# Unveiling the shared etiology between gastrointestinal disorders and valvular heart diseases through a genome-wide pleiotropy study

**DOI:** 10.1038/s44325-025-00054-w

**Published:** 2025-05-22

**Authors:** Jing Xu, Zeye Liu, Lianlian Wu, Fengbo Pei, Hong Jiang, Chen Cheng, Weixian Yang, Jiansong Yuan, Renato Polimanti, Yuejin Yang

**Affiliations:** 1 State Key Laboratory of Cardiovascular Disease, National Center for Cardiovascular Diseases, Fuwai Hospital, Chinese Academy of Medical Sciences & Peking Union Medical College, Beijing, China; 2 Department of Cardiac Surgery, Peking University People’s Hospital, Peking University, Beijing, China; 3 Gastroenterology Department, Renmin Hospital of Wuhan University, Wuhan University, Wuhan, China; 4 Departments of Psychiatry and of Epidemiology (Chronic Diseases), Yale University Schools of Medicine and of Public Health, New Haven, CT USA

**Keywords:** Cardiovascular biology, Cardiovascular diseases

## Abstract

This study explores the genetic link between gastrointestinal disorders and valvular heart disease (VHD), aiming to clarify shared mechanisms through a genome-wide pleiotropy analysis. We assessed the genetic correlation between six gastrointestinal disorders—including GERD, IBS, PUD, IBD, Crohn’s disease, and ulcerative colitis—and VHD, employing methods like linkage disequilibrium score regression and pleiotropic analysis under a composite null hypothesis. Our results show significant genetic correlations, particularly with GERD (rg = 0.211), IBS (rg = 0.23), and PUD (rg = 0.21). Fifteen variants and 64 genes were identified with pleiotropic effects, implicating pathways associated with other conditions like heart defects and dermatitis. Additionally, gut microbiota, specifically *Butyricicoccus*, showed a causal effect on VHD and GERD. This study underscores that gastrointestinal-VHD comorbidity may stem from shared genetic pathways and microbial influences.

## Introduction

Gastrointestinal disorders and valvular heart diseases (VHD) are among the most prevalent medical conditions globally, posing severe healthcare burdens and decreased quality of life for patients^1,2^. Gastrointestinal disorders have diverse clinical manifestations, reflecting different underlying pathophysiological processes, like chronic inflammation, impaired gut motility, and altered gastric acid secretion^3^. Similarly, VHD comprises a group of conditions affecting the normal functioning of the heart valves, leading to reduced blood flow and increased strain on the heart^4^. Both gastrointestinal disorders and VHD present multiple levels of complexity and heterogeneity, making them crucial areas of investigation in contemporary medicine.

Despite the distinct pathophysiological backgrounds of gastrointestinal disorders and VHD, they may share an underlying pathogenetic basis, as vascular diseases and inflammation are known to be involved in the development of these conditions^5,6^. Additionally, the burgeoning area of research supporting the gut-heart axis interconnects the gastrointestinal and cardiovascular systems, presenting complex and bidirectional communication^7,8^. This interaction involves various physiological processes, molecular mechanisms, and signaling pathways, which are influenced by the gut microbiome, diet, and metabolism, among other factors. Alterations in the gut-heart axis have been linked to the development and progression of several gastrointestinal and cardiovascular diseases^9^. Microbiome metabolites, hormonal regulation, and gastrointestinal inflammation can all impact vascular function and are closely linked to cardiac-related diseases^10^. Previous research has primarily focused on the relationship between gastrointestinal function and coronary artery disease^11^, heart failure^12^, and atherosclerosis^13^. However, while multiple studies highlighted the interplay between digestive tract and heart with a potential role for gut microbiome^7,14^, there have been few efforts in assessing the relationship between VHD and gastrointestinal disorders.

With advancements in genome-wide association studies (GWAS), there is growing evidence suggesting the role of genetic factors in the pathophysiology of both gastrointestinal disorders and VHD. GWAS have successfully identified several genetic loci associated with the risk for gastrointestinal disorders, further underscoring the importance of genetic factors in these diseases^15–18^. Additionally, previous research particularly highlighted the critical role of genetic variations in contributing to VHD susceptibility^19,20^. However, it is unclear on the extent of the pleiotropy between gastrointestinal disorders and VHD. Therefore, this study aims to explore the potential shared genetic pathways between gastrointestinal disorders and VHD, investigating their associations and potential mechanisms to illuminate new insights into their co-morbidity and inform more integrated treatment approaches.

**Fig. 1:**
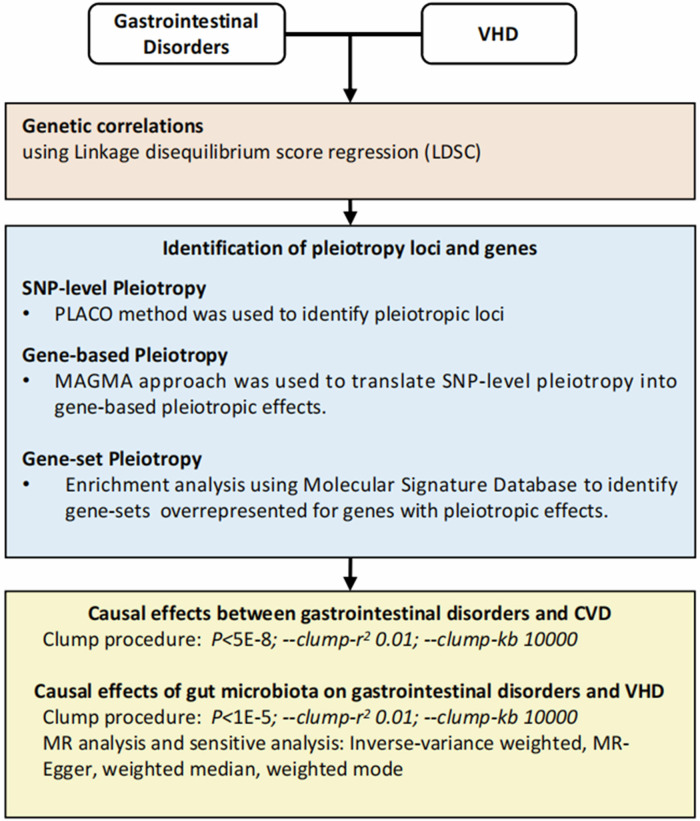
Schematic overview of the study design. The workflow includes three major steps: (1) genetic correlationanalysis between gastrointestinal disorders and valvular heart diseases (VHD) using LDSC; (2) identification ofpleiotropic loci and genes at SNP, gene, and gene-set levels; and (3) Mendelian randomization (MR) analysis toassess causal effects between gastrointestinal disorders, gut microbiota, and VHD.

In this study, we aimed to investigate the shared genetic architecture of VHD with respect to six gastrointestinal disorders (gastro-esophageal reflux disease [GORD], irritable bowel syndrome [IBS], peptic ulcer disease [PUD], inflammatory bowel disease [IBD], Crohn’s disease [CD], and ulcerative colitis [UC]) applying different analytic approaches (Fig. 1) to large-scale genome-wide datasets (Table 1). By elucidating potential links between these complex and heterogeneous conditions, we hope to generate knowledge that may facilitate breakthroughs in our understanding of their pathogenesis and treatment.

**Table 1:** Sample size and data source of the GWAS datasets used in the present study

Abbreviation	Phenotype	Case	Control	Ref
GORD	Gastro-esophageal reflux disease	54,854	401,473	^16^
IBS	Irritable bowel syndrome	53,400	433,201	^16^
PUD	Peptic ulcer disease	16,666	439,661	^16^
IBD	Inflammatory bowel disease	25,042	34,915	^18^
CD	Crohn’s disease	12,194	34915	^18^
UC	Ulcerative colitis	12,366	34,915	^18^
VHD	Valvular heart diseases	72,756	237,263	^21^
VHD	Valvular heart diseases (external validation)	25,070	440,457	^38^

*CD* Crohn’s disease, *GORD* gastro-esophageal reflux disease, *IBD* inflammatory bowel disease, *IBS* irritable bowel syndrome, *PUD* peptic ulcer disease, *UC* ulcerative colitis, *VHD* valvular heart diseases.

## Methods

### Data resources

The GWAS datasets investigated in the present study are summarized in Table 1. GWAS of GORD, IBS, and PUD were previously conducted in 456,327 individuals from the UK Biobank^16^. IBD, CD, and UC GWAS were generated from a meta-analysis of multiple cohorts of European descent^18^. VHD GWAS data were obtained from the Finngen Project Release 8 (available at https://r8.finngen.fi/, ID: I9_VHD)^21^. GWAS data on gut microbiota were generated from 18,340 subjects assessed for 211 taxa, including 131 genera, 35 families, 20 orders, 16 classes and 9 phyla^22^.

### Genetic correlation analysis

Linkage Disequilibrium Score Regression (LDSC)^23^ was utilized to estimate the genetic correlation between gastrointestinal disorders and VHD. LDSC genetic correlation analysis was conducted following the instructions available at https://github.com/bulik/ldsc/wiki/Heritability-and-Genetic-Correlation. Specifically, as recommended, we tested 1,217,311 variants present in the HapMap 3 reference panel and pre-computed linkage disequilibrium (LD) scores based on 1000 Genomes Project reference data on individuals of European ancestry^24^. Strict quality control was implemented for SNPs, including: (i) exclusion of non-biallelic SNPs and those with ambiguous allele coding; (ii) exclusion of SNPs without an rs label; (iii) removal of duplicate SNPs or those not included in the 1000 Genomes Project or with mismatched alleles; (iv) exclusion of SNPs located in the major histocompatibility complex (chr6: 28.5–33.5 Mb) due to their complex LD structure in LDSC analysis; (v) retention of SNPs with a minor allele frequency (MAF) > 0.01. As there was no sample overlap between gastrointestinal disorders and VHD (see description of “data resources” above), we estimated with LDSC genetic covariance intercept constrained to zero to maximize the statistical power of our genetic correlation analyses.

### Pleiotropic analysis

PLACO (Pleiotropic analysis under composite null hypothesis) is a method to investigate variants with pleiotropic effect across multiple traits using genome-wide association statistics^25^. We calculated the square of Z-scores for each variant and removed SNPs with excessively high Z^2^ (>80). Additionally, taking into account the potential correlation between gastrointestinal disorders and VHD, we estimated the correlation matrix of Z. We then used the horizontal α-cross joint test (IUT) method to test the non-multi-effect hypothesis. The final *P* value of the IUT test is the maximum of the *P* values testing H_0_ and H_1_. We considered statistically significant pleiotropic effects surviving genome-wide multiple testing correction (*P* < 5 × 10^−8^).

Based on the PLACO results, we further mapped the identified loci to nearby genes and explored the common biological mechanisms of these multi-effect loci. We used Multi-marker Analysis of GenoMic Annotation approach^26^ to genes underlying the SNP-level pleiotropic effects. Gene-based pleiotropic effects were defined considering a Bonferroni correction accounting for the number of genes tested (*P* < 3 × 10^−6^). FUMA tool^27^ the Molecular Signature Database (MSigDB)^28^ were used to investigate the gene-set enrichment underlying the gene-based pleiotropic effects.

### Summary data-based Mendelian randomization

To follow up the pleiotropy analysis, we used the summary data-based Mendelian randomization (SMR) approach^29^. This method integrates genome-wide association statistics with expression quantitative trait loci (eQTL) data to identify genes whose expression levels are associated with the traits through shared causal genetic variants. In our analysis, we leveraged whole-blood eQTL datasets available from eQTLGen^30^ and GTEx V8^31^. The Heterogeneity in Dependent Instruments (HEIDI) test was performed to distinguish pleiotropy from LD. We considered statistically significant SMR effects those surviving Bonferroni correction accounting for the number of tests performed (*P* < 9.68 × 10^−5^) with HEIDI *P* > 0.05.

### Two-sample Mendelian randomization

We applied the two-sample Mendelian randomization (MR) approach that permits to estimate the effect of an exposure on an outcome of interest using genetic information^32^. Specifically, we investigated the bidirectional relationship between gastrointestinal disorders and VHD. PLINK^33^ was used to define the genetic instruments considering variants association with the exposure of interest (*P* < 5 × 10^−8^) that were LD independent of each other. The r^2^ threshold for instrumental variables was set at 0.001, and the window was set to 500 kb. To ensure the strength of instrumental variables, we calculated the *r*^2^ and F-statistics for each instrumental variable^34^. The formula for F-statistics is as follows:

$$F=\left(\frac{n-1-k}{k}\right)\left(\frac{{r}^{2}}{1-{r}^{2}}\right)$$

where r^2^ represents the proportion of variance explained by instrumental variables, *n* represents the sample size, and *k* represents the number of SNPs. The main MR method used was the inverse variance weighted method (IVW), which requires that instrumental variables (IV) satisfy three assumptions: (i) IV should be related to exposure; (ii) IV should not be associated with confounding factors related to exposure and outcome; (iii) The effect of IV on the outcome is entirely achieved through exposure. Several sensitivity analyses were conducted. First, the Q test of IVW and MR-Egger can detect potential violations of assumptions by heterogeneity among various IVs^35^. Secondly, we applied MR-Egger to estimate the level of horizontal pleiotropy based on its intercept to ensure that genetic variation is independent of exposure and outcome^36^. Additional analyses using MR methods with different modeling assumptions and advantages (weighted median and mode) were performed to increase the stability and robustness of the results. The MR analysis was performed in R3.5.3 software, and MR analysis used the MendelianRandomization package^37^.

We also applied two-sample MR to investigate the possible causal effect of gut microbiota on both VHD and gastrointestinal disorders. Genome-wide association statistics were obtained from MiBioGen consortium initiative (https://mibiogen.gcc.rug.nl/)^22^. This performed GWAS of 21 gut microbiota taxa, including 35 families, 20 orders, 16 classes, 9 phyla, and 131 genera assessed using 16S fecal microbiome gene typing in 18,340 individuals of European descent^22^. First, we used a threshold of *P* < 1 × 10^−5^ to select significant SNPs for each gut microbiota (Supplementary Table 1). Then, we used the clumping procedure in PLINK software to exclude independent instrumental variables with *r*^2^ < 0.001^33^, which was calculated from the European 1000 Genome Projects reference panel^24^. If the IV was missing from the result dataset, proxies with *r*^2^ > 0.8 were added. Finally, to quantify the strength of IV, we also calculated the proportion of variance explained (PVE) and F-statistics (F statistics > 10) for each gut microbiota instrumental variable. Because this is an exploratory analysis, we did not apply a stringent multiple testing correction and considered associations with *P* < 0.01 as significant.

### Validation in an independent VHD GWAS dataset

To assess the robustness of our findings, we performed an external validation using an independent large-scale GWAS dataset of VHD from a previously published meta-analysis^38^. We screened for the most significant SNPs in each previously identified pleiotropic region by extracting all SNPs located within the candidate loci boundaries based on chromosomal positions. For each region, SNPs were ranked by ascending *P* values, and the top SNP (smallest *P*) was retained as the representative variant. We then evaluated whether these lead SNPs showed consistent effect direction and statistical significance in the independent VHD GWAS dataset.

## Results

To assess the genome-wide pleiotropy between VHD and gastrointestinal disorders, we estimated genetic correlation using LDSC approach^23^. After considering Bonferroni multiple testing correction (*P* < 0.008; Supplementary Table 2), VHD showed positive genetic correlation with GORD (*r*_*g*_ = 0.211, *P* = 5.41 × 10^−12^), IBS (*r*_*g*_ = 0.227, *P* = 1.16 × 10^−11^), PUD (*r*_*g*_ = 0.2104, *P* = 1.06 × 10^−6^), IBD (*r*_*g*_ = 0.066, *P* = 0.006), and CD (*r*_*g*_ = 0.078, *P* = 0.001).

**Fig. 2:**
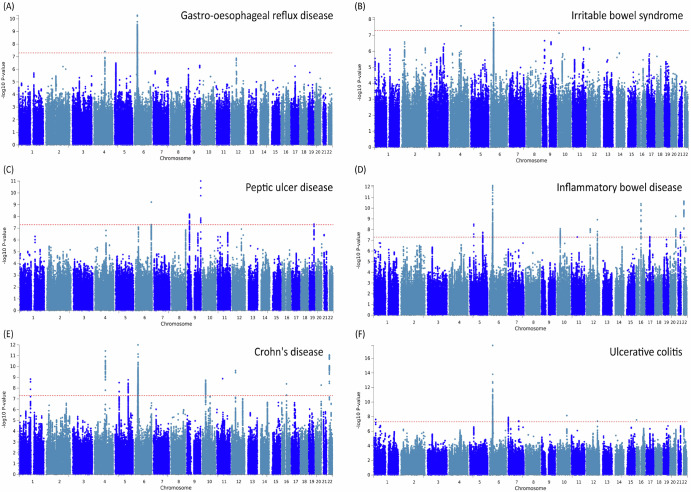
Manhattan plot of the pleiotropic genes (*P* < 3.00E-06) between gastrointestinal tract diseases and VHD. VHD valvular heart diseases.

To follow up the genetic correlations observed, we used PLACO^25^ approach to identify specific loci shared VHD to gastrointestinal disorders. This analysis uncovered 15 pleiotropic loci (Table 2). The Manhattan plot of the pleiotropic genes (*P* < 3.00E-06) between gastrointestinal tract diseases and VHD can be seen in Fig. 2. To strengthen the credibility of our findings, we validated the pleiotropic loci in an independent GWAS dataset of VHD. Among the loci previously identified, five SNPs surpassed the conventional genome-wide significance threshold (*P* < 5 × 10^−8^), and an additional 12 loci reached suggestive significance (*P* < 1 × 10^−3^) in the external VHD dataset. The direction of effect was consistent with the discovery dataset, supporting the reliability of the identified associations (Supplementary Table 3). Across the top signals, multiple pleiotropic variants clustered within the MHC region: rs431722 (UC-VHD pleiotropy *P* = 1.73 × 10^−18^); rs1061808 (IBD-VHD pleiotropy *P* = 7.66 × 10^−13^); rs2394976 (CD-VHD pleiotropy *P* = 9.99 × 10^−13^); rs3131062 (GORD-VHD pleiotropy *P* = 5.25 × 10^−11^); rs9281054 (IBS-VHD pleiotropy *P* = 7.83 × 10^−9^). Although they were located in the same regions, these pleiotropic appear to be independent with partial LD observed only between rs3131062 and rs2394976 (*r*^2^ = 0.38) and between rs431722 and rs1061808 (*r*^2^ = 0.42). Overlapping signals of VHD pleiotropy with the gastrointestinal disorders investigated were observed also in several other genomic regions. To maximize the statistical power of our analysis, we conducted a gene-based analysis of the pleiotropic effects, identifying 64 loci surviving Bonferroni multiple testing correction (*P* < 3 × 10^−6^; Supplementary Table 4): 36 genes for VHD-IBD pleiotropy; 28 genes for VHD-CD pleiotropy; 23 genes for VHD-UC pleiotropy; 16 genes for VHD-UC pleiotropy; three genes for VHD-PUD pleiotropy; two genes for VHD-IBS pleiotropy. There was a consistent overlap of VHD pleiotropy genes across gastrointestinal disorders. For example, four genes within MHC region showed evidence of VHD pleiotropy with four gastrointestinal disorders: *C6orf48* (VHD-pleiotropy: CD-*P* = 1 × 10^−8^; GORD *P* = 4.83 × 10^−7^; IBD *P* = 3.8 × 10^−8^; UC *P* = 1.7 × 10^−8^), *MSH5* (VHD-pleiotropy: CD-*P* = 1.55 × 10^−10^; GORD *P* = 4.40 × 10^−9^; IBD *P* = 1.56 × 10^−9^; IBS *P* = 1.79 × 10^−6^), *PRRC2A* (VHD-pleiotropy: CD-*P* = 8.41 × 10^−7^; GORD *P* = 1.48 × 10^−7^; IBD *P* = 2.24 × 10^−6^; IBS *P* = 2.28 × 10^−7^), and *PBX2* (VHD-pleiotropy: CD-*P* = 2.27 × 10^−6^; GORD *P* = 1.76 × 10^−6^; IBD *P* = 3.15 × 10^−7^; UC *P* = 4.45 × 10^−13^). The gene-set analysis identified that the pleiotropic genes identified were enriched for loci associated with a range of health outcomes (Supplementary Table 5). While the majority of the enrichments were shared across VHD pleiotropy with the gastrointestinal disorders investigated, we also identified mechanisms that appear to be VHD-related mechanism specific to each disorder (Table 3). Among them, the strongest ones were related to conotruncal heart defects (*P* = 7.65 × 10^−5^) for VHD-CD pleiotropic genes, atopic dermatitis (*P* = 3.63 × 10^−6^) for VHD-IBD pleiotropic genes, menopause age of onset for VHD-IBS pleiotropic genes (*P* = 2.33 × 10^−5^), Stevens-Johnson syndrome and toxic epidermal necrolysis (*P* = 4.08 × 10^−11^) for VHD-UC pleiotropic genes.

**Table 2:** Pleiotropic loci (*P* < 5E-8) between gastrointestinal disorders and VHD

VHD Pleiotropy	Locus boundary	Lead SNP	Pleiotropy *P* value	Mapped Gene(s)
CD	1:113,819,380-114,564,892	rs6679677	1.48E-09	*PHTF1, RSBN1*
	4:111,570,369-111,733,700	rs11724067	3.69E-12	*PITX2, LOC729065*
	5:130,438,101-131,955,528	rs25890	1.72E-09	*CSF2, P4HA2*
	5:40,199,659-40,954,627	rs72745992	3.10E-09	intergenic
	6:29,236,722-33,103,592	rs2394976	9.99E-13	*HLA-C, HLA-B*
	10:35,182,402-35,862,021	rs12264698	1.84E-09	*CREM*
	16:50,164,049-50,820,433	rs1906053	4.08E-09	*BRD7, NKD1*
	20:62,191,558-62,649,655	rs381331	5.33E-09	*GMEB2*
	22:21,747,094-22,070,146	rs5749502	8.54E-12	*UBE2L3*
GORD	4:111,486,625-111,639,446	rs2723296	3.94E-08	*PITX2, LOC729065*
	6:29,764,188-33,103,592	rs3131062	5.25E-11	*IER3, DDR1*
IBD	5:130,438,101-131,955,528	rs25890	1.85E-08	*CSF2, P4HA2*
	5:39,666,312-41,004,406	rs10941516	3.18E-09	*LOC285634, LOC100127944*
	6:30,023,079-33,103,592	rs1061808	7.66E-13	*AGPAT1*
	10:35,184,091-35,877,103	rs12764283	8.19E-09	*LOC100129613, CCNY*
	12:111,884,608-113,168,993	rs61941348	2.62E-08	*PTPN11*
	16:50,627,378-50,846,162	rs17312836	3.79E-11	*NOD2*
	17:40,261,284-41,459,600	rs8073836	4.70E-08	*STAT3*
	20:62,191,558-62,649,655	rs381331	5.70E-10	*GMEB2*
	21:40,476,322-40,867,898	rs34989336	1.85E-08	*LOC391282*
	22:21,747,094-22,070,146	rs5749502	2.17E-11	*UBE2L3*
	4:111,486,625-111,639,446	rs2723294	2.54E-08	*PITX2, LOC729065*
	6:29,783,305-33,103,592	rs9281054	7.83E-09	*IER3, DDR1*
PUD	6:160,703,358-161,341,729	rs56393506	6.02E-10	*LPA, PLG*
	9:136,041,865-136,368,406	rs115478735	9.61E-12	intergenic
	9:21,948,078-22,125,913	rs4977575	6.48E-09	*LOC100130239, LOC729983*
	19:49,092,627-49,334,991	rs485186	4.48E-08	*FUT2*
UC	1:20,083,582-20,140,036	rs4655208	2.39E-08	*TMCO4, RNF186*
	6:30,049,966-33,348,012	rs431722	1.73E-18	*NOTCH4*
	7:107,461,908-107,603,431	rs4730273	3.94E-08	*LOC100128307, DLD*
	7:2,725,463-2,930,941	rs2260230	1.25E-08	*GNA12*
	10:101,109,760-101,341,427	rs10748782	7.14E-09	*GOT1, NKX2-3*

*CD* Crohn’s disease, *GORD* gastro-esophageal reflux disease, *IBD* inflammatory bowel disease, *IBS* irritable bowel syndrome, *PUD* peptic ulcer disease, *UC* ulcerative colitis, *VHD* valvular heart diseases.

**Table 3:** Unique gene-set enrichments of VHD pleiotropic genes with each of the gastrointestinal disorders

VHD Pleiotropy	Gene-Set	Genes, *n*	*P* value	genes
CD	Conotruncal heart defects	2	7.65E-05	*GTF2H4, VARS*
	Alopecia areata	3	2.21E-04	*BCL2L15, AP4B1, DCLRE1B*
	Systemic seropositive rheumatic diseases	2	2.58E-04	*AP4B1, YDJC*
IBD	Atopic dermatitis	4	3.63E-06	*PUS10, ATF6B, GPSM3, STAT3*
	Vogt-Koyanagi-Harada syndrome	2	6.29E-05	*IL23R, HLA-DRA*
	Food antigen IgG levels	2	1.08E-04	*NOTCH4, HLA-DRA*
	Systemic lupus erythematosus	5	2.43E-04	*MSH5, NOTCH4, STAT3, UBE2L3, YDJC*
	Lung cancer	4	2.71E-04	*HCG27, MSH5, NOTCH4, HLA-DRA*
IBS	Menopause (age at onset)	2	2.33E-05	*PRRC2A, MSH5*
UC	Stevens-Johnson syndrome and toxic epidermal necrolysis	4	4.08E-11	*PSORS1C1, CCHCR1, TCF19, POU5F1*
	Multiple myeloma	5	6.87E-11	*PSORS1C1, CDSN, CCHCR1, TCF19, POU5F1*
	Chronic hepatitis B infection	3	4.39E-07	*TCF19, NOTCH4, HLA-DPB1*
	Hematology traits	3	2.42E-06	*PSORS1C1, CDSN, CCHCR1*
	Carotid plaque	2	1.19E-05	*CFDP1, TMEM170A*
	Drug-induced Stevens-Johnson syndrome or toxic epidermal necrolysis	2	2.51E-05	*PSORS1C1, POU5F1*
	Systemic sclerosis	3	3.79E-05	*PSORS1C1, NOTCH4, HLA-DPB1*
	White blood cell count	4	4.83E-05	*PSORS1C1, CDSN, CCHCR1, HCG27*
	Chronic obstructive pulmonary disease or coronary artery disease	2	1.25E-04	*CFDP1, TMEM170A*
	Response to hepatitis B vaccine	2	1.81E-04	*PSORS1C1, HLA-DPB1*
	Chronic obstructive pulmonary disease-related biomarkers	2	2.99E-04	*PSORS1C1, CCHCR1*
	Chronic obstructive pulmonary disease	3	4.53E-04	*AGER, AMZ1, CFDP1*
	Night sleep phenotypes	4	6.14E-04	*PSORS1C1, CCHCR1, TCF19, POU5F1*
	Feeling nervous	2	8.65E-04	*PSORS1C1, CCHCR1*
	Automobile speeding propensity	2	8.65E-04	*CDSN, ATF6B*

*CD* Crohn’s disease, *GORD* gastro-esophageal reflux disease, *IBD* inflammatory bowel disease, *IBS* irritable bowel syndrome, *PUD* peptic ulcer disease *UC* ulcerative colitis, *VHD* valvular heart diseases.

Our analysis using SMR approach^29^ identified 16 Bonferroni-significant gene-disease associations mediated by whole-blood transcriptomic regulation (SMR *P* < 9.68 × 10^−5^, HEIDI *P* > 0.05; Supplementary Table 6A). Although no locus showed Bonferroni-significant convergence between both VHD and gastrointestinal disorders, five genes (*CFDP1*, *STAT3*, *IMPDH2*, *UBE2L3*, and *STMN3*) presenting Bonferroni-significant association with at least one of the gastrointestinal disorders tested (Supplementary Table 6A) also had a nominally significant effect on VHD (SMR *P* < 0.05, HEIDI *P* > 0.05; Supplementary Table 6B). *AGER* gene was the only Bonferroni-significant effect observed with respect to VHD (SMR beta=0.212, *P* = 4.82 × 10^−5^; HEIDI *P* = 0.092). For this locus, HEIDI test indicated that its relationship with gastrointestinal disorders was driven by LD rather than pleiotropy (HEIDI *P* < 0.05; Supplementary Table 6C).

**Fig. 3:**
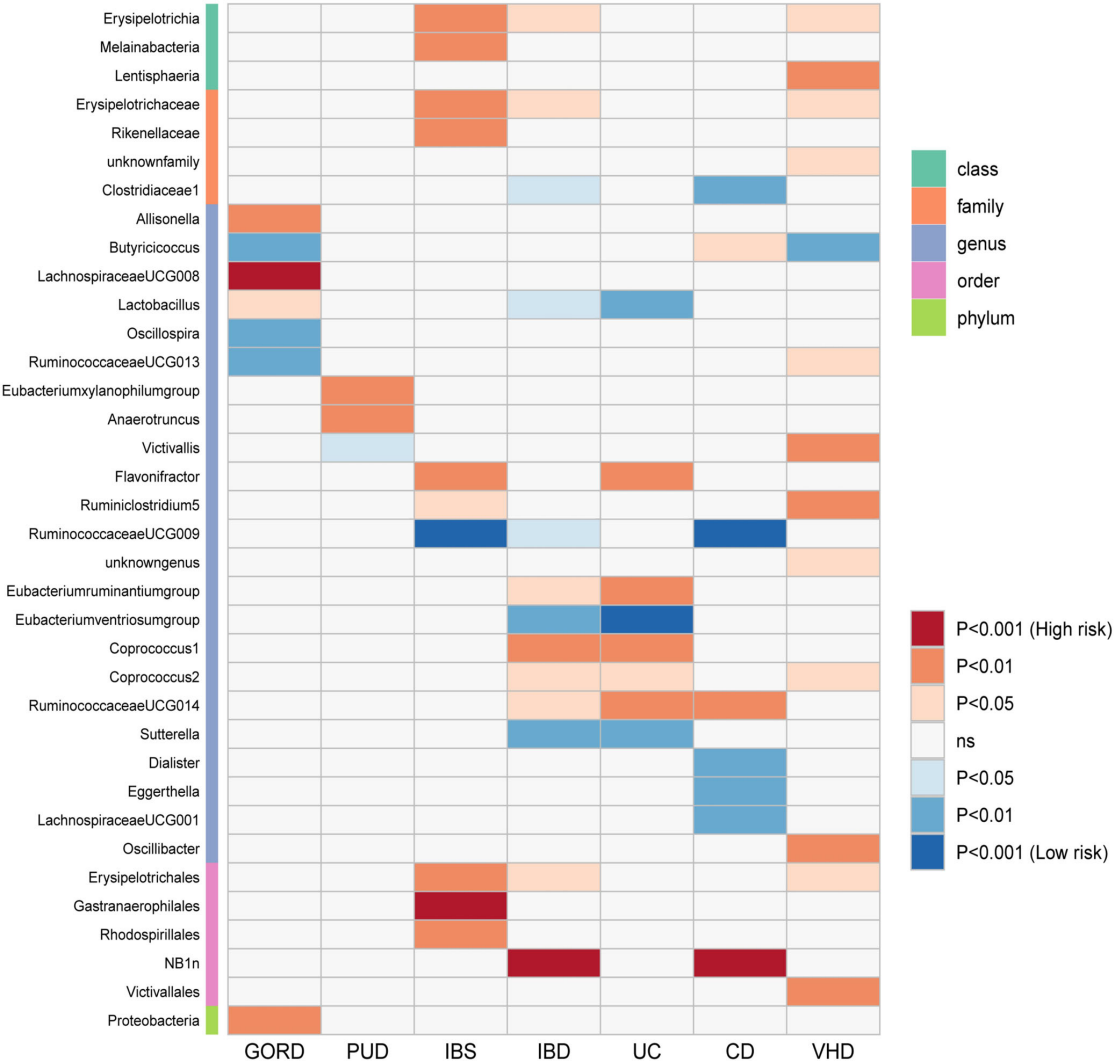
Potential causal relationship of gut microbiota with gastrointestinal disorders and VHD. VHD valvular heart diseases.

While we observed locus-specific pleiotropy between gastrointestinal disorders and VHD, there was no evidence of cause-effect relationships (i.e., vertical pleiotropy) among them through the two-sample MR analyses performed (*P* > 0.05; Supplementary Table 7). However, two-sample MR approach permitted us to observe that gut microbiota can affect both VHD and gastrointestinal disorders (Fig. 3; Supplementary Table 8). Among them, *Butyricicoccus* genus had a genetically inferred causal effect on VHD (beta = −0.133, *P* = 0.004) and GORD (beta = −0.1, *P* = 0.005). Considering a more stringent multiple testing correction (*P* < 2.37 × 10^−4^), we observed that *Lachnospiraceae* genus also affected GORD (beta = 0.91, *P* = 1.79 × 10^−4^). This genus did not show any effect on the other illnesses investigated (*P* > 0.15). Conversely, *Ruminococcaceae* genus showed Bonferroni significant effect on IBS (beta = −0.109, *P* = 2.06 × 10^−4^) and nominally significant associations with CD (beta = −0.242, *P* = 0.001) and IBD (beta = −0.109, *P* = 0.039).

## Discussion

In recent decades, multiple epidemiological studies highlighted the comorbidity between VHD comorbidities and gastrointestinal disorders^39,40^. Additionally, multiple research efforts confirmed the presence of a gut-heart axis, suggesting a complex and bidirectional communication between digestive tract and heart^7^. Although prior GWAS studies have established the role of genetic factors in the pathophysiology of both gastrointestinal disorders and VHD^15,19,20,41^, the scope and underlying genomic mechanisms of their interrelationship have remained elusive. To address this gap, our study employed genomic association analysis, offering novel insights into the phenotypic and genetic association underlying VHD comorbidity with gastrointestinal disorders. Specifically, we identified positive genetic correlations of VHD with GORD, IBS, PUD, IBD, and CD, suggesting that VHD pleiotropic mechanisms are widespread across gastrointestinal disorders.

In line with this hypothesis, our PLACO analysis identified 64 pleiotropic genes that survived Bonferroni multiple testing correction. These genes linked VHD to various gastrointestinal disorders. Intriguingly, several of these genes were localized within the MHC region, an area known for its crucial role in immune responses^42^. For instance, the gene *MSH5*, implicated in both VHD and gastrointestinal disorders, plays a role in DNA repair and meiotic recombination^43,44^, suggesting that DNA instability might be a shared pathophysiological mechanism. Similarly, genes like *PRRC2A* and *PBX2*^45,46^, which are involved in cellular signaling and transcriptional regulation, could potentially affect both cardiac valvular structures and gastrointestinal mucosal integrity. In addition to genes in MHC regions, non-MHC genes like *PHTF1* and *RSBN1* may have distinct roles in cellular processes that might be pivotal in understanding the multifactorial nature of these diseases^47,48^. Similarly, *PITX2* and *LOC729065*, previously associated with gastrointestinal disorders,^49,50^ could be key in deciphering the molecular interplay between cardiac and gastrointestinal physiology. Additionally, the identification of genes like *NOD2*, a known actor in immune response and inflammatory processes, aligns with the proposition of shared inflammatory pathways between VHD and gastrointestinal disorders^51^. These findings point out that multiple molecular pathways contribute to the comorbidity between VHD and gastrointestinal disorders. This is also supported by the fact that several of the genes identified are also related to other outcomes related to different health domains. For instance, the enrichment of genes associated with conotruncal heart defects in VHD-CD pleiotropy could imply a shared developmental or structural etiology that may predispose individuals to both cardiovascular and gastrointestinal complications, emphasizing the importance of exploring congenital abnormalities. Similarly, the enrichment of genes related to atopic dermatitis in VHD-IBD pleiotropy could signify an underlying immune dysregulation affecting multiple organ systems. This is particularly pertinent given that both atopic dermatitis and IBD are conditions with known immune components^52,53^, as is VHD to some extent^54^. It is also intriguing that our enrichment analysis highlighted genes related to the age of onset for menopause in VHD-IBS pleiotropy. This could point to hormonal factors as additional mechanisms impacting both cardiovascular and gastrointestinal health^55,56^. In addition, the association with menopause age of onset for VHD-IBS pleiotropic genes raises intriguing possibilities regarding the role of hormonal changes and their potential impact on both gastrointestinal and cardiac physiology. Hormonal fluctuations and their influences on inflammatory responses, tissue integrity, and cellular signaling can be pivotal in understanding the shared pathophysiology between these disorders^57^. Lastly, the strong association with Stevens-Johnson syndrome and toxic epidermal necrolysis for VHD-UC pleiotropic genes underscores the importance of examining skin manifestations and severe mucocutaneous reactions as they may reflect systemic abnormalities and shared immunological pathways involved in both VHD and gastrointestinal disorders. Taken together, the enrichment findings add an additional layer of complexity to our understanding of the genetic underpinnings of these comorbid conditions, suggesting that the mechanisms linking VHD and gastrointestinal disorders may extend beyond the immediate disease pathways to involve broader biological systems and networks.

Integrating whole-blood transcriptomic information in our pleiotropy analysis using the SMR approach, we uncovered multiple loci that are likely associated with VHD and gastrointestinal diseases through genetically regulated transcriptomic changes in whole blood. However, we observed limited convergence which may be due to the need to investigate transcriptomic profiles of blood, heart, and gastrointestinal cells. Additionally, the SMR analysis highlighted that effects located in the MHC region such as the one observed for the *AGER* locus may appear to be shared between VHD and gastrointestinal diseases due to LD patterns rather than actual pleiotropic effects.

Beyond locus-specific pleiotropic mechanisms, the present study uncovered the potential role of gut microbiome in VHD comorbidity with gastrointestinal disorders, expanding the current knowledge regarding the role of gut microbiome on host health^58,59^. In particular, our findings highlighted potential causal effects of *Butyricicoccus*, *Lachnospiraceae*, and *Ruminococcaceae* genera, *Butyricicoccus* is known for its role in butyrate production, a short-chain fatty acid that has anti-inflammatory effects and is essential for maintaining gut integrity^60^. The genetically inferred protective effect of *Butyricicoccus* on both VHD and GORD supports that butyrate-producing bacteria may have cardioprotective roles^61^. Similarly, the Lachnospiraceae family, found to significantly affect GORD in our study, is recognized for its role in affecting gut health as well as possible microbiota signature in heart failure^62,63^. On the other hand, Ruminococcaceae showed potential effects on IBS, CD, and IBD. This genus is part of another family of butyrate producers^64^ that have been implicated in altered gut motility and even systemic inflammation^65^.

The findings from our study have potential implications for clinical practice and could contribute to design other studies, including clinical trials, to improve the prevention and management of VHD in patients with gastrointestinal disorders. For instance, individuals identified as genetically predisposed to both gastrointestinal disorders and VHD could benefit from targeted screening programs, allowing for timely diagnosis and management of potential cardiovascular complications. Additionally, our research highlights the potential role of gut microbiota in influencing both gastrointestinal health and cardiovascular outcomes. This suggests that dietary interventions or probiotic therapies could be explored as preventive measures. By promoting a healthy gut microbiome, healthcare providers may be able to mitigate the risk of developing VHD while simultaneously improving gastrointestinal health. Integrating these insights into clinical practice encourages a multidisciplinary approach, wherein cardiologists and gastroenterologists collaborate to monitor and manage patients with comorbid conditions. This holistic care model not only enhances patient outcomes but also addresses the complex interplay between gastrointestinal and cardiovascular health, ultimately reducing the healthcare burden associated with these conditions.

While our study provides significant insights into the genetic and microbial factors influencing the comorbidity of VHD and gastrointestinal disorders, several limitations warrant consideration. Firstly, the cross-sectional nature of our data limits our ability to establish causal relationships. Although we employed Mendelian randomization to infer directionality, the observational design restricts our understanding of temporal dynamics between these conditions. Secondly, while our sample size is substantial, it may not fully encompass the genetic and microbial diversity present in different populations, potentially affecting the generalizability of our findings. Thirdly, our focus on pleiotropic effects of specific genes and microbial genera did not account for environmental factors such as diet and lifestyle, which can significantly influence both VHD and gastrointestinal disorders. Additionally, the lack of detailed clinical data on participants may limit our ability to control for confounding variables that could impact the observed associations. Lastly, while we identified key microbial genera, the precise functional mechanisms by which these genera exert their effects remain speculative and necessitate further experimental validation to confirm their roles in the comorbidity of these conditions.

In summary, our study sheds new light on the genetic and microbial factors influencing the comorbidity of VHD and gastrointestinal disorders. We identified positive genetic correlations and significant pleiotropic loci, as well as key microbial genera that contribute to these conditions. These findings provide new insights and evidence for future research and potential therapeutic interventions.

## Supplementary information

Supplementary material

## Data Availability

All data generated or analyzed during this study are included in this published article and its Supplementary Information files. GWAS summary statistics used in this study are summarized in Table 1 and detailed in the "Data resources" subsection of the Methods section, with specific references provided therein. The analysis code used in this study has now been made publicly available on GitHub and can be accessed via the following link: https://github.com/JingXu0701/Gastro_CVD/blob/main/code_to_scxs.R.
