# Supplementary material for: Unveiling the shared etiology between gastrointestinal disorders and valvular heart diseases through a genome-wide pleiotropy study
Source: NPJ Cardiovasc Health. 2025 May 22;2:17. doi: 10.1038/s44325-025-00054-w (PMC12912330; doi:10.1038/s44325-025-00054-w)
Supplement: Supplementary file 1 — Supplementary material [file 44325_2025_54_MOESM1_ESM.pdf]

SUPPLEMENTARY MATERIAL

Supplementary table 1. Gut microbiota instrumental variable.

Supplementary table 2. Genetic correlation of valvular heart disease with gastrointestinal disorders. Bonferroni significant results are indicated in red.

Supplementary table 3. Validation of pleiotropic loci in an independent valvular heart disease GWAS dataset.

Supplementary table 4. Gene-based pleiotropy signals of VHD with gastrointestinal disorders. P values surviving Bonferroni correction are indicated in red.

Supplementary table 5. Gene-set enrichment with respect to gene-based pleiotropy signals survuving false discovery rate multiple testing correction (FDR q<0.05).

Supplementary table 6. Results of the summary-based Mendelian randomization integrating whole-blood expression quantitative trait loci. For each disease-gene association, we report the strongest effect observed between GTEX and eQTLGen datasets. A) Bonferroni-significant disease-gene association with HEIDI $P > 0.05$. B) Nominally significant effect on VHD of Bonferroni-significant gastrointestinal loci. C) Effect of *AGER* gene on VHD and gastrointestinal disorders.

Supplementary table 7. Mendelian randomization between gastrointestinal disorders and VHD.

Supplementary table 8. Mendelian randomization testing the effect of gut microbiota on VHD and gastrointestinal diseases. Effect surviving Bonferroni multiple testing correction (P<2.37E-04) are highlihgted in bold red. Effect surviving the exploratory multiple testing correction (P<0.01) are indicated in red.

Supplementary table 1. Gut microbiota instrumental variable.

Gut	SNP	CHR	POS	A1	A2	BETA	SE	Z	P	N
class.Actinobacteria.id.419	rs687670	2	136740900	C	T	0.10557796	0.012309665	8.470087449	2.45E-17	16313
class.Actinobacteria.id.419	rs7581382	2	135775130	C	T	-0.085112891	0.014138109	-6.523275039	6.88E-11	15292
class.Actinobacteria.id.419	rs12022129	1	207003374	G	A	0.069756094	0.013264752	5.291363233	1.21E-07	16310
class.Actinobacteria.id.419	rs8047955	16	81776768	A	G	0.05758155	0.011676216	4.92730228	8.34E-07	16306
class.Actinobacteria.id.419	rs7322849	13	112859829	T	C	0.094446681	0.019297598	4.984633521	6.21E-07	16313
class.Actinobacteria.id.419	rs72767435	15	94852795	T	C	-0.126352156	0.027362598	-4.702651659	2.57E-06	12898
class.Actinobacteria.id.419	rs134366	22	35567355	G	A	0.111876589	0.023500975	4.810666	1.50E-06	14302
class.Actinobacteria.id.419	rs10841473	12	20378911	G	C	-0.058376469	0.012335331	-4.700630378	2.59E-06	16311
class.Actinobacteria.id.419	rs11738544	5	502594	A	G	0.055031092	0.011501729	4.698248492	2.62E-06	16182
class.Actinobacteria.id.419	rs1515761	10	126110032	T	C	0.076231731	0.016988402	4.566319462	4.96E-06	15525
class.Actinobacteria.id.419	rs1961273	2	137602926	C	T	0.060296939	0.0126047	4.780067332	1.75E-06	16313
class.Actinobacteria.id.419	rs12899991	15	49208992	A	T	0.071992324	0.015466108	4.609923242	4.03E-06	16182
class.Actinobacteria.id.419	rs12049045	1	114665485	A	G	0.05104521	0.01143249	4.449021983	8.63E-06	16311
class.Actinobacteria.id.419	rs1376754	2	161611969	G	A	0.050924155	0.011250292	4.502564005	6.71E-06	16182
class.Actinobacteria.id.419	rs11655079	17	75348675	T	C	-0.056067443	0.01242856	-4.529043738	5.93E-06	16309
class.Actinobacteria.id.419	rs7700479	5	18019549	T	A	-0.076965305	0.017393272	-4.449139926	8.62E-06	16313
class.Actinobacteria.id.419	rs80083040	8	65654844	T	G	0.155783797	0.034801661	4.449150685	8.62E-06	8003
class.Actinobacteria.id.419	rs961091	7	95654718	G	A	0.050125533	0.01125498	4.447787388	8.68E-06	16311
class.Actinobacteria.id.419	rs4945008	11	71221248	A	G	0.054074066	0.012139345	4.548913054	5.39E-06	16313
class.Actinobacteria.id.419	rs857444	6	14617591	C	T	0.050955263	0.011539048	4.441766529	8.92E-06	16312
class.Alphaproteobacteria.id.2379	rs9813022	3	13726736	A	G	-0.075127704	0.015386652	-4.881403724	1.05E-06	9593
class.Alphaproteobacteria.id.2379	rs76784716	2	169033340	A	G	0.133271299	0.026773761	5.022835714	5.09E-07	9593
class.Alphaproteobacteria.id.2379	rs140912403	9	95517807	C	T	-0.160677133	0.031770756	-4.98476783	6.20E-07	8520
class.Alphaproteobacteria.id.2379	rs34569731	2	75630344	A	G	0.070514454	0.015756373	4.482448112	7.38E-06	9115
class.Alphaproteobacteria.id.2379	rs10803434	1	14238305	A	T	-0.069274224	0.01488663	-4.635873227	3.55E-06	9115
class.Alphaproteobacteria.id.2379	rs55876211	3	84471266	C	T	-0.080810954	0.01841593	-4.477948191	7.54E-06	9589
class.Alphaproteobacteria.id.2379	rs7960664	12	89471235	G	A	0.096948894	0.021514853	4.443687332	8.84E-06	9596
class.Alphaproteobacteria.id.2379	rs17061716	3	59982336	G	C	0.092734145	0.020756923	4.432085968	9.33E-06	9465
class.Alphaproteobacteria.id.2379	rs12977163	19	28641647	G	C	0.069023356	0.015382895	4.4894166	7.14E-06	9115
class.Alphaproteobacteria.id.2379	rs62285697	3	176407911	C	T	0.080525582	0.018091166	4.422438163	9.76E-06	9115

Gut	SNP	CHR	POS	A1	A2	BETA	SE	Z	P	N
class.Bacilli.id.1673	rs2952251	8	10143164	A	G	-0.059886787	0.012390272	-4.876664128	1.08E-06	17360
class.Bacilli.id.1673	rs77558518	5	174173171	A	G	-0.107254772	0.022287875	-4.834066003	1.34E-06	15836
class.Bacilli.id.1673	rs57872228	1	200418805	C	T	-0.071477336	0.014664695	-4.907599909	9.22E-07	17375
class.Bacilli.id.1673	rs35344081	16	991253	G	A	0.061952894	0.012706646	4.888712568	1.01E-06	17374
class.Bacilli.id.1673	rs78938557	7	36349586	T	C	0.108007225	0.023285767	4.878507992	1.07E-06	15226
class.Bacilli.id.1673	rs11730038	4	98049499	G	A	-0.063074683	0.012871737	-4.757479224	1.96E-06	17377
class.Bacilli.id.1673	rs76717940	3	94153993	T	A	0.156331484	0.032594546	4.87784685	1.07E-06	8017
class.Bacilli.id.1673	rs74352383	6	67570929	T	A	-0.104495633	0.021708314	-4.807601866	1.53E-06	16519
class.Bacilli.id.1673	rs9581006	13	24973509	C	T	0.225309054	0.046769705	4.775279645	1.79E-06	4253
class.Bacilli.id.1673	rs11110282	12	100585559	A	G	-0.101189316	0.021737883	-4.570982741	4.85E-06	16026
class.Bacilli.id.1673	rs4028634	17	40835649	T	C	0.052061417	0.010984163	4.733427329	2.21E-06	17377
class.Bacilli.id.1673	rs694949	15	58738075	A	G	-0.081145978	0.018004225	-4.47626987	7.60E-06	16600
class.Bacilli.id.1673	rs12797734	11	8332350	T	C	0.057215761	0.012679292	4.487343892	7.21E-06	16902
class.Bacilli.id.1673	rs28564647	9	101101172	T	G	-0.061303431	0.013757893	-4.470272874	7.81E-06	17368
class.Bacilli.id.1673	rs7627280	3	4802165	C	T	0.051120831	0.011464776	4.44437923	8.81E-06	17375
class.Bacilli.id.1673	rs4459992	4	7431487	T	C	0.053585401	0.011636181	4.596408163	4.30E-06	17362
class.Bacilli.id.1673	rs34989881	19	51959855	A	G	0.111055893	0.024603898	4.507944703	6.55E-06	12966
class.Bacilli.id.1673	rs111552159	4	184681021	C	G	0.128031637	0.028692104	4.436857163	9.13E-06	11350
class.Bacilli.id.1673	rs74663707	3	184371224	C	T	0.097915445	0.022407373	4.453279264	8.46E-06	15769
class.Bacteroidia.id.912	rs55773148	13	70523029	G	A	-0.121513597	0.023675903	-5.073977531	3.90E-07	11349
class.Bacteroidia.id.912	rs7631304	3	89339527	G	A	-0.064577053	0.013295739	-4.926587522	8.37E-07	18334
class.Bacteroidia.id.912	rs73975615	17	6461200	G	A	-0.207017892	0.044263343	-4.852986633	1.22E-06	3350
class.Bacteroidia.id.912	rs111845179	14	59317502	T	C	0.102647804	0.021384281	4.907172446	9.24E-07	16393
class.Bacteroidia.id.912	rs2032750	2	53831026	T	C	-0.050839276	0.010680731	-4.76218434	1.92E-06	17853
class.Bacteroidia.id.912	rs7546249	1	176792690	T	A	-0.056711256	0.01183859	-4.804919264	1.55E-06	18333
class.Bacteroidia.id.912	rs929878	16	74290641	C	T	-0.054853206	0.012152954	-4.576253529	4.73E-06	18334
class.Bacteroidia.id.912	rs13291169	9	116888284	C	G	0.068967579	0.014876501	4.625010725	3.75E-06	17513
class.Bacteroidia.id.912	rs17343978	22	27433885	A	C	-0.055205684	0.012027767	-4.455769606	8.36E-06	18334
class.Bacteroidia.id.912	rs72706335	1	157495438	T	C	-0.222409754	0.049345816	-4.474607184	7.66E-06	3819
class.Bacteroidia.id.912	rs11146701	10	39062269	A	G	0.047359582	0.010540996	4.491200742	7.08E-06	18220
class.Bacteroidia.id.912	rs62531359	8	70916181	T	G	0.065572807	0.014989058	4.437839192	9.09E-06	18320

Gut	SNP	CHR	POS	A1	A2	BETA	SE	Z	P	N
class.Bacteroidia.id.912	rs4146051	2	213189994	A	G	-0.107377003	0.024566124	-4.445610503	8.76E-06	11372
class.Bacteroidia.id.912	rs79585701	8	13110185	A	C	0.064682288	0.01496282	4.417478809	9.99E-06	18334
class.Bacteroidia.id.912	rs4916508	3	195936789	G	A	-0.046672941	0.010531247	-4.452916809	8.47E-06	18324
class.Bacteroidia.id.912	rs62575403	9	136493820	C	T	0.140054866	0.031112824	4.491832787	7.06E-06	9668
class.Betaproteobacteria.id.2867	rs4033856	4	45642485	C	T	0.083242265	0.016723677	5.019832426	5.17E-07	15945
class.Betaproteobacteria.id.2867	rs2367850	3	193456274	G	C	0.064005972	0.013037436	4.909785173	9.12E-07	16717
class.Betaproteobacteria.id.2867	rs12467854	2	239888758	G	C	0.06413625	0.013176625	4.87309522	1.10E-06	17038
class.Betaproteobacteria.id.2867	rs6087811	20	30596130	T	G	-0.097817738	0.019852201	-4.949431749	7.44E-07	16184
class.Betaproteobacteria.id.2867	rs62395635	5	173497796	T	C	0.109740893	0.02360002	4.6749038	2.94E-06	15108
class.Betaproteobacteria.id.2867	rs2613606	7	111285025	C	T	-0.051310589	0.010909103	-4.734296144	2.20E-06	17146
class.Betaproteobacteria.id.2867	rs1928341	1	153240013	A	G	0.052558097	0.011040784	4.751884819	2.02E-06	17150
class.Betaproteobacteria.id.2867	rs72747231	9	84206018	C	G	-0.137280671	0.029033768	-4.747928522	2.06E-06	11344
class.Betaproteobacteria.id.2867	rs11128180	3	70592215	A	G	0.059294412	0.012876923	4.629164652	3.67E-06	17151
class.Betaproteobacteria.id.2867	rs2321387	13	58689340	G	A	-0.049459619	0.010942399	-4.533538919	5.80E-06	17149
class.Betaproteobacteria.id.2867	rs1511453	4	23588462	A	G	0.092349246	0.019915827	4.575199961	4.76E-06	17085
class.Betaproteobacteria.id.2867	rs9964679	18	26569901	A	G	0.052911029	0.011486591	4.571355264	4.85E-06	17151
class.Betaproteobacteria.id.2867	rs56386628	8	112732674	C	T	-0.062225169	0.013568762	-4.531034614	5.87E-06	17147
class.Betaproteobacteria.id.2867	rs320161	9	104899117	A	G	0.057228557	0.012619964	4.483803401	7.33E-06	17035
class.Betaproteobacteria.id.2867	rs75242906	15	56786407	C	T	-0.121083253	0.028074987	-4.433471361	9.27E-06	10803
class.Clostridia.id.1859	rs6815608	4	152131744	T	C	0.103803179	0.021088299	5.068075757	4.02E-07	16731
class.Clostridia.id.1859	rs112334273	21	40703251	G	A	0.064100812	0.012740366	5.078010723	3.81E-07	18217
class.Clostridia.id.1859	rs6814436	4	161507301	T	C	0.073999481	0.015081514	4.898624679	9.65E-07	18336
class.Clostridia.id.1859	rs72915163	18	48792829	T	C	-0.058102511	0.012055746	-4.833290492	1.34E-06	18317
class.Clostridia.id.1859	rs10774377	12	5942519	A	G	0.052659008	0.011385834	4.654732866	3.24E-06	18330
class.Clostridia.id.1859	rs13179700	5	149077788	T	C	0.051193667	0.010956865	4.646824914	3.37E-06	18338
class.Clostridia.id.1859	rs6934062	6	163743050	G	C	0.052440576	0.01161154	4.508432731	6.53E-06	17859
class.Clostridia.id.1859	rs1842454	5	105060362	G	A	-0.054720208	0.012733828	-4.446692994	8.72E-06	18340
class.Clostridia.id.1859	rs10209007	2	231676041	G	C	0.079417263	0.018242714	4.419489467	9.89E-06	17812
class.Clostridia.id.1859	rs76767978	3	131371980	G	A	0.069888247	0.015631881	4.454777994	8.40E-06	18329
class.Clostridia.id.1859	rs13105690	4	7420184	T	C	-0.052909681	0.011817976	-4.445276151	8.78E-06	18340
class.Clostridia.id.1859	rs76860606	6	12397187	C	G	0.094703578	0.022511672	4.478082006	7.53E-06	17579

Gut	SNP	CHR	POS	A1	A2	BETA	SE	Z	P	N
class.Clostridia.id.1859	rs72738886	5	35770550	T	C	0.086616952	0.019039264	4.458788875	8.24E-06	16934
class.Clostridia.id.1859	rs7269302	20	50803760	A	T	-0.096398079	0.021200675	-4.43893655	9.04E-06	17405
class.Clostridia.id.1859	rs6797343	3	89268714	G	T	0.059227896	0.013455633	4.431534836	9.36E-06	18340
class.Coriobacteriia.id.809	rs719099	10	65799217	A	G	0.077840173	0.01558151	5.010348118	5.43E-07	17687
class.Coriobacteriia.id.809	rs240104	1	176571431	T	C	-0.060342897	0.012684911	-4.809111417	1.52E-06	17577
class.Coriobacteriia.id.809	rs11250875	10	1922731	T	C	0.060749978	0.013093887	4.572106901	4.83E-06	17690
class.Coriobacteriia.id.809	rs34739816	17	37376685	G	T	0.096501674	0.020766582	4.617517509	3.88E-06	16593
class.Coriobacteriia.id.809	rs76779974	5	125460084	C	G	0.0774888	0.017187698	4.539794605	5.63E-06	16419
class.Coriobacteriia.id.809	rs80046645	5	459415	C	G	0.255839805	0.056199532	4.579679319	4.66E-06	3204
class.Coriobacteriia.id.809	rs45480394	19	55857777	T	G	-0.050426168	0.011330684	-4.423433012	9.71E-06	17413
class.Coriobacteriia.id.809	rs1816223	12	11494021	A	G	-0.058626474	0.012900576	-4.571556654	4.84E-06	17688
class.Coriobacteriia.id.809	rs7898585	10	5644995	T	C	0.073150094	0.01641856	4.554673113	5.25E-06	17691
class.Coriobacteriia.id.809	rs67561917	20	62072077	A	G	-0.071436723	0.015411343	-4.548955316	5.39E-06	17333
class.Coriobacteriia.id.809	rs3025411	9	136512906	A	G	0.092654285	0.020946781	4.458095088	8.27E-06	15671
class.Coriobacteriia.id.809	rs11656361	17	8121332	A	C	0.077300587	0.01755297	4.464644853	8.02E-06	16665
class.Coriobacteriia.id.809	rs1397793	5	90471451	G	A	-0.049885455	0.011244635	-4.422117946	9.77E-06	17527
class.Coriobacteriia.id.809	rs11073596	15	86433579	T	G	0.051021228	0.011433922	4.46135018	8.14E-06	17527
class.Coriobacteriia.id.809	rs2442778	3	11654412	G	A	-0.116408329	0.025854513	-4.439256238	9.03E-06	13472
class.Coriobacteriia.id.809	rs7540303	1	179629191	C	T	0.048322746	0.010906179	4.425509713	9.62E-06	17691
class.Coriobacteriia.id.809	rs62448869	7	24464799	T	A	-0.048605943	0.010898242	-4.468615174	7.87E-06	17527
class.Coriobacteriia.id.809	rs13307134	7	105084680	C	T	0.056568602	0.012626474	4.470620179	7.80E-06	17527
class.Coriobacteriia.id.809	rs4880783	10	1223742	G	T	0.056502976	0.012505544	4.530118208	5.90E-06	17691
class.Coriobacteriia.id.809	rs12974142	19	52895166	G	A	0.078979608	0.017720479	4.451876291	8.51E-06	17413
class.Coriobacteriia.id.809	rs8010111	14	39660509	G	A	-0.10337688	0.022928631	-4.496866139	6.90E-06	15996
class.Deltaproteobacteria.id.3087	rs6058181	20	33694801	C	T	0.082571565	0.016595819	5.100055448	3.40E-07	15100
class.Deltaproteobacteria.id.3087	rs4506934	17	2856662	C	T	-0.093651362	0.020120034	-4.633873303	3.59E-06	15061
class.Deltaproteobacteria.id.3087	rs17791387	9	81834426	A	G	-0.073588121	0.015424623	-4.798044097	1.60E-06	15071
class.Deltaproteobacteria.id.3087	rs2838334	21	45064961	G	A	0.056172159	0.012412391	4.546848915	5.45E-06	15168
class.Deltaproteobacteria.id.3087	rs9928243	16	71541641	C	A	-0.053845737	0.011771796	-4.563916294	5.02E-06	15167
class.Deltaproteobacteria.id.3087	rs11599763	10	11855599	T	C	-0.054421883	0.011739659	-4.614457659	3.94E-06	15168
class.Deltaproteobacteria.id.3087	rs112381107	9	15269705	C	T	0.206594184	0.045690815	4.580866121	4.63E-06	4457

Gut	SNP	CHR	POS	A1	A2	BETA	SE	Z	P	N
class.Deltaproteobacteria.id.3087	rs17084793	18	69312914	G	A	-0.071076166	0.015953009	-4.53772861	5.69E-06	15164
class.Deltaproteobacteria.id.3087	rs2692012	1	203991605	A	G	0.1103349	0.025333574	4.661321165	3.14E-06	13104
class.Deltaproteobacteria.id.3087	rs1035691	11	10658735	A	G	-0.055175977	0.012160175	-4.424832414	9.65E-06	15168
class.Deltaproteobacteria.id.3087	rs62020470	15	96161065	A	G	-0.058541133	0.012935848	-4.571137443	4.85E-06	15168
class.Deltaproteobacteria.id.3087	rs16851319	1	203308383	G	C	-0.07055156	0.015084942	-4.538005572	5.68E-06	15154
class.Deltaproteobacteria.id.3087	rs55744759	8	57867671	A	G	-0.077969339	0.017073844	-4.484348709	7.31E-06	15168
class.Erysipelotrichia.id.2147	rs62504403	8	38803551	C	T	0.068115892	0.012788339	5.305661665	1.12E-07	16981
class.Erysipelotrichia.id.2147	rs7234058	18	5830507	T	C	-0.094575892	0.019409083	-4.909660622	9.12E-07	16874
class.Erysipelotrichia.id.2147	rs17530232	13	40385457	A	G	0.103049982	0.022464754	4.685613075	2.79E-06	16622
class.Erysipelotrichia.id.2147	rs35161940	17	70327224	T	C	-0.080604056	0.01676409	-4.769690104	1.85E-06	18213
class.Erysipelotrichia.id.2147	rs10781552	10	133897233	C	T	-0.055221236	0.011607425	-4.722368313	2.33E-06	17789
class.Erysipelotrichia.id.2147	rs8003149	14	56156504	C	T	0.053879756	0.01168879	4.60702288	4.08E-06	18213
class.Erysipelotrichia.id.2147	rs4078432	14	48997206	C	T	-0.060892934	0.013376363	-4.599698061	4.23E-06	17789
class.Erysipelotrichia.id.2147	rs1074800	5	3002546	A	G	0.049243827	0.010887044	4.521309241	6.15E-06	17789
class.Erysipelotrichia.id.2147	rs56970041	14	80357610	T	G	0.072414447	0.016447064	4.548481456	5.40E-06	18217
class.Erysipelotrichia.id.2147	rs7826267	8	2954952	T	G	-0.083907147	0.019913089	-4.433199344	9.28E-06	17586
class.Erysipelotrichia.id.2147	rs1884466	1	64139196	C	T	-0.047536345	0.01069386	-4.427639255	9.53E-06	18205
class.Erysipelotrichia.id.2147	rs290833	1	97457427	T	G	-0.049737371	0.011137647	-4.464409174	8.03E-06	17789
class.Gammaproteobacteria.id.3303	rs6706173	2	167986992	A	C	0.074250965	0.014639234	5.199807093	1.99E-07	16020
class.Gammaproteobacteria.id.3303	rs11181912	12	43572952	G	A	-0.057915255	0.011879636	-4.892671813	9.95E-07	16017
class.Gammaproteobacteria.id.3303	rs79795896	18	48185248	A	G	-0.159209919	0.03513731	-4.467225985	7.92E-06	7577
class.Gammaproteobacteria.id.3303	rs2234691	10	48385682	T	C	0.076290532	0.017196192	4.454914973	8.39E-06	16011
class.Gammaproteobacteria.id.3303	rs6469506	8	115123937	T	A	0.054142416	0.011633307	4.651842907	3.29E-06	16023
class.Gammaproteobacteria.id.3303	rs12404135	1	186502694	A	G	-0.078884268	0.017241506	-4.442430132	8.89E-06	15231
class.Gammaproteobacteria.id.3303	rs9973122	18	75633634	T	A	0.074061387	0.016259636	4.500792154	6.77E-06	15650
class.Gammaproteobacteria.id.3303	rs9494710	6	137612554	C	T	-0.054967253	0.012092397	-4.584714759	4.55E-06	15650
class.Gammaproteobacteria.id.3303	rs75101789	3	18615903	C	T	0.072901201	0.016300252	4.445031752	8.79E-06	16024
class.Lentisphaeria.id.2250	rs2825714	21	21023966	A	G	-0.137410136	0.02892458	-4.783567792	1.72E-06	4734
class.Lentisphaeria.id.2250	rs17114848	15	25162535	G	A	0.152376929	0.032433167	4.60835594	4.06E-06	4734
class.Lentisphaeria.id.2250	rs77599476	20	61394262	A	G	0.230291775	0.048016811	4.767925577	1.86E-06	3722
class.Lentisphaeria.id.2250	rs72640280	1	11943792	A	G	0.220206945	0.048619648	4.557348316	5.18E-06	3816

Gut	SNP	CHR	POS	A1	A2	BETA	SE	Z	P	N
class.Lentisphaeria.id.2250	rs62570196	9	111086170	C	T	-0.216350467	0.043986577	-4.876607762	1.08E-06	3712
class.Lentisphaeria.id.2250	rs11764871	7	146808977	G	T	0.105796117	0.023346418	4.513221575	6.39E-06	4725
class.Lentisphaeria.id.2250	rs2731834	5	94720245	C	G	0.109437835	0.023692997	4.599081581	4.24E-06	4733
class.Lentisphaeria.id.2250	rs2031282	13	20687179	A	G	0.122368503	0.027032933	4.59236071	4.38E-06	4734
class.Lentisphaeria.id.2250	rs1002941	15	101242690	G	A	0.105025041	0.023348442	4.461248306	8.15E-06	4734
class.Lentisphaeria.id.2250	rs73113483	3	6223568	T	A	-0.131216985	0.0288713	-4.448097775	8.66E-06	4718
class.Melainabacteria.id.1589	rs9864379	3	14306949	T	C	-0.159734555	0.029255297	-5.438915141	5.36E-08	5777
class.Melainabacteria.id.1589	rs11150282	16	80493705	T	C	0.09894875	0.019697164	4.990288491	6.03E-07	5873
class.Melainabacteria.id.1589	rs16851659	1	230523039	G	C	-0.089610337	0.01864323	-4.84363222	1.27E-06	5884
class.Melainabacteria.id.1589	rs73074665	7	20010624	A	T	0.166215674	0.035572757	4.680191177	2.87E-06	5758
class.Melainabacteria.id.1589	rs4129395	9	115975389	G	A	0.08961869	0.01851058	4.813841652	1.48E-06	5887
class.Melainabacteria.id.1589	rs79790072	15	100747683	T	C	0.226721783	0.048784054	4.651825902	3.29E-06	4107
class.Melainabacteria.id.1589	rs10738747	9	26184578	G	A	0.081462422	0.018440929	4.418082555	9.96E-06	5884
class.Melainabacteria.id.1589	rs1221147	9	122046549	T	A	0.123968094	0.027895845	4.490447485	7.11E-06	5768
class.Melainabacteria.id.1589	rs113884518	9	24648997	T	C	-0.20535201	0.045498234	-4.463671729	8.06E-06	4442
class.Melainabacteria.id.1589	rs789069	18	1008278	A	C	-0.103539228	0.023428675	-4.498410305	6.85E-06	5879
class.Melainabacteria.id.1589	rs367480	11	2937631	G	A	-0.08375796	0.018579584	-4.459914143	8.20E-06	5887
class.Melainabacteria.id.1589	rs28678345	17	53906328	T	C	0.214848361	0.047087794	4.503292067	6.69E-06	4323
class.Melainabacteria.id.1589	rs10148250	14	107061448	G	A	0.086192825	0.019338072	4.447900847	8.67E-06	5453
class.Methanobacteria.id.119	rs73457410	13	41956181	A	G	0.215339755	0.04366952	4.823790925	1.41E-06	3498
class.Methanobacteria.id.119	rs6776814	3	15053083	T	C	-0.199565931	0.04118249	-4.79452384	1.63E-06	3385
class.Methanobacteria.id.119	rs894996	4	104418307	C	A	0.216999069	0.044907554	4.766388791	1.88E-06	3582
class.Methanobacteria.id.119	rs75208022	12	21338861	C	T	-0.227244713	0.048763001	-4.529189927	5.92E-06	3203
class.Methanobacteria.id.119	rs12825290	12	43701132	C	G	-0.216781075	0.049349641	-4.523738926	6.08E-06	3144
class.Methanobacteria.id.119	rs56131665	5	178160975	G	A	0.178661188	0.039297982	4.520138571	6.18E-06	3572
class.Methanobacteria.id.119	rs11018665	11	89349287	A	T	0.111465287	0.025051554	4.508829011	6.52E-06	3698
class.Methanobacteria.id.119	rs4257531	3	2086167	G	A	0.164498365	0.03649593	4.480597376	7.44E-06	3162
class.Methanobacteria.id.119	rs6508769	19	28827760	T	C	0.15351913	0.034451866	4.459234474	8.23E-06	3319
class.Methanobacteria.id.119	rs73068003	7	10773932	G	T	-0.158140381	0.035180317	-4.453559383	8.45E-06	3692
class.Methanobacteria.id.119	rs10424197	19	46439321	G	A	-0.111274175	0.024751678	-4.433329849	9.28E-06	3698
class.Mollicutes.id.3920	rs74603314	14	46519718	T	C	0.221639279	0.046291765	4.80379142	1.56E-06	4499

Gut	SNP	CHR	POS	A1	A2	BETA	SE	Z	P	N
class.Mollicutes.id.3920	rs10108398	8	59440824	G	A	0.076914215	0.015395275	4.87465347	1.09E-06	10932
class.Mollicutes.id.3920	rs11890098	2	157532549	A	G	0.074438031	0.015338858	4.900368368	9.57E-07	11392
class.Mollicutes.id.3920	rs72901605	11	47103877	T	C	-0.084185237	0.01781192	-4.653852616	3.26E-06	10526
class.Mollicutes.id.3920	rs3768491	1	109965986	A	G	-0.068105197	0.01490611	-4.599575976	4.23E-06	11002
class.Mollicutes.id.3920	rs17214486	14	97159674	C	A	0.060989958	0.013562263	4.505917879	6.61E-06	11387
class.Mollicutes.id.3920	rs12566890	1	61850864	T	G	-0.101147132	0.023097845	-4.630323303	3.65E-06	11371
class.Mollicutes.id.3920	rs78169027	11	108439087	A	G	-0.108282943	0.023728915	-4.530798993	5.88E-06	10542
class.Mollicutes.id.3920	rs28537087	15	95309676	G	A	0.08208727	0.018831168	4.463212567	8.07E-06	11382
class.Mollicutes.id.3920	rs2464826	7	79490250	A	C	0.094423946	0.021180616	4.454853483	8.40E-06	10998
class.Negativicutes.id.2164	rs1643968	5	165266628	T	C	-0.056528439	0.011229806	-5.062130941	4.15E-07	18170
class.Negativicutes.id.2164	rs60274479	16	21249925	T	C	-0.065962286	0.013413689	-4.861958016	1.16E-06	17610
class.Negativicutes.id.2164	rs13086907	3	142135117	G	A	0.062531199	0.013173503	4.758034029	1.95E-06	18163
class.Negativicutes.id.2164	rs73232831	4	17413426	G	A	-0.151828859	0.03149318	-4.766501422	1.87E-06	9382
class.Negativicutes.id.2164	rs71405394	15	101245088	G	A	-0.114177822	0.02404996	-4.737256816	2.17E-06	13861
class.Negativicutes.id.2164	rs61249479	9	124912908	A	C	0.077707533	0.01686282	4.673943117	2.95E-06	18165
class.Negativicutes.id.2164	rs1447205	8	41231597	G	C	-0.050205329	0.010838203	-4.67544014	2.93E-06	18165
class.Negativicutes.id.2164	rs4463806	10	115597993	T	C	-0.05439664	0.012936525	-4.47041991	7.81E-06	18160
class.Negativicutes.id.2164	rs9423647	10	5579818	G	A	0.047817029	0.010528201	4.524245342	6.06E-06	18164
class.Negativicutes.id.2164	rs2834062	21	34377485	A	G	0.0489057	0.010884164	4.453728035	8.44E-06	18171
class.Negativicutes.id.2164	rs1649999	10	80086072	A	G	0.074898861	0.01664577	4.476682001	7.58E-06	18036
class.Negativicutes.id.2164	rs1135612	7	75609677	G	A	0.052978507	0.01191767	4.433697196	9.26E-06	18164
class.Verrucomicrobiae.id.4029	rs11729256	4	95027272	T	C	0.074979802	0.015017677	4.968971477	6.73E-07	11861
class.Verrucomicrobiae.id.4029	rs9349825	6	56341481	A	G	-0.070403566	0.014712789	-4.705144056	2.54E-06	11858
class.Verrucomicrobiae.id.4029	rs74542928	4	100544188	T	C	0.112163707	0.023642195	4.794327123	1.63E-06	11216
class.Verrucomicrobiae.id.4029	rs3995795	10	11389614	C	T	0.064127531	0.014099468	4.557136659	5.19E-06	11690
class.Verrucomicrobiae.id.4029	rs2602429	16	81063149	C	T	0.074684437	0.015619438	4.701468957	2.58E-06	11690
class.Verrucomicrobiae.id.4029	rs4242783	10	5064327	G	A	0.068929219	0.014769297	4.697308453	2.64E-06	11690
class.Verrucomicrobiae.id.4029	rs11184341	1	105422565	G	C	0.065534193	0.014223078	4.604526701	4.13E-06	11864
class.Verrucomicrobiae.id.4029	rs61779207	1	41074472	G	A	-0.075830853	0.016776157	-4.502279537	6.72E-06	11591
class.Verrucomicrobiae.id.4029	rs117107102	18	49473635	A	G	0.20468288	0.043157251	4.676493858	2.92E-06	4975
class.Verrucomicrobiae.id.4029	rs12908520	15	97570657	G	A	0.061892558	0.013094626	4.736758778	2.17E-06	11863

Gut	SNP	CHR	POS	A1	A2	BETA	SE	Z	P	N
class.Verrucomicrobiae.id.4029	rs111862613	12	130309670	T	C	0.090698957	0.019674596	4.62541648	3.74E-06	11621
class.Verrucomicrobiae.id.4029	rs941682	20	31867840	G	A	-0.063143598	0.014376741	-4.425766881	9.61E-06	11809
family.Acidaminococcaceae.id.2166	rs6923842	6	5723181	T	C	-0.079610621	0.016919247	-4.732928636	2.21E-06	11904
family.Acidaminococcaceae.id.2166	rs6589457	11	114701288	A	G	0.165910976	0.034951601	4.723773984	2.32E-06	7386
family.Acidaminococcaceae.id.2166	rs2933324	9	107117653	A	G	-0.066255706	0.014025652	-4.730764362	2.24E-06	12525
family.Acidaminococcaceae.id.2166	rs262812	6	158647999	T	C	-0.065674997	0.014225656	-4.654412665	3.25E-06	12099
family.Acidaminococcaceae.id.2166	rs74540770	3	186553389	G	A	-0.108969098	0.024353266	-4.491027408	7.09E-06	10606
family.Acidaminococcaceae.id.2166	rs6427992	1	203102563	G	C	-0.059585662	0.012960033	-4.599098093	4.24E-06	12527
family.Acidaminococcaceae.id.2166	rs45497800	20	61991833	T	C	-0.117862982	0.025735121	-4.531224478	5.86E-06	11316
family.Acidaminococcaceae.id.2166	rs78702810	3	18462531	T	C	-0.143755204	0.032324372	-4.435997767	9.16E-06	7992
family.Actinomycetaceae.id.421	rs35011108	6	133007480	A	G	0.24182648	0.050379638	4.771707717	1.83E-06	3630
family.Actinomycetaceae.id.421	rs2889192	9	76394568	G	T	0.088753744	0.019500862	4.630708314	3.64E-06	7710
family.Actinomycetaceae.id.421	rs34583783	6	67207371	G	T	0.123755794	0.026426988	4.545530195	5.48E-06	7710
family.Actinomycetaceae.id.421	rs58484246	15	29850991	T	C	0.0761407	0.016900672	4.52205286	6.12E-06	7710
family.Actinomycetaceae.id.421	rs4073240	6	169224781	G	A	0.074754607	0.016468358	4.524518052	6.05E-06	7710
family.Alcaligenaceae.id.2875	rs62191117	2	239900775	A	G	0.068430199	0.013363598	5.13917787	2.76E-07	16688
family.Alcaligenaceae.id.2875	rs9537886	13	58545246	A	C	-0.057088849	0.011068258	-5.169192126	2.35E-07	16807
family.Alcaligenaceae.id.2875	rs4033856	4	45642485	C	T	0.081833964	0.016893105	4.886005218	1.03E-06	15623
family.Alcaligenaceae.id.2875	rs67737557	20	29836081	C	G	0.073840948	0.015167785	4.88440342	1.04E-06	15876
family.Alcaligenaceae.id.2875	rs17000015	4	75867283	A	T	-0.060925748	0.012536315	-4.868025712	1.13E-06	16377
family.Alcaligenaceae.id.2875	rs2367850	3	193456274	G	C	0.062910409	0.013171198	4.769557669	1.85E-06	16377
family.Alcaligenaceae.id.2875	rs7638039	3	70588939	T	C	0.060333529	0.012777892	4.692051309	2.70E-06	16791
family.Alcaligenaceae.id.2875	rs6087811	20	30596130	T	G	-0.092704374	0.020045669	-4.646974946	3.37E-06	15868
family.Alcaligenaceae.id.2875	rs62395635	5	173497796	T	C	0.110582886	0.023851434	4.648291244	3.35E-06	14802
family.Alcaligenaceae.id.2875	rs112159068	6	138305038	A	T	0.131483982	0.028108868	4.635262793	3.56E-06	11656
family.Alcaligenaceae.id.2875	rs6969323	7	104214487	A	C	-0.059356404	0.012913653	-4.617398244	3.89E-06	16810
family.Alcaligenaceae.id.2875	rs7302582	12	1396151	G	C	-0.106816521	0.023298022	-4.648996142	3.34E-06	15113
family.Alcaligenaceae.id.2875	rs1153990	5	104233461	A	G	-0.058726971	0.012830567	-4.527524534	5.97E-06	16808
family.Alcaligenaceae.id.2875	rs9964679	18	26569901	A	G	0.053705118	0.011600815	4.588057899	4.47E-06	16810
family.Alcaligenaceae.id.2875	rs112135816	9	82485327	T	G	-0.077505169	0.017155357	-4.553477833	5.28E-06	15545
family.Alcaligenaceae.id.2875	rs2040996	7	111294074	T	C	-0.049092245	0.011023144	-4.470623755	7.80E-06	16801

Gut	SNP	CHR	POS	A1	A2	BETA	SE	Z	P	N
family.Alcaligenaceae.id.2875	rs147968	16	85945839	C	T	0.048947517	0.011032087	4.436884836	9.13E-06	16810
family.Alcaligenaceae.id.2875	rs74776516	11	20763367	T	G	-0.09440518	0.021328653	-4.498281731	6.85E-06	14502
family.Alcaligenaceae.id.2875	rs28480294	15	71522509	T	C	-0.051696664	0.011550326	-4.506003551	6.61E-06	16377
family.Alcaligenaceae.id.2875	rs79846372	4	83635863	A	G	0.088245866	0.019832763	4.494114318	6.99E-06	16186
family.Bacteroidaceae.id.917	rs6795673	3	10593224	C	T	0.053856525	0.010525127	5.100974685	3.38E-07	18301
family.Bacteroidaceae.id.917	rs28757219	6	29143451	T	A	0.081839827	0.017031866	4.840642393	1.29E-06	14833
family.Bacteroidaceae.id.917	rs9507307	13	24910476	C	T	0.060445621	0.012912793	4.740957737	2.13E-06	18184
family.Bacteroidaceae.id.917	rs66474973	20	58605432	G	T	0.081251539	0.016447899	4.966714765	6.81E-07	17405
family.Bacteroidaceae.id.917	rs11585893	1	10644351	A	G	-0.074074561	0.014763325	-4.775219844	1.80E-06	17821
family.Bacteroidaceae.id.917	rs495004	9	112855426	C	G	-0.060747491	0.012986536	-4.643779611	3.42E-06	17707
family.Bacteroidaceae.id.917	rs17619981	19	24342250	T	G	0.088097761	0.018700207	4.693413983	2.69E-06	15801
family.Bacteroidaceae.id.917	rs2023437	14	22045949	T	C	-0.07823245	0.016763399	-4.564023942	5.02E-06	17484
family.Bacteroidaceae.id.917	rs13207588	6	41519430	A	G	-0.059204982	0.013119468	-4.479407749	7.49E-06	17707
family.Bacteroidaceae.id.917	rs2366421	3	62019400	T	A	-0.05281527	0.011712437	-4.474767651	7.65E-06	18299
family.Bacteroidaceae.id.917	rs1340391	1	102960989	T	C	-0.059200382	0.013224416	-4.502050394	6.73E-06	18302
family.BacteroidalesS24.7group.id.11173	rs738193	22	25943322	T	C	0.084732776	0.016585588	5.077634559	3.82E-07	7778
family.BacteroidalesS24.7group.id.11173	rs941000	7	90714667	C	T	0.085026574	0.016338237	5.113910618	3.16E-07	7778
family.BacteroidalesS24.7group.id.11173	rs689695	15	47352866	C	A	0.081457787	0.016705675	4.84277449	1.28E-06	7954
family.BacteroidalesS24.7group.id.11173	rs17043785	2	53139362	T	C	-0.176186835	0.034713295	-5.02182162	5.12E-07	5208
family.BacteroidalesS24.7group.id.11173	rs10872669	6	151515172	A	G	-0.123070536	0.027566518	-4.428476662	9.49E-06	7368
family.BacteroidalesS24.7group.id.11173	rs12748533	1	242767252	G	T	-0.082110804	0.017270056	-4.700898483	2.59E-06	7948
family.BacteroidalesS24.7group.id.11173	rs6831034	4	149300138	T	A	-0.095654602	0.020522806	-4.522755847	6.10E-06	7576
family.BacteroidalesS24.7group.id.11173	rs61508842	3	158653545	T	C	0.122574311	0.027238851	4.469839849	7.83E-06	7576
family.BacteroidalesS24.7group.id.11173	rs78609301	6	63236747	A	G	-0.086706514	0.019570857	-4.490879392	7.09E-06	7953
family.BacteroidalesS24.7group.id.11173	rs11135366	5	164763068	C	G	0.084214168	0.01837388	4.445268389	8.78E-06	7953
family.BacteroidalesS24.7group.id.11173	rs7217209	17	21205200	C	T	0.084301877	0.018730791	4.45397817	8.43E-06	7745
family.Bifidobacteriaceae.id.433	rs687670	2	136740900	C	T	0.111068768	0.012910884	8.468270288	2.49E-17	14915
family.Bifidobacteriaceae.id.433	rs7581382	2	135775130	C	T	-0.090211798	0.01478989	-6.570874875	5.00E-11	13923
family.Bifidobacteriaceae.id.433	rs7322849	13	112859829	T	C	0.110675689	0.020099551	5.636013515	1.74E-08	14915
family.Bifidobacteriaceae.id.433	rs1961273	2	137602926	C	T	0.068812456	0.013169905	5.229758778	1.70E-07	14915
family.Bifidobacteriaceae.id.433	rs76671854	5	169748189	C	G	-0.089298109	0.018317684	-4.88461344	1.04E-06	13923

Gut	SNP	CHR	POS	A1	A2	BETA	SE	Z	P	N
family.Bifidobacteriaceae.id.433	rs4957061	5	521096	T	C	0.056987252	0.011690731	4.863771462	1.15E-06	14795
family.Bifidobacteriaceae.id.433	rs10841473	12	20378911	G	C	-0.061303123	0.012884445	-4.726816492	2.28E-06	14914
family.Bifidobacteriaceae.id.433	rs4567981	2	49906864	T	A	0.057776313	0.011738841	4.914949465	8.88E-07	14795
family.Bifidobacteriaceae.id.433	rs677208	18	4304187	G	C	0.072858928	0.016482072	4.526350411	6.00E-06	14915
family.Bifidobacteriaceae.id.433	rs12446429	16	898055	T	C	0.081009916	0.019073029	4.451459465	8.53E-06	14008
family.Bifidobacteriaceae.id.433	rs11655079	17	75348675	T	C	-0.058614646	0.012995163	-4.529059711	5.92E-06	14912
family.Bifidobacteriaceae.id.433	rs540489	17	72897722	T	G	-0.06325184	0.013816951	-4.549861933	5.37E-06	14688
family.Bifidobacteriaceae.id.433	rs857444	6	14617591	C	T	0.05539651	0.012066937	4.620676066	3.82E-06	14914
family.Bifidobacteriaceae.id.433	rs10857328	4	161211924	T	A	0.056200033	0.012639517	4.48646831	7.24E-06	14915
family.Bifidobacteriaceae.id.433	rs10831953	11	13098051	G	A	0.053753749	0.012374596	4.418313309	9.95E-06	14915
family.Bifidobacteriaceae.id.433	rs7174549	15	92463303	C	T	0.055172631	0.01246547	4.497744987	6.87E-06	14915
family.Bifidobacteriaceae.id.433	rs73797465	5	142793467	T	G	-0.094261173	0.020842122	-4.571195472	4.85E-06	14795
family.Bifidobacteriaceae.id.433	rs55888705	4	1517826	A	G	0.053672952	0.012058615	4.448130649	8.66E-06	14914
family.Bifidobacteriaceae.id.433	rs6899771	6	97406220	A	G	-0.09143118	0.020260353	-4.485334979	7.28E-06	14150
family.Bifidobacteriaceae.id.433	rs7588568	2	189805077	A	C	-0.060715954	0.013060506	-4.625862767	3.73E-06	14913
family.Christensenellaceae.id.1866	rs62573205	9	137786999	G	A	-0.065480354	0.013350288	-4.812768966	1.49E-06	16838
family.Christensenellaceae.id.1866	rs870002	8	62220151	C	T	0.048895121	0.010981691	4.508030186	6.54E-06	16847
family.Christensenellaceae.id.1866	rs12657403	5	3174785	A	G	0.078107959	0.017188664	4.541227719	5.59E-06	16245
family.Christensenellaceae.id.1866	rs117186816	11	123936128	G	A	-0.206475619	0.043636232	-4.508796364	6.52E-06	4310
family.Christensenellaceae.id.1866	rs9608766	22	29828040	T	C	-0.068213442	0.01495997	-4.649287432	3.33E-06	16185
family.Christensenellaceae.id.1866	rs4076564	6	169640257	G	A	-0.188623195	0.039209045	-4.463555917	8.06E-06	3730
family.Christensenellaceae.id.1866	rs4805124	19	35884202	C	G	0.048650752	0.010906688	4.455681344	8.36E-06	16847
family.Christensenellaceae.id.1866	rs72706624	9	15518852	G	T	0.088262607	0.019757489	4.513471704	6.38E-06	14869
family.Christensenellaceae.id.1866	rs7211194	17	13541941	C	T	0.049209379	0.011091636	4.418474917	9.94E-06	16436
family.Christensenellaceae.id.1866	rs892686	9	83043376	A	G	0.050610664	0.010965057	4.605049966	4.12E-06	16847
family.Christensenellaceae.id.1866	rs12380890	9	38002256	A	G	-0.050397219	0.011104738	-4.534232773	5.78E-06	16846
family.Clostridiaceae1.id.1869	rs12186080	3	132596627	G	A	0.074823577	0.016245981	4.550983791	5.34E-06	14244
family.Clostridiaceae1.id.1869	rs2795528	10	43270264	G	A	-0.180968849	0.039091059	-4.621690208	3.81E-06	6411
family.Clostridiaceae1.id.1869	rs550843	6	165722832	T	C	-0.073478656	0.016838326	-4.490933008	7.09E-06	14234
family.Clostridiaceae1.id.1869	rs12341505	9	136710881	G	A	0.081450333	0.017933832	4.584770142	4.54E-06	14250
family.Clostridiaceae1.id.1869	rs4723021	7	30934659	T	C	-0.106376242	0.024194378	-4.481228314	7.42E-06	13682

Gut	SNP	CHR	POS	A1	A2	BETA	SE	Z	P	N
family.Clostridiaceae1.id.1869	rs881532	22	47806774	A	G	-0.053303513	0.011901062	-4.467967814	7.90E-06	14250
family.Clostridiaceae1.id.1869	rs2817172	1	3041519	C	T	0.056336654	0.012392023	4.553731571	5.27E-06	13789
family.Clostridiaceae1.id.1869	rs10875374	1	101804809	C	T	-0.053675937	0.011943586	-4.46257091	8.10E-06	14253
family.Clostridiaceae1.id.1869	rs12490337	3	111482845	C	G	-0.06169436	0.013693009	-4.496590635	6.91E-06	14253
family.Clostridiaceae1.id.1869	rs62397761	6	48093014	A	G	0.061580452	0.013619788	4.43806641	9.08E-06	14248
family.Clostridiaceae1.id.1869	rs56188186	16	87710690	A	G	0.096777351	0.02174714	4.458736001	8.24E-06	13218
family.ClostridialesvadinBB60group.id.11286	rs55682560	15	87392290	C	T	-0.131519432	0.026131877	-5.027454421	4.97E-07	9183
family.ClostridialesvadinBB60group.id.11286	rs9979874	21	38612024	G	C	-0.073892457	0.015091139	-4.881519575	1.05E-06	10506
family.ClostridialesvadinBB60group.id.11286	rs28691777	17	58149405	C	T	0.137133741	0.026699624	4.962561049	6.96E-07	10199
family.ClostridialesvadinBB60group.id.11286	rs6588624	1	56849539	G	A	-0.066231659	0.013814656	-4.775471324	1.79E-06	10512
family.ClostridialesvadinBB60group.id.11286	rs7538034	1	71391694	T	G	-0.078597952	0.016598223	-4.719258778	2.37E-06	10420
family.ClostridialesvadinBB60group.id.11286	rs66714985	8	3305122	A	C	0.116907778	0.025244679	4.571029445	4.85E-06	10512
family.ClostridialesvadinBB60group.id.11286	rs118104867	8	123977538	C	T	0.214463834	0.04550976	4.642872677	3.44E-06	4095
family.ClostridialesvadinBB60group.id.11286	rs2191834	2	229942951	G	T	0.074637506	0.015913614	4.707971146	2.50E-06	10511
family.ClostridialesvadinBB60group.id.11286	rs7226487	18	74373125	A	G	-0.064368248	0.013870145	-4.634221964	3.58E-06	10505
family.ClostridialesvadinBB60group.id.11286	rs10517600	4	155632958	T	G	0.062699295	0.01393642	4.498990761	6.83E-06	10512
family.ClostridialesvadinBB60group.id.11286	rs17121075	14	85856796	G	A	0.07692543	0.017223352	4.467492686	7.91E-06	10512
family.ClostridialesvadinBB60group.id.11286	rs10904722	10	6714424	C	T	-0.067231397	0.014712327	-4.562767694	5.05E-06	10512
family.ClostridialesvadinBB60group.id.11286	rs6755871	2	202450739	C	G	-0.061382475	0.013897576	-4.432132023	9.33E-06	10500
family.ClostridialesvadinBB60group.id.11286	rs34088226	5	4308946	A	G	-0.117806973	0.026923974	-4.474412635	7.66E-06	9716
family.ClostridialesvadinBB60group.id.11286	rs13409132	2	205616346	A	G	-0.165418684	0.035215443	-4.59285117	4.37E-06	5913
family.ClostridialesvadinBB60group.id.11286	rs989682	3	15564453	A	G	0.070193983	0.01553641	4.498387002	6.85E-06	10129
family.Coriobacteriaceae.id.811	rs719099	10	65799217	A	G	0.077840173	0.01558151	5.010348118	5.43E-07	17687
family.Coriobacteriaceae.id.811	rs240104	1	176571431	T	C	-0.060342897	0.012684911	-4.809111417	1.52E-06	17577
family.Coriobacteriaceae.id.811	rs11250875	10	1922731	T	C	0.060749978	0.013093887	4.572106901	4.83E-06	17690
family.Coriobacteriaceae.id.811	rs34739816	17	37376685	G	T	0.096501674	0.020766582	4.617517509	3.88E-06	16593
family.Coriobacteriaceae.id.811	rs76779974	5	125460084	C	G	0.0774888	0.017187698	4.539794605	5.63E-06	16419
family.Coriobacteriaceae.id.811	rs80046645	5	459415	C	G	0.255839805	0.056199532	4.579679319	4.66E-06	3204
family.Coriobacteriaceae.id.811	rs45480394	19	55857777	T	G	-0.050426168	0.011330684	-4.423433012	9.71E-06	17413
family.Coriobacteriaceae.id.811	rs1816223	12	11494021	A	G	-0.058626474	0.012900576	-4.571556654	4.84E-06	17688
family.Coriobacteriaceae.id.811	rs7898585	10	5644995	T	C	0.073150094	0.01641856	4.554673113	5.25E-06	17691

Gut	SNP	CHR	POS	A1	A2	BETA	SE	Z	P	N
family.Coriobacteriaceae.id.811	rs67561917	20	62072077	A	G	-0.071436723	0.015411343	-4.548955316	5.39E-06	17333
family.Coriobacteriaceae.id.811	rs3025411	9	136512906	A	G	0.092654285	0.020946781	4.458095088	8.27E-06	15671
family.Coriobacteriaceae.id.811	rs11656361	17	8121332	A	C	0.077300587	0.01755297	4.464644853	8.02E-06	16665
family.Coriobacteriaceae.id.811	rs1397793	5	90471451	G	A	-0.049885455	0.011244635	-4.422117946	9.77E-06	17527
family.Coriobacteriaceae.id.811	rs11073596	15	86433579	T	G	0.051021228	0.011433922	4.46135018	8.14E-06	17527
family.Coriobacteriaceae.id.811	rs2442778	3	11654412	G	A	-0.116408329	0.025854513	-4.439256238	9.03E-06	13472
family.Coriobacteriaceae.id.811	rs7540303	1	179629191	C	T	0.048322746	0.010906179	4.425509713	9.62E-06	17691
family.Coriobacteriaceae.id.811	rs62448869	7	24464799	T	A	-0.048605943	0.010898242	-4.468615174	7.87E-06	17527
family.Coriobacteriaceae.id.811	rs13307134	7	105084680	C	T	0.056568602	0.012626474	4.470620179	7.80E-06	17527
family.Coriobacteriaceae.id.811	rs4880783	10	1223742	G	T	0.056502976	0.012505544	4.530118208	5.90E-06	17691
family.Coriobacteriaceae.id.811	rs12974142	19	52895166	G	A	0.078979608	0.017720479	4.451876291	8.51E-06	17413
family.Coriobacteriaceae.id.811	rs8010111	14	39660509	G	A	-0.10337688	0.022928631	-4.496866139	6.90E-06	15996
family.Defluviitaleaceae.id.1924	rs72731813	4	147414743	C	T	-0.149751171	0.02934152	-5.139291053	2.76E-07	7786
family.Defluviitaleaceae.id.1924	rs55658617	21	41811003	T	C	0.17731425	0.03618445	4.824141085	1.41E-06	6676
family.Defluviitaleaceae.id.1924	rs9725395	1	85205632	A	G	-0.138356652	0.029518507	-4.644216337	3.41E-06	8031
family.Defluviitaleaceae.id.1924	rs17051335	4	122202910	C	T	-0.13431353	0.029220144	-4.583107395	4.58E-06	8031
family.Defluviitaleaceae.id.1924	rs112893842	9	8786663	T	C	0.11076127	0.023254479	4.688636368	2.75E-06	8203
family.Defluviitaleaceae.id.1924	rs28696126	7	79734541	A	T	-0.107106351	0.023817267	-4.542342002	5.56E-06	7830
family.Defluviitaleaceae.id.1924	rs1908593	18	59026148	T	C	0.070222973	0.015660058	4.468989578	7.86E-06	8203
family.Defluviitaleaceae.id.1924	rs9608282	22	24804081	T	G	0.139013635	0.029942857	4.581872291	4.61E-06	7736
family.Defluviitaleaceae.id.1924	rs12122999	1	118782748	G	T	-0.081676551	0.018093841	-4.520053576	6.18E-06	7830
family.Defluviitaleaceae.id.1924	rs4344384	10	66407366	G	T	0.070880349	0.015612105	4.531374792	5.86E-06	8200
family.Defluviitaleaceae.id.1924	rs540220	9	93331308	T	C	-0.123953592	0.028981413	-4.428675082	9.48E-06	7887
family.Desulfovibrionaceae.id.3169	rs6058181	20	33694801	C	T	0.083474185	0.016611075	5.143329138	2.70E-07	15074
family.Desulfovibrionaceae.id.3169	rs4506934	17	2856662	C	T	-0.094338072	0.020137994	-4.660108457	3.16E-06	15035
family.Desulfovibrionaceae.id.3169	rs2692012	1	203991605	A	G	0.11423063	0.025356811	4.802891965	1.56E-06	13080
family.Desulfovibrionaceae.id.3169	rs11599763	10	11855599	T	C	-0.055593074	0.011749345	-4.708371573	2.50E-06	15142
family.Desulfovibrionaceae.id.3169	rs2838334	21	45064961	G	A	0.057141929	0.012422992	4.620739404	3.82E-06	15142
family.Desulfovibrionaceae.id.3169	rs112381107	9	15269705	C	T	0.211435354	0.045696154	4.683603538	2.82E-06	4455
family.Desulfovibrionaceae.id.3169	rs16851319	1	203308383	G	C	-0.073269696	0.015096375	-4.716743939	2.40E-06	15128
family.Desulfovibrionaceae.id.3169	rs17791387	9	81834426	A	G	-0.072925066	0.015438052	-4.743865696	2.10E-06	15045

Gut	SNP	CHR	POS	A1	A2	BETA	SE	Z	P	N
family.Desulfovibrionaceae.id.3169	rs9928243	16	71541641	C	A	-0.054173165	0.011781876	-4.587550093	4.48E-06	15141
family.Desulfovibrionaceae.id.3169	rs7164160	15	96159374	A	T	-0.057445479	0.012946842	-4.483481375	7.34E-06	15142
family.Desulfovibrionaceae.id.3169	rs72647048	8	57865378	T	C	-0.077008881	0.017089038	-4.425793274	9.61E-06	15142
family.Enterobacteriaceae.id.3469	rs78143293	18	57672335	A	G	-0.084852127	0.017041521	-4.856359027	1.20E-06	13572
family.Enterobacteriaceae.id.3469	rs111229068	11	2563450	A	T	0.110560883	0.024171888	4.630175311	3.65E-06	12350
family.Enterobacteriaceae.id.3469	rs62210023	20	55340092	A	G	0.060675357	0.013012497	4.661992323	3.13E-06	13515
family.Enterobacteriaceae.id.3469	rs504442	18	55145547	T	G	0.084159032	0.01894789	4.557731073	5.17E-06	13578
family.Enterobacteriaceae.id.3469	rs80319214	2	4015933	C	G	0.099040406	0.021587746	4.495120205	6.95E-06	12641
family.Enterobacteriaceae.id.3469	rs11026530	11	22379097	T	C	0.082240818	0.018637912	4.429919283	9.43E-06	13485
family.Enterobacteriaceae.id.3469	rs2374342	2	42133542	C	A	0.058292653	0.01261946	4.586092616	4.52E-06	13577
family.Enterobacteriaceae.id.3469	rs79757635	13	110840418	C	A	0.075859672	0.017128562	4.43240967	9.32E-06	13419
family.Enterobacteriaceae.id.3469	rs35673018	16	54327745	G	A	0.08996415	0.020293422	4.47528099	7.63E-06	13326
family.Enterobacteriaceae.id.3469	rs61973590	14	27718039	C	G	-0.060539207	0.013369618	-4.451294856	8.54E-06	13570
family.Enterobacteriaceae.id.3469	rs4792380	17	13521040	A	T	0.115597849	0.025768301	4.428484728	9.49E-06	12763
family.Erysipelotrichaceae.id.2149	rs62504403	8	38803551	C	T	0.068115892	0.012788339	5.305661665	1.12E-07	16981
family.Erysipelotrichaceae.id.2149	rs7234058	18	5830507	T	C	-0.094575892	0.019409083	-4.909660622	9.12E-07	16874
family.Erysipelotrichaceae.id.2149	rs17530232	13	40385457	A	G	0.103049982	0.022464754	4.685613075	2.79E-06	16622
family.Erysipelotrichaceae.id.2149	rs35161940	17	70327224	T	C	-0.080604056	0.01676409	-4.769690104	1.85E-06	18213
family.Erysipelotrichaceae.id.2149	rs10781552	10	133897233	C	T	-0.055221236	0.011607425	-4.722368313	2.33E-06	17789
family.Erysipelotrichaceae.id.2149	rs8003149	14	56156504	C	T	0.053879756	0.01168879	4.60702288	4.08E-06	18213
family.Erysipelotrichaceae.id.2149	rs4078432	14	48997206	C	T	-0.060892934	0.013376363	-4.599698061	4.23E-06	17789
family.Erysipelotrichaceae.id.2149	rs1074800	5	3002546	A	G	0.049243827	0.010887044	4.521309241	6.15E-06	17789
family.Erysipelotrichaceae.id.2149	rs56970041	14	80357610	T	G	0.072414447	0.016447064	4.548481456	5.40E-06	18217
family.Erysipelotrichaceae.id.2149	rs7826267	8	2954952	T	G	-0.083907147	0.019913089	-4.433199344	9.28E-06	17586
family.Erysipelotrichaceae.id.2149	rs1884466	1	64139196	C	T	-0.047536345	0.01069386	-4.427639255	9.53E-06	18205
family.Erysipelotrichaceae.id.2149	rs290833	1	97457427	T	G	-0.049737371	0.011137647	-4.464409174	8.03E-06	17789
family.FamilyXI.id.1936	rs2155352	11	95357209	A	G	-0.150537618	0.030231918	-4.971773481	6.63E-07	3150
family.FamilyXI.id.1936	rs697771	16	54115200	A	G	-0.117677217	0.025149135	-4.657951253	3.19E-06	3152
family.FamilyXI.id.1936	rs10759623	9	115821485	C	T	-0.162114556	0.032150976	-4.998322552	5.78E-07	3156
family.FamilyXI.id.1936	rs11547158	7	148921732	A	G	-0.177599232	0.037279461	-4.692247834	2.70E-06	3133
family.FamilyXI.id.1936	rs488164	1	239995451	T	G	0.117997561	0.025515955	4.573438205	4.80E-06	3149

Gut	SNP	CHR	POS	A1	A2	BETA	SE	Z	P	N
family.FamilyXI.id.1936	rs3733511	4	119955787	A	G	0.128253369	0.02747177	4.645926314	3.39E-06	3156
family.FamilyXI.id.1936	rs6025153	20	55348292	G	C	0.118618541	0.026196893	4.49154472	7.07E-06	3130
family.FamilyXI.id.1936	rs78222136	16	64956642	G	C	0.224834617	0.051167198	4.454403932	8.41E-06	3136
family.FamilyXI.id.1936	rs17379710	11	35334668	T	C	-0.116383434	0.025212482	-4.612890726	3.97E-06	3156
family.FamilyXI.id.1936	rs2156611	18	43345864	T	C	-0.112486332	0.025109889	-4.429924576	9.43E-06	3155
family.FamilyXIII.id.1957	rs118170811	10	102366896	A	G	0.151619208	0.031711234	4.774273165	1.80E-06	9645
family.FamilyXIII.id.1957	rs12643275	4	162211872	A	T	-0.055090712	0.011647397	-4.715157896	2.42E-06	16269
family.FamilyXIII.id.1957	rs1999289	21	19685992	A	T	-0.063736569	0.013926964	-4.705048856	2.54E-06	16269
family.FamilyXIII.id.1957	rs482905	1	166557202	G	T	0.059652844	0.012717031	4.626250638	3.72E-06	16737
family.FamilyXIII.id.1957	rs3098182	15	50802980	G	T	0.05085114	0.011014044	4.60930158	4.04E-06	16731
family.FamilyXIII.id.1957	rs6797051	3	88995218	C	T	-0.080598449	0.017136413	-4.569332286	4.89E-06	16737
family.FamilyXIII.id.1957	rs66753613	1	39212021	G	A	0.065113557	0.014425125	4.46297678	8.08E-06	16269
family.FamilyXIII.id.1957	rs7514702	1	186914023	T	C	-0.066323634	0.014162562	-4.615768981	3.92E-06	16737
family.FamilyXIII.id.1957	rs7076829	10	7906746	T	A	-0.051639411	0.011231289	-4.575057454	4.76E-06	16269
family.FamilyXIII.id.1957	rs10404377	19	16174657	C	A	-0.050318303	0.011165005	-4.493984671	6.99E-06	16269
family.FamilyXIII.id.1957	rs4293737	3	32373530	A	G	-0.112090617	0.027130494	-4.455114449	8.38E-06	5704
family.FamilyXIII.id.1957	rs1887048	1	65170838	G	A	0.057997854	0.012960206	4.42478341	9.65E-06	16717
family.FamilyXIII.id.1957	rs12904405	15	94105415	A	G	-0.085189628	0.019268843	-4.474079485	7.67E-06	16643
family.Lachnospiraceae.id.1987	rs9929145	16	76559184	G	A	-0.125721183	0.024523339	-5.134036058	2.84E-07	15866
family.Lachnospiraceae.id.1987	rs11755180	6	161957425	C	G	-0.04922703	0.010489407	-4.693186732	2.69E-06	18333
family.Lachnospiraceae.id.1987	rs35524804	9	100113683	T	C	-0.060748124	0.012521578	-4.712248076	2.45E-06	17859
family.Lachnospiraceae.id.1987	rs11979110	7	130436459	T	C	-0.050092357	0.010506859	-4.772622409	1.82E-06	18339
family.Lachnospiraceae.id.1987	rs79086868	9	133791605	T	C	0.077672344	0.016447234	4.669848238	3.01E-06	17522
family.Lachnospiraceae.id.1987	rs112040820	17	80469064	A	G	0.054958365	0.011708564	4.71489425	2.42E-06	17512
family.Lachnospiraceae.id.1987	rs2159863	4	10245354	A	G	-0.058583102	0.012874212	-4.627807585	3.70E-06	18340
family.Lachnospiraceae.id.1987	rs11139361	9	72179847	T	C	0.049446281	0.011050331	4.598148398	4.26E-06	18333
family.Lachnospiraceae.id.1987	rs1205443	20	36876868	A	G	0.0501415	0.011215719	4.485086464	7.29E-06	17779
family.Lachnospiraceae.id.1987	rs7359994	19	29408858	T	C	-0.050314217	0.01124817	-4.550219843	5.36E-06	18340
family.Lachnospiraceae.id.1987	rs146660815	6	46664437	T	C	-0.215113896	0.047677775	-4.508382114	6.53E-06	4112
family.Lachnospiraceae.id.1987	rs959845	4	186906801	C	T	-0.049366584	0.010780126	-4.557788688	5.17E-06	17859
family.Lachnospiraceae.id.1987	rs10402491	19	13425799	C	T	0.066170067	0.014850954	4.476617077	7.58E-06	18334

Gut	SNP	CHR	POS	A1	A2	BETA	SE	Z	P	N
family.Lachnospiraceae.id.1987	rs13005175	2	232469722	A	G	0.099348285	0.021839504	4.455476469	8.37E-06	16893
family.Lachnospiraceae.id.1987	rs2910921	5	32348593	T	C	0.160302066	0.035832011	4.454223425	8.42E-06	6549
family.Lachnospiraceae.id.1987	rs12760724	1	214031969	A	C	-0.048456589	0.010793397	-4.485704646	7.27E-06	18340
family.Lachnospiraceae.id.1987	rs3127230	10	103392503	C	T	-0.050353667	0.011220058	-4.519340812	6.20E-06	18340
family.Lachnospiraceae.id.1987	rs11841382	13	38004672	G	T	-0.071812797	0.017461974	-4.426493524	9.58E-06	18340
family.Lactobacillaceae.id.1836	rs16861661	1	18501459	G	A	-0.193287418	0.037929323	-5.143177068	2.70E-07	5385
family.Lactobacillaceae.id.1836	rs921925	19	6928017	A	C	0.0999419	0.020168726	4.998695378	5.77E-07	7053
family.Lactobacillaceae.id.1836	rs768253	8	69010103	T	G	-0.079234443	0.017060426	-4.632794143	3.61E-06	7059
family.Lactobacillaceae.id.1836	rs6092149	20	54192992	A	T	-0.082564564	0.017028222	-4.827643523	1.38E-06	7006
family.Lactobacillaceae.id.1836	rs328312	16	73890910	T	A	0.082529013	0.016822496	4.919170514	8.69E-07	7051
family.Lactobacillaceae.id.1836	rs11674854	2	28903275	C	T	-0.083022375	0.017526743	-4.700484156	2.60E-06	6750
family.Lactobacillaceae.id.1836	rs820999	1	16773387	T	G	-0.075829079	0.017015119	-4.442653464	8.89E-06	7058
family.Lactobacillaceae.id.1836	rs77478751	3	173151665	A	G	-0.219177718	0.047356625	-4.527822481	5.96E-06	3704
family.Lactobacillaceae.id.1836	rs74599091	1	179720669	A	G	0.191612904	0.042503931	4.47349259	7.70E-06	4951
family.Lactobacillaceae.id.1836	rs9345899	6	67559323	A	G	-0.12404185	0.027894638	-4.42948364	9.45E-06	6602
family.Lactobacillaceae.id.1836	rs1590596	13	23836941	C	G	-0.103810248	0.022013537	-4.616675383	3.90E-06	6750
family.Lactobacillaceae.id.1836	rs62314653	4	109896462	C	A	0.177081987	0.039226901	4.506457196	6.59E-06	6016
family.Methanobacteriaceae.id.121	rs73457410	13	41956181	A	G	0.215339755	0.04366952	4.823790925	1.41E-06	3498
family.Methanobacteriaceae.id.121	rs6776814	3	15053083	T	C	-0.199565931	0.04118249	-4.79452384	1.63E-06	3385
family.Methanobacteriaceae.id.121	rs894996	4	104418307	C	A	0.216999069	0.044907554	4.766388791	1.88E-06	3582
family.Methanobacteriaceae.id.121	rs75208022	12	21338861	C	T	-0.227244713	0.048763001	-4.529189927	5.92E-06	3203
family.Methanobacteriaceae.id.121	rs12825290	12	43701132	C	G	-0.216781075	0.049349641	-4.523738926	6.08E-06	3144
family.Methanobacteriaceae.id.121	rs56131665	5	178160975	G	A	0.178661188	0.039297982	4.520138571	6.18E-06	3572
family.Methanobacteriaceae.id.121	rs11018665	11	89349287	A	T	0.111465287	0.025051554	4.508829011	6.52E-06	3698
family.Methanobacteriaceae.id.121	rs4257531	3	2086167	G	A	0.164498365	0.03649593	4.480597376	7.44E-06	3162
family.Methanobacteriaceae.id.121	rs6508769	19	28827760	T	C	0.15351913	0.034451866	4.459234474	8.23E-06	3319
family.Methanobacteriaceae.id.121	rs73068003	7	10773932	G	T	-0.158140381	0.035180317	-4.453559383	8.45E-06	3692
family.Methanobacteriaceae.id.121	rs10424197	19	46439321	G	A	-0.111274175	0.024751678	-4.433329849	9.28E-06	3698
family.Oxalobacteraceae.id.2966	rs4428215	3	171947435	G	A	0.125609687	0.023005262	5.455653217	4.88E-08	5167
family.Oxalobacteraceae.id.2966	rs1569853	6	38550301	T	C	-0.140042787	0.028170393	-4.949203312	7.45E-07	4997
family.Oxalobacteraceae.id.2966	rs6000536	22	37421469	C	T	-0.118382797	0.024135485	-4.950898071	7.39E-07	5166

Gut	SNP	CHR	POS	A1	A2	BETA	SE	Z	P	N
family.Oxalobacteraceae.id.2966	rs36057338	4	189935314	G	T	0.181604096	0.03987425	4.517484282	6.26E-06	4738
family.Oxalobacteraceae.id.2966	rs7993559	13	23443627	C	A	0.092105903	0.020111361	4.563228761	5.04E-06	4989
family.Oxalobacteraceae.id.2966	rs10465181	9	87500772	C	T	0.096407873	0.020012818	4.809407928	1.51E-06	5165
family.Oxalobacteraceae.id.2966	rs12002250	9	19682558	A	C	0.196145295	0.044523026	4.543529119	5.53E-06	4726
family.Oxalobacteraceae.id.2966	rs111966731	15	93941937	T	C	0.204031062	0.044574616	4.583929713	4.56E-06	4387
family.Oxalobacteraceae.id.2966	rs17138946	16	5984558	G	T	-0.1894439	0.043024819	-4.46278621	8.09E-06	4776
family.Oxalobacteraceae.id.2966	rs11246212	11	610277	T	C	0.136070377	0.029179663	4.586454732	4.51E-06	5130
family.Oxalobacteraceae.id.2966	rs12509763	4	84254259	C	G	-0.163319668	0.036082292	-4.463890359	8.05E-06	5129
family.Oxalobacteraceae.id.2966	rs561239	12	18793583	A	G	0.10552502	0.023676874	4.487872356	7.19E-06	4989
family.Oxalobacteraceae.id.2966	rs62435498	7	1794263	C	A	0.181521209	0.040060813	4.480171538	7.46E-06	4780
family.Oxalobacteraceae.id.2966	rs80330081	4	67427392	A	C	-0.187559843	0.042368782	-4.504914205	6.64E-06	4672
family.Pasteurellaceae.id.3689	rs12191680	6	129224488	C	G	0.102213615	0.019718945	5.120392596	3.05E-07	9408
family.Pasteurellaceae.id.3689	rs9382510	6	55448491	C	T	-0.088186524	0.016996482	-5.159507554	2.48E-07	9410
family.Pasteurellaceae.id.3689	rs10840326	11	9951672	C	G	-0.0717634	0.01492293	-4.819605688	1.44E-06	9154
family.Pasteurellaceae.id.3689	rs76022354	10	94306385	C	T	0.242889667	0.050040603	4.771501022	1.83E-06	3692
family.Pasteurellaceae.id.3689	rs72756943	5	26531908	G	A	0.139879974	0.030302876	4.647970039	3.35E-06	8020
family.Pasteurellaceae.id.3689	rs78909003	9	105650242	T	C	-0.241159469	0.049837229	-4.748537868	2.05E-06	3638
family.Pasteurellaceae.id.3689	rs6092684	20	57274077	A	T	0.068464719	0.014673987	4.642595614	3.44E-06	9366
family.Pasteurellaceae.id.3689	rs4822728	22	26891808	T	C	0.068547871	0.014903232	4.576930843	4.72E-06	9412
family.Pasteurellaceae.id.3689	rs35510	12	115491973	A	G	0.122729003	0.026495751	4.610565017	4.02E-06	8671
family.Pasteurellaceae.id.3689	rs10965428	9	22718481	C	A	-0.119902083	0.025821904	-4.596740955	4.29E-06	8873
family.Pasteurellaceae.id.3689	rs16970009	17	32862601	A	G	0.187381473	0.042957182	4.48430178	7.32E-06	4703
family.Pasteurellaceae.id.3689	rs12050685	15	73477482	A	G	-0.067094787	0.015238983	-4.435517722	9.19E-06	9411
family.Pasteurellaceae.id.3689	rs73139353	3	97972214	A	C	-0.222572658	0.048463428	-4.44686776	8.71E-06	4047
family.Pasteurellaceae.id.3689	rs6972479	7	116918060	A	G	-0.078186189	0.01753656	-4.472026835	7.75E-06	8616
family.Pasteurellaceae.id.3689	rs9895850	17	64535013	T	C	-0.176406461	0.041016971	-4.43793724	9.08E-06	4737
family.Pasteurellaceae.id.3689	rs111582866	16	48742489	G	A	-0.113957275	0.025640298	-4.4916826	7.07E-06	8616
family.Pasteurellaceae.id.3689	rs62568866	9	6818307	T	A	-0.118018253	0.025920615	-4.486902005	7.23E-06	8020
family.Pasteurellaceae.id.3689	rs9938097	16	84977389	T	C	-0.071038642	0.015802434	-4.459215025	8.23E-06	9402
family.Peptococcaceae.id.2024	rs35703006	8	28614217	G	T	0.081265478	0.016435533	5.028327988	4.95E-07	10715
family.Peptococcaceae.id.2024	rs4990837	8	3580757	A	G	0.091038704	0.018586948	4.781266297	1.74E-06	10718

Gut	SNP	CHR	POS	A1	A2	BETA	SE	Z	P	N
family.Peptococcaceae.id.2024	rs150600492	10	129175962	A	C	0.135773843	0.028975651	4.723951633	2.31E-06	9761
family.Peptococcaceae.id.2024	rs12634826	3	182984048	T	G	-0.073988509	0.015093124	-4.889467351	1.01E-06	10381
family.Peptococcaceae.id.2024	rs12992764	2	188895743	T	G	0.068421261	0.014113692	4.8164184	1.46E-06	10444
family.Peptococcaceae.id.2024	rs75898026	13	113561082	A	G	-0.082177853	0.017354142	-4.751203182	2.02E-06	10444
family.Peptococcaceae.id.2024	rs117452796	9	9423378	A	G	-0.257982233	0.054956896	-4.660595379	3.15E-06	3161
family.Peptococcaceae.id.2024	rs75430375	5	89252884	C	T	-0.147940821	0.031749824	-4.644531535	3.41E-06	8375
family.Peptococcaceae.id.2024	rs12144792	1	27418225	C	T	0.064399031	0.014124573	4.532737199	5.82E-06	10718
family.Peptococcaceae.id.2024	rs11787651	9	15134457	A	G	0.128888239	0.028876388	4.457560852	8.29E-06	9921
family.Peptostreptococcaceae.id.2042	rs61841503	10	17019559	G	A	0.091965839	0.016133103	5.734133655	9.80E-09	15333
family.Peptostreptococcaceae.id.2042	rs12377846	9	16786784	C	A	-0.252000426	0.051162723	-4.954208346	7.26E-07	3736
family.Peptostreptococcaceae.id.2042	rs2644627	8	26726641	C	G	-0.055850562	0.011224002	-4.980764455	6.33E-07	16059
family.Peptostreptococcaceae.id.2042	rs59987323	22	37976422	C	T	-0.053707832	0.011443008	-4.692351725	2.70E-06	15584
family.Peptostreptococcaceae.id.2042	rs9573937	13	77351196	A	G	-0.069363688	0.01429074	-4.785467938	1.71E-06	16061
family.Peptostreptococcaceae.id.2042	rs75819860	18	65324280	T	A	0.126263781	0.026838281	4.709164315	2.49E-06	11697
family.Peptostreptococcaceae.id.2042	rs117020988	7	46710977	C	T	0.182411122	0.037212625	4.886212663	1.03E-06	6831
family.Peptostreptococcaceae.id.2042	rs1520207	3	151781175	C	T	0.052565835	0.011247608	4.659779821	3.17E-06	16057
family.Peptostreptococcaceae.id.2042	rs76982728	7	44312824	T	C	0.124202832	0.026692325	4.65495101	3.24E-06	11185
family.Peptostreptococcaceae.id.2042	rs12986312	19	17414138	T	G	0.057181848	0.01259472	4.534749673	5.77E-06	15970
family.Peptostreptococcaceae.id.2042	rs10805326	4	14324623	G	A	0.0566637	0.012275984	4.609625205	4.03E-06	16061
family.Peptostreptococcaceae.id.2042	rs1467258	17	38216487	G	A	0.072583976	0.016232118	4.467929348	7.90E-06	15584
family.Peptostreptococcaceae.id.2042	rs77540684	10	14650535	T	G	0.106725055	0.024563751	4.461544017	8.14E-06	13495
family.Peptostreptococcaceae.id.2042	rs6721459	2	67852836	A	G	0.050982344	0.011197512	4.561557225	5.08E-06	16061
family.Porphyromonadaceae.id.943	rs10119172	9	2233969	C	G	-0.073732406	0.014258993	-5.275456426	1.32E-07	18022
family.Porphyromonadaceae.id.943	rs17065783	3	62035586	A	G	-0.059132994	0.012223515	-4.77539906	1.79E-06	18028
family.Porphyromonadaceae.id.943	rs6953849	7	69251692	A	G	0.071814891	0.015072427	4.712680093	2.44E-06	18028
family.Porphyromonadaceae.id.943	rs10858364	9	138076081	G	T	0.055315986	0.012084992	4.59581422	4.31E-06	18028
family.Porphyromonadaceae.id.943	rs12486680	3	191568066	A	C	0.09846386	0.021857289	4.596772432	4.29E-06	17108
family.Porphyromonadaceae.id.943	rs3111851	3	189854046	C	G	0.058189615	0.012114715	4.734591189	2.19E-06	18024
family.Porphyromonadaceae.id.943	rs7330827	13	23531802	T	C	-0.103841997	0.023735535	-4.463878768	8.05E-06	13754
family.Porphyromonadaceae.id.943	rs35961441	1	240929774	A	C	0.091525669	0.020730567	4.455393911	8.37E-06	16374
family.Porphyromonadaceae.id.943	rs10762312	10	71571863	G	A	-0.052368014	0.011881432	-4.447104636	8.70E-06	18028

Gut	SNP	CHR	POS	A1	A2	BETA	SE	Z	P	N
family.Porphyromonadaceae.id.943	rs35233670	17	63750903	T	C	-0.047357561	0.010601956	-4.467608944	7.91E-06	18024
family.Porphyromonadaceae.id.943	rs864093	4	149825977	A	C	-0.052793912	0.011749938	-4.426037205	9.60E-06	18027
family.Porphyromonadaceae.id.943	rs1980561	14	63386516	A	G	-0.048528896	0.010928377	-4.441210678	8.95E-06	18028
family.Prevotellaceae.id.960	rs9958960	18	63018983	G	A	-0.091480658	0.01736113	-5.315570589	1.06E-07	14061
family.Prevotellaceae.id.960	rs4493272	2	118910912	T	C	-0.060499212	0.011802729	-5.121999931	3.02E-07	14867
family.Prevotellaceae.id.960	rs3860225	1	111161677	A	G	0.083936026	0.016793605	5.00810629	5.50E-07	14871
family.Prevotellaceae.id.960	rs912860	14	33706908	G	A	-0.229046147	0.048312418	-4.905916588	9.30E-07	3479
family.Prevotellaceae.id.960	rs12118202	1	210681370	T	C	-0.075198589	0.014726515	-5.006518317	5.54E-07	14869
family.Prevotellaceae.id.960	rs2206482	20	9771109	T	G	-0.056910511	0.011748649	-4.839178904	1.30E-06	14808
family.Prevotellaceae.id.960	rs9586501	13	105075823	G	A	0.059268113	0.012732933	4.700923489	2.59E-06	14868
family.Prevotellaceae.id.960	rs12057990	1	99470269	C	T	0.058987846	0.013226834	4.440505078	8.97E-06	14867
family.Prevotellaceae.id.960	rs148376875	3	170126707	T	G	0.08451502	0.017916322	4.74546349	2.08E-06	14289
family.Prevotellaceae.id.960	rs4685827	3	4864877	T	C	-0.0679529	0.014540716	-4.687034591	2.77E-06	14867
family.Prevotellaceae.id.960	rs10131942	14	78643095	C	G	0.070121224	0.015832621	4.449470896	8.61E-06	14871
family.Prevotellaceae.id.960	rs34660375	5	180283881	A	G	-0.080589392	0.01782913	-4.481752124	7.40E-06	14289
family.Prevotellaceae.id.960	rs7252711	19	17460999	A	G	-0.074087571	0.016192575	-4.542243725	5.57E-06	14540
family.Prevotellaceae.id.960	rs3758087	8	23714609	T	C	0.056419924	0.012465508	4.449530706	8.61E-06	14871
family.Prevotellaceae.id.960	rs34071565	11	119712522	T	A	-0.156699931	0.034788229	-4.540899603	5.60E-06	8136
family.Prevotellaceae.id.960	rs13069367	3	71801830	A	C	-0.053976797	0.011968733	-4.481993867	7.39E-06	14860
family.Prevotellaceae.id.960	rs7975087	12	21404832	C	A	-0.060144779	0.013545406	-4.476305272	7.59E-06	14871
family.Prevotellaceae.id.960	rs2278540	3	32408916	G	A	0.055424915	0.012326469	4.453811466	8.44E-06	14847
family.Rhodospirillaceae.id.2717	rs9813022	3	13726736	A	G	-0.08424439	0.016358324	-5.155375247	2.53E-07	8498
family.Rhodospirillaceae.id.2717	rs3754624	2	225633812	C	T	0.09713892	0.020006404	4.784421581	1.71E-06	8496
family.Rhodospirillaceae.id.2717	rs55876211	3	84471266	C	T	-0.091185056	0.019639521	-4.680140386	2.87E-06	8494
family.Rhodospirillaceae.id.2717	rs76784716	2	169033340	A	G	0.136049347	0.028576745	4.812588552	1.49E-06	8498
family.Rhodospirillaceae.id.2717	rs4278423	10	2670553	T	C	0.107529262	0.023572494	4.662743914	3.12E-06	8501
family.Rhodospirillaceae.id.2717	rs1549633	5	27945645	A	C	0.099931243	0.021863395	4.577577291	4.70E-06	8010
family.Rhodospirillaceae.id.2717	rs74354280	4	134192918	C	T	-0.091189567	0.02049069	-4.503862203	6.67E-06	8501
family.Rhodospirillaceae.id.2717	rs61933850	12	73139398	G	A	0.164808335	0.036064663	4.486925838	7.23E-06	6830
family.Rhodospirillaceae.id.2717	rs7001029	8	131958403	C	T	0.117365754	0.026132422	4.550671283	5.35E-06	8044
family.Rhodospirillaceae.id.2717	rs1035406	5	119372737	G	A	-0.113540052	0.025086266	-4.531937821	5.84E-06	8483

Gut	SNP	CHR	POS	A1	A2	BETA	SE	Z	P	N
family.Rhodospirillaceae.id.2717	rs72714493	1	92206218	A	G	0.08168583	0.018079132	4.483314864	7.35E-06	8497
family.Rhodospirillaceae.id.2717	rs12977163	19	28641647	G	C	0.07309515	0.016349062	4.475425033	7.63E-06	8044
family.Rhodospirillaceae.id.2717	rs4918686	10	113501109	G	T	0.071720972	0.015601659	4.60298833	4.16E-06	8501
family.Rhodospirillaceae.id.2717	rs13336560	16	88554243	C	T	-0.070171864	0.01580613	-4.435794658	9.17E-06	8010
family.Rhodospirillaceae.id.2717	rs6679026	1	78619512	T	C	0.111983836	0.025108815	4.418326826	9.95E-06	8497
family.Rhodospirillaceae.id.2717	rs1923415	6	89268715	A	G	-0.099923761	0.022732585	-4.425161702	9.64E-06	8501
family.Rikenellaceae.id.967	rs9603208	13	38044689	G	T	0.082117307	0.015959564	5.2071129	1.92E-07	17680
family.Rikenellaceae.id.967	rs67705352	1	19790706	T	G	-0.054968793	0.01105338	-4.973497615	6.58E-07	17676
family.Rikenellaceae.id.967	rs4264350	15	71658642	T	C	-0.052639287	0.010855489	-4.832495415	1.35E-06	17668
family.Rikenellaceae.id.967	rs62532512	9	14158854	C	A	-0.050425666	0.010739924	-4.687601419	2.76E-06	17672
family.Rikenellaceae.id.967	rs67281112	7	135778919	G	C	0.063581352	0.013745123	4.631243976	3.63E-06	17199
family.Rikenellaceae.id.967	rs6837275	4	187842232	A	G	0.057035488	0.011886994	4.818419461	1.45E-06	17199
family.Rikenellaceae.id.967	rs9578457	13	22867356	G	A	-0.141478496	0.031584793	-4.611669087	3.99E-06	9400
family.Rikenellaceae.id.967	rs2833282	21	32496710	G	A	0.071113715	0.015696614	4.595793459	4.31E-06	17680
family.Rikenellaceae.id.967	rs77885767	14	21548634	C	T	-0.156209293	0.033648181	-4.681564046	2.85E-06	8478
family.Rikenellaceae.id.967	rs8130320	21	40580258	A	G	-0.048923962	0.010684335	-4.576533186	4.73E-06	17680
family.Rikenellaceae.id.967	rs74474130	14	90270821	T	G	0.137747171	0.029568753	4.632441802	3.61E-06	11055
family.Rikenellaceae.id.967	rs7242694	18	10338470	C	T	-0.061867432	0.013488231	-4.533293834	5.81E-06	17199
family.Rikenellaceae.id.967	rs36021379	21	28515912	A	G	-0.065600501	0.014490275	-4.487830875	7.20E-06	17199
family.Rikenellaceae.id.967	rs10217435	9	86052655	C	T	-0.088375611	0.019750648	-4.509188767	6.51E-06	17679
family.Rikenellaceae.id.967	rs2447496	8	99188924	G	A	-0.054920309	0.012190433	-4.523376057	6.09E-06	17199
family.Rikenellaceae.id.967	rs35909684	8	144441740	A	C	-0.0850181	0.01915389	-4.490720707	7.10E-06	17565
family.Rikenellaceae.id.967	rs4783173	16	85396629	C	G	0.048151437	0.011000589	4.491747896	7.06E-06	17086
family.Rikenellaceae.id.967	rs7832304	8	134444155	T	G	-0.072426204	0.016062997	-4.446965356	8.71E-06	16912
family.Rikenellaceae.id.967	rs10832801	11	17589129	A	C	-0.053476324	0.012266628	-4.478862947	7.50E-06	17199
family.Rikenellaceae.id.967	rs9389714	6	100244667	C	T	-0.063684112	0.014341562	-4.444964066	8.79E-06	17680
family.Rikenellaceae.id.967	rs6744030	2	174257127	C	T	0.069674656	0.015710296	4.432489186	9.32E-06	17564
family.Ruminococcaceae.id.2050	rs55793120	12	47384118	T	C	0.138220105	0.02661521	5.259639829	1.44E-07	12405
family.Ruminococcaceae.id.2050	rs2113833	2	218216801	T	C	0.169146233	0.035500935	4.864984863	1.14E-06	4140
family.Ruminococcaceae.id.2050	rs17376049	1	61425267	T	C	0.084853361	0.017211379	4.953309112	7.30E-07	18333
family.Ruminococcaceae.id.2050	rs56199908	9	2801371	T	C	-0.199326846	0.041044434	-4.790442107	1.66E-06	5618

Gut	SNP	CHR	POS	A1	A2	BETA	SE	Z	P	N
family.Ruminococcaceae.id.2050	rs10093275	8	69732887	C	T	0.053459485	0.011676961	4.550483488	5.35E-06	18335
family.Ruminococcaceae.id.2050	rs76724913	1	24473669	T	G	0.090105292	0.020310437	4.425881039	9.60E-06	17516
family.Ruminococcaceae.id.2050	rs1612733	1	107661606	T	C	0.108708367	0.023800599	4.600027282	4.22E-06	15016
family.Ruminococcaceae.id.2050	rs2426816	20	58278102	T	A	-0.048015186	0.010601121	-4.524821729	6.04E-06	17774
family.Ruminococcaceae.id.2050	rs3009418	1	147744468	C	A	0.092760682	0.021012315	4.447433431	8.69E-06	16407
family.Ruminococcaceae.id.2050	rs10166469	2	29829167	T	C	-0.053336034	0.012020913	-4.451642278	8.52E-06	18314
family.Ruminococcaceae.id.2050	rs1158100	8	4555684	A	G	-0.049053898	0.010986476	-4.449461591	8.61E-06	18333
family.Ruminococcaceae.id.2050	rs499272	11	116485496	G	C	0.070643126	0.016045997	4.432988336	9.29E-06	17854
family.Streptococcaceae.id.1850	rs11110281	12	100584014	T	C	-0.130554059	0.022594298	-5.673221352	1.40E-08	15316
family.Streptococcaceae.id.1850	rs2952251	8	10143164	A	G	-0.063929755	0.012652464	-5.082633933	3.72E-07	16612
family.Streptococcaceae.id.1850	rs4968759	17	61298020	A	G	-0.054403465	0.011127118	-4.914106942	8.92E-07	16624
family.Streptococcaceae.id.1850	rs395407	5	169689604	G	C	-0.082685467	0.017253602	-4.835876345	1.33E-06	16088
family.Streptococcaceae.id.1850	rs35344081	16	991253	G	A	0.06093492	0.012970335	4.697128087	2.64E-06	16624
family.Streptococcaceae.id.1850	rs2370083	14	97526750	G	T	-0.084275076	0.018450934	-4.598440333	4.26E-06	16516
family.Streptococcaceae.id.1850	rs77558518	5	174173171	A	G	-0.104238822	0.022806042	-4.626334341	3.72E-06	15161
family.Streptococcaceae.id.1850	rs76717940	3	94153993	T	A	0.150605742	0.033407945	4.66478794	3.09E-06	7720
family.Streptococcaceae.id.1850	rs10028567	4	53657577	C	T	-0.093402667	0.019034346	-4.626165883	3.72E-06	16549
family.Streptococcaceae.id.1850	rs72739637	15	41663393	A	G	0.092798286	0.019202095	4.772267508	1.82E-06	15111
family.Streptococcaceae.id.1850	rs16950051	12	120729505	A	G	0.107007503	0.023697285	4.551042451	5.34E-06	14431
family.Streptococcaceae.id.1850	rs77968078	1	240441766	G	A	-0.099301329	0.022478765	-4.466973247	7.93E-06	15519
family.Streptococcaceae.id.1850	rs28718126	8	15312558	A	G	0.109069282	0.02468682	4.430296645	9.41E-06	13529
family.Streptococcaceae.id.1850	rs957755	7	46778653	T	G	-0.064244942	0.014270246	-4.481411363	7.42E-06	16627
family.Streptococcaceae.id.1850	rs6806351	3	132058723	T	C	-0.061920915	0.013574394	-4.495583982	6.94E-06	16198
family.Streptococcaceae.id.1850	rs9903102	17	80576624	C	A	-0.069301513	0.015409626	-4.568252998	4.92E-06	16623
family.Streptococcaceae.id.1850	rs6563952	16	73391982	G	C	0.080193069	0.017857592	4.447046316	8.71E-06	16627
family.unknownfamily.id.1000001214	rs9864379	3	14306949	T	C	-0.160521381	0.029275217	-5.463866794	4.66E-08	5768
family.unknownfamily.id.1000001214	rs11150282	16	80493705	T	C	0.098234998	0.019712655	4.951601318	7.36E-07	5864
family.unknownfamily.id.1000001214	rs16851659	1	230523039	G	C	-0.089516816	0.01865552	-4.844354276	1.27E-06	5875
family.unknownfamily.id.1000001214	rs4129395	9	115975389	G	A	0.090426365	0.01852532	4.852780703	1.22E-06	5878
family.unknownfamily.id.1000001214	rs1221147	9	122046549	T	A	0.126305018	0.027916132	4.570759409	4.86E-06	5759
family.unknownfamily.id.1000001214	rs79790072	15	100747683	T	C	0.226102517	0.048801659	4.636820109	3.54E-06	4104

Gut	SNP	CHR	POS	A1	A2	BETA	SE	Z	P	N
family.unknownfamily.id.1000001214	rs73074665	7	20010624	A	T	0.164631221	0.035603665	4.631868601	3.62E-06	5749
family.unknownfamily.id.1000001214	rs113884518	9	24648997	T	C	-0.205909694	0.045537561	-4.472255993	7.74E-06	4434
family.unknownfamily.id.1000001214	rs789069	18	1008278	A	C	-0.104129854	0.023445913	-4.509435534	6.50E-06	5870
family.unknownfamily.id.1000001214	rs367480	11	2937631	G	A	-0.084164921	0.018594792	-4.478294302	7.52E-06	5878
family.unknownfamily.id.1000001214	rs8028558	15	62055753	A	G	0.083459982	0.018850404	4.421929013	9.78E-06	5875
family.unknownfamily.id.1000001214	rs28678345	17	53906328	T	C	0.213082993	0.047137686	4.463596364	8.06E-06	4315
family.unknownfamily.id.1000005471	rs74603314	14	46519718	T	C	0.230767094	0.048970099	4.727196832	2.28E-06	4033
family.unknownfamily.id.1000005471	rs76373661	3	3771148	G	A	0.090821805	0.020298596	4.558199311	5.16E-06	10359
family.unknownfamily.id.1000005471	rs17235252	7	82536037	T	C	-0.12200211	0.02554629	-4.737961619	2.16E-06	9531
family.unknownfamily.id.1000005471	rs638542	1	109845252	G	A	-0.070591966	0.015704899	-4.557652462	5.17E-06	9970
family.unknownfamily.id.1000005471	rs62188991	2	177374176	G	C	-0.110864082	0.02413552	-4.553860627	5.27E-06	9531
family.unknownfamily.id.1000005471	rs949341	11	112386785	G	A	0.065660222	0.014721644	4.472450651	7.73E-06	10355
family.unknownfamily.id.1000005471	rs13100746	3	165846506	C	T	0.063897883	0.014268538	4.485085885	7.29E-06	10359
family.unknownfamily.id.1000005471	rs515984	10	3521459	T	C	-0.087545066	0.019068514	-4.505757535	6.61E-06	10359
family.unknownfamily.id.1000005471	rs7706512	5	17443654	G	A	0.065735114	0.013897929	4.727583749	2.27E-06	10359
family.unknownfamily.id.1000005471	rs11779863	8	15406830	G	A	-0.077276806	0.017217056	-4.503369443	6.69E-06	10353
family.unknownfamily.id.1000005471	rs7801843	7	4105705	A	G	-0.086949468	0.01945094	-4.428960862	9.47E-06	10359
family.unknownfamily.id.1000005471	rs10071529	5	176287195	G	C	0.125104505	0.027867407	4.448578118	8.64E-06	10147
family.unknownfamily.id.1000005471	rs739151	22	42741466	C	G	0.065265852	0.013977274	4.664554003	3.09E-06	10337
family.unknownfamily.id.1000005471	rs114184530	9	116018011	T	C	-0.1545446	0.033999183	-4.468553952	7.88E-06	7196
family.unknownfamily.id.1000005471	rs12566890	1	61850864	T	G	-0.10313363	0.024177477	-4.462206342	8.11E-06	10339
family.unknownfamily.id.1000005471	rs3932485	3	184193482	C	T	0.062620895	0.014162324	4.418723133	9.93E-06	10359
family.unknownfamily.id.1000005471	rs7853673	9	116711241	G	A	-0.062438944	0.013983251	-4.501974774	6.73E-06	10359
family.unknownfamily.id.1000006161	rs60583455	7	1321675	T	C	0.108933641	0.021176966	5.150655336	2.60E-07	4773
family.unknownfamily.id.1000006161	rs2172426	8	18229020	C	T	-0.102119829	0.019894658	-5.112795913	3.17E-07	5099
family.unknownfamily.id.1000006161	rs11251024	10	2095726	G	A	0.104201088	0.020673705	4.971957983	6.63E-07	5107
family.unknownfamily.id.1000006161	rs267959	5	10737802	A	G	0.098861186	0.020967556	4.698567115	2.62E-06	5107
family.unknownfamily.id.1000006161	rs11606187	11	91589600	A	G	-0.154539114	0.032628781	-4.65084005	3.31E-06	5102
family.unknownfamily.id.1000006161	rs7911787	10	98619568	G	T	-0.223070508	0.047023233	-4.645761783	3.39E-06	3879
family.unknownfamily.id.1000006161	rs72671304	14	39425971	T	C	0.172321792	0.037002808	4.622262246	3.80E-06	4887
family.unknownfamily.id.1000006161	rs13385922	2	234081324	T	C	0.092949422	0.020134823	4.613036483	3.97E-06	5106

Gut	SNP	CHR	POS	A1	A2	BETA	SE	Z	P	N
family.unknownfamily.id.1000006161	rs55921101	10	127094261	A	T	-0.109245446	0.023620072	-4.595294284	4.32E-06	5105
family.unknownfamily.id.1000006161	rs9542068	13	70306831	T	C	0.099070907	0.021814934	4.508674582	6.52E-06	5107
family.unknownfamily.id.1000006161	rs2930903	19	887824	C	G	0.090833483	0.020943373	4.49849862	6.84E-06	4789
family.unknownfamily.id.1000006161	rs60775321	15	97318671	T	C	-0.096242537	0.021384172	-4.49053038	7.10E-06	5101
family.unknownfamily.id.1000006161	rs8126061	20	3405239	T	C	-0.159135032	0.035190375	-4.482905922	7.36E-06	5089
family.unknownfamily.id.1000006161	rs166849	2	200778866	G	A	0.09110531	0.020223942	4.472280499	7.74E-06	5107
family.Veillonellaceae.id.2172	rs12741784	1	50088819	C	T	-0.062145234	0.011926612	-5.280981376	1.28E-07	15867
family.Veillonellaceae.id.2172	rs79535861	13	38326692	A	C	0.100666153	0.020701553	4.801239024	1.58E-06	15061
family.Veillonellaceae.id.2172	rs111810795	14	102897930	C	T	-0.08671084	0.018057347	-4.78273339	1.73E-06	15605
family.Veillonellaceae.id.2172	rs12186441	5	132646303	G	A	0.208095725	0.045399862	4.585520341	4.53E-06	5236
family.Veillonellaceae.id.2172	rs4797169	18	462180	T	C	0.058779888	0.012829669	4.587146393	4.49E-06	15974
family.Veillonellaceae.id.2172	rs12668619	7	21637810	A	G	0.055300975	0.011765373	4.702207362	2.57E-06	15971
family.Veillonellaceae.id.2172	rs2175069	4	23317124	A	G	-0.052675165	0.01148863	-4.58063885	4.64E-06	15976
family.Veillonellaceae.id.2172	rs1442060	4	46366067	G	A	-0.051402432	0.011212452	-4.586529359	4.51E-06	15976
family.Veillonellaceae.id.2172	rs1447205	8	41231597	G	C	-0.052068875	0.011565567	-4.483321953	7.35E-06	15971
family.Veillonellaceae.id.2172	rs114889439	13	61597625	A	G	-0.253809201	0.053916917	-4.519783834	6.19E-06	3268
family.Veillonellaceae.id.2172	rs4461038	15	78546996	A	G	-0.055496494	0.011946736	-4.625623216	3.73E-06	15973
family.Veillonellaceae.id.2172	rs6909981	6	75534152	C	T	-0.063869651	0.014176514	-4.545666143	5.48E-06	15976
family.Veillonellaceae.id.2172	rs2561116	5	38348199	T	G	-0.083616969	0.018715906	-4.468277935	7.89E-06	15976
family.Veillonellaceae.id.2172	rs61264131	6	139188534	A	C	0.202366989	0.046438799	4.501566559	6.75E-06	3075
family.Veillonellaceae.id.2172	rs9345168	6	92380922	C	A	0.050910225	0.011325295	4.452468603	8.49E-06	15976
family.Veillonellaceae.id.2172	rs6692542	1	244391618	A	G	0.053439722	0.011825371	4.447692921	8.68E-06	15976
family.Veillonellaceae.id.2172	rs2585520	13	78785141	G	T	-0.090497192	0.020042803	-4.553772926	5.27E-06	15501
family.Veillonellaceae.id.2172	rs75768969	13	101617913	C	G	-0.070377296	0.015685477	-4.487915952	7.19E-06	15975
family.Veillonellaceae.id.2172	rs1693340	18	30208691	T	C	0.081893428	0.018245018	4.433938453	9.25E-06	15058
family.Veillonellaceae.id.2172	rs4263802	8	138319508	G	A	0.050941954	0.011492112	4.480408497	7.45E-06	15976
family.Verrucomicrobiaceae.id.4036	rs11729256	4	95027272	T	C	0.074979802	0.015017677	4.968971477	6.73E-07	11861
family.Verrucomicrobiaceae.id.4036	rs9349825	6	56341481	A	G	-0.070435405	0.014712796	-4.707082462	2.51E-06	11858
family.Verrucomicrobiaceae.id.4036	rs74542928	4	100544188	T	C	0.112123956	0.023642178	4.792624866	1.65E-06	11216
family.Verrucomicrobiaceae.id.4036	rs3995795	10	11389614	C	T	0.064217981	0.0140994	4.563728335	5.03E-06	11690
family.Verrucomicrobiaceae.id.4036	rs2602429	16	81063149	C	T	0.074551564	0.015619484	4.692460195	2.70E-06	11690

Gut	SNP	CHR	POS	A1	A2	BETA	SE	Z	P	N
family.Verrucomicrobiaceae.id.4036	rs4242783	10	5064327	G	A	0.068799208	0.01476936	4.688329533	2.75E-06	11690
family.Verrucomicrobiaceae.id.4036	rs11184341	1	105422565	G	C	0.065530075	0.014223048	4.604305769	4.14E-06	11864
family.Verrucomicrobiaceae.id.4036	rs61779207	1	41074472	G	A	-0.075881119	0.01677619	-4.505224153	6.63E-06	11591
family.Verrucomicrobiaceae.id.4036	rs117107102	18	49473635	A	G	0.20468288	0.043157251	4.676493858	2.92E-06	4975
family.Verrucomicrobiaceae.id.4036	rs12908520	15	97570657	G	A	0.061910394	0.013094609	4.738657785	2.15E-06	11863
family.Verrucomicrobiaceae.id.4036	rs111862613	12	130309670	T	C	0.090706497	0.01967463	4.625815629	3.73E-06	11621
family.Verrucomicrobiaceae.id.4036	rs941682	20	31867840	G	A	-0.06315257	0.014376725	-4.426416617	9.58E-06	11809
family.Victivallaceae.id.2255	rs4396289	11	9316730	C	T	-0.152917575	0.028852532	-5.24797237	1.54E-07	3913
family.Victivallaceae.id.2255	rs2944282	7	57589803	T	C	-0.124196623	0.025702577	-4.802622103	1.57E-06	3555
family.Victivallaceae.id.2255	rs11764871	7	146808977	G	T	0.12709902	0.025655983	4.948132452	7.49E-07	3909
family.Victivallaceae.id.2255	rs6545794	2	60484909	A	G	-0.19778562	0.041049541	-4.992183606	5.97E-07	3916
family.Victivallaceae.id.2255	rs2546105	5	94692439	A	T	0.126643495	0.025711059	4.886775112	1.03E-06	3917
family.Victivallaceae.id.2255	rs62570196	9	111086170	C	T	-0.246101492	0.048307716	-5.143370873	2.70E-07	3091
family.Victivallaceae.id.2255	rs7077363	10	95286954	G	A	0.14894216	0.031836622	4.682515368	2.83E-06	3912
family.Victivallaceae.id.2255	rs67832247	2	2649472	C	T	-0.123489124	0.026669507	-4.655114382	3.24E-06	3555
family.Victivallaceae.id.2255	rs34962571	12	131103256	A	C	-0.186945459	0.04184118	-4.517810525	6.25E-06	3237
family.Victivallaceae.id.2255	rs7860510	9	94128796	G	C	0.164855226	0.037512763	4.425972063	9.60E-06	3912
family.Victivallaceae.id.2255	rs7627405	3	9968555	C	T	-0.134158069	0.030142747	-4.460046267	8.19E-06	3555
family.Victivallaceae.id.2255	rs61702987	2	31211657	T	C	0.145501293	0.030000733	4.665464203	3.08E-06	3906
family.Victivallaceae.id.2255	rs7314815	12	102525047	G	A	0.101087856	0.02255936	4.512696519	6.40E-06	3912
family.Victivallaceae.id.2255	rs11671100	19	711637	A	C	-0.160034432	0.034947236	-4.607243528	4.08E-06	3331
genus..Clostridiuminnocuumgroup.id.14397	rs6890185	5	71186626	T	C	0.113423668	0.023313729	4.868859021	1.12E-06	4166
genus..Clostridiuminnocuumgroup.id.14397	rs61267978	18	6348227	T	C	0.14707953	0.032087502	4.54151557	5.59E-06	4165
genus..Clostridiuminnocuumgroup.id.14397	rs1948423	3	103025517	T	A	-0.108859115	0.023424976	-4.639407488	3.49E-06	4165
genus..Clostridiuminnocuumgroup.id.14397	rs6577484	1	8419420	G	A	0.16042475	0.036085736	4.454573373	8.41E-06	3858
genus..Clostridiuminnocuumgroup.id.14397	rs10074000	5	113126539	T	C	-0.102648064	0.022750829	-4.493705865	7.00E-06	4048
genus..Clostridiuminnocuumgroup.id.14397	rs1942371	18	69274308	G	A	-0.15793795	0.034186957	-4.608114243	4.06E-06	3971
genus..Clostridiuminnocuumgroup.id.14397	rs40656	5	9368158	C	T	0.142664382	0.031102116	4.449296474	8.62E-06	4048
genus..Clostridiuminnocuumgroup.id.14397	rs71564433	6	143718234	T	A	-0.126745947	0.027465666	-4.470598529	7.80E-06	4164
genus..Clostridiuminnocuumgroup.id.14397	rs77845139	15	59704208	A	G	-0.11499293	0.02571863	-4.454568321	8.41E-06	4048
genus..Clostridiuminnocuumgroup.id.14397	rs10506058	12	30268485	A	G	0.099704805	0.022192573	4.441716091	8.92E-06	4163

Gut	SNP	CHR	POS	A1	A2	BETA	SE	Z	P	N
genus..Clostridiuminnocuumgroup.id.14397	rs4869133	5	95717619	G	A	-0.180590537	0.040950516	-4.486374206	7.24E-06	3735
genus..Eubacteriumbrachygroup.id.11296	rs112617308	10	94185213	T	C	-0.170873361	0.036284397	-4.718121316	2.38E-06	4001
genus..Eubacteriumbrachygroup.id.11296	rs9613196	22	26922534	T	A	-0.238718472	0.052856104	-4.565297565	4.99E-06	3004
genus..Eubacteriumbrachygroup.id.11296	rs62348779	5	17460096	T	C	-0.201480569	0.043286015	-4.623059058	3.78E-06	3827
genus..Eubacteriumbrachygroup.id.11296	rs2913110	10	22892667	C	T	0.105143062	0.022941973	4.584164213	4.56E-06	4001
genus..Eubacteriumbrachygroup.id.11296	rs4862235	4	184628931	G	A	0.104806298	0.022575135	4.625806646	3.73E-06	4000
genus..Eubacteriumbrachygroup.id.11296	rs12151423	2	218237281	A	G	0.101328627	0.022732445	4.433524493	9.27E-06	3854
genus..Eubacteriumbrachygroup.id.11296	rs73199919	4	6856564	T	C	-0.236716837	0.053133485	-4.461067458	8.16E-06	3008
genus..Eubacteriumbrachygroup.id.11296	rs6591893	11	80236833	G	A	0.108225435	0.024031859	4.483483276	7.34E-06	3999
genus..Eubacteriumbrachygroup.id.11296	rs720439	22	48190598	A	G	-0.111927002	0.025125245	-4.492830695	7.03E-06	4001
genus..Eubacteriumbrachygroup.id.11296	rs13139592	4	144665178	T	C	-0.145996616	0.032719209	-4.466054552	7.97E-06	3842
genus..Eubacteriumbrachygroup.id.11296	rs1384962	14	23060552	A	G	0.120888267	0.026626427	4.493903753	6.99E-06	4001
genus..Eubacteriumcoprostanoligenesgroup.id.11375	rs17159861	7	31085162	C	T	0.096222758	0.016838828	5.724420667	1.04E-08	17380
genus..Eubacteriumcoprostanoligenesgroup.id.11375	rs9648214	7	16380806	T	C	-0.082871942	0.016430987	-5.156186511	2.52E-07	17379
genus..Eubacteriumcoprostanoligenesgroup.id.11375	rs115325767	1	200638489	G	C	0.078332188	0.017471885	4.698408001	2.62E-06	16555
genus..Eubacteriumcoprostanoligenesgroup.id.11375	rs12906958	15	36911598	C	T	-0.053315925	0.011593477	-4.594012738	4.35E-06	17380
genus..Eubacteriumcoprostanoligenesgroup.id.11375	rs4076415	15	86440997	T	G	0.051515202	0.011029435	4.754464074	1.99E-06	16960
genus..Eubacteriumcoprostanoligenesgroup.id.11375	rs6762473	3	127139574	C	A	0.052158365	0.011235251	4.598221477	4.26E-06	17377
genus..Eubacteriumcoprostanoligenesgroup.id.11375	rs73109678	7	33603122	G	A	-0.079695599	0.017227516	-4.629619009	3.66E-06	17379
genus..Eubacteriumcoprostanoligenesgroup.id.11375	rs2644213	10	84506096	G	A	0.053884526	0.012124458	4.420300421	9.86E-06	17380
genus..Eubacteriumcoprostanoligenesgroup.id.11375	rs11720857	3	113794507	C	T	0.063077578	0.014448482	4.433709278	9.26E-06	16960
genus..Eubacteriumcoprostanoligenesgroup.id.11375	rs4717831	7	73306506	A	T	0.078678267	0.017403801	4.435703922	9.18E-06	16854
genus..Eubacteriumcoprostanoligenesgroup.id.11375	rs62024432	15	97869293	C	T	-0.076969763	0.017210135	-4.478922549	7.50E-06	17271
genus..Eubacteriumcoprostanoligenesgroup.id.11375	rs76898927	3	81595477	G	A	0.12305502	0.026637491	4.573790331	4.79E-06	12197
genus..Eubacteriumcoprostanoligenesgroup.id.11375	rs11052069	12	32713919	T	C	0.047783175	0.01078333	4.431005005	9.38E-06	17380
genus..Eubacteriumcoprostanoligenesgroup.id.11375	rs79895140	2	50797401	T	C	-0.06410167	0.014115288	-4.449222773	8.62E-06	16139
genus..Eubacteriumeligensgroup.id.14372	rs4583233	16	81820486	A	C	0.067020477	0.012812178	5.133864886	2.84E-07	14327
genus..Eubacteriumeligensgroup.id.14372	rs72839198	2	115197171	C	G	0.155658995	0.037561222	4.661499621	3.14E-06	4926
genus..Eubacteriumeligensgroup.id.14372	rs74606150	14	39301409	C	G	-0.196453545	0.042564951	-4.599008564	4.25E-06	5381
genus..Eubacteriumeligensgroup.id.14372	rs6923695	6	70956815	T	G	0.103267603	0.02295245	4.570136641	4.87E-06	12891
genus..Eubacteriumeligensgroup.id.14372	rs56080211	2	110260993	C	T	0.123092697	0.028274315	4.436510219	9.14E-06	10699

Gut	SNP	CHR	POS	A1	A2	BETA	SE	Z	P	N
genus..Eubacteriumeligensgroup.id.14372	rs158115	14	77619186	G	C	0.091527053	0.019061087	4.422825417	9.74E-06	14678
genus..Eubacteriumeligensgroup.id.14372	rs2200429	13	107563458	A	G	-0.088882459	0.019847898	-4.552659091	5.30E-06	14761
genus..Eubacteriumeligensgroup.id.14372	rs182318	11	31098691	G	A	-0.082494113	0.019561497	-4.454768688	8.40E-06	14777
genus..Eubacteriumeligensgroup.id.14372	rs12719051	7	51365152	A	G	0.091662637	0.020818348	4.490139295	7.12E-06	12799
genus..Eubacteriumfissicatenagroup.id.14373	rs3771393	2	71145246	C	T	0.130842344	0.026667104	4.951111172	7.38E-07	3792
genus..Eubacteriumfissicatenagroup.id.14373	rs2733072	8	5433699	G	A	0.109644168	0.022830587	4.812452632	1.49E-06	3833
genus..Eubacteriumfissicatenagroup.id.14373	rs7104872	11	115165111	G	A	0.138611743	0.029190553	4.690040941	2.73E-06	3829
genus..Eubacteriumfissicatenagroup.id.14373	rs151257695	7	73043561	A	G	0.209509779	0.045484723	4.664292313	3.10E-06	3587
genus..Eubacteriumfissicatenagroup.id.14373	rs11876297	18	45753272	T	C	0.131469398	0.028171203	4.695038571	2.67E-06	3396
genus..Eubacteriumfissicatenagroup.id.14373	rs6934739	6	39940437	A	G	0.111463384	0.025278805	4.42257244	9.75E-06	3831
genus..Eubacteriumfissicatenagroup.id.14373	rs10147907	14	89484373	T	G	0.172262807	0.039600983	4.458008645	8.27E-06	3833
genus..Eubacteriumfissicatenagroup.id.14373	rs1768152	3	39604954	T	C	0.139488571	0.031618584	4.447178856	8.70E-06	3590
genus..Eubacteriumfissicatenagroup.id.14373	rs11818408	10	96758467	G	A	0.10585035	0.023711404	4.459890532	8.20E-06	3590
genus..Eubacteriumhalliigroup.id.11338	rs13116360	4	111885431	T	C	0.154124445	0.029718533	5.126941594	2.94E-07	10323
genus..Eubacteriumhalliigroup.id.11338	rs10953312	7	100635591	C	A	-0.055637169	0.011141731	-4.988067949	6.10E-07	16130
genus..Eubacteriumhalliigroup.id.11338	rs10798999	1	34308917	C	T	0.060166074	0.012675245	4.699689081	2.61E-06	16561
genus..Eubacteriumhalliigroup.id.11338	rs60254196	7	148856720	A	G	-0.052283163	0.011186491	-4.692662693	2.70E-06	16238
genus..Eubacteriumhalliigroup.id.11338	rs10808115	7	100635375	A	C	-0.050474741	0.010992248	-4.590375889	4.42E-06	16566
genus..Eubacteriumhalliigroup.id.11338	rs74018587	15	62014160	C	T	0.208942799	0.043821974	4.627415464	3.70E-06	3039
genus..Eubacteriumhalliigroup.id.11338	rs6550770	3	23663416	T	C	-0.198087408	0.044354487	-4.572343054	4.82E-06	4279
genus..Eubacteriumhalliigroup.id.11338	rs10501370	11	58040621	C	T	-0.115588894	0.025248889	-4.547868998	5.42E-06	13824
genus..Eubacteriumhalliigroup.id.11338	rs78056098	11	123789877	G	T	-0.050741114	0.011375822	-4.457601602	8.29E-06	16568
genus..Eubacteriumhalliigroup.id.11338	rs138531890	3	195238086	A	G	0.153138471	0.034945641	4.547426158	5.43E-06	7710
genus..Eubacteriumhalliigroup.id.11338	rs368871882	1	37546787	C	T	0.232067304	0.05161821	4.459070104	8.23E-06	3039
genus..Eubacteriumhalliigroup.id.11338	rs28584818	3	64664456	A	G	0.126115364	0.026862747	4.590005289	4.43E-06	11095
genus..Eubacteriumhalliigroup.id.11338	rs117748144	11	11771637	T	C	-0.126581958	0.028711955	-4.468961756	7.86E-06	10861
genus..Eubacteriumhalliigroup.id.11338	rs17474256	1	104524676	G	A	0.081080509	0.018457309	4.429288401	9.45E-06	16460
genus..Eubacteriumhalliigroup.id.11338	rs630939	18	48384463	C	T	-0.050891423	0.011435211	-4.436164521	9.16E-06	16238
genus..Eubacteriumhalliigroup.id.11338	rs17074066	4	183709536	T	C	-0.081398624	0.018927232	-4.431705905	9.35E-06	15804
genus..Eubacteriumnodatumgroup.id.11297	rs34297067	14	52185888	A	G	-0.186906548	0.034147492	-5.401792191	6.60E-08	3271
genus..Eubacteriumnodatumgroup.id.11297	rs113893692	9	28245518	C	T	-0.18510716	0.040357325	-4.535122175	5.76E-06	3195

Gut	SNP	CHR	POS	A1	A2	BETA	SE	Z	P	N
genus..Eubacteriumnodatumgroup.id.11297	rs77910827	9	96597985	C	T	0.201788441	0.041356826	4.911162256	9.05E-07	3271
genus..Eubacteriumnodatumgroup.id.11297	rs61841040	10	9659749	G	T	0.160621141	0.034160929	4.635462131	3.56E-06	3295
genus..Eubacteriumnodatumgroup.id.11297	rs9425984	1	34529736	T	C	-0.130233832	0.029234653	-4.487487438	7.21E-06	3294
genus..Eubacteriumnodatumgroup.id.11297	rs10263623	7	64894137	C	T	0.193495904	0.043902338	4.442134987	8.91E-06	3080
genus..Eubacteriumnodatumgroup.id.11297	rs11006576	10	61322015	A	G	-0.110167094	0.024592963	-4.465541778	7.99E-06	3295
genus..Eubacteriumnodatumgroup.id.11297	rs7880204	1	171060821	T	C	-0.125453808	0.027530312	-4.498643459	6.84E-06	3294
genus..Eubacteriumnodatumgroup.id.11297	rs6818880	4	94712747	A	G	-0.110080596	0.024596445	-4.469905895	7.83E-06	3291
genus..Eubacteriumnodatumgroup.id.11297	rs10458299	7	135052348	T	C	-0.187752781	0.041969011	-4.455567831	8.37E-06	3271
genus..Eubacteriumnodatumgroup.id.11297	rs7827125	8	4954912	C	T	0.122299815	0.027133143	4.48847314	7.17E-06	3294
genus..Eubacteriumoxidoreducensgroup.id.11339	rs12423772	12	94515340	G	T	0.140979641	0.029502889	4.697841471	2.63E-06	5321
genus..Eubacteriumoxidoreducensgroup.id.11339	rs2973294	4	37526679	G	T	0.092355871	0.019543871	4.717435208	2.39E-06	5378
genus..Eubacteriumoxidoreducensgroup.id.11339	rs34561138	16	86480355	G	A	0.216145345	0.04599197	4.707183597	2.51E-06	4186
genus..Eubacteriumoxidoreducensgroup.id.11339	rs440215	5	106882394	C	T	0.093276592	0.019529391	4.791659801	1.65E-06	5377
genus..Eubacteriumoxidoreducensgroup.id.11339	rs1425962	4	187905399	G	C	0.090610394	0.020061232	4.484082152	7.32E-06	5321
genus..Eubacteriumoxidoreducensgroup.id.11339	rs12129908	1	195064019	C	A	0.08932725	0.019833287	4.533728175	5.80E-06	5377
genus..Eubacteriumrectalegroup.id.14374	rs35398954	15	93006765	A	G	-0.090140156	0.017460171	-5.011545752	5.40E-07	16621
genus..Eubacteriumrectalegroup.id.14374	rs117151453	12	20066025	G	C	-0.113140609	0.02435414	-4.769774815	1.84E-06	13908
genus..Eubacteriumrectalegroup.id.14374	rs16960159	18	37703874	C	G	-0.156595964	0.03362338	-4.606180964	4.10E-06	8334
genus..Eubacteriumrectalegroup.id.14374	rs314726	2	125553155	T	C	0.052882354	0.010948624	4.827919729	1.38E-06	16966
genus..Eubacteriumrectalegroup.id.14374	rs10797540	1	234530370	A	G	0.050317907	0.010838946	4.637069314	3.53E-06	17350
genus..Eubacteriumrectalegroup.id.14374	rs10248854	7	121281238	C	A	-0.052782677	0.01134635	-4.600896783	4.21E-06	17348
genus..Eubacteriumrectalegroup.id.14374	rs3980709	1	170900221	A	T	-0.062270145	0.014057159	-4.49813442	6.86E-06	17340
genus..Eubacteriumrectalegroup.id.14374	rs2884897	11	8129537	A	G	-0.129363699	0.028902068	-4.511263623	6.44E-06	10813
genus..Eubacteriumrectalegroup.id.14374	rs10892089	11	98523638	C	G	-0.063552174	0.014007233	-4.518830651	6.22E-06	17351
genus..Eubacteriumrectalegroup.id.14374	rs143694765	1	116082909	T	C	0.087002523	0.01977896	4.42256305	9.75E-06	15124
genus..Eubacteriumrectalegroup.id.14374	rs59427698	9	129425745	A	G	-0.057603509	0.013085337	-4.549824965	5.37E-06	16613
genus..Eubacteriumrectalegroup.id.14374	rs62547233	9	92013419	A	G	0.0536319	0.012020063	4.41938534	9.90E-06	17347
genus..Eubacteriumrectalegroup.id.14374	rs58498416	15	87881513	C	T	-0.051731578	0.011819644	-4.4241071	9.68E-06	17350
genus..Eubacteriumruminantiumgroup.id.11340	rs2116427	5	121182996	A	G	0.091146051	0.018235307	5.039248033	4.67E-07	7739
genus..Eubacteriumruminantiumgroup.id.11340	rs139749	22	25303055	C	T	-0.084543927	0.017179078	-4.921497503	8.59E-07	7314
genus..Eubacteriumruminantiumgroup.id.11340	rs72836424	10	129037453	C	T	-0.139824589	0.030068963	-4.698463969	2.62E-06	7314

Gut	SNP	CHR	POS	A1	A2	BETA	SE	Z	P	N
genus..Eubacteriumruminantiumgroup.id.11340	rs2229917	9	130980937	A	G	0.153537929	0.032392215	4.737947558	2.16E-06	7050
genus..Eubacteriumruminantiumgroup.id.11340	rs10131724	14	52219066	A	C	-0.199832279	0.041457713	-4.717582999	2.39E-06	5071
genus..Eubacteriumruminantiumgroup.id.11340	rs16891896	5	33897589	G	A	-0.174787011	0.039057268	-4.718046237	2.38E-06	4753
genus..Eubacteriumruminantiumgroup.id.11340	rs7000472	8	138626268	A	G	-0.076228208	0.016523022	-4.607853301	4.07E-06	7739
genus..Eubacteriumruminantiumgroup.id.11340	rs13025464	2	201610645	T	C	-0.073707715	0.016378577	-4.494650667	6.97E-06	7738
genus..Eubacteriumruminantiumgroup.id.11340	rs2418654	2	71247578	C	T	-0.074887891	0.016585161	-4.520393734	6.17E-06	7314
genus..Eubacteriumruminantiumgroup.id.11340	rs6676699	1	197978281	G	T	-0.088812364	0.019644717	-4.513395422	6.38E-06	7708
genus..Eubacteriumruminantiumgroup.id.11340	rs73139629	12	63306003	A	C	-0.115097848	0.024790941	-4.550102007	5.36E-06	7314
genus..Eubacteriumruminantiumgroup.id.11340	rs112375806	22	48749548	T	A	0.143141085	0.029375749	4.532764979	5.82E-06	7123
genus..Eubacteriumruminantiumgroup.id.11340	rs57340348	6	130030114	T	C	-0.097942933	0.021216595	-4.567626347	4.93E-06	7314
genus..Eubacteriumruminantiumgroup.id.11340	rs2817174	1	3044181	C	T	-0.073430579	0.016368685	-4.468701233	7.87E-06	7710
genus..Eubacteriumruminantiumgroup.id.11340	rs10923018	1	88523399	G	A	0.072643786	0.016092406	4.499998031	6.80E-06	7739
genus..Eubacteriumruminantiumgroup.id.11340	rs11637981	15	61289622	G	T	-0.073257996	0.016088796	-4.5471891	5.44E-06	7718
genus..Eubacteriumruminantiumgroup.id.11340	rs17519472	12	30360954	C	T	0.10780414	0.023398378	4.577593479	4.70E-06	7710
genus..Eubacteriumruminantiumgroup.id.11340	rs209813	6	11953633	G	A	-0.103488116	0.023639057	-4.434433815	9.23E-06	7264
genus..Eubacteriumventriosumgroup.id.11341	rs73615400	20	50140919	T	C	-0.095630317	0.01934276	-4.900976789	9.54E-07	15146
genus..Eubacteriumventriosumgroup.id.11341	rs57199565	3	190223732	T	C	0.078339435	0.01602571	4.936091663	7.97E-07	14913
genus..Eubacteriumventriosumgroup.id.11341	rs11617697	13	98785900	A	G	-0.143348528	0.028624073	-4.955416123	7.22E-07	11497
genus..Eubacteriumventriosumgroup.id.11341	rs16884680	8	114445055	G	T	-0.090642843	0.019190901	-4.781526079	1.74E-06	15289
genus..Eubacteriumventriosumgroup.id.11341	rs12964517	18	22460785	G	A	0.058719806	0.012345332	4.745980462	2.07E-06	15289
genus..Eubacteriumventriosumgroup.id.11341	rs876734	12	52423996	C	T	-0.061869648	0.013253231	-4.678552198	2.89E-06	14913
genus..Eubacteriumventriosumgroup.id.11341	rs3809430	14	44975510	T	C	-0.054817114	0.011842566	-4.635941214	3.55E-06	15287
genus..Eubacteriumventriosumgroup.id.11341	rs72783037	16	60348381	C	A	0.065892572	0.014374684	4.507887733	6.55E-06	15286
genus..Eubacteriumventriosumgroup.id.11341	rs73849225	4	126371814	T	C	0.097571148	0.022438827	4.556185846	5.21E-06	14573
genus..Eubacteriumventriosumgroup.id.11341	rs9316536	13	51938576	T	G	-0.081711077	0.018312095	-4.469638479	7.84E-06	15289
genus..Eubacteriumventriosumgroup.id.11341	rs35179274	14	51960625	C	T	-0.062726329	0.013785975	-4.535088949	5.76E-06	15287
genus..Eubacteriumventriosumgroup.id.11341	rs78250280	1	147291928	G	A	0.074976242	0.016440448	4.647617394	3.36E-06	14835
genus..Eubacteriumventriosumgroup.id.11341	rs66746423	1	109398264	C	T	0.0751501	0.016497843	4.522497503	6.11E-06	14578
genus..Eubacteriumventriosumgroup.id.11341	rs6704822	2	203661779	A	G	0.073917922	0.016670495	4.505480289	6.62E-06	15289
genus..Eubacteriumventriosumgroup.id.11341	rs13082419	3	107736434	C	T	-0.071552205	0.016136969	-4.426896244	9.56E-06	14501
genus..Eubacteriumventriosumgroup.id.11341	rs66830358	3	171895485	T	A	0.052875209	0.011797227	4.497702599	6.87E-06	14913

Gut	SNP	CHR	POS	A1	A2	BETA	SE	Z	P	N
genus..Eubacteriumxylanophilumgroup.id.14375	rs17830032	20	57995506	G	A	-0.160597431	0.031051453	-5.166206568	2.39E-07	9527
genus..Eubacteriumxylanophilumgroup.id.14375	rs13239072	7	43566344	G	A	0.068742734	0.014272832	4.772688795	1.82E-06	13307
genus..Eubacteriumxylanophilumgroup.id.14375	rs79582700	17	66689958	C	G	-0.095167651	0.019996897	-4.715615498	2.41E-06	13256
genus..Eubacteriumxylanophilumgroup.id.14375	rs12980122	19	8255478	C	G	-0.108273525	0.023825715	-4.563977213	5.02E-06	12039
genus..Eubacteriumxylanophilumgroup.id.14375	rs2213117	11	131009660	T	G	0.087779736	0.018901228	4.600573821	4.21E-06	12878
genus..Eubacteriumxylanophilumgroup.id.14375	rs10140184	14	73181783	A	C	0.057679453	0.01259899	4.566530713	4.96E-06	12930
genus..Eubacteriumxylanophilumgroup.id.14375	rs10917203	1	22627266	A	C	0.061263327	0.013091688	4.660687614	3.15E-06	12930
genus..Eubacteriumxylanophilumgroup.id.14375	rs112176119	16	73696771	C	T	-0.113455468	0.024576196	-4.64948435	3.33E-06	12199
genus..Eubacteriumxylanophilumgroup.id.14375	rs1999224	9	130498120	G	T	-0.094905143	0.020350281	-4.624506379	3.75E-06	13309
genus..Eubacteriumxylanophilumgroup.id.14375	rs75586835	4	87891938	A	G	-0.114497555	0.026345472	-4.430830574	9.39E-06	12550
genus..Eubacteriumxylanophilumgroup.id.14375	rs4654122	1	246780557	C	G	0.055350808	0.012358794	4.487804417	7.20E-06	13309
genus..Ruminococcusgauvreauiigroup.id.11342	rs2047242	10	29201860	A	G	-0.06760301	0.013373773	-5.160438457	2.46E-07	13369
genus..Ruminococcusgauvreauiigroup.id.11342	rs71386687	16	2767894	T	G	0.121036908	0.02385996	5.129339327	2.91E-07	11340
genus..Ruminococcusgauvreauiigroup.id.11342	rs9870933	3	112372317	A	G	0.062164279	0.012607163	4.923725375	8.49E-07	13382
genus..Ruminococcusgauvreauiigroup.id.11342	rs1391597	12	61119266	C	T	0.059028381	0.012487351	4.768107754	1.86E-06	13382
genus..Ruminococcusgauvreauiigroup.id.11342	rs431418	5	166419552	A	G	-0.094737043	0.02101571	-4.543351878	5.54E-06	13289
genus..Ruminococcusgauvreauiigroup.id.11342	rs10931481	2	191954852	G	A	0.061009638	0.013048446	4.646135256	3.38E-06	13382
genus..Ruminococcusgauvreauiigroup.id.11342	rs289410	15	85563483	G	A	-0.065492577	0.013910104	-4.727354811	2.27E-06	13380
genus..Ruminococcusgauvreauiigroup.id.11342	rs2105937	1	20552072	A	G	0.058021543	0.012780587	4.560486614	5.10E-06	13364
genus..Ruminococcusgauvreauiigroup.id.11342	rs2166943	8	137090850	A	C	0.056697379	0.012349673	4.553455861	5.28E-06	13377
genus..Ruminococcusgauvreauiigroup.id.11342	rs12539819	7	153487396	C	T	0.110653536	0.02406836	4.58713285	4.49E-06	11860
genus..Ruminococcusgauvreauiigroup.id.11342	rs12079579	1	161880482	A	G	0.095532379	0.021343619	4.56323037	5.04E-06	12986
genus..Ruminococcusgauvreauiigroup.id.11342	rs73802842	4	18824860	C	A	0.07368081	0.016966478	4.479544294	7.48E-06	13382
genus..Ruminococcusgauvreauiigroup.id.11342	rs13188803	5	112627773	T	A	0.070941873	0.015685519	4.485462741	7.28E-06	12834
genus..Ruminococcusgnavusgroup.id.14376	rs13163520	5	18662686	G	A	-0.127377481	0.023388325	-5.43080156	5.61E-08	6326
genus..Ruminococcusgnavusgroup.id.14376	rs934940	2	122096018	A	C	-0.105044905	0.022958834	-4.689625966	2.74E-06	6309
genus..Ruminococcusgnavusgroup.id.14376	rs9872758	3	66701616	T	C	0.084923254	0.017664313	4.79090666	1.66E-06	6398
genus..Ruminococcusgnavusgroup.id.14376	rs2909242	8	129224680	C	A	-0.090996476	0.018351012	-4.95030916	7.41E-07	6401
genus..Ruminococcusgnavusgroup.id.14376	rs3124783	9	135838591	A	G	-0.115989721	0.02490991	-4.695066836	2.67E-06	6401
genus..Ruminococcusgnavusgroup.id.14376	rs62167033	2	144700007	T	C	0.185289033	0.039629529	4.63933708	3.50E-06	5194
genus..Ruminococcusgnavusgroup.id.14376	rs12989336	2	36259341	G	A	-0.084686061	0.01879361	-4.490126654	7.12E-06	6401

Gut	SNP	CHR	POS	A1	A2	BETA	SE	Z	P	N
genus..Ruminococcusgnavusgroup.id.14376	rs12136548	1	115168097	C	T	0.090194566	0.019647574	4.664325381	3.10E-06	6401
genus..Ruminococcusgnavusgroup.id.14376	rs78399089	3	146290310	T	C	0.144444798	0.032661769	4.505234195	6.63E-06	5944
genus..Ruminococcusgnavusgroup.id.14376	rs11597105	10	6512581	A	G	0.114672834	0.025065269	4.495254423	6.95E-06	6328
genus..Ruminococcusgnavusgroup.id.14376	rs11864644	16	433078	T	C	-0.139803729	0.031825335	-4.564418654	5.01E-06	6241
genus..Ruminococcusgnavusgroup.id.14376	rs4388134	4	190110710	C	T	-0.090499668	0.020354415	-4.437136001	9.12E-06	6399
genus..Ruminococcustorquesgroup.id.14377	rs773123	12	56494998	T	A	0.082400465	0.017386159	4.799634361	1.59E-06	17011
genus..Ruminococcustorquesgroup.id.14377	rs8103840	19	49254955	C	T	-0.051946898	0.010752535	-4.842237952	1.28E-06	17353
genus..Ruminococcustorquesgroup.id.14377	rs8080469	17	14532347	G	A	0.049072662	0.010703625	4.63934067	3.50E-06	17798
genus..Ruminococcustorquesgroup.id.14377	rs77034621	8	76769843	T	G	-0.151552842	0.033587775	-4.523980794	6.07E-06	8567
genus..Ruminococcustorquesgroup.id.14377	rs73130967	7	54781314	A	T	0.077037483	0.016826423	4.627144951	3.71E-06	17011
genus..Ruminococcustorquesgroup.id.14377	rs1972694	15	25326425	T	A	-0.061409435	0.013732055	-4.44162288	8.93E-06	17353
genus..Ruminococcustorquesgroup.id.14377	rs12434631	14	26130404	A	G	0.074696994	0.015340053	4.686994202	2.77E-06	17353
genus..Ruminococcustorquesgroup.id.14377	rs158487	5	55576026	A	G	0.053178139	0.011634088	4.478464979	7.52E-06	17798
genus..Ruminococcustorquesgroup.id.14377	rs60603763	10	3901226	C	G	0.1243283	0.027528605	4.531013081	5.87E-06	11700
genus..Ruminococcustorquesgroup.id.14377	rs13154778	5	56952803	T	A	0.056335821	0.012952644	4.488951431	7.16E-06	17795
genus..Ruminococcustorquesgroup.id.14377	rs1475330	6	67262102	T	C	0.052349242	0.011820049	4.461617093	8.13E-06	17353
genus..Ruminococcustorquesgroup.id.14377	rs10967781	9	27225258	C	A	0.050799379	0.011332229	4.455529289	8.37E-06	17791
genus..Ruminococcustorquesgroup.id.14377	rs8141465	22	21358653	A	G	0.048077756	0.010744211	4.424907227	9.65E-06	17791
genus.Actinomyces.id.423	rs34583783	6	67207371	G	T	0.126595837	0.026846063	4.587526297	4.49E-06	7468
genus.Actinomyces.id.423	rs2715439	15	99492313	C	T	0.074668399	0.016482184	4.517074144	6.27E-06	7468
genus.Actinomyces.id.423	rs4146653	10	4782841	G	A	0.098522403	0.021418242	4.587006138	4.50E-06	7468
genus.Actinomyces.id.423	rs35011108	6	133007480	A	G	0.232633598	0.051204357	4.514777775	6.34E-06	3515
genus.Actinomyces.id.423	rs4073240	6	169224781	G	A	0.074968682	0.016736817	4.466721491	7.94E-06	7468
genus.Actinomyces.id.423	rs7915461	10	127532121	T	C	0.187759792	0.040163611	4.529232083	5.92E-06	5282
genus.Actinomyces.id.423	rs71315246	3	101352439	A	G	-0.09698092	0.021925041	-4.420884208	9.83E-06	7468
genus.Actinomyces.id.423	rs10787984	10	121356829	G	C	0.094315957	0.021351309	4.425474512	9.62E-06	7468
genus.Adlercreutzia.id.812	rs2147798	1	201061818	C	G	0.092295902	0.019212124	4.82517954	1.40E-06	8243
genus.Adlercreutzia.id.812	rs80078995	5	94059857	A	T	-0.113239745	0.023258622	-4.801656596	1.57E-06	8243
genus.Adlercreutzia.id.812	rs2717140	18	75009770	C	T	-0.119225209	0.025108114	-4.748832666	2.05E-06	8243
genus.Adlercreutzia.id.812	rs9490822	6	123909083	C	T	-0.073451202	0.015578896	-4.70455471	2.54E-06	8243
genus.Adlercreutzia.id.812	rs12522517	5	31991834	A	T	-0.104884371	0.023469475	-4.590940166	4.41E-06	8187

Gut	SNP	CHR	POS	A1	A2	BETA	SE	Z	P	N
genus.Adlercreutzia.id.812	rs13231526	7	48844151	C	A	0.143237051	0.031165443	4.57275415	4.81E-06	8051
genus.Adlercreutzia.id.812	rs6664405	1	69444163	T	C	-0.095308388	0.02107517	-4.555174309	5.23E-06	8051
genus.Adlercreutzia.id.812	rs1046175	10	135204950	C	G	0.112757932	0.025599407	4.514091038	6.36E-06	8051
genus.Adlercreutzia.id.812	rs9915817	17	48864179	T	C	0.074921889	0.016833007	4.459457661	8.22E-06	8243
genus.Adlercreutzia.id.812	rs55719207	3	105355971	G	A	-0.069924275	0.015803609	-4.425878781	9.61E-06	8243
genus.Adlercreutzia.id.812	rs11604400	11	98386085	C	T	-0.102524228	0.023483368	-4.422763724	9.74E-06	8107
genus.Akkermansia.id.4037	rs11729256	4	95027272	T	C	0.075047253	0.015018395	4.973251197	6.58E-07	11860
genus.Akkermansia.id.4037	rs9349825	6	56341481	A	G	-0.070340692	0.014713291	-4.700216385	2.60E-06	11857
genus.Akkermansia.id.4037	rs74542928	4	100544188	T	C	0.112622737	0.023643438	4.813890081	1.48E-06	11215
genus.Akkermansia.id.4037	rs3995795	10	11389614	C	T	0.064117532	0.014099927	4.555768074	5.22E-06	11689
genus.Akkermansia.id.4037	rs2602429	16	81063149	C	T	0.074535241	0.015620127	4.691228767	2.72E-06	11689
genus.Akkermansia.id.4037	rs61779207	1	41074472	G	A	-0.076053856	0.01677691	-4.515352326	6.32E-06	11590
genus.Akkermansia.id.4037	rs11184341	1	105422565	G	C	0.065585479	0.01422364	4.608106095	4.06E-06	11863
genus.Akkermansia.id.4037	rs4242783	10	5064327	G	A	0.068545381	0.014770062	4.670957732	3.00E-06	11689
genus.Akkermansia.id.4037	rs117107102	18	49473635	A	G	0.204406166	0.043162894	4.670041675	3.01E-06	4974
genus.Akkermansia.id.4037	rs12908520	15	97570657	G	A	0.061772023	0.01309539	4.728233573	2.26E-06	11862
genus.Akkermansia.id.4037	rs111862613	12	130309670	T	C	0.091119928	0.019674815	4.645510965	3.39E-06	11620
genus.Akkermansia.id.4037	rs941682	20	31867840	G	A	-0.063295745	0.01437768	-4.435843075	9.17E-06	11808
genus.Alistipes.id.968	rs67281112	7	135778919	G	C	0.063457498	0.013784589	4.615803306	3.92E-06	17090
genus.Alistipes.id.968	rs67705352	1	19790706	T	G	-0.053240398	0.011086401	-4.792004949	1.65E-06	17567
genus.Alistipes.id.968	rs11769002	7	62443498	G	A	-0.052879265	0.010938851	-4.81805879	1.45E-06	17571
genus.Alistipes.id.968	rs7129639	11	17511427	C	A	-0.052495538	0.010958377	-4.776444511	1.78E-06	17571
genus.Alistipes.id.968	rs1689282	9	14080129	A	C	-0.052006142	0.011394196	-4.553511345	5.28E-06	17090
genus.Alistipes.id.968	rs11958296	5	177755179	A	G	-0.098122204	0.021827201	-4.432938561	9.30E-06	16214
genus.Alistipes.id.968	rs8130320	21	40580258	A	G	-0.049001972	0.01071701	-4.571774025	4.84E-06	17571
genus.Alistipes.id.968	rs2875322	11	130887552	T	C	-0.058094464	0.013143238	-4.445297461	8.78E-06	17459
genus.Alistipes.id.968	rs2290844	10	127787142	C	T	0.081396908	0.019163185	4.43749107	9.10E-06	17571
genus.Alistipes.id.968	rs2450745	8	144428720	A	C	-0.080523959	0.018470178	-4.490118359	7.12E-06	17571
genus.Alistipes.id.968	rs62576416	9	112120264	T	C	0.049264678	0.010973282	4.478876941	7.50E-06	17564
genus.Alistipes.id.968	rs1107244	13	38057357	G	A	0.075855776	0.017118221	4.633774119	3.59E-06	17571
genus.Alistipes.id.968	rs34417064	17	52776479	A	G	-0.04820195	0.010686843	-4.493460739	7.01E-06	17570

Gut	SNP	CHR	POS	A1	A2	BETA	SE	Z	P	N
genus.Alistipes.id.968	rs12990744	2	178000949	C	T	-0.077661791	0.017298561	-4.45970518	8.21E-06	16694
genus.Alistipes.id.968	rs4810359	20	41191335	A	G	-0.065242793	0.014615527	-4.479075853	7.50E-06	17380
genus.Allisonella.id.2174	rs602075	9	79110160	A	G	0.168974277	0.029697566	5.511111432	3.57E-08	3212
genus.Allisonella.id.2174	rs35778461	9	100150556	C	T	0.146678707	0.029718557	4.85460075	1.21E-06	3212
genus.Allisonella.id.2174	rs6742198	2	33607071	G	A	0.149151971	0.031648438	4.648403361	3.35E-06	3182
genus.Allisonella.id.2174	rs1901739	5	114286707	T	G	0.115768513	0.024862618	4.633922673	3.59E-06	3212
genus.Allisonella.id.2174	rs76904847	7	137968010	G	A	0.148523492	0.033485691	4.52332694	6.09E-06	3182
genus.Allisonella.id.2174	rs35110698	12	28018998	T	C	-0.146321972	0.032085207	-4.53636452	5.72E-06	3212
genus.Allisonella.id.2174	rs7898615	10	122413451	T	G	0.167966358	0.037358631	4.442964827	8.87E-06	3212
genus.Allisonella.id.2174	rs594561	11	88632972	C	T	0.112230607	0.025168069	4.430281151	9.41E-06	3212
genus.Alloprevotella.id.961	rs12675596	8	69994816	G	T	0.145668934	0.029067823	4.898904314	9.64E-07	3008
genus.Alloprevotella.id.961	rs58212166	4	189076705	A	G	-0.161505876	0.035910233	-4.466899561	7.94E-06	3005
genus.Alloprevotella.id.961	rs34619204	21	40993008	G	A	-0.156036945	0.034439247	-4.443714285	8.84E-06	3003
genus.Alloprevotella.id.961	rs17380632	3	79794395	A	T	0.125563764	0.028040851	4.43350798	9.27E-06	3009
genus.Alloprevotella.id.961	rs4680035	3	152805600	A	G	-0.119599445	0.025944593	-4.565186066	4.99E-06	3007
genus.Alloprevotella.id.961	rs4364940	1	234126516	A	G	0.126417748	0.028220735	4.450087512	8.58E-06	3008
genus.Alloprevotella.id.961	rs2154444	21	36053643	T	G	0.138234323	0.030896071	4.45555742	8.37E-06	3009
genus.Anaerofilum.id.2053	rs816292	12	117811409	T	C	-0.113002639	0.022040046	-5.147459273	2.64E-07	5017
genus.Anaerofilum.id.2053	rs79598899	2	191139151	C	T	0.182585804	0.035728447	5.081367144	3.75E-07	4709
genus.Anaerofilum.id.2053	rs712981	3	129686434	A	C	0.100759617	0.020290248	4.966103534	6.83E-07	5051
genus.Anaerofilum.id.2053	rs17105491	10	125294406	G	C	-0.19310744	0.04111866	-4.802344082	1.57E-06	4856
genus.Anaerofilum.id.2053	rs10794359	11	1051715	T	C	-0.095352431	0.020059979	-4.730928211	2.23E-06	4994
genus.Anaerofilum.id.2053	rs17096874	14	30991358	C	T	-0.126336895	0.02689189	-4.680604546	2.86E-06	5051
genus.Anaerofilum.id.2053	rs356049	17	6450532	G	A	0.132602907	0.028980209	4.507537414	6.56E-06	4994
genus.Anaerofilum.id.2053	rs17012738	2	76698142	T	G	0.090341716	0.020023943	4.486458527	7.24E-06	5051
genus.Anaerofilum.id.2053	rs9299345	9	104339812	T	C	-0.136399548	0.030237163	-4.464033723	8.04E-06	5051
genus.Anaerofilum.id.2053	rs4244069	12	67167461	G	A	-0.146774518	0.032659424	-4.42127491	9.81E-06	5030
genus.Anaerostipes.id.1991	rs7193624	16	77574020	C	T	0.075064239	0.015072346	5.01314262	5.35E-07	16926
genus.Anaerostipes.id.1991	rs62215703	21	25873624	G	A	0.064496549	0.013670195	4.755690138	1.98E-06	15550
genus.Anaerostipes.id.1991	rs2396460	2	228018671	T	C	-0.051265275	0.010955542	-4.677009802	2.91E-06	16912
genus.Anaerostipes.id.1991	rs3900776	9	13525082	G	A	-0.1100132	0.023630171	-4.688834571	2.75E-06	14694

Gut	SNP	CHR	POS	A1	A2	BETA	SE	Z	P	N
genus.Anaerostipes.id.1991	rs1328008	10	117331998	G	A	-0.050821969	0.010944337	-4.618180577	3.87E-06	16926
genus.Anaerostipes.id.1991	rs62157625	2	142774333	T	C	0.088573041	0.018554786	4.818131718	1.45E-06	15215
genus.Anaerostipes.id.1991	rs6726833	2	39351569	C	A	-0.087749727	0.018940832	-4.649825083	3.32E-06	16924
genus.Anaerostipes.id.1991	rs6474958	9	1582701	A	G	-0.050006507	0.011200088	-4.501585881	6.74E-06	16918
genus.Anaerostipes.id.1991	rs6854026	4	169690814	T	C	-0.050848158	0.010907505	-4.657818288	3.20E-06	16921
genus.Anaerostipes.id.1991	rs78735375	19	1497547	A	C	-0.13742857	0.030530436	-4.551209459	5.33E-06	10360
genus.Anaerostipes.id.1991	rs7823228	8	128397494	G	C	-0.062108471	0.013661403	-4.532027466	5.84E-06	16445
genus.Anaerostipes.id.1991	rs13376554	1	67595108	A	T	0.19738899	0.04586424	4.472789265	7.72E-06	3101
genus.Anaerostipes.id.1991	rs10502061	11	105615253	A	G	0.083575226	0.019201667	4.466919696	7.94E-06	16825
genus.Anaerotruncus.id.2054	rs6563550	13	38058413	T	C	0.087713486	0.017674473	5.169662276	2.35E-07	16566
genus.Anaerotruncus.id.2054	rs4669806	2	12200752	G	T	0.057638903	0.012299403	4.714706258	2.42E-06	16566
genus.Anaerotruncus.id.2054	rs10150232	14	30418008	A	G	0.056708789	0.012487702	4.503743812	6.68E-06	16117
genus.Anaerotruncus.id.2054	rs1272208	9	78630894	G	T	-0.061174271	0.012983073	-4.597309455	4.28E-06	16117
genus.Anaerotruncus.id.2054	rs12056802	8	74713100	C	G	0.077419353	0.017717789	4.521965924	6.13E-06	16010
genus.Anaerotruncus.id.2054	rs11018566	11	89040226	A	G	-0.156465176	0.036603226	-4.521610439	6.14E-06	6278
genus.Anaerotruncus.id.2054	rs7675045	4	172808992	A	T	-0.049781529	0.01105734	-4.496170507	6.92E-06	16566
genus.Anaerotruncus.id.2054	rs1431492	3	150855371	C	T	-0.06549964	0.014618811	-4.483128728	7.36E-06	16560
genus.Anaerotruncus.id.2054	rs9347879	6	165015261	T	C	0.050618001	0.011049001	4.600078056	4.22E-06	16566
genus.Anaerotruncus.id.2054	rs17734739	2	211663702	T	C	0.066005195	0.014908026	4.481048496	7.43E-06	15927
genus.Anaerotruncus.id.2054	rs6494922	15	33459867	A	G	0.090310638	0.020225652	4.50543796	6.62E-06	16067
genus.Anaerotruncus.id.2054	rs7155595	14	77502546	C	A	0.053933621	0.011890292	4.477568045	7.55E-06	16553
genus.Anaerotruncus.id.2054	rs115414803	4	88163243	A	C	-0.144355888	0.031752395	-4.498771549	6.83E-06	9273
genus.Anaerotruncus.id.2054	rs2704155	11	54908652	T	A	-0.106234694	0.023481456	-4.547903566	5.42E-06	12419
genus.Bacteroides.id.918	rs6795673	3	10593224	C	T	0.053856525	0.010525127	5.100974685	3.38E-07	18301
genus.Bacteroides.id.918	rs28757219	6	29143451	T	A	0.081839827	0.017031866	4.840642393	1.29E-06	14833
genus.Bacteroides.id.918	rs9507307	13	24910476	C	T	0.060445621	0.012912793	4.740957737	2.13E-06	18184
genus.Bacteroides.id.918	rs66474973	20	58605432	G	T	0.081251539	0.016447899	4.966714765	6.81E-07	17405
genus.Bacteroides.id.918	rs11585893	1	10644351	A	G	-0.074074561	0.014763325	-4.775219844	1.80E-06	17821
genus.Bacteroides.id.918	rs495004	9	112855426	C	G	-0.060747491	0.012986536	-4.643779611	3.42E-06	17707
genus.Bacteroides.id.918	rs17619981	19	24342250	T	G	0.088097761	0.018700207	4.693413983	2.69E-06	15801
genus.Bacteroides.id.918	rs2023437	14	22045949	T	C	-0.07823245	0.016763399	-4.564023942	5.02E-06	17484

Gut	SNP	CHR	POS	A1	A2	BETA	SE	Z	P	N
genus.Bacteroides.id.918	rs13207588	6	41519430	A	G	-0.059204982	0.013119468	-4.479407749	7.49E-06	17707
genus.Bacteroides.id.918	rs2366421	3	62019400	T	A	-0.05281527	0.011712437	-4.474767651	7.65E-06	18299
genus.Bacteroides.id.918	rs1340391	1	102960989	T	C	-0.059200382	0.013224416	-4.502050394	6.73E-06	18302
genus.Barnesiella.id.944	rs2276875	4	5730954	A	G	-0.069717565	0.013953704	-5.040207467	4.65E-07	13893
genus.Barnesiella.id.944	rs2428166	6	110831148	G	A	-0.165858717	0.033730907	-4.923321676	8.51E-07	8091
genus.Barnesiella.id.944	rs60316894	12	54872438	C	T	-0.121478156	0.025183972	-4.857188513	1.19E-06	12717
genus.Barnesiella.id.944	rs35177866	3	181589155	A	G	0.091712545	0.019013343	4.674435431	2.95E-06	13394
genus.Barnesiella.id.944	rs13242616	7	122021518	T	C	-0.058374651	0.012317572	-4.725971785	2.29E-06	13945
genus.Barnesiella.id.944	rs79795328	4	114546595	A	G	-0.081864467	0.01764407	-4.599600616	4.23E-06	13657
genus.Barnesiella.id.944	rs2057922	7	10076378	G	C	0.091524071	0.019462277	4.62059908	3.83E-06	14208
genus.Barnesiella.id.944	rs199035	6	23435415	G	A	0.055949483	0.011971558	4.670618566	3.00E-06	13945
genus.Barnesiella.id.944	rs62251337	3	44327429	A	G	-0.069076268	0.014937412	-4.599177934	4.24E-06	13945
genus.Barnesiella.id.944	rs77455852	5	54107036	T	G	-0.089149084	0.019560057	-4.659905618	3.16E-06	13394
genus.Barnesiella.id.944	rs11155559	6	148601905	T	C	0.095602282	0.021296077	4.441843526	8.92E-06	13383
genus.Barnesiella.id.944	rs12909713	15	87145913	C	T	-0.055071556	0.012003894	-4.567096111	4.95E-06	14205
genus.Barnesiella.id.944	rs113258194	2	180020849	A	G	0.098992031	0.021412093	4.484421926	7.31E-06	13945
genus.Barnesiella.id.944	rs28479800	1	206216607	A	T	-0.091520023	0.021000228	-4.466952394	7.93E-06	12399
genus.Barnesiella.id.944	rs76181748	8	105458416	C	T	-0.077855934	0.017163996	-4.500587132	6.78E-06	14201
genus.Barnesiella.id.944	rs72684847	4	102616869	T	C	-0.114369278	0.025387327	-4.501055309	6.76E-06	12984
genus.Barnesiella.id.944	rs28418786	8	25834694	C	G	-0.079132421	0.017495701	-4.510212575	6.48E-06	13698
genus.Bifidobacterium.id.436	rs687670	2	136740900	C	T	0.11144998	0.012972177	8.463162131	2.60E-17	14778
genus.Bifidobacterium.id.436	rs7581382	2	135775130	C	T	-0.091347733	0.014847735	-6.644956487	3.03E-11	13806
genus.Bifidobacterium.id.436	rs7322849	13	112859829	T	C	0.112428453	0.020181262	5.717083536	1.08E-08	14778
genus.Bifidobacterium.id.436	rs1961273	2	137602926	C	T	0.067403609	0.013231896	5.093850066	3.51E-07	14778
genus.Bifidobacterium.id.436	rs10841473	12	20378911	G	C	-0.062420654	0.012943849	-4.792736086	1.65E-06	14777
genus.Bifidobacterium.id.436	rs56108664	5	537890	T	C	0.072997098	0.015792589	4.713063643	2.44E-06	13916
genus.Bifidobacterium.id.436	rs2686790	7	48090746	T	C	0.070741011	0.015792595	4.479011959	7.50E-06	14778
genus.Bifidobacterium.id.436	rs4567981	2	49906864	T	A	0.056208395	0.011792309	4.760795626	1.93E-06	14666
genus.Bifidobacterium.id.436	rs2491158	10	126089703	G	A	0.071262413	0.015983004	4.463925418	8.05E-06	14021
genus.Bifidobacterium.id.436	rs76671854	5	169748189	C	G	-0.084605457	0.01840032	-4.613643973	3.96E-06	13806
genus.Bifidobacterium.id.436	rs75344046	21	31861790	C	T	0.232354091	0.050597867	4.570589512	4.86E-06	3856

Gut	SNP	CHR	POS	A1	A2	BETA	SE	Z	P	N
genus.Bifidobacterium.id.436	rs540489	17	72897722	T	G	-0.063764086	0.013874591	-4.556772025	5.19E-06	14561
genus.Bifidobacterium.id.436	rs857444	6	14617591	C	T	0.055823437	0.012121924	4.634907826	3.57E-06	14777
genus.Bifidobacterium.id.436	rs12022129	1	207003374	G	A	0.061935632	0.013893747	4.465279423	8.00E-06	14776
genus.Bifidobacterium.id.436	rs62181700	2	189805784	G	A	-0.062464331	0.013120526	-4.73668125	2.17E-06	14774
genus.Bifidobacterium.id.436	rs55888705	4	1517826	A	G	0.054631934	0.012113865	4.503950037	6.67E-06	14777
genus.Bifidobacterium.id.436	rs5746486	22	18354272	T	C	-0.053621605	0.012080057	-4.439912332	9.00E-06	14778
genus.Bifidobacterium.id.436	rs73797465	5	142793467	T	G	-0.095356633	0.02092365	-4.592407441	4.38E-06	14666
genus.Bilophila.id.3170	rs116261629	6	20505628	G	C	0.128112276	0.026135828	4.920705183	8.62E-07	11276
genus.Bilophila.id.3170	rs1571225	9	4940871	C	T	0.082682569	0.017062147	4.868780227	1.12E-06	12854
genus.Bilophila.id.3170	rs3827020	20	61980991	C	T	0.076647909	0.016064223	4.775934231	1.79E-06	12628
genus.Bilophila.id.3170	rs1241171	1	103299921	G	A	-0.069265389	0.015014825	-4.599239166	4.24E-06	12856
genus.Bilophila.id.3170	rs7802841	7	138583840	C	A	0.067009659	0.013770056	4.778108524	1.77E-06	12856
genus.Bilophila.id.3170	rs8013541	14	67640571	A	T	-0.05719913	0.01256729	-4.545696996	5.48E-06	12856
genus.Bilophila.id.3170	rs6793291	3	194419531	C	A	0.112729046	0.024163294	4.66320646	3.11E-06	11051
genus.Bilophila.id.3170	rs1917709	7	123174986	A	T	0.118509423	0.02674196	4.482725503	7.37E-06	10886
genus.Bilophila.id.3170	rs542415	15	50011554	T	C	-0.061362319	0.013343594	-4.577330175	4.71E-06	12777
genus.Bilophila.id.3170	rs72676854	8	109990939	T	C	0.123208756	0.026882038	4.540102787	5.62E-06	11616
genus.Bilophila.id.3170	rs1969927	12	100786847	G	A	0.056458855	0.012693476	4.43818176	9.07E-06	12777
genus.Bilophila.id.3170	rs60178956	8	90878338	G	A	-0.062481785	0.014148499	-4.463685157	8.06E-06	12777
genus.Bilophila.id.3170	rs2728491	7	47211732	G	T	-0.062741568	0.013945646	-4.514970738	6.33E-06	12856
genus.Bilophila.id.3170	rs2713349	7	9510367	A	T	0.061722789	0.014017424	4.448865855	8.63E-06	12853
genus.Bilophila.id.3170	rs9899990	17	9121579	A	G	-0.102702281	0.023387821	-4.438307381	9.07E-06	12088
genus.Bilophila.id.3170	rs11069458	13	102402685	T	C	-0.068094659	0.015502996	-4.47293092	7.72E-06	12856
genus.Blautia.id.1992	rs11149971	16	77238810	C	T	0.117604747	0.023389975	4.884040877	1.04E-06	16255
genus.Blautia.id.1992	rs12453000	17	16895873	C	T	0.062533456	0.012998711	4.845777301	1.26E-06	17815
genus.Blautia.id.1992	rs115043014	1	175757311	G	A	-0.206605438	0.043991974	-4.557119378	5.19E-06	4902
genus.Blautia.id.1992	rs67794373	7	124210039	C	T	0.060170889	0.012344281	4.891632696	1.00E-06	18158
genus.Blautia.id.1992	rs117001700	7	68396262	T	C	0.196414252	0.044112197	4.443781902	8.84E-06	4965
genus.Blautia.id.1992	rs72973581	19	1043103	A	G	0.125169075	0.026540793	4.781567394	1.74E-06	12764
genus.Blautia.id.1992	rs4926264	19	13420917	T	C	0.082620681	0.017823907	4.560510934	5.10E-06	18276
genus.Blautia.id.1992	rs7860714	9	1866537	A	G	-0.050217775	0.010985269	-4.606680852	4.09E-06	18162

Gut	SNP	CHR	POS	A1	A2	BETA	SE	Z	P	N
genus.Blautia.id.1992	rs682885	11	87805911	A	G	-0.049337611	0.010735883	-4.587270057	4.49E-06	18274
genus.Blautia.id.1992	rs2788271	1	219117743	T	G	-0.057559633	0.013349169	-4.488760326	7.16E-06	18276
genus.Blautia.id.1992	rs113271346	14	78062644	C	T	0.078268222	0.017211647	4.498176944	6.85E-06	17465
genus.Blautia.id.1992	rs3005511	6	74605806	A	G	0.050108496	0.01107676	4.51982588	6.19E-06	18276
genus.Blautia.id.1992	rs16892041	6	9298640	T	C	-0.062260496	0.014180398	-4.444240636	8.82E-06	18276
genus.Butyricicoccus.id.2055	rs56221232	2	153453410	T	C	0.082802732	0.016740066	4.944871828	7.62E-07	17133
genus.Butyricicoccus.id.2055	rs7322368	13	100213990	T	C	0.081573314	0.018316651	4.544071055	5.52E-06	17134
genus.Butyricicoccus.id.2055	rs75238760	18	22899113	T	A	0.061942289	0.013994184	4.49994836	6.80E-06	17022
genus.Butyricicoccus.id.2055	rs12585793	13	27018240	T	C	-0.262206199	0.056472877	-4.533846691	5.79E-06	3135
genus.Butyricicoccus.id.2055	rs62478070	7	157688150	T	G	0.224039318	0.049495932	4.528595695	5.94E-06	4007
genus.Butyricicoccus.id.2055	rs4962426	10	126908208	G	T	0.061421574	0.013597912	4.482282301	7.38E-06	16889
genus.Butyricicoccus.id.2055	rs10084203	2	191304149	A	G	0.054969862	0.012356262	4.450014144	8.59E-06	17135
genus.Butyricicoccus.id.2055	rs12034718	1	67379595	A	G	0.0701199	0.01582135	4.426513024	9.58E-06	16237
genus.Butyricimonas.id.945	rs113054641	21	16550389	G	A	-0.144880972	0.027457379	-5.225490144	1.74E-07	9949
genus.Butyricimonas.id.945	rs62390301	5	159867647	T	C	-0.087298337	0.017489288	-4.950060171	7.42E-07	10634
genus.Butyricimonas.id.945	rs7083431	10	72711156	A	C	0.070353941	0.014443868	4.91552247	8.85E-07	10737
genus.Butyricimonas.id.945	rs2642760	5	62119947	G	C	0.071335713	0.014539571	4.921671865	8.58E-07	10634
genus.Butyricimonas.id.945	rs782080	3	127468661	T	A	0.065348802	0.01379735	4.731017211	2.23E-06	10733
genus.Butyricimonas.id.945	rs1862649	16	22172866	G	A	0.113123952	0.024817639	4.574984011	4.76E-06	10123
genus.Butyricimonas.id.945	rs12304031	12	129438771	G	A	-0.086278048	0.019684629	-4.502981573	6.70E-06	10658
genus.Butyricimonas.id.945	rs1701950	19	51465096	G	C	-0.105055622	0.024104658	-4.473938999	7.68E-06	9875
genus.Butyricimonas.id.945	rs71428626	2	83093264	G	T	-0.133188396	0.028990548	-4.573164558	4.80E-06	9776
genus.Butyricimonas.id.945	rs78453362	3	78740009	A	G	-0.14948019	0.032707281	-4.608384367	4.06E-06	8207
genus.Butyricimonas.id.945	rs326049	5	997217	C	G	0.075969365	0.01676173	4.460247655	8.19E-06	10557
genus.Butyricimonas.id.945	rs9657374	8	4834406	C	T	0.068071943	0.014817248	4.586675988	4.50E-06	10737
genus.Butyricimonas.id.945	rs12458763	18	34091618	A	C	0.122036628	0.026958664	4.513839014	6.37E-06	10383
genus.Butyricimonas.id.945	rs270727	1	45082825	G	C	-0.069417267	0.015079545	-4.54933504	5.38E-06	10634
genus.Butyricimonas.id.945	rs72814525	10	72443070	A	G	0.066415351	0.01497662	4.458695794	8.25E-06	10736
genus.Butyricimonas.id.945	rs11228830	11	56664962	A	G	0.135264872	0.029841132	4.507758178	6.55E-06	8974
genus.Butyrivibrio.id.1993	rs72723662	14	76065563	C	T	0.224078206	0.044933642	4.938752059	7.86E-07	3073
genus.Butyrivibrio.id.1993	rs7412979	1	235580646	C	G	0.186778577	0.038911205	4.785494247	1.71E-06	3480

Gut	SNP	CHR	POS	A1	A2	BETA	SE	Z	P	N
genus.Butyrivibrio.id.1993	rs7752361	6	111780705	A	G	-0.119230279	0.023997488	-4.943046072	7.69E-07	3481
genus.Butyrivibrio.id.1993	rs4537857	13	27292666	T	C	-0.124589674	0.026099163	-4.774457323	1.80E-06	3482
genus.Butyrivibrio.id.1993	rs11761679	7	150596929	T	C	0.154775363	0.032153939	4.733913618	2.20E-06	3482
genus.Butyrivibrio.id.1993	rs16941336	17	20575697	C	T	0.127487508	0.026793591	4.807038771	1.53E-06	3482
genus.Butyrivibrio.id.1993	rs77356209	18	10267733	T	C	0.216874487	0.04832994	4.504137738	6.66E-06	3379
genus.Butyrivibrio.id.1993	rs74622183	14	34150977	A	G	-0.201027447	0.042820448	-4.711626262	2.46E-06	3478
genus.Butyrivibrio.id.1993	rs7763512	6	21960091	G	A	0.119845684	0.025336711	4.663646266	3.11E-06	3259
genus.Butyrivibrio.id.1993	rs142855850	10	22587900	A	G	0.205013198	0.045713757	4.498128426	6.86E-06	3234
genus.Butyrivibrio.id.1993	rs9349693	6	53939724	A	G	0.117972728	0.025986063	4.542861293	5.55E-06	3482
genus.Butyrivibrio.id.1993	rs486484	20	811507	A	G	-0.108348689	0.024000793	-4.505848043	6.61E-06	3456
genus.Butyrivibrio.id.1993	rs17163238	5	128976021	G	A	0.140986912	0.030911712	4.544294395	5.51E-06	3482
genus.Butyrivibrio.id.1993	rs4928024	3	54288503	A	G	-0.174709994	0.038933257	-4.460249916	8.19E-06	3481
genus.Butyrivibrio.id.1993	rs1007475	6	148403405	G	T	0.118065426	0.026127086	4.467250977	7.92E-06	3482
genus.Butyrivibrio.id.1993	rs16934069	9	119693164	T	C	-0.133784151	0.029944364	-4.443296318	8.86E-06	3482
genus.CandidatusSoleaferrea.id.11350	rs4294381	1	224688107	T	C	0.112196526	0.023186007	4.82953849	1.37E-06	6145
genus.CandidatusSoleaferrea.id.11350	rs10090365	8	138638199	A	G	-0.083441348	0.018098882	-4.602569891	4.17E-06	6145
genus.CandidatusSoleaferrea.id.11350	rs4678258	3	137945192	T	C	0.098613616	0.021564627	4.543536233	5.53E-06	6101
genus.CandidatusSoleaferrea.id.11350	rs10108780	8	123882833	A	G	-0.09283404	0.01998066	-4.630900266	3.64E-06	5999
genus.CandidatusSoleaferrea.id.11350	rs386526	10	82879688	C	G	0.081845856	0.018008617	4.456502565	8.33E-06	6144
genus.CandidatusSoleaferrea.id.11350	rs36155147	7	352368	C	T	0.104966126	0.024099527	4.548172288	5.41E-06	5958
genus.CandidatusSoleaferrea.id.11350	rs11153159	6	109376647	G	C	-0.128050003	0.028544611	-4.590812673	4.42E-06	6101
genus.CandidatusSoleaferrea.id.11350	rs10809135	9	10611282	T	C	0.08348579	0.01824263	4.545836903	5.47E-06	5999
genus.CandidatusSoleaferrea.id.11350	rs6489992	12	115352769	A	G	-0.084043749	0.018703073	-4.46820537	7.89E-06	6142
genus.CandidatusSoleaferrea.id.11350	rs6494306	15	62394540	A	G	-0.09693036	0.021422086	-4.533533402	5.80E-06	6145
genus.CandidatusSoleaferrea.id.11350	rs12500231	4	52935445	A	T	0.08138533	0.018269552	4.474015791	7.68E-06	6145
genus.CandidatusSoleaferrea.id.11350	rs7400877	14	76403542	T	C	-0.095108487	0.021279907	-4.432967694	9.29E-06	6145
genus.CandidatusSoleaferrea.id.11350	rs2193878	3	190349049	T	A	0.228255107	0.050924135	4.429265308	9.46E-06	3088
genus.CandidatusSoleaferrea.id.11350	rs830149	19	47991441	C	G	0.184646616	0.039723748	4.426356176	9.58E-06	5065
genus.Catenibacterium.id.2153	rs12404911	1	240118443	C	T	0.140717216	0.030414222	4.685050361	2.80E-06	3239
genus.Catenibacterium.id.2153	rs77285108	4	122282768	G	A	-0.161826131	0.035344674	-4.631697189	3.63E-06	3210
genus.Catenibacterium.id.2153	rs73128290	7	57364320	A	G	0.129726249	0.02845626	4.596633523	4.29E-06	3239

Gut	SNP	CHR	POS	A1	A2	BETA	SE	Z	P	N
genus.Catenibacterium.id.2153	rs212393	6	159485742	G	A	-0.135253856	0.02862088	-4.631806624	3.62E-06	3239
genus.Catenibacterium.id.2153	rs7742829	6	105089665	C	T	0.114110133	0.025109895	4.540680758	5.61E-06	3239
genus.ChristensenellaceaeR.7group.id.11283	rs999354	19	34076956	T	A	0.058024779	0.011718514	4.961170834	7.01E-07	16324
genus.ChristensenellaceaeR.7group.id.11283	rs78521377	10	126448374	C	T	0.12499178	0.027479689	4.540701214	5.61E-06	12605
genus.ChristensenellaceaeR.7group.id.11283	rs62467127	7	118096925	C	T	0.114108279	0.025198272	4.65412335	3.25E-06	12719
genus.ChristensenellaceaeR.7group.id.11283	rs17081797	18	67555560	A	G	-0.090432475	0.020425314	-4.648701848	3.34E-06	16324
genus.ChristensenellaceaeR.7group.id.11283	rs62132810	19	49279227	A	G	-0.082885316	0.017962272	-4.538442988	5.67E-06	15066
genus.ChristensenellaceaeR.7group.id.11283	rs60954665	19	35883736	T	G	0.049814958	0.011078727	4.489717584	7.13E-06	16324
genus.ChristensenellaceaeR.7group.id.11283	rs79150079	5	29352420	C	A	0.121546581	0.027097732	4.430120624	9.42E-06	11994
genus.ChristensenellaceaeR.7group.id.11283	rs62190261	2	230943099	A	C	0.095839425	0.021472282	4.446200643	8.74E-06	15274
genus.ChristensenellaceaeR.7group.id.11283	rs892686	9	83043376	A	G	0.051409887	0.011135975	4.613019762	3.97E-06	16325
genus.ChristensenellaceaeR.7group.id.11283	rs73952017	18	1779609	C	T	-0.086216444	0.019435393	-4.453258312	8.46E-06	15274
genus.Clostridiumsensustricto1.id.1873	rs550843	6	165722832	T	C	-0.078324645	0.016920943	-4.748362223	2.05E-06	14102
genus.Clostridiumsensustricto1.id.1873	rs2795528	10	43270264	G	A	-0.184314695	0.039220419	-4.690874569	2.72E-06	6366
genus.Clostridiumsensustricto1.id.1873	rs2817172	1	3041519	C	T	0.058138957	0.012449219	4.687431551	2.77E-06	13658
genus.Clostridiumsensustricto1.id.1873	rs115807074	5	84997131	A	G	-0.227359786	0.049257523	-4.595511955	4.32E-06	4229
genus.Clostridiumsensustricto1.id.1873	rs116847295	12	43485015	C	T	0.110021418	0.024603155	4.583106862	4.58E-06	12910
genus.Clostridiumsensustricto1.id.1873	rs12341505	9	136710881	G	A	0.081071775	0.018012111	4.572397904	4.82E-06	14118
genus.Clostridiumsensustricto1.id.1873	rs11586026	1	83252771	A	T	0.110966819	0.025049788	4.443520394	8.85E-06	13095
genus.Clostridiumsensustricto1.id.1873	rs12490337	3	111482845	C	G	-0.061687867	0.0137581	-4.479133839	7.49E-06	14121
genus.Collinsella.id.815	rs9541268	13	68610061	C	A	0.09596998	0.019731218	4.916863286	8.79E-07	12914
genus.Collinsella.id.815	rs2671662	17	47529492	C	G	-0.056775091	0.011944997	-4.732254116	2.22E-06	14334
genus.Collinsella.id.815	rs73052258	2	195739311	G	A	0.093017814	0.020265842	4.784305008	1.72E-06	14334
genus.Collinsella.id.815	rs2103510	21	29355467	G	A	0.078655101	0.016821672	4.714357859	2.42E-06	14334
genus.Collinsella.id.815	rs75672793	4	149818294	A	G	-0.108908003	0.024052154	-4.521665559	6.14E-06	12601
genus.Collinsella.id.815	rs10890671	11	107123199	T	C	-0.053740144	0.011884842	-4.508740628	6.52E-06	14334
genus.Collinsella.id.815	rs12921100	16	77283458	A	T	0.056375862	0.012701892	4.459223786	8.23E-06	14334
genus.Collinsella.id.815	rs59414781	15	50026113	C	G	0.066886563	0.014977291	4.436408314	9.15E-06	14334
genus.Collinsella.id.815	rs62448871	7	24465024	C	A	-0.054030922	0.012032414	-4.500401347	6.78E-06	14334
genus.Collinsella.id.815	rs1496626	19	31760841	T	C	-0.072230674	0.016160046	-4.500425671	6.78E-06	14334
genus.Collinsella.id.815	rs149807560	19	56526640	C	A	-0.104264209	0.023584097	-4.490707509	7.10E-06	13473

Gut	SNP	CHR	POS	A1	A2	BETA	SE	Z	P	N
genus.Collinsella.id.815	rs11597285	10	8589813	G	T	-0.053794651	0.012054849	-4.431081895	9.38E-06	14334
genus.Coprobacter.id.949	rs305411	1	88233128	A	G	0.129218483	0.026451784	4.889862418	1.01E-06	6777
genus.Coprobacter.id.949	rs3828477	3	46483717	G	T	-0.091217951	0.019569041	-4.678505322	2.89E-06	6798
genus.Coprobacter.id.949	rs55672356	4	34852443	T	A	-0.193354203	0.041381488	-4.689636917	2.74E-06	4903
genus.Coprobacter.id.949	rs143662916	11	103549974	C	T	0.25326389	0.05401412	4.665911429	3.07E-06	3083
genus.Coprobacter.id.949	rs213863	6	97234939	C	T	-0.088741189	0.018837727	-4.720830981	2.35E-06	6798
genus.Coprobacter.id.949	rs72821405	6	4714797	T	C	-0.147365313	0.031997805	-4.575202371	4.76E-06	6177
genus.Coprobacter.id.949	rs11532348	12	98289796	C	T	-0.103930603	0.022682638	-4.536784641	5.71E-06	6364
genus.Coprobacter.id.949	rs12996055	2	137223458	A	C	0.092181727	0.020940499	4.46314881	8.08E-06	6570
genus.Coprobacter.id.949	rs12684609	9	137784868	T	C	0.100826957	0.022036643	4.522831812	6.10E-06	6364
genus.Coprobacter.id.949	rs5011652	15	91166608	G	C	0.089959749	0.02000097	4.544349933	5.51E-06	6783
genus.Coprobacter.id.949	rs74919520	2	32242452	G	A	0.125702917	0.02762957	4.534893243	5.76E-06	6387
genus.Coprobacter.id.949	rs28402691	4	96389240	T	C	0.110695393	0.025119913	4.426968133	9.56E-06	6387
genus.Coprobacter.id.949	rs189356	20	57574851	G	A	0.078112515	0.017191637	4.517313786	6.26E-06	6763
genus.Coprobacter.id.949	rs76001613	4	3852385	C	G	0.215910371	0.049301859	4.436908906	9.13E-06	3385
genus.Coprococcus1.id.11301	rs4277593	20	4319420	G	A	-0.058564915	0.010991363	-5.302913118	1.14E-07	16651
genus.Coprococcus1.id.11301	rs56405618	4	174468316	A	G	-0.089630001	0.018650815	-4.801864762	1.57E-06	16379
genus.Coprococcus1.id.11301	rs74101919	1	95424321	T	C	-0.071898851	0.014465115	-4.885262334	1.03E-06	16732
genus.Coprococcus1.id.11301	rs1010560	1	30400301	C	A	0.058023212	0.012272151	4.757592681	1.96E-06	16723
genus.Coprococcus1.id.11301	rs73031725	11	134622282	T	C	0.167590996	0.035522381	4.75553952	1.98E-06	7206
genus.Coprococcus1.id.11301	rs1576241	6	72529277	A	G	-0.051034349	0.010953456	-4.649424505	3.33E-06	16729
genus.Coprococcus1.id.11301	rs73167075	3	165693143	T	C	0.057313703	0.01275731	4.450460439	8.57E-06	16733
genus.Coprococcus1.id.11301	rs12794898	11	124554061	G	T	0.090329854	0.019721215	4.568370197	4.92E-06	15186
genus.Coprococcus1.id.11301	rs946513	10	15913820	C	T	0.205898923	0.046001796	4.449212965	8.62E-06	4310
genus.Coprococcus1.id.11301	rs7784490	7	132556835	C	G	-0.051854255	0.011313593	-4.584427144	4.55E-06	16733
genus.Coprococcus1.id.11301	rs12886051	14	29898456	G	C	-0.052178208	0.011805063	-4.464975457	8.01E-06	16633
genus.Coprococcus1.id.11301	rs2907920	19	2600717	A	G	0.056117333	0.012682384	4.474660876	7.65E-06	16040
genus.Coprococcus2.id.11302	rs59936925	20	49266770	A	T	0.117085317	0.023399473	4.904313748	9.38E-07	10931
genus.Coprococcus2.id.11302	rs6677933	1	112139008	C	T	-0.080440499	0.016421626	-4.858126895	1.19E-06	11483
genus.Coprococcus2.id.11302	rs1958519	14	86833255	T	A	0.066528846	0.013855806	4.801232369	1.58E-06	11009
genus.Coprococcus2.id.11302	rs2482516	9	25554068	C	T	0.075441464	0.016461865	4.576715374	4.72E-06	11483

Gut	SNP	CHR	POS	A1	A2	BETA	SE	Z	P	N
genus.Coprococcus2.id.11302	rs35890118	10	129715759	A	G	-0.066536003	0.014766106	-4.458341592	8.26E-06	11478
genus.Coprococcus2.id.11302	rs10121347	9	16806694	C	G	0.092580538	0.021945877	4.456927632	8.31E-06	11457
genus.Coprococcus2.id.11302	rs9426473	1	4210455	A	G	0.072736094	0.016166159	4.515831925	6.31E-06	11486
genus.Coprococcus2.id.11302	rs6894272	5	33114638	T	C	-0.113458171	0.025300319	-4.427525145	9.53E-06	11001
genus.Coprococcus2.id.11302	rs35916879	4	131232223	C	G	0.068393956	0.015212626	4.547152542	5.44E-06	11484
genus.Coprococcus2.id.11302	rs12634070	3	180544880	T	C	0.073648589	0.016492134	4.418234597	9.95E-06	11457
genus.Coprococcus2.id.11302	rs61823518	1	223688236	A	C	-0.095538071	0.021572431	-4.503555947	6.68E-06	10980
genus.Coprococcus3.id.11303	rs8100692	19	40032132	T	C	0.057741093	0.011347595	5.061276455	4.16E-07	15829
genus.Coprococcus3.id.11303	rs62481985	8	4128340	G	C	-0.058238523	0.011505221	-5.060773065	4.18E-07	15472
genus.Coprococcus3.id.11303	rs178271	22	21331556	T	C	0.145256923	0.029423868	4.940034444	7.81E-07	10794
genus.Coprococcus3.id.11303	rs13394391	2	240727657	C	T	-0.070930158	0.015091785	-4.733835123	2.20E-06	15834
genus.Coprococcus3.id.11303	rs11080344	17	26104511	C	T	0.051692096	0.011305408	4.573915906	4.79E-06	15835
genus.Coprococcus3.id.11303	rs7521171	1	149998497	G	A	-0.05964344	0.012927857	-4.595244669	4.32E-06	15472
genus.Coprococcus3.id.11303	rs11077359	17	76343961	T	C	-0.064544278	0.014883314	-4.425184616	9.64E-06	15378
genus.Coprococcus3.id.11303	rs6994742	8	8540070	C	T	0.055130618	0.012392938	4.469614036	7.84E-06	15834
genus.Coprococcus3.id.11303	rs13247359	7	76357726	G	A	0.051210989	0.011303088	4.483986969	7.33E-06	15833
genus.Coprococcus3.id.11303	rs4575475	14	99141751	G	A	0.061951375	0.013778722	4.49238963	7.04E-06	15154
genus.Coprococcus3.id.11303	rs10810043	9	13799809	A	G	0.051551783	0.011592302	4.433437387	9.27E-06	15833
genus.DefluviitaleaceaeUCG011.id.11287	rs72731813	4	147414743	C	T	-0.147382972	0.029379613	-5.053795414	4.33E-07	7767
genus.DefluviitaleaceaeUCG011.id.11287	rs112893842	9	8786663	T	C	0.113810202	0.023280562	4.818647501	1.45E-06	8183
genus.DefluviitaleaceaeUCG011.id.11287	rs9725395	1	85205632	A	G	-0.138339669	0.029554002	-4.637926927	3.52E-06	8011
genus.DefluviitaleaceaeUCG011.id.11287	rs55658617	21	41811003	T	C	0.174368775	0.036222554	4.738513178	2.15E-06	6663
genus.DefluviitaleaceaeUCG011.id.11287	rs9608282	22	24804081	T	G	0.14293919	0.029979298	4.706468123	2.52E-06	7716
genus.DefluviitaleaceaeUCG011.id.11287	rs28696126	7	79734541	A	T	-0.106502748	0.023847256	-4.505295727	6.63E-06	7811
genus.DefluviitaleaceaeUCG011.id.11287	rs12122999	1	118782748	G	T	-0.0827292	0.018115427	-4.572219895	4.83E-06	7811
genus.DefluviitaleaceaeUCG011.id.11287	rs4344384	10	66407366	G	T	0.071597782	0.015630912	4.571936184	4.83E-06	8180
genus.DefluviitaleaceaeUCG011.id.11287	rs2892880	4	120741726	G	A	0.081775542	0.018168647	4.498955544	6.83E-06	8182
genus.Desulfovibrio.id.3173	rs11160353	14	97116904	T	A	-0.069175564	0.014728505	-4.692184592	2.70E-06	9234
genus.Desulfovibrio.id.3173	rs2853179	8	105462047	C	T	0.081197078	0.017423443	4.714387232	2.42E-06	8949
genus.Desulfovibrio.id.3173	rs16863365	2	198089911	A	G	0.109402491	0.022696375	4.775287912	1.79E-06	8776
genus.Desulfovibrio.id.3173	rs12031543	1	68664315	T	C	-0.127184982	0.028188722	-4.50788704	6.55E-06	8855

Gut	SNP	CHR	POS	A1	A2	BETA	SE	Z	P	N
genus.Desulfovibrio.id.3173	rs13066142	3	67478973	G	A	0.11914059	0.025089055	4.622761322	3.79E-06	9241
genus.Desulfovibrio.id.3173	rs1294544	14	90986846	A	C	0.068878169	0.015363458	4.471263826	7.78E-06	9241
genus.Desulfovibrio.id.3173	rs2590913	13	64264446	G	A	0.154495517	0.033939625	4.504459475	6.65E-06	7225
genus.Desulfovibrio.id.3173	rs2032031	10	130170270	A	G	-0.065493057	0.014868084	-4.436513341	9.14E-06	9241
genus.Desulfovibrio.id.3173	rs72647089	8	57972916	T	G	-0.10659436	0.023933668	-4.457357039	8.30E-06	8985
genus.Desulfovibrio.id.3173	rs7729080	5	52653978	C	A	-0.070286961	0.015769108	-4.418068335	9.96E-06	9241
genus.Desulfovibrio.id.3173	rs4797774	18	13447995	G	A	0.21258194	0.04700444	4.539336466	5.64E-06	3476
genus.Desulfovibrio.id.3173	rs6580353	5	139410687	T	C	0.0770979	0.016973529	4.567380563	4.94E-06	9241
genus.Dialister.id.2183	rs11166701	8	138295725	G	A	-0.065530102	0.013186	-5.007733944	5.51E-07	11720
genus.Dialister.id.2183	rs517089	11	10967286	T	A	0.076227029	0.017041108	4.55914068	5.14E-06	11988
genus.Dialister.id.2183	rs4747450	10	23199653	C	A	0.066850856	0.014768536	4.532198799	5.84E-06	11987
genus.Dialister.id.2183	rs2314294	16	9336862	T	C	0.086593192	0.019371895	4.463079917	8.08E-06	11339
genus.Dialister.id.2183	rs4753063	11	92267129	G	A	-0.059629704	0.013005185	-4.570878298	4.86E-06	11989
genus.Dialister.id.2183	rs2435610	7	150890034	A	C	0.064713149	0.014331424	4.528774182	5.93E-06	11984
genus.Dialister.id.2183	rs10938938	4	23294359	G	A	-0.077351446	0.017091491	-4.482694941	7.37E-06	11401
genus.Dialister.id.2183	rs10138457	14	102458052	T	C	-0.113053794	0.026193253	-4.468385495	7.88E-06	10968
genus.Dialister.id.2183	rs11071887	15	32955095	T	C	0.066240302	0.014631484	4.52943842	5.91E-06	11989
genus.Dialister.id.2183	rs764177	3	45809449	C	A	-0.060145359	0.013536587	-4.425818696	9.61E-06	11988
genus.Dialister.id.2183	rs76680460	9	25447080	G	A	-0.16129611	0.036433211	-4.460100276	8.19E-06	7176
genus.Dialister.id.2183	rs75416973	1	172809633	A	G	0.072722304	0.016450983	4.429141117	9.46E-06	11402
genus.Dorea.id.1997	rs13279148	8	127905865	G	A	0.071530899	0.015087753	4.729311159	2.25E-06	17605
genus.Dorea.id.1997	rs62503162	8	15768657	A	G	-0.09740913	0.019437924	-4.948831085	7.47E-07	16210
genus.Dorea.id.1997	rs78772518	8	13165357	A	C	0.138259714	0.028360928	4.646254926	3.38E-06	10858
genus.Dorea.id.1997	rs12216169	6	116078810	T	A	0.088172222	0.019368315	4.551312335	5.33E-06	16086
genus.Dorea.id.1997	rs73729431	6	25078050	C	T	-0.137450296	0.029998324	-4.659655189	3.17E-06	9596
genus.Dorea.id.1997	rs4793307	17	70733523	C	T	0.057416514	0.012247163	4.611024219	4.01E-06	17600
genus.Dorea.id.1997	rs11150408	16	81803497	T	G	0.04882539	0.010906337	4.491938968	7.06E-06	17215
genus.Dorea.id.1997	rs345219	3	6826520	T	G	-0.049736844	0.011262081	-4.444721005	8.80E-06	17215
genus.Dorea.id.1997	rs62583469	9	106815466	T	A	-0.063413594	0.014222695	-4.534460732	5.78E-06	16819
genus.Dorea.id.1997	rs3005511	6	74605806	A	G	0.051612659	0.011285226	4.553063945	5.29E-06	17610
genus.Dorea.id.1997	rs1899291	4	60992340	C	T	0.069721719	0.015028462	4.583761026	4.57E-06	17609

Gut	SNP	CHR	POS	A1	A2	BETA	SE	Z	P	N
genus.Dorea.id.1997	rs3752849	11	8729057	G	A	0.16384587	0.036607992	4.473899857	7.68E-06	5322
genus.Dorea.id.1997	rs12537781	7	154978183	T	C	-0.055544008	0.012522298	-4.4363107	9.15E-06	17608
genus.Eggerthella.id.819	rs3851328	2	45453805	T	G	-0.107774251	0.023652669	-4.602430504	4.18E-06	5147
genus.Eggerthella.id.819	rs2240838	7	38335954	A	G	0.098058185	0.01976528	4.951561151	7.36E-07	5147
genus.Eggerthella.id.819	rs2223081	21	29778032	G	A	0.102571659	0.022110762	4.617234094	3.89E-06	5147
genus.Eggerthella.id.819	rs112205261	1	9136139	T	C	-0.188617927	0.040361319	-4.648320634	3.35E-06	5036
genus.Eggerthella.id.819	rs4985746	17	17098213	G	A	0.110522311	0.024765265	4.536682241	5.71E-06	4906
genus.Eggerthella.id.819	rs1784446	11	102461599	G	A	0.090772774	0.019809579	4.55528905	5.23E-06	5147
genus.Eggerthella.id.819	rs76663501	20	55647869	C	T	0.175349437	0.03785764	4.572224336	4.83E-06	4814
genus.Eggerthella.id.819	rs6430926	2	141337454	C	T	0.08797364	0.019712748	4.455463242	8.37E-06	5147
genus.Eggerthella.id.819	rs13070736	3	20807454	A	C	-0.12133107	0.027202382	-4.475615502	7.62E-06	5147
genus.Eggerthella.id.819	rs67490567	15	65691324	T	C	0.108494846	0.024545741	4.441289077	8.94E-06	5083
genus.Eisenbergiella.id.11304	rs3812426	8	50822676	G	A	0.106447316	0.0224159	4.691139956	2.72E-06	5583
genus.Eisenbergiella.id.11304	rs1508033	15	53375201	A	C	0.091545961	0.019578652	4.655695661	3.23E-06	5580
genus.Eisenbergiella.id.11304	rs12278566	11	124764527	T	A	-0.121064706	0.025165804	-4.792153513	1.65E-06	5582
genus.Eisenbergiella.id.11304	rs2683098	15	36178565	C	T	0.107318939	0.022515029	4.730697297	2.24E-06	5582
genus.Eisenbergiella.id.11304	rs11079158	17	53367641	T	C	0.100626785	0.022546032	4.483336639	7.35E-06	5582
genus.Eisenbergiella.id.11304	rs13258851	8	55330814	A	G	0.137001443	0.030212434	4.472073519	7.75E-06	5583
genus.Eisenbergiella.id.11304	rs12710729	2	19973210	C	A	0.089338392	0.019906928	4.42056978	9.84E-06	5583
genus.Eisenbergiella.id.11304	rs12257723	10	109417101	A	C	-0.095274183	0.021156892	-4.443487907	8.85E-06	5582
genus.Eisenbergiella.id.11304	rs4462860	21	21610254	G	A	0.093907092	0.020110429	4.603113807	4.16E-06	5460
genus.Eisenbergiella.id.11304	rs11027642	11	24001840	C	T	0.129005538	0.028484392	4.568225166	4.92E-06	5583
genus.Eisenbergiella.id.11304	rs1553971	3	111419486	T	G	0.120964524	0.026305423	4.553806828	5.27E-06	5583
genus.Enterorhabdus.id.820	rs11098863	4	127336602	T	A	-0.096600831	0.016267701	-5.928536867	3.06E-09	7524
genus.Enterorhabdus.id.820	rs7923280	10	20280612	A	T	0.086001907	0.016914834	5.017362784	5.24E-07	7524
genus.Enterorhabdus.id.820	rs114731706	2	19611953	T	G	0.182308046	0.038229783	4.736592063	2.17E-06	5956
genus.Enterorhabdus.id.820	rs3017103	11	62174193	A	G	0.098089701	0.020899456	4.674936776	2.94E-06	7524
genus.Enterorhabdus.id.820	rs73331712	12	69515102	T	C	0.261989512	0.055123019	4.571207014	4.85E-06	3109
genus.Enterorhabdus.id.820	rs77655283	2	236439879	G	A	0.132987641	0.029830551	4.53064378	5.88E-06	7494
genus.Enterorhabdus.id.820	rs10098492	8	113109756	T	C	0.132323148	0.029368842	4.512535363	6.41E-06	7361
genus.Erysipelatoclostridium.id.11381	rs7221249	17	10177708	A	G	0.083986052	0.01427414	5.871852805	4.31E-09	9781

Gut	SNP	CHR	POS	A1	A2	BETA	SE	Z	P	N
genus.Erysipelatoclostridium.id.11381	rs710230	1	42333631	T	C	0.143381274	0.028151388	4.980830499	6.33E-07	9016
genus.Erysipelatoclostridium.id.11381	rs4697572	4	25446787	A	G	-0.081063643	0.016330822	-4.945535056	7.59E-07	9779
genus.Erysipelatoclostridium.id.11381	rs58236560	11	121876913	G	T	-0.11115688	0.023442572	-4.737735273	2.16E-06	9368
genus.Erysipelatoclostridium.id.11381	rs622418	9	90586043	A	G	-0.066805108	0.014322449	-4.628594416	3.68E-06	9783
genus.Erysipelatoclostridium.id.11381	rs17804233	5	77815056	T	C	-0.066291401	0.014420319	-4.582884656	4.59E-06	9783
genus.Erysipelatoclostridium.id.11381	rs1434153	2	34595533	G	A	-0.06845264	0.01524432	-4.498205457	6.85E-06	9662
genus.Erysipelatoclostridium.id.11381	rs340991	5	34892042	A	G	-0.074048187	0.015889138	-4.624809756	3.75E-06	9781
genus.Erysipelatoclostridium.id.11381	rs6474512	8	38780606	A	C	0.067014995	0.014320143	4.669764292	3.02E-06	9783
genus.Erysipelatoclostridium.id.11381	rs61806970	1	167818597	C	T	0.142547998	0.032068815	4.437662545	9.09E-06	8644
genus.Erysipelatoclostridium.id.11381	rs9652192	13	46368728	C	A	0.065979098	0.014800864	4.493023704	7.02E-06	9782
genus.Erysipelatoclostridium.id.11381	rs45480394	19	55857777	T	G	-0.068713443	0.015183535	-4.474429849	7.66E-06	9604
genus.Erysipelatoclostridium.id.11381	rs34528142	17	71411325	C	G	-0.087555073	0.019555627	-4.521957255	6.13E-06	9604
genus.Erysipelatoclostridium.id.11381	rs16936671	10	36394609	C	T	-0.096802556	0.021788273	-4.524876041	6.04E-06	9488
genus.Erysipelatoclostridium.id.11381	rs3804326	6	24292185	A	G	0.141469838	0.033635154	4.420380501	9.85E-06	7028
genus.ErysipelotrichaceaeUCG003.id.11384	rs28568391	10	125628540	A	G	-0.058391081	0.011874052	-4.97814886	6.42E-07	14544
genus.ErysipelotrichaceaeUCG003.id.11384	rs11994308	8	91660344	C	T	0.115417413	0.024250252	4.835872448	1.33E-06	13558
genus.ErysipelotrichaceaeUCG003.id.11384	rs76502207	15	86522244	T	C	0.14484376	0.028992913	4.978399775	6.41E-07	10740
genus.ErysipelotrichaceaeUCG003.id.11384	rs2302840	18	43072987	T	C	-0.087196551	0.017687036	-4.840782448	1.29E-06	14897
genus.ErysipelotrichaceaeUCG003.id.11384	rs10164067	18	46614602	T	G	-0.103260131	0.021240502	-4.867383188	1.13E-06	14427
genus.ErysipelotrichaceaeUCG003.id.11384	rs8053479	16	4076830	A	G	-0.083821185	0.018650912	-4.532313105	5.83E-06	13813
genus.ErysipelotrichaceaeUCG003.id.11384	rs17798136	7	33684926	G	A	0.15881755	0.034767431	4.655243481	3.24E-06	7421
genus.ErysipelotrichaceaeUCG003.id.11384	rs11666127	19	57828980	A	G	-0.07191871	0.016137058	-4.467764079	7.90E-06	13813
genus.ErysipelotrichaceaeUCG003.id.11384	rs62403464	6	24220160	T	C	-0.073174326	0.015651572	-4.642788377	3.44E-06	14899
genus.ErysipelotrichaceaeUCG003.id.11384	rs59068084	4	113256737	T	G	0.056477243	0.012022387	4.662927917	3.12E-06	14544
genus.ErysipelotrichaceaeUCG003.id.11384	rs73074432	7	20554814	C	T	0.072153999	0.016437785	4.417385417	9.99E-06	14217
genus.ErysipelotrichaceaeUCG003.id.11384	rs214586	6	18213637	T	C	-0.1064546	0.024038036	-4.452150035	8.50E-06	13505
genus.ErysipelotrichaceaeUCG003.id.11384	rs4758231	11	7765096	G	T	-0.05517544	0.012204675	-4.507839186	6.55E-06	14890
genus.ErysipelotrichaceaeUCG003.id.11384	rs74988980	16	80167277	G	A	-0.133141406	0.034857439	-4.448610444	8.64E-06	5944
genus.ErysipelotrichaceaeUCG003.id.11384	rs75949021	2	175403951	T	C	-0.16966706	0.037423793	-4.63466993	3.58E-06	6721
genus.ErysipelotrichaceaeUCG003.id.11384	rs6875357	5	32345671	C	T	0.165673441	0.035383116	4.503118062	6.70E-06	7034
genus.ErysipelotrichaceaeUCG003.id.11384	rs79396538	14	47935928	C	G	0.084814752	0.019114638	4.448808743	8.63E-06	13750

Gut	SNP	CHR	POS	A1	A2	BETA	SE	Z	P	N
genus.ErysipelotrichaceaeUCG003.id.11384	rs12251396	10	873148	A	G	-0.070539949	0.015876598	-4.427834516	9.52E-06	14211
genus.Escherichia.Shigella.id.3504	rs2267739	7	31136897	G	C	0.115505392	0.02376277	4.821506455	1.42E-06	11853
genus.Escherichia.Shigella.id.3504	rs1154904	11	134774845	A	G	-0.061337641	0.01306443	-4.668267238	3.04E-06	12002
genus.Escherichia.Shigella.id.3504	rs73208162	21	39324484	A	G	-0.119295648	0.024838994	-4.734688952	2.19E-06	11073
genus.Escherichia.Shigella.id.3504	rs113513883	7	140678199	A	G	0.172283306	0.038041024	4.553456401	5.28E-06	6280
genus.Escherichia.Shigella.id.3504	rs7502686	17	14173290	G	C	-0.136383738	0.030257643	-4.52980328	5.90E-06	9990
genus.Escherichia.Shigella.id.3504	rs57024273	2	236515155	T	C	0.062617236	0.013991866	4.423651661	9.70E-06	11853
genus.Escherichia.Shigella.id.3504	rs113127095	13	23253392	A	G	0.151009395	0.032343436	4.649123037	3.33E-06	8433
genus.Escherichia.Shigella.id.3504	rs117092367	10	69394914	A	T	0.11736182	0.026453289	4.424866189	9.65E-06	10304
genus.Escherichia.Shigella.id.3504	rs112767262	16	3750623	T	C	0.073296333	0.01635886	4.459728281	8.21E-06	11771
genus.Escherichia.Shigella.id.3504	rs592299	9	136179347	T	C	-0.059200433	0.012948643	-4.574689314	4.77E-06	11995
genus.Escherichia.Shigella.id.3504	rs4731451	7	128054100	G	A	-0.060988547	0.013515708	-4.479748621	7.47E-06	12001
genus.Escherichia.Shigella.id.3504	rs35555519	16	77711641	C	G	0.101975959	0.022288306	4.568028971	4.92E-06	12002
genus.Escherichia.Shigella.id.3504	rs2798105	1	48961903	A	G	-0.100850689	0.02220448	-4.458721071	8.25E-06	11296
genus.Faecalibacterium.id.2057	rs12320842	12	83502773	C	G	0.094832958	0.016396224	5.777774553	7.57E-09	17638
genus.Faecalibacterium.id.2057	rs6910935	6	131587157	A	G	0.134863869	0.027703163	4.828090463	1.38E-06	12640
genus.Faecalibacterium.id.2057	rs1271565	14	64044097	C	T	-0.057625117	0.011964928	-4.839495828	1.30E-06	17638
genus.Faecalibacterium.id.2057	rs75499067	16	6099863	C	T	0.227584711	0.046552213	4.778682048	1.76E-06	4526
genus.Faecalibacterium.id.2057	rs28376661	15	47404021	C	G	0.05048173	0.010936797	4.629554351	3.66E-06	18087
genus.Faecalibacterium.id.2057	rs10927394	1	245267339	G	T	-0.232262107	0.051236432	-4.493116132	7.02E-06	3495
genus.Faecalibacterium.id.2057	rs114946999	2	37125235	C	T	-0.086156499	0.018960043	-4.537380107	5.70E-06	15673
genus.Faecalibacterium.id.2057	rs79656633	1	111036827	T	C	0.145630704	0.032301873	4.461584899	8.14E-06	9080
genus.Faecalibacterium.id.2057	rs61875484	10	128614990	C	G	0.081795263	0.018369023	4.435634426	9.18E-06	16718
genus.Faecalibacterium.id.2057	rs12753492	1	14201546	A	C	0.064130474	0.014993333	4.444668918	8.80E-06	17525
genus.Faecalibacterium.id.2057	rs2835874	21	39027901	T	C	-0.086635636	0.019649581	-4.477731278	7.54E-06	17567
genus.Faecalibacterium.id.2057	rs11776390	8	38593645	T	C	-0.078353971	0.017183046	-4.512832573	6.40E-06	16718
genus.FamilyXIIIAD3011group.id.11293	rs62200412	20	4618639	C	T	-0.080085132	0.016383437	-4.997687579	5.80E-07	13661
genus.FamilyXIIIAD3011group.id.11293	rs16840310	1	240694163	A	G	-0.060805195	0.012214901	-4.9683893	6.75E-07	14201
genus.FamilyXIIIAD3011group.id.11293	rs72730932	1	192019835	C	A	-0.089955866	0.017710652	-4.964504641	6.89E-07	13102
genus.FamilyXIIIAD3011group.id.11293	rs16940167	15	58683697	C	T	0.073255157	0.015989797	4.616196257	3.91E-06	14201
genus.FamilyXIIIAD3011group.id.11293	rs17156849	7	28603985	G	A	-0.112890921	0.024529236	-4.601643225	4.19E-06	13352

Gut	SNP	CHR	POS	A1	A2	BETA	SE	Z	P	N
genus.FamilyXIIIAD3011group.id.11293	rs62029761	16	16006242	A	G	0.128752725	0.027600154	4.61713025	3.89E-06	9655
genus.FamilyXIIIAD3011group.id.11293	rs1848458	3	3556533	A	G	0.068746194	0.015053281	4.480569145	7.44E-06	14195
genus.FamilyXIIIAD3011group.id.11293	rs12812672	12	18127597	T	C	-0.096065015	0.020828023	-4.703445755	2.56E-06	13565
genus.FamilyXIIIAD3011group.id.11293	rs9276029	6	32695336	A	G	-0.081138392	0.018566861	-4.441638126	8.93E-06	13566
genus.FamilyXIIIAD3011group.id.11293	rs12911842	15	40047706	A	T	-0.081197635	0.018333544	-4.496496258	6.91E-06	14125
genus.FamilyXIIIAD3011group.id.11293	rs149302	5	14159925	T	C	-0.064563538	0.014322495	-4.47950014	7.48E-06	14192
genus.FamilyXIIIAD3011group.id.11293	rs9837139	3	29811865	A	G	0.10752022	0.024048177	4.446992751	8.71E-06	13527
genus.FamilyXIIIAD3011group.id.11293	rs739451	9	136678954	C	T	0.064958894	0.014753414	4.468470285	7.88E-06	14197
genus.FamilyXIIIAD3011group.id.11293	rs11126423	2	74195551	C	T	0.090439883	0.019630134	4.52949466	5.91E-06	13735
genus.FamilyXIIIUCG001.id.11294	rs12049454	1	85228325	T	C	-0.064729775	0.013406537	-4.860013185	1.17E-06	12748
genus.FamilyXIIIUCG001.id.11294	rs3842897	1	183503664	G	A	-0.112639326	0.024275096	-4.556671811	5.20E-06	12407
genus.FamilyXIIIUCG001.id.11294	rs116979587	15	25151194	T	A	-0.121678937	0.02608207	-4.667667498	3.05E-06	11989
genus.FamilyXIIIUCG001.id.11294	rs60069152	3	188211909	G	C	-0.074205843	0.015793894	-4.653796817	3.26E-06	12738
genus.FamilyXIIIUCG001.id.11294	rs62414802	6	76598635	C	T	-0.061190403	0.013456987	-4.596726133	4.29E-06	12750
genus.FamilyXIIIUCG001.id.11294	rs2276529	21	45153079	C	G	-0.075940605	0.016509926	-4.550306947	5.36E-06	12748
genus.FamilyXIIIUCG001.id.11294	rs112362903	17	33761027	A	G	-0.149047048	0.033327141	-4.468480964	7.88E-06	8579
genus.FamilyXIIIUCG001.id.11294	rs8076666	17	78223817	A	G	0.088655005	0.019809411	4.464694658	8.02E-06	12265
genus.FamilyXIIIUCG001.id.11294	rs76463770	3	45479090	A	G	0.193129687	0.04198659	4.62341379	3.77E-06	5651
genus.Flavonifractor.id.2059	rs12030302	1	77887835	A	G	-0.069227582	0.013746695	-5.004235978	5.61E-07	10805
genus.Flavonifractor.id.2059	rs806808	10	32381186	T	C	0.0667272	0.013655748	4.859013418	1.18E-06	10801
genus.Flavonifractor.id.2059	rs12790684	11	44844949	A	G	0.076003353	0.016080212	4.769074593	1.85E-06	10708
genus.Flavonifractor.id.2059	rs11811696	1	237351354	T	C	-0.116062715	0.024101172	-4.746129014	2.07E-06	10221
genus.Flavonifractor.id.2059	rs11642826	16	6696929	G	C	0.146860461	0.032519094	4.504627546	6.65E-06	8151
genus.Flavonifractor.id.2059	rs114873521	5	168218403	C	T	-0.130066614	0.029408684	-4.489913129	7.13E-06	9156
genus.Flavonifractor.id.2059	rs6761463	2	50201547	G	C	-0.083391953	0.018459615	-4.462285246	8.11E-06	10805
genus.Flavonifractor.id.2059	rs798674	16	96987	G	C	0.063712527	0.014160814	4.493011807	7.02E-06	10583
genus.Flavonifractor.id.2059	rs12038887	1	102287083	C	G	0.094271903	0.021164606	4.431176653	9.37E-06	10583
genus.Fusicatenibacter.id.11305	rs4378146	1	24927625	A	C	-0.061669056	0.012525884	-4.955768058	7.20E-07	17384
genus.Fusicatenibacter.id.11305	rs62353480	5	29595000	A	G	-0.070139694	0.014558884	-4.802409463	1.57E-06	16119
genus.Fusicatenibacter.id.11305	rs704418	3	64252803	T	C	0.073912422	0.015107283	4.940950109	7.77E-07	16626
genus.Fusicatenibacter.id.11305	rs2132128	8	15360718	G	A	-0.07719139	0.016034448	-4.877046852	1.08E-06	17370

Gut	SNP	CHR	POS	A1	A2	BETA	SE	Z	P	N
genus.Fusicatenibacter.id.11305	rs206581	18	10370596	A	G	-0.056833856	0.012789397	-4.440918842	8.96E-06	17384
genus.Fusicatenibacter.id.11305	rs62187631	2	226649570	T	C	-0.07105711	0.015924014	-4.584751752	4.55E-06	16522
genus.Fusicatenibacter.id.11305	rs2025938	10	112177119	G	A	-0.096729791	0.020542557	-4.671621223	2.99E-06	15626
genus.Fusicatenibacter.id.11305	rs7069626	10	125464544	T	C	0.050901437	0.011138084	4.547980153	5.42E-06	17384
genus.Fusicatenibacter.id.11305	rs3303	10	120447429	T	C	-0.095365713	0.020406992	-4.614579817	3.94E-06	16877
genus.Fusicatenibacter.id.11305	rs2039204	9	130371107	T	A	-0.04974145	0.010794137	-4.614428916	3.94E-06	17383
genus.Fusicatenibacter.id.11305	rs8028026	15	88353215	A	G	-0.079214582	0.018050473	-4.463496386	8.06E-06	16522
genus.Fusicatenibacter.id.11305	rs792108	2	5532793	T	C	-0.050834518	0.011384326	-4.452304706	8.50E-06	16978
genus.Fusicatenibacter.id.11305	rs60254196	7	148856720	A	G	-0.049242701	0.010936491	-4.546004306	5.47E-06	16978
genus.Fusicatenibacter.id.11305	rs9905659	17	70697344	G	A	-0.06161703	0.013657585	-4.484458612	7.31E-06	17384
genus.Fusicatenibacter.id.11305	rs8063430	16	73821805	T	C	-0.104026455	0.022216952	-4.56779135	4.93E-06	15552
genus.Fusicatenibacter.id.11305	rs6515626	20	25257001	G	A	0.141575028	0.031355872	4.485142019	7.29E-06	7796
genus.Fusicatenibacter.id.11305	rs10439674	21	42681392	A	G	-0.057212907	0.013000675	-4.473780822	7.68E-06	17381
genus.Fusicatenibacter.id.11305	rs167879	20	55937366	C	T	-0.065954324	0.014876261	-4.531057137	5.87E-06	16542
genus.Fusicatenibacter.id.11305	rs73103914	12	58658311	A	G	-0.059732424	0.013445773	-4.457408721	8.30E-06	17384
genus.Gordonibacter.id.821	rs76287110	3	187450354	A	T	-0.242964921	0.046617731	-5.232220045	1.67E-07	3483
genus.Gordonibacter.id.821	rs7294633	12	29024534	C	T	0.128670034	0.024994637	5.09772311	3.44E-07	3723
genus.Gordonibacter.id.821	rs7220558	17	10148653	A	T	0.116884144	0.02348337	4.969680142	6.71E-07	3723
genus.Gordonibacter.id.821	rs71545975	7	47144505	A	G	-0.153972004	0.033891756	-4.492492523	7.04E-06	3556
genus.Gordonibacter.id.821	rs35042269	4	8142607	C	A	-0.180292677	0.040329634	-4.462298527	8.11E-06	3595
genus.Gordonibacter.id.821	rs72714787	4	136768501	C	A	0.181404779	0.037710172	4.820932085	1.43E-06	3723
genus.Gordonibacter.id.821	rs322296	7	136937189	G	A	0.178689528	0.037721957	4.6101274	4.02E-06	3595
genus.Gordonibacter.id.821	rs72939513	1	83136096	A	G	-0.213989639	0.049059194	-4.465666497	7.98E-06	3435
genus.Gordonibacter.id.821	rs3765837	1	210628449	T	G	-0.190728517	0.043360843	-4.488606546	7.17E-06	3556
genus.Gordonibacter.id.821	rs13412653	2	30158494	A	C	0.107597447	0.023922776	4.449391136	8.61E-06	3723
genus.Gordonibacter.id.821	rs117347059	7	129433205	G	C	-0.128261008	0.028512264	-4.435940163	9.17E-06	3684
genus.Gordonibacter.id.821	rs16955299	17	52957210	G	A	-0.196422874	0.043353771	-4.513714634	6.37E-06	3572
genus.Gordonibacter.id.821	rs4596722	9	88787144	A	G	0.102908274	0.023157103	4.438547392	9.06E-06	3723
genus.Gordonibacter.id.821	rs768830	7	18970607	G	A	0.149852407	0.033331454	4.47181562	7.76E-06	3723
genus.Gordonibacter.id.821	rs61934597	12	131024079	C	T	-0.172494533	0.038856681	-4.455578655	8.37E-06	3684
genus.Haemophilus.id.3698	rs12191680	6	129224488	C	G	0.106544332	0.020032321	5.256386957	1.47E-07	9115

Gut	SNP	CHR	POS	A1	A2	BETA	SE	Z	P	N
genus.Haemophilus.id.3698	rs9382510	6	55448491	C	T	-0.093521046	0.017264852	-5.388103317	7.12E-08	9117
genus.Haemophilus.id.3698	rs76022354	10	94306385	C	T	0.244637568	0.050550437	4.771310305	1.83E-06	3618
genus.Haemophilus.id.3698	rs111582866	16	48742489	G	A	-0.124265408	0.026016016	-4.843850237	1.27E-06	8370
genus.Haemophilus.id.3698	rs9895850	17	64535013	T	C	-0.192956876	0.041675519	-4.739675749	2.14E-06	4592
genus.Haemophilus.id.3698	rs35509	12	115493337	G	A	0.128248955	0.026877911	4.752464823	2.01E-06	8430
genus.Haemophilus.id.3698	rs78909003	9	105650242	T	C	-0.246255715	0.050392389	-4.790043746	1.67E-06	3559
genus.Haemophilus.id.3698	rs9328464	6	8350917	T	C	0.072309489	0.014879949	4.82179998	1.42E-06	9119
genus.Haemophilus.id.3698	rs4822728	22	26891808	T	C	0.070586099	0.015138761	4.640332606	3.48E-06	9119
genus.Haemophilus.id.3698	rs10840326	11	9951672	C	G	-0.067738403	0.015146258	-4.482848275	7.37E-06	8888
genus.Haemophilus.id.3698	rs12876183	13	85114550	T	A	0.074920105	0.016710973	4.425432981	9.62E-06	9110
genus.Haemophilus.id.3698	rs10781340	9	78752170	G	A	0.094891501	0.020322072	4.595232969	4.32E-06	9037
genus.Holdemanella.id.11393	rs761624	6	163603171	C	G	0.095980128	0.017953732	5.268190237	1.38E-07	7706
genus.Holdemanella.id.11393	rs607782	6	4148609	T	C	-0.08542142	0.017251511	-4.956039341	7.19E-07	7456
genus.Holdemanella.id.11393	rs75764681	10	4494630	T	C	-0.283102607	0.059899873	-4.759291749	1.94E-06	3003
genus.Holdemanella.id.11393	rs73011279	19	15020095	T	C	-0.096166927	0.019933645	-4.830712076	1.36E-06	7456
genus.Holdemanella.id.11393	rs4541991	9	105004835	T	C	-0.092733374	0.019443459	-4.743464035	2.10E-06	7657
genus.Holdemanella.id.11393	rs1830029	7	14728950	C	G	-0.095409594	0.021093039	-4.550679944	5.35E-06	7456
genus.Holdemanella.id.11393	rs12513188	4	71000791	G	A	0.090390629	0.019527621	4.579773231	4.65E-06	7700
genus.Holdemanella.id.11393	rs12415649	10	125021315	G	C	0.083938435	0.019056694	4.468419806	7.88E-06	7662
genus.Holdemanella.id.11393	rs17586763	13	50932063	T	C	-0.227284509	0.051013457	-4.472819549	7.72E-06	3817
genus.Holdemanella.id.11393	rs34187114	8	138623633	C	A	-0.104505235	0.022600956	-4.559197309	5.13E-06	7704
genus.Holdemanella.id.11393	rs35228298	3	85392882	G	A	0.093483215	0.020288588	4.484792442	7.30E-06	7456
genus.Holdemanella.id.11393	rs62113381	19	7668225	T	C	-0.105447171	0.023202912	-4.543271158	5.54E-06	7310
genus.Holdemania.id.2157	rs1867876	11	18768068	T	C	0.084291862	0.016219181	5.140588011	2.74E-07	9109
genus.Holdemania.id.2157	rs9500080	6	105737322	C	T	0.092676436	0.01788862	5.064946612	4.09E-07	9106
genus.Holdemania.id.2157	rs6133067	20	3839754	T	C	0.091082873	0.017856087	5.019920458	5.17E-07	8863
genus.Holdemania.id.2157	rs150096134	12	39589195	T	A	0.162139279	0.033208945	4.718348633	2.38E-06	6906
genus.Holdemania.id.2157	rs41438744	14	102553414	C	G	-0.124870498	0.02701295	-4.712718515	2.44E-06	8839
genus.Holdemania.id.2157	rs77293403	5	77388406	A	G	0.164556483	0.034177476	4.777524727	1.77E-06	7585
genus.Holdemania.id.2157	rs80149660	10	129908282	C	T	-0.23298738	0.051918311	-4.524908141	6.04E-06	3283
genus.Holdemania.id.2157	rs116500994	13	81685543	G	T	-0.137550456	0.029338596	-4.721181527	2.34E-06	8432

Gut	SNP	CHR	POS	A1	A2	BETA	SE	Z	P	N
genus.Holdemania.id.2157	rs55888180	17	76443497	C	G	0.128670991	0.028279464	4.53023046	5.89E-06	8464
genus.Holdemania.id.2157	rs4146507	5	106209093	C	T	0.079488043	0.017700231	4.486810483	7.23E-06	9108
genus.Holdemania.id.2157	rs111745969	15	93110222	A	G	0.120676888	0.026578103	4.627125456	3.71E-06	8233
genus.Holdemania.id.2157	rs113593397	8	123973840	A	G	-0.128934656	0.028252607	-4.431516217	9.36E-06	8296
genus.Holdemania.id.2157	rs10885477	10	115332413	T	C	-0.13513664	0.030189444	-4.449717463	8.60E-06	8690
genus.Holdemania.id.2157	rs9529719	13	70856516	T	C	0.074037972	0.016049433	4.527566289	5.97E-06	9107
genus.Holdemania.id.2157	rs967319	3	60615680	T	C	0.078863839	0.017674402	4.45519873	8.38E-06	9108
genus.Holdemania.id.2157	rs12701617	7	38292857	A	G	-0.066061737	0.014942681	-4.427833287	9.52E-06	9106
genus.Holdemania.id.2157	rs73139538	7	63323145	G	A	-0.148593595	0.032746911	-4.471376459	7.77E-06	8095
genus.Howardella.id.2000	rs901099	11	77901287	T	G	-0.127180025	0.025092403	-4.974886839	6.53E-07	3647
genus.Howardella.id.2000	rs1484873	18	43206985	A	G	-0.227831171	0.046335026	-4.703357613	2.56E-06	3608
genus.Howardella.id.2000	rs17167098	7	133154356	G	A	-0.16936566	0.035206781	-4.868720876	1.12E-06	3586
genus.Howardella.id.2000	rs609430	4	169178315	T	G	-0.112045365	0.023932786	-4.648422063	3.34E-06	3798
genus.Howardella.id.2000	rs36081916	7	93527414	T	C	-0.181229725	0.040298718	-4.577733747	4.70E-06	3216
genus.Howardella.id.2000	rs12452946	17	17253288	A	G	-0.105833103	0.022894084	-4.621817368	3.80E-06	3800
genus.Howardella.id.2000	rs61771805	1	72972799	A	T	-0.136778682	0.029681112	-4.60996354	4.03E-06	3612
genus.Howardella.id.2000	rs672217	18	60125134	G	A	0.164145673	0.034999266	4.637606172	3.52E-06	3611
genus.Howardella.id.2000	rs3791893	2	218819396	A	G	0.147035334	0.034023014	4.428316227	9.50E-06	3795
genus.Howardella.id.2000	rs10048062	15	98476920	C	T	-0.14735223	0.033653121	-4.449957401	8.59E-06	3800
genus.Howardella.id.2000	rs2154047	14	95452797	C	A	-0.192557008	0.042009328	-4.417813912	9.97E-06	3623
genus.Hungatella.id.11306	rs13128780	4	166058998	T	C	-0.149725257	0.031277743	-4.780341366	1.75E-06	3999
genus.Hungatella.id.11306	rs72759041	15	89577376	G	T	-0.126025023	0.028224238	-4.618730264	3.86E-06	3910
genus.Hungatella.id.11306	rs10044993	5	58424440	C	A	0.139546965	0.031674777	4.463406477	8.07E-06	4209
genus.Hungatella.id.11306	rs13249325	8	14853525	T	G	-0.100022583	0.022588331	-4.42394161	9.69E-06	4209
genus.Hungatella.id.11306	rs17092615	14	95973619	G	A	0.152234676	0.033786729	4.482517271	7.38E-06	4009
genus.Intestinibacter.id.11345	rs10805326	4	14324623	G	A	0.077515061	0.013966468	5.512116543	3.55E-08	12303
genus.Intestinibacter.id.11345	rs4327025	15	92446683	G	A	-0.081034559	0.015439666	-5.235739105	1.64E-07	12303
genus.Intestinibacter.id.11345	rs478972	11	125663184	T	C	-0.142680973	0.02971163	-4.772458413	1.82E-06	10333
genus.Intestinibacter.id.11345	rs6875660	5	159686256	C	T	0.089045542	0.01939052	4.666897941	3.06E-06	12293
genus.Intestinibacter.id.11345	rs16938435	9	21502923	T	C	-0.112188506	0.023543887	-4.774564334	1.80E-06	12303
genus.Intestinibacter.id.11345	rs6062862	20	61325223	A	G	0.092450812	0.020465817	4.503643086	6.68E-06	12189

Gut	SNP	CHR	POS	A1	A2	BETA	SE	Z	P	N
genus.Intestinibacter.id.11345	rs118030283	16	5993076	G	A	-0.15182897	0.032447117	-4.694362896	2.67E-06	8830
genus.Intestinibacter.id.11345	rs11109097	12	97928437	C	T	0.062427238	0.013853923	4.544949516	5.49E-06	11886
genus.Intestinibacter.id.11345	rs447950	5	148846426	A	G	0.062834174	0.01366523	4.539487625	5.64E-06	12303
genus.Intestinibacter.id.11345	rs62430350	6	170918194	T	C	0.151095721	0.035147611	4.498536496	6.84E-06	7650
genus.Intestinibacter.id.11345	rs2702387	4	179361262	A	G	0.060855918	0.013214549	4.598279392	4.26E-06	12291
genus.Intestinibacter.id.11345	rs68093214	3	70657785	C	T	0.066225756	0.014987729	4.433822427	9.26E-06	11886
genus.Intestinibacter.id.11345	rs2098844	11	127703160	C	T	-0.057529528	0.012841562	-4.500205306	6.79E-06	12291
genus.Intestinimonas.id.2062	rs11258178	10	13124513	A	G	0.066074232	0.013413886	4.9618651	6.98E-07	11766
genus.Intestinimonas.id.2062	rs12226153	11	94364293	A	G	-0.151142451	0.030692229	-5.021696536	5.12E-07	9023
genus.Intestinimonas.id.2062	rs4784055	16	58818770	T	C	-0.175323954	0.038599023	-4.918564477	8.72E-07	3193
genus.Intestinimonas.id.2062	rs716604	2	6514088	A	G	0.081806835	0.016599238	4.922002693	8.57E-07	12090
genus.Intestinimonas.id.2062	rs2930225	16	85327902	G	T	0.072952384	0.015294848	4.832806136	1.35E-06	12003
genus.Intestinimonas.id.2062	rs62240188	3	10611142	G	A	0.130073573	0.026717884	4.733843275	2.20E-06	10252
genus.Intestinimonas.id.2062	rs2731794	5	17209391	C	T	0.120632118	0.025752925	4.761936856	1.92E-06	11079
genus.Intestinimonas.id.2062	rs7170984	15	93819735	T	C	-0.06581157	0.014076733	-4.671948452	2.98E-06	12090
genus.Intestinimonas.id.2062	rs12566247	1	43767569	T	A	0.063708441	0.013535548	4.73500139	2.19E-06	12076
genus.Intestinimonas.id.2062	rs1859797	7	22007303	G	A	0.060367955	0.013179253	4.605116664	4.12E-06	11766
genus.Intestinimonas.id.2062	rs17067892	8	3763540	C	T	0.107193792	0.025001344	4.51341299	6.38E-06	11544
genus.Intestinimonas.id.2062	rs72982915	2	140898088	C	T	0.183157909	0.040274272	4.568529929	4.91E-06	5591
genus.Intestinimonas.id.2062	rs994794	4	114608814	G	C	-0.141904818	0.031507362	-4.484601713	7.31E-06	9250
genus.Intestinimonas.id.2062	rs4113676	20	6218922	A	C	-0.218506906	0.049015185	-4.481375353	7.42E-06	3170
genus.Intestinimonas.id.2062	rs9823439	3	17187502	T	C	-0.058194711	0.013145465	-4.420248216	9.86E-06	11766
genus.Intestinimonas.id.2062	rs62427239	6	155737906	C	A	0.162658132	0.036764706	4.430418177	9.41E-06	6385
genus.Intestinimonas.id.2062	rs2276760	3	150620956	A	G	-0.06852335	0.015254563	-4.469490227	7.84E-06	12081
genus.Intestinimonas.id.2062	rs6934519	6	67200802	C	T	0.069237276	0.015115352	4.450437217	8.57E-06	12089
genus.Intestinimonas.id.2062	rs1000888	13	23343542	C	G	-0.058503766	0.01321901	-4.423466051	9.71E-06	12090
genus.Lachnoclostridium.id.11308	rs6112314	20	19300846	A	C	-0.056171476	0.010817419	-5.162854285	2.43E-07	17842
genus.Lachnoclostridium.id.11308	rs62285313	3	177470032	A	G	0.086420325	0.018156544	4.800423112	1.58E-06	16991
genus.Lachnoclostridium.id.11308	rs615997	3	23037786	T	C	0.05117524	0.010649056	4.750736295	2.03E-06	17911
genus.Lachnoclostridium.id.11308	rs61915992	12	28169306	A	T	0.080387577	0.01722087	4.694492331	2.67E-06	16773
genus.Lachnoclostridium.id.11308	rs789029	18	1053252	C	T	-0.06412885	0.013797406	-4.624596497	3.75E-06	17922

Gut	SNP	CHR	POS	A1	A2	BETA	SE	Z	P	N
genus.Lachnoclostridium.id.11308	rs12566975	1	185091351	T	C	-0.04680969	0.010578675	-4.426621573	9.57E-06	17921
genus.Lachnoclostridium.id.11308	rs4738679	8	59370320	G	A	-0.052026675	0.011404049	-4.590703569	4.42E-06	17921
genus.Lachnoclostridium.id.11308	rs1031599	3	66725825	G	T	-0.078627049	0.017564435	-4.515600322	6.31E-06	17398
genus.Lachnoclostridium.id.11308	rs72829893	17	46694541	G	T	0.117472377	0.026810315	4.541797525	5.58E-06	12404
genus.Lachnoclostridium.id.11308	rs3821998	4	38694566	C	A	-0.0864066	0.019251946	-4.502352979	6.72E-06	17920
genus.Lachnoclostridium.id.11308	rs2385421	18	19743455	A	G	0.074618572	0.018073408	4.48955627	7.14E-06	17903
genus.Lachnoclostridium.id.11308	rs1528479	2	167244051	G	A	-0.049779849	0.011191926	-4.425100004	9.64E-06	17477
genus.Lachnoclostridium.id.11308	rs1997204	12	102046595	T	C	-0.108074801	0.024202203	-4.527420314	5.97E-06	14138
genus.Lachnoclostridium.id.11308	rs62028349	16	27217620	G	C	0.046998893	0.010597086	4.435862732	9.17E-06	17922
genus.Lachnospira.id.2004	rs13157098	5	176802250	A	G	-0.076805837	0.015531203	-4.991437684	5.99E-07	15947
genus.Lachnospira.id.2004	rs4923324	11	25962890	G	A	-0.061732096	0.013338657	-4.713420453	2.44E-06	16490
genus.Lachnospira.id.2004	rs56791201	2	57701660	T	C	0.051822993	0.011069673	4.67579098	2.93E-06	16486
genus.Lachnospira.id.2004	rs2326833	6	6893668	C	G	-0.078495035	0.017109015	-4.582409926	4.60E-06	15632
genus.Lachnospira.id.2004	rs4686798	3	186445436	T	C	0.053182605	0.011374743	4.689453615	2.74E-06	16497
genus.Lachnospira.id.2004	rs159484	4	111995627	G	A	0.079466817	0.017699872	4.503757973	6.68E-06	16487
genus.Lachnospira.id.2004	rs2520509	12	91006514	A	G	0.051906257	0.011577959	4.481312464	7.42E-06	16500
genus.LachnospiraceaeFCS020group.id.11314	rs7249113	19	2571232	G	A	0.067948308	0.013349587	5.08270544	3.72E-07	13534
genus.LachnospiraceaeFCS020group.id.11314	rs12078956	1	35328576	C	G	0.106101007	0.022290421	4.738382735	2.15E-06	13101
genus.LachnospiraceaeFCS020group.id.11314	rs369444	16	23631307	C	G	0.125481334	0.025900786	4.660902367	3.15E-06	12465
genus.LachnospiraceaeFCS020group.id.11314	rs72793667	2	53946431	A	G	-0.116880798	0.024658906	-4.794082291	1.63E-06	12396
genus.LachnospiraceaeFCS020group.id.11314	rs1363769	19	17865305	T	C	-0.200628437	0.044937217	-4.800635392	1.58E-06	3555
genus.LachnospiraceaeFCS020group.id.11314	rs10093861	8	121244406	G	A	-0.056886893	0.012115188	-4.667003415	3.06E-06	13772
genus.LachnospiraceaeFCS020group.id.11314	rs9788306	13	40419314	C	T	-0.062803973	0.013074422	-4.826981479	1.39E-06	13772
genus.LachnospiraceaeFCS020group.id.11314	rs113859143	5	109403564	G	C	-0.108906331	0.024205472	-4.70373063	2.55E-06	13238
genus.LachnospiraceaeFCS020group.id.11314	rs9919338	1	118425334	G	C	-0.055333855	0.012062109	-4.568739198	4.91E-06	13768
genus.LachnospiraceaeFCS020group.id.11314	rs35035870	11	3248876	T	C	-0.190615272	0.041440269	-4.698539696	2.62E-06	5642
genus.LachnospiraceaeFCS020group.id.11314	rs2862811	3	165996415	T	C	0.056492728	0.012173604	4.615626599	3.92E-06	13772
genus.LachnospiraceaeFCS020group.id.11314	rs1254846	10	44346094	G	A	0.105962631	0.023250118	4.541081592	5.60E-06	13172
genus.LachnospiraceaeFCS020group.id.11314	rs4452603	6	71966268	T	G	0.060420825	0.013596453	4.440334783	8.98E-06	13487
genus.LachnospiraceaeFCS020group.id.11314	rs2322265	4	166588085	C	T	-0.0666305	0.014157654	-4.556226164	5.21E-06	13772
genus.LachnospiraceaeFCS020group.id.11314	rs3999074	10	65563647	G	T	-0.05505865	0.012184677	-4.507795194	6.55E-06	13772

Gut	SNP	CHR	POS	A1	A2	BETA	SE	Z	P	N
genus.LachnospiraceaeFCS020group.id.11314	rs9308097	4	165786478	A	G	0.05538101	0.012365944	4.479754313	7.47E-06	13542
genus.LachnospiraceaeNC2004group.id.11316	rs6116753	20	5330700	G	A	0.099474834	0.020913933	4.67619259	2.92E-06	6278
genus.LachnospiraceaeNC2004group.id.11316	rs3756315	5	149544722	A	G	-0.088346946	0.01883968	-4.649597957	3.33E-06	6328
genus.LachnospiraceaeNC2004group.id.11316	rs12127733	1	234157721	G	A	0.115181538	0.024600625	4.663687082	3.11E-06	6226
genus.LachnospiraceaeNC2004group.id.11316	rs1331592	9	122239986	C	G	0.094880626	0.02083688	4.550825343	5.34E-06	6328
genus.LachnospiraceaeNC2004group.id.11316	rs1928659	9	29334137	T	C	0.102522036	0.022644542	4.520395869	6.17E-06	5516
genus.LachnospiraceaeNC2004group.id.11316	rs1929743	13	76829562	T	C	0.083720631	0.019031847	4.43858802	9.06E-06	6330
genus.LachnospiraceaeNC2004group.id.11316	rs12863463	13	47512191	G	A	-0.156364942	0.03453716	-4.524989997	6.04E-06	5516
genus.LachnospiraceaeNC2004group.id.11316	rs117467633	17	21157406	T	C	-0.169687489	0.038316857	-4.436739439	9.13E-06	5077
genus.LachnospiraceaeNC2004group.id.11316	rs12208226	6	22725908	C	A	-0.154739126	0.034037233	-4.422680174	9.75E-06	5620
genus.LachnospiraceaeND3007group.id.11317	rs9932954	16	1100633	A	G	-0.056186355	0.011598415	-4.84773098	1.25E-06	15222
genus.LachnospiraceaeND3007group.id.11317	rs13110238	4	7678735	C	G	-0.063697828	0.01420413	-4.587862306	4.48E-06	15019
genus.LachnospiraceaeND3007group.id.11317	rs72776675	10	14137663	T	C	-0.064706741	0.014797022	-4.446775304	8.72E-06	15302
genus.LachnospiraceaeND3007group.id.11317	rs2861203	3	172950553	G	A	0.057230395	0.012727879	4.482733	7.37E-06	15305
genus.LachnospiraceaeNK4A136group.id.11319	rs12362320	11	3922055	G	C	0.057316768	0.011546623	4.934339592	8.04E-07	16764
genus.LachnospiraceaeNK4A136group.id.11319	rs954878	1	54578401	A	G	-0.052065624	0.010908221	-4.776691487	1.78E-06	17227
genus.LachnospiraceaeNK4A136group.id.11319	rs7832116	8	4842929	A	G	-0.071475594	0.015170307	-4.634915098	3.57E-06	17219
genus.LachnospiraceaeNK4A136group.id.11319	rs7616165	3	190415145	G	T	-0.230541982	0.048346456	-4.686887823	2.77E-06	4094
genus.LachnospiraceaeNK4A136group.id.11319	rs76193507	3	161749981	A	G	-0.229730157	0.049977561	-4.675603662	2.93E-06	3578
genus.LachnospiraceaeNK4A136group.id.11319	rs11263806	17	35240097	A	G	-0.052461533	0.011675801	-4.562070317	5.07E-06	17236
genus.LachnospiraceaeNK4A136group.id.11319	rs73044693	19	51256120	A	G	-0.107579587	0.022988374	-4.634815689	3.57E-06	12773
genus.LachnospiraceaeNK4A136group.id.11319	rs160061	5	6116659	A	G	0.051383089	0.010809407	4.741446355	2.12E-06	17234
genus.LachnospiraceaeNK4A136group.id.11319	rs7073658	10	62175024	T	G	-0.049962145	0.010969284	-4.553778009	5.27E-06	17236
genus.LachnospiraceaeNK4A136group.id.11319	rs68104925	14	100177681	T	C	-0.054910353	0.011538497	-4.719195356	2.37E-06	17227
genus.LachnospiraceaeNK4A136group.id.11319	rs12611395	19	21806125	A	G	-0.090253198	0.019965236	-4.532448475	5.83E-06	16486
genus.LachnospiraceaeNK4A136group.id.11319	rs2880566	17	30016653	T	C	0.059957809	0.013469261	4.540418215	5.61E-06	17232
genus.LachnospiraceaeNK4A136group.id.11319	rs59805249	5	90289997	T	C	0.09361625	0.020798243	4.429302236	9.45E-06	15195
genus.LachnospiraceaeNK4A136group.id.11319	rs10952110	7	8497922	G	T	0.048770868	0.010961435	4.437969979	9.08E-06	16764
genus.LachnospiraceaeNK4A136group.id.11319	rs4955932	3	55181375	T	C	-0.049228303	0.010938782	-4.492044633	7.05E-06	17236
genus.LachnospiraceaeUCG001.id.11321	rs985416	3	148269083	C	T	0.097025845	0.018180694	5.258050154	1.46E-07	9917
genus.LachnospiraceaeUCG001.id.11321	rs16810	3	42571729	C	T	0.068356504	0.014067088	4.84691034	1.25E-06	10150

Gut	SNP	CHR	POS	A1	A2	BETA	SE	Z	P	N
genus.LachnospiraceaeUCG001.id.11321	rs2050911	1	82384407	G	A	0.075146621	0.015393431	4.871689806	1.11E-06	10148
genus.LachnospiraceaeUCG001.id.11321	rs10815577	9	7273249	C	G	-0.06837061	0.014369051	-4.784395447	1.72E-06	10149
genus.LachnospiraceaeUCG001.id.11321	rs573933	9	14477851	T	C	-0.107898495	0.023234631	-4.663743895	3.11E-06	10150
genus.LachnospiraceaeUCG001.id.11321	rs78848836	1	53773248	A	G	-0.118867977	0.025975321	-4.646159792	3.38E-06	9506
genus.LachnospiraceaeUCG001.id.11321	rs9403580	6	145006780	C	T	0.107800527	0.022977722	4.640965671	3.47E-06	10095
genus.LachnospiraceaeUCG001.id.11321	rs7213933	17	36155074	T	A	-0.081626876	0.018380006	-4.439502529	9.02E-06	9961
genus.LachnospiraceaeUCG001.id.11321	rs8104225	19	13962924	A	G	0.089209817	0.019766483	4.464058598	8.04E-06	9908
genus.LachnospiraceaeUCG001.id.11321	rs74034332	16	79092359	G	A	0.168045475	0.038263689	4.649121741	3.33E-06	6004
genus.LachnospiraceaeUCG001.id.11321	rs62496417	7	96965504	T	G	-0.074850403	0.016568056	-4.530837121	5.88E-06	10150
genus.LachnospiraceaeUCG001.id.11321	rs2371284	12	56256262	T	C	-0.076189302	0.017012675	-4.477289554	7.56E-06	9961
genus.LachnospiraceaeUCG001.id.11321	rs12131224	1	166199362	C	T	0.117095415	0.025909955	4.481885986	7.40E-06	9676
genus.LachnospiraceaeUCG001.id.11321	rs4981345	14	21455973	T	C	-0.068197297	0.014983138	-4.523380963	6.09E-06	9961
genus.LachnospiraceaeUCG001.id.11321	rs79476906	1	108102470	T	A	-0.087088012	0.019700842	-4.457962108	8.27E-06	9917
genus.LachnospiraceaeUCG001.id.11321	rs7341608	8	56839500	T	C	-0.078475713	0.017764768	-4.428678035	9.48E-06	9917
genus.LachnospiraceaeUCG004.id.11324	rs12747809	1	240359089	G	A	-0.062197002	0.012568463	-4.920128327	8.65E-07	14464
genus.LachnospiraceaeUCG004.id.11324	rs2882478	2	49904589	G	A	-0.057709061	0.011833839	-4.854302561	1.21E-06	14466
genus.LachnospiraceaeUCG004.id.11324	rs12673420	7	71303007	G	A	0.055435065	0.011837515	4.672138824	2.98E-06	14463
genus.LachnospiraceaeUCG004.id.11324	rs2444793	6	81318836	C	T	-0.054243345	0.011818754	-4.574624187	4.77E-06	14463
genus.LachnospiraceaeUCG004.id.11324	rs2726805	4	183247601	A	G	0.054839706	0.01208444	4.515928673	6.30E-06	14466
genus.LachnospiraceaeUCG004.id.11324	rs11128180	3	70592215	A	G	0.06482876	0.014012621	4.586037664	4.52E-06	14466
genus.LachnospiraceaeUCG004.id.11324	rs12202257	6	78609654	G	A	-0.055087554	0.01241856	-4.492816006	7.03E-06	14448
genus.LachnospiraceaeUCG004.id.11324	rs12894272	14	40536481	A	G	0.057979201	0.012522898	4.594422829	4.34E-06	14463
genus.LachnospiraceaeUCG004.id.11324	rs233486	6	14817584	A	G	-0.079908769	0.017765984	-4.516582325	6.28E-06	13710
genus.LachnospiraceaeUCG004.id.11324	rs35182105	12	24572337	A	G	-0.109724303	0.024221006	-4.570523162	4.87E-06	13170
genus.LachnospiraceaeUCG004.id.11324	rs12072562	1	105287469	T	C	0.133103727	0.030356565	4.491458891	7.07E-06	9176
genus.LachnospiraceaeUCG004.id.11324	rs2706242	13	51292902	G	C	-0.09013993	0.019971219	-4.420693127	9.84E-06	14026
genus.LachnospiraceaeUCG004.id.11324	rs7629954	3	154242406	A	G	0.108440892	0.023824987	4.534565512	5.77E-06	12568
genus.LachnospiraceaeUCG008.id.11328	rs10793103	11	74391775	C	T	0.097434405	0.018127922	5.338935868	9.35E-08	7014
genus.LachnospiraceaeUCG008.id.11328	rs9873555	3	100496680	G	C	-0.121161831	0.023346742	-5.164297987	2.41E-07	6577
genus.LachnospiraceaeUCG008.id.11328	rs13024781	2	168628871	T	C	-0.079876878	0.016884555	-4.725839742	2.29E-06	7016
genus.LachnospiraceaeUCG008.id.11328	rs10741777	11	19572557	T	C	-0.097384219	0.019479136	-4.943161044	7.69E-07	7014

Gut	SNP	CHR	POS	A1	A2	BETA	SE	Z	P	N
genus.LachnospiraceaeUCG008.id.11328	rs955844	16	84992314	A	C	0.112064162	0.022836934	4.773564368	1.81E-06	6863
genus.LachnospiraceaeUCG008.id.11328	rs67078837	4	114220647	T	C	-0.08458483	0.017069269	-4.943280372	7.68E-07	7016
genus.LachnospiraceaeUCG008.id.11328	rs62277846	2	228100140	C	T	0.102292484	0.021231902	4.799715062	1.59E-06	7016
genus.LachnospiraceaeUCG008.id.11328	rs10801803	1	90818872	G	A	-0.117029089	0.024308454	-4.824962726	1.40E-06	7017
genus.LachnospiraceaeUCG008.id.11328	rs61944774	12	129539818	A	G	0.179838121	0.039379746	4.514680675	6.34E-06	6000
genus.LachnospiraceaeUCG008.id.11328	rs75356640	15	31697284	G	A	0.136523454	0.030312828	4.420834197	9.83E-06	6423
genus.LachnospiraceaeUCG008.id.11328	rs57091572	6	81214803	A	G	-0.110442734	0.023580145	-4.680753515	2.86E-06	6950
genus.LachnospiraceaeUCG010.id.11330	rs11192447	10	83848397	A	G	0.126592141	0.024345002	5.038596695	4.69E-07	12695
genus.LachnospiraceaeUCG010.id.11330	rs9981767	21	44079875	A	C	0.065506222	0.01319942	4.892353371	9.96E-07	12816
genus.LachnospiraceaeUCG010.id.11330	rs74315802	14	29872341	G	T	0.086726244	0.018347717	4.658186968	3.19E-06	12715
genus.LachnospiraceaeUCG010.id.11330	rs12346653	9	92267239	C	T	0.065768558	0.013956927	4.692171283	2.70E-06	12816
genus.LachnospiraceaeUCG010.id.11330	rs10414815	19	42586245	T	C	0.104529604	0.023025257	4.599268724	4.24E-06	11564
genus.LachnospiraceaeUCG010.id.11330	rs72761829	1	224897491	A	T	0.111883594	0.023889286	4.70197973	2.58E-06	11488
genus.LachnospiraceaeUCG010.id.11330	rs72894957	2	185862591	G	A	0.222262976	0.048642511	4.537805014	5.68E-06	4316
genus.LachnospiraceaeUCG010.id.11330	rs336138	5	8146371	G	T	0.077954271	0.017186539	4.479432744	7.48E-06	13236
genus.LachnospiraceaeUCG010.id.11330	rs4576377	7	81410263	A	C	-0.057200131	0.012704307	-4.475295899	7.63E-06	13225
genus.LachnospiraceaeUCG010.id.11330	rs2833528	21	33193567	C	T	-0.056214807	0.012776299	-4.418912003	9.92E-06	13233
genus.LachnospiraceaeUCG010.id.11330	rs2153460	9	85576178	A	T	-0.068403784	0.015656159	-4.435799414	9.17E-06	13236
genus.LachnospiraceaeUCG010.id.11330	rs17730011	6	84772845	G	A	-0.070235733	0.015706022	-4.469344339	7.85E-06	13226
genus.Lactobacillus.id.1837	rs921925	19	6928017	A	C	0.098507745	0.020322897	4.897291713	9.72E-07	6952
genus.Lactobacillus.id.1837	rs16861661	1	18501459	G	A	-0.183146977	0.038148013	-4.843130105	1.28E-06	5325
genus.Lactobacillus.id.1837	rs768253	8	69010103	T	G	-0.079195015	0.017179101	-4.59887935	4.25E-06	6958
genus.Lactobacillus.id.1837	rs11674854	2	28903275	C	T	-0.085268613	0.017650364	-4.799326519	1.59E-06	6655
genus.Lactobacillus.id.1837	rs328312	16	73890910	T	A	0.081509206	0.016944121	4.824126457	1.41E-06	6950
genus.Lactobacillus.id.1837	rs6092149	20	54192992	A	T	-0.080134426	0.01714896	-4.651805029	3.29E-06	6906
genus.Lactobacillus.id.1837	rs75127669	6	151027636	C	A	0.13978419	0.031041446	4.498783621	6.83E-06	6840
genus.Lactobacillus.id.1837	rs77478751	3	173151665	A	G	-0.219885755	0.047575911	-4.483959582	7.33E-06	3669
genus.Lactobacillus.id.1837	rs62314653	4	109896462	C	A	0.187691889	0.039458456	4.7301235	2.24E-06	5944
genus.Lactobacillus.id.1837	rs7399658	13	23834968	G	A	-0.107133701	0.022188263	-4.662692289	3.12E-06	6613
genus.Lactobacillus.id.1837	rs12693845	2	199312684	C	T	-0.080544718	0.017742876	-4.440783337	8.96E-06	6958
genus.Lactococcus.id.1851	rs34757988	18	64699550	G	C	0.122293892	0.022897605	5.346838382	8.95E-08	4055

Gut	SNP	CHR	POS	A1	A2	BETA	SE	Z	P	N
genus.Lactococcus.id.1851	rs757872	22	31532568	G	C	0.140799135	0.027581978	5.052142424	4.37E-07	3792
genus.Lactococcus.id.1851	rs123059	17	2699935	T	C	-0.136709792	0.02746936	-4.845077832	1.27E-06	3760
genus.Lactococcus.id.1851	rs4766997	12	113161438	C	T	0.114599246	0.023839024	4.747305209	2.06E-06	4053
genus.Lactococcus.id.1851	rs10417872	19	28767353	T	G	0.118305772	0.024521975	4.84178569	1.29E-06	4056
genus.Lactococcus.id.1851	rs6674304	1	116887742	C	T	0.200757712	0.044212138	4.520218295	6.18E-06	3869
genus.Lactococcus.id.1851	rs7992246	13	113472525	T	C	0.104232064	0.023078643	4.589305011	4.45E-06	4057
genus.Lactococcus.id.1851	rs55910161	10	71518643	C	T	0.146425945	0.030736694	4.719681972	2.36E-06	3836
genus.Lactococcus.id.1851	rs12621813	2	31266049	G	A	0.108422776	0.023997845	4.505929972	6.61E-06	4057
genus.Lactococcus.id.1851	rs2293361	2	54114864	C	T	-0.199221089	0.043096892	-4.825585551	1.40E-06	3787
genus.Lactococcus.id.1851	rs17168302	7	14650221	G	A	0.191856546	0.042476093	4.516533428	6.29E-06	4023
genus.Marvinbryantia.id.2005	rs61884471	11	45826561	G	A	0.124426313	0.024843116	4.889362021	1.01E-06	11559
genus.Marvinbryantia.id.2005	rs2724813	10	12516924	A	G	-0.084076924	0.01675508	-4.982262611	6.28E-07	11610
genus.Marvinbryantia.id.2005	rs1187983	1	58441860	C	T	-0.093545581	0.019317443	-4.750985925	2.02E-06	11896
genus.Marvinbryantia.id.2005	rs2842896	6	132881664	C	T	-0.0649396	0.013114646	-4.954446881	7.25E-07	11709
genus.Marvinbryantia.id.2005	rs11645029	16	5938448	G	C	-0.060618043	0.013155125	-4.603849493	4.15E-06	11709
genus.Marvinbryantia.id.2005	rs12963345	18	46255637	G	C	-0.05968101	0.013228853	-4.506074743	6.60E-06	11709
genus.Marvinbryantia.id.2005	rs2863363	3	166039765	A	G	0.063486062	0.013632279	4.663232508	3.11E-06	11950
genus.Marvinbryantia.id.2005	rs72948274	11	79518683	A	C	-0.126353989	0.02722118	-4.653776523	3.26E-06	11561
genus.Marvinbryantia.id.2005	rs146541147	19	4542097	G	A	0.118845391	0.02684231	4.497897525	6.86E-06	10778
genus.Marvinbryantia.id.2005	rs11620597	13	86079090	T	C	0.119479349	0.027168773	4.470552415	7.80E-06	11031
genus.Marvinbryantia.id.2005	rs8006832	14	21577837	G	T	-0.095241547	0.021669867	-4.506813791	6.58E-06	11412
genus.Marvinbryantia.id.2005	rs3125832	1	211400180	A	C	0.067932264	0.015011987	4.56364482	5.03E-06	11709
genus.Methanobrevibacter.id.123	rs76029318	13	41963791	T	C	0.22284888	0.045431939	4.877291702	1.08E-06	3387
genus.Methanobrevibacter.id.123	rs894996	4	104418307	C	A	0.214212565	0.045604463	4.621178325	3.82E-06	3479
genus.Methanobrevibacter.id.123	rs11018665	11	89349287	A	T	0.113013025	0.025434251	4.49274812	7.03E-06	3586
genus.Methanobrevibacter.id.123	rs1334944	10	112266046	T	C	0.115196507	0.025549236	4.475765656	7.61E-06	3586
genus.Methanobrevibacter.id.123	rs6776814	3	15053083	T	C	-0.188956311	0.041990543	-4.46387514	8.05E-06	3283
genus.Methanobrevibacter.id.123	rs4779844	15	31516919	G	C	0.109638988	0.024797947	4.433238402	9.28E-06	3585
genus.Methanobrevibacter.id.123	rs4802933	19	52925820	A	G	-0.135628431	0.030813872	-4.422906762	9.74E-06	3586
genus.Odoribacter.id.952	rs503751	15	58063881	C	G	0.061829433	0.011888659	5.192378238	2.08E-07	14218
genus.Odoribacter.id.952	rs77779484	12	67655943	G	A	-0.133489405	0.026852607	-4.973880963	6.56E-07	11935

Gut	SNP	CHR	POS	A1	A2	BETA	SE	Z	P	N
genus.Odoribacter.id.952	rs10423795	19	49523088	C	T	0.055069483	0.012116411	4.506861179	6.58E-06	14135
genus.Odoribacter.id.952	rs6856150	4	11907559	G	A	0.088192184	0.019414623	4.52430253	6.06E-06	14597
genus.Odoribacter.id.952	rs10093869	8	1266824	A	G	-0.057779486	0.012538929	-4.628997304	3.67E-06	14218
genus.Odoribacter.id.952	rs4793970	17	46763962	A	G	-0.057632938	0.012915412	-4.525441271	6.03E-06	14687
genus.Odoribacter.id.952	rs74553962	8	69677480	T	G	0.121448819	0.026410563	4.428550699	9.49E-06	11803
genus.Odoribacter.id.952	rs28417404	14	70944224	A	G	-0.072689645	0.016137195	-4.62866409	3.68E-06	14698
genus.Odoribacter.id.952	rs16918425	11	6848360	A	T	0.099978081	0.022378451	4.443505765	8.85E-06	13689
genus.Olsenella.id.822	rs1035588	2	150077190	A	G	-0.108148115	0.023684753	-4.570957261	4.86E-06	3739
genus.Olsenella.id.822	rs62112538	19	4925018	C	T	-0.199430349	0.040703259	-4.857595134	1.19E-06	3630
genus.Olsenella.id.822	rs35225860	1	247642270	A	G	-0.223603655	0.048239131	-4.618222564	3.87E-06	3630
genus.Olsenella.id.822	rs72691585	9	21884425	C	A	-0.249078856	0.052081416	-4.674066215	2.95E-06	3078
genus.Olsenella.id.822	rs9460691	6	10422207	C	A	0.119965803	0.026864494	4.485229718	7.28E-06	3739
genus.Olsenella.id.822	rs17148768	10	10777085	G	A	0.140434164	0.029560175	4.734274537	2.20E-06	3648
genus.Olsenella.id.822	rs61090148	4	174810498	A	G	-0.104782706	0.023134258	-4.511493348	6.44E-06	3739
genus.Olsenella.id.822	rs7540303	1	179629191	C	T	0.108043794	0.023637952	4.551745303	5.32E-06	3739
genus.Olsenella.id.822	rs2759329	1	231960352	G	A	-0.111131408	0.02372184	-4.643111275	3.43E-06	3739
genus.Olsenella.id.822	rs8066522	17	59632873	G	A	-0.106528718	0.02403789	-4.4237339	9.70E-06	3739
genus.Olsenella.id.822	rs2424252	20	19971412	A	G	0.113431653	0.025238795	4.501567432	6.75E-06	3705
genus.Oscillibacter.id.2063	rs234108	1	184942671	A	G	0.074955279	0.015263201	4.908803854	9.16E-07	8694
genus.Oscillibacter.id.2063	rs36095275	14	32270129	C	T	-0.07523683	0.015686113	-4.825536101	1.40E-06	8694
genus.Oscillibacter.id.2063	rs11627628	14	21479605	T	C	0.143960728	0.029022314	4.889836975	1.01E-06	8486
genus.Oscillibacter.id.2063	rs9393920	6	28580593	A	G	-0.074466242	0.015108116	-4.893171758	9.92E-07	8930
genus.Oscillibacter.id.2063	rs133832	22	44834707	A	C	-0.079552688	0.016241059	-4.864547286	1.15E-06	8926
genus.Oscillibacter.id.2063	rs12649930	4	3656291	T	G	0.121588701	0.025961173	4.606562801	4.09E-06	8794
genus.Oscillibacter.id.2063	rs75453768	10	116469806	G	T	0.122120078	0.026862946	4.550676187	5.35E-06	8256
genus.Oscillibacter.id.2063	rs16866406	2	179457147	A	G	0.098876509	0.02087954	4.66553075	3.08E-06	8930
genus.Oscillibacter.id.2063	rs761240	20	49507892	T	G	-0.176639573	0.038881196	-4.749578194	2.04E-06	5185
genus.Oscillibacter.id.2063	rs4506202	8	21598077	A	G	-0.071132334	0.01522611	-4.657016085	3.21E-06	8694
genus.Oscillibacter.id.2063	rs61883564	11	79013843	A	G	-0.1013514	0.022101565	-4.6458297	3.39E-06	8930
genus.Oscillibacter.id.2063	rs16934185	9	1798324	A	G	-0.129567532	0.028156999	-4.592469615	4.38E-06	8492
genus.Oscillibacter.id.2063	rs12417956	11	25973451	C	G	0.078543098	0.017407454	4.525466027	6.03E-06	8928

Gut	SNP	CHR	POS	A1	A2	BETA	SE	Z	P	N
genus.Oscillibacter.id.2063	rs6901560	6	36931994	C	G	0.085503217	0.018693038	4.519126985	6.21E-06	8525
genus.Oscillibacter.id.2063	rs62206502	20	18003390	C	A	-0.068142274	0.015134881	-4.506230157	6.60E-06	8883
genus.Oscillibacter.id.2063	rs137917150	12	82366390	T	A	-0.174992153	0.038839403	-4.581349452	4.62E-06	5733
genus.Oscillibacter.id.2063	rs11990279	8	11116314	T	C	-0.082493414	0.018045748	-4.567193175	4.94E-06	8528
genus.Oscillospira.id.2064	rs12206468	6	18093691	G	A	-0.133015789	0.026973335	-4.883495395	1.04E-06	9554
genus.Oscillospira.id.2064	rs73038677	3	172373615	T	A	-0.083210036	0.016866557	-4.873876279	1.09E-06	10195
genus.Oscillospira.id.2064	rs1954532	14	28151415	T	C	-0.082624583	0.017525166	-4.727971988	2.27E-06	9841
genus.Oscillospira.id.2064	rs28889936	4	89483300	A	C	0.114050943	0.025283847	4.647129883	3.37E-06	9758
genus.Oscillospira.id.2064	rs751183	1	76803093	T	C	-0.07743224	0.017221553	-4.4982575	6.85E-06	9841
genus.Oscillospira.id.2064	rs8076323	17	14881860	A	G	0.07153922	0.015653872	4.540460205	5.61E-06	10193
genus.Oscillospira.id.2064	rs72866977	6	56160483	A	C	-0.130569033	0.028167414	-4.539940558	5.63E-06	9098
genus.Oscillospira.id.2064	rs62422654	6	170474630	C	T	0.089845572	0.019808815	4.51049877	6.47E-06	9769
genus.Oscillospira.id.2064	rs12925026	16	89792856	T	C	0.135590113	0.030677984	4.432603288	9.31E-06	8200
genus.Oxalobacter.id.2978	rs4428215	3	171947435	G	A	0.130292636	0.0242237	5.378504552	7.51E-08	4655
genus.Oxalobacter.id.2978	rs6000536	22	37421469	C	T	-0.130991749	0.025380356	-5.193791591	2.06E-07	4654
genus.Oxalobacter.id.2978	rs10465181	9	87500772	C	T	0.111699595	0.021073812	5.292371979	1.21E-07	4654
genus.Oxalobacter.id.2978	rs36057338	4	189935314	G	T	0.207847028	0.042143922	4.916777783	8.80E-07	4244
genus.Oxalobacter.id.2978	rs6071435	20	59502888	T	A	-0.105512319	0.021488964	-4.877514224	1.07E-06	4635
genus.Oxalobacter.id.2978	rs12002250	9	19682558	A	C	0.217122385	0.046631729	4.82287456	1.42E-06	4297
genus.Oxalobacter.id.2978	rs1569853	6	38550301	T	C	-0.138077533	0.029698126	-4.630660717	3.65E-06	4492
genus.Oxalobacter.id.2978	rs11108500	12	96819204	A	G	-0.199099074	0.04273266	-4.625176906	3.74E-06	4303
genus.Oxalobacter.id.2978	rs10464997	8	20902693	G	A	0.137690928	0.029480352	4.65135953	3.30E-06	4650
genus.Oxalobacter.id.2978	rs111966731	15	93941937	T	C	0.213114454	0.047162014	4.484788437	7.30E-06	3931
genus.Oxalobacter.id.2978	rs3862635	11	126582578	C	T	-0.172142003	0.039402609	-4.435477271	9.19E-06	4469
genus.Parabacteroides.id.954	rs60884758	9	2217340	C	T	-0.07026666	0.014224884	-5.000881009	5.71E-07	17293
genus.Parabacteroides.id.954	rs3860755	5	169360155	G	C	0.055878381	0.011662454	4.784603979	1.71E-06	17294
genus.Parabacteroides.id.954	rs4236095	6	47368687	G	A	0.076196085	0.015704151	4.760467272	1.93E-06	16383
genus.Parabacteroides.id.954	rs115602804	3	191572365	G	A	0.10308013	0.02227358	4.760556983	1.93E-06	16394
genus.Parabacteroides.id.954	rs114567323	3	52354847	T	C	0.186477784	0.040519936	4.53889901	5.65E-06	5859
genus.Parabacteroides.id.954	rs7298818	12	56396953	C	T	0.088883151	0.02009019	4.451272519	8.54E-06	16510
genus.Parabacteroides.id.954	rs6657302	1	85585609	T	C	-0.104519671	0.022552116	-4.422489819	9.76E-06	16552

Gut	SNP	CHR	POS	A1	A2	BETA	SE	Z	P	N
genus.Parabacteroides.id.954	rs11965579	6	165969505	G	C	0.16282395	0.038186321	4.443130888	8.87E-06	3890
genus.Parabacteroides.id.954	rs17141986	16	7045276	G	C	0.050030286	0.011145925	4.485506341	7.27E-06	16825
genus.Parabacteroides.id.954	rs72893646	2	38037203	A	T	-0.071559422	0.015666777	-4.443908595	8.83E-06	16508
genus.Paraprevotella.id.962	rs2081023	5	174606261	A	G	-0.122560702	0.023650724	-5.147628814	2.64E-07	7178
genus.Paraprevotella.id.962	rs9900242	17	69135631	A	G	-0.085296669	0.017521167	-4.865673508	1.14E-06	6900
genus.Paraprevotella.id.962	rs9602779	13	86162420	A	C	-0.10669184	0.02202635	-4.963386352	6.93E-07	7177
genus.Paraprevotella.id.962	rs140997932	3	149298792	T	C	-0.162375604	0.035417814	-4.742478264	2.11E-06	6187
genus.Paraprevotella.id.962	rs145020347	11	114526679	A	G	-0.124650032	0.026232336	-4.609824736	4.03E-06	6859
genus.Paraprevotella.id.962	rs4767113	12	114131978	C	T	0.088246646	0.018381962	4.73939539	2.14E-06	6900
genus.Paraprevotella.id.962	rs3008582	1	195971173	T	C	0.105720143	0.022724571	4.5934446	4.36E-06	7175
genus.Paraprevotella.id.962	rs4756632	11	41079703	G	T	-0.138905488	0.028989683	-4.621080082	3.82E-06	7179
genus.Paraprevotella.id.962	rs10842464	12	25249743	T	C	-0.075821491	0.017256778	-4.506288122	6.60E-06	7178
genus.Paraprevotella.id.962	rs3801748	7	81759508	G	A	0.077972078	0.01716912	4.55648106	5.20E-06	7179
genus.Paraprevotella.id.962	rs7240324	18	69623804	T	G	-0.102290408	0.022694788	-4.52783817	5.96E-06	6900
genus.Paraprevotella.id.962	rs17109926	12	71978728	A	G	-0.098833506	0.021617178	-4.501335265	6.75E-06	7179
genus.Paraprevotella.id.962	rs17785622	6	83459314	A	G	0.248065378	0.052430396	4.76097638	1.93E-06	3288
genus.Parasutterella.id.2892	rs7572229	2	72235444	G	A	0.066272974	0.013273581	4.981265537	6.32E-07	11383
genus.Parasutterella.id.2892	rs35414597	4	15565736	T	A	-0.068494619	0.01421433	-4.809635843	1.51E-06	11386
genus.Parasutterella.id.2892	rs6828768	4	65556925	C	T	0.063685185	0.013264516	4.776549773	1.78E-06	11386
genus.Parasutterella.id.2892	rs10899911	10	44293839	A	G	-0.071710162	0.014815144	-4.863596211	1.15E-06	11386
genus.Parasutterella.id.2892	rs7303158	12	5275540	C	T	0.064685912	0.013425592	4.835553658	1.33E-06	11386
genus.Parasutterella.id.2892	rs78383039	2	178954708	T	C	-0.146314647	0.029711928	-4.801555889	1.57E-06	9174
genus.Parasutterella.id.2892	rs35055552	8	114804024	T	C	0.109554176	0.023543268	4.648194275	3.35E-06	9970
genus.Parasutterella.id.2892	rs2090816	6	137615592	A	C	0.084096728	0.017731494	4.677979708	2.90E-06	11385
genus.Parasutterella.id.2892	rs1403396	12	126498824	A	T	-0.075672871	0.015903462	-4.688274063	2.76E-06	11378
genus.Parasutterella.id.2892	rs55877868	17	14692867	A	C	-0.10445781	0.022808932	-4.679861871	2.87E-06	10588
genus.Parasutterella.id.2892	rs8039785	15	67316307	T	G	0.061834541	0.013300041	4.632031536	3.62E-06	11383
genus.Parasutterella.id.2892	rs823424	8	16674526	G	A	-0.071344819	0.015695821	-4.566770767	4.95E-06	11386
genus.Parasutterella.id.2892	rs7311004	12	53260710	T	C	-0.061752212	0.01364384	-4.529207428	5.92E-06	11384
genus.Parasutterella.id.2892	rs62273907	3	156550415	A	G	0.229468131	0.050226185	4.53052116	5.88E-06	3691
genus.Parasutterella.id.2892	rs6809952	3	193896709	G	A	-0.068495728	0.015089156	-4.461639757	8.13E-06	11077

Gut	SNP	CHR	POS	A1	A2	BETA	SE	Z	P	N
genus.Parasutterella.id.2892	rs11715853	3	30163689	G	A	-0.066295189	0.014611398	-4.518459129	6.23E-06	11386
genus.Peptococcus.id.2037	rs75754569	3	17793504	C	G	0.181434144	0.031942874	5.714721626	1.10E-08	5481
genus.Peptococcus.id.2037	rs77681628	13	91182556	C	T	0.200307214	0.038732756	5.144079124	2.69E-07	4842
genus.Peptococcus.id.2037	rs10031059	4	35358594	T	C	-0.121166531	0.022584403	-5.288035838	1.24E-07	5526
genus.Peptococcus.id.2037	rs6918730	6	99020289	G	A	0.135310568	0.028974201	4.864525779	1.15E-06	5656
genus.Peptococcus.id.2037	rs413827	14	57985112	G	A	0.11022947	0.023752348	4.651152578	3.30E-06	5482
genus.Peptococcus.id.2037	rs2054133	2	33691809	G	A	0.089543257	0.018833153	4.739575636	2.14E-06	5657
genus.Peptococcus.id.2037	rs11001941	10	78716030	G	A	-0.195611127	0.039221681	-4.835430088	1.33E-06	5607
genus.Peptococcus.id.2037	rs7033353	9	104595848	T	G	0.090152001	0.018995009	4.732329923	2.22E-06	5657
genus.Peptococcus.id.2037	rs5770862	22	50973113	T	C	0.162017845	0.035681339	4.656290785	3.22E-06	5431
genus.Peptococcus.id.2037	rs34282744	15	75073172	G	C	0.19179739	0.03998324	4.770757245	1.84E-06	5257
genus.Peptococcus.id.2037	rs72850165	11	968356	T	C	-0.134304018	0.030042318	-4.53569098	5.74E-06	5627
genus.Peptococcus.id.2037	rs77614201	13	89245906	T	C	-0.175863843	0.03875232	-4.537844428	5.68E-06	5329
genus.Peptococcus.id.2037	rs11030569	11	4019788	A	T	-0.174020126	0.037413336	-4.662105281	3.13E-06	5329
genus.Peptococcus.id.2037	rs7766680	6	162239191	G	C	0.09768317	0.021415492	4.638605402	3.51E-06	5655
genus.Peptococcus.id.2037	rs74592222	1	75971700	G	A	0.137957011	0.030296488	4.450842165	8.55E-06	5329
genus.Peptococcus.id.2037	rs36121075	20	43118017	A	G	-0.140670082	0.030627993	-4.494078134	6.99E-06	5301
genus.Peptococcus.id.2037	rs76697713	3	17250814	C	A	0.146302407	0.032234478	4.463878645	8.05E-06	5481
genus.Peptococcus.id.2037	rs12069354	1	216223782	C	T	0.167629782	0.037950049	4.433224929	9.28E-06	5352
genus.Phascolarctobacterium.id.2168	rs75882962	12	53098742	T	C	0.096866054	0.019057501	5.11186909	3.19E-07	10710
genus.Phascolarctobacterium.id.2168	rs56157888	4	183252836	A	C	0.095486383	0.019397634	4.875549392	1.09E-06	10031
genus.Phascolarctobacterium.id.2168	rs56069061	3	168339417	G	A	-0.111305825	0.02306943	-4.766800992	1.87E-06	11007
genus.Phascolarctobacterium.id.2168	rs74540770	3	186553389	G	A	-0.121014393	0.025862072	-4.633074592	3.60E-06	9403
genus.Phascolarctobacterium.id.2168	rs76124218	17	73584441	C	G	-0.159360781	0.03445316	-4.694722455	2.67E-06	7314
genus.Phascolarctobacterium.id.2168	rs6427992	1	203102563	G	C	-0.065245772	0.013750826	-4.744895437	2.09E-06	11113
genus.Phascolarctobacterium.id.2168	rs12618201	2	174436533	A	G	0.06416571	0.013820192	4.646449779	3.38E-06	11113
genus.Phascolarctobacterium.id.2168	rs74847270	22	47841258	A	G	-0.104889022	0.023099793	-4.536078175	5.73E-06	11098
genus.Phascolarctobacterium.id.2168	rs1264476	8	102434276	T	G	0.076733542	0.016604148	4.596538765	4.30E-06	11104
genus.Phascolarctobacterium.id.2168	rs28525131	14	21358155	G	A	-0.118657158	0.026904831	-4.459195923	8.23E-06	10457
genus.Phascolarctobacterium.id.2168	rs7982713	13	71376322	G	A	0.072692152	0.016320086	4.423273815	9.72E-06	10710
genus.Phascolarctobacterium.id.2168	rs11929846	4	17051156	T	C	-0.069719918	0.015796922	-4.442688841	8.88E-06	11114

Gut	SNP	CHR	POS	A1	A2	BETA	SE	Z	P	N
genus.Prevotella7.id.11182	rs430270	3	60474379	A	C	0.13915522	0.029705527	4.680020827	2.87E-06	3318
genus.Prevotella7.id.11182	rs57404562	2	12101762	C	A	0.15548647	0.031605691	4.984389965	6.22E-07	3487
genus.Prevotella7.id.11182	rs9959718	18	71442664	G	A	0.133012021	0.027536133	4.7637178	1.90E-06	3489
genus.Prevotella7.id.11182	rs9608249	22	24613952	A	G	-0.158213329	0.033631703	-4.746773345	2.07E-06	3482
genus.Prevotella7.id.11182	rs2240542	2	242066314	C	T	0.120849888	0.026177616	4.571542761	4.84E-06	3489
genus.Prevotella7.id.11182	rs16937247	11	19943115	G	C	0.146097858	0.035152923	4.425160757	9.64E-06	3486
genus.Prevotella7.id.11182	rs118038478	16	47237609	A	G	0.205675553	0.046891721	4.46911964	7.85E-06	3295
genus.Prevotella7.id.11182	rs12124567	1	3343802	A	G	-0.121281313	0.027501731	-4.42848308	9.49E-06	3283
genus.Prevotella7.id.11182	rs12195431	6	91019654	T	C	0.196527815	0.044232188	4.446497126	8.73E-06	3489
genus.Prevotella7.id.11182	rs9426434	1	29736216	T	C	-0.123657205	0.027851446	-4.42337454	9.72E-06	3318
genus.Prevotella7.id.11182	rs79263163	11	39821773	A	C	-0.143981995	0.031500978	-4.478683595	7.51E-06	3489
genus.Prevotella9.id.11183	rs111509883	19	639161	T	C	0.171130632	0.03476241	4.848515691	1.24E-06	6960
genus.Prevotella9.id.11183	rs2683313	8	19115604	A	G	-0.072457582	0.015160279	-4.787472159	1.69E-06	10260
genus.Prevotella9.id.11183	rs117271932	22	44236460	A	G	0.20808857	0.044039277	4.683715026	2.82E-06	4878
genus.Prevotella9.id.11183	rs10512344	9	109009495	C	G	0.247372806	0.054408651	4.6579822	3.19E-06	3063
genus.Prevotella9.id.11183	rs9428102	1	118852817	A	G	-0.07788445	0.017606807	-4.581463093	4.62E-06	10264
genus.Prevotella9.id.11183	rs746764	20	21470713	T	C	-0.091577152	0.019324484	-4.749422036	2.04E-06	9385
genus.Prevotella9.id.11183	rs16966465	15	38513730	G	C	0.074334818	0.01652906	4.432098835	9.33E-06	10261
genus.Prevotella9.id.11183	rs2104588	10	12497581	T	C	0.105558491	0.023773023	4.461735054	8.13E-06	9268
genus.Prevotella9.id.11183	rs11199734	10	122819400	A	T	0.077020901	0.016946218	4.493600875	7.00E-06	10263
genus.Prevotella9.id.11183	rs7232121	18	19993452	G	C	0.067129188	0.014422959	4.624218866	3.76E-06	9790
genus.Prevotella9.id.11183	rs11685699	2	11232685	C	T	-0.141357386	0.029566474	-4.750861361	2.03E-06	8342
genus.Prevotella9.id.11183	rs9613013	22	26152738	G	A	0.091759081	0.020269664	4.522749406	6.10E-06	9790
genus.Prevotella9.id.11183	rs2495052	1	14161251	A	G	0.083897846	0.018852315	4.440586141	8.97E-06	9790
genus.Prevotella9.id.11183	rs72815774	2	91954502	T	C	-0.176185788	0.039306766	-4.445153575	8.78E-06	5728
genus.Prevotella9.id.11183	rs4968431	17	59421778	G	T	0.064011283	0.014416226	4.450065532	8.58E-06	10271
genus.Prevotella9.id.11183	rs4821647	22	37758730	G	C	0.063695653	0.014398271	4.418283123	9.95E-06	10179
genus.Prevotella9.id.11183	rs7976209	12	1794470	T	C	-0.087078586	0.019795052	-4.485232912	7.28E-06	10263
genus.Prevotella9.id.11183	rs12648235	4	161115744	T	C	0.078590196	0.017767553	4.482112014	7.39E-06	10271
genus.Prevotella9.id.11183	rs7237249	18	71992502	C	T	-0.082376407	0.018212594	-4.441508895	8.93E-06	10271
genus.RikenellaceaeRC9gutgroup.id.11191	rs2900503	9	112657543	G	T	-0.172329153	0.032667127	-5.246184336	1.55E-07	3320

Gut	SNP	CHR	POS	A1	A2	BETA	SE	Z	P	N
genus.RikenellaceaeRC9gutgroup.id.11191	rs17582787	4	149562638	A	G	-0.157739395	0.033992444	-4.63590621	3.55E-06	3320
genus.RikenellaceaeRC9gutgroup.id.11191	rs7113155	11	102429543	G	C	0.114422634	0.024779825	4.553948966	5.26E-06	3334
genus.RikenellaceaeRC9gutgroup.id.11191	rs4270579	4	84698188	G	A	-0.118021261	0.027104126	-4.546425397	5.46E-06	3331
genus.RikenellaceaeRC9gutgroup.id.11191	rs2998141	10	135010198	T	C	-0.136346427	0.029307262	-4.590411388	4.42E-06	3330
genus.RikenellaceaeRC9gutgroup.id.11191	rs4717843	7	73334987	G	T	-0.119376102	0.026054834	-4.576982772	4.72E-06	3220
genus.RikenellaceaeRC9gutgroup.id.11191	rs9887954	1	164800873	G	A	-0.114849386	0.024901293	-4.572995382	4.81E-06	3330
genus.RikenellaceaeRC9gutgroup.id.11191	rs7193937	16	76605358	G	C	0.12415332	0.027741031	4.519873293	6.19E-06	3220
genus.RikenellaceaeRC9gutgroup.id.11191	rs80309088	6	165668732	G	A	0.174049957	0.038342318	4.5842394	4.56E-06	3170
genus.RikenellaceaeRC9gutgroup.id.11191	rs12501673	4	163830516	A	G	0.116395371	0.026225344	4.516405089	6.29E-06	3334
genus.RikenellaceaeRC9gutgroup.id.11191	rs17032291	4	155865671	T	C	-0.169578595	0.036825126	-4.505873687	6.61E-06	3206
genus.RikenellaceaeRC9gutgroup.id.11191	rs2074881	19	1970021	T	C	-0.142235956	0.032390968	-4.429441958	9.45E-06	3315
genus.RikenellaceaeRC9gutgroup.id.11191	rs7712231	5	31406285	A	G	0.156283835	0.035046199	4.465978554	7.97E-06	3334
genus.Romboutsia.id.11347	rs61841503	10	17019559	G	A	0.092887978	0.017145406	5.491029738	4.00E-08	13568
genus.Romboutsia.id.11347	rs10279978	7	5319387	A	G	-0.062220227	0.012767535	-4.86063634	1.17E-06	14143
genus.Romboutsia.id.11347	rs16843578	1	171903885	C	T	-0.087527594	0.019677862	-4.561405816	5.08E-06	14186
genus.Romboutsia.id.11347	rs77702691	13	88725540	A	G	-0.094405983	0.020854443	-4.482720998	7.37E-06	12487
genus.Romboutsia.id.11347	rs75987356	2	16066705	G	A	-0.129578364	0.028045444	-4.502749532	6.71E-06	10466
genus.Romboutsia.id.11347	rs75200530	8	5088054	T	G	-0.190644379	0.042106493	-4.562054629	5.07E-06	5246
genus.Romboutsia.id.11347	rs9567264	13	32720756	C	T	0.058001807	0.01274947	4.535126883	5.76E-06	14180
genus.Romboutsia.id.11347	rs34302036	7	78136725	A	G	0.055026128	0.012083475	4.530778546	5.88E-06	14185
genus.Romboutsia.id.11347	rs7109293	11	133834657	A	G	0.091911164	0.020573834	4.494189498	6.98E-06	14141
genus.Romboutsia.id.11347	rs10091895	8	3391361	T	C	-0.064991806	0.014550099	-4.5056836	6.62E-06	14188
genus.Romboutsia.id.11347	rs114398731	5	57229635	G	C	-0.13103857	0.029381546	-4.466297152	7.96E-06	10326
genus.Romboutsia.id.11347	rs28603357	7	106406824	T	C	-0.214910996	0.047473928	-4.451623384	8.52E-06	3693
genus.Romboutsia.id.11347	rs11221428	11	99665050	T	C	-0.072677936	0.015826331	-4.509879108	6.49E-06	14187
genus.Romboutsia.id.11347	rs9389266	6	135411737	T	G	0.072281496	0.016235341	4.430887265	9.38E-06	14187
genus.Romboutsia.id.11347	rs62504452	7	145760298	A	G	-0.071004771	0.015665426	-4.579615528	4.66E-06	14178
genus.Roseburia.id.2012	rs116270582	4	119349	T	A	-0.153843918	0.032932399	-4.856422894	1.20E-06	9099
genus.Roseburia.id.2012	rs2160994	12	50650057	T	C	0.055068659	0.011248106	4.897659224	9.70E-07	17444
genus.Roseburia.id.2012	rs16910295	11	12009569	T	C	-0.098043368	0.020956968	-4.676993939	2.91E-06	16648
genus.Roseburia.id.2012	rs12740451	1	95178974	T	C	0.069752736	0.015360737	4.483506527	7.34E-06	17837

Gut	SNP	CHR	POS	A1	A2	BETA	SE	Z	P	N
genus.Roseburia.id.2012	rs9300744	13	103117486	C	T	-0.058845175	0.012622789	-4.575485129	4.75E-06	17854
genus.Roseburia.id.2012	rs2943022	5	89598914	T	C	0.049378559	0.010676336	4.605534377	4.11E-06	17854
genus.Roseburia.id.2012	rs6930661	6	12774611	C	T	-0.096158735	0.020497475	-4.709814911	2.48E-06	16945
genus.Roseburia.id.2012	rs4748237	10	16051097	G	C	0.048832628	0.010640433	4.578991593	4.67E-06	17851
genus.Roseburia.id.2012	rs147990086	1	145655256	A	G	-0.057893629	0.013239999	-4.441595773	8.93E-06	17840
genus.Roseburia.id.2012	rs329182	5	125076494	T	C	0.069032724	0.015288408	4.529890639	5.90E-06	17854
genus.Roseburia.id.2012	rs302266	1	194183650	T	C	-0.077731153	0.01729852	-4.46184041	8.13E-06	17056
genus.Roseburia.id.2012	rs75326254	6	166170836	C	T	-0.104627797	0.023089702	-4.478923418	7.50E-06	15893
genus.Roseburia.id.2012	rs55858165	15	86512809	A	C	0.17928439	0.040495413	4.41739518	9.99E-06	5447
genus.Roseburia.id.2012	rs28040	5	96018066	G	C	0.056569289	0.012679199	4.433735751	9.26E-06	17854
genus.Roseburia.id.2012	rs78753150	5	144135655	A	C	0.096873855	0.021406676	4.417538596	9.98E-06	16303
genus.Ruminiclostridium5.id.11355	rs243585	21	19151940	C	G	-0.058593933	0.012101692	-4.835695195	1.33E-06	16906
genus.Ruminiclostridium5.id.11355	rs2286384	12	1891043	G	C	-0.051881398	0.010748225	-4.819584858	1.44E-06	17394
genus.Ruminiclostridium5.id.11355	rs79968837	20	41177956	A	G	-0.095031084	0.019350746	-4.864712867	1.15E-06	16602
genus.Ruminiclostridium5.id.11355	rs2482038	10	12835085	C	A	0.051899121	0.010877683	4.785792371	1.70E-06	17396
genus.Ruminiclostridium5.id.11355	rs113753996	5	32477664	T	C	0.082068892	0.017446254	4.611957261	3.99E-06	16680
genus.Ruminiclostridium5.id.11355	rs1492620	6	50405920	T	C	-0.083050484	0.018007297	-4.637542039	3.53E-06	17397
genus.Ruminiclostridium5.id.11355	rs2791343	8	131316030	T	C	0.051717025	0.011336879	4.543240913	5.54E-06	17392
genus.Ruminiclostridium5.id.11355	rs10827477	10	35262588	A	G	-0.054738653	0.011516344	-4.734887218	2.19E-06	17391
genus.Ruminiclostridium5.id.11355	rs6121460	20	60293505	G	A	0.09329552	0.019920597	4.696925482	2.64E-06	17318
genus.Ruminiclostridium5.id.11355	rs2801960	1	41878680	C	G	0.052097504	0.011511124	4.519154006	6.21E-06	17387
genus.Ruminiclostridium5.id.11355	rs8053158	16	86769909	A	G	-0.074054329	0.015915101	-4.53001102	5.90E-06	17397
genus.Ruminiclostridium5.id.11355	rs1223978	13	108775162	T	C	0.048416888	0.010825421	4.460893666	8.16E-06	17381
genus.Ruminiclostridium5.id.11355	rs73002572	2	103755970	G	C	0.181547526	0.041407845	4.444193849	8.82E-06	3761
genus.Ruminiclostridium5.id.11355	rs2833828	21	33811886	G	A	0.048961687	0.010870209	4.499338845	6.82E-06	17397
genus.Ruminiclostridium5.id.11355	rs4955951	3	55243531	A	G	-0.07137494	0.01658264	-4.418144517	9.96E-06	17397
genus.Ruminiclostridium6.id.11356	rs1756364	14	20954661	G	T	0.099918929	0.019716857	5.154982767	2.54E-07	12933
genus.Ruminiclostridium6.id.11356	rs71414120	14	56938952	T	G	0.200894087	0.040643398	4.877027165	1.08E-06	5567
genus.Ruminiclostridium6.id.11356	rs61060922	16	72136154	T	G	0.159129692	0.032228344	4.875293988	1.09E-06	8955
genus.Ruminiclostridium6.id.11356	rs79968172	1	240503826	G	A	0.116139597	0.02430834	4.790877312	1.66E-06	12802
genus.Ruminiclostridium6.id.11356	rs77193512	11	40289063	A	G	0.073653265	0.015316349	4.840386931	1.30E-06	13380

Gut	SNP	CHR	POS	A1	A2	BETA	SE	Z	P	N
genus.Ruminiclostridium6.id.11356	rs11992182	8	79766499	A	C	0.06252993	0.013787562	4.580061796	4.65E-06	13381
genus.Ruminiclostridium6.id.11356	rs72991535	18	76018244	T	G	0.135603894	0.029517443	4.566740261	4.95E-06	9642
genus.Ruminiclostridium6.id.11356	rs663262	11	86179076	T	C	-0.134980385	0.03107145	-4.645400655	3.39E-06	7876
genus.Ruminiclostridium6.id.11356	rs9555756	13	111703249	A	C	-0.080413824	0.017669194	-4.490630427	7.10E-06	13369
genus.Ruminiclostridium6.id.11356	rs792058	2	5548605	G	A	0.055423396	0.012542318	4.450187575	8.58E-06	12933
genus.Ruminiclostridium6.id.11356	rs10829821	10	132651293	T	C	-0.097605954	0.021607241	-4.640753566	3.47E-06	12802
genus.Ruminiclostridium6.id.11356	rs67479537	19	10115515	T	C	0.118994191	0.026487011	4.432793294	9.30E-06	11613
genus.Ruminiclostridium6.id.11356	rs1871858	15	61047117	C	G	-0.105421541	0.023727442	-4.437143374	9.12E-06	12818
genus.Ruminiclostridium6.id.11356	rs116969552	10	128214403	A	G	-0.166822503	0.037668056	-4.436119772	9.16E-06	6824
genus.Ruminiclostridium6.id.11356	rs73176030	7	101271282	T	C	0.058665044	0.013207973	4.485074883	7.29E-06	13378
genus.Ruminiclostridium6.id.11356	rs35362464	4	36478011	C	A	0.072007723	0.016538673	4.440107865	8.99E-06	13308
genus.Ruminiclostridium9.id.11357	rs57665991	3	157841287	C	G	-0.064150707	0.012333861	-5.192692922	2.07E-07	16725
genus.Ruminiclostridium9.id.11357	rs55875763	3	158345912	G	C	-0.054533127	0.01129737	-4.797987225	1.60E-06	16725
genus.Ruminiclostridium9.id.11357	rs12040548	1	247710285	G	T	0.057042151	0.012235891	4.660872372	3.15E-06	16721
genus.Ruminiclostridium9.id.11357	rs115044523	7	47013498	G	A	-0.098002558	0.020261641	-4.718832376	2.37E-06	16107
genus.Ruminiclostridium9.id.11357	rs12419854	11	68886460	T	A	-0.072797471	0.015559424	-4.658842702	3.18E-06	16027
genus.Ruminiclostridium9.id.11357	rs6082461	20	2210530	A	C	0.058642458	0.013100394	4.570486345	4.87E-06	16650
genus.Ruminiclostridium9.id.11357	rs113048721	9	74214419	C	G	0.060073481	0.013273731	4.606069282	4.10E-06	16719
genus.Ruminiclostridium9.id.11357	rs73592673	6	128568828	A	T	-0.08162902	0.016951466	-4.739760454	2.14E-06	15493
genus.Ruminiclostridium9.id.11357	rs79082720	7	155701010	C	G	0.092857633	0.020487143	4.510439276	6.47E-06	16094
genus.Ruminiclostridium9.id.11357	rs74303178	8	14697544	T	C	0.053274162	0.011927361	4.467452325	7.92E-06	16706
genus.Ruminiclostridium9.id.11357	rs13033315	2	204706865	T	A	0.051119464	0.011141773	4.537944526	5.68E-06	16724
genus.Ruminiclostridium9.id.11357	rs9809789	3	29179983	C	T	-0.071745357	0.015967315	-4.446706695	8.72E-06	16025
genus.Ruminiclostridium9.id.11357	rs9522712	13	90441578	T	C	0.069972768	0.015493905	4.579557059	4.66E-06	16725
genus.Ruminiclostridium9.id.11357	rs78191726	6	67655320	T	C	0.094499761	0.02102058	4.476635065	7.58E-06	16039
genus.Ruminiclostridium9.id.11357	rs2377294	19	34375757	T	G	-0.08812678	0.020012646	-4.441374612	8.94E-06	16716
genus.Ruminiclostridium9.id.11357	rs7137760	12	10702414	C	T	0.050797017	0.011218236	4.491597106	7.07E-06	16722
genus.RuminococcaceaeNK4A214group.id.11358	rs5994253	22	17793012	A	G	-0.081130217	0.015762089	-5.169132579	2.35E-07	15288
genus.RuminococcaceaeNK4A214group.id.11358	rs136761	22	49796014	G	A	-0.058752097	0.01191547	-4.931694038	8.15E-07	14822
genus.RuminococcaceaeNK4A214group.id.11358	rs11586410	1	157338888	G	A	-0.086342178	0.016993683	-5.085698334	3.66E-07	14822
genus.RuminococcaceaeNK4A214group.id.11358	rs34576931	12	78373555	G	C	-0.087331196	0.019488596	-4.577022574	4.72E-06	14678

Gut	SNP	CHR	POS	A1	A2	BETA	SE	Z	P	N
genus.RuminococcaceaeNK4A214group.id.11358	rs114244418	2	235430299	C	G	-0.17526622	0.037277927	-4.633721154	3.59E-06	7123
genus.RuminococcaceaeNK4A214group.id.11358	rs4814689	20	18030357	C	T	-0.108300291	0.023070832	-4.584446983	4.55E-06	14009
genus.RuminococcaceaeNK4A214group.id.11358	rs73158814	7	132042135	C	G	-0.10925732	0.022745845	-4.734313171	2.20E-06	13077
genus.RuminococcaceaeNK4A214group.id.11358	rs12731	2	239087912	A	G	-0.052768427	0.01150534	-4.57020952	4.87E-06	15288
genus.RuminococcaceaeNK4A214group.id.11358	rs7573569	2	141897042	T	C	0.107738536	0.023363816	4.655535851	3.23E-06	14822
genus.RuminococcaceaeNK4A214group.id.11358	rs147475196	3	26509287	A	G	-0.133768553	0.029519585	-4.576643443	4.72E-06	9917
genus.RuminococcaceaeNK4A214group.id.11358	rs62027366	16	24073806	T	C	0.061538935	0.013761184	4.506693699	6.58E-06	14822
genus.RuminococcaceaeNK4A214group.id.11358	rs12642039	4	159932022	T	C	-0.055300297	0.011939746	-4.643229417	3.43E-06	15276
genus.RuminococcaceaeNK4A214group.id.11358	rs35559912	5	35288938	T	C	-0.092507085	0.020367383	-4.569442484	4.89E-06	13976
genus.RuminococcaceaeNK4A214group.id.11358	rs6681678	1	100248591	C	T	-0.100174165	0.024000023	-4.438725548	9.05E-06	13031
genus.RuminococcaceaeNK4A214group.id.11358	rs11241747	5	123846318	C	T	0.053385843	0.012003462	4.506527526	6.59E-06	14822
genus.RuminococcaceaeUCG002.id.11360	rs77564310	15	80713184	A	C	-0.071309265	0.014085525	-5.106250639	3.29E-07	17000
genus.RuminococcaceaeUCG002.id.11360	rs55793120	12	47384118	T	C	0.137396059	0.027413962	5.033870687	4.81E-07	11669
genus.RuminococcaceaeUCG002.id.11360	rs10927423	1	14732458	C	A	-0.071361729	0.014771224	-4.923633652	8.50E-07	17096
genus.RuminococcaceaeUCG002.id.11360	rs67746927	4	12207874	C	G	-0.054237005	0.011035718	-4.908642864	9.17E-07	16651
genus.RuminococcaceaeUCG002.id.11360	rs116974815	11	111712942	C	A	-0.189730541	0.039656625	-4.75089544	2.03E-06	5819
genus.RuminococcaceaeUCG002.id.11360	rs7155595	14	77502546	C	A	0.056992918	0.011698636	4.864182736	1.15E-06	17084
genus.RuminococcaceaeUCG002.id.11360	rs10916131	1	227563126	C	T	-0.069333027	0.014675325	-4.679556032	2.87E-06	17097
genus.RuminococcaceaeUCG002.id.11360	rs79016051	1	238938497	C	T	-0.088774672	0.018942419	-4.721820963	2.34E-06	16937
genus.RuminococcaceaeUCG002.id.11360	rs12463378	19	54475808	A	G	-0.052206066	0.011208649	-4.673906961	2.96E-06	16994
genus.RuminococcaceaeUCG002.id.11360	rs11750293	5	123822114	G	T	-0.057830385	0.012051229	-4.77903738	1.76E-06	17094
genus.RuminococcaceaeUCG002.id.11360	rs2265670	1	190171640	C	G	-0.051240397	0.010928696	-4.671391695	2.99E-06	17096
genus.RuminococcaceaeUCG002.id.11360	rs15256	10	73820548	C	T	0.073237587	0.016833997	4.429239666	9.46E-06	17095
genus.RuminococcaceaeUCG002.id.11360	rs882348	4	23325040	A	G	-0.079994585	0.017861803	-4.54673234	5.45E-06	17097
genus.RuminococcaceaeUCG002.id.11360	rs72874194	2	45178830	G	C	-0.077028853	0.016724954	-4.641037471	3.47E-06	16651
genus.RuminococcaceaeUCG002.id.11360	rs6793778	3	24045453	C	T	-0.055870572	0.012525794	-4.421255932	9.81E-06	17094
genus.RuminococcaceaeUCG002.id.11360	rs7342369	12	129451330	C	A	-0.052780292	0.011602332	-4.538662621	5.66E-06	16651
genus.RuminococcaceaeUCG002.id.11360	rs11607472	11	43344751	A	G	-0.0780229	0.017632484	-4.488082356	7.19E-06	16282
genus.RuminococcaceaeUCG002.id.11360	rs7249614	19	43637890	A	G	-0.049277826	0.011080679	-4.438144646	9.07E-06	16651
genus.RuminococcaceaeUCG002.id.11360	rs76847269	5	141014951	A	G	0.163508017	0.035615077	4.557691152	5.17E-06	7654
genus.RuminococcaceaeUCG002.id.11360	rs6542556	2	120757853	A	G	0.050974014	0.011406021	4.468928727	7.86E-06	17094

Gut	SNP	CHR	POS	A1	A2	BETA	SE	Z	P	N
genus.RuminococcaceaeUCG002.id.11360	rs57079348	13	87920450	T	G	-0.07657295	0.01728195	-4.487150231	7.22E-06	16372
genus.RuminococcaceaeUCG002.id.11360	rs56030423	15	90780618	G	A	-0.098287388	0.021634426	-4.515933357	6.30E-06	14937
genus.RuminococcaceaeUCG002.id.11360	rs10898505	11	86309390	C	T	0.061862475	0.015002269	4.418368465	9.94E-06	17097
genus.RuminococcaceaeUCG002.id.11360	rs362417	14	73589896	G	C	-0.054862993	0.01209839	-4.470599895	7.80E-06	17097
genus.RuminococcaceaeUCG002.id.11360	rs113147300	9	114066670	A	G	-0.075841986	0.016456212	-4.473598783	7.69E-06	16370
genus.RuminococcaceaeUCG003.id.11361	rs73341549	7	51541468	T	C	-0.169857156	0.031876785	-5.251393638	1.51E-07	6744
genus.RuminococcaceaeUCG003.id.11361	rs6759615	2	205238716	A	G	0.10252284	0.020020394	4.938756342	7.86E-07	14410
genus.RuminococcaceaeUCG003.id.11361	rs11613919	12	75496463	G	T	0.072760434	0.015571648	4.795204339	1.63E-06	14309
genus.RuminococcaceaeUCG003.id.11361	rs11243416	9	134416970	T	C	-0.092565265	0.019120311	-4.789878337	1.67E-06	14004
genus.RuminococcaceaeUCG003.id.11361	rs2000751	6	162573342	C	A	0.055920289	0.012225555	4.655077818	3.24E-06	13915
genus.RuminococcaceaeUCG003.id.11361	rs16959793	15	35071718	A	C	-0.062527033	0.013125865	-4.732556813	2.22E-06	14412
genus.RuminococcaceaeUCG003.id.11361	rs10490280	2	37905976	C	T	-0.067209365	0.014330937	-4.60344918	4.16E-06	14004
genus.RuminococcaceaeUCG003.id.11361	rs4532474	6	105781538	G	A	0.076923106	0.017044961	4.572418606	4.82E-06	14412
genus.RuminococcaceaeUCG003.id.11361	rs4629039	18	46485154	T	A	0.055374213	0.012265944	4.508019776	6.54E-06	14404
genus.RuminococcaceaeUCG003.id.11361	rs2523124	7	97348436	T	C	-0.054662483	0.012082341	-4.53414599	5.78E-06	14409
genus.RuminococcaceaeUCG003.id.11361	rs3013089	1	13794594	G	A	-0.05514734	0.012034599	-4.592421082	4.38E-06	14408
genus.RuminococcaceaeUCG003.id.11361	rs78720113	3	41982393	A	G	-0.115348392	0.02496006	-4.476360772	7.59E-06	13078
genus.RuminococcaceaeUCG003.id.11361	rs139730	22	25292719	G	C	-0.057765528	0.013064482	-4.42328533	9.72E-06	14004
genus.RuminococcaceaeUCG004.id.11362	rs6769553	3	54427795	A	G	0.084974255	0.015745058	5.369154093	7.91E-08	9501
genus.RuminococcaceaeUCG004.id.11362	rs12125734	1	103732214	G	T	0.133971622	0.02574165	5.190768391	2.09E-07	8938
genus.RuminococcaceaeUCG004.id.11362	rs511258	3	169966722	G	A	-0.075747368	0.016247466	-4.585892257	4.52E-06	9500
genus.RuminococcaceaeUCG004.id.11362	rs2248146	7	24791998	T	C	0.068945165	0.015364919	4.459894209	8.20E-06	9502
genus.RuminococcaceaeUCG004.id.11362	rs10976229	9	7317307	T	G	0.095975994	0.021455386	4.492331308	7.04E-06	9116
genus.RuminococcaceaeUCG004.id.11362	rs7569771	2	239846337	A	G	-0.075914029	0.01703081	-4.462004735	8.12E-06	9502
genus.RuminococcaceaeUCG004.id.11362	rs872501	8	139253064	G	A	0.116124682	0.025946001	4.533258968	5.81E-06	8473
genus.RuminococcaceaeUCG004.id.11362	rs550351	1	18676399	A	C	0.078587023	0.018003041	4.429817657	9.43E-06	9498
genus.RuminococcaceaeUCG004.id.11362	rs3800154	6	2119329	A	C	-0.079795901	0.017763932	-4.522345715	6.12E-06	9406
genus.RuminococcaceaeUCG004.id.11362	rs11961899	6	132887921	G	A	-0.070782539	0.01613814	-4.435633336	9.18E-06	9501
genus.RuminococcaceaeUCG004.id.11362	rs9818949	3	197683751	G	T	0.085992665	0.018867146	4.549071257	5.39E-06	9116
genus.RuminococcaceaeUCG004.id.11362	rs7123615	11	15771251	C	G	-0.07861572	0.017959154	-4.490914135	7.09E-06	9502
genus.RuminococcaceaeUCG005.id.11363	rs10950694	7	18026604	T	C	0.057762846	0.011412014	5.05526206	4.30E-07	15821

Gut	SNP	CHR	POS	A1	A2	BETA	SE	Z	P	N
genus.RuminococcaceaeUCG005.id.11363	rs34781347	20	16312851	G	A	0.188684782	0.03864836	4.989701146	6.05E-07	6061
genus.RuminococcaceaeUCG005.id.11363	rs12288512	11	27747671	A	G	0.066635035	0.014435565	4.663896168	3.10E-06	15473
genus.RuminococcaceaeUCG005.id.11363	rs60081663	5	32755085	C	G	0.158081538	0.031958601	4.906298341	9.28E-07	9342
genus.RuminococcaceaeUCG005.id.11363	rs114279581	2	198122357	A	G	-0.146604207	0.031603425	-4.656357489	3.22E-06	8465
genus.RuminococcaceaeUCG005.id.11363	rs12458218	18	22575871	T	C	0.067732182	0.014452491	4.715577174	2.41E-06	16099
genus.RuminococcaceaeUCG005.id.11363	rs35166120	8	3099727	C	G	-0.068615325	0.014625203	-4.624555473	3.75E-06	16092
genus.RuminococcaceaeUCG005.id.11363	rs7555878	1	187906034	A	G	0.058667737	0.012527736	4.684224664	2.81E-06	16100
genus.RuminococcaceaeUCG005.id.11363	rs394449	17	7034575	A	T	0.069290871	0.014871206	4.700207502	2.60E-06	15728
genus.RuminococcaceaeUCG005.id.11363	rs2893871	10	62662781	G	A	-0.073643982	0.015547986	-4.636799462	3.54E-06	16103
genus.RuminococcaceaeUCG005.id.11363	rs72776570	10	4240906	C	A	0.087067007	0.019717899	4.55029168	5.36E-06	15376
genus.RuminococcaceaeUCG005.id.11363	rs898577	15	68705697	T	C	-0.12301218	0.028667054	-4.480033706	7.46E-06	9991
genus.RuminococcaceaeUCG005.id.11363	rs7449320	5	154003091	C	A	0.059916334	0.013083635	4.573006196	4.81E-06	16095
genus.RuminococcaceaeUCG005.id.11363	rs7586445	2	239509030	G	A	0.07823492	0.017645763	4.444381635	8.81E-06	15099
genus.RuminococcaceaeUCG005.id.11363	rs55793120	12	47384118	T	C	0.121547709	0.0279546	4.482699808	7.37E-06	11224
genus.RuminococcaceaeUCG009.id.11366	rs8009993	14	39306809	G	C	-0.135936179	0.024485659	-5.473121899	4.42E-08	7389
genus.RuminococcaceaeUCG009.id.11366	rs4708333	6	78106701	T	G	-0.084032963	0.017475702	-4.803076287	1.56E-06	7511
genus.RuminococcaceaeUCG009.id.11366	rs61779334	1	39983022	G	C	-0.138137216	0.029207242	-4.759325197	1.94E-06	6865
genus.RuminococcaceaeUCG009.id.11366	rs4079028	1	202299085	C	T	0.091562303	0.019936034	4.65230555	3.28E-06	7480
genus.RuminococcaceaeUCG009.id.11366	rs2058609	12	12970792	A	G	0.081646419	0.017471971	4.662437843	3.12E-06	7510
genus.RuminococcaceaeUCG009.id.11366	rs12508214	4	7426937	C	T	-0.077464871	0.016893699	-4.575322455	4.75E-06	7511
genus.RuminococcaceaeUCG009.id.11366	rs9558661	13	106650093	T	C	-0.089782261	0.020073862	-4.493431117	7.01E-06	7480
genus.RuminococcaceaeUCG009.id.11366	rs2192926	2	75512668	A	G	-0.089048031	0.019298901	-4.56999085	4.88E-06	7511
genus.RuminococcaceaeUCG009.id.11366	rs113006825	6	51803869	T	C	-0.092889294	0.020803474	-4.465800008	7.98E-06	7165
genus.RuminococcaceaeUCG009.id.11366	rs138460696	2	66396776	A	G	0.13927429	0.031579435	4.42135451	9.81E-06	6865
genus.RuminococcaceaeUCG009.id.11366	rs78410648	19	52399401	A	G	0.120988897	0.027731677	4.424378548	9.67E-06	7510
genus.RuminococcaceaeUCG010.id.11367	rs35506912	15	36607072	G	C	-0.069374287	0.014792554	-4.656956518	3.21E-06	12793
genus.RuminococcaceaeUCG010.id.11367	rs682403	9	135968557	A	G	-0.058816059	0.01246713	-4.719403492	2.37E-06	12864
genus.RuminococcaceaeUCG010.id.11367	rs6958419	7	16349864	C	T	-0.058571831	0.012499376	-4.681947791	2.84E-06	12870
genus.RuminococcaceaeUCG010.id.11367	rs12597105	16	5233941	G	A	0.067085456	0.014441482	4.570182491	4.87E-06	12415
genus.RuminococcaceaeUCG010.id.11367	rs7935775	11	130676996	A	T	-0.063135075	0.013795347	-4.565030156	4.99E-06	12864
genus.RuminococcaceaeUCG010.id.11367	rs73218807	4	31057482	G	A	-0.166210686	0.036793834	-4.511722415	6.43E-06	6714

Gut	SNP	CHR	POS	A1	A2	BETA	SE	Z	P	N
genus.RuminococcaceaeUCG010.id.11367	rs7441445	4	40707798	C	T	-0.056949974	0.012650232	-4.499795969	6.80E-06	12852
genus.RuminococcaceaeUCG011.id.11368	rs1416041	6	105073676	A	C	-0.182339224	0.033989816	-5.390040172	7.04E-08	3632
genus.RuminococcaceaeUCG011.id.11368	rs12724320	1	179370499	C	T	-0.120880657	0.024922917	-4.808606427	1.52E-06	3632
genus.RuminococcaceaeUCG011.id.11368	rs79113084	12	29634919	C	T	-0.152165548	0.031751932	-4.747326119	2.06E-06	3610
genus.RuminococcaceaeUCG011.id.11368	rs9729514	1	219901055	A	G	0.18493353	0.039461636	4.719203944	2.37E-06	3632
genus.RuminococcaceaeUCG011.id.11368	rs2729556	7	111763988	C	T	-0.109096996	0.023369889	-4.658398357	3.19E-06	3632
genus.RuminococcaceaeUCG011.id.11368	rs4490371	3	76205107	T	C	-0.111816319	0.024896269	-4.472080001	7.75E-06	3610
genus.RuminococcaceaeUCG011.id.11368	rs12636310	3	185469491	G	A	0.132725381	0.028203654	4.684068871	2.81E-06	3632
genus.RuminococcaceaeUCG011.id.11368	rs4568515	7	11236089	C	T	0.111163536	0.024535563	4.507719107	6.55E-06	3632
genus.RuminococcaceaeUCG013.id.11370	rs12781711	10	2219930	C	T	-0.065612917	0.011747657	-5.569616054	2.55E-08	16772
genus.RuminococcaceaeUCG013.id.11370	rs12189346	5	141395140	G	A	0.068496125	0.014558037	4.788592668	1.68E-06	16470
genus.RuminococcaceaeUCG013.id.11370	rs75088940	12	30245557	T	C	-0.094296811	0.02007125	-4.703771814	2.55E-06	15638
genus.RuminococcaceaeUCG013.id.11370	rs16918863	10	19780463	A	C	0.111491182	0.024015687	4.603472946	4.16E-06	12643
genus.RuminococcaceaeUCG013.id.11370	rs76973485	3	9534657	G	T	0.194975714	0.041821342	4.648116324	3.35E-06	5299
genus.RuminococcaceaeUCG013.id.11370	rs9565219	13	76583319	T	A	-0.052459177	0.011770144	-4.446569535	8.73E-06	16772
genus.RuminococcaceaeUCG013.id.11370	rs1729063	16	10863459	G	C	-0.053341105	0.01207565	-4.425188561	9.64E-06	16470
genus.RuminococcaceaeUCG013.id.11370	rs7784330	7	32530997	G	A	-0.049832949	0.011206613	-4.460874137	8.16E-06	16770
genus.RuminococcaceaeUCG013.id.11370	rs11581881	1	9361576	C	T	0.066121072	0.014473649	4.57660524	4.73E-06	15730
genus.RuminococcaceaeUCG013.id.11370	rs2428106	9	136620584	C	G	-0.0490863	0.010990264	-4.455339583	8.38E-06	16768
genus.RuminococcaceaeUCG013.id.11370	rs2730183	8	40311885	G	A	-0.048869155	0.010990742	-4.453761809	8.44E-06	16772
genus.RuminococcaceaeUCG013.id.11370	rs9313055	5	3628882	T	C	0.105087207	0.023446005	4.427184011	9.55E-06	15764
genus.RuminococcaceaeUCG013.id.11370	rs12336782	9	13609119	T	C	-0.085604658	0.018931084	-4.449599584	8.60E-06	16772
genus.RuminococcaceaeUCG013.id.11370	rs4385846	10	115650278	G	T	0.05984045	0.013180685	4.510677482	6.46E-06	16772
genus.RuminococcaceaeUCG014.id.11371	rs115777838	5	26110626	T	C	-0.188349525	0.038664295	-5.041320103	4.62E-07	5674
genus.RuminococcaceaeUCG014.id.11371	rs72809222	2	57205854	T	C	0.067177506	0.013983796	4.71543841	2.41E-06	13922
genus.RuminococcaceaeUCG014.id.11371	rs12638134	3	101334609	T	G	0.058254795	0.011965732	4.853757897	1.21E-06	14403
genus.RuminococcaceaeUCG014.id.11371	rs56105232	9	14363769	G	A	0.139275689	0.029913177	4.676875121	2.91E-06	9190
genus.RuminococcaceaeUCG014.id.11371	rs995642	2	134854659	C	T	0.060047967	0.012641687	4.763543689	1.90E-06	14402
genus.RuminococcaceaeUCG014.id.11371	rs10941294	5	36435597	C	T	-0.122057405	0.026001669	-4.716700666	2.40E-06	12615
genus.RuminococcaceaeUCG014.id.11371	rs79640386	18	4084336	T	A	-0.110863476	0.024809787	-4.446266715	8.74E-06	13692
genus.RuminococcaceaeUCG014.id.11371	rs439810	2	119467367	G	C	-0.057712693	0.012667538	-4.49241882	7.04E-06	14403

Gut	SNP	CHR	POS	A1	A2	BETA	SE	Z	P	N
genus.RuminococcaceaeUCG014.id.11371	rs10495392	1	237422989	C	T	-0.082485954	0.018719441	-4.417968638	9.96E-06	13902
genus.RuminococcaceaeUCG014.id.11371	rs62478832	7	109564034	T	A	-0.058128986	0.012903031	-4.524834692	6.04E-06	14331
genus.RuminococcaceaeUCG014.id.11371	rs61898819	11	126024042	A	T	0.060786464	0.01387607	4.418955933	9.92E-06	14325
genus.RuminococcaceaeUCG014.id.11371	rs73186226	3	123829361	G	A	-0.099385194	0.02168186	-4.502448958	6.72E-06	13760
genus.RuminococcaceaeUCG014.id.11371	rs34402072	8	3636548	C	T	-0.068802318	0.015608285	-4.421445917	9.80E-06	13863
genus.RuminococcaceaeUCG014.id.11371	rs74060145	1	15322582	C	G	-0.115766761	0.025419855	-4.447066291	8.71E-06	13011
genus.RuminococcaceaeUCG014.id.11371	rs77627087	16	87759750	C	G	0.067561994	0.015010339	4.481079217	7.43E-06	14390
genus.RuminococcaceaeUCG014.id.11371	rs17296933	19	49175964	C	G	-0.08295439	0.018505763	-4.483663392	7.34E-06	13177
genus.RuminococcaceaeUCG014.id.11371	rs10791168	11	131659000	A	G	-0.066462357	0.015006876	-4.422383034	9.76E-06	14403
genus.Ruminococcus1.id.11373	rs10769159	11	45688595	G	C	-0.064027674	0.011005922	-5.837742606	5.29E-09	16603
genus.Ruminococcus1.id.11373	rs7117576	11	114023573	A	G	0.082950453	0.017089133	4.976339674	6.48E-07	16606
genus.Ruminococcus1.id.11373	rs17781867	17	71256018	C	T	0.099927121	0.021172816	4.757160192	1.96E-06	14349
genus.Ruminococcus1.id.11373	rs7583465	2	10533921	C	T	0.052765423	0.011261892	4.703600836	2.56E-06	16588
genus.Ruminococcus1.id.11373	rs78613526	2	65925212	G	A	0.167496641	0.036763917	4.560045862	5.11E-06	7266
genus.Ruminococcus1.id.11373	rs78572139	5	15944587	G	A	0.1250377	0.027941129	4.555301618	5.23E-06	11667
genus.Ruminococcus1.id.11373	rs3000856	1	12778598	T	A	-0.070999702	0.016276599	-4.433380509	9.28E-06	16126
genus.Ruminococcus1.id.11373	rs6493760	15	55439182	C	T	0.053526195	0.011588457	4.646042926	3.38E-06	16126
genus.Ruminococcus1.id.11373	rs11783695	8	144596903	G	T	-0.07341143	0.016139812	-4.57656241	4.73E-06	16606
genus.Ruminococcus1.id.11373	rs10995816	10	65870347	C	G	-0.07620021	0.017468886	-4.455172502	8.38E-06	16597
genus.Ruminococcus1.id.11373	rs10167839	2	181882879	A	G	0.051950897	0.011630648	4.462721019	8.09E-06	16605
genus.Ruminococcus1.id.11373	rs3819978	1	241789757	C	T	-0.115039796	0.026013681	-4.446173615	8.74E-06	12769
genus.Ruminococcus1.id.11373	rs4849717	2	119481372	T	A	0.132763838	0.029981613	4.444097499	8.83E-06	10484
genus.Ruminococcus1.id.11373	rs6105066	20	13258628	T	C	-0.060705188	0.01342499	-4.562320423	5.06E-06	16049
genus.Ruminococcus2.id.11374	rs78120384	3	106664363	A	G	-0.192815497	0.039204507	-5.104776732	3.31E-07	6082
genus.Ruminococcus2.id.11374	rs12986628	2	238103439	C	T	0.066593727	0.014016808	4.739541503	2.14E-06	14714
genus.Ruminococcus2.id.11374	rs7635831	3	173040924	G	A	0.061834357	0.012903108	4.755399535	1.98E-06	14924
genus.Ruminococcus2.id.11374	rs2997412	13	62067781	A	G	-0.056843902	0.012232461	-4.600184815	4.22E-06	15336
genus.Ruminococcus2.id.11374	rs2368224	2	182656416	T	G	0.199647633	0.043845344	4.631554866	3.63E-06	3026
genus.Ruminococcus2.id.11374	rs4400279	7	16804578	A	G	0.05461138	0.012007747	4.533577766	5.80E-06	15333
genus.Ruminococcus2.id.11374	rs58681734	9	137557890	A	G	0.07238752	0.016078702	4.602138363	4.18E-06	15338
genus.Ruminococcus2.id.11374	rs1819812	7	151435722	G	T	0.084235642	0.018489341	4.55316996	5.28E-06	14868

Gut	SNP	CHR	POS	A1	A2	BETA	SE	Z	P	N
genus.Ruminococcus2.id.11374	rs12406309	1	108074445	A	C	-0.063192979	0.014227548	-4.421770809	9.79E-06	15339
genus.Ruminococcus2.id.11374	rs2846589	18	862477	G	T	0.052209321	0.011638161	4.476463494	7.59E-06	14924
genus.Ruminococcus2.id.11374	rs55707116	9	79919385	C	A	0.08655215	0.01891409	4.465025164	8.01E-06	15336
genus.Ruminococcus2.id.11374	rs75140805	11	12148588	T	G	0.083682495	0.017644509	4.613839714	3.95E-06	14249
genus.Ruminococcus2.id.11374	rs4799823	18	33456427	C	T	0.083700126	0.018226258	4.548561892	5.40E-06	14650
genus.Ruminococcus2.id.11374	rs61791565	4	24655463	T	C	-0.052350302	0.011711882	-4.500188208	6.79E-06	15319
genus.Ruminococcus2.id.11374	rs7693984	4	4268322	G	A	-0.102821803	0.023508663	-4.430045092	9.42E-06	14154
genus.Sellimonas.id.14369	rs13417181	2	174308394	T	C	0.166509314	0.033752138	4.944920983	7.62E-07	3047
genus.Sellimonas.id.14369	rs2371572	2	213214174	A	C	0.127348939	0.025086238	5.048043733	4.46E-07	3154
genus.Sellimonas.id.14369	rs62016462	15	52485441	T	C	0.233969428	0.0484278	4.874744195	1.09E-06	3153
genus.Sellimonas.id.14369	rs113379006	3	42008186	T	C	-0.162758788	0.035702495	-4.487459044	7.21E-06	3154
genus.Sellimonas.id.14369	rs2187447	11	79332972	A	C	0.243457117	0.052769543	4.612525258	3.98E-06	3028
genus.Sellimonas.id.14369	rs72553859	1	247579710	G	C	-0.150458067	0.033262641	-4.549518805	5.38E-06	3154
genus.Sellimonas.id.14369	rs56203279	7	111501524	T	C	-0.124045002	0.026911497	-4.626443304	3.72E-06	3154
genus.Sellimonas.id.14369	rs553697	6	93730393	T	C	-0.153767275	0.033909892	-4.521933535	6.13E-06	3047
genus.Sellimonas.id.14369	rs6433790	2	180277875	C	T	-0.138530029	0.030169765	-4.612636802	3.98E-06	3095
genus.Sellimonas.id.14369	rs7968030	12	100709313	A	T	-0.127110283	0.028061737	-4.542307976	5.56E-06	3095
genus.Sellimonas.id.14369	rs17657328	6	101614312	C	T	0.114571353	0.025361755	4.458548875	8.25E-06	3154
genus.Sellimonas.id.14369	rs41816	7	106250164	A	G	0.132201227	0.029108726	4.454887506	8.39E-06	3154
genus.Senegalimassilia.id.11160	rs11787826	9	34332382	C	A	0.081327043	0.017115415	4.697421959	2.63E-06	6923
genus.Senegalimassilia.id.11160	rs72887800	17	74949284	T	A	-0.082246156	0.017572066	-4.715148891	2.42E-06	6923
genus.Senegalimassilia.id.11160	rs7225245	17	48379615	G	A	0.079173009	0.017042101	4.602216991	4.18E-06	6923
genus.Senegalimassilia.id.11160	rs57512504	13	19489214	T	A	0.081949442	0.017224097	4.750098488	2.03E-06	6923
genus.Senegalimassilia.id.11160	rs10036909	5	127861692	C	T	0.185519381	0.040088092	4.463733277	8.05E-06	4996
genus.Senegalimassilia.id.11160	rs13383270	2	220831701	G	C	0.077467918	0.017084039	4.52500398	6.04E-06	6923
genus.Senegalimassilia.id.11160	rs1990708	2	206654123	A	C	-0.109619487	0.024778524	-4.442166004	8.91E-06	6923
genus.Senegalimassilia.id.11160	rs2017373	14	34431996	C	T	0.078225822	0.017684094	4.428186224	9.50E-06	6923
genus.Slackia.id.825	rs8901	17	74267010	C	T	0.093459378	0.018681296	4.988977179	6.07E-07	6063
genus.Slackia.id.825	rs4492265	7	13523683	A	G	-0.0905757	0.019165844	-4.715662668	2.41E-06	6063
genus.Slackia.id.825	rs16894137	8	96946291	C	T	-0.12279207	0.026304664	-4.691519718	2.71E-06	6063
genus.Slackia.id.825	rs112764253	7	78282725	T	A	0.194713531	0.041159784	4.645308033	3.40E-06	5634

Gut	SNP	CHR	POS	A1	A2	BETA	SE	Z	P	N
genus.Slackia.id.825	rs12440440	15	34041896	A	G	0.090193357	0.019058248	4.697883188	2.63E-06	6063
genus.Slackia.id.825	rs35156985	7	99451715	T	C	-0.155708877	0.034808468	-4.463598131	8.06E-06	5965
genus.Slackia.id.825	rs10409783	19	4555786	A	G	0.095082845	0.021123946	4.473473244	7.70E-06	5941
genus.Slackia.id.825	rs13339230	16	7460475	C	G	0.147054846	0.033063509	4.48126317	7.42E-06	6063
genus.Streptococcus.id.1853	rs11110281	12	100584014	T	C	-0.137518884	0.022739814	-5.956108743	2.58E-09	15120
genus.Streptococcus.id.1853	rs4968759	17	61298020	A	G	-0.051510944	0.011206836	-4.623062258	3.78E-06	16384
genus.Streptococcus.id.1853	rs11764382	7	46774896	A	G	-0.06953447	0.014367132	-4.84185751	1.29E-06	16387
genus.Streptococcus.id.1853	rs72739637	15	41663393	A	G	0.095994182	0.019321261	4.885232916	1.03E-06	14920
genus.Streptococcus.id.1853	rs1918540	11	131649446	G	A	0.059639038	0.012814811	4.71302409	2.44E-06	16373
genus.Streptococcus.id.1853	rs17708276	8	10199548	A	G	-0.079395519	0.017062753	-4.6680336	3.04E-06	16278
genus.Streptococcus.id.1853	rs77558518	5	174173171	A	G	-0.103999229	0.022971364	-4.577369278	4.71E-06	14949
genus.Streptococcus.id.1853	rs11720390	3	94103591	G	A	0.107023904	0.022812145	4.633530289	3.59E-06	15220
genus.Streptococcus.id.1853	rs395407	5	169689604	G	C	-0.079278061	0.01736973	-4.593198618	4.37E-06	15864
genus.Streptococcus.id.1853	rs10448310	9	93556174	A	G	-0.051793473	0.011132356	-4.650764904	3.31E-06	16384
genus.Streptococcus.id.1853	rs8191642	8	11640784	G	A	-0.060418949	0.013670956	-4.462684205	8.09E-06	16384
genus.Streptococcus.id.1853	rs71481756	10	8102404	T	G	0.09310478	0.020794918	4.508955445	6.51E-06	14526
genus.Streptococcus.id.1853	rs2370083	14	97526750	G	T	-0.081683584	0.018585095	-4.422590536	9.75E-06	16277
genus.Streptococcus.id.1853	rs10028567	4	53657577	C	T	-0.092116652	0.019188114	-4.484648311	7.30E-06	16311
genus.Streptococcus.id.1853	rs6806351	3	132058723	T	C	-0.063382863	0.013664741	-4.567375248	4.94E-06	15973
genus.Streptococcus.id.1853	rs9903102	17	80576624	C	A	-0.070948345	0.015527498	-4.602226708	4.18E-06	16384
genus.Streptococcus.id.1853	rs6563952	16	73391982	G	C	0.082734414	0.018003533	4.532776603	5.82E-06	16387
genus.Subdoligranulum.id.2070	rs12638227	3	62327558	G	C	-0.055728761	0.010855202	-5.159426635	2.48E-07	17165
genus.Subdoligranulum.id.2070	rs10065321	5	141857415	T	C	-0.051282785	0.010810523	-4.743405611	2.10E-06	17589
genus.Subdoligranulum.id.2070	rs4347804	2	218215847	A	G	0.166063165	0.035748326	4.735602645	2.18E-06	4312
genus.Subdoligranulum.id.2070	rs6555306	5	4671383	T	C	-0.074077668	0.015546287	-4.684485412	2.81E-06	17591
genus.Subdoligranulum.id.2070	rs2114677	10	125472937	C	T	-0.104176347	0.023082878	-4.691110313	2.72E-06	15566
genus.Subdoligranulum.id.2070	rs2572356	4	48979085	G	A	-0.053103072	0.011785143	-4.523283458	6.09E-06	17165
genus.Subdoligranulum.id.2070	rs75158211	19	29644694	T	C	-0.07234304	0.015933044	-4.478366928	7.52E-06	17165
genus.Subdoligranulum.id.2070	rs2171249	6	153694029	C	T	0.10674366	0.023321252	4.58658438	4.51E-06	14679
genus.Subdoligranulum.id.2070	rs35940633	14	86933898	G	A	-0.051100832	0.011001029	-4.600391397	4.22E-06	17582
genus.Subdoligranulum.id.2070	rs76528319	5	117799448	G	T	-0.143278588	0.031066225	-4.481444968	7.41E-06	8468

Gut	SNP	CHR	POS	A1	A2	BETA	SE	Z	P	N
genus.Subdoligranulum.id.2070	rs1667315	2	232408743	G	A	0.0485017	0.010745207	4.502324409	6.72E-06	17586
genus.Subdoligranulum.id.2070	rs76664262	3	183716408	T	A	0.083407827	0.018535669	4.570163453	4.87E-06	16712
genus.Subdoligranulum.id.2070	rs10497836	2	200288718	C	T	-0.052406498	0.011869733	-4.455250193	8.38E-06	17579
genus.Subdoligranulum.id.2070	rs16962433	16	13684415	A	T	0.085599862	0.018921315	4.474882427	7.65E-06	15857
genus.Sutterella.id.2896	rs13173038	5	58499183	A	G	-0.071807564	0.015162698	-4.690165601	2.73E-06	13245
genus.Sutterella.id.2896	rs2321387	13	58689340	G	A	-0.059287622	0.012450922	-4.767507802	1.87E-06	13252
genus.Sutterella.id.2896	rs7499539	16	85038065	A	G	0.061747829	0.013099845	4.720065441	2.36E-06	13254
genus.Sutterella.id.2896	rs62501473	7	150126751	G	A	0.069423259	0.014941649	4.544044204	5.52E-06	12762
genus.Sutterella.id.2896	rs143438747	1	63278549	T	C	-0.145792693	0.030686706	-4.652445627	3.28E-06	9368
genus.Sutterella.id.2896	rs9350083	6	18583878	T	G	-0.059306886	0.013392944	-4.459169705	8.23E-06	13254
genus.Sutterella.id.2896	rs7638039	3	70588939	T	C	0.064562374	0.014388651	4.448083784	8.66E-06	13236
genus.Sutterella.id.2896	rs11591622	10	102520687	T	G	-0.0688382	0.015137492	-4.509483214	6.50E-06	12604
genus.Sutterella.id.2896	rs2613606	7	111285025	C	T	-0.055679377	0.012411747	-4.487620769	7.20E-06	13249
genus.Sutterella.id.2896	rs1145877	6	82386821	A	G	-0.073548024	0.016241333	-4.487703903	7.20E-06	13251
genus.Sutterella.id.2896	rs607327	11	111695709	C	T	0.057833353	0.012905264	4.505214285	6.63E-06	13251
genus.Sutterella.id.2896	rs2050185	6	147936781	G	A	0.057513842	0.012876636	4.466000407	7.97E-06	13243
genus.Terrisporobacter.id.11348	rs1883097	11	8938819	C	T	0.226398815	0.045464179	5.061583501	4.16E-07	4417
genus.Terrisporobacter.id.11348	rs2569953	3	144148905	A	C	-0.077559072	0.01746408	-4.441098384	8.95E-06	6637
genus.Terrisporobacter.id.11348	rs7184125	16	15920394	T	C	0.091256006	0.020549332	4.45266671	8.48E-06	6232
genus.Terrisporobacter.id.11348	rs2872237	19	17026940	C	A	-0.08145049	0.017594312	-4.612875887	3.97E-06	6632
genus.Terrisporobacter.id.11348	rs58405430	11	20621043	G	T	0.134617158	0.030117736	4.46687366	7.94E-06	6176
genus.Terrisporobacter.id.11348	rs7034891	9	117245252	G	C	-0.079920945	0.01737936	-4.585096693	4.54E-06	6629
genus.Turicibacter.id.2162	rs149744580	2	63366962	A	G	0.169882696	0.031547837	5.390923517	7.01E-08	8459
genus.Turicibacter.id.2162	rs4869133	5	95717619	G	A	0.131186095	0.027196968	4.703795256	2.55E-06	8256
genus.Turicibacter.id.2162	rs12603364	17	43117476	T	C	0.110860918	0.022559841	4.919738749	8.67E-07	8923
genus.Turicibacter.id.2162	rs11054680	12	12242455	T	C	-0.104751216	0.02269975	-4.72424075	2.31E-06	8928
genus.Turicibacter.id.2162	rs55756211	7	130972179	T	C	-0.115115214	0.024070764	-4.68458099	2.81E-06	8941
genus.Turicibacter.id.2162	rs7199484	16	49826299	G	A	-0.07314282	0.016017166	-4.534769763	5.77E-06	8948
genus.Turicibacter.id.2162	rs11649454	16	10181248	G	C	0.095089088	0.020343331	4.653323624	3.27E-06	8943
genus.Turicibacter.id.2162	rs2834977	21	36929643	T	C	-0.095999549	0.020826107	-4.613687113	3.96E-06	8946
genus.Turicibacter.id.2162	rs11666533	19	11864932	C	T	-0.111689111	0.024843618	-4.48268155	7.37E-06	8256

Gut	SNP	CHR	POS	A1	A2	BETA	SE	Z	P	N
genus.Turicibacter.id.2162	rs4247078	15	92605098	C	G	0.071037672	0.015522123	4.546259406	5.46E-06	8948
genus.Turicibacter.id.2162	rs2221441	5	170048048	G	C	0.071036413	0.015343033	4.641632727	3.46E-06	8720
genus.Turicibacter.id.2162	rs3734633	6	126111881	G	A	-0.120956846	0.026830036	-4.551789997	5.32E-06	8626
genus.Tyzzerella3.id.11335	rs67476743	19	1030320	T	G	0.132163631	0.02220797	5.895278989	3.74E-09	4948
genus.Tyzzerella3.id.11335	rs17706273	5	16388259	T	C	-0.140368365	0.027470793	-4.995177695	5.88E-07	5395
genus.Tyzzerella3.id.11335	rs7561370	2	57810406	T	C	0.131341092	0.028629245	4.809108186	1.52E-06	5396
genus.Tyzzerella3.id.11335	rs55799124	17	3738781	A	G	-0.114350138	0.023860417	-4.834396495	1.34E-06	5362
genus.Tyzzerella3.id.11335	rs17809157	7	82547827	A	T	-0.163819473	0.033638641	-4.805800897	1.54E-06	4621
genus.Tyzzerella3.id.11335	rs75091807	13	35023691	G	T	-0.184966239	0.038302914	-4.785368423	1.71E-06	4621
genus.Tyzzerella3.id.11335	rs4904512	14	89595945	T	C	-0.117150935	0.025030561	-4.664566042	3.09E-06	5396
genus.Tyzzerella3.id.11335	rs7333521	13	81593016	T	C	-0.20719145	0.045312058	-4.570052549	4.88E-06	4335
genus.Tyzzerella3.id.11335	rs112102233	10	46098286	A	G	-0.216348445	0.04775752	-4.520249228	6.18E-06	3857
genus.Tyzzerella3.id.11335	rs1232220	6	102680257	G	T	-0.143869315	0.031828041	-4.46766182	7.91E-06	5000
genus.Tyzzerella3.id.11335	rs191093	12	77390329	G	A	0.15900811	0.035330566	4.500985016	6.76E-06	4621
genus.Tyzzerella3.id.11335	rs10898797	11	87588698	C	T	0.122380253	0.027468252	4.443409016	8.85E-06	5387
genus.Tyzzerella3.id.11335	rs6920448	6	5755626	C	T	-0.141080632	0.030544578	-4.603536506	4.15E-06	5010
genus.unknowngenus.id.1000000073	rs55682560	15	87392290	C	T	-0.131519432	0.026131877	-5.027454421	4.97E-07	9183
genus.unknowngenus.id.1000000073	rs9979874	21	38612024	G	C	-0.073892457	0.015091139	-4.881519575	1.05E-06	10506
genus.unknowngenus.id.1000000073	rs28691777	17	58149405	C	T	0.137133741	0.026699624	4.962561049	6.96E-07	10199
genus.unknowngenus.id.1000000073	rs6588624	1	56849539	G	A	-0.066231659	0.013814656	-4.775471324	1.79E-06	10512
genus.unknowngenus.id.1000000073	rs7538034	1	71391694	T	G	-0.078597952	0.016598223	-4.719258778	2.37E-06	10420
genus.unknowngenus.id.1000000073	rs66714985	8	3305122	A	C	0.116907778	0.025244679	4.571029445	4.85E-06	10512
genus.unknowngenus.id.1000000073	rs118104867	8	123977538	C	T	0.214463834	0.04550976	4.642872677	3.44E-06	4095
genus.unknowngenus.id.1000000073	rs2191834	2	229942951	G	T	0.074637506	0.015913614	4.707971146	2.50E-06	10511
genus.unknowngenus.id.1000000073	rs7226487	18	74373125	A	G	-0.064368248	0.013870145	-4.634221964	3.58E-06	10505
genus.unknowngenus.id.1000000073	rs10517600	4	155632958	T	G	0.062699295	0.01393642	4.498990761	6.83E-06	10512
genus.unknowngenus.id.1000000073	rs17121075	14	85856796	G	A	0.07692543	0.017223352	4.467492686	7.91E-06	10512
genus.unknowngenus.id.1000000073	rs10904722	10	6714424	C	T	-0.067231397	0.014712327	-4.562767694	5.05E-06	10512
genus.unknowngenus.id.1000000073	rs6755871	2	202450739	C	G	-0.061382475	0.013897576	-4.432132023	9.33E-06	10500
genus.unknowngenus.id.1000000073	rs34088226	5	4308946	A	G	-0.117806973	0.026923974	-4.474412635	7.66E-06	9716
genus.unknowngenus.id.1000000073	rs13409132	2	205616346	A	G	-0.165418684	0.035215443	-4.59285117	4.37E-06	5913

Gut	SNP	CHR	POS	A1	A2	BETA	SE	Z	P	N
genus.unknowngenus.id.1000000073	rs989682	3	15564453	A	G	0.070193983	0.01553641	4.498387002	6.85E-06	10129
genus.unknowngenus.id.1000001215	rs9864379	3	14306949	T	C	-0.160521381	0.029275217	-5.463866794	4.66E-08	5768
genus.unknowngenus.id.1000001215	rs11150282	16	80493705	T	C	0.098234998	0.019712655	4.951601318	7.36E-07	5864
genus.unknowngenus.id.1000001215	rs16851659	1	230523039	G	C	-0.089516816	0.01865552	-4.844354276	1.27E-06	5875
genus.unknowngenus.id.1000001215	rs4129395	9	115975389	G	A	0.090426365	0.01852532	4.852780703	1.22E-06	5878
genus.unknowngenus.id.1000001215	rs1221147	9	122046549	T	A	0.126305018	0.027916132	4.570759409	4.86E-06	5759
genus.unknowngenus.id.1000001215	rs79790072	15	100747683	T	C	0.226102517	0.048801659	4.636820109	3.54E-06	4104
genus.unknowngenus.id.1000001215	rs73074665	7	20010624	A	T	0.164631221	0.035603665	4.631868601	3.62E-06	5749
genus.unknowngenus.id.1000001215	rs113884518	9	24648997	T	C	-0.205909694	0.045537561	-4.472255993	7.74E-06	4434
genus.unknowngenus.id.1000001215	rs789069	18	1008278	A	C	-0.104129854	0.023445913	-4.509435534	6.50E-06	5870
genus.unknowngenus.id.1000001215	rs367480	11	2937631	G	A	-0.084164921	0.018594792	-4.478294302	7.52E-06	5878
genus.unknowngenus.id.1000001215	rs8028558	15	62055753	A	G	0.083459982	0.018850404	4.421929013	9.78E-06	5875
genus.unknowngenus.id.1000001215	rs28678345	17	53906328	T	C	0.213082993	0.047137686	4.463596364	8.06E-06	4315
genus.unknowngenus.id.1000005472	rs74603314	14	46519718	T	C	0.230767094	0.048970099	4.727196832	2.28E-06	4033
genus.unknowngenus.id.1000005472	rs76373661	3	3771148	G	A	0.090821805	0.020298596	4.558199311	5.16E-06	10359
genus.unknowngenus.id.1000005472	rs17235252	7	82536037	T	C	-0.12200211	0.02554629	-4.737961619	2.16E-06	9531
genus.unknowngenus.id.1000005472	rs638542	1	109845252	G	A	-0.070591966	0.015704899	-4.557652462	5.17E-06	9970
genus.unknowngenus.id.1000005472	rs62188991	2	177374176	G	C	-0.110864082	0.02413552	-4.553860627	5.27E-06	9531
genus.unknowngenus.id.1000005472	rs949341	11	112386785	G	A	0.065660222	0.014721644	4.472450651	7.73E-06	10355
genus.unknowngenus.id.1000005472	rs13100746	3	165846506	C	T	0.063897883	0.014268538	4.485085885	7.29E-06	10359
genus.unknowngenus.id.1000005472	rs515984	10	3521459	T	C	-0.087545066	0.019068514	-4.505757535	6.61E-06	10359
genus.unknowngenus.id.1000005472	rs7706512	5	17443654	G	A	0.065735114	0.013897929	4.727583749	2.27E-06	10359
genus.unknowngenus.id.1000005472	rs11779863	8	15406830	G	A	-0.077276806	0.017217056	-4.503369443	6.69E-06	10353
genus.unknowngenus.id.1000005472	rs7801843	7	4105705	A	G	-0.086949468	0.01945094	-4.428960862	9.47E-06	10359
genus.unknowngenus.id.1000005472	rs10071529	5	176287195	G	C	0.125104505	0.027867407	4.448578118	8.64E-06	10147
genus.unknowngenus.id.1000005472	rs739151	22	42741466	C	G	0.065265852	0.013977274	4.664554003	3.09E-06	10337
genus.unknowngenus.id.1000005472	rs114184530	9	116018011	T	C	-0.1545446	0.033999183	-4.468553952	7.88E-06	7196
genus.unknowngenus.id.1000005472	rs12566890	1	61850864	T	G	-0.10313363	0.024177477	-4.462206342	8.11E-06	10339
genus.unknowngenus.id.1000005472	rs3932485	3	184193482	C	T	0.062620895	0.014162324	4.418723133	9.93E-06	10359
genus.unknowngenus.id.1000005472	rs7853673	9	116711241	G	A	-0.062438944	0.013983251	-4.501974774	6.73E-06	10359
genus.unknowngenus.id.1000005479	rs738193	22	25943322	T	C	0.084732776	0.016585588	5.077634559	3.82E-07	7778

Gut	SNP	CHR	POS	A1	A2	BETA	SE	Z	P	N
genus.unknowngenus.id.1000005479	rs941000	7	90714667	C	T	0.085026574	0.016338237	5.113910618	3.16E-07	7778
genus.unknowngenus.id.1000005479	rs689695	15	47352866	C	A	0.081457787	0.016705675	4.84277449	1.28E-06	7954
genus.unknowngenus.id.1000005479	rs17043785	2	53139362	T	C	-0.176186835	0.034713295	-5.02182162	5.12E-07	5208
genus.unknowngenus.id.1000005479	rs10872669	6	151515172	A	G	-0.123070536	0.027566518	-4.428476662	9.49E-06	7368
genus.unknowngenus.id.1000005479	rs12748533	1	242767252	G	T	-0.082110804	0.017270056	-4.700898483	2.59E-06	7948
genus.unknowngenus.id.1000005479	rs6831034	4	149300138	T	A	-0.095654602	0.020522806	-4.522755847	6.10E-06	7576
genus.unknowngenus.id.1000005479	rs61508842	3	158653545	T	C	0.122574311	0.027238851	4.469839849	7.83E-06	7576
genus.unknowngenus.id.1000005479	rs78609301	6	63236747	A	G	-0.086706514	0.019570857	-4.490879392	7.09E-06	7953
genus.unknowngenus.id.1000005479	rs11135366	5	164763068	C	G	0.084214168	0.01837388	4.445268389	8.78E-06	7953
genus.unknowngenus.id.1000005479	rs7217209	17	21205200	C	T	0.084301877	0.018730791	4.45397817	8.43E-06	7745
genus.unknowngenus.id.1000006162	rs60583455	7	1321675	T	C	0.108933641	0.021176966	5.150655336	2.60E-07	4773
genus.unknowngenus.id.1000006162	rs2172426	8	18229020	C	T	-0.102119829	0.019894658	-5.112795913	3.17E-07	5099
genus.unknowngenus.id.1000006162	rs11251024	10	2095726	G	A	0.104201088	0.020673705	4.971957983	6.63E-07	5107
genus.unknowngenus.id.1000006162	rs267959	5	10737802	A	G	0.098861186	0.020967556	4.698567115	2.62E-06	5107
genus.unknowngenus.id.1000006162	rs11606187	11	91589600	A	G	-0.154539114	0.032628781	-4.65084005	3.31E-06	5102
genus.unknowngenus.id.1000006162	rs7911787	10	98619568	G	T	-0.223070508	0.047023233	-4.645761783	3.39E-06	3879
genus.unknowngenus.id.1000006162	rs72671304	14	39425971	T	C	0.172321792	0.037002808	4.622262246	3.80E-06	4887
genus.unknowngenus.id.1000006162	rs13385922	2	234081324	T	C	0.092949422	0.020134823	4.613036483	3.97E-06	5106
genus.unknowngenus.id.1000006162	rs55921101	10	127094261	A	T	-0.109245446	0.023620072	-4.595294284	4.32E-06	5105
genus.unknowngenus.id.1000006162	rs9542068	13	70306831	T	C	0.099070907	0.021814934	4.508674582	6.52E-06	5107
genus.unknowngenus.id.1000006162	rs2930903	19	887824	C	G	0.090833483	0.020943373	4.49849862	6.84E-06	4789
genus.unknowngenus.id.1000006162	rs60775321	15	97318671	T	C	-0.096242537	0.021384172	-4.49053038	7.10E-06	5101
genus.unknowngenus.id.1000006162	rs8126061	20	3405239	T	C	-0.159135032	0.035190375	-4.482905922	7.36E-06	5089
genus.unknowngenus.id.1000006162	rs166849	2	200778866	G	A	0.09110531	0.020223942	4.472280499	7.74E-06	5107
genus.unknowngenus.id.1868	rs11709800	3	161557749	T	C	0.08798036	0.01663397	5.254739142	1.48E-07	10293
genus.unknowngenus.id.1868	rs12634544	3	12351583	T	C	-0.081305087	0.015881969	-5.15011688	2.60E-07	10300
genus.unknowngenus.id.1868	rs72819830	2	81684934	A	G	0.226766565	0.046721614	4.7965515	1.61E-06	4399
genus.unknowngenus.id.1868	rs6595770	5	126751728	G	T	-0.085122436	0.017689999	-4.75522617	1.98E-06	10295
genus.unknowngenus.id.1868	rs3010848	1	221945123	G	A	-0.069672776	0.014663068	-4.776587682	1.78E-06	10300
genus.unknowngenus.id.1868	rs17291611	10	109292487	A	G	-0.090569118	0.0190904	-4.725293081	2.30E-06	10008
genus.unknowngenus.id.1868	rs10231386	7	6719867	G	C	-0.172992892	0.039040128	-4.581500109	4.62E-06	6324

Gut	SNP	CHR	POS	A1	A2	BETA	SE	Z	P	N
genus.unknowngenus.id.1868	rs4848741	2	122818391	A	G	-0.068241437	0.014940265	-4.543695624	5.53E-06	10301
genus.unknowngenus.id.1868	rs1044939	12	57449705	C	T	0.109022423	0.024201732	4.493173637	7.02E-06	10175
genus.unknowngenus.id.1868	rs138437711	10	134926480	A	G	-0.087483977	0.019221518	-4.579543114	4.66E-06	9966
genus.unknowngenus.id.1868	rs35947731	7	69979141	C	T	-0.131217986	0.028840707	-4.547375144	5.43E-06	9748
genus.unknowngenus.id.1868	rs12930556	16	8870618	C	T	0.064211031	0.013981592	4.584858115	4.54E-06	10301
genus.unknowngenus.id.1868	rs115372687	1	27221334	A	G	0.108994191	0.024326429	4.436864174	9.13E-06	10018
genus.unknowngenus.id.2001	rs2136103	8	118729182	T	C	0.075453281	0.015403846	4.969133155	6.73E-07	10129
genus.unknowngenus.id.2001	rs6516833	21	29651882	A	G	0.134075146	0.027763614	4.721947244	2.34E-06	9310
genus.unknowngenus.id.2001	rs111373036	1	204056176	A	G	-0.085825912	0.017976634	-4.785801867	1.70E-06	10118
genus.unknowngenus.id.2001	rs2599390	8	70926204	G	A	0.094049006	0.019835459	4.668488586	3.03E-06	9530
genus.unknowngenus.id.2001	rs7334707	13	39470153	T	G	0.118993693	0.026649389	4.517738298	6.25E-06	9782
genus.unknowngenus.id.2001	rs79929882	10	130677483	A	G	-0.10445688	0.023239364	-4.529284904	5.92E-06	9257
genus.unknowngenus.id.2001	rs12696594	3	189357616	A	G	-0.063624893	0.014050224	-4.522565114	6.11E-06	10129
genus.unknowngenus.id.2001	rs58850151	18	39835030	T	C	-0.080893583	0.018153964	-4.479455376	7.48E-06	10129
genus.unknowngenus.id.2001	rs55736413	6	129231261	C	T	0.158131595	0.035761447	4.453501905	8.45E-06	6836
genus.unknowngenus.id.2041	rs35703006	8	28614217	G	T	0.092666931	0.019077889	4.912163069	9.01E-07	7940
genus.unknowngenus.id.2041	rs7187855	16	87893751	A	C	0.199941236	0.041830769	4.733573707	2.21E-06	5059
genus.unknowngenus.id.2041	rs16843660	2	196859205	A	G	0.234697293	0.049069984	4.779946314	1.75E-06	3958
genus.unknowngenus.id.2041	rs1032598	1	217548508	G	A	-0.088642593	0.018903938	-4.607477128	4.08E-06	7944
genus.unknowngenus.id.2041	rs553072	6	153504165	G	A	0.109193438	0.02301976	4.628066496	3.69E-06	7768
genus.unknowngenus.id.2041	rs6514318	20	1322594	T	C	0.128197785	0.028176526	4.549521757	5.38E-06	7352
genus.unknowngenus.id.2041	rs2651663	12	101911079	A	G	-0.076255582	0.01686352	-4.539399435	5.64E-06	7939
genus.unknowngenus.id.2041	rs11941716	4	7649861	A	G	0.101243017	0.0224414	4.438321901	9.07E-06	7473
genus.unknowngenus.id.2041	rs249459	5	4994609	A	G	0.073734102	0.016501801	4.461913583	8.12E-06	7943
genus.unknowngenus.id.2041	rs921383	11	77710841	G	A	0.07231528	0.015970608	4.472557343	7.73E-06	7944
genus.unknowngenus.id.2041	rs1962916	8	137542651	A	G	0.073787605	0.016223151	4.521563136	6.14E-06	7941
genus.unknowngenus.id.2071	rs16823675	2	145173029	C	T	-0.076751466	0.014997279	-5.170598043	2.33E-07	12619
genus.unknowngenus.id.2071	rs12147596	14	104006716	C	T	-0.071981785	0.014160884	-5.132311004	2.86E-07	12619
genus.unknowngenus.id.2071	rs35740166	4	105068883	C	T	-0.112245532	0.022682407	-4.925842374	8.40E-07	11607
genus.unknowngenus.id.2071	rs11195523	10	113086067	A	C	0.068927824	0.014535063	4.716341931	2.40E-06	12617
genus.unknowngenus.id.2071	rs11809762	1	175159585	G	A	-0.093463445	0.019019755	-4.788197531	1.68E-06	12611

Gut	SNP	CHR	POS	A1	A2	BETA	SE	Z	P	N
genus.unknowngenus.id.2071	rs4644504	1	221985829	T	C	-0.096932131	0.021614645	-4.53283772	5.82E-06	12614
genus.unknowngenus.id.2071	rs76532867	12	94212918	T	C	0.112352825	0.024236434	4.703402487	2.56E-06	11387
genus.unknowngenus.id.2071	rs72700702	15	35278445	T	C	-0.091726051	0.018900523	-4.79923442	1.59E-06	11968
genus.unknowngenus.id.2071	rs11684166	2	111817641	A	G	-0.076963494	0.016834857	-4.639579458	3.49E-06	12607
genus.unknowngenus.id.2071	rs72707147	15	26339670	C	T	0.110109404	0.024464418	4.498602438	6.84E-06	11607
genus.unknowngenus.id.2071	rs1455639	8	2259412	G	A	0.076030721	0.016983089	4.469536544	7.84E-06	12358
genus.unknowngenus.id.2071	rs10200320	2	197967710	C	T	0.064113853	0.014276919	4.538680094	5.66E-06	12358
genus.unknowngenus.id.2071	rs2898979	4	54596608	G	C	0.090151535	0.020219892	4.474115205	7.67E-06	11708
genus.unknowngenus.id.2071	rs1809136	2	11152180	G	C	0.099459363	0.022863755	4.455497408	8.37E-06	12614
genus.unknowngenus.id.2071	rs17086536	6	124369748	C	A	-0.10085113	0.02243198	-4.6472565	3.36E-06	12612
genus.unknowngenus.id.2071	rs56975773	12	89157081	T	A	0.113282045	0.024885889	4.474617091	7.65E-06	12616
genus.unknowngenus.id.2071	rs34985298	7	93746275	G	A	-0.062352592	0.013771983	-4.456329395	8.34E-06	12618
genus.unknowngenus.id.2071	rs2939766	11	41417982	A	G	-0.059161052	0.013042014	-4.493341327	7.01E-06	12619
genus.unknowngenus.id.2071	rs6007642	22	47653347	C	T	-0.079141186	0.017795845	-4.41813994	9.96E-06	12358
genus.unknowngenus.id.2755	rs9813022	3	13726736	A	G	-0.08227975	0.01640101	-5.022413931	5.10E-07	8454
genus.unknowngenus.id.2755	rs76784716	2	169033340	A	G	0.13975602	0.028644423	4.925131259	8.43E-07	8454
genus.unknowngenus.id.2755	rs55876211	3	84471266	C	T	-0.091476372	0.019691933	-4.680157688	2.87E-06	8450
genus.unknowngenus.id.2755	rs3754624	2	225633812	C	T	0.096447803	0.020058915	4.738135852	2.16E-06	8452
genus.unknowngenus.id.2755	rs1549633	5	27945645	A	C	0.101843635	0.021925199	4.645288777	3.40E-06	7966
genus.unknowngenus.id.2755	rs56250014	8	131949033	A	T	0.119152976	0.025758007	4.653809427	3.26E-06	8457
genus.unknowngenus.id.2755	rs11630875	15	61775734	T	C	0.092587688	0.020402093	4.495679689	6.93E-06	8449
genus.unknowngenus.id.2755	rs12977163	19	28641647	G	C	0.074485326	0.016390887	4.550117499	5.36E-06	8000
genus.unknowngenus.id.2755	rs61933850	12	73139398	G	A	0.165183947	0.03614692	4.481019087	7.43E-06	6796
genus.unknowngenus.id.2755	rs13336560	16	88554243	C	T	-0.0722586	0.01584847	-4.552669002	5.30E-06	7966
genus.unknowngenus.id.2755	rs74354280	4	134192918	C	T	-0.090331598	0.020547223	-4.447177252	8.70E-06	8457
genus.unknowngenus.id.2755	rs2074723	7	50834929	T	C	0.166301821	0.037575071	4.445875301	8.75E-06	6370
genus.unknowngenus.id.2755	rs1035406	5	119372737	G	A	-0.111240646	0.025155958	-4.435747788	9.18E-06	8439
genus.unknowngenus.id.2755	rs3730086	5	67577051	A	G	0.080674605	0.01800426	4.448486373	8.65E-06	8456
genus.unknowngenus.id.2755	rs4918686	10	113501109	G	T	0.069477215	0.015642376	4.445663051	8.76E-06	8457
genus.unknowngenus.id.826	rs55922055	2	3658278	T	C	-0.239699163	0.047604294	-5.175108004	2.28E-07	4010
genus.unknowngenus.id.826	rs740358	7	112149857	G	T	-0.130156684	0.026476273	-4.908419225	9.18E-07	11975

Gut	SNP	CHR	POS	A1	A2	BETA	SE	Z	P	N
genus.unknowngenus.id.826	rs11257188	10	6237156	A	C	-0.109509032	0.022583213	-4.769420117	1.85E-06	13282
genus.unknowngenus.id.826	rs72846345	2	134186590	T	C	0.087766079	0.019090256	4.701325167	2.58E-06	13281
genus.unknowngenus.id.826	rs66494181	2	174484907	T	G	0.068378899	0.014509844	4.713567012	2.43E-06	13128
genus.unknowngenus.id.826	rs6431586	2	234119635	A	G	-0.095415193	0.020571543	-4.690746132	2.72E-06	13205
genus.unknowngenus.id.826	rs10060821	5	83419996	T	C	0.062420757	0.014081774	4.439817671	9.00E-06	13275
genus.unknowngenus.id.826	rs2163025	2	5991969	T	C	0.05734007	0.012563673	4.57025839	4.87E-06	13282
genus.unknowngenus.id.826	rs142372196	13	84660104	A	G	-0.073620071	0.016090736	-4.547175655	5.44E-06	13200
genus.unknowngenus.id.826	rs9571849	13	68440424	T	C	0.137141259	0.03173764	4.462089447	8.12E-06	8407
genus.unknowngenus.id.826	rs7935794	11	62499044	C	G	-0.127423606	0.027406622	-4.664346874	3.10E-06	10767
genus.unknowngenus.id.826	rs599752	19	39539911	C	T	-0.063020708	0.013796438	-4.608537739	4.06E-06	13281
genus.unknowngenus.id.826	rs13147561	4	62227193	A	G	-0.099312671	0.022799364	-4.42670562	9.57E-06	12632
genus.unknowngenus.id.826	rs116151565	8	128668364	A	G	0.111567164	0.024565024	4.519297672	6.20E-06	13128
genus.unknowngenus.id.826	rs10782329	6	153780607	C	T	0.060279093	0.013755249	4.467906581	7.90E-06	12859
genus.unknowngenus.id.959	rs2199860	4	58800779	C	G	0.121445151	0.022290876	5.400521992	6.64E-08	4864
genus.unknowngenus.id.959	rs56210598	6	1605241	C	G	-0.111519964	0.022568865	-4.909723544	9.12E-07	4565
genus.unknowngenus.id.959	rs10892421	11	119498403	C	T	-0.103857922	0.021395334	-4.827023969	1.39E-06	4565
genus.unknowngenus.id.959	rs13186313	5	79470031	G	A	-0.117826679	0.024113575	-4.836480774	1.32E-06	4294
genus.unknowngenus.id.959	rs9534068	13	45802607	A	G	0.098738036	0.021131933	4.666269301	3.07E-06	4862
genus.unknowngenus.id.959	rs75285799	1	159508009	T	C	0.132213274	0.0275812	4.654482562	3.25E-06	4867
genus.unknowngenus.id.959	rs1969793	13	103561362	G	A	0.102670554	0.021893863	4.653917063	3.26E-06	4868
genus.unknowngenus.id.959	rs4766410	12	1765779	C	T	0.096930529	0.021223699	4.551017532	5.34E-06	4565
genus.unknowngenus.id.959	rs11691923	2	157246935	G	A	-0.092345339	0.02068831	-4.455935713	8.35E-06	4862
genus.unknowngenus.id.959	rs216336	12	6148230	A	G	-0.095527251	0.021425873	-4.466531802	7.95E-06	4868
genus.unknowngenus.id.959	rs115909738	15	61402485	A	C	-0.13462672	0.029776062	-4.50972024	6.49E-06	4866
genus.unknowngenus.id.959	rs2317372	8	57438932	C	T	-0.09464114	0.021078843	-4.467066626	7.93E-06	4565
genus.unknowngenus.id.959	rs66473610	7	22843553	T	G	0.181404571	0.040106074	4.444948132	8.79E-06	4059
genus.unknowngenus.id.959	rs12261792	10	11909896	T	G	-0.113723453	0.025624322	-4.426593146	9.57E-06	4868
genus.Veillonella.id.2198	rs2013594	11	44302154	T	C	-0.072069819	0.01551532	-4.6437574	3.42E-06	9283
genus.Veillonella.id.2198	rs12679709	8	13253770	C	G	-0.079349857	0.016481776	-4.777397112	1.78E-06	9290
genus.Veillonella.id.2198	rs742016	22	45604800	A	G	-0.068857084	0.01497691	-4.579678257	4.66E-06	9279
genus.Veillonella.id.2198	rs1882878	21	30010673	A	G	-0.07689658	0.016390829	-4.67213195	2.98E-06	9282

Gut	SNP	CHR	POS	A1	A2	BETA	SE	Z	P	N
genus.Veillonella.id.2198	rs7645873	3	125544032	A	T	0.076138533	0.016422757	4.662888218	3.12E-06	9286
genus.Veillonella.id.2198	rs55807413	11	2956783	A	G	0.107329355	0.023763343	4.544203163	5.51E-06	8610
genus.Veillonella.id.2198	rs62376424	5	119914285	C	T	-0.076221631	0.016349923	-4.630120386	3.65E-06	8999
genus.Veillonella.id.2198	rs7359080	14	106939916	C	A	-0.135390734	0.030351973	-4.481874535	7.40E-06	6189
genus.Veillonella.id.2198	rs11141494	9	89306305	G	A	-0.078342138	0.017439254	-4.422723933	9.75E-06	9291
genus.Veillonella.id.2198	rs6656807	1	178945043	A	G	0.070306243	0.015395397	4.544869058	5.50E-06	8999
genus.Veillonella.id.2198	rs11614532	12	129984555	G	C	0.074453782	0.016521422	4.489878564	7.13E-06	8411
genus.Victivallis.id.2256	rs56349194	11	9312796	A	G	-0.158501176	0.031523075	-4.983096338	6.26E-07	3266
genus.Victivallis.id.2256	rs592514	9	136309841	T	A	-0.181448867	0.039167751	-4.69983272	2.60E-06	3241
genus.Victivallis.id.2256	rs911666	14	96071125	T	C	-0.118575703	0.026314576	-4.474681763	7.65E-06	3267
genus.Victivallis.id.2256	rs7374366	3	60975863	G	A	-0.182273861	0.040503861	-4.48687719	7.23E-06	3235
genus.Victivallis.id.2256	rs4764863	12	102514184	G	A	0.121560633	0.024605824	4.930018055	8.22E-07	3266
genus.Victivallis.id.2256	rs12512543	4	9608445	A	C	-0.1780361	0.037434441	-4.704788011	2.54E-06	3239
genus.Victivallis.id.2256	rs342302	7	106378229	A	G	-0.152754045	0.035157217	-4.460956509	8.16E-06	3265
genus.Victivallis.id.2256	rs4895919	6	131630319	T	C	-0.116917513	0.024751549	-4.688511618	2.75E-06	3254
genus.Victivallis.id.2256	rs11899949	2	38049069	G	A	0.130567821	0.027623889	4.687391063	2.77E-06	3258
genus.Victivallis.id.2256	rs6445926	3	57723877	G	C	0.117030674	0.024976353	4.673697833	2.96E-06	3267
genus.Victivallis.id.2256	rs173120	13	72876881	T	C	0.133809734	0.029012714	4.474681588	7.65E-06	3263
genus.Victivallis.id.2256	rs1882775	21	40405606	A	G	-0.138270295	0.031272974	-4.446493422	8.73E-06	3258
order.Actinomycetales.id.420	rs35011108	6	133007480	A	G	0.24154595	0.050380314	4.766280292	1.88E-06	3630
order.Actinomycetales.id.420	rs2889192	9	76394568	G	T	0.088405157	0.019499679	4.613091674	3.97E-06	7711
order.Actinomycetales.id.420	rs34583783	6	67207371	G	T	0.123687922	0.026425408	4.543269368	5.54E-06	7711
order.Actinomycetales.id.420	rs4073240	6	169224781	G	A	0.074972982	0.016467174	4.538002875	5.68E-06	7711
order.Actinomycetales.id.420	rs58484246	15	29850991	T	C	0.075828017	0.016899684	4.503804135	6.67E-06	7711
order.Bacillales.id.1674	rs74887130	21	28684912	C	G	-0.192165775	0.035881712	-5.299502923	1.16E-07	3218
order.Bacillales.id.1674	rs4617108	7	49423832	A	G	0.248860918	0.052592896	4.755960024	1.98E-06	3018
order.Bacillales.id.1674	rs62640857	12	69332975	A	G	0.148237754	0.032645545	4.587186804	4.49E-06	3235
order.Bacillales.id.1674	rs1287018	20	5826474	G	A	0.141125041	0.031930898	4.419982093	9.87E-06	3207
order.Bacillales.id.1674	rs10410917	19	14970441	T	C	0.114710488	0.02500254	4.541955342	5.57E-06	3239
order.Bacillales.id.1674	rs74420793	4	127766709	A	G	-0.164413018	0.035376325	-4.665864101	3.07E-06	3077
order.Bacillales.id.1674	rs11034576	11	38085711	A	G	0.205764503	0.045331088	4.443280887	8.86E-06	3076

Gut	SNP	CHR	POS	A1	A2	BETA	SE	Z	P	N
order.Bacillales.id.1674	rs10233278	7	117496144	T	C	-0.116318665	0.024852733	-4.638584962	3.51E-06	3239
order.Bacillales.id.1674	rs11207728	1	61827534	G	A	-0.144550179	0.031691157	-4.53594645	5.73E-06	3239
order.Bacillales.id.1674	rs11844714	14	49001226	A	G	-0.143245189	0.032027153	-4.562196691	5.06E-06	3239
order.Bacteroidales.id.913	rs55773148	13	70523029	G	A	-0.121513597	0.023675903	-5.073977531	3.90E-07	11349
order.Bacteroidales.id.913	rs7631304	3	89339527	G	A	-0.064577053	0.013295739	-4.926587522	8.37E-07	18334
order.Bacteroidales.id.913	rs73975615	17	6461200	G	A	-0.207017892	0.044263343	-4.852986633	1.22E-06	3350
order.Bacteroidales.id.913	rs111845179	14	59317502	T	C	0.102647804	0.021384281	4.907172446	9.24E-07	16393
order.Bacteroidales.id.913	rs2032750	2	53831026	T	C	-0.050839276	0.010680731	-4.76218434	1.92E-06	17853
order.Bacteroidales.id.913	rs7546249	1	176792690	T	A	-0.056711256	0.01183859	-4.804919264	1.55E-06	18333
order.Bacteroidales.id.913	rs929878	16	74290641	C	T	-0.054853206	0.012152954	-4.576253529	4.73E-06	18334
order.Bacteroidales.id.913	rs13291169	9	116888284	C	G	0.068967579	0.014876501	4.625010725	3.75E-06	17513
order.Bacteroidales.id.913	rs17343978	22	27433885	A	C	-0.055205684	0.012027767	-4.455769606	8.36E-06	18334
order.Bacteroidales.id.913	rs72706335	1	157495438	T	C	-0.222409754	0.049345816	-4.474607184	7.66E-06	3819
order.Bacteroidales.id.913	rs11146701	10	39062269	A	G	0.047359582	0.010540996	4.491200742	7.08E-06	18220
order.Bacteroidales.id.913	rs62531359	8	70916181	T	G	0.065572807	0.014989058	4.437839192	9.09E-06	18320
order.Bacteroidales.id.913	rs4146051	2	213189994	A	G	-0.107377003	0.024566124	-4.445610503	8.76E-06	11372
order.Bacteroidales.id.913	rs79585701	8	13110185	A	C	0.064682288	0.01496282	4.417478809	9.99E-06	18334
order.Bacteroidales.id.913	rs4916508	3	195936789	G	A	-0.046672941	0.010531247	-4.452916809	8.47E-06	18324
order.Bacteroidales.id.913	rs62575403	9	136493820	C	T	0.140054866	0.031112824	4.491832787	7.06E-06	9668
order.Bifidobacteriales.id.432	rs687670	2	136740900	C	T	0.111068768	0.012910884	8.468270288	2.49E-17	14915
order.Bifidobacteriales.id.432	rs7581382	2	135775130	C	T	-0.090211798	0.01478989	-6.570874875	5.00E-11	13923
order.Bifidobacteriales.id.432	rs7322849	13	112859829	T	C	0.110675689	0.020099551	5.636013515	1.74E-08	14915
order.Bifidobacteriales.id.432	rs1961273	2	137602926	C	T	0.068812456	0.013169905	5.229758778	1.70E-07	14915
order.Bifidobacteriales.id.432	rs76671854	5	169748189	C	G	-0.089298109	0.018317684	-4.88461344	1.04E-06	13923
order.Bifidobacteriales.id.432	rs4957061	5	521096	T	C	0.056987252	0.011690731	4.863771462	1.15E-06	14795
order.Bifidobacteriales.id.432	rs10841473	12	20378911	G	C	-0.061303123	0.012884445	-4.726816492	2.28E-06	14914
order.Bifidobacteriales.id.432	rs4567981	2	49906864	T	A	0.057776313	0.011738841	4.914949465	8.88E-07	14795
order.Bifidobacteriales.id.432	rs677208	18	4304187	G	C	0.072858928	0.016482072	4.526350411	6.00E-06	14915
order.Bifidobacteriales.id.432	rs12446429	16	898055	T	C	0.081009916	0.019073029	4.451459465	8.53E-06	14008
order.Bifidobacteriales.id.432	rs11655079	17	75348675	T	C	-0.058614646	0.012995163	-4.529059711	5.92E-06	14912
order.Bifidobacteriales.id.432	rs540489	17	72897722	T	G	-0.06325184	0.013816951	-4.549861933	5.37E-06	14688

Gut	SNP	CHR	POS	A1	A2	BETA	SE	Z	P	N
order.Bifidobacteriales.id.432	rs857444	6	14617591	C	T	0.05539651	0.012066937	4.620676066	3.82E-06	14914
order.Bifidobacteriales.id.432	rs10857328	4	161211924	T	A	0.056200033	0.012639517	4.48646831	7.24E-06	14915
order.Bifidobacteriales.id.432	rs10831953	11	13098051	G	A	0.053753749	0.012374596	4.418313309	9.95E-06	14915
order.Bifidobacteriales.id.432	rs7174549	15	92463303	C	T	0.055172631	0.01246547	4.497744987	6.87E-06	14915
order.Bifidobacteriales.id.432	rs73797465	5	142793467	T	G	-0.094261173	0.020842122	-4.571195472	4.85E-06	14795
order.Bifidobacteriales.id.432	rs55888705	4	1517826	A	G	0.053672952	0.012058615	4.448130649	8.66E-06	14914
order.Bifidobacteriales.id.432	rs6899771	6	97406220	A	G	-0.09143118	0.020260353	-4.485334979	7.28E-06	14150
order.Bifidobacteriales.id.432	rs7588568	2	189805077	A	C	-0.060715954	0.013060506	-4.625862767	3.73E-06	14913
order.Burkholderiales.id.2874	rs62191117	2	239900775	A	G	0.067999488	0.013247059	5.137040407	2.79E-07	16975
order.Burkholderiales.id.2874	rs6087811	20	30596130	T	G	-0.101586745	0.019881673	-5.13089061	2.88E-07	16135
order.Burkholderiales.id.2874	rs4033856	4	45642485	C	T	0.083344552	0.016748909	5.002191432	5.67E-07	15897
order.Burkholderiales.id.2874	rs2367850	3	193456274	G	C	0.063330043	0.013059522	4.849754915	1.24E-06	16664
order.Burkholderiales.id.2874	rs62395635	5	173497796	T	C	0.109911047	0.023637504	4.677616854	2.90E-06	15062
order.Burkholderiales.id.2874	rs2321387	13	58689340	G	A	-0.050851372	0.010958763	-4.653854616	3.26E-06	17095
order.Burkholderiales.id.2874	rs7638039	3	70588939	T	C	0.058098748	0.012673046	4.571782401	4.84E-06	17078
order.Burkholderiales.id.2874	rs2613606	7	111285025	C	T	-0.049956147	0.010926388	-4.604550922	4.13E-06	17092
order.Burkholderiales.id.2874	rs72747231	9	84206018	C	G	-0.133728739	0.029073834	-4.624106066	3.76E-06	11315
order.Burkholderiales.id.2874	rs1928341	1	153240013	A	G	0.050778221	0.011058697	4.58614354	4.52E-06	17096
order.Burkholderiales.id.2874	rs9964679	18	26569901	A	G	0.052509048	0.011503961	4.524291814	6.06E-06	17097
order.Burkholderiales.id.2874	rs1511453	4	23588462	A	G	0.09109992	0.01994793	4.465157933	8.00E-06	17032
order.Burkholderiales.id.2874	rs75242906	15	56786407	C	T	-0.120974344	0.028127277	-4.422751288	9.75E-06	10766
order.Clostridiales.id.1863	rs6815608	4	152131744	T	C	0.104089026	0.021088307	5.082760526	3.72E-07	16731
order.Clostridiales.id.1863	rs112334273	21	40703251	G	A	0.063938344	0.012740449	5.06553383	4.07E-07	18217
order.Clostridiales.id.1863	rs6814436	4	161507301	T	C	0.074185762	0.015081384	4.910966043	9.06E-07	18336
order.Clostridiales.id.1863	rs72915163	18	48792829	T	C	-0.058024623	0.012055772	-4.82628376	1.39E-06	18317
order.Clostridiales.id.1863	rs10774377	12	5942519	A	G	0.052300318	0.011385929	4.621541485	3.81E-06	18330
order.Clostridiales.id.1863	rs13179700	5	149077788	T	C	0.051095292	0.010956887	4.637815488	3.52E-06	18338
order.Clostridiales.id.1863	rs6934062	6	163743050	G	C	0.05223974	0.011611578	4.490823658	7.09E-06	17859
order.Clostridiales.id.1863	rs10209007	2	231676041	G	C	0.079542419	0.018242704	4.42609206	9.60E-06	17812
order.Clostridiales.id.1863	rs1842454	5	105060362	G	A	-0.054367588	0.012734002	-4.418965681	9.92E-06	18340
order.Clostridiales.id.1863	rs76767978	3	131371980	G	A	0.069980605	0.015631841	4.460532862	8.18E-06	18329

Gut	SNP	CHR	POS	A1	A2	BETA	SE	Z	P	N
order.Clostridiales.id.1863	rs13105690	4	7420184	T	C	-0.052744064	0.011818018	-4.43126785	9.37E-06	18340
order.Clostridiales.id.1863	rs7269302	20	50803760	A	T	-0.096534637	0.02120063	-4.443676595	8.84E-06	17405
order.Clostridiales.id.1863	rs72738886	5	35770550	T	C	0.086533337	0.019039215	4.454324608	8.42E-06	16934
order.Clostridiales.id.1863	rs76860606	6	12397187	C	G	0.094122019	0.022511723	4.450134048	8.58E-06	17579
order.Clostridiales.id.1863	rs290772	2	102126220	G	A	0.084254009	0.01962973	4.417225503	1.00E-05	17129
order.Clostridiales.id.1863	rs6442336	3	12870886	C	T	-0.054772905	0.012416948	-4.425415623	9.63E-06	18333
order.Coriobacteriales.id.810	rs719099	10	65799217	A	G	0.077840173	0.01558151	5.010348118	5.43E-07	17687
order.Coriobacteriales.id.810	rs240104	1	176571431	T	C	-0.060342897	0.012684911	-4.809111417	1.52E-06	17577
order.Coriobacteriales.id.810	rs11250875	10	1922731	T	C	0.060749978	0.013093887	4.572106901	4.83E-06	17690
order.Coriobacteriales.id.810	rs34739816	17	37376685	G	T	0.096501674	0.020766582	4.617517509	3.88E-06	16593
order.Coriobacteriales.id.810	rs76779974	5	125460084	C	G	0.0774888	0.017187698	4.539794605	5.63E-06	16419
order.Coriobacteriales.id.810	rs80046645	5	459415	C	G	0.255839805	0.056199532	4.579679319	4.66E-06	3204
order.Coriobacteriales.id.810	rs45480394	19	55857777	T	G	-0.050426168	0.011330684	-4.423433012	9.71E-06	17413
order.Coriobacteriales.id.810	rs1816223	12	11494021	A	G	-0.058626474	0.012900576	-4.571556654	4.84E-06	17688
order.Coriobacteriales.id.810	rs7898585	10	5644995	T	C	0.073150094	0.01641856	4.554673113	5.25E-06	17691
order.Coriobacteriales.id.810	rs67561917	20	62072077	A	G	-0.071436723	0.015411343	-4.548955316	5.39E-06	17333
order.Coriobacteriales.id.810	rs3025411	9	136512906	A	G	0.092654285	0.020946781	4.458095088	8.27E-06	15671
order.Coriobacteriales.id.810	rs11656361	17	8121332	A	C	0.077300587	0.01755297	4.464644853	8.02E-06	16665
order.Coriobacteriales.id.810	rs1397793	5	90471451	G	A	-0.049885455	0.011244635	-4.422117946	9.77E-06	17527
order.Coriobacteriales.id.810	rs11073596	15	86433579	T	G	0.051021228	0.011433922	4.46135018	8.14E-06	17527
order.Coriobacteriales.id.810	rs2442778	3	11654412	G	A	-0.116408329	0.025854513	-4.439256238	9.03E-06	13472
order.Coriobacteriales.id.810	rs7540303	1	179629191	C	T	0.048322746	0.010906179	4.425509713	9.62E-06	17691
order.Coriobacteriales.id.810	rs62448869	7	24464799	T	A	-0.048605943	0.010898242	-4.468615174	7.87E-06	17527
order.Coriobacteriales.id.810	rs13307134	7	105084680	C	T	0.056568602	0.012626474	4.470620179	7.80E-06	17527
order.Coriobacteriales.id.810	rs4880783	10	1223742	G	T	0.056502976	0.012505544	4.530118208	5.90E-06	17691
order.Coriobacteriales.id.810	rs12974142	19	52895166	G	A	0.078979608	0.017720479	4.451876291	8.51E-06	17413
order.Coriobacteriales.id.810	rs8010111	14	39660509	G	A	-0.10337688	0.022928631	-4.496866139	6.90E-06	15996
order.Desulfovibrionales.id.3156	rs6058181	20	33694801	C	T	0.083676458	0.016609784	5.155637171	2.53E-07	15076
order.Desulfovibrionales.id.3156	rs4506934	17	2856662	C	T	-0.095338347	0.020136702	-4.713918347	2.43E-06	15037
order.Desulfovibrionales.id.3156	rs11599763	10	11855599	T	C	-0.055486167	0.011748632	-4.699579321	2.61E-06	15144
order.Desulfovibrionales.id.3156	rs2838334	21	45064961	G	A	0.056902402	0.01242233	4.602828689	4.17E-06	15144

Gut	SNP	CHR	POS	A1	A2	BETA	SE	Z	P	N
order.Desulfovibrionales.id.3156	rs9928243	16	71541641	C	A	-0.054471273	0.011781078	-4.613137587	3.97E-06	15143
order.Desulfovibrionales.id.3156	rs112381107	9	15269705	C	T	0.210090212	0.045697378	4.65648539	3.22E-06	4455
order.Desulfovibrionales.id.3156	rs16851319	1	203308383	G	C	-0.072913728	0.015095443	-4.692196778	2.70E-06	15130
order.Desulfovibrionales.id.3156	rs17791387	9	81834426	A	G	-0.072750113	0.015436947	-4.729301257	2.25E-06	15047
order.Desulfovibrionales.id.3156	rs186073	3	31133401	T	C	0.052921896	0.011854172	4.446100055	8.74E-06	14841
order.Desulfovibrionales.id.3156	rs2692012	1	203991605	A	G	0.112244115	0.025355338	4.72774997	2.27E-06	13082
order.Desulfovibrionales.id.3156	rs62020470	15	96161065	A	G	-0.057363273	0.012946185	-4.478702505	7.51E-06	15144
order.Desulfovibrionales.id.3156	rs72647048	8	57865378	T	C	-0.077245856	0.017087645	-4.439831342	9.00E-06	15144
order.Enterobacteriales.id.3468	rs78143293	18	57672335	A	G	-0.084852127	0.017041521	-4.856359027	1.20E-06	13572
order.Enterobacteriales.id.3468	rs111229068	11	2563450	A	T	0.110560883	0.024171888	4.630175311	3.65E-06	12350
order.Enterobacteriales.id.3468	rs62210023	20	55340092	A	G	0.060675357	0.013012497	4.661992323	3.13E-06	13515
order.Enterobacteriales.id.3468	rs504442	18	55145547	T	G	0.084159032	0.01894789	4.557731073	5.17E-06	13578
order.Enterobacteriales.id.3468	rs80319214	2	4015933	C	G	0.099040406	0.021587746	4.495120205	6.95E-06	12641
order.Enterobacteriales.id.3468	rs11026530	11	22379097	T	C	0.082240818	0.018637912	4.429919283	9.43E-06	13485
order.Enterobacteriales.id.3468	rs2374342	2	42133542	C	A	0.058292653	0.01261946	4.586092616	4.52E-06	13577
order.Enterobacteriales.id.3468	rs79757635	13	110840418	C	A	0.075859672	0.017128562	4.43240967	9.32E-06	13419
order.Enterobacteriales.id.3468	rs35673018	16	54327745	G	A	0.08996415	0.020293422	4.47528099	7.63E-06	13326
order.Enterobacteriales.id.3468	rs61973590	14	27718039	C	G	-0.060539207	0.013369618	-4.451294856	8.54E-06	13570
order.Enterobacteriales.id.3468	rs4792380	17	13521040	A	T	0.115597849	0.025768301	4.428484728	9.49E-06	12763
order.Erysipelotrichales.id.2148	rs62504403	8	38803551	C	T	0.068115892	0.012788339	5.305661665	1.12E-07	16981
order.Erysipelotrichales.id.2148	rs7234058	18	5830507	T	C	-0.094575892	0.019409083	-4.909660622	9.12E-07	16874
order.Erysipelotrichales.id.2148	rs17530232	13	40385457	A	G	0.103049982	0.022464754	4.685613075	2.79E-06	16622
order.Erysipelotrichales.id.2148	rs35161940	17	70327224	T	C	-0.080604056	0.01676409	-4.769690104	1.85E-06	18213
order.Erysipelotrichales.id.2148	rs10781552	10	133897233	C	T	-0.055221236	0.011607425	-4.722368313	2.33E-06	17789
order.Erysipelotrichales.id.2148	rs8003149	14	56156504	C	T	0.053879756	0.01168879	4.60702288	4.08E-06	18213
order.Erysipelotrichales.id.2148	rs4078432	14	48997206	C	T	-0.060892934	0.013376363	-4.599698061	4.23E-06	17789
order.Erysipelotrichales.id.2148	rs1074800	5	3002546	A	G	0.049243827	0.010887044	4.521309241	6.15E-06	17789
order.Erysipelotrichales.id.2148	rs56970041	14	80357610	T	G	0.072414447	0.016447064	4.548481456	5.40E-06	18217
order.Erysipelotrichales.id.2148	rs7826267	8	2954952	T	G	-0.083907147	0.019913089	-4.433199344	9.28E-06	17586
order.Erysipelotrichales.id.2148	rs1884466	1	64139196	C	T	-0.047536345	0.01069386	-4.427639255	9.53E-06	18205
order.Erysipelotrichales.id.2148	rs290833	1	97457427	T	G	-0.049737371	0.011137647	-4.464409174	8.03E-06	17789

Gut	SNP	CHR	POS	A1	A2	BETA	SE	Z	P	N
order.Gastranaerophilales.id.1591	rs9864379	3	14306949	T	C	-0.160521381	0.029275217	-5.463866794	4.66E-08	5768
order.Gastranaerophilales.id.1591	rs11150282	16	80493705	T	C	0.098234998	0.019712655	4.951601318	7.36E-07	5864
order.Gastranaerophilales.id.1591	rs16851659	1	230523039	G	C	-0.089516816	0.01865552	-4.844354276	1.27E-06	5875
order.Gastranaerophilales.id.1591	rs4129395	9	115975389	G	A	0.090426365	0.01852532	4.852780703	1.22E-06	5878
order.Gastranaerophilales.id.1591	rs1221147	9	122046549	T	A	0.126305018	0.027916132	4.570759409	4.86E-06	5759
order.Gastranaerophilales.id.1591	rs79790072	15	100747683	T	C	0.226102517	0.048801659	4.636820109	3.54E-06	4104
order.Gastranaerophilales.id.1591	rs73074665	7	20010624	A	T	0.164631221	0.035603665	4.631868601	3.62E-06	5749
order.Gastranaerophilales.id.1591	rs113884518	9	24648997	T	C	-0.205909694	0.045537561	-4.472255993	7.74E-06	4434
order.Gastranaerophilales.id.1591	rs789069	18	1008278	A	C	-0.104129854	0.023445913	-4.509435534	6.50E-06	5870
order.Gastranaerophilales.id.1591	rs367480	11	2937631	G	A	-0.084164921	0.018594792	-4.478294302	7.52E-06	5878
order.Gastranaerophilales.id.1591	rs8028558	15	62055753	A	G	0.083459982	0.018850404	4.421929013	9.78E-06	5875
order.Gastranaerophilales.id.1591	rs28678345	17	53906328	T	C	0.213082993	0.047137686	4.463596364	8.06E-06	4315
order.Lactobacillales.id.1800	rs2952251	8	10143164	A	G	-0.06293169	0.012417747	-5.10178713	3.36E-07	17278
order.Lactobacillales.id.1800	rs35344081	16	991253	G	A	0.064244209	0.012735835	5.061456412	4.16E-07	17292
order.Lactobacillales.id.1800	rs76717940	3	94153993	T	A	0.160942577	0.032699971	4.974160051	6.55E-07	7976
order.Lactobacillales.id.1800	rs77558518	5	174173171	A	G	-0.106465937	0.022348355	-4.789215285	1.67E-06	15758
order.Lactobacillales.id.1800	rs11110282	12	100585559	A	G	-0.102389846	0.021784941	-4.613646703	3.96E-06	15956
order.Lactobacillales.id.1800	rs9581006	13	24973509	C	T	0.225829511	0.046922805	4.778513509	1.77E-06	4224
order.Lactobacillales.id.1800	rs4028634	17	40835649	T	C	0.053291462	0.011009978	4.832533791	1.35E-06	17295
order.Lactobacillales.id.1800	rs74352383	6	67570929	T	A	-0.103396459	0.021757973	-4.765883527	1.88E-06	16442
order.Lactobacillales.id.1800	rs78938557	7	36349586	T	C	0.105530209	0.023358105	4.724143542	2.31E-06	15152
order.Lactobacillales.id.1800	rs57872228	1	200418805	C	T	-0.068841481	0.014699394	-4.70150454	2.58E-06	17293
order.Lactobacillales.id.1800	rs11730038	4	98049499	G	A	-0.060538395	0.01290523	-4.560735978	5.10E-06	17295
order.Lactobacillales.id.1800	rs2370083	14	97526750	G	T	-0.080539958	0.018099185	-4.456394727	8.33E-06	17183
order.Lactobacillales.id.1800	rs12797734	11	8332350	T	C	0.057127947	0.012711299	4.471556521	7.77E-06	16820
order.Lactobacillales.id.1800	rs34989881	19	51959855	A	G	0.113478844	0.024645168	4.606575676	4.09E-06	12921
order.Lactobacillales.id.1800	rs11627423	14	33200623	C	A	0.049926314	0.010974412	4.561145553	5.09E-06	17275
order.Lactobacillales.id.1800	rs111552159	4	184681021	C	G	0.128854393	0.028739711	4.457088379	8.31E-06	11312
order.Lactobacillales.id.1800	rs74663707	3	184371224	C	T	0.09825937	0.022461255	4.454839929	8.40E-06	15695
order.Methanobacteriales.id.120	rs73457410	13	41956181	A	G	0.215339755	0.04366952	4.823790925	1.41E-06	3498
order.Methanobacteriales.id.120	rs6776814	3	15053083	T	C	-0.199565931	0.04118249	-4.79452384	1.63E-06	3385

Gut	SNP	CHR	POS	A1	A2	BETA	SE	Z	P	N
order.Methanobacteriales.id.120	rs894996	4	104418307	C	A	0.216999069	0.044907554	4.766388791	1.88E-06	3582
order.Methanobacteriales.id.120	rs75208022	12	21338861	C	T	-0.227244713	0.048763001	-4.529189927	5.92E-06	3203
order.Methanobacteriales.id.120	rs12825290	12	43701132	C	G	-0.216781075	0.049349641	-4.523738926	6.08E-06	3144
order.Methanobacteriales.id.120	rs56131665	5	178160975	G	A	0.178661188	0.039297982	4.520138571	6.18E-06	3572
order.Methanobacteriales.id.120	rs11018665	11	89349287	A	T	0.111465287	0.025051554	4.508829011	6.52E-06	3698
order.Methanobacteriales.id.120	rs4257531	3	2086167	G	A	0.164498365	0.03649593	4.480597376	7.44E-06	3162
order.Methanobacteriales.id.120	rs6508769	19	28827760	T	C	0.15351913	0.034451866	4.459234474	8.23E-06	3319
order.Methanobacteriales.id.120	rs73068003	7	10773932	G	T	-0.158140381	0.035180317	-4.453559383	8.45E-06	3692
order.Methanobacteriales.id.120	rs10424197	19	46439321	G	A	-0.111274175	0.024751678	-4.433329849	9.28E-06	3698
order.MollicutesRF9.id.11579	rs74603314	14	46519718	T	C	0.230767094	0.048970099	4.727196832	2.28E-06	4033
order.MollicutesRF9.id.11579	rs76373661	3	3771148	G	A	0.090821805	0.020298596	4.558199311	5.16E-06	10359
order.MollicutesRF9.id.11579	rs17235252	7	82536037	T	C	-0.12200211	0.02554629	-4.737961619	2.16E-06	9531
order.MollicutesRF9.id.11579	rs638542	1	109845252	G	A	-0.070591966	0.015704899	-4.557652462	5.17E-06	9970
order.MollicutesRF9.id.11579	rs62188991	2	177374176	G	C	-0.110864082	0.02413552	-4.553860627	5.27E-06	9531
order.MollicutesRF9.id.11579	rs949341	11	112386785	G	A	0.065660222	0.014721644	4.472450651	7.73E-06	10355
order.MollicutesRF9.id.11579	rs13100746	3	165846506	C	T	0.063897883	0.014268538	4.485085885	7.29E-06	10359
order.MollicutesRF9.id.11579	rs515984	10	3521459	T	C	-0.087545066	0.019068514	-4.505757535	6.61E-06	10359
order.MollicutesRF9.id.11579	rs7706512	5	17443654	G	A	0.065735114	0.013897929	4.727583749	2.27E-06	10359
order.MollicutesRF9.id.11579	rs11779863	8	15406830	G	A	-0.077276806	0.017217056	-4.503369443	6.69E-06	10353
order.MollicutesRF9.id.11579	rs7801843	7	4105705	A	G	-0.086949468	0.01945094	-4.428960862	9.47E-06	10359
order.MollicutesRF9.id.11579	rs10071529	5	176287195	G	C	0.125104505	0.027867407	4.448578118	8.64E-06	10147
order.MollicutesRF9.id.11579	rs739151	22	42741466	C	G	0.065265852	0.013977274	4.664554003	3.09E-06	10337
order.MollicutesRF9.id.11579	rs114184530	9	116018011	T	C	-0.1545446	0.033999183	-4.468553952	7.88E-06	7196
order.MollicutesRF9.id.11579	rs12566890	1	61850864	T	G	-0.10313363	0.024177477	-4.462206342	8.11E-06	10339
order.MollicutesRF9.id.11579	rs3932485	3	184193482	C	T	0.062620895	0.014162324	4.418723133	9.93E-06	10359
order.MollicutesRF9.id.11579	rs7853673	9	116711241	G	A	-0.062438944	0.013983251	-4.501974774	6.73E-06	10359
order.NB1n.id.3953	rs60583455	7	1321675	T	C	0.108933641	0.021176966	5.150655336	2.60E-07	4773
order.NB1n.id.3953	rs2172426	8	18229020	C	T	-0.102119829	0.019894658	-5.112795913	3.17E-07	5099
order.NB1n.id.3953	rs11251024	10	2095726	G	A	0.104201088	0.020673705	4.971957983	6.63E-07	5107
order.NB1n.id.3953	rs267959	5	10737802	A	G	0.098861186	0.020967556	4.698567115	2.62E-06	5107
order.NB1n.id.3953	rs11606187	11	91589600	A	G	-0.154539114	0.032628781	-4.65084005	3.31E-06	5102

Gut	SNP	CHR	POS	A1	A2	BETA	SE	Z	P	N
order.NB1n.id.3953	rs7911787	10	98619568	G	T	-0.223070508	0.047023233	-4.645761783	3.39E-06	3879
order.NB1n.id.3953	rs72671304	14	39425971	T	C	0.172321792	0.037002808	4.622262246	3.80E-06	4887
order.NB1n.id.3953	rs13385922	2	234081324	T	C	0.092949422	0.020134823	4.613036483	3.97E-06	5106
order.NB1n.id.3953	rs55921101	10	127094261	A	T	-0.109245446	0.023620072	-4.595294284	4.32E-06	5105
order.NB1n.id.3953	rs9542068	13	70306831	T	C	0.099070907	0.021814934	4.508674582	6.52E-06	5107
order.NB1n.id.3953	rs2930903	19	887824	C	G	0.090833483	0.020943373	4.49849862	6.84E-06	4789
order.NB1n.id.3953	rs60775321	15	97318671	T	C	-0.096242537	0.021384172	-4.49053038	7.10E-06	5101
order.NB1n.id.3953	rs8126061	20	3405239	T	C	-0.159135032	0.035190375	-4.482905922	7.36E-06	5089
order.NB1n.id.3953	rs166849	2	200778866	G	A	0.09110531	0.020223942	4.472280499	7.74E-06	5107
order.Pasteurellales.id.3688	rs12191680	6	129224488	C	G	0.102213615	0.019718945	5.120392596	3.05E-07	9408
order.Pasteurellales.id.3688	rs9382510	6	55448491	C	T	-0.088186524	0.016996482	-5.159507554	2.48E-07	9410
order.Pasteurellales.id.3688	rs10840326	11	9951672	C	G	-0.0717634	0.01492293	-4.819605688	1.44E-06	9154
order.Pasteurellales.id.3688	rs76022354	10	94306385	C	T	0.242889667	0.050040603	4.771501022	1.83E-06	3692
order.Pasteurellales.id.3688	rs72756943	5	26531908	G	A	0.139879974	0.030302876	4.647970039	3.35E-06	8020
order.Pasteurellales.id.3688	rs78909003	9	105650242	T	C	-0.241159469	0.049837229	-4.748537868	2.05E-06	3638
order.Pasteurellales.id.3688	rs6092684	20	57274077	A	T	0.068464719	0.014673987	4.642595614	3.44E-06	9366
order.Pasteurellales.id.3688	rs4822728	22	26891808	T	C	0.068547871	0.014903232	4.576930843	4.72E-06	9412
order.Pasteurellales.id.3688	rs35510	12	115491973	A	G	0.122729003	0.026495751	4.610565017	4.02E-06	8671
order.Pasteurellales.id.3688	rs10965428	9	22718481	C	A	-0.119902083	0.025821904	-4.596740955	4.29E-06	8873
order.Pasteurellales.id.3688	rs16970009	17	32862601	A	G	0.187381473	0.042957182	4.48430178	7.32E-06	4703
order.Pasteurellales.id.3688	rs12050685	15	73477482	A	G	-0.067094787	0.015238983	-4.435517722	9.19E-06	9411
order.Pasteurellales.id.3688	rs73139353	3	97972214	A	C	-0.222572658	0.048463428	-4.44686776	8.71E-06	4047
order.Pasteurellales.id.3688	rs6972479	7	116918060	A	G	-0.078186189	0.01753656	-4.472026835	7.75E-06	8616
order.Pasteurellales.id.3688	rs9895850	17	64535013	T	C	-0.176406461	0.041016971	-4.43793724	9.08E-06	4737
order.Pasteurellales.id.3688	rs111582866	16	48742489	G	A	-0.113957275	0.025640298	-4.4916826	7.07E-06	8616
order.Pasteurellales.id.3688	rs62568866	9	6818307	T	A	-0.118018253	0.025920615	-4.486902005	7.23E-06	8020
order.Pasteurellales.id.3688	rs9938097	16	84977389	T	C	-0.071038642	0.015802434	-4.459215025	8.23E-06	9402
order.Rhodospirillales.id.2667	rs9813022	3	13726736	A	G	-0.083079452	0.016264151	-5.118967278	3.07E-07	8600
order.Rhodospirillales.id.2667	rs11630875	15	61775734	T	C	0.094519847	0.02021388	4.627347619	3.70E-06	8595
order.Rhodospirillales.id.2667	rs76784716	2	169033340	A	G	0.136039348	0.02843993	4.837651429	1.31E-06	8600
order.Rhodospirillales.id.2667	rs3754624	2	225633812	C	T	0.094128044	0.01983893	4.693654436	2.68E-06	8598

Gut	SNP	CHR	POS	A1	A2	BETA	SE	Z	P	N
order.Rhodospirillales.id.2667	rs1549633	5	27945645	A	C	0.099673509	0.02166668	4.617696163	3.88E-06	8112
order.Rhodospirillales.id.2667	rs7001029	8	131958403	C	T	0.120725521	0.026098899	4.682736289	2.83E-06	8063
order.Rhodospirillales.id.2667	rs1035406	5	119372737	G	A	-0.114724899	0.024799361	-4.607691949	4.07E-06	8585
order.Rhodospirillales.id.2667	rs4278423	10	2670553	T	C	0.105215078	0.023377775	4.612403305	3.98E-06	8603
order.Rhodospirillales.id.2667	rs61933850	12	73139398	G	A	0.164662572	0.036028532	4.49372618	7.00E-06	6846
order.Rhodospirillales.id.2667	rs77304857	4	134248015	C	A	-0.099743255	0.02221655	-4.525674656	6.02E-06	8602
order.Rhodospirillales.id.2667	rs55876211	3	84471266	C	T	-0.086500238	0.019519668	-4.468605054	7.87E-06	8596
order.Rhodospirillales.id.2667	rs13336560	16	88554243	C	T	-0.069531323	0.0157065	-4.422714288	9.75E-06	8112
order.Rhodospirillales.id.2667	rs3730086	5	67577051	A	G	0.08003537	0.017873721	4.465652241	7.98E-06	8602
order.Rhodospirillales.id.2667	rs4918686	10	113501109	G	T	0.069967294	0.015536322	4.480867825	7.43E-06	8603
order.Selenomonadales.id.2165	rs1643968	5	165266628	T	C	-0.056528439	0.011229806	-5.062130941	4.15E-07	18170
order.Selenomonadales.id.2165	rs60274479	16	21249925	T	C	-0.065962286	0.013413689	-4.861958016	1.16E-06	17610
order.Selenomonadales.id.2165	rs13086907	3	142135117	G	A	0.062531199	0.013173503	4.758034029	1.95E-06	18163
order.Selenomonadales.id.2165	rs73232831	4	17413426	G	A	-0.151828859	0.03149318	-4.766501422	1.87E-06	9382
order.Selenomonadales.id.2165	rs71405394	15	101245088	G	A	-0.114177822	0.02404996	-4.737256816	2.17E-06	13861
order.Selenomonadales.id.2165	rs61249479	9	124912908	A	C	0.077707533	0.01686282	4.673943117	2.95E-06	18165
order.Selenomonadales.id.2165	rs1447205	8	41231597	G	C	-0.050205329	0.010838203	-4.67544014	2.93E-06	18165
order.Selenomonadales.id.2165	rs4463806	10	115597993	T	C	-0.05439664	0.012936525	-4.47041991	7.81E-06	18160
order.Selenomonadales.id.2165	rs9423647	10	5579818	G	A	0.047817029	0.010528201	4.524245342	6.06E-06	18164
order.Selenomonadales.id.2165	rs2834062	21	34377485	A	G	0.0489057	0.010884164	4.453728035	8.44E-06	18171
order.Selenomonadales.id.2165	rs1649999	10	80086072	A	G	0.074898861	0.01664577	4.476682001	7.58E-06	18036
order.Selenomonadales.id.2165	rs1135612	7	75609677	G	A	0.052978507	0.01191767	4.433697196	9.26E-06	18164
order.Verrucomicrobiales.id.4030	rs11729256	4	95027272	T	C	0.074979802	0.015017677	4.968971477	6.73E-07	11861
order.Verrucomicrobiales.id.4030	rs9349825	6	56341481	A	G	-0.070403566	0.014712789	-4.705144056	2.54E-06	11858
order.Verrucomicrobiales.id.4030	rs74542928	4	100544188	T	C	0.112163707	0.023642195	4.794327123	1.63E-06	11216
order.Verrucomicrobiales.id.4030	rs3995795	10	11389614	C	T	0.064127531	0.014099468	4.557136659	5.19E-06	11690
order.Verrucomicrobiales.id.4030	rs2602429	16	81063149	C	T	0.074684437	0.015619438	4.701468957	2.58E-06	11690
order.Verrucomicrobiales.id.4030	rs4242783	10	5064327	G	A	0.068929219	0.014769297	4.697308453	2.64E-06	11690
order.Verrucomicrobiales.id.4030	rs11184341	1	105422565	G	C	0.065534193	0.014223078	4.604526701	4.13E-06	11864
order.Verrucomicrobiales.id.4030	rs61779207	1	41074472	G	A	-0.075830853	0.016776157	-4.502279537	6.72E-06	11591
order.Verrucomicrobiales.id.4030	rs117107102	18	49473635	A	G	0.20468288	0.043157251	4.676493858	2.92E-06	4975

Gut	SNP	CHR	POS	A1	A2	BETA	SE	Z	P	N
order.Verrucomicrobiales.id.4030	rs12908520	15	97570657	G	A	0.061892558	0.013094626	4.736758778	2.17E-06	11863
order.Verrucomicrobiales.id.4030	rs111862613	12	130309670	T	C	0.090698957	0.019674596	4.62541648	3.74E-06	11621
order.Verrucomicrobiales.id.4030	rs941682	20	31867840	G	A	-0.063143598	0.014376741	-4.425766881	9.61E-06	11809
order.Victivallales.id.2254	rs2825714	21	21023966	A	G	-0.137410136	0.02892458	-4.783567792	1.72E-06	4734
order.Victivallales.id.2254	rs17114848	15	25162535	G	A	0.152376929	0.032433167	4.60835594	4.06E-06	4734
order.Victivallales.id.2254	rs77599476	20	61394262	A	G	0.230291775	0.048016811	4.767925577	1.86E-06	3722
order.Victivallales.id.2254	rs72640280	1	11943792	A	G	0.220206945	0.048619648	4.557348316	5.18E-06	3816
order.Victivallales.id.2254	rs62570196	9	111086170	C	T	-0.216350467	0.043986577	-4.876607762	1.08E-06	3712
order.Victivallales.id.2254	rs11764871	7	146808977	G	T	0.105796117	0.023346418	4.513221575	6.39E-06	4725
order.Victivallales.id.2254	rs2731834	5	94720245	C	G	0.109437835	0.023692997	4.599081581	4.24E-06	4733
order.Victivallales.id.2254	rs2031282	13	20687179	A	G	0.122368503	0.027032933	4.59236071	4.38E-06	4734
order.Victivallales.id.2254	rs1002941	15	101242690	G	A	0.105025041	0.023348442	4.461248306	8.15E-06	4734
order.Victivallales.id.2254	rs73113483	3	6223568	T	A	-0.131216985	0.0288713	-4.448097775	8.66E-06	4718
phylum.Actinobacteria.id.400	rs687670	2	136740900	C	T	0.082866806	0.011703132	6.976786933	3.02E-12	17990
phylum.Actinobacteria.id.400	rs4429415	2	213756191	C	T	0.058190752	0.011134335	5.194612825	2.05E-07	17990
phylum.Actinobacteria.id.400	rs10841473	12	20378911	G	C	-0.059657241	0.011753696	-5.047949659	4.47E-07	17987
phylum.Actinobacteria.id.400	rs10193348	2	135829432	G	A	0.073627905	0.014986276	5.09943442	3.41E-07	17990
phylum.Actinobacteria.id.400	rs74037001	14	23847194	G	A	-0.081939134	0.016533368	-4.96950523	6.71E-07	17179
phylum.Actinobacteria.id.400	rs55888705	4	1517826	A	G	0.053422107	0.010997529	4.837863297	1.31E-06	17988
phylum.Actinobacteria.id.400	rs62448869	7	24464799	T	A	-0.052453124	0.010822585	-4.865969948	1.14E-06	17782
phylum.Actinobacteria.id.400	rs8047955	16	81776768	A	G	0.052111493	0.011119219	4.69531004	2.66E-06	17980
phylum.Actinobacteria.id.400	rs34284163	16	89248299	A	T	-0.060331227	0.013685143	-4.444219958	8.82E-06	17987
phylum.Actinobacteria.id.400	rs9833771	3	32362998	T	C	0.049007706	0.010656419	4.607550439	4.07E-06	17990
phylum.Actinobacteria.id.400	rs1397793	5	90471451	G	A	-0.052138694	0.011158738	-4.625459621	3.74E-06	17782
phylum.Actinobacteria.id.400	rs6496870	15	92467422	T	C	0.05107769	0.011380876	4.581458857	4.62E-06	17990
phylum.Actinobacteria.id.400	rs857444	6	14617591	C	T	0.050731493	0.010988454	4.621841104	3.80E-06	17988
phylum.Actinobacteria.id.400	rs12528285	6	92775210	C	T	0.08094693	0.018078413	4.537669938	5.69E-06	17990
phylum.Actinobacteria.id.400	rs80124826	2	239936384	T	C	-0.124191142	0.027856576	-4.445856941	8.75E-06	11999
phylum.Actinobacteria.id.400	rs13192624	6	113129609	T	C	-0.052286185	0.011774241	-4.432243464	9.33E-06	17985
phylum.Actinobacteria.id.400	rs75211493	19	5300465	G	A	0.084078593	0.018473851	4.433492701	9.27E-06	16605
phylum.Actinobacteria.id.400	rs6743026	2	102249583	T	C	0.058944616	0.013462245	4.419710896	9.88E-06	17875

Gut	SNP	CHR	POS	A1	A2	BETA	SE	Z	P	N
phylum.Actinobacteria.id.400	rs11766971	7	155002747	T	C	0.047637595	0.010646046	4.430550871	9.40E-06	17987
phylum.Bacteroidetes.id.905	rs73846128	3	89340254	A	G	-0.066426181	0.013348134	-5.03482099	4.78E-07	18335
phylum.Bacteroidetes.id.905	rs73512608	13	70522683	G	A	-0.123117207	0.02367433	-5.154807648	2.54E-07	11350
phylum.Bacteroidetes.id.905	rs111845179	14	59317502	T	C	0.104176948	0.021383712	4.976576897	6.47E-07	16394
phylum.Bacteroidetes.id.905	rs73975615	17	6461200	G	A	-0.207162752	0.044262873	-4.854960481	1.20E-06	3350
phylum.Bacteroidetes.id.905	rs2032750	2	53831026	T	C	-0.051085659	0.010680423	-4.785179363	1.71E-06	17854
phylum.Bacteroidetes.id.905	rs7546249	1	176792690	T	A	-0.056917331	0.01183824	-4.8222733	1.42E-06	18334
phylum.Bacteroidetes.id.905	rs13291169	9	116888284	C	G	0.070092873	0.014875617	4.699349611	2.61E-06	17514
phylum.Bacteroidetes.id.905	rs17343978	22	27433885	A	C	-0.055604102	0.012027223	-4.486958795	7.22E-06	18335
phylum.Bacteroidetes.id.905	rs929878	16	74290641	C	T	-0.054000099	0.012152588	-4.509071309	6.51E-06	18335
phylum.Bacteroidetes.id.905	rs72706335	1	157495438	T	C	-0.223193178	0.049338227	-4.489737306	7.13E-06	3820
phylum.Bacteroidetes.id.905	rs7999780	13	24963729	G	A	0.054363486	0.01240667	4.4290132	9.47E-06	18325
phylum.Bacteroidetes.id.905	rs6586324	21	43942135	T	C	0.047779239	0.010543692	4.482748261	7.37E-06	18332
phylum.Bacteroidetes.id.905	rs62531359	8	70916181	T	G	0.06577884	0.014988611	4.454136129	8.42E-06	18321
phylum.Bacteroidetes.id.905	rs62575403	9	136493820	C	T	0.145423872	0.031107867	4.673608639	2.96E-06	9669
phylum.Cyanobacteria.id.1500	rs789068	18	1008239	G	A	-0.111384961	0.021292193	-5.243954892	1.57E-07	7110
phylum.Cyanobacteria.id.1500	rs9864379	3	14306949	T	C	-0.138933303	0.026804476	-5.196263332	2.03E-07	6922
phylum.Cyanobacteria.id.1500	rs76531781	7	21647735	T	C	-0.231867725	0.049339267	-4.679670663	2.87E-06	3658
phylum.Cyanobacteria.id.1500	rs7148504	14	100168298	G	T	0.080040381	0.017662085	4.505428788	6.62E-06	7118
phylum.Cyanobacteria.id.1500	rs584122	6	48288456	C	T	-0.151773535	0.032682655	-4.599859933	4.23E-06	6922
phylum.Cyanobacteria.id.1500	rs2314810	17	26104110	C	G	-0.2182567	0.046457143	-4.598882545	4.25E-06	3934
phylum.Cyanobacteria.id.1500	rs2553290	4	37842537	A	T	0.096213138	0.02026167	4.632468583	3.61E-06	7113
phylum.Cyanobacteria.id.1500	rs12555298	9	110489551	G	A	0.097432733	0.021935139	4.462886828	8.09E-06	7096
phylum.Cyanobacteria.id.1500	rs2585223	15	100880888	T	C	0.111364409	0.024774324	4.443339212	8.86E-06	6911
phylum.Cyanobacteria.id.1500	rs61972390	13	100566758	T	C	0.107345656	0.024246867	4.437198809	9.11E-06	6486
phylum.Euryarchaeota.id.55	rs76029318	13	41963791	T	C	0.214866479	0.043844209	4.882739657	1.05E-06	3660
phylum.Euryarchaeota.id.55	rs73031978	3	15019595	C	G	-0.19752268	0.04144971	-4.68935381	2.74E-06	3501
phylum.Euryarchaeota.id.55	rs34928225	6	146840876	T	C	0.199769775	0.042542135	4.594834373	4.33E-06	3483
phylum.Euryarchaeota.id.55	rs7635189	3	15591607	G	A	0.119983324	0.0259356	4.580664853	4.64E-06	3483
phylum.Euryarchaeota.id.55	rs77658038	6	16284785	A	C	-0.160156679	0.034085617	-4.575351855	4.75E-06	3810
phylum.Euryarchaeota.id.55	rs894996	4	104418307	C	A	0.203576769	0.044003319	4.559837815	5.12E-06	3746

Gut	SNP	CHR	POS	A1	A2	BETA	SE	Z	P	N
phylum.Euryarchaeota.id.55	rs56131665	5	178160975	G	A	0.176758917	0.038578653	4.552394056	5.30E-06	3736
phylum.Euryarchaeota.id.55	rs45498998	21	27368994	G	A	-0.131882333	0.029228979	-4.551576899	5.32E-06	3850
phylum.Euryarchaeota.id.55	rs7015093	8	58018853	G	A	-0.118111053	0.02643893	-4.487727417	7.20E-06	3855
phylum.Euryarchaeota.id.55	rs11022995	11	13893667	G	A	-0.103729602	0.022888851	-4.472528591	7.73E-06	3864
phylum.Euryarchaeota.id.55	rs6508769	19	28827760	T	C	0.150528693	0.033709102	4.461901895	8.12E-06	3483
phylum.Euryarchaeota.id.55	rs6064552	20	56042866	T	C	-0.123611632	0.02757488	-4.431965385	9.34E-06	3775
phylum.Firmicutes.id.1672	rs3792064	2	231678413	G	A	0.089734864	0.018253212	4.968278712	6.75E-07	17812
phylum.Firmicutes.id.1672	rs2009919	17	11780026	T	C	0.053645718	0.010629448	5.028350743	4.95E-07	17859
phylum.Firmicutes.id.1672	rs112334273	21	40703251	G	A	0.062745897	0.012741527	4.906649948	9.26E-07	18217
phylum.Firmicutes.id.1672	rs7975768	12	97374875	C	G	0.051273172	0.010922627	4.683162524	2.82E-06	17859
phylum.Firmicutes.id.1672	rs2332027	4	171672038	G	A	-0.048265611	0.010482301	-4.609032878	4.05E-06	18340
phylum.Firmicutes.id.1672	rs7247191	19	22899585	T	C	-0.071303301	0.015742403	-4.576375327	4.73E-06	18340
phylum.Firmicutes.id.1672	rs72771021	10	13841631	C	T	-0.141356654	0.030830333	-4.559629997	5.12E-06	9068
phylum.Firmicutes.id.1672	rs3852931	20	54087963	C	T	-0.048208647	0.010499991	-4.585641931	4.53E-06	18260
phylum.Firmicutes.id.1672	rs2547978	5	98020298	A	G	0.046580183	0.010507469	4.450465062	8.57E-06	18338
phylum.Firmicutes.id.1672	rs36111799	10	39136170	C	T	-0.04807078	0.010542949	-4.557589394	5.17E-06	18226
phylum.Firmicutes.id.1672	rs12554342	9	116840801	G	C	-0.052570302	0.01169079	-4.499588942	6.81E-06	18332
phylum.Firmicutes.id.1672	rs72738886	5	35770550	T	C	0.086460893	0.019039766	4.473948053	7.68E-06	16934
phylum.Firmicutes.id.1672	rs6815608	4	152131744	T	C	0.093576464	0.021086864	4.486549392	7.24E-06	16731
phylum.Firmicutes.id.1672	rs8085381	18	29328305	G	A	0.064729115	0.014621949	4.447871761	8.67E-06	18339
phylum.Firmicutes.id.1672	rs56199908	9	2801371	T	C	-0.186193968	0.041050975	-4.448019657	8.67E-06	5618
phylum.Firmicutes.id.1672	rs7573799	2	80141964	C	G	-0.069844604	0.015393468	-4.463806241	8.05E-06	17859
phylum.Firmicutes.id.1672	rs6814436	4	161507301	T	C	0.068020505	0.015084366	4.499709912	6.80E-06	18336
phylum.Lentisphaerae.id.2238	rs2825714	21	21023966	A	G	-0.138226357	0.028909621	-4.811539832	1.50E-06	4739
phylum.Lentisphaerae.id.2238	rs77599476	20	61394262	A	G	0.229981464	0.047991818	4.763500229	1.90E-06	3726
phylum.Lentisphaerae.id.2238	rs62570196	9	111086170	C	T	-0.217203229	0.043962139	-4.898751732	9.64E-07	3716
phylum.Lentisphaerae.id.2238	rs72640280	1	11943792	A	G	0.220185072	0.048594661	4.556877853	5.19E-06	3820
phylum.Lentisphaerae.id.2238	rs11764871	7	146808977	G	T	0.10842739	0.023334631	4.62733763	3.70E-06	4730
phylum.Lentisphaerae.id.2238	rs17114848	15	25162535	G	A	0.148972108	0.032416219	4.500796279	6.77E-06	4739
phylum.Lentisphaerae.id.2238	rs1002941	15	101242690	G	A	0.107660547	0.023338177	4.595902705	4.31E-06	4739
phylum.Lentisphaerae.id.2238	rs2731834	5	94720245	C	G	0.109934636	0.023684845	4.622249658	3.80E-06	4738

Gut	SNP	CHR	POS	A1	A2	BETA	SE	Z	P	N
phylum.Lentisphaerae.id.2238	rs60995569	10	53930085	T	G	-0.160620002	0.033680552	-4.435311412	9.19E-06	4737
phylum.Lentisphaerae.id.2238	rs2031282	13	20687179	A	G	0.120411947	0.027026292	4.53121195	5.86E-06	4739
phylum.Lentisphaerae.id.2238	rs73113483	3	6223568	T	A	-0.131681316	0.028850337	-4.444886099	8.79E-06	4723
phylum.Proteobacteria.id.2375	rs922773	3	66318042	C	T	-0.080377814	0.015785107	-5.084828806	3.68E-07	18132
phylum.Proteobacteria.id.2375	rs2532663	10	119247234	G	A	-0.125578004	0.025803359	-4.948777214	7.47E-07	13013
phylum.Proteobacteria.id.2375	rs2347697	7	134388240	G	T	0.050228084	0.010896783	4.597829912	4.27E-06	18248
phylum.Proteobacteria.id.2375	rs312757	17	68347701	C	G	-0.051726884	0.011285376	-4.57164955	4.84E-06	18250
phylum.Proteobacteria.id.2375	rs12467198	2	124872681	C	T	0.049826716	0.011137964	4.515837797	6.31E-06	18238
phylum.Proteobacteria.id.2375	rs10750258	11	123997498	A	C	-0.049124751	0.010824018	-4.446723713	8.72E-06	18250
phylum.Proteobacteria.id.2375	rs6707783	2	52053891	C	T	0.084959348	0.018778235	4.46289598	8.09E-06	17324
phylum.Proteobacteria.id.2375	rs72771021	10	13841631	C	T	0.141725865	0.03092421	4.488252697	7.18E-06	9009
phylum.Proteobacteria.id.2375	rs3890996	22	20301800	T	G	-0.047461372	0.010564451	-4.495317176	6.95E-06	18120
phylum.Proteobacteria.id.2375	rs74757828	8	89541526	A	T	0.095128506	0.021692115	4.433985874	9.25E-06	16634
phylum.Proteobacteria.id.2375	rs4340090	12	130331764	C	T	-0.066816186	0.015338746	-4.417389097	9.99E-06	18250
phylum.Proteobacteria.id.2375	rs11715072	3	53720793	G	A	-0.051928888	0.01152464	-4.496623953	6.90E-06	18249
phylum.Tenericutes.id.3919	rs74603314	14	46519718	T	C	0.221639279	0.046291765	4.80379142	1.56E-06	4499
phylum.Tenericutes.id.3919	rs10108398	8	59440824	G	A	0.076914215	0.015395275	4.87465347	1.09E-06	10932
phylum.Tenericutes.id.3919	rs11890098	2	157532549	A	G	0.074438031	0.015338858	4.900368368	9.57E-07	11392
phylum.Tenericutes.id.3919	rs72901605	11	47103877	T	C	-0.084185237	0.01781192	-4.653852616	3.26E-06	10526
phylum.Tenericutes.id.3919	rs3768491	1	109965986	A	G	-0.068105197	0.01490611	-4.599575976	4.23E-06	11002
phylum.Tenericutes.id.3919	rs17214486	14	97159674	C	A	0.060989958	0.013562263	4.505917879	6.61E-06	11387
phylum.Tenericutes.id.3919	rs12566890	1	61850864	T	G	-0.101147132	0.023097845	-4.630323303	3.65E-06	11371
phylum.Tenericutes.id.3919	rs78169027	11	108439087	A	G	-0.108282943	0.023728915	-4.530798993	5.88E-06	10542
phylum.Tenericutes.id.3919	rs28537087	15	95309676	G	A	0.08208727	0.018831168	4.463212567	8.07E-06	11382
phylum.Tenericutes.id.3919	rs2464826	7	79490250	A	C	0.094423946	0.021180616	4.454853483	8.40E-06	10998
phylum.Verrucomicrobia.id.3982	rs74542928	4	100544188	T	C	0.115994333	0.023132372	5.065011642	4.08E-07	11703
phylum.Verrucomicrobia.id.3982	rs11252894	10	5073050	A	C	0.078405494	0.016276917	4.871853908	1.11E-06	12381
phylum.Verrucomicrobia.id.3982	rs2602429	16	81063149	C	T	0.076414823	0.015348672	4.918737817	8.71E-07	12104
phylum.Verrucomicrobia.id.3982	rs11729256	4	95027272	T	C	0.069698414	0.01469534	4.73101332	2.23E-06	12377
phylum.Verrucomicrobia.id.3982	rs45598138	1	55522083	C	A	-0.143901454	0.030540001	-4.73493356	2.19E-06	9932
phylum.Verrucomicrobia.id.3982	rs61779207	1	41074472	G	A	-0.075525262	0.016438461	-4.553354802	5.28E-06	12087

Gut	SNP	CHR	POS	A1	A2	BETA	SE	Z	P	N
phylum.Verrucomicrobia.id.3982	rs76430504	5	40438158	T	C	-0.117530089	0.025458042	-4.639318265	3.50E-06	11624
phylum.Verrucomicrobia.id.3982	rs9349825	6	56341481	A	G	-0.066024311	0.014391139	-4.5169097	6.27E-06	12373
phylum.Verrucomicrobia.id.3982	rs117107102	18	49473635	A	G	0.204282775	0.042756505	4.693983641	2.68E-06	5071
phylum.Verrucomicrobia.id.3982	rs3995795	10	11389614	C	T	0.061091431	0.013859187	4.42326392	9.72E-06	12104
phylum.Verrucomicrobia.id.3982	rs12908520	15	97570657	G	A	0.059491573	0.012823012	4.644774305	3.40E-06	12380

Supplementary table 2. Genetic correlation of valvular heart disease with gastrointestinal disorders. Bonferroni significant results are indicated in red.

Phenotype	rg	se	p
Gastro-oesophageal reflux disease	0.2113	0.0307	5.41E-12
Irritable bowel syndrome	0.2274	0.0335	1.16E-11
Peptic ulcer disease	0.2104	0.0431	1.06E-06
Inflammatory bowel disease	0.0656	0.0239	0.006
Crohn's disease	0.0778	0.0239	0.0011
Ulcerative colitis	0.0404	0.0257	0.1163

Supplementary table 3.Validation of pleiotropic loci in an independent valvular heart disease GWAS dataset

Loci	SNP	CHR	POS	A1	BETA	SE	P
4:111570369-111733700	rs2350269	4	111687124	T	0.147	0.015	2.91E-22
10:35182402-35862021	rs12264697	10	35464168	A	-0.045	0.011	8.85E-05
16:50164049-50820433	rs1209480260	16	50571354	T	2.411	0.731	9.75E-04
6:29236722-33103592	rs474534	6	31938107	G	-0.082	0.017	1.06E-06
5:130438101-131955528	rs746482098	5	130581528	G	-0.797	0.232	5.89E-04
20:62191558-62649655	rs901604953	20	62380046	G	1.023	0.259	7.92E-05
22:21747094-22070146	rs141038677	22	21936651	G	0.337	0.105	1.32E-03
1:113819380-114564892	rs3827733	1	114338589	G	0.048	0.013	2.85E-04
5:40199659-40954627	rs1545334	5	40430118	G	0.046	0.011	1.83E-05
4:111486625-111639446	rs2723320	4	111639446	C	-0.096	0.013	4.35E-13
6:29764188-33103592	rs474534	6	31938107	G	-0.082	0.017	1.06E-06
6:30023079-33103592	rs474534	6	31938107	G	-0.082	0.017	1.06E-06
5:39666312-41004406	rs1545334	5	40430118	G	0.046	0.011	1.83E-05
10:35184091-35877103	rs12264697	10	35464168	A	-0.045	0.011	8.85E-05
16:50627378-50846162	rs530061257	16	50732120	G	1.101	0.339	1.18E-03
21:40476322-40867898	rs4818028	21	40723256	G	0.039	0.011	3.72E-04
12:111884608-113168993	rs78639818	12	113051513	A	-0.092	0.02	6.43E-06
17:40261284-41459600	rs111299379	17	41127468	C	-0.376	0.087	1.39E-05
6:29783305-33103592	rs474534	6	31938107	G	-0.082	0.017	1.06E-06
9:136041865-136368406	rs148343449	9	136360946	A	0.442	0.094	2.67E-06
19:49092627-49334991	rs1037669569	19	49200400	G	1.435	0.368	9.60E-05
9:21948078-22125913	rs7341791	9	22112427	G	0.052	0.01	6.86E-07
6:160703358-161341729	rs140570886	6	161013013	C	0.253	0.051	6.44E-07
10:101109760-101341427	rs78606180	10	101288805	T	0.077	0.019	6.24E-05
7:2725463-2930941	rs798529	7	2772290	C	-0.037	0.011	3.92E-04
6:30049966-33348012	rs474534	6	31938107	G	-0.082	0.017	1.06E-06
1:20083582-20140036	rs1349996243	1	20113938	T	0.913	0.317	0.004
7:107461908-107603431	rs116929910	7	107599283	T	0.091	0.032	0.005

Supplementary table 4. Gene-based pleiotropy signals of VHD with gastrointestinal disorders. P values surviving Bonferroni correction are indicated in red.

Gene	Ensembl ID	CHR	START	STOP	VHD_CD Pleiotropy P	VHD_GORD Pleiotropy P	VHD_IBD Pleiotropy P	VHD_IBS Pleiotropy P	VHD_PUD Pleiotropy P	VHD_UC Pleiotropy P	d
PBX2	ENSG00000204304	6	32152512	32157963	2.27E-06	1.76E-06	3.15E-07	2.79E-03	1.73E-02	4.45E-13	4
C6orf48	ENSG00000204387	6	31802385	31807541	1.01E-08	4.83E-07	3.80E-08	2.73E-05	2.99E-04	1.70E-08	4
MSH5	ENSG00000204410	6	31707725	31732622	1.55E-10	4.40E-09	1.56E-09	1.79E-06	7.83E-04	4.82E-06	4
PRRC2A	ENSG00000204469	6	31588497	31605548	8.41E-07	1.48E-07	2.24E-06	2.28E-07	3.22E-03	6.63E-06	4
AGPAT1	ENSG00000204310	6	32135989	32145873	3.87E-05	9.37E-08	3.02E-12	1.34E-03	5.38E-02	2.39E-13	3
NOTCH4	ENSG00000204301	6	32162620	32191844	8.78E-09	1.01E-04	2.87E-11	8.56E-05	7.92E-02	1.73E-11	3
PPT2	ENSG00000221988	6	32121218	32134011	9.30E-06	9.03E-08	9.12E-11	1.41E-03	6.79E-02	2.77E-11	3
RNF5	ENSG00000204308	6	32146131	32151930	6.18E-03	4.28E-08	1.14E-07	2.41E-04	9.84E-02	9.65E-11	3
AGER	ENSG00000204305	6	32148745	32152101	1.63E-02	1.63E-07	1.23E-06	1.25E-04	2.66E-01	2.22E-10	3
EGFL8	ENSG00000241404	6	32132360	32136058	3.51E-03	4.13E-07	1.49E-07	6.70E-03	9.14E-02	1.33E-09	3
ATF6B	ENSG00000213676	6	32065953	32096030	1.08E-07	7.44E-03	2.00E-07	1.76E-03	2.53E-02	8.62E-07	3
UBE2L3	ENSG00000185651	22	21903736	21978323	7.58E-11	4.43E-03	1.53E-10	1.88E-01	7.73E-03	1.78E-06	3
LSM2	ENSG00000204392	6	31765173	31774761	2.89E-11	1.89E-06	8.54E-09	4.55E-05	4.87E-05	1.13E-05	3
VARS	ENSG00000204394	6	31745295	31763730	2.15E-09	6.24E-08	1.87E-07	9.78E-06	1.94E-03	1.62E-05	3
VWA7	ENSG00000204396	6	31733367	31745108	1.78E-10	5.04E-07	7.80E-07	2.76E-05	3.65E-03	3.11E-05	3
HLA-DQA1	ENSG00000196735	6	32595956	32614839	2.73E-05	1.26E-02	4.19E-09	2.66E-04	4.61E-02	8.07E-10	2
POU5F1	ENSG00000204531	6	31132119	31148508	5.36E-05	1.11E-03	2.80E-06	5.46E-02	1.20E-01	1.07E-09	2
HCG27	ENSG00000206344	6	31165537	31171745	5.17E-05	8.37E-05	1.36E-06	1.13E-02	3.19E-02	1.36E-07	2
GPSM3	ENSG00000213654	6	32158543	32163300	7.06E-03	5.60E-06	1.47E-06	1.46E-04	1.20E-01	3.45E-07	2
DNMT3A	ENSG00000119772	2	25455845	25565459	2.92E-06	3.09E-02	1.76E-07	1.18E-01	3.40E-03	7.29E-06	2
YDJC	ENSG00000161179	22	21982378	21984353	2.64E-09	1.23E-02	2.19E-08	1.35E-01	1.21E-02	1.56E-05	2
CUL2	ENSG00000108094	10	35297479	35379570	8.48E-09	6.40E-02	4.19E-08	3.56E-01	9.78E-02	7.54E-05	2
CREM	ENSG00000095794	10	35415719	35501886	1.06E-08	5.83E-02	6.35E-08	3.12E-01	7.79E-02	1.16E-04	2
ATP6V1G2	ENSG00000213760	6	31512239	31516204	4.59E-07	2.87E-07	5.00E-04	2.14E-04	2.06E-04	1.57E-04	2
IL23R	ENSG00000162594	1	67632083	67725662	6.06E-08	3.60E-01	3.43E-07	2.90E-01	8.37E-01	1.68E-03	2
TCF19	ENSG00000137310	6	31126319	31134936	1.78E-04	9.36E-03	1.19E-04	8.90E-02	8.60E-02	1.09E-08	1
PRRT1	ENSG00000204314	6	32116136	32122150	1.45E-04	6.96E-05	1.26E-05	6.47E-03	1.76E-01	3.11E-08	1
PSORS1C1	ENSG00000204540	6	31082527	31107869	8.94E-06	4.64E-04	3.60E-05	2.18E-03	2.03E-03	6.24E-08	1
AMZ1	ENSG00000174945	7	2719156	2815134	2.19E-01	7.50E-02	1.50E-05	6.72E-02	1.68E-01	1.81E-07	1
CCHCR1	ENSG00000204536	6	31110216	31126015	4.25E-04	2.17E-03	2.49E-04	5.03E-02	2.16E-01	4.42E-07	1
CFDP1	ENSG00000153774	16	75327596	75467383	1.52E-02	6.57E-02	1.07E-05	4.27E-03	2.25E-02	5.03E-07	1
HLA-DPB1	ENSG00000223865	6	33043703	33054978	6.87E-02	2.25E-01	4.22E-04	7.08E-02	3.70E-01	5.27E-07	1
TMEM170A	ENSG00000166822	16	75476952	75499395	1.20E-02	2.83E-02	6.17E-06	8.93E-03	2.53E-02	9.11E-07	1
CDSN	ENSG00000204539	6	31082867	31088223	7.06E-04	4.03E-03	8.40E-04	5.50E-03	7.69E-03	2.82E-06	1
CCDC36	ENSG00000173421	3	49235861	49295537	1.25E-05	2.30E-01	3.16E-07	1.01E-01	2.03E-01	3.45E-06	1
HLA-DRA	ENSG00000204287	6	32407619	32412823	7.04E-04	2.62E-03	1.32E-08	6.21E-03	1.09E-01	3.60E-06	1
STAT3	ENSG00000168610	17	40465342	40540586	6.15E-06	2.45E-01	2.69E-07	2.13E-01	2.73E-01	7.10E-06	1
STMN3	ENSG00000197457	20	62271061	62284780	6.35E-06	3.03E-02	3.53E-07	7.16E-01	1.22E-01	7.13E-06	1
C3orf62	ENSG00000188315	3	49306035	49315342	4.91E-05	7.56E-01	1.34E-06	5.97E-02	1.67E-01	7.30E-06	1
IMPDH2	ENSG00000178035	3	49061758	49066841	1.58E-05	2.62E-01	1.86E-07	1.64E-01	1.42E-01	9.49E-06	1
PUS10	ENSG00000162927	2	61167357	61245394	9.88E-06	1.11E-01	7.48E-07	1.57E-01	2.23E-01	1.95E-05	1
KLHDC8B	ENSG00000185909	3	49209044	49213917	6.35E-05	3.17E-01	2.46E-06	3.56E-02	8.66E-02	2.15E-05	1
GMEB2	ENSG00000101216	20	62218955	62258394	2.29E-05	4.77E-02	8.33E-07	2.15E-01	1.06E-01	2.51E-05	1
QARS	ENSG00000172053	3	49133365	49142553	1.60E-05	1.53E-01	2.43E-06	3.50E-02	3.22E-01	5.81E-05	1
CLIC1	ENSG00000213719	6	31698358	31707540	6.00E-08	3.71E-06	2.16E-05	6.06E-05	1.11E-02	8.55E-04	1
SLC22A3	ENSG00000146477	6	160769300	160876014	4.53E-02	4.28E-04	1.17E-02	9.13E-03	2.15E-06	2.89E-03	1
TRIM15	ENSG00000204610	6	30130993	30140473	1.01E-07	3.71E-03	4.42E-04	1.90E-02	1.09E-01	2.92E-03	1
C1orf141	ENSG00000203963	1	67557848	67697536	8.62E-07	2.77E-01	3.76E-06	1.89E-01	5.09E-01	3.70E-03	1

Gene	Ensembl ID	CHR	START	STOP	VHD_CD Pleiotropy P	VHD_GORD Pleiotropy P	VHD_IBD Pleiotropy P	VHD_IBS Pleiotropy P	VHD_PUD Pleiotropy P	VHD_UC Pleiotropy P	d
P4HA2	ENSG00000072682	5	131527531	131631008	5.35E-06	3.07E-01	2.90E-06	3.12E-01	4.51E-01	4.30E-03	1
C5orf56	ENSG00000197536	5	131746328	131811736	1.56E-06	2.03E-01	4.23E-06	1.22E-01	3.86E-01	4.73E-03	1
ZNF75A	ENSG00000162086	16	3355406	3368852	1.32E-06	7.37E-03	8.61E-05	1.14E-02	8.15E-04	1.10E-02	1
SPATS2L	ENSG00000196141	2	201170604	201346986	1.54E-06	8.65E-03	5.00E-05	9.01E-03	1.68E-03	1.16E-02	1
CCL2	ENSG00000108691	17	32582304	32584222	1.18E-06	2.78E-01	1.15E-05	8.58E-01	1.32E-01	1.19E-02	1
NOD2	ENSG00000167207	16	50727514	50766988	1.54E-05	4.11E-02	7.92E-10	3.65E-02	4.65E-01	1.79E-02	1
GTF2H4	ENSG00000213780	6	30875961	30881883	3.43E-08	3.92E-03	6.98E-05	2.63E-02	2.48E-02	4.38E-02	1
PSCA	ENSG00000167653	8	143751726	143764142	7.30E-01	5.42E-04	1.47E-01	2.13E-01	3.02E-07	5.22E-02	1
HOXC9	ENSG00000180806	12	54388679	54397121	2.26E-01	2.28E-07	7.40E-02	7.17E-02	1.32E-03	5.24E-02	1
HOXC8	ENSG00000037965	12	54402832	54407570	5.36E-01	2.77E-07	5.85E-02	5.51E-01	9.20E-03	6.91E-02	1
SFTA2	ENSG00000196260	6	30899130	30899952	1.87E-05	1.93E-07	1.64E-02	6.29E-05	9.12E-04	1.18E-01	1
BCL2L15	ENSG00000188761	1	114420790	114430169	1.54E-07	7.48E-03	9.08E-05	1.97E-02	1.80E-02	2.64E-01	1
DCLRE1B	ENSG00000118655	1	114447763	114456708	1.12E-07	8.47E-04	2.15E-04	1.30E-02	3.92E-02	2.93E-01	1
FUT2	ENSG00000176920	19	49199228	49209207	6.88E-02	4.71E-01	2.27E-01	6.41E-02	3.50E-08	3.58E-01	1
AP4B1	ENSG00000134262	1	114437370	114447823	6.61E-07	4.46E-02	4.69E-04	7.78E-02	9.33E-02	6.19E-01	1
ATG16L1	ENSG00000085978	2	234118697	234204320	1.91E-07	6.08E-01	2.06E-04	6.34E-01	5.11E-01	8.65E-01	1
BAG6	ENSG00000204463	6	31606805	31620482	5.87E-05	8.49E-05	8.50E-05	3.37E-05	2.34E-02	7.41E-06	0
OTUD3	ENSG00000169914	1	20209006	20239438	8.84E-01	8.89E-01	4.65E-03	5.23E-01	5.32E-01	7.91E-06	0
MICB	ENSG00000204516	6	31462658	31478901	1.46E-05	2.44E-03	3.88E-06	2.14E-04	2.85E-02	8.42E-06	0
PSORS1C2	ENSG00000204538	6	31105313	31107127	6.23E-06	7.92E-03	3.49E-06	1.74E-02	1.70E-03	9.14E-06	0
GNA12	ENSG00000146535	7	2767746	2883958	1.18E-01	2.25E-01	2.96E-04	4.60E-02	1.36E-01	9.87E-06	0
DUSP8	ENSG00000184545	11	1575274	1593150	1.21E-02	3.57E-02	3.55E-05	5.39E-04	4.02E-03	1.00E-05	0
IP6K2	ENSG00000068745	3	48725436	48777786	1.83E-04	3.95E-01	4.11E-06	1.97E-01	1.17E-01	1.05E-05	0
BRD2	ENSG00000204256	6	32936437	32949282	2.87E-03	7.44E-02	4.47E-05	3.18E-02	3.89E-02	1.45E-05	0
HLA-DMA	ENSG00000204257	6	32916390	32936871	1.34E-02	1.16E-01	1.05E-04	2.98E-02	5.79E-02	1.47E-05	0
ZFP90	ENSG00000184939	16	68563993	68609975	1.05E-01	2.29E-02	1.92E-04	1.92E-01	3.16E-01	1.73E-05	0
SAPCD1	ENSG00000228727	6	31730576	31732628	2.62E-02	2.88E-02	7.51E-05	1.06E-01	6.33E-01	2.48E-05	0
SLC26A6	ENSG00000225697	3	48663156	48672926	3.57E-04	4.03E-01	1.03E-05	1.04E-01	7.06E-02	2.72E-05	0
NFKBIL1	ENSG00000204498	6	31514647	31526606	1.99E-05	6.45E-03	3.49E-05	2.58E-02	7.58E-02	3.14E-05	0
SKIV2L	ENSG00000204351	6	31926857	31937532	7.43E-06	7.81E-03	2.86E-05	6.78E-05	1.14E-01	3.33E-05	0
HLA-DPA1	ENSG00000231389	6	33032346	33048552	9.98E-02	3.02E-02	2.02E-02	9.90E-02	4.26E-01	3.43E-05	0
PLA2G2E	ENSG00000188784	1	20246502	20250110	8.13E-01	5.60E-02	3.36E-03	4.41E-01	3.12E-02	4.19E-05	0
FKBPL	ENSG00000204315	6	32096484	32098068	1.67E-05	3.17E-01	9.38E-02	7.05E-03	1.51E-03	4.24E-05	0
QRICH1	ENSG00000198218	3	49067140	49131796	1.76E-04	1.48E-01	5.49E-06	1.18E-01	3.40E-01	4.61E-05	0
SHISA5	ENSG00000164054	3	48509197	48542259	5.26E-04	9.22E-03	3.64E-05	8.03E-03	2.30E-01	7.16E-05	0
C2	ENSG00000166278	6	31865562	31913449	9.33E-06	4.40E-03	1.26E-03	9.23E-04	2.19E-02	7.19E-05	0
EHMT2	ENSG00000204371	6	31847536	31865464	2.15E-04	8.13E-03	1.18E-03	4.60E-04	2.41E-01	7.54E-05	0
ATP6V1G2-DDX39B	ENSG00000254870	6	31497996	31514385	9.60E-06	2.32E-04	3.58E-04	3.25E-04	4.62E-03	7.80E-05	0
NEFH	ENSG00000100285	22	29876219	29887379	3.88E-02	4.01E-01	6.56E-04	3.98E-02	4.35E-01	8.90E-05	0
SLC25A28	ENSG00000155287	10	101370282	101380366	4.62E-03	9.55E-03	9.29E-05	1.96E-02	7.61E-03	9.06E-05	0
SLC9B1	ENSG00000164037	4	103806205	103940896	3.84E-01	9.82E-02	2.08E-03	6.25E-02	8.54E-03	9.37E-05	0
CUTC	ENSG00000119929	10	101462315	101515891	1.03E-02	1.14E-02	2.66E-04	4.56E-02	9.90E-03	9.39E-05	0
CELSR3	ENSG00000008300	3	48673902	48700348	7.13E-04	5.30E-01	3.84E-05	2.10E-01	6.96E-02	9.77E-05	0
RNF145	ENSG00000145860	5	158584417	158637061	6.40E-05	4.67E-04	7.28E-05	2.79E-02	2.54E-02	1.00E-04	0
MON1A	ENSG00000164077	3	49946302	49967606	2.73E-04	2.37E-03	2.97E-05	2.19E-01	2.29E-01	1.02E-04	0
CTSZ	ENSG00000101160	20	57570240	57582302	5.59E-02	3.82E-01	1.91E-03	6.32E-03	1.02E-01	1.19E-04	0
CISD2	ENSG00000145354	4	103790135	103810399	4.66E-01	7.32E-02	2.35E-03	5.13E-02	5.91E-03	1.20E-04	0
DGAT1	ENSG00000185000	8	145539954	145550573	3.94E-04	1.39E-02	5.11E-05	6.99E-02	6.09E-03	1.32E-04	0
C19orf12	ENSG00000131943	19	30191721	30206364	8.07E-01	3.39E-02	1.75E-02	5.00E-02	5.93E-03	1.42E-04	0

Gene	Ensembl ID	CHR	START	STOP	VHD_CD Pleiotropy P	VHD_GORD Pleiotropy P	VHD_IBD Pleiotropy P	VHD_IBS Pleiotropy P	VHD_PUD Pleiotropy P	VHD_UC Pleiotropy P	d
TNFRSF14	ENSG00000157873	1	2487078	2496821	3.11E-01	8.67E-01	1.10E-03	7.39E-01	1.42E-01	1.48E-04	0
UBE2D3	ENSG00000109332	4	103715540	103790053	3.02E-01	6.49E-02	3.31E-03	3.32E-02	5.57E-03	1.67E-04	0
RAC1	ENSG00000136238	7	6414154	6443608	2.47E-02	1.28E-01	2.67E-04	3.96E-01	1.39E-01	1.72E-04	0
SLC22A2	ENSG00000112499	6	160592093	160698670	1.77E-02	3.41E-02	3.71E-04	5.79E-02	3.61E-05	1.77E-04	0
ENTPD7	ENSG00000198018	10	101419263	101465997	2.98E-03	1.03E-02	1.32E-04	1.99E-02	5.38E-03	1.83E-04	0
S1PR5	ENSG00000180739	19	10623623	10628607	5.02E-04	1.05E-01	1.67E-04	5.70E-02	1.94E-02	1.89E-04	0
COX15	ENSG00000014919	10	101471601	101491857	4.76E-03	3.06E-02	1.80E-04	2.85E-02	1.44E-02	1.93E-04	0
CAMKV	ENSG00000164076	3	49895421	49907655	1.78E-03	3.24E-03	1.45E-04	3.97E-01	1.77E-01	1.98E-04	0
NCKIPSD	ENSG00000213672	3	48701364	48723797	7.36E-04	3.96E-01	5.76E-05	2.81E-01	5.26E-02	2.05E-04	0
TMEM89	ENSG00000183396	3	48658192	48659288	6.09E-03	4.51E-01	1.65E-04	1.16E-01	8.96E-02	2.05E-04	0
ACTA1	ENSG00000143632	1	229566992	229569845	1.12E-04	1.31E-02	1.72E-05	3.15E-03	3.98E-01	2.14E-04	0
SLC26A3	ENSG00000091138	7	107405912	107443670	1.20E-01	1.67E-01	1.84E-03	3.76E-02	1.17E-01	2.29E-04	0
IL2	ENSG00000109471	4	123372625	123377880	7.35E-05	5.44E-01	1.10E-05	2.41E-02	4.15E-01	2.30E-04	0
UBLCP1	ENSG00000164332	5	158690089	158713044	1.64E-04	1.20E-01	1.81E-05	3.91E-01	1.69E-01	2.66E-04	0
KIAA1841	ENSG00000162929	2	61293006	61391960	6.28E-04	1.80E-02	3.70E-05	9.84E-02	2.25E-01	2.67E-04	0
ENPEP	ENSG00000138792	4	111286889	111486441	3.54E-02	1.29E-04	1.35E-02	2.80E-04	8.57E-04	2.70E-04	0
HSPA1L	ENSG00000204390	6	31777396	31783437	1.90E-04	4.36E-01	5.34E-05	2.30E-01	1.92E-02	2.90E-04	0
HLA-DMB	ENSG00000242574	6	32902406	32908847	1.41E-02	1.45E-01	7.68E-04	3.89E-02	2.88E-02	3.05E-04	0
C6orf10	ENSG00000204296	6	32256303	32339684	1.09E-03	8.09E-03	1.13E-03	1.04E-01	3.89E-01	3.06E-04	0
TAP2	ENSG00000250264	6	32781544	32806599	7.46E-04	4.58E-02	1.75E-04	1.67E-01	4.44E-01	3.10E-04	0
SLC1A5	ENSG00000105281	19	47278140	47291851	2.35E-01	3.34E-01	1.89E-03	6.25E-01	2.44E-01	3.12E-04	0
HDGFL1	ENSG00000112273	6	22569678	22571892	1.55E-01	5.59E-02	1.03E-02	7.80E-03	1.65E-03	3.22E-04	0
SATB2	ENSG00000119042	2	200134223	200335989	1.30E-01	6.09E-01	5.74E-03	6.59E-02	1.08E-01	3.23E-04	0
EDNRA	ENSG00000151617	4	148402069	148466106	6.01E-04	1.23E-02	9.54E-05	1.22E-01	9.48E-04	3.25E-04	0
COL7A1	ENSG00000114270	3	48601506	48632700	9.56E-04	2.97E-02	1.22E-04	8.62E-03	3.06E-01	3.30E-04	0
MYO9B	ENSG00000099331	19	17186591	17325346	1.17E-01	1.97E-02	2.86E-03	1.61E-03	1.05E-02	3.41E-04	0
MOB2	ENSG00000182208	11	1490687	1522477	3.07E-02	9.00E-02	5.45E-04	6.20E-03	8.02E-02	3.51E-04	0
PFKFB4	ENSG00000114268	3	48555117	48599448	4.17E-03	4.02E-02	1.81E-04	1.79E-02	4.69E-01	3.54E-04	0
SLC9B2	ENSG00000164038	4	103941025	104006986	1.13E-01	2.93E-02	1.45E-02	1.53E-01	5.99E-02	3.61E-04	0
LHB	ENSG00000104826	19	49519237	49520338	4.37E-04	1.93E-02	9.32E-05	3.19E-02	2.72E-01	3.82E-04	0
PARK7	ENSG00000116288	1	8014351	8045565	6.29E-05	3.53E-02	1.22E-05	1.33E-01	2.32E-01	3.84E-04	0
C1orf68	ENSG00000198854	1	152691998	152692905	3.11E-02	3.89E-01	3.19E-03	2.70E-01	8.19E-01	4.04E-04	0
ATG4D	ENSG00000130734	19	10654571	10664094	3.37E-04	9.95E-02	4.45E-05	4.42E-01	2.82E-02	4.09E-04	0
ME2	ENSG00000082212	18	48405419	48474691	2.76E-03	6.09E-01	2.12E-04	4.70E-02	8.62E-02	4.33E-04	0
PRKAR2A	ENSG00000114302	3	48782030	48885279	3.88E-04	2.28E-01	5.19E-05	1.53E-01	6.06E-01	4.52E-04	0
DPH5	ENSG00000117543	1	101455179	101491644	3.28E-01	3.01E-01	2.72E-03	2.57E-01	4.94E-01	4.71E-04	0
MUC22	ENSG00000261272	6	30978251	31003179	5.69E-03	7.10E-03	5.64E-03	9.11E-03	3.48E-01	4.74E-04	0
EFNA1	ENSG00000169242	1	155099936	155107333	4.42E-04	1.77E-02	5.55E-05	1.93E-01	5.33E-02	5.14E-04	0
NR4A1	ENSG00000123358	12	52416616	52453291	2.64E-02	1.67E-01	1.64E-03	2.65E-01	8.93E-02	5.25E-04	0
MANBA	ENSG00000109323	4	103552660	103682151	2.41E-02	1.46E-02	1.37E-01	1.00E-01	3.75E-02	5.39E-04	0
C1orf106	ENSG00000163362	1	200860176	200884863	1.97E-02	4.17E-01	9.89E-05	4.40E-01	6.15E-01	5.45E-04	0
IL27	ENSG00000197272	16	28510683	28523372	1.76E-05	1.32E-01	7.71E-06	7.35E-02	6.42E-02	5.46E-04	0
CCNE1	ENSG00000105173	19	30302805	30315215	4.26E-02	6.64E-02	1.39E-02	1.63E-03	7.81E-02	5.67E-04	0
STAT5B	ENSG00000173757	17	40351186	40428725	8.02E-05	2.27E-01	2.65E-05	3.98E-02	1.95E-01	5.70E-04	0
BOP1	ENSG00000170727	8	145486055	145515082	1.72E-04	2.01E-03	4.06E-05	2.19E-02	1.03E-02	5.74E-04	0
CFB	ENSG00000244255	6	31895475	31919825	2.65E-05	3.09E-02	5.86E-04	5.31E-03	4.93E-02	5.77E-04	0
TRAF6	ENSG00000175104	11	36508577	36531822	7.57E-02	1.33E-02	8.02E-04	2.16E-01	7.01E-03	5.92E-04	0
ARAP2	ENSG00000047365	4	35949843	36246131	3.68E-04	1.42E-01	4.54E-05	1.34E-01	2.45E-02	6.04E-04	0
HSF1	ENSG00000185122	8	145515280	145538385	1.48E-04	1.51E-03	3.91E-05	2.02E-02	4.29E-02	6.13E-04	0

Gene	Ensembl ID	CHR	START	STOP	VHD_CD Pleiotropy P	VHD_GORD Pleiotropy P	VHD_IBD Pleiotropy P	VHD_IBS Pleiotropy P	VHD_PUD Pleiotropy P	VHD_UC Pleiotropy P	d
WRB	ENSG00000182093	21	40752170	40800454	4.66E-03	5.69E-02	2.49E-04	4.24E-02	1.10E-04	6.36E-04	0
COL15A1	ENSG00000204291	9	101705461	101833069	1.15E-01	7.97E-02	1.99E-02	2.11E-04	1.61E-03	6.41E-04	0
MST1R	ENSG00000164078	3	49924435	49941299	4.26E-03	8.04E-03	4.15E-04	4.10E-01	2.75E-01	6.57E-04	0
RTEL1	ENSG00000258366	20	62289163	62328416	1.48E-03	1.46E-01	1.22E-04	8.47E-01	3.10E-01	6.88E-04	0
LST1	ENSG00000204482	6	31553901	31556686	7.43E-03	2.24E-01	3.15E-03	5.57E-02	2.66E-02	6.99E-04	0
GGT7	ENSG00000131067	20	33432523	33460663	5.04E-01	1.17E-02	4.64E-02	5.06E-02	1.86E-02	7.00E-04	0
NUP133	ENSG00000069248	1	229577045	229644103	3.96E-04	6.36E-02	5.65E-05	2.89E-03	3.10E-01	7.07E-04	0
CSNK2B	ENSG00000204435	6	31633013	31638120	1.18E-05	2.16E-05	7.46E-05	3.01E-05	8.30E-03	7.07E-04	0
GCK	ENSG00000106633	7	44183872	44237769	4.59E-03	4.29E-01	3.99E-04	2.68E-02	4.03E-02	7.08E-04	0
PEX13	ENSG00000162928	2	61244360	61279125	1.00E-04	2.04E-01	2.00E-05	1.29E-01	1.30E-01	7.14E-04	0
RPLP2	ENSG00000177600	11	809647	812880	9.91E-02	2.85E-01	3.97E-03	1.06E-02	3.39E-02	7.18E-04	0
PTK2	ENSG00000169398	8	141667999	142012315	1.52E-01	1.18E-01	2.99E-03	4.12E-03	3.96E-02	7.41E-04	0
SESN1	ENSG00000080546	6	109307640	109416022	7.55E-03	1.66E-02	3.92E-04	4.02E-03	1.04E-01	7.47E-04	0
STAT5A	ENSG00000126561	17	40439565	40463961	1.36E-04	2.51E-01	5.26E-05	1.28E-02	9.99E-02	7.66E-04	0
GCAT	ENSG00000100116	22	38203912	38213183	4.13E-02	5.97E-02	2.20E-02	6.98E-02	3.49E-01	8.35E-04	0
KLHL25	ENSG00000183655	15	86302554	86338261	8.02E-04	1.36E-01	6.95E-03	4.55E-03	7.47E-02	8.62E-04	0
DDIT3	ENSG00000175197	12	57910371	57914300	6.45E-02	3.69E-01	6.90E-04	4.36E-01	2.36E-01	9.07E-04	0
FAM213B	ENSG00000157870	1	2517930	2522908	8.29E-02	6.96E-01	1.47E-02	6.31E-01	8.41E-01	9.10E-04	0
THADA	ENSG00000115970	2	43393800	43823185	1.08E-05	5.73E-03	9.29E-06	2.30E-01	8.00E-03	9.12E-04	0
MSX2	ENSG00000120149	5	174151536	174157896	1.56E-03	2.68E-01	8.05E-04	1.46E-03	1.65E-01	9.25E-04	0
CYP4V2	ENSG00000145476	4	187112674	187134610	2.14E-01	4.97E-02	7.47E-03	3.48E-02	1.04E-01	9.34E-04	0
SLC30A7	ENSG00000162695	1	101361632	101447309	5.16E-01	1.93E-01	5.02E-03	1.40E-01	2.89E-01	9.36E-04	0
PRDM4	ENSG00000110851	12	108126643	108155049	7.50E-02	1.27E-02	2.94E-03	2.66E-01	2.65E-02	9.68E-04	0
RAPGEF5	ENSG00000136237	7	22157856	22396763	3.57E-01	3.15E-02	1.66E-02	7.20E-02	3.19E-03	9.77E-04	0
PSMD3	ENSG00000108344	17	38137050	38154213	6.53E-03	4.12E-01	9.68E-04	2.40E-01	5.64E-02	9.82E-04	0
SLC25A22	ENSG00000177542	11	790475	798316	6.83E-02	9.31E-02	4.17E-03	4.43E-02	1.12E-02	9.98E-04	0
ILF2	ENSG00000143621	1	153634512	153643524	2.55E-02	2.52E-05	9.77E-02	1.81E-04	3.68E-02	1.00E-03	0
DAGLB	ENSG00000164535	7	6448757	6523821	6.46E-02	5.58E-01	3.54E-03	5.75E-01	7.20E-01	1.02E-03	0
GPANK1	ENSG00000204438	6	31629006	31634060	1.45E-04	5.75E-04	2.51E-04	6.93E-04	1.27E-02	1.02E-03	0
UCN2	ENSG00000145040	3	48599160	48601206	9.11E-04	1.11E-03	6.71E-04	2.50E-03	3.94E-01	1.02E-03	0
PDDC1	ENSG00000177225	11	767220	777488	5.08E-02	1.45E-01	2.76E-03	2.05E-02	1.35E-02	1.05E-03	0
NFKB1	ENSG00000109320	4	103422486	103538459	2.10E-04	2.54E-01	3.18E-01	4.07E-01	2.39E-01	1.06E-03	0
CABP1	ENSG00000157782	12	121078355	121105127	1.89E-01	8.83E-03	1.63E-02	1.25E-01	1.03E-01	1.07E-03	0
HMGXB4	ENSG00000100281	22	35653445	35691800	4.39E-02	5.05E-02	1.76E-03	9.29E-02	2.86E-01	1.08E-03	0
TOM1	ENSG00000100284	22	35695268	35743985	1.27E-01	3.92E-02	3.14E-03	1.06E-01	3.95E-01	1.10E-03	0
PNPO	ENSG00000108439	17	46018872	46025654	1.29E-03	3.47E-01	2.76E-04	7.73E-03	5.78E-01	1.11E-03	0
ZNF442	ENSG00000198342	19	12460185	12476719	3.49E-02	1.06E-01	1.39E-03	1.93E-01	6.07E-02	1.11E-03	0
HAUS8	ENSG00000131351	19	17160539	17186435	2.88E-03	4.63E-03	7.59E-04	4.00E-05	1.22E-02	1.12E-03	0
DCTN2	ENSG00000175203	12	57923885	57941114	1.10E-01	4.83E-01	1.05E-03	3.18E-01	2.85E-01	1.13E-03	0
TRIM10	ENSG00000204613	6	30119722	30128711	7.54E-04	3.84E-03	4.44E-02	1.59E-02	5.45E-02	1.15E-03	0
MBD6	ENSG00000166987	12	57914493	57923931	8.37E-02	3.84E-01	1.01E-03	3.87E-01	2.05E-01	1.18E-03	0
JAK2	ENSG00000096968	9	4985033	5128183	9.43E-04	2.88E-01	9.69E-03	4.84E-01	2.10E-01	1.18E-03	0
C2orf74	ENSG00000237651	2	61372243	61391964	1.83E-03	1.03E-02	2.49E-04	1.13E-01	3.74E-01	1.21E-03	0
UQCRC1	ENSG00000010256	3	48636435	48648409	1.24E-03	3.59E-02	2.95E-04	1.49E-02	4.99E-01	1.21E-03	0
TTC34	ENSG00000215912	1	2567415	2718286	3.91E-01	4.42E-01	1.27E-02	3.74E-01	4.58E-01	1.22E-03	0
CTXN3	ENSG00000205279	5	126984736	126994322	5.70E-01	2.19E-01	3.77E-03	4.29E-01	6.19E-01	1.27E-03	0
ELL2	ENSG00000118985	5	95220802	95297775	3.32E-03	4.13E-01	3.04E-04	1.32E-01	7.55E-02	1.31E-03	0
MIIP	ENSG00000116691	1	12079523	12092102	1.77E-04	4.39E-01	1.60E-04	4.34E-01	1.17E-02	1.33E-03	0
CARD9	ENSG00000187796	9	139256355	139268133	4.39E-03	4.37E-01	2.48E-02	4.47E-01	6.13E-01	1.35E-03	0

Gene	Ensembl ID	CHR	START	STOP	VHD_CD Pleiotropy P	VHD_GORD Pleiotropy P	VHD_IBD Pleiotropy P	VHD_IBS Pleiotropy P	VHD_PUD Pleiotropy P	VHD_UC Pleiotropy P	d
FAM198B	ENSG00000164125	4	159045626	159094470	5.19E-02	9.16E-01	3.90E-03	1.31E-01	1.04E-01	1.35E-03	0
TCHH	ENSG00000159450	1	152078793	152086556	4.80E-01	4.12E-01	3.02E-02	3.92E-02	4.41E-03	1.37E-03	0
CYP19A1	ENSG00000137869	15	51500254	51630807	3.91E-01	3.11E-03	6.78E-02	3.38E-01	7.64E-02	1.37E-03	0
ZBTB46	ENSG00000130584	20	62375019	62462597	7.42E-04	1.03E-03	2.14E-04	1.06E-01	1.22E-01	1.38E-03	0
ARIH2	ENSG00000177479	3	48956254	49023815	1.42E-03	1.70E-01	1.95E-04	1.36E-01	5.11E-01	1.41E-03	0
SLC4A10	ENSG00000144290	2	162280843	162841792	4.24E-02	2.60E-02	4.64E-01	6.40E-03	1.71E-01	1.47E-03	0
NUCKS1	ENSG00000069275	1	205681947	205719404	1.56E-02	2.10E-01	1.01E-03	1.36E-01	1.02E-04	1.49E-03	0
ZMAT5	ENSG00000100319	22	30126945	30163000	1.02E-02	2.61E-01	3.15E-03	6.76E-03	1.15E-01	1.53E-03	0
C6orf47	ENSG00000204439	6	31626075	31628549	1.44E-03	5.85E-02	2.55E-01	5.20E-02	1.61E-01	1.54E-03	0
CAMK2A	ENSG00000070808	5	149599054	149669854	5.10E-02	4.59E-01	1.80E-03	2.33E-01	1.43E-01	1.72E-03	0
CES2	ENSG00000172831	16	66968347	66978999	7.00E-02	7.59E-01	2.47E-03	3.22E-01	3.06E-01	1.75E-03	0
MRM1	ENSG00000129282	17	34958001	34965407	2.93E-01	5.98E-02	9.16E-03	7.01E-03	1.09E-01	1.85E-03	0
ITPKC	ENSG00000086544	19	41223008	41246765	7.92E-05	7.08E-01	3.60E-05	4.62E-01	1.87E-01	1.88E-03	0
RFPL1	ENSG00000128250	22	29834572	29838444	1.85E-02	5.63E-01	1.57E-03	6.99E-02	1.89E-01	1.90E-03	0
SNAPC4	ENSG00000165684	9	139270029	139293249	1.96E-03	7.06E-01	9.88E-04	5.67E-01	5.37E-01	1.92E-03	0
WDR5B	ENSG00000196981	3	122130715	122134882	4.03E-01	4.33E-03	2.53E-02	1.86E-02	8.49E-01	1.97E-03	0
NPRL2	ENSG00000114388	3	50384761	50388522	8.88E-03	2.26E-01	1.85E-03	1.26E-01	4.27E-02	1.99E-03	0
HOXA11	ENSG00000005073	7	27221129	27224842	2.72E-01	4.53E-01	7.17E-03	2.54E-01	2.19E-02	2.00E-03	0
HNF4A	ENSG00000101076	20	42984340	43061485	3.13E-03	6.70E-02	3.92E-04	3.43E-02	3.30E-03	2.00E-03	0
MLF1	ENSG00000178053	3	158288952	158325041	6.81E-03	1.99E-02	5.46E-04	1.51E-03	4.16E-01	2.03E-03	0
CDKN2D	ENSG00000129355	19	10677138	10679735	1.98E-03	9.11E-02	5.38E-04	8.33E-01	1.96E-01	2.04E-03	0
PPAP2C	ENSG00000141934	19	281040	291393	5.87E-02	3.14E-01	8.13E-03	4.34E-02	3.15E-02	2.05E-03	0
HIST1H2AH	ENSG00000184825	6	27114861	27115317	1.20E-01	8.41E-01	8.80E-03	1.86E-01	8.50E-01	2.09E-03	0
TNFRSF9	ENSG00000049249	1	7979907	8000926	2.46E-04	3.49E-01	8.94E-05	8.59E-01	4.57E-01	2.14E-03	0
OR5B2	ENSG00000172365	11	58189738	58190786	1.04E-01	1.73E-01	4.93E-03	3.00E-01	4.11E-01	2.19E-03	0
TALDO1	ENSG00000177156	11	747329	765024	6.42E-02	2.05E-01	3.86E-03	3.96E-02	1.11E-02	2.19E-03	0
GNRH2	ENSG00000125787	20	3024268	3026393	1.45E-01	1.75E-01	5.18E-03	5.43E-03	6.44E-02	2.23E-03	0
ATP1B3	ENSG00000069849	3	141594966	141645356	3.01E-01	1.66E-01	2.56E-02	1.37E-01	7.34E-01	2.27E-03	0
ARID5B	ENSG00000150347	10	63661059	63856703	5.15E-02	5.38E-02	3.38E-02	2.16E-03	8.42E-02	2.30E-03	0
COLEC10	ENSG00000184374	8	120007691	120118821	1.19E-01	5.39E-02	5.76E-03	3.81E-01	6.10E-02	2.34E-03	0
KPNA1	ENSG00000114030	3	122140796	122233792	6.19E-01	1.88E-02	3.04E-02	3.50E-02	7.90E-01	2.35E-03	0
DHRS11	ENSG00000108272	17	34948228	34957235	1.71E-01	2.25E-01	7.71E-03	8.70E-03	1.36E-01	2.35E-03	0
RILP	ENSG00000167705	17	1549444	1553371	1.69E-01	1.69E-01	4.71E-02	2.68E-02	8.33E-04	2.35E-03	0
DCAKD	ENSG00000172992	17	43100708	43138473	1.21E-02	1.88E-03	3.23E-03	7.10E-03	5.18E-02	2.38E-03	0
GALNT10	ENSG00000164574	5	153570290	153800544	5.52E-01	2.87E-03	1.45E-02	2.22E-02	2.05E-02	2.39E-03	0
PLCG2	ENSG00000197943	16	81772702	81991899	2.81E-02	2.32E-01	7.55E-04	6.24E-01	6.82E-02	2.39E-03	0
RBM5	ENSG00000003756	3	50126341	50156454	8.83E-03	2.05E-02	1.56E-03	3.72E-01	5.79E-01	2.39E-03	0
TRAF3IP3	ENSG00000009790	1	209929377	209955668	1.82E-01	2.35E-01	1.82E-02	1.15E-01	2.15E-01	2.39E-03	0
HSPA1A	ENSG00000204389	6	31783291	31785723	1.04E-01	2.32E-01	4.97E-03	2.05E-01	3.33E-01	2.41E-03	0
ATAT1	ENSG00000137343	6	30594619	30614600	5.70E-04	2.10E-04	2.40E-04	7.99E-03	1.25E-01	2.44E-03	0
MPV17	ENSG00000115204	2	27532360	27548547	2.11E-01	2.47E-01	8.98E-03	1.20E-05	1.44E-01	2.48E-03	0
ITGAV	ENSG00000138448	2	187454792	187545628	1.26E-03	2.30E-01	4.08E-04	1.68E-01	1.69E-01	2.52E-03	0
YEATS4	ENSG00000127337	12	69753483	69784576	6.16E-03	3.00E-01	1.20E-03	8.20E-03	4.88E-02	2.54E-03	0
SCAMP3	ENSG00000116521	1	155225770	155232221	1.87E-03	4.64E-02	7.01E-04	2.25E-02	1.48E-01	2.60E-03	0
NR2F2	ENSG00000185551	15	96869167	96883492	1.13E-01	1.52E-01	1.24E-02	2.85E-01	8.38E-01	2.62E-03	0
MARS	ENSG00000166986	12	57869228	57911352	1.99E-01	4.83E-01	2.70E-03	2.96E-01	2.38E-01	2.65E-03	0
TNFRSF25	ENSG00000215788	1	6521211	6526255	1.93E-01	6.27E-01	3.59E-03	7.98E-01	6.17E-01	2.65E-03	0
PKIG	ENSG00000168734	20	43160426	43252888	2.25E-02	5.83E-01	3.55E-03	5.67E-01	3.81E-02	2.66E-03	0
ZFHX3	ENSG00000140836	16	72816784	73093597	4.04E-01	7.72E-04	6.94E-03	7.83E-02	3.21E-04	2.69E-03	0

Gene	Ensembl ID	CHR	START	STOP	VHD_CD Pleiotropy P	VHD_GORD Pleiotropy P	VHD_IBD Pleiotropy P	VHD_IBS Pleiotropy P	VHD_PUD Pleiotropy P	VHD_UC Pleiotropy P	d
TMCO4	ENSG00000162542	1	20008706	20126438	2.76E-01	7.93E-01	2.73E-02	4.04E-01	9.70E-01	2.69E-03	0
PCDHGC5	ENSG00000240764	5	140868808	140892546	3.33E-04	1.71E-03	3.00E-04	2.24E-01	5.50E-02	2.69E-03	0
DTD1	ENSG00000125821	20	18568537	18744561	3.57E-02	1.21E-03	2.74E-03	2.18E-03	4.24E-02	2.70E-03	0
TNF	ENSG00000232810	6	31543344	31546113	4.19E-03	9.44E-02	4.90E-02	6.33E-02	7.74E-02	2.71E-03	0
OR2H2	ENSG00000204657	6	29555683	29556745	7.21E-03	1.00E-02	1.94E-03	3.49E-01	9.44E-02	2.72E-03	0
ANXA9	ENSG00000143412	1	150954493	150968110	1.44E-02	1.17E-02	1.10E-03	6.63E-04	1.27E-02	2.72E-03	0
GGNBP2	ENSG00000005955	17	34900737	34946278	2.60E-01	2.94E-01	1.11E-02	6.31E-03	1.64E-01	2.73E-03	0
PWP1	ENSG00000136045	12	108079509	108106944	1.16E-01	4.05E-02	6.99E-03	3.50E-01	2.85E-02	2.73E-03	0
TRIM40	ENSG00000204614	6	30103885	30116512	5.28E-02	1.02E-01	1.59E-01	2.34E-02	1.63E-01	2.76E-03	0
NCR3	ENSG00000204475	6	31556672	31560762	4.66E-05	5.91E-04	4.30E-05	7.32E-03	1.18E-01	2.82E-03	0
RPP21	ENSG00000241370	6	30312908	30314661	1.59E-01	6.43E-02	1.12E-01	8.62E-02	2.16E-01	2.87E-03	0
TMEM88	ENSG00000167874	17	7758383	7759417	7.54E-03	4.29E-01	1.50E-02	1.48E-01	3.65E-01	2.88E-03	0
HOXA13	ENSG00000106031	7	27233122	27239725	6.40E-01	4.98E-01	3.43E-02	6.34E-02	2.16E-02	2.92E-03	0
C16orf59	ENSG00000162062	16	2510081	2514964	6.50E-02	7.59E-01	2.14E-01	7.90E-01	7.74E-01	2.94E-03	0
CCDC101	ENSG00000176476	16	28565236	28603111	1.72E-04	4.28E-01	7.73E-05	8.27E-02	2.26E-01	2.99E-03	0
AP1M2	ENSG00000129354	19	10683347	10697991	5.65E-04	4.06E-01	2.28E-04	7.53E-01	1.43E-01	3.11E-03	0
TRIOBP	ENSG00000100106	22	38093011	38172563	9.86E-02	5.66E-02	2.85E-01	3.19E-01	1.22E-01	3.13E-03	0
ACBD3	ENSG00000182827	1	226332380	226374431	1.06E-02	8.20E-02	3.35E-03	6.42E-01	1.49E-02	3.14E-03	0
GPR132	ENSG00000183484	14	105515728	105531782	3.91E-02	2.49E-01	1.96E-03	2.26E-01	1.46E-01	3.14E-03	0
ZFP91	ENSG00000186660	11	58346584	58388515	6.99E-02	9.32E-02	3.56E-03	4.42E-01	7.11E-02	3.16E-03	0
TSPAN4	ENSG00000214063	11	842808	867116	2.77E-02	1.22E-01	6.68E-03	5.30E-02	1.96E-03	3.18E-03	0
C6orf100	ENSG00000204709	6	28911654	28912314	7.99E-04	5.28E-02	5.37E-01	3.81E-01	2.11E-02	3.23E-03	0
APOBR	ENSG00000184730	16	28505970	28510291	2.93E-04	4.82E-01	1.54E-04	4.80E-02	2.22E-01	3.24E-03	0
CELA2A	ENSG00000142615	1	15783223	15798586	1.19E-02	4.29E-01	2.02E-03	5.62E-02	4.29E-02	3.25E-03	0
PANK4	ENSG00000157881	1	2439972	2458039	1.05E-01	8.91E-01	5.61E-02	1.42E-01	2.54E-01	3.34E-03	0
HMGA2	ENSG00000149948	12	66217911	66360075	6.66E-02	7.72E-02	3.83E-02	2.21E-03	2.44E-02	3.46E-03	0
ZNF831	ENSG00000124203	20	57766075	57834168	3.26E-03	4.34E-01	4.44E-04	2.75E-02	1.16E-01	3.52E-03	0
HIRIP3	ENSG00000149929	16	30003645	30007757	5.33E-01	2.70E-02	1.21E-02	4.68E-01	6.23E-01	3.56E-03	0
ERRFI1	ENSG00000116285	1	8064464	8086368	2.35E-04	8.73E-02	1.42E-04	1.32E-01	3.27E-01	3.62E-03	0
LCE4A	ENSG00000187170	1	152680636	152681967	2.09E-01	5.90E-01	2.26E-02	3.23E-01	1.59E-01	3.67E-03	0
ORC4	ENSG00000115947	2	148687968	148779147	2.34E-03	2.76E-01	8.45E-04	5.70E-01	6.42E-01	3.70E-03	0
PLEKHG4B	ENSG00000153404	5	140373	190085	2.50E-02	1.60E-02	3.98E-03	6.38E-01	1.18E-01	3.72E-03	0
LIN28A	ENSG00000131914	1	26737269	26756213	8.27E-03	1.99E-01	1.24E-03	2.08E-01	3.81E-01	3.72E-03	0
GPR65	ENSG00000140030	14	88471468	88478419	1.77E-04	3.45E-01	7.68E-04	4.78E-01	2.78E-01	3.73E-03	0
LCE2B	ENSG00000159455	1	152647771	152659877	1.19E-01	6.88E-01	2.08E-02	8.80E-01	6.65E-01	3.73E-03	0
HLA-B	ENSG00000234745	6	31321649	31324965	1.37E-04	7.75E-03	1.64E-04	4.78E-02	3.15E-01	3.75E-03	0
GPR88	ENSG00000181656	1	101003693	101007574	2.00E-03	1.85E-01	1.01E-03	5.57E-01	4.58E-01	3.76E-03	0
HSD11B1	ENSG00000117594	1	209859510	209908295	7.37E-01	3.44E-01	3.76E-02	2.33E-01	1.38E-01	3.78E-03	0
BICC1	ENSG00000122870	10	60272900	60591195	2.48E-01	1.34E-03	7.66E-03	1.49E-03	7.13E-02	3.84E-03	0
PEX26	ENSG00000215193	22	18560689	18613905	6.46E-02	4.02E-01	1.15E-01	1.77E-01	1.29E-03	3.87E-03	0
PCDHGC4	ENSG00000242419	5	140864741	140892546	3.99E-04	2.05E-03	4.19E-04	2.18E-01	6.25E-02	3.88E-03	0
GTF2I	ENSG00000077809	7	74071994	74175026	2.84E-03	2.92E-02	9.51E-04	1.94E-02	8.91E-02	3.89E-03	0
MAPKAPK2	ENSG00000162889	1	206858289	206907628	4.51E-02	4.17E-01	3.08E-03	2.20E-01	1.73E-01	3.92E-03	0
AIF1	ENSG00000204472	6	31582961	31584798	8.88E-02	9.40E-02	2.33E-02	1.43E-02	2.89E-01	3.93E-03	0
HDAC7	ENSG00000061273	12	48176505	48226915	1.36E-01	1.35E-01	9.01E-03	6.98E-01	4.08E-01	3.97E-03	0
LCE1E	ENSG00000186226	1	152758690	152760902	7.67E-03	3.93E-01	3.37E-03	1.31E-01	3.59E-01	4.01E-03	0
EVX1	ENSG00000106038	7	27282164	27290112	4.34E-01	8.52E-02	1.08E-01	3.41E-01	1.51E-01	4.01E-03	0
FANCD2	ENSG00000144554	3	10068098	10143614	1.84E-01	1.61E-01	1.42E-02	1.42E-01	3.27E-01	4.03E-03	0
ZSCAN23	ENSG00000187987	6	28399707	28411279	2.73E-01	3.42E-01	2.21E-02	7.07E-01	2.67E-01	4.04E-03	0

Gene	Ensembl ID	CHR	START	STOP	VHD_CD Pleiotropy P	VHD_GORD Pleiotropy P	VHD_IBD Pleiotropy P	VHD_IBS Pleiotropy P	VHD_PUD Pleiotropy P	VHD_UC Pleiotropy P	d
ADCY7	ENSG00000121281	16	50280048	50352046	9.61E-06	2.08E-01	7.93E-04	2.43E-01	4.92E-01	4.08E-03	0
SLC6A5	ENSG00000165970	11	20620946	20680831	4.05E-01	6.36E-01	1.18E-02	8.06E-01	3.34E-01	4.09E-03	0
MAP3K8	ENSG00000107968	10	30722866	30750762	5.36E-04	2.48E-01	2.04E-04	1.68E-01	6.35E-01	4.11E-03	0
DPCR1	ENSG00000168631	6	30908749	30921998	2.25E-05	7.86E-06	5.82E-04	2.52E-04	4.36E-03	4.14E-03	0
REL	ENSG00000162924	2	61108656	61158745	1.43E-03	6.16E-01	4.13E-04	3.47E-01	8.53E-01	4.15E-03	0
ARHGAP10	ENSG00000071205	4	148653214	148993931	9.11E-04	2.32E-02	4.09E-04	4.02E-02	9.65E-02	4.20E-03	0
LRRC71	ENSG00000160838	1	156890442	156902886	1.71E-02	3.06E-01	1.91E-03	6.80E-01	1.05E-01	4.28E-03	0
HEMGN	ENSG00000136929	9	100689073	100707138	1.50E-01	6.29E-02	7.73E-02	5.22E-01	1.36E-01	4.30E-03	0
ABCB10	ENSG00000135776	1	229652329	229694442	3.04E-03	8.90E-02	5.36E-04	7.33E-03	2.84E-01	4.34E-03	0
AEN	ENSG00000181026	15	89164527	89175513	2.44E-02	8.32E-02	3.41E-03	4.72E-02	4.48E-02	4.37E-03	0
P4HTM	ENSG00000178467	3	49027319	49044587	1.19E-02	2.37E-01	1.45E-03	2.49E-01	6.86E-01	4.37E-03	0
FAM109A	ENSG00000198324	12	111798455	111806925	9.55E-03	1.23E-01	2.10E-03	1.08E-01	4.29E-02	4.38E-03	0
WASF1	ENSG00000112290	6	110421022	110501207	8.33E-01	1.84E-01	2.88E-02	4.75E-01	1.47E-01	4.40E-03	0
CYP4F22	ENSG00000171954	19	15619304	15663128	9.37E-02	1.67E-01	7.47E-03	3.04E-01	2.06E-01	4.49E-03	0
GNAT1	ENSG00000114349	3	50229045	50233949	4.42E-02	1.74E-02	5.24E-03	2.24E-01	1.37E-01	4.50E-03	0
SMG7	ENSG00000116698	1	183441351	183567381	5.48E-01	1.76E-01	6.20E-02	5.58E-01	3.80E-01	4.55E-03	0
METTL11B	ENSG00000203740	1	170115142	170136931	9.40E-02	5.98E-04	1.01E-02	2.06E-01	1.23E-02	4.56E-03	0
PCDHGC3	ENSG00000240184	5	140855580	140892542	7.78E-04	3.00E-03	5.03E-04	1.94E-01	9.67E-02	4.58E-03	0
NFKBIA	ENSG00000100906	14	35870717	35873955	1.09E-01	2.53E-01	1.08E-02	5.77E-01	3.84E-01	4.60E-03	0
PTPRJ	ENSG00000149177	11	48002113	48189670	5.05E-04	6.84E-03	2.79E-04	7.11E-02	4.21E-01	4.60E-03	0
TUBA8	ENSG00000183785	22	18593097	18629321	2.15E-01	4.57E-01	1.42E-02	6.40E-02	8.50E-03	4.63E-03	0
ATAD3C	ENSG00000215915	1	1385069	1405538	1.35E-02	5.94E-01	3.57E-03	6.64E-01	6.30E-01	4.67E-03	0
GP9	ENSG00000169704	3	128779610	128781249	1.65E-01	3.45E-02	9.27E-03	1.85E-01	1.44E-01	4.70E-03	0
CNTNAP1	ENSG00000108797	17	40834631	40851832	2.26E-02	9.26E-01	3.57E-03	6.10E-01	4.59E-01	4.71E-03	0
USP19	ENSG00000172046	3	49145479	49158371	1.55E-03	5.60E-02	4.78E-04	5.95E-02	1.83E-01	4.71E-03	0
TRIM54	ENSG00000138100	2	27505260	27530307	2.25E-01	1.02E-01	1.12E-02	4.34E-05	3.15E-01	4.76E-03	0
ZNF428	ENSG00000131116	19	44111371	44124026	9.71E-01	4.04E-01	3.01E-02	5.21E-02	6.36E-02	4.80E-03	0
TMEM200A	ENSG00000164484	6	130686879	130764208	7.03E-02	2.56E-01	2.13E-02	4.20E-01	3.10E-01	4.81E-03	0
METTL18	ENSG00000171806	1	169761670	169764107	7.07E-02	1.02E-01	7.24E-03	2.45E-01	2.65E-01	4.81E-03	0
HNF1A	ENSG00000135100	12	121416346	121440315	1.84E-01	1.53E-01	3.90E-02	1.33E-01	2.43E-01	4.92E-03	0
DIMT1	ENSG00000086189	5	61683081	61699766	1.35E-01	9.00E-02	4.14E-03	3.30E-02	5.04E-02	4.95E-03	0
TBX21	ENSG00000073861	17	45810610	45823485	7.22E-03	5.97E-02	1.94E-03	5.15E-02	3.50E-02	4.97E-03	0
CAMSAP2	ENSG00000118200	1	200708686	200829832	7.58E-02	6.19E-01	2.80E-03	3.52E-01	5.29E-01	5.00E-03	0
RPS21	ENSG00000171858	20	60962172	60963576	9.01E-02	1.58E-02	1.59E-02	5.61E-04	8.62E-03	5.00E-03	0
SOX9	ENSG00000125398	17	70117161	70122561	4.68E-01	6.32E-02	3.28E-02	2.30E-01	2.75E-01	5.04E-03	0
CEND1	ENSG00000184524	11	787104	790123	4.55E-02	2.91E-01	7.13E-03	8.62E-02	3.47E-02	5.08E-03	0
TSPAN14	ENSG00000108219	10	82213922	82292879	2.61E-03	5.79E-01	4.18E-04	1.06E-01	2.62E-02	5.08E-03	0
DLG5	ENSG00000151208	10	79550549	79686378	3.57E-04	9.73E-02	7.72E-04	9.82E-03	6.80E-03	5.11E-03	0
SULT1A2	ENSG00000197165	16	28603264	28608430	2.08E-04	4.14E-01	1.22E-04	6.84E-02	9.43E-02	5.14E-03	0
KIAA1598	ENSG00000187164	10	118643742	118886097	1.41E-02	1.39E-01	3.24E-03	6.78E-02	2.60E-01	5.16E-03	0
MED24	ENSG00000008838	17	38175350	38217468	2.16E-02	5.22E-01	4.52E-03	3.99E-01	2.24E-01	5.19E-03	0
SLC25A20	ENSG00000178537	3	48894369	48936426	1.60E-03	8.24E-02	5.05E-04	1.01E-01	4.81E-01	5.19E-03	0
VDR	ENSG00000111424	12	48235320	48336831	1.64E-01	6.49E-01	1.13E-02	1.28E-01	1.97E-01	5.22E-03	0
C9orf3	ENSG00000148120	9	97488983	97849441	1.36E-01	4.55E-02	1.77E-02	1.61E-01	2.91E-02	5.25E-03	0
CDC37	ENSG00000105401	19	10501810	10530797	6.28E-05	1.00E-01	8.82E-05	4.21E-01	6.92E-01	5.28E-03	0
CSH2	ENSG00000213218	17	61949372	61951126	9.46E-01	1.23E-01	9.83E-02	2.94E-01	5.52E-01	5.34E-03	0
KRTAP5-2	ENSG00000205867	11	1618409	1619524	3.52E-01	7.06E-02	2.47E-02	3.34E-01	2.61E-02	5.41E-03	0
PRELID1	ENSG00000169230	5	176730775	176733960	1.11E-01	1.56E-01	6.38E-03	3.38E-01	6.77E-02	5.41E-03	0
FHL5	ENSG00000112214	6	97010424	97064512	7.09E-02	2.87E-01	7.16E-03	2.73E-01	5.56E-01	5.42E-03	0

Gene	Ensembl ID	CHR	START	STOP	VHD_CD Pleiotropy P	VHD_GORD Pleiotropy P	VHD_IBD Pleiotropy P	VHD_IBS Pleiotropy P	VHD_PUD Pleiotropy P	VHD_UC Pleiotropy P	d
C19orf54	ENSG00000188493	19	41246761	41257458	2.99E-04	2.73E-01	1.04E-04	2.38E-01	3.23E-02	5.42E-03	0
CLDN9	ENSG00000213937	16	3062457	3064506	9.46E-02	4.10E-01	2.46E-01	4.37E-01	2.01E-01	5.43E-03	0
CDH16	ENSG00000166589	16	66942025	66952887	9.15E-02	6.01E-01	5.50E-03	8.17E-01	1.11E-01	5.45E-03	0
NOB1	ENSG00000141101	16	69775770	69788843	5.24E-01	7.37E-01	1.49E-02	7.12E-02	2.52E-01	5.48E-03	0
IL21	ENSG00000138684	4	123533783	123542224	7.79E-04	2.13E-01	3.27E-04	4.78E-02	4.54E-01	5.50E-03	0
KRTAP5-3	ENSG00000196224	11	1628795	1629693	6.70E-01	7.46E-02	8.33E-02	1.68E-02	1.28E-01	5.51E-03	0
RORC	ENSG00000143365	1	151778547	151804348	6.83E-03	6.55E-01	1.90E-03	4.29E-01	4.94E-01	5.51E-03	0
METTL10	ENSG00000203791	10	126436718	126480439	1.06E-02	7.75E-01	2.02E-03	9.39E-02	6.78E-01	5.54E-03	0
DUSP28	ENSG00000188542	2	241499471	241503431	1.59E-01	7.36E-01	1.20E-02	5.24E-01	8.19E-01	5.55E-03	0
PTCHD3	ENSG00000182077	10	27687116	27703297	6.50E-01	3.16E-02	2.30E-02	1.11E-01	5.54E-01	5.58E-03	0
LTA	ENSG00000226979	6	31539831	31542101	2.93E-05	3.52E-05	1.34E-02	1.17E-02	1.05E-03	5.62E-03	0
ZDHHC3	ENSG00000163812	3	44956749	45017677	5.48E-01	5.07E-01	1.79E-02	3.34E-01	3.56E-01	5.63E-03	0
NOL12	ENSG00000100101	22	38077680	38170137	1.48E-01	8.24E-02	3.63E-01	4.10E-01	1.32E-01	5.64E-03	0
PNPLA2	ENSG00000177666	11	818902	825573	3.60E-01	5.93E-01	2.38E-02	8.97E-02	3.86E-02	5.65E-03	0
GPR18	ENSG00000125245	13	99906968	99913998	3.88E-03	8.99E-02	5.06E-04	1.95E-01	4.16E-01	5.69E-03	0
GABBR1	ENSG00000204681	6	29523406	29601753	2.52E-03	4.35E-05	4.52E-03	4.04E-02	1.83E-01	5.74E-03	0
KDELR2	ENSG00000136240	7	6485584	6523873	3.81E-02	4.59E-01	6.78E-03	3.95E-01	6.59E-01	5.78E-03	0
ZFP36L2	ENSG00000152518	2	43449541	43453748	1.76E-03	2.76E-02	9.84E-04	4.68E-01	2.64E-02	5.80E-03	0
ARFRP1	ENSG00000101246	20	62329996	62339377	3.04E-03	1.43E-01	4.66E-04	6.39E-01	5.31E-01	5.81E-03	0
SLC6A7	ENSG00000011083	5	149569520	149602351	2.38E-02	3.20E-01	1.81E-03	7.03E-02	1.20E-01	5.87E-03	0
NCF2	ENSG00000116701	1	183524698	183560011	4.94E-01	1.72E-01	4.87E-02	3.86E-01	2.59E-01	5.94E-03	0
ORAOV1	ENSG00000149716	11	69467844	69490184	3.21E-02	7.72E-01	8.94E-03	2.10E-01	5.25E-01	6.10E-03	0
ZNF366	ENSG00000178175	5	71738479	71803554	6.39E-02	2.67E-01	1.26E-02	2.89E-01	2.10E-03	6.12E-03	0
HPGDS	ENSG00000163106	4	95219686	95264027	8.30E-03	5.89E-01	1.76E-03	7.50E-02	6.49E-01	6.14E-03	0
BRD7	ENSG00000166164	16	50347398	50402845	4.32E-05	1.53E-01	1.19E-04	5.61E-01	6.68E-01	6.21E-03	0
CLN3	ENSG00000261832	16	28477974	28503333	6.29E-04	8.75E-01	3.38E-04	1.17E-01	2.77E-01	6.21E-03	0
GPR35	ENSG00000178623	2	241544848	241570676	1.05E-01	2.67E-01	1.17E-02	7.90E-01	7.66E-01	6.22E-03	0
TRIM48	ENSG00000150244	11	55029658	55038595	9.38E-04	3.69E-01	1.13E-03	1.46E-01	8.80E-02	6.24E-03	0
PDE4A	ENSG00000065989	19	10527449	10580305	5.75E-05	1.03E-01	1.05E-04	4.02E-01	4.54E-01	6.28E-03	0
KRTCAP2	ENSG00000163463	1	155141884	155145951	3.45E-03	4.66E-02	1.10E-03	4.69E-01	2.12E-01	6.32E-03	0
MICA	ENSG00000204520	6	31371356	31383092	8.08E-06	4.88E-03	4.82E-04	4.70E-02	2.51E-03	6.33E-03	0
FCGR2B	ENSG00000072694	1	161551101	161648444	6.96E-02	9.73E-01	1.05E-02	1.57E-02	2.07E-01	6.34E-03	0
NOTCH1	ENSG00000148400	9	139388896	139440314	8.56E-03	8.88E-01	2.01E-03	5.91E-01	7.22E-01	6.40E-03	0
DUSP16	ENSG00000111266	12	12628829	12715317	1.30E-01	2.30E-01	1.40E-02	3.25E-01	2.41E-01	6.43E-03	0
RING1	ENSG00000204227	6	33176272	33180499	2.77E-02	1.16E-01	1.46E-01	2.79E-01	2.81E-01	6.50E-03	0
GRID2IP	ENSG00000215045	7	6537093	6591067	2.85E-02	7.52E-01	6.26E-03	5.84E-01	4.12E-01	6.64E-03	0
PRSS50	ENSG00000206549	3	46753605	46854064	2.30E-02	3.33E-02	5.09E-03	4.48E-01	1.01E-01	6.68E-03	0
COL11A2	ENSG00000204248	6	33130458	33160276	2.73E-01	4.83E-01	5.73E-02	3.75E-01	6.20E-01	6.69E-03	0
PRTN3	ENSG00000196415	19	840963	848175	2.36E-01	3.24E-02	3.87E-02	3.82E-01	3.44E-03	6.69E-03	0
TMEM81	ENSG00000174529	1	205052258	205053645	1.27E-01	4.03E-01	1.04E-02	5.38E-02	2.52E-01	6.69E-03	0
LMAN2	ENSG00000169223	5	176758563	176778853	7.31E-02	1.17E-01	7.75E-03	1.82E-01	8.94E-02	6.71E-03	0
RBM6	ENSG00000004534	3	49977440	50137478	2.48E-02	6.51E-02	4.43E-03	5.10E-01	5.44E-01	6.76E-03	0
ARHGAP9	ENSG00000123329	12	57866038	57882597	4.35E-01	6.62E-01	9.52E-03	3.28E-01	4.07E-01	6.82E-03	0
FES	ENSG00000182511	15	91426925	91439006	6.82E-05	6.22E-02	5.96E-05	1.58E-01	1.61E-01	6.85E-03	0
HSPA1B	ENSG00000204388	6	31795512	31798031	1.67E-03	1.64E-01	1.09E-01	4.51E-01	1.75E-02	6.88E-03	0
USP34	ENSG00000115464	2	61414598	61697904	3.56E-02	2.13E-02	3.92E-03	3.36E-01	7.20E-01	6.95E-03	0
MAPK8	ENSG00000107643	10	49514698	49647403	2.16E-03	4.83E-01	1.18E-03	8.63E-02	1.41E-01	6.96E-03	0
CTRC	ENSG00000162438	1	15764935	15775737	8.48E-03	2.15E-01	4.81E-04	6.16E-01	2.15E-02	6.98E-03	0
BRK1	ENSG00000254999	3	10157276	10168874	8.62E-02	9.42E-02	1.82E-02	2.13E-01	5.43E-01	6.98E-03	0

Gene	Ensembl ID	CHR	START	STOP	VHD_CD Pleiotropy P	VHD_GORD Pleiotropy P	VHD_IBD Pleiotropy P	VHD_IBS Pleiotropy P	VHD_PUD Pleiotropy P	VHD_UC Pleiotropy P	d
HORMAD1	ENSG00000143452	1	150670536	150693364	8.10E-03	1.60E-01	2.12E-03	2.40E-01	3.55E-01	7.02E-03	0
MBD5	ENSG00000204406	2	148778580	149275805	1.76E-03	6.15E-02	8.36E-04	7.29E-01	7.32E-02	7.09E-03	0
NKX2-3	ENSG00000119919	10	101292690	101296278	1.21E-03	5.86E-01	3.70E-02	4.96E-01	6.76E-02	7.09E-03	0
C17orf105	ENSG00000231256	17	41857803	41864980	2.11E-01	2.89E-02	9.12E-03	5.67E-03	2.13E-01	7.10E-03	0
SLC26A10	ENSG00000135502	12	58013310	58019934	1.78E-01	3.59E-01	4.87E-03	5.46E-03	2.03E-01	7.11E-03	0
SAMSN1	ENSG00000155307	21	15857549	15955723	7.14E-02	3.97E-01	9.18E-03	8.79E-01	4.39E-01	7.21E-03	0
FAM171B	ENSG00000144369	2	187558698	187630685	2.88E-03	2.38E-01	1.10E-03	9.77E-02	3.70E-01	7.26E-03	0
FAM96B	ENSG00000166595	16	66965959	66968326	1.13E-01	6.91E-01	4.50E-02	1.97E-01	1.63E-01	7.31E-03	0
ATP6V0A1	ENSG00000033627	17	40610862	40674629	1.20E-02	7.92E-01	3.51E-03	4.08E-01	4.04E-01	7.33E-03	0
CDCA4	ENSG00000170779	14	105475910	105487485	4.20E-02	4.23E-02	2.66E-03	6.83E-01	1.23E-01	7.33E-03	0
USP4	ENSG00000114316	3	49315264	49378145	3.75E-03	6.25E-01	1.63E-03	6.87E-01	6.24E-01	7.36E-03	0
SLC35D1	ENSG00000116704	1	67465015	67519782	3.12E-03	2.73E-02	5.91E-02	8.11E-01	1.58E-01	7.38E-03	0
TBR1	ENSG00000136535	2	162272605	162282381	2.92E-01	4.31E-02	3.31E-01	5.11E-04	2.18E-02	7.39E-03	0
INO80E	ENSG00000169592	16	30006615	30017114	6.63E-01	6.18E-02	2.55E-02	7.49E-01	8.42E-01	7.39E-03	0
CCDC124	ENSG00000007080	19	18043825	18054800	3.00E-01	5.57E-01	5.01E-02	9.44E-02	1.28E-01	7.40E-03	0
CD1D	ENSG00000158473	1	158149737	158154686	4.40E-02	3.95E-01	1.38E-02	9.69E-01	1.77E-01	7.52E-03	0
TNFRSF6B	ENSG00000243509	20	62328021	62330037	1.74E-04	3.95E-01	1.08E-04	9.20E-01	6.19E-01	7.54E-03	0
ZNF430	ENSG00000118620	19	21203426	21242856	7.71E-01	2.35E-01	9.70E-02	4.75E-01	1.59E-01	7.63E-03	0
EXOC5	ENSG00000070367	14	57670518	57735726	2.16E-01	1.71E-01	8.93E-02	5.44E-01	2.37E-01	7.65E-03	0
EXOC3	ENSG00000180104	5	443273	472052	2.02E-01	1.81E-01	2.58E-02	2.49E-01	3.34E-01	7.67E-03	0
OR8J3	ENSG00000167822	11	55904247	55905194	2.04E-04	2.09E-01	3.32E-04	1.14E-01	7.42E-03	7.67E-03	0
TIGIT	ENSG00000181847	3	113995760	114029135	1.03E-02	1.55E-02	5.46E-03	6.18E-02	1.28E-01	7.67E-03	0
IL12B	ENSG00000113302	5	158741791	158757895	1.53E-02	2.68E-01	3.36E-03	3.99E-01	1.87E-01	7.69E-03	0
CADM4	ENSG00000105767	19	44126520	44143991	6.84E-01	1.11E-01	2.73E-02	1.17E-02	6.94E-02	7.70E-03	0
BAIAP2L1	ENSG00000006453	7	97920963	98030380	2.09E-01	7.24E-01	1.12E-01	7.96E-03	5.17E-02	7.74E-03	0
PDCL3	ENSG00000115539	2	101179152	101193197	9.32E-02	1.13E-02	3.62E-02	1.99E-02	6.10E-01	7.75E-03	0
DET1	ENSG00000140543	15	89054790	89089906	1.04E-01	3.29E-01	1.37E-02	6.89E-02	3.00E-01	7.75E-03	0
TRIM26	ENSG00000234127	6	30152232	30181204	3.93E-06	4.51E-06	2.30E-04	2.98E-05	6.97E-03	7.76E-03	0
C20orf202	ENSG00000215595	20	1184098	1188918	9.47E-02	1.65E-02	6.62E-02	6.20E-01	4.96E-03	7.81E-03	0
ZNF365	ENSG00000138311	10	64133951	64431771	7.92E-04	8.97E-01	4.18E-04	7.00E-01	7.84E-01	7.84E-03	0
RARRES3	ENSG00000133321	11	63304281	63313934	3.31E-01	4.08E-01	2.04E-02	6.01E-01	5.95E-01	7.86E-03	0
CSF3	ENSG00000108342	17	38171614	38174066	5.81E-03	5.66E-01	2.21E-03	4.47E-01	2.45E-01	7.90E-03	0
MYLPF	ENSG00000180209	16	30382255	30389312	2.84E-01	3.78E-03	7.02E-02	5.10E-02	2.16E-01	7.91E-03	0
OLIG2	ENSG00000205927	21	34398153	34401504	1.06E-02	4.02E-01	3.61E-03	3.22E-03	5.85E-03	8.00E-03	0
MMEL1	ENSG00000142606	1	2522078	2564481	2.02E-01	9.63E-01	8.67E-02	6.06E-01	8.64E-01	8.07E-03	0
RPH3A	ENSG00000089169	12	113008184	113336686	4.96E-04	2.00E-02	7.52E-05	6.66E-01	2.19E-01	8.07E-03	0
GHR	ENSG00000112964	5	42423879	42721979	5.54E-02	3.36E-01	9.09E-02	2.84E-02	3.12E-02	8.07E-03	0
FAM184A	ENSG00000111879	6	119280928	119470552	6.69E-01	2.97E-01	2.54E-01	2.83E-01	3.72E-01	8.07E-03	0
TRIM14	ENSG00000106785	9	100831557	100881494	3.54E-01	2.57E-01	2.34E-02	1.09E-01	8.65E-01	8.23E-03	0
PCDHGA12	ENSG00000253159	5	140810185	140892546	1.21E-02	3.07E-02	2.34E-03	3.24E-01	3.71E-01	8.24E-03	0
ZNF714	ENSG00000160352	19	21264965	21308073	8.45E-01	3.43E-01	1.20E-01	3.68E-01	1.21E-01	8.32E-03	0
PSMC1	ENSG00000100764	14	90722839	90738968	1.82E-01	4.17E-01	1.13E-01	6.10E-01	5.59E-02	8.33E-03	0
MAX	ENSG00000125952	14	65472892	65569413	1.67E-01	1.68E-01	4.90E-02	4.13E-01	1.38E-01	8.38E-03	0
TRMT61B	ENSG00000171103	2	29072687	29093167	1.42E-02	2.21E-02	3.28E-03	1.02E-02	3.63E-01	8.44E-03	0
OR52E8	ENSG00000183269	11	5877904	5878964	7.46E-02	7.48E-01	8.84E-02	2.79E-01	7.73E-01	8.47E-03	0
TLN2	ENSG00000171914	15	62682725	63136830	4.83E-01	4.54E-02	8.82E-02	4.85E-01	4.78E-01	8.47E-03	0
ZNF772	ENSG00000197128	19	57978031	57988938	1.74E-02	4.00E-01	2.63E-03	5.16E-01	4.72E-02	8.49E-03	0
ADAMTS7	ENSG00000136378	15	79051545	79103773	1.76E-01	9.76E-02	2.13E-02	5.69E-02	2.76E-02	8.53E-03	0
WDR6	ENSG00000178252	3	49044495	49053386	2.67E-02	3.97E-01	3.48E-03	2.62E-01	7.00E-01	8.53E-03	0

Gene	Ensembl ID	CHR	START	STOP	VHD_CD Pleiotropy P	VHD_GORD Pleiotropy P	VHD_IBD Pleiotropy P	VHD_IBS Pleiotropy P	VHD_PUD Pleiotropy P	VHD_UC Pleiotropy P	d
LRCH3	ENSG00000186001	3	197518097	197615307	2.77E-02	3.04E-01	9.89E-03	5.18E-01	1.38E-01	8.55E-03	0
C6orf136	ENSG00000204564	6	30614816	30620987	2.95E-02	9.95E-05	1.35E-02	1.15E-02	9.18E-02	8.56E-03	0
MICALL2	ENSG00000164877	7	1468101	1499138	1.62E-01	5.62E-02	3.34E-02	2.96E-02	1.31E-02	8.59E-03	0
DDX59	ENSG00000118197	1	200593024	200639097	8.29E-02	8.08E-01	4.57E-03	4.08E-01	4.56E-01	8.67E-03	0
FBXW12	ENSG00000164049	3	48413709	48442666	5.00E-01	1.65E-01	5.05E-02	5.00E-01	9.47E-03	8.70E-03	0
SEPP1	ENSG00000250722	5	42799982	42887494	3.00E-01	1.94E-01	2.57E-01	7.45E-02	2.25E-01	8.75E-03	0
ADCK3	ENSG00000163050	1	227085237	227175246	1.54E-01	8.85E-02	3.33E-02	8.96E-02	6.16E-01	8.75E-03	0
GNG8	ENSG00000167414	19	47137333	47137942	9.90E-05	8.91E-01	3.01E-04	4.18E-01	5.79E-02	8.83E-03	0
CCDC114	ENSG00000105479	19	48799714	48825151	3.80E-02	8.55E-02	4.16E-01	9.71E-02	2.02E-01	8.86E-03	0
FOLR2	ENSG00000165457	11	71927645	71932994	4.74E-02	1.33E-01	9.45E-03	1.04E-01	1.74E-01	8.92E-03	0
SLC30A3	ENSG00000115194	2	27476552	27498685	3.74E-01	4.86E-01	2.90E-02	2.26E-05	4.48E-01	8.93E-03	0
EPS8L2	ENSG00000177106	11	694438	727727	3.84E-02	4.79E-02	9.60E-03	6.74E-02	6.13E-03	9.00E-03	0
C2CD3	ENSG00000168014	11	73723763	73882255	2.25E-01	1.60E-01	6.72E-02	6.23E-01	1.60E-01	9.02E-03	0
HLA-DQA2	ENSG00000237541	6	32709119	32714992	1.73E-02	2.39E-01	1.08E-02	2.64E-01	4.87E-01	9.02E-03	0
PTRHD1	ENSG00000184924	2	25012603	25016251	2.23E-02	6.72E-02	2.08E-03	4.64E-02	1.77E-01	9.03E-03	0
SCN5A	ENSG00000183873	3	38589548	38691164	4.00E-01	3.29E-01	1.92E-01	6.07E-01	3.67E-01	9.07E-03	0
MUC21	ENSG00000204544	6	30951495	30957680	1.22E-01	8.79E-02	5.36E-02	5.96E-02	3.33E-01	9.07E-03	0
ATRIP	ENSG00000164053	3	48488114	48507115	3.34E-01	1.77E-01	4.37E-02	7.39E-01	1.23E-02	9.12E-03	0
NFE2L1	ENSG00000082641	17	46125691	46138849	4.38E-02	2.62E-01	7.31E-03	2.32E-01	1.58E-01	9.16E-03	0
SMCHD1	ENSG00000101596	18	2655737	2805015	1.69E-03	2.25E-03	1.03E-03	1.19E-01	1.10E-01	9.19E-03	0
CCDC51	ENSG00000164051	3	48473574	48481866	5.88E-01	1.77E-01	5.58E-02	7.76E-01	1.08E-02	9.21E-03	0
FAM216B	ENSG00000179813	13	43355686	43365683	2.40E-01	7.04E-01	2.32E-02	2.51E-02	1.53E-02	9.26E-03	0
JPH3	ENSG00000154118	16	87635441	87731762	6.90E-01	5.45E-01	1.81E-01	8.17E-02	2.19E-01	9.35E-03	0
CXCL5	ENSG00000163735	4	74861359	74864496	8.64E-01	8.77E-01	6.49E-02	9.51E-01	8.90E-01	9.35E-03	0
C2orf69	ENSG00000178074	2	200775979	200820658	8.53E-02	2.23E-01	6.13E-03	1.30E-03	3.16E-01	9.36E-03	0
PTRF	ENSG00000177469	17	40554470	40575535	9.23E-04	9.80E-01	3.39E-04	7.98E-02	9.93E-02	9.40E-03	0
CCKBR	ENSG00000110148	11	6280966	6293357	1.30E-01	8.32E-01	1.07E-02	1.06E-01	1.81E-01	9.44E-03	0
ENSA	ENSG00000143420	1	150573327	150602088	3.54E-04	2.00E-02	3.29E-04	7.09E-02	1.71E-02	9.47E-03	0
SNRPA	ENSG00000077312	19	41256542	41271294	5.30E-03	4.35E-02	1.28E-03	1.68E-01	1.49E-02	9.48E-03	0
QPCTL	ENSG00000011478	19	46195741	46207247	4.07E-03	6.94E-03	3.43E-03	1.86E-02	1.88E-03	9.48E-03	0
LHPP	ENSG00000107902	10	126150403	126306457	2.05E-01	3.88E-01	1.92E-02	1.52E-02	6.48E-02	9.50E-03	0
MFAP3	ENSG00000037749	5	153418466	153600038	3.24E-01	3.45E-01	3.61E-02	9.12E-02	1.29E-01	9.50E-03	0
SMAD6	ENSG00000137834	15	66994566	67074338	1.93E-01	2.47E-01	2.43E-02	1.43E-01	2.17E-02	9.53E-03	0
PTGIR	ENSG00000160013	19	47123725	47128375	6.26E-02	7.97E-01	1.16E-02	4.69E-01	2.56E-01	9.65E-03	0
AMPD3	ENSG00000133805	11	10329860	10529126	4.22E-02	3.34E-01	1.90E-02	3.73E-02	4.02E-02	9.67E-03	0
MSTO1	ENSG00000125459	1	155579979	155718153	8.59E-04	2.87E-01	1.10E-03	7.07E-02	1.74E-01	9.71E-03	0
PPP5C	ENSG00000011485	19	46850251	46896238	5.33E-03	2.62E-01	9.13E-03	5.70E-01	3.20E-01	9.71E-03	0
PCM1	ENSG00000078674	8	17780349	17885478	2.06E-02	3.02E-01	3.64E-02	6.27E-01	1.43E-02	9.71E-03	0
TMBIM1	ENSG00000135926	2	219138915	219157309	2.50E-02	5.75E-01	4.09E-03	2.04E-01	7.47E-01	9.73E-03	0
CPSF6	ENSG00000111605	12	69633317	69668138	4.57E-03	1.74E-01	1.37E-03	6.65E-03	1.31E-01	9.87E-03	0
ZNF14	ENSG00000105708	19	19821280	19843906	8.12E-02	8.81E-02	3.31E-02	1.27E-01	1.82E-01	9.87E-03	0
OR5T2	ENSG00000181718	11	55999490	56000737	7.86E-04	2.32E-01	5.34E-04	9.44E-02	3.49E-02	9.90E-03	0
ERAP1	ENSG00000164307	5	96096521	96143803	4.72E-02	2.79E-01	1.41E-02	2.30E-01	2.92E-01	9.92E-03	0
BAG1	ENSG00000107262	9	33247818	33264761	8.20E-01	1.52E-01	5.60E-02	3.27E-01	1.60E-01	9.93E-03	0
MXD3	ENSG00000213347	5	176728462	176739758	8.63E-02	3.23E-01	7.66E-03	1.60E-01	3.11E-02	9.94E-03	0
LY6G5C	ENSG00000204428	6	31644461	31651817	2.89E-03	3.14E-01	6.12E-02	6.20E-02	3.50E-01	9.96E-03	0
TUBB2B	ENSG00000137285	6	3224495	3231964	2.93E-02	4.67E-01	1.00E-02	1.33E-01	3.62E-01	1.00E-02	0
GRK4	ENSG00000125388	4	2965335	3042474	1.52E-02	3.19E-02	5.33E-03	3.04E-01	1.63E-02	1.00E-02	0
ACVR2A	ENSG00000121989	2	148602086	148688393	1.33E-02	1.21E-01	3.90E-03	6.55E-01	8.67E-01	1.01E-02	0

Gene	Ensembl ID	CHR	START	STOP	VHD_CD Pleiotropy P	VHD_GORD Pleiotropy P	VHD_IBD Pleiotropy P	VHD_IBS Pleiotropy P	VHD_PUD Pleiotropy P	VHD_UC Pleiotropy P	d
TET2	ENSG00000168769	4	106067032	106200973	1.51E-01	1.61E-01	2.93E-02	2.63E-02	8.77E-01	1.01E-02	0
RCN2	ENSG00000117906	15	77223960	77242601	3.50E-02	6.94E-02	1.34E-02	8.70E-02	8.76E-02	1.02E-02	0
MTX1	ENSG00000173171	1	155178490	155183615	4.96E-03	5.55E-02	1.51E-03	4.17E-01	9.04E-02	1.02E-02	0
OR5M10	ENSG00000254834	11	56344212	56345263	6.32E-03	6.71E-02	2.87E-03	1.11E-01	1.99E-01	1.03E-02	0
PCDHGA11	ENSG00000253873	5	140800762	140891835	1.67E-02	3.72E-02	3.29E-03	3.35E-01	3.72E-01	1.03E-02	0
CRELD1	ENSG00000163703	3	9975506	9987097	7.42E-01	2.43E-01	5.64E-02	5.24E-01	8.74E-01	1.03E-02	0
LCA5L	ENSG00000157578	21	40777770	40817731	3.95E-02	1.06E-01	5.37E-03	4.50E-01	9.07E-04	1.04E-02	0
LYZ	ENSG00000090382	12	69742121	69748014	1.60E-02	2.69E-01	3.82E-03	5.78E-02	5.74E-02	1.04E-02	0
ANXA5	ENSG00000164111	4	122589110	122618268	1.04E-01	5.93E-01	1.10E-02	2.79E-01	5.08E-01	1.04E-02	0
GTF3C3	ENSG00000119041	2	197627756	197664449	1.88E-02	2.78E-01	5.12E-03	4.33E-01	4.53E-02	1.04E-02	0
AHSA2	ENSG00000173209	2	61404553	61418338	6.28E-02	4.69E-02	9.97E-03	4.96E-01	4.75E-01	1.04E-02	0
SLC50A1	ENSG00000169241	1	155107820	155111329	3.75E-02	7.05E-02	1.80E-02	1.18E-01	3.63E-01	1.04E-02	0
TRANK1	ENSG00000168016	3	36868311	36986548	3.03E-01	1.05E-01	3.92E-02	1.00E-01	4.69E-01	1.05E-02	0
ANKMY1	ENSG00000144504	2	241418839	241508626	6.05E-01	8.57E-01	3.54E-02	3.30E-01	3.84E-01	1.05E-02	0
KIF14	ENSG00000118193	1	200520628	200589862	1.33E-02	1.10E-01	6.47E-04	3.86E-01	3.96E-01	1.05E-02	0
SDCCAG3	ENSG00000165689	9	139296377	139305061	3.39E-03	4.14E-01	2.93E-03	4.97E-01	4.75E-01	1.05E-02	0
RASAL1	ENSG00000111344	12	113536624	113574044	4.54E-01	3.44E-01	1.06E-01	4.75E-01	4.33E-01	1.06E-02	0
SLC44A4	ENSG00000204385	6	31830969	31846823	2.03E-03	3.24E-03	6.27E-03	9.81E-03	1.24E-01	1.06E-02	0
HYAL1	ENSG00000114378	3	50337320	50349812	8.18E-02	7.99E-03	2.27E-02	6.38E-02	3.90E-01	1.06E-02	0
PCSK9	ENSG00000169174	1	55505221	55530525	1.40E-01	3.91E-01	6.40E-02	3.33E-01	7.68E-02	1.06E-02	0
RAPGEF2	ENSG00000109756	4	160025330	160281321	6.98E-02	5.74E-02	1.51E-02	1.04E-01	1.21E-02	1.06E-02	0
OR5B12	ENSG00000172362	11	58206593	58207646	2.42E-01	3.23E-01	1.56E-02	3.22E-01	7.40E-01	1.07E-02	0
KIR3DL1	ENSG00000167633	19	55235969	55378448	8.47E-01	3.44E-01	7.17E-02	2.25E-01	1.93E-01	1.07E-02	0
SCN2A	ENSG00000136531	2	166095912	166248818	1.80E-01	5.94E-02	1.89E-02	2.54E-01	8.76E-02	1.07E-02	0
CENPE	ENSG00000138778	4	104026963	104119566	7.14E-02	1.46E-03	3.09E-01	6.30E-01	1.01E-02	1.07E-02	0
RBMS1	ENSG00000153250	2	161128662	161350305	3.40E-03	1.60E-02	1.79E-03	3.46E-03	4.09E-02	1.09E-02	0
WBSCR16	ENSG00000174374	7	74441226	74490064	1.47E-03	3.94E-01	1.32E-03	2.48E-01	3.52E-01	1.09E-02	0
OR13C9	ENSG00000136839	9	107379529	107380485	1.79E-01	1.27E-01	2.47E-02	3.73E-02	2.97E-04	1.10E-02	0
EIF6	ENSG00000242372	20	33866714	33872788	2.06E-01	1.70E-01	2.56E-02	2.49E-01	4.73E-01	1.10E-02	0
CDHR4	ENSG00000187492	3	49828165	49837268	2.38E-03	1.28E-01	1.16E-03	7.79E-01	7.33E-01	1.11E-02	0
FGG	ENSG00000171557	4	155525286	155534119	8.97E-02	1.62E-01	2.93E-03	3.51E-02	4.96E-01	1.11E-02	0
FCGR3A	ENSG00000203747	1	161511549	161600917	1.01E-01	4.50E-01	1.93E-02	1.86E-02	6.14E-01	1.11E-02	0
TREX1	ENSG00000213689	3	48506445	48509044	6.90E-02	3.34E-01	1.87E-02	4.93E-01	4.24E-02	1.11E-02	0
FYB	ENSG00000082074	5	39105338	39274630	3.33E-01	1.03E-01	2.08E-02	3.98E-01	6.98E-01	1.11E-02	0
ATP6V1B1	ENSG00000116039	2	71163012	71192536	6.23E-02	1.09E-01	2.33E-03	1.95E-01	2.95E-01	1.12E-02	0
C6orf25	ENSG00000204420	6	31686371	31694491	5.37E-03	5.66E-02	1.43E-02	5.23E-02	5.92E-01	1.12E-02	0
PRKD3	ENSG00000115825	2	37477645	37551951	4.36E-01	3.41E-01	3.50E-02	5.74E-01	9.66E-02	1.12E-02	0
DHFRL1	ENSG00000178700	3	93766680	93782233	2.46E-01	8.07E-01	2.33E-02	7.11E-01	6.82E-01	1.13E-02	0
OTUD7B	ENSG00000163113	1	149909705	149982625	3.49E-02	1.96E-02	1.52E-02	1.60E-02	2.97E-02	1.13E-02	0
PFKFB2	ENSG00000123836	1	207222801	207254369	3.40E-01	4.74E-01	6.56E-02	4.87E-01	6.45E-01	1.14E-02	0
GOLPH3L	ENSG00000143457	1	150618701	150669630	7.64E-03	1.62E-01	2.50E-03	2.18E-01	2.24E-01	1.14E-02	0
PCDHGB7	ENSG00000254122	5	140797427	140892546	1.62E-02	3.65E-02	3.46E-03	3.47E-01	3.74E-01	1.14E-02	0
CDC25C	ENSG00000158402	5	137620954	137674044	3.60E-02	2.77E-03	4.33E-03	1.57E-02	4.57E-02	1.14E-02	0
COL25A1	ENSG00000188517	4	109731877	110223813	8.92E-03	7.45E-01	3.40E-02	2.37E-02	3.91E-01	1.15E-02	0
FAM53B	ENSG00000189319	10	126307861	126432838	1.78E-02	8.95E-01	5.53E-03	8.15E-02	8.91E-01	1.15E-02	0
CCNL2	ENSG00000221978	1	1321091	1334708	2.41E-02	3.21E-01	7.13E-03	5.58E-01	5.20E-01	1.15E-02	0
GSDMA	ENSG00000167914	17	38119226	38134019	1.72E-02	6.81E-01	5.14E-03	4.47E-01	4.66E-01	1.16E-02	0
C6orf106	ENSG00000196821	6	34555065	34664636	3.42E-01	1.78E-03	4.71E-02	9.81E-03	6.73E-01	1.17E-02	0
SENP7	ENSG00000138468	3	101043049	101232085	1.68E-02	5.17E-02	4.78E-03	2.59E-01	2.42E-02	1.17E-02	0

Gene	Ensembl ID	CHR	START	STOP	VHD_CD Pleiotropy P	VHD_GORD Pleiotropy P	VHD_IBD Pleiotropy P	VHD_IBS Pleiotropy P	VHD_PUD Pleiotropy P	VHD_UC Pleiotropy P	d
PSMB9	ENSG00000240065	6	32811913	32827362	1.84E-02	1.40E-01	9.39E-03	5.38E-01	1.77E-02	1.17E-02	0
C5orf30	ENSG00000181751	5	102594403	102614361	6.58E-02	7.74E-03	8.97E-03	2.66E-04	5.62E-02	1.18E-02	0
TNNT3	ENSG00000130595	11	1940792	1959936	1.47E-02	4.06E-01	3.32E-03	1.07E-01	3.64E-01	1.18E-02	0
CABP7	ENSG00000100314	22	30116073	30127828	4.47E-02	8.40E-01	8.26E-03	7.41E-02	2.86E-01	1.18E-02	0
GTPBP4	ENSG00000107937	10	1034338	1065876	2.25E-01	7.62E-02	4.85E-02	2.63E-01	1.17E-02	1.19E-02	0
MMP3	ENSG00000149968	11	102706532	102714534	1.01E-02	1.62E-01	7.76E-01	2.01E-01	9.03E-03	1.19E-02	0
TMEM62	ENSG00000137842	15	43415477	43477344	4.53E-01	7.99E-02	2.20E-02	2.63E-01	1.78E-02	1.19E-02	0
TYW5	ENSG00000162971	2	200794698	200820459	9.88E-02	1.96E-01	9.14E-03	2.36E-03	3.69E-01	1.20E-02	0
KIR3DL2	ENSG00000240403	19	55361898	55378662	6.72E-01	3.10E-01	6.52E-02	4.90E-01	1.78E-01	1.20E-02	0
ST7L	ENSG00000007341	1	113066140	113163447	3.95E-02	6.91E-01	8.73E-03	6.51E-01	1.41E-01	1.21E-02	0
ZDHHC19	ENSG00000163958	3	195924320	195938308	4.41E-02	2.78E-02	2.81E-02	1.12E-01	2.31E-01	1.21E-02	0
SULT1A1	ENSG00000196502	16	28616903	28634946	1.19E-03	4.57E-01	8.34E-04	6.60E-02	1.99E-01	1.21E-02	0
TFDP2	ENSG00000114126	3	141663277	141868386	6.56E-01	1.42E-01	1.14E-01	1.03E-01	3.85E-01	1.21E-02	0
INSL4	ENSG00000120211	9	5231419	5235304	2.99E-02	6.23E-01	6.62E-03	9.17E-01	4.43E-01	1.21E-02	0
PCDHGA10	ENSG00000253846	5	140792743	140892546	1.60E-02	3.58E-02	3.53E-03	3.62E-01	3.81E-01	1.22E-02	0
NKD1	ENSG00000140807	16	50582241	50670647	1.19E-03	6.18E-02	1.19E-02	1.44E-01	1.91E-01	1.22E-02	0
TGFBR2	ENSG00000163513	3	30647994	30735634	2.70E-02	7.72E-01	6.95E-03	4.39E-01	1.76E-01	1.23E-02	0
PCDHGA9	ENSG00000261934	5	140782520	140892546	1.77E-02	3.99E-02	3.50E-03	3.76E-01	3.60E-01	1.23E-02	0
OR12D3	ENSG00000112462	6	29341200	29343068	3.24E-04	1.99E-02	6.49E-03	1.60E-01	4.43E-02	1.23E-02	0
EZH1	ENSG00000108799	17	40852293	40897071	3.28E-02	6.97E-01	1.16E-02	1.89E-01	2.46E-01	1.23E-02	0
LYZL4	ENSG00000157093	3	42438570	42452092	1.49E-01	7.36E-02	1.26E-02	5.43E-01	7.78E-02	1.24E-02	0
RUSC1	ENSG00000160753	1	155290687	155300905	2.21E-03	5.45E-01	3.74E-03	3.23E-01	7.99E-02	1.24E-02	0
PTX4	ENSG00000251692	16	1535887	1538982	1.46E-01	3.82E-01	1.56E-02	2.42E-01	3.08E-01	1.24E-02	0
MLF2	ENSG00000089693	12	6857170	6876641	2.66E-01	7.80E-02	3.88E-01	4.21E-02	2.55E-01	1.24E-02	0
NRM	ENSG00000137404	6	30655824	30659197	6.86E-05	8.16E-04	4.48E-03	5.15E-02	3.72E-02	1.24E-02	0
IER3	ENSG00000137331	6	30710976	30712331	5.97E-01	1.21E-01	3.07E-02	4.71E-01	2.97E-01	1.25E-02	0
ELAC1	ENSG00000141642	18	48494361	48514491	1.52E-02	2.24E-01	5.56E-03	1.09E-02	7.95E-01	1.25E-02	0
VAX1	ENSG00000148704	10	118888032	118897812	4.35E-02	1.21E-01	1.46E-02	7.39E-02	2.03E-01	1.25E-02	0
APOM	ENSG00000204444	6	31620193	31625987	6.99E-06	1.79E-04	3.98E-04	2.07E-03	1.36E-02	1.25E-02	0
PLXNB1	ENSG00000164050	3	48445261	48471594	4.40E-01	2.30E-01	5.56E-02	8.16E-01	1.00E-02	1.26E-02	0
OR13C2	ENSG00000257019	9	107366924	107367951	1.31E-01	1.08E-01	2.60E-02	3.21E-02	2.71E-04	1.26E-02	0
LCE3D	ENSG00000163202	1	152551857	152552980	8.49E-02	4.36E-01	2.80E-02	5.95E-01	2.96E-01	1.27E-02	0
CNOT1	ENSG00000125107	16	58553855	58663790	3.65E-01	1.74E-01	5.20E-02	5.31E-02	7.51E-01	1.27E-02	0
C6orf15	ENSG00000204542	6	31079000	31080336	3.55E-03	2.49E-03	2.18E-02	8.22E-03	4.54E-02	1.27E-02	0
IL18RAP	ENSG00000115607	2	103035149	103069025	3.59E-04	2.33E-01	5.16E-04	3.49E-02	6.62E-01	1.27E-02	0
HES7	ENSG00000179111	17	8023908	8027410	5.26E-02	1.51E-01	1.90E-02	4.32E-02	2.01E-02	1.28E-02	0
TRIM27	ENSG00000204713	6	28870779	28891766	6.58E-04	1.34E-03	2.47E-01	2.30E-01	3.89E-02	1.28E-02	0
PCK1	ENSG00000124253	20	56136136	56141513	1.60E-01	4.11E-01	1.73E-02	1.46E-01	5.55E-01	1.28E-02	0
BARX1	ENSG00000131668	9	96713905	96717654	3.32E-01	8.39E-02	4.87E-02	2.63E-01	8.54E-03	1.29E-02	0
SLC9A4	ENSG00000180251	2	103089762	103150431	1.97E-04	1.38E-01	9.51E-04	4.84E-02	7.12E-02	1.29E-02	0
TIGD7	ENSG00000140993	16	3348832	3355729	1.04E-05	8.00E-03	1.79E-04	6.20E-02	1.68E-03	1.29E-02	0
PRPF8	ENSG00000174231	17	1553923	1588176	5.73E-01	4.97E-01	5.02E-02	5.00E-02	2.93E-03	1.29E-02	0
KCNH7	ENSG00000184611	2	163227917	163695240	2.11E-02	1.80E-01	6.65E-03	3.34E-02	6.84E-01	1.29E-02	0
USP31	ENSG00000103404	16	23072727	23160591	1.26E-02	3.74E-04	3.42E-03	6.06E-02	3.43E-03	1.30E-02	0
OR5B3	ENSG00000172769	11	58169937	58170882	2.86E-01	1.12E-01	2.78E-02	2.89E-01	8.64E-01	1.30E-02	0
NCR2	ENSG00000096264	6	41303393	41318625	5.72E-01	6.10E-01	5.23E-02	3.46E-01	4.76E-01	1.31E-02	0
TRIO	ENSG00000038382	5	14143811	14532235	8.10E-02	1.92E-01	1.62E-01	1.27E-01	1.71E-02	1.31E-02	0
LPA	ENSG00000198670	6	160952515	161087407	1.43E-01	2.53E-01	1.47E-02	4.40E-02	9.81E-04	1.32E-02	0
AQP10	ENSG00000143595	1	154293566	154297801	3.89E-04	8.18E-03	5.95E-04	1.25E-04	1.45E-01	1.33E-02	0

Gene	Ensembl ID	CHR	START	STOP	VHD_CD Pleiotropy P	VHD_GORD Pleiotropy P	VHD_IBD Pleiotropy P	VHD_IBS Pleiotropy P	VHD_PUD Pleiotropy P	VHD_UC Pleiotropy P	d
IL12RB1	ENSG00000096996	19	18169805	18209754	9.50E-04	2.13E-01	1.80E-01	1.79E-01	1.91E-02	1.34E-02	0
LARP4B	ENSG00000107929	10	855484	977564	3.57E-01	5.40E-02	1.37E-01	2.34E-01	3.37E-01	1.34E-02	0
CORO2A	ENSG00000106789	9	100883257	100954922	2.39E-01	1.27E-01	6.00E-02	4.04E-01	6.58E-01	1.34E-02	0
INTS10	ENSG00000104613	8	19674651	19709594	3.10E-01	9.35E-01	3.36E-02	3.79E-01	2.67E-01	1.35E-02	0
OLIG1	ENSG00000184221	21	34442450	34444726	5.89E-02	5.56E-01	1.81E-02	2.19E-02	7.02E-02	1.35E-02	0
LY6G6F	ENSG00000204424	6	31674640	31685581	4.10E-05	8.57E-03	1.43E-03	2.15E-02	3.77E-02	1.35E-02	0
FGFR1OP	ENSG00000213066	6	167412670	167466201	1.07E-02	5.14E-01	8.97E-04	1.12E-01	1.21E-01	1.36E-02	0
CHAC1	ENSG00000128965	15	41245160	41248710	6.60E-01	2.58E-01	1.14E-01	4.60E-01	1.31E-01	1.36E-02	0
OR14J1	ENSG00000204695	6	29274403	29275519	4.47E-03	3.15E-01	1.00E-01	4.74E-02	1.77E-01	1.36E-02	0
ASH1L	ENSG00000116539	1	155305059	155532598	1.51E-04	2.92E-01	2.88E-04	1.12E-01	2.53E-01	1.36E-02	0
ACHE	ENSG00000087085	7	100487615	100494594	2.63E-02	6.68E-01	7.41E-03	4.61E-01	6.95E-01	1.37E-02	0
SRRM5	ENSG00000226763	19	44100769	44118649	9.13E-01	4.46E-01	8.39E-02	2.24E-02	7.66E-02	1.37E-02	0
ARHGEF12	ENSG00000196914	11	120207787	120360645	6.15E-02	2.52E-02	7.92E-03	3.54E-01	1.65E-01	1.37E-02	0
ITGAL	ENSG00000005844	16	30483979	30534506	3.89E-01	1.01E-01	2.27E-02	1.77E-01	4.77E-01	1.38E-02	0
DTX1	ENSG00000135144	12	113494514	113535833	1.78E-01	2.06E-01	4.86E-02	3.17E-01	2.27E-01	1.38E-02	0
OR5D18	ENSG00000186119	11	55587086	55588073	2.74E-01	2.43E-02	3.42E-02	1.57E-02	2.06E-01	1.38E-02	0
OR5B17	ENSG00000197786	11	58125597	58126542	2.68E-01	1.53E-01	2.33E-02	3.68E-01	8.35E-01	1.38E-02	0
POSTN	ENSG00000133110	13	38136720	38172981	1.97E-01	5.12E-01	8.29E-02	2.65E-01	6.98E-01	1.40E-02	0
NDFIP1	ENSG00000131507	5	141488070	141534005	6.21E-04	1.39E-01	1.20E-03	1.31E-01	1.10E-01	1.40E-02	0
AK3	ENSG00000147853	9	4711155	4742043	4.64E-01	4.56E-02	5.20E-02	7.27E-02	8.32E-02	1.40E-02	0
IKZF2	ENSG00000030419	2	213864429	214017151	2.38E-02	6.41E-02	6.74E-03	8.87E-02	1.29E-02	1.40E-02	0
PLA2R1	ENSG00000153246	2	160788519	160919121	3.82E-02	7.92E-02	1.42E-02	1.68E-01	4.87E-01	1.40E-02	0
MTMR3	ENSG00000100330	22	30279144	30426855	4.80E-03	8.27E-01	1.38E-03	8.41E-02	1.36E-01	1.42E-02	0
TRIM39-RPP21	ENSG00000248167	6	30297359	30314631	2.30E-04	1.61E-04	9.79E-04	5.38E-03	2.39E-02	1.42E-02	0
FAM83C	ENSG00000125998	20	33873534	33880204	2.96E-01	1.21E-01	3.70E-02	1.90E-01	7.09E-01	1.43E-02	0
TM9SF4	ENSG00000101337	20	30697309	30755061	2.24E-02	8.24E-01	5.79E-03	1.48E-01	1.25E-01	1.43E-02	0
OR5AU1	ENSG00000169327	14	21623026	21624222	1.68E-01	1.24E-01	1.55E-01	7.97E-02	1.78E-01	1.43E-02	0
PCDHGB6	ENSG00000253305	5	140787770	140892546	1.59E-02	3.86E-02	3.31E-03	3.77E-01	3.75E-01	1.43E-02	0
PARP9	ENSG00000138496	3	122246771	122283424	4.06E-01	7.82E-02	6.94E-02	6.03E-02	8.19E-01	1.44E-02	0
IRF6	ENSG00000117595	1	209959036	209979465	2.35E-03	1.82E-01	1.77E-03	2.96E-02	4.58E-01	1.44E-02	0
NSD1	ENSG00000165671	5	176560026	176727216	5.51E-01	3.08E-01	4.43E-02	2.33E-02	3.90E-02	1.44E-02	0
INTS3	ENSG00000143624	1	153700543	153746555	1.50E-01	3.50E-03	4.62E-01	2.86E-02	1.20E-01	1.44E-02	0
PRSS45	ENSG00000188086	3	46783582	46854048	4.63E-02	6.59E-02	1.29E-02	4.84E-01	2.43E-01	1.44E-02	0
CFD	ENSG00000197766	19	859453	863453	1.23E-02	1.99E-01	5.17E-03	6.19E-02	3.22E-01	1.46E-02	0
ADCK4	ENSG00000123815	19	41197434	41224112	1.39E-03	7.98E-01	8.74E-04	4.64E-01	4.71E-02	1.46E-02	0
SCNN1G	ENSG00000166828	16	23194036	23228204	2.34E-02	4.79E-03	8.14E-03	2.47E-01	4.51E-03	1.47E-02	0
NCF1	ENSG00000158517	7	74188309	74203659	1.32E-01	1.11E-02	1.05E-01	4.66E-01	1.73E-01	1.47E-02	0
PCDHGB3	ENSG00000262209	5	140749831	140892546	2.88E-02	4.89E-02	5.16E-03	4.33E-01	3.62E-01	1.49E-02	0
TMEM136	ENSG00000181264	11	120195838	120204391	1.88E-01	6.47E-02	3.33E-02	2.78E-01	1.27E-01	1.49E-02	0
FGA	ENSG00000171560	4	155504278	155511918	3.60E-01	9.11E-02	2.91E-02	3.13E-02	5.01E-01	1.50E-02	0
TUBE1	ENSG00000074935	6	112391980	112408732	1.58E-01	2.62E-01	1.55E-02	8.52E-02	3.60E-01	1.50E-02	0
TTPAL	ENSG00000124120	20	43104526	43123244	5.05E-02	1.52E-01	2.50E-02	3.20E-01	1.37E-01	1.50E-02	0
SYNGR1	ENSG00000100321	22	39745930	39781593	5.15E-03	2.52E-01	3.59E-03	4.37E-01	2.30E-01	1.51E-02	0
CDX1	ENSG00000113722	5	149546358	149564120	4.05E-02	5.18E-01	8.22E-03	1.35E-01	1.88E-01	1.51E-02	0
EYA3	ENSG00000158161	1	28296855	28415207	4.64E-01	7.21E-01	4.05E-02	2.89E-01	1.03E-01	1.51E-02	0
PCDHGB2	ENSG00000253910	5	140739703	140892546	3.46E-02	5.08E-02	7.50E-03	4.34E-01	3.67E-01	1.52E-02	0
ARMC2	ENSG00000118690	6	109169619	109295186	2.00E-01	1.86E-01	2.99E-02	2.75E-01	3.59E-01	1.53E-02	0
FCGR2A	ENSG00000143226	1	161475220	161493803	1.46E-01	4.14E-01	5.18E-02	3.85E-01	4.12E-01	1.53E-02	0
OR5V1	ENSG00000243729	6	29323007	29399744	2.02E-04	1.07E-01	2.00E-03	2.56E-01	1.63E-02	1.53E-02	0

Gene	Ensembl ID	CHR	START	STOP	VHD_CD Pleiotropy P	VHD_GORD Pleiotropy P	VHD_IBD Pleiotropy P	VHD_IBS Pleiotropy P	VHD_PUD Pleiotropy P	VHD_UC Pleiotropy P	d
CHST6	ENSG00000183196	16	75510949	75529282	3.37E-01	4.62E-01	3.21E-02	1.13E-01	2.66E-01	1.54E-02	0
SMAD2	ENSG00000175387	18	45357922	45457515	3.84E-02	2.66E-02	1.35E-02	6.18E-01	1.06E-01	1.54E-02	0
S100A13	ENSG00000189171	1	153591263	153606568	1.37E-01	4.79E-04	2.34E-01	1.62E-04	6.01E-02	1.55E-02	0
GPX6	ENSG00000198704	6	28471073	28495992	1.05E-02	5.79E-01	2.85E-02	4.95E-01	5.06E-01	1.55E-02	0
SMAD4	ENSG00000141646	18	48494410	48611415	1.37E-02	2.73E-01	6.06E-03	1.45E-02	8.28E-01	1.55E-02	0
MRPL24	ENSG00000143314	1	156707095	156711382	6.67E-03	3.37E-02	5.51E-03	1.75E-01	6.01E-03	1.56E-02	0
LY96	ENSG00000154589	8	74903587	74941322	4.13E-02	1.72E-03	4.74E-02	3.58E-01	4.31E-01	1.56E-02	0
C2orf16	ENSG00000221843	2	27799389	27805588	2.38E-02	2.84E-01	7.41E-03	4.80E-02	1.32E-01	1.56E-02	0
CENPO	ENSG00000138092	2	25016005	25045245	1.44E-02	1.71E-01	3.28E-03	2.63E-01	4.32E-01	1.56E-02	0
CCNY	ENSG00000108100	10	35535953	35860852	2.93E-04	2.16E-01	5.01E-04	9.10E-01	4.61E-01	1.56E-02	0
KLKB1	ENSG00000164344	4	187130133	187179625	2.29E-02	4.55E-02	7.02E-03	8.67E-02	1.72E-02	1.57E-02	0
TRAF5	ENSG00000082512	1	211499957	211548288	6.23E-01	2.68E-02	2.32E-01	7.60E-01	3.81E-01	1.57E-02	0
LEF1	ENSG00000138795	4	108968701	109090112	1.03E-01	5.01E-01	6.58E-02	8.43E-01	6.99E-01	1.57E-02	0
RAB19	ENSG00000146955	7	140103843	140126050	8.87E-03	3.01E-01	1.02E-02	8.62E-02	3.99E-01	1.57E-02	0
UCKL1	ENSG00000198276	20	62571186	62587769	1.50E-01	3.98E-01	1.16E-02	3.26E-01	1.42E-01	1.58E-02	0
B4GALNT1	ENSG00000135454	12	58017193	58027138	3.34E-01	6.68E-01	3.67E-02	5.08E-03	1.09E-01	1.58E-02	0
MCCD1	ENSG00000204511	6	31496494	31498009	5.40E-04	1.89E-03	5.39E-03	2.28E-03	9.86E-02	1.59E-02	0
C3orf35	ENSG00000198590	3	37427760	37476988	3.90E-01	2.90E-02	3.21E-01	1.96E-02	4.83E-01	1.60E-02	0
AAMP	ENSG00000127837	2	219128850	219134980	4.58E-02	8.19E-01	3.72E-03	2.88E-01	8.09E-01	1.60E-02	0
PCDHGA3	ENSG00000254245	5	140723601	140892546	5.04E-02	5.40E-02	7.23E-03	4.44E-01	3.69E-01	1.60E-02	0
GINS2	ENSG00000131153	16	85709804	85723679	1.28E-02	5.42E-03	4.46E-03	1.31E-01	4.39E-01	1.60E-02	0
GNAL	ENSG00000141404	18	11688955	11885684	1.08E-01	1.22E-01	2.21E-02	8.34E-05	1.03E-02	1.60E-02	0
FADS1	ENSG00000149485	11	61567099	61596790	2.15E-05	2.04E-01	1.97E-04	1.24E-02	9.76E-04	1.61E-02	0
PCDHGA4	ENSG00000262576	5	140734768	140892546	3.97E-02	4.99E-02	7.03E-03	4.34E-01	3.67E-01	1.62E-02	0
BCAM	ENSG00000187244	19	45312328	45324673	3.21E-01	6.34E-01	6.62E-02	5.98E-01	7.97E-02	1.63E-02	0
SCAF1	ENSG00000126461	19	50145382	50161899	9.58E-02	3.97E-01	1.83E-02	9.92E-01	1.75E-01	1.63E-02	0
PCDHGA5	ENSG00000253485	5	140743898	140892546	3.33E-02	4.92E-02	5.78E-03	4.35E-01	3.66E-01	1.63E-02	0
DLL4	ENSG00000128917	15	41221538	41231237	1.53E-01	1.94E-01	1.53E-01	3.41E-01	4.30E-01	1.64E-02	0
PRPSAP2	ENSG00000141127	17	18743398	18834581	6.59E-01	2.83E-01	9.15E-02	7.60E-01	1.67E-01	1.64E-02	0
AASDHPPT	ENSG00000149313	11	105946228	105969437	1.28E-02	1.38E-02	4.41E-03	7.53E-01	1.89E-05	1.64E-02	0
NOP14	ENSG00000087269	4	2939660	2965112	4.31E-02	3.03E-02	1.06E-02	1.69E-01	3.81E-02	1.65E-02	0
OR5J2	ENSG00000174957	11	55944094	55945032	5.44E-04	1.01E-01	8.09E-04	8.77E-02	3.95E-03	1.65E-02	0
RSPH6A	ENSG00000104941	19	46298968	46318577	1.74E-03	6.24E-02	7.19E-04	3.58E-01	4.53E-02	1.65E-02	0
NR3C2	ENSG00000151623	4	148999913	149365850	1.26E-02	2.00E-02	5.35E-03	1.70E-01	3.43E-02	1.65E-02	0
PCDHGA7	ENSG00000253537	5	140762467	140892546	2.44E-02	4.47E-02	4.47E-03	4.30E-01	3.76E-01	1.66E-02	0
SH2D4B	ENSG00000178217	10	82297658	82406316	1.80E-02	2.06E-01	2.26E-03	1.91E-01	8.96E-03	1.66E-02	0
HLA-DQB2	ENSG00000232629	6	32723875	32731311	7.55E-02	4.09E-01	4.44E-02	3.02E-01	5.22E-01	1.67E-02	0
CHMP5	ENSG00000086065	9	33264940	33281977	9.14E-01	3.74E-01	9.10E-02	5.66E-01	2.38E-01	1.67E-02	0
NADK	ENSG00000008130	1	1682671	1711896	3.28E-02	4.55E-01	6.08E-03	4.38E-01	4.40E-01	1.67E-02	0
GBA	ENSG00000177628	1	155204243	155214490	6.03E-03	1.25E-01	3.84E-03	1.17E-01	7.16E-01	1.68E-02	0
HIF3A	ENSG00000124440	19	46800303	46846690	1.20E-01	2.00E-01	2.35E-02	1.15E-01	1.26E-01	1.69E-02	0
NAA35	ENSG00000135040	9	88556061	88637213	3.92E-01	7.69E-01	4.61E-02	8.39E-01	6.29E-01	1.69E-02	0
PCDHGA6	ENSG00000253731	5	140753651	140892546	3.00E-02	4.98E-02	5.05E-03	4.32E-01	3.67E-01	1.70E-02	0
PCDHGA2	ENSG00000081853	5	140718539	140892546	4.33E-02	5.47E-02	8.56E-03	4.44E-01	3.81E-01	1.71E-02	0
ZNF318	ENSG00000171467	6	43274872	43337216	1.05E-04	1.73E-02	9.20E-04	6.05E-01	6.27E-02	1.72E-02	0
FCGR3B	ENSG00000162747	1	161592986	161601753	1.68E-01	6.71E-01	3.11E-02	6.64E-03	6.80E-01	1.73E-02	0
C12orf10	ENSG00000139637	12	53693470	53700961	4.23E-02	2.65E-01	1.24E-02	3.86E-01	1.25E-01	1.73E-02	0
PCDHGA8	ENSG00000253767	5	140772381	140892546	2.67E-02	5.50E-02	5.97E-03	4.13E-01	3.59E-01	1.73E-02	0
CALCOCO2	ENSG00000136436	17	46908350	46943884	7.37E-04	4.80E-02	1.69E-03	1.45E-01	3.75E-03	1.73E-02	0

Gene	Ensembl ID	CHR	START	STOP	VHD_CD Pleiotropy P	VHD_GORD Pleiotropy P	VHD_IBD Pleiotropy P	VHD_IBS Pleiotropy P	VHD_PUD Pleiotropy P	VHD_UC Pleiotropy P	d
NSRP1	ENSG00000126653	17	28442539	28513493	5.65E-01	2.73E-02	5.70E-02	2.35E-01	2.43E-01	1.74E-02	0
CAMK2N2	ENSG00000163888	3	183977001	183979251	2.17E-01	5.36E-02	4.92E-02	1.17E-02	9.37E-03	1.74E-02	0
CAPN13	ENSG00000162949	2	30945637	31043408	1.59E-01	5.29E-01	1.21E-02	4.62E-01	1.82E-01	1.74E-02	0
CXCL11	ENSG00000169248	4	76954835	76962568	1.47E-02	4.75E-01	4.18E-03	6.21E-01	1.27E-01	1.74E-02	0
ZNF263	ENSG00000006194	16	3313743	3351401	3.15E-06	7.91E-03	1.86E-04	9.15E-03	1.42E-03	1.74E-02	0
CAD	ENSG00000084774	2	27440258	27466811	6.91E-01	3.39E-01	8.24E-02	3.76E-05	2.16E-01	1.74E-02	0
ASTL	ENSG00000188886	2	96789589	96804175	2.09E-01	4.61E-01	2.20E-01	9.28E-02	3.05E-01	1.75E-02	0
RHOU	ENSG00000116574	1	228870824	228882416	2.95E-01	2.94E-01	1.05E-01	2.93E-02	3.54E-02	1.75E-02	0
PGM2L1	ENSG00000165434	11	74041363	74109518	4.72E-02	3.22E-01	4.05E-01	2.11E-01	6.95E-01	1.75E-02	0
CEBPZ	ENSG00000115816	2	37428755	37458856	1.75E-01	7.93E-01	1.97E-02	6.49E-01	1.42E-01	1.76E-02	0
RPTN	ENSG00000215853	1	152126071	152131704	1.73E-01	8.89E-01	2.55E-02	8.45E-02	3.61E-02	1.76E-02	0
AP5M1	ENSG00000053770	14	57735627	57756797	2.56E-01	9.13E-01	1.55E-01	9.28E-01	6.50E-01	1.77E-02	0
PCDHGB4	ENSG00000253953	5	140767452	140892546	2.72E-02	5.55E-02	4.96E-03	4.18E-01	3.60E-01	1.77E-02	0
PCYT1A	ENSG00000161217	3	195941093	196014828	2.45E-02	1.49E-01	7.01E-03	2.66E-01	6.79E-01	1.77E-02	0
ZNF24	ENSG00000172466	18	32912176	32925446	3.43E-01	3.94E-01	1.57E-01	2.50E-03	8.43E-02	1.78E-02	0
NEUROD2	ENSG00000171532	17	37759789	37766030	4.87E-02	5.28E-01	1.25E-02	6.71E-01	2.47E-02	1.79E-02	0
DUSP3	ENSG00000108861	17	41843489	41856356	5.58E-01	8.02E-02	3.49E-02	3.96E-02	2.44E-01	1.79E-02	0
UPK3B	ENSG00000243566	7	76139745	76648340	2.39E-01	2.56E-01	3.73E-02	3.71E-01	7.63E-01	1.79E-02	0
COL6A3	ENSG00000163359	2	238232646	238323018	2.52E-02	7.14E-02	6.01E-02	1.97E-01	1.79E-01	1.80E-02	0
FLOT1	ENSG00000137312	6	30695486	30710510	7.67E-06	7.76E-03	1.23E-04	6.35E-02	7.24E-02	1.81E-02	0
DNAJC22	ENSG00000178401	12	49740700	49751309	1.09E-01	1.21E-01	3.95E-01	4.44E-01	2.12E-02	1.81E-02	0
MORN3	ENSG00000139714	12	122089024	122110537	3.18E-02	2.97E-01	1.40E-02	3.81E-02	3.13E-02	1.82E-02	0
RAVER2	ENSG00000162437	1	65210778	65298915	5.37E-02	1.70E-01	1.46E-02	7.78E-02	2.90E-01	1.82E-02	0
LCE1C	ENSG00000197084	1	152777311	152779107	1.04E-02	2.91E-01	8.16E-03	2.33E-01	5.18E-01	1.82E-02	0
PHLDB3	ENSG00000176531	19	43979106	44009087	9.42E-02	1.21E-01	2.02E-01	3.22E-03	1.16E-02	1.82E-02	0
ORAI1	ENSG00000182500	12	122064455	122080583	2.56E-02	2.08E-01	2.05E-02	1.22E-01	4.69E-02	1.83E-02	0
ZNF672	ENSG00000171161	1	249132409	249143716	1.94E-01	1.08E-01	8.41E-02	6.27E-01	5.43E-01	1.83E-02	0
RHOG	ENSG00000177105	11	3848208	3862213	1.31E-01	1.42E-01	1.19E-02	1.24E-01	2.92E-01	1.83E-02	0
CHPF2	ENSG00000033100	7	150929575	150935908	6.22E-02	1.22E-02	6.00E-02	1.18E-01	3.78E-02	1.84E-02	0
ZEB1	ENSG00000148516	10	31607424	31818742	5.85E-01	1.50E-01	6.66E-02	4.96E-01	6.37E-02	1.84E-02	0
HS2ST1	ENSG00000153936	1	87380331	87602334	7.03E-02	3.16E-01	4.24E-02	3.36E-02	1.79E-01	1.84E-02	0
KCNK3	ENSG00000171303	2	26915619	26956288	2.33E-01	3.81E-01	2.42E-01	5.43E-02	3.02E-03	1.85E-02	0
ELOVL6	ENSG00000170522	4	110967002	111120355	2.48E-01	2.66E-04	1.29E-01	2.27E-03	4.51E-02	1.86E-02	0
HIST1H4K	ENSG00000197914	6	27798952	27799305	2.72E-02	8.38E-03	5.65E-03	2.12E-02	1.18E-01	1.86E-02	0
STX19	ENSG00000178750	3	93733213	93747454	1.96E-01	3.87E-01	3.58E-02	7.61E-01	5.14E-01	1.88E-02	0
PCDHGA1	ENSG00000204956	5	140710252	140892546	5.22E-02	6.23E-02	1.07E-02	4.75E-01	4.00E-01	1.88E-02	0
PCDHGB1	ENSG00000254221	5	140729828	140892546	3.75E-02	5.40E-02	8.04E-03	4.45E-01	3.76E-01	1.89E-02	0
BTBD9	ENSG00000183826	6	38136227	38607924	4.11E-02	1.82E-01	2.12E-01	5.81E-02	8.18E-02	1.90E-02	0
MORN1	ENSG00000116151	1	2252692	2323146	2.26E-01	5.34E-02	1.58E-01	1.58E-04	2.32E-01	1.90E-02	0
C11orf30	ENSG00000158636	11	76155967	76264069	8.03E-03	9.11E-01	4.03E-03	3.71E-01	1.70E-01	1.90E-02	0
CTSF	ENSG00000174080	11	66330934	66336312	1.21E-01	2.02E-01	2.88E-01	1.17E-01	9.27E-01	1.90E-02	0
HRASLS2	ENSG00000133328	11	63320242	63330855	1.14E-01	1.38E-01	2.01E-02	3.42E-02	1.35E-01	1.92E-02	0
CHTOP	ENSG00000160679	1	153606525	153618782	2.16E-02	1.77E-03	2.90E-01	1.64E-04	5.33E-02	1.92E-02	0
MAP3K13	ENSG00000073803	3	185000729	185206885	5.43E-02	1.23E-01	2.82E-02	2.19E-01	8.64E-02	1.92E-02	0
LGR6	ENSG00000133067	1	202163029	202288909	5.16E-02	5.97E-02	1.51E-01	2.61E-01	2.09E-01	1.93E-02	0
HIVEP2	ENSG00000010818	6	143072604	143266338	1.23E-01	1.24E-03	7.56E-02	4.54E-02	1.71E-02	1.93E-02	0
ATP6V0C	ENSG00000185883	16	2563871	2570219	4.32E-02	8.36E-02	1.45E-02	7.71E-01	8.86E-01	1.93E-02	0
GON4L	ENSG00000116580	1	155719508	155829191	3.05E-03	2.14E-01	3.45E-03	7.28E-02	1.71E-01	1.93E-02	0
CTSS	ENSG00000163131	1	150702672	150738433	5.32E-03	2.97E-01	2.36E-03	1.37E-01	1.61E-01	1.94E-02	0

Gene	Ensembl ID	CHR	START	STOP	VHD_CD Pleiotropy P	VHD_GORD Pleiotropy P	VHD_IBD Pleiotropy P	VHD_IBS Pleiotropy P	VHD_PUD Pleiotropy P	VHD_UC Pleiotropy P	d
INPPL1	ENSG00000165458	11	71934745	71950149	1.57E-01	3.55E-01	4.16E-02	1.26E-01	8.02E-02	1.95E-02	0
ZFP57	ENSG00000204644	6	29640169	29648887	2.30E-04	3.06E-02	9.03E-04	3.19E-02	5.79E-02	1.95E-02	0
LY6G5B	ENSG00000240053	6	31637944	31641553	2.05E-02	2.85E-01	1.91E-01	3.00E-02	3.73E-01	1.95E-02	0
COX8C	ENSG00000187581	14	93813537	93814702	1.80E-02	1.79E-01	6.16E-03	5.66E-01	2.32E-01	1.95E-02	0
WIPF1	ENSG00000115935	2	175424300	175547644	2.24E-03	6.77E-02	4.52E-03	2.59E-02	4.01E-02	1.95E-02	0
HLA-DOA	ENSG00000204252	6	32971955	32977389	8.70E-02	2.27E-01	2.45E-02	2.70E-01	8.19E-01	1.95E-02	0
KIF5C	ENSG00000168280	2	149632819	149883273	6.79E-01	1.08E-01	9.91E-02	2.50E-01	4.06E-01	1.96E-02	0
HLA-DOB	ENSG00000241106	6	32780540	32784825	3.90E-03	1.76E-01	1.30E-02	1.17E-01	7.20E-01	1.96E-02	0
FIGNL1	ENSG00000132436	7	50511831	50518088	6.09E-01	6.48E-01	8.07E-02	9.89E-02	3.59E-01	1.96E-02	0
OR5M1	ENSG00000255012	11	56380031	56380978	1.32E-02	4.94E-02	2.89E-03	7.37E-02	7.58E-02	1.96E-02	0
PRG4	ENSG00000116690	1	186265405	186283694	2.04E-01	4.28E-01	5.29E-02	1.43E-01	8.20E-01	1.96E-02	0
ZKSCAN3	ENSG00000189298	6	28317691	28336947	5.73E-02	6.11E-02	1.58E-02	5.35E-01	2.25E-01	1.97E-02	0
TECPR1	ENSG00000205356	7	97843936	97881563	4.90E-01	5.02E-01	1.37E-01	6.53E-02	2.29E-02	1.98E-02	0
PDP2	ENSG00000172840	16	66912492	66929657	5.04E-02	4.94E-01	9.93E-03	6.14E-01	3.47E-01	2.00E-02	0
MAP3K10	ENSG00000130758	19	40697651	40721482	1.52E-01	1.11E-01	1.59E-02	4.76E-01	1.47E-01	2.00E-02	0
NFASC	ENSG00000163531	1	204797779	204991950	2.62E-02	9.72E-01	1.51E-02	2.90E-01	6.53E-01	2.00E-02	0
ATP8B3	ENSG00000130270	19	1782074	1812275	1.33E-02	9.48E-02	3.11E-02	2.95E-01	4.73E-02	2.01E-02	0
SCYL3	ENSG00000000457	1	169818772	169863408	1.33E-01	1.58E-01	3.45E-02	6.98E-01	5.59E-01	2.01E-02	0
GPX4	ENSG00000167468	19	1103936	1106787	6.41E-04	6.87E-01	6.00E-04	3.71E-01	5.44E-01	2.01E-02	0
SLC15A4	ENSG00000139370	12	129277739	129308528	4.93E-01	4.12E-01	2.87E-02	5.39E-01	1.84E-01	2.02E-02	0
PAM	ENSG00000145730	5	102089685	102366809	3.04E-01	2.00E-01	2.40E-02	5.22E-04	4.99E-02	2.02E-02	0
PARVG	ENSG00000138964	22	44568836	44615413	4.07E-01	4.37E-01	2.91E-02	4.36E-01	1.43E-01	2.02E-02	0
WISP3	ENSG00000112761	6	112375275	112392171	1.90E-01	4.05E-01	2.96E-02	1.16E-01	3.25E-01	2.02E-02	0
TMEM180	ENSG00000138111	10	104221149	104236802	8.70E-02	1.66E-01	1.86E-02	6.23E-01	4.59E-01	2.02E-02	0
VARS2	ENSG00000137411	6	30876019	30894236	4.60E-04	1.10E-02	1.57E-03	4.76E-03	9.62E-03	2.03E-02	0
EBF1	ENSG00000164330	5	158122928	158526769	5.95E-05	1.24E-01	8.13E-04	2.38E-01	4.62E-01	2.03E-02	0
EFR3B	ENSG00000084710	2	25264999	25378243	4.12E-05	6.80E-02	1.25E-04	8.40E-03	1.93E-01	2.04E-02	0
ZFP36L1	ENSG00000185650	14	69254377	69263190	2.20E-02	6.56E-01	8.46E-03	2.83E-01	4.25E-01	2.05E-02	0
MBD3	ENSG00000071655	19	1576639	1592882	2.48E-01	1.98E-02	4.13E-02	1.77E-02	7.63E-02	2.05E-02	0
PDE2A	ENSG00000186642	11	72287185	72385635	3.50E-03	9.16E-03	1.44E-02	9.51E-02	2.89E-01	2.06E-02	0
SKAP1	ENSG00000141293	17	46210802	46507637	1.18E-02	3.11E-01	5.26E-03	4.14E-03	2.94E-01	2.06E-02	0
RPL18A	ENSG00000105640	19	17970685	17974962	6.07E-01	8.14E-01	4.98E-02	1.29E-01	1.57E-01	2.07E-02	0
ZPBP2	ENSG00000186075	17	38024417	38034149	1.97E-02	9.50E-01	1.20E-01	9.16E-01	8.49E-01	2.07E-02	0
NF2	ENSG00000186575	22	29999545	30094587	3.02E-02	6.00E-01	1.04E-02	1.69E-02	5.81E-01	2.07E-02	0
ANKRD65	ENSG00000235098	1	1353800	1357149	9.94E-02	4.76E-01	2.84E-02	7.38E-01	4.37E-01	2.08E-02	0
FAM220A	ENSG00000178397	7	6369040	6388612	6.23E-02	3.26E-01	1.54E-02	6.16E-01	8.65E-01	2.08E-02	0
CHID1	ENSG00000177830	11	867357	915058	4.81E-02	2.02E-01	2.33E-02	8.23E-02	2.78E-03	2.08E-02	0
MED22	ENSG00000148297	9	136205160	136214986	6.18E-03	3.53E-01	5.48E-03	4.00E-01	3.18E-02	2.09E-02	0
KPRP	ENSG00000203786	1	152730506	152734529	2.49E-03	2.72E-01	2.64E-03	1.85E-01	4.66E-01	2.09E-02	0
SCRT1	ENSG00000170616	8	145554228	145559943	9.24E-02	6.26E-01	5.09E-02	1.92E-01	1.29E-02	2.09E-02	0
PPT1	ENSG00000131238	1	40538379	40563375	7.30E-02	4.49E-01	6.16E-02	3.54E-03	4.10E-02	2.10E-02	0
DZIP1L	ENSG00000158163	3	137780832	137834660	6.86E-01	2.51E-01	2.87E-02	9.64E-03	6.26E-02	2.10E-02	0
AP3M2	ENSG00000070718	8	42010464	42029191	9.27E-01	4.42E-01	1.53E-01	5.06E-03	4.61E-01	2.11E-02	0
FAM195B	ENSG00000225663	17	79780287	79791178	1.99E-01	2.29E-01	1.68E-01	4.20E-01	2.63E-01	2.11E-02	0
C1orf112	ENSG00000000460	1	169631245	169823221	6.34E-02	2.37E-01	1.50E-02	6.61E-01	7.92E-01	2.11E-02	0
SETD6	ENSG00000103037	16	58549383	58554431	2.91E-01	1.04E-01	7.41E-02	7.28E-02	5.26E-01	2.11E-02	0
SPPL3	ENSG00000157837	12	121200313	121342174	4.36E-01	3.19E-01	1.65E-01	2.48E-01	2.19E-01	2.12E-02	0
KIFAP3	ENSG00000075945	1	169890467	170054349	6.98E-02	8.17E-02	2.14E-02	5.39E-01	4.83E-01	2.12E-02	0
DSE	ENSG00000111817	6	116575336	116762424	8.21E-01	5.60E-02	1.26E-01	7.95E-01	3.77E-01	2.12E-02	0

Gene	Ensembl ID	CHR	START	STOP	VHD_CD Pleiotropy P	VHD_GORD Pleiotropy P	VHD_IBD Pleiotropy P	VHD_IBS Pleiotropy P	VHD_PUD Pleiotropy P	VHD_UC Pleiotropy P	d
SNRK	ENSG00000163788	3	43328004	43466256	1.26E-01	4.50E-02	6.11E-02	6.12E-01	1.73E-01	2.14E-02	0
DNAJC5G	ENSG00000163793	2	27498289	27504367	2.74E-01	7.21E-02	3.50E-02	1.46E-04	5.36E-01	2.16E-02	0
PPP1R1C	ENSG00000150722	2	182818968	182996125	7.33E-01	1.98E-01	2.25E-01	2.09E-01	2.96E-01	2.16E-02	0
CBX1	ENSG00000108468	17	46147414	46178883	6.22E-02	2.80E-01	1.83E-02	1.80E-01	2.13E-01	2.17E-02	0
OPA1	ENSG00000198836	3	193310933	193415612	7.80E-02	5.33E-01	1.28E-02	7.24E-01	7.16E-02	2.17E-02	0
PLXNC1	ENSG00000136040	12	94542499	94701451	3.58E-01	9.19E-02	5.30E-02	4.15E-01	1.44E-01	2.18E-02	0
RAB4B	ENSG00000167578	19	41284121	41302847	3.26E-03	3.01E-03	1.10E-03	9.08E-02	9.38E-02	2.18E-02	0
THBS3	ENSG00000169231	1	155165379	155178842	1.66E-02	2.01E-01	7.10E-03	3.18E-01	5.51E-01	2.19E-02	0
MRPS18B	ENSG00000204568	6	30585486	30594172	9.07E-01	3.59E-01	7.75E-02	1.31E-01	4.94E-01	2.20E-02	0
FAM213A	ENSG00000122378	10	82167585	82192753	3.60E-02	4.66E-01	8.24E-03	1.86E-01	1.06E-01	2.20E-02	0
KANSL3	ENSG00000114982	2	97258907	97308524	3.97E-01	1.14E-01	2.43E-01	6.23E-02	2.34E-01	2.20E-02	0
KRT13	ENSG00000171401	17	39657233	39661957	3.14E-02	4.03E-02	9.78E-03	9.18E-01	2.25E-01	2.21E-02	0
UBE2Q1	ENSG00000160714	1	154521053	154531504	5.63E-03	1.27E-04	8.52E-03	4.43E-03	4.30E-03	2.21E-02	0
CCDC136	ENSG00000128596	7	128430811	128462186	1.80E-02	3.62E-01	6.44E-01	2.84E-01	2.84E-01	2.21E-02	0
DOK2	ENSG00000147443	8	21766384	21771371	4.11E-03	1.46E-01	5.50E-03	3.33E-02	4.43E-01	2.21E-02	0
HBS1L	ENSG00000112339	6	135281516	135424194	6.99E-02	4.33E-01	3.43E-02	3.22E-01	3.29E-01	2.22E-02	0
OXCT1	ENSG00000083720	5	41730167	41870621	2.88E-02	1.82E-01	7.58E-03	1.70E-02	5.00E-02	2.22E-02	0
NMT1	ENSG00000136448	17	43128978	43186384	3.55E-02	2.68E-03	2.16E-02	1.04E-02	1.13E-01	2.22E-02	0
BRI3	ENSG00000164713	7	97881691	97937162	3.54E-01	7.88E-01	2.00E-01	2.94E-02	1.02E-01	2.22E-02	0
PRRT3	ENSG00000163704	3	9987226	9994078	6.40E-01	3.53E-01	1.84E-01	7.64E-01	9.27E-01	2.22E-02	0
TMEM140	ENSG00000146859	7	134832824	134850967	1.53E-02	1.85E-02	2.00E-02	4.47E-01	2.57E-01	2.22E-02	0
CDC42BPA	ENSG00000143776	1	227177566	227506175	1.96E-02	5.95E-04	3.40E-02	5.34E-02	1.45E-01	2.23E-02	0
CPB2	ENSG00000080618	13	46627321	46679211	2.26E-01	2.39E-01	1.42E-01	4.38E-02	2.93E-01	2.23E-02	0
GNB2L1	ENSG00000204628	5	180663909	180675096	5.11E-01	2.98E-01	2.92E-01	5.28E-02	2.47E-01	2.23E-02	0
SLFNL1	ENSG00000171790	1	41481269	41488909	3.35E-01	1.13E-01	6.32E-02	5.91E-02	4.82E-01	2.25E-02	0
DNAJC27	ENSG00000115137	2	25166505	25194963	1.63E-03	5.01E-01	1.17E-03	4.95E-03	6.65E-01	2.25E-02	0
PNKD	ENSG00000127838	2	219135115	219211516	3.75E-02	7.39E-01	8.61E-03	2.20E-01	8.00E-01	2.25E-02	0
LEMD1	ENSG00000186007	1	205350506	205425082	3.85E-02	3.65E-01	8.16E-03	3.75E-01	1.99E-01	2.26E-02	0
ASCL4	ENSG00000187855	12	108168162	108170421	1.59E-01	3.25E-01	2.84E-02	4.41E-01	1.36E-01	2.27E-02	0
KDM4D	ENSG00000186280	11	94706845	94732682	3.89E-02	1.17E-01	2.60E-02	3.07E-01	1.46E-01	2.27E-02	0
GIN1	ENSG00000145723	5	102421704	102455855	9.85E-02	2.04E-02	1.89E-02	3.66E-04	1.94E-02	2.28E-02	0
S100A14	ENSG00000189334	1	153586731	153589462	3.04E-01	3.43E-04	4.08E-01	3.41E-04	1.77E-01	2.28E-02	0
ECE2	ENSG00000145194	3	183967438	184010819	1.25E-02	5.32E-02	7.90E-03	3.49E-03	1.38E-01	2.29E-02	0
BEAN1	ENSG00000166546	16	66461200	66527432	6.98E-02	2.60E-01	1.84E-02	1.74E-01	2.78E-01	2.29E-02	0
VPREB3	ENSG00000128218	22	24094930	24096655	6.02E-02	8.56E-02	1.43E-02	4.00E-01	1.89E-01	2.30E-02	0
HSDL2	ENSG00000119471	9	115142217	115234690	6.74E-02	4.68E-03	1.30E-02	8.25E-01	5.70E-01	2.30E-02	0
TNFRSF11B	ENSG00000164761	8	119935796	119964439	1.64E-02	5.59E-02	2.88E-02	1.90E-01	5.70E-01	2.30E-02	0
C6orf1	ENSG00000186577	6	34214157	34217247	4.57E-01	8.25E-02	9.43E-02	1.27E-01	7.94E-01	2.30E-02	0
SCAPER	ENSG00000140386	15	76640526	77197785	2.05E-02	3.77E-02	1.28E-02	5.99E-02	4.99E-02	2.30E-02	0
NBEAL2	ENSG00000160796	3	47021173	47051193	3.76E-01	5.67E-01	2.92E-02	5.76E-01	2.93E-01	2.30E-02	0
GPR146	ENSG00000164849	7	1084212	1098897	8.49E-01	3.74E-02	1.26E-01	4.08E-01	7.02E-01	2.31E-02	0
COX7A2	ENSG00000112695	6	75947391	75960039	7.25E-02	1.23E-01	8.35E-01	4.29E-01	7.98E-01	2.31E-02	0
FBXL8	ENSG00000135722	16	67193834	67198473	1.47E-05	9.84E-03	5.70E-05	4.18E-02	7.21E-03	2.31E-02	0
C12orf43	ENSG00000157895	12	121440225	121454305	2.82E-01	3.07E-01	1.84E-01	1.72E-01	5.61E-01	2.31E-02	0
RBM24	ENSG00000112183	6	17281577	17294106	9.04E-01	6.56E-01	4.40E-01	4.28E-01	8.77E-01	2.32E-02	0
CCDC57	ENSG00000176155	17	80059336	80170706	6.46E-01	6.85E-02	2.46E-01	3.02E-01	4.68E-01	2.33E-02	0
BSN	ENSG00000164061	3	49591922	49708978	3.26E-02	3.25E-01	1.11E-02	7.82E-01	5.86E-01	2.33E-02	0
NPR1	ENSG00000169418	1	153651113	153666468	1.28E-01	2.10E-02	5.00E-01	2.66E-02	1.96E-01	2.34E-02	0
HSD17B8	ENSG00000204228	6	33172419	33174608	1.71E-01	1.27E-01	1.26E-01	9.38E-01	1.95E-01	2.35E-02	0

Gene	Ensembl ID	CHR	START	STOP	VHD_CD Pleiotropy P	VHD_GORD Pleiotropy P	VHD_IBD Pleiotropy P	VHD_IBS Pleiotropy P	VHD_PUD Pleiotropy P	VHD_UC Pleiotropy P	d
SNAPIN	ENSG00000143553	1	153631130	153634306	3.38E-01	9.61E-04	8.91E-01	4.44E-03	3.22E-02	2.36E-02	0
LNX2	ENSG00000139517	13	28120050	28194541	5.47E-02	7.82E-01	1.67E-02	1.66E-01	7.94E-01	2.36E-02	0
OIT3	ENSG00000138315	10	74653339	74692791	6.61E-02	6.58E-02	2.08E-02	6.00E-01	2.31E-02	2.37E-02	0
LAMB2	ENSG00000172037	3	49158547	49170551	1.30E-03	4.04E-02	9.11E-04	3.42E-01	6.17E-01	2.37E-02	0
MIER1	ENSG00000198160	1	67390578	67454302	2.47E-03	9.36E-03	3.23E-02	5.90E-01	8.44E-02	2.38E-02	0
SIPA1L1	ENSG00000197555	14	71787166	72207946	2.93E-01	3.58E-03	1.42E-01	1.39E-01	2.66E-02	2.38E-02	0
ZBTB20	ENSG00000181722	3	114056941	114866118	1.55E-01	1.11E-01	7.06E-02	1.27E-02	1.26E-01	2.39E-02	0
DEK	ENSG00000124795	6	18224099	18265054	1.03E-01	2.35E-01	3.32E-02	7.54E-02	4.83E-03	2.39E-02	0
SYT2	ENSG00000143858	1	202559724	202679545	3.99E-01	4.69E-01	5.15E-02	3.36E-01	5.01E-02	2.39E-02	0
OR5D13	ENSG00000198877	11	55540914	55541858	4.48E-04	1.12E-01	9.87E-04	2.60E-01	1.42E-02	2.39E-02	0
EPYC	ENSG00000083782	12	91357456	91398803	1.59E-01	7.72E-01	3.05E-01	1.69E-01	1.41E-01	2.40E-02	0
DDAH2	ENSG00000213722	6	31694815	31698394	4.46E-01	6.03E-02	1.51E-01	3.10E-02	7.38E-01	2.41E-02	0
NFYB	ENSG00000120837	12	104510855	104532067	1.53E-01	3.54E-02	7.76E-02	2.86E-01	1.39E-01	2.41E-02	0
GPR183	ENSG00000169508	13	99946784	99959659	7.66E-03	5.82E-01	3.57E-03	6.42E-01	5.30E-01	2.41E-02	0
GPR126	ENSG00000112414	6	142622991	142767403	2.47E-02	7.52E-03	1.39E-02	4.30E-01	2.78E-02	2.42E-02	0
TMEM150B	ENSG00000180061	19	55824169	55845416	3.09E-01	7.12E-01	6.56E-02	7.67E-01	8.01E-01	2.42E-02	0
GPR25	ENSG00000170128	1	200842083	200843306	2.06E-01	2.35E-01	3.12E-02	4.45E-02	3.10E-01	2.42E-02	0
OR1J1	ENSG00000136834	9	125239220	125240235	3.58E-02	8.71E-02	3.32E-02	3.74E-01	2.28E-01	2.43E-02	0
BMF	ENSG00000104081	15	40380091	40401093	4.36E-01	1.07E-01	8.73E-02	1.40E-01	2.41E-02	2.43E-02	0
WDR66	ENSG00000158023	12	122355768	122441833	1.59E-01	1.77E-01	5.14E-02	8.40E-01	1.62E-01	2.44E-02	0
RTN4	ENSG00000115310	2	55199325	55339757	3.02E-02	4.26E-01	4.28E-01	4.84E-03	3.00E-02	2.45E-02	0
ZNF74	ENSG00000185252	22	20748405	20762745	2.05E-02	2.57E-01	1.34E-02	3.56E-01	1.16E-01	2.45E-02	0
EFNA3	ENSG00000143590	1	155036224	155060014	1.31E-03	9.06E-02	9.18E-04	4.55E-01	1.45E-01	2.46E-02	0
CCNT2	ENSG00000082258	2	135675805	135716912	3.55E-03	2.57E-02	4.28E-03	4.84E-01	4.31E-01	2.47E-02	0
PPP1R10	ENSG00000204569	6	30568177	30586389	2.03E-01	5.85E-01	3.61E-02	6.33E-01	8.21E-01	2.47E-02	0
FOXO1	ENSG00000150907	13	41129804	41240734	5.90E-01	7.16E-02	1.07E-01	1.50E-01	2.55E-01	2.47E-02	0
TPR	ENSG00000047410	1	186280784	186344825	2.43E-01	2.33E-01	7.28E-02	7.80E-02	6.41E-01	2.48E-02	0
HLA-DQB1	ENSG00000179344	6	32627244	32636160	4.40E-03	4.43E-03	1.42E-03	5.27E-03	3.15E-02	2.48E-02	0
PIGU	ENSG00000101464	20	33148346	33264910	5.93E-01	5.17E-02	2.18E-01	4.51E-01	6.38E-02	2.48E-02	0
DDX28	ENSG00000182810	16	68055179	68057770	5.50E-02	1.31E-01	1.02E-01	8.49E-03	3.14E-02	2.48E-02	0
FAR1	ENSG00000197601	11	13690217	13753893	1.05E-01	3.21E-01	2.86E-02	6.21E-01	4.95E-01	2.48E-02	0
SPATA6L	ENSG00000106686	9	4553386	4666674	6.30E-02	1.81E-01	3.60E-02	8.62E-01	9.31E-01	2.48E-02	0
LTB	ENSG00000227507	6	31548302	31550299	6.04E-03	1.62E-02	3.79E-01	2.48E-02	4.09E-02	2.49E-02	0
ANKFY1	ENSG00000185722	17	4067201	4167274	6.17E-01	7.36E-02	1.09E-01	1.18E-01	5.68E-01	2.50E-02	0
CABLES2	ENSG00000149679	20	60963688	60982341	1.03E-01	3.41E-01	3.28E-02	4.73E-03	1.38E-02	2.50E-02	0
SSTR2	ENSG00000180616	17	71161151	71167185	1.61E-01	3.18E-01	2.01E-02	5.17E-01	3.52E-02	2.50E-02	0
PLEKHG3	ENSG00000126822	14	65170820	65213610	2.87E-01	3.75E-01	2.95E-02	6.90E-02	6.13E-02	2.51E-02	0
RNF24	ENSG00000101236	20	3907956	3996229	5.36E-01	2.93E-01	9.40E-02	3.79E-02	2.83E-01	2.52E-02	0
RBM42	ENSG00000126254	19	36119932	36128588	2.41E-01	2.26E-01	2.25E-01	4.32E-02	2.06E-01	2.52E-02	0
ARHGEF11	ENSG00000132694	1	156904632	157015162	5.35E-02	4.03E-01	1.14E-02	8.16E-01	1.92E-01	2.53E-02	0
PROX2	ENSG00000119608	14	75319736	75330537	1.57E-01	1.00E-01	5.01E-02	1.52E-01	4.23E-01	2.54E-02	0
ACTR5	ENSG00000101442	20	37377085	37400834	7.41E-02	2.12E-01	1.68E-02	6.89E-01	2.06E-01	2.54E-02	0
ASAP2	ENSG00000151693	2	9346894	9545812	4.39E-01	5.99E-01	6.11E-02	4.23E-01	7.55E-01	2.55E-02	0
GGNBP1	ENSG00000204188	6	33551515	33556803	7.40E-02	2.55E-01	8.57E-02	2.96E-01	1.60E-01	2.55E-02	0
DVL3	ENSG00000161202	3	183873176	183891398	3.65E-05	1.42E-02	4.67E-04	1.20E-02	7.61E-03	2.58E-02	0
CCDC58	ENSG00000160124	3	122078438	122102078	5.85E-01	1.32E-01	2.12E-01	9.64E-02	6.70E-01	2.58E-02	0
NCOA6	ENSG00000198646	20	33284722	33413452	4.02E-01	3.29E-02	2.80E-01	2.45E-01	3.02E-02	2.58E-02	0
ATXN2	ENSG00000204842	12	111890018	112037480	3.99E-02	1.17E-01	9.68E-03	3.39E-01	1.75E-01	2.58E-02	0
TACC1	ENSG00000147526	8	38585704	38710546	2.72E-01	2.07E-01	3.94E-02	2.29E-01	6.42E-01	2.59E-02	0

Gene	Ensembl ID	CHR	START	STOP	VHD_CD Pleiotropy P	VHD_GORD Pleiotropy P	VHD_IBD Pleiotropy P	VHD_IBS Pleiotropy P	VHD_PUD Pleiotropy P	VHD_UC Pleiotropy P	d
NREP	ENSG00000134986	5	110998318	111333161	1.88E-02	5.12E-01	2.30E-02	1.73E-01	3.50E-01	2.59E-02	0
OR10X1	ENSG00000186400	1	158548709	158549689	1.70E-01	7.47E-01	4.63E-02	1.18E-01	5.04E-01	2.60E-02	0
CXCL1	ENSG00000163739	4	74735110	74736959	4.09E-01	1.04E-01	7.71E-02	5.00E-01	5.91E-03	2.60E-02	0
CDC14A	ENSG00000079335	1	100810584	100985833	1.49E-02	4.96E-03	3.79E-02	2.48E-01	1.23E-01	2.60E-02	0
ENO4	ENSG00000188316	10	118609023	118671299	3.90E-02	6.20E-01	1.60E-02	8.70E-02	3.33E-01	2.60E-02	0
NOA1	ENSG00000084092	4	57829536	57844989	2.56E-02	2.17E-01	1.35E-02	4.60E-02	2.85E-01	2.61E-02	0
FIGLA	ENSG00000183733	2	71004442	71017775	2.14E-01	8.72E-02	3.37E-02	5.51E-02	4.53E-01	2.62E-02	0
IPO8	ENSG00000133704	12	30781922	30848920	2.23E-01	7.45E-01	4.00E-02	4.79E-01	7.60E-01	2.62E-02	0
AXL	ENSG00000167601	19	41725108	41767671	1.90E-01	5.51E-02	8.74E-02	3.97E-01	7.80E-03	2.62E-02	0
CLCNKB	ENSG00000184908	1	16370272	16383803	3.24E-01	2.22E-01	2.19E-01	5.15E-01	8.68E-01	2.62E-02	0
ARL13B	ENSG00000169379	3	93698983	93774512	4.75E-01	4.40E-01	8.09E-02	7.34E-01	5.53E-01	2.62E-02	0
C1orf27	ENSG00000157181	1	186344890	186390510	2.63E-01	3.25E-01	8.36E-02	9.51E-02	5.05E-01	2.62E-02	0
SETD2	ENSG00000181555	3	47057919	47205457	2.20E-01	4.12E-01	3.11E-02	4.41E-01	1.59E-01	2.64E-02	0
ASCC2	ENSG00000100325	22	30184597	30234271	3.55E-02	1.63E-01	2.69E-02	6.79E-03	2.30E-01	2.64E-02	0
STRN	ENSG00000115808	2	37070783	37193615	1.66E-01	3.25E-01	2.16E-02	1.80E-01	1.72E-01	2.64E-02	0
PPAP2B	ENSG00000162407	1	56960419	57110974	3.25E-01	4.65E-01	4.10E-02	3.95E-01	8.92E-02	2.64E-02	0
WIZ	ENSG00000011451	19	15532319	15560762	2.50E-01	6.95E-01	1.43E-01	9.84E-01	3.97E-01	2.66E-02	0
HCFC1R1	ENSG00000103145	16	3072621	3074287	1.00E-02	9.65E-01	6.80E-01	8.86E-01	7.88E-01	2.66E-02	0
PRH2	ENSG00000134551	12	11081835	11084940	6.56E-03	7.56E-01	5.20E-03	3.05E-01	9.34E-02	2.66E-02	0
CD151	ENSG00000177697	11	832843	839831	5.67E-01	1.37E-01	1.45E-01	1.95E-01	1.23E-01	2.66E-02	0
POLR2L	ENSG00000177700	11	837356	842545	5.66E-01	1.48E-01	1.26E-01	1.92E-01	1.31E-01	2.67E-02	0
HOXA2	ENSG00000105996	7	27139721	27142430	5.03E-01	2.94E-01	2.28E-01	2.33E-01	4.78E-01	2.67E-02	0
CD2BP2	ENSG00000169217	16	30362087	30366682	2.97E-01	1.49E-02	1.18E-01	6.52E-02	1.99E-01	2.67E-02	0
BCAP29	ENSG00000075790	7	107220422	107269615	8.20E-04	1.70E-02	3.10E-03	5.04E-02	2.48E-04	2.68E-02	0
SLC27A2	ENSG00000140284	15	50474393	50528592	2.75E-01	7.30E-01	5.57E-02	4.65E-01	3.18E-01	2.68E-02	0
TM9SF2	ENSG00000125304	13	100153671	100216260	5.59E-02	1.41E-01	1.24E-02	4.73E-01	2.83E-01	2.68E-02	0
SLC2A10	ENSG00000197496	20	45338126	45364965	1.03E-01	2.86E-01	1.62E-02	4.92E-01	6.65E-02	2.69E-02	0
HIPK2	ENSG00000064393	7	139246316	139477577	2.51E-01	4.46E-01	6.34E-02	7.64E-02	1.84E-01	2.69E-02	0
MCCC2	ENSG00000131844	5	70883115	70954531	3.27E-02	8.80E-01	1.95E-01	1.89E-01	3.91E-02	2.70E-02	0
LRRC3C	ENSG00000204913	17	38097727	38101000	2.54E-02	3.20E-01	1.47E-02	2.02E-01	7.43E-01	2.71E-02	0
RRNAD1	ENSG00000143303	1	156698234	156706752	1.06E-01	4.99E-01	2.23E-02	6.41E-02	2.43E-01	2.71E-02	0
SPDYA	ENSG00000163806	2	29005383	29073477	4.86E-02	4.84E-02	1.25E-02	1.30E-02	2.27E-01	2.71E-02	0
YLPM1	ENSG00000119596	14	75230069	75322244	1.67E-01	1.30E-01	3.33E-02	2.61E-01	4.36E-01	2.71E-02	0
PC	ENSG00000173599	11	66615704	66725847	4.80E-01	9.66E-02	1.65E-01	2.06E-01	5.06E-01	2.72E-02	0
DEFB113	ENSG00000214642	6	49936390	49937338	4.05E-01	3.63E-01	7.24E-02	2.68E-01	2.69E-01	2.72E-02	0
TGM4	ENSG00000163810	3	44916100	44956482	2.12E-01	6.29E-01	2.58E-02	1.97E-01	4.07E-01	2.73E-02	0
NICN1	ENSG00000145029	3	49460379	49466759	4.29E-02	8.49E-01	8.21E-03	8.20E-01	7.18E-01	2.76E-02	0
KRT222	ENSG00000213424	17	38810917	38821433	3.50E-01	6.15E-01	4.14E-02	7.35E-01	6.54E-01	2.76E-02	0
SPRR2E	ENSG00000203785	1	153065611	153078660	1.30E-02	8.38E-02	4.85E-02	2.44E-01	5.02E-03	2.77E-02	0
C19orf40	ENSG00000131944	19	33463115	33469128	1.11E-01	2.23E-02	4.04E-02	1.34E-01	5.47E-01	2.77E-02	0
ERP44	ENSG00000023318	9	102741461	102861322	2.10E-02	5.20E-02	1.07E-02	1.01E-01	1.10E-02	2.78E-02	0
KCNH6	ENSG00000173826	17	61600695	61626338	9.11E-01	1.32E-01	1.74E-01	1.31E-01	5.64E-02	2.78E-02	0
SHMT1	ENSG00000176974	17	18231187	18266856	1.08E-01	1.39E-01	2.33E-02	2.51E-01	6.48E-01	2.78E-02	0
PRDX3	ENSG00000165672	10	120927215	120938345	2.16E-02	2.25E-01	1.24E-02	3.87E-03	2.59E-02	2.79E-02	0
ARF1	ENSG00000143761	1	228270361	228286912	1.01E-03	1.46E-02	2.55E-03	3.35E-03	5.54E-02	2.79E-02	0
S100A16	ENSG00000188643	1	153579362	153585621	5.86E-01	1.59E-03	3.44E-01	1.51E-04	1.49E-01	2.79E-02	0
CLIP1	ENSG00000130779	12	122755979	122907179	1.99E-02	2.41E-03	2.22E-02	4.33E-03	3.48E-03	2.79E-02	0
KIAA0895	ENSG00000164542	7	36363830	36429734	3.05E-01	4.90E-01	3.35E-01	2.31E-02	5.93E-01	2.79E-02	0
PDCD11	ENSG00000148843	10	105156405	105206049	1.33E-01	8.60E-02	6.28E-02	3.67E-01	5.29E-01	2.79E-02	0

Gene	Ensembl ID	CHR	START	STOP	VHD_CD Pleiotropy P	VHD_GORD Pleiotropy P	VHD_IBD Pleiotropy P	VHD_IBS Pleiotropy P	VHD_PUD Pleiotropy P	VHD_UC Pleiotropy P	d
AHRR	ENSG00000063438	5	304291	438406	1.39E-01	1.92E-02	4.04E-02	1.43E-01	4.51E-01	2.80E-02	0
C11orf21	ENSG00000110665	11	2316875	2324279	3.40E-02	6.62E-01	2.92E-02	4.38E-01	1.30E-02	2.80E-02	0
PRMT6	ENSG00000198890	1	107599267	107601916	1.44E-01	2.03E-01	4.56E-02	2.05E-01	4.19E-03	2.82E-02	0
CPEB3	ENSG00000107864	10	93806449	94050844	2.11E-01	8.12E-01	2.38E-02	2.95E-01	7.71E-01	2.82E-02	0
RPS6KL1	ENSG00000198208	14	75370657	75390099	1.72E-01	5.47E-02	1.27E-01	4.08E-01	3.48E-01	2.82E-02	0
OS9	ENSG00000135506	12	58087738	58115340	8.72E-02	4.99E-01	2.43E-02	2.60E-03	3.84E-01	2.83E-02	0
GUCY2D	ENSG00000132518	17	7905912	7923657	1.32E-01	7.11E-02	3.73E-01	1.12E-01	1.94E-01	2.83E-02	0
RRM2	ENSG00000171848	2	10262455	10271545	4.16E-01	6.09E-01	5.73E-01	7.04E-01	4.17E-01	2.83E-02	0
TMEM70	ENSG00000175606	8	74884672	74895018	4.77E-02	6.05E-03	5.30E-02	6.10E-01	4.33E-01	2.84E-02	0
SMG6	ENSG00000070366	17	1963133	2207065	1.95E-02	3.39E-03	5.13E-03	7.59E-03	1.53E-03	2.85E-02	0
CR2	ENSG00000117322	1	207627575	207663240	5.31E-01	9.14E-01	6.57E-02	2.16E-01	1.95E-01	2.85E-02	0
C7	ENSG00000112936	5	40909354	40983041	4.08E-04	5.42E-01	1.44E-03	3.10E-01	3.02E-03	2.86E-02	0
GLTPD1	ENSG00000224051	1	1260136	1264277	4.04E-02	2.51E-01	1.77E-02	7.69E-01	8.19E-01	2.86E-02	0
CD302	ENSG00000241399	2	160625364	160654753	5.97E-01	5.43E-02	1.92E-01	1.56E-02	1.16E-01	2.87E-02	0
PCSK7	ENSG00000160613	11	117075053	117103241	4.52E-01	5.76E-02	1.20E-01	2.28E-01	1.19E-01	2.87E-02	0
HDAC3	ENSG00000171720	5	141000443	141016437	1.06E-01	2.09E-01	6.16E-02	8.03E-02	5.34E-01	2.87E-02	0
NAE1	ENSG00000159593	16	66836778	66907159	5.93E-02	1.65E-01	1.75E-02	1.84E-01	5.43E-01	2.87E-02	0
SLC28A3	ENSG00000197506	9	86890372	86955672	7.02E-02	4.92E-01	1.21E-02	6.03E-04	3.99E-02	2.88E-02	0
TBC1D30	ENSG00000111490	12	65174589	65274812	4.72E-01	8.65E-02	6.40E-02	3.68E-02	8.82E-02	2.88E-02	0
MDC1	ENSG00000137337	6	30667584	30685666	2.34E-01	7.39E-02	3.13E-02	7.07E-01	2.36E-01	2.88E-02	0
YRDC	ENSG00000196449	1	38268616	38273857	3.37E-01	1.79E-01	5.86E-02	6.38E-02	3.73E-01	2.88E-02	0
NOS2	ENSG00000007171	17	26083792	26127525	2.59E-02	8.86E-02	1.63E-02	3.11E-01	3.89E-01	2.88E-02	0
IMPG2	ENSG00000081148	3	100941390	101039404	2.28E-04	8.94E-02	9.79E-04	2.15E-01	1.07E-01	2.89E-02	0
CES3	ENSG00000172828	16	66995140	67009051	4.57E-02	4.22E-01	1.33E-02	5.04E-01	1.73E-01	2.89E-02	0
PTPDC1	ENSG00000158079	9	96793076	96872138	1.40E-01	2.12E-01	3.47E-02	1.97E-01	2.02E-02	2.89E-02	0
SNRPC	ENSG00000124562	6	34725183	34741571	7.26E-01	6.78E-03	8.34E-02	1.26E-02	7.03E-01	2.90E-02	0
RBM34	ENSG00000188739	1	235294498	235324772	1.16E-02	2.85E-01	1.33E-02	2.80E-01	5.66E-02	2.90E-02	0
PTPRC	ENSG00000081237	1	198607801	198726545	2.92E-02	5.91E-01	5.60E-03	3.86E-01	9.12E-01	2.90E-02	0
SOSTDC1	ENSG00000171243	7	16501106	16570205	6.71E-01	4.35E-01	1.03E-01	4.71E-02	5.26E-01	2.91E-02	0
MTX3	ENSG00000177034	5	79275584	79287082	2.37E-02	8.08E-03	8.68E-03	5.29E-02	3.61E-01	2.92E-02	0
UFL1	ENSG00000014123	6	96969471	97003152	1.34E-01	4.28E-01	3.08E-02	2.94E-01	7.56E-01	2.92E-02	0
DNAJC18	ENSG00000170464	5	138744279	138780180	3.66E-01	4.27E-03	3.30E-02	5.69E-01	2.56E-01	2.93E-02	0
XRCC1	ENSG00000073050	19	44047192	44084625	3.12E-01	5.84E-01	3.07E-01	2.28E-01	1.21E-01	2.93E-02	0
CALHM2	ENSG00000138172	10	105206543	105212660	1.11E-01	1.13E-01	2.28E-02	3.59E-01	2.85E-01	2.93E-02	0
GAL3ST2	ENSG00000154252	2	242716240	242743623	2.16E-02	4.13E-02	1.15E-02	1.32E-01	2.75E-01	2.95E-02	0
C7orf49	ENSG00000122783	7	134777115	134855547	1.23E-02	1.79E-02	2.58E-02	4.31E-01	2.79E-01	2.96E-02	0
PVRL4	ENSG00000143217	1	161040785	161059389	1.78E-02	4.39E-01	9.69E-03	2.10E-01	7.78E-01	2.97E-02	0
TC2N	ENSG00000165929	14	92246095	92333880	6.68E-02	2.32E-02	2.07E-02	2.31E-01	4.09E-01	2.97E-02	0
FEN1	ENSG00000168496	11	61560109	61564716	1.38E-04	4.81E-01	6.52E-04	3.15E-02	2.92E-03	2.98E-02	0
CCDC152	ENSG00000198865	5	42756903	42802462	4.49E-02	2.46E-01	2.29E-01	3.19E-03	5.32E-02	2.98E-02	0
CDH6	ENSG00000113361	5	31193857	31329253	1.95E-01	3.20E-01	1.16E-01	4.18E-02	3.15E-01	2.98E-02	0
UPF1	ENSG00000005007	19	18942747	18979045	3.60E-02	2.56E-02	9.67E-03	2.36E-01	1.76E-01	2.98E-02	0
PCDH7	ENSG00000169851	4	30722037	31148422	5.93E-02	4.09E-02	2.09E-02	1.59E-01	7.65E-02	3.00E-02	0
ENAM	ENSG00000132464	4	71494461	71552533	2.49E-01	4.75E-01	6.35E-02	5.10E-01	1.38E-01	3.00E-02	0
CNOT7	ENSG00000198791	8	17086737	17104387	6.99E-01	4.21E-01	1.14E-01	1.36E-01	7.30E-01	3.00E-02	0
GGTLC1	ENSG00000149435	20	23965690	23969416	6.84E-01	2.81E-01	3.19E-01	7.87E-01	5.46E-01	3.01E-02	0
CSRNP3	ENSG00000178662	2	166326157	166545917	4.60E-03	1.89E-01	3.81E-03	7.50E-02	1.31E-02	3.01E-02	0
FOXA3	ENSG00000170608	19	46367247	46377055	2.92E-03	1.41E-02	2.23E-03	8.42E-01	1.80E-02	3.03E-02	0
SELL	ENSG00000188404	1	169659808	169680839	3.47E-02	2.15E-01	1.38E-02	4.69E-01	8.03E-01	3.03E-02	0

Gene	Ensembl ID	CHR	START	STOP	VHD_CD Pleiotropy P	VHD_GORD Pleiotropy P	VHD_IBD Pleiotropy P	VHD_IBS Pleiotropy P	VHD_PUD Pleiotropy P	VHD_UC Pleiotropy P	d
L3MBTL3	ENSG00000198945	6	130334844	130462594	6.64E-01	1.97E-01	6.57E-02	3.98E-01	4.19E-01	3.03E-02	0
MRPS2	ENSG00000122140	9	138391830	138396519	6.28E-02	1.17E-01	3.08E-02	5.48E-01	2.46E-01	3.04E-02	0
ZGPAT	ENSG00000197114	20	62338817	62367494	1.17E-02	1.82E-01	4.48E-03	8.77E-01	5.36E-01	3.04E-02	0
CPS1	ENSG00000021826	2	211342406	211543831	5.63E-01	8.18E-02	1.22E-01	2.83E-01	3.56E-01	3.04E-02	0
HIST1H4I	ENSG00000198339	6	27107076	27108418	7.53E-02	6.64E-01	2.41E-02	3.69E-01	6.93E-01	3.05E-02	0
MTAP	ENSG00000099810	9	21802542	21931646	5.95E-04	2.51E-01	1.23E-03	2.92E-01	1.21E-01	3.05E-02	0
ACTR1A	ENSG00000138107	10	104238986	104262482	9.05E-02	1.72E-01	3.34E-02	9.24E-01	3.07E-01	3.05E-02	0
GOT1	ENSG00000120053	10	101156627	101190381	6.83E-02	6.90E-01	1.13E-02	2.89E-01	6.34E-01	3.05E-02	0
KRTAP8-1	ENSG00000183640	21	32185016	32185570	5.92E-02	1.89E-02	3.52E-02	4.61E-01	1.33E-02	3.06E-02	0
NFAT5	ENSG00000102908	16	69598997	69738569	4.67E-01	7.14E-01	4.95E-02	1.85E-01	4.94E-01	3.07E-02	0
SNX11	ENSG00000002919	17	46180719	46200436	1.68E-01	3.29E-01	3.46E-02	3.99E-01	1.74E-01	3.07E-02	0
RXRB	ENSG00000204231	6	33161365	33168630	1.75E-01	1.53E-01	1.33E-01	9.52E-01	2.24E-01	3.07E-02	0
CABIN1	ENSG00000099991	22	24407642	24574596	3.24E-01	1.10E-01	6.73E-02	3.18E-01	5.05E-01	3.07E-02	0
DMWD	ENSG00000185800	19	46286205	46296060	7.96E-03	9.73E-02	5.23E-03	4.78E-01	3.04E-02	3.07E-02	0
C7orf50	ENSG00000146540	7	1036622	1177896	4.79E-01	2.04E-02	1.05E-01	2.31E-01	3.72E-01	3.08E-02	0
ARHGAP42	ENSG00000165895	11	100558384	100862668	1.04E-01	2.47E-03	6.15E-02	1.19E-02	4.76E-01	3.08E-02	0
DAP3	ENSG00000132676	1	155657751	155708801	3.11E-03	3.22E-01	4.24E-03	5.61E-02	1.40E-01	3.09E-02	0
EPB42	ENSG00000166947	15	43398423	43513481	6.25E-01	1.48E-01	5.72E-02	3.51E-01	4.52E-02	3.09E-02	0
NCK1	ENSG00000158092	3	136581050	136668665	1.75E-02	1.86E-02	7.27E-03	1.30E-02	5.25E-01	3.10E-02	0
TAB1	ENSG00000100324	22	39795746	39833065	7.23E-03	1.34E-01	5.33E-03	7.59E-01	2.32E-01	3.10E-02	0
PF4	ENSG00000163737	4	74846794	74847841	8.97E-01	5.25E-01	7.54E-02	9.92E-02	6.29E-01	3.10E-02	0
ACD	ENSG00000102977	16	67691415	67694713	4.25E-02	2.07E-01	4.13E-01	7.13E-02	5.17E-01	3.11E-02	0
THEM5	ENSG00000196407	1	151819739	151826173	1.19E-01	3.42E-01	3.74E-02	1.44E-01	8.75E-01	3.11E-02	0
CDC40	ENSG00000168438	6	110501344	110575478	6.03E-01	2.84E-01	1.58E-01	2.85E-01	2.80E-01	3.12E-02	0
ZNFX1	ENSG00000124201	20	47854483	47894963	2.46E-01	7.44E-02	5.32E-01	2.96E-01	5.67E-01	3.12E-02	0
ZNF397	ENSG00000186812	18	32820994	32847097	1.28E-01	2.32E-01	6.33E-02	6.61E-03	9.23E-02	3.13E-02	0
NTSR1	ENSG00000101188	20	61340189	61394123	1.19E-02	4.65E-02	2.91E-02	2.36E-01	2.20E-02	3.13E-02	0
DEPDC1	ENSG00000024526	1	68939835	68962904	2.96E-02	3.82E-02	1.31E-02	3.78E-01	6.40E-01	3.14E-02	0
BCL7A	ENSG00000110987	12	122457328	122499948	6.96E-02	2.26E-01	3.92E-02	9.22E-01	6.65E-02	3.15E-02	0
MOGAT2	ENSG00000166391	11	75428864	75444003	5.26E-01	3.19E-01	6.11E-02	8.18E-01	3.68E-02	3.16E-02	0
ZBTB40	ENSG00000184677	1	22778344	22857650	6.35E-01	2.13E-01	5.46E-02	6.38E-01	3.50E-01	3.16E-02	0
PDCD6	ENSG00000249915	5	271736	353971	1.38E-01	1.37E-02	2.93E-02	8.18E-02	1.89E-01	3.17E-02	0
ZNF48	ENSG00000180035	16	30389427	30411429	9.00E-01	2.22E-02	2.13E-01	1.65E-01	3.02E-01	3.18E-02	0
SLC35E2	ENSG00000215790	1	1656277	1677431	3.21E-02	1.57E-01	3.99E-03	1.74E-01	1.71E-01	3.18E-02	0
RPL31	ENSG00000071082	2	101618177	101640494	3.83E-02	2.08E-04	1.82E-02	1.16E-01	1.11E-01	3.19E-02	0
RNF123	ENSG00000164068	3	49726932	49758962	6.81E-03	1.86E-01	5.39E-03	6.32E-01	7.37E-01	3.19E-02	0
SMARCD3	ENSG00000082014	7	150935850	150974982	4.86E-02	5.32E-05	5.64E-02	7.23E-02	1.92E-03	3.20E-02	0
NFX1	ENSG00000086102	9	33290509	33371155	7.45E-01	5.87E-01	1.34E-01	5.13E-01	2.23E-01	3.20E-02	0
RHBDF2	ENSG00000129667	17	74466973	74497872	1.42E-02	2.06E-01	6.41E-03	4.10E-01	2.20E-01	3.21E-02	0
PRRC1	ENSG00000164244	5	126853301	126890781	5.99E-01	5.52E-01	2.77E-01	3.42E-01	3.20E-02	3.22E-02	0
STAT4	ENSG00000138378	2	191894302	192016322	2.36E-02	1.95E-01	1.13E-02	1.09E-01	4.06E-01	3.22E-02	0
FOSL2	ENSG00000075426	2	28615315	28640179	1.77E-03	1.86E-01	1.02E-03	8.66E-01	4.95E-01	3.23E-02	0
NOVA2	ENSG00000104967	19	46436992	46476804	1.17E-01	3.96E-01	2.63E-02	2.49E-01	4.09E-01	3.23E-02	0
ME3	ENSG00000151376	11	86152150	86383678	5.40E-02	1.64E-01	1.61E-02	2.45E-01	2.68E-01	3.24E-02	0
SFXN4	ENSG00000183605	10	120900279	120925179	2.43E-02	1.97E-01	1.08E-02	1.83E-02	1.56E-02	3.24E-02	0
OR12D2	ENSG00000168787	6	29364416	29365448	3.52E-02	6.64E-01	1.85E-02	5.82E-01	1.14E-01	3.24E-02	0
XXYLT1	ENSG00000173950	3	194789008	194991896	3.34E-02	2.78E-01	6.01E-02	1.61E-01	1.16E-01	3.24E-02	0
C14orf79	ENSG00000140104	14	105452112	105476819	9.41E-02	1.20E-01	1.60E-02	7.83E-01	1.81E-01	3.25E-02	0
SUMO2	ENSG00000188612	17	73163408	73179078	3.21E-01	8.23E-03	1.62E-01	4.56E-01	4.21E-02	3.26E-02	0

Gene	Ensembl ID	CHR	START	STOP	VHD_CD Pleiotropy P	VHD_GORD Pleiotropy P	VHD_IBD Pleiotropy P	VHD_IBS Pleiotropy P	VHD_PUD Pleiotropy P	VHD_UC Pleiotropy P	d
RSRC1	ENSG00000174891	3	157823644	158263519	2.00E-01	1.74E-01	4.09E-02	9.61E-02	3.27E-01	3.26E-02	0
CDKN1A	ENSG00000124762	6	36644305	36655116	5.69E-02	8.09E-04	1.26E-01	1.86E-01	1.63E-01	3.26E-02	0
GPC1	ENSG00000063660	2	241375088	241407493	5.34E-01	6.12E-01	8.61E-02	4.65E-01	5.41E-01	3.26E-02	0
MITF	ENSG00000187098	3	69788586	70017488	3.71E-01	4.85E-01	8.13E-02	4.64E-01	5.56E-01	3.27E-02	0
FOSB	ENSG00000125740	19	45971253	45978437	2.90E-02	8.75E-02	5.74E-01	4.07E-01	5.48E-03	3.27E-02	0
GOLGA4	ENSG00000144674	3	37284668	37408242	3.41E-01	1.69E-01	2.65E-01	2.56E-02	6.51E-01	3.27E-02	0
FAM162A	ENSG00000114023	3	122103023	122131181	7.08E-01	1.28E-01	1.49E-01	1.18E-01	5.88E-01	3.27E-02	0
OR8J1	ENSG00000172487	11	56127691	56128764	7.90E-04	2.63E-01	2.39E-03	8.04E-02	5.76E-02	3.27E-02	0
RUVBL2	ENSG00000183207	19	49496705	49519252	2.63E-02	1.53E-01	1.95E-02	5.30E-02	2.56E-01	3.28E-02	0
KCNJ5	ENSG00000120457	11	128761251	128790930	9.60E-02	3.90E-01	2.11E-01	3.58E-02	3.15E-03	3.28E-02	0
PCLO	ENSG00000186472	7	82383329	82792246	8.07E-02	5.78E-02	2.17E-02	2.51E-05	3.86E-02	3.28E-02	0
HNRNPA1	ENSG00000135486	12	54673977	54680872	9.34E-04	2.51E-01	1.27E-03	2.65E-01	3.77E-02	3.28E-02	0
GLP2R	ENSG00000065325	17	9725523	9795419	1.72E-01	7.81E-02	1.53E-01	3.37E-02	2.28E-02	3.30E-02	0
ASPG	ENSG00000166183	14	104552016	104579098	8.86E-03	4.94E-01	2.65E-03	4.25E-02	2.81E-02	3.31E-02	0
PAPPA-AS1	ENSG00000256040	9	119160439	119162885	3.00E-02	1.08E-01	1.55E-01	6.89E-01	4.50E-01	3.31E-02	0
OR56B4	ENSG00000180919	11	6128914	6130065	7.38E-02	2.15E-01	5.03E-02	9.61E-01	2.66E-01	3.31E-02	0
KIAA0753	ENSG00000198920	17	6481468	6544247	2.99E-02	4.99E-02	1.72E-02	2.66E-01	7.14E-01	3.32E-02	0
CTNNB1	ENSG00000168036	3	41236328	41301587	1.87E-01	6.55E-01	3.80E-02	8.63E-01	2.23E-01	3.32E-02	0
SPRR2G	ENSG00000159516	1	153122058	153123345	5.94E-02	8.48E-02	7.80E-01	6.50E-01	8.88E-03	3.32E-02	0
SEC31A	ENSG00000138674	4	83739814	83822319	2.46E-02	1.13E-01	9.37E-03	3.53E-01	2.07E-01	3.32E-02	0
EFCAB5	ENSG00000176927	17	28256218	28435470	6.36E-01	7.77E-02	1.19E-01	3.81E-01	5.35E-01	3.32E-02	0
AP5B1	ENSG00000254470	11	65543364	65548273	1.35E-02	1.91E-01	6.55E-03	5.87E-01	5.90E-02	3.32E-02	0
GHSR	ENSG00000121853	3	172162923	172166246	6.29E-01	4.80E-01	1.68E-01	2.13E-01	5.34E-02	3.33E-02	0
CA14	ENSG00000118298	1	150229554	150237478	2.86E-01	4.31E-02	3.62E-02	2.16E-01	7.05E-01	3.33E-02	0
RHBDL3	ENSG00000141314	17	30593195	30651680	8.43E-02	2.69E-02	2.44E-01	4.60E-01	1.74E-01	3.34E-02	0
DIAPH1	ENSG00000131504	5	140894583	140998622	1.03E-01	1.68E-01	2.02E-02	1.23E-01	3.48E-01	3.34E-02	0
PHOX2A	ENSG00000165462	11	71950121	71956708	2.16E-01	3.61E-01	6.42E-02	3.69E-01	2.47E-01	3.34E-02	0
SIX5	ENSG00000177045	19	46268043	46272484	1.44E-02	1.74E-01	7.39E-03	6.43E-01	3.32E-02	3.35E-02	0
TCF3	ENSG00000071564	19	1609291	1652604	1.45E-01	3.50E-01	2.52E-02	5.30E-01	8.81E-01	3.35E-02	0
DMPK	ENSG00000104936	19	46272975	46285810	2.39E-02	1.65E-01	1.13E-02	3.63E-01	9.30E-03	3.35E-02	0
CEP57L1	ENSG00000183137	6	109416313	109485135	7.15E-02	8.23E-02	1.23E-02	1.85E-02	1.35E-01	3.35E-02	0
GTF2IRD1	ENSG00000006704	7	73868120	74016931	1.50E-01	2.04E-01	6.00E-02	3.21E-01	4.33E-01	3.36E-02	0
DOK7	ENSG00000175920	4	3465033	3503200	5.38E-02	1.94E-02	7.95E-03	5.37E-01	2.73E-01	3.39E-02	0
CASP5	ENSG00000137757	11	104864962	104893895	7.64E-02	2.02E-01	5.30E-02	5.92E-01	8.31E-02	3.40E-02	0
GPR15	ENSG00000154165	3	98250743	98251960	6.44E-02	5.00E-01	2.06E-02	3.10E-02	2.53E-01	3.41E-02	0
LPXN	ENSG00000110031	11	58294344	58345693	2.81E-01	2.22E-01	4.83E-02	6.49E-01	2.18E-01	3.41E-02	0
C20orf166	ENSG00000174407	20	61147660	61167971	7.36E-03	2.52E-01	5.18E-03	2.87E-02	2.03E-01	3.41E-02	0
PPP4R2	ENSG00000163605	3	73045936	73118350	4.73E-01	2.96E-01	6.71E-02	2.93E-01	5.69E-01	3.42E-02	0
IL1R2	ENSG00000115590	2	102608306	102645006	3.27E-02	1.37E-01	1.91E-02	9.84E-01	1.46E-01	3.42E-02	0
TBC1D10B	ENSG00000169221	16	30368423	30381585	7.06E-01	2.59E-02	2.85E-01	1.02E-01	2.34E-01	3.42E-02	0
MKNK2	ENSG00000099875	19	2037470	2051243	9.94E-02	1.30E-01	1.81E-02	1.47E-01	3.48E-01	3.43E-02	0
NKG7	ENSG00000105374	19	51874860	51875969	4.03E-01	3.41E-01	9.09E-02	1.28E-01	1.31E-01	3.43E-02	0
PPP3CA	ENSG00000138814	4	101944566	102269435	4.56E-04	1.56E-02	8.61E-04	2.39E-01	8.04E-02	3.44E-02	0
RIPPLY2	ENSG00000203877	6	84562985	84567234	8.60E-01	3.47E-02	9.29E-02	1.33E-01	4.75E-02	3.44E-02	0
ZNF396	ENSG00000186496	18	32946661	32957301	5.47E-01	4.17E-01	1.56E-01	6.72E-03	5.50E-02	3.44E-02	0
AHCY	ENSG00000101444	20	32868074	32899608	5.20E-01	3.59E-01	1.62E-01	6.11E-01	3.18E-01	3.45E-02	0
ACAP3	ENSG00000131584	1	1227756	1244989	2.08E-01	6.95E-01	4.09E-02	4.16E-01	6.60E-01	3.46E-02	0
PTGDR	ENSG00000168229	14	52734431	52743442	6.06E-01	1.47E-01	2.00E-01	3.81E-02	2.44E-02	3.47E-02	0
EDEM2	ENSG00000088298	20	33703167	33865928	6.80E-01	3.65E-01	2.29E-01	2.05E-01	7.95E-01	3.47E-02	0

Gene	Ensembl ID	CHR	START	STOP	VHD_CD Pleiotropy P	VHD_GORD Pleiotropy P	VHD_IBD Pleiotropy P	VHD_IBS Pleiotropy P	VHD_PUD Pleiotropy P	VHD_UC Pleiotropy P	d
GNB1	ENSG00000078369	1	1716729	1822495	1.54E-01	6.93E-01	2.25E-02	5.08E-01	7.08E-01	3.48E-02	0
OR5AR1	ENSG00000172459	11	56431138	56432163	1.36E-02	4.88E-01	7.64E-03	4.00E-01	8.75E-01	3.48E-02	0
PRR12	ENSG00000126464	19	50094900	50129696	2.43E-02	4.63E-01	1.13E-02	1.79E-01	4.94E-02	3.48E-02	0
AGBL3	ENSG00000146856	7	134671259	134832715	6.36E-03	1.40E-02	2.66E-02	3.45E-01	2.95E-01	3.48E-02	0
UBAC2	ENSG00000134882	13	99853028	100038688	1.88E-02	2.90E-01	6.58E-03	5.78E-01	6.84E-01	3.48E-02	0
SLC22A7	ENSG00000137204	6	43263432	43273276	1.15E-03	2.10E-02	3.19E-03	6.95E-01	2.47E-02	3.49E-02	0
HIST1H2BM	ENSG00000196374	6	27782822	27783267	9.51E-01	2.96E-01	9.83E-02	3.94E-01	3.82E-01	3.49E-02	0
KDM6B	ENSG00000132510	17	7743222	7758114	3.10E-02	3.87E-02	4.28E-02	4.62E-02	6.44E-02	3.50E-02	0
ABHD16A	ENSG00000204427	6	31654726	31671221	5.27E-04	3.23E-02	3.26E-02	1.64E-02	4.20E-02	3.50E-02	0
RASEF	ENSG00000165105	9	85594500	85678092	2.65E-01	7.96E-01	3.16E-02	4.66E-01	8.39E-01	3.50E-02	0
SP2	ENSG00000167182	17	45973516	46006323	2.34E-02	3.26E-01	1.26E-02	1.86E-01	2.31E-01	3.52E-02	0
PPP1R18	ENSG00000146112	6	30644166	30655672	3.09E-03	2.91E-04	8.28E-03	1.09E-02	1.86E-02	3.53E-02	0
CADM2	ENSG00000175161	3	85008132	86123579	1.97E-01	6.54E-01	3.92E-02	2.39E-03	1.89E-01	3.53E-02	0
ADRA2B	ENSG00000222040	2	96778707	96781984	3.69E-02	3.46E-01	8.28E-02	5.18E-02	3.04E-01	3.54E-02	0
KCNJ6	ENSG00000157542	21	38979678	39288749	3.74E-01	2.85E-01	6.01E-02	5.11E-01	5.21E-01	3.55E-02	0
ROMO1	ENSG00000125995	20	34287194	34288906	3.46E-01	1.63E-01	8.08E-02	1.37E-01	3.34E-01	3.56E-02	0
COX18	ENSG00000163626	4	73921797	73935472	6.34E-01	5.01E-01	1.28E-01	2.67E-02	1.50E-01	3.56E-02	0
GUCY1B3	ENSG00000061918	4	156680144	156728743	1.27E-01	3.63E-01	8.73E-02	4.18E-01	7.71E-01	3.58E-02	0
ARHGAP31	ENSG00000031081	3	119013220	119139561	1.51E-01	1.53E-01	9.59E-02	4.24E-01	3.47E-01	3.59E-02	0
RCOR3	ENSG00000117625	1	211431719	211489727	6.75E-01	2.46E-01	2.14E-01	7.71E-01	2.96E-01	3.59E-02	0
GNAO1	ENSG00000087258	16	56225302	56391356	6.05E-02	5.20E-01	1.78E-02	1.42E-01	5.63E-02	3.60E-02	0
RILPL1	ENSG00000188026	12	123955925	124018265	2.96E-01	4.06E-01	8.33E-02	5.19E-02	2.81E-01	3.60E-02	0
RNF144B	ENSG00000137393	6	18368779	18469105	2.00E-01	5.59E-01	5.30E-02	3.55E-01	1.68E-01	3.60E-02	0
WNK4	ENSG00000126562	17	40932696	40948954	3.07E-01	1.27E-01	7.54E-02	3.40E-01	3.44E-01	3.60E-02	0
LCP1	ENSG00000136167	13	46700055	46786006	1.09E-02	7.33E-01	1.44E-02	7.30E-01	3.70E-01	3.60E-02	0
MOGS	ENSG00000115275	2	74688184	74692537	2.75E-02	7.56E-01	1.81E-02	7.28E-01	9.27E-01	3.60E-02	0
KLC1	ENSG00000126214	14	104028233	104167888	1.36E-02	2.43E-01	1.97E-02	5.90E-01	7.66E-01	3.61E-02	0
APPL2	ENSG00000136044	12	105567074	105630016	5.20E-01	5.81E-02	8.72E-02	5.98E-03	1.15E-01	3.61E-02	0
UQCR10	ENSG00000184076	22	30163358	30166402	8.39E-02	1.88E-01	6.90E-02	1.04E-02	2.06E-01	3.61E-02	0
RAET1G	ENSG00000203722	6	150238014	150244257	7.53E-01	1.89E-01	6.76E-02	7.06E-01	7.54E-02	3.61E-02	0
OR52I2	ENSG00000226288	11	4608021	4609135	5.16E-01	2.57E-01	5.38E-01	6.07E-01	3.53E-01	3.62E-02	0
CSTA	ENSG00000121552	3	122044091	122060819	7.85E-02	4.96E-02	3.13E-01	1.54E-03	3.17E-02	3.62E-02	0
SMAD9	ENSG00000120693	13	37418968	37494902	4.53E-01	2.19E-01	3.26E-02	5.55E-01	4.80E-01	3.62E-02	0
PDGFB	ENSG00000100311	22	39619364	39640756	2.83E-01	5.74E-01	6.90E-02	6.74E-01	1.36E-01	3.63E-02	0
TRIM39	ENSG00000204599	6	30294256	30311506	9.29E-05	3.75E-05	4.44E-04	4.04E-03	1.64E-02	3.63E-02	0
TMEM201	ENSG00000188807	1	9648932	9674935	2.13E-01	4.98E-01	5.81E-02	1.15E-01	4.67E-01	3.63E-02	0
ANK2	ENSG00000145362	4	113739265	114304896	4.75E-01	3.71E-01	1.26E-01	2.62E-01	5.91E-01	3.63E-02	0
IL1A	ENSG00000115008	2	113531492	113542167	5.76E-01	1.16E-01	1.00E-01	4.19E-01	6.79E-01	3.64E-02	0
LYSMD4	ENSG00000183060	15	100255902	100273766	7.30E-01	1.08E-01	1.47E-01	1.37E-01	4.34E-01	3.64E-02	0
UBE2G1	ENSG00000132388	17	4172554	4269923	3.06E-01	5.21E-02	9.10E-02	1.18E-01	4.19E-01	3.64E-02	0
SLC44A2	ENSG00000129353	19	10713133	10755235	8.72E-02	2.28E-01	2.77E-02	6.80E-03	8.42E-03	3.65E-02	0
TCF4	ENSG00000196628	18	52889562	53332018	4.48E-02	1.89E-02	2.81E-02	3.13E-01	3.76E-01	3.65E-02	0
KRTAP10-10	ENSG00000221859	21	46057273	46058370	2.14E-01	9.36E-02	1.05E-01	6.10E-01	3.70E-01	3.66E-02	0
ZNF608	ENSG00000168916	5	123972608	124084500	2.59E-01	8.62E-02	8.42E-02	1.18E-01	7.10E-02	3.67E-02	0
PGAP1	ENSG00000197121	2	197697728	197792520	2.14E-02	4.82E-01	1.66E-02	4.87E-01	2.31E-01	3.67E-02	0
DUSP5	ENSG00000138166	10	112257596	112271302	5.71E-03	8.55E-01	5.15E-03	5.98E-02	2.28E-01	3.68E-02	0
ST8SIA4	ENSG00000113532	5	100142639	100238970	9.19E-01	6.89E-01	1.19E-01	1.79E-01	8.75E-01	3.68E-02	0
PPP1R11	ENSG00000204619	6	30034486	30038110	4.07E-02	6.25E-02	5.22E-02	5.41E-02	3.56E-01	3.68E-02	0
PMPCA	ENSG00000165688	9	139305110	139318213	4.16E-03	5.75E-01	1.24E-02	5.87E-01	4.05E-01	3.69E-02	0

Gene	Ensembl ID	CHR	START	STOP	VHD_CD Pleiotropy P	VHD_GORD Pleiotropy P	VHD_IBD Pleiotropy P	VHD_IBS Pleiotropy P	VHD_PUD Pleiotropy P	VHD_UC Pleiotropy P	d
FADS2	ENSG00000134824	11	61560452	61634826	2.52E-05	1.33E-01	2.74E-04	2.17E-02	7.09E-04	3.69E-02	0
COL4A2	ENSG00000134871	13	110958159	111165374	1.34E-01	3.64E-01	3.24E-02	2.16E-01	3.68E-01	3.69E-02	0
C17orf67	ENSG00000214226	17	54869274	54916134	7.57E-03	2.55E-01	5.31E-03	8.84E-01	8.58E-01	3.69E-02	0
NAP1L5	ENSG00000177432	4	89617066	89619386	2.85E-02	3.79E-01	2.05E-02	5.65E-01	5.14E-02	3.70E-02	0
HMGN1	ENSG00000205581	21	40714241	40721573	2.93E-03	1.93E-01	1.50E-03	3.35E-02	9.11E-03	3.70E-02	0
SUFU	ENSG00000107882	10	104263744	104393292	1.07E-01	2.58E-01	3.09E-02	8.97E-01	4.24E-01	3.70E-02	0
ALDOA	ENSG00000149925	16	30064411	30081778	2.03E-01	4.28E-01	4.90E-02	7.07E-01	3.06E-01	3.70E-02	0
DGAT2	ENSG00000062282	11	75470557	75512579	6.12E-01	3.55E-02	2.75E-01	1.16E-01	1.50E-01	3.70E-02	0
LOXL1	ENSG00000129038	15	74218330	74244478	1.89E-02	1.13E-01	2.62E-01	8.53E-03	4.20E-01	3.71E-02	0
VWA2	ENSG00000165816	10	115999018	116051272	1.02E-01	4.18E-01	3.30E-02	6.30E-02	6.36E-01	3.72E-02	0
KRTAP5-5	ENSG00000185940	11	1651033	1652160	3.63E-02	6.14E-02	3.93E-01	4.27E-03	3.92E-02	3.72E-02	0
OR6C68	ENSG00000205327	12	55886147	55887100	3.42E-01	4.42E-01	2.26E-01	9.40E-01	3.09E-01	3.73E-02	0
CALML5	ENSG00000178372	10	5540658	5541533	1.85E-01	3.09E-01	5.06E-02	1.10E-01	1.17E-01	3.73E-02	0
HIST1H2AG	ENSG00000196787	6	27100832	27103070	1.52E-01	4.46E-02	9.87E-02	2.93E-01	4.74E-01	3.73E-02	0
TRAF3IP2	ENSG00000056972	6	111877657	111927481	1.39E-01	1.19E-01	3.81E-02	2.54E-01	1.17E-01	3.74E-02	0
SERINC3	ENSG00000132824	20	43124862	43150750	5.70E-02	1.65E-01	3.55E-02	2.27E-01	6.86E-02	3.75E-02	0
LEPRE1	ENSG00000117385	1	43212006	43232755	9.52E-03	2.36E-01	4.16E-01	4.29E-01	1.59E-02	3.75E-02	0
OR10P1	ENSG00000175398	12	56030644	56031638	5.25E-02	7.61E-02	2.57E-02	5.24E-01	9.32E-02	3.75E-02	0
ZNF692	ENSG00000171163	1	249144205	249153343	7.17E-01	2.28E-01	1.83E-01	5.34E-01	6.07E-01	3.76E-02	0
ANKRD62	ENSG00000181626	18	12093852	12129748	4.49E-01	1.03E-01	3.54E-01	1.55E-02	6.50E-02	3.78E-02	0
CHMP4C	ENSG00000164695	8	82644669	82671750	8.28E-01	3.68E-01	1.83E-01	3.61E-01	2.95E-01	3.78E-02	0
MYO1C	ENSG00000197879	17	1367392	1396106	4.52E-02	9.43E-02	7.85E-03	4.11E-03	1.22E-01	3.79E-02	0
NAAA	ENSG00000138744	4	76831809	76862204	2.14E-02	1.37E-01	1.42E-02	5.01E-01	6.86E-02	3.80E-02	0
WWP2	ENSG00000198373	16	69796209	69975644	3.88E-01	2.43E-01	6.73E-02	5.87E-02	1.38E-01	3.80E-02	0
FERMT1	ENSG00000101311	20	6055492	6104191	1.49E-02	1.59E-01	9.04E-03	1.52E-01	9.70E-02	3.81E-02	0
TSEN15	ENSG00000198860	1	184020811	184043346	4.91E-02	5.84E-01	8.89E-03	1.75E-02	5.73E-03	3.81E-02	0
PRTFDC1	ENSG00000099256	10	25137536	25241533	4.82E-01	6.56E-02	5.39E-02	5.36E-02	5.21E-01	3.81E-02	0
MTF1	ENSG00000188786	1	38275239	38325292	5.66E-01	6.97E-02	8.13E-02	4.64E-03	3.40E-01	3.82E-02	0
TPD52	ENSG00000076554	8	80870571	81143467	1.63E-02	1.51E-01	2.80E-02	8.74E-02	1.47E-01	3.82E-02	0
ZNF750	ENSG00000141579	17	80787311	80798454	7.77E-01	8.55E-02	2.18E-01	5.46E-01	9.48E-01	3.83E-02	0
CD28	ENSG00000178562	2	204571198	204603635	1.99E-01	5.86E-01	3.26E-02	4.56E-01	2.28E-01	3.84E-02	0
PNLIPRP1	ENSG00000187021	10	118349897	118368687	3.51E-01	1.72E-01	5.11E-01	2.33E-01	2.57E-01	3.84E-02	0
PTEN	ENSG00000171862	10	89622870	89731687	1.92E-01	1.19E-01	9.26E-02	1.05E-01	6.49E-01	3.84E-02	0
SGTB	ENSG00000197860	5	64961755	65018862	1.52E-01	2.27E-03	2.24E-01	5.78E-01	4.95E-01	3.85E-02	0
PEMT	ENSG00000133027	17	17408877	17495022	5.11E-02	2.26E-01	4.85E-02	3.82E-02	2.85E-01	3.86E-02	0
NHP2	ENSG00000145912	5	177576461	177580968	7.92E-01	2.81E-02	2.58E-01	2.35E-01	5.88E-01	3.86E-02	0
ZNF652	ENSG00000198740	17	47366568	47439835	9.64E-02	4.63E-01	3.63E-01	5.98E-01	4.50E-02	3.87E-02	0
TOE1	ENSG00000132773	1	45805342	45809647	4.12E-01	4.44E-01	3.22E-01	1.98E-01	3.86E-01	3.88E-02	0
SLC35G2	ENSG00000168917	3	136537489	136574734	1.35E-02	2.14E-02	7.50E-03	1.75E-02	3.72E-01	3.88E-02	0
CDCA2	ENSG00000184661	8	25316513	25365436	8.90E-02	4.59E-01	2.38E-02	2.34E-01	2.41E-01	3.88E-02	0
EBLN2	ENSG00000255423	3	73110810	73112488	6.90E-01	2.73E-01	7.48E-02	5.01E-01	4.38E-01	3.88E-02	0
MRPL20	ENSG00000242485	1	1337288	1342693	8.66E-02	3.12E-01	2.63E-02	6.32E-01	6.62E-01	3.89E-02	0
PLEKHJ1	ENSG00000104886	19	2230002	2237703	1.12E-01	3.44E-03	1.19E-01	6.14E-01	1.42E-03	3.89E-02	0
CTDNEP1	ENSG00000175826	17	7146910	7155810	4.32E-01	5.31E-01	4.12E-02	6.61E-02	5.91E-01	3.89E-02	0
TRIM8	ENSG00000171206	10	104404253	104418164	2.47E-02	2.59E-01	2.02E-02	8.39E-01	1.28E-01	3.90E-02	0
TRPS1	ENSG00000104447	8	116420724	116821899	5.81E-01	5.91E-01	1.03E-01	6.99E-02	8.17E-01	3.90E-02	0
UHRF1BP1	ENSG00000065060	6	34759857	34850915	6.36E-01	8.46E-03	1.16E-01	1.56E-02	6.51E-01	3.90E-02	0
GADD45G	ENSG00000130222	9	92219928	92221470	1.03E-02	1.12E-02	7.09E-03	6.55E-02	1.71E-02	3.91E-02	0
PDHX	ENSG00000110435	11	34937376	35042138	1.69E-02	5.76E-02	2.45E-02	1.68E-01	4.70E-02	3.91E-02	0

Gene	Ensembl ID	CHR	START	STOP	VHD_CD Pleiotropy P	VHD_GORD Pleiotropy P	VHD_IBD Pleiotropy P	VHD_IBS Pleiotropy P	VHD_PUD Pleiotropy P	VHD_UC Pleiotropy P	d
NPTX2	ENSG00000106236	7	98246609	98259180	7.98E-01	1.41E-01	1.33E-01	2.40E-01	6.52E-01	3.91E-02	0
FSIP2	ENSG00000188738	2	186603355	186698017	1.77E-01	3.74E-01	2.54E-01	1.78E-01	1.77E-02	3.91E-02	0
CNTF	ENSG00000242689	11	58390146	58393198	2.23E-01	8.98E-02	4.42E-02	2.72E-01	5.54E-02	3.92E-02	0
RPS6KA4	ENSG00000162302	11	64126620	64139687	6.50E-04	1.34E-01	1.12E-03	1.02E-01	2.42E-01	3.93E-02	0
RND3	ENSG00000115963	2	151324709	151395525	6.15E-01	9.76E-02	2.78E-01	1.24E-01	3.99E-01	3.93E-02	0
SNRPD2	ENSG00000125743	19	46190712	46195827	1.38E-03	2.51E-01	6.00E-03	8.30E-02	2.09E-02	3.94E-02	0
RBM26	ENSG00000139746	13	79885962	79980612	1.05E-01	4.77E-01	3.35E-01	2.03E-01	1.04E-01	3.95E-02	0
SELE	ENSG00000007908	1	169691781	169733846	8.23E-02	2.50E-01	2.83E-02	7.87E-01	8.75E-01	3.95E-02	0
C15orf43	ENSG00000167014	15	45248900	45271427	1.01E-01	8.74E-01	7.90E-01	2.23E-01	5.76E-01	3.95E-02	0
GXYLT2	ENSG00000172986	3	72937224	73047289	6.88E-01	1.21E-01	1.75E-01	2.36E-01	7.98E-02	3.96E-02	0
CNGB1	ENSG00000070729	16	57917503	58005020	2.61E-01	1.97E-01	4.38E-02	5.47E-03	8.65E-02	3.96E-02	0
NFKBIZ	ENSG00000144802	3	101546835	101579866	5.20E-03	9.78E-02	3.06E-03	5.78E-01	3.06E-02	3.96E-02	0
CLDN17	ENSG00000156282	21	31538241	31538971	6.00E-01	1.30E-01	1.45E-01	4.66E-01	2.14E-01	3.97E-02	0
C11orf42	ENSG00000180878	11	6226796	6232362	6.51E-02	3.60E-02	1.37E-02	2.33E-01	8.32E-04	3.98E-02	0
SSH2	ENSG00000141298	17	27952956	28257294	4.33E-01	1.32E-01	1.70E-01	1.51E-01	7.93E-01	3.98E-02	0
IDI2	ENSG00000148377	10	1064847	1071799	3.66E-01	1.77E-01	1.89E-01	2.67E-01	2.62E-02	3.99E-02	0
FAM189B	ENSG00000160767	1	155216996	155225274	2.97E-01	1.38E-01	7.35E-02	2.17E-02	1.88E-01	3.99E-02	0
SPATA2	ENSG00000158480	20	48519928	48532080	8.60E-01	4.97E-01	1.62E-01	1.89E-01	3.86E-01	3.99E-02	0
KRTAP1-3	ENSG00000221880	17	39190142	39191107	1.33E-01	2.26E-02	8.08E-02	8.22E-02	2.36E-02	4.01E-02	0
ZNF471	ENSG00000196263	19	57019212	57041590	4.74E-02	3.56E-02	3.40E-02	3.06E-02	6.71E-02	4.01E-02	0
CYP11B1	ENSG00000160882	8	143953772	143961262	4.51E-01	2.58E-02	6.78E-02	2.97E-01	1.82E-01	4.01E-02	0
KL	ENSG00000133116	13	33590207	33640282	1.52E-01	8.49E-02	4.36E-02	5.23E-02	1.46E-01	4.01E-02	0
DDR1	ENSG00000204580	6	30844198	30867933	4.23E-06	1.34E-03	3.84E-04	5.07E-03	1.49E-02	4.01E-02	0
DSG4	ENSG00000175065	18	28956740	28994875	4.07E-01	1.16E-01	1.65E-01	5.11E-01	3.75E-01	4.01E-02	0
FOXN2	ENSG00000170802	2	48541776	48606433	7.06E-02	3.85E-01	6.03E-02	4.76E-02	7.20E-02	4.02E-02	0
PIM1	ENSG00000137193	6	37137979	37143202	8.70E-03	2.70E-04	1.03E-02	2.85E-01	1.86E-01	4.02E-02	0
ITGB2	ENSG00000160255	21	46305868	46351904	3.59E-01	4.39E-01	1.76E-01	3.39E-01	1.19E-01	4.02E-02	0
PUS3	ENSG00000110060	11	125763381	125773116	3.41E-01	9.15E-02	8.30E-02	2.63E-01	1.18E-01	4.03E-02	0
SCO2	ENSG00000130489	22	50961997	50964868	1.79E-01	9.76E-01	3.19E-02	6.44E-01	2.43E-01	4.04E-02	0
SAR1B	ENSG00000152700	5	133936834	133984961	5.93E-01	7.46E-01	6.60E-01	3.15E-01	2.30E-01	4.04E-02	0
TRIM68	ENSG00000167333	11	4619902	4629489	4.96E-01	1.97E-01	3.39E-01	4.79E-01	2.82E-01	4.06E-02	0
HCAR3	ENSG00000255398	12	123199303	123201439	1.73E-02	3.35E-01	8.25E-03	1.54E-01	1.54E-01	4.06E-02	0
LITAF	ENSG00000189067	16	11641853	11730237	2.29E-03	2.43E-01	3.55E-03	2.09E-01	2.10E-01	4.06E-02	0
ZGLP1	ENSG00000220201	19	10415479	10420556	9.56E-03	3.70E-02	1.21E-02	3.89E-02	4.43E-02	4.07E-02	0
XPO6	ENSG00000169180	16	28109300	28223241	2.43E-01	8.55E-01	5.21E-02	3.23E-01	7.72E-02	4.07E-02	0
FAM162B	ENSG00000183807	6	117073363	117086886	3.67E-01	4.47E-01	1.44E-01	5.29E-01	2.01E-01	4.07E-02	0
PCSK1	ENSG00000175426	5	95726119	95769847	7.40E-01	2.81E-02	8.65E-02	1.13E-01	1.83E-01	4.08E-02	0
MUM1	ENSG00000160953	19	1285890	1378430	9.47E-02	5.67E-03	2.97E-02	7.61E-02	1.53E-01	4.08E-02	0
SDAD1	ENSG00000198301	4	76862103	76912115	7.11E-03	1.08E-01	5.43E-03	3.19E-01	9.86E-02	4.08E-02	0
SLC26A9	ENSG00000174502	1	205882176	205912588	2.34E-01	8.31E-01	6.58E-02	4.34E-01	1.39E-01	4.09E-02	0
ARHGEF26	ENSG00000114790	3	153838792	153975616	4.63E-03	3.79E-02	7.15E-03	3.28E-01	2.37E-01	4.09E-02	0
CACFD1	ENSG00000160325	9	136325089	136335970	1.23E-01	1.78E-01	9.77E-02	1.06E-01	1.15E-02	4.10E-02	0
CCNT1	ENSG00000129315	12	49082247	49110681	5.50E-01	6.98E-01	6.88E-02	4.23E-01	4.92E-01	4.11E-02	0
CPSF3L	ENSG00000127054	1	1246965	1260071	6.65E-02	5.15E-01	3.15E-02	9.64E-01	7.11E-01	4.12E-02	0
C10orf54	ENSG00000107738	10	73507316	73533255	2.29E-01	5.85E-01	1.05E-01	9.71E-01	1.80E-01	4.12E-02	0
MED16	ENSG00000175221	19	867962	893218	7.87E-02	4.10E-01	2.90E-02	1.07E-01	3.04E-01	4.13E-02	0
DEFB108B	ENSG00000184276	11	71544246	71548756	7.15E-01	9.82E-02	1.51E-01	3.89E-01	4.87E-01	4.13E-02	0
VPS37A	ENSG00000155975	8	17104080	17159936	6.43E-01	4.56E-01	2.16E-01	7.82E-02	3.61E-02	4.13E-02	0
YWHAE	ENSG00000108953	17	1247566	1303672	1.22E-01	1.41E-03	2.25E-02	4.96E-03	2.85E-01	4.14E-02	0

Gene	Ensembl ID	CHR	START	STOP	VHD_CD Pleiotropy P	VHD_GORD Pleiotropy P	VHD_IBD Pleiotropy P	VHD_IBS Pleiotropy P	VHD_PUD Pleiotropy P	VHD_UC Pleiotropy P	d
CASR	ENSG00000036828	3	121902530	122005342	7.98E-03	1.63E-02	2.28E-02	3.72E-02	1.32E-02	4.14E-02	0
MXRA8	ENSG00000162576	1	1288069	1297157	5.37E-02	2.44E-01	3.28E-02	6.32E-01	7.82E-01	4.15E-02	0
TMEM171	ENSG00000157111	5	72416119	72427644	1.50E-01	5.79E-01	2.74E-02	1.56E-01	9.46E-01	4.16E-02	0
NOS3	ENSG00000164867	7	150688083	150711676	2.11E-03	3.77E-03	2.24E-03	2.63E-02	1.25E-01	4.16E-02	0
AGRN	ENSG00000188157	1	955503	991496	8.10E-01	3.38E-03	1.23E-01	2.34E-01	2.75E-02	4.16E-02	0
COX8A	ENSG00000176340	11	63742079	63744015	5.55E-01	8.13E-01	2.00E-01	3.65E-01	3.96E-01	4.16E-02	0
SLC5A6	ENSG00000138074	2	27422455	27435826	9.25E-01	4.91E-01	1.59E-01	2.13E-04	2.58E-01	4.17E-02	0
BDP1	ENSG00000145734	5	70751442	70863649	1.12E-01	9.26E-01	2.68E-01	1.20E-01	1.01E-01	4.18E-02	0
ZNF311	ENSG00000197935	6	28962562	28973093	7.77E-02	6.70E-03	5.71E-01	5.67E-01	1.69E-01	4.18E-02	0
CCDC90B	ENSG00000137500	11	82970139	82997450	2.44E-02	3.23E-02	4.54E-01	1.41E-01	4.71E-01	4.18E-02	0
USP12	ENSG00000152484	13	27640293	27746033	1.56E-01	1.62E-01	6.14E-02	7.38E-02	5.74E-03	4.18E-02	0
TMEM30A	ENSG00000112697	6	75962640	75994684	4.34E-02	1.01E-01	7.10E-01	6.68E-01	9.37E-01	4.18E-02	0
DTNB	ENSG00000138101	2	25600067	25896503	5.80E-02	3.61E-02	3.78E-02	2.03E-01	1.99E-01	4.18E-02	0
LTK	ENSG00000062524	15	41795836	41806085	8.38E-01	4.53E-01	1.56E-01	3.42E-01	1.41E-01	4.19E-02	0
SCMH1	ENSG00000010803	1	41492872	41707826	3.38E-01	1.41E-01	7.03E-02	1.01E-01	6.94E-01	4.19E-02	0
DAGLA	ENSG00000134780	11	61447905	61514473	1.35E-03	2.92E-01	4.16E-03	4.77E-01	7.34E-02	4.19E-02	0
ITCH	ENSG00000078747	20	32951041	33099198	4.75E-01	5.47E-02	1.18E-01	4.09E-01	2.14E-01	4.21E-02	0
NIPSNAP1	ENSG00000184117	22	29950797	29977326	2.54E-02	4.10E-01	2.17E-02	4.48E-02	5.18E-01	4.22E-02	0
GPA33	ENSG00000143167	1	167022073	167059868	6.49E-01	4.69E-01	1.60E-01	2.02E-01	6.26E-01	4.22E-02	0
GSTA3	ENSG00000174156	6	52761437	52774483	7.86E-01	2.49E-02	1.55E-01	6.81E-01	2.23E-01	4.22E-02	0
INO80	ENSG00000128908	15	41271078	41408552	4.33E-01	5.33E-01	1.04E-01	9.52E-02	4.34E-01	4.22E-02	0
CHD4	ENSG00000111642	12	6679249	6716642	1.84E-02	1.92E-02	4.05E-02	1.98E-01	1.05E-01	4.23E-02	0
ZNF148	ENSG00000163848	3	124944405	125094198	6.16E-02	2.04E-02	6.65E-01	6.50E-01	1.48E-01	4.24E-02	0
UCP3	ENSG00000175564	11	73711326	73720480	4.59E-02	1.42E-02	5.34E-02	7.72E-01	9.40E-02	4.24E-02	0
REV3L	ENSG00000009413	6	111620234	111804918	2.48E-01	3.25E-01	4.39E-02	1.52E-01	1.67E-01	4.25E-02	0
AZIN1	ENSG00000155096	8	103838585	103906092	5.60E-01	5.10E-01	1.72E-01	2.14E-02	4.57E-01	4.25E-02	0
WDR88	ENSG00000166359	19	33622996	33666701	4.28E-01	6.08E-01	4.56E-02	4.50E-01	8.70E-01	4.25E-02	0
FAM129B	ENSG00000136830	9	130267618	130341268	1.55E-02	3.11E-01	4.40E-01	1.43E-02	2.09E-02	4.26E-02	0
CALR	ENSG00000179218	19	13049392	13055303	2.17E-01	1.06E-02	6.05E-02	2.30E-01	3.12E-01	4.26E-02	0
SRP14	ENSG00000140319	15	40327940	40331389	3.06E-01	6.29E-01	1.06E-01	4.30E-01	3.82E-01	4.27E-02	0
CYC1	ENSG00000179091	8	145149930	145152428	4.28E-03	5.94E-01	3.80E-03	4.08E-02	3.35E-01	4.27E-02	0
KCNH2	ENSG00000055118	7	150642049	150675403	8.90E-03	5.98E-03	2.95E-02	1.88E-02	7.56E-01	4.28E-02	0
DDX55	ENSG00000111364	12	124086624	124105488	5.17E-01	6.36E-01	4.88E-02	4.70E-01	8.94E-02	4.28E-02	0
PPID	ENSG00000171497	4	159630286	159644548	1.58E-01	6.43E-02	3.57E-02	9.55E-01	8.89E-01	4.29E-02	0
C5orf28	ENSG00000151881	5	43444354	43483995	1.23E-01	6.59E-02	2.65E-02	2.18E-02	8.17E-02	4.29E-02	0
GATA3	ENSG00000107485	10	8095567	8117161	4.02E-01	7.62E-01	8.13E-02	6.78E-01	6.04E-01	4.30E-02	0
CCDC89	ENSG00000179071	11	85394893	85397320	2.85E-01	2.41E-02	2.31E-01	3.36E-01	4.94E-01	4.31E-02	0
GCDH	ENSG00000105607	19	13001840	13025021	1.85E-01	4.85E-03	2.78E-02	9.04E-02	2.09E-01	4.31E-02	0
NANS	ENSG00000095380	9	100819021	100845357	7.84E-02	2.68E-01	1.63E-02	4.31E-02	5.81E-01	4.32E-02	0
FKBP15	ENSG00000119321	9	115923286	115983641	7.04E-01	4.60E-01	9.95E-02	2.57E-01	2.54E-01	4.32E-02	0
MUC19	ENSG00000205592	12	40787197	40964632	2.27E-04	2.12E-01	3.35E-04	1.47E-01	1.18E-01	4.32E-02	0
TMEM143	ENSG00000161558	19	48835613	48867494	5.44E-02	2.87E-01	7.56E-01	4.05E-01	2.45E-01	4.32E-02	0
FURIN	ENSG00000140564	15	91411822	91426688	1.62E-03	3.74E-02	4.47E-03	9.50E-02	2.74E-01	4.32E-02	0
WNT4	ENSG00000162552	1	22443798	22470462	3.89E-01	2.67E-01	9.04E-02	7.14E-01	7.00E-02	4.33E-02	0
TRAF3	ENSG00000131323	14	103243813	103377837	4.43E-01	6.79E-03	1.05E-01	8.32E-02	5.84E-02	4.34E-02	0
NT5C	ENSG00000125458	17	73126320	73127890	4.02E-01	7.76E-03	2.18E-01	4.35E-01	9.07E-02	4.34E-02	0
VCAM1	ENSG00000162692	1	101185298	101204601	5.66E-01	5.81E-01	1.52E-01	4.54E-01	2.95E-01	4.36E-02	0
KIF2A	ENSG00000068796	5	61601989	61833076	3.28E-01	1.17E-01	3.37E-02	9.99E-02	6.17E-02	4.36E-02	0
SPTLC2	ENSG00000100596	14	77972340	78083116	4.74E-04	1.38E-01	1.25E-03	3.60E-01	6.36E-02	4.36E-02	0

Gene	Ensembl ID	CHR	START	STOP	VHD_CD Pleiotropy P	VHD_GORD Pleiotropy P	VHD_IBD Pleiotropy P	VHD_IBS Pleiotropy P	VHD_PUD Pleiotropy P	VHD_UC Pleiotropy P	d
CAPN10	ENSG00000142330	2	241526133	241557122	5.47E-01	1.04E-01	9.82E-02	8.90E-01	6.94E-01	4.36E-02	0
SLC9A8	ENSG00000197818	20	48429250	48508779	4.18E-01	4.67E-01	1.89E-01	1.56E-01	1.07E-01	4.36E-02	0
TBX5	ENSG00000089225	12	114791736	114846247	1.30E-01	6.42E-02	6.58E-02	3.38E-01	1.07E-01	4.37E-02	0
MND1	ENSG00000121211	4	154265801	154336270	7.37E-01	4.42E-01	2.98E-01	9.55E-01	4.21E-01	4.37E-02	0
C9orf163	ENSG00000196366	9	139377947	139380518	8.02E-03	8.74E-01	3.06E-03	6.33E-01	4.97E-01	4.38E-02	0
KRTAP4-8	ENSG00000204880	17	39253233	39254393	6.32E-01	1.67E-01	2.38E-01	7.85E-02	1.39E-01	4.38E-02	0
ADAM15	ENSG00000143537	1	155023042	155035252	2.09E-03	3.63E-01	3.04E-03	2.92E-01	4.85E-02	4.39E-02	0
NDUFA6	ENSG00000184983	22	42481529	42486959	1.74E-01	5.99E-01	6.64E-02	9.51E-02	1.71E-01	4.40E-02	0
ZBTB16	ENSG00000109906	11	113930315	114121398	2.46E-01	5.15E-01	3.84E-02	2.08E-01	5.95E-01	4.40E-02	0
TMEM41A	ENSG00000163900	3	185194284	185216845	3.58E-01	6.26E-02	9.74E-02	7.87E-01	2.77E-01	4.41E-02	0
CBWD1	ENSG00000172785	9	121041	188979	3.49E-02	2.72E-01	1.06E-01	6.09E-01	4.02E-01	4.41E-02	0
CAPG	ENSG00000042493	2	85621871	85645555	6.64E-01	3.60E-01	2.40E-01	3.71E-01	3.61E-01	4.42E-02	0
SPINK4	ENSG00000122711	9	33218363	33248565	7.11E-01	3.53E-01	2.25E-01	5.29E-01	5.42E-02	4.43E-02	0
ALDH8A1	ENSG00000118514	6	135238528	135271260	4.17E-02	5.48E-02	2.11E-02	3.21E-01	3.20E-01	4.43E-02	0
MBTPS1	ENSG00000140943	16	84087368	84150511	7.84E-01	3.61E-01	2.31E-01	4.99E-01	3.67E-01	4.45E-02	0
NDUFB1	ENSG00000183648	14	92582466	92588261	3.47E-01	7.17E-01	8.75E-02	8.33E-01	4.60E-01	4.45E-02	0
IFITM2	ENSG00000185201	11	307631	315272	4.33E-05	1.35E-02	5.44E-04	9.81E-02	1.35E-01	4.46E-02	0
PAQR8	ENSG00000170915	6	52226219	52272575	1.84E-02	7.88E-01	2.83E-01	5.94E-01	8.18E-02	4.46E-02	0
TRPC4AP	ENSG00000100991	20	33590207	33680674	4.03E-01	2.69E-01	2.66E-01	1.40E-01	4.10E-01	4.46E-02	0
APOA2	ENSG00000158874	1	161192082	161193421	1.35E-01	3.88E-01	5.70E-02	4.18E-01	6.90E-01	4.47E-02	0
RIIAD1	ENSG00000178796	1	151682909	151702281	2.55E-02	9.32E-01	2.80E-02	8.23E-01	2.94E-01	4.47E-02	0
OR4C16	ENSG00000181935	11	55339604	55340536	2.51E-01	1.02E-01	6.41E-02	4.55E-02	3.67E-01	4.47E-02	0
ZC3H13	ENSG00000123200	13	46528600	46626894	2.97E-01	6.94E-01	3.81E-01	3.80E-01	1.44E-01	4.48E-02	0
CRAT	ENSG00000095321	9	131857089	131873468	1.32E-03	4.02E-02	7.23E-02	6.08E-03	3.72E-02	4.48E-02	0
ADORA1	ENSG00000163485	1	203059782	203136533	5.63E-02	9.14E-02	2.32E-02	1.71E-01	3.05E-03	4.48E-02	0
PDZRN4	ENSG00000165966	12	41582250	41968392	1.12E-01	1.51E-01	3.07E-01	8.48E-05	5.27E-01	4.49E-02	0
ACSS2	ENSG00000131069	20	33459949	33515769	5.67E-01	1.84E-01	2.51E-01	5.80E-01	5.50E-01	4.49E-02	0
RHOB	ENSG00000143878	2	20646835	20649200	9.74E-01	9.65E-01	3.18E-01	1.76E-01	8.47E-02	4.51E-02	0
TIE1	ENSG00000066056	1	43766664	43788779	2.21E-01	1.01E-01	5.59E-02	3.03E-02	1.12E-01	4.51E-02	0
RHOT1	ENSG00000126858	17	30469473	30580393	1.82E-01	6.48E-03	4.01E-01	2.39E-01	3.96E-01	4.52E-02	0
STT3B	ENSG00000163527	3	31574130	31679112	6.97E-01	5.09E-02	1.59E-01	8.54E-01	5.54E-01	4.52E-02	0
NOP2	ENSG00000111641	12	6666029	6677857	5.73E-02	3.79E-02	1.01E-01	1.29E-01	1.80E-01	4.52E-02	0
CCDC85A	ENSG00000055813	2	56411258	56613308	2.30E-01	3.14E-02	1.54E-01	1.60E-01	6.69E-01	4.54E-02	0
CRIP3	ENSG00000146215	6	43267448	43276535	6.44E-04	6.43E-03	5.25E-03	5.73E-01	2.41E-02	4.56E-02	0
SRP54	ENSG00000100883	14	35451163	35498773	2.10E-02	2.71E-01	1.48E-02	1.12E-01	2.35E-01	4.56E-02	0
ZNF622	ENSG00000173545	5	16451628	16465901	8.80E-01	7.30E-02	1.06E-01	5.92E-01	3.83E-01	4.56E-02	0
IBTK	ENSG00000005700	6	82879700	82957471	1.22E-01	3.70E-01	3.20E-02	5.72E-01	2.13E-01	4.56E-02	0
ABHD16B	ENSG00000183260	20	62492566	62494341	2.59E-02	3.17E-01	1.85E-02	1.04E-01	3.49E-01	4.57E-02	0
LRCH1	ENSG00000136141	13	47127303	47327175	1.02E-01	2.63E-01	4.94E-02	2.47E-01	4.19E-01	4.57E-02	0
IP6K1	ENSG00000176095	3	49761727	49823975	3.76E-02	1.92E-01	1.96E-02	5.26E-01	7.67E-01	4.57E-02	0
FSIP1	ENSG00000150667	15	39892232	40075031	2.33E-01	7.45E-02	1.22E-01	4.50E-01	2.92E-01	4.58E-02	0
AMFR	ENSG00000159461	16	56395364	56459450	1.26E-01	3.19E-01	4.05E-02	9.13E-02	6.41E-02	4.58E-02	0
PDCD5	ENSG00000105185	19	33071974	33078358	2.00E-01	5.51E-02	8.39E-02	3.71E-02	4.48E-01	4.58E-02	0
KIF9	ENSG00000088727	3	47269516	47324941	3.02E-01	4.68E-01	5.60E-02	5.59E-01	2.47E-01	4.58E-02	0
MED31	ENSG00000108590	17	6546635	6554954	7.50E-02	3.62E-02	3.11E-02	4.76E-01	6.19E-01	4.59E-02	0
SBNO2	ENSG00000064932	19	1107636	1174282	2.47E-04	1.04E-01	5.66E-04	1.57E-01	4.75E-01	4.59E-02	0
SHOX2	ENSG00000168779	3	157814948	157824292	3.24E-01	1.77E-02	1.95E-01	1.24E-02	6.71E-01	4.59E-02	0
SWAP70	ENSG00000133789	11	9685624	9774538	1.03E-01	4.04E-01	8.81E-01	4.89E-02	9.29E-01	4.59E-02	0
CHST3	ENSG00000122863	10	73724123	73773322	4.61E-01	1.83E-01	6.07E-02	1.03E-01	4.65E-01	4.60E-02	0

Gene	Ensembl ID	CHR	START	STOP	VHD_CD Pleiotropy P	VHD_GORD Pleiotropy P	VHD_IBD Pleiotropy P	VHD_IBS Pleiotropy P	VHD_PUD Pleiotropy P	VHD_UC Pleiotropy P	d
CELA3B	ENSG00000219073	1	22303514	22325135	7.01E-02	7.85E-01	2.15E-01	1.87E-01	5.27E-01	4.61E-02	0
PGLYRP2	ENSG00000161031	19	15579456	15609767	2.64E-01	1.47E-01	5.18E-02	4.40E-01	3.16E-01	4.61E-02	0
TRIM24	ENSG00000122779	7	138145079	138274738	2.69E-02	3.29E-01	1.63E-02	4.45E-01	6.35E-01	4.63E-02	0
RPP40	ENSG00000124787	6	4994966	5004297	3.92E-02	7.70E-01	1.68E-02	4.36E-02	4.74E-01	4.64E-02	0
ETS1	ENSG00000134954	11	128328656	128457453	5.98E-03	1.37E-02	3.66E-03	2.38E-01	3.48E-01	4.65E-02	0
S100A1	ENSG00000160678	1	153600402	153604513	3.14E-01	1.76E-03	7.19E-01	1.13E-03	1.14E-01	4.65E-02	0
SLC2A4RG	ENSG00000125520	20	62371214	62374858	2.81E-02	2.44E-01	1.05E-02	9.15E-01	4.19E-01	4.67E-02	0
FAM63A	ENSG00000143409	1	150969025	150980851	1.41E-02	3.95E-02	1.56E-02	1.60E-03	8.11E-03	4.68E-02	0
B3GAT1	ENSG00000109956	11	134248398	134281812	4.25E-02	4.03E-01	1.06E-02	1.90E-02	2.96E-02	4.68E-02	0
PIK3CD	ENSG00000171608	1	9711790	9789172	4.82E-01	2.08E-02	1.76E-01	1.76E-01	5.07E-03	4.68E-02	0
INSL6	ENSG00000120210	9	5131979	5185668	5.15E-02	5.86E-01	1.49E-03	7.72E-01	4.45E-01	4.68E-02	0
CYB5R4	ENSG00000065615	6	84569362	84677143	7.65E-01	4.50E-02	1.50E-01	1.91E-01	5.73E-02	4.69E-02	0
KCNK6	ENSG00000099337	19	38810484	38819660	6.08E-01	1.41E-01	5.89E-02	5.26E-01	8.04E-01	4.69E-02	0
ABCC8	ENSG00000006071	11	17414432	17498449	2.56E-01	7.77E-02	3.35E-02	6.29E-01	7.54E-01	4.70E-02	0
ACAD10	ENSG00000111271	12	112123857	112194903	2.93E-02	5.17E-02	1.66E-02	4.40E-01	3.09E-01	4.70E-02	0
RTN3	ENSG00000133318	11	63448918	63527363	1.43E-02	1.15E-01	1.29E-02	3.09E-01	1.29E-01	4.71E-02	0
TEX101	ENSG00000131126	19	43905648	43922763	8.20E-01	7.32E-01	3.05E-01	5.53E-01	4.44E-01	4.72E-02	0
C7orf65	ENSG00000221845	7	47694842	47701246	2.98E-01	5.91E-01	7.39E-02	3.84E-01	9.31E-02	4.72E-02	0
GML	ENSG00000104499	8	143915663	143997922	4.73E-01	4.87E-02	8.52E-02	3.99E-01	1.05E-01	4.73E-02	0
ZSCAN30	ENSG00000186814	18	32831032	32870196	2.18E-01	4.90E-01	1.02E-01	1.19E-01	2.00E-01	4.73E-02	0
OR52I1	ENSG00000232268	11	4615037	4616243	3.39E-01	2.05E-01	2.76E-01	7.19E-01	6.96E-01	4.73E-02	0
ARHGDIA	ENSG00000141522	17	79825597	79829282	8.27E-02	1.31E-01	3.77E-01	1.87E-01	2.16E-01	4.74E-02	0
TXNDC17	ENSG00000129235	17	6544078	6547861	1.84E-01	1.05E-01	6.92E-02	4.64E-01	6.78E-01	4.75E-02	0
HOXA5	ENSG00000106004	7	27180671	27183287	4.01E-02	5.36E-01	2.07E-02	4.02E-01	6.94E-01	4.76E-02	0
SLCO1A2	ENSG00000084453	12	21417534	21572528	1.28E-01	1.53E-01	2.38E-02	2.42E-01	2.39E-01	4.77E-02	0
RHOD	ENSG00000173156	11	66824289	66839484	3.28E-01	8.14E-01	8.27E-02	6.26E-02	8.22E-01	4.77E-02	0
SFT2D1	ENSG00000198818	6	166733216	166756079	1.23E-01	2.44E-02	5.66E-02	5.52E-02	1.48E-01	4.77E-02	0
IFNGR1	ENSG00000027697	6	137518621	137540586	4.97E-02	5.05E-01	3.41E-01	4.27E-01	1.86E-01	4.77E-02	0
ZC3H12A	ENSG00000163874	1	37940153	37949980	1.58E-02	4.02E-01	1.34E-01	3.24E-02	3.36E-01	4.78E-02	0
CST6	ENSG00000175315	11	65779312	65780976	1.60E-01	5.91E-01	8.06E-02	1.34E-01	6.17E-01	4.79E-02	0
GALK1	ENSG00000108479	17	73747675	73761792	3.81E-01	2.08E-01	5.23E-02	5.59E-01	4.30E-01	4.79E-02	0
DEPDC1B	ENSG00000035499	5	59892739	59996017	8.08E-01	3.73E-01	6.28E-01	6.40E-01	6.81E-02	4.79E-02	0
NDUFB3	ENSG00000119013	2	201936156	201950473	2.79E-01	8.36E-02	7.16E-02	9.41E-01	6.36E-01	4.79E-02	0
AHCYL1	ENSG00000168710	1	110527308	110566357	4.81E-01	5.46E-01	9.18E-02	6.09E-01	6.35E-01	4.81E-02	0
ABCC6	ENSG00000091262	16	16242785	16317379	8.89E-01	4.54E-01	1.84E-01	2.93E-01	2.52E-01	4.81E-02	0
SEC16A	ENSG00000148396	9	139334549	139372141	1.05E-02	8.91E-01	3.02E-03	7.15E-01	7.18E-01	4.82E-02	0
SYT13	ENSG00000019505	11	45261852	45307870	5.75E-01	9.34E-01	4.74E-01	5.73E-01	6.45E-01	4.84E-02	0
RALGDS	ENSG00000160271	9	135973107	136039301	3.51E-01	9.71E-01	4.62E-02	1.27E-01	2.67E-01	4.85E-02	0
SNAPC1	ENSG00000023608	14	62229075	62263146	1.29E-01	6.07E-02	5.74E-02	7.79E-02	6.52E-02	4.86E-02	0
GTF3C2	ENSG00000115207	2	27548716	27579868	3.00E-02	3.44E-01	2.58E-02	2.02E-02	4.41E-01	4.86E-02	0
PAK4	ENSG00000130669	19	39616410	39673456	8.29E-01	1.15E-01	4.06E-01	4.13E-01	1.60E-01	4.86E-02	0
ACE	ENSG00000264813	17	61562184	61599209	3.82E-01	1.69E-01	8.59E-02	1.64E-01	4.02E-02	4.87E-02	0
ISL2	ENSG00000159556	15	76629065	76634817	3.23E-02	3.23E-01	2.42E-02	7.16E-02	7.36E-02	4.87E-02	0
LMO1	ENSG00000166407	11	8245851	8290263	8.79E-01	2.24E-01	1.60E-01	4.49E-01	2.41E-01	4.87E-02	0
C19orf33	ENSG00000167644	19	38794801	38795649	3.59E-02	1.84E-01	1.25E-02	5.03E-01	3.17E-01	4.88E-02	0
CDKN3	ENSG00000100526	14	54863567	54886936	2.38E-01	9.17E-02	1.54E-01	8.11E-01	2.55E-01	4.88E-02	0
CPOX	ENSG00000080819	3	98239976	98312567	5.06E-01	8.19E-01	1.26E-01	5.37E-01	3.67E-01	4.88E-02	0
MAP1LC3A	ENSG00000101460	20	33134658	33148149	7.55E-01	1.32E-01	3.74E-01	7.36E-01	7.12E-02	4.89E-02	0
C10orf128	ENSG00000204161	10	50362773	50396630	3.80E-03	4.38E-01	2.36E-03	4.84E-01	2.85E-01	4.90E-02	0

Gene	Ensembl ID	CHR	START	STOP	VHD_CD Pleiotropy P	VHD_GORD Pleiotropy P	VHD_IBD Pleiotropy P	VHD_IBS Pleiotropy P	VHD_PUD Pleiotropy P	VHD_UC Pleiotropy P	d
SMAD3	ENSG00000166949	15	67356101	67487533	6.27E-03	5.17E-01	4.74E-03	5.95E-01	8.11E-01	4.91E-02	0
ATAD2B	ENSG00000119778	2	23971534	24149984	2.04E-01	4.03E-02	2.94E-02	1.17E-01	1.72E-01	4.91E-02	0
INPP5B	ENSG00000204084	1	38326369	38412729	3.42E-01	1.12E-01	8.65E-02	2.19E-02	5.98E-02	4.92E-02	0
PTPLA	ENSG00000165996	10	17631958	17659376	5.24E-01	8.82E-01	1.02E-01	2.42E-01	1.99E-01	4.93E-02	0
DDX60	ENSG00000137628	4	169137444	169239958	1.84E-01	2.43E-01	1.15E-01	7.10E-01	4.68E-01	4.94E-02	0
ESR2	ENSG00000140009	14	64550950	64804830	9.98E-02	6.62E-02	5.90E-02	1.94E-01	1.44E-02	4.94E-02	0
ALDH3B2	ENSG00000132746	11	67429633	67448671	2.40E-01	1.74E-01	9.54E-02	4.26E-01	4.00E-01	4.95E-02	0
ZFAND4	ENSG00000172671	10	46111043	46168228	9.10E-02	3.65E-01	6.19E-02	1.65E-05	3.21E-01	4.95E-02	0
PLA2G5	ENSG00000127472	1	20354672	20417683	2.74E-01	2.46E-01	4.86E-01	7.77E-02	1.12E-02	4.95E-02	0
TRDMT1	ENSG00000107614	10	17184253	17244053	2.40E-02	5.37E-02	5.93E-02	2.58E-02	1.41E-01	4.96E-02	0
DDX21	ENSG00000165732	10	70715884	70744829	4.75E-01	6.27E-01	4.95E-01	1.96E-01	4.40E-02	4.97E-02	0
RSF1	ENSG00000048649	11	77371041	77532063	8.71E-02	6.87E-03	4.30E-02	1.00E-01	1.86E-01	4.99E-02	0
CCDC127	ENSG00000164366	5	196986	218330	4.40E-01	6.89E-02	9.09E-02	4.35E-01	2.41E-01	4.99E-02	0
ATAD3B	ENSG00000160072	1	1407143	1433228	2.23E-02	4.89E-01	1.87E-02	7.77E-01	8.79E-01	4.99E-02	0
CYLD	ENSG00000083799	16	50775961	50835846	4.76E-02	1.17E-01	1.17E-05	1.10E-01	8.57E-01	4.99E-02	0
UACA	ENSG00000137831	15	70946893	71055932	2.48E-01	4.14E-01	1.23E-01	6.23E-02	7.29E-02	4.99E-02	0
INTS2	ENSG00000108506	17	59942731	60005377	1.54E-01	4.13E-02	1.05E-01	4.08E-02	6.79E-02	5.00E-02	0
HDGF	ENSG00000143321	1	156711899	156736717	7.77E-02	5.39E-01	2.61E-02	7.68E-02	3.77E-01	5.00E-02	0
FST	ENSG00000134363	5	52776239	52782964	3.13E-01	1.32E-01	2.06E-01	3.03E-01	5.18E-01	5.00E-02	0
RNF114	ENSG00000124226	20	48552948	48570429	8.57E-01	7.42E-01	1.72E-01	4.27E-01	3.09E-01	5.01E-02	0
NR1H2	ENSG00000131408	19	50832910	50886239	3.39E-01	1.45E-01	7.94E-02	2.42E-01	7.58E-02	5.01E-02	0
ACADL	ENSG00000115361	2	211052663	211090215	8.56E-02	6.94E-02	1.56E-02	7.18E-02	5.42E-01	5.01E-02	0
FDX1L	ENSG00000267673	19	10416103	10426691	8.13E-03	1.01E-01	6.49E-03	6.30E-02	1.23E-01	5.01E-02	0
LMOD1	ENSG00000163431	1	201865580	201915715	1.72E-01	7.06E-02	4.95E-02	2.16E-01	8.08E-02	5.01E-02	0
THOC5	ENSG00000100296	22	29901868	29951205	4.95E-02	4.88E-01	5.39E-02	3.83E-02	5.60E-01	5.01E-02	0
CLEC12B	ENSG00000256660	12	10163226	10171218	1.92E-01	3.00E-01	4.27E-02	4.46E-01	2.16E-01	5.01E-02	0
SRFBP1	ENSG00000151304	5	121297656	121411265	3.70E-01	2.30E-02	1.10E-01	1.53E-01	1.80E-01	5.02E-02	0
TPCN2	ENSG00000162341	11	68816365	68858072	4.70E-01	7.36E-01	1.21E-01	4.89E-01	3.56E-01	5.02E-02	0
LZTFL1	ENSG00000163818	3	45864808	45957534	2.98E-01	3.53E-01	5.12E-02	3.06E-01	1.52E-01	5.02E-02	0
BARX2	ENSG00000043039	11	129245835	129322171	7.04E-02	1.58E-02	1.30E-01	2.69E-01	7.86E-02	5.02E-02	0
TMPRSS7	ENSG00000176040	3	111753690	111800116	4.22E-02	2.29E-01	5.19E-02	2.97E-01	6.00E-03	5.03E-02	0
TLR2	ENSG00000137462	4	154622652	154626851	3.26E-01	7.70E-01	1.91E-01	6.92E-01	4.18E-01	5.04E-02	0
TBC1D2	ENSG00000095383	9	100961311	101017915	1.59E-01	4.94E-02	1.29E-01	4.29E-01	2.25E-01	5.05E-02	0
OR8K1	ENSG00000150261	11	56113421	56114507	7.86E-02	1.31E-01	3.48E-02	1.37E-01	2.84E-01	5.05E-02	0
XIRP1	ENSG00000168334	3	39224701	39234087	7.91E-03	5.98E-01	2.07E-01	1.42E-01	6.44E-02	5.05E-02	0
BLM	ENSG00000197299	15	91260558	91358859	1.51E-01	8.13E-03	9.84E-02	6.32E-01	2.69E-02	5.07E-02	0
HBQ1	ENSG00000086506	16	230452	231180	3.31E-01	6.50E-01	3.47E-01	4.32E-01	5.72E-01	5.08E-02	0
JAK1	ENSG00000162434	1	65298912	65432187	5.53E-01	3.34E-01	8.81E-02	1.69E-01	1.15E-01	5.09E-02	0
HIC1	ENSG00000177374	17	1957448	1962981	3.62E-02	5.49E-02	2.76E-02	1.75E-01	3.91E-01	5.10E-02	0
IDE	ENSG00000119912	10	94211441	94333833	8.39E-03	3.11E-01	6.49E-03	1.61E-01	6.61E-01	5.11E-02	0
INPP5K	ENSG00000132376	17	1397865	1420182	3.27E-02	2.55E-01	1.00E-02	1.88E-02	3.90E-02	5.11E-02	0
ARHGEF25	ENSG00000240771	12	58003963	58013162	1.65E-01	2.89E-01	5.59E-02	5.99E-02	1.05E-01	5.11E-02	0
LRPAP1	ENSG00000163956	4	3508103	3534286	1.97E-01	4.43E-01	2.79E-02	3.41E-01	5.51E-01	5.12E-02	0
SIX1	ENSG00000126778	14	61110133	61124977	1.62E-01	8.88E-01	3.30E-01	2.17E-01	6.13E-02	5.12E-02	0
SH3PXD2A	ENSG00000107957	10	105348285	105615301	2.53E-01	4.79E-02	2.61E-01	1.36E-02	3.01E-01	5.12E-02	0
ADAMTSL3	ENSG00000156218	15	84322838	84708594	4.19E-02	7.63E-02	2.79E-02	7.58E-02	6.05E-03	5.13E-02	0
DCST2	ENSG00000163354	1	154990996	155006257	2.77E-02	2.38E-03	1.31E-02	3.65E-01	5.49E-02	5.14E-02	0
MYH7B	ENSG00000078814	20	33563206	33590240	4.50E-01	2.32E-01	2.88E-01	2.47E-01	5.76E-01	5.14E-02	0
SCAI	ENSG00000173611	9	127704887	127905785	2.37E-01	1.25E-02	9.93E-02	1.06E-03	6.17E-03	5.15E-02	0

Gene	Ensembl ID	CHR	START	STOP	VHD_CD Pleiotropy P	VHD_GORD Pleiotropy P	VHD_IBD Pleiotropy P	VHD_IBS Pleiotropy P	VHD_PUD Pleiotropy P	VHD_UC Pleiotropy P	d
ACTR2	ENSG00000138071	2	65454887	65498387	8.67E-03	2.40E-02	7.77E-03	1.13E-01	6.95E-01	5.15E-02	0
SOX2	ENSG00000181449	3	181429714	181432221	2.48E-01	3.77E-01	8.94E-02	4.36E-03	4.40E-01	5.16E-02	0
PPP2R4	ENSG00000119383	9	131873229	131911225	2.95E-03	4.35E-02	8.49E-02	1.01E-02	3.97E-02	5.16E-02	0
CD274	ENSG00000120217	9	5450503	5470566	6.11E-01	7.98E-01	1.09E-01	6.16E-01	9.60E-01	5.17E-02	0
KRTAP4-7	ENSG00000240871	17	39240459	39241396	1.38E-01	1.24E-01	1.13E-01	5.61E-02	2.71E-01	5.17E-02	0
RBM43	ENSG00000184898	2	152104454	152118393	2.53E-02	6.89E-01	1.17E-02	3.22E-01	8.15E-01	5.18E-02	0
TUSC2	ENSG00000114383	3	50357458	50365682	5.21E-02	1.27E-02	2.04E-02	1.29E-01	4.99E-02	5.19E-02	0
SPATA12	ENSG00000186451	3	57094469	57109460	4.90E-01	1.80E-01	1.43E-01	3.11E-01	2.08E-01	5.19E-02	0
CACNA1S	ENSG00000081248	1	201008642	201081694	2.72E-01	7.00E-01	4.10E-02	3.69E-01	7.56E-01	5.19E-02	0
SYCE2	ENSG00000161860	19	13009600	13030090	2.15E-01	7.16E-03	3.44E-02	1.81E-01	3.08E-01	5.19E-02	0
NMNAT2	ENSG00000157064	1	183217372	183387737	4.91E-01	4.65E-01	2.85E-01	2.22E-01	2.87E-01	5.21E-02	0
MRGPRX1	ENSG00000170255	11	18955360	18961054	1.70E-01	4.69E-01	4.41E-02	8.79E-01	5.56E-01	5.22E-02	0
YKT6	ENSG00000106636	7	44240567	44253893	3.07E-01	8.39E-01	7.20E-02	6.01E-01	1.57E-01	5.22E-02	0
C5orf34	ENSG00000172244	5	43486803	43515247	6.35E-02	8.93E-02	2.65E-02	1.67E-02	1.12E-01	5.22E-02	0
CMIP	ENSG00000153815	16	81478775	81745367	1.10E-01	6.92E-01	7.63E-02	3.70E-01	7.78E-02	5.22E-02	0
ATP12A	ENSG00000075673	13	25254549	25285921	5.66E-01	7.10E-01	2.24E-01	7.36E-01	5.53E-01	5.23E-02	0
ELFN2	ENSG00000166897	22	37764000	37823505	5.86E-01	4.07E-01	1.48E-01	3.07E-01	6.42E-01	5.24E-02	0
SMPD3	ENSG00000103056	16	68392231	68482591	5.65E-01	7.54E-01	1.42E-01	4.37E-01	4.51E-01	5.24E-02	0
SHISA7	ENSG00000187902	19	55940107	55954230	1.83E-01	3.04E-01	1.47E-01	1.85E-01	5.74E-01	5.25E-02	0
PCGF6	ENSG00000156374	10	105062553	105110891	2.98E-01	3.55E-01	1.76E-01	7.49E-01	4.41E-01	5.26E-02	0
C9orf69	ENSG00000238227	9	139006427	139010731	2.37E-01	8.24E-01	1.07E-01	4.27E-01	4.88E-01	5.26E-02	0
AAAS	ENSG00000094914	12	53701240	53718648	3.57E-02	7.10E-01	2.58E-02	5.34E-01	1.55E-01	5.27E-02	0
C17orf100	ENSG00000212734	17	6554971	6556791	2.11E-02	5.76E-02	5.12E-02	2.88E-01	2.97E-01	5.27E-02	0
RBBP9	ENSG00000089050	20	18467184	18477887	4.28E-01	2.16E-02	1.73E-01	2.97E-01	1.84E-01	5.27E-02	0
PRKAA1	ENSG00000132356	5	40759481	40798476	4.25E-06	6.85E-01	1.25E-04	1.41E-01	4.99E-03	5.28E-02	0
KLF9	ENSG00000119138	9	72999503	73029540	9.98E-01	2.89E-01	2.13E-01	7.74E-01	2.72E-01	5.28E-02	0
PPP1R27	ENSG00000182676	17	79791368	79792926	1.43E-01	2.36E-01	2.90E-01	2.44E-01	2.80E-01	5.28E-02	0
OBSCN	ENSG00000154358	1	228395831	228566577	2.89E-03	5.51E-02	1.58E-03	1.20E-02	1.00E-01	5.29E-02	0
CEACAM6	ENSG00000086548	19	42254885	42276113	8.85E-02	1.49E-01	4.25E-02	2.88E-01	2.02E-01	5.29E-02	0
FAM133B	ENSG00000234545	7	92190107	92219708	3.49E-01	4.50E-02	1.95E-01	6.25E-01	4.89E-01	5.29E-02	0
ARFGAP3	ENSG00000242247	22	43192508	43254112	2.90E-01	4.10E-01	7.36E-02	5.32E-01	4.86E-01	5.29E-02	0
ADAP1	ENSG00000105963	7	937540	995043	1.32E-01	1.25E-01	1.70E-01	1.82E-01	4.72E-03	5.29E-02	0
FCN2	ENSG00000160339	9	137772654	137779924	6.80E-03	1.45E-01	5.03E-03	1.77E-01	7.55E-02	5.31E-02	0
XPR1	ENSG00000143324	1	180601140	180859387	4.67E-01	1.99E-01	3.11E-01	5.80E-01	2.04E-01	5.31E-02	0
ZNF124	ENSG00000196418	1	247285277	247335318	1.37E-01	1.40E-01	4.84E-02	5.05E-01	8.36E-03	5.33E-02	0
TRIM64C	ENSG00000214891	11	49075266	49080664	3.08E-03	2.18E-01	6.57E-03	6.42E-02	2.62E-01	5.34E-02	0
TTBK2	ENSG00000128881	15	43030932	43213007	9.16E-02	4.41E-01	1.07E-01	3.88E-02	2.38E-02	5.34E-02	0
HORMAD2	ENSG00000176635	22	30476163	30573064	2.38E-02	9.25E-01	9.97E-03	2.34E-01	2.36E-01	5.34E-02	0
ZBTB2	ENSG00000181472	6	151685252	151712683	6.88E-03	1.77E-01	1.07E-02	1.97E-01	1.80E-01	5.35E-02	0
AQP11	ENSG00000178301	11	77300436	77321400	1.24E-01	9.44E-03	4.41E-02	1.89E-01	2.18E-01	5.35E-02	0
HSPA5	ENSG00000044574	9	127997132	128003609	3.68E-02	2.05E-01	9.69E-02	7.01E-03	3.36E-02	5.36E-02	0
TRA2A	ENSG00000164548	7	23544399	23571660	3.71E-03	3.61E-01	9.13E-03	5.41E-01	2.42E-02	5.38E-02	0
CALHM1	ENSG00000185933	10	105213144	105218645	1.74E-01	2.96E-01	8.31E-02	3.89E-01	3.86E-01	5.38E-02	0
PPP4C	ENSG00000149923	16	30087299	30096698	9.89E-01	7.61E-02	2.15E-01	5.99E-01	8.15E-01	5.40E-02	0
ADCY3	ENSG00000138031	2	25042038	25142708	4.81E-03	2.68E-01	5.61E-03	1.75E-01	7.51E-01	5.40E-02	0
SYTL2	ENSG00000137501	11	85405267	85522184	3.34E-01	7.97E-02	1.44E-01	3.14E-01	3.06E-01	5.40E-02	0
PPIL4	ENSG00000131013	6	149825869	149867174	4.60E-01	2.42E-01	5.75E-02	5.17E-02	5.55E-03	5.41E-02	0
KRTAP4-5	ENSG00000198271	17	39305178	39306054	2.68E-01	9.43E-02	6.59E-02	3.19E-01	4.01E-02	5.42E-02	0
GSTM3	ENSG00000134202	1	110276554	110284384	6.40E-02	8.33E-01	3.08E-02	3.30E-01	6.71E-01	5.42E-02	0

Gene	Ensembl ID	CHR	START	STOP	VHD_CD Pleiotropy P	VHD_GORD Pleiotropy P	VHD_IBD Pleiotropy P	VHD_IBS Pleiotropy P	VHD_PUD Pleiotropy P	VHD_UC Pleiotropy P	d
LRP2	ENSG00000081479	2	169983619	170219195	7.23E-01	3.15E-01	3.66E-01	7.69E-01	7.68E-01	5.43E-02	0
BRD9	ENSG00000028310	5	850406	892939	6.70E-01	8.78E-01	2.32E-01	1.72E-01	7.65E-02	5.44E-02	0
RUFY4	ENSG00000188282	2	218899683	218955304	1.95E-01	8.22E-01	4.55E-02	3.71E-01	4.86E-01	5.44E-02	0
MARK2	ENSG00000072518	11	63606400	63678491	8.68E-02	5.03E-02	3.35E-01	3.29E-01	6.03E-02	5.45E-02	0
ABCA7	ENSG00000064687	19	1040102	1065571	4.07E-02	4.91E-02	1.18E-01	1.88E-01	4.11E-01	5.45E-02	0
CLCN3	ENSG00000109572	4	170533784	170644824	1.27E-02	6.13E-01	3.04E-03	1.14E-01	1.10E-01	5.47E-02	0
CRABP1	ENSG00000166426	15	78632666	78640572	5.94E-01	6.77E-02	1.57E-01	1.20E-01	4.24E-02	5.47E-02	0
C2CD4C	ENSG00000183186	19	405438	409139	9.88E-03	3.66E-02	1.50E-02	2.88E-01	5.74E-01	5.47E-02	0
ECE1	ENSG00000117298	1	21543740	21671997	5.90E-02	8.66E-02	1.81E-02	3.68E-01	1.94E-02	5.48E-02	0
NRIP1	ENSG00000180530	21	16333556	16437321	8.79E-02	9.92E-03	1.91E-02	2.89E-01	2.13E-02	5.49E-02	0
DLD	ENSG00000091140	7	107531415	107572175	6.67E-01	8.83E-01	1.23E-01	4.65E-01	6.96E-01	5.49E-02	0
DNASE1L2	ENSG00000167968	16	2285817	2288712	1.68E-01	7.68E-01	5.02E-02	3.92E-02	3.18E-01	5.49E-02	0
REST	ENSG00000084093	4	57774075	57802010	4.24E-02	8.60E-02	3.71E-02	5.12E-02	1.63E-01	5.51E-02	0
ITGB1	ENSG00000150093	10	33189247	33294720	1.19E-01	4.69E-01	3.59E-02	2.30E-01	4.52E-02	5.52E-02	0
ARID5A	ENSG00000196843	2	97202480	97218375	1.75E-01	4.51E-01	8.81E-02	3.79E-01	2.65E-01	5.52E-02	0
GPHN	ENSG00000171723	14	66974125	67648520	6.31E-02	1.02E-01	3.47E-02	1.11E-01	2.76E-01	5.52E-02	0
JAKMIP3	ENSG00000188385	10	133918175	133998313	2.52E-01	4.48E-01	4.19E-01	4.41E-02	1.82E-02	5.53E-02	0
BANK1	ENSG00000153064	4	102332443	102995969	8.61E-03	2.99E-01	7.33E-03	4.19E-01	5.11E-01	5.54E-02	0
C21orf49	ENSG00000205930	21	34144411	34266043	1.92E-01	1.31E-01	4.47E-02	1.97E-01	7.01E-01	5.54E-02	0
TNFAIP6	ENSG00000123610	2	152214106	152236560	3.31E-02	1.80E-02	4.26E-01	4.48E-01	4.96E-02	5.54E-02	0
PAPOLG	ENSG00000115421	2	60983365	61029220	1.36E-03	2.85E-03	4.59E-03	2.73E-01	3.42E-01	5.54E-02	0
MED13	ENSG00000108510	17	60019966	60142643	1.04E-01	2.71E-02	1.21E-01	2.92E-02	6.17E-02	5.55E-02	0
C2orf47	ENSG00000162972	2	200820040	200873263	8.67E-02	1.70E-01	1.70E-02	3.76E-02	6.51E-01	5.56E-02	0
TES	ENSG00000135269	7	115850547	115898837	4.15E-02	6.75E-02	2.12E-02	9.92E-03	2.07E-02	5.56E-02	0
LY75	ENSG00000054219	2	160628362	160761260	3.46E-01	1.25E-01	1.26E-01	1.11E-01	1.52E-01	5.56E-02	0
DEFB115	ENSG00000215547	20	29845467	29847435	4.74E-02	1.53E-01	6.12E-02	4.07E-01	7.12E-02	5.57E-02	0
PDXP	ENSG00000241360	22	38054734	38062941	2.92E-01	4.24E-01	1.16E-01	7.37E-02	2.74E-01	5.58E-02	0
ZNF678	ENSG00000181450	1	227751244	227865144	1.54E-01	3.11E-03	4.01E-01	1.13E-01	7.54E-02	5.59E-02	0
LSP1	ENSG00000269099	13	25591541	25591675	1.49E-01	8.23E-01	3.39E-01	2.46E-01	9.82E-01	5.59E-02	0
CD164	ENSG00000135535	6	109687717	109703762	2.75E-02	3.33E-01	2.23E-02	2.98E-01	4.19E-01	5.61E-02	0
RASD2	ENSG00000100302	22	35936915	35950048	9.24E-01	6.70E-01	2.85E-01	3.69E-01	2.14E-01	5.63E-02	0
C15orf38	ENSG00000242498	15	90443159	90456188	6.47E-02	8.53E-01	3.20E-02	4.89E-01	7.82E-01	5.63E-02	0
TADA1	ENSG00000152382	1	166825747	166845564	2.34E-01	5.80E-02	1.42E-01	1.42E-01	2.42E-01	5.64E-02	0
IQCF3	ENSG00000229972	3	51851620	51864876	3.45E-01	2.23E-01	5.28E-02	1.13E-01	2.72E-01	5.64E-02	0
AKAP13	ENSG00000170776	15	85923802	86292586	7.97E-02	1.68E-01	4.36E-02	5.17E-02	3.46E-01	5.65E-02	0
OR13C5	ENSG00000255800	9	107360650	107361788	3.50E-01	7.10E-02	7.71E-02	1.53E-02	4.74E-05	5.65E-02	0
DPY19L1	ENSG00000173852	7	34968488	35077883	2.46E-01	2.14E-01	9.75E-02	2.82E-01	5.91E-01	5.65E-02	0
RCVRN	ENSG00000109047	17	9799637	9808938	1.79E-02	2.02E-02	4.51E-01	8.68E-04	2.79E-01	5.65E-02	0
SMARCE1	ENSG00000073584	17	38781214	38804760	3.99E-01	7.14E-01	7.46E-02	8.18E-01	6.21E-01	5.66E-02	0
OR51G2	ENSG00000176893	11	4935900	4936922	9.15E-03	1.60E-01	4.53E-01	1.22E-01	1.71E-01	5.66E-02	0
ASIP	ENSG00000101440	20	32782375	32857150	5.18E-01	1.15E-01	2.68E-01	4.23E-01	2.83E-01	5.67E-02	0
PAFAH1B2	ENSG00000168092	11	117014983	117047610	4.90E-01	5.60E-02	1.57E-01	1.31E-01	1.14E-01	5.68E-02	0
POU6F1	ENSG00000184271	12	51580719	51611477	1.57E-01	6.88E-02	1.03E-01	7.59E-01	4.60E-01	5.69E-02	0
OR8B2	ENSG00000204293	11	124252291	124253258	3.18E-01	3.16E-01	1.25E-01	9.42E-01	1.66E-01	5.70E-02	0
PFDN5	ENSG00000123349	12	53689075	53693234	6.43E-02	2.88E-01	3.29E-02	5.17E-01	1.47E-01	5.70E-02	0
BTBD7	ENSG00000011114	14	93703896	93799438	6.70E-02	3.30E-01	2.42E-02	4.45E-01	4.58E-01	5.70E-02	0
TAP1	ENSG00000168394	6	32812986	32821755	1.50E-01	1.79E-01	1.05E-01	4.62E-01	1.92E-02	5.70E-02	0
CYP11B2	ENSG00000179142	8	143991975	143999259	5.04E-01	3.50E-02	1.21E-01	4.47E-01	1.18E-01	5.70E-02	0
IPCEF1	ENSG00000074706	6	154475631	154677926	6.30E-01	6.58E-01	2.02E-01	6.18E-01	4.76E-01	5.70E-02	0

Gene	Ensembl ID	CHR	START	STOP	VHD_CD Pleiotropy P	VHD_GORD Pleiotropy P	VHD_IBD Pleiotropy P	VHD_IBS Pleiotropy P	VHD_PUD Pleiotropy P	VHD_UC Pleiotropy P	d
PRR5L	ENSG00000135362	11	36317838	36486754	2.02E-02	1.10E-01	1.31E-02	4.27E-01	9.72E-02	5.71E-02	0
ITGA2B	ENSG00000005961	17	42449548	42466873	1.94E-02	1.11E-02	4.51E-01	6.29E-03	5.07E-02	5.71E-02	0
ELAVL3	ENSG00000196361	19	11562141	11591861	3.73E-01	6.45E-01	6.69E-01	1.62E-01	5.78E-01	5.71E-02	0
IFNE	ENSG00000184995	9	21480841	21482312	5.73E-01	3.89E-02	3.48E-01	7.04E-01	7.95E-01	5.72E-02	0
ZNF575	ENSG00000176472	19	44029649	44040282	8.28E-02	3.21E-01	7.13E-01	1.49E-01	4.56E-02	5.72E-02	0
FCRL5	ENSG00000143297	1	157483167	157522310	3.88E-02	1.33E-01	3.55E-02	7.48E-01	1.04E-01	5.74E-02	0
SETD5	ENSG00000168137	3	9439299	9520924	2.41E-01	1.79E-02	6.29E-02	5.02E-02	8.31E-01	5.74E-02	0
SIK3	ENSG00000160584	11	116714118	116969153	6.95E-01	1.08E-01	1.96E-01	1.81E-01	1.18E-01	5.75E-02	0
HOXA10	ENSG00000253293	7	27210210	27219880	9.34E-02	6.83E-02	9.19E-02	4.94E-02	3.85E-01	5.75E-02	0
TNFSF15	ENSG00000181634	9	117546915	117568406	5.04E-02	9.88E-01	7.49E-02	6.59E-01	9.86E-01	5.76E-02	0
MUT	ENSG00000146085	6	49398073	49430904	2.75E-02	8.32E-02	4.73E-01	8.50E-02	6.15E-02	5.77E-02	0
HLX	ENSG00000136630	1	221051699	221058401	1.24E-01	7.20E-01	5.20E-02	6.89E-01	7.89E-01	5.78E-02	0
SLC13A1	ENSG00000081800	7	122753585	122840040	2.26E-01	3.27E-02	7.05E-02	9.47E-02	1.87E-01	5.78E-02	0
NFKBIE	ENSG00000146232	6	44225903	44233500	8.50E-03	3.11E-01	8.47E-03	1.85E-01	4.84E-01	5.80E-02	0
BMP4	ENSG00000125378	14	54416454	54425479	3.72E-01	9.67E-02	1.19E-01	5.26E-01	5.21E-01	5.80E-02	0
KLHDC1	ENSG00000197776	14	50159823	50219870	1.07E-02	2.43E-01	1.53E-02	9.76E-01	6.67E-01	5.80E-02	0
OR13C8	ENSG00000186943	9	107331449	107332411	2.17E-01	2.15E-02	9.95E-02	2.99E-02	1.58E-05	5.80E-02	0
KCNH4	ENSG00000089558	17	40308909	40333296	5.01E-02	5.15E-01	2.19E-02	8.33E-01	4.41E-01	5.81E-02	0
LOXL4	ENSG00000138131	10	100007447	100028007	1.20E-01	1.26E-02	2.66E-02	1.09E-02	1.69E-01	5.82E-02	0
NUP37	ENSG00000075188	12	102467967	102513902	1.43E-01	2.44E-01	5.41E-02	3.47E-01	5.06E-01	5.82E-02	0
P2RY11	ENSG00000244165	19	10222214	10226048	6.77E-02	2.74E-02	2.12E-02	3.28E-01	6.31E-02	5.83E-02	0
USP6	ENSG00000129204	17	5019733	5078329	2.27E-01	5.41E-02	9.00E-02	9.23E-03	5.65E-02	5.84E-02	0
DAOA	ENSG00000182346	13	106118216	106143383	3.79E-01	3.45E-02	1.94E-01	5.56E-01	3.89E-02	5.85E-02	0
P4HB	ENSG00000185624	17	79801035	79818570	1.20E-01	4.51E-01	3.43E-01	2.85E-01	2.06E-01	5.85E-02	0
CTU2	ENSG00000174177	16	88772871	88781794	2.42E-01	1.01E-01	4.29E-02	1.23E-01	1.76E-01	5.86E-02	0
RTKN2	ENSG00000182010	10	63942794	64028466	2.96E-02	1.40E-01	2.54E-02	5.56E-02	5.37E-01	5.87E-02	0
DENND1B	ENSG00000213047	1	197473878	197744826	6.10E-03	6.34E-02	7.20E-03	1.12E-01	5.53E-02	5.88E-02	0
FNDC3A	ENSG00000102531	13	49550048	49783915	2.54E-03	4.00E-02	1.04E-03	3.35E-01	1.52E-01	5.88E-02	0
SLC25A33	ENSG00000171612	1	9599541	9645237	6.89E-02	9.53E-02	4.44E-02	1.13E-01	7.23E-01	5.88E-02	0
C20orf62	ENSG00000168746	20	43080624	43093984	2.74E-01	4.77E-01	9.18E-02	7.27E-01	4.54E-01	5.89E-02	0
CAMK2G	ENSG00000148660	10	75572259	75634343	5.93E-02	7.23E-01	4.02E-02	1.90E-01	6.90E-01	5.89E-02	0
ATOH8	ENSG00000168874	2	85978467	86015189	4.91E-01	2.51E-02	1.35E-01	2.59E-01	1.84E-02	5.89E-02	0
TMIE	ENSG00000181585	3	46742823	46752376	1.50E-01	3.53E-03	2.70E-02	2.34E-01	8.62E-04	5.90E-02	0
PSMG1	ENSG00000183527	21	40546695	40555777	1.03E-02	2.41E-02	9.98E-03	5.51E-01	1.92E-02	5.90E-02	0
DAG1	ENSG00000173402	3	49506146	49573048	7.43E-02	6.13E-01	3.16E-02	9.03E-01	8.05E-01	5.92E-02	0
CATSPERG	ENSG00000099338	19	38826415	38861589	5.35E-01	6.27E-02	5.85E-02	5.69E-01	8.99E-01	5.92E-02	0
ABCC10	ENSG00000124574	6	43395104	43418168	2.56E-02	6.81E-01	2.82E-02	7.57E-01	6.57E-02	5.92E-02	0
LIF	ENSG00000128342	22	30636436	30642840	5.40E-01	3.82E-01	7.64E-02	8.19E-01	3.64E-01	5.92E-02	0
LAPTM5	ENSG00000162511	1	31205316	31230667	6.23E-01	8.85E-01	5.54E-02	2.72E-01	4.32E-01	5.93E-02	0
PTCH1	ENSG00000185920	9	98205262	98279339	5.66E-01	1.76E-02	1.62E-01	1.63E-01	3.95E-01	5.94E-02	0
FGFR4	ENSG00000160867	5	176513887	176525145	7.48E-01	2.87E-01	1.16E-01	7.39E-02	2.16E-01	5.94E-02	0
SLC17A6	ENSG00000091664	11	22359643	22401049	1.28E-01	6.38E-01	3.43E-01	5.95E-01	1.57E-01	5.94E-02	0
RBBP5	ENSG00000117222	1	205055270	205091143	3.16E-01	4.03E-01	7.55E-02	2.66E-01	4.61E-01	5.95E-02	0
GNGT2	ENSG00000167083	17	47280153	47287936	7.17E-02	1.75E-01	1.41E-01	2.86E-01	3.91E-01	5.96E-02	0
SLC5A1	ENSG00000100170	22	32439019	32509016	4.08E-01	4.93E-02	1.08E-01	1.98E-01	2.41E-01	5.96E-02	0
ATL3	ENSG00000184743	11	63391559	63439393	1.93E-02	1.03E-01	1.90E-02	3.55E-01	2.14E-01	5.96E-02	0
PPP1R3C	ENSG00000119938	10	93388199	93392811	3.70E-02	4.78E-01	1.02E-01	4.72E-01	5.28E-01	5.97E-02	0
SERTAD1	ENSG00000197019	19	40927499	40931932	3.22E-01	2.89E-01	1.42E-01	7.31E-02	5.08E-01	5.97E-02	0
CLK2	ENSG00000176444	1	155232659	155248282	6.79E-02	2.59E-01	3.71E-02	2.65E-02	2.10E-01	5.97E-02	0

Gene	Ensembl ID	CHR	START	STOP	VHD_CD Pleiotropy P	VHD_GORD Pleiotropy P	VHD_IBD Pleiotropy P	VHD_IBS Pleiotropy P	VHD_PUD Pleiotropy P	VHD_UC Pleiotropy P	d
SSSCA1	ENSG00000173465	11	65337901	65341413	2.97E-02	1.08E-01	1.39E-02	7.03E-01	1.59E-01	5.98E-02	0
ZNF502	ENSG00000196653	3	44754135	44765323	1.63E-01	9.71E-01	5.15E-02	2.59E-01	1.49E-01	5.99E-02	0
PPP1R12A	ENSG00000058272	12	80167343	80329240	5.03E-02	4.84E-01	4.75E-02	6.09E-01	3.07E-02	5.99E-02	0
THRA	ENSG00000126351	17	38214543	38250120	2.09E-02	3.58E-01	1.98E-02	3.57E-01	2.64E-01	6.02E-02	0
CCL20	ENSG00000115009	2	228678558	228682272	6.87E-01	8.65E-02	2.63E-01	1.36E-01	2.36E-01	6.02E-02	0
NFE2	ENSG00000123405	12	54685895	54694905	3.14E-03	4.81E-01	2.50E-03	1.75E-01	4.97E-01	6.03E-02	0
FBXO46	ENSG00000177051	19	46213887	46234162	4.37E-02	1.13E-01	1.36E-02	6.00E-01	1.10E-01	6.03E-02	0
ZNF571	ENSG00000180479	19	38045684	38085673	2.07E-01	1.16E-01	8.12E-02	2.57E-02	1.36E-01	6.04E-02	0
PUSL1	ENSG00000169972	1	1243947	1247057	1.06E-01	6.83E-01	6.89E-02	9.76E-01	8.31E-01	6.05E-02	0
USP18	ENSG00000184979	22	18632666	18660164	5.72E-01	4.08E-01	1.27E-01	4.00E-01	1.86E-01	6.05E-02	0
ANKRD17	ENSG00000132466	4	73939093	74124515	5.05E-01	6.82E-01	2.04E-01	1.95E-02	1.17E-01	6.05E-02	0
NAGLU	ENSG00000108784	17	40688190	40696467	1.18E-02	9.97E-01	1.46E-02	4.89E-01	1.53E-01	6.06E-02	0
FAM26E	ENSG00000178033	6	116832809	116845955	6.50E-01	9.40E-01	1.40E-01	8.92E-01	8.22E-01	6.06E-02	0
PDCD6IP	ENSG00000170248	3	33839844	33911194	4.26E-01	5.47E-01	1.10E-01	3.45E-01	2.63E-01	6.07E-02	0
HIST1H2BK	ENSG00000197903	6	27106073	27114619	1.47E-01	2.33E-01	4.56E-02	2.87E-01	6.70E-01	6.08E-02	0
C4orf6	ENSG00000082929	4	5526296	5529528	2.36E-01	8.65E-01	4.68E-02	5.16E-01	5.70E-01	6.10E-02	0
MYO19	ENSG00000141140	17	34851477	34899284	4.91E-01	6.71E-01	1.18E-01	5.85E-02	3.91E-01	6.11E-02	0
TBX2	ENSG00000121068	17	59477257	59486827	8.97E-01	6.98E-01	1.75E-01	5.78E-01	5.49E-02	6.11E-02	0
LETM2	ENSG00000165046	8	38243725	38267045	1.29E-01	3.13E-01	5.95E-02	4.67E-02	1.63E-01	6.11E-02	0
SH2D6	ENSG00000152292	2	85645844	85664152	7.30E-01	3.28E-01	2.89E-01	3.68E-01	5.75E-01	6.11E-02	0
USP36	ENSG00000055483	17	76783463	76837523	3.33E-01	5.50E-01	7.21E-02	9.64E-01	9.71E-01	6.12E-02	0
S100A8	ENSG00000143546	1	153362508	153363664	4.36E-01	1.84E-01	2.41E-01	5.47E-02	4.25E-01	6.12E-02	0
SUSD2	ENSG00000099994	22	24577227	24585078	4.39E-01	2.56E-01	1.03E-01	2.92E-01	7.43E-01	6.12E-02	0
BEST2	ENSG00000039987	19	12862516	12869272	1.67E-01	6.99E-01	9.66E-02	5.74E-02	6.39E-01	6.13E-02	0
HEATR5B	ENSG00000008869	2	37195526	37311485	1.13E-02	5.87E-01	1.43E-02	1.62E-01	2.68E-01	6.13E-02	0
HERPUD1	ENSG00000051108	16	56965960	56977798	2.12E-01	4.69E-01	8.37E-02	4.19E-01	3.03E-01	6.13E-02	0
DDX27	ENSG00000124228	20	47835884	47860614	2.70E-01	1.57E-01	6.19E-01	3.74E-01	5.04E-01	6.13E-02	0
CLCNKA	ENSG00000186510	1	16345370	16360545	1.32E-01	3.45E-01	1.62E-01	8.55E-01	5.00E-01	6.14E-02	0
SLC1A4	ENSG00000115902	2	65215611	65250999	2.69E-01	1.44E-01	1.31E-01	7.52E-01	2.31E-01	6.15E-02	0
APOH	ENSG00000091583	17	64208151	64252643	1.35E-01	8.16E-01	5.31E-02	2.66E-01	1.80E-01	6.15E-02	0
HLA-G	ENSG00000204632	6	29794744	29798902	2.55E-02	1.69E-01	3.27E-02	2.09E-01	1.15E-01	6.16E-02	0
FAM25A	ENSG00000188100	10	88780063	88784487	3.08E-01	2.28E-01	5.79E-02	1.87E-01	7.52E-01	6.17E-02	0
SENP6	ENSG00000112701	6	76311225	76427997	5.58E-01	4.68E-01	4.31E-01	2.83E-01	3.24E-01	6.18E-02	0
TRPC7	ENSG00000069018	5	135548999	135732730	8.42E-01	1.00E-01	4.25E-01	1.55E-01	1.26E-01	6.18E-02	0
HIST1H1B	ENSG00000184357	6	27834570	27835359	2.63E-01	4.38E-01	1.33E-01	4.96E-01	1.27E-01	6.19E-02	0
NPAT	ENSG00000149308	11	108027942	108093369	2.56E-01	3.55E-01	6.69E-02	1.16E-01	1.76E-01	6.20E-02	0
SAMD3	ENSG00000164483	6	130465460	130686570	4.71E-01	3.52E-01	2.04E-01	2.57E-01	4.75E-01	6.20E-02	0
ITLN2	ENSG00000158764	1	160914815	160924589	4.92E-02	6.17E-01	3.06E-02	6.74E-01	1.88E-01	6.21E-02	0
OR5M11	ENSG00000255223	11	56309746	56310757	7.08E-02	1.04E-01	8.63E-02	7.02E-02	6.31E-01	6.21E-02	0
AMACR	ENSG00000242110	5	33986283	34008220	7.52E-02	1.01E-02	4.32E-02	9.65E-02	4.75E-01	6.22E-02	0
ANXA10	ENSG00000109511	4	169013666	169108841	5.11E-01	4.55E-01	1.65E-01	3.48E-01	2.83E-01	6.22E-02	0
FAM71F2	ENSG00000205085	7	128312342	128326929	7.93E-02	7.89E-01	4.48E-01	1.48E-01	1.03E-01	6.22E-02	0
OR1A1	ENSG00000172146	17	3118915	3119844	8.00E-01	9.60E-01	1.27E-01	1.69E-01	6.42E-01	6.22E-02	0
CEPT1	ENSG00000134255	1	111682249	111727724	1.74E-01	9.09E-01	2.56E-02	4.11E-01	1.64E-01	6.24E-02	0
ARNT	ENSG00000143437	1	150782181	150849244	1.83E-02	3.42E-01	8.96E-03	1.14E-01	8.59E-02	6.26E-02	0
ENPP4	ENSG00000001561	6	46097730	46114436	1.49E-01	1.05E-01	2.58E-01	7.31E-01	2.47E-01	6.26E-02	0
IFITM10	ENSG00000244242	11	1753640	1771821	8.79E-03	4.29E-02	1.45E-02	1.56E-01	4.94E-01	6.26E-02	0
SPEG	ENSG00000072195	2	220299568	220363009	8.56E-01	9.98E-03	1.33E-01	1.99E-01	1.46E-02	6.27E-02	0
TRIM46	ENSG00000163462	1	155145873	155157447	4.09E-02	4.85E-01	2.91E-02	5.58E-01	1.36E-01	6.27E-02	0

Gene	Ensembl ID	CHR	START	STOP	VHD_CD Pleiotropy P	VHD_GORD Pleiotropy P	VHD_IBD Pleiotropy P	VHD_IBS Pleiotropy P	VHD_PUD Pleiotropy P	VHD_UC Pleiotropy P	d
SFRP1	ENSG00000104332	8	41119481	41167016	6.12E-03	5.30E-01	8.93E-03	4.92E-01	1.47E-01	6.28E-02	0
CISH	ENSG00000114737	3	50643921	50649262	1.24E-01	1.25E-01	2.58E-02	4.45E-01	3.65E-01	6.28E-02	0
POC5	ENSG00000152359	5	74969949	75013313	3.72E-01	7.77E-01	4.57E-01	4.46E-01	8.23E-02	6.28E-02	0
CCDC116	ENSG00000161180	22	21987005	21991616	2.88E-02	2.28E-01	1.24E-02	1.19E-01	1.41E-01	6.28E-02	0
FN1	ENSG00000115414	2	216225163	216300895	3.74E-02	1.02E-01	2.85E-02	3.93E-03	8.30E-02	6.29E-02	0
FGF10	ENSG00000070193	5	44303646	44389808	7.67E-02	7.04E-03	2.37E-02	6.89E-01	2.58E-01	6.30E-02	0
TBX20	ENSG00000164532	7	35242042	35293758	1.27E-02	9.97E-02	2.52E-02	1.09E-01	7.23E-01	6.33E-02	0
HIST1H1A	ENSG00000124610	6	26017260	26018040	6.04E-02	1.71E-01	3.04E-02	7.06E-03	5.64E-01	6.33E-02	0
RPE	ENSG00000197713	2	210867289	210886300	2.16E-01	3.83E-02	2.95E-02	1.22E-01	3.77E-01	6.33E-02	0
PIK3R5	ENSG00000141506	17	8782233	8869029	2.66E-01	2.50E-01	1.09E-01	8.54E-01	5.06E-01	6.34E-02	0
TRIM33	ENSG00000197323	1	114935399	115053781	4.03E-01	2.53E-01	5.76E-01	8.16E-02	2.79E-01	6.34E-02	0
ZSWIM6	ENSG00000130449	5	60628100	60841997	2.87E-01	4.85E-01	8.57E-02	4.17E-01	5.14E-01	6.34E-02	0
CES4A	ENSG00000172824	16	67022492	67043661	6.56E-02	2.98E-01	6.16E-02	1.02E-01	8.87E-02	6.35E-02	0
NUCB2	ENSG00000070081	11	17229700	17371521	7.61E-02	1.25E-01	3.62E-02	2.31E-02	5.17E-01	6.35E-02	0
PPIL6	ENSG00000185250	6	109711418	109762374	3.78E-02	2.17E-01	3.52E-02	3.58E-01	3.12E-01	6.35E-02	0
IKBIP	ENSG00000166130	12	99007183	99038891	1.58E-01	2.02E-01	8.10E-02	1.67E-01	2.38E-01	6.36E-02	0
SRPRB	ENSG00000144867	3	133502877	133544616	4.36E-01	2.56E-01	4.75E-02	6.39E-01	3.84E-01	6.36E-02	0
CMTM7	ENSG00000153551	3	32433163	32524559	9.39E-02	1.79E-01	1.34E-01	1.89E-01	5.10E-02	6.36E-02	0
CCNE2	ENSG00000175305	8	95891998	95908906	1.20E-01	9.61E-02	3.00E-02	1.14E-02	2.16E-01	6.37E-02	0
SGSM2	ENSG00000141258	17	2240792	2284352	1.19E-01	8.57E-02	6.74E-02	4.79E-01	1.52E-01	6.37E-02	0
TMED10	ENSG00000170348	14	75598173	75643334	3.75E-01	2.63E-01	2.69E-01	8.63E-01	1.65E-01	6.37E-02	0
ARMC12	ENSG00000157343	6	35704809	35716856	1.09E-01	5.30E-01	7.38E-02	3.95E-02	6.37E-01	6.38E-02	0
SMG8	ENSG00000167447	17	57286761	57292608	1.64E-01	3.89E-01	9.57E-02	5.00E-02	2.20E-01	6.39E-02	0
BTAF1	ENSG00000095564	10	93683526	93790082	1.76E-01	2.37E-01	3.40E-02	2.71E-01	6.07E-01	6.40E-02	0
CSRNP1	ENSG00000144655	3	39183346	39196053	8.71E-02	5.27E-01	1.08E-01	8.50E-01	1.95E-01	6.40E-02	0
UBE2U	ENSG00000177414	1	64669310	64733051	2.80E-01	8.04E-01	1.29E-01	2.10E-01	8.77E-01	6.41E-02	0
AAGAB	ENSG00000103591	15	67493371	67547533	3.69E-02	6.04E-02	2.38E-02	6.24E-01	2.43E-01	6.42E-02	0
YOD1	ENSG00000180667	1	207217194	207226325	5.46E-01	7.65E-01	1.96E-01	8.33E-01	5.80E-01	6.43E-02	0
KCNJ3	ENSG00000162989	2	155554811	155714863	1.51E-01	1.78E-01	6.58E-02	9.63E-02	6.25E-02	6.44E-02	0
ZNF18	ENSG00000154957	17	11880756	11900827	6.69E-01	1.35E-01	1.20E-01	8.13E-01	1.45E-01	6.45E-02	0
MAPK3	ENSG00000102882	16	30125426	30134827	5.17E-01	5.58E-01	1.76E-01	5.46E-01	7.02E-01	6.45E-02	0
CCDC61	ENSG00000104983	19	46498339	46524576	1.30E-01	2.90E-01	7.69E-02	4.93E-02	3.65E-01	6.46E-02	0
VPS13D	ENSG00000048707	1	12290124	12572099	3.34E-01	3.79E-01	4.02E-01	6.13E-01	5.79E-01	6.46E-02	0
APOA1	ENSG00000118137	11	116706467	116708666	3.76E-01	4.60E-01	1.32E-01	6.21E-01	1.31E-01	6.46E-02	0
CCL11	ENSG00000172156	17	32612687	32615353	1.80E-05	6.70E-01	3.50E-04	8.75E-01	8.35E-01	6.47E-02	0
SEC61A2	ENSG00000065665	10	12171636	12211960	2.74E-01	2.75E-02	5.25E-02	6.07E-01	2.06E-01	6.48E-02	0
SLC41A1	ENSG00000133065	1	205758221	205782876	2.75E-01	1.92E-01	7.03E-02	1.16E-01	5.48E-03	6.48E-02	0
PKDREJ	ENSG00000130943	22	46651560	46659219	4.82E-02	8.58E-01	4.45E-02	4.34E-01	3.90E-01	6.49E-02	0
PEAR1	ENSG00000187800	1	156863490	156886226	3.70E-01	9.01E-01	1.38E-01	6.54E-01	2.50E-01	6.49E-02	0
FBXO7	ENSG00000100225	22	32870663	32894818	9.64E-02	2.92E-01	4.58E-02	5.83E-01	4.68E-01	6.50E-02	0
CAV2	ENSG00000105971	7	115927434	116148595	1.55E-01	2.52E-02	8.06E-02	1.15E-02	1.11E-01	6.51E-02	0
DOC2A	ENSG00000149927	16	30016830	30034591	6.21E-01	4.01E-01	1.42E-01	7.22E-01	5.72E-01	6.52E-02	0
SCARF1	ENSG00000074660	17	1537152	1549041	3.64E-01	3.25E-01	3.34E-01	5.87E-02	4.00E-02	6.53E-02	0
TCP10L	ENSG00000242220	21	33948862	33959683	5.08E-01	1.26E-01	7.74E-02	5.97E-01	3.19E-01	6.53E-02	0
CALM3	ENSG00000160014	19	47104331	47114050	1.34E-01	6.68E-01	8.24E-02	4.29E-01	5.34E-01	6.53E-02	0
FAM126B	ENSG00000155744	2	201838441	201936394	3.91E-01	6.31E-02	8.93E-02	9.51E-01	6.43E-01	6.54E-02	0
OXA1L	ENSG00000155463	14	23235731	23241007	6.52E-01	2.79E-01	1.40E-01	6.15E-01	3.80E-01	6.54E-02	0
FBXW9	ENSG00000132004	19	12798867	12807457	4.43E-02	8.33E-01	3.28E-02	1.41E-01	9.93E-02	6.54E-02	0
ARHGEF2	ENSG00000116584	1	155916630	155976861	2.27E-02	9.86E-02	2.17E-01	3.07E-02	1.44E-01	6.54E-02	0

Gene	Ensembl ID	CHR	START	STOP	VHD_CD Pleiotropy P	VHD_GORD Pleiotropy P	VHD_IBD Pleiotropy P	VHD_IBS Pleiotropy P	VHD_PUD Pleiotropy P	VHD_UC Pleiotropy P	d
SDHAF1	ENSG00000205138	19	36486090	36487220	3.13E-01	3.14E-01	6.89E-02	2.47E-01	8.97E-02	6.54E-02	0
SPATA24	ENSG00000170469	5	138732252	138739777	2.88E-01	7.14E-03	3.70E-02	4.50E-01	3.36E-01	6.54E-02	0
PSMD8	ENSG00000099341	19	38865176	38874464	5.38E-01	4.84E-02	1.03E-01	2.47E-01	8.22E-01	6.54E-02	0
ATL1	ENSG00000198513	14	50999227	51099786	4.00E-01	7.10E-01	1.22E-01	4.17E-01	4.02E-01	6.55E-02	0
OR10Q1	ENSG00000180475	11	57995354	57996390	6.31E-01	7.74E-02	1.09E-01	1.27E-01	3.30E-01	6.55E-02	0
KRTAP2-1	ENSG00000212725	17	39202793	39203568	2.28E-01	6.74E-02	1.01E-01	2.27E-01	3.15E-01	6.55E-02	0
THSD4	ENSG00000187720	15	71389291	72075722	6.99E-01	2.71E-02	2.30E-01	7.64E-02	1.15E-01	6.56E-02	0
C5orf55	ENSG00000221990	5	441645	443258	8.60E-01	3.90E-01	2.46E-01	6.86E-01	8.21E-01	6.56E-02	0
GMPPA	ENSG00000144591	2	220363589	220371710	7.78E-01	3.51E-02	1.86E-01	6.20E-01	2.06E-02	6.56E-02	0
TINAG	ENSG00000137251	6	54172657	54254950	7.30E-01	4.54E-01	2.84E-01	2.10E-01	5.37E-01	6.57E-02	0
GK5	ENSG00000175066	3	141882414	141944449	7.17E-01	6.55E-01	2.88E-01	5.99E-01	4.41E-01	6.57E-02	0
KRI1	ENSG00000129347	19	10663761	10676713	1.47E-02	9.55E-02	1.63E-02	5.58E-01	2.43E-01	6.57E-02	0
BDKRB2	ENSG00000168398	14	96671016	96710666	7.83E-01	6.84E-01	5.99E-01	6.72E-01	8.85E-01	6.58E-02	0
CCR9	ENSG00000173585	3	45927996	45944667	2.61E-01	4.57E-01	7.37E-02	5.64E-01	5.04E-01	6.58E-02	0
GGA1	ENSG00000100083	22	38004481	38029571	5.99E-01	6.90E-01	3.38E-01	3.55E-02	1.23E-01	6.59E-02	0
LY6E	ENSG00000160932	8	144099399	144105249	2.98E-01	1.40E-01	3.47E-01	4.27E-01	5.37E-01	6.59E-02	0
PAIP1	ENSG00000172239	5	43526369	43557860	1.21E-01	9.39E-02	5.15E-02	4.02E-02	1.03E-01	6.59E-02	0
GCKR	ENSG00000084734	2	27719709	27746554	4.41E-03	2.27E-01	7.26E-03	1.77E-02	1.52E-01	6.60E-02	0
MOV10	ENSG00000155363	1	113215763	113243368	2.55E-02	8.47E-01	1.35E-02	8.25E-01	3.96E-01	6.60E-02	0
USP14	ENSG00000101557	18	158383	214629	4.54E-02	3.95E-01	2.19E-02	3.59E-01	4.81E-01	6.60E-02	0
STEAP1B	ENSG00000105889	7	22459063	22672544	4.54E-02	7.96E-01	4.58E-02	6.07E-01	2.13E-01	6.60E-02	0
PLA2G12B	ENSG00000138308	10	74694928	74714536	1.11E-03	2.76E-01	2.24E-03	5.13E-01	3.87E-03	6.61E-02	0
TMED2	ENSG00000086598	12	124069078	124083116	2.41E-01	6.73E-01	4.42E-02	4.54E-01	3.57E-01	6.61E-02	0
NUDT18	ENSG00000173566	8	21964383	21966932	7.68E-02	6.62E-02	3.16E-02	3.13E-04	3.10E-01	6.61E-02	0
TPD52L2	ENSG00000101150	20	62496596	62522898	1.58E-01	3.55E-01	4.85E-02	4.38E-01	3.52E-01	6.61E-02	0
KIF4B	ENSG00000226650	5	154393260	154397685	8.43E-03	1.04E-01	1.78E-02	6.04E-02	5.64E-02	6.61E-02	0
IL17REL	ENSG00000188263	22	50432942	50451088	5.18E-01	9.49E-01	1.37E-01	6.91E-01	7.32E-01	6.62E-02	0
KCTD9	ENSG00000104756	8	25285366	25315992	1.18E-01	3.16E-01	4.13E-02	2.40E-01	3.12E-01	6.64E-02	0
RGS22	ENSG00000132554	8	100973164	101143496	7.48E-01	5.17E-01	1.33E-01	1.69E-02	3.23E-01	6.64E-02	0
ZNRD1	ENSG00000066379	6	30026676	30032686	5.28E-03	5.16E-02	7.81E-02	9.86E-02	2.43E-01	6.64E-02	0
CACNG3	ENSG00000006116	16	24266874	24374122	1.10E-01	3.48E-01	1.85E-02	8.53E-01	7.33E-01	6.64E-02	0
NGDN	ENSG00000129460	14	23938897	23979071	2.88E-01	4.85E-03	4.53E-01	1.48E-01	1.87E-01	6.66E-02	0
HTT	ENSG00000197386	4	3076408	3245676	1.31E-01	1.08E-01	4.88E-02	4.02E-01	1.36E-01	6.66E-02	0
ST5	ENSG00000166444	11	8714898	8932498	2.78E-01	5.25E-01	6.48E-02	2.13E-01	5.18E-01	6.67E-02	0
SESTD1	ENSG00000187231	2	179966419	180129517	3.79E-01	4.77E-01	2.57E-01	2.41E-01	5.83E-01	6.67E-02	0
PPP6C	ENSG00000119414	9	127908852	127952218	7.92E-01	6.87E-02	2.26E-01	1.66E-03	3.09E-02	6.67E-02	0
NT5C2	ENSG00000076685	10	104845940	104953056	5.96E-02	4.58E-02	4.82E-02	1.55E-01	3.48E-01	6.68E-02	0
ETHE1	ENSG00000105755	19	44010871	44031396	3.24E-01	9.75E-02	4.32E-01	2.52E-01	4.49E-02	6.69E-02	0
PSMD6	ENSG00000163636	3	63996225	64009658	7.62E-01	6.60E-01	1.83E-01	9.90E-01	8.85E-01	6.69E-02	0
STX7	ENSG00000079950	6	132767006	132834337	5.05E-02	1.01E-01	2.51E-02	8.22E-01	2.57E-01	6.69E-02	0
GDPD1	ENSG00000153982	17	57297828	57353328	2.61E-02	1.46E-01	1.20E-01	9.31E-02	3.30E-02	6.69E-02	0
ZNF644	ENSG00000122482	1	91380859	91487829	3.43E-01	6.90E-02	9.56E-02	1.47E-01	4.84E-01	6.70E-02	0
PLVAP	ENSG00000130300	19	17462257	17488159	2.27E-01	1.79E-01	7.15E-02	4.39E-02	1.72E-01	6.71E-02	0
MAML2	ENSG00000184384	11	95709762	96076344	8.30E-01	4.67E-01	2.76E-01	7.08E-01	8.94E-01	6.71E-02	0
KANK4	ENSG00000132854	1	62702651	62785085	3.32E-02	3.88E-01	1.34E-01	1.25E-01	2.22E-01	6.71E-02	0
PLEK	ENSG00000115956	2	68592305	68624585	6.89E-02	3.69E-01	2.85E-02	2.05E-01	7.15E-01	6.71E-02	0
MTMR11	ENSG00000014914	1	149900543	149908791	1.08E-01	1.24E-01	1.78E-01	3.10E-02	3.54E-02	6.71E-02	0
IDH1	ENSG00000138413	2	209100951	209130798	7.34E-01	4.23E-01	3.92E-01	2.83E-01	5.64E-01	6.72E-02	0
FAM210B	ENSG00000124098	20	54933971	54943719	4.54E-02	7.29E-02	1.62E-02	3.83E-01	5.78E-01	6.73E-02	0

Gene	Ensembl ID	CHR	START	STOP	VHD_CD Pleiotropy P	VHD_GORD Pleiotropy P	VHD_IBD Pleiotropy P	VHD_IBS Pleiotropy P	VHD_PUD Pleiotropy P	VHD_UC Pleiotropy P	d
SPATA3	ENSG00000173699	2	231860836	231889770	3.26E-02	2.18E-01	2.69E-02	3.59E-01	6.91E-01	6.73E-02	0
APOBEC3F	ENSG00000128394	22	39436609	39449915	1.26E-01	6.51E-01	7.01E-01	7.01E-01	3.48E-01	6.74E-02	0
NCLN	ENSG00000125912	19	3185561	3209573	7.98E-02	3.53E-01	5.53E-01	5.81E-01	8.83E-01	6.75E-02	0
KRTAP3-2	ENSG00000212900	17	39155447	39156138	9.10E-01	7.93E-01	2.66E-01	2.18E-01	1.71E-01	6.75E-02	0
SUGP1	ENSG00000105705	19	19386827	19431653	4.49E-02	4.16E-02	4.37E-02	1.85E-01	3.62E-02	6.75E-02	0
RNF166	ENSG00000158717	16	88762903	88772829	2.88E-01	4.10E-02	3.71E-02	6.72E-02	2.33E-01	6.76E-02	0
ADAMTSL4	ENSG00000143382	1	150521884	150533413	1.00E-01	3.91E-01	5.02E-02	1.07E-01	3.87E-02	6.76E-02	0
CAPZA1	ENSG00000116489	1	113161795	113214241	1.57E-01	8.48E-01	6.95E-02	7.97E-01	2.88E-01	6.78E-02	0
CYP26C1	ENSG00000187553	10	94821021	94828454	7.10E-02	6.44E-02	4.09E-02	5.17E-02	8.91E-03	6.78E-02	0
B4GALT2	ENSG00000117411	1	44444615	44456840	1.56E-01	5.46E-02	8.10E-01	2.16E-01	9.23E-02	6.79E-02	0
GRID1	ENSG00000182771	10	87359312	88126250	4.09E-01	1.98E-02	1.89E-01	5.83E-02	1.16E-01	6.79E-02	0
ELMOD3	ENSG00000115459	2	85581517	85618875	6.42E-01	7.25E-01	2.53E-01	5.30E-01	1.93E-01	6.79E-02	0
YIPF5	ENSG00000145817	5	143537723	143550278	6.63E-01	1.28E-01	9.44E-02	5.96E-01	2.05E-02	6.81E-02	0
C2orf71	ENSG00000179270	2	29283842	29297127	1.53E-01	4.65E-01	1.90E-01	2.01E-01	6.15E-03	6.81E-02	0
NEK9	ENSG00000119638	14	75548822	75594047	4.93E-01	3.01E-01	3.39E-01	7.60E-01	2.02E-01	6.82E-02	0
DEFB119	ENSG00000180483	20	29964967	29978406	4.18E-02	1.88E-02	3.17E-02	7.28E-02	1.56E-01	6.84E-02	0
TRAIP	ENSG00000183763	3	49866034	49894007	7.52E-02	2.91E-01	3.62E-02	6.68E-01	7.58E-01	6.85E-02	0
PSG3	ENSG00000221826	19	43225790	43244721	4.59E-01	4.91E-01	2.74E-01	1.49E-01	6.45E-01	6.85E-02	0
AMT	ENSG00000145020	3	49454211	49460186	6.67E-04	4.44E-01	2.53E-03	6.15E-01	9.12E-01	6.86E-02	0
RIBC2	ENSG00000128408	22	45809572	45828376	3.55E-01	3.48E-01	1.32E-01	5.59E-01	1.08E-01	6.87E-02	0
RAB11FIP3	ENSG00000090565	16	475619	573011	1.36E-02	9.35E-03	1.52E-02	3.98E-02	3.12E-02	6.87E-02	0
C1orf35	ENSG00000143793	1	228288427	228293112	1.15E-01	3.37E-01	1.23E-01	1.46E-02	7.00E-01	6.88E-02	0
KIAA1109	ENSG00000138688	4	123073488	123283913	8.16E-03	1.54E-01	1.24E-02	7.88E-03	8.87E-01	6.88E-02	0
TMEM104	ENSG00000109066	17	72772622	72835918	4.36E-01	5.69E-01	2.52E-01	6.42E-02	1.58E-01	6.88E-02	0
ZBTB17	ENSG00000116809	1	16268364	16302627	1.81E-01	7.22E-01	1.12E-01	4.33E-01	6.63E-01	6.89E-02	0
ORC5	ENSG00000164815	7	103766788	103848495	6.03E-02	2.35E-01	7.58E-01	2.06E-01	4.57E-01	6.89E-02	0
HENMT1	ENSG00000162639	1	109190912	109204148	6.13E-01	9.40E-02	7.73E-01	4.99E-02	1.61E-02	6.89E-02	0
PTBP3	ENSG00000119314	9	114980715	115095947	1.46E-01	1.33E-01	4.42E-02	4.27E-01	1.25E-01	6.90E-02	0
ZKSCAN4	ENSG00000187626	6	28212401	28227011	8.14E-02	4.67E-02	1.08E-01	2.54E-01	2.45E-01	6.90E-02	0
ZFP64	ENSG00000020256	20	50668202	50820847	3.92E-01	7.35E-01	8.54E-02	8.36E-01	9.32E-01	6.90E-02	0
BRWD1	ENSG00000185658	21	40556102	40693485	1.97E-02	6.89E-02	1.42E-02	4.88E-01	9.37E-03	6.90E-02	0
ABI3	ENSG00000108798	17	47287589	47300587	2.61E-01	2.12E-01	3.11E-01	2.87E-01	3.19E-01	6.91E-02	0
CYP2D6	ENSG00000100197	22	42522501	42526908	1.23E-01	8.18E-01	5.96E-02	2.91E-01	4.15E-01	6.91E-02	0
GGCX	ENSG00000115486	2	85771846	85788670	3.61E-02	1.30E-02	1.52E-02	8.47E-02	1.08E-01	6.92E-02	0
TRIP13	ENSG00000071539	5	892758	919472	5.76E-01	9.76E-01	2.27E-01	1.83E-01	9.92E-02	6.92E-02	0
MAP2K4	ENSG00000065559	17	11924141	12047147	8.82E-01	1.74E-01	2.36E-01	7.69E-01	2.04E-01	6.92E-02	0
RP1	ENSG00000104237	8	55528627	55543394	6.99E-01	3.18E-01	2.61E-01	3.55E-01	1.73E-01	6.92E-02	0
TBCB	ENSG00000105254	19	36605191	36616849	3.70E-01	3.71E-01	9.91E-02	1.75E-01	9.79E-02	6.92E-02	0
CXCR2	ENSG00000180871	2	218990012	219001976	1.26E-01	7.31E-01	4.03E-02	6.74E-01	5.62E-01	6.93E-02	0
LGI4	ENSG00000153902	19	35615417	35633355	5.85E-02	4.94E-01	2.27E-02	3.87E-01	5.05E-01	6.93E-02	0
SPECC1	ENSG00000128487	17	19912657	20222339	2.55E-01	2.65E-01	4.30E-02	3.50E-02	8.16E-02	6.94E-02	0
DYNLRB1	ENSG00000125971	20	33104214	33128762	4.55E-01	2.46E-02	8.65E-02	3.43E-01	5.95E-02	6.95E-02	0
CFHR4	ENSG00000134365	1	196819371	196888102	1.60E-01	7.82E-01	3.41E-01	2.16E-01	3.99E-01	6.95E-02	0
TEKT1	ENSG00000167858	17	6692452	6735080	1.95E-01	8.06E-02	7.39E-01	9.03E-02	7.58E-01	6.96E-02	0
FOLH1	ENSG00000086205	11	49168187	49230222	1.54E-02	2.28E-02	9.82E-03	1.00E-01	3.19E-04	6.96E-02	0
TP53I3	ENSG00000115129	2	24300303	24308731	6.33E-02	6.01E-01	3.54E-02	3.06E-02	9.66E-02	6.97E-02	0
ZBTB7C	ENSG00000184828	18	45553044	45937123	1.99E-01	2.16E-02	9.21E-02	1.97E-01	5.32E-01	6.98E-02	0
DLEU1	ENSG00000176124	13	50656307	51297372	3.11E-01	3.47E-01	1.36E-01	4.82E-01	9.39E-01	6.99E-02	0
ROGDI	ENSG00000067836	16	4846969	4852951	7.30E-02	1.34E-01	5.55E-02	1.48E-01	3.26E-02	7.00E-02	0

Gene	Ensembl ID	CHR	START	STOP	VHD_CD Pleiotropy P	VHD_GORD Pleiotropy P	VHD_IBD Pleiotropy P	VHD_IBS Pleiotropy P	VHD_PUD Pleiotropy P	VHD_UC Pleiotropy P	d
PCNP	ENSG00000081154	3	101292939	101313281	7.64E-03	1.78E-01	1.20E-02	8.22E-02	2.73E-02	7.00E-02	0
DEFB121	ENSG00000204548	20	29992648	30000641	2.64E-02	1.72E-02	1.55E-02	1.06E-01	1.67E-01	7.00E-02	0
PTCH2	ENSG00000117425	1	45285516	45308735	2.30E-01	6.97E-01	6.61E-02	8.19E-01	2.10E-01	7.01E-02	0
ZNF669	ENSG00000188295	1	247263264	247267674	1.28E-01	1.09E-01	5.61E-02	3.56E-01	3.63E-01	7.02E-02	0
BNIP2	ENSG00000140299	15	59951345	59981733	1.86E-01	2.51E-01	5.30E-02	5.63E-01	4.60E-01	7.02E-02	0
OR5H15	ENSG00000233412	3	97887544	97888485	4.93E-01	8.60E-01	1.34E-01	2.08E-01	4.82E-01	7.04E-02	0
MAU2	ENSG00000129933	19	19431490	19469563	5.57E-02	2.40E-02	2.72E-02	1.32E-01	2.42E-02	7.04E-02	0
ZNF576	ENSG00000124444	19	44100544	44105309	9.17E-01	7.88E-01	2.59E-01	1.66E-02	1.02E-01	7.04E-02	0
FBXL7	ENSG00000183580	5	15500305	15939900	2.27E-01	7.76E-01	1.47E-01	4.01E-01	2.14E-01	7.04E-02	0
STRA8	ENSG00000146857	7	134916731	134943244	6.41E-01	7.45E-01	3.65E-01	7.32E-01	5.56E-01	7.05E-02	0
MPL	ENSG00000117400	1	43803478	43818443	2.16E-01	5.45E-02	8.35E-02	8.47E-02	2.56E-01	7.05E-02	0
FNDC1	ENSG00000164694	6	159590429	159693141	3.22E-02	3.22E-01	2.21E-02	4.61E-01	6.13E-01	7.06E-02	0
GLUD1	ENSG00000148672	10	88810243	88854623	2.41E-01	3.22E-01	4.48E-02	3.57E-01	2.11E-01	7.06E-02	0
WNT11	ENSG00000085741	11	75897369	75921780	3.21E-01	4.61E-01	6.71E-02	3.90E-01	2.82E-01	7.06E-02	0
ENDOD1	ENSG00000149218	11	94822974	94865809	1.24E-02	1.38E-01	8.68E-03	1.31E-02	9.25E-01	7.06E-02	0
CLEC3B	ENSG00000163815	3	45043040	45077563	6.66E-01	3.45E-01	1.75E-01	5.82E-01	1.65E-01	7.07E-02	0
BPIFB6	ENSG00000167104	20	31619454	31631853	8.05E-01	8.52E-02	1.12E-01	7.26E-01	2.90E-02	7.07E-02	0
WDR38	ENSG00000136918	9	127615755	127620160	2.17E-01	4.61E-03	8.30E-02	3.93E-02	7.62E-03	7.07E-02	0
RPRD2	ENSG00000163125	1	150335567	150449042	2.42E-01	1.75E-01	1.92E-01	3.11E-01	8.25E-01	7.09E-02	0
BPIFA2	ENSG00000131050	20	31749574	31769218	5.12E-01	5.02E-02	1.46E-01	1.99E-01	3.50E-01	7.09E-02	0
MAP2K6	ENSG00000108984	17	67410839	67539472	2.48E-01	5.14E-02	1.50E-01	2.05E-01	1.11E-01	7.11E-02	0
KLF16	ENSG00000129911	19	1852399	1863567	6.09E-02	2.81E-01	2.90E-02	6.61E-02	2.15E-01	7.11E-02	0
KIAA1033	ENSG00000136051	12	105501102	105562912	6.48E-01	2.37E-01	2.18E-01	6.97E-02	2.06E-01	7.13E-02	0
LRRN4CL	ENSG00000177363	11	62453874	62457371	3.30E-01	1.41E-01	2.30E-01	3.63E-01	7.73E-01	7.13E-02	0
EIF3G	ENSG00000130811	19	10225693	10230596	1.75E-01	2.79E-01	5.87E-02	8.89E-01	2.12E-01	7.13E-02	0
C7orf26	ENSG00000146576	7	6629648	6648357	4.30E-02	6.82E-01	3.84E-02	7.98E-01	7.47E-01	7.15E-02	0
SHE	ENSG00000169291	1	154442248	154474589	2.40E-03	1.06E-03	1.56E-02	1.51E-02	1.30E-02	7.15E-02	0
MARS2	ENSG00000247626	2	198570087	198573113	5.26E-01	2.79E-01	2.14E-01	8.64E-01	7.10E-01	7.15E-02	0
PSMD7	ENSG00000103035	16	74330673	74340186	3.25E-01	9.82E-01	4.93E-02	9.94E-01	3.63E-01	7.16E-02	0
HAUS5	ENSG00000249115	19	36103646	36116251	3.34E-01	5.90E-01	2.46E-01	1.07E-01	6.16E-02	7.16E-02	0
CDH1	ENSG00000039068	16	68771128	68869451	4.27E-01	6.08E-01	1.57E-01	4.30E-01	4.10E-01	7.17E-02	0
CPT1A	ENSG00000110090	11	68522088	68611878	1.30E-02	3.06E-01	1.98E-02	1.06E-02	3.90E-01	7.18E-02	0
INTS8	ENSG00000164941	8	95825539	95893974	2.24E-01	1.02E-01	6.04E-02	1.19E-02	1.77E-01	7.18E-02	0
EPGN	ENSG00000182585	4	75174190	75181024	2.25E-01	1.21E-01	2.12E-01	9.21E-03	2.76E-01	7.18E-02	0
ATP8B2	ENSG00000143515	1	154298029	154323783	3.90E-05	1.06E-02	1.54E-03	1.18E-03	2.48E-01	7.19E-02	0
ACRBP	ENSG00000111644	12	6747241	6756626	3.77E-01	8.37E-02	2.74E-01	9.71E-02	5.76E-01	7.19E-02	0
TRIM31	ENSG00000204616	6	30070674	30080883	5.29E-06	1.99E-03	6.21E-04	8.57E-04	2.41E-01	7.20E-02	0
ALB	ENSG00000163631	4	74262831	74287129	4.07E-01	9.28E-01	1.05E-01	4.19E-02	4.48E-02	7.21E-02	0
CAMKK2	ENSG00000110931	12	121675497	121736111	6.15E-01	1.79E-02	2.41E-01	1.88E-01	2.74E-01	7.21E-02	0
UNC119B	ENSG00000175970	12	121148238	121161443	1.27E-01	4.27E-02	1.97E-01	1.02E-01	3.31E-01	7.21E-02	0
DAB2IP	ENSG00000136848	9	124329336	124547809	8.49E-02	8.58E-01	3.24E-02	1.62E-01	5.29E-01	7.22E-02	0
IPO4	ENSG00000196497	14	24649425	24658170	5.79E-02	8.19E-01	3.88E-02	4.07E-01	1.35E-01	7.22E-02	0
CAMK2D	ENSG00000145349	4	114372188	114683083	7.53E-02	5.84E-01	3.03E-02	1.71E-02	6.89E-02	7.23E-02	0
FCGRT	ENSG00000104870	19	50010073	50029590	1.08E-01	5.61E-01	5.06E-01	7.14E-01	5.01E-01	7.24E-02	0
ZNF660	ENSG00000144792	3	44619715	44641186	1.94E-01	7.56E-01	8.43E-02	2.32E-01	7.98E-02	7.25E-02	0
MB21D1	ENSG00000164430	6	74123238	74161999	3.48E-01	2.06E-01	1.98E-01	9.84E-02	1.96E-01	7.26E-02	0
APH1A	ENSG00000117362	1	150237804	150241980	4.51E-01	2.21E-02	1.24E-01	4.04E-02	2.00E-01	7.26E-02	0
SPRYD7	ENSG00000123178	13	50486842	50510626	4.67E-01	1.11E-01	1.59E-01	1.86E-01	4.94E-01	7.26E-02	0
PGP	ENSG00000184207	16	2261998	2264808	1.22E-01	2.49E-01	2.49E-01	1.98E-01	1.78E-01	7.27E-02	0

Gene	Ensembl ID	CHR	START	STOP	VHD_CD Pleiotropy P	VHD_GORD Pleiotropy P	VHD_IBD Pleiotropy P	VHD_IBS Pleiotropy P	VHD_PUD Pleiotropy P	VHD_UC Pleiotropy P	d
LRRK2	ENSG00000188906	12	40590546	40763087	3.37E-06	7.61E-02	5.13E-05	1.47E-01	2.90E-02	7.27E-02	0
HLA-A	ENSG00000206503	6	29909037	29913661	4.35E-03	1.54E-02	1.35E-01	3.16E-03	2.36E-02	7.28E-02	0
B4GALT5	ENSG00000158470	20	48249482	48330415	8.39E-01	1.29E-01	2.83E-01	1.76E-01	3.57E-02	7.28E-02	0
FXYD1	ENSG00000266964	19	35629712	35634013	1.07E-01	8.07E-01	4.12E-02	7.02E-01	7.38E-01	7.29E-02	0
HCFC2	ENSG00000111727	12	104458235	104498639	1.26E-01	9.96E-02	4.88E-02	5.59E-01	3.97E-01	7.29E-02	0
FAM53C	ENSG00000120709	5	137667624	137685416	7.93E-02	9.83E-03	2.02E-02	5.83E-02	1.26E-01	7.30E-02	0
IFT140	ENSG00000187535	16	1560428	1662111	9.33E-02	4.79E-01	6.82E-01	8.76E-01	5.69E-01	7.30E-02	0
CRTC2	ENSG00000160741	1	153920145	153931101	1.46E-01	1.99E-01	7.21E-01	1.67E-01	5.94E-01	7.31E-02	0
ZNF771	ENSG00000179965	16	30418618	30440920	8.20E-01	1.07E-01	3.69E-01	3.42E-01	3.48E-01	7.32E-02	0
PADI2	ENSG00000117115	1	17393256	17445948	4.77E-01	3.42E-01	2.00E-01	4.62E-01	1.32E-01	7.33E-02	0
H6PD	ENSG00000049239	1	9294834	9331396	6.15E-02	7.06E-01	6.34E-02	4.85E-02	2.73E-01	7.33E-02	0
C9orf116	ENSG00000160345	9	138387027	138393580	1.61E-01	6.18E-01	8.11E-02	3.67E-01	7.58E-01	7.33E-02	0
SPIB	ENSG00000269404	19	50922195	50934570	2.08E-01	4.55E-01	1.07E-01	4.51E-01	5.63E-01	7.33E-02	0
AASS	ENSG00000008311	7	121715701	121784334	9.29E-02	5.32E-01	2.01E-01	5.85E-01	1.82E-01	7.33E-02	0
ZNF445	ENSG00000185219	3	44481262	44519162	6.70E-02	8.82E-01	5.37E-02	8.53E-01	2.61E-01	7.34E-02	0
BLVRB	ENSG00000090013	19	40953696	40971747	6.95E-01	1.05E-01	1.42E-01	7.67E-02	7.53E-01	7.34E-02	0
TYMP	ENSG00000025708	22	50964181	50968485	6.43E-02	9.83E-01	2.71E-02	7.03E-01	2.44E-01	7.34E-02	0
DHX38	ENSG00000140829	16	72127461	72146811	6.80E-02	4.34E-01	4.43E-02	3.57E-01	2.30E-01	7.35E-02	0
ANP32B	ENSG00000136938	9	100745643	100778225	3.19E-01	1.28E-01	1.61E-01	4.04E-01	5.30E-01	7.36E-02	0
ROCK1	ENSG00000067900	18	18526867	18691812	3.42E-01	7.12E-01	2.73E-01	1.51E-01	7.52E-02	7.37E-02	0
QPCT	ENSG00000115828	2	37571717	37600465	2.40E-01	4.47E-01	8.98E-02	1.14E-01	6.52E-02	7.37E-02	0
ZNF648	ENSG00000179930	1	182023705	182030847	2.72E-01	3.39E-01	6.43E-02	2.16E-01	5.41E-02	7.37E-02	0
CLK1	ENSG00000013441	2	201717732	201729422	4.70E-01	6.03E-02	9.45E-02	7.95E-01	8.42E-01	7.38E-02	0
C10orf35	ENSG00000171224	10	71390007	71393352	2.61E-02	6.87E-02	2.14E-02	8.99E-02	7.40E-01	7.39E-02	0
RICTOR	ENSG00000164327	5	38938021	39074510	3.85E-02	9.48E-01	5.44E-02	4.60E-01	6.25E-01	7.39E-02	0
ADCY10	ENSG00000143199	1	167778625	167883453	2.68E-01	3.53E-01	1.49E-01	4.70E-02	7.18E-02	7.40E-02	0
BCL2L12	ENSG00000126453	19	50168823	50177173	5.13E-02	2.80E-01	3.74E-02	8.81E-01	1.83E-01	7.41E-02	0
HINT1	ENSG00000169567	5	130494720	130507428	1.68E-02	1.55E-02	1.50E-02	2.42E-01	3.93E-01	7.43E-02	0
OR13D1	ENSG00000179055	9	107456660	107457766	3.14E-02	1.55E-01	3.62E-02	7.77E-02	1.66E-03	7.43E-02	0
NDUFS5	ENSG00000168653	1	39491990	39500308	4.21E-01	3.06E-01	2.08E-01	4.21E-01	2.16E-01	7.44E-02	0
MTTP	ENSG00000138823	4	100484918	100545156	5.66E-01	5.27E-01	1.69E-01	2.06E-01	5.24E-01	7.44E-02	0
TRIP6	ENSG00000087077	7	100464760	100471076	4.92E-02	6.46E-01	2.26E-02	5.48E-01	8.70E-01	7.46E-02	0
ADAMTS13	ENSG00000160323	9	136279478	136324508	3.95E-02	7.23E-01	3.59E-02	5.00E-01	1.39E-01	7.47E-02	0
IRX1	ENSG00000170549	5	3596168	3601517	2.93E-01	7.30E-02	5.69E-02	2.13E-02	1.50E-01	7.47E-02	0
RAD51B	ENSG00000182185	14	68286496	69196935	3.87E-01	2.36E-01	9.07E-02	2.84E-01	4.32E-01	7.48E-02	0
ANKRD33B	ENSG00000164236	5	10564442	10650308	2.83E-01	8.28E-01	1.38E-01	3.27E-01	2.40E-01	7.48E-02	0
OR2J3	ENSG00000204701	6	29079668	29080603	3.25E-02	3.54E-04	3.23E-01	1.83E-01	1.34E-02	7.48E-02	0
MEFV	ENSG00000103313	16	3292028	3306627	2.51E-03	3.50E-01	1.18E-01	2.06E-02	5.40E-01	7.48E-02	0
DNAJC5	ENSG00000101152	20	62526518	62567384	4.84E-02	7.63E-02	1.35E-01	2.01E-01	9.13E-02	7.48E-02	0
SPG11	ENSG00000104133	15	44854894	44955876	4.84E-01	7.30E-01	1.83E-01	3.78E-02	9.29E-01	7.49E-02	0
PRRG2	ENSG00000126460	19	50083903	50094272	1.00E-01	2.04E-01	3.88E-02	1.90E-01	4.42E-01	7.51E-02	0
ZC3H12D	ENSG00000178199	6	149768794	149806197	2.64E-01	3.64E-01	9.78E-02	3.20E-01	7.67E-02	7.51E-02	0
TRIB1	ENSG00000173334	8	126442563	126450647	3.73E-03	2.59E-01	4.80E-03	2.69E-01	4.08E-02	7.51E-02	0
OR6P1	ENSG00000186440	1	158532441	158533394	4.15E-01	8.24E-01	1.28E-01	2.47E-01	6.67E-01	7.51E-02	0
NRIP3	ENSG00000175352	11	9002123	9025596	1.17E-01	5.30E-01	5.90E-02	2.97E-02	4.44E-01	7.51E-02	0
DDC	ENSG00000132437	7	50526134	50633154	7.32E-01	4.51E-01	2.20E-01	2.77E-01	1.20E-01	7.51E-02	0
FBXO4	ENSG00000151876	5	41925356	41941845	1.11E-02	1.11E-01	9.92E-03	9.16E-02	3.33E-01	7.52E-02	0
ANKRD42	ENSG00000137494	11	82904781	82971736	7.36E-02	2.76E-02	7.09E-01	2.40E-01	7.26E-01	7.52E-02	0
HLA-F	ENSG00000204642	6	29690552	29706305	1.39E-01	2.29E-01	1.18E-01	2.30E-01	3.60E-01	7.53E-02	0

Gene	Ensembl ID	CHR	START	STOP	VHD_CD Pleiotropy P	VHD_GORD Pleiotropy P	VHD_IBD Pleiotropy P	VHD_IBS Pleiotropy P	VHD_PUD Pleiotropy P	VHD_UC Pleiotropy P	d
CCND3	ENSG00000112576	6	41902671	42018095	7.53E-02	7.81E-01	3.58E-02	2.53E-01	4.43E-01	7.54E-02	0
C9orf156	ENSG00000136932	9	100666771	100684852	6.14E-01	3.20E-01	3.85E-01	7.27E-01	4.14E-01	7.55E-02	0
WDR93	ENSG00000140527	15	90234028	90286869	6.80E-02	2.53E-02	4.46E-01	2.80E-01	6.16E-01	7.55E-02	0
VGLL2	ENSG00000170162	6	117586721	117594728	1.54E-01	6.05E-01	8.42E-01	6.11E-01	1.00E-01	7.55E-02	0
TEN1	ENSG00000257949	17	73975301	73996667	5.72E-01	4.61E-01	1.44E-01	5.44E-01	2.90E-01	7.56E-02	0
CDKL4	ENSG00000205111	2	39402787	39456729	5.83E-01	4.39E-01	1.11E-01	1.28E-01	3.26E-01	7.56E-02	0
DERL2	ENSG00000072849	17	5374571	5390131	2.03E-01	4.81E-01	1.75E-01	5.41E-01	1.89E-01	7.56E-02	0
PBLD	ENSG00000108187	10	70042417	70092806	1.29E-01	1.49E-01	5.02E-02	6.97E-02	4.26E-01	7.57E-02	0
SIRPG	ENSG00000089012	20	1609798	1638425	7.20E-01	5.91E-01	3.74E-01	5.04E-02	3.04E-01	7.57E-02	0
MAPKAPK5	ENSG00000089022	12	112279782	112334343	4.83E-01	3.14E-02	1.45E-01	6.30E-01	4.49E-01	7.57E-02	0
KRTAP2-3	ENSG00000212724	17	39215495	39216344	5.95E-01	6.60E-02	1.79E-01	3.14E-01	5.94E-01	7.58E-02	0
PVALB	ENSG00000100362	22	37196728	37215523	8.97E-03	8.10E-02	2.68E-02	4.72E-02	2.11E-02	7.58E-02	0
HMGA1	ENSG00000137309	6	34204650	34214008	3.13E-01	6.15E-02	1.33E-01	8.40E-02	6.45E-01	7.58E-02	0
PHIP	ENSG00000146247	6	79645584	79787953	3.75E-01	1.30E-03	9.98E-02	5.67E-01	2.63E-01	7.58E-02	0
MYBPH	ENSG00000133055	1	203136939	203144941	4.05E-01	1.36E-01	1.24E-01	1.27E-01	1.49E-02	7.59E-02	0
ADA	ENSG00000196839	20	43248163	43280874	7.84E-02	5.23E-01	4.75E-02	7.21E-01	6.47E-01	7.60E-02	0
CHD2	ENSG00000173575	15	93426526	93571237	4.69E-01	4.25E-01	1.57E-01	2.02E-01	1.81E-01	7.60E-02	0
SLC12A9	ENSG00000146828	7	100424442	100464631	9.13E-02	7.81E-01	3.18E-02	5.23E-01	8.81E-01	7.61E-02	0
EVPL	ENSG00000167880	17	74000583	74023533	1.56E-01	3.44E-01	2.34E-01	6.71E-01	3.02E-01	7.63E-02	0
SEMA3F	ENSG00000001617	3	50192478	50226508	6.79E-02	2.13E-02	3.52E-02	1.57E-01	1.40E-01	7.64E-02	0
IDI1	ENSG00000067064	10	1085848	1095110	3.96E-01	3.65E-01	2.39E-01	3.61E-01	5.24E-02	7.66E-02	0
TMEM241	ENSG00000134490	18	20777108	21017925	2.98E-01	3.54E-02	1.23E-01	5.19E-02	1.29E-01	7.67E-02	0
GALC	ENSG00000054983	14	88304164	88460009	9.57E-04	4.55E-01	7.05E-03	8.22E-01	6.60E-01	7.68E-02	0
SLC6A4	ENSG00000108576	17	28521337	28563020	5.79E-01	1.16E-01	1.73E-01	4.54E-01	3.54E-01	7.68E-02	0
GNB2	ENSG00000172354	7	100271154	100276797	4.32E-01	9.56E-01	1.27E-01	5.01E-01	9.59E-01	7.68E-02	0
IAH1	ENSG00000134330	2	9613787	9636672	9.84E-02	8.33E-01	3.96E-02	3.96E-01	3.22E-01	7.68E-02	0
VIL1	ENSG00000127831	2	219283815	219318018	5.26E-03	7.30E-02	1.11E-01	3.68E-02	7.42E-02	7.69E-02	0
SYNGR4	ENSG00000105467	19	48867652	48879633	2.53E-01	1.61E-01	4.38E-01	2.03E-01	1.83E-01	7.70E-02	0
ZPBP	ENSG00000042813	7	49890017	50160925	1.52E-01	5.85E-01	1.02E-01	5.96E-02	5.80E-01	7.71E-02	0
C5orf63	ENSG00000164241	5	126378250	126409184	1.69E-01	1.06E-02	5.77E-02	4.43E-01	3.65E-01	7.72E-02	0
RPL30	ENSG00000156482	8	99037079	99058697	5.54E-01	4.77E-02	1.18E-01	6.36E-01	9.41E-03	7.72E-02	0
OR8G5	ENSG00000255298	11	124134723	124135763	6.16E-03	4.79E-02	1.11E-01	7.46E-02	4.28E-03	7.73E-02	0
TNRC6B	ENSG00000100354	22	40440821	40731812	6.46E-02	1.21E-01	1.69E-01	3.31E-01	4.41E-01	7.73E-02	0
SLC34A1	ENSG00000131183	5	176806236	176825849	3.50E-01	5.13E-01	7.55E-02	7.35E-01	7.74E-02	7.73E-02	0
NOSIP	ENSG00000142546	19	50058968	50093519	3.98E-02	1.85E-01	2.40E-02	1.76E-01	5.91E-01	7.74E-02	0
PTPRA	ENSG00000132670	20	2844830	3019722	2.15E-01	5.30E-01	2.68E-01	6.29E-02	1.89E-01	7.74E-02	0
GTPBP3	ENSG00000130299	19	17445729	17453544	4.58E-01	1.53E-01	1.04E-01	1.10E-02	2.37E-02	7.75E-02	0
SLC22A14	ENSG00000144671	3	38323785	38360066	2.15E-01	7.60E-02	9.27E-02	8.19E-02	6.23E-01	7.75E-02	0
OSCP1	ENSG00000116885	1	36881428	36916086	2.44E-02	4.31E-02	3.48E-01	3.73E-01	3.34E-02	7.75E-02	0
ZDHHC24	ENSG00000174165	11	66288108	66313709	1.88E-01	6.19E-01	2.79E-01	2.39E-01	6.48E-01	7.76E-02	0
ERCC1	ENSG00000012061	19	45910591	45982086	2.07E-02	2.90E-02	2.10E-01	1.12E-01	2.00E-02	7.77E-02	0
CDK15	ENSG00000138395	2	202655184	202760273	1.98E-01	4.75E-01	8.14E-02	3.47E-01	4.57E-01	7.78E-02	0
SEL1L	ENSG00000071537	14	81937893	82000205	1.54E-01	2.88E-01	7.74E-02	4.03E-01	1.35E-01	7.80E-02	0
VSTM2A	ENSG00000170419	7	54610018	54638773	6.24E-02	3.05E-01	2.99E-02	6.65E-02	8.86E-02	7.81E-02	0
PROSER1	ENSG00000120685	13	39584003	39612252	2.15E-01	7.98E-02	1.05E-01	2.34E-01	3.13E-01	7.81E-02	0
FYN	ENSG00000010810	6	111981535	112194655	2.81E-01	7.43E-02	4.57E-01	2.69E-01	8.73E-01	7.82E-02	0
DSTYK	ENSG00000133059	1	205111632	205180727	4.90E-01	6.11E-01	1.17E-01	3.47E-01	8.21E-01	7.82E-02	0
SLBP	ENSG00000163950	4	1694527	1714282	1.86E-01	2.91E-01	3.81E-02	7.32E-01	1.83E-01	7.83E-02	0
DPP3	ENSG00000254986	11	66247484	66277130	2.69E-01	5.69E-01	3.28E-01	2.73E-01	6.44E-01	7.84E-02	0

Gene	Ensembl ID	CHR	START	STOP	VHD_CD Pleiotropy P	VHD_GORD Pleiotropy P	VHD_IBD Pleiotropy P	VHD_IBS Pleiotropy P	VHD_PUD Pleiotropy P	VHD_UC Pleiotropy P	d
TGM2	ENSG00000198959	20	36756863	36794980	6.23E-01	6.63E-01	2.30E-01	8.12E-01	3.53E-02	7.84E-02	0
CDKN2C	ENSG00000123080	1	51426417	51440305	4.27E-01	6.37E-02	2.41E-01	4.35E-02	1.33E-01	7.84E-02	0
ARGLU1	ENSG00000134884	13	107194021	107220512	1.19E-01	1.08E-01	1.12E-01	1.40E-01	6.84E-01	7.85E-02	0
OR2K2	ENSG00000171133	9	114089763	114090800	3.03E-02	1.65E-01	6.42E-01	3.05E-02	2.44E-01	7.86E-02	0
SLC35B4	ENSG00000205060	7	133974084	134001803	1.79E-01	3.72E-01	6.08E-02	7.57E-01	1.77E-01	7.87E-02	0
ABI1	ENSG00000136754	10	27035522	27150016	6.50E-01	3.68E-01	2.10E-01	3.01E-02	9.98E-02	7.87E-02	0
DHX16	ENSG00000204560	6	30620896	30640814	1.41E-01	7.10E-02	1.21E-01	6.87E-02	2.58E-01	7.90E-02	0
PPIF	ENSG00000108179	10	81107225	81115093	4.85E-01	6.28E-01	1.16E-01	5.04E-01	1.94E-01	7.90E-02	0
FAM24A	ENSG00000203795	10	124670217	124672627	5.84E-01	8.99E-01	1.43E-01	7.08E-01	4.83E-01	7.90E-02	0
PRM3	ENSG00000178257	16	11367144	11367452	9.90E-02	4.66E-01	4.53E-02	3.91E-01	4.24E-01	7.90E-02	0
IL4I1	ENSG00000104951	19	50392911	50432796	8.11E-01	3.58E-01	1.99E-01	3.05E-01	2.67E-01	7.91E-02	0
POMC	ENSG00000115138	2	25383722	25391772	7.58E-04	1.02E-01	6.52E-03	1.55E-01	2.47E-01	7.91E-02	0
RHOC	ENSG00000155366	1	113243728	113250056	1.89E-02	9.54E-01	1.43E-02	3.57E-01	9.19E-01	7.91E-02	0
TRAPPC3	ENSG00000054116	1	36602173	36615098	1.41E-01	2.89E-01	4.17E-01	1.25E-01	1.43E-01	7.95E-02	0
DNLZ	ENSG00000213221	9	139253932	139258241	2.49E-02	6.88E-01	2.86E-02	6.09E-01	6.99E-01	7.95E-02	0
SOX17	ENSG00000164736	8	55370495	55373448	3.57E-02	3.83E-01	1.30E-01	7.01E-01	4.53E-01	7.95E-02	0
KSR1	ENSG00000141068	17	25783670	25953461	4.67E-02	5.66E-01	2.92E-01	2.57E-01	8.27E-01	7.96E-02	0
ZNF625	ENSG00000257591	19	12251032	12267546	1.60E-01	4.00E-02	1.04E-01	2.16E-01	1.60E-02	7.96E-02	0
EXOSC7	ENSG00000075914	3	45016733	45077558	7.61E-01	3.95E-01	1.76E-01	6.00E-01	2.15E-01	7.97E-02	0
ZMIZ1	ENSG00000108175	10	80828792	81076276	4.58E-04	3.05E-01	1.52E-03	2.43E-01	2.65E-01	7.97E-02	0
ADD1	ENSG00000087274	4	2845584	2931803	1.27E-02	2.92E-01	7.49E-02	3.18E-01	3.20E-01	7.98E-02	0
TOMM20	ENSG00000173726	1	235272651	235292251	1.17E-02	3.64E-01	2.12E-02	4.39E-01	1.18E-01	7.98E-02	0
WNT2	ENSG00000105989	7	116916685	116963343	2.62E-01	6.75E-01	1.34E-01	8.04E-01	3.32E-01	7.99E-02	0
RPS15	ENSG00000115268	19	1438358	1440583	3.19E-01	5.27E-02	1.64E-01	3.09E-02	6.90E-01	8.00E-02	0
KCTD13	ENSG00000174943	16	29916333	29938356	8.65E-02	1.73E-01	4.31E-01	1.49E-01	3.88E-01	8.00E-02	0
FARSA	ENSG00000179115	19	13033293	13044851	1.80E-01	1.80E-02	7.51E-02	2.28E-01	2.29E-01	8.01E-02	0
C11orf40	ENSG00000171987	11	4592653	4599050	2.28E-01	1.16E-01	2.81E-01	5.92E-01	6.38E-01	8.01E-02	0
STK33	ENSG00000130413	11	8413418	8615836	3.37E-01	3.56E-01	2.10E-01	6.91E-01	5.89E-01	8.02E-02	0
TGFB2	ENSG00000092969	1	218519577	218617961	1.98E-03	1.01E-01	5.60E-03	3.84E-01	3.80E-02	8.02E-02	0
HILPDA	ENSG00000135245	7	128095903	128098472	1.09E-02	1.77E-01	1.92E-01	1.14E-01	2.04E-02	8.03E-02	0
PIGR	ENSG00000162896	1	207101863	207119811	4.48E-01	6.94E-01	1.11E-01	3.48E-01	2.95E-01	8.04E-02	0
FSD1	ENSG00000105255	19	4304597	4323840	1.69E-02	8.91E-02	9.78E-03	3.56E-02	1.07E-01	8.04E-02	0
NME7	ENSG00000143156	1	169101769	169337205	3.94E-01	1.65E-01	1.26E-01	2.43E-02	6.67E-03	8.04E-02	0
MAP3K3	ENSG00000198909	17	61699775	61773663	9.25E-01	6.05E-02	2.37E-01	1.27E-01	5.29E-01	8.04E-02	0
NUDT5	ENSG00000165609	10	12207324	12238143	2.03E-01	3.61E-02	3.96E-02	4.42E-01	1.76E-01	8.05E-02	0
KCNQ4	ENSG00000117013	1	41249684	41306124	2.96E-01	4.46E-01	1.31E-01	3.48E-01	2.48E-01	8.06E-02	0
APOBEC3B	ENSG00000179750	22	39378352	39388809	4.26E-01	7.06E-01	1.44E-01	3.94E-01	2.63E-01	8.06E-02	0
PTPN11	ENSG00000179295	12	112856155	112947717	6.22E-02	4.13E-02	3.62E-02	5.63E-02	1.79E-01	8.08E-02	0
HDLBP	ENSG00000115677	2	242166679	242256476	3.94E-01	8.97E-01	1.80E-01	1.75E-01	6.21E-01	8.08E-02	0
SERBP1	ENSG00000142864	1	67873493	67896098	1.44E-01	4.12E-01	3.37E-02	9.65E-01	4.43E-01	8.09E-02	0
TSPAN3	ENSG00000140391	15	77336359	77376326	1.66E-02	7.72E-01	4.39E-02	2.64E-01	5.36E-01	8.11E-02	0
DONSON	ENSG00000159147	21	34931848	34961014	7.81E-04	5.63E-01	4.94E-03	2.57E-02	5.73E-02	8.12E-02	0
BAK1	ENSG00000030110	6	33540329	33548019	2.90E-01	4.44E-01	2.73E-01	6.91E-01	4.38E-01	8.12E-02	0
RBM15	ENSG00000162775	1	110881128	110889299	8.67E-01	2.04E-01	3.65E-01	2.72E-01	8.46E-02	8.12E-02	0
ACTL6B	ENSG00000077080	7	100240720	100254084	3.64E-01	7.20E-01	1.43E-01	7.15E-01	7.23E-01	8.12E-02	0
KRTAP2-2	ENSG00000214518	17	39210750	39211482	7.56E-01	1.03E-01	2.07E-01	1.91E-01	3.60E-01	8.13E-02	0
UBXN2A	ENSG00000173960	2	24150155	24227779	2.17E-01	6.24E-02	4.57E-02	2.27E-01	1.89E-01	8.13E-02	0
CD48	ENSG00000117091	1	160648536	160681641	4.50E-03	2.08E-01	6.68E-03	3.90E-02	4.57E-01	8.13E-02	0
SH2B3	ENSG00000111252	12	111843752	111889427	1.45E-01	1.56E-01	4.52E-02	4.09E-01	1.64E-01	8.13E-02	0

Gene	Ensembl ID	CHR	START	STOP	VHD_CD Pleiotropy P	VHD_GORD Pleiotropy P	VHD_IBD Pleiotropy P	VHD_IBS Pleiotropy P	VHD_PUD Pleiotropy P	VHD_UC Pleiotropy P	d
PLCXD2	ENSG00000240891	3	111393523	111565294	1.18E-01	1.81E-01	1.75E-01	2.60E-01	6.24E-01	8.13E-02	0
DNAJC24	ENSG00000170946	11	31391387	31453396	5.15E-01	4.25E-02	1.06E-01	1.98E-01	2.19E-02	8.14E-02	0
SNRNP35	ENSG00000184209	12	123942188	123957701	4.05E-01	7.48E-01	1.49E-01	1.04E-01	9.90E-02	8.14E-02	0
SLC29A4	ENSG00000164638	7	5314000	5346501	1.37E-01	4.80E-01	2.51E-02	1.32E-01	4.70E-01	8.14E-02	0
ATXN3	ENSG00000066427	14	92524896	92572965	4.42E-01	2.31E-01	2.04E-01	5.91E-01	6.13E-01	8.14E-02	0
ZC2HC1C	ENSG00000119703	14	75530873	75545126	5.98E-01	2.74E-01	3.38E-01	7.65E-01	2.70E-01	8.16E-02	0
FLNC	ENSG00000128591	7	128470431	128499328	3.82E-02	8.42E-01	8.29E-01	3.75E-01	3.89E-01	8.16E-02	0
C11orf65	ENSG00000166323	11	108179246	108338258	3.32E-01	1.73E-01	1.52E-01	7.29E-02	1.40E-01	8.17E-02	0
CTSK	ENSG00000143387	1	150768684	150780799	1.06E-01	2.64E-01	6.72E-02	1.37E-01	2.39E-01	8.17E-02	0
UNC79	ENSG00000133958	14	93799565	94174222	1.70E-01	1.98E-01	4.99E-02	2.03E-01	6.72E-01	8.17E-02	0
CASS4	ENSG00000087589	20	54987168	55034396	1.20E-01	3.50E-01	8.50E-02	7.07E-01	1.97E-01	8.17E-02	0
SEC23B	ENSG00000101310	20	18488137	18542059	3.83E-01	7.06E-02	9.74E-02	1.93E-01	5.01E-01	8.18E-02	0
KANSL2	ENSG00000139620	12	49047184	49076021	6.64E-01	7.22E-01	1.11E-01	3.94E-01	5.74E-01	8.18E-02	0
ZBTB34	ENSG00000177125	9	129622944	129648157	1.74E-01	4.16E-01	4.75E-01	6.86E-03	1.60E-01	8.18E-02	0
APOPT1	ENSG00000256053	14	104029299	104073860	1.68E-02	2.82E-01	2.79E-02	6.73E-01	6.70E-01	8.19E-02	0
MRPS15	ENSG00000116898	1	36921319	36930038	1.58E-02	2.47E-02	1.97E-01	2.95E-01	1.58E-02	8.22E-02	0
MFSD5	ENSG00000182544	12	53645035	53648189	2.35E-01	1.70E-02	5.63E-02	2.51E-01	1.23E-01	8.23E-02	0
C22orf42	ENSG00000205856	22	32544993	32555309	6.08E-01	1.83E-02	3.86E-01	6.86E-02	2.24E-01	8.23E-02	0
ZNF37A	ENSG00000075407	10	38383264	38414472	6.69E-01	6.27E-01	2.39E-01	9.66E-01	2.88E-01	8.23E-02	0
SLITRK6	ENSG00000184564	13	86366925	86373623	2.79E-02	4.36E-01	6.69E-01	2.50E-02	3.14E-02	8.23E-02	0
KIAA1024L	ENSG00000186367	5	129083772	129102425	5.39E-02	3.58E-01	3.80E-02	1.78E-02	9.51E-02	8.24E-02	0
TDRKH	ENSG00000182134	1	151742583	151763892	3.73E-02	8.72E-01	2.94E-02	7.63E-01	3.63E-01	8.24E-02	0
C11orf45	ENSG00000174370	11	128769460	128775930	2.86E-01	5.55E-01	1.06E-01	9.68E-02	3.24E-01	8.25E-02	0
GPR149	ENSG00000174948	3	154055461	154147504	1.74E-01	6.22E-02	1.05E-01	1.28E-01	1.29E-01	8.25E-02	0
EHD3	ENSG00000013016	2	31456880	31492313	7.43E-01	1.95E-01	1.57E-01	4.79E-01	2.99E-01	8.25E-02	0
LAMA5	ENSG00000130702	20	60883011	60942368	2.22E-01	1.15E-01	8.25E-02	9.93E-03	7.86E-02	8.26E-02	0
S1PR2	ENSG00000267534	19	10334520	10341948	2.31E-02	3.81E-01	2.68E-02	2.38E-01	1.52E-01	8.26E-02	0
LPAR5	ENSG00000184574	12	6728001	6745613	3.03E-02	8.53E-03	6.65E-02	1.43E-01	1.07E-01	8.26E-02	0
SERTAD3	ENSG00000167565	19	40946751	40950612	9.41E-01	2.54E-01	2.11E-01	3.02E-01	5.64E-01	8.27E-02	0
TRAPPC9	ENSG00000167632	8	140742586	141468678	4.69E-01	1.39E-01	3.12E-01	3.27E-01	7.46E-02	8.27E-02	0
ZNF639	ENSG00000121864	3	179040779	179053323	3.49E-04	9.92E-02	4.31E-03	6.24E-02	4.58E-01	8.28E-02	0
SNX10	ENSG00000086300	7	26331541	26413949	2.97E-02	2.49E-03	7.15E-02	3.01E-02	5.84E-02	8.28E-02	0
NUP98	ENSG00000110713	11	3692313	3819022	6.62E-01	3.92E-01	9.15E-02	6.92E-02	1.44E-01	8.30E-02	0
MYBL1	ENSG00000185697	8	67474410	67526482	1.47E-01	1.41E-01	7.26E-02	3.35E-01	6.28E-01	8.31E-02	0
UNC5D	ENSG00000156687	8	35092975	35654068	4.75E-01	6.44E-01	1.88E-01	3.25E-01	3.91E-02	8.31E-02	0
MCM9	ENSG00000111877	6	119134605	119256327	4.69E-01	7.02E-02	2.75E-01	1.88E-01	1.99E-01	8.32E-02	0
TLR3	ENSG00000164342	4	186990306	187009223	1.07E-01	3.40E-01	3.93E-02	9.69E-01	1.80E-01	8.33E-02	0
RAG1	ENSG00000166349	11	36532259	36614706	3.58E-01	9.77E-02	8.30E-02	5.22E-01	3.71E-01	8.33E-02	0
EIF2B1	ENSG00000111361	12	124104953	124118313	5.33E-01	5.64E-01	1.23E-01	4.37E-01	7.53E-02	8.33E-02	0
KIAA1614	ENSG00000135835	1	180882290	180920750	7.72E-01	6.05E-02	5.23E-01	9.77E-01	4.66E-01	8.34E-02	0
FBXO9	ENSG00000112146	6	52916789	52965671	9.37E-01	7.46E-01	2.76E-01	4.87E-01	4.50E-01	8.34E-02	0
SNX17	ENSG00000115234	2	27593389	27599995	1.28E-02	2.09E-01	1.27E-02	4.67E-02	2.81E-01	8.34E-02	0
MAPK13	ENSG00000156711	6	36095586	36107842	4.23E-02	6.39E-01	2.50E-02	7.52E-02	3.18E-01	8.35E-02	0
WDR78	ENSG00000152763	1	67278568	67390570	8.21E-03	2.44E-02	1.11E-01	7.36E-01	2.54E-01	8.35E-02	0
LEFTY2	ENSG00000143768	1	226124298	226129189	7.83E-03	3.25E-02	2.82E-02	6.04E-01	5.26E-03	8.37E-02	0
FCAMR	ENSG00000162897	1	207131310	207143970	3.11E-01	2.06E-01	7.05E-02	2.92E-01	9.24E-02	8.37E-02	0
DDX43	ENSG00000080007	6	74104471	74127292	4.27E-01	2.61E-02	1.92E-01	1.01E-02	1.06E-01	8.37E-02	0
KRT81	ENSG00000205426	12	52679697	52685318	8.63E-01	4.60E-01	1.37E-01	5.81E-01	1.10E-01	8.38E-02	0
ZNF540	ENSG00000171817	19	38042308	38104998	2.56E-01	1.14E-01	9.81E-02	2.34E-02	1.29E-01	8.38E-02	0

Gene	Ensembl ID	CHR	START	STOP	VHD_CD Pleiotropy P	VHD_GORD Pleiotropy P	VHD_IBD Pleiotropy P	VHD_IBS Pleiotropy P	VHD_PUD Pleiotropy P	VHD_UC Pleiotropy P	d
ILK	ENSG00000166333	11	6624961	6632102	3.00E-02	5.48E-01	3.98E-02	7.94E-02	3.97E-02	8.39E-02	0
PLLP	ENSG00000102934	16	57290004	57318599	1.75E-01	4.23E-02	4.94E-01	1.99E-01	3.06E-01	8.39E-02	0
ZNF521	ENSG00000198795	18	22641890	22932154	3.70E-01	5.15E-01	5.19E-02	8.10E-02	5.35E-01	8.40E-02	0
LCE2A	ENSG00000187173	1	152670840	152671918	3.27E-01	4.33E-01	4.29E-01	6.73E-01	2.86E-01	8.40E-02	0
SPP1	ENSG00000118785	4	88896819	88904562	6.23E-01	8.15E-01	1.04E-01	1.69E-01	3.78E-01	8.40E-02	0
DYNC1LI2	ENSG00000135720	16	66754796	66785701	1.20E-01	3.70E-01	4.74E-02	1.48E-01	5.12E-01	8.40E-02	0
FAM177A1	ENSG00000151327	14	35514113	35582336	1.19E-02	4.89E-01	1.55E-02	2.58E-01	2.27E-01	8.42E-02	0
SURF4	ENSG00000148248	9	136228325	136242970	1.72E-02	4.06E-01	2.16E-02	2.86E-01	6.77E-02	8.42E-02	0
ATM	ENSG00000149311	11	108093211	108239829	2.79E-01	2.60E-01	1.01E-01	1.54E-01	1.83E-01	8.42E-02	0
JTB	ENSG00000143543	1	153946745	153950164	4.83E-02	3.02E-01	9.00E-01	1.79E-01	3.30E-01	8.44E-02	0
ZZEF1	ENSG00000074755	17	3907739	4046314	2.93E-01	2.52E-01	3.05E-01	1.89E-01	6.16E-01	8.45E-02	0
LIPM	ENSG00000173239	10	90562487	90580303	3.73E-01	8.87E-01	1.14E-01	1.43E-01	2.94E-01	8.45E-02	0
CA7	ENSG00000168748	16	66878282	66888056	5.01E-02	1.32E-01	2.80E-02	1.32E-01	5.22E-01	8.45E-02	0
PRSS46	ENSG00000261603	3	46761073	46777919	7.80E-02	5.72E-02	5.10E-02	3.87E-01	8.13E-02	8.45E-02	0
CREB3L4	ENSG00000143578	1	153940010	153946839	2.06E-01	2.81E-01	8.49E-01	1.55E-01	5.56E-01	8.46E-02	0
BTN2A2	ENSG00000124508	6	26383324	26395102	1.47E-01	2.55E-02	6.02E-02	1.70E-01	1.92E-01	8.46E-02	0
CLEC1B	ENSG00000165682	12	10138241	10166023	1.42E-01	8.03E-02	6.07E-02	6.71E-01	1.51E-01	8.47E-02	0
WDYHV1	ENSG00000156795	8	124428965	124479470	4.81E-01	1.82E-01	3.56E-01	6.59E-01	6.25E-01	8.48E-02	0
ATP13A4	ENSG00000127249	3	193119866	193310900	5.21E-01	2.47E-01	7.57E-02	3.94E-01	1.58E-01	8.49E-02	0
MLNR	ENSG00000102539	13	49794474	49796513	7.92E-02	4.36E-03	1.52E-02	4.78E-01	7.75E-02	8.49E-02	0
DCTPP1	ENSG00000179958	16	30434940	30441396	9.60E-01	6.59E-02	4.08E-01	3.41E-01	3.51E-01	8.49E-02	0
GRK7	ENSG00000114124	3	141496990	141537332	4.23E-01	7.57E-01	2.35E-01	2.26E-01	6.88E-01	8.50E-02	0
OR51F2	ENSG00000176925	11	4842551	4843686	2.61E-01	4.74E-01	1.16E-01	2.07E-01	8.75E-02	8.51E-02	0
SPATA19	ENSG00000166118	11	133710526	133715433	3.77E-02	7.85E-02	3.81E-02	3.41E-02	9.31E-02	8.51E-02	0
CAPRIN2	ENSG00000110888	12	30862486	30907885	4.31E-01	5.84E-01	1.00E-01	5.58E-01	4.23E-01	8.52E-02	0
KRTAP1-4	ENSG00000204887	17	39185949	39186356	1.98E-01	5.12E-02	1.32E-01	1.68E-01	6.59E-02	8.54E-02	0
ANO2	ENSG00000047617	12	5641035	6055398	1.32E-01	8.70E-01	7.06E-01	9.12E-01	1.56E-01	8.54E-02	0
RANBP9	ENSG00000010017	6	13621730	13711796	2.76E-01	1.76E-02	3.79E-01	7.41E-01	4.14E-01	8.55E-02	0
CYBRD1	ENSG00000071967	2	172378757	172414643	4.28E-02	6.96E-01	1.59E-02	2.41E-01	5.61E-01	8.55E-02	0
PACSIN1	ENSG00000124507	6	34433916	34503006	2.74E-01	2.27E-02	2.45E-01	8.24E-03	4.56E-01	8.56E-02	0
TFRC	ENSG00000072274	3	195754054	195809060	7.51E-01	6.62E-02	2.10E-01	1.97E-01	6.42E-01	8.56E-02	0
PLEKHG5	ENSG00000171680	1	6526152	6580121	5.63E-01	7.30E-01	9.60E-02	9.56E-01	7.85E-01	8.57E-02	0
GSS	ENSG00000100983	20	33516236	33543620	6.79E-01	1.71E-01	3.68E-01	3.98E-01	4.05E-01	8.57E-02	0
ARPC5L	ENSG00000136950	9	127624409	127640003	2.11E-01	4.75E-03	9.93E-02	1.26E-02	6.40E-03	8.58E-02	0
GCM1	ENSG00000137270	6	52991762	53013627	9.68E-01	2.61E-01	3.72E-01	2.73E-01	5.85E-01	8.59E-02	0
ACTR3C	ENSG00000106526	7	149941005	150020814	5.24E-01	4.14E-01	2.78E-01	1.16E-01	3.68E-01	8.60E-02	0
SPACA3	ENSG00000141316	17	31297394	31324895	2.65E-01	1.22E-01	2.75E-01	1.55E-02	1.35E-01	8.60E-02	0
IPO9	ENSG00000198700	1	201798269	201853422	1.86E-01	3.09E-02	5.36E-02	1.91E-01	5.29E-02	8.61E-02	0
UFSP1	ENSG00000176125	7	100486346	100487339	5.25E-02	6.00E-01	2.81E-02	2.69E-01	6.55E-01	8.61E-02	0
PRKAR2B	ENSG00000005249	7	106685094	106802256	1.09E-01	4.91E-01	6.61E-02	5.25E-02	2.11E-01	8.61E-02	0
ZC3H4	ENSG00000130749	19	47567444	47617009	2.78E-01	1.47E-01	4.00E-01	2.39E-01	9.59E-01	8.63E-02	0
RPS6KA1	ENSG00000117676	1	26856252	26901521	1.30E-02	2.95E-01	9.59E-03	6.33E-02	1.83E-01	8.63E-02	0
NUAK2	ENSG00000163545	1	205271187	205290883	2.54E-01	1.89E-01	1.22E-01	1.02E-01	1.41E-01	8.63E-02	0
SON	ENSG00000159140	21	34914924	34949812	5.72E-04	4.32E-01	4.95E-03	1.86E-02	6.57E-02	8.64E-02	0
CLIP4	ENSG00000115295	2	29320571	29412509	1.31E-01	2.01E-01	2.06E-01	1.84E-01	2.04E-01	8.64E-02	0
CENPK	ENSG00000123219	5	64813593	64858998	1.72E-01	2.66E-02	2.83E-01	7.47E-01	2.92E-01	8.64E-02	0
NLRX1	ENSG00000160703	11	119037277	119054725	9.32E-01	1.49E-01	1.15E-01	3.16E-01	1.72E-01	8.65E-02	0
KRTAP5-11	ENSG00000204571	11	71292901	71314399	1.20E-02	2.07E-01	1.99E-02	2.09E-02	2.90E-02	8.65E-02	0
METTL13	ENSG00000010165	1	171750788	171783163	8.04E-01	2.91E-01	2.80E-01	5.42E-02	6.58E-01	8.65E-02	0

Gene	Ensembl ID	CHR	START	STOP	VHD_CD Pleiotropy P	VHD_GORD Pleiotropy P	VHD_IBD Pleiotropy P	VHD_IBS Pleiotropy P	VHD_PUD Pleiotropy P	VHD_UC Pleiotropy P	d
PCDHB1	ENSG00000171815	5	140430979	140433512	3.76E-01	3.32E-02	8.14E-02	7.08E-01	9.66E-01	8.65E-02	0
ZNF610	ENSG00000167554	19	52839498	52871031	8.43E-01	7.14E-01	1.86E-01	5.31E-01	4.82E-01	8.67E-02	0
DVL1	ENSG00000107404	1	1270656	1284730	9.30E-02	3.26E-01	6.26E-02	6.03E-01	7.39E-01	8.67E-02	0
PELP1	ENSG00000141456	17	4574679	4607632	2.90E-01	5.48E-01	9.70E-02	9.82E-01	2.34E-01	8.67E-02	0
IBA57	ENSG00000181873	1	228353516	228369958	9.60E-03	1.73E-02	1.89E-02	1.90E-03	6.39E-02	8.68E-02	0
CEP170	ENSG00000143702	1	243287730	243418650	1.00E-01	2.59E-01	8.88E-02	1.31E-01	5.50E-02	8.69E-02	0
LMAN1	ENSG00000074695	18	56995055	57027194	1.63E-01	3.22E-01	7.25E-02	3.74E-01	5.45E-01	8.69E-02	0
NOD1	ENSG00000106100	7	30464143	30518400	5.30E-01	5.47E-01	7.93E-02	2.55E-01	7.42E-01	8.69E-02	0
MBOAT1	ENSG00000172197	6	20100935	20212670	7.26E-01	2.96E-01	1.60E-01	5.19E-02	4.68E-01	8.70E-02	0
SGOL2	ENSG00000163535	2	201374731	201448505	8.99E-02	2.82E-01	1.03E-01	9.83E-03	1.90E-02	8.70E-02	0
DUSP10	ENSG00000143507	1	221874766	221915518	5.48E-01	2.81E-01	1.25E-01	2.09E-01	2.33E-01	8.70E-02	0
SGCA	ENSG00000108823	17	48241575	48253292	4.70E-01	6.47E-01	1.60E-01	5.27E-01	5.38E-01	8.72E-02	0
LENG1	ENSG00000105617	19	54658899	54663620	4.26E-01	4.69E-01	6.78E-02	6.06E-01	2.03E-01	8.72E-02	0
NOMO3	ENSG00000103226	16	16326352	16388668	7.93E-01	4.90E-01	5.88E-01	8.71E-01	7.68E-01	8.72E-02	0
TOP3A	ENSG00000177302	17	18174742	18218321	1.95E-01	3.34E-01	4.69E-02	4.32E-01	8.15E-01	8.73E-02	0
PLEKHA5	ENSG00000052126	12	19282648	19529334	9.41E-01	1.88E-02	2.36E-01	1.23E-01	1.87E-01	8.73E-02	0
CPT1C	ENSG00000169169	19	50194155	50216988	3.10E-02	8.03E-01	3.28E-02	9.16E-01	7.51E-01	8.74E-02	0
HOXC11	ENSG00000123388	12	54366910	54371427	6.79E-02	2.03E-01	2.73E-01	3.37E-01	1.26E-01	8.74E-02	0
IL18R1	ENSG00000115604	2	102927989	103015218	1.46E-02	4.72E-01	1.58E-02	1.57E-01	5.78E-01	8.75E-02	0
RMND5B	ENSG00000145916	5	177557997	177577566	4.73E-01	3.21E-02	2.17E-01	3.03E-01	6.11E-01	8.75E-02	0
DDX42	ENSG00000198231	17	61850963	61896677	9.39E-02	1.00E-01	1.43E-01	1.27E-01	2.87E-01	8.76E-02	0
NFATC3	ENSG00000072736	16	68118654	68263162	3.22E-02	7.85E-02	5.36E-01	4.45E-02	7.64E-02	8.76E-02	0
RIOK3	ENSG00000101782	18	21032787	21066567	2.60E-01	1.64E-02	3.00E-01	1.47E-02	7.60E-02	8.76E-02	0
STXBP3	ENSG00000116266	1	109289296	109352148	2.39E-01	2.71E-01	2.65E-01	5.54E-01	6.00E-01	8.77E-02	0
RNF186	ENSG00000178828	1	20140523	20141771	8.33E-01	2.14E-01	4.70E-01	7.58E-01	7.68E-01	8.78E-02	0
SERINC4	ENSG00000184716	15	44086360	44092419	1.57E-01	1.17E-01	6.93E-02	3.17E-01	2.49E-01	8.78E-02	0
MRPL52	ENSG00000172590	14	23299088	23304246	1.29E-01	2.94E-01	1.14E-01	3.31E-01	5.96E-01	8.79E-02	0
IL25	ENSG00000166090	14	23842018	23845612	4.81E-01	6.60E-01	3.17E-01	4.72E-01	3.04E-01	8.80E-02	0
NIPSNAP3B	ENSG00000165028	9	107526438	107539738	1.38E-01	5.53E-02	1.74E-01	1.35E-01	1.43E-02	8.80E-02	0
CALB2	ENSG00000172137	16	71392616	71424341	8.43E-01	4.30E-01	2.54E-01	2.82E-01	2.53E-01	8.80E-02	0
PEX2	ENSG00000164751	8	77892494	77913280	2.11E-01	1.42E-01	1.25E-01	7.63E-02	1.99E-01	8.81E-02	0
FTSJ3	ENSG00000108592	17	61896793	61907372	6.69E-02	1.55E-01	2.20E-01	1.00E-01	2.48E-01	8.81E-02	0
SIGLEC12	ENSG00000254521	19	51994611	52005043	1.44E-01	4.12E-01	5.88E-02	1.48E-01	1.37E-01	8.82E-02	0
PEX11A	ENSG00000166821	15	90220995	90234014	6.39E-02	3.37E-01	8.77E-02	5.60E-01	5.64E-01	8.82E-02	0
TSSK2	ENSG00000206203	22	19118321	19120134	4.66E-01	6.15E-01	1.49E-01	1.57E-02	4.49E-02	8.83E-02	0
MIB1	ENSG00000101752	18	19284918	19450918	6.87E-02	8.26E-01	4.23E-02	8.64E-01	9.06E-02	8.84E-02	0
EYA2	ENSG00000064655	20	45523263	45817492	7.36E-01	7.85E-01	3.38E-01	5.21E-01	3.08E-01	8.84E-02	0
OTOGL	ENSG00000165899	12	80603233	80772870	2.13E-01	7.78E-01	2.59E-02	5.68E-01	7.63E-01	8.84E-02	0
MRAP2	ENSG00000135324	6	84743475	84800600	4.48E-01	2.27E-01	3.65E-01	8.02E-01	7.40E-01	8.85E-02	0
RGS14	ENSG00000169220	5	176784838	176799602	1.45E-01	5.54E-01	5.34E-02	6.38E-01	2.83E-01	8.86E-02	0
PIK3CG	ENSG00000105851	7	106505723	106547590	5.30E-01	2.29E-01	3.45E-01	1.87E-01	6.68E-01	8.87E-02	0
FAM57B	ENSG00000149926	16	30035748	30064299	7.95E-01	7.47E-01	2.14E-01	7.54E-01	7.82E-01	8.88E-02	0
NUP62	ENSG00000213024	19	50410082	50433020	7.48E-01	2.58E-01	1.44E-01	2.13E-01	1.96E-01	8.88E-02	0
ERLIN2	ENSG00000147475	8	37594117	37616619	4.81E-01	1.17E-01	6.33E-01	2.30E-01	8.85E-01	8.89E-02	0
IFFO1	ENSG00000010295	12	6647541	6665239	1.54E-01	1.66E-01	3.15E-01	3.58E-01	7.09E-01	8.89E-02	0
NARS2	ENSG00000137513	11	78147007	78285919	4.65E-01	2.08E-01	1.49E-01	2.90E-01	6.11E-01	8.90E-02	0
SLC41A3	ENSG00000114544	3	125725198	125820404	1.67E-01	1.20E-01	1.18E-01	2.44E-01	1.00E-01	8.90E-02	0
APOBEC3G	ENSG00000239713	22	39436924	39483748	3.65E-01	4.75E-01	7.56E-01	4.53E-01	1.78E-01	8.90E-02	0
RBM48	ENSG00000127993	7	92158087	92167319	2.29E-01	1.29E-01	1.94E-01	4.83E-01	4.66E-01	8.91E-02	0

Gene	Ensembl ID	CHR	START	STOP	VHD_CD Pleiotropy P	VHD_GORD Pleiotropy P	VHD_IBD Pleiotropy P	VHD_IBS Pleiotropy P	VHD_PUD Pleiotropy P	VHD_UC Pleiotropy P	d
SUV420H2	ENSG00000133247	19	55851221	55859488	9.48E-01	7.07E-01	2.46E-01	5.55E-01	6.54E-01	8.91E-02	0
TDRD10	ENSG00000163239	1	154474695	154520623	3.45E-03	1.27E-03	1.59E-02	2.09E-02	1.87E-02	8.92E-02	0
SRR	ENSG00000167720	17	2206677	2228554	2.15E-01	1.63E-01	5.92E-02	8.54E-02	5.67E-02	8.93E-02	0
PARP16	ENSG00000138617	15	65526798	65592956	1.85E-01	5.26E-02	1.18E-01	3.53E-01	5.27E-01	8.93E-02	0
ACSS1	ENSG00000154930	20	24986868	25039616	1.43E-01	4.46E-01	5.41E-01	7.79E-02	5.54E-01	8.94E-02	0
MTIF3	ENSG00000122033	13	28009776	28024739	1.72E-02	5.00E-01	1.96E-02	8.03E-02	2.51E-02	8.95E-02	0
TNRC6A	ENSG00000090905	16	24741016	24838953	8.50E-03	3.63E-01	1.62E-02	6.78E-01	1.28E-01	8.95E-02	0
CEBPB	ENSG00000172216	20	48807376	48809212	8.27E-02	8.95E-02	2.09E-01	4.88E-01	7.27E-01	8.95E-02	0
MIPOL1	ENSG00000151338	14	37667118	38021566	3.26E-02	2.55E-02	1.08E-01	2.98E-02	8.46E-04	8.96E-02	0
SYNPO2	ENSG00000172403	4	119809996	119982402	2.64E-01	7.47E-01	6.65E-02	4.64E-02	4.14E-01	8.96E-02	0
WNK1	ENSG00000060237	12	861759	1020618	8.94E-02	5.15E-01	1.24E-01	9.17E-01	7.80E-01	8.96E-02	0
RCAN1	ENSG00000159200	21	35885440	35987441	1.80E-01	8.07E-02	4.67E-02	1.64E-01	6.94E-02	8.98E-02	0
PIK3C2A	ENSG00000011405	11	17099277	17229530	1.82E-01	3.13E-01	1.52E-01	1.66E-02	4.55E-01	8.98E-02	0
ATP13A2	ENSG00000159363	1	17312453	17338423	5.50E-01	1.45E-01	6.82E-01	2.47E-01	6.90E-01	8.98E-02	0
FNDC3B	ENSG00000075420	3	171757418	172119455	7.11E-02	5.09E-01	4.40E-02	5.64E-01	2.84E-01	8.99E-02	0
IGLL5	ENSG00000254709	22	23229960	23238287	7.26E-02	1.69E-01	3.15E-02	8.95E-02	1.79E-01	8.99E-02	0
CRYGN	ENSG00000127377	7	151125921	151137899	7.58E-02	5.61E-02	9.53E-02	9.95E-01	1.39E-01	8.99E-02	0
SSTR1	ENSG00000139874	14	38677204	38682272	8.39E-01	2.78E-01	3.93E-01	3.02E-01	3.32E-01	8.99E-02	0
ZNF20	ENSG00000132010	19	12203658	12251222	3.06E-01	1.21E-01	1.67E-01	1.97E-01	3.61E-02	9.01E-02	0
MXRA7	ENSG00000182534	17	74668633	74707098	5.33E-01	8.18E-01	3.36E-01	5.94E-01	3.66E-01	9.02E-02	0
CENPM	ENSG00000100162	22	42334725	42343168	8.26E-02	7.04E-01	8.21E-01	9.57E-02	2.91E-01	9.02E-02	0
IL6R	ENSG00000160712	1	154377669	154441926	1.77E-04	5.04E-04	9.45E-03	1.36E-02	1.73E-02	9.03E-02	0
C12orf66	ENSG00000174206	12	64580096	64616076	4.59E-03	2.33E-01	1.64E-02	2.63E-01	6.56E-03	9.03E-02	0
HIST1H2BC	ENSG00000180596	6	26115101	26124154	1.33E-02	2.37E-02	2.34E-02	3.29E-01	6.86E-01	9.04E-02	0
FAM175B	ENSG00000165660	10	126490354	126525239	3.55E-02	3.41E-01	2.03E-02	9.34E-02	6.51E-01	9.04E-02	0
DGKE	ENSG00000153933	17	54911460	54946036	1.63E-02	1.53E-01	2.01E-02	2.08E-01	8.67E-01	9.05E-02	0
NRBP1	ENSG00000115216	2	27650657	27665126	4.01E-02	2.24E-01	3.07E-02	7.54E-02	4.02E-01	9.07E-02	0
LMBRD2	ENSG00000164187	5	36098514	36152063	4.22E-01	6.08E-01	1.44E-01	5.81E-02	4.46E-01	9.08E-02	0
PLCH2	ENSG00000149527	1	2357419	2436969	6.49E-01	7.46E-01	2.83E-01	6.67E-01	6.42E-01	9.08E-02	0
TMCO3	ENSG00000150403	13	114145310	114204542	1.94E-01	9.72E-02	4.65E-01	3.06E-01	1.08E-01	9.08E-02	0
IRAK1BP1	ENSG00000146243	6	79577189	79656157	4.19E-01	1.21E-03	1.21E-01	6.18E-01	5.86E-01	9.08E-02	0
SPPL2C	ENSG00000185294	17	43922256	43924438	3.95E-02	1.30E-02	4.02E-02	5.47E-01	4.04E-02	9.08E-02	0
NMI	ENSG00000123609	2	152126979	152146571	6.27E-02	6.92E-01	4.08E-02	3.84E-01	4.86E-01	9.09E-02	0
PPIP5K2	ENSG00000145725	5	102455853	102548500	5.28E-02	4.37E-02	2.24E-02	4.68E-03	3.37E-02	9.11E-02	0
PCYOX1L	ENSG00000145882	5	148737570	148749216	5.78E-02	2.67E-01	3.07E-01	1.68E-02	1.39E-01	9.13E-02	0
PM20D1	ENSG00000162877	1	205797150	205819260	1.66E-01	3.49E-01	1.04E-01	7.97E-02	1.78E-02	9.13E-02	0
ADAD1	ENSG00000164113	4	123300121	123350957	1.13E-02	1.30E-01	1.28E-02	1.08E-01	8.25E-01	9.14E-02	0
CFHR2	ENSG00000080910	1	196788898	196928356	2.13E-01	8.35E-01	4.24E-01	3.20E-01	4.91E-01	9.14E-02	0
ZNF788	ENSG00000214189	19	12203078	12248050	3.92E-01	2.39E-01	1.56E-01	2.00E-01	1.24E-01	9.14E-02	0
PATL2	ENSG00000229474	15	44957930	45003514	1.34E-01	6.51E-01	8.62E-02	1.64E-02	6.34E-01	9.14E-02	0
HOXA6	ENSG00000106006	7	27185015	27190222	3.08E-01	8.38E-01	2.01E-01	2.24E-01	3.55E-01	9.14E-02	0
F11	ENSG00000088926	4	187187099	187210835	7.88E-02	8.70E-02	1.68E-01	3.18E-01	5.03E-03	9.14E-02	0
FANCE	ENSG00000112039	6	35420138	35434880	8.69E-01	7.87E-01	2.85E-01	3.81E-01	6.59E-01	9.15E-02	0
ADNP	ENSG00000101126	20	49505585	49547958	5.89E-01	4.49E-01	2.08E-01	4.10E-01	3.50E-02	9.15E-02	0
SLC12A3	ENSG00000070915	16	56899119	56949762	1.93E-01	4.99E-01	8.63E-02	2.76E-01	1.12E-01	9.15E-02	0
CELA3A	ENSG00000142789	1	22328149	22339032	4.32E-02	9.30E-01	1.14E-01	2.09E-01	9.50E-01	9.16E-02	0
OR4E2	ENSG00000221977	14	22133297	22134238	1.15E-01	2.00E-01	4.73E-02	5.23E-01	3.24E-01	9.17E-02	0
DOCK2	ENSG00000134516	5	169064251	169510386	7.90E-01	3.87E-01	4.33E-01	8.72E-01	3.69E-01	9.17E-02	0
LYSMD3	ENSG00000176018	5	89811428	89825401	7.82E-01	2.50E-03	3.58E-01	3.27E-02	7.81E-02	9.18E-02	0

Gene	Ensembl ID	CHR	START	STOP	VHD_CD Pleiotropy P	VHD_GORD Pleiotropy P	VHD_IBD Pleiotropy P	VHD_IBS Pleiotropy P	VHD_PUD Pleiotropy P	VHD_UC Pleiotropy P	d
WHSC1L1	ENSG00000147548	8	38127215	38239790	1.59E-01	5.07E-01	6.68E-02	4.61E-02	2.51E-01	9.19E-02	0
TLL1	ENSG00000038295	4	166794410	167025047	9.14E-01	6.52E-01	3.10E-01	5.31E-01	7.34E-01	9.20E-02	0
RGS5	ENSG00000143248	1	163080911	163291577	1.13E-01	1.37E-01	1.94E-02	2.35E-01	2.43E-02	9.22E-02	0
LY6G6D	ENSG00000244355	6	31683133	31685581	6.20E-01	6.06E-01	1.72E-01	7.75E-01	4.93E-01	9.23E-02	0
AURKB	ENSG00000178999	17	8108056	8113918	5.24E-01	4.90E-02	1.79E-01	1.17E-01	1.78E-01	9.24E-02	0
RBM39	ENSG00000131051	20	34291531	34330234	5.35E-01	4.59E-01	1.82E-01	4.86E-01	5.26E-01	9.25E-02	0
PSMB8	ENSG00000204264	6	32808494	32812480	2.18E-01	1.22E-01	9.56E-02	6.13E-01	7.23E-02	9.26E-02	0
IKBKE	ENSG00000143466	1	206643791	206670223	8.31E-01	9.93E-01	2.01E-01	7.71E-01	5.57E-01	9.28E-02	0
DAZAP2	ENSG00000183283	12	51632076	51665146	4.29E-01	1.08E-01	2.61E-01	2.12E-01	3.95E-01	9.28E-02	0
FOXL1	ENSG00000176678	16	86609974	86615303	3.05E-01	1.32E-01	2.96E-01	5.61E-01	1.72E-01	9.29E-02	0
ENPP5	ENSG00000112796	6	46126924	46138708	9.59E-02	1.18E-01	3.10E-01	5.78E-01	2.06E-01	9.30E-02	0
CASP4	ENSG00000196954	11	104813593	104840163	9.63E-03	4.20E-01	2.42E-01	3.67E-03	2.14E-01	9.31E-02	0
WDR65	ENSG00000243710	1	43637820	43720029	1.17E-01	3.37E-02	6.63E-02	1.97E-01	2.26E-03	9.31E-02	0
PFDN1	ENSG00000113068	5	139624624	139682706	2.63E-01	1.57E-01	1.57E-01	3.86E-02	4.78E-01	9.32E-02	0
TRPM4	ENSG00000130529	19	49660998	49715093	3.50E-01	5.11E-01	1.15E-01	6.16E-01	3.43E-01	9.33E-02	0
ARHGEF5	ENSG00000050327	7	144052381	144077725	8.01E-01	7.61E-01	1.94E-01	1.71E-01	3.88E-01	9.35E-02	0
ZDHHC6	ENSG00000023041	10	114190058	114206672	5.52E-01	8.53E-02	1.96E-01	2.91E-01	6.20E-01	9.36E-02	0
CATSPERB	ENSG00000133962	14	92047040	92247051	7.61E-02	2.02E-01	3.55E-02	6.02E-01	8.11E-01	9.37E-02	0
RAD23A	ENSG00000179262	19	13056669	13064456	6.13E-02	1.94E-02	3.67E-01	4.66E-01	1.32E-01	9.38E-02	0
IFT172	ENSG00000138002	2	27667238	27712656	2.20E-02	2.49E-01	2.58E-02	3.81E-02	3.28E-01	9.38E-02	0
HTR6	ENSG00000158748	1	19991780	20006055	4.44E-01	5.08E-01	6.81E-01	1.56E-01	8.63E-01	9.39E-02	0
ARRDC5	ENSG00000205784	19	4890449	4902879	2.08E-01	1.26E-01	2.80E-01	3.16E-03	1.19E-02	9.39E-02	0
DLG2	ENSG00000150672	11	83166055	85338966	1.46E-01	1.75E-01	8.21E-01	1.35E-01	5.78E-01	9.40E-02	0
PRSS42	ENSG00000178055	3	46871032	46875585	1.62E-01	2.25E-02	9.66E-02	3.65E-01	2.18E-01	9.40E-02	0
STRADA	ENSG00000266173	17	61780192	61819330	6.42E-02	1.02E-01	1.37E-01	1.24E-01	2.88E-01	9.40E-02	0
UBE2E2	ENSG00000182247	3	23244511	23633284	2.44E-02	1.54E-01	1.81E-02	9.83E-03	9.97E-03	9.41E-02	0
PLAT	ENSG00000104368	8	42032236	42065242	6.91E-01	7.90E-01	4.51E-01	7.97E-03	5.96E-01	9.41E-02	0
PKMYT1	ENSG00000127564	16	3018025	3030540	7.56E-01	8.92E-01	2.24E-01	9.04E-01	9.25E-01	9.41E-02	0
GLI1	ENSG00000111087	12	57853918	57866045	7.62E-01	7.96E-01	1.23E-01	2.98E-01	7.62E-01	9.42E-02	0
OR1F1	ENSG00000168124	16	3254247	3255188	3.79E-01	7.43E-01	1.60E-01	7.39E-01	1.20E-01	9.42E-02	0
SHROOM3	ENSG00000138771	4	77356253	77704406	1.10E-03	3.21E-03	1.41E-02	5.24E-02	1.28E-02	9.42E-02	0
KCNN3	ENSG00000143603	1	154669931	154842756	1.44E-02	3.21E-03	5.52E-03	8.84E-02	1.40E-02	9.43E-02	0
FNTB	ENSG00000257365	14	65381203	65529368	4.27E-01	1.90E-01	3.12E-01	4.18E-01	5.77E-01	9.44E-02	0
CDK3	ENSG00000250506	17	73996987	74002080	1.03E-01	2.29E-01	4.63E-01	7.21E-01	3.04E-01	9.44E-02	0
HEXIM1	ENSG00000186834	17	43224684	43229468	1.17E-01	3.98E-03	1.29E-01	7.29E-02	1.87E-02	9.45E-02	0
LRRC15	ENSG00000172061	3	194075976	194090472	3.62E-01	3.25E-01	4.19E-01	3.21E-01	1.93E-01	9.46E-02	0
C16orf62	ENSG00000103544	16	19566562	19718115	8.66E-01	3.08E-01	3.59E-01	6.99E-01	1.30E-01	9.47E-02	0
NEK10	ENSG00000163491	3	27151576	27410951	4.70E-02	2.74E-01	3.04E-02	3.18E-01	1.05E-01	9.47E-02	0
COG5	ENSG00000164597	7	106842000	107204959	5.00E-03	1.34E-01	1.75E-02	2.67E-02	2.51E-03	9.47E-02	0
CENPQ	ENSG00000031691	6	49431091	49460820	1.21E-02	1.26E-01	2.71E-01	1.31E-01	6.81E-02	9.48E-02	0
MATN1	ENSG00000162510	1	31184124	31196434	8.27E-01	6.57E-01	1.80E-01	1.39E-01	1.14E-01	9.48E-02	0
RIPK3	ENSG00000129465	14	24805227	24809251	1.65E-02	7.08E-03	4.48E-02	6.66E-02	8.59E-02	9.49E-02	0
TP53INP2	ENSG00000078804	20	33292094	33301243	3.07E-01	2.35E-02	3.11E-01	3.27E-01	6.25E-02	9.49E-02	0
MKRN1	ENSG00000133606	7	140152840	140179369	2.39E-01	2.05E-01	1.21E-01	2.63E-01	2.35E-01	9.50E-02	0
TIMP3	ENSG00000100234	22	33197687	33259030	2.67E-01	1.87E-01	2.01E-01	5.78E-01	2.67E-01	9.51E-02	0
POLD2	ENSG00000106628	7	44154286	44163957	5.62E-02	3.17E-01	3.11E-02	6.20E-01	7.44E-01	9.51E-02	0
PPP2R3C	ENSG00000092020	14	35554673	35591723	3.35E-02	6.02E-01	3.07E-02	2.56E-01	1.53E-01	9.54E-02	0
VLDLR	ENSG00000147852	9	2621834	2660053	8.95E-02	4.21E-01	1.78E-01	8.43E-01	6.82E-01	9.55E-02	0
AATK	ENSG00000181409	17	79091095	79139877	4.04E-02	1.98E-01	3.31E-02	7.32E-01	1.10E-01	9.55E-02	0

Gene	Ensembl ID	CHR	START	STOP	VHD_CD Pleiotropy P	VHD_GORD Pleiotropy P	VHD_IBD Pleiotropy P	VHD_IBS Pleiotropy P	VHD_PUD Pleiotropy P	VHD_UC Pleiotropy P	d
PPP1R36	ENSG00000165807	14	65016620	65056098	1.59E-01	3.43E-01	9.13E-02	3.25E-01	6.44E-02	9.56E-02	0
SKP2	ENSG00000145604	5	36152091	36184421	7.42E-01	7.07E-01	1.70E-01	5.61E-02	4.90E-01	9.56E-02	0
CEP19	ENSG00000174007	3	196433148	196439164	5.95E-01	9.18E-01	1.55E-01	4.12E-01	6.86E-01	9.56E-02	0
GBX1	ENSG00000164900	7	150845676	150871832	7.13E-01	3.85E-02	1.83E-01	1.07E-01	2.48E-03	9.56E-02	0
RHOA	ENSG00000067560	3	49396578	49450431	1.38E-01	6.92E-01	5.46E-02	9.59E-01	9.45E-01	9.57E-02	0
UBB	ENSG00000170315	17	16284112	16286059	1.58E-01	3.04E-01	1.32E-01	6.14E-01	8.55E-01	9.57E-02	0
NOM1	ENSG00000146909	7	156742417	156765876	7.40E-01	7.90E-01	2.65E-01	3.94E-01	3.15E-01	9.57E-02	0
KIN	ENSG00000151657	10	7792925	7829990	6.71E-01	3.93E-01	4.19E-01	3.09E-01	4.30E-01	9.58E-02	0
ZBTB12	ENSG00000204366	6	31867384	31869769	6.22E-01	1.61E-01	3.44E-01	3.99E-01	4.76E-01	9.58E-02	0
MAD2L1BP	ENSG00000124688	6	43597277	43608689	8.74E-02	2.07E-01	6.54E-02	9.00E-02	5.81E-02	9.58E-02	0
DDX50	ENSG00000107625	10	70661034	70706603	4.52E-01	6.43E-01	8.21E-01	1.79E-01	5.45E-02	9.58E-02	0
APLP1	ENSG00000105290	19	36358801	36370693	6.14E-02	4.22E-01	5.32E-02	4.66E-01	7.46E-01	9.58E-02	0
CDH3	ENSG00000062038	16	68670092	68756519	5.47E-01	4.97E-01	1.67E-01	3.80E-01	6.91E-01	9.59E-02	0
MEP1B	ENSG00000141434	18	29765032	29800367	2.01E-01	4.89E-01	2.50E-01	5.40E-01	6.79E-01	9.60E-02	0
SHB	ENSG00000107338	9	37919131	38069210	3.05E-01	5.19E-02	6.42E-02	2.55E-02	6.42E-02	9.60E-02	0
CLDN24	ENSG00000185758	4	184242917	184243579	1.46E-01	4.16E-02	6.22E-02	3.64E-01	3.80E-01	9.62E-02	0
GLUL	ENSG00000135821	1	182350839	182361341	1.51E-01	2.71E-01	3.36E-01	4.09E-01	4.33E-01	9.62E-02	0
PRDM16	ENSG00000142611	1	2985732	3355185	4.03E-01	7.95E-02	3.74E-01	5.41E-01	1.55E-01	9.63E-02	0
IL1B	ENSG00000125538	2	113587328	113594480	2.36E-01	5.53E-03	1.20E-01	4.45E-01	9.22E-02	9.63E-02	0
KRIT1	ENSG00000001631	7	91828283	91875480	4.46E-02	3.56E-02	1.18E-01	1.17E-01	3.16E-02	9.64E-02	0
ROS1	ENSG00000047936	6	117609463	117747018	2.30E-02	5.13E-01	2.82E-02	3.29E-01	6.98E-02	9.64E-02	0
ZMAT3	ENSG00000172667	3	178735011	178790067	1.27E-01	8.40E-02	1.02E-01	2.98E-01	3.29E-01	9.64E-02	0
MSANTD4	ENSG00000170903	11	105866350	105893130	2.90E-02	1.14E-01	4.90E-02	5.00E-01	1.09E-01	9.64E-02	0
YY1AP1	ENSG00000163374	1	155629237	155658791	8.45E-03	4.52E-01	8.64E-03	1.71E-01	5.00E-01	9.65E-02	0
C5orf52	ENSG00000187658	5	157098561	157107151	1.62E-01	5.92E-01	1.77E-01	3.82E-01	4.39E-02	9.65E-02	0
KRTAP1-5	ENSG00000221852	17	39182278	39183454	2.01E-01	2.64E-02	8.88E-02	1.54E-01	1.47E-02	9.66E-02	0
LY6K	ENSG00000160886	8	143781529	143786545	4.78E-01	8.80E-03	4.10E-01	5.96E-01	4.77E-06	9.66E-02	0
C1QTNF7	ENSG00000163145	4	15341442	15447790	2.30E-01	9.97E-02	3.85E-01	7.93E-01	4.35E-01	9.66E-02	0
CCDC47	ENSG00000108588	17	61822610	61853711	7.43E-02	1.11E-01	1.48E-01	1.21E-01	2.95E-01	9.67E-02	0
ALKBH5	ENSG00000091542	17	18086392	18113268	3.19E-02	1.83E-03	2.30E-02	8.14E-02	1.88E-01	9.67E-02	0
C20orf194	ENSG00000088854	20	3229951	3388272	4.84E-02	2.93E-01	4.94E-01	2.94E-02	1.12E-02	9.69E-02	0
ISG15	ENSG00000187608	1	948803	949920	1.48E-01	1.16E-01	2.48E-01	4.06E-01	2.06E-01	9.70E-02	0
SMCR8	ENSG00000176994	17	18218624	18226517	2.20E-01	2.42E-01	8.04E-02	5.41E-01	8.05E-01	9.70E-02	0
AXDND1	ENSG00000162779	1	179334855	179523870	6.39E-01	9.51E-01	6.09E-01	2.74E-01	3.51E-02	9.71E-02	0
EIF2B2	ENSG00000119718	14	75469614	75476292	5.28E-01	6.38E-01	5.15E-01	6.63E-01	4.76E-01	9.72E-02	0
CLN8	ENSG00000182372	8	1703944	1734738	1.72E-03	3.13E-02	3.18E-03	1.42E-01	2.49E-03	9.74E-02	0
OR4X1	ENSG00000176567	11	48285413	48286330	7.77E-02	5.76E-03	5.59E-02	2.04E-02	1.60E-01	9.75E-02	0
PELI3	ENSG00000174516	11	66234216	66244808	2.89E-01	8.39E-01	4.21E-01	3.79E-01	7.96E-01	9.76E-02	0
OR8H2	ENSG00000181767	11	55872519	55873457	2.06E-02	5.74E-01	2.65E-02	1.60E-01	2.64E-01	9.77E-02	0
INSRR	ENSG00000027644	1	156809855	156828810	3.01E-01	4.22E-01	6.23E-02	6.56E-02	2.57E-01	9.79E-02	0
TDRD1	ENSG00000095627	10	115939029	115992063	1.43E-01	4.23E-01	7.17E-02	1.21E-01	6.92E-01	9.79E-02	0
C5orf24	ENSG00000181904	5	134181370	134195427	1.44E-01	5.01E-01	4.12E-01	1.48E-01	4.04E-01	9.80E-02	0
ITGAX	ENSG00000140678	16	31366455	31394318	1.85E-01	3.73E-01	1.52E-01	8.22E-02	2.61E-01	9.80E-02	0
ISM1	ENSG00000101230	20	13202418	13281298	1.29E-01	4.60E-01	7.56E-02	1.49E-01	2.00E-01	9.80E-02	0
PRSS33	ENSG00000103355	16	2833954	2837949	2.77E-01	1.21E-01	1.30E-01	3.74E-01	3.58E-01	9.81E-02	0
AGPAT6	ENSG00000158669	8	41434706	41482520	1.66E-02	1.01E-01	1.82E-02	9.27E-01	4.91E-01	9.81E-02	0
FAM86C1	ENSG00000158483	11	71498556	71512282	4.66E-01	1.30E-01	2.71E-01	4.80E-01	6.60E-01	9.82E-02	0
COX6A1	ENSG00000111775	12	120875893	120878545	3.56E-01	2.95E-01	4.16E-01	1.23E-02	5.97E-01	9.82E-02	0
ADCK2	ENSG00000133597	7	140372953	140396061	1.23E-01	6.39E-01	6.79E-02	2.28E-01	2.05E-01	9.82E-02	0

Gene	Ensembl ID	CHR	START	STOP	VHD_CD Pleiotropy P	VHD_GORD Pleiotropy P	VHD_IBD Pleiotropy P	VHD_IBS Pleiotropy P	VHD_PUD Pleiotropy P	VHD_UC Pleiotropy P	d
RAI1	ENSG00000108557	17	17584787	17714767	1.80E-01	2.62E-01	7.64E-02	7.53E-02	3.60E-01	9.84E-02	0
SIDT2	ENSG00000149577	11	117049449	117068160	6.58E-01	6.59E-02	2.55E-01	6.31E-01	9.75E-02	9.84E-02	0
CHMP2A	ENSG00000130724	19	59062933	59066491	2.04E-01	1.57E-01	1.06E-01	5.87E-01	2.62E-01	9.85E-02	0
PHYHIP	ENSG00000168490	8	22077222	22089854	2.69E-01	2.58E-01	4.05E-01	4.20E-01	8.05E-01	9.85E-02	0
OR5H14	ENSG00000236032	3	97868170	97869249	7.08E-01	7.82E-01	1.96E-01	4.08E-01	8.29E-01	9.86E-02	0
CPNE8	ENSG00000139117	12	39040624	39301232	7.02E-01	8.52E-02	2.25E-01	2.10E-01	1.12E-01	9.87E-02	0
ATP4B	ENSG00000186009	13	114303119	114312501	5.11E-01	3.23E-01	2.65E-01	5.38E-01	5.79E-01	9.87E-02	0
CCDC53	ENSG00000120860	12	102406705	102455927	2.00E-01	4.01E-01	7.97E-02	5.22E-01	5.80E-01	9.87E-02	0
RD3	ENSG00000198570	1	211649864	211666259	8.89E-01	5.45E-01	2.94E-01	4.76E-01	7.05E-01	9.87E-02	0
ZSWIM2	ENSG00000163012	2	187692562	187713935	1.82E-01	3.55E-01	8.63E-02	3.24E-02	2.25E-01	9.88E-02	0
IL1RL1	ENSG00000115602	2	102927962	102968497	2.21E-02	5.66E-01	1.95E-02	2.84E-01	4.74E-01	9.89E-02	0
C21orf59	ENSG00000159079	21	33964389	33985176	3.54E-01	1.78E-01	1.83E-01	6.60E-02	1.18E-01	9.89E-02	0
CLCN6	ENSG00000011021	1	11866207	11903201	1.16E-01	2.37E-01	3.68E-02	1.22E-01	1.27E-01	9.91E-02	0
OGFR	ENSG00000060491	20	61436187	61445352	3.49E-01	7.35E-02	3.60E-01	9.28E-03	1.81E-02	9.91E-02	0
APIP	ENSG00000149089	11	34874641	34938046	1.21E-02	9.36E-02	3.03E-02	1.82E-01	1.96E-01	9.91E-02	0
ETFA	ENSG00000140374	15	76507696	76603813	8.78E-02	3.63E-01	4.60E-02	2.21E-01	2.21E-01	9.92E-02	0
C9orf9	ENSG00000165698	9	135753414	135765588	3.58E-01	8.06E-01	1.16E-01	2.12E-01	1.96E-01	9.94E-02	0
NEU1	ENSG00000204386	6	31825436	31830683	2.09E-05	1.11E-04	2.67E-03	6.70E-04	6.23E-03	9.94E-02	0
GFM1	ENSG00000168827	3	158362067	158410364	1.26E-01	1.24E-01	6.57E-02	8.36E-02	8.97E-01	9.94E-02	0
RNF7	ENSG00000114125	3	141457046	141466402	2.78E-01	5.34E-01	2.42E-01	5.41E-01	3.99E-02	9.95E-02	0
METRN	ENSG00000103260	16	765115	769655	5.67E-02	3.68E-01	2.80E-02	5.18E-01	1.21E-02	9.95E-02	0
PRPF3	ENSG00000117360	1	150293925	150325671	2.93E-01	2.02E-01	2.04E-01	1.84E-01	6.93E-01	9.95E-02	0
DEAF1	ENSG00000177030	11	644233	706715	3.10E-03	4.05E-03	4.83E-03	5.23E-02	8.67E-02	9.96E-02	0
WDR11	ENSG00000120008	10	122610687	122669036	3.51E-01	6.67E-01	5.29E-01	4.11E-01	3.55E-01	9.97E-02	0
SDHB	ENSG00000117118	1	17345217	17380665	3.67E-01	6.91E-01	4.15E-01	3.24E-01	4.18E-02	9.97E-02	0
UBE2D4	ENSG00000078967	7	43966037	43995735	1.15E-02	2.03E-01	1.46E-02	2.90E-01	8.33E-03	9.97E-02	0
HMGCS2	ENSG00000134240	1	120290619	120311528	3.41E-01	2.55E-02	1.05E-01	1.18E-01	1.60E-01	9.97E-02	0
C3orf58	ENSG00000181744	3	143690640	143767561	1.78E-01	2.25E-02	4.74E-02	7.31E-01	6.09E-01	9.98E-02	0
KBTBD13	ENSG00000234438	15	65369154	65372276	5.40E-02	2.58E-02	6.25E-02	5.12E-02	8.21E-02	9.99E-02	0
CCDC8	ENSG00000169515	19	46913629	46916841	3.23E-01	8.36E-01	5.49E-02	1.49E-01	7.40E-01	9.99E-02	0
IGSF9B	ENSG00000080854	11	133778459	133826880	4.76E-02	7.46E-02	8.25E-02	3.12E-01	4.15E-01	1.00E-01	0
LPIN3	ENSG00000132793	20	39969560	39989222	1.33E-01	7.87E-01	8.92E-02	2.19E-01	7.08E-01	1.00E-01	0
MYCBPAP	ENSG00000136449	17	48585745	48608862	8.40E-02	6.94E-04	7.98E-02	3.09E-02	4.32E-01	1.00E-01	0
CPA4	ENSG00000128510	7	129932974	129964020	2.72E-01	1.38E-01	9.76E-02	6.38E-01	2.18E-01	1.00E-01	0
FAM35A	ENSG00000122376	10	88853918	88951225	2.95E-01	4.31E-01	8.27E-02	3.10E-01	2.54E-01	1.00E-01	0
TRIM52	ENSG00000183718	5	180681417	180688119	2.19E-01	7.19E-02	4.93E-01	1.26E-01	4.59E-01	1.00E-01	0
POLM	ENSG00000122678	7	44111846	44122139	8.69E-02	1.28E-01	4.50E-02	7.47E-01	9.27E-02	1.00E-01	0
PRMT1	ENSG00000126457	19	50179043	50192286	1.07E-01	1.76E-01	7.57E-02	6.44E-01	5.12E-01	1.00E-01	0
GFAP	ENSG00000131095	17	42982376	42994305	6.68E-01	6.73E-01	2.53E-01	2.89E-01	4.68E-01	1.00E-01	0
TMEM160	ENSG00000130748	19	47549165	47551888	6.05E-01	8.46E-01	1.92E-01	2.93E-01	4.26E-01	1.00E-01	0
FAM134C	ENSG00000141699	17	40731531	40762641	1.83E-01	9.38E-01	1.12E-01	8.42E-01	7.50E-01	1.01E-01	0
ARRB2	ENSG00000141480	17	4613784	4624794	2.11E-01	5.73E-01	7.39E-02	8.29E-01	3.74E-01	1.01E-01	0
PDZK1IP1	ENSG00000162366	1	47649265	47656716	8.10E-01	4.04E-01	1.76E-01	2.89E-01	4.67E-01	1.01E-01	0
HR	ENSG00000168453	8	21971928	21990897	3.46E-02	7.55E-02	5.56E-02	1.62E-02	2.86E-02	1.01E-01	0
URGCP	ENSG00000106608	7	43915493	43966010	3.23E-02	2.16E-01	2.69E-02	1.75E-01	1.28E-02	1.01E-01	0
UBXN10	ENSG00000162543	1	20512578	20522541	9.13E-02	6.94E-01	6.83E-02	1.65E-01	9.25E-01	1.01E-01	0
NCOA4	ENSG00000138293	10	51565108	51590734	2.91E-01	6.41E-01	2.96E-01	7.41E-03	1.53E-01	1.01E-01	0
RCE1	ENSG00000173653	11	66610306	66614017	4.02E-01	1.92E-02	1.09E-01	4.73E-01	3.89E-01	1.01E-01	0
PFN4	ENSG00000176732	2	24338241	24346347	1.53E-01	6.01E-01	5.70E-02	2.99E-02	1.29E-01	1.01E-01	0

Gene	Ensembl ID	CHR	START	STOP	VHD_CD Pleiotropy P	VHD_GORD Pleiotropy P	VHD_IBD Pleiotropy P	VHD_IBS Pleiotropy P	VHD_PUD Pleiotropy P	VHD_UC Pleiotropy P	d
FOXQ1	ENSG00000164379	6	1312675	1314992	2.37E-01	3.83E-01	8.44E-02	6.06E-02	1.44E-01	1.01E-01	0
DCBLD1	ENSG00000164465	6	117774980	117891021	3.77E-02	4.14E-01	3.18E-02	1.28E-01	2.78E-01	1.01E-01	0
SRRT	ENSG00000087087	7	100472733	100486285	1.01E-01	7.22E-01	5.92E-02	6.29E-01	8.39E-01	1.01E-01	0
PHLDB2	ENSG00000144824	3	111451344	111695364	1.07E-01	3.99E-03	1.67E-01	8.93E-02	8.51E-02	1.01E-01	0
TM4SF19	ENSG00000145107	3	196046213	196065374	2.80E-02	1.10E-01	2.41E-02	2.09E-01	8.30E-01	1.01E-01	0
NANOS2	ENSG00000188425	19	46416475	46418036	2.19E-01	7.90E-01	9.16E-02	9.21E-01	1.82E-01	1.01E-01	0
POLR1D	ENSG00000186184	13	28194903	28241548	2.93E-01	7.42E-01	8.98E-02	6.70E-01	9.12E-01	1.01E-01	0
GLCCI1	ENSG00000106415	7	8008425	8133902	9.70E-01	4.48E-01	3.75E-01	7.86E-01	8.24E-01	1.01E-01	0
WRAP53	ENSG00000141499	17	7589389	7606820	4.33E-01	3.43E-01	3.31E-01	3.51E-01	2.52E-01	1.02E-01	0
RAN	ENSG00000132341	12	131356424	131362223	1.36E-01	4.62E-01	9.88E-02	6.38E-01	5.17E-01	1.02E-01	0
C8B	ENSG00000021852	1	57394883	57431813	2.18E-02	2.89E-01	3.23E-02	3.72E-01	2.38E-01	1.02E-01	0
TSPAN13	ENSG00000106537	7	16793160	16824161	6.55E-01	7.07E-01	1.99E-01	1.05E-01	3.60E-01	1.02E-01	0
EIF2B4	ENSG00000115211	2	27587219	27593353	4.93E-02	6.29E-01	2.97E-02	2.14E-01	7.23E-01	1.02E-01	0
SYCP3	ENSG00000139351	12	102122426	102133250	7.49E-01	3.81E-01	3.60E-01	5.03E-01	2.11E-01	1.02E-01	0
SPIN1	ENSG00000106723	9	91003334	91093609	2.78E-02	1.77E-01	2.62E-01	9.65E-02	2.13E-01	1.02E-01	0
IRF3	ENSG00000126456	19	50162826	50169132	1.16E-01	6.85E-01	6.78E-02	9.87E-01	1.51E-01	1.02E-01	0
CSNK1A1	ENSG00000113712	5	148871760	148931007	5.31E-02	6.28E-01	3.33E-02	3.97E-01	1.33E-01	1.02E-01	0
HCAR1	ENSG00000196917	12	123104824	123215390	1.63E-01	5.15E-02	5.91E-02	6.48E-02	3.45E-01	1.02E-01	0
CERS2	ENSG00000143418	1	150933059	150947479	2.07E-02	2.08E-01	1.44E-02	8.41E-02	1.39E-01	1.02E-01	0
ACYP1	ENSG00000119640	14	75519924	75536186	5.64E-01	4.05E-01	3.84E-01	9.45E-01	2.53E-01	1.02E-01	0
NAGA	ENSG00000198951	22	42454358	42466846	1.39E-01	6.25E-01	8.38E-02	8.95E-02	1.36E-01	1.02E-01	0
HOXA7	ENSG00000122592	7	27193335	27197555	9.07E-02	1.77E-01	1.89E-01	2.77E-02	4.84E-02	1.02E-01	0
ERCC4	ENSG00000175595	16	14014014	14046202	9.34E-01	2.44E-01	2.27E-01	9.82E-01	2.83E-01	1.02E-01	0
CDC123	ENSG00000151465	10	12237964	12292588	3.62E-02	2.07E-02	4.99E-02	1.89E-01	6.47E-02	1.02E-01	0
DDX25	ENSG00000109832	11	125773271	125793158	2.50E-01	9.17E-02	1.89E-01	1.55E-01	1.92E-01	1.02E-01	0
OR4A15	ENSG00000181958	11	55135360	55136394	3.19E-01	1.03E-01	1.44E-01	9.52E-02	3.45E-01	1.02E-01	0
CCDC14	ENSG00000175455	3	123616152	123680564	2.52E-01	4.49E-02	1.31E-01	6.25E-01	9.06E-02	1.02E-01	0
ATXN7	ENSG00000163635	3	63850233	63989138	8.72E-01	5.31E-01	2.91E-01	6.54E-01	8.88E-01	1.02E-01	0
OR5D14	ENSG00000186113	11	55563032	55563976	2.87E-01	5.36E-01	9.92E-02	3.20E-01	4.12E-01	1.02E-01	0
DCUN1D2	ENSG00000150401	13	114110134	114145267	4.83E-01	1.86E-01	4.83E-01	6.22E-02	1.64E-01	1.03E-01	0
ORC6	ENSG00000091651	16	46723555	46732306	2.18E-01	1.54E-02	6.45E-02	4.80E-01	6.06E-03	1.03E-01	0
OLR1	ENSG00000173391	12	10310902	10324737	7.82E-01	5.83E-02	1.65E-01	5.08E-01	7.87E-03	1.03E-01	0
ADH7	ENSG00000196344	4	100333418	100356894	2.97E-01	1.59E-01	3.12E-01	4.30E-02	1.64E-01	1.03E-01	0
MFSD2A	ENSG00000168389	1	40420802	40435638	2.63E-01	1.37E-01	1.95E-01	2.41E-02	1.60E-01	1.03E-01	0
LOX	ENSG00000113083	5	121398890	121413980	1.55E-01	7.06E-02	8.18E-02	1.04E-01	3.04E-01	1.03E-01	0
MRPL11	ENSG00000174547	11	66202546	66234209	4.69E-01	6.81E-01	4.80E-01	2.70E-01	4.60E-01	1.03E-01	0
TMEM238	ENSG00000233493	19	55890613	55895966	2.39E-02	4.93E-01	2.07E-02	7.08E-01	4.76E-01	1.03E-01	0
BBS1	ENSG00000174483	11	66278077	66301098	3.28E-01	7.51E-01	4.45E-01	2.35E-01	6.19E-01	1.03E-01	0
PLCL2	ENSG00000154822	3	16844159	17132086	1.87E-02	5.71E-01	9.39E-02	2.17E-01	9.06E-03	1.03E-01	0
NPTXR	ENSG00000221890	22	39214457	39239987	2.93E-01	1.58E-01	1.81E-01	5.43E-01	4.55E-01	1.03E-01	0
DLEC1	ENSG00000008226	3	38080696	38165516	8.72E-01	4.56E-01	4.48E-01	3.67E-01	7.98E-01	1.03E-01	0
GRASP	ENSG00000161835	12	52400724	52409673	5.68E-01	7.74E-01	1.58E-01	2.22E-02	7.13E-01	1.03E-01	0
FZR1	ENSG00000105325	19	3506271	3538328	2.68E-01	6.72E-01	5.93E-01	8.15E-01	5.36E-01	1.04E-01	0
CCDC150	ENSG00000144395	2	197504278	197628214	9.84E-02	3.17E-01	7.13E-02	2.61E-01	3.65E-01	1.04E-01	0
EGR2	ENSG00000122877	10	64571756	64679660	5.39E-05	8.80E-01	7.83E-04	7.42E-01	1.62E-01	1.04E-01	0
C5orf22	ENSG00000082213	5	31532373	31555165	4.33E-01	7.82E-01	3.69E-01	2.78E-01	5.75E-01	1.04E-01	0
KCNV2	ENSG00000168263	9	2717502	2730037	3.78E-01	2.13E-01	1.32E-01	4.89E-02	3.45E-01	1.04E-01	0
GIT1	ENSG00000108262	17	27900487	27921072	3.58E-01	5.56E-01	1.97E-01	6.60E-01	4.07E-01	1.04E-01	0
POP7	ENSG00000172336	7	100303676	100305118	1.10E-01	7.35E-01	7.17E-02	6.09E-01	2.45E-01	1.04E-01	0

Gene	Ensembl ID	CHR	START	STOP	VHD_CD Pleiotropy P	VHD_GORD Pleiotropy P	VHD_IBD Pleiotropy P	VHD_IBS Pleiotropy P	VHD_PUD Pleiotropy P	VHD_UC Pleiotropy P	d
ZP1	ENSG00000149506	11	60635035	60643166	1.65E-01	9.08E-01	1.06E-01	6.86E-01	5.77E-01	1.04E-01	0
FAM209B	ENSG00000213714	20	55108302	55111576	2.19E-02	9.95E-02	3.26E-02	8.74E-01	1.57E-01	1.04E-01	0
GUK1	ENSG00000143774	1	228327663	228336685	4.40E-03	5.82E-03	1.11E-02	6.83E-04	3.99E-02	1.04E-01	0
PPAN	ENSG00000130810	19	10216965	10225414	1.35E-01	8.17E-02	5.05E-02	5.43E-01	1.33E-01	1.04E-01	0
MAT2A	ENSG00000168906	2	85766288	85772403	7.97E-02	9.62E-03	4.10E-02	2.29E-01	1.03E-01	1.04E-01	0
TARS2	ENSG00000143374	1	150459887	150480078	2.74E-01	1.77E-01	1.58E-01	3.58E-01	8.16E-01	1.05E-01	0
SLC45A3	ENSG00000158715	1	205626979	205649587	1.06E-01	8.32E-01	1.42E-01	2.12E-01	3.47E-03	1.05E-01	0
VPS45	ENSG00000136631	1	150039369	150117505	4.68E-02	4.43E-01	2.54E-01	1.02E-01	1.03E-01	1.05E-01	0
ANKRD22	ENSG00000152766	10	90581889	90611575	6.99E-02	3.67E-01	3.61E-02	6.07E-01	3.64E-01	1.05E-01	0
CLNS1A	ENSG00000074201	11	77225981	77348850	9.79E-02	1.10E-02	5.73E-02	2.08E-01	2.26E-01	1.05E-01	0
SLC25A26	ENSG00000144741	3	66119285	66438540	1.91E-01	3.30E-01	1.92E-01	2.26E-01	3.28E-01	1.05E-01	0
SPN	ENSG00000197471	16	29674300	29682187	7.02E-01	8.11E-01	2.36E-01	8.63E-01	6.56E-01	1.05E-01	0
HECTD3	ENSG00000126107	1	45468212	45477001	6.30E-02	2.56E-01	7.20E-02	3.79E-01	2.90E-01	1.05E-01	0
IL31	ENSG00000204671	12	122656577	122658746	8.38E-01	3.57E-01	1.23E-01	3.21E-02	5.70E-01	1.05E-01	0
CRHR1	ENSG00000120088	17	43699267	43913194	4.20E-02	1.36E-02	4.82E-02	4.52E-01	4.47E-02	1.05E-01	0
BGLAP	ENSG00000242252	1	156211753	156213112	5.35E-02	5.08E-01	5.23E-01	3.38E-01	2.17E-01	1.05E-01	0
FAM71D	ENSG00000172717	14	67656110	67695267	2.08E-01	2.52E-01	8.46E-02	3.67E-01	3.24E-01	1.05E-01	0
FKBP3	ENSG00000100442	14	45584803	45604522	3.88E-01	6.95E-01	2.51E-01	8.60E-01	1.93E-01	1.05E-01	0
KRTAP4-6	ENSG00000198090	17	39295685	39296739	1.31E-01	1.04E-01	1.18E-01	3.23E-02	2.36E-01	1.05E-01	0
MLEC	ENSG00000110917	12	121124672	121139667	1.30E-01	1.32E-01	2.80E-01	7.23E-02	4.69E-01	1.06E-01	0
XRCC2	ENSG00000196584	7	152341864	152373250	6.28E-01	2.86E-02	4.17E-01	5.85E-02	5.97E-01	1.06E-01	0
HSD17B1	ENSG00000108786	17	40701232	40707231	1.19E-01	7.18E-01	1.16E-01	4.68E-01	5.03E-01	1.06E-01	0
CYGB	ENSG00000161544	17	74523438	74547257	7.24E-01	2.28E-01	2.32E-01	3.45E-01	7.69E-01	1.06E-01	0
SPICE1	ENSG00000163611	3	113161565	113234034	9.13E-02	1.22E-01	1.18E-01	3.74E-01	2.83E-01	1.06E-01	0
LYST	ENSG00000143669	1	235824341	236046940	8.12E-01	4.80E-01	3.12E-01	2.31E-01	6.87E-01	1.06E-01	0
CHD7	ENSG00000171316	8	61591337	61779465	6.89E-02	2.85E-04	1.26E-01	1.58E-02	3.22E-02	1.06E-01	0
HES5	ENSG00000197921	1	2460184	2461684	5.07E-01	7.00E-01	2.88E-01	7.18E-01	7.52E-01	1.06E-01	0
POU4F1	ENSG00000152192	13	79172497	79177673	3.17E-01	4.39E-01	1.59E-01	1.65E-01	5.80E-01	1.06E-01	0
ICK	ENSG00000112144	6	52866077	52926600	9.52E-01	8.81E-01	3.00E-01	4.52E-01	8.85E-01	1.06E-01	0
ZDHHC4	ENSG00000136247	7	6617065	6629005	5.92E-02	8.05E-01	6.75E-02	7.09E-01	5.56E-01	1.06E-01	0
SMTNL1	ENSG00000214872	11	57308979	57318108	3.33E-01	9.07E-02	1.67E-01	3.85E-01	5.15E-01	1.06E-01	0
SH3BGR	ENSG00000185437	21	40817781	40887433	5.51E-02	3.85E-01	7.99E-02	5.70E-03	1.27E-01	1.06E-01	0
HIST1H4E	ENSG00000198518	6	26204858	26206266	1.80E-01	4.16E-01	7.27E-02	3.07E-01	4.37E-01	1.06E-01	0
SH3BGRL3	ENSG00000142669	1	26605667	26608007	4.81E-01	6.59E-01	1.10E-01	7.70E-01	3.90E-01	1.06E-01	0
RSAD1	ENSG00000136444	17	48556161	48563336	5.86E-01	5.56E-01	1.78E-01	3.74E-01	4.62E-01	1.06E-01	0
LPAR2	ENSG00000064547	19	19734477	19739739	5.13E-01	9.55E-02	3.57E-01	6.46E-02	1.95E-01	1.06E-01	0
WDR91	ENSG00000105875	7	134868590	134896316	3.80E-01	2.43E-01	2.48E-01	3.96E-01	2.27E-01	1.06E-01	0
SAA2	ENSG00000134339	11	18260770	18270190	5.30E-01	6.92E-01	1.70E-01	6.02E-01	8.19E-01	1.06E-01	0
KBTBD3	ENSG00000182359	11	105921825	105948492	1.72E-02	8.80E-02	3.18E-02	7.52E-01	6.56E-04	1.06E-01	0
PDLIM4	ENSG00000131435	5	131593364	131609147	2.56E-03	4.89E-01	5.05E-03	4.88E-01	2.43E-01	1.07E-01	0
C1orf122	ENSG00000197982	1	38272651	38275126	2.65E-01	6.72E-01	1.12E-01	4.06E-01	8.60E-01	1.07E-01	0
NDST4	ENSG00000138653	4	115748919	116035032	3.93E-01	7.87E-01	2.92E-01	1.77E-01	4.36E-03	1.07E-01	0
SAMHD1	ENSG00000101347	20	35518632	35580246	3.18E-01	8.65E-02	8.86E-01	8.30E-01	8.86E-02	1.07E-01	0
ST3GAL5	ENSG00000115525	2	86066267	86116137	9.87E-01	2.64E-01	3.51E-01	6.04E-01	8.30E-01	1.07E-01	0
LGALS1	ENSG00000100097	22	38071615	38075813	3.58E-01	7.10E-01	3.75E-01	1.35E-01	4.41E-01	1.07E-01	0
WIPF2	ENSG00000171475	17	38375556	38440388	9.17E-03	7.51E-01	1.84E-02	4.72E-01	1.20E-01	1.07E-01	0
RNASEH2C	ENSG00000172922	11	65482367	65488418	2.98E-01	9.48E-02	1.44E-01	6.22E-02	5.50E-01	1.07E-01	0
DIDO1	ENSG00000101191	20	61509090	61569304	1.03E-01	2.02E-01	3.18E-01	1.77E-01	4.36E-01	1.07E-01	0
EXOC7	ENSG00000182473	17	74077087	74117657	5.99E-01	3.93E-01	1.93E-01	1.98E-01	5.44E-01	1.07E-01	0

Gene	Ensembl ID	CHR	START	STOP	VHD_CD Pleiotropy P	VHD_GORD Pleiotropy P	VHD_IBD Pleiotropy P	VHD_IBS Pleiotropy P	VHD_PUD Pleiotropy P	VHD_UC Pleiotropy P	d
NAV1	ENSG00000134369	1	201592411	201796102	8.34E-03	1.88E-02	5.55E-03	1.92E-01	3.88E-01	1.07E-01	0
CLDN6	ENSG00000184697	16	3064713	3070072	4.87E-01	8.93E-01	3.71E-01	3.99E-01	7.67E-01	1.07E-01	0
S100A7	ENSG00000143556	1	153430220	153433177	6.19E-01	5.65E-01	3.57E-01	9.35E-02	3.26E-01	1.07E-01	0
TSR1	ENSG00000167721	17	2225797	2240801	4.37E-01	6.37E-01	7.49E-02	6.67E-02	3.69E-01	1.07E-01	0
FBXL6	ENSG00000182325	8	145579091	145583036	3.05E-01	5.50E-01	7.31E-02	5.51E-01	1.85E-01	1.07E-01	0
FLT3LG	ENSG00000090554	19	49977464	49989488	2.73E-01	3.41E-01	3.37E-01	9.54E-01	9.23E-01	1.07E-01	0
ALDH2	ENSG00000111275	12	112204691	112247782	5.01E-01	2.52E-02	1.63E-01	6.65E-01	6.14E-01	1.07E-01	0
MTHFR	ENSG00000177000	1	11845780	11866977	1.36E-01	2.52E-01	6.71E-02	1.90E-01	2.54E-01	1.07E-01	0
AADACL2	ENSG00000197953	3	151451704	151479127	2.88E-01	3.17E-01	1.37E-01	2.28E-01	9.98E-02	1.07E-01	0
PHF1	ENSG00000112511	6	33378176	33384230	2.27E-01	4.06E-01	9.02E-02	1.41E-01	2.14E-01	1.07E-01	0
FAM109B	ENSG00000177096	22	42470255	42475445	2.34E-01	6.62E-01	1.55E-01	8.97E-02	2.40E-01	1.07E-01	0
DRAM1	ENSG00000136048	12	102271129	102405908	5.84E-02	2.63E-01	5.12E-02	1.82E-01	9.06E-02	1.07E-01	0
PHC2	ENSG00000134686	1	33789224	33896653	4.68E-01	4.62E-01	1.21E-01	8.21E-01	9.33E-01	1.07E-01	0
P2RX4	ENSG00000135124	12	121647660	121671909	5.61E-01	7.31E-02	2.31E-01	1.59E-01	9.95E-02	1.07E-01	0
OAF	ENSG00000184232	11	120081475	120101041	7.24E-01	8.22E-02	1.24E-01	2.55E-01	6.96E-02	1.07E-01	0
FAIM3	ENSG00000162894	1	207077731	207096592	5.96E-01	8.24E-01	1.76E-01	8.68E-01	7.72E-01	1.08E-01	0
GSTM1	ENSG00000134184	1	110230436	110251661	2.35E-01	8.01E-01	1.03E-01	8.02E-01	2.67E-01	1.08E-01	0
PNPT1	ENSG00000138035	2	55861400	55921045	1.43E-01	7.00E-01	1.32E-01	1.12E-01	6.80E-01	1.08E-01	0
RUNX3	ENSG00000020633	1	25226002	25291612	8.93E-02	1.92E-01	9.36E-02	1.36E-01	6.89E-01	1.08E-01	0
TTYH1	ENSG00000167614	19	54926393	54948080	8.59E-01	3.52E-01	7.49E-01	1.03E-01	7.67E-02	1.08E-01	0
TUBB1	ENSG00000101162	20	57594309	57601709	1.50E-01	7.23E-01	6.54E-02	3.78E-01	1.11E-01	1.08E-01	0
ASAP1	ENSG00000153317	8	131064353	131455906	6.76E-01	1.63E-01	4.05E-01	2.22E-01	2.54E-01	1.08E-01	0
DYNC1H1	ENSG00000197102	14	102430865	102517129	5.67E-01	7.23E-01	1.25E-01	4.64E-01	7.31E-01	1.08E-01	0
MRFAP1L1	ENSG00000178988	4	6709428	6711607	6.77E-01	4.82E-01	2.30E-01	2.84E-01	6.14E-01	1.08E-01	0
LANCL1	ENSG00000115365	2	211295973	211342376	6.28E-01	1.36E-01	2.15E-01	2.48E-01	3.47E-01	1.08E-01	0
LCE3E	ENSG00000185966	1	152538130	152539248	9.21E-02	3.21E-01	7.87E-02	2.16E-01	4.85E-01	1.08E-01	0
C11orf74	ENSG00000166352	11	36616051	36694823	2.67E-01	2.81E-01	1.00E-01	3.95E-01	5.31E-01	1.08E-01	0
USP54	ENSG00000166348	10	75257296	75385711	5.41E-01	1.26E-01	3.21E-01	3.86E-02	5.56E-02	1.08E-01	0
AKR1A1	ENSG00000117448	1	46016215	46035721	2.17E-01	3.27E-01	1.34E-01	2.59E-01	3.83E-01	1.09E-01	0
FDPS	ENSG00000160752	1	155278539	155290457	1.28E-01	3.01E-01	2.03E-01	1.50E-01	4.35E-01	1.09E-01	0
DIO3	ENSG00000197406	14	102027688	102029789	1.92E-01	4.13E-01	6.86E-01	5.48E-02	5.88E-01	1.09E-01	0
MAGEF1	ENSG00000177383	3	184428156	184429836	1.47E-01	3.46E-01	7.80E-02	3.58E-01	5.33E-01	1.09E-01	0
NEK4	ENSG00000114904	3	52744800	52804965	4.31E-01	2.43E-01	2.22E-01	2.27E-01	7.45E-01	1.09E-01	0
ATXN7L3B	ENSG00000253719	12	74931551	74935223	6.26E-01	6.68E-01	6.37E-01	5.00E-01	6.64E-01	1.09E-01	0
ZNF300	ENSG00000145908	5	150273954	150284545	2.09E-01	3.47E-01	7.40E-01	5.14E-01	1.37E-01	1.09E-01	0
RBPMS	ENSG00000157110	8	30241944	30429778	6.00E-01	5.69E-02	3.98E-01	2.71E-01	2.34E-01	1.09E-01	0
ZIK1	ENSG00000171649	19	58089824	58105145	8.61E-01	4.61E-01	3.09E-01	8.16E-01	6.71E-01	1.09E-01	0
CAMKMT	ENSG00000143919	2	44589089	44999731	5.86E-01	2.28E-01	1.19E-01	7.10E-02	7.14E-02	1.09E-01	0
SPCS3	ENSG00000129128	4	177241115	177253396	2.82E-01	5.22E-01	1.35E-01	1.49E-01	9.49E-01	1.09E-01	0
PITPNA	ENSG00000174238	17	1421012	1466110	8.61E-03	1.38E-01	1.34E-02	2.07E-01	2.01E-01	1.09E-01	0
ZC3HAV1	ENSG00000105939	7	138728266	138794465	3.42E-01	2.47E-01	2.01E-01	2.05E-01	6.28E-01	1.09E-01	0
LTC4S	ENSG00000213316	5	179220981	179223648	7.13E-01	7.23E-01	2.06E-01	8.61E-01	6.50E-01	1.09E-01	0
SCFD1	ENSG00000092108	14	31091318	31205018	3.81E-01	3.21E-01	8.88E-02	1.60E-01	8.39E-01	1.09E-01	0
ZFP30	ENSG00000120784	19	38104650	38183238	3.61E-01	3.01E-01	2.17E-01	6.67E-02	1.13E-01	1.09E-01	0
CPNE2	ENSG00000140848	16	57126449	57181878	3.31E-01	4.95E-01	1.57E-01	3.19E-02	7.48E-01	1.09E-01	0
GALNT4	ENSG00000257594	12	89913185	89920039	1.10E-01	1.95E-01	3.95E-01	4.05E-01	2.69E-01	1.09E-01	0
RNF217	ENSG00000146373	6	125283691	125413779	1.45E-01	6.03E-01	7.35E-02	2.78E-01	9.29E-02	1.09E-01	0
CEP85	ENSG00000130695	1	26560691	26605299	5.47E-01	7.75E-01	1.13E-01	7.13E-01	6.21E-01	1.09E-01	0
TCTEX1D2	ENSG00000213123	3	196018090	196045170	7.24E-02	3.92E-01	6.69E-02	4.66E-01	6.57E-01	1.09E-01	0

Gene	Ensembl ID	CHR	START	STOP	VHD_CD Pleiotropy P	VHD_GORD Pleiotropy P	VHD_IBD Pleiotropy P	VHD_IBS Pleiotropy P	VHD_PUD Pleiotropy P	VHD_UC Pleiotropy P	d
RPS8	ENSG00000142937	1	45240923	45244451	2.74E-02	9.85E-01	3.19E-02	8.11E-01	7.72E-01	1.10E-01	0
DOT1L	ENSG00000104885	19	2164148	2232577	3.96E-02	7.42E-03	7.18E-02	2.90E-01	1.26E-02	1.10E-01	0
ITFG3	ENSG00000167930	16	284545	319942	6.55E-01	9.64E-01	3.08E-01	7.66E-02	4.58E-02	1.10E-01	0
ILDR2	ENSG00000143195	1	166882443	166944719	5.28E-01	3.37E-01	1.57E-01	4.83E-01	4.57E-01	1.10E-01	0
OR4C15	ENSG00000181939	11	55321783	55322895	3.66E-01	1.42E-01	1.65E-01	9.33E-02	4.47E-01	1.10E-01	0
ANXA3	ENSG00000138772	4	79472673	79531597	5.25E-01	2.45E-01	3.08E-01	2.40E-01	1.57E-01	1.10E-01	0
FAM179B	ENSG00000198718	14	45431411	45543634	2.81E-01	7.35E-01	2.43E-01	8.05E-01	1.23E-01	1.10E-01	0
DLST	ENSG00000119689	14	75348594	75370448	2.53E-01	1.01E-01	3.48E-01	4.23E-01	5.92E-01	1.10E-01	0
FAM13B	ENSG00000031003	5	137273649	137387650	4.17E-03	2.92E-01	1.12E-02	1.09E-01	3.55E-02	1.10E-01	0
SEC24A	ENSG00000113615	5	133984479	134063513	4.51E-01	5.37E-01	3.86E-01	4.36E-01	2.35E-01	1.10E-01	0
TMEM200C	ENSG00000206432	18	5882071	5895954	5.80E-02	4.17E-01	1.25E-01	3.73E-01	1.55E-02	1.10E-01	0
ACVR1B	ENSG00000135503	12	52345451	52390862	4.04E-01	1.80E-01	1.54E-01	5.08E-02	5.49E-01	1.10E-01	0
TWISTNB	ENSG00000105849	7	19735085	19748710	3.49E-01	7.04E-02	1.36E-01	3.73E-01	3.33E-01	1.10E-01	0
PLEKHB1	ENSG00000021300	11	73357223	73373864	7.71E-01	2.40E-01	3.74E-01	9.05E-01	2.09E-01	1.10E-01	0
DAZAP1	ENSG00000071626	19	1407568	1435719	2.55E-01	2.22E-01	3.18E-01	1.87E-01	6.59E-01	1.10E-01	0
RNF19A	ENSG00000034677	8	101269288	101348446	4.58E-01	3.99E-01	8.96E-02	1.10E-01	2.35E-01	1.10E-01	0
TBX15	ENSG00000092607	1	119425669	119532179	6.13E-01	2.96E-01	2.36E-01	3.11E-01	2.67E-01	1.10E-01	0
GIPR	ENSG00000010310	19	46171502	46186982	5.92E-02	2.07E-02	1.70E-01	1.21E-01	7.41E-03	1.11E-01	0
ADSSL1	ENSG00000185100	14	105190523	105213662	5.63E-01	1.64E-02	3.26E-01	2.80E-01	7.85E-02	1.11E-01	0
KANSL1L	ENSG00000144445	2	210886147	211036107	1.79E-01	5.88E-02	3.84E-02	3.09E-01	5.35E-01	1.11E-01	0
SREBF2	ENSG00000198911	22	42229109	42303312	9.26E-02	1.19E-01	2.81E-01	1.99E-01	8.58E-02	1.11E-01	0
ZC3H12C	ENSG00000149289	11	109964087	110042566	1.45E-01	6.88E-01	6.42E-01	6.24E-01	2.72E-01	1.11E-01	0
COX19	ENSG00000240230	7	938415	1015235	1.51E-01	5.97E-02	1.91E-01	2.36E-01	7.48E-03	1.11E-01	0
UBE2D2	ENSG00000131508	5	138906016	139008018	2.80E-01	1.01E-01	9.28E-02	9.74E-01	1.84E-01	1.11E-01	0
ZNF681	ENSG00000196172	19	23921997	23941693	7.16E-02	6.26E-02	4.17E-02	4.92E-01	9.02E-02	1.11E-01	0
ILF3	ENSG00000129351	19	10764937	10803093	2.14E-01	8.32E-01	1.03E-01	3.89E-03	2.39E-02	1.11E-01	0
HBP1	ENSG00000105856	7	106809406	106842974	1.51E-02	2.39E-01	3.42E-02	4.73E-02	3.28E-02	1.11E-01	0
APOBEC3D	ENSG00000243811	22	39410368	39429281	9.21E-02	3.07E-01	7.78E-01	8.84E-01	4.50E-01	1.11E-01	0
DRD4	ENSG00000069696	11	637293	640706	1.85E-01	2.93E-01	7.91E-02	4.35E-01	7.97E-02	1.11E-01	0
USP16	ENSG00000156256	21	30396950	30426809	4.81E-01	1.12E-01	2.42E-01	1.52E-01	2.10E-01	1.11E-01	0
BCL11A	ENSG00000119866	2	60678302	60780702	5.07E-01	5.01E-01	1.49E-01	5.19E-01	4.33E-01	1.11E-01	0
HPSE	ENSG00000173083	4	84213614	84256306	2.93E-01	9.17E-01	1.20E-01	7.84E-01	3.35E-01	1.11E-01	0
LTF	ENSG00000012223	3	46477136	46526724	5.86E-01	5.79E-01	2.15E-01	8.48E-01	6.36E-01	1.12E-01	0
DOLPP1	ENSG00000167130	9	131843379	131852717	1.57E-02	6.65E-02	5.79E-01	5.11E-02	9.60E-02	1.12E-01	0
ARHGAP22	ENSG00000128805	10	49654077	49864310	2.28E-01	7.60E-01	6.95E-02	7.18E-01	2.78E-01	1.12E-01	0
CCND1	ENSG00000110092	11	69455855	69469242	9.75E-02	8.15E-01	5.54E-02	9.67E-01	5.70E-01	1.12E-01	0
LXN	ENSG00000079257	3	158363611	158390482	1.17E-01	1.12E-01	5.87E-02	8.39E-02	9.07E-01	1.12E-01	0
S100A10	ENSG00000197747	1	151955391	151966866	4.55E-01	5.15E-01	2.17E-01	1.82E-01	2.46E-01	1.12E-01	0
HFE	ENSG00000010704	6	26087509	26098571	7.35E-02	5.84E-02	6.07E-02	7.53E-02	1.96E-01	1.12E-01	0
NAMPT	ENSG00000105835	7	105888731	105926772	4.12E-01	9.71E-01	4.45E-01	5.16E-01	5.60E-01	1.12E-01	0
CRY2	ENSG00000121671	11	45868669	45904798	6.63E-01	7.79E-01	1.91E-01	8.62E-01	3.33E-01	1.12E-01	0
INA	ENSG00000148798	10	105036920	105050108	4.28E-01	2.84E-01	3.00E-01	6.37E-01	3.88E-01	1.12E-01	0
TBC1D8	ENSG00000204634	2	101624079	101869328	2.94E-01	1.44E-04	1.54E-01	2.21E-01	2.77E-01	1.12E-01	0
TNPO2	ENSG00000105576	19	12810008	12834825	9.70E-02	6.35E-01	7.60E-02	1.93E-01	1.56E-01	1.12E-01	0
IFI16	ENSG00000163565	1	158969758	159024945	5.77E-01	1.56E-01	2.33E-01	4.97E-03	4.33E-02	1.12E-01	0
BCAT2	ENSG00000105552	19	49298319	49314286	3.61E-01	4.01E-01	1.45E-01	4.71E-01	8.02E-03	1.12E-01	0
DHRS4L2	ENSG00000187630	14	24439148	24475617	6.11E-03	9.20E-01	1.62E-02	1.79E-02	6.46E-01	1.12E-01	0
RASL12	ENSG00000103710	15	65345679	65369028	9.28E-02	6.80E-03	6.82E-02	8.92E-03	1.08E-01	1.12E-01	0
FBN2	ENSG00000138829	5	127593601	127994878	4.79E-01	8.36E-02	3.03E-01	1.49E-01	1.96E-01	1.12E-01	0

Gene	Ensembl ID	CHR	START	STOP	VHD_CD Pleiotropy P	VHD_GORD Pleiotropy P	VHD_IBD Pleiotropy P	VHD_IBS Pleiotropy P	VHD_PUD Pleiotropy P	VHD_UC Pleiotropy P	d
ZNF419	ENSG00000105136	19	57999079	58006048	1.22E-02	6.30E-01	1.74E-02	3.03E-01	7.73E-02	1.12E-01	0
MLYCD	ENSG00000103150	16	83932731	83949787	3.94E-02	1.38E-01	7.47E-02	2.91E-01	1.96E-01	1.12E-01	0
MST1	ENSG00000173531	3	49721380	49726934	5.83E-02	2.88E-01	4.41E-02	6.21E-01	6.88E-01	1.12E-01	0
TMEM80	ENSG00000177042	11	695428	705028	3.21E-01	2.22E-02	2.16E-01	1.91E-01	6.98E-02	1.13E-01	0
LTV1	ENSG00000135521	6	144164481	144184949	4.91E-01	1.71E-01	4.16E-01	5.85E-01	9.71E-02	1.13E-01	0
MTERF	ENSG00000127989	7	91321323	91510034	1.07E-01	1.76E-01	1.99E-01	2.44E-01	2.37E-01	1.13E-01	0
FKBP10	ENSG00000141756	17	39968932	39979465	1.11E-01	1.39E-01	6.45E-02	6.86E-01	3.77E-01	1.13E-01	0
CCNF	ENSG00000162063	16	2479395	2508855	3.34E-03	6.26E-01	1.12E-01	1.87E-01	1.27E-01	1.13E-01	0
OR6Y1	ENSG00000197532	1	158516918	158517895	3.11E-01	6.98E-01	1.14E-01	2.55E-01	5.61E-01	1.13E-01	0
LRSAM1	ENSG00000148356	9	130213765	130265780	1.92E-01	1.15E-01	1.34E-01	1.55E-01	2.83E-01	1.13E-01	0
PRKD2	ENSG00000105287	19	47177532	47220384	9.24E-01	8.80E-01	2.97E-01	7.86E-01	3.56E-01	1.13E-01	0
ERCC5	ENSG00000134899	13	103497194	103528345	1.69E-02	4.47E-01	4.32E-01	3.60E-01	3.97E-01	1.13E-01	0
RNF38	ENSG00000137075	9	36336393	36487545	3.15E-01	3.98E-01	2.44E-01	5.24E-01	9.04E-01	1.13E-01	0
FITM2	ENSG00000197296	20	42931478	42939809	4.81E-01	1.27E-02	1.52E-01	1.62E-01	4.93E-01	1.13E-01	0
IL20	ENSG00000162891	1	207038699	207042568	6.66E-02	9.54E-01	1.68E-02	7.55E-01	9.63E-01	1.13E-01	0
BCL2L11	ENSG00000153094	2	111876955	111926024	5.75E-03	1.38E-01	6.03E-02	8.34E-01	7.80E-01	1.13E-01	0
QRICH2	ENSG00000129646	17	74270130	74303761	2.16E-01	3.74E-01	1.06E-01	5.45E-01	9.03E-01	1.13E-01	0
ABLIM3	ENSG00000173210	5	148521046	148640105	2.47E-01	6.86E-02	5.11E-01	2.17E-02	3.19E-02	1.13E-01	0
CNNM2	ENSG00000148842	10	104678050	104849978	6.22E-02	4.52E-02	5.84E-02	1.74E-01	3.52E-01	1.13E-01	0
FCN3	ENSG00000142748	1	27695603	27701315	3.32E-01	8.58E-02	2.61E-01	9.24E-01	2.46E-01	1.14E-01	0
C3orf70	ENSG00000187068	3	184795838	184870802	6.83E-01	7.24E-02	2.54E-01	4.99E-01	1.41E-01	1.14E-01	0
FZD3	ENSG00000104290	8	28351729	28431775	8.80E-02	2.25E-01	6.54E-02	5.56E-02	3.63E-01	1.14E-01	0
PPP1R1B	ENSG00000131771	17	37782993	37792879	8.01E-02	9.06E-01	3.42E-02	9.26E-01	2.19E-01	1.14E-01	0
PGBD1	ENSG00000137338	6	28249314	28270326	1.68E-01	4.46E-02	1.65E-01	3.90E-01	7.16E-01	1.14E-01	0
GRB14	ENSG00000115290	2	165349322	165478358	3.73E-01	2.22E-01	1.46E-01	7.34E-02	1.15E-01	1.14E-01	0
LAMA4	ENSG00000112769	6	112429963	112576141	3.04E-01	6.08E-01	9.84E-02	3.26E-01	5.03E-01	1.14E-01	0
QTRT1	ENSG00000213339	19	10812106	10824113	2.72E-02	2.37E-01	2.58E-02	6.47E-03	1.05E-02	1.14E-01	0
LY9	ENSG00000122224	1	160765864	160798051	7.26E-02	7.03E-01	1.01E-01	5.86E-01	7.56E-01	1.14E-01	0
FAM153B	ENSG00000182230	5	175487692	175559261	6.93E-02	1.30E-01	7.08E-02	1.81E-01	2.92E-01	1.14E-01	0
CDRT15L2	ENSG00000214819	17	20483037	20484224	5.27E-01	4.73E-01	1.45E-01	7.23E-01	4.89E-01	1.14E-01	0
DOK6	ENSG00000206052	18	67068291	67516323	2.10E-01	6.55E-01	4.54E-02	8.40E-01	4.97E-03	1.14E-01	0
C20orf78	ENSG00000149443	20	18790372	18810797	2.94E-02	1.21E-02	3.79E-02	7.46E-01	3.13E-02	1.14E-01	0
HIBADH	ENSG00000106049	7	27565061	27702614	2.20E-02	1.08E-02	1.59E-01	4.16E-01	2.45E-01	1.14E-01	0
TCF7L2	ENSG00000148737	10	114710009	114927437	1.91E-01	4.77E-01	7.94E-02	7.84E-01	7.46E-01	1.14E-01	0
PRCD	ENSG00000214140	17	74523668	74549660	7.51E-01	2.64E-01	2.43E-01	3.69E-01	8.13E-01	1.14E-01	0
ISL1	ENSG00000016082	5	50678921	50690564	1.13E-01	5.31E-02	1.81E-01	4.11E-01	3.33E-01	1.15E-01	0
C7orf71	ENSG00000222004	7	26677490	26686889	1.67E-01	8.18E-02	4.68E-01	4.97E-01	4.88E-01	1.15E-01	0
ALX3	ENSG00000156150	1	110602616	110613322	4.49E-01	1.95E-01	8.71E-02	2.11E-01	5.38E-01	1.15E-01	0
OSMR	ENSG00000145623	5	38845960	38945698	5.94E-02	8.95E-01	5.84E-02	6.65E-01	5.46E-01	1.15E-01	0
GORAB	ENSG00000120370	1	170501270	170522587	7.02E-02	4.63E-01	4.82E-02	6.79E-01	1.68E-01	1.15E-01	0
RABEPK	ENSG00000136933	9	127962821	127996437	7.44E-01	8.43E-02	3.20E-01	2.91E-03	3.94E-02	1.15E-01	0
PRR18	ENSG00000176381	6	166719168	166721936	1.75E-02	1.04E-01	2.15E-02	2.58E-01	2.62E-01	1.15E-01	0
GOPC	ENSG00000047932	6	117639374	117923691	5.72E-03	4.98E-01	1.51E-02	1.82E-01	4.32E-02	1.15E-01	0
C10orf55	ENSG00000222047	10	75669727	75682535	5.52E-01	5.96E-01	1.84E-01	5.54E-01	9.44E-01	1.15E-01	0
ABCB5	ENSG00000004846	7	20654830	20816658	6.06E-01	8.54E-01	1.49E-01	8.77E-01	4.48E-01	1.15E-01	0
CARD14	ENSG00000141527	17	78143791	78183130	7.22E-01	1.84E-01	3.89E-01	2.22E-01	3.91E-01	1.15E-01	0
SNRPB	ENSG00000125835	20	2442280	2451499	3.47E-01	8.43E-01	1.07E-01	7.98E-01	7.62E-01	1.15E-01	0
C19orf71	ENSG00000183397	19	3539152	3544028	2.96E-01	6.70E-01	6.49E-01	8.79E-01	7.31E-01	1.16E-01	0
SAV1	ENSG00000151748	14	51098776	51135049	6.02E-01	8.26E-01	2.18E-01	7.51E-01	4.08E-01	1.16E-01	0

Gene	Ensembl ID	CHR	START	STOP	VHD_CD Pleiotropy P	VHD_GORD Pleiotropy P	VHD_IBD Pleiotropy P	VHD_IBS Pleiotropy P	VHD_PUD Pleiotropy P	VHD_UC Pleiotropy P	d
SETDB1	ENSG00000143379	1	150898739	150937213	4.36E-02	2.31E-01	2.20E-02	1.37E-01	2.01E-01	1.16E-01	0
SAFB2	ENSG00000130254	19	5587010	5624057	4.10E-01	7.12E-01	1.59E-01	7.83E-01	8.97E-01	1.16E-01	0
SCARA3	ENSG00000168077	8	27491385	27534293	3.34E-01	1.98E-01	5.90E-01	4.99E-01	1.87E-01	1.16E-01	0
RNASET2	ENSG00000026297	6	167342992	167370679	5.14E-03	8.08E-01	7.02E-03	6.78E-01	8.59E-01	1.16E-01	0
PIK3CA	ENSG00000121879	3	178865902	178957881	1.37E-02	4.94E-03	2.00E-02	1.74E-01	2.44E-02	1.16E-01	0
RPS18	ENSG00000231500	6	33239787	33244287	2.63E-02	3.81E-01	4.54E-02	3.86E-01	9.62E-02	1.16E-01	0
OR6C6	ENSG00000188324	12	55688072	55689016	2.36E-01	8.71E-01	1.25E-01	1.60E-01	1.59E-02	1.16E-01	0
POGLUT1	ENSG00000163389	3	119187785	119213555	5.88E-01	6.30E-01	2.33E-01	6.19E-01	8.83E-01	1.16E-01	0
ARSK	ENSG00000164291	5	94890778	94940768	5.96E-01	4.38E-01	3.53E-01	1.91E-01	1.82E-02	1.16E-01	0
ZNF518B	ENSG00000178163	4	10441498	10459034	5.71E-01	4.20E-03	3.43E-01	9.27E-01	1.03E-01	1.16E-01	0
ADAR	ENSG00000160710	1	154554538	154600475	1.33E-01	5.01E-03	1.13E-01	1.23E-01	3.14E-01	1.16E-01	0
MS4A12	ENSG00000071203	11	60260251	60274903	2.48E-01	9.69E-01	1.39E-01	2.64E-01	7.21E-01	1.16E-01	0
MMP24	ENSG00000125966	20	33814457	33864801	8.50E-01	5.73E-01	2.26E-01	7.76E-01	9.21E-01	1.17E-01	0
SLC17A7	ENSG00000104888	19	49932658	49945617	3.89E-03	7.90E-02	1.72E-02	1.18E-01	1.16E-01	1.17E-01	0
LEPREL4	ENSG00000141696	17	39958199	39968856	1.25E-01	4.06E-01	7.67E-02	4.95E-01	5.50E-01	1.17E-01	0
ACTB	ENSG00000075624	7	5566782	5603415	4.36E-01	5.29E-01	2.54E-01	6.51E-01	4.03E-01	1.17E-01	0
CYTL1	ENSG00000170891	4	5016313	5021199	2.71E-01	8.22E-01	1.49E-01	2.25E-01	1.85E-01	1.17E-01	0
HIVEP1	ENSG00000095951	6	12008995	12165232	7.12E-01	1.38E-01	3.04E-01	2.34E-01	3.40E-01	1.17E-01	0
TRAPPC10	ENSG00000160218	21	45432200	45526433	1.21E-02	4.89E-01	1.80E-02	2.83E-01	7.04E-01	1.17E-01	0
SIVA1	ENSG00000184990	14	105219437	105234831	4.63E-01	3.26E-02	2.49E-01	5.58E-01	6.31E-02	1.17E-01	0
ZC2HC1B	ENSG00000118491	6	144185573	144259483	2.73E-01	2.25E-01	1.59E-01	4.53E-01	6.99E-02	1.17E-01	0
GTF3C4	ENSG00000125484	9	135545422	135570342	7.07E-01	3.83E-01	2.40E-01	2.53E-01	2.08E-01	1.17E-01	0
HNRNPA2B1	ENSG00000122566	7	26229547	26241149	2.52E-01	1.97E-01	1.62E-01	1.80E-01	3.00E-01	1.17E-01	0
PILRA	ENSG00000085514	7	99965153	99997719	2.35E-01	9.51E-01	8.75E-02	3.86E-01	7.89E-01	1.17E-01	0
USP5	ENSG00000111667	12	6961292	6975796	2.23E-01	8.96E-01	3.72E-01	8.17E-01	9.23E-01	1.17E-01	0
ROPN1L	ENSG00000145491	5	10441636	10472141	4.82E-01	2.55E-01	9.32E-02	3.62E-01	4.72E-01	1.17E-01	0
LAMP1	ENSG00000185896	13	113951556	113977987	7.90E-01	3.03E-01	2.91E-01	2.42E-01	7.21E-01	1.17E-01	0
YARS2	ENSG00000139131	12	32880424	32908836	1.61E-01	1.48E-01	6.69E-01	7.57E-02	6.21E-01	1.18E-01	0
EXOC2	ENSG00000112685	6	485133	693117	4.92E-01	3.22E-01	1.46E-01	7.86E-01	4.12E-01	1.18E-01	0
DIO1	ENSG00000211452	1	54356912	54376759	2.31E-01	9.86E-02	6.08E-01	2.84E-01	1.37E-04	1.18E-01	0
LIN9	ENSG00000183814	1	226418850	226497570	1.30E-01	5.48E-01	7.47E-02	4.73E-01	2.17E-01	1.18E-01	0
NQO1	ENSG00000181019	16	69740899	69760854	7.54E-01	8.48E-01	1.60E-01	2.53E-01	3.98E-01	1.18E-01	0
GGPS1	ENSG00000152904	1	235490665	235507847	5.71E-02	2.24E-02	6.45E-02	4.39E-02	5.85E-02	1.18E-01	0
STRAP	ENSG00000023734	12	16035325	16056412	8.16E-02	3.36E-01	1.09E-01	6.46E-01	3.93E-01	1.18E-01	0
FXYD3	ENSG00000089356	19	35606732	35615228	2.01E-01	9.01E-01	7.08E-02	8.18E-01	8.50E-01	1.18E-01	0
TMEM74B	ENSG00000125895	20	1161205	1166059	3.28E-01	4.23E-01	2.74E-01	5.36E-02	3.87E-01	1.18E-01	0
PSMF1	ENSG00000125818	20	1093906	1160596	1.98E-01	2.49E-01	4.48E-01	2.45E-01	3.51E-01	1.18E-01	0
ATL2	ENSG00000119787	2	38522022	38604427	2.29E-01	8.91E-01	5.47E-01	8.96E-01	3.62E-01	1.18E-01	0
PEX1	ENSG00000127980	7	92116334	92157845	2.66E-01	1.80E-01	2.27E-01	5.82E-01	3.95E-01	1.18E-01	0
AKAP8	ENSG00000105127	19	15464196	15490609	4.48E-01	9.13E-01	2.33E-01	9.82E-01	9.20E-01	1.18E-01	0
CHEK1	ENSG00000149554	11	125495036	125546150	4.65E-01	5.59E-01	3.17E-01	5.01E-01	2.91E-01	1.18E-01	0
GPR55	ENSG00000135898	2	231772033	231825781	7.15E-02	6.21E-01	4.15E-02	3.74E-01	5.73E-01	1.18E-01	0
THEMIS	ENSG00000172673	6	128029217	128239776	2.99E-03	2.37E-01	6.00E-03	2.21E-01	4.26E-02	1.18E-01	0
DPM1	ENSG00000000419	20	49551404	49575092	7.83E-01	5.63E-01	2.68E-01	3.55E-01	1.16E-01	1.18E-01	0
KCNMB3	ENSG00000171121	3	178957530	178984790	2.20E-02	3.00E-02	2.82E-02	2.06E-01	1.19E-01	1.19E-01	0
CDON	ENSG00000064309	11	125825691	125933230	1.91E-01	2.32E-01	1.26E-01	5.60E-01	3.49E-01	1.19E-01	0
KRT86	ENSG00000170442	12	52643084	52702947	7.95E-01	3.68E-01	1.94E-01	6.06E-01	2.20E-01	1.19E-01	0
TIGD2	ENSG00000180346	4	90033968	90036050	6.32E-02	7.08E-01	8.33E-02	5.61E-01	5.59E-01	1.19E-01	0
MYO10	ENSG00000145555	5	16665395	16936372	1.95E-01	8.90E-01	1.14E-01	9.68E-01	8.34E-01	1.19E-01	0

Gene	Ensembl ID	CHR	START	STOP	VHD_CD Pleiotropy P	VHD_GORD Pleiotropy P	VHD_IBD Pleiotropy P	VHD_IBS Pleiotropy P	VHD_PUD Pleiotropy P	VHD_UC Pleiotropy P	d
CACNG1	ENSG00000108878	17	65040706	65052909	1.99E-01	4.92E-01	7.29E-02	6.04E-01	3.16E-01	1.19E-01	0
COL9A3	ENSG00000092758	20	61447596	61472511	3.23E-01	1.12E-01	2.52E-01	4.33E-01	1.05E-01	1.19E-01	0
AHR	ENSG00000106546	7	17338246	17385776	7.31E-01	5.37E-01	4.52E-01	6.84E-01	7.92E-01	1.19E-01	0
ZNF197	ENSG00000186448	3	44626380	44689963	4.44E-01	8.77E-01	2.66E-01	4.24E-01	1.06E-01	1.19E-01	0
BIRC7	ENSG00000101197	20	61867235	61871859	2.12E-02	1.16E-01	1.70E-02	1.21E-01	3.69E-01	1.19E-01	0
SLC31A2	ENSG00000136867	9	115913222	115926417	7.71E-01	4.42E-01	3.79E-01	3.40E-01	1.91E-01	1.19E-01	0
VAMP3	ENSG00000049245	1	7831329	7841492	1.12E-01	6.12E-01	8.61E-02	5.00E-01	7.09E-01	1.19E-01	0
C14orf182	ENSG00000214900	14	50448430	50474238	5.30E-01	9.61E-01	5.12E-01	8.71E-01	8.24E-01	1.19E-01	0
OR52E4	ENSG00000180974	11	5905501	5906527	4.92E-02	1.80E-01	5.40E-02	2.43E-01	9.41E-01	1.19E-01	0
C8orf44	ENSG00000213865	8	67579850	67597799	3.38E-01	1.66E-01	9.65E-02	2.52E-01	7.07E-01	1.19E-01	0
GADD45A	ENSG00000116717	1	68150744	68154021	1.95E-01	1.86E-01	1.27E-01	4.03E-01	1.25E-01	1.19E-01	0
WIPI1	ENSG00000070540	17	66417089	66453654	5.00E-02	2.44E-01	2.77E-01	3.21E-02	5.95E-01	1.19E-01	0
CWC15	ENSG00000150316	11	94695787	94706776	6.15E-02	1.33E-01	7.81E-02	7.68E-01	2.16E-01	1.20E-01	0
IL31RA	ENSG00000164509	5	55147207	55218678	1.95E-01	1.18E-01	1.13E-01	6.50E-01	6.83E-01	1.20E-01	0
B3GALNT1	ENSG00000169255	3	160801671	160823172	9.20E-01	2.07E-01	2.58E-01	1.23E-01	9.09E-02	1.20E-01	0
DCAF7	ENSG00000136485	17	61627822	61671639	3.06E-01	2.74E-01	2.61E-01	1.29E-01	1.87E-01	1.20E-01	0
KDELC1	ENSG00000134901	13	103436631	103451357	1.44E-01	3.59E-01	1.56E-01	1.44E-01	1.24E-01	1.20E-01	0
SCRG1	ENSG00000164106	4	174305852	174327531	6.06E-02	3.76E-01	4.73E-01	1.76E-01	6.45E-01	1.20E-01	0
STT3A	ENSG00000134910	11	125461607	125495110	5.50E-01	7.29E-01	3.20E-01	4.84E-01	2.40E-01	1.20E-01	0
EZR	ENSG00000092820	6	159186773	159240444	2.82E-01	1.49E-01	1.04E-01	1.62E-01	2.66E-03	1.20E-01	0
ORAI2	ENSG00000160991	7	102073553	102097268	3.69E-02	3.75E-01	3.44E-02	9.23E-02	3.18E-01	1.20E-01	0
FFAR1	ENSG00000126266	19	35842445	35843367	4.18E-01	3.85E-01	5.29E-01	3.94E-01	9.02E-01	1.20E-01	0
XRCC3	ENSG00000126215	14	104163946	104181841	3.12E-02	3.81E-01	7.76E-02	5.88E-01	7.71E-01	1.20E-01	0
KCTD18	ENSG00000155729	2	201353675	201384507	2.88E-01	4.45E-01	1.32E-01	1.06E-02	3.42E-02	1.20E-01	0
CNTN2	ENSG00000184144	1	205012325	205047627	1.97E-01	1.98E-01	8.24E-02	3.03E-01	5.31E-01	1.20E-01	0
FRY	ENSG00000073910	13	32605437	32870794	6.48E-01	1.17E-01	2.04E-01	5.90E-01	5.05E-01	1.20E-01	0
SGPP1	ENSG00000126821	14	64150932	64194757	6.05E-01	2.40E-01	3.81E-01	9.46E-02	4.59E-01	1.20E-01	0
FBRS	ENSG00000156860	16	30669752	30682135	2.47E-02	1.17E-01	5.27E-02	3.23E-01	1.80E-01	1.20E-01	0
TTC18	ENSG00000156042	10	75013517	75118617	6.94E-01	4.85E-01	2.32E-01	9.85E-02	3.90E-02	1.20E-01	0
TMEM120B	ENSG00000188735	12	122150658	122220907	2.15E-01	1.40E-01	1.86E-01	4.22E-02	1.43E-01	1.20E-01	0
FCHSD1	ENSG00000197948	5	141018869	141030986	4.07E-01	4.91E-01	3.03E-01	1.75E-01	7.94E-01	1.20E-01	0
OPRM1	ENSG00000112038	6	154331631	154568001	6.35E-01	9.11E-01	3.64E-01	7.00E-01	4.48E-01	1.20E-01	0
QRSL1	ENSG00000130348	6	107077453	107116292	3.25E-01	5.39E-01	1.23E-01	4.08E-01	2.94E-01	1.21E-01	0
ITGB3	ENSG00000259753	17	45331263	45421658	1.89E-01	2.95E-01	4.90E-02	1.94E-01	1.22E-01	1.21E-01	0
PIK3R3	ENSG00000117461	1	46505812	46642160	2.42E-01	1.82E-01	1.48E-01	5.67E-01	4.26E-01	1.21E-01	0
TSFM	ENSG00000123297	12	58176372	58201854	8.68E-03	1.38E-01	1.40E-02	1.99E-03	6.35E-01	1.21E-01	0
TMEM204	ENSG00000131634	16	1578689	1605581	2.69E-01	3.97E-01	6.71E-01	9.36E-01	5.59E-01	1.21E-01	0
SLC25A3	ENSG00000075415	12	98987369	98995946	3.78E-01	1.92E-01	5.37E-01	6.40E-01	4.00E-03	1.21E-01	0
DNAJC25	ENSG00000059769	9	114393632	114432508	4.04E-01	3.25E-01	1.03E-01	1.36E-01	5.78E-02	1.21E-01	0
OR1L1	ENSG00000173679	9	125423845	125424927	8.04E-02	8.72E-01	1.80E-02	9.25E-01	8.62E-01	1.21E-01	0
PPBP	ENSG00000163736	4	74852755	74853914	4.56E-01	8.92E-01	1.01E-01	3.67E-01	4.30E-01	1.21E-01	0
RIN1	ENSG00000174791	11	66097713	66104311	3.06E-01	7.96E-01	9.63E-02	3.05E-01	1.60E-01	1.21E-01	0
FGF5	ENSG00000138675	4	81187753	81257834	2.43E-02	5.57E-02	5.46E-02	5.82E-02	1.20E-01	1.21E-01	0
TNPO3	ENSG00000064419	7	128594948	128695198	5.79E-01	8.95E-01	3.63E-01	5.84E-01	9.80E-01	1.21E-01	0
SLC22A24	ENSG00000197658	11	62847412	62911693	1.31E-01	2.45E-03	6.21E-02	4.19E-02	5.93E-01	1.21E-01	0
ACER3	ENSG00000078124	11	76571911	76737841	2.91E-01	8.13E-01	1.58E-01	5.54E-01	8.01E-01	1.22E-01	0
ANO8	ENSG00000074855	19	17434032	17445638	3.59E-01	1.60E-01	9.39E-02	1.20E-02	6.70E-02	1.22E-01	0
PRMT3	ENSG00000185238	11	20409076	20530840	1.24E-01	3.60E-01	5.92E-02	8.74E-01	2.36E-01	1.22E-01	0
OPRD1	ENSG00000116329	1	29138654	29190208	2.11E-02	1.08E-01	2.10E-02	4.40E-01	4.48E-01	1.22E-01	0

Gene	Ensembl ID	CHR	START	STOP	VHD_CD Pleiotropy P	VHD_GORD Pleiotropy P	VHD_IBD Pleiotropy P	VHD_IBS Pleiotropy P	VHD_PUD Pleiotropy P	VHD_UC Pleiotropy P	d
DCC	ENSG00000187323	18	49866542	51057784	3.44E-02	2.38E-02	4.22E-02	4.12E-05	4.25E-02	1.22E-01	0
DERL3	ENSG00000099958	22	24176690	24181315	2.47E-01	1.11E-01	1.27E-01	5.99E-01	1.69E-01	1.22E-01	0
TTLL1	ENSG00000100271	22	43435522	43485434	5.76E-01	4.16E-01	2.35E-01	4.00E-01	4.43E-01	1.22E-01	0
LGALS9	ENSG00000168961	17	25956824	25976586	1.24E-01	3.13E-01	9.54E-02	8.15E-02	8.58E-01	1.22E-01	0
ZNF266	ENSG00000174652	19	9523272	9546254	6.15E-01	2.45E-01	5.86E-01	8.10E-01	7.84E-01	1.22E-01	0
GLDN	ENSG00000186417	15	51633826	51700210	5.67E-01	5.08E-02	3.70E-01	2.13E-01	7.43E-01	1.22E-01	0
C2orf66	ENSG00000187944	2	197669726	197675000	1.95E-01	6.21E-01	1.32E-01	8.54E-01	9.34E-02	1.22E-01	0
ANPEP	ENSG00000166825	15	90328120	90358633	3.55E-01	2.22E-01	8.64E-01	7.04E-01	4.05E-01	1.22E-01	0
CALB1	ENSG00000104327	8	91070836	91107703	1.17E-02	1.58E-01	5.69E-02	2.44E-01	2.88E-02	1.22E-01	0
SALL3	ENSG00000256463	18	76740275	76762677	6.67E-01	6.91E-01	7.61E-01	2.74E-01	2.53E-01	1.22E-01	0
VAV3	ENSG00000134215	1	108113782	108507766	1.82E-01	4.35E-02	1.16E-01	2.48E-01	2.97E-01	1.22E-01	0
ZNF707	ENSG00000181135	8	144766622	144796068	2.53E-01	3.72E-01	1.08E-01	6.82E-01	6.71E-01	1.22E-01	0
RAVER1	ENSG00000161847	19	10426888	10444316	1.69E-01	9.37E-02	5.91E-02	2.26E-01	1.73E-01	1.22E-01	0
UQCR11	ENSG00000127540	19	1597171	1605480	8.26E-01	1.18E-01	3.94E-01	3.42E-01	2.50E-01	1.22E-01	0
MACF1	ENSG00000127603	1	39546988	39952849	9.52E-03	3.97E-02	1.79E-01	2.82E-04	3.17E-01	1.22E-01	0
FKBP1B	ENSG00000119782	2	24272571	24286551	1.64E-01	2.63E-01	5.26E-02	1.32E-02	2.85E-01	1.22E-01	0
FAM149A	ENSG00000109794	4	187025573	187093821	1.40E-01	8.23E-01	5.32E-02	2.01E-01	3.49E-01	1.22E-01	0
KIAA1279	ENSG00000198954	10	70748487	70776738	3.96E-01	3.50E-01	7.75E-01	2.65E-01	3.09E-02	1.23E-01	0
MAEA	ENSG00000090316	4	1283639	1333935	3.54E-01	5.32E-01	1.80E-01	6.33E-01	1.12E-01	1.23E-01	0
PSMB6	ENSG00000142507	17	4699439	4701790	3.30E-02	6.72E-01	7.57E-02	1.48E-01	2.05E-01	1.23E-01	0
IPO11	ENSG00000086200	5	61699799	61924409	5.03E-01	3.42E-01	9.76E-02	1.34E-01	2.71E-01	1.23E-01	0
CCT5	ENSG00000150753	5	10250033	10266524	5.01E-01	1.71E-01	4.02E-01	6.19E-02	1.50E-01	1.23E-01	0
WDR75	ENSG00000115368	2	190306159	190340291	2.63E-01	2.47E-01	4.48E-01	7.90E-01	2.32E-01	1.23E-01	0
B3GALNT2	ENSG00000162885	1	235613238	235667781	9.07E-01	4.27E-02	2.99E-01	1.89E-01	4.91E-01	1.23E-01	0
STAMBPL1	ENSG00000138134	10	90639491	90734910	3.60E-01	7.83E-01	1.70E-01	1.38E-01	8.04E-01	1.23E-01	0
PSMC3	ENSG00000165916	11	47440320	47447993	6.66E-02	4.09E-02	4.75E-02	2.25E-01	3.49E-01	1.23E-01	0
MAPT	ENSG00000186868	17	43971748	44105700	4.47E-02	1.19E-02	5.30E-02	3.81E-01	4.26E-02	1.23E-01	0
GNPTAB	ENSG00000111670	12	102139275	102224716	6.15E-01	7.54E-01	4.05E-01	2.23E-01	2.51E-01	1.23E-01	0
HNMT	ENSG00000150540	2	138721590	138773930	3.80E-01	1.75E-02	6.36E-01	4.04E-01	3.38E-01	1.23E-01	0
ROPN1B	ENSG00000114547	3	125687987	125702297	1.57E-01	2.02E-01	2.64E-01	1.64E-01	4.89E-01	1.23E-01	0
COPS4	ENSG00000138663	4	83955600	83996971	7.39E-02	1.80E-01	4.41E-02	3.34E-01	2.80E-01	1.23E-01	0
RPL11	ENSG00000142676	1	24018269	24022915	1.51E-01	6.72E-01	1.58E-01	2.75E-01	5.87E-01	1.23E-01	0
PACSIN2	ENSG00000100266	22	43231418	43411151	6.76E-01	5.89E-01	2.05E-01	3.55E-01	6.52E-01	1.23E-01	0
IP6K3	ENSG00000161896	6	33689444	33714762	6.40E-01	6.01E-01	4.12E-01	1.16E-01	6.33E-01	1.23E-01	0
MORC2	ENSG00000133422	22	31321117	31364284	2.11E-02	2.27E-01	3.33E-02	4.87E-01	7.44E-02	1.23E-01	0
LONRF2	ENSG00000170500	2	100889753	100939195	3.18E-01	5.49E-01	4.07E-01	7.69E-02	7.12E-01	1.23E-01	0
NFS1	ENSG00000244005	20	34255977	34287281	3.08E-01	4.67E-02	1.12E-01	1.03E-01	2.99E-01	1.24E-01	0
SYNRG	ENSG00000006114	17	35874900	35969544	3.58E-01	4.71E-01	1.89E-01	6.59E-01	3.70E-01	1.24E-01	0
LRRC61	ENSG00000127399	7	150019728	150035239	5.12E-01	1.86E-01	1.55E-01	1.73E-02	2.02E-01	1.24E-01	0
EPRS	ENSG00000136628	1	220141943	220220000	1.26E-01	1.13E-01	5.88E-02	2.67E-01	3.23E-01	1.24E-01	0
ZBTB24	ENSG00000112365	6	109783797	109804440	6.80E-02	2.71E-01	1.48E-01	4.54E-01	3.60E-01	1.24E-01	0
GRK5	ENSG00000198873	10	120967101	121215131	1.38E-01	1.36E-01	1.86E-01	3.48E-02	3.08E-03	1.24E-01	0
RPL13A	ENSG00000142541	19	49990811	49995565	3.84E-01	6.77E-01	5.40E-01	9.38E-01	3.90E-01	1.24E-01	0
APBA2	ENSG00000034053	15	29129629	29410518	7.70E-01	7.45E-01	2.83E-01	5.61E-02	3.06E-01	1.24E-01	0
IL10RA	ENSG00000110324	11	117857063	117872196	5.90E-02	7.96E-01	3.20E-02	9.54E-01	4.11E-01	1.24E-01	0
CELF5	ENSG00000161082	19	3224701	3297074	3.35E-01	9.06E-01	6.56E-01	7.25E-01	6.70E-01	1.24E-01	0
POFUT1	ENSG00000101346	20	30795683	30826470	3.78E-02	4.69E-01	5.76E-02	1.49E-02	4.21E-01	1.24E-01	0
C10orf118	ENSG00000165813	10	115880621	115933979	1.93E-01	5.65E-01	9.22E-02	1.43E-01	4.27E-01	1.24E-01	0
MPP4	ENSG00000082126	2	202509593	202563417	1.86E-01	2.98E-01	1.50E-01	2.60E-01	5.27E-01	1.24E-01	0

Gene	Ensembl ID	CHR	START	STOP	VHD_CD Pleiotropy P	VHD_GORD Pleiotropy P	VHD_IBD Pleiotropy P	VHD_IBS Pleiotropy P	VHD_PUD Pleiotropy P	VHD_UC Pleiotropy P	d
MTMR6	ENSG00000139505	13	25802307	25862147	9.80E-01	1.03E-01	2.33E-01	1.99E-01	2.01E-01	1.24E-01	0
PLOD2	ENSG00000152952	3	145787227	145881440	3.25E-01	2.55E-01	1.36E-01	9.12E-03	1.41E-01	1.24E-01	0
EBNA1BP2	ENSG00000117395	1	43629846	43736607	1.34E-01	5.35E-02	6.16E-02	2.68E-01	2.97E-03	1.25E-01	0
PTPN2	ENSG00000175354	18	12785477	12929642	2.74E-03	6.20E-01	4.57E-03	5.68E-01	3.82E-01	1.25E-01	0
FAM21C	ENSG00000172661	10	46222648	46288409	1.93E-01	6.87E-01	2.40E-01	3.59E-05	4.04E-01	1.25E-01	0
STK25	ENSG00000115694	2	242432089	242449145	3.27E-01	8.48E-01	1.14E-01	7.53E-02	4.23E-01	1.25E-01	0
SLC22A5	ENSG00000197375	5	131705444	131731306	3.08E-03	6.29E-01	4.13E-03	7.96E-01	7.26E-01	1.25E-01	0
G2E3	ENSG00000092140	14	31028329	31089269	5.26E-01	2.12E-01	9.49E-02	1.28E-01	8.33E-01	1.25E-01	0
TERF1	ENSG00000147601	8	73921099	73960357	1.13E-02	1.23E-01	2.16E-02	1.23E-02	6.57E-02	1.25E-01	0
ZNF473	ENSG00000142528	19	50528971	50556671	3.87E-01	9.07E-02	1.27E-01	4.70E-01	3.64E-02	1.25E-01	0
SLC38A7	ENSG00000103042	16	58699013	58719008	3.27E-01	5.25E-02	5.16E-01	6.97E-02	2.96E-01	1.25E-01	0
HPS3	ENSG00000163755	3	148847371	148891519	2.75E-01	4.31E-01	1.51E-01	6.47E-01	4.96E-01	1.25E-01	0
ETFDH	ENSG00000171503	4	159593277	159630775	1.90E-01	1.94E-01	7.34E-02	3.99E-01	8.55E-01	1.25E-01	0
SPRR2B	ENSG00000196805	1	153042700	153113927	5.17E-03	1.71E-01	1.81E-02	4.00E-01	3.81E-02	1.25E-01	0
MPP5	ENSG00000072415	14	67707826	67802536	6.91E-02	1.54E-01	2.03E-01	6.06E-01	3.34E-01	1.25E-01	0
ZBTB7B	ENSG00000160685	1	154975127	154990998	1.24E-02	2.93E-03	9.14E-03	2.55E-01	2.33E-03	1.25E-01	0
CDK5RAP3	ENSG00000108465	17	46045176	46059140	1.21E-01	2.80E-01	2.61E-01	3.04E-02	2.94E-01	1.25E-01	0
SPEN	ENSG00000065526	1	16174359	16266955	4.76E-01	6.93E-01	1.83E-01	6.29E-01	8.44E-01	1.25E-01	0
LTN1	ENSG00000198862	21	30300466	30365270	1.31E-01	1.69E-01	9.66E-02	2.13E-01	3.34E-01	1.25E-01	0
DCT	ENSG00000080166	13	95089558	95131936	3.80E-02	1.74E-02	7.21E-02	1.38E-01	2.09E-01	1.25E-01	0
AP1M1	ENSG00000072958	19	16308389	16346160	4.91E-01	9.67E-02	2.22E-01	5.48E-01	4.16E-02	1.26E-01	0
PLEKHH3	ENSG00000068137	17	40819932	40829048	5.96E-02	8.83E-01	2.69E-02	9.35E-01	5.95E-01	1.26E-01	0
WDR26	ENSG00000162923	1	224572845	224624735	2.31E-01	2.15E-01	1.36E-01	2.56E-01	3.06E-01	1.26E-01	0
DNAJC9	ENSG00000213551	10	74943120	75008620	3.72E-01	4.13E-01	1.47E-01	5.52E-02	2.73E-02	1.26E-01	0
GJB2	ENSG00000165474	13	20761609	20767037	1.29E-02	1.32E-01	2.52E-02	3.48E-01	3.28E-01	1.26E-01	0
C19orf35	ENSG00000188305	19	2274631	2282181	5.83E-01	6.01E-02	1.10E-01	8.86E-01	7.04E-03	1.26E-01	0
ANP32E	ENSG00000143401	1	150190717	150208504	2.59E-01	2.63E-01	1.23E-01	4.25E-01	5.98E-01	1.26E-01	0
RABEP2	ENSG00000177548	16	28915742	28947847	5.30E-02	5.28E-01	3.47E-02	3.79E-01	7.19E-01	1.26E-01	0
ART3	ENSG00000156219	4	76932337	77033955	1.01E-01	4.43E-01	8.72E-02	7.97E-01	2.08E-01	1.26E-01	0
STH	ENSG00000256762	17	44076616	44077060	4.74E-02	1.21E-02	5.26E-02	3.93E-01	4.00E-02	1.26E-01	0
ARC	ENSG00000198576	8	143692405	143696833	3.85E-03	1.02E-01	1.11E-02	1.78E-02	8.72E-01	1.26E-01	0
GOLGA1	ENSG00000136935	9	127640646	127710771	1.34E-01	5.70E-03	8.57E-02	1.95E-02	5.22E-03	1.26E-01	0
SSR1	ENSG00000124783	6	7268539	7347679	3.45E-01	1.10E-01	4.63E-01	1.15E-01	7.86E-02	1.26E-01	0
LIPH	ENSG00000163898	3	185224050	185270401	6.54E-01	1.41E-01	2.35E-01	7.35E-01	7.97E-01	1.26E-01	0
FGFBP3	ENSG00000174721	10	93666346	93669240	3.32E-02	2.54E-01	4.26E-02	8.55E-01	3.87E-01	1.26E-01	0
ABCC9	ENSG00000069431	12	21950335	22094336	6.89E-02	3.84E-01	9.57E-02	9.04E-01	2.75E-02	1.26E-01	0
HADH	ENSG00000138796	4	108910870	108956331	3.09E-01	6.08E-01	2.88E-01	7.47E-01	7.68E-01	1.26E-01	0
USP30	ENSG00000135093	12	109460894	109525831	2.76E-01	2.90E-01	1.56E-01	4.15E-01	2.54E-01	1.26E-01	0
FBXL20	ENSG00000108306	17	37415384	37558776	1.48E-02	3.76E-01	1.35E-02	7.19E-02	7.47E-03	1.27E-01	0
CHAMP1	ENSG00000198824	13	115079988	115092796	6.50E-01	5.75E-01	5.19E-01	4.17E-02	3.57E-02	1.27E-01	0
ERMP1	ENSG00000099219	9	5765076	5833117	4.35E-01	4.97E-01	1.86E-01	7.64E-01	7.46E-01	1.27E-01	0
DPY30	ENSG00000162961	2	32092878	32264881	1.27E-01	8.10E-02	7.11E-02	3.72E-01	4.79E-01	1.27E-01	0
RAB3D	ENSG00000105514	19	11432722	11456946	1.29E-01	2.31E-03	2.60E-01	2.73E-02	6.51E-02	1.27E-01	0
PANK2	ENSG00000125779	20	3869486	3907605	9.02E-01	4.21E-01	2.44E-01	7.58E-02	3.55E-01	1.27E-01	0
NCAPH	ENSG00000121152	2	97001525	97039583	5.27E-01	4.50E-02	4.54E-01	1.04E-01	8.92E-02	1.27E-01	0
AEBP2	ENSG00000139154	12	19556979	19873735	4.80E-01	6.16E-01	3.05E-01	2.33E-01	8.41E-01	1.27E-01	0
GNAI2	ENSG00000114353	3	50263724	50296787	6.04E-02	1.23E-01	3.67E-02	1.68E-01	6.70E-01	1.27E-01	0
EPO	ENSG00000130427	7	100318423	100321323	7.57E-01	9.97E-01	3.32E-01	4.70E-01	6.65E-01	1.27E-01	0
FNDC4	ENSG00000115226	2	27714750	27718112	3.20E-02	2.89E-01	3.24E-02	8.68E-02	6.30E-01	1.27E-01	0

Gene	Ensembl ID	CHR	START	STOP	VHD_CD Pleiotropy P	VHD_GORD Pleiotropy P	VHD_IBD Pleiotropy P	VHD_IBS Pleiotropy P	VHD_PUD Pleiotropy P	VHD_UC Pleiotropy P	d
TRIM23	ENSG00000113595	5	64885507	64921802	1.34E-01	4.00E-02	3.07E-01	6.69E-01	4.24E-01	1.27E-01	0
ONECUT1	ENSG00000169856	15	53049637	53083273	9.01E-01	1.94E-01	3.09E-01	9.24E-01	8.07E-02	1.27E-01	0
SNX13	ENSG00000071189	7	17830385	17980124	1.93E-01	2.33E-03	1.76E-01	3.81E-01	3.62E-01	1.27E-01	0
CELF3	ENSG00000159409	1	151674880	151689290	1.69E-01	9.44E-01	2.06E-01	9.81E-01	4.77E-01	1.28E-01	0
TMEM144	ENSG00000164124	4	159122756	159176563	3.97E-01	2.94E-01	2.17E-01	3.40E-01	4.16E-01	1.28E-01	0
ABCF3	ENSG00000161204	3	183903811	183911800	3.28E-03	3.03E-02	8.89E-03	2.64E-01	2.68E-03	1.28E-01	0
NTN5	ENSG00000142233	19	49164664	49176338	5.91E-04	5.79E-01	3.16E-03	8.38E-02	1.58E-04	1.28E-01	0
PAIP2	ENSG00000120727	5	138677276	138705406	2.18E-01	6.44E-01	5.59E-02	5.60E-01	2.27E-01	1.28E-01	0
TMEM11	ENSG00000178307	17	21100593	21117937	7.62E-02	1.15E-01	7.51E-02	7.13E-02	1.67E-01	1.28E-01	0
ZNF132	ENSG00000131849	19	58944181	58951589	2.28E-01	7.82E-01	1.02E-01	5.34E-02	2.40E-01	1.28E-01	0
SCD5	ENSG00000145284	4	83550692	83720010	5.13E-01	5.96E-01	3.49E-01	7.45E-01	5.87E-01	1.28E-01	0
RGL2	ENSG00000237441	6	33259431	33267101	2.25E-01	1.45E-02	1.54E-01	4.35E-01	3.89E-01	1.28E-01	0
AOC3	ENSG00000131471	17	41003201	41010147	2.01E-01	3.78E-01	1.62E-01	2.20E-01	2.43E-01	1.28E-01	0
SCNN1B	ENSG00000168447	16	23289552	23392620	8.24E-01	9.56E-01	2.81E-01	5.39E-01	7.21E-01	1.28E-01	0
MON2	ENSG00000061987	12	62860597	62991363	4.73E-01	9.30E-02	2.33E-01	5.74E-03	3.52E-01	1.28E-01	0
ASIC2	ENSG00000108684	17	31340105	32501983	2.99E-01	3.13E-01	9.33E-01	8.59E-01	2.49E-01	1.28E-01	0
PIGW	ENSG00000184886	17	34890847	34895159	7.52E-01	8.14E-01	2.57E-01	5.77E-02	4.49E-01	1.28E-01	0
TGFBR3	ENSG00000069702	1	92145902	92371892	2.80E-01	3.85E-01	1.00E-01	3.49E-01	2.89E-01	1.28E-01	0
PAIP2B	ENSG00000124374	2	71409869	71454213	1.59E-01	4.15E-01	1.23E-01	3.05E-01	6.37E-01	1.28E-01	0
LGALS16	ENSG00000249861	19	40146534	40151287	8.22E-01	7.73E-01	2.06E-01	2.91E-01	7.17E-01	1.28E-01	0
DDX31	ENSG00000125485	9	135468384	135545788	3.56E-01	3.99E-01	2.36E-01	1.82E-01	6.15E-02	1.28E-01	0
ASPM	ENSG00000066279	1	197053258	197115824	3.49E-01	9.48E-01	3.57E-01	2.97E-01	1.05E-01	1.28E-01	0
SLC22A8	ENSG00000149452	11	62756626	62783311	1.64E-01	7.26E-01	6.22E-02	8.56E-01	8.50E-01	1.28E-01	0
MAST2	ENSG00000086015	1	46252659	46501796	2.27E-01	3.26E-01	1.28E-01	4.18E-01	6.69E-01	1.28E-01	0
DPEP2	ENSG00000167261	16	68021297	68034489	1.01E-01	1.53E-01	8.42E-01	4.62E-02	1.36E-01	1.28E-01	0
XRN1	ENSG00000114127	3	142025449	142166904	6.81E-02	2.97E-02	6.40E-01	5.53E-02	3.01E-02	1.29E-01	0
SLC23A1	ENSG00000170482	5	138702885	138720242	5.62E-01	1.46E-02	1.15E-01	4.27E-01	7.47E-01	1.29E-01	0
SNAI3	ENSG00000185669	16	88744090	88752901	3.32E-01	3.29E-01	8.92E-02	1.46E-01	3.79E-01	1.29E-01	0
CAST	ENSG00000153113	5	95860971	96115299	2.66E-01	8.49E-01	2.27E-01	2.03E-01	7.55E-01	1.29E-01	0
CXCL14	ENSG00000145824	5	134906373	134914969	6.27E-01	9.99E-02	8.02E-01	1.45E-01	4.05E-01	1.29E-01	0
STX1B	ENSG00000099365	16	31000577	31021949	9.32E-02	3.29E-01	5.57E-02	1.85E-01	6.08E-01	1.29E-01	0
TFEC	ENSG00000105967	7	115575202	115799950	1.60E-01	2.70E-02	1.08E-01	4.33E-02	9.37E-02	1.29E-01	0
DALRD3	ENSG00000178149	3	49052921	49059726	1.59E-01	1.23E-02	6.06E-02	9.17E-02	3.03E-01	1.29E-01	0
NAIF1	ENSG00000171169	9	130823512	130830485	3.09E-01	6.67E-01	2.84E-01	1.73E-01	6.23E-01	1.29E-01	0
IRGQ	ENSG00000167378	19	44088521	44100287	9.74E-01	6.30E-01	1.85E-01	5.98E-02	2.41E-01	1.29E-01	0
LRFN4	ENSG00000173621	11	66624118	66627946	8.02E-01	1.14E-02	3.38E-01	7.03E-01	6.10E-01	1.29E-01	0
EEF1A2	ENSG00000101210	20	62119366	62130505	4.91E-03	1.22E-01	4.45E-02	1.31E-01	5.07E-01	1.29E-01	0
TMEM50A	ENSG00000183726	1	25664408	25688852	7.80E-01	5.97E-01	4.78E-01	5.80E-01	9.83E-01	1.30E-01	0
ALOX5AP	ENSG00000132965	13	31309645	31338556	2.03E-02	2.31E-01	3.44E-02	1.74E-01	2.98E-01	1.30E-01	0
NMB	ENSG00000197696	15	85198360	85201794	6.90E-03	3.04E-01	2.00E-02	1.52E-01	1.44E-02	1.30E-01	0
NR3C1	ENSG00000113580	5	142657496	142815077	8.17E-01	8.89E-01	3.11E-01	3.46E-01	2.31E-01	1.30E-01	0
MFAP2	ENSG00000117122	1	17300997	17307330	2.15E-01	3.83E-02	8.46E-01	2.54E-01	4.63E-01	1.30E-01	0
C1orf145	ENSG00000162913	1	228391207	228401365	3.44E-03	4.84E-02	6.17E-03	1.42E-02	6.97E-02	1.30E-01	0
GPR17	ENSG00000144230	2	128403439	128410213	1.24E-01	1.80E-01	7.64E-02	2.33E-01	6.14E-01	1.30E-01	0
ZNF584	ENSG00000171574	19	58912871	58929694	7.19E-02	7.75E-01	6.61E-02	1.50E-01	2.92E-01	1.30E-01	0
ADPRHL1	ENSG00000153531	13	114076260	114107839	7.13E-01	5.11E-01	1.13E-01	1.81E-01	6.18E-01	1.30E-01	0
ALKBH6	ENSG00000239382	19	36500022	36505141	2.49E-01	3.80E-01	7.95E-02	4.46E-01	2.62E-01	1.30E-01	0
NPPA	ENSG00000175206	1	11905766	11908402	2.64E-01	4.65E-01	6.46E-02	2.85E-01	1.21E-01	1.30E-01	0
MTG1	ENSG00000148824	10	135207598	135234811	6.21E-01	3.51E-01	4.34E-01	5.03E-02	2.32E-01	1.30E-01	0

Gene	Ensembl ID	CHR	START	STOP	VHD_CD Pleiotropy P	VHD_GORD Pleiotropy P	VHD_IBD Pleiotropy P	VHD_IBS Pleiotropy P	VHD_PUD Pleiotropy P	VHD_UC Pleiotropy P	d
CCDC102B	ENSG00000150636	18	66382446	66722426	3.06E-01	2.38E-01	2.22E-01	8.43E-02	4.22E-03	1.30E-01	0
CLIP3	ENSG00000105270	19	36505562	36524245	5.04E-01	4.33E-01	1.14E-01	2.52E-01	1.17E-01	1.31E-01	0
RFESD	ENSG00000175449	5	94982458	95020477	2.56E-01	2.72E-01	4.30E-01	1.92E-01	4.33E-02	1.31E-01	0
LRRC63	ENSG00000173988	13	46786083	46851501	1.29E-01	4.51E-01	6.50E-02	5.16E-01	2.37E-01	1.31E-01	0
MAP4K4	ENSG00000071054	2	102313312	102511149	1.38E-01	6.76E-01	7.76E-02	5.10E-01	2.85E-01	1.31E-01	0
OR5R1	ENSG00000174942	11	56184734	56185708	1.53E-01	2.79E-01	2.18E-01	2.26E-01	5.33E-01	1.31E-01	0
HSPA12A	ENSG00000165868	10	118430703	118502085	6.95E-01	1.84E-01	2.73E-01	5.53E-02	1.70E-01	1.31E-01	0
KLHL28	ENSG00000179454	14	45393522	45511525	2.87E-01	6.89E-01	2.53E-01	6.45E-01	1.45E-01	1.31E-01	0
SOS1	ENSG00000115904	2	39208537	39351486	4.38E-02	5.90E-01	3.80E-02	6.06E-03	3.67E-02	1.31E-01	0
COPS7A	ENSG00000111652	12	6832907	6841041	8.15E-01	2.09E-01	4.82E-01	3.19E-01	8.43E-01	1.31E-01	0
TMEM126B	ENSG00000171204	11	85339629	85347580	3.62E-01	2.04E-02	5.07E-01	5.96E-01	1.28E-01	1.31E-01	0
C6orf203	ENSG00000130349	6	107349407	107372546	8.24E-01	6.44E-01	4.25E-01	5.08E-01	9.53E-02	1.31E-01	0
PRR11	ENSG00000068489	17	57232860	57282066	1.67E-02	1.36E-01	4.19E-02	6.76E-02	9.83E-02	1.31E-01	0
KLHL18	ENSG00000114648	3	47324407	47388306	2.07E-01	4.99E-01	9.44E-02	8.14E-01	8.24E-01	1.31E-01	0
PGAP2	ENSG00000148985	11	3818954	3847601	1.90E-01	3.70E-01	1.40E-01	5.82E-01	4.17E-01	1.31E-01	0
POTEJ	ENSG00000222038	2	131369054	131415664	3.02E-01	4.44E-01	3.92E-01	8.49E-01	6.55E-01	1.31E-01	0
ZNF460	ENSG00000197714	19	57791419	57804937	6.19E-03	1.89E-02	1.90E-02	5.62E-01	1.01E-02	1.31E-01	0
PLEK2	ENSG00000100558	14	67853700	67878917	4.89E-01	5.80E-01	2.18E-01	5.03E-01	5.82E-01	1.31E-01	0
IER5L	ENSG00000188483	9	131937835	131940540	1.18E-02	4.86E-02	1.26E-01	3.57E-03	4.56E-01	1.32E-01	0
CARD6	ENSG00000132357	5	40841286	40860275	1.27E-03	7.62E-02	9.43E-03	1.13E-01	2.61E-02	1.32E-01	0
ZNF664	ENSG00000179195	12	124456392	124499986	2.06E-01	5.60E-02	7.17E-02	2.73E-02	2.97E-01	1.32E-01	0
VPS52	ENSG00000223501	6	33218049	33239824	3.89E-02	3.88E-01	5.29E-02	5.46E-01	8.19E-02	1.32E-01	0
OR13A1	ENSG00000256574	10	45798102	45811056	1.91E-01	2.58E-01	8.50E-02	1.60E-01	3.78E-01	1.32E-01	0
ICAM1	ENSG00000090339	19	10381511	10397291	5.99E-02	5.15E-01	4.87E-02	3.23E-01	8.63E-02	1.32E-01	0
CCDC158	ENSG00000163749	4	77234154	77343021	1.03E-02	1.40E-04	4.81E-01	1.28E-01	4.52E-04	1.32E-01	0
AMPD1	ENSG00000116748	1	115215719	115238239	1.70E-01	2.85E-01	7.99E-02	6.77E-01	2.19E-01	1.32E-01	0
FHOD1	ENSG00000135723	16	67263290	67281561	3.17E-01	4.67E-01	3.09E-01	3.37E-01	2.19E-01	1.32E-01	0
F2RL3	ENSG00000127533	19	16999671	17003417	1.10E-02	9.88E-03	9.06E-02	5.10E-02	3.37E-02	1.32E-01	0
FAM196B	ENSG00000204767	5	169291268	169407744	5.89E-01	8.48E-01	3.67E-01	8.53E-01	6.64E-01	1.32E-01	0
LATS1	ENSG00000131023	6	149979289	150039392	1.27E-01	4.34E-02	1.12E-01	3.82E-01	2.15E-01	1.32E-01	0
HIST1H4L	ENSG00000198558	6	27840926	27841289	1.32E-01	9.13E-03	4.43E-02	2.74E-01	2.89E-01	1.32E-01	0
DCAF4L2	ENSG00000176566	8	88882973	88886296	4.46E-01	2.33E-01	2.16E-01	7.26E-02	5.67E-01	1.32E-01	0
OR7E24	ENSG00000237521	19	9361606	9362820	6.21E-01	1.56E-01	4.08E-01	7.88E-01	1.83E-01	1.32E-01	0
KRTAP10-12	ENSG00000189169	21	46117087	46117959	1.96E-01	4.33E-01	1.24E-01	7.71E-01	3.97E-01	1.32E-01	0
ZNF391	ENSG00000124613	6	27342394	27371683	3.94E-01	2.30E-01	1.62E-01	2.48E-01	6.46E-01	1.32E-01	0
CYP3A7	ENSG00000160870	7	99302660	99332819	2.55E-02	3.99E-01	1.36E-01	1.91E-01	3.55E-01	1.32E-01	0
CASKIN2	ENSG00000177303	17	73496342	73511664	7.00E-01	3.89E-01	4.70E-01	8.60E-01	5.60E-01	1.32E-01	0
TMEM192	ENSG00000170088	4	165995574	166129701	2.15E-01	3.86E-01	8.15E-01	8.63E-01	5.35E-01	1.32E-01	0
RASSF5	ENSG00000136653	1	206680879	206762616	6.88E-01	9.73E-01	2.34E-01	4.49E-01	4.13E-01	1.33E-01	0
SIX4	ENSG00000100625	14	61176246	61191066	2.88E-01	3.50E-01	1.79E-01	2.00E-01	4.88E-01	1.33E-01	0
CDC42	ENSG00000070831	1	22379120	22419437	4.98E-01	3.37E-01	2.04E-01	6.61E-01	3.73E-01	1.33E-01	0
TMEM115	ENSG00000126062	3	50392180	50397041	8.50E-02	5.75E-02	4.53E-02	1.02E-01	4.90E-01	1.33E-01	0
KIF15	ENSG00000163808	3	44803209	44914868	1.42E-01	9.34E-01	8.36E-02	3.51E-01	4.57E-02	1.33E-01	0
HSPA6	ENSG00000173110	1	161494036	161496681	8.61E-01	6.64E-01	2.17E-01	6.52E-01	7.38E-01	1.33E-01	0
CASP1	ENSG00000137752	11	104896170	104972158	2.86E-01	6.19E-01	1.97E-01	8.59E-01	1.36E-01	1.33E-01	0
PRICKLE4	ENSG00000124593	6	41748087	41757879	7.77E-01	5.73E-01	2.31E-01	1.90E-01	4.36E-01	1.33E-01	0
GPR12	ENSG00000132975	13	27329341	27334922	6.51E-01	3.37E-02	1.62E-01	4.97E-02	3.16E-01	1.33E-01	0
CCDC103	ENSG00000167131	17	42976510	42982758	4.71E-01	4.70E-01	1.97E-01	1.76E-01	4.45E-01	1.33E-01	0
PTAR1	ENSG00000188647	9	72324438	72374875	4.04E-01	4.51E-01	4.68E-01	7.25E-01	9.54E-02	1.33E-01	0

Gene	Ensembl ID	CHR	START	STOP	VHD_CD Pleiotropy P	VHD_GORD Pleiotropy P	VHD_IBD Pleiotropy P	VHD_IBS Pleiotropy P	VHD_PUD Pleiotropy P	VHD_UC Pleiotropy P	d
PPWD1	ENSG00000113593	5	64859063	64883376	1.28E-01	3.58E-02	2.23E-01	6.71E-01	4.15E-01	1.33E-01	0
SUCNR1	ENSG00000198829	3	151591431	151602405	6.75E-01	9.06E-01	2.34E-01	2.91E-01	3.23E-01	1.33E-01	0
OR3A1	ENSG00000180090	17	3194929	3195876	2.13E-01	9.92E-01	1.08E-01	6.53E-01	2.93E-01	1.34E-01	0
CDYL2	ENSG00000166446	16	80631803	80838226	4.46E-03	2.13E-01	7.30E-03	7.85E-01	4.29E-01	1.34E-01	0
KAT5	ENSG00000172977	11	65479467	65487075	2.18E-01	1.67E-01	1.18E-01	8.70E-02	4.97E-01	1.34E-01	0
RALBP1	ENSG00000017797	18	9475007	9538114	2.98E-01	3.19E-01	2.37E-01	4.60E-01	1.02E-01	1.34E-01	0
ADAMTS8	ENSG00000134917	11	130274820	130298888	6.49E-01	3.50E-01	8.67E-01	7.96E-01	8.57E-01	1.34E-01	0
BACH2	ENSG00000112182	6	90636248	91006627	5.53E-02	4.59E-01	2.87E-02	5.15E-01	6.08E-01	1.34E-01	0
DNASE1	ENSG00000213918	16	3661729	3730144	4.95E-01	2.40E-01	2.28E-01	1.86E-01	5.50E-01	1.34E-01	0
ERBB2IP	ENSG00000112851	5	65222303	65378377	3.37E-01	5.42E-01	1.76E-01	2.73E-01	1.72E-01	1.34E-01	0
TSHZ3	ENSG00000121297	19	31765851	31840453	1.79E-01	2.35E-01	9.56E-02	3.68E-02	2.35E-01	1.34E-01	0
SLC30A10	ENSG00000196660	1	219858769	220131989	5.10E-01	4.41E-01	1.50E-01	7.46E-01	2.45E-01	1.34E-01	0
ERC2	ENSG00000187672	3	55542336	56502391	3.18E-01	5.08E-02	1.71E-01	4.96E-01	3.41E-01	1.34E-01	0
H1F0	ENSG00000189060	22	38201114	38203442	2.83E-01	1.83E-02	1.84E-01	3.72E-01	1.28E-01	1.34E-01	0
PTOV1	ENSG00000104960	19	50353992	50364001	8.54E-01	5.08E-01	4.88E-01	3.86E-01	6.95E-01	1.34E-01	0
CAP1	ENSG00000131236	1	40505905	40538321	1.18E-01	7.68E-01	1.31E-01	3.33E-02	1.12E-01	1.34E-01	0
MRPS28	ENSG00000147586	8	80830952	80942524	1.66E-01	4.48E-01	1.76E-01	1.39E-01	4.84E-01	1.34E-01	0
JMJD6	ENSG00000070495	17	74708919	74722866	7.73E-01	8.03E-01	4.81E-01	1.87E-01	3.46E-01	1.34E-01	0
IRF5	ENSG00000128604	7	128577666	128590089	7.82E-01	9.41E-01	3.70E-01	8.06E-01	9.97E-01	1.34E-01	0
SLC30A1	ENSG00000170385	1	211744910	211752084	6.05E-01	5.16E-02	3.36E-01	1.40E-01	1.85E-01	1.35E-01	0
PPL	ENSG00000118898	16	4932508	5010742	2.07E-01	5.38E-01	7.70E-02	5.33E-01	2.61E-01	1.35E-01	0
ST7	ENSG00000004866	7	116593292	116870157	5.96E-01	6.96E-01	4.28E-01	5.15E-01	3.87E-01	1.35E-01	0
ERAP2	ENSG00000164308	5	96211643	96255420	4.99E-03	2.72E-01	1.01E-02	7.69E-01	5.47E-01	1.35E-01	0
GDAP1L1	ENSG00000124194	20	42875887	42909013	1.16E-01	1.97E-02	8.31E-02	2.08E-01	3.02E-01	1.35E-01	0
KANSL1	ENSG00000120071	17	44107282	44302733	5.44E-02	1.66E-02	6.00E-02	3.92E-01	5.69E-02	1.35E-01	0
EHHADH	ENSG00000113790	3	184908412	184999778	2.38E-01	2.04E-01	1.42E-01	3.41E-01	1.75E-01	1.35E-01	0
ATG10	ENSG00000152348	5	81267844	81572676	2.12E-01	5.00E-03	1.81E-01	8.50E-01	4.51E-01	1.35E-01	0
TCFL5	ENSG00000101190	20	61472467	61493115	4.60E-01	2.02E-01	1.50E-01	5.47E-01	4.81E-01	1.35E-01	0
ATP5SL	ENSG00000105341	19	41937223	41946622	3.52E-01	6.09E-01	1.70E-01	7.82E-01	4.66E-01	1.35E-01	0
FRS3	ENSG00000137218	6	41737914	41754280	7.71E-01	5.74E-01	2.72E-01	3.67E-01	5.67E-01	1.35E-01	0
TH	ENSG00000180176	11	2185159	2193107	1.00E-01	5.42E-01	1.00E-01	7.38E-01	6.50E-02	1.35E-01	0
WDR89	ENSG00000140006	14	64063757	64108579	6.13E-01	3.86E-01	5.22E-01	1.38E-01	3.37E-01	1.35E-01	0
EIF4H	ENSG00000106682	7	73588575	73611431	4.33E-01	1.53E-02	1.97E-01	6.37E-02	1.66E-01	1.35E-01	0
IL1RN	ENSG00000136689	2	113864791	113891593	6.60E-01	5.24E-01	3.95E-01	4.99E-01	1.77E-01	1.35E-01	0
RPA2	ENSG00000117748	1	28218035	28241257	3.15E-02	2.20E-01	5.05E-02	1.16E-01	3.32E-01	1.35E-01	0
MFSD12	ENSG00000161091	19	3538259	3574288	4.49E-01	6.72E-01	2.61E-01	6.78E-01	8.82E-01	1.35E-01	0
MICU1	ENSG00000107745	10	74127098	74385899	4.55E-02	5.27E-01	4.11E-02	5.46E-02	2.57E-01	1.35E-01	0
CYSTM1	ENSG00000120306	5	139554227	139661637	3.14E-01	2.39E-01	1.57E-01	5.78E-02	4.03E-01	1.35E-01	0
STMN2	ENSG00000104435	8	80523049	80578410	1.03E-01	8.55E-01	1.55E-01	6.06E-01	8.23E-01	1.35E-01	0
FRAT1	ENSG00000165879	10	99079022	99081672	3.95E-01	2.20E-02	3.22E-01	4.22E-01	1.39E-01	1.36E-01	0
PRPF4	ENSG00000136875	9	116037623	116055185	1.44E-01	4.00E-01	1.52E-01	4.54E-01	1.99E-01	1.36E-01	0
MYOF	ENSG00000138119	10	95066186	95242074	7.24E-01	5.63E-01	4.44E-01	2.12E-01	4.32E-01	1.36E-01	0
SLC30A6	ENSG00000152683	2	32390933	32449448	1.72E-01	5.53E-01	5.71E-02	1.13E-01	5.49E-01	1.36E-01	0
PSTPIP1	ENSG00000140368	15	77285700	77329673	1.11E-02	4.79E-01	3.35E-02	8.63E-02	1.39E-01	1.36E-01	0
PPP1CB	ENSG00000213639	2	28974506	29025806	8.81E-02	4.31E-02	3.59E-02	1.29E-02	2.34E-01	1.36E-01	0
CEP89	ENSG00000121289	19	33369902	33462897	5.17E-01	1.83E-01	3.27E-01	1.51E-01	7.66E-01	1.36E-01	0
FCAR	ENSG00000186431	19	55385704	55401838	9.08E-01	5.22E-01	4.28E-01	7.84E-01	3.06E-01	1.36E-01	0
C9orf85	ENSG00000155621	9	74526426	74600970	6.66E-01	4.10E-01	1.40E-01	4.56E-01	3.53E-01	1.36E-01	0
MOG	ENSG00000204655	6	29624758	29640149	4.87E-02	6.04E-01	8.11E-02	2.85E-01	1.69E-01	1.36E-01	0

Gene	Ensembl ID	CHR	START	STOP	VHD_CD Pleiotropy P	VHD_GORD Pleiotropy P	VHD_IBD Pleiotropy P	VHD_IBS Pleiotropy P	VHD_PUD Pleiotropy P	VHD_UC Pleiotropy P	d
EN1	ENSG00000163064	2	119599747	119605254	2.26E-01	1.47E-01	3.58E-01	6.65E-01	1.49E-01	1.36E-01	0
NFIC	ENSG00000141905	19	3359561	3469215	4.24E-01	3.06E-01	2.62E-01	7.90E-02	3.34E-01	1.36E-01	0
ZYG11B	ENSG00000162378	1	53192126	53293014	4.82E-01	5.15E-01	3.30E-01	7.59E-01	3.28E-01	1.36E-01	0
MLLT1	ENSG00000130382	19	6212966	6279959	1.31E-01	2.60E-01	8.91E-02	3.71E-01	4.12E-02	1.36E-01	0
AFP	ENSG00000081051	4	74296855	74321891	4.67E-01	5.50E-01	1.79E-01	5.70E-02	8.55E-02	1.36E-01	0
EFNA4	ENSG00000243364	1	155036207	155042029	2.43E-02	7.66E-01	2.91E-02	4.74E-01	4.09E-01	1.36E-01	0
TCEA2	ENSG00000171703	20	62681189	62703700	7.13E-01	4.41E-01	3.04E-01	8.60E-01	6.39E-01	1.36E-01	0
ATP6V1D	ENSG00000100554	14	67761088	67826982	4.76E-02	1.19E-01	2.45E-01	6.40E-01	2.83E-01	1.37E-01	0
IRGM	ENSG00000237693	5	150226085	150280298	1.64E-01	6.84E-01	4.79E-01	7.01E-01	4.02E-01	1.37E-01	0
IPO13	ENSG00000117408	1	44412611	44433694	8.15E-01	1.80E-01	5.37E-01	8.79E-02	1.52E-01	1.37E-01	0
FADS3	ENSG00000221968	11	61640991	61659523	3.11E-03	5.67E-02	2.94E-02	5.03E-02	5.75E-02	1.37E-01	0
ASPHD1	ENSG00000174939	16	29911696	29931185	1.13E-01	3.36E-01	4.54E-01	2.87E-01	3.98E-01	1.37E-01	0
NOMO1	ENSG00000103512	16	14927538	14990017	1.27E-01	1.12E-01	7.20E-02	5.19E-02	1.38E-01	1.37E-01	0
TTC23	ENSG00000103852	15	99676528	99791428	2.64E-01	7.58E-01	8.39E-02	4.37E-01	2.10E-01	1.37E-01	0
KIAA0907	ENSG00000132680	1	155882834	155904191	6.67E-02	4.22E-02	3.12E-01	1.06E-02	8.15E-02	1.37E-01	0
MLLT4	ENSG00000130396	6	168227602	168372703	4.54E-01	1.13E-01	3.20E-01	2.87E-01	2.50E-01	1.37E-01	0
RNF219	ENSG00000152193	13	79188426	79233314	2.00E-01	3.79E-01	1.69E-01	1.28E-01	5.39E-01	1.37E-01	0
RAD52	ENSG00000002016	12	1021243	1099219	3.36E-02	2.63E-02	1.14E-01	4.52E-02	5.44E-01	1.37E-01	0
ZNF264	ENSG00000083844	19	57702868	57724724	2.33E-01	1.06E-01	1.99E-01	5.18E-01	2.52E-01	1.37E-01	0
CDC26	ENSG00000176386	9	116018115	116037869	2.78E-01	5.60E-01	1.35E-01	3.78E-01	2.23E-01	1.37E-01	0
C7orf57	ENSG00000164746	7	48075108	48100901	3.95E-01	7.23E-01	9.69E-02	1.55E-01	4.13E-01	1.37E-01	0
STK17A	ENSG00000164543	7	43622357	43666385	4.15E-01	1.49E-01	1.45E-01	1.21E-02	1.17E-01	1.38E-01	0
LRIT1	ENSG00000148602	10	85991349	86001217	2.08E-01	3.53E-01	7.11E-01	3.73E-01	1.50E-02	1.38E-01	0
SPSB1	ENSG00000171621	1	9352939	9429591	1.03E-01	8.83E-01	5.17E-02	7.62E-01	4.84E-01	1.38E-01	0
BET1	ENSG00000105829	7	93592074	93633694	3.20E-01	9.41E-01	2.35E-01	5.90E-01	9.27E-01	1.38E-01	0
HEG1	ENSG00000173706	3	124684554	124774802	1.23E-01	3.37E-01	6.61E-02	5.79E-02	4.58E-02	1.38E-01	0
KDR	ENSG00000128052	4	55944644	55991756	4.52E-01	8.11E-01	1.90E-01	3.60E-02	9.28E-01	1.38E-01	0
CETN3	ENSG00000153140	5	89688078	89705603	8.30E-01	2.15E-01	5.44E-01	4.34E-01	1.16E-01	1.38E-01	0
TFB1M	ENSG00000029639	6	155578643	155635627	3.55E-01	2.14E-01	2.16E-01	7.36E-01	6.00E-01	1.38E-01	0
DNAJC15	ENSG00000120675	13	43597339	43683045	3.35E-01	4.49E-01	1.89E-01	4.03E-01	8.89E-02	1.38E-01	0
ADAMTS5	ENSG00000154736	21	28290231	28338832	5.40E-01	5.71E-01	3.33E-01	4.19E-01	3.34E-01	1.38E-01	0
UPF2	ENSG00000151461	10	11962021	12085169	3.44E-01	5.84E-01	1.47E-01	2.92E-01	3.89E-01	1.38E-01	0
RNLS	ENSG00000184719	10	90033621	90344287	2.51E-01	8.40E-01	8.57E-01	3.51E-01	4.37E-01	1.38E-01	0
SYT11	ENSG00000132718	1	155829300	155854990	5.00E-02	2.63E-01	5.82E-02	2.71E-02	1.48E-01	1.38E-01	0
ELAVL4	ENSG00000162374	1	50513686	50669458	1.68E-01	4.71E-01	3.12E-02	7.64E-01	1.76E-01	1.38E-01	0
ITPA	ENSG00000125877	20	3189514	3204516	4.85E-01	3.10E-01	7.01E-01	7.69E-01	4.40E-01	1.38E-01	0
SLC27A4	ENSG00000167114	9	131102925	131123749	1.62E-01	5.19E-02	2.16E-01	4.29E-02	8.79E-02	1.38E-01	0
CDH15	ENSG00000129910	16	89238175	89261900	5.86E-01	1.33E-01	2.07E-01	1.52E-01	2.06E-01	1.38E-01	0
PPA2	ENSG00000138777	4	106290234	106395238	2.53E-01	4.04E-01	2.92E-01	1.13E-01	8.12E-01	1.38E-01	0
KLF1	ENSG00000105610	19	12995237	12997995	7.62E-02	4.89E-01	6.74E-02	2.97E-01	2.62E-01	1.39E-01	0
RLN3	ENSG00000171136	19	14138960	14141854	5.32E-01	4.80E-01	2.09E-01	2.69E-01	2.30E-01	1.39E-01	0
ZRANB1	ENSG00000019995	10	126630692	126676758	1.25E-01	2.63E-04	4.80E-01	1.42E-01	8.01E-04	1.39E-01	0
C15orf27	ENSG00000169758	15	76352178	76521462	1.47E-01	8.31E-01	7.94E-02	6.65E-01	6.73E-01	1.39E-01	0
OR5H6	ENSG00000230301	3	97983088	97984200	7.06E-01	9.03E-01	4.07E-01	2.01E-01	8.25E-02	1.39E-01	0
RBPJ	ENSG00000168214	4	26165077	26436541	4.13E-01	6.07E-01	5.04E-01	5.02E-01	3.18E-01	1.39E-01	0
ZCCHC8	ENSG00000033030	12	122957417	122985518	3.54E-02	5.60E-02	4.91E-02	3.70E-02	5.76E-02	1.39E-01	0
GLYR1	ENSG00000140632	16	4853204	4897343	2.52E-02	4.35E-01	5.71E-02	5.69E-01	1.53E-01	1.39E-01	0
HTR4	ENSG00000164270	5	147830595	148056798	1.64E-01	8.61E-01	8.17E-02	9.18E-01	6.51E-01	1.39E-01	0
ENAH	ENSG00000154380	1	225674537	225840844	1.16E-01	1.82E-01	4.11E-01	2.44E-01	5.26E-02	1.39E-01	0

Gene	Ensembl ID	CHR	START	STOP	VHD_CD Pleiotropy P	VHD_GORD Pleiotropy P	VHD_IBD Pleiotropy P	VHD_IBS Pleiotropy P	VHD_PUD Pleiotropy P	VHD_UC Pleiotropy P	d
ZNF324B	ENSG00000249471	19	58962971	58969200	1.59E-01	7.02E-01	1.02E-01	7.42E-02	1.38E-01	1.39E-01	0
PROCA1	ENSG00000167525	17	27030215	27038872	4.40E-01	3.14E-01	1.22E-01	2.60E-01	4.42E-01	1.39E-01	0
DDHD2	ENSG00000085788	8	38082736	38133076	1.38E-01	6.16E-01	8.06E-02	6.63E-02	4.21E-01	1.39E-01	0
KIF23	ENSG00000137807	15	69706585	69740764	1.16E-01	2.58E-01	1.60E-01	6.65E-01	2.97E-01	1.39E-01	0
MLH3	ENSG00000119684	14	75480467	75518235	6.14E-01	5.49E-01	4.21E-01	8.84E-01	2.94E-01	1.39E-01	0
ST6GAL2	ENSG00000144057	2	107418056	107503564	8.35E-01	3.52E-01	2.67E-01	3.28E-02	8.59E-01	1.39E-01	0
ANKRD54	ENSG00000100124	22	38226862	38245334	6.85E-01	6.39E-01	4.25E-01	1.61E-01	3.48E-01	1.40E-01	0
TRIM5	ENSG00000132256	11	5684425	5959849	9.20E-02	3.34E-02	6.17E-02	3.80E-01	1.18E-01	1.40E-01	0
RASGEF1C	ENSG00000146090	5	179527795	179636153	5.36E-01	8.07E-01	1.72E-01	5.25E-01	9.15E-01	1.40E-01	0
DDA1	ENSG00000130311	19	17420327	17434110	6.45E-01	3.29E-01	1.67E-01	5.01E-02	1.45E-01	1.40E-01	0
LENG8	ENSG00000167615	19	54960065	54973217	9.18E-01	1.89E-01	5.77E-01	2.54E-02	5.90E-02	1.40E-01	0
FRG1B	ENSG00000149531	20	29611857	29634010	1.79E-01	5.71E-03	1.67E-01	1.93E-01	5.20E-02	1.40E-01	0
THAP9	ENSG00000168152	4	83821837	83841122	1.21E-01	2.33E-01	9.15E-02	3.51E-01	3.93E-01	1.40E-01	0
CDC25A	ENSG00000164045	3	48198636	48229892	1.20E-01	1.65E-01	2.17E-01	7.79E-01	1.33E-01	1.40E-01	0
SDHC	ENSG00000143252	1	161284047	161332984	3.17E-02	6.29E-01	3.27E-02	6.77E-01	6.22E-02	1.40E-01	0
OTUB1	ENSG00000167770	11	63753325	63769283	7.27E-01	6.84E-02	3.09E-01	4.91E-01	8.30E-01	1.40E-01	0
CATSPER1	ENSG00000175294	11	65784223	65793988	1.49E-01	2.58E-01	8.23E-02	9.44E-02	8.95E-02	1.40E-01	0
NPC1	ENSG00000141458	18	21086148	21166862	1.25E-02	2.17E-02	1.24E-02	6.57E-03	9.45E-02	1.40E-01	0
OAZ3	ENSG00000143450	1	151735445	151743808	1.09E-01	6.99E-01	6.54E-02	7.26E-01	4.42E-01	1.40E-01	0
YWHAQ	ENSG00000134308	2	9724101	9771143	1.96E-01	8.37E-01	9.07E-02	5.61E-01	4.17E-01	1.40E-01	0
LRIG3	ENSG00000139263	12	59265931	59314303	7.57E-02	8.31E-02	3.07E-01	7.01E-01	2.92E-02	1.40E-01	0
ZNF140	ENSG00000196387	12	133656424	133684130	6.51E-02	1.99E-02	9.21E-02	2.00E-01	6.14E-02	1.40E-01	0
ITLN1	ENSG00000179914	1	160846329	160854960	1.14E-01	3.41E-01	1.01E-01	8.50E-01	2.42E-01	1.40E-01	0
ZNF446	ENSG00000083838	19	58985384	58992597	6.60E-02	8.20E-01	7.84E-02	9.59E-02	1.70E-01	1.40E-01	0
RECQL	ENSG00000004700	12	21621845	21654603	2.90E-01	1.07E-01	2.09E-01	3.15E-01	2.95E-01	1.40E-01	0
FGL1	ENSG00000104760	8	17721889	17767874	1.27E-02	6.83E-01	3.06E-02	2.11E-01	1.99E-02	1.41E-01	0
SOCS3	ENSG00000184557	17	76352864	76356158	2.65E-01	4.70E-01	1.27E-01	5.16E-01	1.96E-01	1.41E-01	0
CXCL9	ENSG00000138755	4	76922428	76928641	4.20E-02	2.11E-01	4.71E-02	5.11E-01	1.49E-01	1.41E-01	0
C3orf33	ENSG00000174928	3	155480401	155524140	5.80E-01	4.38E-01	3.60E-01	3.04E-01	7.59E-02	1.41E-01	0
BTNL2	ENSG00000204290	6	32361740	32374905	7.61E-02	1.74E-01	1.87E-03	1.38E-01	7.92E-01	1.41E-01	0
SCARB1	ENSG00000073060	12	125261402	125367214	3.42E-01	2.61E-01	1.62E-01	4.40E-02	1.62E-01	1.41E-01	0
MCM6	ENSG00000076003	2	136597196	136633996	2.29E-02	3.49E-01	3.13E-02	1.03E-02	8.70E-01	1.41E-01	0
FUCA2	ENSG00000001036	6	143815948	143832827	1.67E-01	2.14E-01	7.56E-02	5.06E-01	9.45E-02	1.41E-01	0
HPSE2	ENSG00000172987	10	100218875	100995619	8.08E-02	2.20E-01	5.80E-02	1.57E-01	1.19E-01	1.41E-01	0
CXCR1	ENSG00000163464	2	219027568	219031718	1.25E-01	4.94E-01	9.07E-02	3.26E-01	4.53E-01	1.41E-01	0
UBXN7	ENSG00000163960	3	196074533	196159345	2.44E-01	5.06E-01	9.06E-02	5.04E-01	7.39E-01	1.41E-01	0
APBB1	ENSG00000166313	11	6416355	6440644	8.91E-02	1.45E-01	4.43E-02	7.02E-02	6.65E-02	1.41E-01	0
ADARB1	ENSG00000197381	21	46493768	46646478	4.19E-01	2.70E-01	1.10E-01	3.71E-01	5.73E-01	1.41E-01	0
KCNA2	ENSG00000177301	1	111136202	111174096	2.54E-02	2.24E-01	2.99E-02	1.94E-03	2.53E-01	1.41E-01	0
ZSWIM5	ENSG00000162415	1	45482071	45771881	2.89E-02	2.46E-01	3.87E-02	6.28E-01	1.23E-01	1.41E-01	0
PSG8	ENSG00000124467	19	43256838	43359843	9.37E-01	6.14E-01	6.54E-01	3.82E-01	9.19E-01	1.41E-01	0
HMX1	ENSG00000215612	4	8847802	8873543	6.91E-01	2.38E-01	3.34E-01	1.41E-01	5.60E-01	1.41E-01	0
PLAU	ENSG00000122861	10	75668935	75677255	2.36E-01	6.03E-01	1.25E-01	4.88E-01	8.99E-01	1.41E-01	0
LYRM7	ENSG00000186687	5	130506503	130541119	2.94E-02	9.64E-02	2.56E-02	4.19E-01	6.57E-01	1.41E-01	0
ACADS	ENSG00000122971	12	121163538	121177811	2.84E-01	8.28E-02	4.51E-01	1.49E-01	4.30E-01	1.41E-01	0
ACLY	ENSG00000131473	17	40023161	40086795	1.11E-01	1.29E-01	5.99E-02	8.08E-01	7.19E-01	1.41E-01	0
VHL	ENSG00000134086	3	10182692	10193904	8.34E-01	9.06E-01	2.19E-01	1.32E-01	6.09E-01	1.41E-01	0
SPNS1	ENSG00000169682	16	28985542	28995869	8.79E-02	6.99E-01	9.99E-02	4.60E-01	3.73E-01	1.41E-01	0
ZNF439	ENSG00000171291	19	11959541	11994565	3.09E-01	8.17E-01	1.32E-01	9.28E-01	5.70E-01	1.42E-01	0

Gene	Ensembl ID	CHR	START	STOP	VHD_CD Pleiotropy P	VHD_GORD Pleiotropy P	VHD_IBD Pleiotropy P	VHD_IBS Pleiotropy P	VHD_PUD Pleiotropy P	VHD_UC Pleiotropy P	d
FER	ENSG00000151422	5	108083523	108532542	1.81E-01	3.77E-01	1.64E-01	5.23E-01	1.54E-01	1.42E-01	0
ZNF165	ENSG00000197279	6	28048753	28057341	4.70E-01	3.06E-01	2.60E-01	6.79E-01	7.52E-01	1.42E-01	0
ATP10D	ENSG00000145246	4	47487305	47595503	1.41E-01	3.98E-02	6.91E-02	1.66E-01	2.01E-01	1.42E-01	0
PDE1A	ENSG00000115252	2	183004763	183387919	2.56E-01	2.11E-01	1.53E-01	4.12E-01	8.93E-01	1.42E-01	0
NAT10	ENSG00000135372	11	34127149	34169217	2.66E-01	2.74E-01	1.33E-01	7.88E-01	8.14E-01	1.42E-01	0
OR2T4	ENSG00000196944	1	248524883	248525929	8.96E-01	7.04E-02	1.14E-01	1.37E-01	1.16E-01	1.42E-01	0
COPB2	ENSG00000184432	3	139074442	139108574	5.22E-02	8.82E-01	5.49E-02	9.27E-02	1.51E-01	1.42E-01	0
NPC1L1	ENSG00000015520	7	44552134	44580914	3.02E-01	9.43E-02	1.97E-01	7.55E-02	3.16E-01	1.42E-01	0
VAT1	ENSG00000108828	17	41166622	41177140	4.87E-01	6.89E-01	2.27E-01	1.82E-01	2.06E-01	1.42E-01	0
ACTL7B	ENSG00000148156	9	111616871	111619239	4.17E-01	3.79E-01	1.90E-01	5.63E-01	7.19E-01	1.42E-01	0
GATAD2A	ENSG00000167491	19	19496635	19619740	1.51E-01	5.72E-02	9.14E-02	3.59E-01	7.45E-02	1.42E-01	0
LRRC46	ENSG00000141294	17	45908993	45915079	3.69E-01	8.01E-01	2.63E-01	4.03E-01	4.90E-01	1.42E-01	0
UBE2QL1	ENSG00000215218	5	6448736	6495022	1.56E-01	2.77E-01	7.13E-02	7.91E-01	8.89E-01	1.42E-01	0
MED1	ENSG00000125686	17	37560538	37607539	1.94E-02	2.63E-01	1.83E-02	5.95E-02	5.31E-03	1.42E-01	0
SDCBP2	ENSG00000125775	20	1290619	1309883	5.38E-02	9.23E-01	2.81E-01	7.24E-01	2.99E-01	1.42E-01	0
RCL1	ENSG00000120158	9	4792869	4885917	2.09E-01	5.62E-01	1.30E-01	3.45E-01	5.24E-01	1.43E-01	0
TMEM194B	ENSG00000189362	2	191369068	191399448	2.72E-01	9.89E-02	6.30E-01	1.16E-01	6.82E-02	1.43E-01	0
BCL2L10	ENSG00000137875	15	52401460	52404972	6.57E-02	8.28E-01	4.92E-02	2.61E-01	3.76E-01	1.43E-01	0
RNASE6	ENSG00000169413	14	21249210	21250626	2.12E-01	3.12E-01	9.34E-02	5.01E-01	2.01E-01	1.43E-01	0
OR2T6	ENSG00000198104	1	248550910	248551836	6.42E-01	6.74E-02	1.20E-01	5.15E-02	1.48E-01	1.43E-01	0
ADHFE1	ENSG00000147576	8	67342420	67383836	1.07E-01	8.33E-01	5.45E-01	2.08E-01	4.42E-01	1.43E-01	0
UBE2W	ENSG00000104343	8	74692332	74791145	1.33E-01	9.35E-04	6.19E-01	6.78E-01	2.21E-01	1.43E-01	0
AGAP3	ENSG00000133612	7	150782918	150841523	2.26E-01	3.64E-02	9.68E-02	1.12E-01	1.26E-01	1.43E-01	0
RASSF6	ENSG00000169435	4	74437267	74486348	5.42E-01	6.46E-01	2.07E-01	3.92E-01	2.39E-01	1.43E-01	0
C8orf74	ENSG00000171060	8	10530147	10558103	3.59E-01	5.14E-02	1.98E-01	3.41E-01	1.56E-01	1.44E-01	0
SPINT2	ENSG00000167642	19	38734675	38783254	4.02E-02	6.64E-01	1.89E-02	8.33E-01	5.81E-01	1.44E-01	0
C11orf85	ENSG00000168070	11	64704989	64739557	2.55E-02	8.09E-01	2.56E-02	4.59E-01	6.60E-01	1.44E-01	0
SPTBN2	ENSG00000173898	11	66452719	66496697	5.59E-01	1.64E-01	2.33E-01	7.23E-01	6.54E-01	1.44E-01	0
AFAP1L1	ENSG00000157510	5	148651434	148721365	4.07E-01	5.68E-01	2.44E-01	2.71E-02	1.94E-01	1.44E-01	0
METTL14	ENSG00000145388	4	119606523	119636588	4.16E-02	3.39E-02	2.68E-02	3.23E-01	1.65E-01	1.44E-01	0
IL12RB2	ENSG00000081985	1	67773047	67862583	1.23E-01	7.22E-01	5.92E-02	9.27E-01	7.20E-01	1.44E-01	0
ZSCAN22	ENSG00000182318	19	58838385	58853698	1.46E-01	3.30E-01	9.92E-02	1.04E-01	5.65E-01	1.44E-01	0
ST7-OT4	ENSG00000214188	7	116593953	116738860	4.02E-01	8.11E-01	3.47E-01	2.65E-01	3.37E-01	1.44E-01	0
FBXO16	ENSG00000214050	8	28205804	28347835	4.67E-01	4.80E-01	2.24E-01	5.06E-01	5.79E-01	1.44E-01	0
ASNA1	ENSG00000198356	19	12847953	12859137	2.88E-02	2.34E-01	1.33E-01	1.43E-02	1.07E-01	1.44E-01	0
PDCD4	ENSG00000150593	10	112631565	112659764	9.97E-01	6.41E-01	6.09E-01	8.57E-01	8.35E-01	1.44E-01	0
MTL5	ENSG00000132749	11	68474908	68519032	4.81E-02	2.41E-01	8.44E-02	8.67E-02	1.79E-01	1.44E-01	0
TMEM14A	ENSG00000096092	6	52535907	52551386	1.79E-01	6.57E-01	1.87E-01	6.60E-01	2.51E-01	1.44E-01	0
AEBP1	ENSG00000106624	7	44143960	44154161	4.61E-02	6.17E-02	4.41E-02	3.04E-02	3.68E-01	1.44E-01	0
COMMD7	ENSG00000149600	20	31290493	31331803	3.13E-01	2.47E-01	1.24E-01	3.94E-01	9.90E-01	1.44E-01	0
DCDC1	ENSG00000170959	11	30851916	31391357	2.04E-01	3.77E-03	1.11E-01	2.01E-02	4.61E-04	1.44E-01	0
KIAA1671	ENSG00000197077	22	25348697	25593415	3.34E-01	4.02E-01	2.08E-01	8.73E-01	8.46E-01	1.45E-01	0
SMARCB1	ENSG00000099956	22	24129150	24176703	2.60E-01	2.33E-01	9.51E-02	6.15E-01	2.87E-01	1.45E-01	0
CDK12	ENSG00000167258	17	37617764	37721160	2.00E-02	3.03E-01	1.94E-02	6.94E-02	1.05E-02	1.45E-01	0
PTMS	ENSG00000159335	12	6874682	6880116	4.96E-01	7.97E-02	3.89E-01	2.81E-02	1.37E-01	1.45E-01	0
CRK	ENSG00000167193	17	1323983	1366456	1.45E-01	4.06E-01	5.46E-02	2.21E-01	7.79E-01	1.45E-01	0
USO1	ENSG00000138768	4	76649777	76735382	4.34E-01	5.60E-01	1.64E-01	4.31E-01	2.18E-01	1.45E-01	0
PRUNE	ENSG00000143363	1	150980896	151008189	5.22E-03	7.35E-02	2.23E-02	2.15E-02	7.96E-02	1.45E-01	0
NPHS2	ENSG00000116218	1	179519674	179545087	4.51E-01	9.11E-01	4.78E-01	1.44E-01	3.33E-02	1.45E-01	0

Gene	Ensembl ID	CHR	START	STOP	VHD_CD Pleiotropy P	VHD_GORD Pleiotropy P	VHD_IBD Pleiotropy P	VHD_IBS Pleiotropy P	VHD_PUD Pleiotropy P	VHD_UC Pleiotropy P	d
DAND5	ENSG00000179284	19	13075973	13085567	2.45E-01	3.98E-01	1.92E-01	1.57E-01	3.61E-01	1.45E-01	0
RGS21	ENSG00000253148	1	192286122	192336415	2.39E-01	3.91E-01	1.83E-01	1.36E-01	1.09E-01	1.45E-01	0
UBR1	ENSG00000159459	15	43235095	43398311	4.72E-01	6.49E-01	1.34E-01	1.47E-01	7.60E-02	1.45E-01	0
DUOXA2	ENSG00000140274	15	45406519	45410619	7.87E-02	5.69E-01	5.40E-01	5.75E-01	3.87E-01	1.45E-01	0
TRIML2	ENSG00000179046	4	189012427	189030757	4.03E-01	7.42E-01	2.50E-01	3.29E-01	2.88E-01	1.45E-01	0
KAZN	ENSG00000189337	1	14925200	15444539	7.62E-02	2.66E-01	7.35E-02	2.24E-01	4.84E-01	1.45E-01	0
FN3K	ENSG00000167363	17	80693451	80709073	5.45E-01	4.72E-02	4.45E-01	6.94E-01	5.91E-01	1.45E-01	0
FARP2	ENSG00000006607	2	242295658	242434256	5.95E-01	9.17E-01	3.55E-01	1.02E-01	5.96E-01	1.45E-01	0
SLC16A4	ENSG00000168679	1	110905470	110933704	8.27E-01	3.34E-01	4.15E-01	3.33E-01	1.68E-01	1.45E-01	0
NDC80	ENSG00000080986	18	2571510	2616634	1.23E-01	1.10E-02	8.79E-02	1.49E-01	2.13E-01	1.46E-01	0
AURKAIP1	ENSG00000175756	1	1309110	1310875	2.35E-01	5.55E-01	8.48E-02	8.26E-01	6.57E-01	1.46E-01	0
CRYBB1	ENSG00000100122	22	26995242	27014052	1.37E-01	5.89E-01	7.33E-02	8.57E-01	4.67E-01	1.46E-01	0
NUP43	ENSG00000120253	6	150045451	150070801	3.64E-01	4.58E-02	4.31E-01	7.50E-01	2.41E-01	1.46E-01	0
TOM1L2	ENSG00000175662	17	17746828	17875736	6.19E-01	1.04E-01	4.21E-01	4.63E-03	9.54E-02	1.46E-01	0
MNAT1	ENSG00000020426	14	61201460	61436671	6.25E-01	6.49E-01	1.92E-01	3.23E-01	5.88E-01	1.46E-01	0
SF3B4	ENSG00000143368	1	149895209	149900236	2.37E-01	2.96E-01	3.96E-01	7.06E-02	1.05E-01	1.46E-01	0
FAM149B1	ENSG00000138286	10	74927924	75004262	3.85E-01	4.36E-01	1.42E-01	4.69E-02	2.79E-02	1.46E-01	0
ZFPM1	ENSG00000179588	16	88519725	88603424	6.54E-03	1.45E-01	4.13E-02	8.19E-02	2.10E-03	1.46E-01	0
MTHFS	ENSG00000136371	15	80125927	80189721	6.16E-01	6.63E-01	1.93E-01	4.03E-01	8.22E-01	1.46E-01	0
ANKRD18A	ENSG00000273170	9	38540566	38577204	9.35E-01	6.55E-01	3.40E-01	1.92E-01	1.45E-01	1.47E-01	0
P2RY14	ENSG00000174944	3	150929905	150996255	1.89E-02	3.05E-02	3.21E-02	2.10E-03	2.16E-01	1.47E-01	0
DEFB129	ENSG00000125903	20	207899	210527	3.69E-02	3.57E-02	3.14E-02	3.11E-01	2.87E-02	1.47E-01	0
DGKH	ENSG00000102780	13	42614176	42830714	3.08E-01	4.74E-01	1.87E-01	1.79E-01	2.78E-01	1.47E-01	0
C21orf67	ENSG00000183250	21	46352729	46359828	7.74E-01	9.22E-01	3.64E-01	3.93E-01	8.59E-01	1.47E-01	0
OR52A5	ENSG00000171944	11	5152922	5153872	8.39E-01	7.15E-01	4.13E-01	8.02E-01	8.52E-01	1.47E-01	0
MIER3	ENSG00000155545	5	56215429	56267502	2.39E-01	6.19E-01	1.29E-01	6.41E-01	3.70E-01	1.47E-01	0
TBC1D15	ENSG00000121749	12	72233487	72320629	3.71E-01	2.07E-01	1.98E-01	9.67E-02	9.13E-01	1.47E-01	0
RAB11FIP2	ENSG00000107560	10	119764427	119806114	6.47E-01	6.72E-01	1.65E-01	8.73E-02	7.89E-02	1.47E-01	0
CAPRIN1	ENSG00000135387	11	34073230	34122703	2.90E-01	2.48E-01	1.49E-01	6.78E-01	6.88E-01	1.47E-01	0
RPL37	ENSG00000145592	5	40825364	40835437	7.70E-06	5.43E-01	3.54E-04	1.58E-01	3.68E-03	1.47E-01	0
GTF2A2	ENSG00000140307	15	59930261	59949740	2.64E-01	2.92E-01	1.07E-01	5.54E-01	7.34E-01	1.47E-01	0
RAB2A	ENSG00000104388	8	61429416	61536186	2.87E-01	2.29E-01	1.38E-01	8.10E-01	7.31E-01	1.47E-01	0
PSMC5	ENSG00000087191	17	61904512	61909379	7.51E-02	9.21E-02	2.57E-01	1.16E-01	2.91E-01	1.47E-01	0
ACTRT2	ENSG00000169717	1	2938046	2939465	9.15E-01	3.80E-01	2.28E-01	2.33E-01	2.06E-02	1.48E-01	0
MGAT4B	ENSG00000161013	5	179224597	179233952	4.56E-01	7.39E-01	2.17E-01	9.02E-01	2.46E-01	1.48E-01	0
OR2AT4	ENSG00000171561	11	74799758	74800799	6.24E-01	5.37E-01	2.89E-01	3.66E-01	7.19E-01	1.48E-01	0
B3GNT2	ENSG00000170340	2	62423248	62451866	7.34E-01	3.81E-01	6.59E-01	2.66E-01	9.27E-01	1.48E-01	0
DZANK1	ENSG00000089091	20	18364011	18447925	5.15E-01	3.23E-01	2.54E-01	6.15E-01	3.93E-01	1.48E-01	0
OR1D2	ENSG00000184166	17	2995352	2996290	8.90E-01	9.19E-02	2.65E-01	9.18E-01	6.86E-01	1.48E-01	0
HDDC2	ENSG00000111906	6	125541108	125623282	2.04E-01	3.81E-01	1.12E-01	5.15E-01	7.75E-01	1.48E-01	0
THUMPD2	ENSG00000138050	2	39963200	40006407	3.60E-01	8.99E-01	1.97E-01	4.04E-01	4.21E-01	1.48E-01	0
REPIN1	ENSG00000214022	7	150065278	150071133	2.10E-01	1.22E-01	2.99E-01	2.76E-02	2.72E-01	1.48E-01	0
C1orf192	ENSG00000188931	1	161334521	161337664	3.35E-02	5.68E-01	3.78E-02	7.00E-01	5.43E-02	1.48E-01	0
TBCD	ENSG00000141556	17	80709940	80900724	5.88E-01	5.28E-02	4.12E-01	5.11E-01	8.94E-01	1.48E-01	0
C10orf53	ENSG00000178645	10	50887697	50918307	7.21E-01	2.91E-02	4.78E-01	2.99E-01	2.37E-01	1.48E-01	0
ELL	ENSG00000105656	19	18553473	18632937	1.21E-02	1.72E-01	3.30E-02	2.09E-01	6.70E-01	1.49E-01	0
COPS8	ENSG00000198612	2	237993955	238009109	5.25E-02	6.64E-01	4.47E-02	3.86E-01	2.81E-01	1.49E-01	0
SZT2	ENSG00000198198	1	43855553	43918321	6.85E-02	7.93E-02	7.62E-02	1.81E-03	5.81E-02	1.49E-01	0
ARL2	ENSG00000213465	11	64781585	64789656	4.15E-02	7.97E-01	3.07E-02	3.26E-01	5.70E-01	1.49E-01	0

Gene	Ensembl ID	CHR	START	STOP	VHD_CD Pleiotropy P	VHD_GORD Pleiotropy P	VHD_IBD Pleiotropy P	VHD_IBS Pleiotropy P	VHD_PUD Pleiotropy P	VHD_UC Pleiotropy P	d
PSENEN	ENSG00000205155	19	36236015	36237911	1.78E-02	2.88E-01	1.59E-01	7.09E-02	9.42E-03	1.49E-01	0
HECTD1	ENSG00000092148	14	31569318	31677010	8.27E-02	4.92E-01	6.50E-02	3.92E-01	2.13E-01	1.49E-01	0
EXOSC4	ENSG00000178896	8	145133529	145135550	3.32E-01	4.54E-01	1.48E-01	3.04E-01	3.06E-01	1.49E-01	0
NIN	ENSG00000100503	14	51186481	51297839	2.12E-01	2.71E-01	9.25E-02	2.36E-01	3.57E-01	1.49E-01	0
LRRC10B	ENSG00000204950	11	61276272	61278482	3.32E-01	4.37E-01	4.61E-01	6.34E-01	4.94E-01	1.49E-01	0
SLAMF9	ENSG00000162723	1	159921282	159924044	1.08E-01	4.89E-02	1.03E-01	8.71E-01	4.08E-01	1.49E-01	0
AIF1L	ENSG00000126878	9	133971863	133998539	4.04E-01	5.55E-01	2.10E-01	3.20E-01	5.99E-01	1.49E-01	0
STX17	ENSG00000136874	9	102668915	102732618	1.57E-01	5.92E-01	9.51E-02	6.74E-01	1.19E-01	1.49E-01	0
ABHD11	ENSG00000106077	7	73150424	73153197	6.56E-01	5.82E-01	1.95E-01	6.75E-01	4.51E-01	1.49E-01	0
EXTL2	ENSG00000162694	1	101337943	101361554	5.31E-01	4.68E-01	2.37E-01	3.26E-01	6.19E-01	1.50E-01	0
ACBD4	ENSG00000181513	17	43209967	43221548	2.75E-01	1.55E-02	1.87E-01	2.23E-01	5.67E-02	1.50E-01	0
TRIM29	ENSG00000137699	11	119981983	120056237	7.44E-01	1.98E-01	4.26E-01	2.07E-01	1.22E-01	1.50E-01	0
EXOC6B	ENSG00000144036	2	72403113	73053170	2.68E-01	9.20E-01	1.74E-01	6.70E-01	8.97E-01	1.50E-01	0
GAN	ENSG00000261609	16	81348557	81424489	2.83E-01	4.86E-01	1.78E-01	4.40E-02	8.25E-01	1.50E-01	0
FOXRED2	ENSG00000100350	22	36883237	36903148	2.50E-01	8.67E-01	9.63E-01	8.89E-01	3.37E-01	1.50E-01	0
SLC3A2	ENSG00000168003	11	62623518	62656352	5.67E-01	8.05E-02	1.67E-01	8.07E-01	5.01E-01	1.50E-01	0
MIOX	ENSG00000100253	22	50925213	50929077	2.41E-01	8.20E-01	7.96E-02	5.87E-01	8.90E-01	1.50E-01	0
TMEM109	ENSG00000110108	11	60681346	60690915	3.94E-01	7.66E-01	1.20E-01	7.34E-01	7.85E-01	1.50E-01	0
C2orf68	ENSG00000168887	2	85833777	85839189	4.02E-01	5.14E-02	4.65E-01	9.03E-02	3.58E-01	1.50E-01	0
PAQR4	ENSG00000162073	16	3019246	3023490	6.14E-01	8.94E-01	2.04E-01	9.58E-01	9.04E-01	1.50E-01	0
TLCD1	ENSG00000160606	17	27051366	27054953	3.87E-01	5.19E-01	7.09E-02	6.98E-01	1.66E-01	1.50E-01	0
TMEM221	ENSG00000188051	19	17546318	17559376	3.81E-02	6.80E-01	6.07E-02	5.50E-01	5.42E-02	1.50E-01	0
MRPS16	ENSG00000182180	10	75006510	75012451	5.36E-01	5.24E-01	2.53E-01	1.13E-01	3.66E-02	1.50E-01	0
ACY3	ENSG00000132744	11	67410026	67418130	4.95E-01	5.15E-01	2.91E-01	2.68E-01	5.52E-01	1.50E-01	0
CYP4X1	ENSG00000186377	1	47427036	47516423	1.90E-01	2.98E-01	4.70E-02	1.87E-02	7.11E-02	1.50E-01	0
HSPA14	ENSG00000187522	10	14880163	14913740	6.46E-01	2.87E-02	8.09E-02	6.73E-01	1.70E-01	1.50E-01	0
UTS2	ENSG00000049247	1	7903143	7913572	1.08E-01	7.02E-01	9.36E-02	6.16E-01	8.44E-01	1.50E-01	0
CCDC92	ENSG00000119242	12	124403207	124457378	2.82E-01	8.66E-02	9.13E-02	4.71E-02	3.80E-01	1.50E-01	0
CCDC34	ENSG00000109881	11	27352374	27385415	2.33E-01	2.98E-02	1.81E-01	7.28E-01	3.15E-02	1.50E-01	0
CERS3	ENSG00000154227	15	100940600	101085200	6.18E-01	5.24E-01	2.84E-01	7.07E-01	2.56E-01	1.50E-01	0
C9orf91	ENSG00000157693	9	117373486	117408702	7.18E-02	1.06E-01	5.51E-02	3.19E-01	5.33E-01	1.50E-01	0
HSBP1	ENSG00000230989	16	83841448	83853342	3.63E-01	7.20E-03	1.32E-01	4.25E-01	2.44E-01	1.50E-01	0
MAL2	ENSG00000147676	8	120177273	120257913	5.38E-01	6.66E-01	1.73E-01	4.55E-01	4.69E-01	1.50E-01	0
RLBP1	ENSG00000140522	15	89753100	89764982	5.64E-01	3.18E-01	4.06E-01	5.92E-02	5.26E-01	1.51E-01	0
IGFL3	ENSG00000188624	19	46623328	46627931	2.11E-01	8.94E-02	3.82E-01	1.76E-01	7.76E-01	1.51E-01	0
EFCAB3	ENSG00000172421	17	60447579	60493837	3.65E-01	3.85E-01	1.64E-01	3.77E-01	1.50E-01	1.51E-01	0
NCOA1	ENSG00000084676	2	24714783	24993571	6.32E-02	6.77E-02	2.64E-02	3.99E-01	4.29E-01	1.51E-01	0
CLEC10A	ENSG00000132514	17	6977856	6983626	4.87E-02	3.76E-01	4.86E-02	9.36E-01	8.93E-01	1.51E-01	0
OR4A16	ENSG00000181961	11	55110627	55111707	3.34E-01	3.38E-01	1.78E-01	3.19E-01	2.94E-01	1.51E-01	0
MAFB	ENSG00000204103	20	39314488	39317880	1.82E-01	4.48E-01	7.58E-01	7.25E-02	8.02E-01	1.51E-01	0
YJEFN3	ENSG00000250067	19	19627036	19648390	3.07E-01	1.42E-01	1.81E-01	5.70E-01	3.35E-01	1.51E-01	0
PNISR	ENSG00000132424	6	99845927	99873207	4.25E-02	8.23E-02	7.50E-01	9.36E-01	3.94E-02	1.51E-01	0
PLEKHO1	ENSG00000023902	1	150121373	150136916	8.44E-02	3.64E-01	6.18E-01	1.56E-01	7.34E-02	1.51E-01	0
EME1	ENSG00000154920	17	48450581	48458844	4.18E-01	3.75E-01	2.11E-01	3.06E-01	5.25E-02	1.51E-01	0
SMARCD2	ENSG00000108604	17	61909444	61920425	5.33E-02	1.02E-01	1.88E-01	1.19E-01	2.53E-01	1.51E-01	0
POLR3F	ENSG00000132664	20	18447771	18465287	3.29E-01	1.06E-01	1.51E-01	4.01E-01	3.74E-01	1.51E-01	0
ENPP6	ENSG00000164303	4	185009859	185142383	6.72E-01	1.93E-01	3.23E-01	4.71E-01	3.53E-01	1.51E-01	0
SPOP	ENSG00000121067	17	47676246	47755596	1.59E-01	3.54E-02	1.83E-01	4.89E-01	2.41E-02	1.51E-01	0
DUPD1	ENSG00000188716	10	76797594	76818272	6.31E-02	2.36E-01	4.02E-02	1.01E-01	4.83E-02	1.52E-01	0

Gene	Ensembl ID	CHR	START	STOP	VHD_CD Pleiotropy P	VHD_GORD Pleiotropy P	VHD_IBD Pleiotropy P	VHD_IBS Pleiotropy P	VHD_PUD Pleiotropy P	VHD_UC Pleiotropy P	d
ANKIB1	ENSG00000001629	7	91875548	92030698	6.59E-02	5.25E-02	2.31E-01	1.74E-01	9.08E-02	1.52E-01	0
MYOG	ENSG00000122180	1	203052260	203055164	5.26E-01	3.30E-01	3.21E-01	7.57E-01	7.74E-01	1.52E-01	0
DEFB118	ENSG00000131068	20	29956421	29961726	9.50E-02	1.92E-02	4.74E-02	1.11E-01	1.02E-01	1.52E-01	0
ATP5G2	ENSG00000135390	12	54026510	54071192	1.05E-01	4.92E-01	7.23E-02	2.88E-01	1.21E-01	1.52E-01	0
CCDC79	ENSG00000249961	16	66788879	66835523	1.87E-01	3.38E-01	1.00E-01	1.89E-01	5.05E-01	1.52E-01	0
GDPD5	ENSG00000158555	11	75145685	75236948	1.36E-01	8.73E-02	1.78E-01	2.39E-02	2.51E-01	1.52E-01	0
HOXD1	ENSG00000128645	2	177053307	177055688	1.20E-01	7.92E-01	7.21E-02	1.01E-01	1.82E-01	1.52E-01	0
NCAPD2	ENSG00000010292	12	6602522	6641121	2.06E-02	2.24E-01	2.33E-02	1.70E-01	4.08E-01	1.52E-01	0
BMS1	ENSG00000165733	10	43278249	43330385	7.75E-01	3.67E-01	3.69E-01	7.09E-01	6.03E-01	1.52E-01	0
H2AFX	ENSG00000188486	11	118964564	118966177	7.31E-01	1.21E-01	2.77E-01	3.20E-01	2.48E-01	1.52E-01	0
ZNF615	ENSG00000197619	19	52494585	52511483	1.99E-01	4.53E-02	6.59E-01	2.66E-01	3.96E-01	1.52E-01	0
RECK	ENSG00000122707	9	36036430	36124448	3.65E-01	1.91E-01	4.23E-01	2.64E-01	5.56E-01	1.52E-01	0
CARD17	ENSG00000255221	11	104963196	104972158	6.48E-01	7.13E-01	3.37E-01	7.85E-01	8.82E-02	1.52E-01	0
ZNF512	ENSG00000243943	2	27805897	27858041	3.62E-02	4.57E-01	3.83E-02	1.78E-01	2.88E-01	1.52E-01	0
TAOK2	ENSG00000149930	16	29984962	30003582	3.00E-01	5.69E-01	1.48E-01	6.62E-01	8.87E-01	1.52E-01	0
ANO5	ENSG00000171714	11	22214722	22304903	8.78E-01	2.52E-01	1.55E-01	3.21E-05	4.95E-01	1.52E-01	0
SWI5	ENSG00000175854	9	131037658	131051269	6.54E-01	2.91E-03	7.37E-01	2.04E-01	3.66E-03	1.52E-01	0
WBP2NL	ENSG00000183066	22	42394729	42454460	5.33E-01	5.67E-01	2.76E-01	2.24E-01	1.28E-01	1.52E-01	0
ALOX5	ENSG00000012779	10	45869661	45941561	2.69E-01	5.18E-01	6.63E-02	1.16E-01	5.59E-01	1.52E-01	0
CCDC62	ENSG00000130783	12	123258874	123312075	4.88E-02	5.91E-02	1.40E-01	2.91E-01	2.30E-02	1.53E-01	0
C17orf82	ENSG00000187013	17	59489112	59490641	3.77E-01	9.23E-01	2.48E-01	2.86E-01	1.56E-01	1.53E-01	0
DTX3L	ENSG00000163840	3	122283085	122294050	5.18E-01	4.03E-01	2.74E-01	3.15E-01	6.82E-01	1.53E-01	0
TNIK	ENSG00000154310	3	170779128	171178197	5.49E-01	8.28E-02	4.81E-01	4.97E-01	2.32E-01	1.53E-01	0
ALG3	ENSG00000214160	3	183960089	183967336	2.20E-01	9.48E-02	3.96E-01	1.05E-02	1.96E-01	1.53E-01	0
INTS5	ENSG00000185085	11	62414320	62420774	2.96E-02	1.11E-01	3.66E-02	6.14E-03	2.27E-01	1.53E-01	0
SLC22A11	ENSG00000168065	11	64323098	64340347	1.51E-01	2.04E-01	5.07E-02	6.83E-01	1.61E-01	1.53E-01	0
CHPT1	ENSG00000111666	12	102090725	102137918	6.03E-01	6.97E-01	5.74E-01	5.09E-01	4.71E-01	1.53E-01	0
NOS1	ENSG00000089250	12	117645947	117889975	1.30E-01	3.04E-02	1.52E-01	3.51E-02	1.89E-02	1.53E-01	0
ATP8B4	ENSG00000104043	15	50150435	50475014	4.19E-01	6.59E-01	4.23E-01	3.59E-01	2.12E-01	1.53E-01	0
TXNL4B	ENSG00000140830	16	72078188	72128330	4.90E-02	3.07E-01	5.05E-02	5.10E-01	3.61E-01	1.53E-01	0
CYP4F11	ENSG00000171903	19	16023177	16045677	6.26E-01	2.54E-01	7.40E-01	1.51E-01	3.74E-01	1.53E-01	0
TJAP1	ENSG00000137221	6	43445261	43474294	1.27E-01	6.66E-02	1.18E-01	5.30E-01	4.77E-01	1.53E-01	0
C11orf91	ENSG00000205177	11	33719807	33722347	8.85E-01	4.55E-01	3.27E-01	5.48E-01	8.40E-01	1.53E-01	0
CPEB2	ENSG00000137449	4	15004298	15071777	3.86E-02	4.84E-01	1.09E-01	5.57E-01	2.43E-01	1.53E-01	0
ST6GALNAC4	ENSG00000136840	9	130670165	130679317	2.74E-01	5.63E-01	1.77E-01	7.40E-01	4.60E-01	1.53E-01	0
SCGB2A1	ENSG00000124939	11	61976140	61981408	1.25E-01	1.68E-01	1.08E-01	4.14E-01	4.71E-01	1.53E-01	0
SHC1	ENSG00000160691	1	154934774	154946871	1.12E-01	1.48E-01	1.02E-01	1.18E-01	6.76E-02	1.53E-01	0
ITPR3	ENSG00000096433	6	33588142	33664351	3.83E-01	6.61E-01	3.67E-01	2.80E-01	3.31E-01	1.53E-01	0
SLC7A8	ENSG00000092068	14	23594504	23652883	2.88E-01	2.71E-02	7.83E-02	7.45E-01	7.70E-01	1.53E-01	0
LSM10	ENSG00000181817	1	36856839	36863493	2.65E-01	1.98E-01	1.42E-01	3.47E-01	8.90E-02	1.53E-01	0
PTGER4	ENSG00000171522	5	40679600	40693837	7.85E-04	7.57E-01	8.37E-03	7.89E-02	1.77E-02	1.53E-01	0
UBXN4	ENSG00000144224	2	136499189	136542625	3.05E-02	4.80E-01	2.17E-02	9.82E-03	7.50E-01	1.54E-01	0
MATR3	ENSG00000015479	5	138609441	138667360	1.47E-01	5.88E-01	2.10E-01	6.84E-01	4.76E-01	1.54E-01	0
TMEM2	ENSG00000135048	9	74298282	74431606	4.22E-01	7.48E-01	2.26E-01	3.80E-02	5.73E-01	1.54E-01	0
MINK1	ENSG00000141503	17	4736683	4801356	3.37E-01	5.71E-01	1.39E-01	5.09E-01	3.76E-01	1.54E-01	0
TMEM9B	ENSG00000175348	11	8968841	8986558	4.15E-01	9.09E-01	2.14E-01	1.31E-01	6.13E-01	1.54E-01	0
TEX264	ENSG00000164081	3	51696709	51738339	3.31E-01	4.68E-01	8.79E-02	5.60E-01	7.15E-01	1.54E-01	0
OR2L3	ENSG00000198128	1	248223984	248224922	5.87E-01	5.89E-02	2.57E-01	4.74E-01	6.12E-01	1.54E-01	0
RABGGTA	ENSG00000100949	14	24734744	24740945	3.90E-01	5.72E-01	3.32E-01	7.74E-01	1.52E-02	1.54E-01	0

Gene	Ensembl ID	CHR	START	STOP	VHD_CD Pleiotropy P	VHD_GORD Pleiotropy P	VHD_IBD Pleiotropy P	VHD_IBS Pleiotropy P	VHD_PUD Pleiotropy P	VHD_UC Pleiotropy P	d
DHX34	ENSG00000134815	19	47852538	47885961	5.96E-01	5.51E-01	4.26E-01	1.90E-01	3.47E-01	1.54E-01	0
DPAGT1	ENSG00000172269	11	118967213	118979041	8.94E-01	2.43E-01	2.70E-01	3.96E-01	6.30E-01	1.54E-01	0
FGD5	ENSG00000154783	3	14860469	14975895	7.24E-02	2.95E-01	3.69E-02	3.96E-01	1.10E-01	1.54E-01	0
SMC1B	ENSG00000077935	22	45739944	45809500	2.54E-01	3.35E-01	2.08E-01	6.84E-01	2.91E-01	1.54E-01	0
HLA-E	ENSG00000204592	6	30457244	30461982	1.50E-01	2.64E-01	6.03E-01	5.93E-01	1.80E-01	1.54E-01	0
PDGFA	ENSG00000197461	7	536895	559933	2.58E-01	2.79E-01	1.12E-01	3.38E-01	1.01E-01	1.54E-01	0
KIFC3	ENSG00000140859	16	57792129	57896957	1.73E-01	3.12E-01	1.99E-01	3.68E-01	1.31E-01	1.54E-01	0
UBXN11	ENSG00000158062	1	26607819	26644854	5.98E-01	5.60E-01	1.63E-01	5.81E-01	7.64E-01	1.54E-01	0
TAOK1	ENSG00000160551	17	27717482	27878922	6.82E-01	6.01E-01	5.44E-01	2.99E-01	4.12E-01	1.54E-01	0
C11orf80	ENSG00000173715	11	66511922	66610987	6.89E-01	5.88E-03	4.17E-01	2.62E-01	6.61E-01	1.54E-01	0
YIPF3	ENSG00000137207	6	43479565	43484728	2.25E-01	1.93E-01	1.86E-01	7.09E-01	9.39E-01	1.55E-01	0
FGF17	ENSG00000158815	8	21899909	21906320	7.92E-03	1.14E-02	2.09E-02	2.91E-01	2.94E-01	1.55E-01	0
USP24	ENSG00000162402	1	55532032	55680786	8.72E-01	8.88E-01	2.43E-01	8.46E-01	6.05E-01	1.55E-01	0
ZNF491	ENSG00000177599	19	11908482	11920437	2.14E-01	4.86E-01	1.05E-01	2.54E-01	1.78E-01	1.55E-01	0
PLIN1	ENSG00000166819	15	90207596	90222658	2.50E-01	7.91E-01	2.03E-01	8.43E-01	4.12E-01	1.55E-01	0
P2RY12	ENSG00000169313	3	151055168	151102600	6.92E-03	1.06E-02	2.54E-02	7.09E-05	6.25E-01	1.55E-01	0
KRT32	ENSG00000108759	17	39616063	39623681	5.69E-01	4.67E-01	5.75E-01	3.93E-01	1.86E-01	1.55E-01	0
TSPAN5	ENSG00000168785	4	99391518	99579780	8.15E-01	6.35E-01	5.62E-01	2.72E-01	6.42E-01	1.55E-01	0
CILP2	ENSG00000160161	19	19649057	19657468	2.69E-01	9.51E-02	1.76E-01	6.20E-01	4.89E-01	1.55E-01	0
CD22	ENSG00000012124	19	35810164	35838258	7.86E-01	8.87E-01	5.11E-01	8.28E-01	6.58E-01	1.55E-01	0
TREM2	ENSG00000095970	6	41126244	41130924	7.11E-01	7.14E-01	2.04E-01	3.12E-01	4.53E-01	1.55E-01	0
MAN2B2	ENSG00000013288	4	6576902	6625089	3.21E-02	9.19E-01	7.62E-02	7.17E-01	6.36E-01	1.55E-01	0
HELZ	ENSG00000198265	17	65066554	65242105	2.61E-01	7.82E-01	8.99E-02	5.25E-01	2.20E-01	1.55E-01	0
CAV1	ENSG00000105974	7	116164839	116201233	1.62E-02	2.33E-03	5.52E-02	1.48E-03	9.10E-03	1.55E-01	0
ITGA6	ENSG00000091409	2	173292082	173371181	5.33E-01	4.65E-02	3.84E-01	2.03E-01	5.98E-02	1.55E-01	0
MLN	ENSG00000096395	6	33762450	33771788	4.02E-01	2.92E-01	2.72E-01	8.09E-02	6.75E-01	1.55E-01	0
FNDC7	ENSG00000143107	1	109255279	109285365	1.68E-01	1.26E-01	1.66E-01	4.12E-01	6.58E-01	1.55E-01	0
TMEM126A	ENSG00000171202	11	85359011	85367591	2.90E-01	7.20E-02	3.09E-01	4.35E-01	1.32E-01	1.55E-01	0
ANGPTL4	ENSG00000167772	19	8428173	8439257	2.37E-01	8.50E-01	1.44E-01	7.64E-01	2.89E-01	1.55E-01	0
ZNF589	ENSG00000164048	3	48282590	48340743	5.51E-02	1.55E-01	1.73E-01	4.39E-01	1.33E-01	1.55E-01	0
SP7	ENSG00000170374	12	53720362	53739099	1.73E-01	4.06E-01	1.03E-01	1.15E-01	5.77E-02	1.55E-01	0
NLE1	ENSG00000073536	17	33455772	33469334	2.77E-01	7.71E-02	2.88E-01	5.84E-01	6.75E-02	1.55E-01	0
EMP1	ENSG00000134531	12	13349650	13369708	4.55E-02	1.70E-01	1.12E-01	7.30E-02	4.02E-01	1.55E-01	0
ITPKA	ENSG00000137825	15	41785591	41795747	9.30E-01	9.73E-01	6.05E-01	5.80E-01	5.55E-01	1.55E-01	0
STAMBP	ENSG00000124356	2	74056086	74100786	3.82E-01	2.41E-02	8.18E-01	5.83E-02	1.95E-02	1.55E-01	0
EIF4G1	ENSG00000114867	3	184032283	184053146	1.56E-01	7.64E-02	8.62E-02	5.61E-03	3.36E-02	1.55E-01	0
RGS10	ENSG00000148908	10	121259340	121302220	2.61E-01	2.97E-01	1.16E-01	7.76E-01	4.12E-01	1.55E-01	0
KPNA5	ENSG00000196911	6	117002350	117063029	6.81E-01	8.02E-01	2.47E-01	8.96E-01	6.84E-01	1.56E-01	0
CEBPE	ENSG00000092067	14	23586513	23588825	2.85E-01	3.35E-01	1.94E-01	4.12E-01	3.73E-01	1.56E-01	0
SPAM1	ENSG00000106304	7	123565286	123611468	4.41E-01	3.11E-02	1.42E-01	3.44E-01	1.09E-01	1.56E-01	0
RPL9	ENSG00000163682	4	39455744	39460568	2.68E-02	3.33E-02	1.98E-02	1.17E-02	4.07E-01	1.56E-01	0
PIGF	ENSG00000151665	2	46808076	46844258	4.48E-01	6.05E-01	4.98E-01	2.83E-01	3.23E-01	1.56E-01	0
PTPN12	ENSG00000127947	7	77166592	77269388	5.60E-01	3.71E-01	6.58E-01	2.93E-01	7.24E-01	1.56E-01	0
AP3D1	ENSG00000065000	19	2100988	2164464	2.21E-02	6.49E-02	8.61E-02	5.58E-02	6.52E-03	1.56E-01	0
SFR1	ENSG00000156384	10	105881816	105886143	4.49E-01	1.99E-01	1.98E-01	5.39E-01	2.60E-01	1.56E-01	0
SGIP1	ENSG00000118473	1	66999066	67213982	3.05E-01	5.32E-01	1.03E-01	9.37E-02	6.90E-01	1.56E-01	0
UBAP1L	ENSG00000246922	15	65385338	65407535	2.55E-01	7.73E-01	3.19E-01	5.01E-01	3.49E-01	1.56E-01	0
ARPC1A	ENSG00000241685	7	98923521	98985787	5.06E-02	7.14E-01	3.71E-02	3.92E-01	6.63E-02	1.56E-01	0
ASF1A	ENSG00000111875	6	119215384	119230332	2.66E-01	1.91E-01	2.22E-01	2.19E-01	3.21E-01	1.56E-01	0

Gene	Ensembl ID	CHR	START	STOP	VHD_CD Pleiotropy P	VHD_GORD Pleiotropy P	VHD_IBD Pleiotropy P	VHD_IBS Pleiotropy P	VHD_PUD Pleiotropy P	VHD_UC Pleiotropy P	d
DNAJC7	ENSG00000168259	17	40128451	40173394	7.47E-02	3.82E-01	3.61E-02	5.80E-01	8.37E-01	1.56E-01	0
DIRC3	ENSG00000231672	2	218148742	218621316	5.81E-01	6.32E-01	7.41E-01	2.66E-01	4.68E-01	1.56E-01	0
TMEM150C	ENSG00000249242	4	83404323	83483510	4.91E-01	6.11E-01	3.28E-01	3.15E-01	2.93E-01	1.56E-01	0
RPL27A	ENSG00000166441	11	8703958	8736306	9.04E-02	3.94E-01	1.06E-01	8.73E-01	2.89E-01	1.56E-01	0
CLPX	ENSG00000166855	15	65440557	65477680	2.10E-01	8.42E-01	2.76E-01	2.17E-01	9.14E-02	1.56E-01	0
PRELP	ENSG00000188783	1	203444956	203460480	4.02E-01	2.81E-02	4.96E-01	9.07E-01	3.19E-01	1.56E-01	0
SUDS3	ENSG00000111707	12	118814185	118855840	9.80E-01	1.14E-01	4.56E-01	9.56E-03	5.61E-02	1.56E-01	0
DEFB116	ENSG00000215545	20	29891015	29896388	5.45E-02	1.60E-02	4.20E-02	3.04E-02	2.13E-01	1.56E-01	0
DLL3	ENSG00000090932	19	39989535	39999121	4.29E-01	8.63E-02	2.89E-01	3.96E-01	6.95E-01	1.56E-01	0
MUC4	ENSG00000145113	3	195473636	195539148	4.52E-01	2.99E-01	1.86E-01	5.25E-01	1.39E-01	1.56E-01	0
HIST1H1C	ENSG00000187837	6	26055968	26056699	8.18E-02	3.60E-03	2.97E-01	8.52E-02	5.44E-01	1.56E-01	0
NKAPL	ENSG00000189134	6	28227098	28228736	4.23E-02	1.85E-02	7.69E-02	3.08E-01	7.95E-01	1.56E-01	0
SLC46A1	ENSG00000076351	17	26721661	26734215	9.79E-01	2.78E-01	3.99E-01	8.43E-01	2.13E-01	1.57E-01	0
C1orf54	ENSG00000118292	1	150240600	150253327	3.84E-01	8.36E-02	1.79E-01	1.14E-01	3.56E-01	1.57E-01	0
QKI	ENSG00000112531	6	163835032	163999628	4.28E-01	7.97E-01	4.32E-01	1.64E-01	6.89E-02	1.57E-01	0
TRIM13	ENSG00000204977	13	50570024	50594617	8.93E-01	1.13E-01	3.90E-01	2.49E-01	8.22E-01	1.57E-01	0
PGM3	ENSG00000013375	6	83870869	83903655	2.65E-01	2.69E-01	1.94E-01	1.89E-01	6.90E-01	1.57E-01	0
ECD	ENSG00000122882	10	74889913	74928813	4.10E-01	5.77E-01	1.59E-01	2.92E-02	3.13E-02	1.57E-01	0
ANKRD6	ENSG00000135299	6	90142889	90343553	2.15E-01	3.73E-01	4.15E-01	2.18E-01	1.30E-01	1.57E-01	0
PAX8	ENSG00000125618	2	113973574	114036527	1.72E-01	6.91E-01	8.92E-02	9.08E-01	7.94E-01	1.57E-01	0
C5AR1	ENSG00000197405	19	47793280	47825323	4.17E-01	5.06E-01	1.99E-01	4.42E-01	4.58E-01	1.57E-01	0
SLC35C2	ENSG00000080189	20	44978167	44993043	8.14E-01	4.70E-01	5.55E-01	9.03E-01	6.38E-01	1.57E-01	0
HECA	ENSG00000112406	6	139456249	139501939	1.79E-01	2.15E-01	1.44E-01	1.70E-01	2.08E-01	1.57E-01	0
MYO1B	ENSG00000128641	2	192109911	192290115	5.51E-01	4.05E-02	5.23E-01	1.38E-01	4.13E-01	1.57E-01	0
ASB6	ENSG00000148331	9	132399171	132404444	1.55E-01	5.13E-01	6.20E-02	5.94E-01	3.24E-01	1.57E-01	0
COL14A1	ENSG00000187955	8	121072019	121384275	4.17E-01	3.19E-03	3.72E-01	4.00E-01	2.75E-01	1.57E-01	0
NINJ1	ENSG00000131669	9	95883771	95896570	8.69E-01	6.70E-01	2.82E-01	4.30E-01	4.94E-01	1.57E-01	0
ZNF253	ENSG00000256771	19	19976695	20005483	9.37E-01	4.68E-01	2.64E-01	2.58E-01	2.89E-01	1.57E-01	0
KRTAP10-11	ENSG00000243489	21	46066331	46067564	9.77E-01	1.60E-01	5.59E-01	4.74E-01	3.11E-01	1.58E-01	0
PAG1	ENSG00000076641	8	81880045	82024303	7.00E-01	9.09E-01	2.48E-01	9.75E-01	9.32E-01	1.58E-01	0
ACSL5	ENSG00000197142	10	114133776	114188138	2.97E-01	9.78E-02	1.94E-01	5.71E-01	4.43E-01	1.58E-01	0
CARS2	ENSG00000134905	13	111293759	111365950	3.42E-03	4.52E-01	4.31E-02	5.61E-02	7.22E-03	1.58E-01	0
CDCA3	ENSG00000111665	12	6953957	6961230	2.17E-01	6.62E-01	4.48E-01	7.37E-01	8.66E-01	1.58E-01	0
GNB3	ENSG00000111664	12	6949118	6956557	2.17E-01	8.12E-01	4.48E-01	8.26E-01	9.60E-01	1.58E-01	0
TMPRSS11A	ENSG00000187054	4	68775103	68829858	1.31E-02	4.85E-02	4.92E-02	1.75E-01	4.60E-02	1.58E-01	0
RALB	ENSG00000144118	2	120997640	121052289	2.68E-01	1.50E-01	3.27E-01	2.42E-01	2.95E-01	1.58E-01	0
HHIP	ENSG00000164161	4	145567173	145666423	2.97E-02	4.54E-01	5.82E-02	7.02E-01	1.26E-01	1.58E-01	0
ATP6AP1L	ENSG00000205464	5	81575281	81682796	9.00E-02	9.84E-02	1.47E-01	7.36E-01	4.03E-01	1.58E-01	0
ZNF420	ENSG00000197050	19	37498759	37621216	1.17E-03	8.26E-02	4.91E-03	5.31E-01	2.87E-01	1.58E-01	0
ANGPTL7	ENSG00000171819	1	11249398	11256038	4.13E-01	4.99E-01	3.77E-01	1.18E-01	6.04E-01	1.58E-01	0
CMTM6	ENSG00000091317	3	32522804	32544900	5.32E-01	6.78E-01	3.49E-01	3.40E-01	1.20E-01	1.58E-01	0
GATAD2B	ENSG00000143614	1	153777201	153895451	6.45E-02	1.76E-01	5.88E-01	6.72E-02	2.12E-01	1.58E-01	0
ADK	ENSG00000156110	10	75910960	76469061	3.77E-01	7.77E-01	1.10E-01	8.62E-01	7.12E-01	1.58E-01	0
TTC33	ENSG00000113638	5	40714577	40756077	5.97E-05	7.90E-01	2.12E-03	1.23E-01	9.74E-03	1.58E-01	0
RRP15	ENSG00000067533	1	218458629	218511325	9.54E-04	2.36E-01	6.97E-03	4.28E-03	2.53E-02	1.58E-01	0
CASP6	ENSG00000138794	4	110609785	110624739	1.88E-01	3.80E-02	2.23E-01	5.67E-02	9.44E-02	1.59E-01	0
TOP1	ENSG00000198900	20	39657458	39753127	1.35E-01	7.04E-01	1.54E-01	5.39E-01	3.48E-01	1.59E-01	0
ANTXR1	ENSG00000169604	2	69240310	69476459	8.41E-01	6.06E-01	2.10E-01	8.07E-01	7.55E-01	1.59E-01	0
ZFYVE20	ENSG00000131381	3	15111580	15140670	6.44E-01	1.02E-01	2.63E-01	7.07E-01	3.27E-02	1.59E-01	0

Gene	Ensembl ID	CHR	START	STOP	VHD_CD Pleiotropy P	VHD_GORD Pleiotropy P	VHD_IBD Pleiotropy P	VHD_IBS Pleiotropy P	VHD_PUD Pleiotropy P	VHD_UC Pleiotropy P	d
FKBP5	ENSG00000096060	6	35541362	35696360	4.86E-02	4.52E-01	6.68E-02	3.31E-01	5.44E-01	1.59E-01	0
SHANK3	ENSG00000251322	22	51112843	51171726	6.99E-01	1.02E-01	5.54E-01	9.40E-02	5.41E-01	1.59E-01	0
LIN54	ENSG00000189308	4	83831126	83934079	1.65E-01	1.28E-01	9.87E-02	2.02E-01	3.40E-01	1.59E-01	0
NPTX1	ENSG00000171246	17	78440948	78451643	2.25E-01	1.34E-03	3.04E-01	5.58E-01	2.54E-03	1.59E-01	0
WNT2B	ENSG00000134245	1	113009163	113072787	1.46E-02	6.05E-01	2.49E-02	1.95E-01	5.30E-02	1.59E-01	0
F11R	ENSG00000158769	1	160965001	160991138	5.16E-01	7.32E-01	1.70E-01	3.82E-01	5.72E-01	1.59E-01	0
POLR2E	ENSG00000099817	19	1086594	1095598	1.20E-02	9.95E-02	2.29E-02	2.99E-01	2.41E-01	1.59E-01	0
SUPT6H	ENSG00000109111	17	26989109	27029697	4.83E-01	4.09E-01	1.53E-01	6.94E-01	7.05E-01	1.59E-01	0
DNM1L	ENSG00000087470	12	32832134	32898486	1.38E-01	1.47E-01	5.84E-01	7.52E-02	5.72E-01	1.59E-01	0
KERA	ENSG00000139330	12	91444268	91451760	5.89E-01	7.92E-01	2.90E-01	3.68E-01	2.69E-01	1.59E-01	0
KLK4	ENSG00000167749	19	51409608	51413994	5.88E-01	5.11E-01	1.60E-01	6.60E-01	8.79E-01	1.60E-01	0
KDELR1	ENSG00000105438	19	48885827	48894810	1.04E-01	3.89E-01	3.46E-01	3.90E-01	5.15E-01	1.60E-01	0
TEAD3	ENSG00000007866	6	35441374	35464853	8.09E-01	4.70E-01	2.81E-01	7.58E-01	7.93E-01	1.60E-01	0
TCEA3	ENSG00000204219	1	23707554	23751233	8.74E-01	6.02E-01	5.62E-01	7.61E-01	8.39E-01	1.60E-01	0
TSN	ENSG00000211460	2	122494679	122525429	4.33E-01	3.61E-01	3.26E-01	3.51E-01	6.16E-02	1.60E-01	0
DDX52	ENSG00000141141	17	35969787	36003493	2.26E-01	3.00E-01	1.34E-01	2.10E-01	6.14E-01	1.60E-01	0
FRYL	ENSG00000075539	4	48499378	48782339	1.39E-02	8.81E-01	5.18E-02	2.58E-01	4.41E-01	1.60E-01	0
OR51B2	ENSG00000184881	11	5344541	5345582	2.76E-01	5.78E-01	2.73E-01	2.34E-01	1.60E-01	1.60E-01	0
SPATA9	ENSG00000145757	5	94987885	95034415	2.04E-01	1.39E-01	4.10E-01	3.31E-01	1.03E-01	1.60E-01	0
BCL6B	ENSG00000161940	17	6926339	6933609	5.50E-01	1.68E-01	2.32E-01	3.64E-01	1.05E-01	1.60E-01	0
NKX1-1	ENSG00000235608	4	1396720	1400119	2.95E-02	3.86E-01	6.78E-02	8.20E-01	3.25E-01	1.60E-01	0
OR4D5	ENSG00000171014	11	123810250	123811344	3.63E-01	3.49E-01	1.93E-01	8.97E-01	3.86E-01	1.60E-01	0
KIAA0408	ENSG00000189367	6	127761488	127780536	6.06E-01	8.83E-02	6.65E-01	6.01E-01	1.12E-01	1.60E-01	0
TRAF7	ENSG00000131653	16	2205699	2228130	1.01E-01	5.63E-01	1.00E-01	5.41E-01	6.48E-01	1.60E-01	0
C18orf8	ENSG00000141452	18	21083473	21111746	3.16E-02	1.85E-02	2.65E-02	5.65E-03	5.31E-02	1.60E-01	0
IL19	ENSG00000142224	1	206972215	207016324	2.26E-01	9.62E-01	6.87E-02	9.53E-01	8.90E-01	1.60E-01	0
CNTD2	ENSG00000105219	19	40728115	40732597	3.32E-01	6.36E-01	1.05E-01	8.56E-01	2.00E-01	1.60E-01	0
EXOC3L4	ENSG00000205436	14	103566481	103576896	3.20E-01	2.54E-01	3.73E-01	6.97E-01	7.58E-01	1.60E-01	0
PRPF39	ENSG00000185246	14	45553302	45585485	6.84E-01	4.93E-01	3.37E-01	6.57E-01	1.69E-01	1.60E-01	0
OCIAD1	ENSG00000109180	4	48807229	48863834	7.48E-02	7.23E-01	8.07E-02	6.88E-01	2.49E-01	1.61E-01	0
ABCA8	ENSG00000141338	17	66863433	66951533	8.35E-01	2.37E-01	2.75E-01	8.51E-01	6.28E-01	1.61E-01	0
HOXD10	ENSG00000128710	2	176973518	176984670	9.20E-02	1.43E-01	5.38E-02	5.46E-01	4.28E-01	1.61E-01	0
RAPSN	ENSG00000165917	11	47459308	47470730	8.59E-02	1.99E-01	7.12E-02	7.93E-02	6.00E-01	1.61E-01	0
ZNF184	ENSG00000096654	6	27418522	27440897	1.75E-01	3.86E-02	2.15E-01	3.31E-01	5.28E-01	1.61E-01	0
RBM4	ENSG00000173933	11	66384097	66434153	6.48E-01	3.70E-01	4.01E-01	3.71E-01	5.63E-01	1.61E-01	0
HSP90AA1	ENSG00000080824	14	102547075	102606036	1.38E-01	5.73E-01	8.81E-02	2.78E-01	5.45E-01	1.61E-01	0
CD4	ENSG00000010610	12	6896024	6929974	3.28E-01	8.57E-01	1.24E-01	3.08E-01	8.10E-01	1.61E-01	0
KRTAP19-8	ENSG00000206102	21	32410478	32410795	2.04E-01	5.83E-01	1.44E-01	3.75E-01	2.67E-01	1.61E-01	0
TOMM6	ENSG00000214736	6	41755400	41757636	3.74E-01	7.03E-01	2.61E-01	7.30E-02	2.76E-01	1.61E-01	0
ZNF257	ENSG00000197134	19	22235254	22274282	6.59E-02	4.02E-01	6.50E-01	2.13E-01	3.51E-02	1.61E-01	0
IGFL4	ENSG00000204869	19	46543006	46580376	2.64E-02	1.42E-01	5.51E-02	1.75E-01	1.39E-02	1.61E-01	0
TRAPPC1	ENSG00000170043	17	7833663	7835441	2.14E-01	2.55E-02	3.56E-01	4.03E-01	1.63E-01	1.62E-01	0
GNL2	ENSG00000134697	1	38032417	38061536	1.04E-01	2.31E-01	2.33E-01	9.98E-02	2.46E-01	1.62E-01	0
SLC35E1	ENSG00000127526	19	16660642	16683193	6.64E-01	3.52E-01	7.15E-01	3.54E-01	5.23E-01	1.62E-01	0
GART	ENSG00000159131	21	34876238	34915797	1.04E-03	7.96E-01	6.66E-03	8.62E-02	5.15E-02	1.62E-01	0
ERBB3	ENSG00000065361	12	56473641	56497289	2.08E-01	1.26E-04	3.43E-01	5.66E-01	1.18E-01	1.62E-01	0
RELA	ENSG00000173039	11	65421067	65430565	5.56E-02	1.69E-01	5.02E-02	2.15E-01	2.30E-01	1.62E-01	0
EIF2AK3	ENSG00000172071	2	88856259	88927094	1.36E-01	8.47E-01	9.60E-02	4.30E-01	4.58E-02	1.62E-01	0
KIAA2013	ENSG00000116685	1	11979648	11986485	1.55E-01	9.01E-02	7.19E-02	6.03E-01	2.16E-01	1.62E-01	0

Gene	Ensembl ID	CHR	START	STOP	VHD_CD Pleiotropy P	VHD_GORD Pleiotropy P	VHD_IBD Pleiotropy P	VHD_IBS Pleiotropy P	VHD_PUD Pleiotropy P	VHD_UC Pleiotropy P	d
C16orf92	ENSG00000167194	16	30034655	30039057	8.61E-01	5.41E-01	2.30E-01	4.93E-01	5.45E-01	1.62E-01	0
DNAJB1	ENSG00000132002	19	14625582	14640582	1.60E-01	9.81E-02	6.07E-02	5.56E-02	1.07E-01	1.62E-01	0
KIF2C	ENSG00000142945	1	45205490	45233439	2.23E-01	9.58E-01	1.67E-01	7.59E-01	2.71E-01	1.62E-01	0
USP37	ENSG00000135913	2	219314974	219433084	4.81E-03	5.26E-02	9.52E-02	1.20E-01	7.09E-02	1.62E-01	0
NMUR2	ENSG00000132911	5	151771093	151812929	3.98E-02	3.73E-01	2.96E-02	4.28E-03	2.28E-02	1.62E-01	0
PLCH1	ENSG00000114805	3	155093369	155462856	6.82E-01	1.83E-01	5.17E-01	1.88E-01	2.00E-02	1.62E-01	0
ZNF852	ENSG00000178917	3	44540462	44552128	1.70E-01	6.12E-01	1.71E-01	9.14E-01	1.84E-01	1.62E-01	0
DEFA5	ENSG00000164816	8	6912831	6914256	2.82E-01	8.34E-01	1.87E-01	5.94E-01	6.88E-01	1.62E-01	0
ZC3H15	ENSG00000065548	2	187350883	187374090	8.66E-01	6.07E-01	3.56E-01	3.80E-01	4.96E-01	1.62E-01	0
RASA3	ENSG00000185989	13	114747194	114898086	1.14E-01	1.03E-01	6.89E-02	4.48E-01	1.75E-01	1.62E-01	0
C17orf89	ENSG00000224877	17	79213039	79215081	6.20E-01	9.42E-01	3.62E-01	2.47E-01	3.63E-01	1.62E-01	0
B3GNT1	ENSG00000174684	11	66112843	66115163	2.61E-01	3.46E-01	1.08E-01	1.17E-01	1.32E-01	1.62E-01	0
MEMO1	ENSG00000162959	2	32090129	32236299	1.54E-01	9.15E-02	9.62E-02	4.18E-01	4.87E-01	1.63E-01	0
HIST1H3J	ENSG00000197153	6	27858093	27860884	1.97E-01	6.09E-02	1.55E-01	3.49E-01	2.24E-01	1.63E-01	0
MUTYH	ENSG00000132781	1	45794835	45806142	4.18E-01	7.11E-01	5.44E-01	7.28E-01	3.31E-01	1.63E-01	0
NUF2	ENSG00000143228	1	163236366	163325554	3.91E-01	2.33E-01	1.03E-01	2.96E-01	2.31E-02	1.63E-01	0
CC2D2A	ENSG00000048342	4	15471489	15603180	3.56E-02	7.90E-02	3.22E-01	1.38E-01	5.85E-01	1.63E-01	0
BNC2	ENSG00000173068	9	16409501	16870841	4.42E-02	4.87E-01	6.68E-02	1.72E-01	5.35E-01	1.63E-01	0
VASP	ENSG00000125753	19	46009837	46030241	1.06E-01	6.14E-01	1.43E-01	4.99E-01	1.71E-01	1.63E-01	0
OPRL1	ENSG00000125510	20	62711526	62731996	6.63E-01	6.16E-01	2.53E-01	6.14E-01	3.41E-01	1.63E-01	0
METAP1D	ENSG00000172878	2	172864490	172947158	7.49E-02	4.03E-01	4.27E-01	3.30E-01	4.45E-01	1.63E-01	0
MYRIP	ENSG00000170011	3	39850405	40301812	6.92E-01	7.67E-01	4.37E-01	5.86E-01	8.87E-02	1.63E-01	0
ANKRD55	ENSG00000164512	5	55395507	55529186	2.43E-02	6.98E-01	8.42E-02	7.59E-01	1.90E-01	1.63E-01	0
GSG1	ENSG00000111305	12	13236494	13256619	3.32E-01	9.62E-01	3.40E-01	4.60E-01	2.20E-01	1.63E-01	0
LBH	ENSG00000213626	2	30454397	30546596	4.91E-01	5.70E-01	2.49E-01	1.38E-01	4.66E-01	1.63E-01	0
HHEX	ENSG00000152804	10	94447945	94455403	1.99E-01	1.60E-01	1.41E-01	7.32E-01	3.19E-01	1.63E-01	0
DR1	ENSG00000117505	1	93811445	93835055	5.58E-02	2.90E-01	3.92E-02	2.17E-01	2.69E-01	1.63E-01	0
ARID4B	ENSG00000054267	1	235294949	235491534	5.87E-02	3.34E-02	9.89E-02	7.79E-02	2.33E-02	1.63E-01	0
C14orf64	ENSG00000246223	14	98391947	98444461	3.41E-01	2.25E-01	1.28E-01	4.56E-01	4.92E-01	1.64E-01	0
FAT4	ENSG00000196159	4	126237554	126414087	5.90E-01	1.84E-01	3.49E-01	8.80E-02	2.46E-01	1.64E-01	0
IGFBP4	ENSG00000141753	17	38599702	38613983	7.08E-01	8.03E-01	2.30E-01	9.22E-01	7.12E-01	1.64E-01	0
RIT1	ENSG00000143622	1	155867599	155881195	4.70E-02	1.16E-01	1.73E-01	1.36E-02	1.34E-01	1.64E-01	0
PPARD	ENSG00000112033	6	35310335	35395968	6.21E-01	9.45E-01	4.00E-01	6.00E-01	5.17E-01	1.64E-01	0
CCDC66	ENSG00000180376	3	56591189	56655846	6.64E-02	1.41E-01	2.15E-01	3.69E-01	4.66E-01	1.64E-01	0
NLGN2	ENSG00000169992	17	7308193	7323179	8.30E-01	9.08E-01	2.98E-01	6.86E-01	5.12E-01	1.64E-01	0
ZNF484	ENSG00000127081	9	95607874	95640304	2.77E-02	5.62E-01	2.84E-02	3.52E-01	4.04E-01	1.64E-01	0
BCAN	ENSG00000132692	1	156611182	156629324	1.60E-01	9.86E-02	1.62E-01	3.08E-01	2.87E-02	1.64E-01	0
MORF4L1	ENSG00000185787	15	79102829	79190475	5.90E-01	2.41E-02	1.48E-01	3.03E-02	1.13E-02	1.64E-01	0
KLHL30	ENSG00000168427	2	239047363	239061588	6.57E-01	6.80E-01	2.16E-01	5.85E-01	4.16E-02	1.64E-01	0
TSSK6	ENSG00000178093	19	19623227	19626838	2.79E-01	2.41E-01	1.72E-01	5.40E-01	5.27E-01	1.64E-01	0
RRAS	ENSG00000126458	19	50138549	50143458	8.24E-01	5.18E-01	2.63E-01	9.64E-01	7.22E-01	1.64E-01	0
PLAGL2	ENSG00000126003	20	30780306	30795594	1.46E-01	7.07E-01	2.12E-01	3.90E-02	5.02E-01	1.64E-01	0
KRT20	ENSG00000171431	17	39032193	39041479	5.46E-02	6.68E-01	5.14E-02	5.80E-01	6.36E-01	1.64E-01	0
RPS10	ENSG00000124614	6	34385231	34393902	3.03E-01	3.56E-01	4.86E-01	1.59E-01	5.88E-01	1.64E-01	0
SLC9A3R1	ENSG00000109062	17	72744791	72765492	1.98E-01	6.36E-01	1.57E-01	1.39E-01	7.79E-01	1.64E-01	0
ANKRD13B	ENSG00000198720	17	27916787	27941779	4.90E-01	3.92E-01	4.12E-01	4.06E-01	6.12E-01	1.64E-01	0
SMPD4	ENSG00000136699	2	130908981	130940323	6.70E-01	3.52E-02	2.40E-01	6.22E-01	7.89E-03	1.65E-01	0
TM6SF2	ENSG00000213996	19	19375173	19384200	1.15E-01	8.11E-02	1.04E-01	3.24E-01	1.19E-01	1.65E-01	0
ERBB4	ENSG00000178568	2	212240446	213403565	1.47E-01	1.13E-01	5.40E-02	4.94E-01	2.47E-01	1.65E-01	0

Gene	Ensembl ID	CHR	START	STOP	VHD_CD Pleiotropy P	VHD_GORD Pleiotropy P	VHD_IBD Pleiotropy P	VHD_IBS Pleiotropy P	VHD_PUD Pleiotropy P	VHD_UC Pleiotropy P	d
FYTTD1	ENSG00000122068	3	197464050	197514467	7.77E-01	4.97E-01	5.42E-01	6.61E-01	3.23E-01	1.65E-01	0
COL10A1	ENSG00000123500	6	116440086	116479910	6.42E-01	3.17E-02	2.81E-01	3.30E-01	1.50E-01	1.65E-01	0
NDUFA13	ENSG00000186010	19	19626545	19644285	3.37E-01	1.31E-01	1.92E-01	5.87E-01	3.64E-01	1.65E-01	0
DUSP18	ENSG00000167065	22	31048038	31063877	4.18E-01	5.59E-01	3.58E-01	5.78E-01	8.78E-01	1.65E-01	0
CCDC129	ENSG00000180347	7	31553704	31698334	5.10E-01	9.69E-01	1.47E-01	6.84E-01	1.33E-01	1.65E-01	0
CXXC5	ENSG00000171604	5	139026884	139063467	6.04E-02	1.12E-01	8.51E-02	2.42E-01	1.56E-01	1.65E-01	0
GAST	ENSG00000184502	17	39868578	39872221	5.83E-03	4.88E-01	1.20E-02	1.70E-02	7.74E-04	1.65E-01	0
EIF5	ENSG00000100664	14	103799881	103811362	7.86E-02	2.73E-01	5.51E-02	6.54E-02	7.97E-02	1.65E-01	0
BLNK	ENSG00000095585	10	97951458	98031344	8.64E-02	6.26E-01	1.07E-01	6.46E-01	7.81E-01	1.65E-01	0
HNRNPA3	ENSG00000170144	2	178077291	178088686	4.58E-01	6.13E-01	4.83E-01	2.60E-01	4.07E-01	1.65E-01	0
PLA2G2A	ENSG00000188257	1	20301925	20306932	6.58E-02	1.62E-01	3.00E-02	4.98E-01	3.42E-03	1.65E-01	0
OXNAD1	ENSG00000154814	3	16306706	16391806	8.92E-02	8.50E-01	7.44E-02	6.49E-02	6.33E-01	1.65E-01	0
OASL	ENSG00000135114	12	121458095	121477045	5.81E-01	3.41E-01	5.36E-01	8.64E-01	3.15E-01	1.65E-01	0
HSD17B13	ENSG00000170509	4	88224941	88244058	2.26E-01	6.82E-01	2.43E-01	1.98E-01	2.98E-01	1.65E-01	0
CNTN1	ENSG00000018236	12	41086244	41466220	1.31E-01	8.21E-01	8.37E-02	2.78E-01	1.06E-01	1.65E-01	0
VWA3A	ENSG00000175267	16	22103859	22168287	3.08E-01	1.94E-01	7.95E-01	9.96E-02	1.72E-03	1.65E-01	0
CACNB1	ENSG00000067191	17	37329709	37353956	2.13E-01	3.27E-01	1.17E-01	5.33E-01	1.39E-01	1.66E-01	0
ANO10	ENSG00000160746	3	43396351	43733086	4.18E-01	4.99E-02	1.20E-01	6.51E-01	1.66E-01	1.66E-01	0
BZW1	ENSG00000082153	2	201675317	201688569	5.12E-01	3.30E-01	2.46E-01	2.84E-02	4.77E-01	1.66E-01	0
PRKAG3	ENSG00000115592	2	219687106	219696809	3.25E-01	5.92E-01	7.79E-01	5.00E-01	2.52E-01	1.66E-01	0
EIF3D	ENSG00000100353	22	36906897	36925483	8.43E-01	5.47E-01	4.79E-01	4.70E-01	7.87E-03	1.66E-01	0
C9orf92	ENSG00000205549	9	16203933	16276311	1.51E-01	5.86E-01	1.73E-01	2.81E-01	6.12E-01	1.66E-01	0
SLC16A10	ENSG00000112394	6	111408781	111552397	7.41E-01	6.97E-01	2.15E-01	1.69E-01	5.46E-01	1.66E-01	0
PRPF19	ENSG00000110107	11	60658202	60674060	2.89E-01	9.97E-01	1.44E-01	7.88E-01	6.77E-01	1.66E-01	0
RNF182	ENSG00000180537	6	13924677	13980533	7.03E-01	6.52E-01	5.58E-01	6.37E-01	5.56E-01	1.66E-01	0
RETNLB	ENSG00000163515	3	108462271	108476231	1.59E-01	9.32E-02	9.66E-02	6.55E-01	6.08E-01	1.66E-01	0
CYP8B1	ENSG00000180432	3	42897497	42917633	3.10E-01	3.59E-01	1.31E-01	7.25E-01	1.53E-01	1.66E-01	0
STYX	ENSG00000198252	14	53196898	53241716	5.36E-01	1.48E-01	4.35E-01	4.25E-01	3.81E-01	1.66E-01	0
ZNF486	ENSG00000256229	19	20278037	20311299	5.23E-01	2.27E-01	4.24E-01	9.16E-02	2.50E-01	1.66E-01	0
ENTPD4	ENSG00000197217	8	23243296	23315208	4.38E-01	9.56E-01	4.15E-01	9.00E-01	5.03E-01	1.66E-01	0
KPNA2	ENSG00000182481	17	66031635	66042958	9.82E-01	1.78E-01	3.36E-01	9.36E-01	2.82E-01	1.66E-01	0
KCNU1	ENSG00000215262	8	36641842	36793646	6.15E-01	2.03E-01	4.55E-01	2.11E-01	4.67E-01	1.66E-01	0
RPRD1A	ENSG00000141425	18	33564350	33647539	5.63E-01	2.35E-01	3.66E-01	1.30E-02	7.29E-01	1.67E-01	0
RIMS2	ENSG00000176406	8	104512976	105268322	2.92E-01	1.32E-02	2.14E-01	7.86E-01	3.97E-02	1.67E-01	0
OR4K14	ENSG00000169484	14	20482419	20483352	5.09E-01	3.07E-02	1.64E-01	3.12E-01	2.28E-01	1.67E-01	0
VPS37D	ENSG00000176428	7	73082155	73086442	2.59E-01	1.90E-01	2.58E-01	2.15E-01	2.02E-02	1.67E-01	0
CCR7	ENSG00000126353	17	38710021	38721724	1.70E-01	7.93E-01	1.13E-01	2.56E-01	9.93E-01	1.67E-01	0
SPATA7	ENSG00000042317	14	88851268	88936694	4.91E-01	2.36E-01	3.63E-01	4.10E-02	1.93E-01	1.67E-01	0
GRIA4	ENSG00000152578	11	105480721	105852819	2.33E-01	5.90E-01	2.09E-01	8.16E-01	3.66E-01	1.67E-01	0
WDFY2	ENSG00000139668	13	52158644	52336171	3.18E-01	4.59E-02	2.24E-01	3.84E-03	4.02E-01	1.67E-01	0
HOXD13	ENSG00000128714	2	176957619	176960666	2.56E-01	3.05E-01	1.22E-01	6.80E-01	9.59E-01	1.67E-01	0
DST	ENSG00000151914	6	56322785	56819426	8.92E-01	5.16E-01	3.88E-01	7.26E-01	7.82E-01	1.67E-01	0
DNMT1	ENSG00000130816	19	10244021	10341962	7.98E-02	6.89E-01	3.94E-02	1.83E-02	1.83E-01	1.67E-01	0
DEPDC5	ENSG00000100150	22	32149944	32303012	5.29E-01	4.89E-01	3.95E-01	4.59E-01	2.43E-01	1.67E-01	0
YWHAG	ENSG00000170027	7	75956116	75988348	1.35E-01	8.14E-01	3.01E-01	1.80E-01	4.04E-01	1.67E-01	0
SUN3	ENSG00000164744	7	48026745	48068716	3.30E-01	4.16E-01	1.57E-01	1.93E-01	8.84E-02	1.67E-01	0
TRA2B	ENSG00000136527	3	185633694	185655924	2.92E-01	3.70E-01	8.44E-01	2.73E-01	5.91E-01	1.67E-01	0
PGAP3	ENSG00000161395	17	37827375	37853050	9.31E-02	9.94E-01	7.60E-02	6.76E-01	7.70E-01	1.67E-01	0
TRIM42	ENSG00000155890	3	140396881	140419992	3.05E-01	3.98E-01	2.47E-01	3.07E-01	8.57E-01	1.67E-01	0

Gene	Ensembl ID	CHR	START	STOP	VHD_CD Pleiotropy P	VHD_GORD Pleiotropy P	VHD_IBD Pleiotropy P	VHD_IBS Pleiotropy P	VHD_PUD Pleiotropy P	VHD_UC Pleiotropy P	d
BTBD19	ENSG00000222009	1	45274154	45281257	7.19E-01	6.18E-01	3.46E-01	1.00E+00	4.57E-01	1.67E-01	0
CBLC	ENSG00000142273	19	45281126	45303891	7.04E-01	5.47E-01	5.18E-01	6.93E-01	5.29E-01	1.67E-01	0
CNNM1	ENSG00000119946	10	101088856	101154087	3.47E-01	2.64E-01	1.47E-01	1.38E-01	3.59E-01	1.68E-01	0
F3	ENSG00000117525	1	94994781	95007356	9.77E-02	6.92E-01	1.48E-01	2.36E-01	8.70E-02	1.68E-01	0
DCST1	ENSG00000163357	1	155006300	155023406	1.13E-02	1.10E-02	1.61E-02	6.13E-01	2.33E-01	1.68E-01	0
MED8	ENSG00000159479	1	43849588	43855479	8.56E-01	1.88E-01	5.92E-01	2.96E-01	6.33E-01	1.68E-01	0
GPBAR1	ENSG00000179921	2	219124219	219128582	2.09E-01	7.49E-01	1.07E-01	4.81E-01	8.35E-01	1.68E-01	0
NAA11	ENSG00000156269	4	80146848	80247204	2.93E-01	3.10E-01	4.96E-01	9.76E-02	1.06E-01	1.68E-01	0
POLH	ENSG00000170734	6	43543887	43586701	4.10E-01	2.78E-01	2.51E-01	5.51E-01	3.73E-01	1.68E-01	0
ZAR1	ENSG00000182223	4	48492269	48496406	2.24E-02	5.34E-01	4.45E-02	1.35E-01	2.77E-01	1.68E-01	0
PTPN21	ENSG00000070778	14	88932122	89021077	4.46E-01	2.64E-01	2.60E-01	8.24E-02	2.52E-01	1.68E-01	0
SPHK1	ENSG00000176170	17	74372665	74383941	6.63E-01	8.92E-01	5.91E-01	7.71E-01	6.50E-01	1.68E-01	0
VWA1	ENSG00000179403	1	1370241	1378262	2.31E-01	3.92E-01	4.01E-01	8.93E-02	5.19E-01	1.68E-01	0
TMEM179B	ENSG00000185475	11	62554887	62557877	3.18E-01	3.93E-01	2.17E-01	3.47E-01	6.96E-01	1.68E-01	0
OR8B12	ENSG00000170953	11	124412578	124413575	2.32E-01	2.30E-01	6.52E-01	1.40E-01	8.71E-02	1.68E-01	0
VWA3B	ENSG00000168658	2	98703579	98929762	4.84E-01	1.38E-01	1.44E-01	7.83E-01	5.06E-01	1.68E-01	0
DYDC1	ENSG00000170788	10	82095861	82116511	5.12E-02	5.94E-01	3.15E-02	2.64E-01	3.88E-01	1.69E-01	0
ZNF607	ENSG00000198182	19	38187264	38210691	1.56E-01	3.31E-01	1.21E-01	2.58E-01	2.26E-01	1.69E-01	0
P4HA3	ENSG00000149380	11	73946846	74022702	2.30E-01	8.64E-02	3.04E-01	7.75E-01	7.26E-01	1.69E-01	0
RGL4	ENSG00000159496	22	24030323	24041363	7.57E-02	7.82E-02	8.37E-02	4.31E-01	9.65E-02	1.69E-01	0
AP1S1	ENSG00000106367	7	100797678	100804877	4.20E-01	1.16E-01	1.76E-01	4.01E-01	5.26E-01	1.69E-01	0
VCPIP1	ENSG00000175073	8	67540722	67579452	2.55E-01	1.89E-01	1.24E-01	4.55E-01	8.44E-01	1.69E-01	0
ZNF567	ENSG00000189042	19	37178514	37218603	1.67E-01	1.23E-01	7.27E-01	1.56E-01	6.95E-01	1.69E-01	0
RGS9BP	ENSG00000186326	19	33166313	33169206	3.97E-01	9.17E-01	1.94E-01	3.73E-01	8.51E-01	1.69E-01	0
GANAB	ENSG00000089597	11	62392298	62414104	3.65E-01	2.48E-01	2.45E-01	3.07E-02	2.18E-01	1.69E-01	0
CKS1B	ENSG00000173207	1	154947129	154951725	1.12E-01	8.35E-01	1.12E-01	8.24E-01	9.07E-01	1.69E-01	0
SRCAP	ENSG00000080603	16	30709530	30755602	3.97E-02	6.94E-01	4.99E-02	3.63E-01	3.86E-01	1.69E-01	0
ZNF536	ENSG00000198597	19	30719197	31204445	6.79E-01	4.47E-01	3.29E-01	7.03E-01	2.19E-01	1.69E-01	0
TOP2A	ENSG00000131747	17	38544768	38574202	4.47E-01	9.12E-01	1.88E-01	9.59E-01	7.55E-01	1.69E-01	0
KIF26A	ENSG00000066735	14	104605060	104647231	7.64E-01	9.01E-01	4.08E-01	8.51E-01	7.92E-01	1.69E-01	0
RACGAP1	ENSG00000161800	12	50370706	50426919	6.10E-02	4.75E-01	1.08E-01	3.41E-01	2.57E-01	1.70E-01	0
C3orf52	ENSG00000114529	3	111805182	111849851	4.81E-02	4.26E-01	5.30E-02	2.52E-01	6.06E-01	1.70E-01	0
GOLT1B	ENSG00000111711	12	21654715	21671342	4.02E-01	3.01E-01	4.07E-01	9.95E-02	2.06E-01	1.70E-01	0
RBM8A	ENSG00000131795	1	145507598	145513536	4.53E-01	1.78E-01	1.84E-01	3.25E-01	8.99E-01	1.70E-01	0
FBXO6	ENSG00000116663	1	11724181	11734411	2.15E-01	5.05E-01	1.01E-01	1.05E-02	4.14E-02	1.70E-01	0
CABP5	ENSG00000105507	19	48533210	48547310	5.00E-01	4.37E-01	1.95E-01	5.91E-01	9.11E-01	1.70E-01	0
CORO6	ENSG00000167549	17	27941774	27949925	5.15E-01	2.07E-01	5.19E-01	2.26E-01	4.87E-01	1.70E-01	0
ACBD5	ENSG00000107897	10	27484146	27531059	5.81E-01	7.66E-01	3.10E-01	2.90E-01	6.29E-01	1.70E-01	0
VGLL4	ENSG00000144560	3	11597544	11762220	2.13E-03	6.63E-01	1.06E-02	4.19E-02	4.36E-01	1.70E-01	0
EML4	ENSG00000143924	2	42396490	42559688	1.32E-01	4.15E-01	2.06E-01	2.52E-01	4.39E-01	1.70E-01	0
PLEKHA1	ENSG00000107679	10	124134212	124191867	4.91E-01	7.53E-01	2.57E-01	6.87E-01	5.95E-01	1.70E-01	0
FAM101A	ENSG00000178882	12	124457788	124800570	1.59E-01	1.65E-01	8.77E-02	3.34E-01	3.98E-01	1.70E-01	0
RFX5	ENSG00000143390	1	151313116	151319833	5.48E-02	3.98E-01	7.39E-02	3.83E-01	1.42E-01	1.70E-01	0
RPAIN	ENSG00000129197	17	5322961	5336196	3.01E-01	1.65E-01	1.78E-01	2.83E-01	1.86E-01	1.70E-01	0
HN1L	ENSG00000206053	16	1728257	1752281	7.48E-01	3.76E-01	3.87E-01	3.80E-01	7.91E-01	1.70E-01	0
OSR2	ENSG00000164920	8	99956631	99964332	1.18E-01	3.70E-01	6.25E-02	3.90E-02	3.26E-01	1.70E-01	0
CKAP2L	ENSG00000169607	2	113493930	113522254	2.75E-01	2.23E-01	3.31E-01	1.37E-01	3.91E-01	1.70E-01	0
OR2B3	ENSG00000204703	6	29053985	29055090	4.18E-01	5.01E-01	4.25E-01	6.48E-01	5.86E-01	1.70E-01	0
ALS2	ENSG00000003393	2	202565277	202645912	1.78E-01	3.15E-01	4.33E-02	2.59E-01	6.99E-01	1.70E-01	0

Gene	Ensembl ID	CHR	START	STOP	VHD_CD Pleiotropy P	VHD_GORD Pleiotropy P	VHD_IBD Pleiotropy P	VHD_IBS Pleiotropy P	VHD_PUD Pleiotropy P	VHD_UC Pleiotropy P	d
TMTC4	ENSG00000125247	13	101256181	101327347	3.63E-01	9.58E-01	1.69E-01	7.08E-01	8.38E-01	1.70E-01	0
RBM22	ENSG00000086589	5	150070356	150080669	5.61E-01	4.09E-01	3.35E-01	9.48E-02	5.96E-01	1.70E-01	0
WFDC1	ENSG00000103175	16	84328252	84363450	7.23E-02	6.43E-01	2.59E-01	4.45E-01	1.47E-01	1.71E-01	0
LRRC42	ENSG00000116212	1	54411750	54433841	7.26E-01	6.57E-01	2.35E-01	8.58E-01	8.15E-02	1.71E-01	0
NQO2	ENSG00000124588	6	2988221	3019996	4.16E-01	2.57E-01	2.47E-01	7.55E-01	1.76E-01	1.71E-01	0
GLYAT	ENSG00000149124	11	58407899	58499447	6.40E-01	5.11E-01	3.17E-01	8.01E-01	3.63E-01	1.71E-01	0
EPHA1	ENSG00000146904	7	143087382	143105985	2.44E-01	1.67E-01	1.39E-01	6.11E-02	4.35E-01	1.71E-01	0
CLPSL2	ENSG00000196748	6	35744371	35747329	1.05E-01	1.26E-01	1.82E-01	1.26E-01	2.68E-02	1.71E-01	0
GJA1	ENSG00000152661	6	121756838	121770873	4.60E-01	2.10E-01	1.66E-01	1.91E-01	3.52E-01	1.71E-01	0
MICAL1	ENSG00000135596	6	109765265	109787171	7.17E-02	3.80E-01	9.37E-02	6.01E-01	4.08E-01	1.71E-01	0
HIST1H2AC	ENSG00000180573	6	26124373	26139344	2.23E-02	2.18E-02	3.36E-02	4.43E-01	5.07E-02	1.71E-01	0
CPAMD8	ENSG00000160111	19	17003758	17137625	1.19E-01	1.24E-01	2.14E-01	1.12E-01	1.47E-02	1.71E-01	0
KCNRG	ENSG00000198553	13	50589390	50595058	8.40E-01	8.22E-02	4.20E-01	2.62E-01	7.31E-01	1.71E-01	0
TAF10	ENSG00000166337	11	6627526	6633898	8.39E-02	3.48E-01	1.19E-01	1.69E-01	6.16E-02	1.72E-01	0
PPARGC1B	ENSG00000155846	5	149109861	149234585	1.05E-01	4.14E-01	1.66E-01	1.19E-01	2.68E-01	1.72E-01	0
C5orf64	ENSG00000178722	5	60933535	61047590	1.79E-02	4.19E-02	1.15E-01	3.00E-01	1.18E-01	1.72E-01	0
FAM13C	ENSG00000148541	10	61005890	61122939	2.25E-01	7.98E-02	1.41E-01	9.05E-02	2.45E-02	1.72E-01	0
STARD13	ENSG00000133121	13	33677272	33924767	5.17E-01	4.48E-01	1.70E-01	2.82E-01	7.44E-01	1.72E-01	0
WDR62	ENSG00000075702	19	36545783	36596008	3.37E-01	3.54E-01	1.29E-01	4.34E-01	7.73E-02	1.72E-01	0
ETV1	ENSG00000006468	7	13930853	14031050	5.87E-01	1.47E-01	4.71E-01	1.79E-01	6.48E-01	1.72E-01	0
LZTS1	ENSG00000061337	8	20103676	20161474	2.60E-01	3.23E-01	6.77E-02	1.94E-01	1.16E-01	1.72E-01	0
ARMC6	ENSG00000105676	19	19144384	19170563	2.34E-01	5.73E-01	1.87E-01	3.69E-01	5.52E-02	1.72E-01	0
F13B	ENSG00000143278	1	197008321	197036397	4.57E-01	9.88E-01	4.39E-01	4.20E-01	2.41E-01	1.72E-01	0
AP2M1	ENSG00000161203	3	183892477	183901879	3.00E-03	4.89E-02	1.02E-02	3.53E-01	1.20E-02	1.72E-01	0
UTF1	ENSG00000171794	10	135043778	135045062	3.88E-01	8.49E-01	1.88E-01	7.06E-01	1.87E-01	1.72E-01	0
PTP4A2	ENSG00000184007	1	32372022	32410457	4.92E-01	6.71E-01	3.81E-01	9.08E-01	3.91E-01	1.72E-01	0
AAED1	ENSG00000158122	9	99401859	99417585	3.15E-01	8.44E-01	2.12E-01	4.84E-02	9.30E-01	1.73E-01	0
GATAD1	ENSG00000157259	7	92076767	92088150	2.49E-01	5.69E-02	2.29E-01	6.27E-01	3.62E-01	1.73E-01	0
P2RX2	ENSG00000187848	12	133195366	133198972	4.08E-01	7.27E-01	7.06E-01	4.81E-01	2.93E-01	1.73E-01	0
FAT2	ENSG00000086570	5	150883654	150948505	5.15E-01	1.00E-01	3.26E-01	1.99E-01	2.69E-01	1.73E-01	0
CFL2	ENSG00000165410	14	35179593	35184029	3.05E-01	2.66E-01	4.75E-01	2.79E-01	4.82E-01	1.73E-01	0
PRSS22	ENSG00000005001	16	2902728	2908171	4.30E-01	1.68E-01	1.99E-01	2.52E-02	5.36E-01	1.73E-01	0
LRRC9	ENSG00000131951	14	60386431	60530277	6.42E-01	5.01E-01	2.57E-01	1.78E-01	5.77E-01	1.73E-01	0
NR2F6	ENSG00000160113	19	17342692	17356749	6.42E-01	3.44E-02	2.96E-01	2.48E-01	5.34E-01	1.73E-01	0
ARHGAP12	ENSG00000165322	10	32094365	32217742	1.55E-01	4.15E-01	7.63E-02	5.73E-01	7.70E-02	1.73E-01	0
ESPL1	ENSG00000135476	12	53662083	53687427	1.32E-01	3.40E-01	2.46E-01	5.07E-02	1.60E-01	1.73E-01	0
HYI	ENSG00000178922	1	43916824	43919660	9.30E-02	1.81E-01	9.53E-02	2.65E-03	6.21E-02	1.73E-01	0
MAP3K6	ENSG00000142733	1	27681675	27693383	4.94E-01	2.61E-01	2.06E-01	6.82E-01	4.89E-01	1.73E-01	0
SAMD5	ENSG00000203727	6	147830063	148058683	7.77E-01	2.77E-01	3.26E-01	9.47E-01	6.86E-01	1.73E-01	0
LDHA	ENSG00000134333	11	18415935	18429972	4.35E-02	6.35E-01	4.79E-02	1.84E-01	3.09E-01	1.73E-01	0
PEX11G	ENSG00000104883	19	7541761	7562335	7.18E-02	9.29E-02	1.07E-01	9.45E-02	7.84E-03	1.73E-01	0
SFXN2	ENSG00000156398	10	104474295	104503249	1.77E-01	7.96E-02	2.58E-01	8.49E-01	4.24E-01	1.74E-01	0
POP5	ENSG00000167272	12	121016567	121019201	4.82E-01	2.49E-01	2.60E-01	5.09E-01	6.11E-01	1.74E-01	0
CCDC65	ENSG00000139537	12	49297893	49325623	7.48E-01	9.21E-01	2.90E-01	6.11E-01	8.23E-01	1.74E-01	0
TRPM2	ENSG00000142185	21	45770046	45862964	5.18E-01	4.06E-01	2.36E-01	6.20E-01	8.21E-01	1.74E-01	0
SCN9A	ENSG00000169432	2	167051695	167232503	7.70E-02	3.27E-01	5.47E-02	1.55E-01	8.41E-02	1.74E-01	0
KRT24	ENSG00000167916	17	38854243	38860002	4.40E-01	6.45E-01	1.24E-01	8.19E-01	6.70E-01	1.74E-01	0
POM121L12	ENSG00000221900	7	53103349	53104617	7.23E-02	4.55E-01	1.23E-01	5.11E-02	3.97E-01	1.74E-01	0
PLA2G2F	ENSG00000158786	1	20465819	20476879	6.25E-01	4.26E-01	2.92E-01	1.64E-01	1.99E-01	1.74E-01	0

	Lung cancer	4	2.71E-04	HCG27, MSH5, NOTCH4, HLA-DRA
	HIV-1 control	2	3.56E-04	VARS, NOTCH4
	Oleic acid (18:1n-9) levels	2	3.56E-04	PPT2, EGFL8, AGPAT1
	Type 1 diabetes and autoimmune thyroid diseases	2	5.61E-04	PRRC2A, NOTCH4
VHD_IBS	Takayasu arteritis	2	1.34E-05	PRRC2A, MSH5
	Menopause (age at onset)	2	2.33E-05	PRRC2A, MSH5
VHD_UC	Ulcerative colitis	16	7.12E-23	PSORS1C1, CDSN, CCHCR1, TCF19, POU5F1, HCG27, ATF6B, PPT2, EGFL8, AGPAT1, RNF5, AGER, PBX2, GPSM3, NOTCH4, AMZ1, UBE2L3
	Inflammatory bowel disease	16	1.66E-19	PSORS1C1, CDSN, CCHCR1, TCF19, POU5F1, HCG27, ATF6B, PPT2, EGFL8, AGPAT1, RNF5, AGER, PBX2, GPSM3, NOTCH4, AMZ1, UBE2L3
	Autism spectrum disorder or schizophrenia	15	5.36E-19	PSORS1C1, CDSN, CCHCR1, TCF19, POU5F1, HCG27, ATF6B, PPT2, EGFL8, AGPAT1, RNF5, AGER, PBX2, GPSM3, NOTCH4, HLA-DPB1
	Prostate cancer	9	3.13E-11	CCHCR1, ATF6B, PPT2, EGFL8, AGPAT1, RNF5, AGER, PBX2, GPSM3, NOTCH4
	Stevens-Johnson syndrome and toxic epidermal necrolysis	4	4.08E-11	PSORS1C1, CCHCR1, TCF19, POU5F1
	Multiple myeloma	5	6.87E-11	PSORS1C1, CDSN, CCHCR1, TCF19, POU5F1
	idosis (non-Lofgren's syndrome without extrapulmonary manifestat	5	1.02E-10	ATF6B, AGER, GPSM3, NOTCH4, HLA-DPB1
	Idiopathic membranous nephropathy	4	3.83E-10	PSORS1C1, TCF19, PPT2, EGFL8, RNF5
	Takayasu arteritis	5	1.14E-08	PSORS1C1, CDSN, HCG27, ATF6B, NOTCH4
	Advanced glycation end-product levels	3	1.02E-07	RNF5, AGER, NOTCH4
	Chronic hepatitis B infection	3	4.39E-07	TCF19, NOTCH4, HLA-DPB1
	-induced agranulocytosis/granulocytopenia in treatment-resistant scl	3	1.85E-06	AGPAT1, PBX2, NOTCH4
	Hematology traits	3	2.42E-06	PSORS1C1, CDSN, CCHCR1
	Carotid plaque	2	1.19E-05	CFDP1, TMEM170A
	rug-induced Stevens-Johnson syndrome or toxic epidermal necrolys	2	2.51E-05	PSORS1C1, POU5F1
	Cold sores	2	2.51E-05	POU5F1, HCG27
	Systemic sclerosis	3	3.79E-05	PSORS1C1, NOTCH4, HLA-DPB1
	Hepatitis B	2	4.29E-05	HLA-DPB1, UBE2L3
	Epstein-Barr virus immune response (EBNA-1)	2	4.29E-05	PSORS1C1, NOTCH4
	White blood cell count	4	4.83E-05	PSORS1C1, CDSN, CCHCR1, HCG27
	Chronic obstructive pulmonary disease or coronary artery disease	2	1.25E-04	CFDP1, TMEM170A
	Oleic acid (18:1n-9) levels	2	1.42E-04	PPT2, EGFL8, AGPAT1
	HIV-1 control	2	1.42E-04	CDSN, NOTCH4
	Response to hepatitis B vaccine	2	1.81E-04	PSORS1C1, HLA-DPB1
	Blood protein levels	8	1.88E-04	PSORS1C1, CDSN, HCG27, ATF6B, AGER, GPSM3, NOTCH4, HLA-DPB1
	Type 1 diabetes and autoimmune thyroid diseases	2	2.25E-04	PSORS1C1, NOTCH4
	Chronic obstructive pulmonary disease-related biomarkers	2	2.99E-04	PSORS1C1, CCHCR1
	Psoriasis	3	4.06E-04	CCHCR1, HCG27, UBE2L3
	Chronic obstructive pulmonary disease	3	4.53E-04	AGER, AMZ1, CFDP1
	Night sleep phenotypes	4	6.14E-04	PSORS1C1, CCHCR1, TCF19, POU5F1
	Feeling nervous	2	8.65E-04	PSORS1C1, CCHCR1
	Automobile speeding propensity	2	8.65E-04	CDSN, ATF6B

	Lung cancer	4	2.71E-04	HCG27, MSH5, NOTCH4, HLA-DRA
	HIV-1 control	2	3.56E-04	VARS, NOTCH4
	Oleic acid (18:1n-9) levels	2	3.56E-04	PPT2, EGFL8, AGPAT1
	Type 1 diabetes and autoimmune thyroid diseases	2	5.61E-04	PRRC2A, NOTCH4
VHD_IBS	Takayasu arteritis	2	1.34E-05	PRRC2A, MSH5
	Menopause (age at onset)	2	2.33E-05	PRRC2A, MSH5
VHD_UC	Ulcerative colitis	16	7.12E-23	PSORS1C1, CDSN, CCHCR1, TCF19, POU5F1, HCG27, ATF6B, PPT2, EGFL8, AGPAT1, RNF5, AGER, PBX2, GPSM3, NOTCH4, AMZ1, UBE2L3
	Inflammatory bowel disease	16	1.66E-19	PSORS1C1, CDSN, CCHCR1, TCF19, POU5F1, HCG27, ATF6B, PPT2, EGFL8, AGPAT1, RNF5, AGER, PBX2, GPSM3, NOTCH4, AMZ1, UBE2L3
	Autism spectrum disorder or schizophrenia	15	5.36E-19	PSORS1C1, CDSN, CCHCR1, TCF19, POU5F1, HCG27, ATF6B, PPT2, EGFL8, AGPAT1, RNF5, AGER, PBX2, GPSM3, NOTCH4, HLA-DPB1
	Prostate cancer	9	3.13E-11	CCHCR1, ATF6B, PPT2, EGFL8, AGPAT1, RNF5, AGER, PBX2, GPSM3, NOTCH4
	Stevens-Johnson syndrome and toxic epidermal necrolysis	4	4.08E-11	PSORS1C1, CCHCR1, TCF19, POU5F1
	Multiple myeloma	5	6.87E-11	PSORS1C1, CDSN, CCHCR1, TCF19, POU5F1
	›idosis (non-Lofgren's syndrome without extrapulmonary manifesta	5	1.02E-10	ATF6B, AGER, GPSM3, NOTCH4, HLA-DPB1
	Idiopathic membranous nephropathy	4	3.83E-10	PSORS1C1, TCF19, PPT2, EGFL8, RNF5
	Takayasu arteritis	5	1.14E-08	PSORS1C1, CDSN, HCG27, ATF6B, NOTCH4
	Advanced glycation end-product levels	3	1.02E-07	RNF5, AGER, NOTCH4
	Chronic hepatitis B infection	3	4.39E-07	TCF19, NOTCH4, HLA-DPB1
	-induced agranulocytosis/granulocytopenia in treatment-resistant scl	3	1.85E-06	AGPAT1, PBX2, NOTCH4
	Hematology traits	3	2.42E-06	PSORS1C1, CDSN, CCHCR1
	Carotid plaque	2	1.19E-05	CFDP1, TMEM170A
	rug-induced Stevens-Johnson syndrome or toxic epidermal necrolys	2	2.51E-05	PSORS1C1, POU5F1
	Cold sores	2	2.51E-05	POU5F1, HCG27
	Systemic sclerosis	3	3.79E-05	PSORS1C1, NOTCH4, HLA-DPB1
	Hepatitis B	2	4.29E-05	HLA-DPB1, UBE2L3
	Epstein-Barr virus immune response (EBNA-1)	2	4.29E-05	PSORS1C1, NOTCH4
	White blood cell count	4	4.83E-05	PSORS1C1, CDSN, CCHCR1, HCG27
	Chronic obstructive pulmonary disease or coronary artery disease	2	1.25E-04	CFDP1, TMEM170A
	Oleic acid (18:1n-9) levels	2	1.42E-04	PPT2, EGFL8, AGPAT1
	HIV-1 control	2	1.42E-04	CDSN, NOTCH4
	Response to hepatitis B vaccine	2	1.81E-04	PSORS1C1, HLA-DPB1
	Blood protein levels	8	1.88E-04	PSORS1C1, CDSN, HCG27, ATF6B, AGER, GPSM3, NOTCH4, HLA-DPB1
	Type 1 diabetes and autoimmune thyroid diseases	2	2.25E-04	PSORS1C1, NOTCH4
	Chronic obstructive pulmonary disease-related biomarkers	2	2.99E-04	PSORS1C1, CCHCR1
	Psoriasis	3	4.06E-04	CCHCR1, HCG27, UBE2L3
	Chronic obstructive pulmonary disease	3	4.53E-04	AGER, AMZ1, CFDP1
	Night sleep phenotypes	4	6.14E-04	PSORS1C1, CCHCR1, TCF19, POU5F1
	Feeling nervous	2	8.65E-04	PSORS1C1, CCHCR1
	Automobile speeding propensity	2	8.65E-04	CDSN, ATF6B

	Lung cancer	4	2.71E-04	HCG27, MSH5, NOTCH4, HLA-DRA
	HIV-1 control	2	3.56E-04	VARS, NOTCH4
	Oleic acid (18:1n-9) levels	2	3.56E-04	PPT2, EGFL8, AGPAT1
	Type 1 diabetes and autoimmune thyroid diseases	2	5.61E-04	PRRC2A, NOTCH4
VHD_IBS	Takayasu arteritis	2	1.34E-05	PRRC2A, MSH5
	Menopause (age at onset)	2	2.33E-05	PRRC2A, MSH5
VHD_UC	Ulcerative colitis	16	7.12E-23	PSORS1C1, CDSN, CCHCR1, TCF19, POU5F1, HCG27, ATF6B, PPT2, EGFL8, AGPAT1, RNF5, AGER, PBX2, GPSM3, NOTCH4, AMZ1, UBE2L3
	Inflammatory bowel disease	16	1.66E-19	PSORS1C1, CDSN, CCHCR1, TCF19, POU5F1, HCG27, ATF6B, PPT2, EGFL8, AGPAT1, RNF5, AGER, PBX2, GPSM3, NOTCH4, AMZ1, UBE2L3
	Autism spectrum disorder or schizophrenia	15	5.36E-19	PSORS1C1, CDSN, CCHCR1, TCF19, POU5F1, HCG27, ATF6B, PPT2, EGFL8, AGPAT1, RNF5, AGER, PBX2, GPSM3, NOTCH4, HLA-DPB1
	Prostate cancer	9	3.13E-11	CCHCR1, ATF6B, PPT2, EGFL8, AGPAT1, RNF5, AGER, PBX2, GPSM3, NOTCH4
	Stevens-Johnson syndrome and toxic epidermal necrolysis	4	4.08E-11	PSORS1C1, CCHCR1, TCF19, POU5F1
	Multiple myeloma	5	6.87E-11	PSORS1C1, CDSN, CCHCR1, TCF19, POU5F1
	oidosis (non-Lofgren's syndrome without extrapulmonary manifestat	5	1.02E-10	ATF6B, AGER, GPSM3, NOTCH4, HLA-DPB1
	Idiopathic membranous nephropathy	4	3.83E-10	PSORS1C1, TCF19, PPT2, EGFL8, RNF5
	Takayasu arteritis	5	1.14E-08	PSORS1C1, CDSN, HCG27, ATF6B, NOTCH4
	Advanced glycation end-product levels	3	1.02E-07	RNF5, AGER, NOTCH4
	Chronic hepatitis B infection	3	4.39E-07	TCF19, NOTCH4, HLA-DPB1
	-induced agranulocytosis/granulocytopenia in treatment-resistant scl	3	1.85E-06	AGPAT1, PBX2, NOTCH4
	Hematology traits	3	2.42E-06	PSORS1C1, CDSN, CCHCR1
	Carotid plaque	2	1.19E-05	CFDP1, TMEM170A
	rug-induced Stevens-Johnson syndrome or toxic epidermal necrolys	2	2.51E-05	PSORS1C1, POU5F1
	Cold sores	2	2.51E-05	POU5F1, HCG27
	Systemic sclerosis	3	3.79E-05	PSORS1C1, NOTCH4, HLA-DPB1
	Hepatitis B	2	4.29E-05	HLA-DPB1, UBE2L3
	Epstein-Barr virus immune response (EBNA-1)	2	4.29E-05	PSORS1C1, NOTCH4
	White blood cell count	4	4.83E-05	PSORS1C1, CDSN, CCHCR1, HCG27
	Chronic obstructive pulmonary disease or coronary artery disease	2	1.25E-04	CFDP1, TMEM170A
	Oleic acid (18:1n-9) levels	2	1.42E-04	PPT2, EGFL8, AGPAT1
	HIV-1 control	2	1.42E-04	CDSN, NOTCH4
	Response to hepatitis B vaccine	2	1.81E-04	PSORS1C1, HLA-DPB1
	Blood protein levels	8	1.88E-04	PSORS1C1, CDSN, HCG27, ATF6B, AGER, GPSM3, NOTCH4, HLA-DPB1
	Type 1 diabetes and autoimmune thyroid diseases	2	2.25E-04	PSORS1C1, NOTCH4
	Chronic obstructive pulmonary disease-related biomarkers	2	2.99E-04	PSORS1C1, CCHCR1
	Psoriasis	3	4.06E-04	CCHCR1, HCG27, UBE2L3
	Chronic obstructive pulmonary disease	3	4.53E-04	AGER, AMZ1, CFDP1
	Night sleep phenotypes	4	6.14E-04	PSORS1C1, CCHCR1, TCF19, POU5F1
	Feeling nervous	2	8.65E-04	PSORS1C1, CCHCR1
	Automobile speeding propensity	2	8.65E-04	CDSN, ATF6B

	Lung cancer	4	2.71E-04	HCG27, MSH5, NOTCH4, HLA-DRA
	HIV-1 control	2	3.56E-04	VARS, NOTCH4
	Oleic acid (18:1n-9) levels	2	3.56E-04	PPT2, EGFL8, AGPAT1
	Type 1 diabetes and autoimmune thyroid diseases	2	5.61E-04	PRRC2A, NOTCH4
VHD_IBS	Takayasu arteritis	2	1.34E-05	PRRC2A, MSH5
	Menopause (age at onset)	2	2.33E-05	PRRC2A, MSH5
VHD_UC	Ulcerative colitis	16	7.12E-23	PSORS1C1, CDSN, CCHCR1, TCF19, POU5F1, HCG27, ATF6B, PPT2, EGFL8, AGPAT1, RNF5, AGER, PBX2, GPSM3, NOTCH4, AMZ1, UBE2L3
	Inflammatory bowel disease	16	1.66E-19	PSORS1C1, CDSN, CCHCR1, TCF19, POU5F1, HCG27, ATF6B, PPT2, EGFL8, AGPAT1, RNF5, AGER, PBX2, GPSM3, NOTCH4, AMZ1, UBE2L3
	Autism spectrum disorder or schizophrenia	15	5.36E-19	PSORS1C1, CDSN, CCHCR1, TCF19, POU5F1, HCG27, ATF6B, PPT2, EGFL8, AGPAT1, RNF5, AGER, PBX2, GPSM3, NOTCH4, HLA-DPB1
	Prostate cancer	9	3.13E-11	CCHCR1, ATF6B, PPT2, EGFL8, AGPAT1, RNF5, AGER, PBX2, GPSM3, NOTCH4
	Stevens-Johnson syndrome and toxic epidermal necrolysis	4	4.08E-11	PSORS1C1, CCHCR1, TCF19, POU5F1
	Multiple myeloma	5	6.87E-11	PSORS1C1, CDSN, CCHCR1, TCF19, POU5F1
	›idosis (non-Lofgren's syndrome without extrapulmonary manifesta	5	1.02E-10	ATF6B, AGER, GPSM3, NOTCH4, HLA-DPB1
	Idiopathic membranous nephropathy	4	3.83E-10	PSORS1C1, TCF19, PPT2, EGFL8, RNF5
	Takayasu arteritis	5	1.14E-08	PSORS1C1, CDSN, HCG27, ATF6B, NOTCH4
	Advanced glycation end-product levels	3	1.02E-07	RNF5, AGER, NOTCH4
	Chronic hepatitis B infection	3	4.39E-07	TCF19, NOTCH4, HLA-DPB1
	-induced agranulocytosis/granulocytopenia in treatment-resistant scl	3	1.85E-06	AGPAT1, PBX2, NOTCH4
	Hematology traits	3	2.42E-06	PSORS1C1, CDSN, CCHCR1
	Carotid plaque	2	1.19E-05	CFDP1, TMEM170A
	rug-induced Stevens-Johnson syndrome or toxic epidermal necrolys	2	2.51E-05	PSORS1C1, POU5F1
	Cold sores	2	2.51E-05	POU5F1, HCG27
	Systemic sclerosis	3	3.79E-05	PSORS1C1, NOTCH4, HLA-DPB1
	Hepatitis B	2	4.29E-05	HLA-DPB1, UBE2L3
	Epstein-Barr virus immune response (EBNA-1)	2	4.29E-05	PSORS1C1, NOTCH4
	White blood cell count	4	4.83E-05	PSORS1C1, CDSN, CCHCR1, HCG27
	Chronic obstructive pulmonary disease or coronary artery disease	2	1.25E-04	CFDP1, TMEM170A
	Oleic acid (18:1n-9) levels	2	1.42E-04	PPT2, EGFL8, AGPAT1
	HIV-1 control	2	1.42E-04	CDSN, NOTCH4
	Response to hepatitis B vaccine	2	1.81E-04	PSORS1C1, HLA-DPB1
	Blood protein levels	8	1.88E-04	PSORS1C1, CDSN, HCG27, ATF6B, AGER, GPSM3, NOTCH4, HLA-DPB1
	Type 1 diabetes and autoimmune thyroid diseases	2	2.25E-04	PSORS1C1, NOTCH4
	Chronic obstructive pulmonary disease-related biomarkers	2	2.99E-04	PSORS1C1, CCHCR1
	Psoriasis	3	4.06E-04	CCHCR1, HCG27, UBE2L3
	Chronic obstructive pulmonary disease	3	4.53E-04	AGER, AMZ1, CFDP1
	Night sleep phenotypes	4	6.14E-04	PSORS1C1, CCHCR1, TCF19, POU5F1
	Feeling nervous	2	8.65E-04	PSORS1C1, CCHCR1
	Automobile speeding propensity	2	8.65E-04	CDSN, ATF6B

	Lung cancer	4	2.71E-04	HCG27, MSH5, NOTCH4, HLA-DRA
	HIV-1 control	2	3.56E-04	VARS, NOTCH4
	Oleic acid (18:1n-9) levels	2	3.56E-04	PPT2, EGFL8, AGPAT1
	Type 1 diabetes and autoimmune thyroid diseases	2	5.61E-04	PRRC2A, NOTCH4
VHD_IBS	Takayasu arteritis	2	1.34E-05	PRRC2A, MSH5
	Menopause (age at onset)	2	2.33E-05	PRRC2A, MSH5
VHD_UC	Ulcerative colitis	16	7.12E-23	PSORS1C1, CDSN, CCHCR1, TCF19, POU5F1, HCG27, ATF6B, PPT2, EGFL8, AGPAT1, RNF5, AGER, PBX2, GPSM3, NOTCH4, AMZ1, UBE2L3
	Inflammatory bowel disease	16	1.66E-19	PSORS1C1, CDSN, CCHCR1, TCF19, POU5F1, HCG27, ATF6B, PPT2, EGFL8, AGPAT1, RNF5, AGER, PBX2, GPSM3, NOTCH4, AMZ1, UBE2L3
	Autism spectrum disorder or schizophrenia	15	5.36E-19	PSORS1C1, CDSN, CCHCR1, TCF19, POU5F1, HCG27, ATF6B, PPT2, EGFL8, AGPAT1, RNF5, AGER, PBX2, GPSM3, NOTCH4, HLA-DPB1
	Prostate cancer	9	3.13E-11	CCHCR1, ATF6B, PPT2, EGFL8, AGPAT1, RNF5, AGER, PBX2, GPSM3, NOTCH4
	Stevens-Johnson syndrome and toxic epidermal necrolysis	4	4.08E-11	PSORS1C1, CCHCR1, TCF19, POU5F1
	Multiple myeloma	5	6.87E-11	PSORS1C1, CDSN, CCHCR1, TCF19, POU5F1
	idosis (non-Lofgren's syndrome without extrapulmonary manifesta	5	1.02E-10	ATF6B, AGER, GPSM3, NOTCH4, HLA-DPB1
	Idiopathic membranous nephropathy	4	3.83E-10	PSORS1C1, TCF19, PPT2, EGFL8, RNF5
	Takayasu arteritis	5	1.14E-08	PSORS1C1, CDSN, HCG27, ATF6B, NOTCH4
	Advanced glycation end-product levels	3	1.02E-07	RNF5, AGER, NOTCH4
	Chronic hepatitis B infection	3	4.39E-07	TCF19, NOTCH4, HLA-DPB1
	induced agranulocytosis/granulocytopenia in treatment-resistant sc	3	1.85E-06	AGPAT1, PBX2, NOTCH4
	Hematology traits	3	2.42E-06	PSORS1C1, CDSN, CCHCR1
	Carotid plaque	2	1.19E-05	CFDP1, TMEM170A
	rug-induced Stevens-Johnson syndrome or toxic epidermal necrolys	2	2.51E-05	PSORS1C1, POU5F1
	Cold sores	2	2.51E-05	POU5F1, HCG27
	Systemic sclerosis	3	3.79E-05	PSORS1C1, NOTCH4, HLA-DPB1
	Hepatitis B	2	4.29E-05	HLA-DPB1, UBE2L3
	Epstein-Barr virus immune response (EBNA-1)	2	4.29E-05	PSORS1C1, NOTCH4
	White blood cell count	4	4.83E-05	PSORS1C1, CDSN, CCHCR1, HCG27
	Chronic obstructive pulmonary disease or coronary artery disease	2	1.25E-04	CFDP1, TMEM170A
	Oleic acid (18:1n-9) levels	2	1.42E-04	PPT2, EGFL8, AGPAT1
	HIV-1 control	2	1.42E-04	CDSN, NOTCH4
	Response to hepatitis B vaccine	2	1.81E-04	PSORS1C1, HLA-DPB1
	Blood protein levels	8	1.88E-04	PSORS1C1, CDSN, HCG27, ATF6B, AGER, GPSM3, NOTCH4, HLA-DPB1
	Type 1 diabetes and autoimmune thyroid diseases	2	2.25E-04	PSORS1C1, NOTCH4
	Chronic obstructive pulmonary disease-related biomarkers	2	2.99E-04	PSORS1C1, CCHCR1
	Psoriasis	3	4.06E-04	CCHCR1, HCG27, UBE2L3
	Chronic obstructive pulmonary disease	3	4.53E-04	AGER, AMZ1, CFDP1
	Night sleep phenotypes	4	6.14E-04	PSORS1C1, CCHCR1, TCF19, POU5F1
	Feeling nervous	2	8.65E-04	PSORS1C1, CCHCR1
	Automobile speeding propensity	2	8.65E-04	CDSN, ATF6B

	Lung cancer	4	2.71E-04	HCG27, MSH5, NOTCH4, HLA-DRA
	HIV-1 control	2	3.56E-04	VARS, NOTCH4
	Oleic acid (18:1n-9) levels	2	3.56E-04	PPT2, EGFL8, AGPAT1
	Type 1 diabetes and autoimmune thyroid diseases	2	5.61E-04	PRRC2A, NOTCH4
VHD_IBS	Takayasu arteritis	2	1.34E-05	PRRC2A, MSH5
	Menopause (age at onset)	2	2.33E-05	PRRC2A, MSH5
VHD_UC	Ulcerative colitis	16	7.12E-23	PSORS1C1, CDSN, CCHCR1, TCF19, POU5F1, HCG27, ATF6B, PPT2, EGFL8, AGPAT1, RNF5, AGER, PBX2, GPSM3, NOTCH4, AMZ1, UBE2L3
	Inflammatory bowel disease	16	1.66E-19	PSORS1C1, CDSN, CCHCR1, TCF19, POU5F1, HCG27, ATF6B, PPT2, EGFL8, AGPAT1, RNF5, AGER, PBX2, GPSM3, NOTCH4, AMZ1, UBE2L3
	Autism spectrum disorder or schizophrenia	15	5.36E-19	PSORS1C1, CDSN, CCHCR1, TCF19, POU5F1, HCG27, ATF6B, PPT2, EGFL8, AGPAT1, RNF5, AGER, PBX2, GPSM3, NOTCH4, HLA-DPB1
	Prostate cancer	9	3.13E-11	CCHCR1, ATF6B, PPT2, EGFL8, AGPAT1, RNF5, AGER, PBX2, GPSM3, NOTCH4
	Stevens-Johnson syndrome and toxic epidermal necrolysis	4	4.08E-11	PSORS1C1, CCHCR1, TCF19, POU5F1
	Multiple myeloma	5	6.87E-11	PSORS1C1, CDSN, CCHCR1, TCF19, POU5F1
	idosis (non-Lofgren's syndrome without extrapulmonary manifestat	5	1.02E-10	ATF6B, AGER, GPSM3, NOTCH4, HLA-DPB1
	Idiopathic membranous nephropathy	4	3.83E-10	PSORS1C1, TCF19, PPT2, EGFL8, RNF5
	Takayasu arteritis	5	1.14E-08	PSORS1C1, CDSN, HCG27, ATF6B, NOTCH4
	Advanced glycation end-product levels	3	1.02E-07	RNF5, AGER, NOTCH4
	Chronic hepatitis B infection	3	4.39E-07	TCF19, NOTCH4, HLA-DPB1
	-induced agranulocytosis/granulocytopenia in treatment-resistant scl	3	1.85E-06	AGPAT1, PBX2, NOTCH4
	Hematology traits	3	2.42E-06	PSORS1C1, CDSN, CCHCR1
	Carotid plaque	2	1.19E-05	CFDP1, TMEM170A
	rug-induced Stevens-Johnson syndrome or toxic epidermal necrolys	2	2.51E-05	PSORS1C1, POU5F1
	Cold sores	2	2.51E-05	POU5F1, HCG27
	Systemic sclerosis	3	3.79E-05	PSORS1C1, NOTCH4, HLA-DPB1
	Hepatitis B	2	4.29E-05	HLA-DPB1, UBE2L3
	Epstein-Barr virus immune response (EBNA-1)	2	4.29E-05	PSORS1C1, NOTCH4
	White blood cell count	4	4.83E-05	PSORS1C1, CDSN, CCHCR1, HCG27
	Chronic obstructive pulmonary disease or coronary artery disease	2	1.25E-04	CFDP1, TMEM170A
	Oleic acid (18:1n-9) levels	2	1.42E-04	PPT2, EGFL8, AGPAT1
	HIV-1 control	2	1.42E-04	CDSN, NOTCH4
	Response to hepatitis B vaccine	2	1.81E-04	PSORS1C1, HLA-DPB1
	Blood protein levels	8	1.88E-04	PSORS1C1, CDSN, HCG27, ATF6B, AGER, GPSM3, NOTCH4, HLA-DPB1
	Type 1 diabetes and autoimmune thyroid diseases	2	2.25E-04	PSORS1C1, NOTCH4
	Chronic obstructive pulmonary disease-related biomarkers	2	2.99E-04	PSORS1C1, CCHCR1
	Psoriasis	3	4.06E-04	CCHCR1, HCG27, UBE2L3
	Chronic obstructive pulmonary disease	3	4.53E-04	AGER, AMZ1, CFDP1
	Night sleep phenotypes	4	6.14E-04	PSORS1C1, CCHCR1, TCF19, POU5F1
	Feeling nervous	2	8.65E-04	PSORS1C1, CCHCR1
	Automobile speeding propensity	2	8.65E-04	CDSN, ATF6B

	Lung cancer	4	2.71E-04	HCG27, MSH5, NOTCH4, HLA-DRA
	HIV-1 control	2	3.56E-04	VARS, NOTCH4
	Oleic acid (18:1n-9) levels	2	3.56E-04	PPT2, EGFL8, AGPAT1
	Type 1 diabetes and autoimmune thyroid diseases	2	5.61E-04	PRRC2A, NOTCH4
VHD_IBS	Takayasu arteritis	2	1.34E-05	PRRC2A, MSH5
	Menopause (age at onset)	2	2.33E-05	PRRC2A, MSH5
VHD_UC	Ulcerative colitis	16	7.12E-23	PSORS1C1, CDSN, CCHCR1, TCF19, POU5F1, HCG27, ATF6B, PPT2, EGFL8, AGPAT1, RNF5, AGER, PBX2, GPSM3, NOTCH4, AMZ1, UBE2L3
	Inflammatory bowel disease	16	1.66E-19	PSORS1C1, CDSN, CCHCR1, TCF19, POU5F1, HCG27, ATF6B, PPT2, EGFL8, AGPAT1, RNF5, AGER, PBX2, GPSM3, NOTCH4, AMZ1, UBE2L3
	Autism spectrum disorder or schizophrenia	15	5.36E-19	PSORS1C1, CDSN, CCHCR1, TCF19, POU5F1, HCG27, ATF6B, PPT2, EGFL8, AGPAT1, RNF5, AGER, PBX2, GPSM3, NOTCH4, HLA-DPB1
	Prostate cancer	9	3.13E-11	CCHCR1, ATF6B, PPT2, EGFL8, AGPAT1, RNF5, AGER, PBX2, GPSM3, NOTCH4
	Stevens-Johnson syndrome and toxic epidermal necrolysis	4	4.08E-11	PSORS1C1, CCHCR1, TCF19, POU5F1
	Multiple myeloma	5	6.87E-11	PSORS1C1, CDSN, CCHCR1, TCF19, POU5F1
	oidosis (non-Lofgren's syndrome without extrapulmonary manifesta	5	1.02E-10	ATF6B, AGER, GPSM3, NOTCH4, HLA-DPB1
	Idiopathic membranous nephropathy	4	3.83E-10	PSORS1C1, TCF19, PPT2, EGFL8, RNF5
	Takayasu arteritis	5	1.14E-08	PSORS1C1, CDSN, HCG27, ATF6B, NOTCH4
	Advanced glycation end-product levels	3	1.02E-07	RNF5, AGER, NOTCH4
	Chronic hepatitis B infection	3	4.39E-07	TCF19, NOTCH4, HLA-DPB1
	-induced agranulocytosis/granulocytopenia in treatment-resistant scl	3	1.85E-06	AGPAT1, PBX2, NOTCH4
	Hematology traits	3	2.42E-06	PSORS1C1, CDSN, CCHCR1
	Carotid plaque	2	1.19E-05	CFDP1, TMEM170A
	rug-induced Stevens-Johnson syndrome or toxic epidermal necrolys	2	2.51E-05	PSORS1C1, POU5F1
	Cold sores	2	2.51E-05	POU5F1, HCG27
	Systemic sclerosis	3	3.79E-05	PSORS1C1, NOTCH4, HLA-DPB1
	Hepatitis B	2	4.29E-05	HLA-DPB1, UBE2L3
	Epstein-Barr virus immune response (EBNA-1)	2	4.29E-05	PSORS1C1, NOTCH4
	White blood cell count	4	4.83E-05	PSORS1C1, CDSN, CCHCR1, HCG27
	Chronic obstructive pulmonary disease or coronary artery disease	2	1.25E-04	CFDP1, TMEM170A
	Oleic acid (18:1n-9) levels	2	1.42E-04	PPT2, EGFL8, AGPAT1
	HIV-1 control	2	1.42E-04	CDSN, NOTCH4
	Response to hepatitis B vaccine	2	1.81E-04	PSORS1C1, HLA-DPB1
	Blood protein levels	8	1.88E-04	PSORS1C1, CDSN, HCG27, ATF6B, AGER, GPSM3, NOTCH4, HLA-DPB1
	Type 1 diabetes and autoimmune thyroid diseases	2	2.25E-04	PSORS1C1, NOTCH4
	Chronic obstructive pulmonary disease-related biomarkers	2	2.99E-04	PSORS1C1, CCHCR1
	Psoriasis	3	4.06E-04	CCHCR1, HCG27, UBE2L3
	Chronic obstructive pulmonary disease	3	4.53E-04	AGER, AMZ1, CFDP1
	Night sleep phenotypes	4	6.14E-04	PSORS1C1, CCHCR1, TCF19, POU5F1
	Feeling nervous	2	8.65E-04	PSORS1C1, CCHCR1
	Automobile speeding propensity	2	8.65E-04	CDSN, ATF6B

	Lung cancer	4	2.71E-04	HCG27, MSH5, NOTCH4, HLA-DRA
	HIV-1 control	2	3.56E-04	VARS, NOTCH4
	Oleic acid (18:1n-9) levels	2	3.56E-04	PPT2, EGFL8, AGPAT1
	Type 1 diabetes and autoimmune thyroid diseases	2	5.61E-04	PRRC2A, NOTCH4
VHD_IBS	Takayasu arteritis	2	1.34E-05	PRRC2A, MSH5
	Menopause (age at onset)	2	2.33E-05	PRRC2A, MSH5
VHD_UC	Ulcerative colitis	16	7.12E-23	PSORS1C1, CDSN, CCHCR1, TCF19, POU5F1, HCG27, ATF6B, PPT2, EGFL8, AGPAT1, RNF5, AGER, PBX2, GPSM3, NOTCH4, AMZ1, UBE2L3
	Inflammatory bowel disease	16	1.66E-19	PSORS1C1, CDSN, CCHCR1, TCF19, POU5F1, HCG27, ATF6B, PPT2, EGFL8, AGPAT1, RNF5, AGER, PBX2, GPSM3, NOTCH4, AMZ1, UBE2L3
	Autism spectrum disorder or schizophrenia	15	5.36E-19	PSORS1C1, CDSN, CCHCR1, TCF19, POU5F1, HCG27, ATF6B, PPT2, EGFL8, AGPAT1, RNF5, AGER, PBX2, GPSM3, NOTCH4, HLA-DPB1
	Prostate cancer	9	3.13E-11	CCHCR1, ATF6B, PPT2, EGFL8, AGPAT1, RNF5, AGER, PBX2, GPSM3, NOTCH4
	Stevens-Johnson syndrome and toxic epidermal necrolysis	4	4.08E-11	PSORS1C1, CCHCR1, TCF19, POU5F1
	Multiple myeloma	5	6.87E-11	PSORS1C1, CDSN, CCHCR1, TCF19, POU5F1
	oidosis (non-Lofgren's syndrome without extrapulmonary manifestat	5	1.02E-10	ATF6B, AGER, GPSM3, NOTCH4, HLA-DPB1
	Idiopathic membranous nephropathy	4	3.83E-10	PSORS1C1, TCF19, PPT2, EGFL8, RNF5
	Takayasu arteritis	5	1.14E-08	PSORS1C1, CDSN, HCG27, ATF6B, NOTCH4
	Advanced glycation end-product levels	3	1.02E-07	RNF5, AGER, NOTCH4
	Chronic hepatitis B infection	3	4.39E-07	TCF19, NOTCH4, HLA-DPB1
	-induced agranulocytosis/granulocytopenia in treatment-resistant scl	3	1.85E-06	AGPAT1, PBX2, NOTCH4
	Hematology traits	3	2.42E-06	PSORS1C1, CDSN, CCHCR1
	Carotid plaque	2	1.19E-05	CFDP1, TMEM170A
	rug-induced Stevens-Johnson syndrome or toxic epidermal necrolys	2	2.51E-05	PSORS1C1, POU5F1
	Cold sores	2	2.51E-05	POU5F1, HCG27
	Systemic sclerosis	3	3.79E-05	PSORS1C1, NOTCH4, HLA-DPB1
	Hepatitis B	2	4.29E-05	HLA-DPB1, UBE2L3
	Epstein-Barr virus immune response (EBNA-1)	2	4.29E-05	PSORS1C1, NOTCH4
	White blood cell count	4	4.83E-05	PSORS1C1, CDSN, CCHCR1, HCG27
	Chronic obstructive pulmonary disease or coronary artery disease	2	1.25E-04	CFDP1, TMEM170A
	Oleic acid (18:1n-9) levels	2	1.42E-04	PPT2, EGFL8, AGPAT1
	HIV-1 control	2	1.42E-04	CDSN, NOTCH4
	Response to hepatitis B vaccine	2	1.81E-04	PSORS1C1, HLA-DPB1
	Blood protein levels	8	1.88E-04	PSORS1C1, CDSN, HCG27, ATF6B, AGER, GPSM3, NOTCH4, HLA-DPB1
	Type 1 diabetes and autoimmune thyroid diseases	2	2.25E-04	PSORS1C1, NOTCH4
	Chronic obstructive pulmonary disease-related biomarkers	2	2.99E-04	PSORS1C1, CCHCR1
	Psoriasis	3	4.06E-04	CCHCR1, HCG27, UBE2L3
	Chronic obstructive pulmonary disease	3	4.53E-04	AGER, AMZ1, CFDP1
	Night sleep phenotypes	4	6.14E-04	PSORS1C1, CCHCR1, TCF19, POU5F1
	Feeling nervous	2	8.65E-04	PSORS1C1, CCHCR1
	Automobile speeding propensity	2	8.65E-04	CDSN, ATF6B

	Lung cancer	4	2.71E-04	HCG27, MSH5, NOTCH4, HLA-DRA
	HIV-1 control	2	3.56E-04	VARS, NOTCH4
	Oleic acid (18:1n-9) levels	2	3.56E-04	PPT2, EGFL8, AGPAT1
	Type 1 diabetes and autoimmune thyroid diseases	2	5.61E-04	PRRC2A, NOTCH4
VHD_IBS	Takayasu arteritis	2	1.34E-05	PRRC2A, MSH5
	Menopause (age at onset)	2	2.33E-05	PRRC2A, MSH5
VHD_UC	Ulcerative colitis	16	7.12E-23	PSORS1C1, CDSN, CCHCR1, TCF19, POU5F1, HCG27, ATF6B, PPT2, EGFL8, AGPAT1, RNF5, AGER, PBX2, GPSM3, NOTCH4, AMZ1, UBE2L3
	Inflammatory bowel disease	16	1.66E-19	PSORS1C1, CDSN, CCHCR1, TCF19, POU5F1, HCG27, ATF6B, PPT2, EGFL8, AGPAT1, RNF5, AGER, PBX2, GPSM3, NOTCH4, AMZ1, UBE2L3
	Autism spectrum disorder or schizophrenia	15	5.36E-19	PSORS1C1, CDSN, CCHCR1, TCF19, POU5F1, HCG27, ATF6B, PPT2, EGFL8, AGPAT1, RNF5, AGER, PBX2, GPSM3, NOTCH4, HLA-DPB1
	Prostate cancer	9	3.13E-11	CCHCR1, ATF6B, PPT2, EGFL8, AGPAT1, RNF5, AGER, PBX2, GPSM3, NOTCH4
	Stevens-Johnson syndrome and toxic epidermal necrolysis	4	4.08E-11	PSORS1C1, CCHCR1, TCF19, POU5F1
	Multiple myeloma	5	6.87E-11	PSORS1C1, CDSN, CCHCR1, TCF19, POU5F1
	›idosis (non-Lofgren's syndrome without extrapulmonary manifesta	5	1.02E-10	ATF6B, AGER, GPSM3, NOTCH4, HLA-DPB1
	Idiopathic membranous nephropathy	4	3.83E-10	PSORS1C1, TCF19, PPT2, EGFL8, RNF5
	Takayasu arteritis	5	1.14E-08	PSORS1C1, CDSN, HCG27, ATF6B, NOTCH4
	Advanced glycation end-product levels	3	1.02E-07	RNF5, AGER, NOTCH4
	Chronic hepatitis B infection	3	4.39E-07	TCF19, NOTCH4, HLA-DPB1
	-induced agranulocytosis/granulocytopenia in treatment-resistant scl	3	1.85E-06	AGPAT1, PBX2, NOTCH4
	Hematology traits	3	2.42E-06	PSORS1C1, CDSN, CCHCR1
	Carotid plaque	2	1.19E-05	CFDP1, TMEM170A
	rug-induced Stevens-Johnson syndrome or toxic epidermal necrolys	2	2.51E-05	PSORS1C1, POU5F1
	Cold sores	2	2.51E-05	POU5F1, HCG27
	Systemic sclerosis	3	3.79E-05	PSORS1C1, NOTCH4, HLA-DPB1
	Hepatitis B	2	4.29E-05	HLA-DPB1, UBE2L3
	Epstein-Barr virus immune response (EBNA-1)	2	4.29E-05	PSORS1C1, NOTCH4
	White blood cell count	4	4.83E-05	PSORS1C1, CDSN, CCHCR1, HCG27
	Chronic obstructive pulmonary disease or coronary artery disease	2	1.25E-04	CFDP1, TMEM170A
	Oleic acid (18:1n-9) levels	2	1.42E-04	PPT2, EGFL8, AGPAT1
	HIV-1 control	2	1.42E-04	CDSN, NOTCH4
	Response to hepatitis B vaccine	2	1.81E-04	PSORS1C1, HLA-DPB1
	Blood protein levels	8	1.88E-04	PSORS1C1, CDSN, HCG27, ATF6B, AGER, GPSM3, NOTCH4, HLA-DPB1
	Type 1 diabetes and autoimmune thyroid diseases	2	2.25E-04	PSORS1C1, NOTCH4
	Chronic obstructive pulmonary disease-related biomarkers	2	2.99E-04	PSORS1C1, CCHCR1
	Psoriasis	3	4.06E-04	CCHCR1, HCG27, UBE2L3
	Chronic obstructive pulmonary disease	3	4.53E-04	AGER, AMZ1, CFDP1
	Night sleep phenotypes	4	6.14E-04	PSORS1C1, CCHCR1, TCF19, POU5F1
	Feeling nervous	2	8.65E-04	PSORS1C1, CCHCR1
	Automobile speeding propensity	2	8.65E-04	CDSN, ATF6B

	Lung cancer	4	2.71E-04	HCG27, MSH5, NOTCH4, HLA-DRA
	HIV-1 control	2	3.56E-04	VARS, NOTCH4
	Oleic acid (18:1n-9) levels	2	3.56E-04	PPT2, EGFL8, AGPAT1
	Type 1 diabetes and autoimmune thyroid diseases	2	5.61E-04	PRRC2A, NOTCH4
VHD_IBS	Takayasu arteritis	2	1.34E-05	PRRC2A, MSH5
	Menopause (age at onset)	2	2.33E-05	PRRC2A, MSH5
VHD_UC	Ulcerative colitis	16	7.12E-23	PSORS1C1, CDSN, CCHCR1, TCF19, POU5F1, HCG27, ATF6B, PPT2, EGFL8, AGPAT1, RNF5, AGER, PBX2, GPSM3, NOTCH4, AMZ1, UBE2L3
	Inflammatory bowel disease	16	1.66E-19	PSORS1C1, CDSN, CCHCR1, TCF19, POU5F1, HCG27, ATF6B, PPT2, EGFL8, AGPAT1, RNF5, AGER, PBX2, GPSM3, NOTCH4, AMZ1, UBE2L3
	Autism spectrum disorder or schizophrenia	15	5.36E-19	PSORS1C1, CDSN, CCHCR1, TCF19, POU5F1, HCG27, ATF6B, PPT2, EGFL8, AGPAT1, RNF5, AGER, PBX2, GPSM3, NOTCH4, HLA-DPB1
	Prostate cancer	9	3.13E-11	CCHCR1, ATF6B, PPT2, EGFL8, AGPAT1, RNF5, AGER, PBX2, GPSM3, NOTCH4
	Stevens-Johnson syndrome and toxic epidermal necrolysis	4	4.08E-11	PSORS1C1, CCHCR1, TCF19, POU5F1
	Multiple myeloma	5	6.87E-11	PSORS1C1, CDSN, CCHCR1, TCF19, POU5F1
	idosis (non-Lofgren's syndrome without extrapulmonary manifesta	5	1.02E-10	ATF6B, AGER, GPSM3, NOTCH4, HLA-DPB1
	Idiopathic membranous nephropathy	4	3.83E-10	PSORS1C1, TCF19, PPT2, EGFL8, RNF5
	Takayasu arteritis	5	1.14E-08	PSORS1C1, CDSN, HCG27, ATF6B, NOTCH4
	Advanced glycation end-product levels	3	1.02E-07	RNF5, AGER, NOTCH4
	Chronic hepatitis B infection	3	4.39E-07	TCF19, NOTCH4, HLA-DPB1
	induced agranulocytosis/granulocytopenia in treatment-resistant scl	3	1.85E-06	AGPAT1, PBX2, NOTCH4
	Hematology traits	3	2.42E-06	PSORS1C1, CDSN, CCHCR1
	Carotid plaque	2	1.19E-05	CFDP1, TMEM170A
	rug-induced Stevens-Johnson syndrome or toxic epidermal necrolys	2	2.51E-05	PSORS1C1, POU5F1
	Cold sores	2	2.51E-05	POU5F1, HCG27
	Systemic sclerosis	3	3.79E-05	PSORS1C1, NOTCH4, HLA-DPB1
	Hepatitis B	2	4.29E-05	HLA-DPB1, UBE2L3
	Epstein-Barr virus immune response (EBNA-1)	2	4.29E-05	PSORS1C1, NOTCH4
	White blood cell count	4	4.83E-05	PSORS1C1, CDSN, CCHCR1, HCG27
	Chronic obstructive pulmonary disease or coronary artery disease	2	1.25E-04	CFDP1, TMEM170A
	Oleic acid (18:1n-9) levels	2	1.42E-04	PPT2, EGFL8, AGPAT1
	HIV-1 control	2	1.42E-04	CDSN, NOTCH4
	Response to hepatitis B vaccine	2	1.81E-04	PSORS1C1, HLA-DPB1
	Blood protein levels	8	1.88E-04	PSORS1C1, CDSN, HCG27, ATF6B, AGER, GPSM3, NOTCH4, HLA-DPB1
	Type 1 diabetes and autoimmune thyroid diseases	2	2.25E-04	PSORS1C1, NOTCH4
	Chronic obstructive pulmonary disease-related biomarkers	2	2.99E-04	PSORS1C1, CCHCR1
	Psoriasis	3	4.06E-04	CCHCR1, HCG27, UBE2L3
	Chronic obstructive pulmonary disease	3	4.53E-04	AGER, AMZ1, CFDP1
	Night sleep phenotypes	4	6.14E-04	PSORS1C1, CCHCR1, TCF19, POU5F1
	Feeling nervous	2	8.65E-04	PSORS1C1, CCHCR1
	Automobile speeding propensity	2	8.65E-04	CDSN, ATF6B

	Lung cancer	4	2.71E-04	HCG27, MSH5, NOTCH4, HLA-DRA
	HIV-1 control	2	3.56E-04	VARS, NOTCH4
	Oleic acid (18:1n-9) levels	2	3.56E-04	PPT2, EGFL8, AGPAT1
	Type 1 diabetes and autoimmune thyroid diseases	2	5.61E-04	PRRC2A, NOTCH4
VHD_IBS	Takayasu arteritis	2	1.34E-05	PRRC2A, MSH5
	Menopause (age at onset)	2	2.33E-05	PRRC2A, MSH5
VHD_UC	Ulcerative colitis	16	7.12E-23	PSORS1C1, CDSN, CCHCR1, TCF19, POU5F1, HCG27, ATF6B, PPT2, EGFL8, AGPAT1, RNF5, AGER, PBX2, GPSM3, NOTCH4, AMZ1, UBE2L3
	Inflammatory bowel disease	16	1.66E-19	PSORS1C1, CDSN, CCHCR1, TCF19, POU5F1, HCG27, ATF6B, PPT2, EGFL8, AGPAT1, RNF5, AGER, PBX2, GPSM3, NOTCH4, AMZ1, UBE2L3
	Autism spectrum disorder or schizophrenia	15	5.36E-19	PSORS1C1, CDSN, CCHCR1, TCF19, POU5F1, HCG27, ATF6B, PPT2, EGFL8, AGPAT1, RNF5, AGER, PBX2, GPSM3, NOTCH4, HLA-DPB1
	Prostate cancer	9	3.13E-11	CCHCR1, ATF6B, PPT2, EGFL8, AGPAT1, RNF5, AGER, PBX2, GPSM3, NOTCH4
	Stevens-Johnson syndrome and toxic epidermal necrolysis	4	4.08E-11	PSORS1C1, CCHCR1, TCF19, POU5F1
	Multiple myeloma	5	6.87E-11	PSORS1C1, CDSN, CCHCR1, TCF19, POU5F1
	›idosis (non-Lofgren's syndrome without extrapulmonary manifesta	5	1.02E-10	ATF6B, AGER, GPSM3, NOTCH4, HLA-DPB1
	Idiopathic membranous nephropathy	4	3.83E-10	PSORS1C1, TCF19, PPT2, EGFL8, RNF5
	Takayasu arteritis	5	1.14E-08	PSORS1C1, CDSN, HCG27, ATF6B, NOTCH4
	Advanced glycation end-product levels	3	1.02E-07	RNF5, AGER, NOTCH4
	Chronic hepatitis B infection	3	4.39E-07	TCF19, NOTCH4, HLA-DPB1
	-induced agranulocytosis/granulocytopenia in treatment-resistant scl	3	1.85E-06	AGPAT1, PBX2, NOTCH4
	Hematology traits	3	2.42E-06	PSORS1C1, CDSN, CCHCR1
	Carotid plaque	2	1.19E-05	CFDP1, TMEM170A
	rug-induced Stevens-Johnson syndrome or toxic epidermal necrolys	2	2.51E-05	PSORS1C1, POU5F1
	Cold sores	2	2.51E-05	POU5F1, HCG27
	Systemic sclerosis	3	3.79E-05	PSORS1C1, NOTCH4, HLA-DPB1
	Hepatitis B	2	4.29E-05	HLA-DPB1, UBE2L3
	Epstein-Barr virus immune response (EBNA-1)	2	4.29E-05	PSORS1C1, NOTCH4
	White blood cell count	4	4.83E-05	PSORS1C1, CDSN, CCHCR1, HCG27
	Chronic obstructive pulmonary disease or coronary artery disease	2	1.25E-04	CFDP1, TMEM170A
	Oleic acid (18:1n-9) levels	2	1.42E-04	PPT2, EGFL8, AGPAT1
	HIV-1 control	2	1.42E-04	CDSN, NOTCH4
	Response to hepatitis B vaccine	2	1.81E-04	PSORS1C1, HLA-DPB1
	Blood protein levels	8	1.88E-04	PSORS1C1, CDSN, HCG27, ATF6B, AGER, GPSM3, NOTCH4, HLA-DPB1
	Type 1 diabetes and autoimmune thyroid diseases	2	2.25E-04	PSORS1C1, NOTCH4
	Chronic obstructive pulmonary disease-related biomarkers	2	2.99E-04	PSORS1C1, CCHCR1
	Psoriasis	3	4.06E-04	CCHCR1, HCG27, UBE2L3
	Chronic obstructive pulmonary disease	3	4.53E-04	AGER, AMZ1, CFDP1
	Night sleep phenotypes	4	6.14E-04	PSORS1C1, CCHCR1, TCF19, POU5F1
	Feeling nervous	2	8.65E-04	PSORS1C1, CCHCR1
	Automobile speeding propensity	2	8.65E-04	CDSN, ATF6B

	Lung cancer	4	2.71E-04	HCG27, MSH5, NOTCH4, HLA-DRA
	HIV-1 control	2	3.56E-04	VARS, NOTCH4
	Oleic acid (18:1n-9) levels	2	3.56E-04	PPT2, EGFL8, AGPAT1
	Type 1 diabetes and autoimmune thyroid diseases	2	5.61E-04	PRRC2A, NOTCH4
VHD_IBS	Takayasu arteritis	2	1.34E-05	PRRC2A, MSH5
	Menopause (age at onset)	2	2.33E-05	PRRC2A, MSH5
VHD_UC	Ulcerative colitis	16	7.12E-23	PSORS1C1, CDSN, CCHCR1, TCF19, POU5F1, HCG27, ATF6B, PPT2, EGFL8, AGPAT1, RNF5, AGER, PBX2, GPSM3, NOTCH4, AMZ1, UBE2L3
	Inflammatory bowel disease	16	1.66E-19	PSORS1C1, CDSN, CCHCR1, TCF19, POU5F1, HCG27, ATF6B, PPT2, EGFL8, AGPAT1, RNF5, AGER, PBX2, GPSM3, NOTCH4, AMZ1, UBE2L3
	Autism spectrum disorder or schizophrenia	15	5.36E-19	PSORS1C1, CDSN, CCHCR1, TCF19, POU5F1, HCG27, ATF6B, PPT2, EGFL8, AGPAT1, RNF5, AGER, PBX2, GPSM3, NOTCH4, HLA-DPB1
	Prostate cancer	9	3.13E-11	CCHCR1, ATF6B, PPT2, EGFL8, AGPAT1, RNF5, AGER, PBX2, GPSM3, NOTCH4
	Stevens-Johnson syndrome and toxic epidermal necrolysis	4	4.08E-11	PSORS1C1, CCHCR1, TCF19, POU5F1
	Multiple myeloma	5	6.87E-11	PSORS1C1, CDSN, CCHCR1, TCF19, POU5F1
	idosis (non-Lofgren's syndrome without extrapulmonary manifesta	5	1.02E-10	ATF6B, AGER, GPSM3, NOTCH4, HLA-DPB1
	Idiopathic membranous nephropathy	4	3.83E-10	PSORS1C1, TCF19, PPT2, EGFL8, RNF5
	Takayasu arteritis	5	1.14E-08	PSORS1C1, CDSN, HCG27, ATF6B, NOTCH4
	Advanced glycation end-product levels	3	1.02E-07	RNF5, AGER, NOTCH4
	Chronic hepatitis B infection	3	4.39E-07	TCF19, NOTCH4, HLA-DPB1
	-induced agranulocytosis/granulocytopenia in treatment-resistant scl	3	1.85E-06	AGPAT1, PBX2, NOTCH4
	Hematology traits	3	2.42E-06	PSORS1C1, CDSN, CCHCR1
	Carotid plaque	2	1.19E-05	CFDP1, TMEM170A
	rug-induced Stevens-Johnson syndrome or toxic epidermal necrolys	2	2.51E-05	PSORS1C1, POU5F1
	Cold sores	2	2.51E-05	POU5F1, HCG27
	Systemic sclerosis	3	3.79E-05	PSORS1C1, NOTCH4, HLA-DPB1
	Hepatitis B	2	4.29E-05	HLA-DPB1, UBE2L3
	Epstein-Barr virus immune response (EBNA-1)	2	4.29E-05	PSORS1C1, NOTCH4
	White blood cell count	4	4.83E-05	PSORS1C1, CDSN, CCHCR1, HCG27
	Chronic obstructive pulmonary disease or coronary artery disease	2	1.25E-04	CFDP1, TMEM170A
	Oleic acid (18:1n-9) levels	2	1.42E-04	PPT2, EGFL8, AGPAT1
	HIV-1 control	2	1.42E-04	CDSN, NOTCH4
	Response to hepatitis B vaccine	2	1.81E-04	PSORS1C1, HLA-DPB1
	Blood protein levels	8	1.88E-04	PSORS1C1, CDSN, HCG27, ATF6B, AGER, GPSM3, NOTCH4, HLA-DPB1
	Type 1 diabetes and autoimmune thyroid diseases	2	2.25E-04	PSORS1C1, NOTCH4
	Chronic obstructive pulmonary disease-related biomarkers	2	2.99E-04	PSORS1C1, CCHCR1
	Psoriasis	3	4.06E-04	CCHCR1, HCG27, UBE2L3
	Chronic obstructive pulmonary disease	3	4.53E-04	AGER, AMZ1, CFDP1
	Night sleep phenotypes	4	6.14E-04	PSORS1C1, CCHCR1, TCF19, POU5F1
	Feeling nervous	2	8.65E-04	PSORS1C1, CCHCR1
	Automobile speeding propensity	2	8.65E-04	CDSN, ATF6B

	Lung cancer	4	2.71E-04	HCG27, MSH5, NOTCH4, HLA-DRA
	HIV-1 control	2	3.56E-04	VARS, NOTCH4
	Oleic acid (18:1n-9) levels	2	3.56E-04	PPT2, EGFL8, AGPAT1
	Type 1 diabetes and autoimmune thyroid diseases	2	5.61E-04	PRRC2A, NOTCH4
VHD_IBS	Takayasu arteritis	2	1.34E-05	PRRC2A, MSH5
	Menopause (age at onset)	2	2.33E-05	PRRC2A, MSH5
VHD_UC	Ulcerative colitis	16	7.12E-23	PSORS1C1, CDSN, CCHCR1, TCF19, POU5F1, HCG27, ATF6B, PPT2, EGFL8, AGPAT1, RNF5, AGER, PBX2, GPSM3, NOTCH4, AMZ1, UBE2L3
	Inflammatory bowel disease	16	1.66E-19	PSORS1C1, CDSN, CCHCR1, TCF19, POU5F1, HCG27, ATF6B, PPT2, EGFL8, AGPAT1, RNF5, AGER, PBX2, GPSM3, NOTCH4, AMZ1, UBE2L3
	Autism spectrum disorder or schizophrenia	15	5.36E-19	PSORS1C1, CDSN, CCHCR1, TCF19, POU5F1, HCG27, ATF6B, PPT2, EGFL8, AGPAT1, RNF5, AGER, PBX2, GPSM3, NOTCH4, HLA-DPB1
	Prostate cancer	9	3.13E-11	CCHCR1, ATF6B, PPT2, EGFL8, AGPAT1, RNF5, AGER, PBX2, GPSM3, NOTCH4
	Stevens-Johnson syndrome and toxic epidermal necrolysis	4	4.08E-11	PSORS1C1, CCHCR1, TCF19, POU5F1
	Multiple myeloma	5	6.87E-11	PSORS1C1, CDSN, CCHCR1, TCF19, POU5F1
	›idosis (non-Lofgren's syndrome without extrapulmonary manifesta	5	1.02E-10	ATF6B, AGER, GPSM3, NOTCH4, HLA-DPB1
	Idiopathic membranous nephropathy	4	3.83E-10	PSORS1C1, TCF19, PPT2, EGFL8, RNF5
	Takayasu arteritis	5	1.14E-08	PSORS1C1, CDSN, HCG27, ATF6B, NOTCH4
	Advanced glycation end-product levels	3	1.02E-07	RNF5, AGER, NOTCH4
	Chronic hepatitis B infection	3	4.39E-07	TCF19, NOTCH4, HLA-DPB1
	-induced agranulocytosis/granulocytopenia in treatment-resistant scl	3	1.85E-06	AGPAT1, PBX2, NOTCH4
	Hematology traits	3	2.42E-06	PSORS1C1, CDSN, CCHCR1
	Carotid plaque	2	1.19E-05	CFDP1, TMEM170A
	rug-induced Stevens-Johnson syndrome or toxic epidermal necrolys	2	2.51E-05	PSORS1C1, POU5F1
	Cold sores	2	2.51E-05	POU5F1, HCG27
	Systemic sclerosis	3	3.79E-05	PSORS1C1, NOTCH4, HLA-DPB1
	Hepatitis B	2	4.29E-05	HLA-DPB1, UBE2L3
	Epstein-Barr virus immune response (EBNA-1)	2	4.29E-05	PSORS1C1, NOTCH4
	White blood cell count	4	4.83E-05	PSORS1C1, CDSN, CCHCR1, HCG27
	Chronic obstructive pulmonary disease or coronary artery disease	2	1.25E-04	CFDP1, TMEM170A
	Oleic acid (18:1n-9) levels	2	1.42E-04	PPT2, EGFL8, AGPAT1
	HIV-1 control	2	1.42E-04	CDSN, NOTCH4
	Response to hepatitis B vaccine	2	1.81E-04	PSORS1C1, HLA-DPB1
	Blood protein levels	8	1.88E-04	PSORS1C1, CDSN, HCG27, ATF6B, AGER, GPSM3, NOTCH4, HLA-DPB1
	Type 1 diabetes and autoimmune thyroid diseases	2	2.25E-04	PSORS1C1, NOTCH4
	Chronic obstructive pulmonary disease-related biomarkers	2	2.99E-04	PSORS1C1, CCHCR1
	Psoriasis	3	4.06E-04	CCHCR1, HCG27, UBE2L3
	Chronic obstructive pulmonary disease	3	4.53E-04	AGER, AMZ1, CFDP1
	Night sleep phenotypes	4	6.14E-04	PSORS1C1, CCHCR1, TCF19, POU5F1
	Feeling nervous	2	8.65E-04	PSORS1C1, CCHCR1
	Automobile speeding propensity	2	8.65E-04	CDSN, ATF6B

	Lung cancer	4	2.71E-04	HCG27, MSH5, NOTCH4, HLA-DRA
	HIV-1 control	2	3.56E-04	VARS, NOTCH4
	Oleic acid (18:1n-9) levels	2	3.56E-04	PPT2, EGFL8, AGPAT1
	Type 1 diabetes and autoimmune thyroid diseases	2	5.61E-04	PRRC2A, NOTCH4
VHD_IBS	Takayasu arteritis	2	1.34E-05	PRRC2A, MSH5
	Menopause (age at onset)	2	2.33E-05	PRRC2A, MSH5
VHD_UC	Ulcerative colitis	16	7.12E-23	PSORS1C1, CDSN, CCHCR1, TCF19, POU5F1, HCG27, ATF6B, PPT2, EGFL8, AGPAT1, RNF5, AGER, PBX2, GPSM3, NOTCH4, AMZ1, UBE2L3
	Inflammatory bowel disease	16	1.66E-19	PSORS1C1, CDSN, CCHCR1, TCF19, POU5F1, HCG27, ATF6B, PPT2, EGFL8, AGPAT1, RNF5, AGER, PBX2, GPSM3, NOTCH4, AMZ1, UBE2L3
	Autism spectrum disorder or schizophrenia	15	5.36E-19	PSORS1C1, CDSN, CCHCR1, TCF19, POU5F1, HCG27, ATF6B, PPT2, EGFL8, AGPAT1, RNF5, AGER, PBX2, GPSM3, NOTCH4, HLA-DPB1
	Prostate cancer	9	3.13E-11	CCHCR1, ATF6B, PPT2, EGFL8, AGPAT1, RNF5, AGER, PBX2, GPSM3, NOTCH4
	Stevens-Johnson syndrome and toxic epidermal necrolysis	4	4.08E-11	PSORS1C1, CCHCR1, TCF19, POU5F1
	Multiple myeloma	5	6.87E-11	PSORS1C1, CDSN, CCHCR1, TCF19, POU5F1
	›idosis (non-Lofgren's syndrome without extrapulmonary manifesta	5	1.02E-10	ATF6B, AGER, GPSM3, NOTCH4, HLA-DPB1
	Idiopathic membranous nephropathy	4	3.83E-10	PSORS1C1, TCF19, PPT2, EGFL8, RNF5
	Takayasu arteritis	5	1.14E-08	PSORS1C1, CDSN, HCG27, ATF6B, NOTCH4
	Advanced glycation end-product levels	3	1.02E-07	RNF5, AGER, NOTCH4
	Chronic hepatitis B infection	3	4.39E-07	TCF19, NOTCH4, HLA-DPB1
	-induced agranulocytosis/granulocytopenia in treatment-resistant scl	3	1.85E-06	AGPAT1, PBX2, NOTCH4
	Hematology traits	3	2.42E-06	PSORS1C1, CDSN, CCHCR1
	Carotid plaque	2	1.19E-05	CFDP1, TMEM170A
	rug-induced Stevens-Johnson syndrome or toxic epidermal necrolys	2	2.51E-05	PSORS1C1, POU5F1
	Cold sores	2	2.51E-05	POU5F1, HCG27
	Systemic sclerosis	3	3.79E-05	PSORS1C1, NOTCH4, HLA-DPB1
	Hepatitis B	2	4.29E-05	HLA-DPB1, UBE2L3
	Epstein-Barr virus immune response (EBNA-1)	2	4.29E-05	PSORS1C1, NOTCH4
	White blood cell count	4	4.83E-05	PSORS1C1, CDSN, CCHCR1, HCG27
	Chronic obstructive pulmonary disease or coronary artery disease	2	1.25E-04	CFDP1, TMEM170A
	Oleic acid (18:1n-9) levels	2	1.42E-04	PPT2, EGFL8, AGPAT1
	HIV-1 control	2	1.42E-04	CDSN, NOTCH4
	Response to hepatitis B vaccine	2	1.81E-04	PSORS1C1, HLA-DPB1
	Blood protein levels	8	1.88E-04	PSORS1C1, CDSN, HCG27, ATF6B, AGER, GPSM3, NOTCH4, HLA-DPB1
	Type 1 diabetes and autoimmune thyroid diseases	2	2.25E-04	PSORS1C1, NOTCH4
	Chronic obstructive pulmonary disease-related biomarkers	2	2.99E-04	PSORS1C1, CCHCR1
	Psoriasis	3	4.06E-04	CCHCR1, HCG27, UBE2L3
	Chronic obstructive pulmonary disease	3	4.53E-04	AGER, AMZ1, CFDP1
	Night sleep phenotypes	4	6.14E-04	PSORS1C1, CCHCR1, TCF19, POU5F1
	Feeling nervous	2	8.65E-04	PSORS1C1, CCHCR1
	Automobile speeding propensity	2	8.65E-04	CDSN, ATF6B

	Lung cancer	4	2.71E-04	HCG27, MSH5, NOTCH4, HLA-DRA
	HIV-1 control	2	3.56E-04	VARS, NOTCH4
	Oleic acid (18:1n-9) levels	2	3.56E-04	PPT2, EGFL8, AGPAT1
	Type 1 diabetes and autoimmune thyroid diseases	2	5.61E-04	PRRC2A, NOTCH4
VHD_IBS	Takayasu arteritis	2	1.34E-05	PRRC2A, MSH5
	Menopause (age at onset)	2	2.33E-05	PRRC2A, MSH5
VHD_UC	Ulcerative colitis	16	7.12E-23	PSORS1C1, CDSN, CCHCR1, TCF19, POU5F1, HCG27, ATF6B, PPT2, EGFL8, AGPAT1, RNF5, AGER, PBX2, GPSM3, NOTCH4, AMZ1, UBE2L3
	Inflammatory bowel disease	16	1.66E-19	PSORS1C1, CDSN, CCHCR1, TCF19, POU5F1, HCG27, ATF6B, PPT2, EGFL8, AGPAT1, RNF5, AGER, PBX2, GPSM3, NOTCH4, AMZ1, UBE2L3
	Autism spectrum disorder or schizophrenia	15	5.36E-19	PSORS1C1, CDSN, CCHCR1, TCF19, POU5F1, HCG27, ATF6B, PPT2, EGFL8, AGPAT1, RNF5, AGER, PBX2, GPSM3, NOTCH4, HLA-DPB1
	Prostate cancer	9	3.13E-11	CCHCR1, ATF6B, PPT2, EGFL8, AGPAT1, RNF5, AGER, PBX2, GPSM3, NOTCH4
	Stevens-Johnson syndrome and toxic epidermal necrolysis	4	4.08E-11	PSORS1C1, CCHCR1, TCF19, POU5F1
	Multiple myeloma	5	6.87E-11	PSORS1C1, CDSN, CCHCR1, TCF19, POU5F1
	oidosis (non-Lofgren's syndrome without extrapulmonary manifesta	5	1.02E-10	ATF6B, AGER, GPSM3, NOTCH4, HLA-DPB1
	Idiopathic membranous nephropathy	4	3.83E-10	PSORS1C1, TCF19, PPT2, EGFL8, RNF5
	Takayasu arteritis	5	1.14E-08	PSORS1C1, CDSN, HCG27, ATF6B, NOTCH4
	Advanced glycation end-product levels	3	1.02E-07	RNF5, AGER, NOTCH4
	Chronic hepatitis B infection	3	4.39E-07	TCF19, NOTCH4, HLA-DPB1
	-induced agranulocytosis/granulocytopenia in treatment-resistant scl	3	1.85E-06	AGPAT1, PBX2, NOTCH4
	Hematology traits	3	2.42E-06	PSORS1C1, CDSN, CCHCR1
	Carotid plaque	2	1.19E-05	CFDP1, TMEM170A
	rug-induced Stevens-Johnson syndrome or toxic epidermal necrolys	2	2.51E-05	PSORS1C1, POU5F1
	Cold sores	2	2.51E-05	POU5F1, HCG27
	Systemic sclerosis	3	3.79E-05	PSORS1C1, NOTCH4, HLA-DPB1
	Hepatitis B	2	4.29E-05	HLA-DPB1, UBE2L3
	Epstein-Barr virus immune response (EBNA-1)	2	4.29E-05	PSORS1C1, NOTCH4
	White blood cell count	4	4.83E-05	PSORS1C1, CDSN, CCHCR1, HCG27
	Chronic obstructive pulmonary disease or coronary artery disease	2	1.25E-04	CFDP1, TMEM170A
	Oleic acid (18:1n-9) levels	2	1.42E-04	PPT2, EGFL8, AGPAT1
	HIV-1 control	2	1.42E-04	CDSN, NOTCH4
	Response to hepatitis B vaccine	2	1.81E-04	PSORS1C1, HLA-DPB1
	Blood protein levels	8	1.88E-04	PSORS1C1, CDSN, HCG27, ATF6B, AGER, GPSM3, NOTCH4, HLA-DPB1
	Type 1 diabetes and autoimmune thyroid diseases	2	2.25E-04	PSORS1C1, NOTCH4
	Chronic obstructive pulmonary disease-related biomarkers	2	2.99E-04	PSORS1C1, CCHCR1
	Psoriasis	3	4.06E-04	CCHCR1, HCG27, UBE2L3
	Chronic obstructive pulmonary disease	3	4.53E-04	AGER, AMZ1, CFDP1
	Night sleep phenotypes	4	6.14E-04	PSORS1C1, CCHCR1, TCF19, POU5F1
	Feeling nervous	2	8.65E-04	PSORS1C1, CCHCR1
	Automobile speeding propensity	2	8.65E-04	CDSN, ATF6B

	Lung cancer	4	2.71E-04	HCG27, MSH5, NOTCH4, HLA-DRA
	HIV-1 control	2	3.56E-04	VARS, NOTCH4
	Oleic acid (18:1n-9) levels	2	3.56E-04	PPT2, EGFL8, AGPAT1
	Type 1 diabetes and autoimmune thyroid diseases	2	5.61E-04	PRRC2A, NOTCH4
VHD_IBS	Takayasu arteritis	2	1.34E-05	PRRC2A, MSH5
	Menopause (age at onset)	2	2.33E-05	PRRC2A, MSH5
VHD_UC	Ulcerative colitis	16	7.12E-23	PSORS1C1, CDSN, CCHCR1, TCF19, POU5F1, HCG27, ATF6B, PPT2, EGFL8, AGPAT1, RNF5, AGER, PBX2, GPSM3, NOTCH4, AMZ1, UBE2L3
	Inflammatory bowel disease	16	1.66E-19	PSORS1C1, CDSN, CCHCR1, TCF19, POU5F1, HCG27, ATF6B, PPT2, EGFL8, AGPAT1, RNF5, AGER, PBX2, GPSM3, NOTCH4, AMZ1, UBE2L3
	Autism spectrum disorder or schizophrenia	15	5.36E-19	PSORS1C1, CDSN, CCHCR1, TCF19, POU5F1, HCG27, ATF6B, PPT2, EGFL8, AGPAT1, RNF5, AGER, PBX2, GPSM3, NOTCH4, HLA-DPB1
	Prostate cancer	9	3.13E-11	CCHCR1, ATF6B, PPT2, EGFL8, AGPAT1, RNF5, AGER, PBX2, GPSM3, NOTCH4
	Stevens-Johnson syndrome and toxic epidermal necrolysis	4	4.08E-11	PSORS1C1, CCHCR1, TCF19, POU5F1
	Multiple myeloma	5	6.87E-11	PSORS1C1, CDSN, CCHCR1, TCF19, POU5F1
	idosis (non-Lofgren's syndrome without extrapulmonary manifesta	5	1.02E-10	ATF6B, AGER, GPSM3, NOTCH4, HLA-DPB1
	Idiopathic membranous nephropathy	4	3.83E-10	PSORS1C1, TCF19, PPT2, EGFL8, RNF5
	Takayasu arteritis	5	1.14E-08	PSORS1C1, CDSN, HCG27, ATF6B, NOTCH4
	Advanced glycation end-product levels	3	1.02E-07	RNF5, AGER, NOTCH4
	Chronic hepatitis B infection	3	4.39E-07	TCF19, NOTCH4, HLA-DPB1
	-induced agranulocytosis/granulocytopenia in treatment-resistant sc	3	1.85E-06	AGPAT1, PBX2, NOTCH4
	Hematology traits	3	2.42E-06	PSORS1C1, CDSN, CCHCR1
	Carotid plaque	2	1.19E-05	CFDP1, TMEM170A
	rug-induced Stevens-Johnson syndrome or toxic epidermal necrolys	2	2.51E-05	PSORS1C1, POU5F1
	Cold sores	2	2.51E-05	POU5F1, HCG27
	Systemic sclerosis	3	3.79E-05	PSORS1C1, NOTCH4, HLA-DPB1
	Hepatitis B	2	4.29E-05	HLA-DPB1, UBE2L3
	Epstein-Barr virus immune response (EBNA-1)	2	4.29E-05	PSORS1C1, NOTCH4
	White blood cell count	4	4.83E-05	PSORS1C1, CDSN, CCHCR1, HCG27
	Chronic obstructive pulmonary disease or coronary artery disease	2	1.25E-04	CFDP1, TMEM170A
	Oleic acid (18:1n-9) levels	2	1.42E-04	PPT2, EGFL8, AGPAT1
	HIV-1 control	2	1.42E-04	CDSN, NOTCH4
	Response to hepatitis B vaccine	2	1.81E-04	PSORS1C1, HLA-DPB1
	Blood protein levels	8	1.88E-04	PSORS1C1, CDSN, HCG27, ATF6B, AGER, GPSM3, NOTCH4, HLA-DPB1
	Type 1 diabetes and autoimmune thyroid diseases	2	2.25E-04	PSORS1C1, NOTCH4
	Chronic obstructive pulmonary disease-related biomarkers	2	2.99E-04	PSORS1C1, CCHCR1
	Psoriasis	3	4.06E-04	CCHCR1, HCG27, UBE2L3
	Chronic obstructive pulmonary disease	3	4.53E-04	AGER, AMZ1, CFDP1
	Night sleep phenotypes	4	6.14E-04	PSORS1C1, CCHCR1, TCF19, POU5F1
	Feeling nervous	2	8.65E-04	PSORS1C1, CCHCR1
	Automobile speeding propensity	2	8.65E-04	CDSN, ATF6B

	Lung cancer	4	2.71E-04	HCG27, MSH5, NOTCH4, HLA-DRA
	HIV-1 control	2	3.56E-04	VARS, NOTCH4
	Oleic acid (18:1n-9) levels	2	3.56E-04	PPT2, EGFL8, AGPAT1
	Type 1 diabetes and autoimmune thyroid diseases	2	5.61E-04	PRRC2A, NOTCH4
VHD_IBS	Takayasu arteritis	2	1.34E-05	PRRC2A, MSH5
	Menopause (age at onset)	2	2.33E-05	PRRC2A, MSH5
VHD_UC	Ulcerative colitis	16	7.12E-23	PSORS1C1, CDSN, CCHCR1, TCF19, POU5F1, HCG27, ATF6B, PPT2, EGFL8, AGPAT1, RNF5, AGER, PBX2, GPSM3, NOTCH4, AMZ1, UBE2L3
	Inflammatory bowel disease	16	1.66E-19	PSORS1C1, CDSN, CCHCR1, TCF19, POU5F1, HCG27, ATF6B, PPT2, EGFL8, AGPAT1, RNF5, AGER, PBX2, GPSM3, NOTCH4, AMZ1, UBE2L3
	Autism spectrum disorder or schizophrenia	15	5.36E-19	PSORS1C1, CDSN, CCHCR1, TCF19, POU5F1, HCG27, ATF6B, PPT2, EGFL8, AGPAT1, RNF5, AGER, PBX2, GPSM3, NOTCH4, HLA-DPB1
	Prostate cancer	9	3.13E-11	CCHCR1, ATF6B, PPT2, EGFL8, AGPAT1, RNF5, AGER, PBX2, GPSM3, NOTCH4
	Stevens-Johnson syndrome and toxic epidermal necrolysis	4	4.08E-11	PSORS1C1, CCHCR1, TCF19, POU5F1
	Multiple myeloma	5	6.87E-11	PSORS1C1, CDSN, CCHCR1, TCF19, POU5F1
	idosis (non-Lofgren's syndrome without extrapulmonary manifesta	5	1.02E-10	ATF6B, AGER, GPSM3, NOTCH4, HLA-DPB1
	Idiopathic membranous nephropathy	4	3.83E-10	PSORS1C1, TCF19, PPT2, EGFL8, RNF5
	Takayasu arteritis	5	1.14E-08	PSORS1C1, CDSN, HCG27, ATF6B, NOTCH4
	Advanced glycation end-product levels	3	1.02E-07	RNF5, AGER, NOTCH4
	Chronic hepatitis B infection	3	4.39E-07	TCF19, NOTCH4, HLA-DPB1
	induced agranulocytosis/granulocytopenia in treatment-resistant scl	3	1.85E-06	AGPAT1, PBX2, NOTCH4
	Hematology traits	3	2.42E-06	PSORS1C1, CDSN, CCHCR1
	Carotid plaque	2	1.19E-05	CFDP1, TMEM170A
	rug-induced Stevens-Johnson syndrome or toxic epidermal necrolys	2	2.51E-05	PSORS1C1, POU5F1
	Cold sores	2	2.51E-05	POU5F1, HCG27
	Systemic sclerosis	3	3.79E-05	PSORS1C1, NOTCH4, HLA-DPB1
	Hepatitis B	2	4.29E-05	HLA-DPB1, UBE2L3
	Epstein-Barr virus immune response (EBNA-1)	2	4.29E-05	PSORS1C1, NOTCH4
	White blood cell count	4	4.83E-05	PSORS1C1, CDSN, CCHCR1, HCG27
	Chronic obstructive pulmonary disease or coronary artery disease	2	1.25E-04	CFDP1, TMEM170A
	Oleic acid (18:1n-9) levels	2	1.42E-04	PPT2, EGFL8, AGPAT1
	HIV-1 control	2	1.42E-04	CDSN, NOTCH4
	Response to hepatitis B vaccine	2	1.81E-04	PSORS1C1, HLA-DPB1
	Blood protein levels	8	1.88E-04	PSORS1C1, CDSN, HCG27, ATF6B, AGER, GPSM3, NOTCH4, HLA-DPB1
	Type 1 diabetes and autoimmune thyroid diseases	2	2.25E-04	PSORS1C1, NOTCH4
	Chronic obstructive pulmonary disease-related biomarkers	2	2.99E-04	PSORS1C1, CCHCR1
	Psoriasis	3	4.06E-04	CCHCR1, HCG27, UBE2L3
	Chronic obstructive pulmonary disease	3	4.53E-04	AGER, AMZ1, CFDP1
	Night sleep phenotypes	4	6.14E-04	PSORS1C1, CCHCR1, TCF19, POU5F1
	Feeling nervous	2	8.65E-04	PSORS1C1, CCHCR1
	Automobile speeding propensity	2	8.65E-04	CDSN, ATF6B

	Lung cancer	4	2.71E-04	HCG27, MSH5, NOTCH4, HLA-DRA
	HIV-1 control	2	3.56E-04	VARS, NOTCH4
	Oleic acid (18:1n-9) levels	2	3.56E-04	PPT2, EGFL8, AGPAT1
	Type 1 diabetes and autoimmune thyroid diseases	2	5.61E-04	PRRC2A, NOTCH4
VHD_IBS	Takayasu arteritis	2	1.34E-05	PRRC2A, MSH5
	Menopause (age at onset)	2	2.33E-05	PRRC2A, MSH5
VHD_UC	Ulcerative colitis	16	7.12E-23	PSORS1C1, CDSN, CCHCR1, TCF19, POU5F1, HCG27, ATF6B, PPT2, EGFL8, AGPAT1, RNF5, AGER, PBX2, GPSM3, NOTCH4, AMZ1, UBE2L3
	Inflammatory bowel disease	16	1.66E-19	PSORS1C1, CDSN, CCHCR1, TCF19, POU5F1, HCG27, ATF6B, PPT2, EGFL8, AGPAT1, RNF5, AGER, PBX2, GPSM3, NOTCH4, AMZ1, UBE2L3
	Autism spectrum disorder or schizophrenia	15	5.36E-19	PSORS1C1, CDSN, CCHCR1, TCF19, POU5F1, HCG27, ATF6B, PPT2, EGFL8, AGPAT1, RNF5, AGER, PBX2, GPSM3, NOTCH4, HLA-DPB1
	Prostate cancer	9	3.13E-11	CCHCR1, ATF6B, PPT2, EGFL8, AGPAT1, RNF5, AGER, PBX2, GPSM3, NOTCH4
	Stevens-Johnson syndrome and toxic epidermal necrolysis	4	4.08E-11	PSORS1C1, CCHCR1, TCF19, POU5F1
	Multiple myeloma	5	6.87E-11	PSORS1C1, CDSN, CCHCR1, TCF19, POU5F1
	idosis (non-Lofgren's syndrome without extrapulmonary manifestat	5	1.02E-10	ATF6B, AGER, GPSM3, NOTCH4, HLA-DPB1
	Idiopathic membranous nephropathy	4	3.83E-10	PSORS1C1, TCF19, PPT2, EGFL8, RNF5
	Takayasu arteritis	5	1.14E-08	PSORS1C1, CDSN, HCG27, ATF6B, NOTCH4
	Advanced glycation end-product levels	3	1.02E-07	RNF5, AGER, NOTCH4
	Chronic hepatitis B infection	3	4.39E-07	TCF19, NOTCH4, HLA-DPB1
	induced agranulocytosis/granulocytopenia in treatment-resistant scl	3	1.85E-06	AGPAT1, PBX2, NOTCH4
	Hematology traits	3	2.42E-06	PSORS1C1, CDSN, CCHCR1
	Carotid plaque	2	1.19E-05	CFDP1, TMEM170A
	rug-induced Stevens-Johnson syndrome or toxic epidermal necrolys	2	2.51E-05	PSORS1C1, POU5F1
	Cold sores	2	2.51E-05	POU5F1, HCG27
	Systemic sclerosis	3	3.79E-05	PSORS1C1, NOTCH4, HLA-DPB1
	Hepatitis B	2	4.29E-05	HLA-DPB1, UBE2L3
	Epstein-Barr virus immune response (EBNA-1)	2	4.29E-05	PSORS1C1, NOTCH4
	White blood cell count	4	4.83E-05	PSORS1C1, CDSN, CCHCR1, HCG27
	Chronic obstructive pulmonary disease or coronary artery disease	2	1.25E-04	CFDP1, TMEM170A
	Oleic acid (18:1n-9) levels	2	1.42E-04	PPT2, EGFL8, AGPAT1
	HIV-1 control	2	1.42E-04	CDSN, NOTCH4
	Response to hepatitis B vaccine	2	1.81E-04	PSORS1C1, HLA-DPB1
	Blood protein levels	8	1.88E-04	PSORS1C1, CDSN, HCG27, ATF6B, AGER, GPSM3, NOTCH4, HLA-DPB1
	Type 1 diabetes and autoimmune thyroid diseases	2	2.25E-04	PSORS1C1, NOTCH4
	Chronic obstructive pulmonary disease-related biomarkers	2	2.99E-04	PSORS1C1, CCHCR1
	Psoriasis	3	4.06E-04	CCHCR1, HCG27, UBE2L3
	Chronic obstructive pulmonary disease	3	4.53E-04	AGER, AMZ1, CFDP1
	Night sleep phenotypes	4	6.14E-04	PSORS1C1, CCHCR1, TCF19, POU5F1
	Feeling nervous	2	8.65E-04	PSORS1C1, CCHCR1
	Automobile speeding propensity	2	8.65E-04	CDSN, ATF6B

	Lung cancer	4	2.71E-04	HCG27, MSH5, NOTCH4, HLA-DRA
	HIV-1 control	2	3.56E-04	VARS, NOTCH4
	Oleic acid (18:1n-9) levels	2	3.56E-04	PPT2, EGFL8, AGPAT1
	Type 1 diabetes and autoimmune thyroid diseases	2	5.61E-04	PRRC2A, NOTCH4
VHD_IBS	Takayasu arteritis	2	1.34E-05	PRRC2A, MSH5
	Menopause (age at onset)	2	2.33E-05	PRRC2A, MSH5
VHD_UC	Ulcerative colitis	16	7.12E-23	PSORS1C1, CDSN, CCHCR1, TCF19, POU5F1, HCG27, ATF6B, PPT2, EGFL8, AGPAT1, RNF5, AGER, PBX2, GPSM3, NOTCH4, AMZ1, UBE2L3
	Inflammatory bowel disease	16	1.66E-19	PSORS1C1, CDSN, CCHCR1, TCF19, POU5F1, HCG27, ATF6B, PPT2, EGFL8, AGPAT1, RNF5, AGER, PBX2, GPSM3, NOTCH4, AMZ1, UBE2L3
	Autism spectrum disorder or schizophrenia	15	5.36E-19	PSORS1C1, CDSN, CCHCR1, TCF19, POU5F1, HCG27, ATF6B, PPT2, EGFL8, AGPAT1, RNF5, AGER, PBX2, GPSM3, NOTCH4, HLA-DPB1
	Prostate cancer	9	3.13E-11	CCHCR1, ATF6B, PPT2, EGFL8, AGPAT1, RNF5, AGER, PBX2, GPSM3, NOTCH4
	Stevens-Johnson syndrome and toxic epidermal necrolysis	4	4.08E-11	PSORS1C1, CCHCR1, TCF19, POU5F1
	Multiple myeloma	5	6.87E-11	PSORS1C1, CDSN, CCHCR1, TCF19, POU5F1
	›idosis (non-Lofgren's syndrome without extrapulmonary manifestat	5	1.02E-10	ATF6B, AGER, GPSM3, NOTCH4, HLA-DPB1
	Idiopathic membranous nephropathy	4	3.83E-10	PSORS1C1, TCF19, PPT2, EGFL8, RNF5
	Takayasu arteritis	5	1.14E-08	PSORS1C1, CDSN, HCG27, ATF6B, NOTCH4
	Advanced glycation end-product levels	3	1.02E-07	RNF5, AGER, NOTCH4
	Chronic hepatitis B infection	3	4.39E-07	TCF19, NOTCH4, HLA-DPB1
	-induced agranulocytosis/granulocytopenia in treatment-resistant scl	3	1.85E-06	AGPAT1, PBX2, NOTCH4
	Hematology traits	3	2.42E-06	PSORS1C1, CDSN, CCHCR1
	Carotid plaque	2	1.19E-05	CFDP1, TMEM170A
	rug-induced Stevens-Johnson syndrome or toxic epidermal necrolys	2	2.51E-05	PSORS1C1, POU5F1
	Cold sores	2	2.51E-05	POU5F1, HCG27
	Systemic sclerosis	3	3.79E-05	PSORS1C1, NOTCH4, HLA-DPB1
	Hepatitis B	2	4.29E-05	HLA-DPB1, UBE2L3
	Epstein-Barr virus immune response (EBNA-1)	2	4.29E-05	PSORS1C1, NOTCH4
	White blood cell count	4	4.83E-05	PSORS1C1, CDSN, CCHCR1, HCG27
	Chronic obstructive pulmonary disease or coronary artery disease	2	1.25E-04	CFDP1, TMEM170A
	Oleic acid (18:1n-9) levels	2	1.42E-04	PPT2, EGFL8, AGPAT1
	HIV-1 control	2	1.42E-04	CDSN, NOTCH4
	Response to hepatitis B vaccine	2	1.81E-04	PSORS1C1, HLA-DPB1
	Blood protein levels	8	1.88E-04	PSORS1C1, CDSN, HCG27, ATF6B, AGER, GPSM3, NOTCH4, HLA-DPB1
	Type 1 diabetes and autoimmune thyroid diseases	2	2.25E-04	PSORS1C1, NOTCH4
	Chronic obstructive pulmonary disease-related biomarkers	2	2.99E-04	PSORS1C1, CCHCR1
	Psoriasis	3	4.06E-04	CCHCR1, HCG27, UBE2L3
	Chronic obstructive pulmonary disease	3	4.53E-04	AGER, AMZ1, CFDP1
	Night sleep phenotypes	4	6.14E-04	PSORS1C1, CCHCR1, TCF19, POU5F1
	Feeling nervous	2	8.65E-04	PSORS1C1, CCHCR1
	Automobile speeding propensity	2	8.65E-04	CDSN, ATF6B

	Lung cancer	4	2.71E-04	HCG27, MSH5, NOTCH4, HLA-DRA
	HIV-1 control	2	3.56E-04	VARS, NOTCH4
	Oleic acid (18:1n-9) levels	2	3.56E-04	PPT2, EGFL8, AGPAT1
	Type 1 diabetes and autoimmune thyroid diseases	2	5.61E-04	PRRC2A, NOTCH4
VHD_IBS	Takayasu arteritis	2	1.34E-05	PRRC2A, MSH5
	Menopause (age at onset)	2	2.33E-05	PRRC2A, MSH5
VHD_UC	Ulcerative colitis	16	7.12E-23	PSORS1C1, CDSN, CCHCR1, TCF19, POU5F1, HCG27, ATF6B, PPT2, EGFL8, AGPAT1, RNF5, AGER, PBX2, GPSM3, NOTCH4, AMZ1, UBE2L3
	Inflammatory bowel disease	16	1.66E-19	PSORS1C1, CDSN, CCHCR1, TCF19, POU5F1, HCG27, ATF6B, PPT2, EGFL8, AGPAT1, RNF5, AGER, PBX2, GPSM3, NOTCH4, AMZ1, UBE2L3
	Autism spectrum disorder or schizophrenia	15	5.36E-19	PSORS1C1, CDSN, CCHCR1, TCF19, POU5F1, HCG27, ATF6B, PPT2, EGFL8, AGPAT1, RNF5, AGER, PBX2, GPSM3, NOTCH4, HLA-DPB1
	Prostate cancer	9	3.13E-11	CCHCR1, ATF6B, PPT2, EGFL8, AGPAT1, RNF5, AGER, PBX2, GPSM3, NOTCH4
	Stevens-Johnson syndrome and toxic epidermal necrolysis	4	4.08E-11	PSORS1C1, CCHCR1, TCF19, POU5F1
	Multiple myeloma	5	6.87E-11	PSORS1C1, CDSN, CCHCR1, TCF19, POU5F1
	oidosis (non-Lofgren's syndrome without extrapulmonary manifestat	5	1.02E-10	ATF6B, AGER, GPSM3, NOTCH4, HLA-DPB1
	Idiopathic membranous nephropathy	4	3.83E-10	PSORS1C1, TCF19, PPT2, EGFL8, RNF5
	Takayasu arteritis	5	1.14E-08	PSORS1C1, CDSN, HCG27, ATF6B, NOTCH4
	Advanced glycation end-product levels	3	1.02E-07	RNF5, AGER, NOTCH4
	Chronic hepatitis B infection	3	4.39E-07	TCF19, NOTCH4, HLA-DPB1
	-induced agranulocytosis/granulocytopenia in treatment-resistant scl	3	1.85E-06	AGPAT1, PBX2, NOTCH4
	Hematology traits	3	2.42E-06	PSORS1C1, CDSN, CCHCR1
	Carotid plaque	2	1.19E-05	CFDP1, TMEM170A
	rug-induced Stevens-Johnson syndrome or toxic epidermal necrolys	2	2.51E-05	PSORS1C1, POU5F1
	Cold sores	2	2.51E-05	POU5F1, HCG27
	Systemic sclerosis	3	3.79E-05	PSORS1C1, NOTCH4, HLA-DPB1
	Hepatitis B	2	4.29E-05	HLA-DPB1, UBE2L3
	Epstein-Barr virus immune response (EBNA-1)	2	4.29E-05	PSORS1C1, NOTCH4
	White blood cell count	4	4.83E-05	PSORS1C1, CDSN, CCHCR1, HCG27
	Chronic obstructive pulmonary disease or coronary artery disease	2	1.25E-04	CFDP1, TMEM170A
	Oleic acid (18:1n-9) levels	2	1.42E-04	PPT2, EGFL8, AGPAT1
	HIV-1 control	2	1.42E-04	CDSN, NOTCH4
	Response to hepatitis B vaccine	2	1.81E-04	PSORS1C1, HLA-DPB1
	Blood protein levels	8	1.88E-04	PSORS1C1, CDSN, HCG27, ATF6B, AGER, GPSM3, NOTCH4, HLA-DPB1
	Type 1 diabetes and autoimmune thyroid diseases	2	2.25E-04	PSORS1C1, NOTCH4
	Chronic obstructive pulmonary disease-related biomarkers	2	2.99E-04	PSORS1C1, CCHCR1
	Psoriasis	3	4.06E-04	CCHCR1, HCG27, UBE2L3
	Chronic obstructive pulmonary disease	3	4.53E-04	AGER, AMZ1, CFDP1
	Night sleep phenotypes	4	6.14E-04	PSORS1C1, CCHCR1, TCF19, POU5F1
	Feeling nervous	2	8.65E-04	PSORS1C1, CCHCR1
	Automobile speeding propensity	2	8.65E-04	CDSN, ATF6B

	Lung cancer	4	2.71E-04	HCG27, MSH5, NOTCH4, HLA-DRA
	HIV-1 control	2	3.56E-04	VARS, NOTCH4
	Oleic acid (18:1n-9) levels	2	3.56E-04	PPT2, EGFL8, AGPAT1
	Type 1 diabetes and autoimmune thyroid diseases	2	5.61E-04	PRRC2A, NOTCH4
VHD_IBS	Takayasu arteritis	2	1.34E-05	PRRC2A, MSH5
	Menopause (age at onset)	2	2.33E-05	PRRC2A, MSH5
VHD_UC	Ulcerative colitis	16	7.12E-23	PSORS1C1, CDSN, CCHCR1, TCF19, POU5F1, HCG27, ATF6B, PPT2, EGFL8, AGPAT1, RNF5, AGER, PBX2, GPSM3, NOTCH4, AMZ1, UBE2L3
	Inflammatory bowel disease	16	1.66E-19	PSORS1C1, CDSN, CCHCR1, TCF19, POU5F1, HCG27, ATF6B, PPT2, EGFL8, AGPAT1, RNF5, AGER, PBX2, GPSM3, NOTCH4, AMZ1, UBE2L3
	Autism spectrum disorder or schizophrenia	15	5.36E-19	PSORS1C1, CDSN, CCHCR1, TCF19, POU5F1, HCG27, ATF6B, PPT2, EGFL8, AGPAT1, RNF5, AGER, PBX2, GPSM3, NOTCH4, HLA-DPB1
	Prostate cancer	9	3.13E-11	CCHCR1, ATF6B, PPT2, EGFL8, AGPAT1, RNF5, AGER, PBX2, GPSM3, NOTCH4
	Stevens-Johnson syndrome and toxic epidermal necrolysis	4	4.08E-11	PSORS1C1, CCHCR1, TCF19, POU5F1
	Multiple myeloma	5	6.87E-11	PSORS1C1, CDSN, CCHCR1, TCF19, POU5F1
	oidosis (non-Lofgren's syndrome without extrapulmonary manifesta	5	1.02E-10	ATF6B, AGER, GPSM3, NOTCH4, HLA-DPB1
	Idiopathic membranous nephropathy	4	3.83E-10	PSORS1C1, TCF19, PPT2, EGFL8, RNF5
	Takayasu arteritis	5	1.14E-08	PSORS1C1, CDSN, HCG27, ATF6B, NOTCH4
	Advanced glycation end-product levels	3	1.02E-07	RNF5, AGER, NOTCH4
	Chronic hepatitis B infection	3	4.39E-07	TCF19, NOTCH4, HLA-DPB1
	-induced agranulocytosis/granulocytopenia in treatment-resistant scl	3	1.85E-06	AGPAT1, PBX2, NOTCH4
	Hematology traits	3	2.42E-06	PSORS1C1, CDSN, CCHCR1
	Carotid plaque	2	1.19E-05	CFDP1, TMEM170A
	rug-induced Stevens-Johnson syndrome or toxic epidermal necrolys	2	2.51E-05	PSORS1C1, POU5F1
	Cold sores	2	2.51E-05	POU5F1, HCG27
	Systemic sclerosis	3	3.79E-05	PSORS1C1, NOTCH4, HLA-DPB1
	Hepatitis B	2	4.29E-05	HLA-DPB1, UBE2L3
	Epstein-Barr virus immune response (EBNA-1)	2	4.29E-05	PSORS1C1, NOTCH4
	White blood cell count	4	4.83E-05	PSORS1C1, CDSN, CCHCR1, HCG27
	Chronic obstructive pulmonary disease or coronary artery disease	2	1.25E-04	CFDP1, TMEM170A
	Oleic acid (18:1n-9) levels	2	1.42E-04	PPT2, EGFL8, AGPAT1
	HIV-1 control	2	1.42E-04	CDSN, NOTCH4
	Response to hepatitis B vaccine	2	1.81E-04	PSORS1C1, HLA-DPB1
	Blood protein levels	8	1.88E-04	PSORS1C1, CDSN, HCG27, ATF6B, AGER, GPSM3, NOTCH4, HLA-DPB1
	Type 1 diabetes and autoimmune thyroid diseases	2	2.25E-04	PSORS1C1, NOTCH4
	Chronic obstructive pulmonary disease-related biomarkers	2	2.99E-04	PSORS1C1, CCHCR1
	Psoriasis	3	4.06E-04	CCHCR1, HCG27, UBE2L3
	Chronic obstructive pulmonary disease	3	4.53E-04	AGER, AMZ1, CFDP1
	Night sleep phenotypes	4	6.14E-04	PSORS1C1, CCHCR1, TCF19, POU5F1
	Feeling nervous	2	8.65E-04	PSORS1C1, CCHCR1
	Automobile speeding propensity	2	8.65E-04	CDSN, ATF6B

	Lung cancer	4	2.71E-04	HCG27, MSH5, NOTCH4, HLA-DRA
	HIV-1 control	2	3.56E-04	VARS, NOTCH4
	Oleic acid (18:1n-9) levels	2	3.56E-04	PPT2, EGFL8, AGPAT1
	Type 1 diabetes and autoimmune thyroid diseases	2	5.61E-04	PRRC2A, NOTCH4
VHD_IBS	Takayasu arteritis	2	1.34E-05	PRRC2A, MSH5
	Menopause (age at onset)	2	2.33E-05	PRRC2A, MSH5
VHD_UC	Ulcerative colitis	16	7.12E-23	PSORS1C1, CDSN, CCHCR1, TCF19, POU5F1, HCG27, ATF6B, PPT2, EGFL8, AGPAT1, RNF5, AGER, PBX2, GPSM3, NOTCH4, AMZ1, UBE2L3
	Inflammatory bowel disease	16	1.66E-19	PSORS1C1, CDSN, CCHCR1, TCF19, POU5F1, HCG27, ATF6B, PPT2, EGFL8, AGPAT1, RNF5, AGER, PBX2, GPSM3, NOTCH4, AMZ1, UBE2L3
	Autism spectrum disorder or schizophrenia	15	5.36E-19	PSORS1C1, CDSN, CCHCR1, TCF19, POU5F1, HCG27, ATF6B, PPT2, EGFL8, AGPAT1, RNF5, AGER, PBX2, GPSM3, NOTCH4, HLA-DPB1
	Prostate cancer	9	3.13E-11	CCHCR1, ATF6B, PPT2, EGFL8, AGPAT1, RNF5, AGER, PBX2, GPSM3, NOTCH4
	Stevens-Johnson syndrome and toxic epidermal necrolysis	4	4.08E-11	PSORS1C1, CCHCR1, TCF19, POU5F1
	Multiple myeloma	5	6.87E-11	PSORS1C1, CDSN, CCHCR1, TCF19, POU5F1
	ɔidosis (non-Lofgren's syndrome without extrapulmonary manifestat	5	1.02E-10	ATF6B, AGER, GPSM3, NOTCH4, HLA-DPB1
	Idiopathic membranous nephropathy	4	3.83E-10	PSORS1C1, TCF19, PPT2, EGFL8, RNF5
	Takayasu arteritis	5	1.14E-08	PSORS1C1, CDSN, HCG27, ATF6B, NOTCH4
	Advanced glycation end-product levels	3	1.02E-07	RNF5, AGER, NOTCH4
	Chronic hepatitis B infection	3	4.39E-07	TCF19, NOTCH4, HLA-DPB1
	-induced agranulocytosis/granulocytopenia in treatment-resistant scl	3	1.85E-06	AGPAT1, PBX2, NOTCH4
	Hematology traits	3	2.42E-06	PSORS1C1, CDSN, CCHCR1
	Carotid plaque	2	1.19E-05	CFDP1, TMEM170A
	rug-induced Stevens-Johnson syndrome or toxic epidermal necrolys	2	2.51E-05	PSORS1C1, POU5F1
	Cold sores	2	2.51E-05	POU5F1, HCG27
	Systemic sclerosis	3	3.79E-05	PSORS1C1, NOTCH4, HLA-DPB1
	Hepatitis B	2	4.29E-05	HLA-DPB1, UBE2L3
	Epstein-Barr virus immune response (EBNA-1)	2	4.29E-05	PSORS1C1, NOTCH4
	White blood cell count	4	4.83E-05	PSORS1C1, CDSN, CCHCR1, HCG27
	Chronic obstructive pulmonary disease or coronary artery disease	2	1.25E-04	CFDP1, TMEM170A
	Oleic acid (18:1n-9) levels	2	1.42E-04	PPT2, EGFL8, AGPAT1
	HIV-1 control	2	1.42E-04	CDSN, NOTCH4
	Response to hepatitis B vaccine	2	1.81E-04	PSORS1C1, HLA-DPB1
	Blood protein levels	8	1.88E-04	PSORS1C1, CDSN, HCG27, ATF6B, AGER, GPSM3, NOTCH4, HLA-DPB1
	Type 1 diabetes and autoimmune thyroid diseases	2	2.25E-04	PSORS1C1, NOTCH4
	Chronic obstructive pulmonary disease-related biomarkers	2	2.99E-04	PSORS1C1, CCHCR1
	Psoriasis	3	4.06E-04	CCHCR1, HCG27, UBE2L3
	Chronic obstructive pulmonary disease	3	4.53E-04	AGER, AMZ1, CFDP1
	Night sleep phenotypes	4	6.14E-04	PSORS1C1, CCHCR1, TCF19, POU5F1
	Feeling nervous	2	8.65E-04	PSORS1C1, CCHCR1
	Automobile speeding propensity	2	8.65E-04	CDSN, ATF6B

	Lung cancer	4	2.71E-04	HCG27, MSH5, NOTCH4, HLA-DRA
	HIV-1 control	2	3.56E-04	VARS, NOTCH4
	Oleic acid (18:1n-9) levels	2	3.56E-04	PPT2, EGFL8, AGPAT1
	Type 1 diabetes and autoimmune thyroid diseases	2	5.61E-04	PRRC2A, NOTCH4
VHD_IBS	Takayasu arteritis	2	1.34E-05	PRRC2A, MSH5
	Menopause (age at onset)	2	2.33E-05	PRRC2A, MSH5
VHD_UC	Ulcerative colitis	16	7.12E-23	PSORS1C1, CDSN, CCHCR1, TCF19, POU5F1, HCG27, ATF6B, PPT2, EGFL8, AGPAT1, RNF5, AGER, PBX2, GPSM3, NOTCH4, AMZ1, UBE2L3
	Inflammatory bowel disease	16	1.66E-19	PSORS1C1, CDSN, CCHCR1, TCF19, POU5F1, HCG27, ATF6B, PPT2, EGFL8, AGPAT1, RNF5, AGER, PBX2, GPSM3, NOTCH4, AMZ1, UBE2L3
	Autism spectrum disorder or schizophrenia	15	5.36E-19	PSORS1C1, CDSN, CCHCR1, TCF19, POU5F1, HCG27, ATF6B, PPT2, EGFL8, AGPAT1, RNF5, AGER, PBX2, GPSM3, NOTCH4, HLA-DPB1
	Prostate cancer	9	3.13E-11	CCHCR1, ATF6B, PPT2, EGFL8, AGPAT1, RNF5, AGER, PBX2, GPSM3, NOTCH4
	Stevens-Johnson syndrome and toxic epidermal necrolysis	4	4.08E-11	PSORS1C1, CCHCR1, TCF19, POU5F1
	Multiple myeloma	5	6.87E-11	PSORS1C1, CDSN, CCHCR1, TCF19, POU5F1
	idosis (non-Lofgren's syndrome without extrapulmonary manifesta	5	1.02E-10	ATF6B, AGER, GPSM3, NOTCH4, HLA-DPB1
	Idiopathic membranous nephropathy	4	3.83E-10	PSORS1C1, TCF19, PPT2, EGFL8, RNF5
	Takayasu arteritis	5	1.14E-08	PSORS1C1, CDSN, HCG27, ATF6B, NOTCH4
	Advanced glycation end-product levels	3	1.02E-07	RNF5, AGER, NOTCH4
	Chronic hepatitis B infection	3	4.39E-07	TCF19, NOTCH4, HLA-DPB1
	induced agranulocytosis/granulocytopenia in treatment-resistant scl	3	1.85E-06	AGPAT1, PBX2, NOTCH4
	Hematology traits	3	2.42E-06	PSORS1C1, CDSN, CCHCR1
	Carotid plaque	2	1.19E-05	CFDP1, TMEM170A
	rug-induced Stevens-Johnson syndrome or toxic epidermal necrolys	2	2.51E-05	PSORS1C1, POU5F1
	Cold sores	2	2.51E-05	POU5F1, HCG27
	Systemic sclerosis	3	3.79E-05	PSORS1C1, NOTCH4, HLA-DPB1
	Hepatitis B	2	4.29E-05	HLA-DPB1, UBE2L3
	Epstein-Barr virus immune response (EBNA-1)	2	4.29E-05	PSORS1C1, NOTCH4
	White blood cell count	4	4.83E-05	PSORS1C1, CDSN, CCHCR1, HCG27
	Chronic obstructive pulmonary disease or coronary artery disease	2	1.25E-04	CFDP1, TMEM170A
	Oleic acid (18:1n-9) levels	2	1.42E-04	PPT2, EGFL8, AGPAT1
	HIV-1 control	2	1.42E-04	CDSN, NOTCH4
	Response to hepatitis B vaccine	2	1.81E-04	PSORS1C1, HLA-DPB1
	Blood protein levels	8	1.88E-04	PSORS1C1, CDSN, HCG27, ATF6B, AGER, GPSM3, NOTCH4, HLA-DPB1
	Type 1 diabetes and autoimmune thyroid diseases	2	2.25E-04	PSORS1C1, NOTCH4
	Chronic obstructive pulmonary disease-related biomarkers	2	2.99E-04	PSORS1C1, CCHCR1
	Psoriasis	3	4.06E-04	CCHCR1, HCG27, UBE2L3
	Chronic obstructive pulmonary disease	3	4.53E-04	AGER, AMZ1, CFDP1
	Night sleep phenotypes	4	6.14E-04	PSORS1C1, CCHCR1, TCF19, POU5F1
	Feeling nervous	2	8.65E-04	PSORS1C1, CCHCR1
	Automobile speeding propensity	2	8.65E-04	CDSN, ATF6B

	Lung cancer	4	2.71E-04	HCG27, MSH5, NOTCH4, HLA-DRA
	HIV-1 control	2	3.56E-04	VARS, NOTCH4
	Oleic acid (18:1n-9) levels	2	3.56E-04	PPT2, EGFL8, AGPAT1
	Type 1 diabetes and autoimmune thyroid diseases	2	5.61E-04	PRRC2A, NOTCH4
VHD_IBS	Takayasu arteritis	2	1.34E-05	PRRC2A, MSH5
	Menopause (age at onset)	2	2.33E-05	PRRC2A, MSH5
VHD_UC	Ulcerative colitis	16	7.12E-23	PSORS1C1, CDSN, CCHCR1, TCF19, POU5F1, HCG27, ATF6B, PPT2, EGFL8, AGPAT1, RNF5, AGER, PBX2, GPSM3, NOTCH4, AMZ1, UBE2L3
	Inflammatory bowel disease	16	1.66E-19	PSORS1C1, CDSN, CCHCR1, TCF19, POU5F1, HCG27, ATF6B, PPT2, EGFL8, AGPAT1, RNF5, AGER, PBX2, GPSM3, NOTCH4, AMZ1, UBE2L3
	Autism spectrum disorder or schizophrenia	15	5.36E-19	PSORS1C1, CDSN, CCHCR1, TCF19, POU5F1, HCG27, ATF6B, PPT2, EGFL8, AGPAT1, RNF5, AGER, PBX2, GPSM3, NOTCH4, HLA-DPB1
	Prostate cancer	9	3.13E-11	CCHCR1, ATF6B, PPT2, EGFL8, AGPAT1, RNF5, AGER, PBX2, GPSM3, NOTCH4
	Stevens-Johnson syndrome and toxic epidermal necrolysis	4	4.08E-11	PSORS1C1, CCHCR1, TCF19, POU5F1
	Multiple myeloma	5	6.87E-11	PSORS1C1, CDSN, CCHCR1, TCF19, POU5F1
	idosis (non-Lofgren's syndrome without extrapulmonary manifesta	5	1.02E-10	ATF6B, AGER, GPSM3, NOTCH4, HLA-DPB1
	Idiopathic membranous nephropathy	4	3.83E-10	PSORS1C1, TCF19, PPT2, EGFL8, RNF5
	Takayasu arteritis	5	1.14E-08	PSORS1C1, CDSN, HCG27, ATF6B, NOTCH4
	Advanced glycation end-product levels	3	1.02E-07	RNF5, AGER, NOTCH4
	Chronic hepatitis B infection	3	4.39E-07	TCF19, NOTCH4, HLA-DPB1
	-induced agranulocytosis/granulocytopenia in treatment-resistant scl	3	1.85E-06	AGPAT1, PBX2, NOTCH4
	Hematology traits	3	2.42E-06	PSORS1C1, CDSN, CCHCR1
	Carotid plaque	2	1.19E-05	CFDP1, TMEM170A
	rug-induced Stevens-Johnson syndrome or toxic epidermal necrolys	2	2.51E-05	PSORS1C1, POU5F1
	Cold sores	2	2.51E-05	POU5F1, HCG27
	Systemic sclerosis	3	3.79E-05	PSORS1C1, NOTCH4, HLA-DPB1
	Hepatitis B	2	4.29E-05	HLA-DPB1, UBE2L3
	Epstein-Barr virus immune response (EBNA-1)	2	4.29E-05	PSORS1C1, NOTCH4
	White blood cell count	4	4.83E-05	PSORS1C1, CDSN, CCHCR1, HCG27
	Chronic obstructive pulmonary disease or coronary artery disease	2	1.25E-04	CFDP1, TMEM170A
	Oleic acid (18:1n-9) levels	2	1.42E-04	PPT2, EGFL8, AGPAT1
	HIV-1 control	2	1.42E-04	CDSN, NOTCH4
	Response to hepatitis B vaccine	2	1.81E-04	PSORS1C1, HLA-DPB1
	Blood protein levels	8	1.88E-04	PSORS1C1, CDSN, HCG27, ATF6B, AGER, GPSM3, NOTCH4, HLA-DPB1
	Type 1 diabetes and autoimmune thyroid diseases	2	2.25E-04	PSORS1C1, NOTCH4
	Chronic obstructive pulmonary disease-related biomarkers	2	2.99E-04	PSORS1C1, CCHCR1
	Psoriasis	3	4.06E-04	CCHCR1, HCG27, UBE2L3
	Chronic obstructive pulmonary disease	3	4.53E-04	AGER, AMZ1, CFDP1
	Night sleep phenotypes	4	6.14E-04	PSORS1C1, CCHCR1, TCF19, POU5F1
	Feeling nervous	2	8.65E-04	PSORS1C1, CCHCR1
	Automobile speeding propensity	2	8.65E-04	CDSN, ATF6B

	Lung cancer	4	2.71E-04	HCG27, MSH5, NOTCH4, HLA-DRA
	HIV-1 control	2	3.56E-04	VARS, NOTCH4
	Oleic acid (18:1n-9) levels	2	3.56E-04	PPT2, EGFL8, AGPAT1
	Type 1 diabetes and autoimmune thyroid diseases	2	5.61E-04	PRRC2A, NOTCH4
VHD_IBS	Takayasu arteritis	2	1.34E-05	PRRC2A, MSH5
	Menopause (age at onset)	2	2.33E-05	PRRC2A, MSH5
VHD_UC	Ulcerative colitis	16	7.12E-23	PSORS1C1, CDSN, CCHCR1, TCF19, POU5F1, HCG27, ATF6B, PPT2, EGFL8, AGPAT1, RNF5, AGER, PBX2, GPSM3, NOTCH4, AMZ1, UBE2L3
	Inflammatory bowel disease	16	1.66E-19	PSORS1C1, CDSN, CCHCR1, TCF19, POU5F1, HCG27, ATF6B, PPT2, EGFL8, AGPAT1, RNF5, AGER, PBX2, GPSM3, NOTCH4, AMZ1, UBE2L3
	Autism spectrum disorder or schizophrenia	15	5.36E-19	PSORS1C1, CDSN, CCHCR1, TCF19, POU5F1, HCG27, ATF6B, PPT2, EGFL8, AGPAT1, RNF5, AGER, PBX2, GPSM3, NOTCH4, HLA-DPB1
	Prostate cancer	9	3.13E-11	CCHCR1, ATF6B, PPT2, EGFL8, AGPAT1, RNF5, AGER, PBX2, GPSM3, NOTCH4
	Stevens-Johnson syndrome and toxic epidermal necrolysis	4	4.08E-11	PSORS1C1, CCHCR1, TCF19, POU5F1
	Multiple myeloma	5	6.87E-11	PSORS1C1, CDSN, CCHCR1, TCF19, POU5F1
	oidosis (non-Lofgren's syndrome without extrapulmonary manifesta	5	1.02E-10	ATF6B, AGER, GPSM3, NOTCH4, HLA-DPB1
	Idiopathic membranous nephropathy	4	3.83E-10	PSORS1C1, TCF19, PPT2, EGFL8, RNF5
	Takayasu arteritis	5	1.14E-08	PSORS1C1, CDSN, HCG27, ATF6B, NOTCH4
	Advanced glycation end-product levels	3	1.02E-07	RNF5, AGER, NOTCH4
	Chronic hepatitis B infection	3	4.39E-07	TCF19, NOTCH4, HLA-DPB1
	-induced agranulocytosis/granulocytopenia in treatment-resistant scl	3	1.85E-06	AGPAT1, PBX2, NOTCH4
	Hematology traits	3	2.42E-06	PSORS1C1, CDSN, CCHCR1
	Carotid plaque	2	1.19E-05	CFDP1, TMEM170A
	rug-induced Stevens-Johnson syndrome or toxic epidermal necrolys	2	2.51E-05	PSORS1C1, POU5F1
	Cold sores	2	2.51E-05	POU5F1, HCG27
	Systemic sclerosis	3	3.79E-05	PSORS1C1, NOTCH4, HLA-DPB1
	Hepatitis B	2	4.29E-05	HLA-DPB1, UBE2L3
	Epstein-Barr virus immune response (EBNA-1)	2	4.29E-05	PSORS1C1, NOTCH4
	White blood cell count	4	4.83E-05	PSORS1C1, CDSN, CCHCR1, HCG27
	Chronic obstructive pulmonary disease or coronary artery disease	2	1.25E-04	CFDP1, TMEM170A
	Oleic acid (18:1n-9) levels	2	1.42E-04	PPT2, EGFL8, AGPAT1
	HIV-1 control	2	1.42E-04	CDSN, NOTCH4
	Response to hepatitis B vaccine	2	1.81E-04	PSORS1C1, HLA-DPB1
	Blood protein levels	8	1.88E-04	PSORS1C1, CDSN, HCG27, ATF6B, AGER, GPSM3, NOTCH4, HLA-DPB1
	Type 1 diabetes and autoimmune thyroid diseases	2	2.25E-04	PSORS1C1, NOTCH4
	Chronic obstructive pulmonary disease-related biomarkers	2	2.99E-04	PSORS1C1, CCHCR1
	Psoriasis	3	4.06E-04	CCHCR1, HCG27, UBE2L3
	Chronic obstructive pulmonary disease	3	4.53E-04	AGER, AMZ1, CFDP1
	Night sleep phenotypes	4	6.14E-04	PSORS1C1, CCHCR1, TCF19, POU5F1
	Feeling nervous	2	8.65E-04	PSORS1C1, CCHCR1
	Automobile speeding propensity	2	8.65E-04	CDSN, ATF6B

	Lung cancer	4	2.71E-04	HCG27, MSH5, NOTCH4, HLA-DRA
	HIV-1 control	2	3.56E-04	VARS, NOTCH4
	Oleic acid (18:1n-9) levels	2	3.56E-04	PPT2, EGFL8, AGPAT1
	Type 1 diabetes and autoimmune thyroid diseases	2	5.61E-04	PRRC2A, NOTCH4
VHD_IBS	Takayasu arteritis	2	1.34E-05	PRRC2A, MSH5
	Menopause (age at onset)	2	2.33E-05	PRRC2A, MSH5
VHD_UC	Ulcerative colitis	16	7.12E-23	PSORS1C1, CDSN, CCHCR1, TCF19, POU5F1, HCG27, ATF6B, PPT2, EGFL8, AGPAT1, RNF5, AGER, PBX2, GPSM3, NOTCH4, AMZ1, UBE2L3
	Inflammatory bowel disease	16	1.66E-19	PSORS1C1, CDSN, CCHCR1, TCF19, POU5F1, HCG27, ATF6B, PPT2, EGFL8, AGPAT1, RNF5, AGER, PBX2, GPSM3, NOTCH4, AMZ1, UBE2L3
	Autism spectrum disorder or schizophrenia	15	5.36E-19	PSORS1C1, CDSN, CCHCR1, TCF19, POU5F1, HCG27, ATF6B, PPT2, EGFL8, AGPAT1, RNF5, AGER, PBX2, GPSM3, NOTCH4, HLA-DPB1
	Prostate cancer	9	3.13E-11	CCHCR1, ATF6B, PPT2, EGFL8, AGPAT1, RNF5, AGER, PBX2, GPSM3, NOTCH4
	Stevens-Johnson syndrome and toxic epidermal necrolysis	4	4.08E-11	PSORS1C1, CCHCR1, TCF19, POU5F1
	Multiple myeloma	5	6.87E-11	PSORS1C1, CDSN, CCHCR1, TCF19, POU5F1
	idosis (non-Lofgren's syndrome without extrapulmonary manifesta	5	1.02E-10	ATF6B, AGER, GPSM3, NOTCH4, HLA-DPB1
	Idiopathic membranous nephropathy	4	3.83E-10	PSORS1C1, TCF19, PPT2, EGFL8, RNF5
	Takayasu arteritis	5	1.14E-08	PSORS1C1, CDSN, HCG27, ATF6B, NOTCH4
	Advanced glycation end-product levels	3	1.02E-07	RNF5, AGER, NOTCH4
	Chronic hepatitis B infection	3	4.39E-07	TCF19, NOTCH4, HLA-DPB1
	-induced agranulocytosis/granulocytopenia in treatment-resistant scl	3	1.85E-06	AGPAT1, PBX2, NOTCH4
	Hematology traits	3	2.42E-06	PSORS1C1, CDSN, CCHCR1
	Carotid plaque	2	1.19E-05	CFDP1, TMEM170A
	rug-induced Stevens-Johnson syndrome or toxic epidermal necrolys	2	2.51E-05	PSORS1C1, POU5F1
	Cold sores	2	2.51E-05	POU5F1, HCG27
	Systemic sclerosis	3	3.79E-05	PSORS1C1, NOTCH4, HLA-DPB1
	Hepatitis B	2	4.29E-05	HLA-DPB1, UBE2L3
	Epstein-Barr virus immune response (EBNA-1)	2	4.29E-05	PSORS1C1, NOTCH4
	White blood cell count	4	4.83E-05	PSORS1C1, CDSN, CCHCR1, HCG27
	Chronic obstructive pulmonary disease or coronary artery disease	2	1.25E-04	CFDP1, TMEM170A
	Oleic acid (18:1n-9) levels	2	1.42E-04	PPT2, EGFL8, AGPAT1
	HIV-1 control	2	1.42E-04	CDSN, NOTCH4
	Response to hepatitis B vaccine	2	1.81E-04	PSORS1C1, HLA-DPB1
	Blood protein levels	8	1.88E-04	PSORS1C1, CDSN, HCG27, ATF6B, AGER, GPSM3, NOTCH4, HLA-DPB1
	Type 1 diabetes and autoimmune thyroid diseases	2	2.25E-04	PSORS1C1, NOTCH4
	Chronic obstructive pulmonary disease-related biomarkers	2	2.99E-04	PSORS1C1, CCHCR1
	Psoriasis	3	4.06E-04	CCHCR1, HCG27, UBE2L3
	Chronic obstructive pulmonary disease	3	4.53E-04	AGER, AMZ1, CFDP1
	Night sleep phenotypes	4	6.14E-04	PSORS1C1, CCHCR1, TCF19, POU5F1
	Feeling nervous	2	8.65E-04	PSORS1C1, CCHCR1
	Automobile speeding propensity	2	8.65E-04	CDSN, ATF6B

	Lung cancer	4	2.71E-04	HCG27, MSH5, NOTCH4, HLA-DRA
	HIV-1 control	2	3.56E-04	VARS, NOTCH4
	Oleic acid (18:1n-9) levels	2	3.56E-04	PPT2, EGFL8, AGPAT1
	Type 1 diabetes and autoimmune thyroid diseases	2	5.61E-04	PRRC2A, NOTCH4
VHD_IBS	Takayasu arteritis	2	1.34E-05	PRRC2A, MSH5
	Menopause (age at onset)	2	2.33E-05	PRRC2A, MSH5
VHD_UC	Ulcerative colitis	16	7.12E-23	PSORS1C1, CDSN, CCHCR1, TCF19, POU5F1, HCG27, ATF6B, PPT2, EGFL8, AGPAT1, RNF5, AGER, PBX2, GPSM3, NOTCH4, AMZ1, UBE2L3
	Inflammatory bowel disease	16	1.66E-19	PSORS1C1, CDSN, CCHCR1, TCF19, POU5F1, HCG27, ATF6B, PPT2, EGFL8, AGPAT1, RNF5, AGER, PBX2, GPSM3, NOTCH4, AMZ1, UBE2L3
	Autism spectrum disorder or schizophrenia	15	5.36E-19	PSORS1C1, CDSN, CCHCR1, TCF19, POU5F1, HCG27, ATF6B, PPT2, EGFL8, AGPAT1, RNF5, AGER, PBX2, GPSM3, NOTCH4, HLA-DPB1
	Prostate cancer	9	3.13E-11	CCHCR1, ATF6B, PPT2, EGFL8, AGPAT1, RNF5, AGER, PBX2, GPSM3, NOTCH4
	Stevens-Johnson syndrome and toxic epidermal necrolysis	4	4.08E-11	PSORS1C1, CCHCR1, TCF19, POU5F1
	Multiple myeloma	5	6.87E-11	PSORS1C1, CDSN, CCHCR1, TCF19, POU5F1
	idosis (non-Lofgren's syndrome without extrapulmonary manifesta	5	1.02E-10	ATF6B, AGER, GPSM3, NOTCH4, HLA-DPB1
	Idiopathic membranous nephropathy	4	3.83E-10	PSORS1C1, TCF19, PPT2, EGFL8, RNF5
	Takayasu arteritis	5	1.14E-08	PSORS1C1, CDSN, HCG27, ATF6B, NOTCH4
	Advanced glycation end-product levels	3	1.02E-07	RNF5, AGER, NOTCH4
	Chronic hepatitis B infection	3	4.39E-07	TCF19, NOTCH4, HLA-DPB1
	-induced agranulocytosis/granulocytopenia in treatment-resistant scl	3	1.85E-06	AGPAT1, PBX2, NOTCH4
	Hematology traits	3	2.42E-06	PSORS1C1, CDSN, CCHCR1
	Carotid plaque	2	1.19E-05	CFDP1, TMEM170A
	rug-induced Stevens-Johnson syndrome or toxic epidermal necrolys	2	2.51E-05	PSORS1C1, POU5F1
	Cold sores	2	2.51E-05	POU5F1, HCG27
	Systemic sclerosis	3	3.79E-05	PSORS1C1, NOTCH4, HLA-DPB1
	Hepatitis B	2	4.29E-05	HLA-DPB1, UBE2L3
	Epstein-Barr virus immune response (EBNA-1)	2	4.29E-05	PSORS1C1, NOTCH4
	White blood cell count	4	4.83E-05	PSORS1C1, CDSN, CCHCR1, HCG27
	Chronic obstructive pulmonary disease or coronary artery disease	2	1.25E-04	CFDP1, TMEM170A
	Oleic acid (18:1n-9) levels	2	1.42E-04	PPT2, EGFL8, AGPAT1
	HIV-1 control	2	1.42E-04	CDSN, NOTCH4
	Response to hepatitis B vaccine	2	1.81E-04	PSORS1C1, HLA-DPB1
	Blood protein levels	8	1.88E-04	PSORS1C1, CDSN, HCG27, ATF6B, AGER, GPSM3, NOTCH4, HLA-DPB1
	Type 1 diabetes and autoimmune thyroid diseases	2	2.25E-04	PSORS1C1, NOTCH4
	Chronic obstructive pulmonary disease-related biomarkers	2	2.99E-04	PSORS1C1, CCHCR1
	Psoriasis	3	4.06E-04	CCHCR1, HCG27, UBE2L3
	Chronic obstructive pulmonary disease	3	4.53E-04	AGER, AMZ1, CFDP1
	Night sleep phenotypes	4	6.14E-04	PSORS1C1, CCHCR1, TCF19, POU5F1
	Feeling nervous	2	8.65E-04	PSORS1C1, CCHCR1
	Automobile speeding propensity	2	8.65E-04	CDSN, ATF6B

	Lung cancer	4	2.71E-04	HCG27, MSH5, NOTCH4, HLA-DRA
	HIV-1 control	2	3.56E-04	VARS, NOTCH4
	Oleic acid (18:1n-9) levels	2	3.56E-04	PPT2, EGFL8, AGPAT1
	Type 1 diabetes and autoimmune thyroid diseases	2	5.61E-04	PRRC2A, NOTCH4
VHD_IBS	Takayasu arteritis	2	1.34E-05	PRRC2A, MSH5
	Menopause (age at onset)	2	2.33E-05	PRRC2A, MSH5
VHD_UC	Ulcerative colitis	16	7.12E-23	PSORS1C1, CDSN, CCHCR1, TCF19, POU5F1, HCG27, ATF6B, PPT2, EGFL8, AGPAT1, RNF5, AGER, PBX2, GPSM3, NOTCH4, AMZ1, UBE2L3
	Inflammatory bowel disease	16	1.66E-19	PSORS1C1, CDSN, CCHCR1, TCF19, POU5F1, HCG27, ATF6B, PPT2, EGFL8, AGPAT1, RNF5, AGER, PBX2, GPSM3, NOTCH4, AMZ1, UBE2L3
	Autism spectrum disorder or schizophrenia	15	5.36E-19	PSORS1C1, CDSN, CCHCR1, TCF19, POU5F1, HCG27, ATF6B, PPT2, EGFL8, AGPAT1, RNF5, AGER, PBX2, GPSM3, NOTCH4, HLA-DPB1
	Prostate cancer	9	3.13E-11	CCHCR1, ATF6B, PPT2, EGFL8, AGPAT1, RNF5, AGER, PBX2, GPSM3, NOTCH4
	Stevens-Johnson syndrome and toxic epidermal necrolysis	4	4.08E-11	PSORS1C1, CCHCR1, TCF19, POU5F1
	Multiple myeloma	5	6.87E-11	PSORS1C1, CDSN, CCHCR1, TCF19, POU5F1
	idosis (non-Lofgren's syndrome without extrapulmonary manifesta	5	1.02E-10	ATF6B, AGER, GPSM3, NOTCH4, HLA-DPB1
	Idiopathic membranous nephropathy	4	3.83E-10	PSORS1C1, TCF19, PPT2, EGFL8, RNF5
	Takayasu arteritis	5	1.14E-08	PSORS1C1, CDSN, HCG27, ATF6B, NOTCH4
	Advanced glycation end-product levels	3	1.02E-07	RNF5, AGER, NOTCH4
	Chronic hepatitis B infection	3	4.39E-07	TCF19, NOTCH4, HLA-DPB1
	-induced agranulocytosis/granulocytopenia in treatment-resistant scl	3	1.85E-06	AGPAT1, PBX2, NOTCH4
	Hematology traits	3	2.42E-06	PSORS1C1, CDSN, CCHCR1
	Carotid plaque	2	1.19E-05	CFDP1, TMEM170A
	rug-induced Stevens-Johnson syndrome or toxic epidermal necrolys	2	2.51E-05	PSORS1C1, POU5F1
	Cold sores	2	2.51E-05	POU5F1, HCG27
	Systemic sclerosis	3	3.79E-05	PSORS1C1, NOTCH4, HLA-DPB1
	Hepatitis B	2	4.29E-05	HLA-DPB1, UBE2L3
	Epstein-Barr virus immune response (EBNA-1)	2	4.29E-05	PSORS1C1, NOTCH4
	White blood cell count	4	4.83E-05	PSORS1C1, CDSN, CCHCR1, HCG27
	Chronic obstructive pulmonary disease or coronary artery disease	2	1.25E-04	CFDP1, TMEM170A
	Oleic acid (18:1n-9) levels	2	1.42E-04	PPT2, EGFL8, AGPAT1
	HIV-1 control	2	1.42E-04	CDSN, NOTCH4
	Response to hepatitis B vaccine	2	1.81E-04	PSORS1C1, HLA-DPB1
	Blood protein levels	8	1.88E-04	PSORS1C1, CDSN, HCG27, ATF6B, AGER, GPSM3, NOTCH4, HLA-DPB1
	Type 1 diabetes and autoimmune thyroid diseases	2	2.25E-04	PSORS1C1, NOTCH4
	Chronic obstructive pulmonary disease-related biomarkers	2	2.99E-04	PSORS1C1, CCHCR1
	Psoriasis	3	4.06E-04	CCHCR1, HCG27, UBE2L3
	Chronic obstructive pulmonary disease	3	4.53E-04	AGER, AMZ1, CFDP1
	Night sleep phenotypes	4	6.14E-04	PSORS1C1, CCHCR1, TCF19, POU5F1
	Feeling nervous	2	8.65E-04	PSORS1C1, CCHCR1
	Automobile speeding propensity	2	8.65E-04	CDSN, ATF6B

	Lung cancer	4	2.71E-04	HCG27, MSH5, NOTCH4, HLA-DRA
	HIV-1 control	2	3.56E-04	VARS, NOTCH4
	Oleic acid (18:1n-9) levels	2	3.56E-04	PPT2, EGFL8, AGPAT1
	Type 1 diabetes and autoimmune thyroid diseases	2	5.61E-04	PRRC2A, NOTCH4
VHD_IBS	Takayasu arteritis	2	1.34E-05	PRRC2A, MSH5
	Menopause (age at onset)	2	2.33E-05	PRRC2A, MSH5
VHD_UC	Ulcerative colitis	16	7.12E-23	PSORS1C1, CDSN, CCHCR1, TCF19, POU5F1, HCG27, ATF6B, PPT2, EGFL8, AGPAT1, RNF5, AGER, PBX2, GPSM3, NOTCH4, AMZ1, UBE2L3
	Inflammatory bowel disease	16	1.66E-19	PSORS1C1, CDSN, CCHCR1, TCF19, POU5F1, HCG27, ATF6B, PPT2, EGFL8, AGPAT1, RNF5, AGER, PBX2, GPSM3, NOTCH4, AMZ1, UBE2L3
	Autism spectrum disorder or schizophrenia	15	5.36E-19	PSORS1C1, CDSN, CCHCR1, TCF19, POU5F1, HCG27, ATF6B, PPT2, EGFL8, AGPAT1, RNF5, AGER, PBX2, GPSM3, NOTCH4, HLA-DPB1
	Prostate cancer	9	3.13E-11	CCHCR1, ATF6B, PPT2, EGFL8, AGPAT1, RNF5, AGER, PBX2, GPSM3, NOTCH4
	Stevens-Johnson syndrome and toxic epidermal necrolysis	4	4.08E-11	PSORS1C1, CCHCR1, TCF19, POU5F1
	Multiple myeloma	5	6.87E-11	PSORS1C1, CDSN, CCHCR1, TCF19, POU5F1
	oidosis (non-Lofgren's syndrome without extrapulmonary manifesta	5	1.02E-10	ATF6B, AGER, GPSM3, NOTCH4, HLA-DPB1
	Idiopathic membranous nephropathy	4	3.83E-10	PSORS1C1, TCF19, PPT2, EGFL8, RNF5
	Takayasu arteritis	5	1.14E-08	PSORS1C1, CDSN, HCG27, ATF6B, NOTCH4
	Advanced glycation end-product levels	3	1.02E-07	RNF5, AGER, NOTCH4
	Chronic hepatitis B infection	3	4.39E-07	TCF19, NOTCH4, HLA-DPB1
	-induced agranulocytosis/granulocytopenia in treatment-resistant scl	3	1.85E-06	AGPAT1, PBX2, NOTCH4
	Hematology traits	3	2.42E-06	PSORS1C1, CDSN, CCHCR1
	Carotid plaque	2	1.19E-05	CFDP1, TMEM170A
	rug-induced Stevens-Johnson syndrome or toxic epidermal necrolys	2	2.51E-05	PSORS1C1, POU5F1
	Cold sores	2	2.51E-05	POU5F1, HCG27
	Systemic sclerosis	3	3.79E-05	PSORS1C1, NOTCH4, HLA-DPB1
	Hepatitis B	2	4.29E-05	HLA-DPB1, UBE2L3
	Epstein-Barr virus immune response (EBNA-1)	2	4.29E-05	PSORS1C1, NOTCH4
	White blood cell count	4	4.83E-05	PSORS1C1, CDSN, CCHCR1, HCG27
	Chronic obstructive pulmonary disease or coronary artery disease	2	1.25E-04	CFDP1, TMEM170A
	Oleic acid (18:1n-9) levels	2	1.42E-04	PPT2, EGFL8, AGPAT1
	HIV-1 control	2	1.42E-04	CDSN, NOTCH4
	Response to hepatitis B vaccine	2	1.81E-04	PSORS1C1, HLA-DPB1
	Blood protein levels	8	1.88E-04	PSORS1C1, CDSN, HCG27, ATF6B, AGER, GPSM3, NOTCH4, HLA-DPB1
	Type 1 diabetes and autoimmune thyroid diseases	2	2.25E-04	PSORS1C1, NOTCH4
	Chronic obstructive pulmonary disease-related biomarkers	2	2.99E-04	PSORS1C1, CCHCR1
	Psoriasis	3	4.06E-04	CCHCR1, HCG27, UBE2L3
	Chronic obstructive pulmonary disease	3	4.53E-04	AGER, AMZ1, CFDP1
	Night sleep phenotypes	4	6.14E-04	PSORS1C1, CCHCR1, TCF19, POU5F1
	Feeling nervous	2	8.65E-04	PSORS1C1, CCHCR1
	Automobile speeding propensity	2	8.65E-04	CDSN, ATF6B

	Lung cancer	4	2.71E-04	HCG27, MSH5, NOTCH4, HLA-DRA
	HIV-1 control	2	3.56E-04	VARS, NOTCH4
	Oleic acid (18:1n-9) levels	2	3.56E-04	PPT2, EGFL8, AGPAT1
	Type 1 diabetes and autoimmune thyroid diseases	2	5.61E-04	PRRC2A, NOTCH4
VHD_IBS	Takayasu arteritis	2	1.34E-05	PRRC2A, MSH5
	Menopause (age at onset)	2	2.33E-05	PRRC2A, MSH5
VHD_UC	Ulcerative colitis	16	7.12E-23	PSORS1C1, CDSN, CCHCR1, TCF19, POU5F1, HCG27, ATF6B, PPT2, EGFL8, AGPAT1, RNF5, AGER, PBX2, GPSM3, NOTCH4, AMZ1, UBE2L3
	Inflammatory bowel disease	16	1.66E-19	PSORS1C1, CDSN, CCHCR1, TCF19, POU5F1, HCG27, ATF6B, PPT2, EGFL8, AGPAT1, RNF5, AGER, PBX2, GPSM3, NOTCH4, AMZ1, UBE2L3
	Autism spectrum disorder or schizophrenia	15	5.36E-19	PSORS1C1, CDSN, CCHCR1, TCF19, POU5F1, HCG27, ATF6B, PPT2, EGFL8, AGPAT1, RNF5, AGER, PBX2, GPSM3, NOTCH4, HLA-DPB1
	Prostate cancer	9	3.13E-11	CCHCR1, ATF6B, PPT2, EGFL8, AGPAT1, RNF5, AGER, PBX2, GPSM3, NOTCH4
	Stevens-Johnson syndrome and toxic epidermal necrolysis	4	4.08E-11	PSORS1C1, CCHCR1, TCF19, POU5F1
	Multiple myeloma	5	6.87E-11	PSORS1C1, CDSN, CCHCR1, TCF19, POU5F1
	idosis (non-Lofgren's syndrome without extrapulmonary manifesta	5	1.02E-10	ATF6B, AGER, GPSM3, NOTCH4, HLA-DPB1
	Idiopathic membranous nephropathy	4	3.83E-10	PSORS1C1, TCF19, PPT2, EGFL8, RNF5
	Takayasu arteritis	5	1.14E-08	PSORS1C1, CDSN, HCG27, ATF6B, NOTCH4
	Advanced glycation end-product levels	3	1.02E-07	RNF5, AGER, NOTCH4
	Chronic hepatitis B infection	3	4.39E-07	TCF19, NOTCH4, HLA-DPB1
	induced agranulocytosis/granulocytopenia in treatment-resistant scl	3	1.85E-06	AGPAT1, PBX2, NOTCH4
	Hematology traits	3	2.42E-06	PSORS1C1, CDSN, CCHCR1
	Carotid plaque	2	1.19E-05	CFDP1, TMEM170A
	rug-induced Stevens-Johnson syndrome or toxic epidermal necrolys	2	2.51E-05	PSORS1C1, POU5F1
	Cold sores	2	2.51E-05	POU5F1, HCG27
	Systemic sclerosis	3	3.79E-05	PSORS1C1, NOTCH4, HLA-DPB1
	Hepatitis B	2	4.29E-05	HLA-DPB1, UBE2L3
	Epstein-Barr virus immune response (EBNA-1)	2	4.29E-05	PSORS1C1, NOTCH4
	White blood cell count	4	4.83E-05	PSORS1C1, CDSN, CCHCR1, HCG27
	Chronic obstructive pulmonary disease or coronary artery disease	2	1.25E-04	CFDP1, TMEM170A
	Oleic acid (18:1n-9) levels	2	1.42E-04	PPT2, EGFL8, AGPAT1
	HIV-1 control	2	1.42E-04	CDSN, NOTCH4
	Response to hepatitis B vaccine	2	1.81E-04	PSORS1C1, HLA-DPB1
	Blood protein levels	8	1.88E-04	PSORS1C1, CDSN, HCG27, ATF6B, AGER, GPSM3, NOTCH4, HLA-DPB1
	Type 1 diabetes and autoimmune thyroid diseases	2	2.25E-04	PSORS1C1, NOTCH4
	Chronic obstructive pulmonary disease-related biomarkers	2	2.99E-04	PSORS1C1, CCHCR1
	Psoriasis	3	4.06E-04	CCHCR1, HCG27, UBE2L3
	Chronic obstructive pulmonary disease	3	4.53E-04	AGER, AMZ1, CFDP1
	Night sleep phenotypes	4	6.14E-04	PSORS1C1, CCHCR1, TCF19, POU5F1
	Feeling nervous	2	8.65E-04	PSORS1C1, CCHCR1
	Automobile speeding propensity	2	8.65E-04	CDSN, ATF6B

	Lung cancer	4	2.71E-04	HCG27, MSH5, NOTCH4, HLA-DRA
	HIV-1 control	2	3.56E-04	VARS, NOTCH4
	Oleic acid (18:1n-9) levels	2	3.56E-04	PPT2, EGFL8, AGPAT1
	Type 1 diabetes and autoimmune thyroid diseases	2	5.61E-04	PRRC2A, NOTCH4
VHD_IBS	Takayasu arteritis	2	1.34E-05	PRRC2A, MSH5
	Menopause (age at onset)	2	2.33E-05	PRRC2A, MSH5
VHD_UC	Ulcerative colitis	16	7.12E-23	PSORS1C1, CDSN, CCHCR1, TCF19, POU5F1, HCG27, ATF6B, PPT2, EGFL8, AGPAT1, RNF5, AGER, PBX2, GPSM3, NOTCH4, AMZ1, UBE2L3
	Inflammatory bowel disease	16	1.66E-19	PSORS1C1, CDSN, CCHCR1, TCF19, POU5F1, HCG27, ATF6B, PPT2, EGFL8, AGPAT1, RNF5, AGER, PBX2, GPSM3, NOTCH4, AMZ1, UBE2L3
	Autism spectrum disorder or schizophrenia	15	5.36E-19	PSORS1C1, CDSN, CCHCR1, TCF19, POU5F1, HCG27, ATF6B, PPT2, EGFL8, AGPAT1, RNF5, AGER, PBX2, GPSM3, NOTCH4, HLA-DPB1
	Prostate cancer	9	3.13E-11	CCHCR1, ATF6B, PPT2, EGFL8, AGPAT1, RNF5, AGER, PBX2, GPSM3, NOTCH4
	Stevens-Johnson syndrome and toxic epidermal necrolysis	4	4.08E-11	PSORS1C1, CCHCR1, TCF19, POU5F1
	Multiple myeloma	5	6.87E-11	PSORS1C1, CDSN, CCHCR1, TCF19, POU5F1
	ɔidosis (non-Lofgren's syndrome without extrapulmonary manifestat	5	1.02E-10	ATF6B, AGER, GPSM3, NOTCH4, HLA-DPB1
	Idiopathic membranous nephropathy	4	3.83E-10	PSORS1C1, TCF19, PPT2, EGFL8, RNF5
	Takayasu arteritis	5	1.14E-08	PSORS1C1, CDSN, HCG27, ATF6B, NOTCH4
	Advanced glycation end-product levels	3	1.02E-07	RNF5, AGER, NOTCH4
	Chronic hepatitis B infection	3	4.39E-07	TCF19, NOTCH4, HLA-DPB1
	-induced agranulocytosis/granulocytopenia in treatment-resistant scl	3	1.85E-06	AGPAT1, PBX2, NOTCH4
	Hematology traits	3	2.42E-06	PSORS1C1, CDSN, CCHCR1
	Carotid plaque	2	1.19E-05	CFDP1, TMEM170A
	rug-induced Stevens-Johnson syndrome or toxic epidermal necrolys	2	2.51E-05	PSORS1C1, POU5F1
	Cold sores	2	2.51E-05	POU5F1, HCG27
	Systemic sclerosis	3	3.79E-05	PSORS1C1, NOTCH4, HLA-DPB1
	Hepatitis B	2	4.29E-05	HLA-DPB1, UBE2L3
	Epstein-Barr virus immune response (EBNA-1)	2	4.29E-05	PSORS1C1, NOTCH4
	White blood cell count	4	4.83E-05	PSORS1C1, CDSN, CCHCR1, HCG27
	Chronic obstructive pulmonary disease or coronary artery disease	2	1.25E-04	CFDP1, TMEM170A
	Oleic acid (18:1n-9) levels	2	1.42E-04	PPT2, EGFL8, AGPAT1
	HIV-1 control	2	1.42E-04	CDSN, NOTCH4
	Response to hepatitis B vaccine	2	1.81E-04	PSORS1C1, HLA-DPB1
	Blood protein levels	8	1.88E-04	PSORS1C1, CDSN, HCG27, ATF6B, AGER, GPSM3, NOTCH4, HLA-DPB1
	Type 1 diabetes and autoimmune thyroid diseases	2	2.25E-04	PSORS1C1, NOTCH4
	Chronic obstructive pulmonary disease-related biomarkers	2	2.99E-04	PSORS1C1, CCHCR1
	Psoriasis	3	4.06E-04	CCHCR1, HCG27, UBE2L3
	Chronic obstructive pulmonary disease	3	4.53E-04	AGER, AMZ1, CFDP1
	Night sleep phenotypes	4	6.14E-04	PSORS1C1, CCHCR1, TCF19, POU5F1
	Feeling nervous	2	8.65E-04	PSORS1C1, CCHCR1
	Automobile speeding propensity	2	8.65E-04	CDSN, ATF6B

	Lung cancer	4	2.71E-04	HCG27, MSH5, NOTCH4, HLA-DRA
	HIV-1 control	2	3.56E-04	VARS, NOTCH4
	Oleic acid (18:1n-9) levels	2	3.56E-04	PPT2, EGFL8, AGPAT1
	Type 1 diabetes and autoimmune thyroid diseases	2	5.61E-04	PRRC2A, NOTCH4
VHD_IBS	Takayasu arteritis	2	1.34E-05	PRRC2A, MSH5
	Menopause (age at onset)	2	2.33E-05	PRRC2A, MSH5
VHD_UC	Ulcerative colitis	16	7.12E-23	PSORS1C1, CDSN, CCHCR1, TCF19, POU5F1, HCG27, ATF6B, PPT2, EGFL8, AGPAT1, RNF5, AGER, PBX2, GPSM3, NOTCH4, AMZ1, UBE2L3
	Inflammatory bowel disease	16	1.66E-19	PSORS1C1, CDSN, CCHCR1, TCF19, POU5F1, HCG27, ATF6B, PPT2, EGFL8, AGPAT1, RNF5, AGER, PBX2, GPSM3, NOTCH4, AMZ1, UBE2L3
	Autism spectrum disorder or schizophrenia	15	5.36E-19	PSORS1C1, CDSN, CCHCR1, TCF19, POU5F1, HCG27, ATF6B, PPT2, EGFL8, AGPAT1, RNF5, AGER, PBX2, GPSM3, NOTCH4, HLA-DPB1
	Prostate cancer	9	3.13E-11	CCHCR1, ATF6B, PPT2, EGFL8, AGPAT1, RNF5, AGER, PBX2, GPSM3, NOTCH4
	Stevens-Johnson syndrome and toxic epidermal necrolysis	4	4.08E-11	PSORS1C1, CCHCR1, TCF19, POU5F1
	Multiple myeloma	5	6.87E-11	PSORS1C1, CDSN, CCHCR1, TCF19, POU5F1
	idosis (non-Lofgren's syndrome without extrapulmonary manifesta	5	1.02E-10	ATF6B, AGER, GPSM3, NOTCH4, HLA-DPB1
	Idiopathic membranous nephropathy	4	3.83E-10	PSORS1C1, TCF19, PPT2, EGFL8, RNF5
	Takayasu arteritis	5	1.14E-08	PSORS1C1, CDSN, HCG27, ATF6B, NOTCH4
	Advanced glycation end-product levels	3	1.02E-07	RNF5, AGER, NOTCH4
	Chronic hepatitis B infection	3	4.39E-07	TCF19, NOTCH4, HLA-DPB1
	induced agranulocytosis/granulocytopenia in treatment-resistant scl	3	1.85E-06	AGPAT1, PBX2, NOTCH4
	Hematology traits	3	2.42E-06	PSORS1C1, CDSN, CCHCR1
	Carotid plaque	2	1.19E-05	CFDP1, TMEM170A
	rug-induced Stevens-Johnson syndrome or toxic epidermal necrolys	2	2.51E-05	PSORS1C1, POU5F1
	Cold sores	2	2.51E-05	POU5F1, HCG27
	Systemic sclerosis	3	3.79E-05	PSORS1C1, NOTCH4, HLA-DPB1
	Hepatitis B	2	4.29E-05	HLA-DPB1, UBE2L3
	Epstein-Barr virus immune response (EBNA-1)	2	4.29E-05	PSORS1C1, NOTCH4
	White blood cell count	4	4.83E-05	PSORS1C1, CDSN, CCHCR1, HCG27
	Chronic obstructive pulmonary disease or coronary artery disease	2	1.25E-04	CFDP1, TMEM170A
	Oleic acid (18:1n-9) levels	2	1.42E-04	PPT2, EGFL8, AGPAT1
	HIV-1 control	2	1.42E-04	CDSN, NOTCH4
	Response to hepatitis B vaccine	2	1.81E-04	PSORS1C1, HLA-DPB1
	Blood protein levels	8	1.88E-04	PSORS1C1, CDSN, HCG27, ATF6B, AGER, GPSM3, NOTCH4, HLA-DPB1
	Type 1 diabetes and autoimmune thyroid diseases	2	2.25E-04	PSORS1C1, NOTCH4
	Chronic obstructive pulmonary disease-related biomarkers	2	2.99E-04	PSORS1C1, CCHCR1
	Psoriasis	3	4.06E-04	CCHCR1, HCG27, UBE2L3
	Chronic obstructive pulmonary disease	3	4.53E-04	AGER, AMZ1, CFDP1
	Night sleep phenotypes	4	6.14E-04	PSORS1C1, CCHCR1, TCF19, POU5F1
	Feeling nervous	2	8.65E-04	PSORS1C1, CCHCR1
	Automobile speeding propensity	2	8.65E-04	CDSN, ATF6B

	Lung cancer	4	2.71E-04	HCG27, MSH5, NOTCH4, HLA-DRA
	HIV-1 control	2	3.56E-04	VARS, NOTCH4
	Oleic acid (18:1n-9) levels	2	3.56E-04	PPT2, EGFL8, AGPAT1
	Type 1 diabetes and autoimmune thyroid diseases	2	5.61E-04	PRRC2A, NOTCH4
VHD_IBS	Takayasu arteritis	2	1.34E-05	PRRC2A, MSH5
	Menopause (age at onset)	2	2.33E-05	PRRC2A, MSH5
VHD_UC	Ulcerative colitis	16	7.12E-23	PSORS1C1, CDSN, CCHCR1, TCF19, POU5F1, HCG27, ATF6B, PPT2, EGFL8, AGPAT1, RNF5, AGER, PBX2, GPSM3, NOTCH4, AMZ1, UBE2L3
	Inflammatory bowel disease	16	1.66E-19	PSORS1C1, CDSN, CCHCR1, TCF19, POU5F1, HCG27, ATF6B, PPT2, EGFL8, AGPAT1, RNF5, AGER, PBX2, GPSM3, NOTCH4, AMZ1, UBE2L3
	Autism spectrum disorder or schizophrenia	15	5.36E-19	PSORS1C1, CDSN, CCHCR1, TCF19, POU5F1, HCG27, ATF6B, PPT2, EGFL8, AGPAT1, RNF5, AGER, PBX2, GPSM3, NOTCH4, HLA-DPB1
	Prostate cancer	9	3.13E-11	CCHCR1, ATF6B, PPT2, EGFL8, AGPAT1, RNF5, AGER, PBX2, GPSM3, NOTCH4
	Stevens-Johnson syndrome and toxic epidermal necrolysis	4	4.08E-11	PSORS1C1, CCHCR1, TCF19, POU5F1
	Multiple myeloma	5	6.87E-11	PSORS1C1, CDSN, CCHCR1, TCF19, POU5F1
	›idosis (non-Lofgren's syndrome without extrapulmonary manifesta	5	1.02E-10	ATF6B, AGER, GPSM3, NOTCH4, HLA-DPB1
	Idiopathic membranous nephropathy	4	3.83E-10	PSORS1C1, TCF19, PPT2, EGFL8, RNF5
	Takayasu arteritis	5	1.14E-08	PSORS1C1, CDSN, HCG27, ATF6B, NOTCH4
	Advanced glycation end-product levels	3	1.02E-07	RNF5, AGER, NOTCH4
	Chronic hepatitis B infection	3	4.39E-07	TCF19, NOTCH4, HLA-DPB1
	-induced agranulocytosis/granulocytopenia in treatment-resistant scl	3	1.85E-06	AGPAT1, PBX2, NOTCH4
	Hematology traits	3	2.42E-06	PSORS1C1, CDSN, CCHCR1
	Carotid plaque	2	1.19E-05	CFDP1, TMEM170A
	rug-induced Stevens-Johnson syndrome or toxic epidermal necrolys	2	2.51E-05	PSORS1C1, POU5F1
	Cold sores	2	2.51E-05	POU5F1, HCG27
	Systemic sclerosis	3	3.79E-05	PSORS1C1, NOTCH4, HLA-DPB1
	Hepatitis B	2	4.29E-05	HLA-DPB1, UBE2L3
	Epstein-Barr virus immune response (EBNA-1)	2	4.29E-05	PSORS1C1, NOTCH4
	White blood cell count	4	4.83E-05	PSORS1C1, CDSN, CCHCR1, HCG27
	Chronic obstructive pulmonary disease or coronary artery disease	2	1.25E-04	CFDP1, TMEM170A
	Oleic acid (18:1n-9) levels	2	1.42E-04	PPT2, EGFL8, AGPAT1
	HIV-1 control	2	1.42E-04	CDSN, NOTCH4
	Response to hepatitis B vaccine	2	1.81E-04	PSORS1C1, HLA-DPB1
	Blood protein levels	8	1.88E-04	PSORS1C1, CDSN, HCG27, ATF6B, AGER, GPSM3, NOTCH4, HLA-DPB1
	Type 1 diabetes and autoimmune thyroid diseases	2	2.25E-04	PSORS1C1, NOTCH4
	Chronic obstructive pulmonary disease-related biomarkers	2	2.99E-04	PSORS1C1, CCHCR1
	Psoriasis	3	4.06E-04	CCHCR1, HCG27, UBE2L3
	Chronic obstructive pulmonary disease	3	4.53E-04	AGER, AMZ1, CFDP1
	Night sleep phenotypes	4	6.14E-04	PSORS1C1, CCHCR1, TCF19, POU5F1
	Feeling nervous	2	8.65E-04	PSORS1C1, CCHCR1
	Automobile speeding propensity	2	8.65E-04	CDSN, ATF6B

Supplementary table 6. Results of the summary-based Mendelian randomization integrating whole-blood expression quantitative trait loci. For each disease-gene association, we report the strongest effect observed between GTEX and eQTLGen datasets.

A) Bonferroni-significant disease-gene association with HEIDI P > 0.05.

Disease	Gene	chr	gene_bp	topSNP	topSNP_bp	A1	A2	Freq	b_SMR	se_SMR	p_SMR	p_HEIDI
CD	DNMT3A	2	25510652	rs7578575	25488819	A	T	0.329	-0.834	0.164	3.51E-07	0.383
IBD	DNMT3A	2	25510652	rs7578575	25488819	A	T	0.329	-0.745	0.130	9.22E-09	0.515
UC	DNMT3A	2	25510652	rs7578575	25488819	A	T	0.329	-0.627	0.156	5.73E-05	0.541
UC	CFDP1	16	75327596	rs9934007	75390532	C	T	0.754	0.273	0.070	9.61E-05	0.973
CD	STAT3	17	40502964	rs1053004	40466092	G	A	0.380	0.702	0.128	4.40E-08	0.056
CD	IMPDH2	3	49064299	rs9813813	49207093	A	T	0.110	-1.233	0.294	2.74E-05	0.213
IBD	IMPDH2	3	49064299	rs9813813	49207093	A	T	0.110	-1.328	0.258	2.57E-07	0.071
UC	IMPDH2	3	49064299	rs9813813	49207093	A	T	0.110	-1.445	0.307	2.53E-06	0.050
IBD	UBE2L3	22	21941029	rs2070512	21949411	C	A	0.194	0.163	0.021	1.06E-14	0.169
UC	UBE2L3	22	21941029	rs2070512	21949411	C	A	0.194	0.116	0.027	1.76E-05	0.698
CD	BCL2L15	1	1.14E+08	rs1217393	1.14E+08	A	G	0.271	-0.705	0.170	3.39E-05	0.184
CD	STMN3	20	62271061	rs909333	62270637	G	T	0.207	0.547	0.102	8.31E-08	0.200
IBD	STMN3	20	62271061	rs909333	62270637	G	T	0.207	0.555	0.087	2.01E-10	0.055
UC	STMN3	20	62271061	rs909333	62270637	G	T	0.207	0.498	0.098	4.04E-07	0.062
GORD	PBX2	6	32154964	rs693906	31835164	C	G	0.113	0.154	0.036	1.65E-05	0.058
VHD	AGER	6	32150423	rs693906	31835164	C	G	0.113	0.212	0.052	4.82E-05	0.092

B) Nominally significant effect on VHD of Bonferroni-significant gastrointestinal loci.

Disease	Gene	chr	Probe_bp	topSNP	topSNP_bp	A1	A2	Freq	b_SMR	se_SMR	p_SMR	p_HEIDI
VHD	CFDP1	16	75397489	rs7201989	75471499	G	A	0.246	-0.056	0.018	0.001	0.119
VHD	STAT3	17	40502964	rs1053004	40466092	G	A	0.380	0.093	0.047	0.048	0.948
VHD	IMPDH2	3	49064299	rs34037363	48821036	T	C	0.114	-0.209	0.105	0.047	0.089
VHD	UBE2L3	22	21941029	rs2070512	21949411	C	A	0.194	-0.029	0.009	0.002	0.623
VHD	STMN3	20	62271061	rs909333	62270637	G	T	0.207	-0.076	0.036	0.036	0.093

C) Effect of AGER gene on VHD and gastrointestinal disorders.

Disease	Gene	chr	Probe_bp	topSNP	topSNP_bp	A1	A2	Freq	b_SMR	se_SMR	p_SMR	p_HEIDI
CD	AGER	6	32150423	rs693906	31835164	C	G	0.113	-0.312	0.120	0.009	6.16E-08
GORD	AGER	6	32150423	rs693906	31835164	C	G	0.113	-0.209	0.049	2.02E-05	0.022
IBD	AGER	6	32148745	rs204995	32154285	G	A	0.217	-0.252	0.108	0.020	1.60E-04
IBS	AGER	6	32150423	rs693906	31835164	C	G	0.113	-0.168	0.052	0.001	0.045
UC	AGER	6	32148745	rs204995	32154285	G	A	0.217	-0.870	0.185	2.59E-06	0.002
VHD	AGER	6	32150423	rs693906	31835164	C	G	0.113	0.212	0.052	4.82E-05	0.092

Supplementary table 6. Results of the summary-based Mendelian randomization integrating whole-blood expression quantitative trait loci. For each disease-gene association, we report the strongest effect observed between GTEX and eQTLGen datasets.

A) Bonferroni-significant disease-gene association with HEIDI P > 0.05.

Disease	Gene	chr	gene_bp	topSNP	topSNP_bp	A1	A2	Freq	b_SMR	se_SMR	p_SMR	p_HEIDI
CD	DNMT3A	2	25510652	rs7578575	25488819	A	T	0.329	-0.834	0.164	3.51E-07	0.383
IBD	DNMT3A	2	25510652	rs7578575	25488819	A	T	0.329	-0.745	0.130	9.22E-09	0.515
UC	DNMT3A	2	25510652	rs7578575	25488819	A	T	0.329	-0.627	0.156	5.73E-05	0.541
UC	CFDP1	16	75327596	rs9934007	75390532	C	T	0.754	0.273	0.070	9.61E-05	0.973
CD	STAT3	17	40502964	rs1053004	40466092	G	A	0.380	0.702	0.128	4.40E-08	0.056
CD	IMPDH2	3	49064299	rs9813813	49207093	A	T	0.110	-1.233	0.294	2.74E-05	0.213
IBD	IMPDH2	3	49064299	rs9813813	49207093	A	T	0.110	-1.328	0.258	2.57E-07	0.071
UC	IMPDH2	3	49064299	rs9813813	49207093	A	T	0.110	-1.445	0.307	2.53E-06	0.050
IBD	UBE2L3	22	21941029	rs2070512	21949411	C	A	0.194	0.163	0.021	1.06E-14	0.169
UC	UBE2L3	22	21941029	rs2070512	21949411	C	A	0.194	0.116	0.027	1.76E-05	0.698
CD	BCL2L15	1	1.14E+08	rs1217393	1.14E+08	A	G	0.271	-0.705	0.170	3.39E-05	0.184
CD	STMN3	20	62271061	rs909333	62270637	G	T	0.207	0.547	0.102	8.31E-08	0.200
IBD	STMN3	20	62271061	rs909333	62270637	G	T	0.207	0.555	0.087	2.01E-10	0.055
UC	STMN3	20	62271061	rs909333	62270637	G	T	0.207	0.498	0.098	4.04E-07	0.062
GORD	PBX2	6	32154964	rs693906	31835164	C	G	0.113	0.154	0.036	1.65E-05	0.058
VHD	AGER	6	32150423	rs693906	31835164	C	G	0.113	0.212	0.052	4.82E-05	0.092

B) Nominally significant effect on VHD of Bonferroni-significant gastrointestinal loci.

Disease	Gene	chr	Probe_bp	topSNP	topSNP_bp	A1	A2	Freq	b_SMR	se_SMR	p_SMR	p_HEIDI
VHD	CFDP1	16	75397489	rs7201989	75471499	G	A	0.246	-0.056	0.018	0.001	0.119
VHD	STAT3	17	40502964	rs1053004	40466092	G	A	0.380	0.093	0.047	0.048	0.948
VHD	IMPDH2	3	49064299	rs34037363	48821036	T	C	0.114	-0.209	0.105	0.047	0.089
VHD	UBE2L3	22	21941029	rs2070512	21949411	C	A	0.194	-0.029	0.009	0.002	0.623
VHD	STMN3	20	62271061	rs909333	62270637	G	T	0.207	-0.076	0.036	0.036	0.093

C) Effect of AGER gene on VHD and gastrointestinal disorders.

Disease	Gene	chr	Probe_bp	topSNP	topSNP_bp	A1	A2	Freq	b_SMR	se_SMR	p_SMR	p_HEIDI
CD	AGER	6	32150423	rs693906	31835164	C	G	0.113	-0.312	0.120	0.009	6.16E-08
GORD	AGER	6	32150423	rs693906	31835164	C	G	0.113	-0.209	0.049	2.02E-05	0.022
IBD	AGER	6	32148745	rs204995	32154285	G	A	0.217	-0.252	0.108	0.020	1.60E-04
IBS	AGER	6	32150423	rs693906	31835164	C	G	0.113	-0.168	0.052	0.001	0.045
UC	AGER	6	32148745	rs204995	32154285	G	A	0.217	-0.870	0.185	2.59E-06	0.002
VHD	AGER	6	32150423	rs693906	31835164	C	G	0.113	0.212	0.052	4.82E-05	0.092

Supplementary table 6. Results of the summary-based Mendelian randomization integrating whole-blood expression quantitative trait loci. For each disease-gene association, we report the strongest effect observed between GTEX and eQTLGen datasets.

A) Bonferroni-significant disease-gene association with HEIDI P > 0.05.

Disease	Gene	chr	gene_bp	topSNP	topSNP_bp	A1	A2	Freq	b_SMR	se_SMR	p_SMR	p_HEIDI
CD	DNMT3A	2	25510652	rs7578575	25488819	A	T	0.329	-0.834	0.164	3.51E-07	0.383
IBD	DNMT3A	2	25510652	rs7578575	25488819	A	T	0.329	-0.745	0.130	9.22E-09	0.515
UC	DNMT3A	2	25510652	rs7578575	25488819	A	T	0.329	-0.627	0.156	5.73E-05	0.541
UC	CFDP1	16	75327596	rs9934007	75390532	C	T	0.754	0.273	0.070	9.61E-05	0.973
CD	STAT3	17	40502964	rs1053004	40466092	G	A	0.380	0.702	0.128	4.40E-08	0.056
CD	IMPDH2	3	49064299	rs9813813	49207093	A	T	0.110	-1.233	0.294	2.74E-05	0.213
IBD	IMPDH2	3	49064299	rs9813813	49207093	A	T	0.110	-1.328	0.258	2.57E-07	0.071
UC	IMPDH2	3	49064299	rs9813813	49207093	A	T	0.110	-1.445	0.307	2.53E-06	0.050
IBD	UBE2L3	22	21941029	rs2070512	21949411	C	A	0.194	0.163	0.021	1.06E-14	0.169
UC	UBE2L3	22	21941029	rs2070512	21949411	C	A	0.194	0.116	0.027	1.76E-05	0.698
CD	BCL2L15	1	1.14E+08	rs1217393	1.14E+08	A	G	0.271	-0.705	0.170	3.39E-05	0.184
CD	STMN3	20	62271061	rs909333	62270637	G	T	0.207	0.547	0.102	8.31E-08	0.200
IBD	STMN3	20	62271061	rs909333	62270637	G	T	0.207	0.555	0.087	2.01E-10	0.055
UC	STMN3	20	62271061	rs909333	62270637	G	T	0.207	0.498	0.098	4.04E-07	0.062
GORD	PBX2	6	32154964	rs693906	31835164	C	G	0.113	0.154	0.036	1.65E-05	0.058
VHD	AGER	6	32150423	rs693906	31835164	C	G	0.113	0.212	0.052	4.82E-05	0.092

B) Nominally significant effect on VHD of Bonferroni-significant gastrointestinal loci.

Disease	Gene	chr	Probe_bp	topSNP	topSNP_bp	A1	A2	Freq	b_SMR	se_SMR	p_SMR	p_HEIDI
VHD	CFDP1	16	75397489	rs7201989	75471499	G	A	0.246	-0.056	0.018	0.001	0.119
VHD	STAT3	17	40502964	rs1053004	40466092	G	A	0.380	0.093	0.047	0.048	0.948
VHD	IMPDH2	3	49064299	rs34037363	48821036	T	C	0.114	-0.209	0.105	0.047	0.089
VHD	UBE2L3	22	21941029	rs2070512	21949411	C	A	0.194	-0.029	0.009	0.002	0.623
VHD	STMN3	20	62271061	rs909333	62270637	G	T	0.207	-0.076	0.036	0.036	0.093

C) Effect of AGER gene on VHD and gastrointestinal disorders.

Disease	Gene	chr	Probe_bp	topSNP	topSNP_bp	A1	A2	Freq	b_SMR	se_SMR	p_SMR	p_HEIDI
CD	AGER	6	32150423	rs693906	31835164	C	G	0.113	-0.312	0.120	0.009	6.16E-08
GORD	AGER	6	32150423	rs693906	31835164	C	G	0.113	-0.209	0.049	2.02E-05	0.022
IBD	AGER	6	32148745	rs204995	32154285	G	A	0.217	-0.252	0.108	0.020	1.60E-04
IBS	AGER	6	32150423	rs693906	31835164	C	G	0.113	-0.168	0.052	0.001	0.045
UC	AGER	6	32148745	rs204995	32154285	G	A	0.217	-0.870	0.185	2.59E-06	0.002
VHD	AGER	6	32150423	rs693906	31835164	C	G	0.113	0.212	0.052	4.82E-05	0.092

Supplementary table 6. Results of the summary-based Mendelian randomization integrating whole-blood expression quantitative trait loci. For each disease-gene association, we report the strongest effect observed between GTEX and eQTLGen datasets.

A) Bonferroni-significant disease-gene association with HEIDI P > 0.05.

Disease	Gene	chr	gene_bp	topSNP	topSNP_bp	A1	A2	Freq	b_SMR	se_SMR	p_SMR	p_HEIDI
CD	DNMT3A	2	25510652	rs7578575	25488819	A	T	0.329	-0.834	0.164	3.51E-07	0.383
IBD	DNMT3A	2	25510652	rs7578575	25488819	A	T	0.329	-0.745	0.130	9.22E-09	0.515
UC	DNMT3A	2	25510652	rs7578575	25488819	A	T	0.329	-0.627	0.156	5.73E-05	0.541
UC	CFDP1	16	75327596	rs9934007	75390532	C	T	0.754	0.273	0.070	9.61E-05	0.973
CD	STAT3	17	40502964	rs1053004	40466092	G	A	0.380	0.702	0.128	4.40E-08	0.056
CD	IMPDH2	3	49064299	rs9813813	49207093	A	T	0.110	-1.233	0.294	2.74E-05	0.213
IBD	IMPDH2	3	49064299	rs9813813	49207093	A	T	0.110	-1.328	0.258	2.57E-07	0.071
UC	IMPDH2	3	49064299	rs9813813	49207093	A	T	0.110	-1.445	0.307	2.53E-06	0.050
IBD	UBE2L3	22	21941029	rs2070512	21949411	C	A	0.194	0.163	0.021	1.06E-14	0.169
UC	UBE2L3	22	21941029	rs2070512	21949411	C	A	0.194	0.116	0.027	1.76E-05	0.698
CD	BCL2L15	1	1.14E+08	rs1217393	1.14E+08	A	G	0.271	-0.705	0.170	3.39E-05	0.184
CD	STMN3	20	62271061	rs909333	62270637	G	T	0.207	0.547	0.102	8.31E-08	0.200
IBD	STMN3	20	62271061	rs909333	62270637	G	T	0.207	0.555	0.087	2.01E-10	0.055
UC	STMN3	20	62271061	rs909333	62270637	G	T	0.207	0.498	0.098	4.04E-07	0.062
GORD	PBX2	6	32154964	rs693906	31835164	C	G	0.113	0.154	0.036	1.65E-05	0.058
VHD	AGER	6	32150423	rs693906	31835164	C	G	0.113	0.212	0.052	4.82E-05	0.092

B) Nominally significant effect on VHD of Bonferroni-significant gastrointestinal loci.

Disease	Gene	chr	Probe_bp	topSNP	topSNP_bp	A1	A2	Freq	b_SMR	se_SMR	p_SMR	p_HEIDI
VHD	CFDP1	16	75397489	rs7201989	75471499	G	A	0.246	-0.056	0.018	0.001	0.119
VHD	STAT3	17	40502964	rs1053004	40466092	G	A	0.380	0.093	0.047	0.048	0.948
VHD	IMPDH2	3	49064299	rs34037363	48821036	T	C	0.114	-0.209	0.105	0.047	0.089
VHD	UBE2L3	22	21941029	rs2070512	21949411	C	A	0.194	-0.029	0.009	0.002	0.623
VHD	STMN3	20	62271061	rs909333	62270637	G	T	0.207	-0.076	0.036	0.036	0.093

C) Effect of AGER gene on VHD and gastrointestinal disorders.

Disease	Gene	chr	Probe_bp	topSNP	topSNP_bp	A1	A2	Freq	b_SMR	se_SMR	p_SMR	p_HEIDI
CD	AGER	6	32150423	rs693906	31835164	C	G	0.113	-0.312	0.120	0.009	6.16E-08
GORD	AGER	6	32150423	rs693906	31835164	C	G	0.113	-0.209	0.049	2.02E-05	0.022
IBD	AGER	6	32148745	rs204995	32154285	G	A	0.217	-0.252	0.108	0.020	1.60E-04
IBS	AGER	6	32150423	rs693906	31835164	C	G	0.113	-0.168	0.052	0.001	0.045
UC	AGER	6	32148745	rs204995	32154285	G	A	0.217	-0.870	0.185	2.59E-06	0.002
VHD	AGER	6	32150423	rs693906	31835164	C	G	0.113	0.212	0.052	4.82E-05	0.092

Supplementary table 6. Results of the summary-based Mendelian randomization integrating whole-blood expression quantitative trait loci. For each disease-gene association, we report the strongest effect observed between GTEX and eQTLGen datasets.

A) Bonferroni-significant disease-gene association with HEIDI P > 0.05.

Disease	Gene	chr	gene_bp	topSNP	topSNP_bp	A1	A2	Freq	b_SMR	se_SMR	p_SMR	p_HEIDI
CD	DNMT3A	2	25510652	rs7578575	25488819	A	T	0.329	-0.834	0.164	3.51E-07	0.383
IBD	DNMT3A	2	25510652	rs7578575	25488819	A	T	0.329	-0.745	0.130	9.22E-09	0.515
UC	DNMT3A	2	25510652	rs7578575	25488819	A	T	0.329	-0.627	0.156	5.73E-05	0.541
UC	CFDP1	16	75327596	rs9934007	75390532	C	T	0.754	0.273	0.070	9.61E-05	0.973
CD	STAT3	17	40502964	rs1053004	40466092	G	A	0.380	0.702	0.128	4.40E-08	0.056
CD	IMPDH2	3	49064299	rs9813813	49207093	A	T	0.110	-1.233	0.294	2.74E-05	0.213
IBD	IMPDH2	3	49064299	rs9813813	49207093	A	T	0.110	-1.328	0.258	2.57E-07	0.071
UC	IMPDH2	3	49064299	rs9813813	49207093	A	T	0.110	-1.445	0.307	2.53E-06	0.050
IBD	UBE2L3	22	21941029	rs2070512	21949411	C	A	0.194	0.163	0.021	1.06E-14	0.169
UC	UBE2L3	22	21941029	rs2070512	21949411	C	A	0.194	0.116	0.027	1.76E-05	0.698
CD	BCL2L15	1	1.14E+08	rs1217393	1.14E+08	A	G	0.271	-0.705	0.170	3.39E-05	0.184
CD	STMN3	20	62271061	rs909333	62270637	G	T	0.207	0.547	0.102	8.31E-08	0.200
IBD	STMN3	20	62271061	rs909333	62270637	G	T	0.207	0.555	0.087	2.01E-10	0.055
UC	STMN3	20	62271061	rs909333	62270637	G	T	0.207	0.498	0.098	4.04E-07	0.062
GORD	PBX2	6	32154964	rs693906	31835164	C	G	0.113	0.154	0.036	1.65E-05	0.058
VHD	AGER	6	32150423	rs693906	31835164	C	G	0.113	0.212	0.052	4.82E-05	0.092

B) Nominally significant effect on VHD of Bonferroni-significant gastrointestinal loci.

Disease	Gene	chr	Probe_bp	topSNP	topSNP_bp	A1	A2	Freq	b_SMR	se_SMR	p_SMR	p_HEIDI
VHD	CFDP1	16	75397489	rs7201989	75471499	G	A	0.246	-0.056	0.018	0.001	0.119
VHD	STAT3	17	40502964	rs1053004	40466092	G	A	0.380	0.093	0.047	0.048	0.948
VHD	IMPDH2	3	49064299	rs34037363	48821036	T	C	0.114	-0.209	0.105	0.047	0.089
VHD	UBE2L3	22	21941029	rs2070512	21949411	C	A	0.194	-0.029	0.009	0.002	0.623
VHD	STMN3	20	62271061	rs909333	62270637	G	T	0.207	-0.076	0.036	0.036	0.093

C) Effect of AGER gene on VHD and gastrointestinal disorders.

Disease	Gene	chr	Probe_bp	topSNP	topSNP_bp	A1	A2	Freq	b_SMR	se_SMR	p_SMR	p_HEIDI
CD	AGER	6	32150423	rs693906	31835164	C	G	0.113	-0.312	0.120	0.009	6.16E-08
GORD	AGER	6	32150423	rs693906	31835164	C	G	0.113	-0.209	0.049	2.02E-05	0.022
IBD	AGER	6	32148745	rs204995	32154285	G	A	0.217	-0.252	0.108	0.020	1.60E-04
IBS	AGER	6	32150423	rs693906	31835164	C	G	0.113	-0.168	0.052	0.001	0.045
UC	AGER	6	32148745	rs204995	32154285	G	A	0.217	-0.870	0.185	2.59E-06	0.002
VHD	AGER	6	32150423	rs693906	31835164	C	G	0.113	0.212	0.052	4.82E-05	0.092

Supplementary table 6. Results of the summary-based Mendelian randomization integrating whole-blood expression quantitative trait loci. For each disease-gene association, we report the strongest effect observed between GTEX and eQTLGen datasets.

A) Bonferroni-significant disease-gene association with HEIDI P > 0.05.

Disease	Gene	chr	gene_bp	topSNP	topSNP_bp	A1	A2	Freq	b_SMR	se_SMR	p_SMR	p_HEIDI
CD	DNMT3A	2	25510652	rs7578575	25488819	A	T	0.329	-0.834	0.164	3.51E-07	0.383
IBD	DNMT3A	2	25510652	rs7578575	25488819	A	T	0.329	-0.745	0.130	9.22E-09	0.515
UC	DNMT3A	2	25510652	rs7578575	25488819	A	T	0.329	-0.627	0.156	5.73E-05	0.541
UC	CFDP1	16	75327596	rs9934007	75390532	C	T	0.754	0.273	0.070	9.61E-05	0.973
CD	STAT3	17	40502964	rs1053004	40466092	G	A	0.380	0.702	0.128	4.40E-08	0.056
CD	IMPDH2	3	49064299	rs9813813	49207093	A	T	0.110	-1.233	0.294	2.74E-05	0.213
IBD	IMPDH2	3	49064299	rs9813813	49207093	A	T	0.110	-1.328	0.258	2.57E-07	0.071
UC	IMPDH2	3	49064299	rs9813813	49207093	A	T	0.110	-1.445	0.307	2.53E-06	0.050
IBD	UBE2L3	22	21941029	rs2070512	21949411	C	A	0.194	0.163	0.021	1.06E-14	0.169
UC	UBE2L3	22	21941029	rs2070512	21949411	C	A	0.194	0.116	0.027	1.76E-05	0.698
CD	BCL2L15	1	1.14E+08	rs1217393	1.14E+08	A	G	0.271	-0.705	0.170	3.39E-05	0.184
CD	STMN3	20	62271061	rs909333	62270637	G	T	0.207	0.547	0.102	8.31E-08	0.200
IBD	STMN3	20	62271061	rs909333	62270637	G	T	0.207	0.555	0.087	2.01E-10	0.055
UC	STMN3	20	62271061	rs909333	62270637	G	T	0.207	0.498	0.098	4.04E-07	0.062
GORD	PBX2	6	32154964	rs693906	31835164	C	G	0.113	0.154	0.036	1.65E-05	0.058
VHD	AGER	6	32150423	rs693906	31835164	C	G	0.113	0.212	0.052	4.82E-05	0.092

B) Nominally significant effect on VHD of Bonferroni-significant gastrointestinal loci.

Disease	Gene	chr	Probe_bp	topSNP	topSNP_bp	A1	A2	Freq	b_SMR	se_SMR	p_SMR	p_HEIDI
VHD	CFDP1	16	75397489	rs7201989	75471499	G	A	0.246	-0.056	0.018	0.001	0.119
VHD	STAT3	17	40502964	rs1053004	40466092	G	A	0.380	0.093	0.047	0.048	0.948
VHD	IMPDH2	3	49064299	rs34037363	48821036	T	C	0.114	-0.209	0.105	0.047	0.089
VHD	UBE2L3	22	21941029	rs2070512	21949411	C	A	0.194	-0.029	0.009	0.002	0.623
VHD	STMN3	20	62271061	rs909333	62270637	G	T	0.207	-0.076	0.036	0.036	0.093

C) Effect of AGER gene on VHD and gastrointestinal disorders.

Disease	Gene	chr	Probe_bp	topSNP	topSNP_bp	A1	A2	Freq	b_SMR	se_SMR	p_SMR	p_HEIDI
CD	AGER	6	32150423	rs693906	31835164	C	G	0.113	-0.312	0.120	0.009	6.16E-08
GORD	AGER	6	32150423	rs693906	31835164	C	G	0.113	-0.209	0.049	2.02E-05	0.022
IBD	AGER	6	32148745	rs204995	32154285	G	A	0.217	-0.252	0.108	0.020	1.60E-04
IBS	AGER	6	32150423	rs693906	31835164	C	G	0.113	-0.168	0.052	0.001	0.045
UC	AGER	6	32148745	rs204995	32154285	G	A	0.217	-0.870	0.185	2.59E-06	0.002
VHD	AGER	6	32150423	rs693906	31835164	C	G	0.113	0.212	0.052	4.82E-05	0.092

Supplementary table 6. Results of the summary-based Mendelian randomization integrating whole-blood expression quantitative trait loci. For each disease-gene association, we report the strongest effect observed between GTEX and eQTLGen datasets.

A) Bonferroni-significant disease-gene association with HEIDI P > 0.05.

Disease	Gene	chr	gene_bp	topSNP	topSNP_bp	A1	A2	Freq	b_SMR	se_SMR	p_SMR	p_HEIDI
CD	DNMT3A	2	25510652	rs7578575	25488819	A	T	0.329	-0.834	0.164	3.51E-07	0.383
IBD	DNMT3A	2	25510652	rs7578575	25488819	A	T	0.329	-0.745	0.130	9.22E-09	0.515
UC	DNMT3A	2	25510652	rs7578575	25488819	A	T	0.329	-0.627	0.156	5.73E-05	0.541
UC	CFDP1	16	75327596	rs9934007	75390532	C	T	0.754	0.273	0.070	9.61E-05	0.973
CD	STAT3	17	40502964	rs1053004	40466092	G	A	0.380	0.702	0.128	4.40E-08	0.056
CD	IMPDH2	3	49064299	rs9813813	49207093	A	T	0.110	-1.233	0.294	2.74E-05	0.213
IBD	IMPDH2	3	49064299	rs9813813	49207093	A	T	0.110	-1.328	0.258	2.57E-07	0.071
UC	IMPDH2	3	49064299	rs9813813	49207093	A	T	0.110	-1.445	0.307	2.53E-06	0.050
IBD	UBE2L3	22	21941029	rs2070512	21949411	C	A	0.194	0.163	0.021	1.06E-14	0.169
UC	UBE2L3	22	21941029	rs2070512	21949411	C	A	0.194	0.116	0.027	1.76E-05	0.698
CD	BCL2L15	1	1.14E+08	rs1217393	1.14E+08	A	G	0.271	-0.705	0.170	3.39E-05	0.184
CD	STMN3	20	62271061	rs909333	62270637	G	T	0.207	0.547	0.102	8.31E-08	0.200
IBD	STMN3	20	62271061	rs909333	62270637	G	T	0.207	0.555	0.087	2.01E-10	0.055
UC	STMN3	20	62271061	rs909333	62270637	G	T	0.207	0.498	0.098	4.04E-07	0.062
GORD	PBX2	6	32154964	rs693906	31835164	C	G	0.113	0.154	0.036	1.65E-05	0.058
VHD	AGER	6	32150423	rs693906	31835164	C	G	0.113	0.212	0.052	4.82E-05	0.092

B) Nominally significant effect on VHD of Bonferroni-significant gastrointestinal loci.

Disease	Gene	chr	Probe_bp	topSNP	topSNP_bp	A1	A2	Freq	b_SMR	se_SMR	p_SMR	p_HEIDI
VHD	CFDP1	16	75397489	rs7201989	75471499	G	A	0.246	-0.056	0.018	0.001	0.119
VHD	STAT3	17	40502964	rs1053004	40466092	G	A	0.380	0.093	0.047	0.048	0.948
VHD	IMPDH2	3	49064299	rs34037363	48821036	T	C	0.114	-0.209	0.105	0.047	0.089
VHD	UBE2L3	22	21941029	rs2070512	21949411	C	A	0.194	-0.029	0.009	0.002	0.623
VHD	STMN3	20	62271061	rs909333	62270637	G	T	0.207	-0.076	0.036	0.036	0.093

C) Effect of AGER gene on VHD and gastrointestinal disorders.

Disease	Gene	chr	Probe_bp	topSNP	topSNP_bp	A1	A2	Freq	b_SMR	se_SMR	p_SMR	p_HEIDI
CD	AGER	6	32150423	rs693906	31835164	C	G	0.113	-0.312	0.120	0.009	6.16E-08
GORD	AGER	6	32150423	rs693906	31835164	C	G	0.113	-0.209	0.049	2.02E-05	0.022
IBD	AGER	6	32148745	rs204995	32154285	G	A	0.217	-0.252	0.108	0.020	1.60E-04
IBS	AGER	6	32150423	rs693906	31835164	C	G	0.113	-0.168	0.052	0.001	0.045
UC	AGER	6	32148745	rs204995	32154285	G	A	0.217	-0.870	0.185	2.59E-06	0.002
VHD	AGER	6	32150423	rs693906	31835164	C	G	0.113	0.212	0.052	4.82E-05	0.092

Supplementary table 6. Results of the summary-based Mendelian randomization integrating whole-blood expression quantitative trait loci. For each disease-gene association, we report the strongest effect observed between GTEX and eQTLGen datasets.

A) Bonferroni-significant disease-gene association with HEIDI P > 0.05.

Disease	Gene	chr	gene_bp	topSNP	topSNP_bp	A1	A2	Freq	b_SMR	se_SMR	p_SMR	p_HEIDI
CD	DNMT3A	2	25510652	rs7578575	25488819	A	T	0.329	-0.834	0.164	3.51E-07	0.383
IBD	DNMT3A	2	25510652	rs7578575	25488819	A	T	0.329	-0.745	0.130	9.22E-09	0.515
UC	DNMT3A	2	25510652	rs7578575	25488819	A	T	0.329	-0.627	0.156	5.73E-05	0.541
UC	CFDP1	16	75327596	rs9934007	75390532	C	T	0.754	0.273	0.070	9.61E-05	0.973
CD	STAT3	17	40502964	rs1053004	40466092	G	A	0.380	0.702	0.128	4.40E-08	0.056
CD	IMPDH2	3	49064299	rs9813813	49207093	A	T	0.110	-1.233	0.294	2.74E-05	0.213
IBD	IMPDH2	3	49064299	rs9813813	49207093	A	T	0.110	-1.328	0.258	2.57E-07	0.071
UC	IMPDH2	3	49064299	rs9813813	49207093	A	T	0.110	-1.445	0.307	2.53E-06	0.050
IBD	UBE2L3	22	21941029	rs2070512	21949411	C	A	0.194	0.163	0.021	1.06E-14	0.169
UC	UBE2L3	22	21941029	rs2070512	21949411	C	A	0.194	0.116	0.027	1.76E-05	0.698
CD	BCL2L15	1	1.14E+08	rs1217393	1.14E+08	A	G	0.271	-0.705	0.170	3.39E-05	0.184
CD	STMN3	20	62271061	rs909333	62270637	G	T	0.207	0.547	0.102	8.31E-08	0.200
IBD	STMN3	20	62271061	rs909333	62270637	G	T	0.207	0.555	0.087	2.01E-10	0.055
UC	STMN3	20	62271061	rs909333	62270637	G	T	0.207	0.498	0.098	4.04E-07	0.062
GORD	PBX2	6	32154964	rs693906	31835164	C	G	0.113	0.154	0.036	1.65E-05	0.058
VHD	AGER	6	32150423	rs693906	31835164	C	G	0.113	0.212	0.052	4.82E-05	0.092

B) Nominally significant effect on VHD of Bonferroni-significant gastrointestinal loci.

Disease	Gene	chr	Probe_bp	topSNP	topSNP_bp	A1	A2	Freq	b_SMR	se_SMR	p_SMR	p_HEIDI
VHD	CFDP1	16	75397489	rs7201989	75471499	G	A	0.246	-0.056	0.018	0.001	0.119
VHD	STAT3	17	40502964	rs1053004	40466092	G	A	0.380	0.093	0.047	0.048	0.948
VHD	IMPDH2	3	49064299	rs34037363	48821036	T	C	0.114	-0.209	0.105	0.047	0.089
VHD	UBE2L3	22	21941029	rs2070512	21949411	C	A	0.194	-0.029	0.009	0.002	0.623
VHD	STMN3	20	62271061	rs909333	62270637	G	T	0.207	-0.076	0.036	0.036	0.093

C) Effect of AGER gene on VHD and gastrointestinal disorders.

Disease	Gene	chr	Probe_bp	topSNP	topSNP_bp	A1	A2	Freq	b_SMR	se_SMR	p_SMR	p_HEIDI
CD	AGER	6	32150423	rs693906	31835164	C	G	0.113	-0.312	0.120	0.009	6.16E-08
GORD	AGER	6	32150423	rs693906	31835164	C	G	0.113	-0.209	0.049	2.02E-05	0.022
IBD	AGER	6	32148745	rs204995	32154285	G	A	0.217	-0.252	0.108	0.020	1.60E-04
IBS	AGER	6	32150423	rs693906	31835164	C	G	0.113	-0.168	0.052	0.001	0.045
UC	AGER	6	32148745	rs204995	32154285	G	A	0.217	-0.870	0.185	2.59E-06	0.002
VHD	AGER	6	32150423	rs693906	31835164	C	G	0.113	0.212	0.052	4.82E-05	0.092

Supplementary table 6. Results of the summary-based Mendelian randomization integrating whole-blood expression quantitative trait loci. For each disease-gene association, we report the strongest effect observed between GTEX and eQTLGen datasets.

A) Bonferroni-significant disease-gene association with HEIDI P > 0.05.

Disease	Gene	chr	gene_bp	topSNP	topSNP_bp	A1	A2	Freq	b_SMR	se_SMR	p_SMR	p_HEIDI
CD	DNMT3A	2	25510652	rs7578575	25488819	A	T	0.329	-0.834	0.164	3.51E-07	0.383
IBD	DNMT3A	2	25510652	rs7578575	25488819	A	T	0.329	-0.745	0.130	9.22E-09	0.515
UC	DNMT3A	2	25510652	rs7578575	25488819	A	T	0.329	-0.627	0.156	5.73E-05	0.541
UC	CFDP1	16	75327596	rs9934007	75390532	C	T	0.754	0.273	0.070	9.61E-05	0.973
CD	STAT3	17	40502964	rs1053004	40466092	G	A	0.380	0.702	0.128	4.40E-08	0.056
CD	IMPDH2	3	49064299	rs9813813	49207093	A	T	0.110	-1.233	0.294	2.74E-05	0.213
IBD	IMPDH2	3	49064299	rs9813813	49207093	A	T	0.110	-1.328	0.258	2.57E-07	0.071
UC	IMPDH2	3	49064299	rs9813813	49207093	A	T	0.110	-1.445	0.307	2.53E-06	0.050
IBD	UBE2L3	22	21941029	rs2070512	21949411	C	A	0.194	0.163	0.021	1.06E-14	0.169
UC	UBE2L3	22	21941029	rs2070512	21949411	C	A	0.194	0.116	0.027	1.76E-05	0.698
CD	BCL2L15	1	1.14E+08	rs1217393	1.14E+08	A	G	0.271	-0.705	0.170	3.39E-05	0.184
CD	STMN3	20	62271061	rs909333	62270637	G	T	0.207	0.547	0.102	8.31E-08	0.200
IBD	STMN3	20	62271061	rs909333	62270637	G	T	0.207	0.555	0.087	2.01E-10	0.055
UC	STMN3	20	62271061	rs909333	62270637	G	T	0.207	0.498	0.098	4.04E-07	0.062
GORD	PBX2	6	32154964	rs693906	31835164	C	G	0.113	0.154	0.036	1.65E-05	0.058
VHD	AGER	6	32150423	rs693906	31835164	C	G	0.113	0.212	0.052	4.82E-05	0.092

B) Nominally significant effect on VHD of Bonferroni-significant gastrointestinal loci.

Disease	Gene	chr	Probe_bp	topSNP	topSNP_bp	A1	A2	Freq	b_SMR	se_SMR	p_SMR	p_HEIDI
VHD	CFDP1	16	75397489	rs7201989	75471499	G	A	0.246	-0.056	0.018	0.001	0.119
VHD	STAT3	17	40502964	rs1053004	40466092	G	A	0.380	0.093	0.047	0.048	0.948
VHD	IMPDH2	3	49064299	rs34037363	48821036	T	C	0.114	-0.209	0.105	0.047	0.089
VHD	UBE2L3	22	21941029	rs2070512	21949411	C	A	0.194	-0.029	0.009	0.002	0.623
VHD	STMN3	20	62271061	rs909333	62270637	G	T	0.207	-0.076	0.036	0.036	0.093

C) Effect of AGER gene on VHD and gastrointestinal disorders.

Disease	Gene	chr	Probe_bp	topSNP	topSNP_bp	A1	A2	Freq	b_SMR	se_SMR	p_SMR	p_HEIDI
CD	AGER	6	32150423	rs693906	31835164	C	G	0.113	-0.312	0.120	0.009	6.16E-08
GORD	AGER	6	32150423	rs693906	31835164	C	G	0.113	-0.209	0.049	2.02E-05	0.022
IBD	AGER	6	32148745	rs204995	32154285	G	A	0.217	-0.252	0.108	0.020	1.60E-04
IBS	AGER	6	32150423	rs693906	31835164	C	G	0.113	-0.168	0.052	0.001	0.045
UC	AGER	6	32148745	rs204995	32154285	G	A	0.217	-0.870	0.185	2.59E-06	0.002
VHD	AGER	6	32150423	rs693906	31835164	C	G	0.113	0.212	0.052	4.82E-05	0.092

Supplementary table 6. Results of the summary-based Mendelian randomization integrating whole-blood expression quantitative trait loci. For each disease-gene association, we report the strongest effect observed between GTEX and eQTLGen datasets.

A) Bonferroni-significant disease-gene association with HEIDI P > 0.05.

Disease	Gene	chr	gene_bp	topSNP	topSNP_bp	A1	A2	Freq	b_SMR	se_SMR	p_SMR	p_HEIDI
CD	DNMT3A	2	25510652	rs7578575	25488819	A	T	0.329	-0.834	0.164	3.51E-07	0.383
IBD	DNMT3A	2	25510652	rs7578575	25488819	A	T	0.329	-0.745	0.130	9.22E-09	0.515
UC	DNMT3A	2	25510652	rs7578575	25488819	A	T	0.329	-0.627	0.156	5.73E-05	0.541
UC	CFDP1	16	75327596	rs9934007	75390532	C	T	0.754	0.273	0.070	9.61E-05	0.973
CD	STAT3	17	40502964	rs1053004	40466092	G	A	0.380	0.702	0.128	4.40E-08	0.056
CD	IMPDH2	3	49064299	rs9813813	49207093	A	T	0.110	-1.233	0.294	2.74E-05	0.213
IBD	IMPDH2	3	49064299	rs9813813	49207093	A	T	0.110	-1.328	0.258	2.57E-07	0.071
UC	IMPDH2	3	49064299	rs9813813	49207093	A	T	0.110	-1.445	0.307	2.53E-06	0.050
IBD	UBE2L3	22	21941029	rs2070512	21949411	C	A	0.194	0.163	0.021	1.06E-14	0.169
UC	UBE2L3	22	21941029	rs2070512	21949411	C	A	0.194	0.116	0.027	1.76E-05	0.698
CD	BCL2L15	1	1.14E+08	rs1217393	1.14E+08	A	G	0.271	-0.705	0.170	3.39E-05	0.184
CD	STMN3	20	62271061	rs909333	62270637	G	T	0.207	0.547	0.102	8.31E-08	0.200
IBD	STMN3	20	62271061	rs909333	62270637	G	T	0.207	0.555	0.087	2.01E-10	0.055
UC	STMN3	20	62271061	rs909333	62270637	G	T	0.207	0.498	0.098	4.04E-07	0.062
GORD	PBX2	6	32154964	rs693906	31835164	C	G	0.113	0.154	0.036	1.65E-05	0.058
VHD	AGER	6	32150423	rs693906	31835164	C	G	0.113	0.212	0.052	4.82E-05	0.092

B) Nominally significant effect on VHD of Bonferroni-significant gastrointestinal loci.

Disease	Gene	chr	Probe_bp	topSNP	topSNP_bp	A1	A2	Freq	b_SMR	se_SMR	p_SMR	p_HEIDI
VHD	CFDP1	16	75397489	rs7201989	75471499	G	A	0.246	-0.056	0.018	0.001	0.119
VHD	STAT3	17	40502964	rs1053004	40466092	G	A	0.380	0.093	0.047	0.048	0.948
VHD	IMPDH2	3	49064299	rs34037363	48821036	T	C	0.114	-0.209	0.105	0.047	0.089
VHD	UBE2L3	22	21941029	rs2070512	21949411	C	A	0.194	-0.029	0.009	0.002	0.623
VHD	STMN3	20	62271061	rs909333	62270637	G	T	0.207	-0.076	0.036	0.036	0.093

C) Effect of AGER gene on VHD and gastrointestinal disorders.

Disease	Gene	chr	Probe_bp	topSNP	topSNP_bp	A1	A2	Freq	b_SMR	se_SMR	p_SMR	p_HEIDI
CD	AGER	6	32150423	rs693906	31835164	C	G	0.113	-0.312	0.120	0.009	6.16E-08
GORD	AGER	6	32150423	rs693906	31835164	C	G	0.113	-0.209	0.049	2.02E-05	0.022
IBD	AGER	6	32148745	rs204995	32154285	G	A	0.217	-0.252	0.108	0.020	1.60E-04
IBS	AGER	6	32150423	rs693906	31835164	C	G	0.113	-0.168	0.052	0.001	0.045
UC	AGER	6	32148745	rs204995	32154285	G	A	0.217	-0.870	0.185	2.59E-06	0.002
VHD	AGER	6	32150423	rs693906	31835164	C	G	0.113	0.212	0.052	4.82E-05	0.092

Supplementary table 6. Results of the summary-based Mendelian randomization integrating whole-blood expression quantitative trait loci. For each disease-gene association, we report the strongest effect observed between GTEX and eQTLGen datasets.

A) Bonferroni-significant disease-gene association with HEIDI P > 0.05.

Disease	Gene	chr	gene_bp	topSNP	topSNP_bp	A1	A2	Freq	b_SMR	se_SMR	p_SMR	p_HEIDI
CD	DNMT3A	2	25510652	rs7578575	25488819	A	T	0.329	-0.834	0.164	3.51E-07	0.383
IBD	DNMT3A	2	25510652	rs7578575	25488819	A	T	0.329	-0.745	0.130	9.22E-09	0.515
UC	DNMT3A	2	25510652	rs7578575	25488819	A	T	0.329	-0.627	0.156	5.73E-05	0.541
UC	CFDP1	16	75327596	rs9934007	75390532	C	T	0.754	0.273	0.070	9.61E-05	0.973
CD	STAT3	17	40502964	rs1053004	40466092	G	A	0.380	0.702	0.128	4.40E-08	0.056
CD	IMPDH2	3	49064299	rs9813813	49207093	A	T	0.110	-1.233	0.294	2.74E-05	0.213
IBD	IMPDH2	3	49064299	rs9813813	49207093	A	T	0.110	-1.328	0.258	2.57E-07	0.071
UC	IMPDH2	3	49064299	rs9813813	49207093	A	T	0.110	-1.445	0.307	2.53E-06	0.050
IBD	UBE2L3	22	21941029	rs2070512	21949411	C	A	0.194	0.163	0.021	1.06E-14	0.169
UC	UBE2L3	22	21941029	rs2070512	21949411	C	A	0.194	0.116	0.027	1.76E-05	0.698
CD	BCL2L15	1	1.14E+08	rs1217393	1.14E+08	A	G	0.271	-0.705	0.170	3.39E-05	0.184
CD	STMN3	20	62271061	rs909333	62270637	G	T	0.207	0.547	0.102	8.31E-08	0.200
IBD	STMN3	20	62271061	rs909333	62270637	G	T	0.207	0.555	0.087	2.01E-10	0.055
UC	STMN3	20	62271061	rs909333	62270637	G	T	0.207	0.498	0.098	4.04E-07	0.062
GORD	PBX2	6	32154964	rs693906	31835164	C	G	0.113	0.154	0.036	1.65E-05	0.058
VHD	AGER	6	32150423	rs693906	31835164	C	G	0.113	0.212	0.052	4.82E-05	0.092

B) Nominally significant effect on VHD of Bonferroni-significant gastrointestinal loci.

Disease	Gene	chr	Probe_bp	topSNP	topSNP_bp	A1	A2	Freq	b_SMR	se_SMR	p_SMR	p_HEIDI
VHD	CFDP1	16	75397489	rs7201989	75471499	G	A	0.246	-0.056	0.018	0.001	0.119
VHD	STAT3	17	40502964	rs1053004	40466092	G	A	0.380	0.093	0.047	0.048	0.948
VHD	IMPDH2	3	49064299	rs34037363	48821036	T	C	0.114	-0.209	0.105	0.047	0.089
VHD	UBE2L3	22	21941029	rs2070512	21949411	C	A	0.194	-0.029	0.009	0.002	0.623
VHD	STMN3	20	62271061	rs909333	62270637	G	T	0.207	-0.076	0.036	0.036	0.093

C) Effect of AGER gene on VHD and gastrointestinal disorders.

Disease	Gene	chr	Probe_bp	topSNP	topSNP_bp	A1	A2	Freq	b_SMR	se_SMR	p_SMR	p_HEIDI
CD	AGER	6	32150423	rs693906	31835164	C	G	0.113	-0.312	0.120	0.009	6.16E-08
GORD	AGER	6	32150423	rs693906	31835164	C	G	0.113	-0.209	0.049	2.02E-05	0.022
IBD	AGER	6	32148745	rs204995	32154285	G	A	0.217	-0.252	0.108	0.020	1.60E-04
IBS	AGER	6	32150423	rs693906	31835164	C	G	0.113	-0.168	0.052	0.001	0.045
UC	AGER	6	32148745	rs204995	32154285	G	A	0.217	-0.870	0.185	2.59E-06	0.002
VHD	AGER	6	32150423	rs693906	31835164	C	G	0.113	0.212	0.052	4.82E-05	0.092

Supplementary table 6. Results of the summary-based Mendelian randomization integrating whole-blood expression quantitative trait loci. For each disease-gene association, we report the strongest effect observed between GTEX and eQTLGen datasets.

A) Bonferroni-significant disease-gene association with HEIDI P > 0.05.

Disease	Gene	chr	gene_bp	topSNP	topSNP_bp	A1	A2	Freq	b_SMR	se_SMR	p_SMR	p_HEIDI
CD	DNMT3A	2	25510652	rs7578575	25488819	A	T	0.329	-0.834	0.164	3.51E-07	0.383
IBD	DNMT3A	2	25510652	rs7578575	25488819	A	T	0.329	-0.745	0.130	9.22E-09	0.515
UC	DNMT3A	2	25510652	rs7578575	25488819	A	T	0.329	-0.627	0.156	5.73E-05	0.541
UC	CFDP1	16	75327596	rs9934007	75390532	C	T	0.754	0.273	0.070	9.61E-05	0.973
CD	STAT3	17	40502964	rs1053004	40466092	G	A	0.380	0.702	0.128	4.40E-08	0.056
CD	IMPDH2	3	49064299	rs9813813	49207093	A	T	0.110	-1.233	0.294	2.74E-05	0.213
IBD	IMPDH2	3	49064299	rs9813813	49207093	A	T	0.110	-1.328	0.258	2.57E-07	0.071
UC	IMPDH2	3	49064299	rs9813813	49207093	A	T	0.110	-1.445	0.307	2.53E-06	0.050
IBD	UBE2L3	22	21941029	rs2070512	21949411	C	A	0.194	0.163	0.021	1.06E-14	0.169
UC	UBE2L3	22	21941029	rs2070512	21949411	C	A	0.194	0.116	0.027	1.76E-05	0.698
CD	BCL2L15	1	1.14E+08	rs1217393	1.14E+08	A	G	0.271	-0.705	0.170	3.39E-05	0.184
CD	STMN3	20	62271061	rs909333	62270637	G	T	0.207	0.547	0.102	8.31E-08	0.200
IBD	STMN3	20	62271061	rs909333	62270637	G	T	0.207	0.555	0.087	2.01E-10	0.055
UC	STMN3	20	62271061	rs909333	62270637	G	T	0.207	0.498	0.098	4.04E-07	0.062
GORD	PBX2	6	32154964	rs693906	31835164	C	G	0.113	0.154	0.036	1.65E-05	0.058
VHD	AGER	6	32150423	rs693906	31835164	C	G	0.113	0.212	0.052	4.82E-05	0.092

B) Nominally significant effect on VHD of Bonferroni-significant gastrointestinal loci.

Disease	Gene	chr	Probe_bp	topSNP	topSNP_bp	A1	A2	Freq	b_SMR	se_SMR	p_SMR	p_HEIDI
VHD	CFDP1	16	75397489	rs7201989	75471499	G	A	0.246	-0.056	0.018	0.001	0.119
VHD	STAT3	17	40502964	rs1053004	40466092	G	A	0.380	0.093	0.047	0.048	0.948
VHD	IMPDH2	3	49064299	rs34037363	48821036	T	C	0.114	-0.209	0.105	0.047	0.089
VHD	UBE2L3	22	21941029	rs2070512	21949411	C	A	0.194	-0.029	0.009	0.002	0.623
VHD	STMN3	20	62271061	rs909333	62270637	G	T	0.207	-0.076	0.036	0.036	0.093

C) Effect of AGER gene on VHD and gastrointestinal disorders.

Disease	Gene	chr	Probe_bp	topSNP	topSNP_bp	A1	A2	Freq	b_SMR	se_SMR	p_SMR	p_HEIDI
CD	AGER	6	32150423	rs693906	31835164	C	G	0.113	-0.312	0.120	0.009	6.16E-08
GORD	AGER	6	32150423	rs693906	31835164	C	G	0.113	-0.209	0.049	2.02E-05	0.022
IBD	AGER	6	32148745	rs204995	32154285	G	A	0.217	-0.252	0.108	0.020	1.60E-04
IBS	AGER	6	32150423	rs693906	31835164	C	G	0.113	-0.168	0.052	0.001	0.045
UC	AGER	6	32148745	rs204995	32154285	G	A	0.217	-0.870	0.185	2.59E-06	0.002
VHD	AGER	6	32150423	rs693906	31835164	C	G	0.113	0.212	0.052	4.82E-05	0.092

Supplementary table 6. Results of the summary-based Mendelian randomization integrating whole-blood expression quantitative trait loci. For each disease-gene association, we report the strongest effect observed between GTEX and eQTLGen datasets.

A) Bonferroni-significant disease-gene association with HEIDI P > 0.05.

Disease	Gene	chr	gene_bp	topSNP	topSNP_bp	A1	A2	Freq	b_SMR	se_SMR	p_SMR	p_HEIDI
CD	DNMT3A	2	25510652	rs7578575	25488819	A	T	0.329	-0.834	0.164	3.51E-07	0.383
IBD	DNMT3A	2	25510652	rs7578575	25488819	A	T	0.329	-0.745	0.130	9.22E-09	0.515
UC	DNMT3A	2	25510652	rs7578575	25488819	A	T	0.329	-0.627	0.156	5.73E-05	0.541
UC	CFDP1	16	75327596	rs9934007	75390532	C	T	0.754	0.273	0.070	9.61E-05	0.973
CD	STAT3	17	40502964	rs1053004	40466092	G	A	0.380	0.702	0.128	4.40E-08	0.056
CD	IMPDH2	3	49064299	rs9813813	49207093	A	T	0.110	-1.233	0.294	2.74E-05	0.213
IBD	IMPDH2	3	49064299	rs9813813	49207093	A	T	0.110	-1.328	0.258	2.57E-07	0.071
UC	IMPDH2	3	49064299	rs9813813	49207093	A	T	0.110	-1.445	0.307	2.53E-06	0.050
IBD	UBE2L3	22	21941029	rs2070512	21949411	C	A	0.194	0.163	0.021	1.06E-14	0.169
UC	UBE2L3	22	21941029	rs2070512	21949411	C	A	0.194	0.116	0.027	1.76E-05	0.698
CD	BCL2L15	1	1.14E+08	rs1217393	1.14E+08	A	G	0.271	-0.705	0.170	3.39E-05	0.184
CD	STMN3	20	62271061	rs909333	62270637	G	T	0.207	0.547	0.102	8.31E-08	0.200
IBD	STMN3	20	62271061	rs909333	62270637	G	T	0.207	0.555	0.087	2.01E-10	0.055
UC	STMN3	20	62271061	rs909333	62270637	G	T	0.207	0.498	0.098	4.04E-07	0.062
GORD	PBX2	6	32154964	rs693906	31835164	C	G	0.113	0.154	0.036	1.65E-05	0.058
VHD	AGER	6	32150423	rs693906	31835164	C	G	0.113	0.212	0.052	4.82E-05	0.092

B) Nominally significant effect on VHD of Bonferroni-significant gastrointestinal loci.

Disease	Gene	chr	Probe_bp	topSNP	topSNP_bp	A1	A2	Freq	b_SMR	se_SMR	p_SMR	p_HEIDI
VHD	CFDP1	16	75397489	rs7201989	75471499	G	A	0.246	-0.056	0.018	0.001	0.119
VHD	STAT3	17	40502964	rs1053004	40466092	G	A	0.380	0.093	0.047	0.048	0.948
VHD	IMPDH2	3	49064299	rs34037363	48821036	T	C	0.114	-0.209	0.105	0.047	0.089
VHD	UBE2L3	22	21941029	rs2070512	21949411	C	A	0.194	-0.029	0.009	0.002	0.623
VHD	STMN3	20	62271061	rs909333	62270637	G	T	0.207	-0.076	0.036	0.036	0.093

C) Effect of AGER gene on VHD and gastrointestinal disorders.

Disease	Gene	chr	Probe_bp	topSNP	topSNP_bp	A1	A2	Freq	b_SMR	se_SMR	p_SMR	p_HEIDI
CD	AGER	6	32150423	rs693906	31835164	C	G	0.113	-0.312	0.120	0.009	6.16E-08
GORD	AGER	6	32150423	rs693906	31835164	C	G	0.113	-0.209	0.049	2.02E-05	0.022
IBD	AGER	6	32148745	rs204995	32154285	G	A	0.217	-0.252	0.108	0.020	1.60E-04
IBS	AGER	6	32150423	rs693906	31835164	C	G	0.113	-0.168	0.052	0.001	0.045
UC	AGER	6	32148745	rs204995	32154285	G	A	0.217	-0.870	0.185	2.59E-06	0.002
VHD	AGER	6	32150423	rs693906	31835164	C	G	0.113	0.212	0.052	4.82E-05	0.092

Supplementary table 6. Results of the summary-based Mendelian randomization integrating whole-blood expression quantitative trait loci. For each disease-gene association, we report the strongest effect observed between GTEX and eQTLGen datasets.

A) Bonferroni-significant disease-gene association with HEIDI P > 0.05.

Disease	Gene	chr	gene_bp	topSNP	topSNP_bp	A1	A2	Freq	b_SMR	se_SMR	p_SMR	p_HEIDI
CD	DNMT3A	2	25510652	rs7578575	25488819	A	T	0.329	-0.834	0.164	3.51E-07	0.383
IBD	DNMT3A	2	25510652	rs7578575	25488819	A	T	0.329	-0.745	0.130	9.22E-09	0.515
UC	DNMT3A	2	25510652	rs7578575	25488819	A	T	0.329	-0.627	0.156	5.73E-05	0.541
UC	CFDP1	16	75327596	rs9934007	75390532	C	T	0.754	0.273	0.070	9.61E-05	0.973
CD	STAT3	17	40502964	rs1053004	40466092	G	A	0.380	0.702	0.128	4.40E-08	0.056
CD	IMPDH2	3	49064299	rs9813813	49207093	A	T	0.110	-1.233	0.294	2.74E-05	0.213
IBD	IMPDH2	3	49064299	rs9813813	49207093	A	T	0.110	-1.328	0.258	2.57E-07	0.071
UC	IMPDH2	3	49064299	rs9813813	49207093	A	T	0.110	-1.445	0.307	2.53E-06	0.050
IBD	UBE2L3	22	21941029	rs2070512	21949411	C	A	0.194	0.163	0.021	1.06E-14	0.169
UC	UBE2L3	22	21941029	rs2070512	21949411	C	A	0.194	0.116	0.027	1.76E-05	0.698
CD	BCL2L15	1	1.14E+08	rs1217393	1.14E+08	A	G	0.271	-0.705	0.170	3.39E-05	0.184
CD	STMN3	20	62271061	rs909333	62270637	G	T	0.207	0.547	0.102	8.31E-08	0.200
IBD	STMN3	20	62271061	rs909333	62270637	G	T	0.207	0.555	0.087	2.01E-10	0.055
UC	STMN3	20	62271061	rs909333	62270637	G	T	0.207	0.498	0.098	4.04E-07	0.062
GORD	PBX2	6	32154964	rs693906	31835164	C	G	0.113	0.154	0.036	1.65E-05	0.058
VHD	AGER	6	32150423	rs693906	31835164	C	G	0.113	0.212	0.052	4.82E-05	0.092

B) Nominally significant effect on VHD of Bonferroni-significant gastrointestinal loci.

Disease	Gene	chr	Probe_bp	topSNP	topSNP_bp	A1	A2	Freq	b_SMR	se_SMR	p_SMR	p_HEIDI
VHD	CFDP1	16	75397489	rs7201989	75471499	G	A	0.246	-0.056	0.018	0.001	0.119
VHD	STAT3	17	40502964	rs1053004	40466092	G	A	0.380	0.093	0.047	0.048	0.948
VHD	IMPDH2	3	49064299	rs34037363	48821036	T	C	0.114	-0.209	0.105	0.047	0.089
VHD	UBE2L3	22	21941029	rs2070512	21949411	C	A	0.194	-0.029	0.009	0.002	0.623
VHD	STMN3	20	62271061	rs909333	62270637	G	T	0.207	-0.076	0.036	0.036	0.093

C) Effect of AGER gene on VHD and gastrointestinal disorders.

Disease	Gene	chr	Probe_bp	topSNP	topSNP_bp	A1	A2	Freq	b_SMR	se_SMR	p_SMR	p_HEIDI
CD	AGER	6	32150423	rs693906	31835164	C	G	0.113	-0.312	0.120	0.009	6.16E-08
GORD	AGER	6	32150423	rs693906	31835164	C	G	0.113	-0.209	0.049	2.02E-05	0.022
IBD	AGER	6	32148745	rs204995	32154285	G	A	0.217	-0.252	0.108	0.020	1.60E-04
IBS	AGER	6	32150423	rs693906	31835164	C	G	0.113	-0.168	0.052	0.001	0.045
UC	AGER	6	32148745	rs204995	32154285	G	A	0.217	-0.870	0.185	2.59E-06	0.002
VHD	AGER	6	32150423	rs693906	31835164	C	G	0.113	0.212	0.052	4.82E-05	0.092

Supplementary table 6. Results of the summary-based Mendelian randomization integrating whole-blood expression quantitative trait loci. For each disease-gene association, we report the strongest effect observed between GTEX and eQTLGen datasets.

A) Bonferroni-significant disease-gene association with HEIDI P > 0.05.

Disease	Gene	chr	gene_bp	topSNP	topSNP_bp	A1	A2	Freq	b_SMR	se_SMR	p_SMR	p_HEIDI
CD	DNMT3A	2	25510652	rs7578575	25488819	A	T	0.329	-0.834	0.164	3.51E-07	0.383
IBD	DNMT3A	2	25510652	rs7578575	25488819	A	T	0.329	-0.745	0.130	9.22E-09	0.515
UC	DNMT3A	2	25510652	rs7578575	25488819	A	T	0.329	-0.627	0.156	5.73E-05	0.541
UC	CFDP1	16	75327596	rs9934007	75390532	C	T	0.754	0.273	0.070	9.61E-05	0.973
CD	STAT3	17	40502964	rs1053004	40466092	G	A	0.380	0.702	0.128	4.40E-08	0.056
CD	IMPDH2	3	49064299	rs9813813	49207093	A	T	0.110	-1.233	0.294	2.74E-05	0.213
IBD	IMPDH2	3	49064299	rs9813813	49207093	A	T	0.110	-1.328	0.258	2.57E-07	0.071
UC	IMPDH2	3	49064299	rs9813813	49207093	A	T	0.110	-1.445	0.307	2.53E-06	0.050
IBD	UBE2L3	22	21941029	rs2070512	21949411	C	A	0.194	0.163	0.021	1.06E-14	0.169
UC	UBE2L3	22	21941029	rs2070512	21949411	C	A	0.194	0.116	0.027	1.76E-05	0.698
CD	BCL2L15	1	1.14E+08	rs1217393	1.14E+08	A	G	0.271	-0.705	0.170	3.39E-05	0.184
CD	STMN3	20	62271061	rs909333	62270637	G	T	0.207	0.547	0.102	8.31E-08	0.200
IBD	STMN3	20	62271061	rs909333	62270637	G	T	0.207	0.555	0.087	2.01E-10	0.055
UC	STMN3	20	62271061	rs909333	62270637	G	T	0.207	0.498	0.098	4.04E-07	0.062
GORD	PBX2	6	32154964	rs693906	31835164	C	G	0.113	0.154	0.036	1.65E-05	0.058
VHD	AGER	6	32150423	rs693906	31835164	C	G	0.113	0.212	0.052	4.82E-05	0.092

B) Nominally significant effect on VHD of Bonferroni-significant gastrointestinal loci.

Disease	Gene	chr	Probe_bp	topSNP	topSNP_bp	A1	A2	Freq	b_SMR	se_SMR	p_SMR	p_HEIDI
VHD	CFDP1	16	75397489	rs7201989	75471499	G	A	0.246	-0.056	0.018	0.001	0.119
VHD	STAT3	17	40502964	rs1053004	40466092	G	A	0.380	0.093	0.047	0.048	0.948
VHD	IMPDH2	3	49064299	rs34037363	48821036	T	C	0.114	-0.209	0.105	0.047	0.089
VHD	UBE2L3	22	21941029	rs2070512	21949411	C	A	0.194	-0.029	0.009	0.002	0.623
VHD	STMN3	20	62271061	rs909333	62270637	G	T	0.207	-0.076	0.036	0.036	0.093

C) Effect of AGER gene on VHD and gastrointestinal disorders.

Disease	Gene	chr	Probe_bp	topSNP	topSNP_bp	A1	A2	Freq	b_SMR	se_SMR	p_SMR	p_HEIDI
CD	AGER	6	32150423	rs693906	31835164	C	G	0.113	-0.312	0.120	0.009	6.16E-08
GORD	AGER	6	32150423	rs693906	31835164	C	G	0.113	-0.209	0.049	2.02E-05	0.022
IBD	AGER	6	32148745	rs204995	32154285	G	A	0.217	-0.252	0.108	0.020	1.60E-04
IBS	AGER	6	32150423	rs693906	31835164	C	G	0.113	-0.168	0.052	0.001	0.045
UC	AGER	6	32148745	rs204995	32154285	G	A	0.217	-0.870	0.185	2.59E-06	0.002
VHD	AGER	6	32150423	rs693906	31835164	C	G	0.113	0.212	0.052	4.82E-05	0.092

Supplementary table 6. Results of the summary-based Mendelian randomization integrating whole-blood expression quantitative trait loci. For each disease-gene association, we report the strongest effect observed between GTEX and eQTLGen datasets.

A) Bonferroni-significant disease-gene association with HEIDI P > 0.05.

Disease	Gene	chr	gene_bp	topSNP	topSNP_bp	A1	A2	Freq	b_SMR	se_SMR	p_SMR	p_HEIDI
CD	DNMT3A	2	25510652	rs7578575	25488819	A	T	0.329	-0.834	0.164	3.51E-07	0.383
IBD	DNMT3A	2	25510652	rs7578575	25488819	A	T	0.329	-0.745	0.130	9.22E-09	0.515
UC	DNMT3A	2	25510652	rs7578575	25488819	A	T	0.329	-0.627	0.156	5.73E-05	0.541
UC	CFDP1	16	75327596	rs9934007	75390532	C	T	0.754	0.273	0.070	9.61E-05	0.973
CD	STAT3	17	40502964	rs1053004	40466092	G	A	0.380	0.702	0.128	4.40E-08	0.056
CD	IMPDH2	3	49064299	rs9813813	49207093	A	T	0.110	-1.233	0.294	2.74E-05	0.213
IBD	IMPDH2	3	49064299	rs9813813	49207093	A	T	0.110	-1.328	0.258	2.57E-07	0.071
UC	IMPDH2	3	49064299	rs9813813	49207093	A	T	0.110	-1.445	0.307	2.53E-06	0.050
IBD	UBE2L3	22	21941029	rs2070512	21949411	C	A	0.194	0.163	0.021	1.06E-14	0.169
UC	UBE2L3	22	21941029	rs2070512	21949411	C	A	0.194	0.116	0.027	1.76E-05	0.698
CD	BCL2L15	1	1.14E+08	rs1217393	1.14E+08	A	G	0.271	-0.705	0.170	3.39E-05	0.184
CD	STMN3	20	62271061	rs909333	62270637	G	T	0.207	0.547	0.102	8.31E-08	0.200
IBD	STMN3	20	62271061	rs909333	62270637	G	T	0.207	0.555	0.087	2.01E-10	0.055
UC	STMN3	20	62271061	rs909333	62270637	G	T	0.207	0.498	0.098	4.04E-07	0.062
GORD	PBX2	6	32154964	rs693906	31835164	C	G	0.113	0.154	0.036	1.65E-05	0.058
VHD	AGER	6	32150423	rs693906	31835164	C	G	0.113	0.212	0.052	4.82E-05	0.092

B) Nominally significant effect on VHD of Bonferroni-significant gastrointestinal loci.

Disease	Gene	chr	Probe_bp	topSNP	topSNP_bp	A1	A2	Freq	b_SMR	se_SMR	p_SMR	p_HEIDI
VHD	CFDP1	16	75397489	rs7201989	75471499	G	A	0.246	-0.056	0.018	0.001	0.119
VHD	STAT3	17	40502964	rs1053004	40466092	G	A	0.380	0.093	0.047	0.048	0.948
VHD	IMPDH2	3	49064299	rs34037363	48821036	T	C	0.114	-0.209	0.105	0.047	0.089
VHD	UBE2L3	22	21941029	rs2070512	21949411	C	A	0.194	-0.029	0.009	0.002	0.623
VHD	STMN3	20	62271061	rs909333	62270637	G	T	0.207	-0.076	0.036	0.036	0.093

C) Effect of AGER gene on VHD and gastrointestinal disorders.

Disease	Gene	chr	Probe_bp	topSNP	topSNP_bp	A1	A2	Freq	b_SMR	se_SMR	p_SMR	p_HEIDI
CD	AGER	6	32150423	rs693906	31835164	C	G	0.113	-0.312	0.120	0.009	6.16E-08
GORD	AGER	6	32150423	rs693906	31835164	C	G	0.113	-0.209	0.049	2.02E-05	0.022
IBD	AGER	6	32148745	rs204995	32154285	G	A	0.217	-0.252	0.108	0.020	1.60E-04
IBS	AGER	6	32150423	rs693906	31835164	C	G	0.113	-0.168	0.052	0.001	0.045
UC	AGER	6	32148745	rs204995	32154285	G	A	0.217	-0.870	0.185	2.59E-06	0.002
VHD	AGER	6	32150423	rs693906	31835164	C	G	0.113	0.212	0.052	4.82E-05	0.092

Supplementary table 6. Results of the summary-based Mendelian randomization integrating whole-blood expression quantitative trait loci. For each disease-gene association, we report the strongest effect observed between GTEX and eQTLGen datasets.

A) Bonferroni-significant disease-gene association with HEIDI P > 0.05.

Disease	Gene	chr	gene_bp	topSNP	topSNP_bp	A1	A2	Freq	b_SMR	se_SMR	p_SMR	p_HEIDI
CD	DNMT3A	2	25510652	rs7578575	25488819	A	T	0.329	-0.834	0.164	3.51E-07	0.383
IBD	DNMT3A	2	25510652	rs7578575	25488819	A	T	0.329	-0.745	0.130	9.22E-09	0.515
UC	DNMT3A	2	25510652	rs7578575	25488819	A	T	0.329	-0.627	0.156	5.73E-05	0.541
UC	CFDP1	16	75327596	rs9934007	75390532	C	T	0.754	0.273	0.070	9.61E-05	0.973
CD	STAT3	17	40502964	rs1053004	40466092	G	A	0.380	0.702	0.128	4.40E-08	0.056
CD	IMPDH2	3	49064299	rs9813813	49207093	A	T	0.110	-1.233	0.294	2.74E-05	0.213
IBD	IMPDH2	3	49064299	rs9813813	49207093	A	T	0.110	-1.328	0.258	2.57E-07	0.071
UC	IMPDH2	3	49064299	rs9813813	49207093	A	T	0.110	-1.445	0.307	2.53E-06	0.050
IBD	UBE2L3	22	21941029	rs2070512	21949411	C	A	0.194	0.163	0.021	1.06E-14	0.169
UC	UBE2L3	22	21941029	rs2070512	21949411	C	A	0.194	0.116	0.027	1.76E-05	0.698
CD	BCL2L15	1	1.14E+08	rs1217393	1.14E+08	A	G	0.271	-0.705	0.170	3.39E-05	0.184
CD	STMN3	20	62271061	rs909333	62270637	G	T	0.207	0.547	0.102	8.31E-08	0.200
IBD	STMN3	20	62271061	rs909333	62270637	G	T	0.207	0.555	0.087	2.01E-10	0.055
UC	STMN3	20	62271061	rs909333	62270637	G	T	0.207	0.498	0.098	4.04E-07	0.062
GORD	PBX2	6	32154964	rs693906	31835164	C	G	0.113	0.154	0.036	1.65E-05	0.058
VHD	AGER	6	32150423	rs693906	31835164	C	G	0.113	0.212	0.052	4.82E-05	0.092

B) Nominally significant effect on VHD of Bonferroni-significant gastrointestinal loci.

Disease	Gene	chr	Probe_bp	topSNP	topSNP_bp	A1	A2	Freq	b_SMR	se_SMR	p_SMR	p_HEIDI
VHD	CFDP1	16	75397489	rs7201989	75471499	G	A	0.246	-0.056	0.018	0.001	0.119
VHD	STAT3	17	40502964	rs1053004	40466092	G	A	0.380	0.093	0.047	0.048	0.948
VHD	IMPDH2	3	49064299	rs34037363	48821036	T	C	0.114	-0.209	0.105	0.047	0.089
VHD	UBE2L3	22	21941029	rs2070512	21949411	C	A	0.194	-0.029	0.009	0.002	0.623
VHD	STMN3	20	62271061	rs909333	62270637	G	T	0.207	-0.076	0.036	0.036	0.093

C) Effect of AGER gene on VHD and gastrointestinal disorders.

Disease	Gene	chr	Probe_bp	topSNP	topSNP_bp	A1	A2	Freq	b_SMR	se_SMR	p_SMR	p_HEIDI
CD	AGER	6	32150423	rs693906	31835164	C	G	0.113	-0.312	0.120	0.009	6.16E-08
GORD	AGER	6	32150423	rs693906	31835164	C	G	0.113	-0.209	0.049	2.02E-05	0.022
IBD	AGER	6	32148745	rs204995	32154285	G	A	0.217	-0.252	0.108	0.020	1.60E-04
IBS	AGER	6	32150423	rs693906	31835164	C	G	0.113	-0.168	0.052	0.001	0.045
UC	AGER	6	32148745	rs204995	32154285	G	A	0.217	-0.870	0.185	2.59E-06	0.002
VHD	AGER	6	32150423	rs693906	31835164	C	G	0.113	0.212	0.052	4.82E-05	0.092

Supplementary table 6. Results of the summary-based Mendelian randomization integrating whole-blood expression quantitative trait loci. For each disease-gene association, we report the strongest effect observed between GTEX and eQTLGen datasets.

A) Bonferroni-significant disease-gene association with HEIDI P > 0.05.

Disease	Gene	chr	gene_bp	topSNP	topSNP_bp	A1	A2	Freq	b_SMR	se_SMR	p_SMR	p_HEIDI
CD	DNMT3A	2	25510652	rs7578575	25488819	A	T	0.329	-0.834	0.164	3.51E-07	0.383
IBD	DNMT3A	2	25510652	rs7578575	25488819	A	T	0.329	-0.745	0.130	9.22E-09	0.515
UC	DNMT3A	2	25510652	rs7578575	25488819	A	T	0.329	-0.627	0.156	5.73E-05	0.541
UC	CFDP1	16	75327596	rs9934007	75390532	C	T	0.754	0.273	0.070	9.61E-05	0.973
CD	STAT3	17	40502964	rs1053004	40466092	G	A	0.380	0.702	0.128	4.40E-08	0.056
CD	IMPDH2	3	49064299	rs9813813	49207093	A	T	0.110	-1.233	0.294	2.74E-05	0.213
IBD	IMPDH2	3	49064299	rs9813813	49207093	A	T	0.110	-1.328	0.258	2.57E-07	0.071
UC	IMPDH2	3	49064299	rs9813813	49207093	A	T	0.110	-1.445	0.307	2.53E-06	0.050
IBD	UBE2L3	22	21941029	rs2070512	21949411	C	A	0.194	0.163	0.021	1.06E-14	0.169
UC	UBE2L3	22	21941029	rs2070512	21949411	C	A	0.194	0.116	0.027	1.76E-05	0.698
CD	BCL2L15	1	1.14E+08	rs1217393	1.14E+08	A	G	0.271	-0.705	0.170	3.39E-05	0.184
CD	STMN3	20	62271061	rs909333	62270637	G	T	0.207	0.547	0.102	8.31E-08	0.200
IBD	STMN3	20	62271061	rs909333	62270637	G	T	0.207	0.555	0.087	2.01E-10	0.055
UC	STMN3	20	62271061	rs909333	62270637	G	T	0.207	0.498	0.098	4.04E-07	0.062
GORD	PBX2	6	32154964	rs693906	31835164	C	G	0.113	0.154	0.036	1.65E-05	0.058
VHD	AGER	6	32150423	rs693906	31835164	C	G	0.113	0.212	0.052	4.82E-05	0.092

B) Nominally significant effect on VHD of Bonferroni-significant gastrointestinal loci.

Disease	Gene	chr	Probe_bp	topSNP	topSNP_bp	A1	A2	Freq	b_SMR	se_SMR	p_SMR	p_HEIDI
VHD	CFDP1	16	75397489	rs7201989	75471499	G	A	0.246	-0.056	0.018	0.001	0.119
VHD	STAT3	17	40502964	rs1053004	40466092	G	A	0.380	0.093	0.047	0.048	0.948
VHD	IMPDH2	3	49064299	rs34037363	48821036	T	C	0.114	-0.209	0.105	0.047	0.089
VHD	UBE2L3	22	21941029	rs2070512	21949411	C	A	0.194	-0.029	0.009	0.002	0.623
VHD	STMN3	20	62271061	rs909333	62270637	G	T	0.207	-0.076	0.036	0.036	0.093

C) Effect of AGER gene on VHD and gastrointestinal disorders.

Disease	Gene	chr	Probe_bp	topSNP	topSNP_bp	A1	A2	Freq	b_SMR	se_SMR	p_SMR	p_HEIDI
CD	AGER	6	32150423	rs693906	31835164	C	G	0.113	-0.312	0.120	0.009	6.16E-08
GORD	AGER	6	32150423	rs693906	31835164	C	G	0.113	-0.209	0.049	2.02E-05	0.022
IBD	AGER	6	32148745	rs204995	32154285	G	A	0.217	-0.252	0.108	0.020	1.60E-04
IBS	AGER	6	32150423	rs693906	31835164	C	G	0.113	-0.168	0.052	0.001	0.045
UC	AGER	6	32148745	rs204995	32154285	G	A	0.217	-0.870	0.185	2.59E-06	0.002
VHD	AGER	6	32150423	rs693906	31835164	C	G	0.113	0.212	0.052	4.82E-05	0.092

Supplementary table 6. Results of the summary-based Mendelian randomization integrating whole-blood expression quantitative trait loci. For each disease-gene association, we report the strongest effect observed between GTEX and eQTLGen datasets.

A) Bonferroni-significant disease-gene association with HEIDI P > 0.05.

Disease	Gene	chr	gene_bp	topSNP	topSNP_bp	A1	A2	Freq	b_SMR	se_SMR	p_SMR	p_HEIDI
CD	DNMT3A	2	25510652	rs7578575	25488819	A	T	0.329	-0.834	0.164	3.51E-07	0.383
IBD	DNMT3A	2	25510652	rs7578575	25488819	A	T	0.329	-0.745	0.130	9.22E-09	0.515
UC	DNMT3A	2	25510652	rs7578575	25488819	A	T	0.329	-0.627	0.156	5.73E-05	0.541
UC	CFDP1	16	75327596	rs9934007	75390532	C	T	0.754	0.273	0.070	9.61E-05	0.973
CD	STAT3	17	40502964	rs1053004	40466092	G	A	0.380	0.702	0.128	4.40E-08	0.056
CD	IMPDH2	3	49064299	rs9813813	49207093	A	T	0.110	-1.233	0.294	2.74E-05	0.213
IBD	IMPDH2	3	49064299	rs9813813	49207093	A	T	0.110	-1.328	0.258	2.57E-07	0.071
UC	IMPDH2	3	49064299	rs9813813	49207093	A	T	0.110	-1.445	0.307	2.53E-06	0.050
IBD	UBE2L3	22	21941029	rs2070512	21949411	C	A	0.194	0.163	0.021	1.06E-14	0.169
UC	UBE2L3	22	21941029	rs2070512	21949411	C	A	0.194	0.116	0.027	1.76E-05	0.698
CD	BCL2L15	1	1.14E+08	rs1217393	1.14E+08	A	G	0.271	-0.705	0.170	3.39E-05	0.184
CD	STMN3	20	62271061	rs909333	62270637	G	T	0.207	0.547	0.102	8.31E-08	0.200
IBD	STMN3	20	62271061	rs909333	62270637	G	T	0.207	0.555	0.087	2.01E-10	0.055
UC	STMN3	20	62271061	rs909333	62270637	G	T	0.207	0.498	0.098	4.04E-07	0.062
GORD	PBX2	6	32154964	rs693906	31835164	C	G	0.113	0.154	0.036	1.65E-05	0.058
VHD	AGER	6	32150423	rs693906	31835164	C	G	0.113	0.212	0.052	4.82E-05	0.092

B) Nominally significant effect on VHD of Bonferroni-significant gastrointestinal loci.

Disease	Gene	chr	Probe_bp	topSNP	topSNP_bp	A1	A2	Freq	b_SMR	se_SMR	p_SMR	p_HEIDI
VHD	CFDP1	16	75397489	rs7201989	75471499	G	A	0.246	-0.056	0.018	0.001	0.119
VHD	STAT3	17	40502964	rs1053004	40466092	G	A	0.380	0.093	0.047	0.048	0.948
VHD	IMPDH2	3	49064299	rs34037363	48821036	T	C	0.114	-0.209	0.105	0.047	0.089
VHD	UBE2L3	22	21941029	rs2070512	21949411	C	A	0.194	-0.029	0.009	0.002	0.623
VHD	STMN3	20	62271061	rs909333	62270637	G	T	0.207	-0.076	0.036	0.036	0.093

C) Effect of AGER gene on VHD and gastrointestinal disorders.

Disease	Gene	chr	Probe_bp	topSNP	topSNP_bp	A1	A2	Freq	b_SMR	se_SMR	p_SMR	p_HEIDI
CD	AGER	6	32150423	rs693906	31835164	C	G	0.113	-0.312	0.120	0.009	6.16E-08
GORD	AGER	6	32150423	rs693906	31835164	C	G	0.113	-0.209	0.049	2.02E-05	0.022
IBD	AGER	6	32148745	rs204995	32154285	G	A	0.217	-0.252	0.108	0.020	1.60E-04
IBS	AGER	6	32150423	rs693906	31835164	C	G	0.113	-0.168	0.052	0.001	0.045
UC	AGER	6	32148745	rs204995	32154285	G	A	0.217	-0.870	0.185	2.59E-06	0.002
VHD	AGER	6	32150423	rs693906	31835164	C	G	0.113	0.212	0.052	4.82E-05	0.092

Supplementary table 6. Results of the summary-based Mendelian randomization integrating whole-blood expression quantitative trait loci. For each disease-gene association, we report the strongest effect observed between GTEX and eQTLGen datasets.

A) Bonferroni-significant disease-gene association with HEIDI P > 0.05.

Disease	Gene	chr	gene_bp	topSNP	topSNP_bp	A1	A2	Freq	b_SMR	se_SMR	p_SMR	p_HEIDI
CD	DNMT3A	2	25510652	rs7578575	25488819	A	T	0.329	-0.834	0.164	3.51E-07	0.383
IBD	DNMT3A	2	25510652	rs7578575	25488819	A	T	0.329	-0.745	0.130	9.22E-09	0.515
UC	DNMT3A	2	25510652	rs7578575	25488819	A	T	0.329	-0.627	0.156	5.73E-05	0.541
UC	CFDP1	16	75327596	rs9934007	75390532	C	T	0.754	0.273	0.070	9.61E-05	0.973
CD	STAT3	17	40502964	rs1053004	40466092	G	A	0.380	0.702	0.128	4.40E-08	0.056
CD	IMPDH2	3	49064299	rs9813813	49207093	A	T	0.110	-1.233	0.294	2.74E-05	0.213
IBD	IMPDH2	3	49064299	rs9813813	49207093	A	T	0.110	-1.328	0.258	2.57E-07	0.071
UC	IMPDH2	3	49064299	rs9813813	49207093	A	T	0.110	-1.445	0.307	2.53E-06	0.050
IBD	UBE2L3	22	21941029	rs2070512	21949411	C	A	0.194	0.163	0.021	1.06E-14	0.169
UC	UBE2L3	22	21941029	rs2070512	21949411	C	A	0.194	0.116	0.027	1.76E-05	0.698
CD	BCL2L15	1	1.14E+08	rs1217393	1.14E+08	A	G	0.271	-0.705	0.170	3.39E-05	0.184
CD	STMN3	20	62271061	rs909333	62270637	G	T	0.207	0.547	0.102	8.31E-08	0.200
IBD	STMN3	20	62271061	rs909333	62270637	G	T	0.207	0.555	0.087	2.01E-10	0.055
UC	STMN3	20	62271061	rs909333	62270637	G	T	0.207	0.498	0.098	4.04E-07	0.062
GORD	PBX2	6	32154964	rs693906	31835164	C	G	0.113	0.154	0.036	1.65E-05	0.058
VHD	AGER	6	32150423	rs693906	31835164	C	G	0.113	0.212	0.052	4.82E-05	0.092

B) Nominally significant effect on VHD of Bonferroni-significant gastrointestinal loci.

Disease	Gene	chr	Probe_bp	topSNP	topSNP_bp	A1	A2	Freq	b_SMR	se_SMR	p_SMR	p_HEIDI
VHD	CFDP1	16	75397489	rs7201989	75471499	G	A	0.246	-0.056	0.018	0.001	0.119
VHD	STAT3	17	40502964	rs1053004	40466092	G	A	0.380	0.093	0.047	0.048	0.948
VHD	IMPDH2	3	49064299	rs34037363	48821036	T	C	0.114	-0.209	0.105	0.047	0.089
VHD	UBE2L3	22	21941029	rs2070512	21949411	C	A	0.194	-0.029	0.009	0.002	0.623
VHD	STMN3	20	62271061	rs909333	62270637	G	T	0.207	-0.076	0.036	0.036	0.093

C) Effect of AGER gene on VHD and gastrointestinal disorders.

Disease	Gene	chr	Probe_bp	topSNP	topSNP_bp	A1	A2	Freq	b_SMR	se_SMR	p_SMR	p_HEIDI
CD	AGER	6	32150423	rs693906	31835164	C	G	0.113	-0.312	0.120	0.009	6.16E-08
GORD	AGER	6	32150423	rs693906	31835164	C	G	0.113	-0.209	0.049	2.02E-05	0.022
IBD	AGER	6	32148745	rs204995	32154285	G	A	0.217	-0.252	0.108	0.020	1.60E-04
IBS	AGER	6	32150423	rs693906	31835164	C	G	0.113	-0.168	0.052	0.001	0.045
UC	AGER	6	32148745	rs204995	32154285	G	A	0.217	-0.870	0.185	2.59E-06	0.002
VHD	AGER	6	32150423	rs693906	31835164	C	G	0.113	0.212	0.052	4.82E-05	0.092

Supplementary table 6. Results of the summary-based Mendelian randomization integrating whole-blood expression quantitative trait loci. For each disease-gene association, we report the strongest effect observed between GTEX and eQTLGen datasets.

A) Bonferroni-significant disease-gene association with HEIDI P > 0.05.

Disease	Gene	chr	gene_bp	topSNP	topSNP_bp	A1	A2	Freq	b_SMR	se_SMR	p_SMR	p_HEIDI
CD	DNMT3A	2	25510652	rs7578575	25488819	A	T	0.329	-0.834	0.164	3.51E-07	0.383
IBD	DNMT3A	2	25510652	rs7578575	25488819	A	T	0.329	-0.745	0.130	9.22E-09	0.515
UC	DNMT3A	2	25510652	rs7578575	25488819	A	T	0.329	-0.627	0.156	5.73E-05	0.541
UC	CFDP1	16	75327596	rs9934007	75390532	C	T	0.754	0.273	0.070	9.61E-05	0.973
CD	STAT3	17	40502964	rs1053004	40466092	G	A	0.380	0.702	0.128	4.40E-08	0.056
CD	IMPDH2	3	49064299	rs9813813	49207093	A	T	0.110	-1.233	0.294	2.74E-05	0.213
IBD	IMPDH2	3	49064299	rs9813813	49207093	A	T	0.110	-1.328	0.258	2.57E-07	0.071
UC	IMPDH2	3	49064299	rs9813813	49207093	A	T	0.110	-1.445	0.307	2.53E-06	0.050
IBD	UBE2L3	22	21941029	rs2070512	21949411	C	A	0.194	0.163	0.021	1.06E-14	0.169
UC	UBE2L3	22	21941029	rs2070512	21949411	C	A	0.194	0.116	0.027	1.76E-05	0.698
CD	BCL2L15	1	1.14E+08	rs1217393	1.14E+08	A	G	0.271	-0.705	0.170	3.39E-05	0.184
CD	STMN3	20	62271061	rs909333	62270637	G	T	0.207	0.547	0.102	8.31E-08	0.200
IBD	STMN3	20	62271061	rs909333	62270637	G	T	0.207	0.555	0.087	2.01E-10	0.055
UC	STMN3	20	62271061	rs909333	62270637	G	T	0.207	0.498	0.098	4.04E-07	0.062
GORD	PBX2	6	32154964	rs693906	31835164	C	G	0.113	0.154	0.036	1.65E-05	0.058
VHD	AGER	6	32150423	rs693906	31835164	C	G	0.113	0.212	0.052	4.82E-05	0.092

B) Nominally significant effect on VHD of Bonferroni-significant gastrointestinal loci.

Disease	Gene	chr	Probe_bp	topSNP	topSNP_bp	A1	A2	Freq	b_SMR	se_SMR	p_SMR	p_HEIDI
VHD	CFDP1	16	75397489	rs7201989	75471499	G	A	0.246	-0.056	0.018	0.001	0.119
VHD	STAT3	17	40502964	rs1053004	40466092	G	A	0.380	0.093	0.047	0.048	0.948
VHD	IMPDH2	3	49064299	rs34037363	48821036	T	C	0.114	-0.209	0.105	0.047	0.089
VHD	UBE2L3	22	21941029	rs2070512	21949411	C	A	0.194	-0.029	0.009	0.002	0.623
VHD	STMN3	20	62271061	rs909333	62270637	G	T	0.207	-0.076	0.036	0.036	0.093

C) Effect of AGER gene on VHD and gastrointestinal disorders.

Disease	Gene	chr	Probe_bp	topSNP	topSNP_bp	A1	A2	Freq	b_SMR	se_SMR	p_SMR	p_HEIDI
CD	AGER	6	32150423	rs693906	31835164	C	G	0.113	-0.312	0.120	0.009	6.16E-08
GORD	AGER	6	32150423	rs693906	31835164	C	G	0.113	-0.209	0.049	2.02E-05	0.022
IBD	AGER	6	32148745	rs204995	32154285	G	A	0.217	-0.252	0.108	0.020	1.60E-04
IBS	AGER	6	32150423	rs693906	31835164	C	G	0.113	-0.168	0.052	0.001	0.045
UC	AGER	6	32148745	rs204995	32154285	G	A	0.217	-0.870	0.185	2.59E-06	0.002
VHD	AGER	6	32150423	rs693906	31835164	C	G	0.113	0.212	0.052	4.82E-05	0.092

Supplementary table 6. Results of the summary-based Mendelian randomization integrating whole-blood expression quantitative trait loci. For each disease-gene association, we report the strongest effect observed between GTEX and eQTLGen datasets.

A) Bonferroni-significant disease-gene association with HEIDI P > 0.05.

Disease	Gene	chr	gene_bp	topSNP	topSNP_bp	A1	A2	Freq	b_SMR	se_SMR	p_SMR	p_HEIDI
CD	DNMT3A	2	25510652	rs7578575	25488819	A	T	0.329	-0.834	0.164	3.51E-07	0.383
IBD	DNMT3A	2	25510652	rs7578575	25488819	A	T	0.329	-0.745	0.130	9.22E-09	0.515
UC	DNMT3A	2	25510652	rs7578575	25488819	A	T	0.329	-0.627	0.156	5.73E-05	0.541
UC	CFDP1	16	75327596	rs9934007	75390532	C	T	0.754	0.273	0.070	9.61E-05	0.973
CD	STAT3	17	40502964	rs1053004	40466092	G	A	0.380	0.702	0.128	4.40E-08	0.056
CD	IMPDH2	3	49064299	rs9813813	49207093	A	T	0.110	-1.233	0.294	2.74E-05	0.213
IBD	IMPDH2	3	49064299	rs9813813	49207093	A	T	0.110	-1.328	0.258	2.57E-07	0.071
UC	IMPDH2	3	49064299	rs9813813	49207093	A	T	0.110	-1.445	0.307	2.53E-06	0.050
IBD	UBE2L3	22	21941029	rs2070512	21949411	C	A	0.194	0.163	0.021	1.06E-14	0.169
UC	UBE2L3	22	21941029	rs2070512	21949411	C	A	0.194	0.116	0.027	1.76E-05	0.698
CD	BCL2L15	1	1.14E+08	rs1217393	1.14E+08	A	G	0.271	-0.705	0.170	3.39E-05	0.184
CD	STMN3	20	62271061	rs909333	62270637	G	T	0.207	0.547	0.102	8.31E-08	0.200
IBD	STMN3	20	62271061	rs909333	62270637	G	T	0.207	0.555	0.087	2.01E-10	0.055
UC	STMN3	20	62271061	rs909333	62270637	G	T	0.207	0.498	0.098	4.04E-07	0.062
GORD	PBX2	6	32154964	rs693906	31835164	C	G	0.113	0.154	0.036	1.65E-05	0.058
VHD	AGER	6	32150423	rs693906	31835164	C	G	0.113	0.212	0.052	4.82E-05	0.092

B) Nominally significant effect on VHD of Bonferroni-significant gastrointestinal loci.

Disease	Gene	chr	Probe_bp	topSNP	topSNP_bp	A1	A2	Freq	b_SMR	se_SMR	p_SMR	p_HEIDI
VHD	CFDP1	16	75397489	rs7201989	75471499	G	A	0.246	-0.056	0.018	0.001	0.119
VHD	STAT3	17	40502964	rs1053004	40466092	G	A	0.380	0.093	0.047	0.048	0.948
VHD	IMPDH2	3	49064299	rs34037363	48821036	T	C	0.114	-0.209	0.105	0.047	0.089
VHD	UBE2L3	22	21941029	rs2070512	21949411	C	A	0.194	-0.029	0.009	0.002	0.623
VHD	STMN3	20	62271061	rs909333	62270637	G	T	0.207	-0.076	0.036	0.036	0.093

C) Effect of AGER gene on VHD and gastrointestinal disorders.

Disease	Gene	chr	Probe_bp	topSNP	topSNP_bp	A1	A2	Freq	b_SMR	se_SMR	p_SMR	p_HEIDI
CD	AGER	6	32150423	rs693906	31835164	C	G	0.113	-0.312	0.120	0.009	6.16E-08
GORD	AGER	6	32150423	rs693906	31835164	C	G	0.113	-0.209	0.049	2.02E-05	0.022
IBD	AGER	6	32148745	rs204995	32154285	G	A	0.217	-0.252	0.108	0.020	1.60E-04
IBS	AGER	6	32150423	rs693906	31835164	C	G	0.113	-0.168	0.052	0.001	0.045
UC	AGER	6	32148745	rs204995	32154285	G	A	0.217	-0.870	0.185	2.59E-06	0.002
VHD	AGER	6	32150423	rs693906	31835164	C	G	0.113	0.212	0.052	4.82E-05	0.092

Supplementary table 6. Results of the summary-based Mendelian randomization integrating whole-blood expression quantitative trait loci. For each disease-gene association, we report the strongest effect observed between GTEX and eQTLGen datasets.

A) Bonferroni-significant disease-gene association with HEIDI P > 0.05.

Disease	Gene	chr	gene_bp	topSNP	topSNP_bp	A1	A2	Freq	b_SMR	se_SMR	p_SMR	p_HEIDI
CD	DNMT3A	2	25510652	rs7578575	25488819	A	T	0.329	-0.834	0.164	3.51E-07	0.383
IBD	DNMT3A	2	25510652	rs7578575	25488819	A	T	0.329	-0.745	0.130	9.22E-09	0.515
UC	DNMT3A	2	25510652	rs7578575	25488819	A	T	0.329	-0.627	0.156	5.73E-05	0.541
UC	CFDP1	16	75327596	rs9934007	75390532	C	T	0.754	0.273	0.070	9.61E-05	0.973
CD	STAT3	17	40502964	rs1053004	40466092	G	A	0.380	0.702	0.128	4.40E-08	0.056
CD	IMPDH2	3	49064299	rs9813813	49207093	A	T	0.110	-1.233	0.294	2.74E-05	0.213
IBD	IMPDH2	3	49064299	rs9813813	49207093	A	T	0.110	-1.328	0.258	2.57E-07	0.071
UC	IMPDH2	3	49064299	rs9813813	49207093	A	T	0.110	-1.445	0.307	2.53E-06	0.050
IBD	UBE2L3	22	21941029	rs2070512	21949411	C	A	0.194	0.163	0.021	1.06E-14	0.169
UC	UBE2L3	22	21941029	rs2070512	21949411	C	A	0.194	0.116	0.027	1.76E-05	0.698
CD	BCL2L15	1	1.14E+08	rs1217393	1.14E+08	A	G	0.271	-0.705	0.170	3.39E-05	0.184
CD	STMN3	20	62271061	rs909333	62270637	G	T	0.207	0.547	0.102	8.31E-08	0.200
IBD	STMN3	20	62271061	rs909333	62270637	G	T	0.207	0.555	0.087	2.01E-10	0.055
UC	STMN3	20	62271061	rs909333	62270637	G	T	0.207	0.498	0.098	4.04E-07	0.062
GORD	PBX2	6	32154964	rs693906	31835164	C	G	0.113	0.154	0.036	1.65E-05	0.058
VHD	AGER	6	32150423	rs693906	31835164	C	G	0.113	0.212	0.052	4.82E-05	0.092

B) Nominally significant effect on VHD of Bonferroni-significant gastrointestinal loci.

Disease	Gene	chr	Probe_bp	topSNP	topSNP_bp	A1	A2	Freq	b_SMR	se_SMR	p_SMR	p_HEIDI
VHD	CFDP1	16	75397489	rs7201989	75471499	G	A	0.246	-0.056	0.018	0.001	0.119
VHD	STAT3	17	40502964	rs1053004	40466092	G	A	0.380	0.093	0.047	0.048	0.948
VHD	IMPDH2	3	49064299	rs34037363	48821036	T	C	0.114	-0.209	0.105	0.047	0.089
VHD	UBE2L3	22	21941029	rs2070512	21949411	C	A	0.194	-0.029	0.009	0.002	0.623
VHD	STMN3	20	62271061	rs909333	62270637	G	T	0.207	-0.076	0.036	0.036	0.093

C) Effect of AGER gene on VHD and gastrointestinal disorders.

Disease	Gene	chr	Probe_bp	topSNP	topSNP_bp	A1	A2	Freq	b_SMR	se_SMR	p_SMR	p_HEIDI
CD	AGER	6	32150423	rs693906	31835164	C	G	0.113	-0.312	0.120	0.009	6.16E-08
GORD	AGER	6	32150423	rs693906	31835164	C	G	0.113	-0.209	0.049	2.02E-05	0.022
IBD	AGER	6	32148745	rs204995	32154285	G	A	0.217	-0.252	0.108	0.020	1.60E-04
IBS	AGER	6	32150423	rs693906	31835164	C	G	0.113	-0.168	0.052	0.001	0.045
UC	AGER	6	32148745	rs204995	32154285	G	A	0.217	-0.870	0.185	2.59E-06	0.002
VHD	AGER	6	32150423	rs693906	31835164	C	G	0.113	0.212	0.052	4.82E-05	0.092

Supplementary table 6. Results of the summary-based Mendelian randomization integrating whole-blood expression quantitative trait loci. For each disease-gene association, we report the strongest effect observed between GTEX and eQTLGen datasets.

A) Bonferroni-significant disease-gene association with HEIDI P > 0.05.

Disease	Gene	chr	gene_bp	topSNP	topSNP_bp	A1	A2	Freq	b_SMR	se_SMR	p_SMR	p_HEIDI
CD	DNMT3A	2	25510652	rs7578575	25488819	A	T	0.329	-0.834	0.164	3.51E-07	0.383
IBD	DNMT3A	2	25510652	rs7578575	25488819	A	T	0.329	-0.745	0.130	9.22E-09	0.515
UC	DNMT3A	2	25510652	rs7578575	25488819	A	T	0.329	-0.627	0.156	5.73E-05	0.541
UC	CFDP1	16	75327596	rs9934007	75390532	C	T	0.754	0.273	0.070	9.61E-05	0.973
CD	STAT3	17	40502964	rs1053004	40466092	G	A	0.380	0.702	0.128	4.40E-08	0.056
CD	IMPDH2	3	49064299	rs9813813	49207093	A	T	0.110	-1.233	0.294	2.74E-05	0.213
IBD	IMPDH2	3	49064299	rs9813813	49207093	A	T	0.110	-1.328	0.258	2.57E-07	0.071
UC	IMPDH2	3	49064299	rs9813813	49207093	A	T	0.110	-1.445	0.307	2.53E-06	0.050
IBD	UBE2L3	22	21941029	rs2070512	21949411	C	A	0.194	0.163	0.021	1.06E-14	0.169
UC	UBE2L3	22	21941029	rs2070512	21949411	C	A	0.194	0.116	0.027	1.76E-05	0.698
CD	BCL2L15	1	1.14E+08	rs1217393	1.14E+08	A	G	0.271	-0.705	0.170	3.39E-05	0.184
CD	STMN3	20	62271061	rs909333	62270637	G	T	0.207	0.547	0.102	8.31E-08	0.200
IBD	STMN3	20	62271061	rs909333	62270637	G	T	0.207	0.555	0.087	2.01E-10	0.055
UC	STMN3	20	62271061	rs909333	62270637	G	T	0.207	0.498	0.098	4.04E-07	0.062
GORD	PBX2	6	32154964	rs693906	31835164	C	G	0.113	0.154	0.036	1.65E-05	0.058
VHD	AGER	6	32150423	rs693906	31835164	C	G	0.113	0.212	0.052	4.82E-05	0.092

B) Nominally significant effect on VHD of Bonferroni-significant gastrointestinal loci.

Disease	Gene	chr	Probe_bp	topSNP	topSNP_bp	A1	A2	Freq	b_SMR	se_SMR	p_SMR	p_HEIDI
VHD	CFDP1	16	75397489	rs7201989	75471499	G	A	0.246	-0.056	0.018	0.001	0.119
VHD	STAT3	17	40502964	rs1053004	40466092	G	A	0.380	0.093	0.047	0.048	0.948
VHD	IMPDH2	3	49064299	rs34037363	48821036	T	C	0.114	-0.209	0.105	0.047	0.089
VHD	UBE2L3	22	21941029	rs2070512	21949411	C	A	0.194	-0.029	0.009	0.002	0.623
VHD	STMN3	20	62271061	rs909333	62270637	G	T	0.207	-0.076	0.036	0.036	0.093

C) Effect of AGER gene on VHD and gastrointestinal disorders.

Disease	Gene	chr	Probe_bp	topSNP	topSNP_bp	A1	A2	Freq	b_SMR	se_SMR	p_SMR	p_HEIDI
CD	AGER	6	32150423	rs693906	31835164	C	G	0.113	-0.312	0.120	0.009	6.16E-08
GORD	AGER	6	32150423	rs693906	31835164	C	G	0.113	-0.209	0.049	2.02E-05	0.022
IBD	AGER	6	32148745	rs204995	32154285	G	A	0.217	-0.252	0.108	0.020	1.60E-04
IBS	AGER	6	32150423	rs693906	31835164	C	G	0.113	-0.168	0.052	0.001	0.045
UC	AGER	6	32148745	rs204995	32154285	G	A	0.217	-0.870	0.185	2.59E-06	0.002
VHD	AGER	6	32150423	rs693906	31835164	C	G	0.113	0.212	0.052	4.82E-05	0.092

Supplementary table 6. Results of the summary-based Mendelian randomization integrating whole-blood expression quantitative trait loci. For each disease-gene association, we report the strongest effect observed between GTEX and eQTLGen datasets.

A) Bonferroni-significant disease-gene association with HEIDI P > 0.05.

Disease	Gene	chr	gene_bp	topSNP	topSNP_bp	A1	A2	Freq	b_SMR	se_SMR	p_SMR	p_HEIDI
CD	DNMT3A	2	25510652	rs7578575	25488819	A	T	0.329	-0.834	0.164	3.51E-07	0.383
IBD	DNMT3A	2	25510652	rs7578575	25488819	A	T	0.329	-0.745	0.130	9.22E-09	0.515
UC	DNMT3A	2	25510652	rs7578575	25488819	A	T	0.329	-0.627	0.156	5.73E-05	0.541
UC	CFDP1	16	75327596	rs9934007	75390532	C	T	0.754	0.273	0.070	9.61E-05	0.973
CD	STAT3	17	40502964	rs1053004	40466092	G	A	0.380	0.702	0.128	4.40E-08	0.056
CD	IMPDH2	3	49064299	rs9813813	49207093	A	T	0.110	-1.233	0.294	2.74E-05	0.213
IBD	IMPDH2	3	49064299	rs9813813	49207093	A	T	0.110	-1.328	0.258	2.57E-07	0.071
UC	IMPDH2	3	49064299	rs9813813	49207093	A	T	0.110	-1.445	0.307	2.53E-06	0.050
IBD	UBE2L3	22	21941029	rs2070512	21949411	C	A	0.194	0.163	0.021	1.06E-14	0.169
UC	UBE2L3	22	21941029	rs2070512	21949411	C	A	0.194	0.116	0.027	1.76E-05	0.698
CD	BCL2L15	1	1.14E+08	rs1217393	1.14E+08	A	G	0.271	-0.705	0.170	3.39E-05	0.184
CD	STMN3	20	62271061	rs909333	62270637	G	T	0.207	0.547	0.102	8.31E-08	0.200
IBD	STMN3	20	62271061	rs909333	62270637	G	T	0.207	0.555	0.087	2.01E-10	0.055
UC	STMN3	20	62271061	rs909333	62270637	G	T	0.207	0.498	0.098	4.04E-07	0.062
GORD	PBX2	6	32154964	rs693906	31835164	C	G	0.113	0.154	0.036	1.65E-05	0.058
VHD	AGER	6	32150423	rs693906	31835164	C	G	0.113	0.212	0.052	4.82E-05	0.092

B) Nominally significant effect on VHD of Bonferroni-significant gastrointestinal loci.

Disease	Gene	chr	Probe_bp	topSNP	topSNP_bp	A1	A2	Freq	b_SMR	se_SMR	p_SMR	p_HEIDI
VHD	CFDP1	16	75397489	rs7201989	75471499	G	A	0.246	-0.056	0.018	0.001	0.119
VHD	STAT3	17	40502964	rs1053004	40466092	G	A	0.380	0.093	0.047	0.048	0.948
VHD	IMPDH2	3	49064299	rs34037363	48821036	T	C	0.114	-0.209	0.105	0.047	0.089
VHD	UBE2L3	22	21941029	rs2070512	21949411	C	A	0.194	-0.029	0.009	0.002	0.623
VHD	STMN3	20	62271061	rs909333	62270637	G	T	0.207	-0.076	0.036	0.036	0.093

C) Effect of AGER gene on VHD and gastrointestinal disorders.

Disease	Gene	chr	Probe_bp	topSNP	topSNP_bp	A1	A2	Freq	b_SMR	se_SMR	p_SMR	p_HEIDI
CD	AGER	6	32150423	rs693906	31835164	C	G	0.113	-0.312	0.120	0.009	6.16E-08
GORD	AGER	6	32150423	rs693906	31835164	C	G	0.113	-0.209	0.049	2.02E-05	0.022
IBD	AGER	6	32148745	rs204995	32154285	G	A	0.217	-0.252	0.108	0.020	1.60E-04
IBS	AGER	6	32150423	rs693906	31835164	C	G	0.113	-0.168	0.052	0.001	0.045
UC	AGER	6	32148745	rs204995	32154285	G	A	0.217	-0.870	0.185	2.59E-06	0.002
VHD	AGER	6	32150423	rs693906	31835164	C	G	0.113	0.212	0.052	4.82E-05	0.092

Supplementary table 6. Results of the summary-based Mendelian randomization integrating whole-blood expression quantitative trait loci. For each disease-gene association, we report the strongest effect observed between GTEX and eQTLGen datasets.

A) Bonferroni-significant disease-gene association with HEIDI P > 0.05.

Disease	Gene	chr	gene_bp	topSNP	topSNP_bp	A1	A2	Freq	b_SMR	se_SMR	p_SMR	p_HEIDI
CD	DNMT3A	2	25510652	rs7578575	25488819	A	T	0.329	-0.834	0.164	3.51E-07	0.383
IBD	DNMT3A	2	25510652	rs7578575	25488819	A	T	0.329	-0.745	0.130	9.22E-09	0.515
UC	DNMT3A	2	25510652	rs7578575	25488819	A	T	0.329	-0.627	0.156	5.73E-05	0.541
UC	CFDP1	16	75327596	rs9934007	75390532	C	T	0.754	0.273	0.070	9.61E-05	0.973
CD	STAT3	17	40502964	rs1053004	40466092	G	A	0.380	0.702	0.128	4.40E-08	0.056
CD	IMPDH2	3	49064299	rs9813813	49207093	A	T	0.110	-1.233	0.294	2.74E-05	0.213
IBD	IMPDH2	3	49064299	rs9813813	49207093	A	T	0.110	-1.328	0.258	2.57E-07	0.071
UC	IMPDH2	3	49064299	rs9813813	49207093	A	T	0.110	-1.445	0.307	2.53E-06	0.050
IBD	UBE2L3	22	21941029	rs2070512	21949411	C	A	0.194	0.163	0.021	1.06E-14	0.169
UC	UBE2L3	22	21941029	rs2070512	21949411	C	A	0.194	0.116	0.027	1.76E-05	0.698
CD	BCL2L15	1	1.14E+08	rs1217393	1.14E+08	A	G	0.271	-0.705	0.170	3.39E-05	0.184
CD	STMN3	20	62271061	rs909333	62270637	G	T	0.207	0.547	0.102	8.31E-08	0.200
IBD	STMN3	20	62271061	rs909333	62270637	G	T	0.207	0.555	0.087	2.01E-10	0.055
UC	STMN3	20	62271061	rs909333	62270637	G	T	0.207	0.498	0.098	4.04E-07	0.062
GORD	PBX2	6	32154964	rs693906	31835164	C	G	0.113	0.154	0.036	1.65E-05	0.058
VHD	AGER	6	32150423	rs693906	31835164	C	G	0.113	0.212	0.052	4.82E-05	0.092

B) Nominally significant effect on VHD of Bonferroni-significant gastrointestinal loci.

Disease	Gene	chr	Probe_bp	topSNP	topSNP_bp	A1	A2	Freq	b_SMR	se_SMR	p_SMR	p_HEIDI
VHD	CFDP1	16	75397489	rs7201989	75471499	G	A	0.246	-0.056	0.018	0.001	0.119
VHD	STAT3	17	40502964	rs1053004	40466092	G	A	0.380	0.093	0.047	0.048	0.948
VHD	IMPDH2	3	49064299	rs34037363	48821036	T	C	0.114	-0.209	0.105	0.047	0.089
VHD	UBE2L3	22	21941029	rs2070512	21949411	C	A	0.194	-0.029	0.009	0.002	0.623
VHD	STMN3	20	62271061	rs909333	62270637	G	T	0.207	-0.076	0.036	0.036	0.093

C) Effect of AGER gene on VHD and gastrointestinal disorders.

Disease	Gene	chr	Probe_bp	topSNP	topSNP_bp	A1	A2	Freq	b_SMR	se_SMR	p_SMR	p_HEIDI
CD	AGER	6	32150423	rs693906	31835164	C	G	0.113	-0.312	0.120	0.009	6.16E-08
GORD	AGER	6	32150423	rs693906	31835164	C	G	0.113	-0.209	0.049	2.02E-05	0.022
IBD	AGER	6	32148745	rs204995	32154285	G	A	0.217	-0.252	0.108	0.020	1.60E-04
IBS	AGER	6	32150423	rs693906	31835164	C	G	0.113	-0.168	0.052	0.001	0.045
UC	AGER	6	32148745	rs204995	32154285	G	A	0.217	-0.870	0.185	2.59E-06	0.002
VHD	AGER	6	32150423	rs693906	31835164	C	G	0.113	0.212	0.052	4.82E-05	0.092

Supplementary table 6. Results of the summary-based Mendelian randomization integrating whole-blood expression quantitative trait loci. For each disease-gene association, we report the strongest effect observed between GTEX and eQTLGen datasets.

A) Bonferroni-significant disease-gene association with HEIDI P > 0.05.

Disease	Gene	chr	gene_bp	topSNP	topSNP_bp	A1	A2	Freq	b_SMR	se_SMR	p_SMR	p_HEIDI
CD	DNMT3A	2	25510652	rs7578575	25488819	A	T	0.329	-0.834	0.164	3.51E-07	0.383
IBD	DNMT3A	2	25510652	rs7578575	25488819	A	T	0.329	-0.745	0.130	9.22E-09	0.515
UC	DNMT3A	2	25510652	rs7578575	25488819	A	T	0.329	-0.627	0.156	5.73E-05	0.541
UC	CFDP1	16	75327596	rs9934007	75390532	C	T	0.754	0.273	0.070	9.61E-05	0.973
CD	STAT3	17	40502964	rs1053004	40466092	G	A	0.380	0.702	0.128	4.40E-08	0.056
CD	IMPDH2	3	49064299	rs9813813	49207093	A	T	0.110	-1.233	0.294	2.74E-05	0.213
IBD	IMPDH2	3	49064299	rs9813813	49207093	A	T	0.110	-1.328	0.258	2.57E-07	0.071
UC	IMPDH2	3	49064299	rs9813813	49207093	A	T	0.110	-1.445	0.307	2.53E-06	0.050
IBD	UBE2L3	22	21941029	rs2070512	21949411	C	A	0.194	0.163	0.021	1.06E-14	0.169
UC	UBE2L3	22	21941029	rs2070512	21949411	C	A	0.194	0.116	0.027	1.76E-05	0.698
CD	BCL2L15	1	1.14E+08	rs1217393	1.14E+08	A	G	0.271	-0.705	0.170	3.39E-05	0.184
CD	STMN3	20	62271061	rs909333	62270637	G	T	0.207	0.547	0.102	8.31E-08	0.200
IBD	STMN3	20	62271061	rs909333	62270637	G	T	0.207	0.555	0.087	2.01E-10	0.055
UC	STMN3	20	62271061	rs909333	62270637	G	T	0.207	0.498	0.098	4.04E-07	0.062
GORD	PBX2	6	32154964	rs693906	31835164	C	G	0.113	0.154	0.036	1.65E-05	0.058
VHD	AGER	6	32150423	rs693906	31835164	C	G	0.113	0.212	0.052	4.82E-05	0.092

B) Nominally significant effect on VHD of Bonferroni-significant gastrointestinal loci.

Disease	Gene	chr	Probe_bp	topSNP	topSNP_bp	A1	A2	Freq	b_SMR	se_SMR	p_SMR	p_HEIDI
VHD	CFDP1	16	75397489	rs7201989	75471499	G	A	0.246	-0.056	0.018	0.001	0.119
VHD	STAT3	17	40502964	rs1053004	40466092	G	A	0.380	0.093	0.047	0.048	0.948
VHD	IMPDH2	3	49064299	rs34037363	48821036	T	C	0.114	-0.209	0.105	0.047	0.089
VHD	UBE2L3	22	21941029	rs2070512	21949411	C	A	0.194	-0.029	0.009	0.002	0.623
VHD	STMN3	20	62271061	rs909333	62270637	G	T	0.207	-0.076	0.036	0.036	0.093

C) Effect of AGER gene on VHD and gastrointestinal disorders.

Disease	Gene	chr	Probe_bp	topSNP	topSNP_bp	A1	A2	Freq	b_SMR	se_SMR	p_SMR	p_HEIDI
CD	AGER	6	32150423	rs693906	31835164	C	G	0.113	-0.312	0.120	0.009	6.16E-08
GORD	AGER	6	32150423	rs693906	31835164	C	G	0.113	-0.209	0.049	2.02E-05	0.022
IBD	AGER	6	32148745	rs204995	32154285	G	A	0.217	-0.252	0.108	0.020	1.60E-04
IBS	AGER	6	32150423	rs693906	31835164	C	G	0.113	-0.168	0.052	0.001	0.045
UC	AGER	6	32148745	rs204995	32154285	G	A	0.217	-0.870	0.185	2.59E-06	0.002
VHD	AGER	6	32150423	rs693906	31835164	C	G	0.113	0.212	0.052	4.82E-05	0.092

Supplementary table 6. Results of the summary-based Mendelian randomization integrating whole-blood expression quantitative trait loci. For each disease-gene association, we report the strongest effect observed between GTEX and eQTLGen datasets.

A) Bonferroni-significant disease-gene association with HEIDI P > 0.05.

Disease	Gene	chr	gene_bp	topSNP	topSNP_bp	A1	A2	Freq	b_SMR	se_SMR	p_SMR	p_HEIDI
CD	DNMT3A	2	25510652	rs7578575	25488819	A	T	0.329	-0.834	0.164	3.51E-07	0.383
IBD	DNMT3A	2	25510652	rs7578575	25488819	A	T	0.329	-0.745	0.130	9.22E-09	0.515
UC	DNMT3A	2	25510652	rs7578575	25488819	A	T	0.329	-0.627	0.156	5.73E-05	0.541
UC	CFDP1	16	75327596	rs9934007	75390532	C	T	0.754	0.273	0.070	9.61E-05	0.973
CD	STAT3	17	40502964	rs1053004	40466092	G	A	0.380	0.702	0.128	4.40E-08	0.056
CD	IMPDH2	3	49064299	rs9813813	49207093	A	T	0.110	-1.233	0.294	2.74E-05	0.213
IBD	IMPDH2	3	49064299	rs9813813	49207093	A	T	0.110	-1.328	0.258	2.57E-07	0.071
UC	IMPDH2	3	49064299	rs9813813	49207093	A	T	0.110	-1.445	0.307	2.53E-06	0.050
IBD	UBE2L3	22	21941029	rs2070512	21949411	C	A	0.194	0.163	0.021	1.06E-14	0.169
UC	UBE2L3	22	21941029	rs2070512	21949411	C	A	0.194	0.116	0.027	1.76E-05	0.698
CD	BCL2L15	1	1.14E+08	rs1217393	1.14E+08	A	G	0.271	-0.705	0.170	3.39E-05	0.184
CD	STMN3	20	62271061	rs909333	62270637	G	T	0.207	0.547	0.102	8.31E-08	0.200
IBD	STMN3	20	62271061	rs909333	62270637	G	T	0.207	0.555	0.087	2.01E-10	0.055
UC	STMN3	20	62271061	rs909333	62270637	G	T	0.207	0.498	0.098	4.04E-07	0.062
GORD	PBX2	6	32154964	rs693906	31835164	C	G	0.113	0.154	0.036	1.65E-05	0.058
VHD	AGER	6	32150423	rs693906	31835164	C	G	0.113	0.212	0.052	4.82E-05	0.092

B) Nominally significant effect on VHD of Bonferroni-significant gastrointestinal loci.

Disease	Gene	chr	Probe_bp	topSNP	topSNP_bp	A1	A2	Freq	b_SMR	se_SMR	p_SMR	p_HEIDI
VHD	CFDP1	16	75397489	rs7201989	75471499	G	A	0.246	-0.056	0.018	0.001	0.119
VHD	STAT3	17	40502964	rs1053004	40466092	G	A	0.380	0.093	0.047	0.048	0.948
VHD	IMPDH2	3	49064299	rs34037363	48821036	T	C	0.114	-0.209	0.105	0.047	0.089
VHD	UBE2L3	22	21941029	rs2070512	21949411	C	A	0.194	-0.029	0.009	0.002	0.623
VHD	STMN3	20	62271061	rs909333	62270637	G	T	0.207	-0.076	0.036	0.036	0.093

C) Effect of AGER gene on VHD and gastrointestinal disorders.

Disease	Gene	chr	Probe_bp	topSNP	topSNP_bp	A1	A2	Freq	b_SMR	se_SMR	p_SMR	p_HEIDI
CD	AGER	6	32150423	rs693906	31835164	C	G	0.113	-0.312	0.120	0.009	6.16E-08
GORD	AGER	6	32150423	rs693906	31835164	C	G	0.113	-0.209	0.049	2.02E-05	0.022
IBD	AGER	6	32148745	rs204995	32154285	G	A	0.217	-0.252	0.108	0.020	1.60E-04
IBS	AGER	6	32150423	rs693906	31835164	C	G	0.113	-0.168	0.052	0.001	0.045
UC	AGER	6	32148745	rs204995	32154285	G	A	0.217	-0.870	0.185	2.59E-06	0.002
VHD	AGER	6	32150423	rs693906	31835164	C	G	0.113	0.212	0.052	4.82E-05	0.092

Supplementary table 6. Results of the summary-based Mendelian randomization integrating whole-blood expression quantitative trait loci. For each disease-gene association, we report the strongest effect observed between GTEX and eQTLGen datasets.

A) Bonferroni-significant disease-gene association with HEIDI P > 0.05.

Disease	Gene	chr	gene_bp	topSNP	topSNP_bp	A1	A2	Freq	b_SMR	se_SMR	p_SMR	p_HEIDI
CD	DNMT3A	2	25510652	rs7578575	25488819	A	T	0.329	-0.834	0.164	3.51E-07	0.383
IBD	DNMT3A	2	25510652	rs7578575	25488819	A	T	0.329	-0.745	0.130	9.22E-09	0.515
UC	DNMT3A	2	25510652	rs7578575	25488819	A	T	0.329	-0.627	0.156	5.73E-05	0.541
UC	CFDP1	16	75327596	rs9934007	75390532	C	T	0.754	0.273	0.070	9.61E-05	0.973
CD	STAT3	17	40502964	rs1053004	40466092	G	A	0.380	0.702	0.128	4.40E-08	0.056
CD	IMPDH2	3	49064299	rs9813813	49207093	A	T	0.110	-1.233	0.294	2.74E-05	0.213
IBD	IMPDH2	3	49064299	rs9813813	49207093	A	T	0.110	-1.328	0.258	2.57E-07	0.071
UC	IMPDH2	3	49064299	rs9813813	49207093	A	T	0.110	-1.445	0.307	2.53E-06	0.050
IBD	UBE2L3	22	21941029	rs2070512	21949411	C	A	0.194	0.163	0.021	1.06E-14	0.169
UC	UBE2L3	22	21941029	rs2070512	21949411	C	A	0.194	0.116	0.027	1.76E-05	0.698
CD	BCL2L15	1	1.14E+08	rs1217393	1.14E+08	A	G	0.271	-0.705	0.170	3.39E-05	0.184
CD	STMN3	20	62271061	rs909333	62270637	G	T	0.207	0.547	0.102	8.31E-08	0.200
IBD	STMN3	20	62271061	rs909333	62270637	G	T	0.207	0.555	0.087	2.01E-10	0.055
UC	STMN3	20	62271061	rs909333	62270637	G	T	0.207	0.498	0.098	4.04E-07	0.062
GORD	PBX2	6	32154964	rs693906	31835164	C	G	0.113	0.154	0.036	1.65E-05	0.058
VHD	AGER	6	32150423	rs693906	31835164	C	G	0.113	0.212	0.052	4.82E-05	0.092

B) Nominally significant effect on VHD of Bonferroni-significant gastrointestinal loci.

Disease	Gene	chr	Probe_bp	topSNP	topSNP_bp	A1	A2	Freq	b_SMR	se_SMR	p_SMR	p_HEIDI
VHD	CFDP1	16	75397489	rs7201989	75471499	G	A	0.246	-0.056	0.018	0.001	0.119
VHD	STAT3	17	40502964	rs1053004	40466092	G	A	0.380	0.093	0.047	0.048	0.948
VHD	IMPDH2	3	49064299	rs34037363	48821036	T	C	0.114	-0.209	0.105	0.047	0.089
VHD	UBE2L3	22	21941029	rs2070512	21949411	C	A	0.194	-0.029	0.009	0.002	0.623
VHD	STMN3	20	62271061	rs909333	62270637	G	T	0.207	-0.076	0.036	0.036	0.093

C) Effect of AGER gene on VHD and gastrointestinal disorders.

Disease	Gene	chr	Probe_bp	topSNP	topSNP_bp	A1	A2	Freq	b_SMR	se_SMR	p_SMR	p_HEIDI
CD	AGER	6	32150423	rs693906	31835164	C	G	0.113	-0.312	0.120	0.009	6.16E-08
GORD	AGER	6	32150423	rs693906	31835164	C	G	0.113	-0.209	0.049	2.02E-05	0.022
IBD	AGER	6	32148745	rs204995	32154285	G	A	0.217	-0.252	0.108	0.020	1.60E-04
IBS	AGER	6	32150423	rs693906	31835164	C	G	0.113	-0.168	0.052	0.001	0.045
UC	AGER	6	32148745	rs204995	32154285	G	A	0.217	-0.870	0.185	2.59E-06	0.002
VHD	AGER	6	32150423	rs693906	31835164	C	G	0.113	0.212	0.052	4.82E-05	0.092

Supplementary table 6. Results of the summary-based Mendelian randomization integrating whole-blood expression quantitative trait loci. For each disease-gene association, we report the strongest effect observed between GTEX and eQTLGen datasets.

A) Bonferroni-significant disease-gene association with HEIDI P > 0.05.

Disease	Gene	chr	gene_bp	topSNP	topSNP_bp	A1	A2	Freq	b_SMR	se_SMR	p_SMR	p_HEIDI
CD	DNMT3A	2	25510652	rs7578575	25488819	A	T	0.329	-0.834	0.164	3.51E-07	0.383
IBD	DNMT3A	2	25510652	rs7578575	25488819	A	T	0.329	-0.745	0.130	9.22E-09	0.515
UC	DNMT3A	2	25510652	rs7578575	25488819	A	T	0.329	-0.627	0.156	5.73E-05	0.541
UC	CFDP1	16	75327596	rs9934007	75390532	C	T	0.754	0.273	0.070	9.61E-05	0.973
CD	STAT3	17	40502964	rs1053004	40466092	G	A	0.380	0.702	0.128	4.40E-08	0.056
CD	IMPDH2	3	49064299	rs9813813	49207093	A	T	0.110	-1.233	0.294	2.74E-05	0.213
IBD	IMPDH2	3	49064299	rs9813813	49207093	A	T	0.110	-1.328	0.258	2.57E-07	0.071
UC	IMPDH2	3	49064299	rs9813813	49207093	A	T	0.110	-1.445	0.307	2.53E-06	0.050
IBD	UBE2L3	22	21941029	rs2070512	21949411	C	A	0.194	0.163	0.021	1.06E-14	0.169
UC	UBE2L3	22	21941029	rs2070512	21949411	C	A	0.194	0.116	0.027	1.76E-05	0.698
CD	BCL2L15	1	1.14E+08	rs1217393	1.14E+08	A	G	0.271	-0.705	0.170	3.39E-05	0.184
CD	STMN3	20	62271061	rs909333	62270637	G	T	0.207	0.547	0.102	8.31E-08	0.200
IBD	STMN3	20	62271061	rs909333	62270637	G	T	0.207	0.555	0.087	2.01E-10	0.055
UC	STMN3	20	62271061	rs909333	62270637	G	T	0.207	0.498	0.098	4.04E-07	0.062
GORD	PBX2	6	32154964	rs693906	31835164	C	G	0.113	0.154	0.036	1.65E-05	0.058
VHD	AGER	6	32150423	rs693906	31835164	C	G	0.113	0.212	0.052	4.82E-05	0.092

B) Nominally significant effect on VHD of Bonferroni-significant gastrointestinal loci.

Disease	Gene	chr	Probe_bp	topSNP	topSNP_bp	A1	A2	Freq	b_SMR	se_SMR	p_SMR	p_HEIDI
VHD	CFDP1	16	75397489	rs7201989	75471499	G	A	0.246	-0.056	0.018	0.001	0.119
VHD	STAT3	17	40502964	rs1053004	40466092	G	A	0.380	0.093	0.047	0.048	0.948
VHD	IMPDH2	3	49064299	rs34037363	48821036	T	C	0.114	-0.209	0.105	0.047	0.089
VHD	UBE2L3	22	21941029	rs2070512	21949411	C	A	0.194	-0.029	0.009	0.002	0.623
VHD	STMN3	20	62271061	rs909333	62270637	G	T	0.207	-0.076	0.036	0.036	0.093

C) Effect of AGER gene on VHD and gastrointestinal disorders.

Disease	Gene	chr	Probe_bp	topSNP	topSNP_bp	A1	A2	Freq	b_SMR	se_SMR	p_SMR	p_HEIDI
CD	AGER	6	32150423	rs693906	31835164	C	G	0.113	-0.312	0.120	0.009	6.16E-08
GORD	AGER	6	32150423	rs693906	31835164	C	G	0.113	-0.209	0.049	2.02E-05	0.022
IBD	AGER	6	32148745	rs204995	32154285	G	A	0.217	-0.252	0.108	0.020	1.60E-04
IBS	AGER	6	32150423	rs693906	31835164	C	G	0.113	-0.168	0.052	0.001	0.045
UC	AGER	6	32148745	rs204995	32154285	G	A	0.217	-0.870	0.185	2.59E-06	0.002
VHD	AGER	6	32150423	rs693906	31835164	C	G	0.113	0.212	0.052	4.82E-05	0.092

Supplementary table 6. Results of the summary-based Mendelian randomization integrating whole-blood expression quantitative trait loci. For each disease-gene association, we report the strongest effect observed between GTEX and eQTLGen datasets.

A) Bonferroni-significant disease-gene association with HEIDI P > 0.05.

Disease	Gene	chr	gene_bp	topSNP	topSNP_bp	A1	A2	Freq	b_SMR	se_SMR	p_SMR	p_HEIDI
CD	DNMT3A	2	25510652	rs7578575	25488819	A	T	0.329	-0.834	0.164	3.51E-07	0.383
IBD	DNMT3A	2	25510652	rs7578575	25488819	A	T	0.329	-0.745	0.130	9.22E-09	0.515
UC	DNMT3A	2	25510652	rs7578575	25488819	A	T	0.329	-0.627	0.156	5.73E-05	0.541
UC	CFDP1	16	75327596	rs9934007	75390532	C	T	0.754	0.273	0.070	9.61E-05	0.973
CD	STAT3	17	40502964	rs1053004	40466092	G	A	0.380	0.702	0.128	4.40E-08	0.056
CD	IMPDH2	3	49064299	rs9813813	49207093	A	T	0.110	-1.233	0.294	2.74E-05	0.213
IBD	IMPDH2	3	49064299	rs9813813	49207093	A	T	0.110	-1.328	0.258	2.57E-07	0.071
UC	IMPDH2	3	49064299	rs9813813	49207093	A	T	0.110	-1.445	0.307	2.53E-06	0.050
IBD	UBE2L3	22	21941029	rs2070512	21949411	C	A	0.194	0.163	0.021	1.06E-14	0.169
UC	UBE2L3	22	21941029	rs2070512	21949411	C	A	0.194	0.116	0.027	1.76E-05	0.698
CD	BCL2L15	1	1.14E+08	rs1217393	1.14E+08	A	G	0.271	-0.705	0.170	3.39E-05	0.184
CD	STMN3	20	62271061	rs909333	62270637	G	T	0.207	0.547	0.102	8.31E-08	0.200
IBD	STMN3	20	62271061	rs909333	62270637	G	T	0.207	0.555	0.087	2.01E-10	0.055
UC	STMN3	20	62271061	rs909333	62270637	G	T	0.207	0.498	0.098	4.04E-07	0.062
GORD	PBX2	6	32154964	rs693906	31835164	C	G	0.113	0.154	0.036	1.65E-05	0.058
VHD	AGER	6	32150423	rs693906	31835164	C	G	0.113	0.212	0.052	4.82E-05	0.092

B) Nominally significant effect on VHD of Bonferroni-significant gastrointestinal loci.

Disease	Gene	chr	Probe_bp	topSNP	topSNP_bp	A1	A2	Freq	b_SMR	se_SMR	p_SMR	p_HEIDI
VHD	CFDP1	16	75397489	rs7201989	75471499	G	A	0.246	-0.056	0.018	0.001	0.119
VHD	STAT3	17	40502964	rs1053004	40466092	G	A	0.380	0.093	0.047	0.048	0.948
VHD	IMPDH2	3	49064299	rs34037363	48821036	T	C	0.114	-0.209	0.105	0.047	0.089
VHD	UBE2L3	22	21941029	rs2070512	21949411	C	A	0.194	-0.029	0.009	0.002	0.623
VHD	STMN3	20	62271061	rs909333	62270637	G	T	0.207	-0.076	0.036	0.036	0.093

C) Effect of AGER gene on VHD and gastrointestinal disorders.

Disease	Gene	chr	Probe_bp	topSNP	topSNP_bp	A1	A2	Freq	b_SMR	se_SMR	p_SMR	p_HEIDI
CD	AGER	6	32150423	rs693906	31835164	C	G	0.113	-0.312	0.120	0.009	6.16E-08
GORD	AGER	6	32150423	rs693906	31835164	C	G	0.113	-0.209	0.049	2.02E-05	0.022
IBD	AGER	6	32148745	rs204995	32154285	G	A	0.217	-0.252	0.108	0.020	1.60E-04
IBS	AGER	6	32150423	rs693906	31835164	C	G	0.113	-0.168	0.052	0.001	0.045
UC	AGER	6	32148745	rs204995	32154285	G	A	0.217	-0.870	0.185	2.59E-06	0.002
VHD	AGER	6	32150423	rs693906	31835164	C	G	0.113	0.212	0.052	4.82E-05	0.092

Supplementary table 6. Results of the summary-based Mendelian randomization integrating whole-blood expression quantitative trait loci. For each disease-gene association, we report the strongest effect observed between GTEX and eQTLGen datasets.

A) Bonferroni-significant disease-gene association with HEIDI P > 0.05.

Disease	Gene	chr	gene_bp	topSNP	topSNP_bp	A1	A2	Freq	b_SMR	se_SMR	p_SMR	p_HEIDI
CD	DNMT3A	2	25510652	rs7578575	25488819	A	T	0.329	-0.834	0.164	3.51E-07	0.383
IBD	DNMT3A	2	25510652	rs7578575	25488819	A	T	0.329	-0.745	0.130	9.22E-09	0.515
UC	DNMT3A	2	25510652	rs7578575	25488819	A	T	0.329	-0.627	0.156	5.73E-05	0.541
UC	CFDP1	16	75327596	rs9934007	75390532	C	T	0.754	0.273	0.070	9.61E-05	0.973
CD	STAT3	17	40502964	rs1053004	40466092	G	A	0.380	0.702	0.128	4.40E-08	0.056
CD	IMPDH2	3	49064299	rs9813813	49207093	A	T	0.110	-1.233	0.294	2.74E-05	0.213
IBD	IMPDH2	3	49064299	rs9813813	49207093	A	T	0.110	-1.328	0.258	2.57E-07	0.071
UC	IMPDH2	3	49064299	rs9813813	49207093	A	T	0.110	-1.445	0.307	2.53E-06	0.050
IBD	UBE2L3	22	21941029	rs2070512	21949411	C	A	0.194	0.163	0.021	1.06E-14	0.169
UC	UBE2L3	22	21941029	rs2070512	21949411	C	A	0.194	0.116	0.027	1.76E-05	0.698
CD	BCL2L15	1	1.14E+08	rs1217393	1.14E+08	A	G	0.271	-0.705	0.170	3.39E-05	0.184
CD	STMN3	20	62271061	rs909333	62270637	G	T	0.207	0.547	0.102	8.31E-08	0.200
IBD	STMN3	20	62271061	rs909333	62270637	G	T	0.207	0.555	0.087	2.01E-10	0.055
UC	STMN3	20	62271061	rs909333	62270637	G	T	0.207	0.498	0.098	4.04E-07	0.062
GORD	PBX2	6	32154964	rs693906	31835164	C	G	0.113	0.154	0.036	1.65E-05	0.058
VHD	AGER	6	32150423	rs693906	31835164	C	G	0.113	0.212	0.052	4.82E-05	0.092

B) Nominally significant effect on VHD of Bonferroni-significant gastrointestinal loci.

Disease	Gene	chr	Probe_bp	topSNP	topSNP_bp	A1	A2	Freq	b_SMR	se_SMR	p_SMR	p_HEIDI
VHD	CFDP1	16	75397489	rs7201989	75471499	G	A	0.246	-0.056	0.018	0.001	0.119
VHD	STAT3	17	40502964	rs1053004	40466092	G	A	0.380	0.093	0.047	0.048	0.948
VHD	IMPDH2	3	49064299	rs34037363	48821036	T	C	0.114	-0.209	0.105	0.047	0.089
VHD	UBE2L3	22	21941029	rs2070512	21949411	C	A	0.194	-0.029	0.009	0.002	0.623
VHD	STMN3	20	62271061	rs909333	62270637	G	T	0.207	-0.076	0.036	0.036	0.093

C) Effect of AGER gene on VHD and gastrointestinal disorders.

Disease	Gene	chr	Probe_bp	topSNP	topSNP_bp	A1	A2	Freq	b_SMR	se_SMR	p_SMR	p_HEIDI
CD	AGER	6	32150423	rs693906	31835164	C	G	0.113	-0.312	0.120	0.009	6.16E-08
GORD	AGER	6	32150423	rs693906	31835164	C	G	0.113	-0.209	0.049	2.02E-05	0.022
IBD	AGER	6	32148745	rs204995	32154285	G	A	0.217	-0.252	0.108	0.020	1.60E-04
IBS	AGER	6	32150423	rs693906	31835164	C	G	0.113	-0.168	0.052	0.001	0.045
UC	AGER	6	32148745	rs204995	32154285	G	A	0.217	-0.870	0.185	2.59E-06	0.002
VHD	AGER	6	32150423	rs693906	31835164	C	G	0.113	0.212	0.052	4.82E-05	0.092

Supplementary table 6. Results of the summary-based Mendelian randomization integrating whole-blood expression quantitative trait loci. For each disease-gene association, we report the strongest effect observed between GTEX and eQTLGen datasets.

A) Bonferroni-significant disease-gene association with HEIDI P > 0.05.

Disease	Gene	chr	gene_bp	topSNP	topSNP_bp	A1	A2	Freq	b_SMR	se_SMR	p_SMR	p_HEIDI
CD	DNMT3A	2	25510652	rs7578575	25488819	A	T	0.329	-0.834	0.164	3.51E-07	0.383
IBD	DNMT3A	2	25510652	rs7578575	25488819	A	T	0.329	-0.745	0.130	9.22E-09	0.515
UC	DNMT3A	2	25510652	rs7578575	25488819	A	T	0.329	-0.627	0.156	5.73E-05	0.541
UC	CFDP1	16	75327596	rs9934007	75390532	C	T	0.754	0.273	0.070	9.61E-05	0.973
CD	STAT3	17	40502964	rs1053004	40466092	G	A	0.380	0.702	0.128	4.40E-08	0.056
CD	IMPDH2	3	49064299	rs9813813	49207093	A	T	0.110	-1.233	0.294	2.74E-05	0.213
IBD	IMPDH2	3	49064299	rs9813813	49207093	A	T	0.110	-1.328	0.258	2.57E-07	0.071
UC	IMPDH2	3	49064299	rs9813813	49207093	A	T	0.110	-1.445	0.307	2.53E-06	0.050
IBD	UBE2L3	22	21941029	rs2070512	21949411	C	A	0.194	0.163	0.021	1.06E-14	0.169
UC	UBE2L3	22	21941029	rs2070512	21949411	C	A	0.194	0.116	0.027	1.76E-05	0.698
CD	BCL2L15	1	1.14E+08	rs1217393	1.14E+08	A	G	0.271	-0.705	0.170	3.39E-05	0.184
CD	STMN3	20	62271061	rs909333	62270637	G	T	0.207	0.547	0.102	8.31E-08	0.200
IBD	STMN3	20	62271061	rs909333	62270637	G	T	0.207	0.555	0.087	2.01E-10	0.055
UC	STMN3	20	62271061	rs909333	62270637	G	T	0.207	0.498	0.098	4.04E-07	0.062
GORD	PBX2	6	32154964	rs693906	31835164	C	G	0.113	0.154	0.036	1.65E-05	0.058
VHD	AGER	6	32150423	rs693906	31835164	C	G	0.113	0.212	0.052	4.82E-05	0.092

B) Nominally significant effect on VHD of Bonferroni-significant gastrointestinal loci.

Disease	Gene	chr	Probe_bp	topSNP	topSNP_bp	A1	A2	Freq	b_SMR	se_SMR	p_SMR	p_HEIDI
VHD	CFDP1	16	75397489	rs7201989	75471499	G	A	0.246	-0.056	0.018	0.001	0.119
VHD	STAT3	17	40502964	rs1053004	40466092	G	A	0.380	0.093	0.047	0.048	0.948
VHD	IMPDH2	3	49064299	rs34037363	48821036	T	C	0.114	-0.209	0.105	0.047	0.089
VHD	UBE2L3	22	21941029	rs2070512	21949411	C	A	0.194	-0.029	0.009	0.002	0.623
VHD	STMN3	20	62271061	rs909333	62270637	G	T	0.207	-0.076	0.036	0.036	0.093

C) Effect of AGER gene on VHD and gastrointestinal disorders.

Disease	Gene	chr	Probe_bp	topSNP	topSNP_bp	A1	A2	Freq	b_SMR	se_SMR	p_SMR	p_HEIDI
CD	AGER	6	32150423	rs693906	31835164	C	G	0.113	-0.312	0.120	0.009	6.16E-08
GORD	AGER	6	32150423	rs693906	31835164	C	G	0.113	-0.209	0.049	2.02E-05	0.022
IBD	AGER	6	32148745	rs204995	32154285	G	A	0.217	-0.252	0.108	0.020	1.60E-04
IBS	AGER	6	32150423	rs693906	31835164	C	G	0.113	-0.168	0.052	0.001	0.045
UC	AGER	6	32148745	rs204995	32154285	G	A	0.217	-0.870	0.185	2.59E-06	0.002
VHD	AGER	6	32150423	rs693906	31835164	C	G	0.113	0.212	0.052	4.82E-05	0.092

Supplementary table 6. Results of the summary-based Mendelian randomization integrating whole-blood expression quantitative trait loci. For each disease-gene association, we report the strongest effect observed between GTEX and eQTLGen datasets.

A) Bonferroni-significant disease-gene association with HEIDI P > 0.05.

Disease	Gene	chr	gene_bp	topSNP	topSNP_bp	A1	A2	Freq	b_SMR	se_SMR	p_SMR	p_HEIDI
CD	DNMT3A	2	25510652	rs7578575	25488819	A	T	0.329	-0.834	0.164	3.51E-07	0.383
IBD	DNMT3A	2	25510652	rs7578575	25488819	A	T	0.329	-0.745	0.130	9.22E-09	0.515
UC	DNMT3A	2	25510652	rs7578575	25488819	A	T	0.329	-0.627	0.156	5.73E-05	0.541
UC	CFDP1	16	75327596	rs9934007	75390532	C	T	0.754	0.273	0.070	9.61E-05	0.973
CD	STAT3	17	40502964	rs1053004	40466092	G	A	0.380	0.702	0.128	4.40E-08	0.056
CD	IMPDH2	3	49064299	rs9813813	49207093	A	T	0.110	-1.233	0.294	2.74E-05	0.213
IBD	IMPDH2	3	49064299	rs9813813	49207093	A	T	0.110	-1.328	0.258	2.57E-07	0.071
UC	IMPDH2	3	49064299	rs9813813	49207093	A	T	0.110	-1.445	0.307	2.53E-06	0.050
IBD	UBE2L3	22	21941029	rs2070512	21949411	C	A	0.194	0.163	0.021	1.06E-14	0.169
UC	UBE2L3	22	21941029	rs2070512	21949411	C	A	0.194	0.116	0.027	1.76E-05	0.698
CD	BCL2L15	1	1.14E+08	rs1217393	1.14E+08	A	G	0.271	-0.705	0.170	3.39E-05	0.184
CD	STMN3	20	62271061	rs909333	62270637	G	T	0.207	0.547	0.102	8.31E-08	0.200
IBD	STMN3	20	62271061	rs909333	62270637	G	T	0.207	0.555	0.087	2.01E-10	0.055
UC	STMN3	20	62271061	rs909333	62270637	G	T	0.207	0.498	0.098	4.04E-07	0.062
GORD	PBX2	6	32154964	rs693906	31835164	C	G	0.113	0.154	0.036	1.65E-05	0.058
VHD	AGER	6	32150423	rs693906	31835164	C	G	0.113	0.212	0.052	4.82E-05	0.092

B) Nominally significant effect on VHD of Bonferroni-significant gastrointestinal loci.

Disease	Gene	chr	Probe_bp	topSNP	topSNP_bp	A1	A2	Freq	b_SMR	se_SMR	p_SMR	p_HEIDI
VHD	CFDP1	16	75397489	rs7201989	75471499	G	A	0.246	-0.056	0.018	0.001	0.119
VHD	STAT3	17	40502964	rs1053004	40466092	G	A	0.380	0.093	0.047	0.048	0.948
VHD	IMPDH2	3	49064299	rs34037363	48821036	T	C	0.114	-0.209	0.105	0.047	0.089
VHD	UBE2L3	22	21941029	rs2070512	21949411	C	A	0.194	-0.029	0.009	0.002	0.623
VHD	STMN3	20	62271061	rs909333	62270637	G	T	0.207	-0.076	0.036	0.036	0.093

C) Effect of AGER gene on VHD and gastrointestinal disorders.

Disease	Gene	chr	Probe_bp	topSNP	topSNP_bp	A1	A2	Freq	b_SMR	se_SMR	p_SMR	p_HEIDI
CD	AGER	6	32150423	rs693906	31835164	C	G	0.113	-0.312	0.120	0.009	6.16E-08
GORD	AGER	6	32150423	rs693906	31835164	C	G	0.113	-0.209	0.049	2.02E-05	0.022
IBD	AGER	6	32148745	rs204995	32154285	G	A	0.217	-0.252	0.108	0.020	1.60E-04
IBS	AGER	6	32150423	rs693906	31835164	C	G	0.113	-0.168	0.052	0.001	0.045
UC	AGER	6	32148745	rs204995	32154285	G	A	0.217	-0.870	0.185	2.59E-06	0.002
VHD	AGER	6	32150423	rs693906	31835164	C	G	0.113	0.212	0.052	4.82E-05	0.092

Supplementary table 6. Results of the summary-based Mendelian randomization integrating whole-blood expression quantitative trait loci. For each disease-gene association, we report the strongest effect observed between GTEX and eQTLGen datasets.

A) Bonferroni-significant disease-gene association with HEIDI P > 0.05.

Disease	Gene	chr	gene_bp	topSNP	topSNP_bp	A1	A2	Freq	b_SMR	se_SMR	p_SMR	p_HEIDI
CD	DNMT3A	2	25510652	rs7578575	25488819	A	T	0.329	-0.834	0.164	3.51E-07	0.383
IBD	DNMT3A	2	25510652	rs7578575	25488819	A	T	0.329	-0.745	0.130	9.22E-09	0.515
UC	DNMT3A	2	25510652	rs7578575	25488819	A	T	0.329	-0.627	0.156	5.73E-05	0.541
UC	CFDP1	16	75327596	rs9934007	75390532	C	T	0.754	0.273	0.070	9.61E-05	0.973
CD	STAT3	17	40502964	rs1053004	40466092	G	A	0.380	0.702	0.128	4.40E-08	0.056
CD	IMPDH2	3	49064299	rs9813813	49207093	A	T	0.110	-1.233	0.294	2.74E-05	0.213
IBD	IMPDH2	3	49064299	rs9813813	49207093	A	T	0.110	-1.328	0.258	2.57E-07	0.071
UC	IMPDH2	3	49064299	rs9813813	49207093	A	T	0.110	-1.445	0.307	2.53E-06	0.050
IBD	UBE2L3	22	21941029	rs2070512	21949411	C	A	0.194	0.163	0.021	1.06E-14	0.169
UC	UBE2L3	22	21941029	rs2070512	21949411	C	A	0.194	0.116	0.027	1.76E-05	0.698
CD	BCL2L15	1	1.14E+08	rs1217393	1.14E+08	A	G	0.271	-0.705	0.170	3.39E-05	0.184
CD	STMN3	20	62271061	rs909333	62270637	G	T	0.207	0.547	0.102	8.31E-08	0.200
IBD	STMN3	20	62271061	rs909333	62270637	G	T	0.207	0.555	0.087	2.01E-10	0.055
UC	STMN3	20	62271061	rs909333	62270637	G	T	0.207	0.498	0.098	4.04E-07	0.062
GORD	PBX2	6	32154964	rs693906	31835164	C	G	0.113	0.154	0.036	1.65E-05	0.058
VHD	AGER	6	32150423	rs693906	31835164	C	G	0.113	0.212	0.052	4.82E-05	0.092

B) Nominally significant effect on VHD of Bonferroni-significant gastrointestinal loci.

Disease	Gene	chr	Probe_bp	topSNP	topSNP_bp	A1	A2	Freq	b_SMR	se_SMR	p_SMR	p_HEIDI
VHD	CFDP1	16	75397489	rs7201989	75471499	G	A	0.246	-0.056	0.018	0.001	0.119
VHD	STAT3	17	40502964	rs1053004	40466092	G	A	0.380	0.093	0.047	0.048	0.948
VHD	IMPDH2	3	49064299	rs34037363	48821036	T	C	0.114	-0.209	0.105	0.047	0.089
VHD	UBE2L3	22	21941029	rs2070512	21949411	C	A	0.194	-0.029	0.009	0.002	0.623
VHD	STMN3	20	62271061	rs909333	62270637	G	T	0.207	-0.076	0.036	0.036	0.093

C) Effect of AGER gene on VHD and gastrointestinal disorders.

Disease	Gene	chr	Probe_bp	topSNP	topSNP_bp	A1	A2	Freq	b_SMR	se_SMR	p_SMR	p_HEIDI
CD	AGER	6	32150423	rs693906	31835164	C	G	0.113	-0.312	0.120	0.009	6.16E-08
GORD	AGER	6	32150423	rs693906	31835164	C	G	0.113	-0.209	0.049	2.02E-05	0.022
IBD	AGER	6	32148745	rs204995	32154285	G	A	0.217	-0.252	0.108	0.020	1.60E-04
IBS	AGER	6	32150423	rs693906	31835164	C	G	0.113	-0.168	0.052	0.001	0.045
UC	AGER	6	32148745	rs204995	32154285	G	A	0.217	-0.870	0.185	2.59E-06	0.002
VHD	AGER	6	32150423	rs693906	31835164	C	G	0.113	0.212	0.052	4.82E-05	0.092

Supplementary table 6. Results of the summary-based Mendelian randomization integrating whole-blood expression quantitative trait loci. For each disease-gene association, we report the strongest effect observed between GTEX and eQTLGen datasets.

A) Bonferroni-significant disease-gene association with HEIDI P > 0.05.

Disease	Gene	chr	gene_bp	topSNP	topSNP_bp	A1	A2	Freq	b_SMR	se_SMR	p_SMR	p_HEIDI
CD	DNMT3A	2	25510652	rs7578575	25488819	A	T	0.329	-0.834	0.164	3.51E-07	0.383
IBD	DNMT3A	2	25510652	rs7578575	25488819	A	T	0.329	-0.745	0.130	9.22E-09	0.515
UC	DNMT3A	2	25510652	rs7578575	25488819	A	T	0.329	-0.627	0.156	5.73E-05	0.541
UC	CFDP1	16	75327596	rs9934007	75390532	C	T	0.754	0.273	0.070	9.61E-05	0.973
CD	STAT3	17	40502964	rs1053004	40466092	G	A	0.380	0.702	0.128	4.40E-08	0.056
CD	IMPDH2	3	49064299	rs9813813	49207093	A	T	0.110	-1.233	0.294	2.74E-05	0.213
IBD	IMPDH2	3	49064299	rs9813813	49207093	A	T	0.110	-1.328	0.258	2.57E-07	0.071
UC	IMPDH2	3	49064299	rs9813813	49207093	A	T	0.110	-1.445	0.307	2.53E-06	0.050
IBD	UBE2L3	22	21941029	rs2070512	21949411	C	A	0.194	0.163	0.021	1.06E-14	0.169
UC	UBE2L3	22	21941029	rs2070512	21949411	C	A	0.194	0.116	0.027	1.76E-05	0.698
CD	BCL2L15	1	1.14E+08	rs1217393	1.14E+08	A	G	0.271	-0.705	0.170	3.39E-05	0.184
CD	STMN3	20	62271061	rs909333	62270637	G	T	0.207	0.547	0.102	8.31E-08	0.200
IBD	STMN3	20	62271061	rs909333	62270637	G	T	0.207	0.555	0.087	2.01E-10	0.055
UC	STMN3	20	62271061	rs909333	62270637	G	T	0.207	0.498	0.098	4.04E-07	0.062
GORD	PBX2	6	32154964	rs693906	31835164	C	G	0.113	0.154	0.036	1.65E-05	0.058
VHD	AGER	6	32150423	rs693906	31835164	C	G	0.113	0.212	0.052	4.82E-05	0.092

B) Nominally significant effect on VHD of Bonferroni-significant gastrointestinal loci.

Disease	Gene	chr	Probe_bp	topSNP	topSNP_bp	A1	A2	Freq	b_SMR	se_SMR	p_SMR	p_HEIDI
VHD	CFDP1	16	75397489	rs7201989	75471499	G	A	0.246	-0.056	0.018	0.001	0.119
VHD	STAT3	17	40502964	rs1053004	40466092	G	A	0.380	0.093	0.047	0.048	0.948
VHD	IMPDH2	3	49064299	rs34037363	48821036	T	C	0.114	-0.209	0.105	0.047	0.089
VHD	UBE2L3	22	21941029	rs2070512	21949411	C	A	0.194	-0.029	0.009	0.002	0.623
VHD	STMN3	20	62271061	rs909333	62270637	G	T	0.207	-0.076	0.036	0.036	0.093

C) Effect of AGER gene on VHD and gastrointestinal disorders.

Disease	Gene	chr	Probe_bp	topSNP	topSNP_bp	A1	A2	Freq	b_SMR	se_SMR	p_SMR	p_HEIDI
CD	AGER	6	32150423	rs693906	31835164	C	G	0.113	-0.312	0.120	0.009	6.16E-08
GORD	AGER	6	32150423	rs693906	31835164	C	G	0.113	-0.209	0.049	2.02E-05	0.022
IBD	AGER	6	32148745	rs204995	32154285	G	A	0.217	-0.252	0.108	0.020	1.60E-04
IBS	AGER	6	32150423	rs693906	31835164	C	G	0.113	-0.168	0.052	0.001	0.045
UC	AGER	6	32148745	rs204995	32154285	G	A	0.217	-0.870	0.185	2.59E-06	0.002
VHD	AGER	6	32150423	rs693906	31835164	C	G	0.113	0.212	0.052	4.82E-05	0.092

Supplementary table 6. Results of the summary-based Mendelian randomization integrating whole-blood expression quantitative trait loci. For each disease-gene association, we report the strongest effect observed between GTEX and eQTLGen datasets.

A) Bonferroni-significant disease-gene association with HEIDI P > 0.05.

Disease	Gene	chr	gene_bp	topSNP	topSNP_bp	A1	A2	Freq	b_SMR	se_SMR	p_SMR	p_HEIDI
CD	DNMT3A	2	25510652	rs7578575	25488819	A	T	0.329	-0.834	0.164	3.51E-07	0.383
IBD	DNMT3A	2	25510652	rs7578575	25488819	A	T	0.329	-0.745	0.130	9.22E-09	0.515
UC	DNMT3A	2	25510652	rs7578575	25488819	A	T	0.329	-0.627	0.156	5.73E-05	0.541
UC	CFDP1	16	75327596	rs9934007	75390532	C	T	0.754	0.273	0.070	9.61E-05	0.973
CD	STAT3	17	40502964	rs1053004	40466092	G	A	0.380	0.702	0.128	4.40E-08	0.056
CD	IMPDH2	3	49064299	rs9813813	49207093	A	T	0.110	-1.233	0.294	2.74E-05	0.213
IBD	IMPDH2	3	49064299	rs9813813	49207093	A	T	0.110	-1.328	0.258	2.57E-07	0.071
UC	IMPDH2	3	49064299	rs9813813	49207093	A	T	0.110	-1.445	0.307	2.53E-06	0.050
IBD	UBE2L3	22	21941029	rs2070512	21949411	C	A	0.194	0.163	0.021	1.06E-14	0.169
UC	UBE2L3	22	21941029	rs2070512	21949411	C	A	0.194	0.116	0.027	1.76E-05	0.698
CD	BCL2L15	1	1.14E+08	rs1217393	1.14E+08	A	G	0.271	-0.705	0.170	3.39E-05	0.184
CD	STMN3	20	62271061	rs909333	62270637	G	T	0.207	0.547	0.102	8.31E-08	0.200
IBD	STMN3	20	62271061	rs909333	62270637	G	T	0.207	0.555	0.087	2.01E-10	0.055
UC	STMN3	20	62271061	rs909333	62270637	G	T	0.207	0.498	0.098	4.04E-07	0.062
GORD	PBX2	6	32154964	rs693906	31835164	C	G	0.113	0.154	0.036	1.65E-05	0.058
VHD	AGER	6	32150423	rs693906	31835164	C	G	0.113	0.212	0.052	4.82E-05	0.092

B) Nominally significant effect on VHD of Bonferroni-significant gastrointestinal loci.

Disease	Gene	chr	Probe_bp	topSNP	topSNP_bp	A1	A2	Freq	b_SMR	se_SMR	p_SMR	p_HEIDI
VHD	CFDP1	16	75397489	rs7201989	75471499	G	A	0.246	-0.056	0.018	0.001	0.119
VHD	STAT3	17	40502964	rs1053004	40466092	G	A	0.380	0.093	0.047	0.048	0.948
VHD	IMPDH2	3	49064299	rs34037363	48821036	T	C	0.114	-0.209	0.105	0.047	0.089
VHD	UBE2L3	22	21941029	rs2070512	21949411	C	A	0.194	-0.029	0.009	0.002	0.623
VHD	STMN3	20	62271061	rs909333	62270637	G	T	0.207	-0.076	0.036	0.036	0.093

C) Effect of AGER gene on VHD and gastrointestinal disorders.

Disease	Gene	chr	Probe_bp	topSNP	topSNP_bp	A1	A2	Freq	b_SMR	se_SMR	p_SMR	p_HEIDI
CD	AGER	6	32150423	rs693906	31835164	C	G	0.113	-0.312	0.120	0.009	6.16E-08
GORD	AGER	6	32150423	rs693906	31835164	C	G	0.113	-0.209	0.049	2.02E-05	0.022
IBD	AGER	6	32148745	rs204995	32154285	G	A	0.217	-0.252	0.108	0.020	1.60E-04
IBS	AGER	6	32150423	rs693906	31835164	C	G	0.113	-0.168	0.052	0.001	0.045
UC	AGER	6	32148745	rs204995	32154285	G	A	0.217	-0.870	0.185	2.59E-06	0.002
VHD	AGER	6	32150423	rs693906	31835164	C	G	0.113	0.212	0.052	4.82E-05	0.092

Supplementary table 6. Results of the summary-based Mendelian randomization integrating whole-blood expression quantitative trait loci. For each disease-gene association, we report the strongest effect observed between GTEX and eQTLGen datasets.

A) Bonferroni-significant disease-gene association with HEIDI P > 0.05.

Disease	Gene	chr	gene_bp	topSNP	topSNP_bp	A1	A2	Freq	b_SMR	se_SMR	p_SMR	p_HEIDI
CD	DNMT3A	2	25510652	rs7578575	25488819	A	T	0.329	-0.834	0.164	3.51E-07	0.383
IBD	DNMT3A	2	25510652	rs7578575	25488819	A	T	0.329	-0.745	0.130	9.22E-09	0.515
UC	DNMT3A	2	25510652	rs7578575	25488819	A	T	0.329	-0.627	0.156	5.73E-05	0.541
UC	CFDP1	16	75327596	rs9934007	75390532	C	T	0.754	0.273	0.070	9.61E-05	0.973
CD	STAT3	17	40502964	rs1053004	40466092	G	A	0.380	0.702	0.128	4.40E-08	0.056
CD	IMPDH2	3	49064299	rs9813813	49207093	A	T	0.110	-1.233	0.294	2.74E-05	0.213
IBD	IMPDH2	3	49064299	rs9813813	49207093	A	T	0.110	-1.328	0.258	2.57E-07	0.071
UC	IMPDH2	3	49064299	rs9813813	49207093	A	T	0.110	-1.445	0.307	2.53E-06	0.050
IBD	UBE2L3	22	21941029	rs2070512	21949411	C	A	0.194	0.163	0.021	1.06E-14	0.169
UC	UBE2L3	22	21941029	rs2070512	21949411	C	A	0.194	0.116	0.027	1.76E-05	0.698
CD	BCL2L15	1	1.14E+08	rs1217393	1.14E+08	A	G	0.271	-0.705	0.170	3.39E-05	0.184
CD	STMN3	20	62271061	rs909333	62270637	G	T	0.207	0.547	0.102	8.31E-08	0.200
IBD	STMN3	20	62271061	rs909333	62270637	G	T	0.207	0.555	0.087	2.01E-10	0.055
UC	STMN3	20	62271061	rs909333	62270637	G	T	0.207	0.498	0.098	4.04E-07	0.062
GORD	PBX2	6	32154964	rs693906	31835164	C	G	0.113	0.154	0.036	1.65E-05	0.058
VHD	AGER	6	32150423	rs693906	31835164	C	G	0.113	0.212	0.052	4.82E-05	0.092

B) Nominally significant effect on VHD of Bonferroni-significant gastrointestinal loci.

Disease	Gene	chr	Probe_bp	topSNP	topSNP_bp	A1	A2	Freq	b_SMR	se_SMR	p_SMR	p_HEIDI
VHD	CFDP1	16	75397489	rs7201989	75471499	G	A	0.246	-0.056	0.018	0.001	0.119
VHD	STAT3	17	40502964	rs1053004	40466092	G	A	0.380	0.093	0.047	0.048	0.948
VHD	IMPDH2	3	49064299	rs34037363	48821036	T	C	0.114	-0.209	0.105	0.047	0.089
VHD	UBE2L3	22	21941029	rs2070512	21949411	C	A	0.194	-0.029	0.009	0.002	0.623
VHD	STMN3	20	62271061	rs909333	62270637	G	T	0.207	-0.076	0.036	0.036	0.093

C) Effect of AGER gene on VHD and gastrointestinal disorders.

Disease	Gene	chr	Probe_bp	topSNP	topSNP_bp	A1	A2	Freq	b_SMR	se_SMR	p_SMR	p_HEIDI
CD	AGER	6	32150423	rs693906	31835164	C	G	0.113	-0.312	0.120	0.009	6.16E-08
GORD	AGER	6	32150423	rs693906	31835164	C	G	0.113	-0.209	0.049	2.02E-05	0.022
IBD	AGER	6	32148745	rs204995	32154285	G	A	0.217	-0.252	0.108	0.020	1.60E-04
IBS	AGER	6	32150423	rs693906	31835164	C	G	0.113	-0.168	0.052	0.001	0.045
UC	AGER	6	32148745	rs204995	32154285	G	A	0.217	-0.870	0.185	2.59E-06	0.002
VHD	AGER	6	32150423	rs693906	31835164	C	G	0.113	0.212	0.052	4.82E-05	0.092

Supplementary table 6. Results of the summary-based Mendelian randomization integrating whole-blood expression quantitative trait loci. For each disease-gene association, we report the strongest effect observed between GTEX and eQTLGen datasets.

A) Bonferroni-significant disease-gene association with HEIDI P > 0.05.

Disease	Gene	chr	gene_bp	topSNP	topSNP_bp	A1	A2	Freq	b_SMR	se_SMR	p_SMR	p_HEIDI
CD	DNMT3A	2	25510652	rs7578575	25488819	A	T	0.329	-0.834	0.164	3.51E-07	0.383
IBD	DNMT3A	2	25510652	rs7578575	25488819	A	T	0.329	-0.745	0.130	9.22E-09	0.515
UC	DNMT3A	2	25510652	rs7578575	25488819	A	T	0.329	-0.627	0.156	5.73E-05	0.541
UC	CFDP1	16	75327596	rs9934007	75390532	C	T	0.754	0.273	0.070	9.61E-05	0.973
CD	STAT3	17	40502964	rs1053004	40466092	G	A	0.380	0.702	0.128	4.40E-08	0.056
CD	IMPDH2	3	49064299	rs9813813	49207093	A	T	0.110	-1.233	0.294	2.74E-05	0.213
IBD	IMPDH2	3	49064299	rs9813813	49207093	A	T	0.110	-1.328	0.258	2.57E-07	0.071
UC	IMPDH2	3	49064299	rs9813813	49207093	A	T	0.110	-1.445	0.307	2.53E-06	0.050
IBD	UBE2L3	22	21941029	rs2070512	21949411	C	A	0.194	0.163	0.021	1.06E-14	0.169
UC	UBE2L3	22	21941029	rs2070512	21949411	C	A	0.194	0.116	0.027	1.76E-05	0.698
CD	BCL2L15	1	1.14E+08	rs1217393	1.14E+08	A	G	0.271	-0.705	0.170	3.39E-05	0.184
CD	STMN3	20	62271061	rs909333	62270637	G	T	0.207	0.547	0.102	8.31E-08	0.200
IBD	STMN3	20	62271061	rs909333	62270637	G	T	0.207	0.555	0.087	2.01E-10	0.055
UC	STMN3	20	62271061	rs909333	62270637	G	T	0.207	0.498	0.098	4.04E-07	0.062
GORD	PBX2	6	32154964	rs693906	31835164	C	G	0.113	0.154	0.036	1.65E-05	0.058
VHD	AGER	6	32150423	rs693906	31835164	C	G	0.113	0.212	0.052	4.82E-05	0.092

B) Nominally significant effect on VHD of Bonferroni-significant gastrointestinal loci.

Disease	Gene	chr	Probe_bp	topSNP	topSNP_bp	A1	A2	Freq	b_SMR	se_SMR	p_SMR	p_HEIDI
VHD	CFDP1	16	75397489	rs7201989	75471499	G	A	0.246	-0.056	0.018	0.001	0.119
VHD	STAT3	17	40502964	rs1053004	40466092	G	A	0.380	0.093	0.047	0.048	0.948
VHD	IMPDH2	3	49064299	rs34037363	48821036	T	C	0.114	-0.209	0.105	0.047	0.089
VHD	UBE2L3	22	21941029	rs2070512	21949411	C	A	0.194	-0.029	0.009	0.002	0.623
VHD	STMN3	20	62271061	rs909333	62270637	G	T	0.207	-0.076	0.036	0.036	0.093

C) Effect of AGER gene on VHD and gastrointestinal disorders.

Disease	Gene	chr	Probe_bp	topSNP	topSNP_bp	A1	A2	Freq	b_SMR	se_SMR	p_SMR	p_HEIDI
CD	AGER	6	32150423	rs693906	31835164	C	G	0.113	-0.312	0.120	0.009	6.16E-08
GORD	AGER	6	32150423	rs693906	31835164	C	G	0.113	-0.209	0.049	2.02E-05	0.022
IBD	AGER	6	32148745	rs204995	32154285	G	A	0.217	-0.252	0.108	0.020	1.60E-04
IBS	AGER	6	32150423	rs693906	31835164	C	G	0.113	-0.168	0.052	0.001	0.045
UC	AGER	6	32148745	rs204995	32154285	G	A	0.217	-0.870	0.185	2.59E-06	0.002
VHD	AGER	6	32150423	rs693906	31835164	C	G	0.113	0.212	0.052	4.82E-05	0.092

Supplementary table 6. Results of the summary-based Mendelian randomization integrating whole-blood expression quantitative trait loci. For each disease-gene association, we report the strongest effect observed between GTEX and eQTLGen datasets.

A) Bonferroni-significant disease-gene association with HEIDI P > 0.05.

Disease	Gene	chr	gene_bp	topSNP	topSNP_bp	A1	A2	Freq	b_SMR	se_SMR	p_SMR	p_HEIDI
CD	DNMT3A	2	25510652	rs7578575	25488819	A	T	0.329	-0.834	0.164	3.51E-07	0.383
IBD	DNMT3A	2	25510652	rs7578575	25488819	A	T	0.329	-0.745	0.130	9.22E-09	0.515
UC	DNMT3A	2	25510652	rs7578575	25488819	A	T	0.329	-0.627	0.156	5.73E-05	0.541
UC	CFDP1	16	75327596	rs9934007	75390532	C	T	0.754	0.273	0.070	9.61E-05	0.973
CD	STAT3	17	40502964	rs1053004	40466092	G	A	0.380	0.702	0.128	4.40E-08	0.056
CD	IMPDH2	3	49064299	rs9813813	49207093	A	T	0.110	-1.233	0.294	2.74E-05	0.213
IBD	IMPDH2	3	49064299	rs9813813	49207093	A	T	0.110	-1.328	0.258	2.57E-07	0.071
UC	IMPDH2	3	49064299	rs9813813	49207093	A	T	0.110	-1.445	0.307	2.53E-06	0.050
IBD	UBE2L3	22	21941029	rs2070512	21949411	C	A	0.194	0.163	0.021	1.06E-14	0.169
UC	UBE2L3	22	21941029	rs2070512	21949411	C	A	0.194	0.116	0.027	1.76E-05	0.698
CD	BCL2L15	1	1.14E+08	rs1217393	1.14E+08	A	G	0.271	-0.705	0.170	3.39E-05	0.184
CD	STMN3	20	62271061	rs909333	62270637	G	T	0.207	0.547	0.102	8.31E-08	0.200
IBD	STMN3	20	62271061	rs909333	62270637	G	T	0.207	0.555	0.087	2.01E-10	0.055
UC	STMN3	20	62271061	rs909333	62270637	G	T	0.207	0.498	0.098	4.04E-07	0.062
GORD	PBX2	6	32154964	rs693906	31835164	C	G	0.113	0.154	0.036	1.65E-05	0.058
VHD	AGER	6	32150423	rs693906	31835164	C	G	0.113	0.212	0.052	4.82E-05	0.092

B) Nominally significant effect on VHD of Bonferroni-significant gastrointestinal loci.

Disease	Gene	chr	Probe_bp	topSNP	topSNP_bp	A1	A2	Freq	b_SMR	se_SMR	p_SMR	p_HEIDI
VHD	CFDP1	16	75397489	rs7201989	75471499	G	A	0.246	-0.056	0.018	0.001	0.119
VHD	STAT3	17	40502964	rs1053004	40466092	G	A	0.380	0.093	0.047	0.048	0.948
VHD	IMPDH2	3	49064299	rs34037363	48821036	T	C	0.114	-0.209	0.105	0.047	0.089
VHD	UBE2L3	22	21941029	rs2070512	21949411	C	A	0.194	-0.029	0.009	0.002	0.623
VHD	STMN3	20	62271061	rs909333	62270637	G	T	0.207	-0.076	0.036	0.036	0.093

C) Effect of AGER gene on VHD and gastrointestinal disorders.

Disease	Gene	chr	Probe_bp	topSNP	topSNP_bp	A1	A2	Freq	b_SMR	se_SMR	p_SMR	p_HEIDI
CD	AGER	6	32150423	rs693906	31835164	C	G	0.113	-0.312	0.120	0.009	6.16E-08
GORD	AGER	6	32150423	rs693906	31835164	C	G	0.113	-0.209	0.049	2.02E-05	0.022
IBD	AGER	6	32148745	rs204995	32154285	G	A	0.217	-0.252	0.108	0.020	1.60E-04
IBS	AGER	6	32150423	rs693906	31835164	C	G	0.113	-0.168	0.052	0.001	0.045
UC	AGER	6	32148745	rs204995	32154285	G	A	0.217	-0.870	0.185	2.59E-06	0.002
VHD	AGER	6	32150423	rs693906	31835164	C	G	0.113	0.212	0.052	4.82E-05	0.092

Supplementary table 6. Results of the summary-based Mendelian randomization integrating whole-blood expression quantitative trait loci. For each disease-gene association, we report the strongest effect observed between GTEX and eQTLGen datasets.

A) Bonferroni-significant disease-gene association with HEIDI P > 0.05.

Disease	Gene	chr	gene_bp	topSNP	topSNP_bp	A1	A2	Freq	b_SMR	se_SMR	p_SMR	p_HEIDI
CD	DNMT3A	2	25510652	rs7578575	25488819	A	T	0.329	-0.834	0.164	3.51E-07	0.383
IBD	DNMT3A	2	25510652	rs7578575	25488819	A	T	0.329	-0.745	0.130	9.22E-09	0.515
UC	DNMT3A	2	25510652	rs7578575	25488819	A	T	0.329	-0.627	0.156	5.73E-05	0.541
UC	CFDP1	16	75327596	rs9934007	75390532	C	T	0.754	0.273	0.070	9.61E-05	0.973
CD	STAT3	17	40502964	rs1053004	40466092	G	A	0.380	0.702	0.128	4.40E-08	0.056
CD	IMPDH2	3	49064299	rs9813813	49207093	A	T	0.110	-1.233	0.294	2.74E-05	0.213
IBD	IMPDH2	3	49064299	rs9813813	49207093	A	T	0.110	-1.328	0.258	2.57E-07	0.071
UC	IMPDH2	3	49064299	rs9813813	49207093	A	T	0.110	-1.445	0.307	2.53E-06	0.050
IBD	UBE2L3	22	21941029	rs2070512	21949411	C	A	0.194	0.163	0.021	1.06E-14	0.169
UC	UBE2L3	22	21941029	rs2070512	21949411	C	A	0.194	0.116	0.027	1.76E-05	0.698
CD	BCL2L15	1	1.14E+08	rs1217393	1.14E+08	A	G	0.271	-0.705	0.170	3.39E-05	0.184
CD	STMN3	20	62271061	rs909333	62270637	G	T	0.207	0.547	0.102	8.31E-08	0.200
IBD	STMN3	20	62271061	rs909333	62270637	G	T	0.207	0.555	0.087	2.01E-10	0.055
UC	STMN3	20	62271061	rs909333	62270637	G	T	0.207	0.498	0.098	4.04E-07	0.062
GORD	PBX2	6	32154964	rs693906	31835164	C	G	0.113	0.154	0.036	1.65E-05	0.058
VHD	AGER	6	32150423	rs693906	31835164	C	G	0.113	0.212	0.052	4.82E-05	0.092

B) Nominally significant effect on VHD of Bonferroni-significant gastrointestinal loci.

Disease	Gene	chr	Probe_bp	topSNP	topSNP_bp	A1	A2	Freq	b_SMR	se_SMR	p_SMR	p_HEIDI
VHD	CFDP1	16	75397489	rs7201989	75471499	G	A	0.246	-0.056	0.018	0.001	0.119
VHD	STAT3	17	40502964	rs1053004	40466092	G	A	0.380	0.093	0.047	0.048	0.948
VHD	IMPDH2	3	49064299	rs34037363	48821036	T	C	0.114	-0.209	0.105	0.047	0.089
VHD	UBE2L3	22	21941029	rs2070512	21949411	C	A	0.194	-0.029	0.009	0.002	0.623
VHD	STMN3	20	62271061	rs909333	62270637	G	T	0.207	-0.076	0.036	0.036	0.093

C) Effect of AGER gene on VHD and gastrointestinal disorders.

Disease	Gene	chr	Probe_bp	topSNP	topSNP_bp	A1	A2	Freq	b_SMR	se_SMR	p_SMR	p_HEIDI
CD	AGER	6	32150423	rs693906	31835164	C	G	0.113	-0.312	0.120	0.009	6.16E-08
GORD	AGER	6	32150423	rs693906	31835164	C	G	0.113	-0.209	0.049	2.02E-05	0.022
IBD	AGER	6	32148745	rs204995	32154285	G	A	0.217	-0.252	0.108	0.020	1.60E-04
IBS	AGER	6	32150423	rs693906	31835164	C	G	0.113	-0.168	0.052	0.001	0.045
UC	AGER	6	32148745	rs204995	32154285	G	A	0.217	-0.870	0.185	2.59E-06	0.002
VHD	AGER	6	32150423	rs693906	31835164	C	G	0.113	0.212	0.052	4.82E-05	0.092

Supplementary table 6. Results of the summary-based Mendelian randomization integrating whole-blood expression quantitative trait loci. For each disease-gene association, we report the strongest effect observed between GTEX and eQTLGen datasets.

A) Bonferroni-significant disease-gene association with HEIDI P > 0.05.

Disease	Gene	chr	gene_bp	topSNP	topSNP_bp	A1	A2	Freq	b_SMR	se_SMR	p_SMR	p_HEIDI
CD	DNMT3A	2	25510652	rs7578575	25488819	A	T	0.329	-0.834	0.164	3.51E-07	0.383
IBD	DNMT3A	2	25510652	rs7578575	25488819	A	T	0.329	-0.745	0.130	9.22E-09	0.515
UC	DNMT3A	2	25510652	rs7578575	25488819	A	T	0.329	-0.627	0.156	5.73E-05	0.541
UC	CFDP1	16	75327596	rs9934007	75390532	C	T	0.754	0.273	0.070	9.61E-05	0.973
CD	STAT3	17	40502964	rs1053004	40466092	G	A	0.380	0.702	0.128	4.40E-08	0.056
CD	IMPDH2	3	49064299	rs9813813	49207093	A	T	0.110	-1.233	0.294	2.74E-05	0.213
IBD	IMPDH2	3	49064299	rs9813813	49207093	A	T	0.110	-1.328	0.258	2.57E-07	0.071
UC	IMPDH2	3	49064299	rs9813813	49207093	A	T	0.110	-1.445	0.307	2.53E-06	0.050
IBD	UBE2L3	22	21941029	rs2070512	21949411	C	A	0.194	0.163	0.021	1.06E-14	0.169
UC	UBE2L3	22	21941029	rs2070512	21949411	C	A	0.194	0.116	0.027	1.76E-05	0.698
CD	BCL2L15	1	1.14E+08	rs1217393	1.14E+08	A	G	0.271	-0.705	0.170	3.39E-05	0.184
CD	STMN3	20	62271061	rs909333	62270637	G	T	0.207	0.547	0.102	8.31E-08	0.200
IBD	STMN3	20	62271061	rs909333	62270637	G	T	0.207	0.555	0.087	2.01E-10	0.055
UC	STMN3	20	62271061	rs909333	62270637	G	T	0.207	0.498	0.098	4.04E-07	0.062
GORD	PBX2	6	32154964	rs693906	31835164	C	G	0.113	0.154	0.036	1.65E-05	0.058
VHD	AGER	6	32150423	rs693906	31835164	C	G	0.113	0.212	0.052	4.82E-05	0.092

B) Nominally significant effect on VHD of Bonferroni-significant gastrointestinal loci.

Disease	Gene	chr	Probe_bp	topSNP	topSNP_bp	A1	A2	Freq	b_SMR	se_SMR	p_SMR	p_HEIDI
VHD	CFDP1	16	75397489	rs7201989	75471499	G	A	0.246	-0.056	0.018	0.001	0.119
VHD	STAT3	17	40502964	rs1053004	40466092	G	A	0.380	0.093	0.047	0.048	0.948
VHD	IMPDH2	3	49064299	rs34037363	48821036	T	C	0.114	-0.209	0.105	0.047	0.089
VHD	UBE2L3	22	21941029	rs2070512	21949411	C	A	0.194	-0.029	0.009	0.002	0.623
VHD	STMN3	20	62271061	rs909333	62270637	G	T	0.207	-0.076	0.036	0.036	0.093

C) Effect of AGER gene on VHD and gastrointestinal disorders.

Disease	Gene	chr	Probe_bp	topSNP	topSNP_bp	A1	A2	Freq	b_SMR	se_SMR	p_SMR	p_HEIDI
CD	AGER	6	32150423	rs693906	31835164	C	G	0.113	-0.312	0.120	0.009	6.16E-08
GORD	AGER	6	32150423	rs693906	31835164	C	G	0.113	-0.209	0.049	2.02E-05	0.022
IBD	AGER	6	32148745	rs204995	32154285	G	A	0.217	-0.252	0.108	0.020	1.60E-04
IBS	AGER	6	32150423	rs693906	31835164	C	G	0.113	-0.168	0.052	0.001	0.045
UC	AGER	6	32148745	rs204995	32154285	G	A	0.217	-0.870	0.185	2.59E-06	0.002
VHD	AGER	6	32150423	rs693906	31835164	C	G	0.113	0.212	0.052	4.82E-05	0.092

Supplementary table 6. Results of the summary-based Mendelian randomization integrating whole-blood expression quantitative trait loci. For each disease-gene association, we report the strongest effect observed between GTEX and eQTLGen datasets.

A) Bonferroni-significant disease-gene association with HEIDI P > 0.05.

Disease	Gene	chr	gene_bp	topSNP	topSNP_bp	A1	A2	Freq	b_SMR	se_SMR	p_SMR	p_HEIDI
CD	DNMT3A	2	25510652	rs7578575	25488819	A	T	0.329	-0.834	0.164	3.51E-07	0.383
IBD	DNMT3A	2	25510652	rs7578575	25488819	A	T	0.329	-0.745	0.130	9.22E-09	0.515
UC	DNMT3A	2	25510652	rs7578575	25488819	A	T	0.329	-0.627	0.156	5.73E-05	0.541
UC	CFDP1	16	75327596	rs9934007	75390532	C	T	0.754	0.273	0.070	9.61E-05	0.973
CD	STAT3	17	40502964	rs1053004	40466092	G	A	0.380	0.702	0.128	4.40E-08	0.056
CD	IMPDH2	3	49064299	rs9813813	49207093	A	T	0.110	-1.233	0.294	2.74E-05	0.213
IBD	IMPDH2	3	49064299	rs9813813	49207093	A	T	0.110	-1.328	0.258	2.57E-07	0.071
UC	IMPDH2	3	49064299	rs9813813	49207093	A	T	0.110	-1.445	0.307	2.53E-06	0.050
IBD	UBE2L3	22	21941029	rs2070512	21949411	C	A	0.194	0.163	0.021	1.06E-14	0.169
UC	UBE2L3	22	21941029	rs2070512	21949411	C	A	0.194	0.116	0.027	1.76E-05	0.698
CD	BCL2L15	1	1.14E+08	rs1217393	1.14E+08	A	G	0.271	-0.705	0.170	3.39E-05	0.184
CD	STMN3	20	62271061	rs909333	62270637	G	T	0.207	0.547	0.102	8.31E-08	0.200
IBD	STMN3	20	62271061	rs909333	62270637	G	T	0.207	0.555	0.087	2.01E-10	0.055
UC	STMN3	20	62271061	rs909333	62270637	G	T	0.207	0.498	0.098	4.04E-07	0.062
GORD	PBX2	6	32154964	rs693906	31835164	C	G	0.113	0.154	0.036	1.65E-05	0.058
VHD	AGER	6	32150423	rs693906	31835164	C	G	0.113	0.212	0.052	4.82E-05	0.092

B) Nominally significant effect on VHD of Bonferroni-significant gastrointestinal loci.

Disease	Gene	chr	Probe_bp	topSNP	topSNP_bp	A1	A2	Freq	b_SMR	se_SMR	p_SMR	p_HEIDI
VHD	CFDP1	16	75397489	rs7201989	75471499	G	A	0.246	-0.056	0.018	0.001	0.119
VHD	STAT3	17	40502964	rs1053004	40466092	G	A	0.380	0.093	0.047	0.048	0.948
VHD	IMPDH2	3	49064299	rs34037363	48821036	T	C	0.114	-0.209	0.105	0.047	0.089
VHD	UBE2L3	22	21941029	rs2070512	21949411	C	A	0.194	-0.029	0.009	0.002	0.623
VHD	STMN3	20	62271061	rs909333	62270637	G	T	0.207	-0.076	0.036	0.036	0.093

C) Effect of AGER gene on VHD and gastrointestinal disorders.

Disease	Gene	chr	Probe_bp	topSNP	topSNP_bp	A1	A2	Freq	b_SMR	se_SMR	p_SMR	p_HEIDI
CD	AGER	6	32150423	rs693906	31835164	C	G	0.113	-0.312	0.120	0.009	6.16E-08
GORD	AGER	6	32150423	rs693906	31835164	C	G	0.113	-0.209	0.049	2.02E-05	0.022
IBD	AGER	6	32148745	rs204995	32154285	G	A	0.217	-0.252	0.108	0.020	1.60E-04
IBS	AGER	6	32150423	rs693906	31835164	C	G	0.113	-0.168	0.052	0.001	0.045
UC	AGER	6	32148745	rs204995	32154285	G	A	0.217	-0.870	0.185	2.59E-06	0.002
VHD	AGER	6	32150423	rs693906	31835164	C	G	0.113	0.212	0.052	4.82E-05	0.092

Supplementary table 6. Results of the summary-based Mendelian randomization integrating whole-blood expression quantitative trait loci. For each disease-gene association, we report the strongest effect observed between GTEX and eQTLGen datasets.

A) Bonferroni-significant disease-gene association with HEIDI P > 0.05.

Disease	Gene	chr	gene_bp	topSNP	topSNP_bp	A1	A2	Freq	b_SMR	se_SMR	p_SMR	p_HEIDI
CD	DNMT3A	2	25510652	rs7578575	25488819	A	T	0.329	-0.834	0.164	3.51E-07	0.383
IBD	DNMT3A	2	25510652	rs7578575	25488819	A	T	0.329	-0.745	0.130	9.22E-09	0.515
UC	DNMT3A	2	25510652	rs7578575	25488819	A	T	0.329	-0.627	0.156	5.73E-05	0.541
UC	CFDP1	16	75327596	rs9934007	75390532	C	T	0.754	0.273	0.070	9.61E-05	0.973
CD	STAT3	17	40502964	rs1053004	40466092	G	A	0.380	0.702	0.128	4.40E-08	0.056
CD	IMPDH2	3	49064299	rs9813813	49207093	A	T	0.110	-1.233	0.294	2.74E-05	0.213
IBD	IMPDH2	3	49064299	rs9813813	49207093	A	T	0.110	-1.328	0.258	2.57E-07	0.071
UC	IMPDH2	3	49064299	rs9813813	49207093	A	T	0.110	-1.445	0.307	2.53E-06	0.050
IBD	UBE2L3	22	21941029	rs2070512	21949411	C	A	0.194	0.163	0.021	1.06E-14	0.169
UC	UBE2L3	22	21941029	rs2070512	21949411	C	A	0.194	0.116	0.027	1.76E-05	0.698
CD	BCL2L15	1	1.14E+08	rs1217393	1.14E+08	A	G	0.271	-0.705	0.170	3.39E-05	0.184
CD	STMN3	20	62271061	rs909333	62270637	G	T	0.207	0.547	0.102	8.31E-08	0.200
IBD	STMN3	20	62271061	rs909333	62270637	G	T	0.207	0.555	0.087	2.01E-10	0.055
UC	STMN3	20	62271061	rs909333	62270637	G	T	0.207	0.498	0.098	4.04E-07	0.062
GORD	PBX2	6	32154964	rs693906	31835164	C	G	0.113	0.154	0.036	1.65E-05	0.058
VHD	AGER	6	32150423	rs693906	31835164	C	G	0.113	0.212	0.052	4.82E-05	0.092

B) Nominally significant effect on VHD of Bonferroni-significant gastrointestinal loci.

Disease	Gene	chr	Probe_bp	topSNP	topSNP_bp	A1	A2	Freq	b_SMR	se_SMR	p_SMR	p_HEIDI
VHD	CFDP1	16	75397489	rs7201989	75471499	G	A	0.246	-0.056	0.018	0.001	0.119
VHD	STAT3	17	40502964	rs1053004	40466092	G	A	0.380	0.093	0.047	0.048	0.948
VHD	IMPDH2	3	49064299	rs34037363	48821036	T	C	0.114	-0.209	0.105	0.047	0.089
VHD	UBE2L3	22	21941029	rs2070512	21949411	C	A	0.194	-0.029	0.009	0.002	0.623
VHD	STMN3	20	62271061	rs909333	62270637	G	T	0.207	-0.076	0.036	0.036	0.093

C) Effect of AGER gene on VHD and gastrointestinal disorders.

Disease	Gene	chr	Probe_bp	topSNP	topSNP_bp	A1	A2	Freq	b_SMR	se_SMR	p_SMR	p_HEIDI
CD	AGER	6	32150423	rs693906	31835164	C	G	0.113	-0.312	0.120	0.009	6.16E-08
GORD	AGER	6	32150423	rs693906	31835164	C	G	0.113	-0.209	0.049	2.02E-05	0.022
IBD	AGER	6	32148745	rs204995	32154285	G	A	0.217	-0.252	0.108	0.020	1.60E-04
IBS	AGER	6	32150423	rs693906	31835164	C	G	0.113	-0.168	0.052	0.001	0.045
UC	AGER	6	32148745	rs204995	32154285	G	A	0.217	-0.870	0.185	2.59E-06	0.002
VHD	AGER	6	32150423	rs693906	31835164	C	G	0.113	0.212	0.052	4.82E-05	0.092

Supplementary table 6. Results of the summary-based Mendelian randomization integrating whole-blood expression quantitative trait loci. For each disease-gene association, we report the strongest effect observed between GTEX and eQTLGen datasets.

A) Bonferroni-significant disease-gene association with HEIDI P > 0.05.

Disease	Gene	chr	gene_bp	topSNP	topSNP_bp	A1	A2	Freq	b_SMR	se_SMR	p_SMR	p_HEIDI
CD	DNMT3A	2	25510652	rs7578575	25488819	A	T	0.329	-0.834	0.164	3.51E-07	0.383
IBD	DNMT3A	2	25510652	rs7578575	25488819	A	T	0.329	-0.745	0.130	9.22E-09	0.515
UC	DNMT3A	2	25510652	rs7578575	25488819	A	T	0.329	-0.627	0.156	5.73E-05	0.541
UC	CFDP1	16	75327596	rs9934007	75390532	C	T	0.754	0.273	0.070	9.61E-05	0.973
CD	STAT3	17	40502964	rs1053004	40466092	G	A	0.380	0.702	0.128	4.40E-08	0.056
CD	IMPDH2	3	49064299	rs9813813	49207093	A	T	0.110	-1.233	0.294	2.74E-05	0.213
IBD	IMPDH2	3	49064299	rs9813813	49207093	A	T	0.110	-1.328	0.258	2.57E-07	0.071
UC	IMPDH2	3	49064299	rs9813813	49207093	A	T	0.110	-1.445	0.307	2.53E-06	0.050
IBD	UBE2L3	22	21941029	rs2070512	21949411	C	A	0.194	0.163	0.021	1.06E-14	0.169
UC	UBE2L3	22	21941029	rs2070512	21949411	C	A	0.194	0.116	0.027	1.76E-05	0.698
CD	BCL2L15	1	1.14E+08	rs1217393	1.14E+08	A	G	0.271	-0.705	0.170	3.39E-05	0.184
CD	STMN3	20	62271061	rs909333	62270637	G	T	0.207	0.547	0.102	8.31E-08	0.200
IBD	STMN3	20	62271061	rs909333	62270637	G	T	0.207	0.555	0.087	2.01E-10	0.055
UC	STMN3	20	62271061	rs909333	62270637	G	T	0.207	0.498	0.098	4.04E-07	0.062
GORD	PBX2	6	32154964	rs693906	31835164	C	G	0.113	0.154	0.036	1.65E-05	0.058
VHD	AGER	6	32150423	rs693906	31835164	C	G	0.113	0.212	0.052	4.82E-05	0.092

B) Nominally significant effect on VHD of Bonferroni-significant gastrointestinal loci.

Disease	Gene	chr	Probe_bp	topSNP	topSNP_bp	A1	A2	Freq	b_SMR	se_SMR	p_SMR	p_HEIDI
VHD	CFDP1	16	75397489	rs7201989	75471499	G	A	0.246	-0.056	0.018	0.001	0.119
VHD	STAT3	17	40502964	rs1053004	40466092	G	A	0.380	0.093	0.047	0.048	0.948
VHD	IMPDH2	3	49064299	rs34037363	48821036	T	C	0.114	-0.209	0.105	0.047	0.089
VHD	UBE2L3	22	21941029	rs2070512	21949411	C	A	0.194	-0.029	0.009	0.002	0.623
VHD	STMN3	20	62271061	rs909333	62270637	G	T	0.207	-0.076	0.036	0.036	0.093

C) Effect of AGER gene on VHD and gastrointestinal disorders.

Disease	Gene	chr	Probe_bp	topSNP	topSNP_bp	A1	A2	Freq	b_SMR	se_SMR	p_SMR	p_HEIDI
CD	AGER	6	32150423	rs693906	31835164	C	G	0.113	-0.312	0.120	0.009	6.16E-08
GORD	AGER	6	32150423	rs693906	31835164	C	G	0.113	-0.209	0.049	2.02E-05	0.022
IBD	AGER	6	32148745	rs204995	32154285	G	A	0.217	-0.252	0.108	0.020	1.60E-04
IBS	AGER	6	32150423	rs693906	31835164	C	G	0.113	-0.168	0.052	0.001	0.045
UC	AGER	6	32148745	rs204995	32154285	G	A	0.217	-0.870	0.185	2.59E-06	0.002
VHD	AGER	6	32150423	rs693906	31835164	C	G	0.113	0.212	0.052	4.82E-05	0.092

Supplementary table 6. Results of the summary-based Mendelian randomization integrating whole-blood expression quantitative trait loci. For each disease-gene association, we report the strongest effect observed between GTEX and eQTLGen datasets.

A) Bonferroni-significant disease-gene association with HEIDI P > 0.05.

Disease	Gene	chr	gene_bp	topSNP	topSNP_bp	A1	A2	Freq	b_SMR	se_SMR	p_SMR	p_HEIDI
CD	DNMT3A	2	25510652	rs7578575	25488819	A	T	0.329	-0.834	0.164	3.51E-07	0.383
IBD	DNMT3A	2	25510652	rs7578575	25488819	A	T	0.329	-0.745	0.130	9.22E-09	0.515
UC	DNMT3A	2	25510652	rs7578575	25488819	A	T	0.329	-0.627	0.156	5.73E-05	0.541
UC	CFDP1	16	75327596	rs9934007	75390532	C	T	0.754	0.273	0.070	9.61E-05	0.973
CD	STAT3	17	40502964	rs1053004	40466092	G	A	0.380	0.702	0.128	4.40E-08	0.056
CD	IMPDH2	3	49064299	rs9813813	49207093	A	T	0.110	-1.233	0.294	2.74E-05	0.213
IBD	IMPDH2	3	49064299	rs9813813	49207093	A	T	0.110	-1.328	0.258	2.57E-07	0.071
UC	IMPDH2	3	49064299	rs9813813	49207093	A	T	0.110	-1.445	0.307	2.53E-06	0.050
IBD	UBE2L3	22	21941029	rs2070512	21949411	C	A	0.194	0.163	0.021	1.06E-14	0.169
UC	UBE2L3	22	21941029	rs2070512	21949411	C	A	0.194	0.116	0.027	1.76E-05	0.698
CD	BCL2L15	1	1.14E+08	rs1217393	1.14E+08	A	G	0.271	-0.705	0.170	3.39E-05	0.184
CD	STMN3	20	62271061	rs909333	62270637	G	T	0.207	0.547	0.102	8.31E-08	0.200
IBD	STMN3	20	62271061	rs909333	62270637	G	T	0.207	0.555	0.087	2.01E-10	0.055
UC	STMN3	20	62271061	rs909333	62270637	G	T	0.207	0.498	0.098	4.04E-07	0.062
GORD	PBX2	6	32154964	rs693906	31835164	C	G	0.113	0.154	0.036	1.65E-05	0.058
VHD	AGER	6	32150423	rs693906	31835164	C	G	0.113	0.212	0.052	4.82E-05	0.092

B) Nominally significant effect on VHD of Bonferroni-significant gastrointestinal loci.

Disease	Gene	chr	Probe_bp	topSNP	topSNP_bp	A1	A2	Freq	b_SMR	se_SMR	p_SMR	p_HEIDI
VHD	CFDP1	16	75397489	rs7201989	75471499	G	A	0.246	-0.056	0.018	0.001	0.119
VHD	STAT3	17	40502964	rs1053004	40466092	G	A	0.380	0.093	0.047	0.048	0.948
VHD	IMPDH2	3	49064299	rs34037363	48821036	T	C	0.114	-0.209	0.105	0.047	0.089
VHD	UBE2L3	22	21941029	rs2070512	21949411	C	A	0.194	-0.029	0.009	0.002	0.623
VHD	STMN3	20	62271061	rs909333	62270637	G	T	0.207	-0.076	0.036	0.036	0.093

C) Effect of AGER gene on VHD and gastrointestinal disorders.

Disease	Gene	chr	Probe_bp	topSNP	topSNP_bp	A1	A2	Freq	b_SMR	se_SMR	p_SMR	p_HEIDI
CD	AGER	6	32150423	rs693906	31835164	C	G	0.113	-0.312	0.120	0.009	6.16E-08
GORD	AGER	6	32150423	rs693906	31835164	C	G	0.113	-0.209	0.049	2.02E-05	0.022
IBD	AGER	6	32148745	rs204995	32154285	G	A	0.217	-0.252	0.108	0.020	1.60E-04
IBS	AGER	6	32150423	rs693906	31835164	C	G	0.113	-0.168	0.052	0.001	0.045
UC	AGER	6	32148745	rs204995	32154285	G	A	0.217	-0.870	0.185	2.59E-06	0.002
VHD	AGER	6	32150423	rs693906	31835164	C	G	0.113	0.212	0.052	4.82E-05	0.092

Supplementary table 6. Results of the summary-based Mendelian randomization integrating whole-blood expression quantitative trait loci. For each disease-gene association, we report the strongest effect observed between GTEX and eQTLGen datasets.

A) Bonferroni-significant disease-gene association with HEIDI P > 0.05.

Disease	Gene	chr	gene_bp	topSNP	topSNP_bp	A1	A2	Freq	b_SMR	se_SMR	p_SMR	p_HEIDI
CD	DNMT3A	2	25510652	rs7578575	25488819	A	T	0.329	-0.834	0.164	3.51E-07	0.383
IBD	DNMT3A	2	25510652	rs7578575	25488819	A	T	0.329	-0.745	0.130	9.22E-09	0.515
UC	DNMT3A	2	25510652	rs7578575	25488819	A	T	0.329	-0.627	0.156	5.73E-05	0.541
UC	CFDP1	16	75327596	rs9934007	75390532	C	T	0.754	0.273	0.070	9.61E-05	0.973
CD	STAT3	17	40502964	rs1053004	40466092	G	A	0.380	0.702	0.128	4.40E-08	0.056
CD	IMPDH2	3	49064299	rs9813813	49207093	A	T	0.110	-1.233	0.294	2.74E-05	0.213
IBD	IMPDH2	3	49064299	rs9813813	49207093	A	T	0.110	-1.328	0.258	2.57E-07	0.071
UC	IMPDH2	3	49064299	rs9813813	49207093	A	T	0.110	-1.445	0.307	2.53E-06	0.050
IBD	UBE2L3	22	21941029	rs2070512	21949411	C	A	0.194	0.163	0.021	1.06E-14	0.169
UC	UBE2L3	22	21941029	rs2070512	21949411	C	A	0.194	0.116	0.027	1.76E-05	0.698
CD	BCL2L15	1	1.14E+08	rs1217393	1.14E+08	A	G	0.271	-0.705	0.170	3.39E-05	0.184
CD	STMN3	20	62271061	rs909333	62270637	G	T	0.207	0.547	0.102	8.31E-08	0.200
IBD	STMN3	20	62271061	rs909333	62270637	G	T	0.207	0.555	0.087	2.01E-10	0.055
UC	STMN3	20	62271061	rs909333	62270637	G	T	0.207	0.498	0.098	4.04E-07	0.062
GORD	PBX2	6	32154964	rs693906	31835164	C	G	0.113	0.154	0.036	1.65E-05	0.058
VHD	AGER	6	32150423	rs693906	31835164	C	G	0.113	0.212	0.052	4.82E-05	0.092

B) Nominally significant effect on VHD of Bonferroni-significant gastrointestinal loci.

Disease	Gene	chr	Probe_bp	topSNP	topSNP_bp	A1	A2	Freq	b_SMR	se_SMR	p_SMR	p_HEIDI
VHD	CFDP1	16	75397489	rs7201989	75471499	G	A	0.246	-0.056	0.018	0.001	0.119
VHD	STAT3	17	40502964	rs1053004	40466092	G	A	0.380	0.093	0.047	0.048	0.948
VHD	IMPDH2	3	49064299	rs34037363	48821036	T	C	0.114	-0.209	0.105	0.047	0.089
VHD	UBE2L3	22	21941029	rs2070512	21949411	C	A	0.194	-0.029	0.009	0.002	0.623
VHD	STMN3	20	62271061	rs909333	62270637	G	T	0.207	-0.076	0.036	0.036	0.093

C) Effect of AGER gene on VHD and gastrointestinal disorders.

Disease	Gene	chr	Probe_bp	topSNP	topSNP_bp	A1	A2	Freq	b_SMR	se_SMR	p_SMR	p_HEIDI
CD	AGER	6	32150423	rs693906	31835164	C	G	0.113	-0.312	0.120	0.009	6.16E-08
GORD	AGER	6	32150423	rs693906	31835164	C	G	0.113	-0.209	0.049	2.02E-05	0.022
IBD	AGER	6	32148745	rs204995	32154285	G	A	0.217	-0.252	0.108	0.020	1.60E-04
IBS	AGER	6	32150423	rs693906	31835164	C	G	0.113	-0.168	0.052	0.001	0.045
UC	AGER	6	32148745	rs204995	32154285	G	A	0.217	-0.870	0.185	2.59E-06	0.002
VHD	AGER	6	32150423	rs693906	31835164	C	G	0.113	0.212	0.052	4.82E-05	0.092

Supplementary table 6. Results of the summary-based Mendelian randomization integrating whole-blood expression quantitative trait loci. For each disease-gene association, we report the strongest effect observed between GTEX and eQTLGen datasets.

A) Bonferroni-significant disease-gene association with HEIDI P > 0.05.

Disease	Gene	chr	gene_bp	topSNP	topSNP_bp	A1	A2	Freq	b_SMR	se_SMR	p_SMR	p_HEIDI
CD	DNMT3A	2	25510652	rs7578575	25488819	A	T	0.329	-0.834	0.164	3.51E-07	0.383
IBD	DNMT3A	2	25510652	rs7578575	25488819	A	T	0.329	-0.745	0.130	9.22E-09	0.515
UC	DNMT3A	2	25510652	rs7578575	25488819	A	T	0.329	-0.627	0.156	5.73E-05	0.541
UC	CFDP1	16	75327596	rs9934007	75390532	C	T	0.754	0.273	0.070	9.61E-05	0.973
CD	STAT3	17	40502964	rs1053004	40466092	G	A	0.380	0.702	0.128	4.40E-08	0.056
CD	IMPDH2	3	49064299	rs9813813	49207093	A	T	0.110	-1.233	0.294	2.74E-05	0.213
IBD	IMPDH2	3	49064299	rs9813813	49207093	A	T	0.110	-1.328	0.258	2.57E-07	0.071
UC	IMPDH2	3	49064299	rs9813813	49207093	A	T	0.110	-1.445	0.307	2.53E-06	0.050
IBD	UBE2L3	22	21941029	rs2070512	21949411	C	A	0.194	0.163	0.021	1.06E-14	0.169
UC	UBE2L3	22	21941029	rs2070512	21949411	C	A	0.194	0.116	0.027	1.76E-05	0.698
CD	BCL2L15	1	1.14E+08	rs1217393	1.14E+08	A	G	0.271	-0.705	0.170	3.39E-05	0.184
CD	STMN3	20	62271061	rs909333	62270637	G	T	0.207	0.547	0.102	8.31E-08	0.200
IBD	STMN3	20	62271061	rs909333	62270637	G	T	0.207	0.555	0.087	2.01E-10	0.055
UC	STMN3	20	62271061	rs909333	62270637	G	T	0.207	0.498	0.098	4.04E-07	0.062
GORD	PBX2	6	32154964	rs693906	31835164	C	G	0.113	0.154	0.036	1.65E-05	0.058
VHD	AGER	6	32150423	rs693906	31835164	C	G	0.113	0.212	0.052	4.82E-05	0.092

B) Nominally significant effect on VHD of Bonferroni-significant gastrointestinal loci.

Disease	Gene	chr	Probe_bp	topSNP	topSNP_bp	A1	A2	Freq	b_SMR	se_SMR	p_SMR	p_HEIDI
VHD	CFDP1	16	75397489	rs7201989	75471499	G	A	0.246	-0.056	0.018	0.001	0.119
VHD	STAT3	17	40502964	rs1053004	40466092	G	A	0.380	0.093	0.047	0.048	0.948
VHD	IMPDH2	3	49064299	rs34037363	48821036	T	C	0.114	-0.209	0.105	0.047	0.089
VHD	UBE2L3	22	21941029	rs2070512	21949411	C	A	0.194	-0.029	0.009	0.002	0.623
VHD	STMN3	20	62271061	rs909333	62270637	G	T	0.207	-0.076	0.036	0.036	0.093

C) Effect of AGER gene on VHD and gastrointestinal disorders.

Disease	Gene	chr	Probe_bp	topSNP	topSNP_bp	A1	A2	Freq	b_SMR	se_SMR	p_SMR	p_HEIDI
CD	AGER	6	32150423	rs693906	31835164	C	G	0.113	-0.312	0.120	0.009	6.16E-08
GORD	AGER	6	32150423	rs693906	31835164	C	G	0.113	-0.209	0.049	2.02E-05	0.022
IBD	AGER	6	32148745	rs204995	32154285	G	A	0.217	-0.252	0.108	0.020	1.60E-04
IBS	AGER	6	32150423	rs693906	31835164	C	G	0.113	-0.168	0.052	0.001	0.045
UC	AGER	6	32148745	rs204995	32154285	G	A	0.217	-0.870	0.185	2.59E-06	0.002
VHD	AGER	6	32150423	rs693906	31835164	C	G	0.113	0.212	0.052	4.82E-05	0.092

Supplementary table 6. Results of the summary-based Mendelian randomization integrating whole-blood expression quantitative trait loci. For each disease-gene association, we report the strongest effect observed between GTEX and eQTLGen datasets.

A) Bonferroni-significant disease-gene association with HEIDI P > 0.05.

Disease	Gene	chr	gene_bp	topSNP	topSNP_bp	A1	A2	Freq	b_SMR	se_SMR	p_SMR	p_HEIDI
CD	DNMT3A	2	25510652	rs7578575	25488819	A	T	0.329	-0.834	0.164	3.51E-07	0.383
IBD	DNMT3A	2	25510652	rs7578575	25488819	A	T	0.329	-0.745	0.130	9.22E-09	0.515
UC	DNMT3A	2	25510652	rs7578575	25488819	A	T	0.329	-0.627	0.156	5.73E-05	0.541
UC	CFDP1	16	75327596	rs9934007	75390532	C	T	0.754	0.273	0.070	9.61E-05	0.973
CD	STAT3	17	40502964	rs1053004	40466092	G	A	0.380	0.702	0.128	4.40E-08	0.056
CD	IMPDH2	3	49064299	rs9813813	49207093	A	T	0.110	-1.233	0.294	2.74E-05	0.213
IBD	IMPDH2	3	49064299	rs9813813	49207093	A	T	0.110	-1.328	0.258	2.57E-07	0.071
UC	IMPDH2	3	49064299	rs9813813	49207093	A	T	0.110	-1.445	0.307	2.53E-06	0.050
IBD	UBE2L3	22	21941029	rs2070512	21949411	C	A	0.194	0.163	0.021	1.06E-14	0.169
UC	UBE2L3	22	21941029	rs2070512	21949411	C	A	0.194	0.116	0.027	1.76E-05	0.698
CD	BCL2L15	1	1.14E+08	rs1217393	1.14E+08	A	G	0.271	-0.705	0.170	3.39E-05	0.184
CD	STMN3	20	62271061	rs909333	62270637	G	T	0.207	0.547	0.102	8.31E-08	0.200
IBD	STMN3	20	62271061	rs909333	62270637	G	T	0.207	0.555	0.087	2.01E-10	0.055
UC	STMN3	20	62271061	rs909333	62270637	G	T	0.207	0.498	0.098	4.04E-07	0.062
GORD	PBX2	6	32154964	rs693906	31835164	C	G	0.113	0.154	0.036	1.65E-05	0.058
VHD	AGER	6	32150423	rs693906	31835164	C	G	0.113	0.212	0.052	4.82E-05	0.092

B) Nominally significant effect on VHD of Bonferroni-significant gastrointestinal loci.

Disease	Gene	chr	Probe_bp	topSNP	topSNP_bp	A1	A2	Freq	b_SMR	se_SMR	p_SMR	p_HEIDI
VHD	CFDP1	16	75397489	rs7201989	75471499	G	A	0.246	-0.056	0.018	0.001	0.119
VHD	STAT3	17	40502964	rs1053004	40466092	G	A	0.380	0.093	0.047	0.048	0.948
VHD	IMPDH2	3	49064299	rs34037363	48821036	T	C	0.114	-0.209	0.105	0.047	0.089
VHD	UBE2L3	22	21941029	rs2070512	21949411	C	A	0.194	-0.029	0.009	0.002	0.623
VHD	STMN3	20	62271061	rs909333	62270637	G	T	0.207	-0.076	0.036	0.036	0.093

C) Effect of AGER gene on VHD and gastrointestinal disorders.

Disease	Gene	chr	Probe_bp	topSNP	topSNP_bp	A1	A2	Freq	b_SMR	se_SMR	p_SMR	p_HEIDI
CD	AGER	6	32150423	rs693906	31835164	C	G	0.113	-0.312	0.120	0.009	6.16E-08
GORD	AGER	6	32150423	rs693906	31835164	C	G	0.113	-0.209	0.049	2.02E-05	0.022
IBD	AGER	6	32148745	rs204995	32154285	G	A	0.217	-0.252	0.108	0.020	1.60E-04
IBS	AGER	6	32150423	rs693906	31835164	C	G	0.113	-0.168	0.052	0.001	0.045
UC	AGER	6	32148745	rs204995	32154285	G	A	0.217	-0.870	0.185	2.59E-06	0.002
VHD	AGER	6	32150423	rs693906	31835164	C	G	0.113	0.212	0.052	4.82E-05	0.092

Supplementary table 6. Results of the summary-based Mendelian randomization integrating whole-blood expression quantitative trait loci. For each disease-gene association, we report the strongest effect observed between GTEX and eQTLGen datasets.

A) Bonferroni-significant disease-gene association with HEIDI P > 0.05.

Disease	Gene	chr	gene_bp	topSNP	topSNP_bp	A1	A2	Freq	b_SMR	se_SMR	p_SMR	p_HEIDI
CD	DNMT3A	2	25510652	rs7578575	25488819	A	T	0.329	-0.834	0.164	3.51E-07	0.383
IBD	DNMT3A	2	25510652	rs7578575	25488819	A	T	0.329	-0.745	0.130	9.22E-09	0.515
UC	DNMT3A	2	25510652	rs7578575	25488819	A	T	0.329	-0.627	0.156	5.73E-05	0.541
UC	CFDP1	16	75327596	rs9934007	75390532	C	T	0.754	0.273	0.070	9.61E-05	0.973
CD	STAT3	17	40502964	rs1053004	40466092	G	A	0.380	0.702	0.128	4.40E-08	0.056
CD	IMPDH2	3	49064299	rs9813813	49207093	A	T	0.110	-1.233	0.294	2.74E-05	0.213
IBD	IMPDH2	3	49064299	rs9813813	49207093	A	T	0.110	-1.328	0.258	2.57E-07	0.071
UC	IMPDH2	3	49064299	rs9813813	49207093	A	T	0.110	-1.445	0.307	2.53E-06	0.050
IBD	UBE2L3	22	21941029	rs2070512	21949411	C	A	0.194	0.163	0.021	1.06E-14	0.169
UC	UBE2L3	22	21941029	rs2070512	21949411	C	A	0.194	0.116	0.027	1.76E-05	0.698
CD	BCL2L15	1	1.14E+08	rs1217393	1.14E+08	A	G	0.271	-0.705	0.170	3.39E-05	0.184
CD	STMN3	20	62271061	rs909333	62270637	G	T	0.207	0.547	0.102	8.31E-08	0.200
IBD	STMN3	20	62271061	rs909333	62270637	G	T	0.207	0.555	0.087	2.01E-10	0.055
UC	STMN3	20	62271061	rs909333	62270637	G	T	0.207	0.498	0.098	4.04E-07	0.062
GORD	PBX2	6	32154964	rs693906	31835164	C	G	0.113	0.154	0.036	1.65E-05	0.058
VHD	AGER	6	32150423	rs693906	31835164	C	G	0.113	0.212	0.052	4.82E-05	0.092

B) Nominally significant effect on VHD of Bonferroni-significant gastrointestinal loci.

Disease	Gene	chr	Probe_bp	topSNP	topSNP_bp	A1	A2	Freq	b_SMR	se_SMR	p_SMR	p_HEIDI
VHD	CFDP1	16	75397489	rs7201989	75471499	G	A	0.246	-0.056	0.018	0.001	0.119
VHD	STAT3	17	40502964	rs1053004	40466092	G	A	0.380	0.093	0.047	0.048	0.948
VHD	IMPDH2	3	49064299	rs34037363	48821036	T	C	0.114	-0.209	0.105	0.047	0.089
VHD	UBE2L3	22	21941029	rs2070512	21949411	C	A	0.194	-0.029	0.009	0.002	0.623
VHD	STMN3	20	62271061	rs909333	62270637	G	T	0.207	-0.076	0.036	0.036	0.093

C) Effect of AGER gene on VHD and gastrointestinal disorders.

Disease	Gene	chr	Probe_bp	topSNP	topSNP_bp	A1	A2	Freq	b_SMR	se_SMR	p_SMR	p_HEIDI
CD	AGER	6	32150423	rs693906	31835164	C	G	0.113	-0.312	0.120	0.009	6.16E-08
GORD	AGER	6	32150423	rs693906	31835164	C	G	0.113	-0.209	0.049	2.02E-05	0.022
IBD	AGER	6	32148745	rs204995	32154285	G	A	0.217	-0.252	0.108	0.020	1.60E-04
IBS	AGER	6	32150423	rs693906	31835164	C	G	0.113	-0.168	0.052	0.001	0.045
UC	AGER	6	32148745	rs204995	32154285	G	A	0.217	-0.870	0.185	2.59E-06	0.002
VHD	AGER	6	32150423	rs693906	31835164	C	G	0.113	0.212	0.052	4.82E-05	0.092

Supplementary table 6. Results of the summary-based Mendelian randomization integrating whole-blood expression quantitative trait loci. For each disease-gene association, we report the strongest effect observed between GTEX and eQTLGen datasets.

A) Bonferroni-significant disease-gene association with HEIDI P > 0.05.

Disease	Gene	chr	gene_bp	topSNP	topSNP_bp	A1	A2	Freq	b_SMR	se_SMR	p_SMR	p_HEIDI
CD	DNMT3A	2	25510652	rs7578575	25488819	A	T	0.329	-0.834	0.164	3.51E-07	0.383
IBD	DNMT3A	2	25510652	rs7578575	25488819	A	T	0.329	-0.745	0.130	9.22E-09	0.515
UC	DNMT3A	2	25510652	rs7578575	25488819	A	T	0.329	-0.627	0.156	5.73E-05	0.541
UC	CFDP1	16	75327596	rs9934007	75390532	C	T	0.754	0.273	0.070	9.61E-05	0.973
CD	STAT3	17	40502964	rs1053004	40466092	G	A	0.380	0.702	0.128	4.40E-08	0.056
CD	IMPDH2	3	49064299	rs9813813	49207093	A	T	0.110	-1.233	0.294	2.74E-05	0.213
IBD	IMPDH2	3	49064299	rs9813813	49207093	A	T	0.110	-1.328	0.258	2.57E-07	0.071
UC	IMPDH2	3	49064299	rs9813813	49207093	A	T	0.110	-1.445	0.307	2.53E-06	0.050
IBD	UBE2L3	22	21941029	rs2070512	21949411	C	A	0.194	0.163	0.021	1.06E-14	0.169
UC	UBE2L3	22	21941029	rs2070512	21949411	C	A	0.194	0.116	0.027	1.76E-05	0.698
CD	BCL2L15	1	1.14E+08	rs1217393	1.14E+08	A	G	0.271	-0.705	0.170	3.39E-05	0.184
CD	STMN3	20	62271061	rs909333	62270637	G	T	0.207	0.547	0.102	8.31E-08	0.200
IBD	STMN3	20	62271061	rs909333	62270637	G	T	0.207	0.555	0.087	2.01E-10	0.055
UC	STMN3	20	62271061	rs909333	62270637	G	T	0.207	0.498	0.098	4.04E-07	0.062
GORD	PBX2	6	32154964	rs693906	31835164	C	G	0.113	0.154	0.036	1.65E-05	0.058
VHD	AGER	6	32150423	rs693906	31835164	C	G	0.113	0.212	0.052	4.82E-05	0.092

B) Nominally significant effect on VHD of Bonferroni-significant gastrointestinal loci.

Disease	Gene	chr	Probe_bp	topSNP	topSNP_bp	A1	A2	Freq	b_SMR	se_SMR	p_SMR	p_HEIDI
VHD	CFDP1	16	75397489	rs7201989	75471499	G	A	0.246	-0.056	0.018	0.001	0.119
VHD	STAT3	17	40502964	rs1053004	40466092	G	A	0.380	0.093	0.047	0.048	0.948
VHD	IMPDH2	3	49064299	rs34037363	48821036	T	C	0.114	-0.209	0.105	0.047	0.089
VHD	UBE2L3	22	21941029	rs2070512	21949411	C	A	0.194	-0.029	0.009	0.002	0.623
VHD	STMN3	20	62271061	rs909333	62270637	G	T	0.207	-0.076	0.036	0.036	0.093

C) Effect of AGER gene on VHD and gastrointestinal disorders.

Disease	Gene	chr	Probe_bp	topSNP	topSNP_bp	A1	A2	Freq	b_SMR	se_SMR	p_SMR	p_HEIDI
CD	AGER	6	32150423	rs693906	31835164	C	G	0.113	-0.312	0.120	0.009	6.16E-08
GORD	AGER	6	32150423	rs693906	31835164	C	G	0.113	-0.209	0.049	2.02E-05	0.022
IBD	AGER	6	32148745	rs204995	32154285	G	A	0.217	-0.252	0.108	0.020	1.60E-04
IBS	AGER	6	32150423	rs693906	31835164	C	G	0.113	-0.168	0.052	0.001	0.045
UC	AGER	6	32148745	rs204995	32154285	G	A	0.217	-0.870	0.185	2.59E-06	0.002
VHD	AGER	6	32150423	rs693906	31835164	C	G	0.113	0.212	0.052	4.82E-05	0.092

Supplementary table 6. Results of the summary-based Mendelian randomization integrating whole-blood expression quantitative trait loci. For each disease-gene association, we report the strongest effect observed between GTEX and eQTLGen datasets.

A) Bonferroni-significant disease-gene association with HEIDI P > 0.05.

Disease	Gene	chr	gene_bp	topSNP	topSNP_bp	A1	A2	Freq	b_SMR	se_SMR	p_SMR	p_HEIDI
CD	DNMT3A	2	25510652	rs7578575	25488819	A	T	0.329	-0.834	0.164	3.51E-07	0.383
IBD	DNMT3A	2	25510652	rs7578575	25488819	A	T	0.329	-0.745	0.130	9.22E-09	0.515
UC	DNMT3A	2	25510652	rs7578575	25488819	A	T	0.329	-0.627	0.156	5.73E-05	0.541
UC	CFDP1	16	75327596	rs9934007	75390532	C	T	0.754	0.273	0.070	9.61E-05	0.973
CD	STAT3	17	40502964	rs1053004	40466092	G	A	0.380	0.702	0.128	4.40E-08	0.056
CD	IMPDH2	3	49064299	rs9813813	49207093	A	T	0.110	-1.233	0.294	2.74E-05	0.213
IBD	IMPDH2	3	49064299	rs9813813	49207093	A	T	0.110	-1.328	0.258	2.57E-07	0.071
UC	IMPDH2	3	49064299	rs9813813	49207093	A	T	0.110	-1.445	0.307	2.53E-06	0.050
IBD	UBE2L3	22	21941029	rs2070512	21949411	C	A	0.194	0.163	0.021	1.06E-14	0.169
UC	UBE2L3	22	21941029	rs2070512	21949411	C	A	0.194	0.116	0.027	1.76E-05	0.698
CD	BCL2L15	1	1.14E+08	rs1217393	1.14E+08	A	G	0.271	-0.705	0.170	3.39E-05	0.184
CD	STMN3	20	62271061	rs909333	62270637	G	T	0.207	0.547	0.102	8.31E-08	0.200
IBD	STMN3	20	62271061	rs909333	62270637	G	T	0.207	0.555	0.087	2.01E-10	0.055
UC	STMN3	20	62271061	rs909333	62270637	G	T	0.207	0.498	0.098	4.04E-07	0.062
GORD	PBX2	6	32154964	rs693906	31835164	C	G	0.113	0.154	0.036	1.65E-05	0.058
VHD	AGER	6	32150423	rs693906	31835164	C	G	0.113	0.212	0.052	4.82E-05	0.092

B) Nominally significant effect on VHD of Bonferroni-significant gastrointestinal loci.

Disease	Gene	chr	Probe_bp	topSNP	topSNP_bp	A1	A2	Freq	b_SMR	se_SMR	p_SMR	p_HEIDI
VHD	CFDP1	16	75397489	rs7201989	75471499	G	A	0.246	-0.056	0.018	0.001	0.119
VHD	STAT3	17	40502964	rs1053004	40466092	G	A	0.380	0.093	0.047	0.048	0.948
VHD	IMPDH2	3	49064299	rs34037363	48821036	T	C	0.114	-0.209	0.105	0.047	0.089
VHD	UBE2L3	22	21941029	rs2070512	21949411	C	A	0.194	-0.029	0.009	0.002	0.623
VHD	STMN3	20	62271061	rs909333	62270637	G	T	0.207	-0.076	0.036	0.036	0.093

C) Effect of AGER gene on VHD and gastrointestinal disorders.

Disease	Gene	chr	Probe_bp	topSNP	topSNP_bp	A1	A2	Freq	b_SMR	se_SMR	p_SMR	p_HEIDI
CD	AGER	6	32150423	rs693906	31835164	C	G	0.113	-0.312	0.120	0.009	6.16E-08
GORD	AGER	6	32150423	rs693906	31835164	C	G	0.113	-0.209	0.049	2.02E-05	0.022
IBD	AGER	6	32148745	rs204995	32154285	G	A	0.217	-0.252	0.108	0.020	1.60E-04
IBS	AGER	6	32150423	rs693906	31835164	C	G	0.113	-0.168	0.052	0.001	0.045
UC	AGER	6	32148745	rs204995	32154285	G	A	0.217	-0.870	0.185	2.59E-06	0.002
VHD	AGER	6	32150423	rs693906	31835164	C	G	0.113	0.212	0.052	4.82E-05	0.092

Supplementary table 6. Results of the summary-based Mendelian randomization integrating whole-blood expression quantitative trait loci. For each disease-gene association, we report the strongest effect observed between GTEX and eQTLGen datasets.

A) Bonferroni-significant disease-gene association with HEIDI P > 0.05.

Disease	Gene	chr	gene_bp	topSNP	topSNP_bp	A1	A2	Freq	b_SMR	se_SMR	p_SMR	p_HEIDI
CD	DNMT3A	2	25510652	rs7578575	25488819	A	T	0.329	-0.834	0.164	3.51E-07	0.383
IBD	DNMT3A	2	25510652	rs7578575	25488819	A	T	0.329	-0.745	0.130	9.22E-09	0.515
UC	DNMT3A	2	25510652	rs7578575	25488819	A	T	0.329	-0.627	0.156	5.73E-05	0.541
UC	CFDP1	16	75327596	rs9934007	75390532	C	T	0.754	0.273	0.070	9.61E-05	0.973
CD	STAT3	17	40502964	rs1053004	40466092	G	A	0.380	0.702	0.128	4.40E-08	0.056
CD	IMPDH2	3	49064299	rs9813813	49207093	A	T	0.110	-1.233	0.294	2.74E-05	0.213
IBD	IMPDH2	3	49064299	rs9813813	49207093	A	T	0.110	-1.328	0.258	2.57E-07	0.071
UC	IMPDH2	3	49064299	rs9813813	49207093	A	T	0.110	-1.445	0.307	2.53E-06	0.050
IBD	UBE2L3	22	21941029	rs2070512	21949411	C	A	0.194	0.163	0.021	1.06E-14	0.169
UC	UBE2L3	22	21941029	rs2070512	21949411	C	A	0.194	0.116	0.027	1.76E-05	0.698
CD	BCL2L15	1	1.14E+08	rs1217393	1.14E+08	A	G	0.271	-0.705	0.170	3.39E-05	0.184
CD	STMN3	20	62271061	rs909333	62270637	G	T	0.207	0.547	0.102	8.31E-08	0.200
IBD	STMN3	20	62271061	rs909333	62270637	G	T	0.207	0.555	0.087	2.01E-10	0.055
UC	STMN3	20	62271061	rs909333	62270637	G	T	0.207	0.498	0.098	4.04E-07	0.062
GORD	PBX2	6	32154964	rs693906	31835164	C	G	0.113	0.154	0.036	1.65E-05	0.058
VHD	AGER	6	32150423	rs693906	31835164	C	G	0.113	0.212	0.052	4.82E-05	0.092

B) Nominally significant effect on VHD of Bonferroni-significant gastrointestinal loci.

Disease	Gene	chr	Probe_bp	topSNP	topSNP_bp	A1	A2	Freq	b_SMR	se_SMR	p_SMR	p_HEIDI
VHD	CFDP1	16	75397489	rs7201989	75471499	G	A	0.246	-0.056	0.018	0.001	0.119
VHD	STAT3	17	40502964	rs1053004	40466092	G	A	0.380	0.093	0.047	0.048	0.948
VHD	IMPDH2	3	49064299	rs34037363	48821036	T	C	0.114	-0.209	0.105	0.047	0.089
VHD	UBE2L3	22	21941029	rs2070512	21949411	C	A	0.194	-0.029	0.009	0.002	0.623
VHD	STMN3	20	62271061	rs909333	62270637	G	T	0.207	-0.076	0.036	0.036	0.093

C) Effect of AGER gene on VHD and gastrointestinal disorders.

Disease	Gene	chr	Probe_bp	topSNP	topSNP_bp	A1	A2	Freq	b_SMR	se_SMR	p_SMR	p_HEIDI
CD	AGER	6	32150423	rs693906	31835164	C	G	0.113	-0.312	0.120	0.009	6.16E-08
GORD	AGER	6	32150423	rs693906	31835164	C	G	0.113	-0.209	0.049	2.02E-05	0.022
IBD	AGER	6	32148745	rs204995	32154285	G	A	0.217	-0.252	0.108	0.020	1.60E-04
IBS	AGER	6	32150423	rs693906	31835164	C	G	0.113	-0.168	0.052	0.001	0.045
UC	AGER	6	32148745	rs204995	32154285	G	A	0.217	-0.870	0.185	2.59E-06	0.002
VHD	AGER	6	32150423	rs693906	31835164	C	G	0.113	0.212	0.052	4.82E-05	0.092

Supplementary table 6. Results of the summary-based Mendelian randomization integrating whole-blood expression quantitative trait loci. For each disease-gene association, we report the strongest effect observed between GTEX and eQTLGen datasets.

A) Bonferroni-significant disease-gene association with HEIDI P > 0.05.

Disease	Gene	chr	gene_bp	topSNP	topSNP_bp	A1	A2	Freq	b_SMR	se_SMR	p_SMR	p_HEIDI
CD	DNMT3A	2	25510652	rs7578575	25488819	A	T	0.329	-0.834	0.164	3.51E-07	0.383
IBD	DNMT3A	2	25510652	rs7578575	25488819	A	T	0.329	-0.745	0.130	9.22E-09	0.515
UC	DNMT3A	2	25510652	rs7578575	25488819	A	T	0.329	-0.627	0.156	5.73E-05	0.541
UC	CFDP1	16	75327596	rs9934007	75390532	C	T	0.754	0.273	0.070	9.61E-05	0.973
CD	STAT3	17	40502964	rs1053004	40466092	G	A	0.380	0.702	0.128	4.40E-08	0.056
CD	IMPDH2	3	49064299	rs9813813	49207093	A	T	0.110	-1.233	0.294	2.74E-05	0.213
IBD	IMPDH2	3	49064299	rs9813813	49207093	A	T	0.110	-1.328	0.258	2.57E-07	0.071
UC	IMPDH2	3	49064299	rs9813813	49207093	A	T	0.110	-1.445	0.307	2.53E-06	0.050
IBD	UBE2L3	22	21941029	rs2070512	21949411	C	A	0.194	0.163	0.021	1.06E-14	0.169
UC	UBE2L3	22	21941029	rs2070512	21949411	C	A	0.194	0.116	0.027	1.76E-05	0.698
CD	BCL2L15	1	1.14E+08	rs1217393	1.14E+08	A	G	0.271	-0.705	0.170	3.39E-05	0.184
CD	STMN3	20	62271061	rs909333	62270637	G	T	0.207	0.547	0.102	8.31E-08	0.200
IBD	STMN3	20	62271061	rs909333	62270637	G	T	0.207	0.555	0.087	2.01E-10	0.055
UC	STMN3	20	62271061	rs909333	62270637	G	T	0.207	0.498	0.098	4.04E-07	0.062
GORD	PBX2	6	32154964	rs693906	31835164	C	G	0.113	0.154	0.036	1.65E-05	0.058
VHD	AGER	6	32150423	rs693906	31835164	C	G	0.113	0.212	0.052	4.82E-05	0.092

B) Nominally significant effect on VHD of Bonferroni-significant gastrointestinal loci.

Disease	Gene	chr	Probe_bp	topSNP	topSNP_bp	A1	A2	Freq	b_SMR	se_SMR	p_SMR	p_HEIDI
VHD	CFDP1	16	75397489	rs7201989	75471499	G	A	0.246	-0.056	0.018	0.001	0.119
VHD	STAT3	17	40502964	rs1053004	40466092	G	A	0.380	0.093	0.047	0.048	0.948
VHD	IMPDH2	3	49064299	rs34037363	48821036	T	C	0.114	-0.209	0.105	0.047	0.089
VHD	UBE2L3	22	21941029	rs2070512	21949411	C	A	0.194	-0.029	0.009	0.002	0.623
VHD	STMN3	20	62271061	rs909333	62270637	G	T	0.207	-0.076	0.036	0.036	0.093

C) Effect of AGER gene on VHD and gastrointestinal disorders.

Disease	Gene	chr	Probe_bp	topSNP	topSNP_bp	A1	A2	Freq	b_SMR	se_SMR	p_SMR	p_HEIDI
CD	AGER	6	32150423	rs693906	31835164	C	G	0.113	-0.312	0.120	0.009	6.16E-08
GORD	AGER	6	32150423	rs693906	31835164	C	G	0.113	-0.209	0.049	2.02E-05	0.022
IBD	AGER	6	32148745	rs204995	32154285	G	A	0.217	-0.252	0.108	0.020	1.60E-04
IBS	AGER	6	32150423	rs693906	31835164	C	G	0.113	-0.168	0.052	0.001	0.045
UC	AGER	6	32148745	rs204995	32154285	G	A	0.217	-0.870	0.185	2.59E-06	0.002
VHD	AGER	6	32150423	rs693906	31835164	C	G	0.113	0.212	0.052	4.82E-05	0.092

Supplementary table 6. Results of the summary-based Mendelian randomization integrating whole-blood expression quantitative trait loci. For each disease-gene association, we report the strongest effect observed between GTEX and eQTLGen datasets.

A) Bonferroni-significant disease-gene association with HEIDI P > 0.05.

Disease	Gene	chr	gene_bp	topSNP	topSNP_bp	A1	A2	Freq	b_SMR	se_SMR	p_SMR	p_HEIDI
CD	DNMT3A	2	25510652	rs7578575	25488819	A	T	0.329	-0.834	0.164	3.51E-07	0.383
IBD	DNMT3A	2	25510652	rs7578575	25488819	A	T	0.329	-0.745	0.130	9.22E-09	0.515
UC	DNMT3A	2	25510652	rs7578575	25488819	A	T	0.329	-0.627	0.156	5.73E-05	0.541
UC	CFDP1	16	75327596	rs9934007	75390532	C	T	0.754	0.273	0.070	9.61E-05	0.973
CD	STAT3	17	40502964	rs1053004	40466092	G	A	0.380	0.702	0.128	4.40E-08	0.056
CD	IMPDH2	3	49064299	rs9813813	49207093	A	T	0.110	-1.233	0.294	2.74E-05	0.213
IBD	IMPDH2	3	49064299	rs9813813	49207093	A	T	0.110	-1.328	0.258	2.57E-07	0.071
UC	IMPDH2	3	49064299	rs9813813	49207093	A	T	0.110	-1.445	0.307	2.53E-06	0.050
IBD	UBE2L3	22	21941029	rs2070512	21949411	C	A	0.194	0.163	0.021	1.06E-14	0.169
UC	UBE2L3	22	21941029	rs2070512	21949411	C	A	0.194	0.116	0.027	1.76E-05	0.698
CD	BCL2L15	1	1.14E+08	rs1217393	1.14E+08	A	G	0.271	-0.705	0.170	3.39E-05	0.184
CD	STMN3	20	62271061	rs909333	62270637	G	T	0.207	0.547	0.102	8.31E-08	0.200
IBD	STMN3	20	62271061	rs909333	62270637	G	T	0.207	0.555	0.087	2.01E-10	0.055
UC	STMN3	20	62271061	rs909333	62270637	G	T	0.207	0.498	0.098	4.04E-07	0.062
GORD	PBX2	6	32154964	rs693906	31835164	C	G	0.113	0.154	0.036	1.65E-05	0.058
VHD	AGER	6	32150423	rs693906	31835164	C	G	0.113	0.212	0.052	4.82E-05	0.092

B) Nominally significant effect on VHD of Bonferroni-significant gastrointestinal loci.

Disease	Gene	chr	Probe_bp	topSNP	topSNP_bp	A1	A2	Freq	b_SMR	se_SMR	p_SMR	p_HEIDI
VHD	CFDP1	16	75397489	rs7201989	75471499	G	A	0.246	-0.056	0.018	0.001	0.119
VHD	STAT3	17	40502964	rs1053004	40466092	G	A	0.380	0.093	0.047	0.048	0.948
VHD	IMPDH2	3	49064299	rs34037363	48821036	T	C	0.114	-0.209	0.105	0.047	0.089
VHD	UBE2L3	22	21941029	rs2070512	21949411	C	A	0.194	-0.029	0.009	0.002	0.623
VHD	STMN3	20	62271061	rs909333	62270637	G	T	0.207	-0.076	0.036	0.036	0.093

C) Effect of AGER gene on VHD and gastrointestinal disorders.

Disease	Gene	chr	Probe_bp	topSNP	topSNP_bp	A1	A2	Freq	b_SMR	se_SMR	p_SMR	p_HEIDI
CD	AGER	6	32150423	rs693906	31835164	C	G	0.113	-0.312	0.120	0.009	6.16E-08
GORD	AGER	6	32150423	rs693906	31835164	C	G	0.113	-0.209	0.049	2.02E-05	0.022
IBD	AGER	6	32148745	rs204995	32154285	G	A	0.217	-0.252	0.108	0.020	1.60E-04
IBS	AGER	6	32150423	rs693906	31835164	C	G	0.113	-0.168	0.052	0.001	0.045
UC	AGER	6	32148745	rs204995	32154285	G	A	0.217	-0.870	0.185	2.59E-06	0.002
VHD	AGER	6	32150423	rs693906	31835164	C	G	0.113	0.212	0.052	4.82E-05	0.092

Supplementary table 6. Results of the summary-based Mendelian randomization integrating whole-blood expression quantitative trait loci. For each disease-gene association, we report the strongest effect observed between GTEX and eQTLGen datasets.

A) Bonferroni-significant disease-gene association with HEIDI P > 0.05.

Disease	Gene	chr	gene_bp	topSNP	topSNP_bp	A1	A2	Freq	b_SMR	se_SMR	p_SMR	p_HEIDI
CD	DNMT3A	2	25510652	rs7578575	25488819	A	T	0.329	-0.834	0.164	3.51E-07	0.383
IBD	DNMT3A	2	25510652	rs7578575	25488819	A	T	0.329	-0.745	0.130	9.22E-09	0.515
UC	DNMT3A	2	25510652	rs7578575	25488819	A	T	0.329	-0.627	0.156	5.73E-05	0.541
UC	CFDP1	16	75327596	rs9934007	75390532	C	T	0.754	0.273	0.070	9.61E-05	0.973
CD	STAT3	17	40502964	rs1053004	40466092	G	A	0.380	0.702	0.128	4.40E-08	0.056
CD	IMPDH2	3	49064299	rs9813813	49207093	A	T	0.110	-1.233	0.294	2.74E-05	0.213
IBD	IMPDH2	3	49064299	rs9813813	49207093	A	T	0.110	-1.328	0.258	2.57E-07	0.071
UC	IMPDH2	3	49064299	rs9813813	49207093	A	T	0.110	-1.445	0.307	2.53E-06	0.050
IBD	UBE2L3	22	21941029	rs2070512	21949411	C	A	0.194	0.163	0.021	1.06E-14	0.169
UC	UBE2L3	22	21941029	rs2070512	21949411	C	A	0.194	0.116	0.027	1.76E-05	0.698
CD	BCL2L15	1	1.14E+08	rs1217393	1.14E+08	A	G	0.271	-0.705	0.170	3.39E-05	0.184
CD	STMN3	20	62271061	rs909333	62270637	G	T	0.207	0.547	0.102	8.31E-08	0.200
IBD	STMN3	20	62271061	rs909333	62270637	G	T	0.207	0.555	0.087	2.01E-10	0.055
UC	STMN3	20	62271061	rs909333	62270637	G	T	0.207	0.498	0.098	4.04E-07	0.062
GORD	PBX2	6	32154964	rs693906	31835164	C	G	0.113	0.154	0.036	1.65E-05	0.058
VHD	AGER	6	32150423	rs693906	31835164	C	G	0.113	0.212	0.052	4.82E-05	0.092

B) Nominally significant effect on VHD of Bonferroni-significant gastrointestinal loci.

Disease	Gene	chr	Probe_bp	topSNP	topSNP_bp	A1	A2	Freq	b_SMR	se_SMR	p_SMR	p_HEIDI
VHD	CFDP1	16	75397489	rs7201989	75471499	G	A	0.246	-0.056	0.018	0.001	0.119
VHD	STAT3	17	40502964	rs1053004	40466092	G	A	0.380	0.093	0.047	0.048	0.948
VHD	IMPDH2	3	49064299	rs34037363	48821036	T	C	0.114	-0.209	0.105	0.047	0.089
VHD	UBE2L3	22	21941029	rs2070512	21949411	C	A	0.194	-0.029	0.009	0.002	0.623
VHD	STMN3	20	62271061	rs909333	62270637	G	T	0.207	-0.076	0.036	0.036	0.093

C) Effect of AGER gene on VHD and gastrointestinal disorders.

Disease	Gene	chr	Probe_bp	topSNP	topSNP_bp	A1	A2	Freq	b_SMR	se_SMR	p_SMR	p_HEIDI
CD	AGER	6	32150423	rs693906	31835164	C	G	0.113	-0.312	0.120	0.009	6.16E-08
GORD	AGER	6	32150423	rs693906	31835164	C	G	0.113	-0.209	0.049	2.02E-05	0.022
IBD	AGER	6	32148745	rs204995	32154285	G	A	0.217	-0.252	0.108	0.020	1.60E-04
IBS	AGER	6	32150423	rs693906	31835164	C	G	0.113	-0.168	0.052	0.001	0.045
UC	AGER	6	32148745	rs204995	32154285	G	A	0.217	-0.870	0.185	2.59E-06	0.002
VHD	AGER	6	32150423	rs693906	31835164	C	G	0.113	0.212	0.052	4.82E-05	0.092

Supplementary table 6. Results of the summary-based Mendelian randomization integrating whole-blood expression quantitative trait loci. For each disease-gene association, we report the strongest effect observed between GTEX and eQTLGen datasets.

A) Bonferroni-significant disease-gene association with HEIDI P > 0.05.

Disease	Gene	chr	gene_bp	topSNP	topSNP_bp	A1	A2	Freq	b_SMR	se_SMR	p_SMR	p_HEIDI
CD	DNMT3A	2	25510652	rs7578575	25488819	A	T	0.329	-0.834	0.164	3.51E-07	0.383
IBD	DNMT3A	2	25510652	rs7578575	25488819	A	T	0.329	-0.745	0.130	9.22E-09	0.515
UC	DNMT3A	2	25510652	rs7578575	25488819	A	T	0.329	-0.627	0.156	5.73E-05	0.541
UC	CFDP1	16	75327596	rs9934007	75390532	C	T	0.754	0.273	0.070	9.61E-05	0.973
CD	STAT3	17	40502964	rs1053004	40466092	G	A	0.380	0.702	0.128	4.40E-08	0.056
CD	IMPDH2	3	49064299	rs9813813	49207093	A	T	0.110	-1.233	0.294	2.74E-05	0.213
IBD	IMPDH2	3	49064299	rs9813813	49207093	A	T	0.110	-1.328	0.258	2.57E-07	0.071
UC	IMPDH2	3	49064299	rs9813813	49207093	A	T	0.110	-1.445	0.307	2.53E-06	0.050
IBD	UBE2L3	22	21941029	rs2070512	21949411	C	A	0.194	0.163	0.021	1.06E-14	0.169
UC	UBE2L3	22	21941029	rs2070512	21949411	C	A	0.194	0.116	0.027	1.76E-05	0.698
CD	BCL2L15	1	1.14E+08	rs1217393	1.14E+08	A	G	0.271	-0.705	0.170	3.39E-05	0.184
CD	STMN3	20	62271061	rs909333	62270637	G	T	0.207	0.547	0.102	8.31E-08	0.200
IBD	STMN3	20	62271061	rs909333	62270637	G	T	0.207	0.555	0.087	2.01E-10	0.055
UC	STMN3	20	62271061	rs909333	62270637	G	T	0.207	0.498	0.098	4.04E-07	0.062
GORD	PBX2	6	32154964	rs693906	31835164	C	G	0.113	0.154	0.036	1.65E-05	0.058
VHD	AGER	6	32150423	rs693906	31835164	C	G	0.113	0.212	0.052	4.82E-05	0.092

B) Nominally significant effect on VHD of Bonferroni-significant gastrointestinal loci.

Disease	Gene	chr	Probe_bp	topSNP	topSNP_bp	A1	A2	Freq	b_SMR	se_SMR	p_SMR	p_HEIDI
VHD	CFDP1	16	75397489	rs7201989	75471499	G	A	0.246	-0.056	0.018	0.001	0.119
VHD	STAT3	17	40502964	rs1053004	40466092	G	A	0.380	0.093	0.047	0.048	0.948
VHD	IMPDH2	3	49064299	rs34037363	48821036	T	C	0.114	-0.209	0.105	0.047	0.089
VHD	UBE2L3	22	21941029	rs2070512	21949411	C	A	0.194	-0.029	0.009	0.002	0.623
VHD	STMN3	20	62271061	rs909333	62270637	G	T	0.207	-0.076	0.036	0.036	0.093

C) Effect of AGER gene on VHD and gastrointestinal disorders.

Disease	Gene	chr	Probe_bp	topSNP	topSNP_bp	A1	A2	Freq	b_SMR	se_SMR	p_SMR	p_HEIDI
CD	AGER	6	32150423	rs693906	31835164	C	G	0.113	-0.312	0.120	0.009	6.16E-08
GORD	AGER	6	32150423	rs693906	31835164	C	G	0.113	-0.209	0.049	2.02E-05	0.022
IBD	AGER	6	32148745	rs204995	32154285	G	A	0.217	-0.252	0.108	0.020	1.60E-04
IBS	AGER	6	32150423	rs693906	31835164	C	G	0.113	-0.168	0.052	0.001	0.045
UC	AGER	6	32148745	rs204995	32154285	G	A	0.217	-0.870	0.185	2.59E-06	0.002
VHD	AGER	6	32150423	rs693906	31835164	C	G	0.113	0.212	0.052	4.82E-05	0.092

Supplementary table 6. Results of the summary-based Mendelian randomization integrating whole-blood expression quantitative trait loci. For each disease-gene association, we report the strongest effect observed between GTEX and eQTLGen datasets.

A) Bonferroni-significant disease-gene association with HEIDI P > 0.05.

Disease	Gene	chr	gene_bp	topSNP	topSNP_bp	A1	A2	Freq	b_SMR	se_SMR	p_SMR	p_HEIDI
CD	DNMT3A	2	25510652	rs7578575	25488819	A	T	0.329	-0.834	0.164	3.51E-07	0.383
IBD	DNMT3A	2	25510652	rs7578575	25488819	A	T	0.329	-0.745	0.130	9.22E-09	0.515
UC	DNMT3A	2	25510652	rs7578575	25488819	A	T	0.329	-0.627	0.156	5.73E-05	0.541
UC	CFDP1	16	75327596	rs9934007	75390532	C	T	0.754	0.273	0.070	9.61E-05	0.973
CD	STAT3	17	40502964	rs1053004	40466092	G	A	0.380	0.702	0.128	4.40E-08	0.056
CD	IMPDH2	3	49064299	rs9813813	49207093	A	T	0.110	-1.233	0.294	2.74E-05	0.213
IBD	IMPDH2	3	49064299	rs9813813	49207093	A	T	0.110	-1.328	0.258	2.57E-07	0.071
UC	IMPDH2	3	49064299	rs9813813	49207093	A	T	0.110	-1.445	0.307	2.53E-06	0.050
IBD	UBE2L3	22	21941029	rs2070512	21949411	C	A	0.194	0.163	0.021	1.06E-14	0.169
UC	UBE2L3	22	21941029	rs2070512	21949411	C	A	0.194	0.116	0.027	1.76E-05	0.698
CD	BCL2L15	1	1.14E+08	rs1217393	1.14E+08	A	G	0.271	-0.705	0.170	3.39E-05	0.184
CD	STMN3	20	62271061	rs909333	62270637	G	T	0.207	0.547	0.102	8.31E-08	0.200
IBD	STMN3	20	62271061	rs909333	62270637	G	T	0.207	0.555	0.087	2.01E-10	0.055
UC	STMN3	20	62271061	rs909333	62270637	G	T	0.207	0.498	0.098	4.04E-07	0.062
GORD	PBX2	6	32154964	rs693906	31835164	C	G	0.113	0.154	0.036	1.65E-05	0.058
VHD	AGER	6	32150423	rs693906	31835164	C	G	0.113	0.212	0.052	4.82E-05	0.092

B) Nominally significant effect on VHD of Bonferroni-significant gastrointestinal loci.

Disease	Gene	chr	Probe_bp	topSNP	topSNP_bp	A1	A2	Freq	b_SMR	se_SMR	p_SMR	p_HEIDI
VHD	CFDP1	16	75397489	rs7201989	75471499	G	A	0.246	-0.056	0.018	0.001	0.119
VHD	STAT3	17	40502964	rs1053004	40466092	G	A	0.380	0.093	0.047	0.048	0.948
VHD	IMPDH2	3	49064299	rs34037363	48821036	T	C	0.114	-0.209	0.105	0.047	0.089
VHD	UBE2L3	22	21941029	rs2070512	21949411	C	A	0.194	-0.029	0.009	0.002	0.623
VHD	STMN3	20	62271061	rs909333	62270637	G	T	0.207	-0.076	0.036	0.036	0.093

C) Effect of AGER gene on VHD and gastrointestinal disorders.

Disease	Gene	chr	Probe_bp	topSNP	topSNP_bp	A1	A2	Freq	b_SMR	se_SMR	p_SMR	p_HEIDI
CD	AGER	6	32150423	rs693906	31835164	C	G	0.113	-0.312	0.120	0.009	6.16E-08
GORD	AGER	6	32150423	rs693906	31835164	C	G	0.113	-0.209	0.049	2.02E-05	0.022
IBD	AGER	6	32148745	rs204995	32154285	G	A	0.217	-0.252	0.108	0.020	1.60E-04
IBS	AGER	6	32150423	rs693906	31835164	C	G	0.113	-0.168	0.052	0.001	0.045
UC	AGER	6	32148745	rs204995	32154285	G	A	0.217	-0.870	0.185	2.59E-06	0.002
VHD	AGER	6	32150423	rs693906	31835164	C	G	0.113	0.212	0.052	4.82E-05	0.092

Supplementary table 6. Results of the summary-based Mendelian randomization integrating whole-blood expression quantitative trait loci. For each disease-gene association, we report the strongest effect observed between GTEX and eQTLGen datasets.

A) Bonferroni-significant disease-gene association with HEIDI P > 0.05.

Disease	Gene	chr	gene_bp	topSNP	topSNP_bp	A1	A2	Freq	b_SMR	se_SMR	p_SMR	p_HEIDI
CD	DNMT3A	2	25510652	rs7578575	25488819	A	T	0.329	-0.834	0.164	3.51E-07	0.383
IBD	DNMT3A	2	25510652	rs7578575	25488819	A	T	0.329	-0.745	0.130	9.22E-09	0.515
UC	DNMT3A	2	25510652	rs7578575	25488819	A	T	0.329	-0.627	0.156	5.73E-05	0.541
UC	CFDP1	16	75327596	rs9934007	75390532	C	T	0.754	0.273	0.070	9.61E-05	0.973
CD	STAT3	17	40502964	rs1053004	40466092	G	A	0.380	0.702	0.128	4.40E-08	0.056
CD	IMPDH2	3	49064299	rs9813813	49207093	A	T	0.110	-1.233	0.294	2.74E-05	0.213
IBD	IMPDH2	3	49064299	rs9813813	49207093	A	T	0.110	-1.328	0.258	2.57E-07	0.071
UC	IMPDH2	3	49064299	rs9813813	49207093	A	T	0.110	-1.445	0.307	2.53E-06	0.050
IBD	UBE2L3	22	21941029	rs2070512	21949411	C	A	0.194	0.163	0.021	1.06E-14	0.169
UC	UBE2L3	22	21941029	rs2070512	21949411	C	A	0.194	0.116	0.027	1.76E-05	0.698
CD	BCL2L15	1	1.14E+08	rs1217393	1.14E+08	A	G	0.271	-0.705	0.170	3.39E-05	0.184
CD	STMN3	20	62271061	rs909333	62270637	G	T	0.207	0.547	0.102	8.31E-08	0.200
IBD	STMN3	20	62271061	rs909333	62270637	G	T	0.207	0.555	0.087	2.01E-10	0.055
UC	STMN3	20	62271061	rs909333	62270637	G	T	0.207	0.498	0.098	4.04E-07	0.062
GORD	PBX2	6	32154964	rs693906	31835164	C	G	0.113	0.154	0.036	1.65E-05	0.058
VHD	AGER	6	32150423	rs693906	31835164	C	G	0.113	0.212	0.052	4.82E-05	0.092

B) Nominally significant effect on VHD of Bonferroni-significant gastrointestinal loci.

Disease	Gene	chr	Probe_bp	topSNP	topSNP_bp	A1	A2	Freq	b_SMR	se_SMR	p_SMR	p_HEIDI
VHD	CFDP1	16	75397489	rs7201989	75471499	G	A	0.246	-0.056	0.018	0.001	0.119
VHD	STAT3	17	40502964	rs1053004	40466092	G	A	0.380	0.093	0.047	0.048	0.948
VHD	IMPDH2	3	49064299	rs34037363	48821036	T	C	0.114	-0.209	0.105	0.047	0.089
VHD	UBE2L3	22	21941029	rs2070512	21949411	C	A	0.194	-0.029	0.009	0.002	0.623
VHD	STMN3	20	62271061	rs909333	62270637	G	T	0.207	-0.076	0.036	0.036	0.093

C) Effect of AGER gene on VHD and gastrointestinal disorders.

Disease	Gene	chr	Probe_bp	topSNP	topSNP_bp	A1	A2	Freq	b_SMR	se_SMR	p_SMR	p_HEIDI
CD	AGER	6	32150423	rs693906	31835164	C	G	0.113	-0.312	0.120	0.009	6.16E-08
GORD	AGER	6	32150423	rs693906	31835164	C	G	0.113	-0.209	0.049	2.02E-05	0.022
IBD	AGER	6	32148745	rs204995	32154285	G	A	0.217	-0.252	0.108	0.020	1.60E-04
IBS	AGER	6	32150423	rs693906	31835164	C	G	0.113	-0.168	0.052	0.001	0.045
UC	AGER	6	32148745	rs204995	32154285	G	A	0.217	-0.870	0.185	2.59E-06	0.002
VHD	AGER	6	32150423	rs693906	31835164	C	G	0.113	0.212	0.052	4.82E-05	0.092

Supplementary table 6. Results of the summary-based Mendelian randomization integrating whole-blood expression quantitative trait loci. For each disease-gene association, we report the strongest effect observed between GTEX and eQTLGen datasets.

A) Bonferroni-significant disease-gene association with HEIDI P > 0.05.

Disease	Gene	chr	gene_bp	topSNP	topSNP_bp	A1	A2	Freq	b_SMR	se_SMR	p_SMR	p_HEIDI
CD	DNMT3A	2	25510652	rs7578575	25488819	A	T	0.329	-0.834	0.164	3.51E-07	0.383
IBD	DNMT3A	2	25510652	rs7578575	25488819	A	T	0.329	-0.745	0.130	9.22E-09	0.515
UC	DNMT3A	2	25510652	rs7578575	25488819	A	T	0.329	-0.627	0.156	5.73E-05	0.541
UC	CFDP1	16	75327596	rs9934007	75390532	C	T	0.754	0.273	0.070	9.61E-05	0.973
CD	STAT3	17	40502964	rs1053004	40466092	G	A	0.380	0.702	0.128	4.40E-08	0.056
CD	IMPDH2	3	49064299	rs9813813	49207093	A	T	0.110	-1.233	0.294	2.74E-05	0.213
IBD	IMPDH2	3	49064299	rs9813813	49207093	A	T	0.110	-1.328	0.258	2.57E-07	0.071
UC	IMPDH2	3	49064299	rs9813813	49207093	A	T	0.110	-1.445	0.307	2.53E-06	0.050
IBD	UBE2L3	22	21941029	rs2070512	21949411	C	A	0.194	0.163	0.021	1.06E-14	0.169
UC	UBE2L3	22	21941029	rs2070512	21949411	C	A	0.194	0.116	0.027	1.76E-05	0.698
CD	BCL2L15	1	1.14E+08	rs1217393	1.14E+08	A	G	0.271	-0.705	0.170	3.39E-05	0.184
CD	STMN3	20	62271061	rs909333	62270637	G	T	0.207	0.547	0.102	8.31E-08	0.200
IBD	STMN3	20	62271061	rs909333	62270637	G	T	0.207	0.555	0.087	2.01E-10	0.055
UC	STMN3	20	62271061	rs909333	62270637	G	T	0.207	0.498	0.098	4.04E-07	0.062
GORD	PBX2	6	32154964	rs693906	31835164	C	G	0.113	0.154	0.036	1.65E-05	0.058
VHD	AGER	6	32150423	rs693906	31835164	C	G	0.113	0.212	0.052	4.82E-05	0.092

B) Nominally significant effect on VHD of Bonferroni-significant gastrointestinal loci.

Disease	Gene	chr	Probe_bp	topSNP	topSNP_bp	A1	A2	Freq	b_SMR	se_SMR	p_SMR	p_HEIDI
VHD	CFDP1	16	75397489	rs7201989	75471499	G	A	0.246	-0.056	0.018	0.001	0.119
VHD	STAT3	17	40502964	rs1053004	40466092	G	A	0.380	0.093	0.047	0.048	0.948
VHD	IMPDH2	3	49064299	rs34037363	48821036	T	C	0.114	-0.209	0.105	0.047	0.089
VHD	UBE2L3	22	21941029	rs2070512	21949411	C	A	0.194	-0.029	0.009	0.002	0.623
VHD	STMN3	20	62271061	rs909333	62270637	G	T	0.207	-0.076	0.036	0.036	0.093

C) Effect of AGER gene on VHD and gastrointestinal disorders.

Disease	Gene	chr	Probe_bp	topSNP	topSNP_bp	A1	A2	Freq	b_SMR	se_SMR	p_SMR	p_HEIDI
CD	AGER	6	32150423	rs693906	31835164	C	G	0.113	-0.312	0.120	0.009	6.16E-08
GORD	AGER	6	32150423	rs693906	31835164	C	G	0.113	-0.209	0.049	2.02E-05	0.022
IBD	AGER	6	32148745	rs204995	32154285	G	A	0.217	-0.252	0.108	0.020	1.60E-04
IBS	AGER	6	32150423	rs693906	31835164	C	G	0.113	-0.168	0.052	0.001	0.045
UC	AGER	6	32148745	rs204995	32154285	G	A	0.217	-0.870	0.185	2.59E-06	0.002
VHD	AGER	6	32150423	rs693906	31835164	C	G	0.113	0.212	0.052	4.82E-05	0.092

Supplementary table 6. Results of the summary-based Mendelian randomization integrating whole-blood expression quantitative trait loci. For each disease-gene association, we report the strongest effect observed between GTEX and eQTLGen datasets.

A) Bonferroni-significant disease-gene association with HEIDI P > 0.05.

Disease	Gene	chr	gene_bp	topSNP	topSNP_bp	A1	A2	Freq	b_SMR	se_SMR	p_SMR	p_HEIDI
CD	DNMT3A	2	25510652	rs7578575	25488819	A	T	0.329	-0.834	0.164	3.51E-07	0.383
IBD	DNMT3A	2	25510652	rs7578575	25488819	A	T	0.329	-0.745	0.130	9.22E-09	0.515
UC	DNMT3A	2	25510652	rs7578575	25488819	A	T	0.329	-0.627	0.156	5.73E-05	0.541
UC	CFDP1	16	75327596	rs9934007	75390532	C	T	0.754	0.273	0.070	9.61E-05	0.973
CD	STAT3	17	40502964	rs1053004	40466092	G	A	0.380	0.702	0.128	4.40E-08	0.056
CD	IMPDH2	3	49064299	rs9813813	49207093	A	T	0.110	-1.233	0.294	2.74E-05	0.213
IBD	IMPDH2	3	49064299	rs9813813	49207093	A	T	0.110	-1.328	0.258	2.57E-07	0.071
UC	IMPDH2	3	49064299	rs9813813	49207093	A	T	0.110	-1.445	0.307	2.53E-06	0.050
IBD	UBE2L3	22	21941029	rs2070512	21949411	C	A	0.194	0.163	0.021	1.06E-14	0.169
UC	UBE2L3	22	21941029	rs2070512	21949411	C	A	0.194	0.116	0.027	1.76E-05	0.698
CD	BCL2L15	1	1.14E+08	rs1217393	1.14E+08	A	G	0.271	-0.705	0.170	3.39E-05	0.184
CD	STMN3	20	62271061	rs909333	62270637	G	T	0.207	0.547	0.102	8.31E-08	0.200
IBD	STMN3	20	62271061	rs909333	62270637	G	T	0.207	0.555	0.087	2.01E-10	0.055
UC	STMN3	20	62271061	rs909333	62270637	G	T	0.207	0.498	0.098	4.04E-07	0.062
GORD	PBX2	6	32154964	rs693906	31835164	C	G	0.113	0.154	0.036	1.65E-05	0.058
VHD	AGER	6	32150423	rs693906	31835164	C	G	0.113	0.212	0.052	4.82E-05	0.092

B) Nominally significant effect on VHD of Bonferroni-significant gastrointestinal loci.

Disease	Gene	chr	Probe_bp	topSNP	topSNP_bp	A1	A2	Freq	b_SMR	se_SMR	p_SMR	p_HEIDI
VHD	CFDP1	16	75397489	rs7201989	75471499	G	A	0.246	-0.056	0.018	0.001	0.119
VHD	STAT3	17	40502964	rs1053004	40466092	G	A	0.380	0.093	0.047	0.048	0.948
VHD	IMPDH2	3	49064299	rs34037363	48821036	T	C	0.114	-0.209	0.105	0.047	0.089
VHD	UBE2L3	22	21941029	rs2070512	21949411	C	A	0.194	-0.029	0.009	0.002	0.623
VHD	STMN3	20	62271061	rs909333	62270637	G	T	0.207	-0.076	0.036	0.036	0.093

C) Effect of AGER gene on VHD and gastrointestinal disorders.

Disease	Gene	chr	Probe_bp	topSNP	topSNP_bp	A1	A2	Freq	b_SMR	se_SMR	p_SMR	p_HEIDI
CD	AGER	6	32150423	rs693906	31835164	C	G	0.113	-0.312	0.120	0.009	6.16E-08
GORD	AGER	6	32150423	rs693906	31835164	C	G	0.113	-0.209	0.049	2.02E-05	0.022
IBD	AGER	6	32148745	rs204995	32154285	G	A	0.217	-0.252	0.108	0.020	1.60E-04
IBS	AGER	6	32150423	rs693906	31835164	C	G	0.113	-0.168	0.052	0.001	0.045
UC	AGER	6	32148745	rs204995	32154285	G	A	0.217	-0.870	0.185	2.59E-06	0.002
VHD	AGER	6	32150423	rs693906	31835164	C	G	0.113	0.212	0.052	4.82E-05	0.092

Supplementary table 6. Results of the summary-based Mendelian randomization integrating whole-blood expression quantitative trait loci. For each disease-gene association, we report the strongest effect observed between GTEX and eQTLGen datasets.

A) Bonferroni-significant disease-gene association with HEIDI P > 0.05.

Disease	Gene	chr	gene_bp	topSNP	topSNP_bp	A1	A2	Freq	b_SMR	se_SMR	p_SMR	p_HEIDI
CD	DNMT3A	2	25510652	rs7578575	25488819	A	T	0.329	-0.834	0.164	3.51E-07	0.383
IBD	DNMT3A	2	25510652	rs7578575	25488819	A	T	0.329	-0.745	0.130	9.22E-09	0.515
UC	DNMT3A	2	25510652	rs7578575	25488819	A	T	0.329	-0.627	0.156	5.73E-05	0.541
UC	CFDP1	16	75327596	rs9934007	75390532	C	T	0.754	0.273	0.070	9.61E-05	0.973
CD	STAT3	17	40502964	rs1053004	40466092	G	A	0.380	0.702	0.128	4.40E-08	0.056
CD	IMPDH2	3	49064299	rs9813813	49207093	A	T	0.110	-1.233	0.294	2.74E-05	0.213
IBD	IMPDH2	3	49064299	rs9813813	49207093	A	T	0.110	-1.328	0.258	2.57E-07	0.071
UC	IMPDH2	3	49064299	rs9813813	49207093	A	T	0.110	-1.445	0.307	2.53E-06	0.050
IBD	UBE2L3	22	21941029	rs2070512	21949411	C	A	0.194	0.163	0.021	1.06E-14	0.169
UC	UBE2L3	22	21941029	rs2070512	21949411	C	A	0.194	0.116	0.027	1.76E-05	0.698
CD	BCL2L15	1	1.14E+08	rs1217393	1.14E+08	A	G	0.271	-0.705	0.170	3.39E-05	0.184
CD	STMN3	20	62271061	rs909333	62270637	G	T	0.207	0.547	0.102	8.31E-08	0.200
IBD	STMN3	20	62271061	rs909333	62270637	G	T	0.207	0.555	0.087	2.01E-10	0.055
UC	STMN3	20	62271061	rs909333	62270637	G	T	0.207	0.498	0.098	4.04E-07	0.062
GORD	PBX2	6	32154964	rs693906	31835164	C	G	0.113	0.154	0.036	1.65E-05	0.058
VHD	AGER	6	32150423	rs693906	31835164	C	G	0.113	0.212	0.052	4.82E-05	0.092

B) Nominally significant effect on VHD of Bonferroni-significant gastrointestinal loci.

Disease	Gene	chr	Probe_bp	topSNP	topSNP_bp	A1	A2	Freq	b_SMR	se_SMR	p_SMR	p_HEIDI
VHD	CFDP1	16	75397489	rs7201989	75471499	G	A	0.246	-0.056	0.018	0.001	0.119
VHD	STAT3	17	40502964	rs1053004	40466092	G	A	0.380	0.093	0.047	0.048	0.948
VHD	IMPDH2	3	49064299	rs34037363	48821036	T	C	0.114	-0.209	0.105	0.047	0.089
VHD	UBE2L3	22	21941029	rs2070512	21949411	C	A	0.194	-0.029	0.009	0.002	0.623
VHD	STMN3	20	62271061	rs909333	62270637	G	T	0.207	-0.076	0.036	0.036	0.093

C) Effect of AGER gene on VHD and gastrointestinal disorders.

Disease	Gene	chr	Probe_bp	topSNP	topSNP_bp	A1	A2	Freq	b_SMR	se_SMR	p_SMR	p_HEIDI
CD	AGER	6	32150423	rs693906	31835164	C	G	0.113	-0.312	0.120	0.009	6.16E-08
GORD	AGER	6	32150423	rs693906	31835164	C	G	0.113	-0.209	0.049	2.02E-05	0.022
IBD	AGER	6	32148745	rs204995	32154285	G	A	0.217	-0.252	0.108	0.020	1.60E-04
IBS	AGER	6	32150423	rs693906	31835164	C	G	0.113	-0.168	0.052	0.001	0.045
UC	AGER	6	32148745	rs204995	32154285	G	A	0.217	-0.870	0.185	2.59E-06	0.002
VHD	AGER	6	32150423	rs693906	31835164	C	G	0.113	0.212	0.052	4.82E-05	0.092

Supplementary table 6. Results of the summary-based Mendelian randomization integrating whole-blood expression quantitative trait loci. For each disease-gene association, we report the strongest effect observed between GTEX and eQTLGen datasets.

A) Bonferroni-significant disease-gene association with HEIDI P > 0.05.

Disease	Gene	chr	gene_bp	topSNP	topSNP_bp	A1	A2	Freq	b_SMR	se_SMR	p_SMR	p_HEIDI
CD	DNMT3A	2	25510652	rs7578575	25488819	A	T	0.329	-0.834	0.164	3.51E-07	0.383
IBD	DNMT3A	2	25510652	rs7578575	25488819	A	T	0.329	-0.745	0.130	9.22E-09	0.515
UC	DNMT3A	2	25510652	rs7578575	25488819	A	T	0.329	-0.627	0.156	5.73E-05	0.541
UC	CFDP1	16	75327596	rs9934007	75390532	C	T	0.754	0.273	0.070	9.61E-05	0.973
CD	STAT3	17	40502964	rs1053004	40466092	G	A	0.380	0.702	0.128	4.40E-08	0.056
CD	IMPDH2	3	49064299	rs9813813	49207093	A	T	0.110	-1.233	0.294	2.74E-05	0.213
IBD	IMPDH2	3	49064299	rs9813813	49207093	A	T	0.110	-1.328	0.258	2.57E-07	0.071
UC	IMPDH2	3	49064299	rs9813813	49207093	A	T	0.110	-1.445	0.307	2.53E-06	0.050
IBD	UBE2L3	22	21941029	rs2070512	21949411	C	A	0.194	0.163	0.021	1.06E-14	0.169
UC	UBE2L3	22	21941029	rs2070512	21949411	C	A	0.194	0.116	0.027	1.76E-05	0.698
CD	BCL2L15	1	1.14E+08	rs1217393	1.14E+08	A	G	0.271	-0.705	0.170	3.39E-05	0.184
CD	STMN3	20	62271061	rs909333	62270637	G	T	0.207	0.547	0.102	8.31E-08	0.200
IBD	STMN3	20	62271061	rs909333	62270637	G	T	0.207	0.555	0.087	2.01E-10	0.055
UC	STMN3	20	62271061	rs909333	62270637	G	T	0.207	0.498	0.098	4.04E-07	0.062
GORD	PBX2	6	32154964	rs693906	31835164	C	G	0.113	0.154	0.036	1.65E-05	0.058
VHD	AGER	6	32150423	rs693906	31835164	C	G	0.113	0.212	0.052	4.82E-05	0.092

B) Nominally significant effect on VHD of Bonferroni-significant gastrointestinal loci.

Disease	Gene	chr	Probe_bp	topSNP	topSNP_bp	A1	A2	Freq	b_SMR	se_SMR	p_SMR	p_HEIDI
VHD	CFDP1	16	75397489	rs7201989	75471499	G	A	0.246	-0.056	0.018	0.001	0.119
VHD	STAT3	17	40502964	rs1053004	40466092	G	A	0.380	0.093	0.047	0.048	0.948
VHD	IMPDH2	3	49064299	rs34037363	48821036	T	C	0.114	-0.209	0.105	0.047	0.089
VHD	UBE2L3	22	21941029	rs2070512	21949411	C	A	0.194	-0.029	0.009	0.002	0.623
VHD	STMN3	20	62271061	rs909333	62270637	G	T	0.207	-0.076	0.036	0.036	0.093

C) Effect of AGER gene on VHD and gastrointestinal disorders.

Disease	Gene	chr	Probe_bp	topSNP	topSNP_bp	A1	A2	Freq	b_SMR	se_SMR	p_SMR	p_HEIDI
CD	AGER	6	32150423	rs693906	31835164	C	G	0.113	-0.312	0.120	0.009	6.16E-08
GORD	AGER	6	32150423	rs693906	31835164	C	G	0.113	-0.209	0.049	2.02E-05	0.022
IBD	AGER	6	32148745	rs204995	32154285	G	A	0.217	-0.252	0.108	0.020	1.60E-04
IBS	AGER	6	32150423	rs693906	31835164	C	G	0.113	-0.168	0.052	0.001	0.045
UC	AGER	6	32148745	rs204995	32154285	G	A	0.217	-0.870	0.185	2.59E-06	0.002
VHD	AGER	6	32150423	rs693906	31835164	C	G	0.113	0.212	0.052	4.82E-05	0.092

Supplementary table 6. Results of the summary-based Mendelian randomization integrating whole-blood expression quantitative trait loci. For each disease-gene association, we report the strongest effect observed between GTEX and eQTLGen datasets.

A) Bonferroni-significant disease-gene association with HEIDI P > 0.05.

Disease	Gene	chr	gene_bp	topSNP	topSNP_bp	A1	A2	Freq	b_SMR	se_SMR	p_SMR	p_HEIDI
CD	DNMT3A	2	25510652	rs7578575	25488819	A	T	0.329	-0.834	0.164	3.51E-07	0.383
IBD	DNMT3A	2	25510652	rs7578575	25488819	A	T	0.329	-0.745	0.130	9.22E-09	0.515
UC	DNMT3A	2	25510652	rs7578575	25488819	A	T	0.329	-0.627	0.156	5.73E-05	0.541
UC	CFDP1	16	75327596	rs9934007	75390532	C	T	0.754	0.273	0.070	9.61E-05	0.973
CD	STAT3	17	40502964	rs1053004	40466092	G	A	0.380	0.702	0.128	4.40E-08	0.056
CD	IMPDH2	3	49064299	rs9813813	49207093	A	T	0.110	-1.233	0.294	2.74E-05	0.213
IBD	IMPDH2	3	49064299	rs9813813	49207093	A	T	0.110	-1.328	0.258	2.57E-07	0.071
UC	IMPDH2	3	49064299	rs9813813	49207093	A	T	0.110	-1.445	0.307	2.53E-06	0.050
IBD	UBE2L3	22	21941029	rs2070512	21949411	C	A	0.194	0.163	0.021	1.06E-14	0.169
UC	UBE2L3	22	21941029	rs2070512	21949411	C	A	0.194	0.116	0.027	1.76E-05	0.698
CD	BCL2L15	1	1.14E+08	rs1217393	1.14E+08	A	G	0.271	-0.705	0.170	3.39E-05	0.184
CD	STMN3	20	62271061	rs909333	62270637	G	T	0.207	0.547	0.102	8.31E-08	0.200
IBD	STMN3	20	62271061	rs909333	62270637	G	T	0.207	0.555	0.087	2.01E-10	0.055
UC	STMN3	20	62271061	rs909333	62270637	G	T	0.207	0.498	0.098	4.04E-07	0.062
GORD	PBX2	6	32154964	rs693906	31835164	C	G	0.113	0.154	0.036	1.65E-05	0.058
VHD	AGER	6	32150423	rs693906	31835164	C	G	0.113	0.212	0.052	4.82E-05	0.092

B) Nominally significant effect on VHD of Bonferroni-significant gastrointestinal loci.

Disease	Gene	chr	Probe_bp	topSNP	topSNP_bp	A1	A2	Freq	b_SMR	se_SMR	p_SMR	p_HEIDI
VHD	CFDP1	16	75397489	rs7201989	75471499	G	A	0.246	-0.056	0.018	0.001	0.119
VHD	STAT3	17	40502964	rs1053004	40466092	G	A	0.380	0.093	0.047	0.048	0.948
VHD	IMPDH2	3	49064299	rs34037363	48821036	T	C	0.114	-0.209	0.105	0.047	0.089
VHD	UBE2L3	22	21941029	rs2070512	21949411	C	A	0.194	-0.029	0.009	0.002	0.623
VHD	STMN3	20	62271061	rs909333	62270637	G	T	0.207	-0.076	0.036	0.036	0.093

C) Effect of AGER gene on VHD and gastrointestinal disorders.

Disease	Gene	chr	Probe_bp	topSNP	topSNP_bp	A1	A2	Freq	b_SMR	se_SMR	p_SMR	p_HEIDI
CD	AGER	6	32150423	rs693906	31835164	C	G	0.113	-0.312	0.120	0.009	6.16E-08
GORD	AGER	6	32150423	rs693906	31835164	C	G	0.113	-0.209	0.049	2.02E-05	0.022
IBD	AGER	6	32148745	rs204995	32154285	G	A	0.217	-0.252	0.108	0.020	1.60E-04
IBS	AGER	6	32150423	rs693906	31835164	C	G	0.113	-0.168	0.052	0.001	0.045
UC	AGER	6	32148745	rs204995	32154285	G	A	0.217	-0.870	0.185	2.59E-06	0.002
VHD	AGER	6	32150423	rs693906	31835164	C	G	0.113	0.212	0.052	4.82E-05	0.092

Supplementary table 6. Results of the summary-based Mendelian randomization integrating whole-blood expression quantitative trait loci. For each disease-gene association, we report the strongest effect observed between GTEX and eQTLGen datasets.

A) Bonferroni-significant disease-gene association with HEIDI P > 0.05.

Disease	Gene	chr	gene_bp	topSNP	topSNP_bp	A1	A2	Freq	b_SMR	se_SMR	p_SMR	p_HEIDI
CD	DNMT3A	2	25510652	rs7578575	25488819	A	T	0.329	-0.834	0.164	3.51E-07	0.383
IBD	DNMT3A	2	25510652	rs7578575	25488819	A	T	0.329	-0.745	0.130	9.22E-09	0.515
UC	DNMT3A	2	25510652	rs7578575	25488819	A	T	0.329	-0.627	0.156	5.73E-05	0.541
UC	CFDP1	16	75327596	rs9934007	75390532	C	T	0.754	0.273	0.070	9.61E-05	0.973
CD	STAT3	17	40502964	rs1053004	40466092	G	A	0.380	0.702	0.128	4.40E-08	0.056
CD	IMPDH2	3	49064299	rs9813813	49207093	A	T	0.110	-1.233	0.294	2.74E-05	0.213
IBD	IMPDH2	3	49064299	rs9813813	49207093	A	T	0.110	-1.328	0.258	2.57E-07	0.071
UC	IMPDH2	3	49064299	rs9813813	49207093	A	T	0.110	-1.445	0.307	2.53E-06	0.050
IBD	UBE2L3	22	21941029	rs2070512	21949411	C	A	0.194	0.163	0.021	1.06E-14	0.169
UC	UBE2L3	22	21941029	rs2070512	21949411	C	A	0.194	0.116	0.027	1.76E-05	0.698
CD	BCL2L15	1	1.14E+08	rs1217393	1.14E+08	A	G	0.271	-0.705	0.170	3.39E-05	0.184
CD	STMN3	20	62271061	rs909333	62270637	G	T	0.207	0.547	0.102	8.31E-08	0.200
IBD	STMN3	20	62271061	rs909333	62270637	G	T	0.207	0.555	0.087	2.01E-10	0.055
UC	STMN3	20	62271061	rs909333	62270637	G	T	0.207	0.498	0.098	4.04E-07	0.062
GORD	PBX2	6	32154964	rs693906	31835164	C	G	0.113	0.154	0.036	1.65E-05	0.058
VHD	AGER	6	32150423	rs693906	31835164	C	G	0.113	0.212	0.052	4.82E-05	0.092

B) Nominally significant effect on VHD of Bonferroni-significant gastrointestinal loci.

Disease	Gene	chr	Probe_bp	topSNP	topSNP_bp	A1	A2	Freq	b_SMR	se_SMR	p_SMR	p_HEIDI
VHD	CFDP1	16	75397489	rs7201989	75471499	G	A	0.246	-0.056	0.018	0.001	0.119
VHD	STAT3	17	40502964	rs1053004	40466092	G	A	0.380	0.093	0.047	0.048	0.948
VHD	IMPDH2	3	49064299	rs34037363	48821036	T	C	0.114	-0.209	0.105	0.047	0.089
VHD	UBE2L3	22	21941029	rs2070512	21949411	C	A	0.194	-0.029	0.009	0.002	0.623
VHD	STMN3	20	62271061	rs909333	62270637	G	T	0.207	-0.076	0.036	0.036	0.093

C) Effect of AGER gene on VHD and gastrointestinal disorders.

Disease	Gene	chr	Probe_bp	topSNP	topSNP_bp	A1	A2	Freq	b_SMR	se_SMR	p_SMR	p_HEIDI
CD	AGER	6	32150423	rs693906	31835164	C	G	0.113	-0.312	0.120	0.009	6.16E-08
GORD	AGER	6	32150423	rs693906	31835164	C	G	0.113	-0.209	0.049	2.02E-05	0.022
IBD	AGER	6	32148745	rs204995	32154285	G	A	0.217	-0.252	0.108	0.020	1.60E-04
IBS	AGER	6	32150423	rs693906	31835164	C	G	0.113	-0.168	0.052	0.001	0.045
UC	AGER	6	32148745	rs204995	32154285	G	A	0.217	-0.870	0.185	2.59E-06	0.002
VHD	AGER	6	32150423	rs693906	31835164	C	G	0.113	0.212	0.052	4.82E-05	0.092

Supplementary table 6. Results of the summary-based Mendelian randomization integrating whole-blood expression quantitative trait loci. For each disease-gene association, we report the strongest effect observed between GTEX and eQTLGen datasets.

A) Bonferroni-significant disease-gene association with HEIDI P > 0.05.

Disease	Gene	chr	gene_bp	topSNP	topSNP_bp	A1	A2	Freq	b_SMR	se_SMR	p_SMR	p_HEIDI
CD	DNMT3A	2	25510652	rs7578575	25488819	A	T	0.329	-0.834	0.164	3.51E-07	0.383
IBD	DNMT3A	2	25510652	rs7578575	25488819	A	T	0.329	-0.745	0.130	9.22E-09	0.515
UC	DNMT3A	2	25510652	rs7578575	25488819	A	T	0.329	-0.627	0.156	5.73E-05	0.541
UC	CFDP1	16	75327596	rs9934007	75390532	C	T	0.754	0.273	0.070	9.61E-05	0.973
CD	STAT3	17	40502964	rs1053004	40466092	G	A	0.380	0.702	0.128	4.40E-08	0.056
CD	IMPDH2	3	49064299	rs9813813	49207093	A	T	0.110	-1.233	0.294	2.74E-05	0.213
IBD	IMPDH2	3	49064299	rs9813813	49207093	A	T	0.110	-1.328	0.258	2.57E-07	0.071
UC	IMPDH2	3	49064299	rs9813813	49207093	A	T	0.110	-1.445	0.307	2.53E-06	0.050
IBD	UBE2L3	22	21941029	rs2070512	21949411	C	A	0.194	0.163	0.021	1.06E-14	0.169
UC	UBE2L3	22	21941029	rs2070512	21949411	C	A	0.194	0.116	0.027	1.76E-05	0.698
CD	BCL2L15	1	1.14E+08	rs1217393	1.14E+08	A	G	0.271	-0.705	0.170	3.39E-05	0.184
CD	STMN3	20	62271061	rs909333	62270637	G	T	0.207	0.547	0.102	8.31E-08	0.200
IBD	STMN3	20	62271061	rs909333	62270637	G	T	0.207	0.555	0.087	2.01E-10	0.055
UC	STMN3	20	62271061	rs909333	62270637	G	T	0.207	0.498	0.098	4.04E-07	0.062
GORD	PBX2	6	32154964	rs693906	31835164	C	G	0.113	0.154	0.036	1.65E-05	0.058
VHD	AGER	6	32150423	rs693906	31835164	C	G	0.113	0.212	0.052	4.82E-05	0.092

B) Nominally significant effect on VHD of Bonferroni-significant gastrointestinal loci.

Disease	Gene	chr	Probe_bp	topSNP	topSNP_bp	A1	A2	Freq	b_SMR	se_SMR	p_SMR	p_HEIDI
VHD	CFDP1	16	75397489	rs7201989	75471499	G	A	0.246	-0.056	0.018	0.001	0.119
VHD	STAT3	17	40502964	rs1053004	40466092	G	A	0.380	0.093	0.047	0.048	0.948
VHD	IMPDH2	3	49064299	rs34037363	48821036	T	C	0.114	-0.209	0.105	0.047	0.089
VHD	UBE2L3	22	21941029	rs2070512	21949411	C	A	0.194	-0.029	0.009	0.002	0.623
VHD	STMN3	20	62271061	rs909333	62270637	G	T	0.207	-0.076	0.036	0.036	0.093

C) Effect of AGER gene on VHD and gastrointestinal disorders.

Disease	Gene	chr	Probe_bp	topSNP	topSNP_bp	A1	A2	Freq	b_SMR	se_SMR	p_SMR	p_HEIDI
CD	AGER	6	32150423	rs693906	31835164	C	G	0.113	-0.312	0.120	0.009	6.16E-08
GORD	AGER	6	32150423	rs693906	31835164	C	G	0.113	-0.209	0.049	2.02E-05	0.022
IBD	AGER	6	32148745	rs204995	32154285	G	A	0.217	-0.252	0.108	0.020	1.60E-04
IBS	AGER	6	32150423	rs693906	31835164	C	G	0.113	-0.168	0.052	0.001	0.045
UC	AGER	6	32148745	rs204995	32154285	G	A	0.217	-0.870	0.185	2.59E-06	0.002
VHD	AGER	6	32150423	rs693906	31835164	C	G	0.113	0.212	0.052	4.82E-05	0.092

Supplementary table 6. Results of the summary-based Mendelian randomization integrating whole-blood expression quantitative trait loci. For each disease-gene association, we report the strongest effect observed between GTEX and eQTLGen datasets.

A) Bonferroni-significant disease-gene association with HEIDI P > 0.05.

Disease	Gene	chr	gene_bp	topSNP	topSNP_bp	A1	A2	Freq	b_SMR	se_SMR	p_SMR	p_HEIDI
CD	DNMT3A	2	25510652	rs7578575	25488819	A	T	0.329	-0.834	0.164	3.51E-07	0.383
IBD	DNMT3A	2	25510652	rs7578575	25488819	A	T	0.329	-0.745	0.130	9.22E-09	0.515
UC	DNMT3A	2	25510652	rs7578575	25488819	A	T	0.329	-0.627	0.156	5.73E-05	0.541
UC	CFDP1	16	75327596	rs9934007	75390532	C	T	0.754	0.273	0.070	9.61E-05	0.973
CD	STAT3	17	40502964	rs1053004	40466092	G	A	0.380	0.702	0.128	4.40E-08	0.056
CD	IMPDH2	3	49064299	rs9813813	49207093	A	T	0.110	-1.233	0.294	2.74E-05	0.213
IBD	IMPDH2	3	49064299	rs9813813	49207093	A	T	0.110	-1.328	0.258	2.57E-07	0.071
UC	IMPDH2	3	49064299	rs9813813	49207093	A	T	0.110	-1.445	0.307	2.53E-06	0.050
IBD	UBE2L3	22	21941029	rs2070512	21949411	C	A	0.194	0.163	0.021	1.06E-14	0.169
UC	UBE2L3	22	21941029	rs2070512	21949411	C	A	0.194	0.116	0.027	1.76E-05	0.698
CD	BCL2L15	1	1.14E+08	rs1217393	1.14E+08	A	G	0.271	-0.705	0.170	3.39E-05	0.184
CD	STMN3	20	62271061	rs909333	62270637	G	T	0.207	0.547	0.102	8.31E-08	0.200
IBD	STMN3	20	62271061	rs909333	62270637	G	T	0.207	0.555	0.087	2.01E-10	0.055
UC	STMN3	20	62271061	rs909333	62270637	G	T	0.207	0.498	0.098	4.04E-07	0.062
GORD	PBX2	6	32154964	rs693906	31835164	C	G	0.113	0.154	0.036	1.65E-05	0.058
VHD	AGER	6	32150423	rs693906	31835164	C	G	0.113	0.212	0.052	4.82E-05	0.092

B) Nominally significant effect on VHD of Bonferroni-significant gastrointestinal loci.

Disease	Gene	chr	Probe_bp	topSNP	topSNP_bp	A1	A2	Freq	b_SMR	se_SMR	p_SMR	p_HEIDI
VHD	CFDP1	16	75397489	rs7201989	75471499	G	A	0.246	-0.056	0.018	0.001	0.119
VHD	STAT3	17	40502964	rs1053004	40466092	G	A	0.380	0.093	0.047	0.048	0.948
VHD	IMPDH2	3	49064299	rs34037363	48821036	T	C	0.114	-0.209	0.105	0.047	0.089
VHD	UBE2L3	22	21941029	rs2070512	21949411	C	A	0.194	-0.029	0.009	0.002	0.623
VHD	STMN3	20	62271061	rs909333	62270637	G	T	0.207	-0.076	0.036	0.036	0.093

C) Effect of AGER gene on VHD and gastrointestinal disorders.

Disease	Gene	chr	Probe_bp	topSNP	topSNP_bp	A1	A2	Freq	b_SMR	se_SMR	p_SMR	p_HEIDI
CD	AGER	6	32150423	rs693906	31835164	C	G	0.113	-0.312	0.120	0.009	6.16E-08
GORD	AGER	6	32150423	rs693906	31835164	C	G	0.113	-0.209	0.049	2.02E-05	0.022
IBD	AGER	6	32148745	rs204995	32154285	G	A	0.217	-0.252	0.108	0.020	1.60E-04
IBS	AGER	6	32150423	rs693906	31835164	C	G	0.113	-0.168	0.052	0.001	0.045
UC	AGER	6	32148745	rs204995	32154285	G	A	0.217	-0.870	0.185	2.59E-06	0.002
VHD	AGER	6	32150423	rs693906	31835164	C	G	0.113	0.212	0.052	4.82E-05	0.092

Supplementary table 6. Results of the summary-based Mendelian randomization integrating whole-blood expression quantitative trait loci. For each disease-gene association, we report the strongest effect observed between GTEX and eQTLGen datasets.

A) Bonferroni-significant disease-gene association with HEIDI P > 0.05.

Disease	Gene	chr	gene_bp	topSNP	topSNP_bp	A1	A2	Freq	b_SMR	se_SMR	p_SMR	p_HEIDI
CD	DNMT3A	2	25510652	rs7578575	25488819	A	T	0.329	-0.834	0.164	3.51E-07	0.383
IBD	DNMT3A	2	25510652	rs7578575	25488819	A	T	0.329	-0.745	0.130	9.22E-09	0.515
UC	DNMT3A	2	25510652	rs7578575	25488819	A	T	0.329	-0.627	0.156	5.73E-05	0.541
UC	CFDP1	16	75327596	rs9934007	75390532	C	T	0.754	0.273	0.070	9.61E-05	0.973
CD	STAT3	17	40502964	rs1053004	40466092	G	A	0.380	0.702	0.128	4.40E-08	0.056
CD	IMPDH2	3	49064299	rs9813813	49207093	A	T	0.110	-1.233	0.294	2.74E-05	0.213
IBD	IMPDH2	3	49064299	rs9813813	49207093	A	T	0.110	-1.328	0.258	2.57E-07	0.071
UC	IMPDH2	3	49064299	rs9813813	49207093	A	T	0.110	-1.445	0.307	2.53E-06	0.050
IBD	UBE2L3	22	21941029	rs2070512	21949411	C	A	0.194	0.163	0.021	1.06E-14	0.169
UC	UBE2L3	22	21941029	rs2070512	21949411	C	A	0.194	0.116	0.027	1.76E-05	0.698
CD	BCL2L15	1	1.14E+08	rs1217393	1.14E+08	A	G	0.271	-0.705	0.170	3.39E-05	0.184
CD	STMN3	20	62271061	rs909333	62270637	G	T	0.207	0.547	0.102	8.31E-08	0.200
IBD	STMN3	20	62271061	rs909333	62270637	G	T	0.207	0.555	0.087	2.01E-10	0.055
UC	STMN3	20	62271061	rs909333	62270637	G	T	0.207	0.498	0.098	4.04E-07	0.062
GORD	PBX2	6	32154964	rs693906	31835164	C	G	0.113	0.154	0.036	1.65E-05	0.058
VHD	AGER	6	32150423	rs693906	31835164	C	G	0.113	0.212	0.052	4.82E-05	0.092

B) Nominally significant effect on VHD of Bonferroni-significant gastrointestinal loci.

Disease	Gene	chr	Probe_bp	topSNP	topSNP_bp	A1	A2	Freq	b_SMR	se_SMR	p_SMR	p_HEIDI
VHD	CFDP1	16	75397489	rs7201989	75471499	G	A	0.246	-0.056	0.018	0.001	0.119
VHD	STAT3	17	40502964	rs1053004	40466092	G	A	0.380	0.093	0.047	0.048	0.948
VHD	IMPDH2	3	49064299	rs34037363	48821036	T	C	0.114	-0.209	0.105	0.047	0.089
VHD	UBE2L3	22	21941029	rs2070512	21949411	C	A	0.194	-0.029	0.009	0.002	0.623
VHD	STMN3	20	62271061	rs909333	62270637	G	T	0.207	-0.076	0.036	0.036	0.093

C) Effect of AGER gene on VHD and gastrointestinal disorders.

Disease	Gene	chr	Probe_bp	topSNP	topSNP_bp	A1	A2	Freq	b_SMR	se_SMR	p_SMR	p_HEIDI
CD	AGER	6	32150423	rs693906	31835164	C	G	0.113	-0.312	0.120	0.009	6.16E-08
GORD	AGER	6	32150423	rs693906	31835164	C	G	0.113	-0.209	0.049	2.02E-05	0.022
IBD	AGER	6	32148745	rs204995	32154285	G	A	0.217	-0.252	0.108	0.020	1.60E-04
IBS	AGER	6	32150423	rs693906	31835164	C	G	0.113	-0.168	0.052	0.001	0.045
UC	AGER	6	32148745	rs204995	32154285	G	A	0.217	-0.870	0.185	2.59E-06	0.002
VHD	AGER	6	32150423	rs693906	31835164	C	G	0.113	0.212	0.052	4.82E-05	0.092

Supplementary table 6. Results of the summary-based Mendelian randomization integrating whole-blood expression quantitative trait loci. For each disease-gene association, we report the strongest effect observed between GTEX and eQTLGen datasets.

A) Bonferroni-significant disease-gene association with HEIDI P > 0.05.

Disease	Gene	chr	gene_bp	topSNP	topSNP_bp	A1	A2	Freq	b_SMR	se_SMR	p_SMR	p_HEIDI
CD	DNMT3A	2	25510652	rs7578575	25488819	A	T	0.329	-0.834	0.164	3.51E-07	0.383
IBD	DNMT3A	2	25510652	rs7578575	25488819	A	T	0.329	-0.745	0.130	9.22E-09	0.515
UC	DNMT3A	2	25510652	rs7578575	25488819	A	T	0.329	-0.627	0.156	5.73E-05	0.541
UC	CFDP1	16	75327596	rs9934007	75390532	C	T	0.754	0.273	0.070	9.61E-05	0.973
CD	STAT3	17	40502964	rs1053004	40466092	G	A	0.380	0.702	0.128	4.40E-08	0.056
CD	IMPDH2	3	49064299	rs9813813	49207093	A	T	0.110	-1.233	0.294	2.74E-05	0.213
IBD	IMPDH2	3	49064299	rs9813813	49207093	A	T	0.110	-1.328	0.258	2.57E-07	0.071
UC	IMPDH2	3	49064299	rs9813813	49207093	A	T	0.110	-1.445	0.307	2.53E-06	0.050
IBD	UBE2L3	22	21941029	rs2070512	21949411	C	A	0.194	0.163	0.021	1.06E-14	0.169
UC	UBE2L3	22	21941029	rs2070512	21949411	C	A	0.194	0.116	0.027	1.76E-05	0.698
CD	BCL2L15	1	1.14E+08	rs1217393	1.14E+08	A	G	0.271	-0.705	0.170	3.39E-05	0.184
CD	STMN3	20	62271061	rs909333	62270637	G	T	0.207	0.547	0.102	8.31E-08	0.200
IBD	STMN3	20	62271061	rs909333	62270637	G	T	0.207	0.555	0.087	2.01E-10	0.055
UC	STMN3	20	62271061	rs909333	62270637	G	T	0.207	0.498	0.098	4.04E-07	0.062
GORD	PBX2	6	32154964	rs693906	31835164	C	G	0.113	0.154	0.036	1.65E-05	0.058
VHD	AGER	6	32150423	rs693906	31835164	C	G	0.113	0.212	0.052	4.82E-05	0.092

B) Nominally significant effect on VHD of Bonferroni-significant gastrointestinal loci.

Disease	Gene	chr	Probe_bp	topSNP	topSNP_bp	A1	A2	Freq	b_SMR	se_SMR	p_SMR	p_HEIDI
VHD	CFDP1	16	75397489	rs7201989	75471499	G	A	0.246	-0.056	0.018	0.001	0.119
VHD	STAT3	17	40502964	rs1053004	40466092	G	A	0.380	0.093	0.047	0.048	0.948
VHD	IMPDH2	3	49064299	rs34037363	48821036	T	C	0.114	-0.209	0.105	0.047	0.089
VHD	UBE2L3	22	21941029	rs2070512	21949411	C	A	0.194	-0.029	0.009	0.002	0.623
VHD	STMN3	20	62271061	rs909333	62270637	G	T	0.207	-0.076	0.036	0.036	0.093

C) Effect of AGER gene on VHD and gastrointestinal disorders.

Disease	Gene	chr	Probe_bp	topSNP	topSNP_bp	A1	A2	Freq	b_SMR	se_SMR	p_SMR	p_HEIDI
CD	AGER	6	32150423	rs693906	31835164	C	G	0.113	-0.312	0.120	0.009	6.16E-08
GORD	AGER	6	32150423	rs693906	31835164	C	G	0.113	-0.209	0.049	2.02E-05	0.022
IBD	AGER	6	32148745	rs204995	32154285	G	A	0.217	-0.252	0.108	0.020	1.60E-04
IBS	AGER	6	32150423	rs693906	31835164	C	G	0.113	-0.168	0.052	0.001	0.045
UC	AGER	6	32148745	rs204995	32154285	G	A	0.217	-0.870	0.185	2.59E-06	0.002
VHD	AGER	6	32150423	rs693906	31835164	C	G	0.113	0.212	0.052	4.82E-05	0.092

Supplementary table 6. Results of the summary-based Mendelian randomization integrating whole-blood expression quantitative trait loci. For each disease-gene association, we report the strongest effect observed between GTEX and eQTLGen datasets.

A) Bonferroni-significant disease-gene association with HEIDI P > 0.05.

Disease	Gene	chr	gene_bp	topSNP	topSNP_bp	A1	A2	Freq	b_SMR	se_SMR	p_SMR	p_HEIDI
CD	DNMT3A	2	25510652	rs7578575	25488819	A	T	0.329	-0.834	0.164	3.51E-07	0.383
IBD	DNMT3A	2	25510652	rs7578575	25488819	A	T	0.329	-0.745	0.130	9.22E-09	0.515
UC	DNMT3A	2	25510652	rs7578575	25488819	A	T	0.329	-0.627	0.156	5.73E-05	0.541
UC	CFDP1	16	75327596	rs9934007	75390532	C	T	0.754	0.273	0.070	9.61E-05	0.973
CD	STAT3	17	40502964	rs1053004	40466092	G	A	0.380	0.702	0.128	4.40E-08	0.056
CD	IMPDH2	3	49064299	rs9813813	49207093	A	T	0.110	-1.233	0.294	2.74E-05	0.213
IBD	IMPDH2	3	49064299	rs9813813	49207093	A	T	0.110	-1.328	0.258	2.57E-07	0.071
UC	IMPDH2	3	49064299	rs9813813	49207093	A	T	0.110	-1.445	0.307	2.53E-06	0.050
IBD	UBE2L3	22	21941029	rs2070512	21949411	C	A	0.194	0.163	0.021	1.06E-14	0.169
UC	UBE2L3	22	21941029	rs2070512	21949411	C	A	0.194	0.116	0.027	1.76E-05	0.698
CD	BCL2L15	1	1.14E+08	rs1217393	1.14E+08	A	G	0.271	-0.705	0.170	3.39E-05	0.184
CD	STMN3	20	62271061	rs909333	62270637	G	T	0.207	0.547	0.102	8.31E-08	0.200
IBD	STMN3	20	62271061	rs909333	62270637	G	T	0.207	0.555	0.087	2.01E-10	0.055
UC	STMN3	20	62271061	rs909333	62270637	G	T	0.207	0.498	0.098	4.04E-07	0.062
GORD	PBX2	6	32154964	rs693906	31835164	C	G	0.113	0.154	0.036	1.65E-05	0.058
VHD	AGER	6	32150423	rs693906	31835164	C	G	0.113	0.212	0.052	4.82E-05	0.092

B) Nominally significant effect on VHD of Bonferroni-significant gastrointestinal loci.

Disease	Gene	chr	Probe_bp	topSNP	topSNP_bp	A1	A2	Freq	b_SMR	se_SMR	p_SMR	p_HEIDI
VHD	CFDP1	16	75397489	rs7201989	75471499	G	A	0.246	-0.056	0.018	0.001	0.119
VHD	STAT3	17	40502964	rs1053004	40466092	G	A	0.380	0.093	0.047	0.048	0.948
VHD	IMPDH2	3	49064299	rs34037363	48821036	T	C	0.114	-0.209	0.105	0.047	0.089
VHD	UBE2L3	22	21941029	rs2070512	21949411	C	A	0.194	-0.029	0.009	0.002	0.623
VHD	STMN3	20	62271061	rs909333	62270637	G	T	0.207	-0.076	0.036	0.036	0.093

C) Effect of AGER gene on VHD and gastrointestinal disorders.

Disease	Gene	chr	Probe_bp	topSNP	topSNP_bp	A1	A2	Freq	b_SMR	se_SMR	p_SMR	p_HEIDI
CD	AGER	6	32150423	rs693906	31835164	C	G	0.113	-0.312	0.120	0.009	6.16E-08
GORD	AGER	6	32150423	rs693906	31835164	C	G	0.113	-0.209	0.049	2.02E-05	0.022
IBD	AGER	6	32148745	rs204995	32154285	G	A	0.217	-0.252	0.108	0.020	1.60E-04
IBS	AGER	6	32150423	rs693906	31835164	C	G	0.113	-0.168	0.052	0.001	0.045
UC	AGER	6	32148745	rs204995	32154285	G	A	0.217	-0.870	0.185	2.59E-06	0.002
VHD	AGER	6	32150423	rs693906	31835164	C	G	0.113	0.212	0.052	4.82E-05	0.092

Supplementary table 6. Results of the summary-based Mendelian randomization integrating whole-blood expression quantitative trait loci. For each disease-gene association, we report the strongest effect observed between GTEX and eQTLGen datasets.

A) Bonferroni-significant disease-gene association with HEIDI P > 0.05.

Disease	Gene	chr	gene_bp	topSNP	topSNP_bp	A1	A2	Freq	b_SMR	se_SMR	p_SMR	p_HEIDI
CD	DNMT3A	2	25510652	rs7578575	25488819	A	T	0.329	-0.834	0.164	3.51E-07	0.383
IBD	DNMT3A	2	25510652	rs7578575	25488819	A	T	0.329	-0.745	0.130	9.22E-09	0.515
UC	DNMT3A	2	25510652	rs7578575	25488819	A	T	0.329	-0.627	0.156	5.73E-05	0.541
UC	CFDP1	16	75327596	rs9934007	75390532	C	T	0.754	0.273	0.070	9.61E-05	0.973
CD	STAT3	17	40502964	rs1053004	40466092	G	A	0.380	0.702	0.128	4.40E-08	0.056
CD	IMPDH2	3	49064299	rs9813813	49207093	A	T	0.110	-1.233	0.294	2.74E-05	0.213
IBD	IMPDH2	3	49064299	rs9813813	49207093	A	T	0.110	-1.328	0.258	2.57E-07	0.071
UC	IMPDH2	3	49064299	rs9813813	49207093	A	T	0.110	-1.445	0.307	2.53E-06	0.050
IBD	UBE2L3	22	21941029	rs2070512	21949411	C	A	0.194	0.163	0.021	1.06E-14	0.169
UC	UBE2L3	22	21941029	rs2070512	21949411	C	A	0.194	0.116	0.027	1.76E-05	0.698
CD	BCL2L15	1	1.14E+08	rs1217393	1.14E+08	A	G	0.271	-0.705	0.170	3.39E-05	0.184
CD	STMN3	20	62271061	rs909333	62270637	G	T	0.207	0.547	0.102	8.31E-08	0.200
IBD	STMN3	20	62271061	rs909333	62270637	G	T	0.207	0.555	0.087	2.01E-10	0.055
UC	STMN3	20	62271061	rs909333	62270637	G	T	0.207	0.498	0.098	4.04E-07	0.062
GORD	PBX2	6	32154964	rs693906	31835164	C	G	0.113	0.154	0.036	1.65E-05	0.058
VHD	AGER	6	32150423	rs693906	31835164	C	G	0.113	0.212	0.052	4.82E-05	0.092

B) Nominally significant effect on VHD of Bonferroni-significant gastrointestinal loci.

Disease	Gene	chr	Probe_bp	topSNP	topSNP_bp	A1	A2	Freq	b_SMR	se_SMR	p_SMR	p_HEIDI
VHD	CFDP1	16	75397489	rs7201989	75471499	G	A	0.246	-0.056	0.018	0.001	0.119
VHD	STAT3	17	40502964	rs1053004	40466092	G	A	0.380	0.093	0.047	0.048	0.948
VHD	IMPDH2	3	49064299	rs34037363	48821036	T	C	0.114	-0.209	0.105	0.047	0.089
VHD	UBE2L3	22	21941029	rs2070512	21949411	C	A	0.194	-0.029	0.009	0.002	0.623
VHD	STMN3	20	62271061	rs909333	62270637	G	T	0.207	-0.076	0.036	0.036	0.093

C) Effect of AGER gene on VHD and gastrointestinal disorders.

Disease	Gene	chr	Probe_bp	topSNP	topSNP_bp	A1	A2	Freq	b_SMR	se_SMR	p_SMR	p_HEIDI
CD	AGER	6	32150423	rs693906	31835164	C	G	0.113	-0.312	0.120	0.009	6.16E-08
GORD	AGER	6	32150423	rs693906	31835164	C	G	0.113	-0.209	0.049	2.02E-05	0.022
IBD	AGER	6	32148745	rs204995	32154285	G	A	0.217	-0.252	0.108	0.020	1.60E-04
IBS	AGER	6	32150423	rs693906	31835164	C	G	0.113	-0.168	0.052	0.001	0.045
UC	AGER	6	32148745	rs204995	32154285	G	A	0.217	-0.870	0.185	2.59E-06	0.002
VHD	AGER	6	32150423	rs693906	31835164	C	G	0.113	0.212	0.052	4.82E-05	0.092

Supplementary table 6. Results of the summary-based Mendelian randomization integrating whole-blood expression quantitative trait loci. For each disease-gene association, we report the strongest effect observed between GTEX and eQTLGen datasets.

A) Bonferroni-significant disease-gene association with HEIDI P > 0.05.

Disease	Gene	chr	gene_bp	topSNP	topSNP_bp	A1	A2	Freq	b_SMR	se_SMR	p_SMR	p_HEIDI
CD	DNMT3A	2	25510652	rs7578575	25488819	A	T	0.329	-0.834	0.164	3.51E-07	0.383
IBD	DNMT3A	2	25510652	rs7578575	25488819	A	T	0.329	-0.745	0.130	9.22E-09	0.515
UC	DNMT3A	2	25510652	rs7578575	25488819	A	T	0.329	-0.627	0.156	5.73E-05	0.541
UC	CFDP1	16	75327596	rs9934007	75390532	C	T	0.754	0.273	0.070	9.61E-05	0.973
CD	STAT3	17	40502964	rs1053004	40466092	G	A	0.380	0.702	0.128	4.40E-08	0.056
CD	IMPDH2	3	49064299	rs9813813	49207093	A	T	0.110	-1.233	0.294	2.74E-05	0.213
IBD	IMPDH2	3	49064299	rs9813813	49207093	A	T	0.110	-1.328	0.258	2.57E-07	0.071
UC	IMPDH2	3	49064299	rs9813813	49207093	A	T	0.110	-1.445	0.307	2.53E-06	0.050
IBD	UBE2L3	22	21941029	rs2070512	21949411	C	A	0.194	0.163	0.021	1.06E-14	0.169
UC	UBE2L3	22	21941029	rs2070512	21949411	C	A	0.194	0.116	0.027	1.76E-05	0.698
CD	BCL2L15	1	1.14E+08	rs1217393	1.14E+08	A	G	0.271	-0.705	0.170	3.39E-05	0.184
CD	STMN3	20	62271061	rs909333	62270637	G	T	0.207	0.547	0.102	8.31E-08	0.200
IBD	STMN3	20	62271061	rs909333	62270637	G	T	0.207	0.555	0.087	2.01E-10	0.055
UC	STMN3	20	62271061	rs909333	62270637	G	T	0.207	0.498	0.098	4.04E-07	0.062
GORD	PBX2	6	32154964	rs693906	31835164	C	G	0.113	0.154	0.036	1.65E-05	0.058
VHD	AGER	6	32150423	rs693906	31835164	C	G	0.113	0.212	0.052	4.82E-05	0.092

B) Nominally significant effect on VHD of Bonferroni-significant gastrointestinal loci.

Disease	Gene	chr	Probe_bp	topSNP	topSNP_bp	A1	A2	Freq	b_SMR	se_SMR	p_SMR	p_HEIDI
VHD	CFDP1	16	75397489	rs7201989	75471499	G	A	0.246	-0.056	0.018	0.001	0.119
VHD	STAT3	17	40502964	rs1053004	40466092	G	A	0.380	0.093	0.047	0.048	0.948
VHD	IMPDH2	3	49064299	rs34037363	48821036	T	C	0.114	-0.209	0.105	0.047	0.089
VHD	UBE2L3	22	21941029	rs2070512	21949411	C	A	0.194	-0.029	0.009	0.002	0.623
VHD	STMN3	20	62271061	rs909333	62270637	G	T	0.207	-0.076	0.036	0.036	0.093

C) Effect of AGER gene on VHD and gastrointestinal disorders.

Disease	Gene	chr	Probe_bp	topSNP	topSNP_bp	A1	A2	Freq	b_SMR	se_SMR	p_SMR	p_HEIDI
CD	AGER	6	32150423	rs693906	31835164	C	G	0.113	-0.312	0.120	0.009	6.16E-08
GORD	AGER	6	32150423	rs693906	31835164	C	G	0.113	-0.209	0.049	2.02E-05	0.022
IBD	AGER	6	32148745	rs204995	32154285	G	A	0.217	-0.252	0.108	0.020	1.60E-04
IBS	AGER	6	32150423	rs693906	31835164	C	G	0.113	-0.168	0.052	0.001	0.045
UC	AGER	6	32148745	rs204995	32154285	G	A	0.217	-0.870	0.185	2.59E-06	0.002
VHD	AGER	6	32150423	rs693906	31835164	C	G	0.113	0.212	0.052	4.82E-05	0.092

Supplementary table 6. Results of the summary-based Mendelian randomization integrating whole-blood expression quantitative trait loci. For each disease-gene association, we report the strongest effect observed between GTEX and eQTLGen datasets.

A) Bonferroni-significant disease-gene association with HEIDI P > 0.05.

Disease	Gene	chr	gene_bp	topSNP	topSNP_bp	A1	A2	Freq	b_SMR	se_SMR	p_SMR	p_HEIDI
CD	DNMT3A	2	25510652	rs7578575	25488819	A	T	0.329	-0.834	0.164	3.51E-07	0.383
IBD	DNMT3A	2	25510652	rs7578575	25488819	A	T	0.329	-0.745	0.130	9.22E-09	0.515
UC	DNMT3A	2	25510652	rs7578575	25488819	A	T	0.329	-0.627	0.156	5.73E-05	0.541
UC	CFDP1	16	75327596	rs9934007	75390532	C	T	0.754	0.273	0.070	9.61E-05	0.973
CD	STAT3	17	40502964	rs1053004	40466092	G	A	0.380	0.702	0.128	4.40E-08	0.056
CD	IMPDH2	3	49064299	rs9813813	49207093	A	T	0.110	-1.233	0.294	2.74E-05	0.213
IBD	IMPDH2	3	49064299	rs9813813	49207093	A	T	0.110	-1.328	0.258	2.57E-07	0.071
UC	IMPDH2	3	49064299	rs9813813	49207093	A	T	0.110	-1.445	0.307	2.53E-06	0.050
IBD	UBE2L3	22	21941029	rs2070512	21949411	C	A	0.194	0.163	0.021	1.06E-14	0.169
UC	UBE2L3	22	21941029	rs2070512	21949411	C	A	0.194	0.116	0.027	1.76E-05	0.698
CD	BCL2L15	1	1.14E+08	rs1217393	1.14E+08	A	G	0.271	-0.705	0.170	3.39E-05	0.184
CD	STMN3	20	62271061	rs909333	62270637	G	T	0.207	0.547	0.102	8.31E-08	0.200
IBD	STMN3	20	62271061	rs909333	62270637	G	T	0.207	0.555	0.087	2.01E-10	0.055
UC	STMN3	20	62271061	rs909333	62270637	G	T	0.207	0.498	0.098	4.04E-07	0.062
GORD	PBX2	6	32154964	rs693906	31835164	C	G	0.113	0.154	0.036	1.65E-05	0.058
VHD	AGER	6	32150423	rs693906	31835164	C	G	0.113	0.212	0.052	4.82E-05	0.092

B) Nominally significant effect on VHD of Bonferroni-significant gastrointestinal loci.

Disease	Gene	chr	Probe_bp	topSNP	topSNP_bp	A1	A2	Freq	b_SMR	se_SMR	p_SMR	p_HEIDI
VHD	CFDP1	16	75397489	rs7201989	75471499	G	A	0.246	-0.056	0.018	0.001	0.119
VHD	STAT3	17	40502964	rs1053004	40466092	G	A	0.380	0.093	0.047	0.048	0.948
VHD	IMPDH2	3	49064299	rs34037363	48821036	T	C	0.114	-0.209	0.105	0.047	0.089
VHD	UBE2L3	22	21941029	rs2070512	21949411	C	A	0.194	-0.029	0.009	0.002	0.623
VHD	STMN3	20	62271061	rs909333	62270637	G	T	0.207	-0.076	0.036	0.036	0.093

C) Effect of AGER gene on VHD and gastrointestinal disorders.

Disease	Gene	chr	Probe_bp	topSNP	topSNP_bp	A1	A2	Freq	b_SMR	se_SMR	p_SMR	p_HEIDI
CD	AGER	6	32150423	rs693906	31835164	C	G	0.113	-0.312	0.120	0.009	6.16E-08
GORD	AGER	6	32150423	rs693906	31835164	C	G	0.113	-0.209	0.049	2.02E-05	0.022
IBD	AGER	6	32148745	rs204995	32154285	G	A	0.217	-0.252	0.108	0.020	1.60E-04
IBS	AGER	6	32150423	rs693906	31835164	C	G	0.113	-0.168	0.052	0.001	0.045
UC	AGER	6	32148745	rs204995	32154285	G	A	0.217	-0.870	0.185	2.59E-06	0.002
VHD	AGER	6	32150423	rs693906	31835164	C	G	0.113	0.212	0.052	4.82E-05	0.092

Supplementary table 6. Results of the summary-based Mendelian randomization integrating whole-blood expression quantitative trait loci. For each disease-gene association, we report the strongest effect observed between GTEX and eQTLGen datasets.

A) Bonferroni-significant disease-gene association with HEIDI P > 0.05.

Disease	Gene	chr	gene_bp	topSNP	topSNP_bp	A1	A2	Freq	b_SMR	se_SMR	p_SMR	p_HEIDI
CD	DNMT3A	2	25510652	rs7578575	25488819	A	T	0.329	-0.834	0.164	3.51E-07	0.383
IBD	DNMT3A	2	25510652	rs7578575	25488819	A	T	0.329	-0.745	0.130	9.22E-09	0.515
UC	DNMT3A	2	25510652	rs7578575	25488819	A	T	0.329	-0.627	0.156	5.73E-05	0.541
UC	CFDP1	16	75327596	rs9934007	75390532	C	T	0.754	0.273	0.070	9.61E-05	0.973
CD	STAT3	17	40502964	rs1053004	40466092	G	A	0.380	0.702	0.128	4.40E-08	0.056
CD	IMPDH2	3	49064299	rs9813813	49207093	A	T	0.110	-1.233	0.294	2.74E-05	0.213
IBD	IMPDH2	3	49064299	rs9813813	49207093	A	T	0.110	-1.328	0.258	2.57E-07	0.071
UC	IMPDH2	3	49064299	rs9813813	49207093	A	T	0.110	-1.445	0.307	2.53E-06	0.050
IBD	UBE2L3	22	21941029	rs2070512	21949411	C	A	0.194	0.163	0.021	1.06E-14	0.169
UC	UBE2L3	22	21941029	rs2070512	21949411	C	A	0.194	0.116	0.027	1.76E-05	0.698
CD	BCL2L15	1	1.14E+08	rs1217393	1.14E+08	A	G	0.271	-0.705	0.170	3.39E-05	0.184
CD	STMN3	20	62271061	rs909333	62270637	G	T	0.207	0.547	0.102	8.31E-08	0.200
IBD	STMN3	20	62271061	rs909333	62270637	G	T	0.207	0.555	0.087	2.01E-10	0.055
UC	STMN3	20	62271061	rs909333	62270637	G	T	0.207	0.498	0.098	4.04E-07	0.062
GORD	PBX2	6	32154964	rs693906	31835164	C	G	0.113	0.154	0.036	1.65E-05	0.058
VHD	AGER	6	32150423	rs693906	31835164	C	G	0.113	0.212	0.052	4.82E-05	0.092

B) Nominally significant effect on VHD of Bonferroni-significant gastrointestinal loci.

Disease	Gene	chr	Probe_bp	topSNP	topSNP_bp	A1	A2	Freq	b_SMR	se_SMR	p_SMR	p_HEIDI
VHD	CFDP1	16	75397489	rs7201989	75471499	G	A	0.246	-0.056	0.018	0.001	0.119
VHD	STAT3	17	40502964	rs1053004	40466092	G	A	0.380	0.093	0.047	0.048	0.948
VHD	IMPDH2	3	49064299	rs34037363	48821036	T	C	0.114	-0.209	0.105	0.047	0.089
VHD	UBE2L3	22	21941029	rs2070512	21949411	C	A	0.194	-0.029	0.009	0.002	0.623
VHD	STMN3	20	62271061	rs909333	62270637	G	T	0.207	-0.076	0.036	0.036	0.093

C) Effect of AGER gene on VHD and gastrointestinal disorders.

Disease	Gene	chr	Probe_bp	topSNP	topSNP_bp	A1	A2	Freq	b_SMR	se_SMR	p_SMR	p_HEIDI
CD	AGER	6	32150423	rs693906	31835164	C	G	0.113	-0.312	0.120	0.009	6.16E-08
GORD	AGER	6	32150423	rs693906	31835164	C	G	0.113	-0.209	0.049	2.02E-05	0.022
IBD	AGER	6	32148745	rs204995	32154285	G	A	0.217	-0.252	0.108	0.020	1.60E-04
IBS	AGER	6	32150423	rs693906	31835164	C	G	0.113	-0.168	0.052	0.001	0.045
UC	AGER	6	32148745	rs204995	32154285	G	A	0.217	-0.870	0.185	2.59E-06	0.002
VHD	AGER	6	32150423	rs693906	31835164	C	G	0.113	0.212	0.052	4.82E-05	0.092

Supplementary table 6. Results of the summary-based Mendelian randomization integrating whole-blood expression quantitative trait loci. For each disease-gene association, we report the strongest effect observed between GTEX and eQTLGen datasets.

A) Bonferroni-significant disease-gene association with HEIDI P > 0.05.

Disease	Gene	chr	gene_bp	topSNP	topSNP_bp	A1	A2	Freq	b_SMR	se_SMR	p_SMR	p_HEIDI
CD	DNMT3A	2	25510652	rs7578575	25488819	A	T	0.329	-0.834	0.164	3.51E-07	0.383
IBD	DNMT3A	2	25510652	rs7578575	25488819	A	T	0.329	-0.745	0.130	9.22E-09	0.515
UC	DNMT3A	2	25510652	rs7578575	25488819	A	T	0.329	-0.627	0.156	5.73E-05	0.541
UC	CFDP1	16	75327596	rs9934007	75390532	C	T	0.754	0.273	0.070	9.61E-05	0.973
CD	STAT3	17	40502964	rs1053004	40466092	G	A	0.380	0.702	0.128	4.40E-08	0.056
CD	IMPDH2	3	49064299	rs9813813	49207093	A	T	0.110	-1.233	0.294	2.74E-05	0.213
IBD	IMPDH2	3	49064299	rs9813813	49207093	A	T	0.110	-1.328	0.258	2.57E-07	0.071
UC	IMPDH2	3	49064299	rs9813813	49207093	A	T	0.110	-1.445	0.307	2.53E-06	0.050
IBD	UBE2L3	22	21941029	rs2070512	21949411	C	A	0.194	0.163	0.021	1.06E-14	0.169
UC	UBE2L3	22	21941029	rs2070512	21949411	C	A	0.194	0.116	0.027	1.76E-05	0.698
CD	BCL2L15	1	1.14E+08	rs1217393	1.14E+08	A	G	0.271	-0.705	0.170	3.39E-05	0.184
CD	STMN3	20	62271061	rs909333	62270637	G	T	0.207	0.547	0.102	8.31E-08	0.200
IBD	STMN3	20	62271061	rs909333	62270637	G	T	0.207	0.555	0.087	2.01E-10	0.055
UC	STMN3	20	62271061	rs909333	62270637	G	T	0.207	0.498	0.098	4.04E-07	0.062
GORD	PBX2	6	32154964	rs693906	31835164	C	G	0.113	0.154	0.036	1.65E-05	0.058
VHD	AGER	6	32150423	rs693906	31835164	C	G	0.113	0.212	0.052	4.82E-05	0.092

B) Nominally significant effect on VHD of Bonferroni-significant gastrointestinal loci.

Disease	Gene	chr	Probe_bp	topSNP	topSNP_bp	A1	A2	Freq	b_SMR	se_SMR	p_SMR	p_HEIDI
VHD	CFDP1	16	75397489	rs7201989	75471499	G	A	0.246	-0.056	0.018	0.001	0.119
VHD	STAT3	17	40502964	rs1053004	40466092	G	A	0.380	0.093	0.047	0.048	0.948
VHD	IMPDH2	3	49064299	rs34037363	48821036	T	C	0.114	-0.209	0.105	0.047	0.089
VHD	UBE2L3	22	21941029	rs2070512	21949411	C	A	0.194	-0.029	0.009	0.002	0.623
VHD	STMN3	20	62271061	rs909333	62270637	G	T	0.207	-0.076	0.036	0.036	0.093

C) Effect of AGER gene on VHD and gastrointestinal disorders.

Disease	Gene	chr	Probe_bp	topSNP	topSNP_bp	A1	A2	Freq	b_SMR	se_SMR	p_SMR	p_HEIDI
CD	AGER	6	32150423	rs693906	31835164	C	G	0.113	-0.312	0.120	0.009	6.16E-08
GORD	AGER	6	32150423	rs693906	31835164	C	G	0.113	-0.209	0.049	2.02E-05	0.022
IBD	AGER	6	32148745	rs204995	32154285	G	A	0.217	-0.252	0.108	0.020	1.60E-04
IBS	AGER	6	32150423	rs693906	31835164	C	G	0.113	-0.168	0.052	0.001	0.045
UC	AGER	6	32148745	rs204995	32154285	G	A	0.217	-0.870	0.185	2.59E-06	0.002
VHD	AGER	6	32150423	rs693906	31835164	C	G	0.113	0.212	0.052	4.82E-05	0.092

Supplementary table 6. Results of the summary-based Mendelian randomization integrating whole-blood expression quantitative trait loci. For each disease-gene association, we report the strongest effect observed between GTEX and eQTLGen datasets.

A) Bonferroni-significant disease-gene association with HEIDI P > 0.05.

Disease	Gene	chr	gene_bp	topSNP	topSNP_bp	A1	A2	Freq	b_SMR	se_SMR	p_SMR	p_HEIDI
CD	DNMT3A	2	25510652	rs7578575	25488819	A	T	0.329	-0.834	0.164	3.51E-07	0.383
IBD	DNMT3A	2	25510652	rs7578575	25488819	A	T	0.329	-0.745	0.130	9.22E-09	0.515
UC	DNMT3A	2	25510652	rs7578575	25488819	A	T	0.329	-0.627	0.156	5.73E-05	0.541
UC	CFDP1	16	75327596	rs9934007	75390532	C	T	0.754	0.273	0.070	9.61E-05	0.973
CD	STAT3	17	40502964	rs1053004	40466092	G	A	0.380	0.702	0.128	4.40E-08	0.056
CD	IMPDH2	3	49064299	rs9813813	49207093	A	T	0.110	-1.233	0.294	2.74E-05	0.213
IBD	IMPDH2	3	49064299	rs9813813	49207093	A	T	0.110	-1.328	0.258	2.57E-07	0.071
UC	IMPDH2	3	49064299	rs9813813	49207093	A	T	0.110	-1.445	0.307	2.53E-06	0.050
IBD	UBE2L3	22	21941029	rs2070512	21949411	C	A	0.194	0.163	0.021	1.06E-14	0.169
UC	UBE2L3	22	21941029	rs2070512	21949411	C	A	0.194	0.116	0.027	1.76E-05	0.698
CD	BCL2L15	1	1.14E+08	rs1217393	1.14E+08	A	G	0.271	-0.705	0.170	3.39E-05	0.184
CD	STMN3	20	62271061	rs909333	62270637	G	T	0.207	0.547	0.102	8.31E-08	0.200
IBD	STMN3	20	62271061	rs909333	62270637	G	T	0.207	0.555	0.087	2.01E-10	0.055
UC	STMN3	20	62271061	rs909333	62270637	G	T	0.207	0.498	0.098	4.04E-07	0.062
GORD	PBX2	6	32154964	rs693906	31835164	C	G	0.113	0.154	0.036	1.65E-05	0.058
VHD	AGER	6	32150423	rs693906	31835164	C	G	0.113	0.212	0.052	4.82E-05	0.092

B) Nominally significant effect on VHD of Bonferroni-significant gastrointestinal loci.

Disease	Gene	chr	Probe_bp	topSNP	topSNP_bp	A1	A2	Freq	b_SMR	se_SMR	p_SMR	p_HEIDI
VHD	CFDP1	16	75397489	rs7201989	75471499	G	A	0.246	-0.056	0.018	0.001	0.119
VHD	STAT3	17	40502964	rs1053004	40466092	G	A	0.380	0.093	0.047	0.048	0.948
VHD	IMPDH2	3	49064299	rs34037363	48821036	T	C	0.114	-0.209	0.105	0.047	0.089
VHD	UBE2L3	22	21941029	rs2070512	21949411	C	A	0.194	-0.029	0.009	0.002	0.623
VHD	STMN3	20	62271061	rs909333	62270637	G	T	0.207	-0.076	0.036	0.036	0.093

C) Effect of AGER gene on VHD and gastrointestinal disorders.

Disease	Gene	chr	Probe_bp	topSNP	topSNP_bp	A1	A2	Freq	b_SMR	se_SMR	p_SMR	p_HEIDI
CD	AGER	6	32150423	rs693906	31835164	C	G	0.113	-0.312	0.120	0.009	6.16E-08
GORD	AGER	6	32150423	rs693906	31835164	C	G	0.113	-0.209	0.049	2.02E-05	0.022
IBD	AGER	6	32148745	rs204995	32154285	G	A	0.217	-0.252	0.108	0.020	1.60E-04
IBS	AGER	6	32150423	rs693906	31835164	C	G	0.113	-0.168	0.052	0.001	0.045
UC	AGER	6	32148745	rs204995	32154285	G	A	0.217	-0.870	0.185	2.59E-06	0.002
VHD	AGER	6	32150423	rs693906	31835164	C	G	0.113	0.212	0.052	4.82E-05	0.092

Supplementary table 6. Results of the summary-based Mendelian randomization integrating whole-blood expression quantitative trait loci. For each disease-gene association, we report the strongest effect observed between GTEX and eQTLGen datasets.

A) Bonferroni-significant disease-gene association with HEIDI P > 0.05.

Disease	Gene	chr	gene_bp	topSNP	topSNP_bp	A1	A2	Freq	b_SMR	se_SMR	p_SMR	p_HEIDI
CD	DNMT3A	2	25510652	rs7578575	25488819	A	T	0.329	-0.834	0.164	3.51E-07	0.383
IBD	DNMT3A	2	25510652	rs7578575	25488819	A	T	0.329	-0.745	0.130	9.22E-09	0.515
UC	DNMT3A	2	25510652	rs7578575	25488819	A	T	0.329	-0.627	0.156	5.73E-05	0.541
UC	CFDP1	16	75327596	rs9934007	75390532	C	T	0.754	0.273	0.070	9.61E-05	0.973
CD	STAT3	17	40502964	rs1053004	40466092	G	A	0.380	0.702	0.128	4.40E-08	0.056
CD	IMPDH2	3	49064299	rs9813813	49207093	A	T	0.110	-1.233	0.294	2.74E-05	0.213
IBD	IMPDH2	3	49064299	rs9813813	49207093	A	T	0.110	-1.328	0.258	2.57E-07	0.071
UC	IMPDH2	3	49064299	rs9813813	49207093	A	T	0.110	-1.445	0.307	2.53E-06	0.050
IBD	UBE2L3	22	21941029	rs2070512	21949411	C	A	0.194	0.163	0.021	1.06E-14	0.169
UC	UBE2L3	22	21941029	rs2070512	21949411	C	A	0.194	0.116	0.027	1.76E-05	0.698
CD	BCL2L15	1	1.14E+08	rs1217393	1.14E+08	A	G	0.271	-0.705	0.170	3.39E-05	0.184
CD	STMN3	20	62271061	rs909333	62270637	G	T	0.207	0.547	0.102	8.31E-08	0.200
IBD	STMN3	20	62271061	rs909333	62270637	G	T	0.207	0.555	0.087	2.01E-10	0.055
UC	STMN3	20	62271061	rs909333	62270637	G	T	0.207	0.498	0.098	4.04E-07	0.062
GORD	PBX2	6	32154964	rs693906	31835164	C	G	0.113	0.154	0.036	1.65E-05	0.058
VHD	AGER	6	32150423	rs693906	31835164	C	G	0.113	0.212	0.052	4.82E-05	0.092

B) Nominally significant effect on VHD of Bonferroni-significant gastrointestinal loci.

Disease	Gene	chr	Probe_bp	topSNP	topSNP_bp	A1	A2	Freq	b_SMR	se_SMR	p_SMR	p_HEIDI
VHD	CFDP1	16	75397489	rs7201989	75471499	G	A	0.246	-0.056	0.018	0.001	0.119
VHD	STAT3	17	40502964	rs1053004	40466092	G	A	0.380	0.093	0.047	0.048	0.948
VHD	IMPDH2	3	49064299	rs34037363	48821036	T	C	0.114	-0.209	0.105	0.047	0.089
VHD	UBE2L3	22	21941029	rs2070512	21949411	C	A	0.194	-0.029	0.009	0.002	0.623
VHD	STMN3	20	62271061	rs909333	62270637	G	T	0.207	-0.076	0.036	0.036	0.093

C) Effect of AGER gene on VHD and gastrointestinal disorders.

Disease	Gene	chr	Probe_bp	topSNP	topSNP_bp	A1	A2	Freq	b_SMR	se_SMR	p_SMR	p_HEIDI
CD	AGER	6	32150423	rs693906	31835164	C	G	0.113	-0.312	0.120	0.009	6.16E-08
GORD	AGER	6	32150423	rs693906	31835164	C	G	0.113	-0.209	0.049	2.02E-05	0.022
IBD	AGER	6	32148745	rs204995	32154285	G	A	0.217	-0.252	0.108	0.020	1.60E-04
IBS	AGER	6	32150423	rs693906	31835164	C	G	0.113	-0.168	0.052	0.001	0.045
UC	AGER	6	32148745	rs204995	32154285	G	A	0.217	-0.870	0.185	2.59E-06	0.002
VHD	AGER	6	32150423	rs693906	31835164	C	G	0.113	0.212	0.052	4.82E-05	0.092

Supplementary table 6. Results of the summary-based Mendelian randomization integrating whole-blood expression quantitative trait loci. For each disease-gene association, we report the strongest effect observed between GTEX and eQTLGen datasets.

A) Bonferroni-significant disease-gene association with HEIDI P > 0.05.

Disease	Gene	chr	gene_bp	topSNP	topSNP_bp	A1	A2	Freq	b_SMR	se_SMR	p_SMR	p_HEIDI
CD	DNMT3A	2	25510652	rs7578575	25488819	A	T	0.329	-0.834	0.164	3.51E-07	0.383
IBD	DNMT3A	2	25510652	rs7578575	25488819	A	T	0.329	-0.745	0.130	9.22E-09	0.515
UC	DNMT3A	2	25510652	rs7578575	25488819	A	T	0.329	-0.627	0.156	5.73E-05	0.541
UC	CFDP1	16	75327596	rs9934007	75390532	C	T	0.754	0.273	0.070	9.61E-05	0.973
CD	STAT3	17	40502964	rs1053004	40466092	G	A	0.380	0.702	0.128	4.40E-08	0.056
CD	IMPDH2	3	49064299	rs9813813	49207093	A	T	0.110	-1.233	0.294	2.74E-05	0.213
IBD	IMPDH2	3	49064299	rs9813813	49207093	A	T	0.110	-1.328	0.258	2.57E-07	0.071
UC	IMPDH2	3	49064299	rs9813813	49207093	A	T	0.110	-1.445	0.307	2.53E-06	0.050
IBD	UBE2L3	22	21941029	rs2070512	21949411	C	A	0.194	0.163	0.021	1.06E-14	0.169
UC	UBE2L3	22	21941029	rs2070512	21949411	C	A	0.194	0.116	0.027	1.76E-05	0.698
CD	BCL2L15	1	1.14E+08	rs1217393	1.14E+08	A	G	0.271	-0.705	0.170	3.39E-05	0.184
CD	STMN3	20	62271061	rs909333	62270637	G	T	0.207	0.547	0.102	8.31E-08	0.200
IBD	STMN3	20	62271061	rs909333	62270637	G	T	0.207	0.555	0.087	2.01E-10	0.055
UC	STMN3	20	62271061	rs909333	62270637	G	T	0.207	0.498	0.098	4.04E-07	0.062
GORD	PBX2	6	32154964	rs693906	31835164	C	G	0.113	0.154	0.036	1.65E-05	0.058
VHD	AGER	6	32150423	rs693906	31835164	C	G	0.113	0.212	0.052	4.82E-05	0.092

B) Nominally significant effect on VHD of Bonferroni-significant gastrointestinal loci.

Disease	Gene	chr	Probe_bp	topSNP	topSNP_bp	A1	A2	Freq	b_SMR	se_SMR	p_SMR	p_HEIDI
VHD	CFDP1	16	75397489	rs7201989	75471499	G	A	0.246	-0.056	0.018	0.001	0.119
VHD	STAT3	17	40502964	rs1053004	40466092	G	A	0.380	0.093	0.047	0.048	0.948
VHD	IMPDH2	3	49064299	rs34037363	48821036	T	C	0.114	-0.209	0.105	0.047	0.089
VHD	UBE2L3	22	21941029	rs2070512	21949411	C	A	0.194	-0.029	0.009	0.002	0.623
VHD	STMN3	20	62271061	rs909333	62270637	G	T	0.207	-0.076	0.036	0.036	0.093

C) Effect of AGER gene on VHD and gastrointestinal disorders.

Disease	Gene	chr	Probe_bp	topSNP	topSNP_bp	A1	A2	Freq	b_SMR	se_SMR	p_SMR	p_HEIDI
CD	AGER	6	32150423	rs693906	31835164	C	G	0.113	-0.312	0.120	0.009	6.16E-08
GORD	AGER	6	32150423	rs693906	31835164	C	G	0.113	-0.209	0.049	2.02E-05	0.022
IBD	AGER	6	32148745	rs204995	32154285	G	A	0.217	-0.252	0.108	0.020	1.60E-04
IBS	AGER	6	32150423	rs693906	31835164	C	G	0.113	-0.168	0.052	0.001	0.045
UC	AGER	6	32148745	rs204995	32154285	G	A	0.217	-0.870	0.185	2.59E-06	0.002
VHD	AGER	6	32150423	rs693906	31835164	C	G	0.113	0.212	0.052	4.82E-05	0.092

Supplementary table 6. Results of the summary-based Mendelian randomization integrating whole-blood expression quantitative trait loci. For each disease-gene association, we report the strongest effect observed between GTEX and eQTLGen datasets.

A) Bonferroni-significant disease-gene association with HEIDI P > 0.05.

Disease	Gene	chr	gene_bp	topSNP	topSNP_bp	A1	A2	Freq	b_SMR	se_SMR	p_SMR	p_HEIDI
CD	DNMT3A	2	25510652	rs7578575	25488819	A	T	0.329	-0.834	0.164	3.51E-07	0.383
IBD	DNMT3A	2	25510652	rs7578575	25488819	A	T	0.329	-0.745	0.130	9.22E-09	0.515
UC	DNMT3A	2	25510652	rs7578575	25488819	A	T	0.329	-0.627	0.156	5.73E-05	0.541
UC	CFDP1	16	75327596	rs9934007	75390532	C	T	0.754	0.273	0.070	9.61E-05	0.973
CD	STAT3	17	40502964	rs1053004	40466092	G	A	0.380	0.702	0.128	4.40E-08	0.056
CD	IMPDH2	3	49064299	rs9813813	49207093	A	T	0.110	-1.233	0.294	2.74E-05	0.213
IBD	IMPDH2	3	49064299	rs9813813	49207093	A	T	0.110	-1.328	0.258	2.57E-07	0.071
UC	IMPDH2	3	49064299	rs9813813	49207093	A	T	0.110	-1.445	0.307	2.53E-06	0.050
IBD	UBE2L3	22	21941029	rs2070512	21949411	C	A	0.194	0.163	0.021	1.06E-14	0.169
UC	UBE2L3	22	21941029	rs2070512	21949411	C	A	0.194	0.116	0.027	1.76E-05	0.698
CD	BCL2L15	1	1.14E+08	rs1217393	1.14E+08	A	G	0.271	-0.705	0.170	3.39E-05	0.184
CD	STMN3	20	62271061	rs909333	62270637	G	T	0.207	0.547	0.102	8.31E-08	0.200
IBD	STMN3	20	62271061	rs909333	62270637	G	T	0.207	0.555	0.087	2.01E-10	0.055
UC	STMN3	20	62271061	rs909333	62270637	G	T	0.207	0.498	0.098	4.04E-07	0.062
GORD	PBX2	6	32154964	rs693906	31835164	C	G	0.113	0.154	0.036	1.65E-05	0.058
VHD	AGER	6	32150423	rs693906	31835164	C	G	0.113	0.212	0.052	4.82E-05	0.092

B) Nominally significant effect on VHD of Bonferroni-significant gastrointestinal loci.

Disease	Gene	chr	Probe_bp	topSNP	topSNP_bp	A1	A2	Freq	b_SMR	se_SMR	p_SMR	p_HEIDI
VHD	CFDP1	16	75397489	rs7201989	75471499	G	A	0.246	-0.056	0.018	0.001	0.119
VHD	STAT3	17	40502964	rs1053004	40466092	G	A	0.380	0.093	0.047	0.048	0.948
VHD	IMPDH2	3	49064299	rs34037363	48821036	T	C	0.114	-0.209	0.105	0.047	0.089
VHD	UBE2L3	22	21941029	rs2070512	21949411	C	A	0.194	-0.029	0.009	0.002	0.623
VHD	STMN3	20	62271061	rs909333	62270637	G	T	0.207	-0.076	0.036	0.036	0.093

C) Effect of AGER gene on VHD and gastrointestinal disorders.

Disease	Gene	chr	Probe_bp	topSNP	topSNP_bp	A1	A2	Freq	b_SMR	se_SMR	p_SMR	p_HEIDI
CD	AGER	6	32150423	rs693906	31835164	C	G	0.113	-0.312	0.120	0.009	6.16E-08
GORD	AGER	6	32150423	rs693906	31835164	C	G	0.113	-0.209	0.049	2.02E-05	0.022
IBD	AGER	6	32148745	rs204995	32154285	G	A	0.217	-0.252	0.108	0.020	1.60E-04
IBS	AGER	6	32150423	rs693906	31835164	C	G	0.113	-0.168	0.052	0.001	0.045
UC	AGER	6	32148745	rs204995	32154285	G	A	0.217	-0.870	0.185	2.59E-06	0.002
VHD	AGER	6	32150423	rs693906	31835164	C	G	0.113	0.212	0.052	4.82E-05	0.092

Supplementary table 6. Results of the summary-based Mendelian randomization integrating whole-blood expression quantitative trait loci. For each disease-gene association, we report the strongest effect observed between GTEX and eQTLGen datasets.

A) Bonferroni-significant disease-gene association with HEIDI P > 0.05.

Disease	Gene	chr	gene_bp	topSNP	topSNP_bp	A1	A2	Freq	b_SMR	se_SMR	p_SMR	p_HEIDI
CD	DNMT3A	2	25510652	rs7578575	25488819	A	T	0.329	-0.834	0.164	3.51E-07	0.383
IBD	DNMT3A	2	25510652	rs7578575	25488819	A	T	0.329	-0.745	0.130	9.22E-09	0.515
UC	DNMT3A	2	25510652	rs7578575	25488819	A	T	0.329	-0.627	0.156	5.73E-05	0.541
UC	CFDP1	16	75327596	rs9934007	75390532	C	T	0.754	0.273	0.070	9.61E-05	0.973
CD	STAT3	17	40502964	rs1053004	40466092	G	A	0.380	0.702	0.128	4.40E-08	0.056
CD	IMPDH2	3	49064299	rs9813813	49207093	A	T	0.110	-1.233	0.294	2.74E-05	0.213
IBD	IMPDH2	3	49064299	rs9813813	49207093	A	T	0.110	-1.328	0.258	2.57E-07	0.071
UC	IMPDH2	3	49064299	rs9813813	49207093	A	T	0.110	-1.445	0.307	2.53E-06	0.050
IBD	UBE2L3	22	21941029	rs2070512	21949411	C	A	0.194	0.163	0.021	1.06E-14	0.169
UC	UBE2L3	22	21941029	rs2070512	21949411	C	A	0.194	0.116	0.027	1.76E-05	0.698
CD	BCL2L15	1	1.14E+08	rs1217393	1.14E+08	A	G	0.271	-0.705	0.170	3.39E-05	0.184
CD	STMN3	20	62271061	rs909333	62270637	G	T	0.207	0.547	0.102	8.31E-08	0.200
IBD	STMN3	20	62271061	rs909333	62270637	G	T	0.207	0.555	0.087	2.01E-10	0.055
UC	STMN3	20	62271061	rs909333	62270637	G	T	0.207	0.498	0.098	4.04E-07	0.062
GORD	PBX2	6	32154964	rs693906	31835164	C	G	0.113	0.154	0.036	1.65E-05	0.058
VHD	AGER	6	32150423	rs693906	31835164	C	G	0.113	0.212	0.052	4.82E-05	0.092

B) Nominally significant effect on VHD of Bonferroni-significant gastrointestinal loci.

Disease	Gene	chr	Probe_bp	topSNP	topSNP_bp	A1	A2	Freq	b_SMR	se_SMR	p_SMR	p_HEIDI
VHD	CFDP1	16	75397489	rs7201989	75471499	G	A	0.246	-0.056	0.018	0.001	0.119
VHD	STAT3	17	40502964	rs1053004	40466092	G	A	0.380	0.093	0.047	0.048	0.948
VHD	IMPDH2	3	49064299	rs34037363	48821036	T	C	0.114	-0.209	0.105	0.047	0.089
VHD	UBE2L3	22	21941029	rs2070512	21949411	C	A	0.194	-0.029	0.009	0.002	0.623
VHD	STMN3	20	62271061	rs909333	62270637	G	T	0.207	-0.076	0.036	0.036	0.093

C) Effect of AGER gene on VHD and gastrointestinal disorders.

Disease	Gene	chr	Probe_bp	topSNP	topSNP_bp	A1	A2	Freq	b_SMR	se_SMR	p_SMR	p_HEIDI
CD	AGER	6	32150423	rs693906	31835164	C	G	0.113	-0.312	0.120	0.009	6.16E-08
GORD	AGER	6	32150423	rs693906	31835164	C	G	0.113	-0.209	0.049	2.02E-05	0.022
IBD	AGER	6	32148745	rs204995	32154285	G	A	0.217	-0.252	0.108	0.020	1.60E-04
IBS	AGER	6	32150423	rs693906	31835164	C	G	0.113	-0.168	0.052	0.001	0.045
UC	AGER	6	32148745	rs204995	32154285	G	A	0.217	-0.870	0.185	2.59E-06	0.002
VHD	AGER	6	32150423	rs693906	31835164	C	G	0.113	0.212	0.052	4.82E-05	0.092

Supplementary table 6. Results of the summary-based Mendelian randomization integrating whole-blood expression quantitative trait loci. For each disease-gene association, we report the strongest effect observed between GTEX and eQTLGen datasets.

A) Bonferroni-significant disease-gene association with HEIDI P > 0.05.

Disease	Gene	chr	gene_bp	topSNP	topSNP_bp	A1	A2	Freq	b_SMR	se_SMR	p_SMR	p_HEIDI
CD	DNMT3A	2	25510652	rs7578575	25488819	A	T	0.329	-0.834	0.164	3.51E-07	0.383
IBD	DNMT3A	2	25510652	rs7578575	25488819	A	T	0.329	-0.745	0.130	9.22E-09	0.515
UC	DNMT3A	2	25510652	rs7578575	25488819	A	T	0.329	-0.627	0.156	5.73E-05	0.541
UC	CFDP1	16	75327596	rs9934007	75390532	C	T	0.754	0.273	0.070	9.61E-05	0.973
CD	STAT3	17	40502964	rs1053004	40466092	G	A	0.380	0.702	0.128	4.40E-08	0.056
CD	IMPDH2	3	49064299	rs9813813	49207093	A	T	0.110	-1.233	0.294	2.74E-05	0.213
IBD	IMPDH2	3	49064299	rs9813813	49207093	A	T	0.110	-1.328	0.258	2.57E-07	0.071
UC	IMPDH2	3	49064299	rs9813813	49207093	A	T	0.110	-1.445	0.307	2.53E-06	0.050
IBD	UBE2L3	22	21941029	rs2070512	21949411	C	A	0.194	0.163	0.021	1.06E-14	0.169
UC	UBE2L3	22	21941029	rs2070512	21949411	C	A	0.194	0.116	0.027	1.76E-05	0.698
CD	BCL2L15	1	1.14E+08	rs1217393	1.14E+08	A	G	0.271	-0.705	0.170	3.39E-05	0.184
CD	STMN3	20	62271061	rs909333	62270637	G	T	0.207	0.547	0.102	8.31E-08	0.200
IBD	STMN3	20	62271061	rs909333	62270637	G	T	0.207	0.555	0.087	2.01E-10	0.055
UC	STMN3	20	62271061	rs909333	62270637	G	T	0.207	0.498	0.098	4.04E-07	0.062
GORD	PBX2	6	32154964	rs693906	31835164	C	G	0.113	0.154	0.036	1.65E-05	0.058
VHD	AGER	6	32150423	rs693906	31835164	C	G	0.113	0.212	0.052	4.82E-05	0.092

B) Nominally significant effect on VHD of Bonferroni-significant gastrointestinal loci.

Disease	Gene	chr	Probe_bp	topSNP	topSNP_bp	A1	A2	Freq	b_SMR	se_SMR	p_SMR	p_HEIDI
VHD	CFDP1	16	75397489	rs7201989	75471499	G	A	0.246	-0.056	0.018	0.001	0.119
VHD	STAT3	17	40502964	rs1053004	40466092	G	A	0.380	0.093	0.047	0.048	0.948
VHD	IMPDH2	3	49064299	rs34037363	48821036	T	C	0.114	-0.209	0.105	0.047	0.089
VHD	UBE2L3	22	21941029	rs2070512	21949411	C	A	0.194	-0.029	0.009	0.002	0.623
VHD	STMN3	20	62271061	rs909333	62270637	G	T	0.207	-0.076	0.036	0.036	0.093

C) Effect of AGER gene on VHD and gastrointestinal disorders.

Disease	Gene	chr	Probe_bp	topSNP	topSNP_bp	A1	A2	Freq	b_SMR	se_SMR	p_SMR	p_HEIDI
CD	AGER	6	32150423	rs693906	31835164	C	G	0.113	-0.312	0.120	0.009	6.16E-08
GORD	AGER	6	32150423	rs693906	31835164	C	G	0.113	-0.209	0.049	2.02E-05	0.022
IBD	AGER	6	32148745	rs204995	32154285	G	A	0.217	-0.252	0.108	0.020	1.60E-04
IBS	AGER	6	32150423	rs693906	31835164	C	G	0.113	-0.168	0.052	0.001	0.045
UC	AGER	6	32148745	rs204995	32154285	G	A	0.217	-0.870	0.185	2.59E-06	0.002
VHD	AGER	6	32150423	rs693906	31835164	C	G	0.113	0.212	0.052	4.82E-05	0.092

Supplementary table 6. Results of the summary-based Mendelian randomization integrating whole-blood expression quantitative trait loci. For each disease-gene association, we report the strongest effect observed between GTEX and eQTLGen datasets.

A) Bonferroni-significant disease-gene association with HEIDI P > 0.05.

Disease	Gene	chr	gene_bp	topSNP	topSNP_bp	A1	A2	Freq	b_SMR	se_SMR	p_SMR	p_HEIDI
CD	DNMT3A	2	25510652	rs7578575	25488819	A	T	0.329	-0.834	0.164	3.51E-07	0.383
IBD	DNMT3A	2	25510652	rs7578575	25488819	A	T	0.329	-0.745	0.130	9.22E-09	0.515
UC	DNMT3A	2	25510652	rs7578575	25488819	A	T	0.329	-0.627	0.156	5.73E-05	0.541
UC	CFDP1	16	75327596	rs9934007	75390532	C	T	0.754	0.273	0.070	9.61E-05	0.973
CD	STAT3	17	40502964	rs1053004	40466092	G	A	0.380	0.702	0.128	4.40E-08	0.056
CD	IMPDH2	3	49064299	rs9813813	49207093	A	T	0.110	-1.233	0.294	2.74E-05	0.213
IBD	IMPDH2	3	49064299	rs9813813	49207093	A	T	0.110	-1.328	0.258	2.57E-07	0.071
UC	IMPDH2	3	49064299	rs9813813	49207093	A	T	0.110	-1.445	0.307	2.53E-06	0.050
IBD	UBE2L3	22	21941029	rs2070512	21949411	C	A	0.194	0.163	0.021	1.06E-14	0.169
UC	UBE2L3	22	21941029	rs2070512	21949411	C	A	0.194	0.116	0.027	1.76E-05	0.698
CD	BCL2L15	1	1.14E+08	rs1217393	1.14E+08	A	G	0.271	-0.705	0.170	3.39E-05	0.184
CD	STMN3	20	62271061	rs909333	62270637	G	T	0.207	0.547	0.102	8.31E-08	0.200
IBD	STMN3	20	62271061	rs909333	62270637	G	T	0.207	0.555	0.087	2.01E-10	0.055
UC	STMN3	20	62271061	rs909333	62270637	G	T	0.207	0.498	0.098	4.04E-07	0.062
GORD	PBX2	6	32154964	rs693906	31835164	C	G	0.113	0.154	0.036	1.65E-05	0.058
VHD	AGER	6	32150423	rs693906	31835164	C	G	0.113	0.212	0.052	4.82E-05	0.092

B) Nominally significant effect on VHD of Bonferroni-significant gastrointestinal loci.

Disease	Gene	chr	Probe_bp	topSNP	topSNP_bp	A1	A2	Freq	b_SMR	se_SMR	p_SMR	p_HEIDI
VHD	CFDP1	16	75397489	rs7201989	75471499	G	A	0.246	-0.056	0.018	0.001	0.119
VHD	STAT3	17	40502964	rs1053004	40466092	G	A	0.380	0.093	0.047	0.048	0.948
VHD	IMPDH2	3	49064299	rs34037363	48821036	T	C	0.114	-0.209	0.105	0.047	0.089
VHD	UBE2L3	22	21941029	rs2070512	21949411	C	A	0.194	-0.029	0.009	0.002	0.623
VHD	STMN3	20	62271061	rs909333	62270637	G	T	0.207	-0.076	0.036	0.036	0.093

C) Effect of AGER gene on VHD and gastrointestinal disorders.

Disease	Gene	chr	Probe_bp	topSNP	topSNP_bp	A1	A2	Freq	b_SMR	se_SMR	p_SMR	p_HEIDI
CD	AGER	6	32150423	rs693906	31835164	C	G	0.113	-0.312	0.120	0.009	6.16E-08
GORD	AGER	6	32150423	rs693906	31835164	C	G	0.113	-0.209	0.049	2.02E-05	0.022
IBD	AGER	6	32148745	rs204995	32154285	G	A	0.217	-0.252	0.108	0.020	1.60E-04
IBS	AGER	6	32150423	rs693906	31835164	C	G	0.113	-0.168	0.052	0.001	0.045
UC	AGER	6	32148745	rs204995	32154285	G	A	0.217	-0.870	0.185	2.59E-06	0.002
VHD	AGER	6	32150423	rs693906	31835164	C	G	0.113	0.212	0.052	4.82E-05	0.092

Supplementary table 6. Results of the summary-based Mendelian randomization integrating whole-blood expression quantitative trait loci. For each disease-gene association, we report the strongest effect observed between GTEX and eQTLGen datasets.

A) Bonferroni-significant disease-gene association with HEIDI P > 0.05.

Disease	Gene	chr	gene_bp	topSNP	topSNP_bp	A1	A2	Freq	b_SMR	se_SMR	p_SMR	p_HEIDI
CD	DNMT3A	2	25510652	rs7578575	25488819	A	T	0.329	-0.834	0.164	3.51E-07	0.383
IBD	DNMT3A	2	25510652	rs7578575	25488819	A	T	0.329	-0.745	0.130	9.22E-09	0.515
UC	DNMT3A	2	25510652	rs7578575	25488819	A	T	0.329	-0.627	0.156	5.73E-05	0.541
UC	CFDP1	16	75327596	rs9934007	75390532	C	T	0.754	0.273	0.070	9.61E-05	0.973
CD	STAT3	17	40502964	rs1053004	40466092	G	A	0.380	0.702	0.128	4.40E-08	0.056
CD	IMPDH2	3	49064299	rs9813813	49207093	A	T	0.110	-1.233	0.294	2.74E-05	0.213
IBD	IMPDH2	3	49064299	rs9813813	49207093	A	T	0.110	-1.328	0.258	2.57E-07	0.071
UC	IMPDH2	3	49064299	rs9813813	49207093	A	T	0.110	-1.445	0.307	2.53E-06	0.050
IBD	UBE2L3	22	21941029	rs2070512	21949411	C	A	0.194	0.163	0.021	1.06E-14	0.169
UC	UBE2L3	22	21941029	rs2070512	21949411	C	A	0.194	0.116	0.027	1.76E-05	0.698
CD	BCL2L15	1	1.14E+08	rs1217393	1.14E+08	A	G	0.271	-0.705	0.170	3.39E-05	0.184
CD	STMN3	20	62271061	rs909333	62270637	G	T	0.207	0.547	0.102	8.31E-08	0.200
IBD	STMN3	20	62271061	rs909333	62270637	G	T	0.207	0.555	0.087	2.01E-10	0.055
UC	STMN3	20	62271061	rs909333	62270637	G	T	0.207	0.498	0.098	4.04E-07	0.062
GORD	PBX2	6	32154964	rs693906	31835164	C	G	0.113	0.154	0.036	1.65E-05	0.058
VHD	AGER	6	32150423	rs693906	31835164	C	G	0.113	0.212	0.052	4.82E-05	0.092

B) Nominally significant effect on VHD of Bonferroni-significant gastrointestinal loci.

Disease	Gene	chr	Probe_bp	topSNP	topSNP_bp	A1	A2	Freq	b_SMR	se_SMR	p_SMR	p_HEIDI
VHD	CFDP1	16	75397489	rs7201989	75471499	G	A	0.246	-0.056	0.018	0.001	0.119
VHD	STAT3	17	40502964	rs1053004	40466092	G	A	0.380	0.093	0.047	0.048	0.948
VHD	IMPDH2	3	49064299	rs34037363	48821036	T	C	0.114	-0.209	0.105	0.047	0.089
VHD	UBE2L3	22	21941029	rs2070512	21949411	C	A	0.194	-0.029	0.009	0.002	0.623
VHD	STMN3	20	62271061	rs909333	62270637	G	T	0.207	-0.076	0.036	0.036	0.093

C) Effect of AGER gene on VHD and gastrointestinal disorders.

Disease	Gene	chr	Probe_bp	topSNP	topSNP_bp	A1	A2	Freq	b_SMR	se_SMR	p_SMR	p_HEIDI
CD	AGER	6	32150423	rs693906	31835164	C	G	0.113	-0.312	0.120	0.009	6.16E-08
GORD	AGER	6	32150423	rs693906	31835164	C	G	0.113	-0.209	0.049	2.02E-05	0.022
IBD	AGER	6	32148745	rs204995	32154285	G	A	0.217	-0.252	0.108	0.020	1.60E-04
IBS	AGER	6	32150423	rs693906	31835164	C	G	0.113	-0.168	0.052	0.001	0.045
UC	AGER	6	32148745	rs204995	32154285	G	A	0.217	-0.870	0.185	2.59E-06	0.002
VHD	AGER	6	32150423	rs693906	31835164	C	G	0.113	0.212	0.052	4.82E-05	0.092

Supplementary table 6. Results of the summary-based Mendelian randomization integrating whole-blood expression quantitative trait loci. For each disease-gene association, we report the strongest effect observed between GTEX and eQTLGen datasets.

A) Bonferroni-significant disease-gene association with HEIDI P > 0.05.

Disease	Gene	chr	gene_bp	topSNP	topSNP_bp	A1	A2	Freq	b_SMR	se_SMR	p_SMR	p_HEIDI
CD	DNMT3A	2	25510652	rs7578575	25488819	A	T	0.329	-0.834	0.164	3.51E-07	0.383
IBD	DNMT3A	2	25510652	rs7578575	25488819	A	T	0.329	-0.745	0.130	9.22E-09	0.515
UC	DNMT3A	2	25510652	rs7578575	25488819	A	T	0.329	-0.627	0.156	5.73E-05	0.541
UC	CFDP1	16	75327596	rs9934007	75390532	C	T	0.754	0.273	0.070	9.61E-05	0.973
CD	STAT3	17	40502964	rs1053004	40466092	G	A	0.380	0.702	0.128	4.40E-08	0.056
CD	IMPDH2	3	49064299	rs9813813	49207093	A	T	0.110	-1.233	0.294	2.74E-05	0.213
IBD	IMPDH2	3	49064299	rs9813813	49207093	A	T	0.110	-1.328	0.258	2.57E-07	0.071
UC	IMPDH2	3	49064299	rs9813813	49207093	A	T	0.110	-1.445	0.307	2.53E-06	0.050
IBD	UBE2L3	22	21941029	rs2070512	21949411	C	A	0.194	0.163	0.021	1.06E-14	0.169
UC	UBE2L3	22	21941029	rs2070512	21949411	C	A	0.194	0.116	0.027	1.76E-05	0.698
CD	BCL2L15	1	1.14E+08	rs1217393	1.14E+08	A	G	0.271	-0.705	0.170	3.39E-05	0.184
CD	STMN3	20	62271061	rs909333	62270637	G	T	0.207	0.547	0.102	8.31E-08	0.200
IBD	STMN3	20	62271061	rs909333	62270637	G	T	0.207	0.555	0.087	2.01E-10	0.055
UC	STMN3	20	62271061	rs909333	62270637	G	T	0.207	0.498	0.098	4.04E-07	0.062
GORD	PBX2	6	32154964	rs693906	31835164	C	G	0.113	0.154	0.036	1.65E-05	0.058
VHD	AGER	6	32150423	rs693906	31835164	C	G	0.113	0.212	0.052	4.82E-05	0.092

B) Nominally significant effect on VHD of Bonferroni-significant gastrointestinal loci.

Disease	Gene	chr	Probe_bp	topSNP	topSNP_bp	A1	A2	Freq	b_SMR	se_SMR	p_SMR	p_HEIDI
VHD	CFDP1	16	75397489	rs7201989	75471499	G	A	0.246	-0.056	0.018	0.001	0.119
VHD	STAT3	17	40502964	rs1053004	40466092	G	A	0.380	0.093	0.047	0.048	0.948
VHD	IMPDH2	3	49064299	rs34037363	48821036	T	C	0.114	-0.209	0.105	0.047	0.089
VHD	UBE2L3	22	21941029	rs2070512	21949411	C	A	0.194	-0.029	0.009	0.002	0.623
VHD	STMN3	20	62271061	rs909333	62270637	G	T	0.207	-0.076	0.036	0.036	0.093

C) Effect of AGER gene on VHD and gastrointestinal disorders.

Disease	Gene	chr	Probe_bp	topSNP	topSNP_bp	A1	A2	Freq	b_SMR	se_SMR	p_SMR	p_HEIDI
CD	AGER	6	32150423	rs693906	31835164	C	G	0.113	-0.312	0.120	0.009	6.16E-08
GORD	AGER	6	32150423	rs693906	31835164	C	G	0.113	-0.209	0.049	2.02E-05	0.022
IBD	AGER	6	32148745	rs204995	32154285	G	A	0.217	-0.252	0.108	0.020	1.60E-04
IBS	AGER	6	32150423	rs693906	31835164	C	G	0.113	-0.168	0.052	0.001	0.045
UC	AGER	6	32148745	rs204995	32154285	G	A	0.217	-0.870	0.185	2.59E-06	0.002
VHD	AGER	6	32150423	rs693906	31835164	C	G	0.113	0.212	0.052	4.82E-05	0.092

Supplementary table 6. Results of the summary-based Mendelian randomization integrating whole-blood expression quantitative trait loci. For each disease-gene association, we report the strongest effect observed between GTEX and eQTLGen datasets.

A) Bonferroni-significant disease-gene association with HEIDI P > 0.05.

Disease	Gene	chr	gene_bp	topSNP	topSNP_bp	A1	A2	Freq	b_SMR	se_SMR	p_SMR	p_HEIDI
CD	DNMT3A	2	25510652	rs7578575	25488819	A	T	0.329	-0.834	0.164	3.51E-07	0.383
IBD	DNMT3A	2	25510652	rs7578575	25488819	A	T	0.329	-0.745	0.130	9.22E-09	0.515
UC	DNMT3A	2	25510652	rs7578575	25488819	A	T	0.329	-0.627	0.156	5.73E-05	0.541
UC	CFDP1	16	75327596	rs9934007	75390532	C	T	0.754	0.273	0.070	9.61E-05	0.973
CD	STAT3	17	40502964	rs1053004	40466092	G	A	0.380	0.702	0.128	4.40E-08	0.056
CD	IMPDH2	3	49064299	rs9813813	49207093	A	T	0.110	-1.233	0.294	2.74E-05	0.213
IBD	IMPDH2	3	49064299	rs9813813	49207093	A	T	0.110	-1.328	0.258	2.57E-07	0.071
UC	IMPDH2	3	49064299	rs9813813	49207093	A	T	0.110	-1.445	0.307	2.53E-06	0.050
IBD	UBE2L3	22	21941029	rs2070512	21949411	C	A	0.194	0.163	0.021	1.06E-14	0.169
UC	UBE2L3	22	21941029	rs2070512	21949411	C	A	0.194	0.116	0.027	1.76E-05	0.698
CD	BCL2L15	1	1.14E+08	rs1217393	1.14E+08	A	G	0.271	-0.705	0.170	3.39E-05	0.184
CD	STMN3	20	62271061	rs909333	62270637	G	T	0.207	0.547	0.102	8.31E-08	0.200
IBD	STMN3	20	62271061	rs909333	62270637	G	T	0.207	0.555	0.087	2.01E-10	0.055
UC	STMN3	20	62271061	rs909333	62270637	G	T	0.207	0.498	0.098	4.04E-07	0.062
GORD	PBX2	6	32154964	rs693906	31835164	C	G	0.113	0.154	0.036	1.65E-05	0.058
VHD	AGER	6	32150423	rs693906	31835164	C	G	0.113	0.212	0.052	4.82E-05	0.092

B) Nominally significant effect on VHD of Bonferroni-significant gastrointestinal loci.

Disease	Gene	chr	Probe_bp	topSNP	topSNP_bp	A1	A2	Freq	b_SMR	se_SMR	p_SMR	p_HEIDI
VHD	CFDP1	16	75397489	rs7201989	75471499	G	A	0.246	-0.056	0.018	0.001	0.119
VHD	STAT3	17	40502964	rs1053004	40466092	G	A	0.380	0.093	0.047	0.048	0.948
VHD	IMPDH2	3	49064299	rs34037363	48821036	T	C	0.114	-0.209	0.105	0.047	0.089
VHD	UBE2L3	22	21941029	rs2070512	21949411	C	A	0.194	-0.029	0.009	0.002	0.623
VHD	STMN3	20	62271061	rs909333	62270637	G	T	0.207	-0.076	0.036	0.036	0.093

C) Effect of AGER gene on VHD and gastrointestinal disorders.

Disease	Gene	chr	Probe_bp	topSNP	topSNP_bp	A1	A2	Freq	b_SMR	se_SMR	p_SMR	p_HEIDI
CD	AGER	6	32150423	rs693906	31835164	C	G	0.113	-0.312	0.120	0.009	6.16E-08
GORD	AGER	6	32150423	rs693906	31835164	C	G	0.113	-0.209	0.049	2.02E-05	0.022
IBD	AGER	6	32148745	rs204995	32154285	G	A	0.217	-0.252	0.108	0.020	1.60E-04
IBS	AGER	6	32150423	rs693906	31835164	C	G	0.113	-0.168	0.052	0.001	0.045
UC	AGER	6	32148745	rs204995	32154285	G	A	0.217	-0.870	0.185	2.59E-06	0.002
VHD	AGER	6	32150423	rs693906	31835164	C	G	0.113	0.212	0.052	4.82E-05	0.092

Supplementary table 6. Results of the summary-based Mendelian randomization integrating whole-blood expression quantitative trait loci. For each disease-gene association, we report the strongest effect observed between GTEX and eQTLGen datasets.

A) Bonferroni-significant disease-gene association with HEIDI P > 0.05.

Disease	Gene	chr	gene_bp	topSNP	topSNP_bp	A1	A2	Freq	b_SMR	se_SMR	p_SMR	p_HEIDI
CD	DNMT3A	2	25510652	rs7578575	25488819	A	T	0.329	-0.834	0.164	3.51E-07	0.383
IBD	DNMT3A	2	25510652	rs7578575	25488819	A	T	0.329	-0.745	0.130	9.22E-09	0.515
UC	DNMT3A	2	25510652	rs7578575	25488819	A	T	0.329	-0.627	0.156	5.73E-05	0.541
UC	CFDP1	16	75327596	rs9934007	75390532	C	T	0.754	0.273	0.070	9.61E-05	0.973
CD	STAT3	17	40502964	rs1053004	40466092	G	A	0.380	0.702	0.128	4.40E-08	0.056
CD	IMPDH2	3	49064299	rs9813813	49207093	A	T	0.110	-1.233	0.294	2.74E-05	0.213
IBD	IMPDH2	3	49064299	rs9813813	49207093	A	T	0.110	-1.328	0.258	2.57E-07	0.071
UC	IMPDH2	3	49064299	rs9813813	49207093	A	T	0.110	-1.445	0.307	2.53E-06	0.050
IBD	UBE2L3	22	21941029	rs2070512	21949411	C	A	0.194	0.163	0.021	1.06E-14	0.169
UC	UBE2L3	22	21941029	rs2070512	21949411	C	A	0.194	0.116	0.027	1.76E-05	0.698
CD	BCL2L15	1	1.14E+08	rs1217393	1.14E+08	A	G	0.271	-0.705	0.170	3.39E-05	0.184
CD	STMN3	20	62271061	rs909333	62270637	G	T	0.207	0.547	0.102	8.31E-08	0.200
IBD	STMN3	20	62271061	rs909333	62270637	G	T	0.207	0.555	0.087	2.01E-10	0.055
UC	STMN3	20	62271061	rs909333	62270637	G	T	0.207	0.498	0.098	4.04E-07	0.062
GORD	PBX2	6	32154964	rs693906	31835164	C	G	0.113	0.154	0.036	1.65E-05	0.058
VHD	AGER	6	32150423	rs693906	31835164	C	G	0.113	0.212	0.052	4.82E-05	0.092

B) Nominally significant effect on VHD of Bonferroni-significant gastrointestinal loci.

Disease	Gene	chr	Probe_bp	topSNP	topSNP_bp	A1	A2	Freq	b_SMR	se_SMR	p_SMR	p_HEIDI
VHD	CFDP1	16	75397489	rs7201989	75471499	G	A	0.246	-0.056	0.018	0.001	0.119
VHD	STAT3	17	40502964	rs1053004	40466092	G	A	0.380	0.093	0.047	0.048	0.948
VHD	IMPDH2	3	49064299	rs34037363	48821036	T	C	0.114	-0.209	0.105	0.047	0.089
VHD	UBE2L3	22	21941029	rs2070512	21949411	C	A	0.194	-0.029	0.009	0.002	0.623
VHD	STMN3	20	62271061	rs909333	62270637	G	T	0.207	-0.076	0.036	0.036	0.093

C) Effect of AGER gene on VHD and gastrointestinal disorders.

Disease	Gene	chr	Probe_bp	topSNP	topSNP_bp	A1	A2	Freq	b_SMR	se_SMR	p_SMR	p_HEIDI
CD	AGER	6	32150423	rs693906	31835164	C	G	0.113	-0.312	0.120	0.009	6.16E-08
GORD	AGER	6	32150423	rs693906	31835164	C	G	0.113	-0.209	0.049	2.02E-05	0.022
IBD	AGER	6	32148745	rs204995	32154285	G	A	0.217	-0.252	0.108	0.020	1.60E-04
IBS	AGER	6	32150423	rs693906	31835164	C	G	0.113	-0.168	0.052	0.001	0.045
UC	AGER	6	32148745	rs204995	32154285	G	A	0.217	-0.870	0.185	2.59E-06	0.002
VHD	AGER	6	32150423	rs693906	31835164	C	G	0.113	0.212	0.052	4.82E-05	0.092

Supplementary table 6. Results of the summary-based Mendelian randomization integrating whole-blood expression quantitative trait loci. For each disease-gene association, we report the strongest effect observed between GTEX and eQTLGen datasets.

A) Bonferroni-significant disease-gene association with HEIDI P > 0.05.

Disease	Gene	chr	gene_bp	topSNP	topSNP_bp	A1	A2	Freq	b_SMR	se_SMR	p_SMR	p_HEIDI
CD	DNMT3A	2	25510652	rs7578575	25488819	A	T	0.329	-0.834	0.164	3.51E-07	0.383
IBD	DNMT3A	2	25510652	rs7578575	25488819	A	T	0.329	-0.745	0.130	9.22E-09	0.515
UC	DNMT3A	2	25510652	rs7578575	25488819	A	T	0.329	-0.627	0.156	5.73E-05	0.541
UC	CFDP1	16	75327596	rs9934007	75390532	C	T	0.754	0.273	0.070	9.61E-05	0.973
CD	STAT3	17	40502964	rs1053004	40466092	G	A	0.380	0.702	0.128	4.40E-08	0.056
CD	IMPDH2	3	49064299	rs9813813	49207093	A	T	0.110	-1.233	0.294	2.74E-05	0.213
IBD	IMPDH2	3	49064299	rs9813813	49207093	A	T	0.110	-1.328	0.258	2.57E-07	0.071
UC	IMPDH2	3	49064299	rs9813813	49207093	A	T	0.110	-1.445	0.307	2.53E-06	0.050
IBD	UBE2L3	22	21941029	rs2070512	21949411	C	A	0.194	0.163	0.021	1.06E-14	0.169
UC	UBE2L3	22	21941029	rs2070512	21949411	C	A	0.194	0.116	0.027	1.76E-05	0.698
CD	BCL2L15	1	1.14E+08	rs1217393	1.14E+08	A	G	0.271	-0.705	0.170	3.39E-05	0.184
CD	STMN3	20	62271061	rs909333	62270637	G	T	0.207	0.547	0.102	8.31E-08	0.200
IBD	STMN3	20	62271061	rs909333	62270637	G	T	0.207	0.555	0.087	2.01E-10	0.055
UC	STMN3	20	62271061	rs909333	62270637	G	T	0.207	0.498	0.098	4.04E-07	0.062
GORD	PBX2	6	32154964	rs693906	31835164	C	G	0.113	0.154	0.036	1.65E-05	0.058
VHD	AGER	6	32150423	rs693906	31835164	C	G	0.113	0.212	0.052	4.82E-05	0.092

B) Nominally significant effect on VHD of Bonferroni-significant gastrointestinal loci.

Disease	Gene	chr	Probe_bp	topSNP	topSNP_bp	A1	A2	Freq	b_SMR	se_SMR	p_SMR	p_HEIDI
VHD	CFDP1	16	75397489	rs7201989	75471499	G	A	0.246	-0.056	0.018	0.001	0.119
VHD	STAT3	17	40502964	rs1053004	40466092	G	A	0.380	0.093	0.047	0.048	0.948
VHD	IMPDH2	3	49064299	rs34037363	48821036	T	C	0.114	-0.209	0.105	0.047	0.089
VHD	UBE2L3	22	21941029	rs2070512	21949411	C	A	0.194	-0.029	0.009	0.002	0.623
VHD	STMN3	20	62271061	rs909333	62270637	G	T	0.207	-0.076	0.036	0.036	0.093

C) Effect of AGER gene on VHD and gastrointestinal disorders.

Disease	Gene	chr	Probe_bp	topSNP	topSNP_bp	A1	A2	Freq	b_SMR	se_SMR	p_SMR	p_HEIDI
CD	AGER	6	32150423	rs693906	31835164	C	G	0.113	-0.312	0.120	0.009	6.16E-08
GORD	AGER	6	32150423	rs693906	31835164	C	G	0.113	-0.209	0.049	2.02E-05	0.022
IBD	AGER	6	32148745	rs204995	32154285	G	A	0.217	-0.252	0.108	0.020	1.60E-04
IBS	AGER	6	32150423	rs693906	31835164	C	G	0.113	-0.168	0.052	0.001	0.045
UC	AGER	6	32148745	rs204995	32154285	G	A	0.217	-0.870	0.185	2.59E-06	0.002
VHD	AGER	6	32150423	rs693906	31835164	C	G	0.113	0.212	0.052	4.82E-05	0.092

Supplementary table 6. Results of the summary-based Mendelian randomization integrating whole-blood expression quantitative trait loci. For each disease-gene association, we report the strongest effect observed between GTEX and eQTLGen datasets.

A) Bonferroni-significant disease-gene association with HEIDI P > 0.05.

Disease	Gene	chr	gene_bp	topSNP	topSNP_bp	A1	A2	Freq	b_SMR	se_SMR	p_SMR	p_HEIDI
CD	DNMT3A	2	25510652	rs7578575	25488819	A	T	0.329	-0.834	0.164	3.51E-07	0.383
IBD	DNMT3A	2	25510652	rs7578575	25488819	A	T	0.329	-0.745	0.130	9.22E-09	0.515
UC	DNMT3A	2	25510652	rs7578575	25488819	A	T	0.329	-0.627	0.156	5.73E-05	0.541
UC	CFDP1	16	75327596	rs9934007	75390532	C	T	0.754	0.273	0.070	9.61E-05	0.973
CD	STAT3	17	40502964	rs1053004	40466092	G	A	0.380	0.702	0.128	4.40E-08	0.056
CD	IMPDH2	3	49064299	rs9813813	49207093	A	T	0.110	-1.233	0.294	2.74E-05	0.213
IBD	IMPDH2	3	49064299	rs9813813	49207093	A	T	0.110	-1.328	0.258	2.57E-07	0.071
UC	IMPDH2	3	49064299	rs9813813	49207093	A	T	0.110	-1.445	0.307	2.53E-06	0.050
IBD	UBE2L3	22	21941029	rs2070512	21949411	C	A	0.194	0.163	0.021	1.06E-14	0.169
UC	UBE2L3	22	21941029	rs2070512	21949411	C	A	0.194	0.116	0.027	1.76E-05	0.698
CD	BCL2L15	1	1.14E+08	rs1217393	1.14E+08	A	G	0.271	-0.705	0.170	3.39E-05	0.184
CD	STMN3	20	62271061	rs909333	62270637	G	T	0.207	0.547	0.102	8.31E-08	0.200
IBD	STMN3	20	62271061	rs909333	62270637	G	T	0.207	0.555	0.087	2.01E-10	0.055
UC	STMN3	20	62271061	rs909333	62270637	G	T	0.207	0.498	0.098	4.04E-07	0.062
GORD	PBX2	6	32154964	rs693906	31835164	C	G	0.113	0.154	0.036	1.65E-05	0.058
VHD	AGER	6	32150423	rs693906	31835164	C	G	0.113	0.212	0.052	4.82E-05	0.092

B) Nominally significant effect on VHD of Bonferroni-significant gastrointestinal loci.

Disease	Gene	chr	Probe_bp	topSNP	topSNP_bp	A1	A2	Freq	b_SMR	se_SMR	p_SMR	p_HEIDI
VHD	CFDP1	16	75397489	rs7201989	75471499	G	A	0.246	-0.056	0.018	0.001	0.119
VHD	STAT3	17	40502964	rs1053004	40466092	G	A	0.380	0.093	0.047	0.048	0.948
VHD	IMPDH2	3	49064299	rs34037363	48821036	T	C	0.114	-0.209	0.105	0.047	0.089
VHD	UBE2L3	22	21941029	rs2070512	21949411	C	A	0.194	-0.029	0.009	0.002	0.623
VHD	STMN3	20	62271061	rs909333	62270637	G	T	0.207	-0.076	0.036	0.036	0.093

C) Effect of AGER gene on VHD and gastrointestinal disorders.

Disease	Gene	chr	Probe_bp	topSNP	topSNP_bp	A1	A2	Freq	b_SMR	se_SMR	p_SMR	p_HEIDI
CD	AGER	6	32150423	rs693906	31835164	C	G	0.113	-0.312	0.120	0.009	6.16E-08
GORD	AGER	6	32150423	rs693906	31835164	C	G	0.113	-0.209	0.049	2.02E-05	0.022
IBD	AGER	6	32148745	rs204995	32154285	G	A	0.217	-0.252	0.108	0.020	1.60E-04
IBS	AGER	6	32150423	rs693906	31835164	C	G	0.113	-0.168	0.052	0.001	0.045
UC	AGER	6	32148745	rs204995	32154285	G	A	0.217	-0.870	0.185	2.59E-06	0.002
VHD	AGER	6	32150423	rs693906	31835164	C	G	0.113	0.212	0.052	4.82E-05	0.092

Supplementary table 6. Results of the summary-based Mendelian randomization integrating whole-blood expression quantitative trait loci. For each disease-gene association, we report the strongest effect observed between GTEX and eQTLGen datasets.

A) Bonferroni-significant disease-gene association with HEIDI P > 0.05.

Disease	Gene	chr	gene_bp	topSNP	topSNP_bp	A1	A2	Freq	b_SMR	se_SMR	p_SMR	p_HEIDI
CD	DNMT3A	2	25510652	rs7578575	25488819	A	T	0.329	-0.834	0.164	3.51E-07	0.383
IBD	DNMT3A	2	25510652	rs7578575	25488819	A	T	0.329	-0.745	0.130	9.22E-09	0.515
UC	DNMT3A	2	25510652	rs7578575	25488819	A	T	0.329	-0.627	0.156	5.73E-05	0.541
UC	CFDP1	16	75327596	rs9934007	75390532	C	T	0.754	0.273	0.070	9.61E-05	0.973
CD	STAT3	17	40502964	rs1053004	40466092	G	A	0.380	0.702	0.128	4.40E-08	0.056
CD	IMPDH2	3	49064299	rs9813813	49207093	A	T	0.110	-1.233	0.294	2.74E-05	0.213
IBD	IMPDH2	3	49064299	rs9813813	49207093	A	T	0.110	-1.328	0.258	2.57E-07	0.071
UC	IMPDH2	3	49064299	rs9813813	49207093	A	T	0.110	-1.445	0.307	2.53E-06	0.050
IBD	UBE2L3	22	21941029	rs2070512	21949411	C	A	0.194	0.163	0.021	1.06E-14	0.169
UC	UBE2L3	22	21941029	rs2070512	21949411	C	A	0.194	0.116	0.027	1.76E-05	0.698
CD	BCL2L15	1	1.14E+08	rs1217393	1.14E+08	A	G	0.271	-0.705	0.170	3.39E-05	0.184
CD	STMN3	20	62271061	rs909333	62270637	G	T	0.207	0.547	0.102	8.31E-08	0.200
IBD	STMN3	20	62271061	rs909333	62270637	G	T	0.207	0.555	0.087	2.01E-10	0.055
UC	STMN3	20	62271061	rs909333	62270637	G	T	0.207	0.498	0.098	4.04E-07	0.062
GORD	PBX2	6	32154964	rs693906	31835164	C	G	0.113	0.154	0.036	1.65E-05	0.058
VHD	AGER	6	32150423	rs693906	31835164	C	G	0.113	0.212	0.052	4.82E-05	0.092

B) Nominally significant effect on VHD of Bonferroni-significant gastrointestinal loci.

Disease	Gene	chr	Probe_bp	topSNP	topSNP_bp	A1	A2	Freq	b_SMR	se_SMR	p_SMR	p_HEIDI
VHD	CFDP1	16	75397489	rs7201989	75471499	G	A	0.246	-0.056	0.018	0.001	0.119
VHD	STAT3	17	40502964	rs1053004	40466092	G	A	0.380	0.093	0.047	0.048	0.948
VHD	IMPDH2	3	49064299	rs34037363	48821036	T	C	0.114	-0.209	0.105	0.047	0.089
VHD	UBE2L3	22	21941029	rs2070512	21949411	C	A	0.194	-0.029	0.009	0.002	0.623
VHD	STMN3	20	62271061	rs909333	62270637	G	T	0.207	-0.076	0.036	0.036	0.093

C) Effect of AGER gene on VHD and gastrointestinal disorders.

Disease	Gene	chr	Probe_bp	topSNP	topSNP_bp	A1	A2	Freq	b_SMR	se_SMR	p_SMR	p_HEIDI
CD	AGER	6	32150423	rs693906	31835164	C	G	0.113	-0.312	0.120	0.009	6.16E-08
GORD	AGER	6	32150423	rs693906	31835164	C	G	0.113	-0.209	0.049	2.02E-05	0.022
IBD	AGER	6	32148745	rs204995	32154285	G	A	0.217	-0.252	0.108	0.020	1.60E-04
IBS	AGER	6	32150423	rs693906	31835164	C	G	0.113	-0.168	0.052	0.001	0.045
UC	AGER	6	32148745	rs204995	32154285	G	A	0.217	-0.870	0.185	2.59E-06	0.002
VHD	AGER	6	32150423	rs693906	31835164	C	G	0.113	0.212	0.052	4.82E-05	0.092

Supplementary table 6. Results of the summary-based Mendelian randomization integrating whole-blood expression quantitative trait loci. For each disease-gene association, we report the strongest effect observed between GTEX and eQTLGen datasets.

A) Bonferroni-significant disease-gene association with HEIDI P > 0.05.

Disease	Gene	chr	gene_bp	topSNP	topSNP_bp	A1	A2	Freq	b_SMR	se_SMR	p_SMR	p_HEIDI
CD	DNMT3A	2	25510652	rs7578575	25488819	A	T	0.329	-0.834	0.164	3.51E-07	0.383
IBD	DNMT3A	2	25510652	rs7578575	25488819	A	T	0.329	-0.745	0.130	9.22E-09	0.515
UC	DNMT3A	2	25510652	rs7578575	25488819	A	T	0.329	-0.627	0.156	5.73E-05	0.541
UC	CFDP1	16	75327596	rs9934007	75390532	C	T	0.754	0.273	0.070	9.61E-05	0.973
CD	STAT3	17	40502964	rs1053004	40466092	G	A	0.380	0.702	0.128	4.40E-08	0.056
CD	IMPDH2	3	49064299	rs9813813	49207093	A	T	0.110	-1.233	0.294	2.74E-05	0.213
IBD	IMPDH2	3	49064299	rs9813813	49207093	A	T	0.110	-1.328	0.258	2.57E-07	0.071
UC	IMPDH2	3	49064299	rs9813813	49207093	A	T	0.110	-1.445	0.307	2.53E-06	0.050
IBD	UBE2L3	22	21941029	rs2070512	21949411	C	A	0.194	0.163	0.021	1.06E-14	0.169
UC	UBE2L3	22	21941029	rs2070512	21949411	C	A	0.194	0.116	0.027	1.76E-05	0.698
CD	BCL2L15	1	1.14E+08	rs1217393	1.14E+08	A	G	0.271	-0.705	0.170	3.39E-05	0.184
CD	STMN3	20	62271061	rs909333	62270637	G	T	0.207	0.547	0.102	8.31E-08	0.200
IBD	STMN3	20	62271061	rs909333	62270637	G	T	0.207	0.555	0.087	2.01E-10	0.055
UC	STMN3	20	62271061	rs909333	62270637	G	T	0.207	0.498	0.098	4.04E-07	0.062
GORD	PBX2	6	32154964	rs693906	31835164	C	G	0.113	0.154	0.036	1.65E-05	0.058
VHD	AGER	6	32150423	rs693906	31835164	C	G	0.113	0.212	0.052	4.82E-05	0.092

B) Nominally significant effect on VHD of Bonferroni-significant gastrointestinal loci.

Disease	Gene	chr	Probe_bp	topSNP	topSNP_bp	A1	A2	Freq	b_SMR	se_SMR	p_SMR	p_HEIDI
VHD	CFDP1	16	75397489	rs7201989	75471499	G	A	0.246	-0.056	0.018	0.001	0.119
VHD	STAT3	17	40502964	rs1053004	40466092	G	A	0.380	0.093	0.047	0.048	0.948
VHD	IMPDH2	3	49064299	rs34037363	48821036	T	C	0.114	-0.209	0.105	0.047	0.089
VHD	UBE2L3	22	21941029	rs2070512	21949411	C	A	0.194	-0.029	0.009	0.002	0.623
VHD	STMN3	20	62271061	rs909333	62270637	G	T	0.207	-0.076	0.036	0.036	0.093

C) Effect of AGER gene on VHD and gastrointestinal disorders.

Disease	Gene	chr	Probe_bp	topSNP	topSNP_bp	A1	A2	Freq	b_SMR	se_SMR	p_SMR	p_HEIDI
CD	AGER	6	32150423	rs693906	31835164	C	G	0.113	-0.312	0.120	0.009	6.16E-08
GORD	AGER	6	32150423	rs693906	31835164	C	G	0.113	-0.209	0.049	2.02E-05	0.022
IBD	AGER	6	32148745	rs204995	32154285	G	A	0.217	-0.252	0.108	0.020	1.60E-04
IBS	AGER	6	32150423	rs693906	31835164	C	G	0.113	-0.168	0.052	0.001	0.045
UC	AGER	6	32148745	rs204995	32154285	G	A	0.217	-0.870	0.185	2.59E-06	0.002
VHD	AGER	6	32150423	rs693906	31835164	C	G	0.113	0.212	0.052	4.82E-05	0.092

Supplementary table 6. Results of the summary-based Mendelian randomization integrating whole-blood expression quantitative trait loci. For each disease-gene association, we report the strongest effect observed between GTEX and eQTLGen datasets.

A) Bonferroni-significant disease-gene association with HEIDI P > 0.05.

Disease	Gene	chr	gene_bp	topSNP	topSNP_bp	A1	A2	Freq	b_SMR	se_SMR	p_SMR	p_HEIDI
CD	DNMT3A	2	25510652	rs7578575	25488819	A	T	0.329	-0.834	0.164	3.51E-07	0.383
IBD	DNMT3A	2	25510652	rs7578575	25488819	A	T	0.329	-0.745	0.130	9.22E-09	0.515
UC	DNMT3A	2	25510652	rs7578575	25488819	A	T	0.329	-0.627	0.156	5.73E-05	0.541
UC	CFDP1	16	75327596	rs9934007	75390532	C	T	0.754	0.273	0.070	9.61E-05	0.973
CD	STAT3	17	40502964	rs1053004	40466092	G	A	0.380	0.702	0.128	4.40E-08	0.056
CD	IMPDH2	3	49064299	rs9813813	49207093	A	T	0.110	-1.233	0.294	2.74E-05	0.213
IBD	IMPDH2	3	49064299	rs9813813	49207093	A	T	0.110	-1.328	0.258	2.57E-07	0.071
UC	IMPDH2	3	49064299	rs9813813	49207093	A	T	0.110	-1.445	0.307	2.53E-06	0.050
IBD	UBE2L3	22	21941029	rs2070512	21949411	C	A	0.194	0.163	0.021	1.06E-14	0.169
UC	UBE2L3	22	21941029	rs2070512	21949411	C	A	0.194	0.116	0.027	1.76E-05	0.698
CD	BCL2L15	1	1.14E+08	rs1217393	1.14E+08	A	G	0.271	-0.705	0.170	3.39E-05	0.184
CD	STMN3	20	62271061	rs909333	62270637	G	T	0.207	0.547	0.102	8.31E-08	0.200
IBD	STMN3	20	62271061	rs909333	62270637	G	T	0.207	0.555	0.087	2.01E-10	0.055
UC	STMN3	20	62271061	rs909333	62270637	G	T	0.207	0.498	0.098	4.04E-07	0.062
GORD	PBX2	6	32154964	rs693906	31835164	C	G	0.113	0.154	0.036	1.65E-05	0.058
VHD	AGER	6	32150423	rs693906	31835164	C	G	0.113	0.212	0.052	4.82E-05	0.092

B) Nominally significant effect on VHD of Bonferroni-significant gastrointestinal loci.

Disease	Gene	chr	Probe_bp	topSNP	topSNP_bp	A1	A2	Freq	b_SMR	se_SMR	p_SMR	p_HEIDI
VHD	CFDP1	16	75397489	rs7201989	75471499	G	A	0.246	-0.056	0.018	0.001	0.119
VHD	STAT3	17	40502964	rs1053004	40466092	G	A	0.380	0.093	0.047	0.048	0.948
VHD	IMPDH2	3	49064299	rs34037363	48821036	T	C	0.114	-0.209	0.105	0.047	0.089
VHD	UBE2L3	22	21941029	rs2070512	21949411	C	A	0.194	-0.029	0.009	0.002	0.623
VHD	STMN3	20	62271061	rs909333	62270637	G	T	0.207	-0.076	0.036	0.036	0.093

C) Effect of AGER gene on VHD and gastrointestinal disorders.

Disease	Gene	chr	Probe_bp	topSNP	topSNP_bp	A1	A2	Freq	b_SMR	se_SMR	p_SMR	p_HEIDI
CD	AGER	6	32150423	rs693906	31835164	C	G	0.113	-0.312	0.120	0.009	6.16E-08
GORD	AGER	6	32150423	rs693906	31835164	C	G	0.113	-0.209	0.049	2.02E-05	0.022
IBD	AGER	6	32148745	rs204995	32154285	G	A	0.217	-0.252	0.108	0.020	1.60E-04
IBS	AGER	6	32150423	rs693906	31835164	C	G	0.113	-0.168	0.052	0.001	0.045
UC	AGER	6	32148745	rs204995	32154285	G	A	0.217	-0.870	0.185	2.59E-06	0.002
VHD	AGER	6	32150423	rs693906	31835164	C	G	0.113	0.212	0.052	4.82E-05	0.092

Supplementary table 6. Results of the summary-based Mendelian randomization integrating whole-blood expression quantitative trait loci. For each disease-gene association, we report the strongest effect observed between GTEX and eQTLGen datasets.

A) Bonferroni-significant disease-gene association with HEIDI P > 0.05.

Disease	Gene	chr	gene_bp	topSNP	topSNP_bp	A1	A2	Freq	b_SMR	se_SMR	p_SMR	p_HEIDI
CD	DNMT3A	2	25510652	rs7578575	25488819	A	T	0.329	-0.834	0.164	3.51E-07	0.383
IBD	DNMT3A	2	25510652	rs7578575	25488819	A	T	0.329	-0.745	0.130	9.22E-09	0.515
UC	DNMT3A	2	25510652	rs7578575	25488819	A	T	0.329	-0.627	0.156	5.73E-05	0.541
UC	CFDP1	16	75327596	rs9934007	75390532	C	T	0.754	0.273	0.070	9.61E-05	0.973
CD	STAT3	17	40502964	rs1053004	40466092	G	A	0.380	0.702	0.128	4.40E-08	0.056
CD	IMPDH2	3	49064299	rs9813813	49207093	A	T	0.110	-1.233	0.294	2.74E-05	0.213
IBD	IMPDH2	3	49064299	rs9813813	49207093	A	T	0.110	-1.328	0.258	2.57E-07	0.071
UC	IMPDH2	3	49064299	rs9813813	49207093	A	T	0.110	-1.445	0.307	2.53E-06	0.050
IBD	UBE2L3	22	21941029	rs2070512	21949411	C	A	0.194	0.163	0.021	1.06E-14	0.169
UC	UBE2L3	22	21941029	rs2070512	21949411	C	A	0.194	0.116	0.027	1.76E-05	0.698
CD	BCL2L15	1	1.14E+08	rs1217393	1.14E+08	A	G	0.271	-0.705	0.170	3.39E-05	0.184
CD	STMN3	20	62271061	rs909333	62270637	G	T	0.207	0.547	0.102	8.31E-08	0.200
IBD	STMN3	20	62271061	rs909333	62270637	G	T	0.207	0.555	0.087	2.01E-10	0.055
UC	STMN3	20	62271061	rs909333	62270637	G	T	0.207	0.498	0.098	4.04E-07	0.062
GORD	PBX2	6	32154964	rs693906	31835164	C	G	0.113	0.154	0.036	1.65E-05	0.058
VHD	AGER	6	32150423	rs693906	31835164	C	G	0.113	0.212	0.052	4.82E-05	0.092

B) Nominally significant effect on VHD of Bonferroni-significant gastrointestinal loci.

Disease	Gene	chr	Probe_bp	topSNP	topSNP_bp	A1	A2	Freq	b_SMR	se_SMR	p_SMR	p_HEIDI
VHD	CFDP1	16	75397489	rs7201989	75471499	G	A	0.246	-0.056	0.018	0.001	0.119
VHD	STAT3	17	40502964	rs1053004	40466092	G	A	0.380	0.093	0.047	0.048	0.948
VHD	IMPDH2	3	49064299	rs34037363	48821036	T	C	0.114	-0.209	0.105	0.047	0.089
VHD	UBE2L3	22	21941029	rs2070512	21949411	C	A	0.194	-0.029	0.009	0.002	0.623
VHD	STMN3	20	62271061	rs909333	62270637	G	T	0.207	-0.076	0.036	0.036	0.093

C) Effect of AGER gene on VHD and gastrointestinal disorders.

Disease	Gene	chr	Probe_bp	topSNP	topSNP_bp	A1	A2	Freq	b_SMR	se_SMR	p_SMR	p_HEIDI
CD	AGER	6	32150423	rs693906	31835164	C	G	0.113	-0.312	0.120	0.009	6.16E-08
GORD	AGER	6	32150423	rs693906	31835164	C	G	0.113	-0.209	0.049	2.02E-05	0.022
IBD	AGER	6	32148745	rs204995	32154285	G	A	0.217	-0.252	0.108	0.020	1.60E-04
IBS	AGER	6	32150423	rs693906	31835164	C	G	0.113	-0.168	0.052	0.001	0.045
UC	AGER	6	32148745	rs204995	32154285	G	A	0.217	-0.870	0.185	2.59E-06	0.002
VHD	AGER	6	32150423	rs693906	31835164	C	G	0.113	0.212	0.052	4.82E-05	0.092

Supplementary table 6. Results of the summary-based Mendelian randomization integrating whole-blood expression quantitative trait loci. For each disease-gene association, we report the strongest effect observed between GTEX and eQTLGen datasets.

A) Bonferroni-significant disease-gene association with HEIDI P > 0.05.

Disease	Gene	chr	gene_bp	topSNP	topSNP_bp	A1	A2	Freq	b_SMR	se_SMR	p_SMR	p_HEIDI
CD	DNMT3A	2	25510652	rs7578575	25488819	A	T	0.329	-0.834	0.164	3.51E-07	0.383
IBD	DNMT3A	2	25510652	rs7578575	25488819	A	T	0.329	-0.745	0.130	9.22E-09	0.515
UC	DNMT3A	2	25510652	rs7578575	25488819	A	T	0.329	-0.627	0.156	5.73E-05	0.541
UC	CFDP1	16	75327596	rs9934007	75390532	C	T	0.754	0.273	0.070	9.61E-05	0.973
CD	STAT3	17	40502964	rs1053004	40466092	G	A	0.380	0.702	0.128	4.40E-08	0.056
CD	IMPDH2	3	49064299	rs9813813	49207093	A	T	0.110	-1.233	0.294	2.74E-05	0.213
IBD	IMPDH2	3	49064299	rs9813813	49207093	A	T	0.110	-1.328	0.258	2.57E-07	0.071
UC	IMPDH2	3	49064299	rs9813813	49207093	A	T	0.110	-1.445	0.307	2.53E-06	0.050
IBD	UBE2L3	22	21941029	rs2070512	21949411	C	A	0.194	0.163	0.021	1.06E-14	0.169
UC	UBE2L3	22	21941029	rs2070512	21949411	C	A	0.194	0.116	0.027	1.76E-05	0.698
CD	BCL2L15	1	1.14E+08	rs1217393	1.14E+08	A	G	0.271	-0.705	0.170	3.39E-05	0.184
CD	STMN3	20	62271061	rs909333	62270637	G	T	0.207	0.547	0.102	8.31E-08	0.200
IBD	STMN3	20	62271061	rs909333	62270637	G	T	0.207	0.555	0.087	2.01E-10	0.055
UC	STMN3	20	62271061	rs909333	62270637	G	T	0.207	0.498	0.098	4.04E-07	0.062
GORD	PBX2	6	32154964	rs693906	31835164	C	G	0.113	0.154	0.036	1.65E-05	0.058
VHD	AGER	6	32150423	rs693906	31835164	C	G	0.113	0.212	0.052	4.82E-05	0.092

B) Nominally significant effect on VHD of Bonferroni-significant gastrointestinal loci.

Disease	Gene	chr	Probe_bp	topSNP	topSNP_bp	A1	A2	Freq	b_SMR	se_SMR	p_SMR	p_HEIDI
VHD	CFDP1	16	75397489	rs7201989	75471499	G	A	0.246	-0.056	0.018	0.001	0.119
VHD	STAT3	17	40502964	rs1053004	40466092	G	A	0.380	0.093	0.047	0.048	0.948
VHD	IMPDH2	3	49064299	rs34037363	48821036	T	C	0.114	-0.209	0.105	0.047	0.089
VHD	UBE2L3	22	21941029	rs2070512	21949411	C	A	0.194	-0.029	0.009	0.002	0.623
VHD	STMN3	20	62271061	rs909333	62270637	G	T	0.207	-0.076	0.036	0.036	0.093

C) Effect of AGER gene on VHD and gastrointestinal disorders.

Disease	Gene	chr	Probe_bp	topSNP	topSNP_bp	A1	A2	Freq	b_SMR	se_SMR	p_SMR	p_HEIDI
CD	AGER	6	32150423	rs693906	31835164	C	G	0.113	-0.312	0.120	0.009	6.16E-08
GORD	AGER	6	32150423	rs693906	31835164	C	G	0.113	-0.209	0.049	2.02E-05	0.022
IBD	AGER	6	32148745	rs204995	32154285	G	A	0.217	-0.252	0.108	0.020	1.60E-04
IBS	AGER	6	32150423	rs693906	31835164	C	G	0.113	-0.168	0.052	0.001	0.045
UC	AGER	6	32148745	rs204995	32154285	G	A	0.217	-0.870	0.185	2.59E-06	0.002
VHD	AGER	6	32150423	rs693906	31835164	C	G	0.113	0.212	0.052	4.82E-05	0.092

Supplementary table 6. Results of the summary-based Mendelian randomization integrating whole-blood expression quantitative trait loci. For each disease-gene association, we report the strongest effect observed between GTEX and eQTLGen datasets.

A) Bonferroni-significant disease-gene association with HEIDI P > 0.05.

Disease	Gene	chr	gene_bp	topSNP	topSNP_bp	A1	A2	Freq	b_SMR	se_SMR	p_SMR	p_HEIDI
CD	DNMT3A	2	25510652	rs7578575	25488819	A	T	0.329	-0.834	0.164	3.51E-07	0.383
IBD	DNMT3A	2	25510652	rs7578575	25488819	A	T	0.329	-0.745	0.130	9.22E-09	0.515
UC	DNMT3A	2	25510652	rs7578575	25488819	A	T	0.329	-0.627	0.156	5.73E-05	0.541
UC	CFDP1	16	75327596	rs9934007	75390532	C	T	0.754	0.273	0.070	9.61E-05	0.973
CD	STAT3	17	40502964	rs1053004	40466092	G	A	0.380	0.702	0.128	4.40E-08	0.056
CD	IMPDH2	3	49064299	rs9813813	49207093	A	T	0.110	-1.233	0.294	2.74E-05	0.213
IBD	IMPDH2	3	49064299	rs9813813	49207093	A	T	0.110	-1.328	0.258	2.57E-07	0.071
UC	IMPDH2	3	49064299	rs9813813	49207093	A	T	0.110	-1.445	0.307	2.53E-06	0.050
IBD	UBE2L3	22	21941029	rs2070512	21949411	C	A	0.194	0.163	0.021	1.06E-14	0.169
UC	UBE2L3	22	21941029	rs2070512	21949411	C	A	0.194	0.116	0.027	1.76E-05	0.698
CD	BCL2L15	1	1.14E+08	rs1217393	1.14E+08	A	G	0.271	-0.705	0.170	3.39E-05	0.184
CD	STMN3	20	62271061	rs909333	62270637	G	T	0.207	0.547	0.102	8.31E-08	0.200
IBD	STMN3	20	62271061	rs909333	62270637	G	T	0.207	0.555	0.087	2.01E-10	0.055
UC	STMN3	20	62271061	rs909333	62270637	G	T	0.207	0.498	0.098	4.04E-07	0.062
GORD	PBX2	6	32154964	rs693906	31835164	C	G	0.113	0.154	0.036	1.65E-05	0.058
VHD	AGER	6	32150423	rs693906	31835164	C	G	0.113	0.212	0.052	4.82E-05	0.092

B) Nominally significant effect on VHD of Bonferroni-significant gastrointestinal loci.

Disease	Gene	chr	Probe_bp	topSNP	topSNP_bp	A1	A2	Freq	b_SMR	se_SMR	p_SMR	p_HEIDI
VHD	CFDP1	16	75397489	rs7201989	75471499	G	A	0.246	-0.056	0.018	0.001	0.119
VHD	STAT3	17	40502964	rs1053004	40466092	G	A	0.380	0.093	0.047	0.048	0.948
VHD	IMPDH2	3	49064299	rs34037363	48821036	T	C	0.114	-0.209	0.105	0.047	0.089
VHD	UBE2L3	22	21941029	rs2070512	21949411	C	A	0.194	-0.029	0.009	0.002	0.623
VHD	STMN3	20	62271061	rs909333	62270637	G	T	0.207	-0.076	0.036	0.036	0.093

C) Effect of AGER gene on VHD and gastrointestinal disorders.

Disease	Gene	chr	Probe_bp	topSNP	topSNP_bp	A1	A2	Freq	b_SMR	se_SMR	p_SMR	p_HEIDI
CD	AGER	6	32150423	rs693906	31835164	C	G	0.113	-0.312	0.120	0.009	6.16E-08
GORD	AGER	6	32150423	rs693906	31835164	C	G	0.113	-0.209	0.049	2.02E-05	0.022
IBD	AGER	6	32148745	rs204995	32154285	G	A	0.217	-0.252	0.108	0.020	1.60E-04
IBS	AGER	6	32150423	rs693906	31835164	C	G	0.113	-0.168	0.052	0.001	0.045
UC	AGER	6	32148745	rs204995	32154285	G	A	0.217	-0.870	0.185	2.59E-06	0.002
VHD	AGER	6	32150423	rs693906	31835164	C	G	0.113	0.212	0.052	4.82E-05	0.092

Supplementary table 6. Results of the summary-based Mendelian randomization integrating whole-blood expression quantitative trait loci. For each disease-gene association, we report the strongest effect observed between GTEX and eQTLGen datasets.

A) Bonferroni-significant disease-gene association with HEIDI P > 0.05.

Disease	Gene	chr	gene_bp	topSNP	topSNP_bp	A1	A2	Freq	b_SMR	se_SMR	p_SMR	p_HEIDI
CD	DNMT3A	2	25510652	rs7578575	25488819	A	T	0.329	-0.834	0.164	3.51E-07	0.383
IBD	DNMT3A	2	25510652	rs7578575	25488819	A	T	0.329	-0.745	0.130	9.22E-09	0.515
UC	DNMT3A	2	25510652	rs7578575	25488819	A	T	0.329	-0.627	0.156	5.73E-05	0.541
UC	CFDP1	16	75327596	rs9934007	75390532	C	T	0.754	0.273	0.070	9.61E-05	0.973
CD	STAT3	17	40502964	rs1053004	40466092	G	A	0.380	0.702	0.128	4.40E-08	0.056
CD	IMPDH2	3	49064299	rs9813813	49207093	A	T	0.110	-1.233	0.294	2.74E-05	0.213
IBD	IMPDH2	3	49064299	rs9813813	49207093	A	T	0.110	-1.328	0.258	2.57E-07	0.071
UC	IMPDH2	3	49064299	rs9813813	49207093	A	T	0.110	-1.445	0.307	2.53E-06	0.050
IBD	UBE2L3	22	21941029	rs2070512	21949411	C	A	0.194	0.163	0.021	1.06E-14	0.169
UC	UBE2L3	22	21941029	rs2070512	21949411	C	A	0.194	0.116	0.027	1.76E-05	0.698
CD	BCL2L15	1	1.14E+08	rs1217393	1.14E+08	A	G	0.271	-0.705	0.170	3.39E-05	0.184
CD	STMN3	20	62271061	rs909333	62270637	G	T	0.207	0.547	0.102	8.31E-08	0.200
IBD	STMN3	20	62271061	rs909333	62270637	G	T	0.207	0.555	0.087	2.01E-10	0.055
UC	STMN3	20	62271061	rs909333	62270637	G	T	0.207	0.498	0.098	4.04E-07	0.062
GORD	PBX2	6	32154964	rs693906	31835164	C	G	0.113	0.154	0.036	1.65E-05	0.058
VHD	AGER	6	32150423	rs693906	31835164	C	G	0.113	0.212	0.052	4.82E-05	0.092

B) Nominally significant effect on VHD of Bonferroni-significant gastrointestinal loci.

Disease	Gene	chr	Probe_bp	topSNP	topSNP_bp	A1	A2	Freq	b_SMR	se_SMR	p_SMR	p_HEIDI
VHD	CFDP1	16	75397489	rs7201989	75471499	G	A	0.246	-0.056	0.018	0.001	0.119
VHD	STAT3	17	40502964	rs1053004	40466092	G	A	0.380	0.093	0.047	0.048	0.948
VHD	IMPDH2	3	49064299	rs34037363	48821036	T	C	0.114	-0.209	0.105	0.047	0.089
VHD	UBE2L3	22	21941029	rs2070512	21949411	C	A	0.194	-0.029	0.009	0.002	0.623
VHD	STMN3	20	62271061	rs909333	62270637	G	T	0.207	-0.076	0.036	0.036	0.093

C) Effect of AGER gene on VHD and gastrointestinal disorders.

Disease	Gene	chr	Probe_bp	topSNP	topSNP_bp	A1	A2	Freq	b_SMR	se_SMR	p_SMR	p_HEIDI
CD	AGER	6	32150423	rs693906	31835164	C	G	0.113	-0.312	0.120	0.009	6.16E-08
GORD	AGER	6	32150423	rs693906	31835164	C	G	0.113	-0.209	0.049	2.02E-05	0.022
IBD	AGER	6	32148745	rs204995	32154285	G	A	0.217	-0.252	0.108	0.020	1.60E-04
IBS	AGER	6	32150423	rs693906	31835164	C	G	0.113	-0.168	0.052	0.001	0.045
UC	AGER	6	32148745	rs204995	32154285	G	A	0.217	-0.870	0.185	2.59E-06	0.002
VHD	AGER	6	32150423	rs693906	31835164	C	G	0.113	0.212	0.052	4.82E-05	0.092

Supplementary table 6. Results of the summary-based Mendelian randomization integrating whole-blood expression quantitative trait loci. For each disease-gene association, we report the strongest effect observed between GTEX and eQTLGen datasets.

A) Bonferroni-significant disease-gene association with HEIDI P > 0.05.

Disease	Gene	chr	gene_bp	topSNP	topSNP_bp	A1	A2	Freq	b_SMR	se_SMR	p_SMR	p_HEIDI
CD	DNMT3A	2	25510652	rs7578575	25488819	A	T	0.329	-0.834	0.164	3.51E-07	0.383
IBD	DNMT3A	2	25510652	rs7578575	25488819	A	T	0.329	-0.745	0.130	9.22E-09	0.515
UC	DNMT3A	2	25510652	rs7578575	25488819	A	T	0.329	-0.627	0.156	5.73E-05	0.541
UC	CFDP1	16	75327596	rs9934007	75390532	C	T	0.754	0.273	0.070	9.61E-05	0.973
CD	STAT3	17	40502964	rs1053004	40466092	G	A	0.380	0.702	0.128	4.40E-08	0.056
CD	IMPDH2	3	49064299	rs9813813	49207093	A	T	0.110	-1.233	0.294	2.74E-05	0.213
IBD	IMPDH2	3	49064299	rs9813813	49207093	A	T	0.110	-1.328	0.258	2.57E-07	0.071
UC	IMPDH2	3	49064299	rs9813813	49207093	A	T	0.110	-1.445	0.307	2.53E-06	0.050
IBD	UBE2L3	22	21941029	rs2070512	21949411	C	A	0.194	0.163	0.021	1.06E-14	0.169
UC	UBE2L3	22	21941029	rs2070512	21949411	C	A	0.194	0.116	0.027	1.76E-05	0.698
CD	BCL2L15	1	1.14E+08	rs1217393	1.14E+08	A	G	0.271	-0.705	0.170	3.39E-05	0.184
CD	STMN3	20	62271061	rs909333	62270637	G	T	0.207	0.547	0.102	8.31E-08	0.200
IBD	STMN3	20	62271061	rs909333	62270637	G	T	0.207	0.555	0.087	2.01E-10	0.055
UC	STMN3	20	62271061	rs909333	62270637	G	T	0.207	0.498	0.098	4.04E-07	0.062
GORD	PBX2	6	32154964	rs693906	31835164	C	G	0.113	0.154	0.036	1.65E-05	0.058
VHD	AGER	6	32150423	rs693906	31835164	C	G	0.113	0.212	0.052	4.82E-05	0.092

B) Nominally significant effect on VHD of Bonferroni-significant gastrointestinal loci.

Disease	Gene	chr	Probe_bp	topSNP	topSNP_bp	A1	A2	Freq	b_SMR	se_SMR	p_SMR	p_HEIDI
VHD	CFDP1	16	75397489	rs7201989	75471499	G	A	0.246	-0.056	0.018	0.001	0.119
VHD	STAT3	17	40502964	rs1053004	40466092	G	A	0.380	0.093	0.047	0.048	0.948
VHD	IMPDH2	3	49064299	rs34037363	48821036	T	C	0.114	-0.209	0.105	0.047	0.089
VHD	UBE2L3	22	21941029	rs2070512	21949411	C	A	0.194	-0.029	0.009	0.002	0.623
VHD	STMN3	20	62271061	rs909333	62270637	G	T	0.207	-0.076	0.036	0.036	0.093

C) Effect of AGER gene on VHD and gastrointestinal disorders.

Disease	Gene	chr	Probe_bp	topSNP	topSNP_bp	A1	A2	Freq	b_SMR	se_SMR	p_SMR	p_HEIDI
CD	AGER	6	32150423	rs693906	31835164	C	G	0.113	-0.312	0.120	0.009	6.16E-08
GORD	AGER	6	32150423	rs693906	31835164	C	G	0.113	-0.209	0.049	2.02E-05	0.022
IBD	AGER	6	32148745	rs204995	32154285	G	A	0.217	-0.252	0.108	0.020	1.60E-04
IBS	AGER	6	32150423	rs693906	31835164	C	G	0.113	-0.168	0.052	0.001	0.045
UC	AGER	6	32148745	rs204995	32154285	G	A	0.217	-0.870	0.185	2.59E-06	0.002
VHD	AGER	6	32150423	rs693906	31835164	C	G	0.113	0.212	0.052	4.82E-05	0.092

Supplementary table 6. Results of the summary-based Mendelian randomization integrating whole-blood expression quantitative trait loci. For each disease-gene association, we report the strongest effect observed between GTEX and eQTLGen datasets.

A) Bonferroni-significant disease-gene association with HEIDI P > 0.05.

Disease	Gene	chr	gene_bp	topSNP	topSNP_bp	A1	A2	Freq	b_SMR	se_SMR	p_SMR	p_HEIDI
CD	DNMT3A	2	25510652	rs7578575	25488819	A	T	0.329	-0.834	0.164	3.51E-07	0.383
IBD	DNMT3A	2	25510652	rs7578575	25488819	A	T	0.329	-0.745	0.130	9.22E-09	0.515
UC	DNMT3A	2	25510652	rs7578575	25488819	A	T	0.329	-0.627	0.156	5.73E-05	0.541
UC	CFDP1	16	75327596	rs9934007	75390532	C	T	0.754	0.273	0.070	9.61E-05	0.973
CD	STAT3	17	40502964	rs1053004	40466092	G	A	0.380	0.702	0.128	4.40E-08	0.056
CD	IMPDH2	3	49064299	rs9813813	49207093	A	T	0.110	-1.233	0.294	2.74E-05	0.213
IBD	IMPDH2	3	49064299	rs9813813	49207093	A	T	0.110	-1.328	0.258	2.57E-07	0.071
UC	IMPDH2	3	49064299	rs9813813	49207093	A	T	0.110	-1.445	0.307	2.53E-06	0.050
IBD	UBE2L3	22	21941029	rs2070512	21949411	C	A	0.194	0.163	0.021	1.06E-14	0.169
UC	UBE2L3	22	21941029	rs2070512	21949411	C	A	0.194	0.116	0.027	1.76E-05	0.698
CD	BCL2L15	1	1.14E+08	rs1217393	1.14E+08	A	G	0.271	-0.705	0.170	3.39E-05	0.184
CD	STMN3	20	62271061	rs909333	62270637	G	T	0.207	0.547	0.102	8.31E-08	0.200
IBD	STMN3	20	62271061	rs909333	62270637	G	T	0.207	0.555	0.087	2.01E-10	0.055
UC	STMN3	20	62271061	rs909333	62270637	G	T	0.207	0.498	0.098	4.04E-07	0.062
GORD	PBX2	6	32154964	rs693906	31835164	C	G	0.113	0.154	0.036	1.65E-05	0.058
VHD	AGER	6	32150423	rs693906	31835164	C	G	0.113	0.212	0.052	4.82E-05	0.092

B) Nominally significant effect on VHD of Bonferroni-significant gastrointestinal loci.

Disease	Gene	chr	Probe_bp	topSNP	topSNP_bp	A1	A2	Freq	b_SMR	se_SMR	p_SMR	p_HEIDI
VHD	CFDP1	16	75397489	rs7201989	75471499	G	A	0.246	-0.056	0.018	0.001	0.119
VHD	STAT3	17	40502964	rs1053004	40466092	G	A	0.380	0.093	0.047	0.048	0.948
VHD	IMPDH2	3	49064299	rs34037363	48821036	T	C	0.114	-0.209	0.105	0.047	0.089
VHD	UBE2L3	22	21941029	rs2070512	21949411	C	A	0.194	-0.029	0.009	0.002	0.623
VHD	STMN3	20	62271061	rs909333	62270637	G	T	0.207	-0.076	0.036	0.036	0.093

C) Effect of AGER gene on VHD and gastrointestinal disorders.

Disease	Gene	chr	Probe_bp	topSNP	topSNP_bp	A1	A2	Freq	b_SMR	se_SMR	p_SMR	p_HEIDI
CD	AGER	6	32150423	rs693906	31835164	C	G	0.113	-0.312	0.120	0.009	6.16E-08
GORD	AGER	6	32150423	rs693906	31835164	C	G	0.113	-0.209	0.049	2.02E-05	0.022
IBD	AGER	6	32148745	rs204995	32154285	G	A	0.217	-0.252	0.108	0.020	1.60E-04
IBS	AGER	6	32150423	rs693906	31835164	C	G	0.113	-0.168	0.052	0.001	0.045
UC	AGER	6	32148745	rs204995	32154285	G	A	0.217	-0.870	0.185	2.59E-06	0.002
VHD	AGER	6	32150423	rs693906	31835164	C	G	0.113	0.212	0.052	4.82E-05	0.092

Supplementary table 6. Results of the summary-based Mendelian randomization integrating whole-blood expression quantitative trait loci. For each disease-gene association, we report the strongest effect observed between GTEX and eQTLGen datasets.

A) Bonferroni-significant disease-gene association with HEIDI P > 0.05.

Disease	Gene	chr	gene_bp	topSNP	topSNP_bp	A1	A2	Freq	b_SMR	se_SMR	p_SMR	p_HEIDI
CD	DNMT3A	2	25510652	rs7578575	25488819	A	T	0.329	-0.834	0.164	3.51E-07	0.383
IBD	DNMT3A	2	25510652	rs7578575	25488819	A	T	0.329	-0.745	0.130	9.22E-09	0.515
UC	DNMT3A	2	25510652	rs7578575	25488819	A	T	0.329	-0.627	0.156	5.73E-05	0.541
UC	CFDP1	16	75327596	rs9934007	75390532	C	T	0.754	0.273	0.070	9.61E-05	0.973
CD	STAT3	17	40502964	rs1053004	40466092	G	A	0.380	0.702	0.128	4.40E-08	0.056
CD	IMPDH2	3	49064299	rs9813813	49207093	A	T	0.110	-1.233	0.294	2.74E-05	0.213
IBD	IMPDH2	3	49064299	rs9813813	49207093	A	T	0.110	-1.328	0.258	2.57E-07	0.071
UC	IMPDH2	3	49064299	rs9813813	49207093	A	T	0.110	-1.445	0.307	2.53E-06	0.050
IBD	UBE2L3	22	21941029	rs2070512	21949411	C	A	0.194	0.163	0.021	1.06E-14	0.169
UC	UBE2L3	22	21941029	rs2070512	21949411	C	A	0.194	0.116	0.027	1.76E-05	0.698
CD	BCL2L15	1	1.14E+08	rs1217393	1.14E+08	A	G	0.271	-0.705	0.170	3.39E-05	0.184
CD	STMN3	20	62271061	rs909333	62270637	G	T	0.207	0.547	0.102	8.31E-08	0.200
IBD	STMN3	20	62271061	rs909333	62270637	G	T	0.207	0.555	0.087	2.01E-10	0.055
UC	STMN3	20	62271061	rs909333	62270637	G	T	0.207	0.498	0.098	4.04E-07	0.062
GORD	PBX2	6	32154964	rs693906	31835164	C	G	0.113	0.154	0.036	1.65E-05	0.058
VHD	AGER	6	32150423	rs693906	31835164	C	G	0.113	0.212	0.052	4.82E-05	0.092

B) Nominally significant effect on VHD of Bonferroni-significant gastrointestinal loci.

Disease	Gene	chr	Probe_bp	topSNP	topSNP_bp	A1	A2	Freq	b_SMR	se_SMR	p_SMR	p_HEIDI
VHD	CFDP1	16	75397489	rs7201989	75471499	G	A	0.246	-0.056	0.018	0.001	0.119
VHD	STAT3	17	40502964	rs1053004	40466092	G	A	0.380	0.093	0.047	0.048	0.948
VHD	IMPDH2	3	49064299	rs34037363	48821036	T	C	0.114	-0.209	0.105	0.047	0.089
VHD	UBE2L3	22	21941029	rs2070512	21949411	C	A	0.194	-0.029	0.009	0.002	0.623
VHD	STMN3	20	62271061	rs909333	62270637	G	T	0.207	-0.076	0.036	0.036	0.093

C) Effect of AGER gene on VHD and gastrointestinal disorders.

Disease	Gene	chr	Probe_bp	topSNP	topSNP_bp	A1	A2	Freq	b_SMR	se_SMR	p_SMR	p_HEIDI
CD	AGER	6	32150423	rs693906	31835164	C	G	0.113	-0.312	0.120	0.009	6.16E-08
GORD	AGER	6	32150423	rs693906	31835164	C	G	0.113	-0.209	0.049	2.02E-05	0.022
IBD	AGER	6	32148745	rs204995	32154285	G	A	0.217	-0.252	0.108	0.020	1.60E-04
IBS	AGER	6	32150423	rs693906	31835164	C	G	0.113	-0.168	0.052	0.001	0.045
UC	AGER	6	32148745	rs204995	32154285	G	A	0.217	-0.870	0.185	2.59E-06	0.002
VHD	AGER	6	32150423	rs693906	31835164	C	G	0.113	0.212	0.052	4.82E-05	0.092

Supplementary table 6. Results of the summary-based Mendelian randomization integrating whole-blood expression quantitative trait loci. For each disease-gene association, we report the strongest effect observed between GTEX and eQTLGen datasets.

A) Bonferroni-significant disease-gene association with HEIDI P > 0.05.

Disease	Gene	chr	gene_bp	topSNP	topSNP_bp	A1	A2	Freq	b_SMR	se_SMR	p_SMR	p_HEIDI
CD	DNMT3A	2	25510652	rs7578575	25488819	A	T	0.329	-0.834	0.164	3.51E-07	0.383
IBD	DNMT3A	2	25510652	rs7578575	25488819	A	T	0.329	-0.745	0.130	9.22E-09	0.515
UC	DNMT3A	2	25510652	rs7578575	25488819	A	T	0.329	-0.627	0.156	5.73E-05	0.541
UC	CFDP1	16	75327596	rs9934007	75390532	C	T	0.754	0.273	0.070	9.61E-05	0.973
CD	STAT3	17	40502964	rs1053004	40466092	G	A	0.380	0.702	0.128	4.40E-08	0.056
CD	IMPDH2	3	49064299	rs9813813	49207093	A	T	0.110	-1.233	0.294	2.74E-05	0.213
IBD	IMPDH2	3	49064299	rs9813813	49207093	A	T	0.110	-1.328	0.258	2.57E-07	0.071
UC	IMPDH2	3	49064299	rs9813813	49207093	A	T	0.110	-1.445	0.307	2.53E-06	0.050
IBD	UBE2L3	22	21941029	rs2070512	21949411	C	A	0.194	0.163	0.021	1.06E-14	0.169
UC	UBE2L3	22	21941029	rs2070512	21949411	C	A	0.194	0.116	0.027	1.76E-05	0.698
CD	BCL2L15	1	1.14E+08	rs1217393	1.14E+08	A	G	0.271	-0.705	0.170	3.39E-05	0.184
CD	STMN3	20	62271061	rs909333	62270637	G	T	0.207	0.547	0.102	8.31E-08	0.200
IBD	STMN3	20	62271061	rs909333	62270637	G	T	0.207	0.555	0.087	2.01E-10	0.055
UC	STMN3	20	62271061	rs909333	62270637	G	T	0.207	0.498	0.098	4.04E-07	0.062
GORD	PBX2	6	32154964	rs693906	31835164	C	G	0.113	0.154	0.036	1.65E-05	0.058
VHD	AGER	6	32150423	rs693906	31835164	C	G	0.113	0.212	0.052	4.82E-05	0.092

B) Nominally significant effect on VHD of Bonferroni-significant gastrointestinal loci.

Disease	Gene	chr	Probe_bp	topSNP	topSNP_bp	A1	A2	Freq	b_SMR	se_SMR	p_SMR	p_HEIDI
VHD	CFDP1	16	75397489	rs7201989	75471499	G	A	0.246	-0.056	0.018	0.001	0.119
VHD	STAT3	17	40502964	rs1053004	40466092	G	A	0.380	0.093	0.047	0.048	0.948
VHD	IMPDH2	3	49064299	rs34037363	48821036	T	C	0.114	-0.209	0.105	0.047	0.089
VHD	UBE2L3	22	21941029	rs2070512	21949411	C	A	0.194	-0.029	0.009	0.002	0.623
VHD	STMN3	20	62271061	rs909333	62270637	G	T	0.207	-0.076	0.036	0.036	0.093

C) Effect of AGER gene on VHD and gastrointestinal disorders.

Disease	Gene	chr	Probe_bp	topSNP	topSNP_bp	A1	A2	Freq	b_SMR	se_SMR	p_SMR	p_HEIDI
CD	AGER	6	32150423	rs693906	31835164	C	G	0.113	-0.312	0.120	0.009	6.16E-08
GORD	AGER	6	32150423	rs693906	31835164	C	G	0.113	-0.209	0.049	2.02E-05	0.022
IBD	AGER	6	32148745	rs204995	32154285	G	A	0.217	-0.252	0.108	0.020	1.60E-04
IBS	AGER	6	32150423	rs693906	31835164	C	G	0.113	-0.168	0.052	0.001	0.045
UC	AGER	6	32148745	rs204995	32154285	G	A	0.217	-0.870	0.185	2.59E-06	0.002
VHD	AGER	6	32150423	rs693906	31835164	C	G	0.113	0.212	0.052	4.82E-05	0.092

Supplementary table 6. Results of the summary-based Mendelian randomization integrating whole-blood expression quantitative trait loci. For each disease-gene association, we report the strongest effect observed between GTEX and eQTLGen datasets.

A) Bonferroni-significant disease-gene association with HEIDI P > 0.05.

Disease	Gene	chr	gene_bp	topSNP	topSNP_bp	A1	A2	Freq	b_SMR	se_SMR	p_SMR	p_HEIDI
CD	DNMT3A	2	25510652	rs7578575	25488819	A	T	0.329	-0.834	0.164	3.51E-07	0.383
IBD	DNMT3A	2	25510652	rs7578575	25488819	A	T	0.329	-0.745	0.130	9.22E-09	0.515
UC	DNMT3A	2	25510652	rs7578575	25488819	A	T	0.329	-0.627	0.156	5.73E-05	0.541
UC	CFDP1	16	75327596	rs9934007	75390532	C	T	0.754	0.273	0.070	9.61E-05	0.973
CD	STAT3	17	40502964	rs1053004	40466092	G	A	0.380	0.702	0.128	4.40E-08	0.056
CD	IMPDH2	3	49064299	rs9813813	49207093	A	T	0.110	-1.233	0.294	2.74E-05	0.213
IBD	IMPDH2	3	49064299	rs9813813	49207093	A	T	0.110	-1.328	0.258	2.57E-07	0.071
UC	IMPDH2	3	49064299	rs9813813	49207093	A	T	0.110	-1.445	0.307	2.53E-06	0.050
IBD	UBE2L3	22	21941029	rs2070512	21949411	C	A	0.194	0.163	0.021	1.06E-14	0.169
UC	UBE2L3	22	21941029	rs2070512	21949411	C	A	0.194	0.116	0.027	1.76E-05	0.698
CD	BCL2L15	1	1.14E+08	rs1217393	1.14E+08	A	G	0.271	-0.705	0.170	3.39E-05	0.184
CD	STMN3	20	62271061	rs909333	62270637	G	T	0.207	0.547	0.102	8.31E-08	0.200
IBD	STMN3	20	62271061	rs909333	62270637	G	T	0.207	0.555	0.087	2.01E-10	0.055
UC	STMN3	20	62271061	rs909333	62270637	G	T	0.207	0.498	0.098	4.04E-07	0.062
GORD	PBX2	6	32154964	rs693906	31835164	C	G	0.113	0.154	0.036	1.65E-05	0.058
VHD	AGER	6	32150423	rs693906	31835164	C	G	0.113	0.212	0.052	4.82E-05	0.092

B) Nominally significant effect on VHD of Bonferroni-significant gastrointestinal loci.

Disease	Gene	chr	Probe_bp	topSNP	topSNP_bp	A1	A2	Freq	b_SMR	se_SMR	p_SMR	p_HEIDI
VHD	CFDP1	16	75397489	rs7201989	75471499	G	A	0.246	-0.056	0.018	0.001	0.119
VHD	STAT3	17	40502964	rs1053004	40466092	G	A	0.380	0.093	0.047	0.048	0.948
VHD	IMPDH2	3	49064299	rs34037363	48821036	T	C	0.114	-0.209	0.105	0.047	0.089
VHD	UBE2L3	22	21941029	rs2070512	21949411	C	A	0.194	-0.029	0.009	0.002	0.623
VHD	STMN3	20	62271061	rs909333	62270637	G	T	0.207	-0.076	0.036	0.036	0.093

C) Effect of AGER gene on VHD and gastrointestinal disorders.

Disease	Gene	chr	Probe_bp	topSNP	topSNP_bp	A1	A2	Freq	b_SMR	se_SMR	p_SMR	p_HEIDI
CD	AGER	6	32150423	rs693906	31835164	C	G	0.113	-0.312	0.120	0.009	6.16E-08
GORD	AGER	6	32150423	rs693906	31835164	C	G	0.113	-0.209	0.049	2.02E-05	0.022
IBD	AGER	6	32148745	rs204995	32154285	G	A	0.217	-0.252	0.108	0.020	1.60E-04
IBS	AGER	6	32150423	rs693906	31835164	C	G	0.113	-0.168	0.052	0.001	0.045
UC	AGER	6	32148745	rs204995	32154285	G	A	0.217	-0.870	0.185	2.59E-06	0.002
VHD	AGER	6	32150423	rs693906	31835164	C	G	0.113	0.212	0.052	4.82E-05	0.092

Supplementary table 6. Results of the summary-based Mendelian randomization integrating whole-blood expression quantitative trait loci. For each disease-gene association, we report the strongest effect observed between GTEX and eQTLGen datasets.

A) Bonferroni-significant disease-gene association with HEIDI P > 0.05.

Disease	Gene	chr	gene_bp	topSNP	topSNP_bp	A1	A2	Freq	b_SMR	se_SMR	p_SMR	p_HEIDI
CD	DNMT3A	2	25510652	rs7578575	25488819	A	T	0.329	-0.834	0.164	3.51E-07	0.383
IBD	DNMT3A	2	25510652	rs7578575	25488819	A	T	0.329	-0.745	0.130	9.22E-09	0.515
UC	DNMT3A	2	25510652	rs7578575	25488819	A	T	0.329	-0.627	0.156	5.73E-05	0.541
UC	CFDP1	16	75327596	rs9934007	75390532	C	T	0.754	0.273	0.070	9.61E-05	0.973
CD	STAT3	17	40502964	rs1053004	40466092	G	A	0.380	0.702	0.128	4.40E-08	0.056
CD	IMPDH2	3	49064299	rs9813813	49207093	A	T	0.110	-1.233	0.294	2.74E-05	0.213
IBD	IMPDH2	3	49064299	rs9813813	49207093	A	T	0.110	-1.328	0.258	2.57E-07	0.071
UC	IMPDH2	3	49064299	rs9813813	49207093	A	T	0.110	-1.445	0.307	2.53E-06	0.050
IBD	UBE2L3	22	21941029	rs2070512	21949411	C	A	0.194	0.163	0.021	1.06E-14	0.169
UC	UBE2L3	22	21941029	rs2070512	21949411	C	A	0.194	0.116	0.027	1.76E-05	0.698
CD	BCL2L15	1	1.14E+08	rs1217393	1.14E+08	A	G	0.271	-0.705	0.170	3.39E-05	0.184
CD	STMN3	20	62271061	rs909333	62270637	G	T	0.207	0.547	0.102	8.31E-08	0.200
IBD	STMN3	20	62271061	rs909333	62270637	G	T	0.207	0.555	0.087	2.01E-10	0.055
UC	STMN3	20	62271061	rs909333	62270637	G	T	0.207	0.498	0.098	4.04E-07	0.062
GORD	PBX2	6	32154964	rs693906	31835164	C	G	0.113	0.154	0.036	1.65E-05	0.058
VHD	AGER	6	32150423	rs693906	31835164	C	G	0.113	0.212	0.052	4.82E-05	0.092

B) Nominally significant effect on VHD of Bonferroni-significant gastrointestinal loci.

Disease	Gene	chr	Probe_bp	topSNP	topSNP_bp	A1	A2	Freq	b_SMR	se_SMR	p_SMR	p_HEIDI
VHD	CFDP1	16	75397489	rs7201989	75471499	G	A	0.246	-0.056	0.018	0.001	0.119
VHD	STAT3	17	40502964	rs1053004	40466092	G	A	0.380	0.093	0.047	0.048	0.948
VHD	IMPDH2	3	49064299	rs34037363	48821036	T	C	0.114	-0.209	0.105	0.047	0.089
VHD	UBE2L3	22	21941029	rs2070512	21949411	C	A	0.194	-0.029	0.009	0.002	0.623
VHD	STMN3	20	62271061	rs909333	62270637	G	T	0.207	-0.076	0.036	0.036	0.093

C) Effect of AGER gene on VHD and gastrointestinal disorders.

Disease	Gene	chr	Probe_bp	topSNP	topSNP_bp	A1	A2	Freq	b_SMR	se_SMR	p_SMR	p_HEIDI
CD	AGER	6	32150423	rs693906	31835164	C	G	0.113	-0.312	0.120	0.009	6.16E-08
GORD	AGER	6	32150423	rs693906	31835164	C	G	0.113	-0.209	0.049	2.02E-05	0.022
IBD	AGER	6	32148745	rs204995	32154285	G	A	0.217	-0.252	0.108	0.020	1.60E-04
IBS	AGER	6	32150423	rs693906	31835164	C	G	0.113	-0.168	0.052	0.001	0.045
UC	AGER	6	32148745	rs204995	32154285	G	A	0.217	-0.870	0.185	2.59E-06	0.002
VHD	AGER	6	32150423	rs693906	31835164	C	G	0.113	0.212	0.052	4.82E-05	0.092

Supplementary table 6. Results of the summary-based Mendelian randomization integrating whole-blood expression quantitative trait loci. For each disease-gene association, we report the strongest effect observed between GTEX and eQTLGen datasets.

A) Bonferroni-significant disease-gene association with HEIDI P > 0.05.

Disease	Gene	chr	gene_bp	topSNP	topSNP_bp	A1	A2	Freq	b_SMR	se_SMR	p_SMR	p_HEIDI
CD	DNMT3A	2	25510652	rs7578575	25488819	A	T	0.329	-0.834	0.164	3.51E-07	0.383
IBD	DNMT3A	2	25510652	rs7578575	25488819	A	T	0.329	-0.745	0.130	9.22E-09	0.515
UC	DNMT3A	2	25510652	rs7578575	25488819	A	T	0.329	-0.627	0.156	5.73E-05	0.541
UC	CFDP1	16	75327596	rs9934007	75390532	C	T	0.754	0.273	0.070	9.61E-05	0.973
CD	STAT3	17	40502964	rs1053004	40466092	G	A	0.380	0.702	0.128	4.40E-08	0.056
CD	IMPDH2	3	49064299	rs9813813	49207093	A	T	0.110	-1.233	0.294	2.74E-05	0.213
IBD	IMPDH2	3	49064299	rs9813813	49207093	A	T	0.110	-1.328	0.258	2.57E-07	0.071
UC	IMPDH2	3	49064299	rs9813813	49207093	A	T	0.110	-1.445	0.307	2.53E-06	0.050
IBD	UBE2L3	22	21941029	rs2070512	21949411	C	A	0.194	0.163	0.021	1.06E-14	0.169
UC	UBE2L3	22	21941029	rs2070512	21949411	C	A	0.194	0.116	0.027	1.76E-05	0.698
CD	BCL2L15	1	1.14E+08	rs1217393	1.14E+08	A	G	0.271	-0.705	0.170	3.39E-05	0.184
CD	STMN3	20	62271061	rs909333	62270637	G	T	0.207	0.547	0.102	8.31E-08	0.200
IBD	STMN3	20	62271061	rs909333	62270637	G	T	0.207	0.555	0.087	2.01E-10	0.055
UC	STMN3	20	62271061	rs909333	62270637	G	T	0.207	0.498	0.098	4.04E-07	0.062
GORD	PBX2	6	32154964	rs693906	31835164	C	G	0.113	0.154	0.036	1.65E-05	0.058
VHD	AGER	6	32150423	rs693906	31835164	C	G	0.113	0.212	0.052	4.82E-05	0.092

B) Nominally significant effect on VHD of Bonferroni-significant gastrointestinal loci.

Disease	Gene	chr	Probe_bp	topSNP	topSNP_bp	A1	A2	Freq	b_SMR	se_SMR	p_SMR	p_HEIDI
VHD	CFDP1	16	75397489	rs7201989	75471499	G	A	0.246	-0.056	0.018	0.001	0.119
VHD	STAT3	17	40502964	rs1053004	40466092	G	A	0.380	0.093	0.047	0.048	0.948
VHD	IMPDH2	3	49064299	rs34037363	48821036	T	C	0.114	-0.209	0.105	0.047	0.089
VHD	UBE2L3	22	21941029	rs2070512	21949411	C	A	0.194	-0.029	0.009	0.002	0.623
VHD	STMN3	20	62271061	rs909333	62270637	G	T	0.207	-0.076	0.036	0.036	0.093

C) Effect of AGER gene on VHD and gastrointestinal disorders.

Disease	Gene	chr	Probe_bp	topSNP	topSNP_bp	A1	A2	Freq	b_SMR	se_SMR	p_SMR	p_HEIDI
CD	AGER	6	32150423	rs693906	31835164	C	G	0.113	-0.312	0.120	0.009	6.16E-08
GORD	AGER	6	32150423	rs693906	31835164	C	G	0.113	-0.209	0.049	2.02E-05	0.022
IBD	AGER	6	32148745	rs204995	32154285	G	A	0.217	-0.252	0.108	0.020	1.60E-04
IBS	AGER	6	32150423	rs693906	31835164	C	G	0.113	-0.168	0.052	0.001	0.045
UC	AGER	6	32148745	rs204995	32154285	G	A	0.217	-0.870	0.185	2.59E-06	0.002
VHD	AGER	6	32150423	rs693906	31835164	C	G	0.113	0.212	0.052	4.82E-05	0.092

Supplementary table 6. Results of the summary-based Mendelian randomization integrating whole-blood expression quantitative trait loci. For each disease-gene association, we report the strongest effect observed between GTEX and eQTLGen datasets.

A) Bonferroni-significant disease-gene association with HEIDI P > 0.05.

Disease	Gene	chr	gene_bp	topSNP	topSNP_bp	A1	A2	Freq	b_SMR	se_SMR	p_SMR	p_HEIDI
CD	DNMT3A	2	25510652	rs7578575	25488819	A	T	0.329	-0.834	0.164	3.51E-07	0.383
IBD	DNMT3A	2	25510652	rs7578575	25488819	A	T	0.329	-0.745	0.130	9.22E-09	0.515
UC	DNMT3A	2	25510652	rs7578575	25488819	A	T	0.329	-0.627	0.156	5.73E-05	0.541
UC	CFDP1	16	75327596	rs9934007	75390532	C	T	0.754	0.273	0.070	9.61E-05	0.973
CD	STAT3	17	40502964	rs1053004	40466092	G	A	0.380	0.702	0.128	4.40E-08	0.056
CD	IMPDH2	3	49064299	rs9813813	49207093	A	T	0.110	-1.233	0.294	2.74E-05	0.213
IBD	IMPDH2	3	49064299	rs9813813	49207093	A	T	0.110	-1.328	0.258	2.57E-07	0.071
UC	IMPDH2	3	49064299	rs9813813	49207093	A	T	0.110	-1.445	0.307	2.53E-06	0.050
IBD	UBE2L3	22	21941029	rs2070512	21949411	C	A	0.194	0.163	0.021	1.06E-14	0.169
UC	UBE2L3	22	21941029	rs2070512	21949411	C	A	0.194	0.116	0.027	1.76E-05	0.698
CD	BCL2L15	1	1.14E+08	rs1217393	1.14E+08	A	G	0.271	-0.705	0.170	3.39E-05	0.184
CD	STMN3	20	62271061	rs909333	62270637	G	T	0.207	0.547	0.102	8.31E-08	0.200
IBD	STMN3	20	62271061	rs909333	62270637	G	T	0.207	0.555	0.087	2.01E-10	0.055
UC	STMN3	20	62271061	rs909333	62270637	G	T	0.207	0.498	0.098	4.04E-07	0.062
GORD	PBX2	6	32154964	rs693906	31835164	C	G	0.113	0.154	0.036	1.65E-05	0.058
VHD	AGER	6	32150423	rs693906	31835164	C	G	0.113	0.212	0.052	4.82E-05	0.092

B) Nominally significant effect on VHD of Bonferroni-significant gastrointestinal loci.

Disease	Gene	chr	Probe_bp	topSNP	topSNP_bp	A1	A2	Freq	b_SMR	se_SMR	p_SMR	p_HEIDI
VHD	CFDP1	16	75397489	rs7201989	75471499	G	A	0.246	-0.056	0.018	0.001	0.119
VHD	STAT3	17	40502964	rs1053004	40466092	G	A	0.380	0.093	0.047	0.048	0.948
VHD	IMPDH2	3	49064299	rs34037363	48821036	T	C	0.114	-0.209	0.105	0.047	0.089
VHD	UBE2L3	22	21941029	rs2070512	21949411	C	A	0.194	-0.029	0.009	0.002	0.623
VHD	STMN3	20	62271061	rs909333	62270637	G	T	0.207	-0.076	0.036	0.036	0.093

C) Effect of AGER gene on VHD and gastrointestinal disorders.

Disease	Gene	chr	Probe_bp	topSNP	topSNP_bp	A1	A2	Freq	b_SMR	se_SMR	p_SMR	p_HEIDI
CD	AGER	6	32150423	rs693906	31835164	C	G	0.113	-0.312	0.120	0.009	6.16E-08
GORD	AGER	6	32150423	rs693906	31835164	C	G	0.113	-0.209	0.049	2.02E-05	0.022
IBD	AGER	6	32148745	rs204995	32154285	G	A	0.217	-0.252	0.108	0.020	1.60E-04
IBS	AGER	6	32150423	rs693906	31835164	C	G	0.113	-0.168	0.052	0.001	0.045
UC	AGER	6	32148745	rs204995	32154285	G	A	0.217	-0.870	0.185	2.59E-06	0.002
VHD	AGER	6	32150423	rs693906	31835164	C	G	0.113	0.212	0.052	4.82E-05	0.092

Supplementary table 6. Results of the summary-based Mendelian randomization integrating whole-blood expression quantitative trait loci. For each disease-gene association, we report the strongest effect observed between GTEX and eQTLGen datasets.

A) Bonferroni-significant disease-gene association with HEIDI P > 0.05.

Disease	Gene	chr	gene_bp	topSNP	topSNP_bp	A1	A2	Freq	b_SMR	se_SMR	p_SMR	p_HEIDI
CD	DNMT3A	2	25510652	rs7578575	25488819	A	T	0.329	-0.834	0.164	3.51E-07	0.383
IBD	DNMT3A	2	25510652	rs7578575	25488819	A	T	0.329	-0.745	0.130	9.22E-09	0.515
UC	DNMT3A	2	25510652	rs7578575	25488819	A	T	0.329	-0.627	0.156	5.73E-05	0.541
UC	CFDP1	16	75327596	rs9934007	75390532	C	T	0.754	0.273	0.070	9.61E-05	0.973
CD	STAT3	17	40502964	rs1053004	40466092	G	A	0.380	0.702	0.128	4.40E-08	0.056
CD	IMPDH2	3	49064299	rs9813813	49207093	A	T	0.110	-1.233	0.294	2.74E-05	0.213
IBD	IMPDH2	3	49064299	rs9813813	49207093	A	T	0.110	-1.328	0.258	2.57E-07	0.071
UC	IMPDH2	3	49064299	rs9813813	49207093	A	T	0.110	-1.445	0.307	2.53E-06	0.050
IBD	UBE2L3	22	21941029	rs2070512	21949411	C	A	0.194	0.163	0.021	1.06E-14	0.169
UC	UBE2L3	22	21941029	rs2070512	21949411	C	A	0.194	0.116	0.027	1.76E-05	0.698
CD	BCL2L15	1	1.14E+08	rs1217393	1.14E+08	A	G	0.271	-0.705	0.170	3.39E-05	0.184
CD	STMN3	20	62271061	rs909333	62270637	G	T	0.207	0.547	0.102	8.31E-08	0.200
IBD	STMN3	20	62271061	rs909333	62270637	G	T	0.207	0.555	0.087	2.01E-10	0.055
UC	STMN3	20	62271061	rs909333	62270637	G	T	0.207	0.498	0.098	4.04E-07	0.062
GORD	PBX2	6	32154964	rs693906	31835164	C	G	0.113	0.154	0.036	1.65E-05	0.058
VHD	AGER	6	32150423	rs693906	31835164	C	G	0.113	0.212	0.052	4.82E-05	0.092

B) Nominally significant effect on VHD of Bonferroni-significant gastrointestinal loci.

Disease	Gene	chr	Probe_bp	topSNP	topSNP_bp	A1	A2	Freq	b_SMR	se_SMR	p_SMR	p_HEIDI
VHD	CFDP1	16	75397489	rs7201989	75471499	G	A	0.246	-0.056	0.018	0.001	0.119
VHD	STAT3	17	40502964	rs1053004	40466092	G	A	0.380	0.093	0.047	0.048	0.948
VHD	IMPDH2	3	49064299	rs34037363	48821036	T	C	0.114	-0.209	0.105	0.047	0.089
VHD	UBE2L3	22	21941029	rs2070512	21949411	C	A	0.194	-0.029	0.009	0.002	0.623
VHD	STMN3	20	62271061	rs909333	62270637	G	T	0.207	-0.076	0.036	0.036	0.093

C) Effect of AGER gene on VHD and gastrointestinal disorders.

Disease	Gene	chr	Probe_bp	topSNP	topSNP_bp	A1	A2	Freq	b_SMR	se_SMR	p_SMR	p_HEIDI
CD	AGER	6	32150423	rs693906	31835164	C	G	0.113	-0.312	0.120	0.009	6.16E-08
GORD	AGER	6	32150423	rs693906	31835164	C	G	0.113	-0.209	0.049	2.02E-05	0.022
IBD	AGER	6	32148745	rs204995	32154285	G	A	0.217	-0.252	0.108	0.020	1.60E-04
IBS	AGER	6	32150423	rs693906	31835164	C	G	0.113	-0.168	0.052	0.001	0.045
UC	AGER	6	32148745	rs204995	32154285	G	A	0.217	-0.870	0.185	2.59E-06	0.002
VHD	AGER	6	32150423	rs693906	31835164	C	G	0.113	0.212	0.052	4.82E-05	0.092

Supplementary table 6. Results of the summary-based Mendelian randomization integrating whole-blood expression quantitative trait loci. For each disease-gene association, we report the strongest effect observed between GTEX and eQTLGen datasets.

A) Bonferroni-significant disease-gene association with HEIDI P > 0.05.

Disease	Gene	chr	gene_bp	topSNP	topSNP_bp	A1	A2	Freq	b_SMR	se_SMR	p_SMR	p_HEIDI
CD	DNMT3A	2	25510652	rs7578575	25488819	A	T	0.329	-0.834	0.164	3.51E-07	0.383
IBD	DNMT3A	2	25510652	rs7578575	25488819	A	T	0.329	-0.745	0.130	9.22E-09	0.515
UC	DNMT3A	2	25510652	rs7578575	25488819	A	T	0.329	-0.627	0.156	5.73E-05	0.541
UC	CFDP1	16	75327596	rs9934007	75390532	C	T	0.754	0.273	0.070	9.61E-05	0.973
CD	STAT3	17	40502964	rs1053004	40466092	G	A	0.380	0.702	0.128	4.40E-08	0.056
CD	IMPDH2	3	49064299	rs9813813	49207093	A	T	0.110	-1.233	0.294	2.74E-05	0.213
IBD	IMPDH2	3	49064299	rs9813813	49207093	A	T	0.110	-1.328	0.258	2.57E-07	0.071
UC	IMPDH2	3	49064299	rs9813813	49207093	A	T	0.110	-1.445	0.307	2.53E-06	0.050
IBD	UBE2L3	22	21941029	rs2070512	21949411	C	A	0.194	0.163	0.021	1.06E-14	0.169
UC	UBE2L3	22	21941029	rs2070512	21949411	C	A	0.194	0.116	0.027	1.76E-05	0.698
CD	BCL2L15	1	1.14E+08	rs1217393	1.14E+08	A	G	0.271	-0.705	0.170	3.39E-05	0.184
CD	STMN3	20	62271061	rs909333	62270637	G	T	0.207	0.547	0.102	8.31E-08	0.200
IBD	STMN3	20	62271061	rs909333	62270637	G	T	0.207	0.555	0.087	2.01E-10	0.055
UC	STMN3	20	62271061	rs909333	62270637	G	T	0.207	0.498	0.098	4.04E-07	0.062
GORD	PBX2	6	32154964	rs693906	31835164	C	G	0.113	0.154	0.036	1.65E-05	0.058
VHD	AGER	6	32150423	rs693906	31835164	C	G	0.113	0.212	0.052	4.82E-05	0.092

B) Nominally significant effect on VHD of Bonferroni-significant gastrointestinal loci.

Disease	Gene	chr	Probe_bp	topSNP	topSNP_bp	A1	A2	Freq	b_SMR	se_SMR	p_SMR	p_HEIDI
VHD	CFDP1	16	75397489	rs7201989	75471499	G	A	0.246	-0.056	0.018	0.001	0.119
VHD	STAT3	17	40502964	rs1053004	40466092	G	A	0.380	0.093	0.047	0.048	0.948
VHD	IMPDH2	3	49064299	rs34037363	48821036	T	C	0.114	-0.209	0.105	0.047	0.089
VHD	UBE2L3	22	21941029	rs2070512	21949411	C	A	0.194	-0.029	0.009	0.002	0.623
VHD	STMN3	20	62271061	rs909333	62270637	G	T	0.207	-0.076	0.036	0.036	0.093

C) Effect of AGER gene on VHD and gastrointestinal disorders.

Disease	Gene	chr	Probe_bp	topSNP	topSNP_bp	A1	A2	Freq	b_SMR	se_SMR	p_SMR	p_HEIDI
CD	AGER	6	32150423	rs693906	31835164	C	G	0.113	-0.312	0.120	0.009	6.16E-08
GORD	AGER	6	32150423	rs693906	31835164	C	G	0.113	-0.209	0.049	2.02E-05	0.022
IBD	AGER	6	32148745	rs204995	32154285	G	A	0.217	-0.252	0.108	0.020	1.60E-04
IBS	AGER	6	32150423	rs693906	31835164	C	G	0.113	-0.168	0.052	0.001	0.045
UC	AGER	6	32148745	rs204995	32154285	G	A	0.217	-0.870	0.185	2.59E-06	0.002
VHD	AGER	6	32150423	rs693906	31835164	C	G	0.113	0.212	0.052	4.82E-05	0.092

Supplementary table 6. Results of the summary-based Mendelian randomization integrating whole-blood expression quantitative trait loci. For each disease-gene association, we report the strongest effect observed between GTEX and eQTLGen datasets.

A) Bonferroni-significant disease-gene association with HEIDI P > 0.05.

Disease	Gene	chr	gene_bp	topSNP	topSNP_bp	A1	A2	Freq	b_SMR	se_SMR	p_SMR	p_HEIDI
CD	DNMT3A	2	25510652	rs7578575	25488819	A	T	0.329	-0.834	0.164	3.51E-07	0.383
IBD	DNMT3A	2	25510652	rs7578575	25488819	A	T	0.329	-0.745	0.130	9.22E-09	0.515
UC	DNMT3A	2	25510652	rs7578575	25488819	A	T	0.329	-0.627	0.156	5.73E-05	0.541
UC	CFDP1	16	75327596	rs9934007	75390532	C	T	0.754	0.273	0.070	9.61E-05	0.973
CD	STAT3	17	40502964	rs1053004	40466092	G	A	0.380	0.702	0.128	4.40E-08	0.056
CD	IMPDH2	3	49064299	rs9813813	49207093	A	T	0.110	-1.233	0.294	2.74E-05	0.213
IBD	IMPDH2	3	49064299	rs9813813	49207093	A	T	0.110	-1.328	0.258	2.57E-07	0.071
UC	IMPDH2	3	49064299	rs9813813	49207093	A	T	0.110	-1.445	0.307	2.53E-06	0.050
IBD	UBE2L3	22	21941029	rs2070512	21949411	C	A	0.194	0.163	0.021	1.06E-14	0.169
UC	UBE2L3	22	21941029	rs2070512	21949411	C	A	0.194	0.116	0.027	1.76E-05	0.698
CD	BCL2L15	1	1.14E+08	rs1217393	1.14E+08	A	G	0.271	-0.705	0.170	3.39E-05	0.184
CD	STMN3	20	62271061	rs909333	62270637	G	T	0.207	0.547	0.102	8.31E-08	0.200
IBD	STMN3	20	62271061	rs909333	62270637	G	T	0.207	0.555	0.087	2.01E-10	0.055
UC	STMN3	20	62271061	rs909333	62270637	G	T	0.207	0.498	0.098	4.04E-07	0.062
GORD	PBX2	6	32154964	rs693906	31835164	C	G	0.113	0.154	0.036	1.65E-05	0.058
VHD	AGER	6	32150423	rs693906	31835164	C	G	0.113	0.212	0.052	4.82E-05	0.092

B) Nominally significant effect on VHD of Bonferroni-significant gastrointestinal loci.

Disease	Gene	chr	Probe_bp	topSNP	topSNP_bp	A1	A2	Freq	b_SMR	se_SMR	p_SMR	p_HEIDI
VHD	CFDP1	16	75397489	rs7201989	75471499	G	A	0.246	-0.056	0.018	0.001	0.119
VHD	STAT3	17	40502964	rs1053004	40466092	G	A	0.380	0.093	0.047	0.048	0.948
VHD	IMPDH2	3	49064299	rs34037363	48821036	T	C	0.114	-0.209	0.105	0.047	0.089
VHD	UBE2L3	22	21941029	rs2070512	21949411	C	A	0.194	-0.029	0.009	0.002	0.623
VHD	STMN3	20	62271061	rs909333	62270637	G	T	0.207	-0.076	0.036	0.036	0.093

C) Effect of AGER gene on VHD and gastrointestinal disorders.

Disease	Gene	chr	Probe_bp	topSNP	topSNP_bp	A1	A2	Freq	b_SMR	se_SMR	p_SMR	p_HEIDI
CD	AGER	6	32150423	rs693906	31835164	C	G	0.113	-0.312	0.120	0.009	6.16E-08
GORD	AGER	6	32150423	rs693906	31835164	C	G	0.113	-0.209	0.049	2.02E-05	0.022
IBD	AGER	6	32148745	rs204995	32154285	G	A	0.217	-0.252	0.108	0.020	1.60E-04
IBS	AGER	6	32150423	rs693906	31835164	C	G	0.113	-0.168	0.052	0.001	0.045
UC	AGER	6	32148745	rs204995	32154285	G	A	0.217	-0.870	0.185	2.59E-06	0.002
VHD	AGER	6	32150423	rs693906	31835164	C	G	0.113	0.212	0.052	4.82E-05	0.092

Supplementary table 6. Results of the summary-based Mendelian randomization integrating whole-blood expression quantitative trait loci. For each disease-gene association, we report the strongest effect observed between GTEX and eQTLGen datasets.

A) Bonferroni-significant disease-gene association with HEIDI P > 0.05.

Disease	Gene	chr	gene_bp	topSNP	topSNP_bp	A1	A2	Freq	b_SMR	se_SMR	p_SMR	p_HEIDI
CD	DNMT3A	2	25510652	rs7578575	25488819	A	T	0.329	-0.834	0.164	3.51E-07	0.383
IBD	DNMT3A	2	25510652	rs7578575	25488819	A	T	0.329	-0.745	0.130	9.22E-09	0.515
UC	DNMT3A	2	25510652	rs7578575	25488819	A	T	0.329	-0.627	0.156	5.73E-05	0.541
UC	CFDP1	16	75327596	rs9934007	75390532	C	T	0.754	0.273	0.070	9.61E-05	0.973
CD	STAT3	17	40502964	rs1053004	40466092	G	A	0.380	0.702	0.128	4.40E-08	0.056
CD	IMPDH2	3	49064299	rs9813813	49207093	A	T	0.110	-1.233	0.294	2.74E-05	0.213
IBD	IMPDH2	3	49064299	rs9813813	49207093	A	T	0.110	-1.328	0.258	2.57E-07	0.071
UC	IMPDH2	3	49064299	rs9813813	49207093	A	T	0.110	-1.445	0.307	2.53E-06	0.050
IBD	UBE2L3	22	21941029	rs2070512	21949411	C	A	0.194	0.163	0.021	1.06E-14	0.169
UC	UBE2L3	22	21941029	rs2070512	21949411	C	A	0.194	0.116	0.027	1.76E-05	0.698
CD	BCL2L15	1	1.14E+08	rs1217393	1.14E+08	A	G	0.271	-0.705	0.170	3.39E-05	0.184
CD	STMN3	20	62271061	rs909333	62270637	G	T	0.207	0.547	0.102	8.31E-08	0.200
IBD	STMN3	20	62271061	rs909333	62270637	G	T	0.207	0.555	0.087	2.01E-10	0.055
UC	STMN3	20	62271061	rs909333	62270637	G	T	0.207	0.498	0.098	4.04E-07	0.062
GORD	PBX2	6	32154964	rs693906	31835164	C	G	0.113	0.154	0.036	1.65E-05	0.058
VHD	AGER	6	32150423	rs693906	31835164	C	G	0.113	0.212	0.052	4.82E-05	0.092

B) Nominally significant effect on VHD of Bonferroni-significant gastrointestinal loci.

Disease	Gene	chr	Probe_bp	topSNP	topSNP_bp	A1	A2	Freq	b_SMR	se_SMR	p_SMR	p_HEIDI
VHD	CFDP1	16	75397489	rs7201989	75471499	G	A	0.246	-0.056	0.018	0.001	0.119
VHD	STAT3	17	40502964	rs1053004	40466092	G	A	0.380	0.093	0.047	0.048	0.948
VHD	IMPDH2	3	49064299	rs34037363	48821036	T	C	0.114	-0.209	0.105	0.047	0.089
VHD	UBE2L3	22	21941029	rs2070512	21949411	C	A	0.194	-0.029	0.009	0.002	0.623
VHD	STMN3	20	62271061	rs909333	62270637	G	T	0.207	-0.076	0.036	0.036	0.093

C) Effect of AGER gene on VHD and gastrointestinal disorders.

Disease	Gene	chr	Probe_bp	topSNP	topSNP_bp	A1	A2	Freq	b_SMR	se_SMR	p_SMR	p_HEIDI
CD	AGER	6	32150423	rs693906	31835164	C	G	0.113	-0.312	0.120	0.009	6.16E-08
GORD	AGER	6	32150423	rs693906	31835164	C	G	0.113	-0.209	0.049	2.02E-05	0.022
IBD	AGER	6	32148745	rs204995	32154285	G	A	0.217	-0.252	0.108	0.020	1.60E-04
IBS	AGER	6	32150423	rs693906	31835164	C	G	0.113	-0.168	0.052	0.001	0.045
UC	AGER	6	32148745	rs204995	32154285	G	A	0.217	-0.870	0.185	2.59E-06	0.002
VHD	AGER	6	32150423	rs693906	31835164	C	G	0.113	0.212	0.052	4.82E-05	0.092

Supplementary table 6. Results of the summary-based Mendelian randomization integrating whole-blood expression quantitative trait loci. For each disease-gene association, we report the strongest effect observed between GTEX and eQTLGen datasets.

A) Bonferroni-significant disease-gene association with HEIDI P > 0.05.

Disease	Gene	chr	gene_bp	topSNP	topSNP_bp	A1	A2	Freq	b_SMR	se_SMR	p_SMR	p_HEIDI
CD	DNMT3A	2	25510652	rs7578575	25488819	A	T	0.329	-0.834	0.164	3.51E-07	0.383
IBD	DNMT3A	2	25510652	rs7578575	25488819	A	T	0.329	-0.745	0.130	9.22E-09	0.515
UC	DNMT3A	2	25510652	rs7578575	25488819	A	T	0.329	-0.627	0.156	5.73E-05	0.541
UC	CFDP1	16	75327596	rs9934007	75390532	C	T	0.754	0.273	0.070	9.61E-05	0.973
CD	STAT3	17	40502964	rs1053004	40466092	G	A	0.380	0.702	0.128	4.40E-08	0.056
CD	IMPDH2	3	49064299	rs9813813	49207093	A	T	0.110	-1.233	0.294	2.74E-05	0.213
IBD	IMPDH2	3	49064299	rs9813813	49207093	A	T	0.110	-1.328	0.258	2.57E-07	0.071
UC	IMPDH2	3	49064299	rs9813813	49207093	A	T	0.110	-1.445	0.307	2.53E-06	0.050
IBD	UBE2L3	22	21941029	rs2070512	21949411	C	A	0.194	0.163	0.021	1.06E-14	0.169
UC	UBE2L3	22	21941029	rs2070512	21949411	C	A	0.194	0.116	0.027	1.76E-05	0.698
CD	BCL2L15	1	1.14E+08	rs1217393	1.14E+08	A	G	0.271	-0.705	0.170	3.39E-05	0.184
CD	STMN3	20	62271061	rs909333	62270637	G	T	0.207	0.547	0.102	8.31E-08	0.200
IBD	STMN3	20	62271061	rs909333	62270637	G	T	0.207	0.555	0.087	2.01E-10	0.055
UC	STMN3	20	62271061	rs909333	62270637	G	T	0.207	0.498	0.098	4.04E-07	0.062
GORD	PBX2	6	32154964	rs693906	31835164	C	G	0.113	0.154	0.036	1.65E-05	0.058
VHD	AGER	6	32150423	rs693906	31835164	C	G	0.113	0.212	0.052	4.82E-05	0.092

B) Nominally significant effect on VHD of Bonferroni-significant gastrointestinal loci.

Disease	Gene	chr	Probe_bp	topSNP	topSNP_bp	A1	A2	Freq	b_SMR	se_SMR	p_SMR	p_HEIDI
VHD	CFDP1	16	75397489	rs7201989	75471499	G	A	0.246	-0.056	0.018	0.001	0.119
VHD	STAT3	17	40502964	rs1053004	40466092	G	A	0.380	0.093	0.047	0.048	0.948
VHD	IMPDH2	3	49064299	rs34037363	48821036	T	C	0.114	-0.209	0.105	0.047	0.089
VHD	UBE2L3	22	21941029	rs2070512	21949411	C	A	0.194	-0.029	0.009	0.002	0.623
VHD	STMN3	20	62271061	rs909333	62270637	G	T	0.207	-0.076	0.036	0.036	0.093

C) Effect of AGER gene on VHD and gastrointestinal disorders.

Disease	Gene	chr	Probe_bp	topSNP	topSNP_bp	A1	A2	Freq	b_SMR	se_SMR	p_SMR	p_HEIDI
CD	AGER	6	32150423	rs693906	31835164	C	G	0.113	-0.312	0.120	0.009	6.16E-08
GORD	AGER	6	32150423	rs693906	31835164	C	G	0.113	-0.209	0.049	2.02E-05	0.022
IBD	AGER	6	32148745	rs204995	32154285	G	A	0.217	-0.252	0.108	0.020	1.60E-04
IBS	AGER	6	32150423	rs693906	31835164	C	G	0.113	-0.168	0.052	0.001	0.045
UC	AGER	6	32148745	rs204995	32154285	G	A	0.217	-0.870	0.185	2.59E-06	0.002
VHD	AGER	6	32150423	rs693906	31835164	C	G	0.113	0.212	0.052	4.82E-05	0.092

Supplementary table 6. Results of the summary-based Mendelian randomization integrating whole-blood expression quantitative trait loci. For each disease-gene association, we report the strongest effect observed between GTEX and eQTLGen datasets.

A) Bonferroni-significant disease-gene association with HEIDI P > 0.05.

Disease	Gene	chr	gene_bp	topSNP	topSNP_bp	A1	A2	Freq	b_SMR	se_SMR	p_SMR	p_HEIDI
CD	DNMT3A	2	25510652	rs7578575	25488819	A	T	0.329	-0.834	0.164	3.51E-07	0.383
IBD	DNMT3A	2	25510652	rs7578575	25488819	A	T	0.329	-0.745	0.130	9.22E-09	0.515
UC	DNMT3A	2	25510652	rs7578575	25488819	A	T	0.329	-0.627	0.156	5.73E-05	0.541
UC	CFDP1	16	75327596	rs9934007	75390532	C	T	0.754	0.273	0.070	9.61E-05	0.973
CD	STAT3	17	40502964	rs1053004	40466092	G	A	0.380	0.702	0.128	4.40E-08	0.056
CD	IMPDH2	3	49064299	rs9813813	49207093	A	T	0.110	-1.233	0.294	2.74E-05	0.213
IBD	IMPDH2	3	49064299	rs9813813	49207093	A	T	0.110	-1.328	0.258	2.57E-07	0.071
UC	IMPDH2	3	49064299	rs9813813	49207093	A	T	0.110	-1.445	0.307	2.53E-06	0.050
IBD	UBE2L3	22	21941029	rs2070512	21949411	C	A	0.194	0.163	0.021	1.06E-14	0.169
UC	UBE2L3	22	21941029	rs2070512	21949411	C	A	0.194	0.116	0.027	1.76E-05	0.698
CD	BCL2L15	1	1.14E+08	rs1217393	1.14E+08	A	G	0.271	-0.705	0.170	3.39E-05	0.184
CD	STMN3	20	62271061	rs909333	62270637	G	T	0.207	0.547	0.102	8.31E-08	0.200
IBD	STMN3	20	62271061	rs909333	62270637	G	T	0.207	0.555	0.087	2.01E-10	0.055
UC	STMN3	20	62271061	rs909333	62270637	G	T	0.207	0.498	0.098	4.04E-07	0.062
GORD	PBX2	6	32154964	rs693906	31835164	C	G	0.113	0.154	0.036	1.65E-05	0.058
VHD	AGER	6	32150423	rs693906	31835164	C	G	0.113	0.212	0.052	4.82E-05	0.092

B) Nominally significant effect on VHD of Bonferroni-significant gastrointestinal loci.

Disease	Gene	chr	Probe_bp	topSNP	topSNP_bp	A1	A2	Freq	b_SMR	se_SMR	p_SMR	p_HEIDI
VHD	CFDP1	16	75397489	rs7201989	75471499	G	A	0.246	-0.056	0.018	0.001	0.119
VHD	STAT3	17	40502964	rs1053004	40466092	G	A	0.380	0.093	0.047	0.048	0.948
VHD	IMPDH2	3	49064299	rs34037363	48821036	T	C	0.114	-0.209	0.105	0.047	0.089
VHD	UBE2L3	22	21941029	rs2070512	21949411	C	A	0.194	-0.029	0.009	0.002	0.623
VHD	STMN3	20	62271061	rs909333	62270637	G	T	0.207	-0.076	0.036	0.036	0.093

C) Effect of AGER gene on VHD and gastrointestinal disorders.

Disease	Gene	chr	Probe_bp	topSNP	topSNP_bp	A1	A2	Freq	b_SMR	se_SMR	p_SMR	p_HEIDI
CD	AGER	6	32150423	rs693906	31835164	C	G	0.113	-0.312	0.120	0.009	6.16E-08
GORD	AGER	6	32150423	rs693906	31835164	C	G	0.113	-0.209	0.049	2.02E-05	0.022
IBD	AGER	6	32148745	rs204995	32154285	G	A	0.217	-0.252	0.108	0.020	1.60E-04
IBS	AGER	6	32150423	rs693906	31835164	C	G	0.113	-0.168	0.052	0.001	0.045
UC	AGER	6	32148745	rs204995	32154285	G	A	0.217	-0.870	0.185	2.59E-06	0.002
VHD	AGER	6	32150423	rs693906	31835164	C	G	0.113	0.212	0.052	4.82E-05	0.092

Supplementary table 6. Results of the summary-based Mendelian randomization integrating whole-blood expression quantitative trait loci. For each disease-gene association, we report the strongest effect observed between GTEX and eQTLGen datasets.

A) Bonferroni-significant disease-gene association with HEIDI P > 0.05.

Disease	Gene	chr	gene_bp	topSNP	topSNP_bp	A1	A2	Freq	b_SMR	se_SMR	p_SMR	p_HEIDI
CD	DNMT3A	2	25510652	rs7578575	25488819	A	T	0.329	-0.834	0.164	3.51E-07	0.383
IBD	DNMT3A	2	25510652	rs7578575	25488819	A	T	0.329	-0.745	0.130	9.22E-09	0.515
UC	DNMT3A	2	25510652	rs7578575	25488819	A	T	0.329	-0.627	0.156	5.73E-05	0.541
UC	CFDP1	16	75327596	rs9934007	75390532	C	T	0.754	0.273	0.070	9.61E-05	0.973
CD	STAT3	17	40502964	rs1053004	40466092	G	A	0.380	0.702	0.128	4.40E-08	0.056
CD	IMPDH2	3	49064299	rs9813813	49207093	A	T	0.110	-1.233	0.294	2.74E-05	0.213
IBD	IMPDH2	3	49064299	rs9813813	49207093	A	T	0.110	-1.328	0.258	2.57E-07	0.071
UC	IMPDH2	3	49064299	rs9813813	49207093	A	T	0.110	-1.445	0.307	2.53E-06	0.050
IBD	UBE2L3	22	21941029	rs2070512	21949411	C	A	0.194	0.163	0.021	1.06E-14	0.169
UC	UBE2L3	22	21941029	rs2070512	21949411	C	A	0.194	0.116	0.027	1.76E-05	0.698
CD	BCL2L15	1	1.14E+08	rs1217393	1.14E+08	A	G	0.271	-0.705	0.170	3.39E-05	0.184
CD	STMN3	20	62271061	rs909333	62270637	G	T	0.207	0.547	0.102	8.31E-08	0.200
IBD	STMN3	20	62271061	rs909333	62270637	G	T	0.207	0.555	0.087	2.01E-10	0.055
UC	STMN3	20	62271061	rs909333	62270637	G	T	0.207	0.498	0.098	4.04E-07	0.062
GORD	PBX2	6	32154964	rs693906	31835164	C	G	0.113	0.154	0.036	1.65E-05	0.058
VHD	AGER	6	32150423	rs693906	31835164	C	G	0.113	0.212	0.052	4.82E-05	0.092

B) Nominally significant effect on VHD of Bonferroni-significant gastrointestinal loci.

Disease	Gene	chr	Probe_bp	topSNP	topSNP_bp	A1	A2	Freq	b_SMR	se_SMR	p_SMR	p_HEIDI
VHD	CFDP1	16	75397489	rs7201989	75471499	G	A	0.246	-0.056	0.018	0.001	0.119
VHD	STAT3	17	40502964	rs1053004	40466092	G	A	0.380	0.093	0.047	0.048	0.948
VHD	IMPDH2	3	49064299	rs34037363	48821036	T	C	0.114	-0.209	0.105	0.047	0.089
VHD	UBE2L3	22	21941029	rs2070512	21949411	C	A	0.194	-0.029	0.009	0.002	0.623
VHD	STMN3	20	62271061	rs909333	62270637	G	T	0.207	-0.076	0.036	0.036	0.093

C) Effect of AGER gene on VHD and gastrointestinal disorders.

Disease	Gene	chr	Probe_bp	topSNP	topSNP_bp	A1	A2	Freq	b_SMR	se_SMR	p_SMR	p_HEIDI
CD	AGER	6	32150423	rs693906	31835164	C	G	0.113	-0.312	0.120	0.009	6.16E-08
GORD	AGER	6	32150423	rs693906	31835164	C	G	0.113	-0.209	0.049	2.02E-05	0.022
IBD	AGER	6	32148745	rs204995	32154285	G	A	0.217	-0.252	0.108	0.020	1.60E-04
IBS	AGER	6	32150423	rs693906	31835164	C	G	0.113	-0.168	0.052	0.001	0.045
UC	AGER	6	32148745	rs204995	32154285	G	A	0.217	-0.870	0.185	2.59E-06	0.002
VHD	AGER	6	32150423	rs693906	31835164	C	G	0.113	0.212	0.052	4.82E-05	0.092

Supplementary table 6. Results of the summary-based Mendelian randomization integrating whole-blood expression quantitative trait loci. For each disease-gene association, we report the strongest effect observed between GTEX and eQTLGen datasets.

A) Bonferroni-significant disease-gene association with HEIDI P > 0.05.

Disease	Gene	chr	gene_bp	topSNP	topSNP_bp	A1	A2	Freq	b_SMR	se_SMR	p_SMR	p_HEIDI
CD	DNMT3A	2	25510652	rs7578575	25488819	A	T	0.329	-0.834	0.164	3.51E-07	0.383
IBD	DNMT3A	2	25510652	rs7578575	25488819	A	T	0.329	-0.745	0.130	9.22E-09	0.515
UC	DNMT3A	2	25510652	rs7578575	25488819	A	T	0.329	-0.627	0.156	5.73E-05	0.541
UC	CFDP1	16	75327596	rs9934007	75390532	C	T	0.754	0.273	0.070	9.61E-05	0.973
CD	STAT3	17	40502964	rs1053004	40466092	G	A	0.380	0.702	0.128	4.40E-08	0.056
CD	IMPDH2	3	49064299	rs9813813	49207093	A	T	0.110	-1.233	0.294	2.74E-05	0.213
IBD	IMPDH2	3	49064299	rs9813813	49207093	A	T	0.110	-1.328	0.258	2.57E-07	0.071
UC	IMPDH2	3	49064299	rs9813813	49207093	A	T	0.110	-1.445	0.307	2.53E-06	0.050
IBD	UBE2L3	22	21941029	rs2070512	21949411	C	A	0.194	0.163	0.021	1.06E-14	0.169
UC	UBE2L3	22	21941029	rs2070512	21949411	C	A	0.194	0.116	0.027	1.76E-05	0.698
CD	BCL2L15	1	1.14E+08	rs1217393	1.14E+08	A	G	0.271	-0.705	0.170	3.39E-05	0.184
CD	STMN3	20	62271061	rs909333	62270637	G	T	0.207	0.547	0.102	8.31E-08	0.200
IBD	STMN3	20	62271061	rs909333	62270637	G	T	0.207	0.555	0.087	2.01E-10	0.055
UC	STMN3	20	62271061	rs909333	62270637	G	T	0.207	0.498	0.098	4.04E-07	0.062
GORD	PBX2	6	32154964	rs693906	31835164	C	G	0.113	0.154	0.036	1.65E-05	0.058
VHD	AGER	6	32150423	rs693906	31835164	C	G	0.113	0.212	0.052	4.82E-05	0.092

B) Nominally significant effect on VHD of Bonferroni-significant gastrointestinal loci.

Disease	Gene	chr	Probe_bp	topSNP	topSNP_bp	A1	A2	Freq	b_SMR	se_SMR	p_SMR	p_HEIDI
VHD	CFDP1	16	75397489	rs7201989	75471499	G	A	0.246	-0.056	0.018	0.001	0.119
VHD	STAT3	17	40502964	rs1053004	40466092	G	A	0.380	0.093	0.047	0.048	0.948
VHD	IMPDH2	3	49064299	rs34037363	48821036	T	C	0.114	-0.209	0.105	0.047	0.089
VHD	UBE2L3	22	21941029	rs2070512	21949411	C	A	0.194	-0.029	0.009	0.002	0.623
VHD	STMN3	20	62271061	rs909333	62270637	G	T	0.207	-0.076	0.036	0.036	0.093

C) Effect of AGER gene on VHD and gastrointestinal disorders.

Disease	Gene	chr	Probe_bp	topSNP	topSNP_bp	A1	A2	Freq	b_SMR	se_SMR	p_SMR	p_HEIDI
CD	AGER	6	32150423	rs693906	31835164	C	G	0.113	-0.312	0.120	0.009	6.16E-08
GORD	AGER	6	32150423	rs693906	31835164	C	G	0.113	-0.209	0.049	2.02E-05	0.022
IBD	AGER	6	32148745	rs204995	32154285	G	A	0.217	-0.252	0.108	0.020	1.60E-04
IBS	AGER	6	32150423	rs693906	31835164	C	G	0.113	-0.168	0.052	0.001	0.045
UC	AGER	6	32148745	rs204995	32154285	G	A	0.217	-0.870	0.185	2.59E-06	0.002
VHD	AGER	6	32150423	rs693906	31835164	C	G	0.113	0.212	0.052	4.82E-05	0.092

Supplementary table 6. Results of the summary-based Mendelian randomization integrating whole-blood expression quantitative trait loci. For each disease-gene association, we report the strongest effect observed between GTEX and eQTLGen datasets.

A) Bonferroni-significant disease-gene association with HEIDI P > 0.05.

Disease	Gene	chr	gene_bp	topSNP	topSNP_bp	A1	A2	Freq	b_SMR	se_SMR	p_SMR	p_HEIDI
CD	DNMT3A	2	25510652	rs7578575	25488819	A	T	0.329	-0.834	0.164	3.51E-07	0.383
IBD	DNMT3A	2	25510652	rs7578575	25488819	A	T	0.329	-0.745	0.130	9.22E-09	0.515
UC	DNMT3A	2	25510652	rs7578575	25488819	A	T	0.329	-0.627	0.156	5.73E-05	0.541
UC	CFDP1	16	75327596	rs9934007	75390532	C	T	0.754	0.273	0.070	9.61E-05	0.973
CD	STAT3	17	40502964	rs1053004	40466092	G	A	0.380	0.702	0.128	4.40E-08	0.056
CD	IMPDH2	3	49064299	rs9813813	49207093	A	T	0.110	-1.233	0.294	2.74E-05	0.213
IBD	IMPDH2	3	49064299	rs9813813	49207093	A	T	0.110	-1.328	0.258	2.57E-07	0.071
UC	IMPDH2	3	49064299	rs9813813	49207093	A	T	0.110	-1.445	0.307	2.53E-06	0.050
IBD	UBE2L3	22	21941029	rs2070512	21949411	C	A	0.194	0.163	0.021	1.06E-14	0.169
UC	UBE2L3	22	21941029	rs2070512	21949411	C	A	0.194	0.116	0.027	1.76E-05	0.698
CD	BCL2L15	1	1.14E+08	rs1217393	1.14E+08	A	G	0.271	-0.705	0.170	3.39E-05	0.184
CD	STMN3	20	62271061	rs909333	62270637	G	T	0.207	0.547	0.102	8.31E-08	0.200
IBD	STMN3	20	62271061	rs909333	62270637	G	T	0.207	0.555	0.087	2.01E-10	0.055
UC	STMN3	20	62271061	rs909333	62270637	G	T	0.207	0.498	0.098	4.04E-07	0.062
GORD	PBX2	6	32154964	rs693906	31835164	C	G	0.113	0.154	0.036	1.65E-05	0.058
VHD	AGER	6	32150423	rs693906	31835164	C	G	0.113	0.212	0.052	4.82E-05	0.092

B) Nominally significant effect on VHD of Bonferroni-significant gastrointestinal loci.

Disease	Gene	chr	Probe_bp	topSNP	topSNP_bp	A1	A2	Freq	b_SMR	se_SMR	p_SMR	p_HEIDI
VHD	CFDP1	16	75397489	rs7201989	75471499	G	A	0.246	-0.056	0.018	0.001	0.119
VHD	STAT3	17	40502964	rs1053004	40466092	G	A	0.380	0.093	0.047	0.048	0.948
VHD	IMPDH2	3	49064299	rs34037363	48821036	T	C	0.114	-0.209	0.105	0.047	0.089
VHD	UBE2L3	22	21941029	rs2070512	21949411	C	A	0.194	-0.029	0.009	0.002	0.623
VHD	STMN3	20	62271061	rs909333	62270637	G	T	0.207	-0.076	0.036	0.036	0.093

C) Effect of AGER gene on VHD and gastrointestinal disorders.

Disease	Gene	chr	Probe_bp	topSNP	topSNP_bp	A1	A2	Freq	b_SMR	se_SMR	p_SMR	p_HEIDI
CD	AGER	6	32150423	rs693906	31835164	C	G	0.113	-0.312	0.120	0.009	6.16E-08
GORD	AGER	6	32150423	rs693906	31835164	C	G	0.113	-0.209	0.049	2.02E-05	0.022
IBD	AGER	6	32148745	rs204995	32154285	G	A	0.217	-0.252	0.108	0.020	1.60E-04
IBS	AGER	6	32150423	rs693906	31835164	C	G	0.113	-0.168	0.052	0.001	0.045
UC	AGER	6	32148745	rs204995	32154285	G	A	0.217	-0.870	0.185	2.59E-06	0.002
VHD	AGER	6	32150423	rs693906	31835164	C	G	0.113	0.212	0.052	4.82E-05	0.092

Supplementary table 6. Results of the summary-based Mendelian randomization integrating whole-blood expression quantitative trait loci. For each disease-gene association, we report the strongest effect observed between GTEX and eQTLGen datasets.

A) Bonferroni-significant disease-gene association with HEIDI P > 0.05.

Disease	Gene	chr	gene_bp	topSNP	topSNP_bp	A1	A2	Freq	b_SMR	se_SMR	p_SMR	p_HEIDI
CD	DNMT3A	2	25510652	rs7578575	25488819	A	T	0.329	-0.834	0.164	3.51E-07	0.383
IBD	DNMT3A	2	25510652	rs7578575	25488819	A	T	0.329	-0.745	0.130	9.22E-09	0.515
UC	DNMT3A	2	25510652	rs7578575	25488819	A	T	0.329	-0.627	0.156	5.73E-05	0.541
UC	CFDP1	16	75327596	rs9934007	75390532	C	T	0.754	0.273	0.070	9.61E-05	0.973
CD	STAT3	17	40502964	rs1053004	40466092	G	A	0.380	0.702	0.128	4.40E-08	0.056
CD	IMPDH2	3	49064299	rs9813813	49207093	A	T	0.110	-1.233	0.294	2.74E-05	0.213
IBD	IMPDH2	3	49064299	rs9813813	49207093	A	T	0.110	-1.328	0.258	2.57E-07	0.071
UC	IMPDH2	3	49064299	rs9813813	49207093	A	T	0.110	-1.445	0.307	2.53E-06	0.050
IBD	UBE2L3	22	21941029	rs2070512	21949411	C	A	0.194	0.163	0.021	1.06E-14	0.169
UC	UBE2L3	22	21941029	rs2070512	21949411	C	A	0.194	0.116	0.027	1.76E-05	0.698
CD	BCL2L15	1	1.14E+08	rs1217393	1.14E+08	A	G	0.271	-0.705	0.170	3.39E-05	0.184
CD	STMN3	20	62271061	rs909333	62270637	G	T	0.207	0.547	0.102	8.31E-08	0.200
IBD	STMN3	20	62271061	rs909333	62270637	G	T	0.207	0.555	0.087	2.01E-10	0.055
UC	STMN3	20	62271061	rs909333	62270637	G	T	0.207	0.498	0.098	4.04E-07	0.062
GORD	PBX2	6	32154964	rs693906	31835164	C	G	0.113	0.154	0.036	1.65E-05	0.058
VHD	AGER	6	32150423	rs693906	31835164	C	G	0.113	0.212	0.052	4.82E-05	0.092

B) Nominally significant effect on VHD of Bonferroni-significant gastrointestinal loci.

Disease	Gene	chr	Probe_bp	topSNP	topSNP_bp	A1	A2	Freq	b_SMR	se_SMR	p_SMR	p_HEIDI
VHD	CFDP1	16	75397489	rs7201989	75471499	G	A	0.246	-0.056	0.018	0.001	0.119
VHD	STAT3	17	40502964	rs1053004	40466092	G	A	0.380	0.093	0.047	0.048	0.948
VHD	IMPDH2	3	49064299	rs34037363	48821036	T	C	0.114	-0.209	0.105	0.047	0.089
VHD	UBE2L3	22	21941029	rs2070512	21949411	C	A	0.194	-0.029	0.009	0.002	0.623
VHD	STMN3	20	62271061	rs909333	62270637	G	T	0.207	-0.076	0.036	0.036	0.093

C) Effect of AGER gene on VHD and gastrointestinal disorders.

Disease	Gene	chr	Probe_bp	topSNP	topSNP_bp	A1	A2	Freq	b_SMR	se_SMR	p_SMR	p_HEIDI
CD	AGER	6	32150423	rs693906	31835164	C	G	0.113	-0.312	0.120	0.009	6.16E-08
GORD	AGER	6	32150423	rs693906	31835164	C	G	0.113	-0.209	0.049	2.02E-05	0.022
IBD	AGER	6	32148745	rs204995	32154285	G	A	0.217	-0.252	0.108	0.020	1.60E-04
IBS	AGER	6	32150423	rs693906	31835164	C	G	0.113	-0.168	0.052	0.001	0.045
UC	AGER	6	32148745	rs204995	32154285	G	A	0.217	-0.870	0.185	2.59E-06	0.002
VHD	AGER	6	32150423	rs693906	31835164	C	G	0.113	0.212	0.052	4.82E-05	0.092

Supplementary table 6. Results of the summary-based Mendelian randomization integrating whole-blood expression quantitative trait loci. For each disease-gene association, we report the strongest effect observed between GTEX and eQTLGen datasets.

A) Bonferroni-significant disease-gene association with HEIDI P > 0.05.

Disease	Gene	chr	gene_bp	topSNP	topSNP_bp	A1	A2	Freq	b_SMR	se_SMR	p_SMR	p_HEIDI
CD	DNMT3A	2	25510652	rs7578575	25488819	A	T	0.329	-0.834	0.164	3.51E-07	0.383
IBD	DNMT3A	2	25510652	rs7578575	25488819	A	T	0.329	-0.745	0.130	9.22E-09	0.515
UC	DNMT3A	2	25510652	rs7578575	25488819	A	T	0.329	-0.627	0.156	5.73E-05	0.541
UC	CFDP1	16	75327596	rs9934007	75390532	C	T	0.754	0.273	0.070	9.61E-05	0.973
CD	STAT3	17	40502964	rs1053004	40466092	G	A	0.380	0.702	0.128	4.40E-08	0.056
CD	IMPDH2	3	49064299	rs9813813	49207093	A	T	0.110	-1.233	0.294	2.74E-05	0.213
IBD	IMPDH2	3	49064299	rs9813813	49207093	A	T	0.110	-1.328	0.258	2.57E-07	0.071
UC	IMPDH2	3	49064299	rs9813813	49207093	A	T	0.110	-1.445	0.307	2.53E-06	0.050
IBD	UBE2L3	22	21941029	rs2070512	21949411	C	A	0.194	0.163	0.021	1.06E-14	0.169
UC	UBE2L3	22	21941029	rs2070512	21949411	C	A	0.194	0.116	0.027	1.76E-05	0.698
CD	BCL2L15	1	1.14E+08	rs1217393	1.14E+08	A	G	0.271	-0.705	0.170	3.39E-05	0.184
CD	STMN3	20	62271061	rs909333	62270637	G	T	0.207	0.547	0.102	8.31E-08	0.200
IBD	STMN3	20	62271061	rs909333	62270637	G	T	0.207	0.555	0.087	2.01E-10	0.055
UC	STMN3	20	62271061	rs909333	62270637	G	T	0.207	0.498	0.098	4.04E-07	0.062
GORD	PBX2	6	32154964	rs693906	31835164	C	G	0.113	0.154	0.036	1.65E-05	0.058
VHD	AGER	6	32150423	rs693906	31835164	C	G	0.113	0.212	0.052	4.82E-05	0.092

B) Nominally significant effect on VHD of Bonferroni-significant gastrointestinal loci.

Disease	Gene	chr	Probe_bp	topSNP	topSNP_bp	A1	A2	Freq	b_SMR	se_SMR	p_SMR	p_HEIDI
VHD	CFDP1	16	75397489	rs7201989	75471499	G	A	0.246	-0.056	0.018	0.001	0.119
VHD	STAT3	17	40502964	rs1053004	40466092	G	A	0.380	0.093	0.047	0.048	0.948
VHD	IMPDH2	3	49064299	rs34037363	48821036	T	C	0.114	-0.209	0.105	0.047	0.089
VHD	UBE2L3	22	21941029	rs2070512	21949411	C	A	0.194	-0.029	0.009	0.002	0.623
VHD	STMN3	20	62271061	rs909333	62270637	G	T	0.207	-0.076	0.036	0.036	0.093

C) Effect of AGER gene on VHD and gastrointestinal disorders.

Disease	Gene	chr	Probe_bp	topSNP	topSNP_bp	A1	A2	Freq	b_SMR	se_SMR	p_SMR	p_HEIDI
CD	AGER	6	32150423	rs693906	31835164	C	G	0.113	-0.312	0.120	0.009	6.16E-08
GORD	AGER	6	32150423	rs693906	31835164	C	G	0.113	-0.209	0.049	2.02E-05	0.022
IBD	AGER	6	32148745	rs204995	32154285	G	A	0.217	-0.252	0.108	0.020	1.60E-04
IBS	AGER	6	32150423	rs693906	31835164	C	G	0.113	-0.168	0.052	0.001	0.045
UC	AGER	6	32148745	rs204995	32154285	G	A	0.217	-0.870	0.185	2.59E-06	0.002
VHD	AGER	6	32150423	rs693906	31835164	C	G	0.113	0.212	0.052	4.82E-05	0.092

Supplementary table 6. Results of the summary-based Mendelian randomization integrating whole-blood expression quantitative trait loci. For each disease-gene association, we report the strongest effect observed between GTEX and eQTLGen datasets.

A) Bonferroni-significant disease-gene association with HEIDI P > 0.05.

Disease	Gene	chr	gene_bp	topSNP	topSNP_bp	A1	A2	Freq	b_SMR	se_SMR	p_SMR	p_HEIDI
CD	DNMT3A	2	25510652	rs7578575	25488819	A	T	0.329	-0.834	0.164	3.51E-07	0.383
IBD	DNMT3A	2	25510652	rs7578575	25488819	A	T	0.329	-0.745	0.130	9.22E-09	0.515
UC	DNMT3A	2	25510652	rs7578575	25488819	A	T	0.329	-0.627	0.156	5.73E-05	0.541
UC	CFDP1	16	75327596	rs9934007	75390532	C	T	0.754	0.273	0.070	9.61E-05	0.973
CD	STAT3	17	40502964	rs1053004	40466092	G	A	0.380	0.702	0.128	4.40E-08	0.056
CD	IMPDH2	3	49064299	rs9813813	49207093	A	T	0.110	-1.233	0.294	2.74E-05	0.213
IBD	IMPDH2	3	49064299	rs9813813	49207093	A	T	0.110	-1.328	0.258	2.57E-07	0.071
UC	IMPDH2	3	49064299	rs9813813	49207093	A	T	0.110	-1.445	0.307	2.53E-06	0.050
IBD	UBE2L3	22	21941029	rs2070512	21949411	C	A	0.194	0.163	0.021	1.06E-14	0.169
UC	UBE2L3	22	21941029	rs2070512	21949411	C	A	0.194	0.116	0.027	1.76E-05	0.698
CD	BCL2L15	1	1.14E+08	rs1217393	1.14E+08	A	G	0.271	-0.705	0.170	3.39E-05	0.184
CD	STMN3	20	62271061	rs909333	62270637	G	T	0.207	0.547	0.102	8.31E-08	0.200
IBD	STMN3	20	62271061	rs909333	62270637	G	T	0.207	0.555	0.087	2.01E-10	0.055
UC	STMN3	20	62271061	rs909333	62270637	G	T	0.207	0.498	0.098	4.04E-07	0.062
GORD	PBX2	6	32154964	rs693906	31835164	C	G	0.113	0.154	0.036	1.65E-05	0.058
VHD	AGER	6	32150423	rs693906	31835164	C	G	0.113	0.212	0.052	4.82E-05	0.092

B) Nominally significant effect on VHD of Bonferroni-significant gastrointestinal loci.

Disease	Gene	chr	Probe_bp	topSNP	topSNP_bp	A1	A2	Freq	b_SMR	se_SMR	p_SMR	p_HEIDI
VHD	CFDP1	16	75397489	rs7201989	75471499	G	A	0.246	-0.056	0.018	0.001	0.119
VHD	STAT3	17	40502964	rs1053004	40466092	G	A	0.380	0.093	0.047	0.048	0.948
VHD	IMPDH2	3	49064299	rs34037363	48821036	T	C	0.114	-0.209	0.105	0.047	0.089
VHD	UBE2L3	22	21941029	rs2070512	21949411	C	A	0.194	-0.029	0.009	0.002	0.623
VHD	STMN3	20	62271061	rs909333	62270637	G	T	0.207	-0.076	0.036	0.036	0.093

C) Effect of AGER gene on VHD and gastrointestinal disorders.

Disease	Gene	chr	Probe_bp	topSNP	topSNP_bp	A1	A2	Freq	b_SMR	se_SMR	p_SMR	p_HEIDI
CD	AGER	6	32150423	rs693906	31835164	C	G	0.113	-0.312	0.120	0.009	6.16E-08
GORD	AGER	6	32150423	rs693906	31835164	C	G	0.113	-0.209	0.049	2.02E-05	0.022
IBD	AGER	6	32148745	rs204995	32154285	G	A	0.217	-0.252	0.108	0.020	1.60E-04
IBS	AGER	6	32150423	rs693906	31835164	C	G	0.113	-0.168	0.052	0.001	0.045
UC	AGER	6	32148745	rs204995	32154285	G	A	0.217	-0.870	0.185	2.59E-06	0.002
VHD	AGER	6	32150423	rs693906	31835164	C	G	0.113	0.212	0.052	4.82E-05	0.092

Supplementary table 6. Results of the summary-based Mendelian randomization integrating whole-blood expression quantitative trait loci. For each disease-gene association, we report the strongest effect observed between GTEX and eQTLGen datasets.

A) Bonferroni-significant disease-gene association with HEIDI P > 0.05.

Disease	Gene	chr	gene_bp	topSNP	topSNP_bp	A1	A2	Freq	b_SMR	se_SMR	p_SMR	p_HEIDI
CD	DNMT3A	2	25510652	rs7578575	25488819	A	T	0.329	-0.834	0.164	3.51E-07	0.383
IBD	DNMT3A	2	25510652	rs7578575	25488819	A	T	0.329	-0.745	0.130	9.22E-09	0.515
UC	DNMT3A	2	25510652	rs7578575	25488819	A	T	0.329	-0.627	0.156	5.73E-05	0.541
UC	CFDP1	16	75327596	rs9934007	75390532	C	T	0.754	0.273	0.070	9.61E-05	0.973
CD	STAT3	17	40502964	rs1053004	40466092	G	A	0.380	0.702	0.128	4.40E-08	0.056
CD	IMPDH2	3	49064299	rs9813813	49207093	A	T	0.110	-1.233	0.294	2.74E-05	0.213
IBD	IMPDH2	3	49064299	rs9813813	49207093	A	T	0.110	-1.328	0.258	2.57E-07	0.071
UC	IMPDH2	3	49064299	rs9813813	49207093	A	T	0.110	-1.445	0.307	2.53E-06	0.050
IBD	UBE2L3	22	21941029	rs2070512	21949411	C	A	0.194	0.163	0.021	1.06E-14	0.169
UC	UBE2L3	22	21941029	rs2070512	21949411	C	A	0.194	0.116	0.027	1.76E-05	0.698
CD	BCL2L15	1	1.14E+08	rs1217393	1.14E+08	A	G	0.271	-0.705	0.170	3.39E-05	0.184
CD	STMN3	20	62271061	rs909333	62270637	G	T	0.207	0.547	0.102	8.31E-08	0.200
IBD	STMN3	20	62271061	rs909333	62270637	G	T	0.207	0.555	0.087	2.01E-10	0.055
UC	STMN3	20	62271061	rs909333	62270637	G	T	0.207	0.498	0.098	4.04E-07	0.062
GORD	PBX2	6	32154964	rs693906	31835164	C	G	0.113	0.154	0.036	1.65E-05	0.058
VHD	AGER	6	32150423	rs693906	31835164	C	G	0.113	0.212	0.052	4.82E-05	0.092

B) Nominally significant effect on VHD of Bonferroni-significant gastrointestinal loci.

Disease	Gene	chr	Probe_bp	topSNP	topSNP_bp	A1	A2	Freq	b_SMR	se_SMR	p_SMR	p_HEIDI
VHD	CFDP1	16	75397489	rs7201989	75471499	G	A	0.246	-0.056	0.018	0.001	0.119
VHD	STAT3	17	40502964	rs1053004	40466092	G	A	0.380	0.093	0.047	0.048	0.948
VHD	IMPDH2	3	49064299	rs34037363	48821036	T	C	0.114	-0.209	0.105	0.047	0.089
VHD	UBE2L3	22	21941029	rs2070512	21949411	C	A	0.194	-0.029	0.009	0.002	0.623
VHD	STMN3	20	62271061	rs909333	62270637	G	T	0.207	-0.076	0.036	0.036	0.093

C) Effect of AGER gene on VHD and gastrointestinal disorders.

Disease	Gene	chr	Probe_bp	topSNP	topSNP_bp	A1	A2	Freq	b_SMR	se_SMR	p_SMR	p_HEIDI
CD	AGER	6	32150423	rs693906	31835164	C	G	0.113	-0.312	0.120	0.009	6.16E-08
GORD	AGER	6	32150423	rs693906	31835164	C	G	0.113	-0.209	0.049	2.02E-05	0.022
IBD	AGER	6	32148745	rs204995	32154285	G	A	0.217	-0.252	0.108	0.020	1.60E-04
IBS	AGER	6	32150423	rs693906	31835164	C	G	0.113	-0.168	0.052	0.001	0.045
UC	AGER	6	32148745	rs204995	32154285	G	A	0.217	-0.870	0.185	2.59E-06	0.002
VHD	AGER	6	32150423	rs693906	31835164	C	G	0.113	0.212	0.052	4.82E-05	0.092

Supplementary table 6. Results of the summary-based Mendelian randomization integrating whole-blood expression quantitative trait loci. For each disease-gene association, we report the strongest effect observed between GTEX and eQTLGen datasets.

A) Bonferroni-significant disease-gene association with HEIDI P > 0.05.

Disease	Gene	chr	gene_bp	topSNP	topSNP_bp	A1	A2	Freq	b_SMR	se_SMR	p_SMR	p_HEIDI
CD	DNMT3A	2	25510652	rs7578575	25488819	A	T	0.329	-0.834	0.164	3.51E-07	0.383
IBD	DNMT3A	2	25510652	rs7578575	25488819	A	T	0.329	-0.745	0.130	9.22E-09	0.515
UC	DNMT3A	2	25510652	rs7578575	25488819	A	T	0.329	-0.627	0.156	5.73E-05	0.541
UC	CFDP1	16	75327596	rs9934007	75390532	C	T	0.754	0.273	0.070	9.61E-05	0.973
CD	STAT3	17	40502964	rs1053004	40466092	G	A	0.380	0.702	0.128	4.40E-08	0.056
CD	IMPDH2	3	49064299	rs9813813	49207093	A	T	0.110	-1.233	0.294	2.74E-05	0.213
IBD	IMPDH2	3	49064299	rs9813813	49207093	A	T	0.110	-1.328	0.258	2.57E-07	0.071
UC	IMPDH2	3	49064299	rs9813813	49207093	A	T	0.110	-1.445	0.307	2.53E-06	0.050
IBD	UBE2L3	22	21941029	rs2070512	21949411	C	A	0.194	0.163	0.021	1.06E-14	0.169
UC	UBE2L3	22	21941029	rs2070512	21949411	C	A	0.194	0.116	0.027	1.76E-05	0.698
CD	BCL2L15	1	1.14E+08	rs1217393	1.14E+08	A	G	0.271	-0.705	0.170	3.39E-05	0.184
CD	STMN3	20	62271061	rs909333	62270637	G	T	0.207	0.547	0.102	8.31E-08	0.200
IBD	STMN3	20	62271061	rs909333	62270637	G	T	0.207	0.555	0.087	2.01E-10	0.055
UC	STMN3	20	62271061	rs909333	62270637	G	T	0.207	0.498	0.098	4.04E-07	0.062
GORD	PBX2	6	32154964	rs693906	31835164	C	G	0.113	0.154	0.036	1.65E-05	0.058
VHD	AGER	6	32150423	rs693906	31835164	C	G	0.113	0.212	0.052	4.82E-05	0.092

B) Nominally significant effect on VHD of Bonferroni-significant gastrointestinal loci.

Disease	Gene	chr	Probe_bp	topSNP	topSNP_bp	A1	A2	Freq	b_SMR	se_SMR	p_SMR	p_HEIDI
VHD	CFDP1	16	75397489	rs7201989	75471499	G	A	0.246	-0.056	0.018	0.001	0.119
VHD	STAT3	17	40502964	rs1053004	40466092	G	A	0.380	0.093	0.047	0.048	0.948
VHD	IMPDH2	3	49064299	rs34037363	48821036	T	C	0.114	-0.209	0.105	0.047	0.089
VHD	UBE2L3	22	21941029	rs2070512	21949411	C	A	0.194	-0.029	0.009	0.002	0.623
VHD	STMN3	20	62271061	rs909333	62270637	G	T	0.207	-0.076	0.036	0.036	0.093

C) Effect of AGER gene on VHD and gastrointestinal disorders.

Disease	Gene	chr	Probe_bp	topSNP	topSNP_bp	A1	A2	Freq	b_SMR	se_SMR	p_SMR	p_HEIDI
CD	AGER	6	32150423	rs693906	31835164	C	G	0.113	-0.312	0.120	0.009	6.16E-08
GORD	AGER	6	32150423	rs693906	31835164	C	G	0.113	-0.209	0.049	2.02E-05	0.022
IBD	AGER	6	32148745	rs204995	32154285	G	A	0.217	-0.252	0.108	0.020	1.60E-04
IBS	AGER	6	32150423	rs693906	31835164	C	G	0.113	-0.168	0.052	0.001	0.045
UC	AGER	6	32148745	rs204995	32154285	G	A	0.217	-0.870	0.185	2.59E-06	0.002
VHD	AGER	6	32150423	rs693906	31835164	C	G	0.113	0.212	0.052	4.82E-05	0.092

Supplementary table 6. Results of the summary-based Mendelian randomization integrating whole-blood expression quantitative trait loci. For each disease-gene association, we report the strongest effect observed between GTEX and eQTLGen datasets.

A) Bonferroni-significant disease-gene association with HEIDI P > 0.05.

Disease	Gene	chr	gene_bp	topSNP	topSNP_bp	A1	A2	Freq	b_SMR	se_SMR	p_SMR	p_HEIDI
CD	DNMT3A	2	25510652	rs7578575	25488819	A	T	0.329	-0.834	0.164	3.51E-07	0.383
IBD	DNMT3A	2	25510652	rs7578575	25488819	A	T	0.329	-0.745	0.130	9.22E-09	0.515
UC	DNMT3A	2	25510652	rs7578575	25488819	A	T	0.329	-0.627	0.156	5.73E-05	0.541
UC	CFDP1	16	75327596	rs9934007	75390532	C	T	0.754	0.273	0.070	9.61E-05	0.973
CD	STAT3	17	40502964	rs1053004	40466092	G	A	0.380	0.702	0.128	4.40E-08	0.056
CD	IMPDH2	3	49064299	rs9813813	49207093	A	T	0.110	-1.233	0.294	2.74E-05	0.213
IBD	IMPDH2	3	49064299	rs9813813	49207093	A	T	0.110	-1.328	0.258	2.57E-07	0.071
UC	IMPDH2	3	49064299	rs9813813	49207093	A	T	0.110	-1.445	0.307	2.53E-06	0.050
IBD	UBE2L3	22	21941029	rs2070512	21949411	C	A	0.194	0.163	0.021	1.06E-14	0.169
UC	UBE2L3	22	21941029	rs2070512	21949411	C	A	0.194	0.116	0.027	1.76E-05	0.698
CD	BCL2L15	1	1.14E+08	rs1217393	1.14E+08	A	G	0.271	-0.705	0.170	3.39E-05	0.184
CD	STMN3	20	62271061	rs909333	62270637	G	T	0.207	0.547	0.102	8.31E-08	0.200
IBD	STMN3	20	62271061	rs909333	62270637	G	T	0.207	0.555	0.087	2.01E-10	0.055
UC	STMN3	20	62271061	rs909333	62270637	G	T	0.207	0.498	0.098	4.04E-07	0.062
GORD	PBX2	6	32154964	rs693906	31835164	C	G	0.113	0.154	0.036	1.65E-05	0.058
VHD	AGER	6	32150423	rs693906	31835164	C	G	0.113	0.212	0.052	4.82E-05	0.092

B) Nominally significant effect on VHD of Bonferroni-significant gastrointestinal loci.

Disease	Gene	chr	Probe_bp	topSNP	topSNP_bp	A1	A2	Freq	b_SMR	se_SMR	p_SMR	p_HEIDI
VHD	CFDP1	16	75397489	rs7201989	75471499	G	A	0.246	-0.056	0.018	0.001	0.119
VHD	STAT3	17	40502964	rs1053004	40466092	G	A	0.380	0.093	0.047	0.048	0.948
VHD	IMPDH2	3	49064299	rs34037363	48821036	T	C	0.114	-0.209	0.105	0.047	0.089
VHD	UBE2L3	22	21941029	rs2070512	21949411	C	A	0.194	-0.029	0.009	0.002	0.623
VHD	STMN3	20	62271061	rs909333	62270637	G	T	0.207	-0.076	0.036	0.036	0.093

C) Effect of AGER gene on VHD and gastrointestinal disorders.

Disease	Gene	chr	Probe_bp	topSNP	topSNP_bp	A1	A2	Freq	b_SMR	se_SMR	p_SMR	p_HEIDI
CD	AGER	6	32150423	rs693906	31835164	C	G	0.113	-0.312	0.120	0.009	6.16E-08
GORD	AGER	6	32150423	rs693906	31835164	C	G	0.113	-0.209	0.049	2.02E-05	0.022
IBD	AGER	6	32148745	rs204995	32154285	G	A	0.217	-0.252	0.108	0.020	1.60E-04
IBS	AGER	6	32150423	rs693906	31835164	C	G	0.113	-0.168	0.052	0.001	0.045
UC	AGER	6	32148745	rs204995	32154285	G	A	0.217	-0.870	0.185	2.59E-06	0.002
VHD	AGER	6	32150423	rs693906	31835164	C	G	0.113	0.212	0.052	4.82E-05	0.092

Supplementary table 6. Results of the summary-based Mendelian randomization integrating whole-blood expression quantitative trait loci. For each disease-gene association, we report the strongest effect observed between GTEX and eQTLGen datasets.

A) Bonferroni-significant disease-gene association with HEIDI P > 0.05.

Disease	Gene	chr	gene_bp	topSNP	topSNP_bp	A1	A2	Freq	b_SMR	se_SMR	p_SMR	p_HEIDI
CD	DNMT3A	2	25510652	rs7578575	25488819	A	T	0.329	-0.834	0.164	3.51E-07	0.383
IBD	DNMT3A	2	25510652	rs7578575	25488819	A	T	0.329	-0.745	0.130	9.22E-09	0.515
UC	DNMT3A	2	25510652	rs7578575	25488819	A	T	0.329	-0.627	0.156	5.73E-05	0.541
UC	CFDP1	16	75327596	rs9934007	75390532	C	T	0.754	0.273	0.070	9.61E-05	0.973
CD	STAT3	17	40502964	rs1053004	40466092	G	A	0.380	0.702	0.128	4.40E-08	0.056
CD	IMPDH2	3	49064299	rs9813813	49207093	A	T	0.110	-1.233	0.294	2.74E-05	0.213
IBD	IMPDH2	3	49064299	rs9813813	49207093	A	T	0.110	-1.328	0.258	2.57E-07	0.071
UC	IMPDH2	3	49064299	rs9813813	49207093	A	T	0.110	-1.445	0.307	2.53E-06	0.050
IBD	UBE2L3	22	21941029	rs2070512	21949411	C	A	0.194	0.163	0.021	1.06E-14	0.169
UC	UBE2L3	22	21941029	rs2070512	21949411	C	A	0.194	0.116	0.027	1.76E-05	0.698
CD	BCL2L15	1	1.14E+08	rs1217393	1.14E+08	A	G	0.271	-0.705	0.170	3.39E-05	0.184
CD	STMN3	20	62271061	rs909333	62270637	G	T	0.207	0.547	0.102	8.31E-08	0.200
IBD	STMN3	20	62271061	rs909333	62270637	G	T	0.207	0.555	0.087	2.01E-10	0.055
UC	STMN3	20	62271061	rs909333	62270637	G	T	0.207	0.498	0.098	4.04E-07	0.062
GORD	PBX2	6	32154964	rs693906	31835164	C	G	0.113	0.154	0.036	1.65E-05	0.058
VHD	AGER	6	32150423	rs693906	31835164	C	G	0.113	0.212	0.052	4.82E-05	0.092

B) Nominally significant effect on VHD of Bonferroni-significant gastrointestinal loci.

Disease	Gene	chr	Probe_bp	topSNP	topSNP_bp	A1	A2	Freq	b_SMR	se_SMR	p_SMR	p_HEIDI
VHD	CFDP1	16	75397489	rs7201989	75471499	G	A	0.246	-0.056	0.018	0.001	0.119
VHD	STAT3	17	40502964	rs1053004	40466092	G	A	0.380	0.093	0.047	0.048	0.948
VHD	IMPDH2	3	49064299	rs34037363	48821036	T	C	0.114	-0.209	0.105	0.047	0.089
VHD	UBE2L3	22	21941029	rs2070512	21949411	C	A	0.194	-0.029	0.009	0.002	0.623
VHD	STMN3	20	62271061	rs909333	62270637	G	T	0.207	-0.076	0.036	0.036	0.093

C) Effect of AGER gene on VHD and gastrointestinal disorders.

Disease	Gene	chr	Probe_bp	topSNP	topSNP_bp	A1	A2	Freq	b_SMR	se_SMR	p_SMR	p_HEIDI
CD	AGER	6	32150423	rs693906	31835164	C	G	0.113	-0.312	0.120	0.009	6.16E-08
GORD	AGER	6	32150423	rs693906	31835164	C	G	0.113	-0.209	0.049	2.02E-05	0.022
IBD	AGER	6	32148745	rs204995	32154285	G	A	0.217	-0.252	0.108	0.020	1.60E-04
IBS	AGER	6	32150423	rs693906	31835164	C	G	0.113	-0.168	0.052	0.001	0.045
UC	AGER	6	32148745	rs204995	32154285	G	A	0.217	-0.870	0.185	2.59E-06	0.002
VHD	AGER	6	32150423	rs693906	31835164	C	G	0.113	0.212	0.052	4.82E-05	0.092

Supplementary table 6. Results of the summary-based Mendelian randomization integrating whole-blood expression quantitative trait loci. For each disease-gene association, we report the strongest effect observed between GTEX and eQTLGen datasets.

A) Bonferroni-significant disease-gene association with HEIDI P > 0.05.

Disease	Gene	chr	gene_bp	topSNP	topSNP_bp	A1	A2	Freq	b_SMR	se_SMR	p_SMR	p_HEIDI
CD	DNMT3A	2	25510652	rs7578575	25488819	A	T	0.329	-0.834	0.164	3.51E-07	0.383
IBD	DNMT3A	2	25510652	rs7578575	25488819	A	T	0.329	-0.745	0.130	9.22E-09	0.515
UC	DNMT3A	2	25510652	rs7578575	25488819	A	T	0.329	-0.627	0.156	5.73E-05	0.541
UC	CFDP1	16	75327596	rs9934007	75390532	C	T	0.754	0.273	0.070	9.61E-05	0.973
CD	STAT3	17	40502964	rs1053004	40466092	G	A	0.380	0.702	0.128	4.40E-08	0.056
CD	IMPDH2	3	49064299	rs9813813	49207093	A	T	0.110	-1.233	0.294	2.74E-05	0.213
IBD	IMPDH2	3	49064299	rs9813813	49207093	A	T	0.110	-1.328	0.258	2.57E-07	0.071
UC	IMPDH2	3	49064299	rs9813813	49207093	A	T	0.110	-1.445	0.307	2.53E-06	0.050
IBD	UBE2L3	22	21941029	rs2070512	21949411	C	A	0.194	0.163	0.021	1.06E-14	0.169
UC	UBE2L3	22	21941029	rs2070512	21949411	C	A	0.194	0.116	0.027	1.76E-05	0.698
CD	BCL2L15	1	1.14E+08	rs1217393	1.14E+08	A	G	0.271	-0.705	0.170	3.39E-05	0.184
CD	STMN3	20	62271061	rs909333	62270637	G	T	0.207	0.547	0.102	8.31E-08	0.200
IBD	STMN3	20	62271061	rs909333	62270637	G	T	0.207	0.555	0.087	2.01E-10	0.055
UC	STMN3	20	62271061	rs909333	62270637	G	T	0.207	0.498	0.098	4.04E-07	0.062
GORD	PBX2	6	32154964	rs693906	31835164	C	G	0.113	0.154	0.036	1.65E-05	0.058
VHD	AGER	6	32150423	rs693906	31835164	C	G	0.113	0.212	0.052	4.82E-05	0.092

B) Nominally significant effect on VHD of Bonferroni-significant gastrointestinal loci.

Disease	Gene	chr	Probe_bp	topSNP	topSNP_bp	A1	A2	Freq	b_SMR	se_SMR	p_SMR	p_HEIDI
VHD	CFDP1	16	75397489	rs7201989	75471499	G	A	0.246	-0.056	0.018	0.001	0.119
VHD	STAT3	17	40502964	rs1053004	40466092	G	A	0.380	0.093	0.047	0.048	0.948
VHD	IMPDH2	3	49064299	rs34037363	48821036	T	C	0.114	-0.209	0.105	0.047	0.089
VHD	UBE2L3	22	21941029	rs2070512	21949411	C	A	0.194	-0.029	0.009	0.002	0.623
VHD	STMN3	20	62271061	rs909333	62270637	G	T	0.207	-0.076	0.036	0.036	0.093

C) Effect of AGER gene on VHD and gastrointestinal disorders.

Disease	Gene	chr	Probe_bp	topSNP	topSNP_bp	A1	A2	Freq	b_SMR	se_SMR	p_SMR	p_HEIDI
CD	AGER	6	32150423	rs693906	31835164	C	G	0.113	-0.312	0.120	0.009	6.16E-08
GORD	AGER	6	32150423	rs693906	31835164	C	G	0.113	-0.209	0.049	2.02E-05	0.022
IBD	AGER	6	32148745	rs204995	32154285	G	A	0.217	-0.252	0.108	0.020	1.60E-04
IBS	AGER	6	32150423	rs693906	31835164	C	G	0.113	-0.168	0.052	0.001	0.045
UC	AGER	6	32148745	rs204995	32154285	G	A	0.217	-0.870	0.185	2.59E-06	0.002
VHD	AGER	6	32150423	rs693906	31835164	C	G	0.113	0.212	0.052	4.82E-05	0.092

Supplementary table 6. Results of the summary-based Mendelian randomization integrating whole-blood expression quantitative trait loci. For each disease-gene association, we report the strongest effect observed between GTEX and eQTLGen datasets.

A) Bonferroni-significant disease-gene association with HEIDI P > 0.05.

Disease	Gene	chr	gene_bp	topSNP	topSNP_bp	A1	A2	Freq	b_SMR	se_SMR	p_SMR	p_HEIDI
CD	DNMT3A	2	25510652	rs7578575	25488819	A	T	0.329	-0.834	0.164	3.51E-07	0.383
IBD	DNMT3A	2	25510652	rs7578575	25488819	A	T	0.329	-0.745	0.130	9.22E-09	0.515
UC	DNMT3A	2	25510652	rs7578575	25488819	A	T	0.329	-0.627	0.156	5.73E-05	0.541
UC	CFDP1	16	75327596	rs9934007	75390532	C	T	0.754	0.273	0.070	9.61E-05	0.973
CD	STAT3	17	40502964	rs1053004	40466092	G	A	0.380	0.702	0.128	4.40E-08	0.056
CD	IMPDH2	3	49064299	rs9813813	49207093	A	T	0.110	-1.233	0.294	2.74E-05	0.213
IBD	IMPDH2	3	49064299	rs9813813	49207093	A	T	0.110	-1.328	0.258	2.57E-07	0.071
UC	IMPDH2	3	49064299	rs9813813	49207093	A	T	0.110	-1.445	0.307	2.53E-06	0.050
IBD	UBE2L3	22	21941029	rs2070512	21949411	C	A	0.194	0.163	0.021	1.06E-14	0.169
UC	UBE2L3	22	21941029	rs2070512	21949411	C	A	0.194	0.116	0.027	1.76E-05	0.698
CD	BCL2L15	1	1.14E+08	rs1217393	1.14E+08	A	G	0.271	-0.705	0.170	3.39E-05	0.184
CD	STMN3	20	62271061	rs909333	62270637	G	T	0.207	0.547	0.102	8.31E-08	0.200
IBD	STMN3	20	62271061	rs909333	62270637	G	T	0.207	0.555	0.087	2.01E-10	0.055
UC	STMN3	20	62271061	rs909333	62270637	G	T	0.207	0.498	0.098	4.04E-07	0.062
GORD	PBX2	6	32154964	rs693906	31835164	C	G	0.113	0.154	0.036	1.65E-05	0.058
VHD	AGER	6	32150423	rs693906	31835164	C	G	0.113	0.212	0.052	4.82E-05	0.092

B) Nominally significant effect on VHD of Bonferroni-significant gastrointestinal loci.

Disease	Gene	chr	Probe_bp	topSNP	topSNP_bp	A1	A2	Freq	b_SMR	se_SMR	p_SMR	p_HEIDI
VHD	CFDP1	16	75397489	rs7201989	75471499	G	A	0.246	-0.056	0.018	0.001	0.119
VHD	STAT3	17	40502964	rs1053004	40466092	G	A	0.380	0.093	0.047	0.048	0.948
VHD	IMPDH2	3	49064299	rs34037363	48821036	T	C	0.114	-0.209	0.105	0.047	0.089
VHD	UBE2L3	22	21941029	rs2070512	21949411	C	A	0.194	-0.029	0.009	0.002	0.623
VHD	STMN3	20	62271061	rs909333	62270637	G	T	0.207	-0.076	0.036	0.036	0.093

C) Effect of AGER gene on VHD and gastrointestinal disorders.

Disease	Gene	chr	Probe_bp	topSNP	topSNP_bp	A1	A2	Freq	b_SMR	se_SMR	p_SMR	p_HEIDI
CD	AGER	6	32150423	rs693906	31835164	C	G	0.113	-0.312	0.120	0.009	6.16E-08
GORD	AGER	6	32150423	rs693906	31835164	C	G	0.113	-0.209	0.049	2.02E-05	0.022
IBD	AGER	6	32148745	rs204995	32154285	G	A	0.217	-0.252	0.108	0.020	1.60E-04
IBS	AGER	6	32150423	rs693906	31835164	C	G	0.113	-0.168	0.052	0.001	0.045
UC	AGER	6	32148745	rs204995	32154285	G	A	0.217	-0.870	0.185	2.59E-06	0.002
VHD	AGER	6	32150423	rs693906	31835164	C	G	0.113	0.212	0.052	4.82E-05	0.092

Supplementary table 6. Results of the summary-based Mendelian randomization integrating whole-blood expression quantitative trait loci. For each disease-gene association, we report the strongest effect observed between GTEX and eQTLGen datasets.

A) Bonferroni-significant disease-gene association with HEIDI P > 0.05.

Disease	Gene	chr	gene_bp	topSNP	topSNP_bp	A1	A2	Freq	b_SMR	se_SMR	p_SMR	p_HEIDI
CD	DNMT3A	2	25510652	rs7578575	25488819	A	T	0.329	-0.834	0.164	3.51E-07	0.383
IBD	DNMT3A	2	25510652	rs7578575	25488819	A	T	0.329	-0.745	0.130	9.22E-09	0.515
UC	DNMT3A	2	25510652	rs7578575	25488819	A	T	0.329	-0.627	0.156	5.73E-05	0.541
UC	CFDP1	16	75327596	rs9934007	75390532	C	T	0.754	0.273	0.070	9.61E-05	0.973
CD	STAT3	17	40502964	rs1053004	40466092	G	A	0.380	0.702	0.128	4.40E-08	0.056
CD	IMPDH2	3	49064299	rs9813813	49207093	A	T	0.110	-1.233	0.294	2.74E-05	0.213
IBD	IMPDH2	3	49064299	rs9813813	49207093	A	T	0.110	-1.328	0.258	2.57E-07	0.071
UC	IMPDH2	3	49064299	rs9813813	49207093	A	T	0.110	-1.445	0.307	2.53E-06	0.050
IBD	UBE2L3	22	21941029	rs2070512	21949411	C	A	0.194	0.163	0.021	1.06E-14	0.169
UC	UBE2L3	22	21941029	rs2070512	21949411	C	A	0.194	0.116	0.027	1.76E-05	0.698
CD	BCL2L15	1	1.14E+08	rs1217393	1.14E+08	A	G	0.271	-0.705	0.170	3.39E-05	0.184
CD	STMN3	20	62271061	rs909333	62270637	G	T	0.207	0.547	0.102	8.31E-08	0.200
IBD	STMN3	20	62271061	rs909333	62270637	G	T	0.207	0.555	0.087	2.01E-10	0.055
UC	STMN3	20	62271061	rs909333	62270637	G	T	0.207	0.498	0.098	4.04E-07	0.062
GORD	PBX2	6	32154964	rs693906	31835164	C	G	0.113	0.154	0.036	1.65E-05	0.058
VHD	AGER	6	32150423	rs693906	31835164	C	G	0.113	0.212	0.052	4.82E-05	0.092

B) Nominally significant effect on VHD of Bonferroni-significant gastrointestinal loci.

Disease	Gene	chr	Probe_bp	topSNP	topSNP_bp	A1	A2	Freq	b_SMR	se_SMR	p_SMR	p_HEIDI
VHD	CFDP1	16	75397489	rs7201989	75471499	G	A	0.246	-0.056	0.018	0.001	0.119
VHD	STAT3	17	40502964	rs1053004	40466092	G	A	0.380	0.093	0.047	0.048	0.948
VHD	IMPDH2	3	49064299	rs34037363	48821036	T	C	0.114	-0.209	0.105	0.047	0.089
VHD	UBE2L3	22	21941029	rs2070512	21949411	C	A	0.194	-0.029	0.009	0.002	0.623
VHD	STMN3	20	62271061	rs909333	62270637	G	T	0.207	-0.076	0.036	0.036	0.093

C) Effect of AGER gene on VHD and gastrointestinal disorders.

Disease	Gene	chr	Probe_bp	topSNP	topSNP_bp	A1	A2	Freq	b_SMR	se_SMR	p_SMR	p_HEIDI
CD	AGER	6	32150423	rs693906	31835164	C	G	0.113	-0.312	0.120	0.009	6.16E-08
GORD	AGER	6	32150423	rs693906	31835164	C	G	0.113	-0.209	0.049	2.02E-05	0.022
IBD	AGER	6	32148745	rs204995	32154285	G	A	0.217	-0.252	0.108	0.020	1.60E-04
IBS	AGER	6	32150423	rs693906	31835164	C	G	0.113	-0.168	0.052	0.001	0.045
UC	AGER	6	32148745	rs204995	32154285	G	A	0.217	-0.870	0.185	2.59E-06	0.002
VHD	AGER	6	32150423	rs693906	31835164	C	G	0.113	0.212	0.052	4.82E-05	0.092

Supplementary table 6. Results of the summary-based Mendelian randomization integrating whole-blood expression quantitative trait loci. For each disease-gene association, we report the strongest effect observed between GTEX and eQTLGen datasets.

A) Bonferroni-significant disease-gene association with HEIDI P > 0.05.

Disease	Gene	chr	gene_bp	topSNP	topSNP_bp	A1	A2	Freq	b_SMR	se_SMR	p_SMR	p_HEIDI
CD	DNMT3A	2	25510652	rs7578575	25488819	A	T	0.329	-0.834	0.164	3.51E-07	0.383
IBD	DNMT3A	2	25510652	rs7578575	25488819	A	T	0.329	-0.745	0.130	9.22E-09	0.515
UC	DNMT3A	2	25510652	rs7578575	25488819	A	T	0.329	-0.627	0.156	5.73E-05	0.541
UC	CFDP1	16	75327596	rs9934007	75390532	C	T	0.754	0.273	0.070	9.61E-05	0.973
CD	STAT3	17	40502964	rs1053004	40466092	G	A	0.380	0.702	0.128	4.40E-08	0.056
CD	IMPDH2	3	49064299	rs9813813	49207093	A	T	0.110	-1.233	0.294	2.74E-05	0.213
IBD	IMPDH2	3	49064299	rs9813813	49207093	A	T	0.110	-1.328	0.258	2.57E-07	0.071
UC	IMPDH2	3	49064299	rs9813813	49207093	A	T	0.110	-1.445	0.307	2.53E-06	0.050
IBD	UBE2L3	22	21941029	rs2070512	21949411	C	A	0.194	0.163	0.021	1.06E-14	0.169
UC	UBE2L3	22	21941029	rs2070512	21949411	C	A	0.194	0.116	0.027	1.76E-05	0.698
CD	BCL2L15	1	1.14E+08	rs1217393	1.14E+08	A	G	0.271	-0.705	0.170	3.39E-05	0.184
CD	STMN3	20	62271061	rs909333	62270637	G	T	0.207	0.547	0.102	8.31E-08	0.200
IBD	STMN3	20	62271061	rs909333	62270637	G	T	0.207	0.555	0.087	2.01E-10	0.055
UC	STMN3	20	62271061	rs909333	62270637	G	T	0.207	0.498	0.098	4.04E-07	0.062
GORD	PBX2	6	32154964	rs693906	31835164	C	G	0.113	0.154	0.036	1.65E-05	0.058
VHD	AGER	6	32150423	rs693906	31835164	C	G	0.113	0.212	0.052	4.82E-05	0.092

B) Nominally significant effect on VHD of Bonferroni-significant gastrointestinal loci.

Disease	Gene	chr	Probe_bp	topSNP	topSNP_bp	A1	A2	Freq	b_SMR	se_SMR	p_SMR	p_HEIDI
VHD	CFDP1	16	75397489	rs7201989	75471499	G	A	0.246	-0.056	0.018	0.001	0.119
VHD	STAT3	17	40502964	rs1053004	40466092	G	A	0.380	0.093	0.047	0.048	0.948
VHD	IMPDH2	3	49064299	rs34037363	48821036	T	C	0.114	-0.209	0.105	0.047	0.089
VHD	UBE2L3	22	21941029	rs2070512	21949411	C	A	0.194	-0.029	0.009	0.002	0.623
VHD	STMN3	20	62271061	rs909333	62270637	G	T	0.207	-0.076	0.036	0.036	0.093

C) Effect of AGER gene on VHD and gastrointestinal disorders.

Disease	Gene	chr	Probe_bp	topSNP	topSNP_bp	A1	A2	Freq	b_SMR	se_SMR	p_SMR	p_HEIDI
CD	AGER	6	32150423	rs693906	31835164	C	G	0.113	-0.312	0.120	0.009	6.16E-08
GORD	AGER	6	32150423	rs693906	31835164	C	G	0.113	-0.209	0.049	2.02E-05	0.022
IBD	AGER	6	32148745	rs204995	32154285	G	A	0.217	-0.252	0.108	0.020	1.60E-04
IBS	AGER	6	32150423	rs693906	31835164	C	G	0.113	-0.168	0.052	0.001	0.045
UC	AGER	6	32148745	rs204995	32154285	G	A	0.217	-0.870	0.185	2.59E-06	0.002
VHD	AGER	6	32150423	rs693906	31835164	C	G	0.113	0.212	0.052	4.82E-05	0.092

Supplementary table 6. Results of the summary-based Mendelian randomization integrating whole-blood expression quantitative trait loci. For each disease-gene association, we report the strongest effect observed between GTEX and eQTLGen datasets.

A) Bonferroni-significant disease-gene association with HEIDI P > 0.05.

Disease	Gene	chr	gene_bp	topSNP	topSNP_bp	A1	A2	Freq	b_SMR	se_SMR	p_SMR	p_HEIDI
CD	DNMT3A	2	25510652	rs7578575	25488819	A	T	0.329	-0.834	0.164	3.51E-07	0.383
IBD	DNMT3A	2	25510652	rs7578575	25488819	A	T	0.329	-0.745	0.130	9.22E-09	0.515
UC	DNMT3A	2	25510652	rs7578575	25488819	A	T	0.329	-0.627	0.156	5.73E-05	0.541
UC	CFDP1	16	75327596	rs9934007	75390532	C	T	0.754	0.273	0.070	9.61E-05	0.973
CD	STAT3	17	40502964	rs1053004	40466092	G	A	0.380	0.702	0.128	4.40E-08	0.056
CD	IMPDH2	3	49064299	rs9813813	49207093	A	T	0.110	-1.233	0.294	2.74E-05	0.213
IBD	IMPDH2	3	49064299	rs9813813	49207093	A	T	0.110	-1.328	0.258	2.57E-07	0.071
UC	IMPDH2	3	49064299	rs9813813	49207093	A	T	0.110	-1.445	0.307	2.53E-06	0.050
IBD	UBE2L3	22	21941029	rs2070512	21949411	C	A	0.194	0.163	0.021	1.06E-14	0.169
UC	UBE2L3	22	21941029	rs2070512	21949411	C	A	0.194	0.116	0.027	1.76E-05	0.698
CD	BCL2L15	1	1.14E+08	rs1217393	1.14E+08	A	G	0.271	-0.705	0.170	3.39E-05	0.184
CD	STMN3	20	62271061	rs909333	62270637	G	T	0.207	0.547	0.102	8.31E-08	0.200
IBD	STMN3	20	62271061	rs909333	62270637	G	T	0.207	0.555	0.087	2.01E-10	0.055
UC	STMN3	20	62271061	rs909333	62270637	G	T	0.207	0.498	0.098	4.04E-07	0.062
GORD	PBX2	6	32154964	rs693906	31835164	C	G	0.113	0.154	0.036	1.65E-05	0.058
VHD	AGER	6	32150423	rs693906	31835164	C	G	0.113	0.212	0.052	4.82E-05	0.092

B) Nominally significant effect on VHD of Bonferroni-significant gastrointestinal loci.

Disease	Gene	chr	Probe_bp	topSNP	topSNP_bp	A1	A2	Freq	b_SMR	se_SMR	p_SMR	p_HEIDI
VHD	CFDP1	16	75397489	rs7201989	75471499	G	A	0.246	-0.056	0.018	0.001	0.119
VHD	STAT3	17	40502964	rs1053004	40466092	G	A	0.380	0.093	0.047	0.048	0.948
VHD	IMPDH2	3	49064299	rs34037363	48821036	T	C	0.114	-0.209	0.105	0.047	0.089
VHD	UBE2L3	22	21941029	rs2070512	21949411	C	A	0.194	-0.029	0.009	0.002	0.623
VHD	STMN3	20	62271061	rs909333	62270637	G	T	0.207	-0.076	0.036	0.036	0.093

C) Effect of AGER gene on VHD and gastrointestinal disorders.

Disease	Gene	chr	Probe_bp	topSNP	topSNP_bp	A1	A2	Freq	b_SMR	se_SMR	p_SMR	p_HEIDI
CD	AGER	6	32150423	rs693906	31835164	C	G	0.113	-0.312	0.120	0.009	6.16E-08
GORD	AGER	6	32150423	rs693906	31835164	C	G	0.113	-0.209	0.049	2.02E-05	0.022
IBD	AGER	6	32148745	rs204995	32154285	G	A	0.217	-0.252	0.108	0.020	1.60E-04
IBS	AGER	6	32150423	rs693906	31835164	C	G	0.113	-0.168	0.052	0.001	0.045
UC	AGER	6	32148745	rs204995	32154285	G	A	0.217	-0.870	0.185	2.59E-06	0.002
VHD	AGER	6	32150423	rs693906	31835164	C	G	0.113	0.212	0.052	4.82E-05	0.092

Supplementary table 6. Results of the summary-based Mendelian randomization integrating whole-blood expression quantitative trait loci. For each disease-gene association, we report the strongest effect observed between GTEX and eQTLGen datasets.

A) Bonferroni-significant disease-gene association with HEIDI P > 0.05.

Disease	Gene	chr	gene_bp	topSNP	topSNP_bp	A1	A2	Freq	b_SMR	se_SMR	p_SMR	p_HEIDI
CD	DNMT3A	2	25510652	rs7578575	25488819	A	T	0.329	-0.834	0.164	3.51E-07	0.383
IBD	DNMT3A	2	25510652	rs7578575	25488819	A	T	0.329	-0.745	0.130	9.22E-09	0.515
UC	DNMT3A	2	25510652	rs7578575	25488819	A	T	0.329	-0.627	0.156	5.73E-05	0.541
UC	CFDP1	16	75327596	rs9934007	75390532	C	T	0.754	0.273	0.070	9.61E-05	0.973
CD	STAT3	17	40502964	rs1053004	40466092	G	A	0.380	0.702	0.128	4.40E-08	0.056
CD	IMPDH2	3	49064299	rs9813813	49207093	A	T	0.110	-1.233	0.294	2.74E-05	0.213
IBD	IMPDH2	3	49064299	rs9813813	49207093	A	T	0.110	-1.328	0.258	2.57E-07	0.071
UC	IMPDH2	3	49064299	rs9813813	49207093	A	T	0.110	-1.445	0.307	2.53E-06	0.050
IBD	UBE2L3	22	21941029	rs2070512	21949411	C	A	0.194	0.163	0.021	1.06E-14	0.169
UC	UBE2L3	22	21941029	rs2070512	21949411	C	A	0.194	0.116	0.027	1.76E-05	0.698
CD	BCL2L15	1	1.14E+08	rs1217393	1.14E+08	A	G	0.271	-0.705	0.170	3.39E-05	0.184
CD	STMN3	20	62271061	rs909333	62270637	G	T	0.207	0.547	0.102	8.31E-08	0.200
IBD	STMN3	20	62271061	rs909333	62270637	G	T	0.207	0.555	0.087	2.01E-10	0.055
UC	STMN3	20	62271061	rs909333	62270637	G	T	0.207	0.498	0.098	4.04E-07	0.062
GORD	PBX2	6	32154964	rs693906	31835164	C	G	0.113	0.154	0.036	1.65E-05	0.058
VHD	AGER	6	32150423	rs693906	31835164	C	G	0.113	0.212	0.052	4.82E-05	0.092

B) Nominally significant effect on VHD of Bonferroni-significant gastrointestinal loci.

Disease	Gene	chr	Probe_bp	topSNP	topSNP_bp	A1	A2	Freq	b_SMR	se_SMR	p_SMR	p_HEIDI
VHD	CFDP1	16	75397489	rs7201989	75471499	G	A	0.246	-0.056	0.018	0.001	0.119
VHD	STAT3	17	40502964	rs1053004	40466092	G	A	0.380	0.093	0.047	0.048	0.948
VHD	IMPDH2	3	49064299	rs34037363	48821036	T	C	0.114	-0.209	0.105	0.047	0.089
VHD	UBE2L3	22	21941029	rs2070512	21949411	C	A	0.194	-0.029	0.009	0.002	0.623
VHD	STMN3	20	62271061	rs909333	62270637	G	T	0.207	-0.076	0.036	0.036	0.093

C) Effect of AGER gene on VHD and gastrointestinal disorders.

Disease	Gene	chr	Probe_bp	topSNP	topSNP_bp	A1	A2	Freq	b_SMR	se_SMR	p_SMR	p_HEIDI
CD	AGER	6	32150423	rs693906	31835164	C	G	0.113	-0.312	0.120	0.009	6.16E-08
GORD	AGER	6	32150423	rs693906	31835164	C	G	0.113	-0.209	0.049	2.02E-05	0.022
IBD	AGER	6	32148745	rs204995	32154285	G	A	0.217	-0.252	0.108	0.020	1.60E-04
IBS	AGER	6	32150423	rs693906	31835164	C	G	0.113	-0.168	0.052	0.001	0.045
UC	AGER	6	32148745	rs204995	32154285	G	A	0.217	-0.870	0.185	2.59E-06	0.002
VHD	AGER	6	32150423	rs693906	31835164	C	G	0.113	0.212	0.052	4.82E-05	0.092

Supplementary table 6. Results of the summary-based Mendelian randomization integrating whole-blood expression quantitative trait loci. For each disease-gene association, we report the strongest effect observed between GTEX and eQTLGen datasets.

A) Bonferroni-significant disease-gene association with HEIDI P > 0.05.

Disease	Gene	chr	gene_bp	topSNP	topSNP_bp	A1	A2	Freq	b_SMR	se_SMR	p_SMR	p_HEIDI
CD	DNMT3A	2	25510652	rs7578575	25488819	A	T	0.329	-0.834	0.164	3.51E-07	0.383
IBD	DNMT3A	2	25510652	rs7578575	25488819	A	T	0.329	-0.745	0.130	9.22E-09	0.515
UC	DNMT3A	2	25510652	rs7578575	25488819	A	T	0.329	-0.627	0.156	5.73E-05	0.541
UC	CFDP1	16	75327596	rs9934007	75390532	C	T	0.754	0.273	0.070	9.61E-05	0.973
CD	STAT3	17	40502964	rs1053004	40466092	G	A	0.380	0.702	0.128	4.40E-08	0.056
CD	IMPDH2	3	49064299	rs9813813	49207093	A	T	0.110	-1.233	0.294	2.74E-05	0.213
IBD	IMPDH2	3	49064299	rs9813813	49207093	A	T	0.110	-1.328	0.258	2.57E-07	0.071
UC	IMPDH2	3	49064299	rs9813813	49207093	A	T	0.110	-1.445	0.307	2.53E-06	0.050
IBD	UBE2L3	22	21941029	rs2070512	21949411	C	A	0.194	0.163	0.021	1.06E-14	0.169
UC	UBE2L3	22	21941029	rs2070512	21949411	C	A	0.194	0.116	0.027	1.76E-05	0.698
CD	BCL2L15	1	1.14E+08	rs1217393	1.14E+08	A	G	0.271	-0.705	0.170	3.39E-05	0.184
CD	STMN3	20	62271061	rs909333	62270637	G	T	0.207	0.547	0.102	8.31E-08	0.200
IBD	STMN3	20	62271061	rs909333	62270637	G	T	0.207	0.555	0.087	2.01E-10	0.055
UC	STMN3	20	62271061	rs909333	62270637	G	T	0.207	0.498	0.098	4.04E-07	0.062
GORD	PBX2	6	32154964	rs693906	31835164	C	G	0.113	0.154	0.036	1.65E-05	0.058
VHD	AGER	6	32150423	rs693906	31835164	C	G	0.113	0.212	0.052	4.82E-05	0.092

B) Nominally significant effect on VHD of Bonferroni-significant gastrointestinal loci.

Disease	Gene	chr	Probe_bp	topSNP	topSNP_bp	A1	A2	Freq	b_SMR	se_SMR	p_SMR	p_HEIDI
VHD	CFDP1	16	75397489	rs7201989	75471499	G	A	0.246	-0.056	0.018	0.001	0.119
VHD	STAT3	17	40502964	rs1053004	40466092	G	A	0.380	0.093	0.047	0.048	0.948
VHD	IMPDH2	3	49064299	rs34037363	48821036	T	C	0.114	-0.209	0.105	0.047	0.089
VHD	UBE2L3	22	21941029	rs2070512	21949411	C	A	0.194	-0.029	0.009	0.002	0.623
VHD	STMN3	20	62271061	rs909333	62270637	G	T	0.207	-0.076	0.036	0.036	0.093

C) Effect of AGER gene on VHD and gastrointestinal disorders.

Disease	Gene	chr	Probe_bp	topSNP	topSNP_bp	A1	A2	Freq	b_SMR	se_SMR	p_SMR	p_HEIDI
CD	AGER	6	32150423	rs693906	31835164	C	G	0.113	-0.312	0.120	0.009	6.16E-08
GORD	AGER	6	32150423	rs693906	31835164	C	G	0.113	-0.209	0.049	2.02E-05	0.022
IBD	AGER	6	32148745	rs204995	32154285	G	A	0.217	-0.252	0.108	0.020	1.60E-04
IBS	AGER	6	32150423	rs693906	31835164	C	G	0.113	-0.168	0.052	0.001	0.045
UC	AGER	6	32148745	rs204995	32154285	G	A	0.217	-0.870	0.185	2.59E-06	0.002
VHD	AGER	6	32150423	rs693906	31835164	C	G	0.113	0.212	0.052	4.82E-05	0.092

Supplementary table 6. Results of the summary-based Mendelian randomization integrating whole-blood expression quantitative trait loci. For each disease-gene association, we report the strongest effect observed between GTEX and eQTLGen datasets.

A) Bonferroni-significant disease-gene association with HEIDI P > 0.05.

Disease	Gene	chr	gene_bp	topSNP	topSNP_bp	A1	A2	Freq	b_SMR	se_SMR	p_SMR	p_HEIDI
CD	DNMT3A	2	25510652	rs7578575	25488819	A	T	0.329	-0.834	0.164	3.51E-07	0.383
IBD	DNMT3A	2	25510652	rs7578575	25488819	A	T	0.329	-0.745	0.130	9.22E-09	0.515
UC	DNMT3A	2	25510652	rs7578575	25488819	A	T	0.329	-0.627	0.156	5.73E-05	0.541
UC	CFDP1	16	75327596	rs9934007	75390532	C	T	0.754	0.273	0.070	9.61E-05	0.973
CD	STAT3	17	40502964	rs1053004	40466092	G	A	0.380	0.702	0.128	4.40E-08	0.056
CD	IMPDH2	3	49064299	rs9813813	49207093	A	T	0.110	-1.233	0.294	2.74E-05	0.213
IBD	IMPDH2	3	49064299	rs9813813	49207093	A	T	0.110	-1.328	0.258	2.57E-07	0.071
UC	IMPDH2	3	49064299	rs9813813	49207093	A	T	0.110	-1.445	0.307	2.53E-06	0.050
IBD	UBE2L3	22	21941029	rs2070512	21949411	C	A	0.194	0.163	0.021	1.06E-14	0.169
UC	UBE2L3	22	21941029	rs2070512	21949411	C	A	0.194	0.116	0.027	1.76E-05	0.698
CD	BCL2L15	1	1.14E+08	rs1217393	1.14E+08	A	G	0.271	-0.705	0.170	3.39E-05	0.184
CD	STMN3	20	62271061	rs909333	62270637	G	T	0.207	0.547	0.102	8.31E-08	0.200
IBD	STMN3	20	62271061	rs909333	62270637	G	T	0.207	0.555	0.087	2.01E-10	0.055
UC	STMN3	20	62271061	rs909333	62270637	G	T	0.207	0.498	0.098	4.04E-07	0.062
GORD	PBX2	6	32154964	rs693906	31835164	C	G	0.113	0.154	0.036	1.65E-05	0.058
VHD	AGER	6	32150423	rs693906	31835164	C	G	0.113	0.212	0.052	4.82E-05	0.092

B) Nominally significant effect on VHD of Bonferroni-significant gastrointestinal loci.

Disease	Gene	chr	Probe_bp	topSNP	topSNP_bp	A1	A2	Freq	b_SMR	se_SMR	p_SMR	p_HEIDI
VHD	CFDP1	16	75397489	rs7201989	75471499	G	A	0.246	-0.056	0.018	0.001	0.119
VHD	STAT3	17	40502964	rs1053004	40466092	G	A	0.380	0.093	0.047	0.048	0.948
VHD	IMPDH2	3	49064299	rs34037363	48821036	T	C	0.114	-0.209	0.105	0.047	0.089
VHD	UBE2L3	22	21941029	rs2070512	21949411	C	A	0.194	-0.029	0.009	0.002	0.623
VHD	STMN3	20	62271061	rs909333	62270637	G	T	0.207	-0.076	0.036	0.036	0.093

C) Effect of AGER gene on VHD and gastrointestinal disorders.

Disease	Gene	chr	Probe_bp	topSNP	topSNP_bp	A1	A2	Freq	b_SMR	se_SMR	p_SMR	p_HEIDI
CD	AGER	6	32150423	rs693906	31835164	C	G	0.113	-0.312	0.120	0.009	6.16E-08
GORD	AGER	6	32150423	rs693906	31835164	C	G	0.113	-0.209	0.049	2.02E-05	0.022
IBD	AGER	6	32148745	rs204995	32154285	G	A	0.217	-0.252	0.108	0.020	1.60E-04
IBS	AGER	6	32150423	rs693906	31835164	C	G	0.113	-0.168	0.052	0.001	0.045
UC	AGER	6	32148745	rs204995	32154285	G	A	0.217	-0.870	0.185	2.59E-06	0.002
VHD	AGER	6	32150423	rs693906	31835164	C	G	0.113	0.212	0.052	4.82E-05	0.092

Supplementary table 6. Results of the summary-based Mendelian randomization integrating whole-blood expression quantitative trait loci. For each disease-gene association, we report the strongest effect observed between GTEX and eQTLGen datasets.

A) Bonferroni-significant disease-gene association with HEIDI P > 0.05.

Disease	Gene	chr	gene_bp	topSNP	topSNP_bp	A1	A2	Freq	b_SMR	se_SMR	p_SMR	p_HEIDI
CD	DNMT3A	2	25510652	rs7578575	25488819	A	T	0.329	-0.834	0.164	3.51E-07	0.383
IBD	DNMT3A	2	25510652	rs7578575	25488819	A	T	0.329	-0.745	0.130	9.22E-09	0.515
UC	DNMT3A	2	25510652	rs7578575	25488819	A	T	0.329	-0.627	0.156	5.73E-05	0.541
UC	CFDP1	16	75327596	rs9934007	75390532	C	T	0.754	0.273	0.070	9.61E-05	0.973
CD	STAT3	17	40502964	rs1053004	40466092	G	A	0.380	0.702	0.128	4.40E-08	0.056
CD	IMPDH2	3	49064299	rs9813813	49207093	A	T	0.110	-1.233	0.294	2.74E-05	0.213
IBD	IMPDH2	3	49064299	rs9813813	49207093	A	T	0.110	-1.328	0.258	2.57E-07	0.071
UC	IMPDH2	3	49064299	rs9813813	49207093	A	T	0.110	-1.445	0.307	2.53E-06	0.050
IBD	UBE2L3	22	21941029	rs2070512	21949411	C	A	0.194	0.163	0.021	1.06E-14	0.169
UC	UBE2L3	22	21941029	rs2070512	21949411	C	A	0.194	0.116	0.027	1.76E-05	0.698
CD	BCL2L15	1	1.14E+08	rs1217393	1.14E+08	A	G	0.271	-0.705	0.170	3.39E-05	0.184
CD	STMN3	20	62271061	rs909333	62270637	G	T	0.207	0.547	0.102	8.31E-08	0.200
IBD	STMN3	20	62271061	rs909333	62270637	G	T	0.207	0.555	0.087	2.01E-10	0.055
UC	STMN3	20	62271061	rs909333	62270637	G	T	0.207	0.498	0.098	4.04E-07	0.062
GORD	PBX2	6	32154964	rs693906	31835164	C	G	0.113	0.154	0.036	1.65E-05	0.058
VHD	AGER	6	32150423	rs693906	31835164	C	G	0.113	0.212	0.052	4.82E-05	0.092

B) Nominally significant effect on VHD of Bonferroni-significant gastrointestinal loci.

Disease	Gene	chr	Probe_bp	topSNP	topSNP_bp	A1	A2	Freq	b_SMR	se_SMR	p_SMR	p_HEIDI
VHD	CFDP1	16	75397489	rs7201989	75471499	G	A	0.246	-0.056	0.018	0.001	0.119
VHD	STAT3	17	40502964	rs1053004	40466092	G	A	0.380	0.093	0.047	0.048	0.948
VHD	IMPDH2	3	49064299	rs34037363	48821036	T	C	0.114	-0.209	0.105	0.047	0.089
VHD	UBE2L3	22	21941029	rs2070512	21949411	C	A	0.194	-0.029	0.009	0.002	0.623
VHD	STMN3	20	62271061	rs909333	62270637	G	T	0.207	-0.076	0.036	0.036	0.093

C) Effect of AGER gene on VHD and gastrointestinal disorders.

Disease	Gene	chr	Probe_bp	topSNP	topSNP_bp	A1	A2	Freq	b_SMR	se_SMR	p_SMR	p_HEIDI
CD	AGER	6	32150423	rs693906	31835164	C	G	0.113	-0.312	0.120	0.009	6.16E-08
GORD	AGER	6	32150423	rs693906	31835164	C	G	0.113	-0.209	0.049	2.02E-05	0.022
IBD	AGER	6	32148745	rs204995	32154285	G	A	0.217	-0.252	0.108	0.020	1.60E-04
IBS	AGER	6	32150423	rs693906	31835164	C	G	0.113	-0.168	0.052	0.001	0.045
UC	AGER	6	32148745	rs204995	32154285	G	A	0.217	-0.870	0.185	2.59E-06	0.002
VHD	AGER	6	32150423	rs693906	31835164	C	G	0.113	0.212	0.052	4.82E-05	0.092

Supplementary table 6. Results of the summary-based Mendelian randomization integrating whole-blood expression quantitative trait loci. For each disease-gene association, we report the strongest effect observed between GTEX and eQTLGen datasets.

A) Bonferroni-significant disease-gene association with HEIDI P > 0.05.

Disease	Gene	chr	gene_bp	topSNP	topSNP_bp	A1	A2	Freq	b_SMR	se_SMR	p_SMR	p_HEIDI
CD	DNMT3A	2	25510652	rs7578575	25488819	A	T	0.329	-0.834	0.164	3.51E-07	0.383
IBD	DNMT3A	2	25510652	rs7578575	25488819	A	T	0.329	-0.745	0.130	9.22E-09	0.515
UC	DNMT3A	2	25510652	rs7578575	25488819	A	T	0.329	-0.627	0.156	5.73E-05	0.541
UC	CFDP1	16	75327596	rs9934007	75390532	C	T	0.754	0.273	0.070	9.61E-05	0.973
CD	STAT3	17	40502964	rs1053004	40466092	G	A	0.380	0.702	0.128	4.40E-08	0.056
CD	IMPDH2	3	49064299	rs9813813	49207093	A	T	0.110	-1.233	0.294	2.74E-05	0.213
IBD	IMPDH2	3	49064299	rs9813813	49207093	A	T	0.110	-1.328	0.258	2.57E-07	0.071
UC	IMPDH2	3	49064299	rs9813813	49207093	A	T	0.110	-1.445	0.307	2.53E-06	0.050
IBD	UBE2L3	22	21941029	rs2070512	21949411	C	A	0.194	0.163	0.021	1.06E-14	0.169
UC	UBE2L3	22	21941029	rs2070512	21949411	C	A	0.194	0.116	0.027	1.76E-05	0.698
CD	BCL2L15	1	1.14E+08	rs1217393	1.14E+08	A	G	0.271	-0.705	0.170	3.39E-05	0.184
CD	STMN3	20	62271061	rs909333	62270637	G	T	0.207	0.547	0.102	8.31E-08	0.200
IBD	STMN3	20	62271061	rs909333	62270637	G	T	0.207	0.555	0.087	2.01E-10	0.055
UC	STMN3	20	62271061	rs909333	62270637	G	T	0.207	0.498	0.098	4.04E-07	0.062
GORD	PBX2	6	32154964	rs693906	31835164	C	G	0.113	0.154	0.036	1.65E-05	0.058
VHD	AGER	6	32150423	rs693906	31835164	C	G	0.113	0.212	0.052	4.82E-05	0.092

B) Nominally significant effect on VHD of Bonferroni-significant gastrointestinal loci.

Disease	Gene	chr	Probe_bp	topSNP	topSNP_bp	A1	A2	Freq	b_SMR	se_SMR	p_SMR	p_HEIDI
VHD	CFDP1	16	75397489	rs7201989	75471499	G	A	0.246	-0.056	0.018	0.001	0.119
VHD	STAT3	17	40502964	rs1053004	40466092	G	A	0.380	0.093	0.047	0.048	0.948
VHD	IMPDH2	3	49064299	rs34037363	48821036	T	C	0.114	-0.209	0.105	0.047	0.089
VHD	UBE2L3	22	21941029	rs2070512	21949411	C	A	0.194	-0.029	0.009	0.002	0.623
VHD	STMN3	20	62271061	rs909333	62270637	G	T	0.207	-0.076	0.036	0.036	0.093

C) Effect of AGER gene on VHD and gastrointestinal disorders.

Disease	Gene	chr	Probe_bp	topSNP	topSNP_bp	A1	A2	Freq	b_SMR	se_SMR	p_SMR	p_HEIDI
CD	AGER	6	32150423	rs693906	31835164	C	G	0.113	-0.312	0.120	0.009	6.16E-08
GORD	AGER	6	32150423	rs693906	31835164	C	G	0.113	-0.209	0.049	2.02E-05	0.022
IBD	AGER	6	32148745	rs204995	32154285	G	A	0.217	-0.252	0.108	0.020	1.60E-04
IBS	AGER	6	32150423	rs693906	31835164	C	G	0.113	-0.168	0.052	0.001	0.045
UC	AGER	6	32148745	rs204995	32154285	G	A	0.217	-0.870	0.185	2.59E-06	0.002
VHD	AGER	6	32150423	rs693906	31835164	C	G	0.113	0.212	0.052	4.82E-05	0.092

Supplementary table 6. Results of the summary-based Mendelian randomization integrating whole-blood expression quantitative trait loci. For each disease-gene association, we report the strongest effect observed between GTEX and eQTLGen datasets.

A) Bonferroni-significant disease-gene association with HEIDI P > 0.05.

Disease	Gene	chr	gene_bp	topSNP	topSNP_bp	A1	A2	Freq	b_SMR	se_SMR	p_SMR	p_HEIDI
CD	DNMT3A	2	25510652	rs7578575	25488819	A	T	0.329	-0.834	0.164	3.51E-07	0.383
IBD	DNMT3A	2	25510652	rs7578575	25488819	A	T	0.329	-0.745	0.130	9.22E-09	0.515
UC	DNMT3A	2	25510652	rs7578575	25488819	A	T	0.329	-0.627	0.156	5.73E-05	0.541
UC	CFDP1	16	75327596	rs9934007	75390532	C	T	0.754	0.273	0.070	9.61E-05	0.973
CD	STAT3	17	40502964	rs1053004	40466092	G	A	0.380	0.702	0.128	4.40E-08	0.056
CD	IMPDH2	3	49064299	rs9813813	49207093	A	T	0.110	-1.233	0.294	2.74E-05	0.213
IBD	IMPDH2	3	49064299	rs9813813	49207093	A	T	0.110	-1.328	0.258	2.57E-07	0.071
UC	IMPDH2	3	49064299	rs9813813	49207093	A	T	0.110	-1.445	0.307	2.53E-06	0.050
IBD	UBE2L3	22	21941029	rs2070512	21949411	C	A	0.194	0.163	0.021	1.06E-14	0.169
UC	UBE2L3	22	21941029	rs2070512	21949411	C	A	0.194	0.116	0.027	1.76E-05	0.698
CD	BCL2L15	1	1.14E+08	rs1217393	1.14E+08	A	G	0.271	-0.705	0.170	3.39E-05	0.184
CD	STMN3	20	62271061	rs909333	62270637	G	T	0.207	0.547	0.102	8.31E-08	0.200
IBD	STMN3	20	62271061	rs909333	62270637	G	T	0.207	0.555	0.087	2.01E-10	0.055
UC	STMN3	20	62271061	rs909333	62270637	G	T	0.207	0.498	0.098	4.04E-07	0.062
GORD	PBX2	6	32154964	rs693906	31835164	C	G	0.113	0.154	0.036	1.65E-05	0.058
VHD	AGER	6	32150423	rs693906	31835164	C	G	0.113	0.212	0.052	4.82E-05	0.092

B) Nominally significant effect on VHD of Bonferroni-significant gastrointestinal loci.

Disease	Gene	chr	Probe_bp	topSNP	topSNP_bp	A1	A2	Freq	b_SMR	se_SMR	p_SMR	p_HEIDI
VHD	CFDP1	16	75397489	rs7201989	75471499	G	A	0.246	-0.056	0.018	0.001	0.119
VHD	STAT3	17	40502964	rs1053004	40466092	G	A	0.380	0.093	0.047	0.048	0.948
VHD	IMPDH2	3	49064299	rs34037363	48821036	T	C	0.114	-0.209	0.105	0.047	0.089
VHD	UBE2L3	22	21941029	rs2070512	21949411	C	A	0.194	-0.029	0.009	0.002	0.623
VHD	STMN3	20	62271061	rs909333	62270637	G	T	0.207	-0.076	0.036	0.036	0.093

C) Effect of AGER gene on VHD and gastrointestinal disorders.

Disease	Gene	chr	Probe_bp	topSNP	topSNP_bp	A1	A2	Freq	b_SMR	se_SMR	p_SMR	p_HEIDI
CD	AGER	6	32150423	rs693906	31835164	C	G	0.113	-0.312	0.120	0.009	6.16E-08
GORD	AGER	6	32150423	rs693906	31835164	C	G	0.113	-0.209	0.049	2.02E-05	0.022
IBD	AGER	6	32148745	rs204995	32154285	G	A	0.217	-0.252	0.108	0.020	1.60E-04
IBS	AGER	6	32150423	rs693906	31835164	C	G	0.113	-0.168	0.052	0.001	0.045
UC	AGER	6	32148745	rs204995	32154285	G	A	0.217	-0.870	0.185	2.59E-06	0.002
VHD	AGER	6	32150423	rs693906	31835164	C	G	0.113	0.212	0.052	4.82E-05	0.092

Supplementary table 6. Results of the summary-based Mendelian randomization integrating whole-blood expression quantitative trait loci. For each disease-gene association, we report the strongest effect observed between GTEX and eQTLGen datasets.

A) Bonferroni-significant disease-gene association with HEIDI P > 0.05.

Disease	Gene	chr	gene_bp	topSNP	topSNP_bp	A1	A2	Freq	b_SMR	se_SMR	p_SMR	p_HEIDI
CD	DNMT3A	2	25510652	rs7578575	25488819	A	T	0.329	-0.834	0.164	3.51E-07	0.383
IBD	DNMT3A	2	25510652	rs7578575	25488819	A	T	0.329	-0.745	0.130	9.22E-09	0.515
UC	DNMT3A	2	25510652	rs7578575	25488819	A	T	0.329	-0.627	0.156	5.73E-05	0.541
UC	CFDP1	16	75327596	rs9934007	75390532	C	T	0.754	0.273	0.070	9.61E-05	0.973
CD	STAT3	17	40502964	rs1053004	40466092	G	A	0.380	0.702	0.128	4.40E-08	0.056
CD	IMPDH2	3	49064299	rs9813813	49207093	A	T	0.110	-1.233	0.294	2.74E-05	0.213
IBD	IMPDH2	3	49064299	rs9813813	49207093	A	T	0.110	-1.328	0.258	2.57E-07	0.071
UC	IMPDH2	3	49064299	rs9813813	49207093	A	T	0.110	-1.445	0.307	2.53E-06	0.050
IBD	UBE2L3	22	21941029	rs2070512	21949411	C	A	0.194	0.163	0.021	1.06E-14	0.169
UC	UBE2L3	22	21941029	rs2070512	21949411	C	A	0.194	0.116	0.027	1.76E-05	0.698
CD	BCL2L15	1	1.14E+08	rs1217393	1.14E+08	A	G	0.271	-0.705	0.170	3.39E-05	0.184
CD	STMN3	20	62271061	rs909333	62270637	G	T	0.207	0.547	0.102	8.31E-08	0.200
IBD	STMN3	20	62271061	rs909333	62270637	G	T	0.207	0.555	0.087	2.01E-10	0.055
UC	STMN3	20	62271061	rs909333	62270637	G	T	0.207	0.498	0.098	4.04E-07	0.062
GORD	PBX2	6	32154964	rs693906	31835164	C	G	0.113	0.154	0.036	1.65E-05	0.058
VHD	AGER	6	32150423	rs693906	31835164	C	G	0.113	0.212	0.052	4.82E-05	0.092

B) Nominally significant effect on VHD of Bonferroni-significant gastrointestinal loci.

Disease	Gene	chr	Probe_bp	topSNP	topSNP_bp	A1	A2	Freq	b_SMR	se_SMR	p_SMR	p_HEIDI
VHD	CFDP1	16	75397489	rs7201989	75471499	G	A	0.246	-0.056	0.018	0.001	0.119
VHD	STAT3	17	40502964	rs1053004	40466092	G	A	0.380	0.093	0.047	0.048	0.948
VHD	IMPDH2	3	49064299	rs34037363	48821036	T	C	0.114	-0.209	0.105	0.047	0.089
VHD	UBE2L3	22	21941029	rs2070512	21949411	C	A	0.194	-0.029	0.009	0.002	0.623
VHD	STMN3	20	62271061	rs909333	62270637	G	T	0.207	-0.076	0.036	0.036	0.093

C) Effect of AGER gene on VHD and gastrointestinal disorders.

Disease	Gene	chr	Probe_bp	topSNP	topSNP_bp	A1	A2	Freq	b_SMR	se_SMR	p_SMR	p_HEIDI
CD	AGER	6	32150423	rs693906	31835164	C	G	0.113	-0.312	0.120	0.009	6.16E-08
GORD	AGER	6	32150423	rs693906	31835164	C	G	0.113	-0.209	0.049	2.02E-05	0.022
IBD	AGER	6	32148745	rs204995	32154285	G	A	0.217	-0.252	0.108	0.020	1.60E-04
IBS	AGER	6	32150423	rs693906	31835164	C	G	0.113	-0.168	0.052	0.001	0.045
UC	AGER	6	32148745	rs204995	32154285	G	A	0.217	-0.870	0.185	2.59E-06	0.002
VHD	AGER	6	32150423	rs693906	31835164	C	G	0.113	0.212	0.052	4.82E-05	0.092

Supplementary table 6. Results of the summary-based Mendelian randomization integrating whole-blood expression quantitative trait loci. For each disease-gene association, we report the strongest effect observed between GTEX and eQTLGen datasets.

A) Bonferroni-significant disease-gene association with HEIDI P > 0.05.

Disease	Gene	chr	gene_bp	topSNP	topSNP_bp	A1	A2	Freq	b_SMR	se_SMR	p_SMR	p_HEIDI
CD	DNMT3A	2	25510652	rs7578575	25488819	A	T	0.329	-0.834	0.164	3.51E-07	0.383
IBD	DNMT3A	2	25510652	rs7578575	25488819	A	T	0.329	-0.745	0.130	9.22E-09	0.515
UC	DNMT3A	2	25510652	rs7578575	25488819	A	T	0.329	-0.627	0.156	5.73E-05	0.541
UC	CFDP1	16	75327596	rs9934007	75390532	C	T	0.754	0.273	0.070	9.61E-05	0.973
CD	STAT3	17	40502964	rs1053004	40466092	G	A	0.380	0.702	0.128	4.40E-08	0.056
CD	IMPDH2	3	49064299	rs9813813	49207093	A	T	0.110	-1.233	0.294	2.74E-05	0.213
IBD	IMPDH2	3	49064299	rs9813813	49207093	A	T	0.110	-1.328	0.258	2.57E-07	0.071
UC	IMPDH2	3	49064299	rs9813813	49207093	A	T	0.110	-1.445	0.307	2.53E-06	0.050
IBD	UBE2L3	22	21941029	rs2070512	21949411	C	A	0.194	0.163	0.021	1.06E-14	0.169
UC	UBE2L3	22	21941029	rs2070512	21949411	C	A	0.194	0.116	0.027	1.76E-05	0.698
CD	BCL2L15	1	1.14E+08	rs1217393	1.14E+08	A	G	0.271	-0.705	0.170	3.39E-05	0.184
CD	STMN3	20	62271061	rs909333	62270637	G	T	0.207	0.547	0.102	8.31E-08	0.200
IBD	STMN3	20	62271061	rs909333	62270637	G	T	0.207	0.555	0.087	2.01E-10	0.055
UC	STMN3	20	62271061	rs909333	62270637	G	T	0.207	0.498	0.098	4.04E-07	0.062
GORD	PBX2	6	32154964	rs693906	31835164	C	G	0.113	0.154	0.036	1.65E-05	0.058
VHD	AGER	6	32150423	rs693906	31835164	C	G	0.113	0.212	0.052	4.82E-05	0.092

B) Nominally significant effect on VHD of Bonferroni-significant gastrointestinal loci.

Disease	Gene	chr	Probe_bp	topSNP	topSNP_bp	A1	A2	Freq	b_SMR	se_SMR	p_SMR	p_HEIDI
VHD	CFDP1	16	75397489	rs7201989	75471499	G	A	0.246	-0.056	0.018	0.001	0.119
VHD	STAT3	17	40502964	rs1053004	40466092	G	A	0.380	0.093	0.047	0.048	0.948
VHD	IMPDH2	3	49064299	rs34037363	48821036	T	C	0.114	-0.209	0.105	0.047	0.089
VHD	UBE2L3	22	21941029	rs2070512	21949411	C	A	0.194	-0.029	0.009	0.002	0.623
VHD	STMN3	20	62271061	rs909333	62270637	G	T	0.207	-0.076	0.036	0.036	0.093

C) Effect of AGER gene on VHD and gastrointestinal disorders.

Disease	Gene	chr	Probe_bp	topSNP	topSNP_bp	A1	A2	Freq	b_SMR	se_SMR	p_SMR	p_HEIDI
CD	AGER	6	32150423	rs693906	31835164	C	G	0.113	-0.312	0.120	0.009	6.16E-08
GORD	AGER	6	32150423	rs693906	31835164	C	G	0.113	-0.209	0.049	2.02E-05	0.022
IBD	AGER	6	32148745	rs204995	32154285	G	A	0.217	-0.252	0.108	0.020	1.60E-04
IBS	AGER	6	32150423	rs693906	31835164	C	G	0.113	-0.168	0.052	0.001	0.045
UC	AGER	6	32148745	rs204995	32154285	G	A	0.217	-0.870	0.185	2.59E-06	0.002
VHD	AGER	6	32150423	rs693906	31835164	C	G	0.113	0.212	0.052	4.82E-05	0.092

Supplementary table 6. Results of the summary-based Mendelian randomization integrating whole-blood expression quantitative trait loci. For each disease-gene association, we report the strongest effect observed between GTEX and eQTLGen datasets.

A) Bonferroni-significant disease-gene association with HEIDI P > 0.05.

Disease	Gene	chr	gene_bp	topSNP	topSNP_bp	A1	A2	Freq	b_SMR	se_SMR	p_SMR	p_HEIDI
CD	DNMT3A	2	25510652	rs7578575	25488819	A	T	0.329	-0.834	0.164	3.51E-07	0.383
IBD	DNMT3A	2	25510652	rs7578575	25488819	A	T	0.329	-0.745	0.130	9.22E-09	0.515
UC	DNMT3A	2	25510652	rs7578575	25488819	A	T	0.329	-0.627	0.156	5.73E-05	0.541
UC	CFDP1	16	75327596	rs9934007	75390532	C	T	0.754	0.273	0.070	9.61E-05	0.973
CD	STAT3	17	40502964	rs1053004	40466092	G	A	0.380	0.702	0.128	4.40E-08	0.056
CD	IMPDH2	3	49064299	rs9813813	49207093	A	T	0.110	-1.233	0.294	2.74E-05	0.213
IBD	IMPDH2	3	49064299	rs9813813	49207093	A	T	0.110	-1.328	0.258	2.57E-07	0.071
UC	IMPDH2	3	49064299	rs9813813	49207093	A	T	0.110	-1.445	0.307	2.53E-06	0.050
IBD	UBE2L3	22	21941029	rs2070512	21949411	C	A	0.194	0.163	0.021	1.06E-14	0.169
UC	UBE2L3	22	21941029	rs2070512	21949411	C	A	0.194	0.116	0.027	1.76E-05	0.698
CD	BCL2L15	1	1.14E+08	rs1217393	1.14E+08	A	G	0.271	-0.705	0.170	3.39E-05	0.184
CD	STMN3	20	62271061	rs909333	62270637	G	T	0.207	0.547	0.102	8.31E-08	0.200
IBD	STMN3	20	62271061	rs909333	62270637	G	T	0.207	0.555	0.087	2.01E-10	0.055
UC	STMN3	20	62271061	rs909333	62270637	G	T	0.207	0.498	0.098	4.04E-07	0.062
GORD	PBX2	6	32154964	rs693906	31835164	C	G	0.113	0.154	0.036	1.65E-05	0.058
VHD	AGER	6	32150423	rs693906	31835164	C	G	0.113	0.212	0.052	4.82E-05	0.092

B) Nominally significant effect on VHD of Bonferroni-significant gastrointestinal loci.

Disease	Gene	chr	Probe_bp	topSNP	topSNP_bp	A1	A2	Freq	b_SMR	se_SMR	p_SMR	p_HEIDI
VHD	CFDP1	16	75397489	rs7201989	75471499	G	A	0.246	-0.056	0.018	0.001	0.119
VHD	STAT3	17	40502964	rs1053004	40466092	G	A	0.380	0.093	0.047	0.048	0.948
VHD	IMPDH2	3	49064299	rs34037363	48821036	T	C	0.114	-0.209	0.105	0.047	0.089
VHD	UBE2L3	22	21941029	rs2070512	21949411	C	A	0.194	-0.029	0.009	0.002	0.623
VHD	STMN3	20	62271061	rs909333	62270637	G	T	0.207	-0.076	0.036	0.036	0.093

C) Effect of AGER gene on VHD and gastrointestinal disorders.

Disease	Gene	chr	Probe_bp	topSNP	topSNP_bp	A1	A2	Freq	b_SMR	se_SMR	p_SMR	p_HEIDI
CD	AGER	6	32150423	rs693906	31835164	C	G	0.113	-0.312	0.120	0.009	6.16E-08
GORD	AGER	6	32150423	rs693906	31835164	C	G	0.113	-0.209	0.049	2.02E-05	0.022
IBD	AGER	6	32148745	rs204995	32154285	G	A	0.217	-0.252	0.108	0.020	1.60E-04
IBS	AGER	6	32150423	rs693906	31835164	C	G	0.113	-0.168	0.052	0.001	0.045
UC	AGER	6	32148745	rs204995	32154285	G	A	0.217	-0.870	0.185	2.59E-06	0.002
VHD	AGER	6	32150423	rs693906	31835164	C	G	0.113	0.212	0.052	4.82E-05	0.092

Supplementary table 6. Results of the summary-based Mendelian randomization integrating whole-blood expression quantitative trait loci. For each disease-gene association, we report the strongest effect observed between GTEX and eQTLGen datasets.

A) Bonferroni-significant disease-gene association with HEIDI P > 0.05.

Disease	Gene	chr	gene_bp	topSNP	topSNP_bp	A1	A2	Freq	b_SMR	se_SMR	p_SMR	p_HEIDI
CD	DNMT3A	2	25510652	rs7578575	25488819	A	T	0.329	-0.834	0.164	3.51E-07	0.383
IBD	DNMT3A	2	25510652	rs7578575	25488819	A	T	0.329	-0.745	0.130	9.22E-09	0.515
UC	DNMT3A	2	25510652	rs7578575	25488819	A	T	0.329	-0.627	0.156	5.73E-05	0.541
UC	CFDP1	16	75327596	rs9934007	75390532	C	T	0.754	0.273	0.070	9.61E-05	0.973
CD	STAT3	17	40502964	rs1053004	40466092	G	A	0.380	0.702	0.128	4.40E-08	0.056
CD	IMPDH2	3	49064299	rs9813813	49207093	A	T	0.110	-1.233	0.294	2.74E-05	0.213
IBD	IMPDH2	3	49064299	rs9813813	49207093	A	T	0.110	-1.328	0.258	2.57E-07	0.071
UC	IMPDH2	3	49064299	rs9813813	49207093	A	T	0.110	-1.445	0.307	2.53E-06	0.050
IBD	UBE2L3	22	21941029	rs2070512	21949411	C	A	0.194	0.163	0.021	1.06E-14	0.169
UC	UBE2L3	22	21941029	rs2070512	21949411	C	A	0.194	0.116	0.027	1.76E-05	0.698
CD	BCL2L15	1	1.14E+08	rs1217393	1.14E+08	A	G	0.271	-0.705	0.170	3.39E-05	0.184
CD	STMN3	20	62271061	rs909333	62270637	G	T	0.207	0.547	0.102	8.31E-08	0.200
IBD	STMN3	20	62271061	rs909333	62270637	G	T	0.207	0.555	0.087	2.01E-10	0.055
UC	STMN3	20	62271061	rs909333	62270637	G	T	0.207	0.498	0.098	4.04E-07	0.062
GORD	PBX2	6	32154964	rs693906	31835164	C	G	0.113	0.154	0.036	1.65E-05	0.058
VHD	AGER	6	32150423	rs693906	31835164	C	G	0.113	0.212	0.052	4.82E-05	0.092

B) Nominally significant effect on VHD of Bonferroni-significant gastrointestinal loci.

Disease	Gene	chr	Probe_bp	topSNP	topSNP_bp	A1	A2	Freq	b_SMR	se_SMR	p_SMR	p_HEIDI
VHD	CFDP1	16	75397489	rs7201989	75471499	G	A	0.246	-0.056	0.018	0.001	0.119
VHD	STAT3	17	40502964	rs1053004	40466092	G	A	0.380	0.093	0.047	0.048	0.948
VHD	IMPDH2	3	49064299	rs34037363	48821036	T	C	0.114	-0.209	0.105	0.047	0.089
VHD	UBE2L3	22	21941029	rs2070512	21949411	C	A	0.194	-0.029	0.009	0.002	0.623
VHD	STMN3	20	62271061	rs909333	62270637	G	T	0.207	-0.076	0.036	0.036	0.093

C) Effect of AGER gene on VHD and gastrointestinal disorders.

Disease	Gene	chr	Probe_bp	topSNP	topSNP_bp	A1	A2	Freq	b_SMR	se_SMR	p_SMR	p_HEIDI
CD	AGER	6	32150423	rs693906	31835164	C	G	0.113	-0.312	0.120	0.009	6.16E-08
GORD	AGER	6	32150423	rs693906	31835164	C	G	0.113	-0.209	0.049	2.02E-05	0.022
IBD	AGER	6	32148745	rs204995	32154285	G	A	0.217	-0.252	0.108	0.020	1.60E-04
IBS	AGER	6	32150423	rs693906	31835164	C	G	0.113	-0.168	0.052	0.001	0.045
UC	AGER	6	32148745	rs204995	32154285	G	A	0.217	-0.870	0.185	2.59E-06	0.002
VHD	AGER	6	32150423	rs693906	31835164	C	G	0.113	0.212	0.052	4.82E-05	0.092

Supplementary table 6. Results of the summary-based Mendelian randomization integrating whole-blood expression quantitative trait loci. For each disease-gene association, we report the strongest effect observed between GTEX and eQTLGen datasets.

A) Bonferroni-significant disease-gene association with HEIDI P > 0.05.

Disease	Gene	chr	gene_bp	topSNP	topSNP_bp	A1	A2	Freq	b_SMR	se_SMR	p_SMR	p_HEIDI
CD	DNMT3A	2	25510652	rs7578575	25488819	A	T	0.329	-0.834	0.164	3.51E-07	0.383
IBD	DNMT3A	2	25510652	rs7578575	25488819	A	T	0.329	-0.745	0.130	9.22E-09	0.515
UC	DNMT3A	2	25510652	rs7578575	25488819	A	T	0.329	-0.627	0.156	5.73E-05	0.541
UC	CFDP1	16	75327596	rs9934007	75390532	C	T	0.754	0.273	0.070	9.61E-05	0.973
CD	STAT3	17	40502964	rs1053004	40466092	G	A	0.380	0.702	0.128	4.40E-08	0.056
CD	IMPDH2	3	49064299	rs9813813	49207093	A	T	0.110	-1.233	0.294	2.74E-05	0.213
IBD	IMPDH2	3	49064299	rs9813813	49207093	A	T	0.110	-1.328	0.258	2.57E-07	0.071
UC	IMPDH2	3	49064299	rs9813813	49207093	A	T	0.110	-1.445	0.307	2.53E-06	0.050
IBD	UBE2L3	22	21941029	rs2070512	21949411	C	A	0.194	0.163	0.021	1.06E-14	0.169
UC	UBE2L3	22	21941029	rs2070512	21949411	C	A	0.194	0.116	0.027	1.76E-05	0.698
CD	BCL2L15	1	1.14E+08	rs1217393	1.14E+08	A	G	0.271	-0.705	0.170	3.39E-05	0.184
CD	STMN3	20	62271061	rs909333	62270637	G	T	0.207	0.547	0.102	8.31E-08	0.200
IBD	STMN3	20	62271061	rs909333	62270637	G	T	0.207	0.555	0.087	2.01E-10	0.055
UC	STMN3	20	62271061	rs909333	62270637	G	T	0.207	0.498	0.098	4.04E-07	0.062
GORD	PBX2	6	32154964	rs693906	31835164	C	G	0.113	0.154	0.036	1.65E-05	0.058
VHD	AGER	6	32150423	rs693906	31835164	C	G	0.113	0.212	0.052	4.82E-05	0.092

B) Nominally significant effect on VHD of Bonferroni-significant gastrointestinal loci.

Disease	Gene	chr	Probe_bp	topSNP	topSNP_bp	A1	A2	Freq	b_SMR	se_SMR	p_SMR	p_HEIDI
VHD	CFDP1	16	75397489	rs7201989	75471499	G	A	0.246	-0.056	0.018	0.001	0.119
VHD	STAT3	17	40502964	rs1053004	40466092	G	A	0.380	0.093	0.047	0.048	0.948
VHD	IMPDH2	3	49064299	rs34037363	48821036	T	C	0.114	-0.209	0.105	0.047	0.089
VHD	UBE2L3	22	21941029	rs2070512	21949411	C	A	0.194	-0.029	0.009	0.002	0.623
VHD	STMN3	20	62271061	rs909333	62270637	G	T	0.207	-0.076	0.036	0.036	0.093

C) Effect of AGER gene on VHD and gastrointestinal disorders.

Disease	Gene	chr	Probe_bp	topSNP	topSNP_bp	A1	A2	Freq	b_SMR	se_SMR	p_SMR	p_HEIDI
CD	AGER	6	32150423	rs693906	31835164	C	G	0.113	-0.312	0.120	0.009	6.16E-08
GORD	AGER	6	32150423	rs693906	31835164	C	G	0.113	-0.209	0.049	2.02E-05	0.022
IBD	AGER	6	32148745	rs204995	32154285	G	A	0.217	-0.252	0.108	0.020	1.60E-04
IBS	AGER	6	32150423	rs693906	31835164	C	G	0.113	-0.168	0.052	0.001	0.045
UC	AGER	6	32148745	rs204995	32154285	G	A	0.217	-0.870	0.185	2.59E-06	0.002
VHD	AGER	6	32150423	rs693906	31835164	C	G	0.113	0.212	0.052	4.82E-05	0.092

Supplementary table 6. Results of the summary-based Mendelian randomization integrating whole-blood expression quantitative trait loci. For each disease-gene association, we report the strongest effect observed between GTEX and eQTLGen datasets.

A) Bonferroni-significant disease-gene association with HEIDI P > 0.05.

Disease	Gene	chr	gene_bp	topSNP	topSNP_bp	A1	A2	Freq	b_SMR	se_SMR	p_SMR	p_HEIDI
CD	DNMT3A	2	25510652	rs7578575	25488819	A	T	0.329	-0.834	0.164	3.51E-07	0.383
IBD	DNMT3A	2	25510652	rs7578575	25488819	A	T	0.329	-0.745	0.130	9.22E-09	0.515
UC	DNMT3A	2	25510652	rs7578575	25488819	A	T	0.329	-0.627	0.156	5.73E-05	0.541
UC	CFDP1	16	75327596	rs9934007	75390532	C	T	0.754	0.273	0.070	9.61E-05	0.973
CD	STAT3	17	40502964	rs1053004	40466092	G	A	0.380	0.702	0.128	4.40E-08	0.056
CD	IMPDH2	3	49064299	rs9813813	49207093	A	T	0.110	-1.233	0.294	2.74E-05	0.213
IBD	IMPDH2	3	49064299	rs9813813	49207093	A	T	0.110	-1.328	0.258	2.57E-07	0.071
UC	IMPDH2	3	49064299	rs9813813	49207093	A	T	0.110	-1.445	0.307	2.53E-06	0.050
IBD	UBE2L3	22	21941029	rs2070512	21949411	C	A	0.194	0.163	0.021	1.06E-14	0.169
UC	UBE2L3	22	21941029	rs2070512	21949411	C	A	0.194	0.116	0.027	1.76E-05	0.698
CD	BCL2L15	1	1.14E+08	rs1217393	1.14E+08	A	G	0.271	-0.705	0.170	3.39E-05	0.184
CD	STMN3	20	62271061	rs909333	62270637	G	T	0.207	0.547	0.102	8.31E-08	0.200
IBD	STMN3	20	62271061	rs909333	62270637	G	T	0.207	0.555	0.087	2.01E-10	0.055
UC	STMN3	20	62271061	rs909333	62270637	G	T	0.207	0.498	0.098	4.04E-07	0.062
GORD	PBX2	6	32154964	rs693906	31835164	C	G	0.113	0.154	0.036	1.65E-05	0.058
VHD	AGER	6	32150423	rs693906	31835164	C	G	0.113	0.212	0.052	4.82E-05	0.092

B) Nominally significant effect on VHD of Bonferroni-significant gastrointestinal loci.

Disease	Gene	chr	Probe_bp	topSNP	topSNP_bp	A1	A2	Freq	b_SMR	se_SMR	p_SMR	p_HEIDI
VHD	CFDP1	16	75397489	rs7201989	75471499	G	A	0.246	-0.056	0.018	0.001	0.119
VHD	STAT3	17	40502964	rs1053004	40466092	G	A	0.380	0.093	0.047	0.048	0.948
VHD	IMPDH2	3	49064299	rs34037363	48821036	T	C	0.114	-0.209	0.105	0.047	0.089
VHD	UBE2L3	22	21941029	rs2070512	21949411	C	A	0.194	-0.029	0.009	0.002	0.623
VHD	STMN3	20	62271061	rs909333	62270637	G	T	0.207	-0.076	0.036	0.036	0.093

C) Effect of AGER gene on VHD and gastrointestinal disorders.

Disease	Gene	chr	Probe_bp	topSNP	topSNP_bp	A1	A2	Freq	b_SMR	se_SMR	p_SMR	p_HEIDI
CD	AGER	6	32150423	rs693906	31835164	C	G	0.113	-0.312	0.120	0.009	6.16E-08
GORD	AGER	6	32150423	rs693906	31835164	C	G	0.113	-0.209	0.049	2.02E-05	0.022
IBD	AGER	6	32148745	rs204995	32154285	G	A	0.217	-0.252	0.108	0.020	1.60E-04
IBS	AGER	6	32150423	rs693906	31835164	C	G	0.113	-0.168	0.052	0.001	0.045
UC	AGER	6	32148745	rs204995	32154285	G	A	0.217	-0.870	0.185	2.59E-06	0.002
VHD	AGER	6	32150423	rs693906	31835164	C	G	0.113	0.212	0.052	4.82E-05	0.092

Supplementary table 6. Results of the summary-based Mendelian randomization integrating whole-blood expression quantitative trait loci. For each disease-gene association, we report the strongest effect observed between GTEX and eQTLGen datasets.

A) Bonferroni-significant disease-gene association with HEIDI P > 0.05.

Disease	Gene	chr	gene_bp	topSNP	topSNP_bp	A1	A2	Freq	b_SMR	se_SMR	p_SMR	p_HEIDI
CD	DNMT3A	2	25510652	rs7578575	25488819	A	T	0.329	-0.834	0.164	3.51E-07	0.383
IBD	DNMT3A	2	25510652	rs7578575	25488819	A	T	0.329	-0.745	0.130	9.22E-09	0.515
UC	DNMT3A	2	25510652	rs7578575	25488819	A	T	0.329	-0.627	0.156	5.73E-05	0.541
UC	CFDP1	16	75327596	rs9934007	75390532	C	T	0.754	0.273	0.070	9.61E-05	0.973
CD	STAT3	17	40502964	rs1053004	40466092	G	A	0.380	0.702	0.128	4.40E-08	0.056
CD	IMPDH2	3	49064299	rs9813813	49207093	A	T	0.110	-1.233	0.294	2.74E-05	0.213
IBD	IMPDH2	3	49064299	rs9813813	49207093	A	T	0.110	-1.328	0.258	2.57E-07	0.071
UC	IMPDH2	3	49064299	rs9813813	49207093	A	T	0.110	-1.445	0.307	2.53E-06	0.050
IBD	UBE2L3	22	21941029	rs2070512	21949411	C	A	0.194	0.163	0.021	1.06E-14	0.169
UC	UBE2L3	22	21941029	rs2070512	21949411	C	A	0.194	0.116	0.027	1.76E-05	0.698
CD	BCL2L15	1	1.14E+08	rs1217393	1.14E+08	A	G	0.271	-0.705	0.170	3.39E-05	0.184
CD	STMN3	20	62271061	rs909333	62270637	G	T	0.207	0.547	0.102	8.31E-08	0.200
IBD	STMN3	20	62271061	rs909333	62270637	G	T	0.207	0.555	0.087	2.01E-10	0.055
UC	STMN3	20	62271061	rs909333	62270637	G	T	0.207	0.498	0.098	4.04E-07	0.062
GORD	PBX2	6	32154964	rs693906	31835164	C	G	0.113	0.154	0.036	1.65E-05	0.058
VHD	AGER	6	32150423	rs693906	31835164	C	G	0.113	0.212	0.052	4.82E-05	0.092

B) Nominally significant effect on VHD of Bonferroni-significant gastrointestinal loci.

Disease	Gene	chr	Probe_bp	topSNP	topSNP_bp	A1	A2	Freq	b_SMR	se_SMR	p_SMR	p_HEIDI
VHD	CFDP1	16	75397489	rs7201989	75471499	G	A	0.246	-0.056	0.018	0.001	0.119
VHD	STAT3	17	40502964	rs1053004	40466092	G	A	0.380	0.093	0.047	0.048	0.948
VHD	IMPDH2	3	49064299	rs34037363	48821036	T	C	0.114	-0.209	0.105	0.047	0.089
VHD	UBE2L3	22	21941029	rs2070512	21949411	C	A	0.194	-0.029	0.009	0.002	0.623
VHD	STMN3	20	62271061	rs909333	62270637	G	T	0.207	-0.076	0.036	0.036	0.093

C) Effect of AGER gene on VHD and gastrointestinal disorders.

Disease	Gene	chr	Probe_bp	topSNP	topSNP_bp	A1	A2	Freq	b_SMR	se_SMR	p_SMR	p_HEIDI
CD	AGER	6	32150423	rs693906	31835164	C	G	0.113	-0.312	0.120	0.009	6.16E-08
GORD	AGER	6	32150423	rs693906	31835164	C	G	0.113	-0.209	0.049	2.02E-05	0.022
IBD	AGER	6	32148745	rs204995	32154285	G	A	0.217	-0.252	0.108	0.020	1.60E-04
IBS	AGER	6	32150423	rs693906	31835164	C	G	0.113	-0.168	0.052	0.001	0.045
UC	AGER	6	32148745	rs204995	32154285	G	A	0.217	-0.870	0.185	2.59E-06	0.002
VHD	AGER	6	32150423	rs693906	31835164	C	G	0.113	0.212	0.052	4.82E-05	0.092

Supplementary table 6. Results of the summary-based Mendelian randomization integrating whole-blood expression quantitative trait loci. For each disease-gene association, we report the strongest effect observed between GTEX and eQTLGen datasets.

A) Bonferroni-significant disease-gene association with HEIDI P > 0.05.

Disease	Gene	chr	gene_bp	topSNP	topSNP_bp	A1	A2	Freq	b_SMR	se_SMR	p_SMR	p_HEIDI
CD	DNMT3A	2	25510652	rs7578575	25488819	A	T	0.329	-0.834	0.164	3.51E-07	0.383
IBD	DNMT3A	2	25510652	rs7578575	25488819	A	T	0.329	-0.745	0.130	9.22E-09	0.515
UC	DNMT3A	2	25510652	rs7578575	25488819	A	T	0.329	-0.627	0.156	5.73E-05	0.541
UC	CFDP1	16	75327596	rs9934007	75390532	C	T	0.754	0.273	0.070	9.61E-05	0.973
CD	STAT3	17	40502964	rs1053004	40466092	G	A	0.380	0.702	0.128	4.40E-08	0.056
CD	IMPDH2	3	49064299	rs9813813	49207093	A	T	0.110	-1.233	0.294	2.74E-05	0.213
IBD	IMPDH2	3	49064299	rs9813813	49207093	A	T	0.110	-1.328	0.258	2.57E-07	0.071
UC	IMPDH2	3	49064299	rs9813813	49207093	A	T	0.110	-1.445	0.307	2.53E-06	0.050
IBD	UBE2L3	22	21941029	rs2070512	21949411	C	A	0.194	0.163	0.021	1.06E-14	0.169
UC	UBE2L3	22	21941029	rs2070512	21949411	C	A	0.194	0.116	0.027	1.76E-05	0.698
CD	BCL2L15	1	1.14E+08	rs1217393	1.14E+08	A	G	0.271	-0.705	0.170	3.39E-05	0.184
CD	STMN3	20	62271061	rs909333	62270637	G	T	0.207	0.547	0.102	8.31E-08	0.200
IBD	STMN3	20	62271061	rs909333	62270637	G	T	0.207	0.555	0.087	2.01E-10	0.055
UC	STMN3	20	62271061	rs909333	62270637	G	T	0.207	0.498	0.098	4.04E-07	0.062
GORD	PBX2	6	32154964	rs693906	31835164	C	G	0.113	0.154	0.036	1.65E-05	0.058
VHD	AGER	6	32150423	rs693906	31835164	C	G	0.113	0.212	0.052	4.82E-05	0.092

B) Nominally significant effect on VHD of Bonferroni-significant gastrointestinal loci.

Disease	Gene	chr	Probe_bp	topSNP	topSNP_bp	A1	A2	Freq	b_SMR	se_SMR	p_SMR	p_HEIDI
VHD	CFDP1	16	75397489	rs7201989	75471499	G	A	0.246	-0.056	0.018	0.001	0.119
VHD	STAT3	17	40502964	rs1053004	40466092	G	A	0.380	0.093	0.047	0.048	0.948
VHD	IMPDH2	3	49064299	rs34037363	48821036	T	C	0.114	-0.209	0.105	0.047	0.089
VHD	UBE2L3	22	21941029	rs2070512	21949411	C	A	0.194	-0.029	0.009	0.002	0.623
VHD	STMN3	20	62271061	rs909333	62270637	G	T	0.207	-0.076	0.036	0.036	0.093

C) Effect of AGER gene on VHD and gastrointestinal disorders.

Disease	Gene	chr	Probe_bp	topSNP	topSNP_bp	A1	A2	Freq	b_SMR	se_SMR	p_SMR	p_HEIDI
CD	AGER	6	32150423	rs693906	31835164	C	G	0.113	-0.312	0.120	0.009	6.16E-08
GORD	AGER	6	32150423	rs693906	31835164	C	G	0.113	-0.209	0.049	2.02E-05	0.022
IBD	AGER	6	32148745	rs204995	32154285	G	A	0.217	-0.252	0.108	0.020	1.60E-04
IBS	AGER	6	32150423	rs693906	31835164	C	G	0.113	-0.168	0.052	0.001	0.045
UC	AGER	6	32148745	rs204995	32154285	G	A	0.217	-0.870	0.185	2.59E-06	0.002
VHD	AGER	6	32150423	rs693906	31835164	C	G	0.113	0.212	0.052	4.82E-05	0.092

Supplementary table 6. Results of the summary-based Mendelian randomization integrating whole-blood expression quantitative trait loci. For each disease-gene association, we report the strongest effect observed between GTEX and eQTLGen datasets.

A) Bonferroni-significant disease-gene association with HEIDI P > 0.05.

Disease	Gene	chr	gene_bp	topSNP	topSNP_bp	A1	A2	Freq	b_SMR	se_SMR	p_SMR	p_HEIDI
CD	DNMT3A	2	25510652	rs7578575	25488819	A	T	0.329	-0.834	0.164	3.51E-07	0.383
IBD	DNMT3A	2	25510652	rs7578575	25488819	A	T	0.329	-0.745	0.130	9.22E-09	0.515
UC	DNMT3A	2	25510652	rs7578575	25488819	A	T	0.329	-0.627	0.156	5.73E-05	0.541
UC	CFDP1	16	75327596	rs9934007	75390532	C	T	0.754	0.273	0.070	9.61E-05	0.973
CD	STAT3	17	40502964	rs1053004	40466092	G	A	0.380	0.702	0.128	4.40E-08	0.056
CD	IMPDH2	3	49064299	rs9813813	49207093	A	T	0.110	-1.233	0.294	2.74E-05	0.213
IBD	IMPDH2	3	49064299	rs9813813	49207093	A	T	0.110	-1.328	0.258	2.57E-07	0.071
UC	IMPDH2	3	49064299	rs9813813	49207093	A	T	0.110	-1.445	0.307	2.53E-06	0.050
IBD	UBE2L3	22	21941029	rs2070512	21949411	C	A	0.194	0.163	0.021	1.06E-14	0.169
UC	UBE2L3	22	21941029	rs2070512	21949411	C	A	0.194	0.116	0.027	1.76E-05	0.698
CD	BCL2L15	1	1.14E+08	rs1217393	1.14E+08	A	G	0.271	-0.705	0.170	3.39E-05	0.184
CD	STMN3	20	62271061	rs909333	62270637	G	T	0.207	0.547	0.102	8.31E-08	0.200
IBD	STMN3	20	62271061	rs909333	62270637	G	T	0.207	0.555	0.087	2.01E-10	0.055
UC	STMN3	20	62271061	rs909333	62270637	G	T	0.207	0.498	0.098	4.04E-07	0.062
GORD	PBX2	6	32154964	rs693906	31835164	C	G	0.113	0.154	0.036	1.65E-05	0.058
VHD	AGER	6	32150423	rs693906	31835164	C	G	0.113	0.212	0.052	4.82E-05	0.092

B) Nominally significant effect on VHD of Bonferroni-significant gastrointestinal loci.

Disease	Gene	chr	Probe_bp	topSNP	topSNP_bp	A1	A2	Freq	b_SMR	se_SMR	p_SMR	p_HEIDI
VHD	CFDP1	16	75397489	rs7201989	75471499	G	A	0.246	-0.056	0.018	0.001	0.119
VHD	STAT3	17	40502964	rs1053004	40466092	G	A	0.380	0.093	0.047	0.048	0.948
VHD	IMPDH2	3	49064299	rs34037363	48821036	T	C	0.114	-0.209	0.105	0.047	0.089
VHD	UBE2L3	22	21941029	rs2070512	21949411	C	A	0.194	-0.029	0.009	0.002	0.623
VHD	STMN3	20	62271061	rs909333	62270637	G	T	0.207	-0.076	0.036	0.036	0.093

C) Effect of AGER gene on VHD and gastrointestinal disorders.

Disease	Gene	chr	Probe_bp	topSNP	topSNP_bp	A1	A2	Freq	b_SMR	se_SMR	p_SMR	p_HEIDI
CD	AGER	6	32150423	rs693906	31835164	C	G	0.113	-0.312	0.120	0.009	6.16E-08
GORD	AGER	6	32150423	rs693906	31835164	C	G	0.113	-0.209	0.049	2.02E-05	0.022
IBD	AGER	6	32148745	rs204995	32154285	G	A	0.217	-0.252	0.108	0.020	1.60E-04
IBS	AGER	6	32150423	rs693906	31835164	C	G	0.113	-0.168	0.052	0.001	0.045
UC	AGER	6	32148745	rs204995	32154285	G	A	0.217	-0.870	0.185	2.59E-06	0.002
VHD	AGER	6	32150423	rs693906	31835164	C	G	0.113	0.212	0.052	4.82E-05	0.092

Supplementary table 6. Results of the summary-based Mendelian randomization integrating whole-blood expression quantitative trait loci. For each disease-gene association, we report the strongest effect observed between GTEX and eQTLGen datasets.

A) Bonferroni-significant disease-gene association with HEIDI P > 0.05.

Disease	Gene	chr	gene_bp	topSNP	topSNP_bp	A1	A2	Freq	b_SMR	se_SMR	p_SMR	p_HEIDI
CD	DNMT3A	2	25510652	rs7578575	25488819	A	T	0.329	-0.834	0.164	3.51E-07	0.383
IBD	DNMT3A	2	25510652	rs7578575	25488819	A	T	0.329	-0.745	0.130	9.22E-09	0.515
UC	DNMT3A	2	25510652	rs7578575	25488819	A	T	0.329	-0.627	0.156	5.73E-05	0.541
UC	CFDP1	16	75327596	rs9934007	75390532	C	T	0.754	0.273	0.070	9.61E-05	0.973
CD	STAT3	17	40502964	rs1053004	40466092	G	A	0.380	0.702	0.128	4.40E-08	0.056
CD	IMPDH2	3	49064299	rs9813813	49207093	A	T	0.110	-1.233	0.294	2.74E-05	0.213
IBD	IMPDH2	3	49064299	rs9813813	49207093	A	T	0.110	-1.328	0.258	2.57E-07	0.071
UC	IMPDH2	3	49064299	rs9813813	49207093	A	T	0.110	-1.445	0.307	2.53E-06	0.050
IBD	UBE2L3	22	21941029	rs2070512	21949411	C	A	0.194	0.163	0.021	1.06E-14	0.169
UC	UBE2L3	22	21941029	rs2070512	21949411	C	A	0.194	0.116	0.027	1.76E-05	0.698
CD	BCL2L15	1	1.14E+08	rs1217393	1.14E+08	A	G	0.271	-0.705	0.170	3.39E-05	0.184
CD	STMN3	20	62271061	rs909333	62270637	G	T	0.207	0.547	0.102	8.31E-08	0.200
IBD	STMN3	20	62271061	rs909333	62270637	G	T	0.207	0.555	0.087	2.01E-10	0.055
UC	STMN3	20	62271061	rs909333	62270637	G	T	0.207	0.498	0.098	4.04E-07	0.062
GORD	PBX2	6	32154964	rs693906	31835164	C	G	0.113	0.154	0.036	1.65E-05	0.058
VHD	AGER	6	32150423	rs693906	31835164	C	G	0.113	0.212	0.052	4.82E-05	0.092

B) Nominally significant effect on VHD of Bonferroni-significant gastrointestinal loci.

Disease	Gene	chr	Probe_bp	topSNP	topSNP_bp	A1	A2	Freq	b_SMR	se_SMR	p_SMR	p_HEIDI
VHD	CFDP1	16	75397489	rs7201989	75471499	G	A	0.246	-0.056	0.018	0.001	0.119
VHD	STAT3	17	40502964	rs1053004	40466092	G	A	0.380	0.093	0.047	0.048	0.948
VHD	IMPDH2	3	49064299	rs34037363	48821036	T	C	0.114	-0.209	0.105	0.047	0.089
VHD	UBE2L3	22	21941029	rs2070512	21949411	C	A	0.194	-0.029	0.009	0.002	0.623
VHD	STMN3	20	62271061	rs909333	62270637	G	T	0.207	-0.076	0.036	0.036	0.093

C) Effect of AGER gene on VHD and gastrointestinal disorders.

Disease	Gene	chr	Probe_bp	topSNP	topSNP_bp	A1	A2	Freq	b_SMR	se_SMR	p_SMR	p_HEIDI
CD	AGER	6	32150423	rs693906	31835164	C	G	0.113	-0.312	0.120	0.009	6.16E-08
GORD	AGER	6	32150423	rs693906	31835164	C	G	0.113	-0.209	0.049	2.02E-05	0.022
IBD	AGER	6	32148745	rs204995	32154285	G	A	0.217	-0.252	0.108	0.020	1.60E-04
IBS	AGER	6	32150423	rs693906	31835164	C	G	0.113	-0.168	0.052	0.001	0.045
UC	AGER	6	32148745	rs204995	32154285	G	A	0.217	-0.870	0.185	2.59E-06	0.002
VHD	AGER	6	32150423	rs693906	31835164	C	G	0.113	0.212	0.052	4.82E-05	0.092

Supplementary table 6. Results of the summary-based Mendelian randomization integrating whole-blood expression quantitative trait loci. For each disease-gene association, we report the strongest effect observed between GTEX and eQTLGen datasets.

A) Bonferroni-significant disease-gene association with HEIDI P > 0.05.

Disease	Gene	chr	gene_bp	topSNP	topSNP_bp	A1	A2	Freq	b_SMR	se_SMR	p_SMR	p_HEIDI
CD	DNMT3A	2	25510652	rs7578575	25488819	A	T	0.329	-0.834	0.164	3.51E-07	0.383
IBD	DNMT3A	2	25510652	rs7578575	25488819	A	T	0.329	-0.745	0.130	9.22E-09	0.515
UC	DNMT3A	2	25510652	rs7578575	25488819	A	T	0.329	-0.627	0.156	5.73E-05	0.541
UC	CFDP1	16	75327596	rs9934007	75390532	C	T	0.754	0.273	0.070	9.61E-05	0.973
CD	STAT3	17	40502964	rs1053004	40466092	G	A	0.380	0.702	0.128	4.40E-08	0.056
CD	IMPDH2	3	49064299	rs9813813	49207093	A	T	0.110	-1.233	0.294	2.74E-05	0.213
IBD	IMPDH2	3	49064299	rs9813813	49207093	A	T	0.110	-1.328	0.258	2.57E-07	0.071
UC	IMPDH2	3	49064299	rs9813813	49207093	A	T	0.110	-1.445	0.307	2.53E-06	0.050
IBD	UBE2L3	22	21941029	rs2070512	21949411	C	A	0.194	0.163	0.021	1.06E-14	0.169
UC	UBE2L3	22	21941029	rs2070512	21949411	C	A	0.194	0.116	0.027	1.76E-05	0.698
CD	BCL2L15	1	1.14E+08	rs1217393	1.14E+08	A	G	0.271	-0.705	0.170	3.39E-05	0.184
CD	STMN3	20	62271061	rs909333	62270637	G	T	0.207	0.547	0.102	8.31E-08	0.200
IBD	STMN3	20	62271061	rs909333	62270637	G	T	0.207	0.555	0.087	2.01E-10	0.055
UC	STMN3	20	62271061	rs909333	62270637	G	T	0.207	0.498	0.098	4.04E-07	0.062
GORD	PBX2	6	32154964	rs693906	31835164	C	G	0.113	0.154	0.036	1.65E-05	0.058
VHD	AGER	6	32150423	rs693906	31835164	C	G	0.113	0.212	0.052	4.82E-05	0.092

B) Nominally significant effect on VHD of Bonferroni-significant gastrointestinal loci.

Disease	Gene	chr	Probe_bp	topSNP	topSNP_bp	A1	A2	Freq	b_SMR	se_SMR	p_SMR	p_HEIDI
VHD	CFDP1	16	75397489	rs7201989	75471499	G	A	0.246	-0.056	0.018	0.001	0.119
VHD	STAT3	17	40502964	rs1053004	40466092	G	A	0.380	0.093	0.047	0.048	0.948
VHD	IMPDH2	3	49064299	rs34037363	48821036	T	C	0.114	-0.209	0.105	0.047	0.089
VHD	UBE2L3	22	21941029	rs2070512	21949411	C	A	0.194	-0.029	0.009	0.002	0.623
VHD	STMN3	20	62271061	rs909333	62270637	G	T	0.207	-0.076	0.036	0.036	0.093

C) Effect of AGER gene on VHD and gastrointestinal disorders.

Disease	Gene	chr	Probe_bp	topSNP	topSNP_bp	A1	A2	Freq	b_SMR	se_SMR	p_SMR	p_HEIDI
CD	AGER	6	32150423	rs693906	31835164	C	G	0.113	-0.312	0.120	0.009	6.16E-08
GORD	AGER	6	32150423	rs693906	31835164	C	G	0.113	-0.209	0.049	2.02E-05	0.022
IBD	AGER	6	32148745	rs204995	32154285	G	A	0.217	-0.252	0.108	0.020	1.60E-04
IBS	AGER	6	32150423	rs693906	31835164	C	G	0.113	-0.168	0.052	0.001	0.045
UC	AGER	6	32148745	rs204995	32154285	G	A	0.217	-0.870	0.185	2.59E-06	0.002
VHD	AGER	6	32150423	rs693906	31835164	C	G	0.113	0.212	0.052	4.82E-05	0.092

Supplementary table 6. Results of the summary-based Mendelian randomization integrating whole-blood expression quantitative trait loci. For each disease-gene association, we report the strongest effect observed between GTEX and eQTLGen datasets.

A) Bonferroni-significant disease-gene association with HEIDI P > 0.05.

Disease	Gene	chr	gene_bp	topSNP	topSNP_bp	A1	A2	Freq	b_SMR	se_SMR	p_SMR	p_HEIDI
CD	DNMT3A	2	25510652	rs7578575	25488819	A	T	0.329	-0.834	0.164	3.51E-07	0.383
IBD	DNMT3A	2	25510652	rs7578575	25488819	A	T	0.329	-0.745	0.130	9.22E-09	0.515
UC	DNMT3A	2	25510652	rs7578575	25488819	A	T	0.329	-0.627	0.156	5.73E-05	0.541
UC	CFDP1	16	75327596	rs9934007	75390532	C	T	0.754	0.273	0.070	9.61E-05	0.973
CD	STAT3	17	40502964	rs1053004	40466092	G	A	0.380	0.702	0.128	4.40E-08	0.056
CD	IMPDH2	3	49064299	rs9813813	49207093	A	T	0.110	-1.233	0.294	2.74E-05	0.213
IBD	IMPDH2	3	49064299	rs9813813	49207093	A	T	0.110	-1.328	0.258	2.57E-07	0.071
UC	IMPDH2	3	49064299	rs9813813	49207093	A	T	0.110	-1.445	0.307	2.53E-06	0.050
IBD	UBE2L3	22	21941029	rs2070512	21949411	C	A	0.194	0.163	0.021	1.06E-14	0.169
UC	UBE2L3	22	21941029	rs2070512	21949411	C	A	0.194	0.116	0.027	1.76E-05	0.698
CD	BCL2L15	1	1.14E+08	rs1217393	1.14E+08	A	G	0.271	-0.705	0.170	3.39E-05	0.184
CD	STMN3	20	62271061	rs909333	62270637	G	T	0.207	0.547	0.102	8.31E-08	0.200
IBD	STMN3	20	62271061	rs909333	62270637	G	T	0.207	0.555	0.087	2.01E-10	0.055
UC	STMN3	20	62271061	rs909333	62270637	G	T	0.207	0.498	0.098	4.04E-07	0.062
GORD	PBX2	6	32154964	rs693906	31835164	C	G	0.113	0.154	0.036	1.65E-05	0.058
VHD	AGER	6	32150423	rs693906	31835164	C	G	0.113	0.212	0.052	4.82E-05	0.092

B) Nominally significant effect on VHD of Bonferroni-significant gastrointestinal loci.

Disease	Gene	chr	Probe_bp	topSNP	topSNP_bp	A1	A2	Freq	b_SMR	se_SMR	p_SMR	p_HEIDI
VHD	CFDP1	16	75397489	rs7201989	75471499	G	A	0.246	-0.056	0.018	0.001	0.119
VHD	STAT3	17	40502964	rs1053004	40466092	G	A	0.380	0.093	0.047	0.048	0.948
VHD	IMPDH2	3	49064299	rs34037363	48821036	T	C	0.114	-0.209	0.105	0.047	0.089
VHD	UBE2L3	22	21941029	rs2070512	21949411	C	A	0.194	-0.029	0.009	0.002	0.623
VHD	STMN3	20	62271061	rs909333	62270637	G	T	0.207	-0.076	0.036	0.036	0.093

C) Effect of AGER gene on VHD and gastrointestinal disorders.

Disease	Gene	chr	Probe_bp	topSNP	topSNP_bp	A1	A2	Freq	b_SMR	se_SMR	p_SMR	p_HEIDI
CD	AGER	6	32150423	rs693906	31835164	C	G	0.113	-0.312	0.120	0.009	6.16E-08
GORD	AGER	6	32150423	rs693906	31835164	C	G	0.113	-0.209	0.049	2.02E-05	0.022
IBD	AGER	6	32148745	rs204995	32154285	G	A	0.217	-0.252	0.108	0.020	1.60E-04
IBS	AGER	6	32150423	rs693906	31835164	C	G	0.113	-0.168	0.052	0.001	0.045
UC	AGER	6	32148745	rs204995	32154285	G	A	0.217	-0.870	0.185	2.59E-06	0.002
VHD	AGER	6	32150423	rs693906	31835164	C	G	0.113	0.212	0.052	4.82E-05	0.092

Supplementary table 6. Results of the summary-based Mendelian randomization integrating whole-blood expression quantitative trait loci. For each disease-gene association, we report the strongest effect observed between GTEX and eQTLGen datasets.

A) Bonferroni-significant disease-gene association with HEIDI P > 0.05.

Disease	Gene	chr	gene_bp	topSNP	topSNP_bp	A1	A2	Freq	b_SMR	se_SMR	p_SMR	p_HEIDI
CD	DNMT3A	2	25510652	rs7578575	25488819	A	T	0.329	-0.834	0.164	3.51E-07	0.383
IBD	DNMT3A	2	25510652	rs7578575	25488819	A	T	0.329	-0.745	0.130	9.22E-09	0.515
UC	DNMT3A	2	25510652	rs7578575	25488819	A	T	0.329	-0.627	0.156	5.73E-05	0.541
UC	CFDP1	16	75327596	rs9934007	75390532	C	T	0.754	0.273	0.070	9.61E-05	0.973
CD	STAT3	17	40502964	rs1053004	40466092	G	A	0.380	0.702	0.128	4.40E-08	0.056
CD	IMPDH2	3	49064299	rs9813813	49207093	A	T	0.110	-1.233	0.294	2.74E-05	0.213
IBD	IMPDH2	3	49064299	rs9813813	49207093	A	T	0.110	-1.328	0.258	2.57E-07	0.071
UC	IMPDH2	3	49064299	rs9813813	49207093	A	T	0.110	-1.445	0.307	2.53E-06	0.050
IBD	UBE2L3	22	21941029	rs2070512	21949411	C	A	0.194	0.163	0.021	1.06E-14	0.169
UC	UBE2L3	22	21941029	rs2070512	21949411	C	A	0.194	0.116	0.027	1.76E-05	0.698
CD	BCL2L15	1	1.14E+08	rs1217393	1.14E+08	A	G	0.271	-0.705	0.170	3.39E-05	0.184
CD	STMN3	20	62271061	rs909333	62270637	G	T	0.207	0.547	0.102	8.31E-08	0.200
IBD	STMN3	20	62271061	rs909333	62270637	G	T	0.207	0.555	0.087	2.01E-10	0.055
UC	STMN3	20	62271061	rs909333	62270637	G	T	0.207	0.498	0.098	4.04E-07	0.062
GORD	PBX2	6	32154964	rs693906	31835164	C	G	0.113	0.154	0.036	1.65E-05	0.058
VHD	AGER	6	32150423	rs693906	31835164	C	G	0.113	0.212	0.052	4.82E-05	0.092

B) Nominally significant effect on VHD of Bonferroni-significant gastrointestinal loci.

Disease	Gene	chr	Probe_bp	topSNP	topSNP_bp	A1	A2	Freq	b_SMR	se_SMR	p_SMR	p_HEIDI
VHD	CFDP1	16	75397489	rs7201989	75471499	G	A	0.246	-0.056	0.018	0.001	0.119
VHD	STAT3	17	40502964	rs1053004	40466092	G	A	0.380	0.093	0.047	0.048	0.948
VHD	IMPDH2	3	49064299	rs34037363	48821036	T	C	0.114	-0.209	0.105	0.047	0.089
VHD	UBE2L3	22	21941029	rs2070512	21949411	C	A	0.194	-0.029	0.009	0.002	0.623
VHD	STMN3	20	62271061	rs909333	62270637	G	T	0.207	-0.076	0.036	0.036	0.093

C) Effect of AGER gene on VHD and gastrointestinal disorders.

Disease	Gene	chr	Probe_bp	topSNP	topSNP_bp	A1	A2	Freq	b_SMR	se_SMR	p_SMR	p_HEIDI
CD	AGER	6	32150423	rs693906	31835164	C	G	0.113	-0.312	0.120	0.009	6.16E-08
GORD	AGER	6	32150423	rs693906	31835164	C	G	0.113	-0.209	0.049	2.02E-05	0.022
IBD	AGER	6	32148745	rs204995	32154285	G	A	0.217	-0.252	0.108	0.020	1.60E-04
IBS	AGER	6	32150423	rs693906	31835164	C	G	0.113	-0.168	0.052	0.001	0.045
UC	AGER	6	32148745	rs204995	32154285	G	A	0.217	-0.870	0.185	2.59E-06	0.002
VHD	AGER	6	32150423	rs693906	31835164	C	G	0.113	0.212	0.052	4.82E-05	0.092

Supplementary table 6. Results of the summary-based Mendelian randomization integrating whole-blood expression quantitative trait loci. For each disease-gene association, we report the strongest effect observed between GTEX and eQTLGen datasets.

A) Bonferroni-significant disease-gene association with HEIDI P > 0.05.

Disease	Gene	chr	gene_bp	topSNP	topSNP_bp	A1	A2	Freq	b_SMR	se_SMR	p_SMR	p_HEIDI
CD	DNMT3A	2	25510652	rs7578575	25488819	A	T	0.329	-0.834	0.164	3.51E-07	0.383
IBD	DNMT3A	2	25510652	rs7578575	25488819	A	T	0.329	-0.745	0.130	9.22E-09	0.515
UC	DNMT3A	2	25510652	rs7578575	25488819	A	T	0.329	-0.627	0.156	5.73E-05	0.541
UC	CFDP1	16	75327596	rs9934007	75390532	C	T	0.754	0.273	0.070	9.61E-05	0.973
CD	STAT3	17	40502964	rs1053004	40466092	G	A	0.380	0.702	0.128	4.40E-08	0.056
CD	IMPDH2	3	49064299	rs9813813	49207093	A	T	0.110	-1.233	0.294	2.74E-05	0.213
IBD	IMPDH2	3	49064299	rs9813813	49207093	A	T	0.110	-1.328	0.258	2.57E-07	0.071
UC	IMPDH2	3	49064299	rs9813813	49207093	A	T	0.110	-1.445	0.307	2.53E-06	0.050
IBD	UBE2L3	22	21941029	rs2070512	21949411	C	A	0.194	0.163	0.021	1.06E-14	0.169
UC	UBE2L3	22	21941029	rs2070512	21949411	C	A	0.194	0.116	0.027	1.76E-05	0.698
CD	BCL2L15	1	1.14E+08	rs1217393	1.14E+08	A	G	0.271	-0.705	0.170	3.39E-05	0.184
CD	STMN3	20	62271061	rs909333	62270637	G	T	0.207	0.547	0.102	8.31E-08	0.200
IBD	STMN3	20	62271061	rs909333	62270637	G	T	0.207	0.555	0.087	2.01E-10	0.055
UC	STMN3	20	62271061	rs909333	62270637	G	T	0.207	0.498	0.098	4.04E-07	0.062
GORD	PBX2	6	32154964	rs693906	31835164	C	G	0.113	0.154	0.036	1.65E-05	0.058
VHD	AGER	6	32150423	rs693906	31835164	C	G	0.113	0.212	0.052	4.82E-05	0.092

B) Nominally significant effect on VHD of Bonferroni-significant gastrointestinal loci.

Disease	Gene	chr	Probe_bp	topSNP	topSNP_bp	A1	A2	Freq	b_SMR	se_SMR	p_SMR	p_HEIDI
VHD	CFDP1	16	75397489	rs7201989	75471499	G	A	0.246	-0.056	0.018	0.001	0.119
VHD	STAT3	17	40502964	rs1053004	40466092	G	A	0.380	0.093	0.047	0.048	0.948
VHD	IMPDH2	3	49064299	rs34037363	48821036	T	C	0.114	-0.209	0.105	0.047	0.089
VHD	UBE2L3	22	21941029	rs2070512	21949411	C	A	0.194	-0.029	0.009	0.002	0.623
VHD	STMN3	20	62271061	rs909333	62270637	G	T	0.207	-0.076	0.036	0.036	0.093

C) Effect of AGER gene on VHD and gastrointestinal disorders.

Disease	Gene	chr	Probe_bp	topSNP	topSNP_bp	A1	A2	Freq	b_SMR	se_SMR	p_SMR	p_HEIDI
CD	AGER	6	32150423	rs693906	31835164	C	G	0.113	-0.312	0.120	0.009	6.16E-08
GORD	AGER	6	32150423	rs693906	31835164	C	G	0.113	-0.209	0.049	2.02E-05	0.022
IBD	AGER	6	32148745	rs204995	32154285	G	A	0.217	-0.252	0.108	0.020	1.60E-04
IBS	AGER	6	32150423	rs693906	31835164	C	G	0.113	-0.168	0.052	0.001	0.045
UC	AGER	6	32148745	rs204995	32154285	G	A	0.217	-0.870	0.185	2.59E-06	0.002
VHD	AGER	6	32150423	rs693906	31835164	C	G	0.113	0.212	0.052	4.82E-05	0.092

Supplementary table 6. Results of the summary-based Mendelian randomization integrating whole-blood expression quantitative trait loci. For each disease-gene association, we report the strongest effect observed between GTEX and eQTLGen datasets.

A) Bonferroni-significant disease-gene association with HEIDI P > 0.05.

Disease	Gene	chr	gene_bp	topSNP	topSNP_bp	A1	A2	Freq	b_SMR	se_SMR	p_SMR	p_HEIDI
CD	DNMT3A	2	25510652	rs7578575	25488819	A	T	0.329	-0.834	0.164	3.51E-07	0.383
IBD	DNMT3A	2	25510652	rs7578575	25488819	A	T	0.329	-0.745	0.130	9.22E-09	0.515
UC	DNMT3A	2	25510652	rs7578575	25488819	A	T	0.329	-0.627	0.156	5.73E-05	0.541
UC	CFDP1	16	75327596	rs9934007	75390532	C	T	0.754	0.273	0.070	9.61E-05	0.973
CD	STAT3	17	40502964	rs1053004	40466092	G	A	0.380	0.702	0.128	4.40E-08	0.056
CD	IMPDH2	3	49064299	rs9813813	49207093	A	T	0.110	-1.233	0.294	2.74E-05	0.213
IBD	IMPDH2	3	49064299	rs9813813	49207093	A	T	0.110	-1.328	0.258	2.57E-07	0.071
UC	IMPDH2	3	49064299	rs9813813	49207093	A	T	0.110	-1.445	0.307	2.53E-06	0.050
IBD	UBE2L3	22	21941029	rs2070512	21949411	C	A	0.194	0.163	0.021	1.06E-14	0.169
UC	UBE2L3	22	21941029	rs2070512	21949411	C	A	0.194	0.116	0.027	1.76E-05	0.698
CD	BCL2L15	1	1.14E+08	rs1217393	1.14E+08	A	G	0.271	-0.705	0.170	3.39E-05	0.184
CD	STMN3	20	62271061	rs909333	62270637	G	T	0.207	0.547	0.102	8.31E-08	0.200
IBD	STMN3	20	62271061	rs909333	62270637	G	T	0.207	0.555	0.087	2.01E-10	0.055
UC	STMN3	20	62271061	rs909333	62270637	G	T	0.207	0.498	0.098	4.04E-07	0.062
GORD	PBX2	6	32154964	rs693906	31835164	C	G	0.113	0.154	0.036	1.65E-05	0.058
VHD	AGER	6	32150423	rs693906	31835164	C	G	0.113	0.212	0.052	4.82E-05	0.092

B) Nominally significant effect on VHD of Bonferroni-significant gastrointestinal loci.

Disease	Gene	chr	Probe_bp	topSNP	topSNP_bp	A1	A2	Freq	b_SMR	se_SMR	p_SMR	p_HEIDI
VHD	CFDP1	16	75397489	rs7201989	75471499	G	A	0.246	-0.056	0.018	0.001	0.119
VHD	STAT3	17	40502964	rs1053004	40466092	G	A	0.380	0.093	0.047	0.048	0.948
VHD	IMPDH2	3	49064299	rs34037363	48821036	T	C	0.114	-0.209	0.105	0.047	0.089
VHD	UBE2L3	22	21941029	rs2070512	21949411	C	A	0.194	-0.029	0.009	0.002	0.623
VHD	STMN3	20	62271061	rs909333	62270637	G	T	0.207	-0.076	0.036	0.036	0.093

C) Effect of AGER gene on VHD and gastrointestinal disorders.

Disease	Gene	chr	Probe_bp	topSNP	topSNP_bp	A1	A2	Freq	b_SMR	se_SMR	p_SMR	p_HEIDI
CD	AGER	6	32150423	rs693906	31835164	C	G	0.113	-0.312	0.120	0.009	6.16E-08
GORD	AGER	6	32150423	rs693906	31835164	C	G	0.113	-0.209	0.049	2.02E-05	0.022
IBD	AGER	6	32148745	rs204995	32154285	G	A	0.217	-0.252	0.108	0.020	1.60E-04
IBS	AGER	6	32150423	rs693906	31835164	C	G	0.113	-0.168	0.052	0.001	0.045
UC	AGER	6	32148745	rs204995	32154285	G	A	0.217	-0.870	0.185	2.59E-06	0.002
VHD	AGER	6	32150423	rs693906	31835164	C	G	0.113	0.212	0.052	4.82E-05	0.092

Supplementary table 6. Results of the summary-based Mendelian randomization integrating whole-blood expression quantitative trait loci. For each disease-gene association, we report the strongest effect observed between GTEX and eQTLGen datasets.

A) Bonferroni-significant disease-gene association with HEIDI P > 0.05.

Disease	Gene	chr	gene_bp	topSNP	topSNP_bp	A1	A2	Freq	b_SMR	se_SMR	p_SMR	p_HEIDI
CD	DNMT3A	2	25510652	rs7578575	25488819	A	T	0.329	-0.834	0.164	3.51E-07	0.383
IBD	DNMT3A	2	25510652	rs7578575	25488819	A	T	0.329	-0.745	0.130	9.22E-09	0.515
UC	DNMT3A	2	25510652	rs7578575	25488819	A	T	0.329	-0.627	0.156	5.73E-05	0.541
UC	CFDP1	16	75327596	rs9934007	75390532	C	T	0.754	0.273	0.070	9.61E-05	0.973
CD	STAT3	17	40502964	rs1053004	40466092	G	A	0.380	0.702	0.128	4.40E-08	0.056
CD	IMPDH2	3	49064299	rs9813813	49207093	A	T	0.110	-1.233	0.294	2.74E-05	0.213
IBD	IMPDH2	3	49064299	rs9813813	49207093	A	T	0.110	-1.328	0.258	2.57E-07	0.071
UC	IMPDH2	3	49064299	rs9813813	49207093	A	T	0.110	-1.445	0.307	2.53E-06	0.050
IBD	UBE2L3	22	21941029	rs2070512	21949411	C	A	0.194	0.163	0.021	1.06E-14	0.169
UC	UBE2L3	22	21941029	rs2070512	21949411	C	A	0.194	0.116	0.027	1.76E-05	0.698
CD	BCL2L15	1	1.14E+08	rs1217393	1.14E+08	A	G	0.271	-0.705	0.170	3.39E-05	0.184
CD	STMN3	20	62271061	rs909333	62270637	G	T	0.207	0.547	0.102	8.31E-08	0.200
IBD	STMN3	20	62271061	rs909333	62270637	G	T	0.207	0.555	0.087	2.01E-10	0.055
UC	STMN3	20	62271061	rs909333	62270637	G	T	0.207	0.498	0.098	4.04E-07	0.062
GORD	PBX2	6	32154964	rs693906	31835164	C	G	0.113	0.154	0.036	1.65E-05	0.058
VHD	AGER	6	32150423	rs693906	31835164	C	G	0.113	0.212	0.052	4.82E-05	0.092

B) Nominally significant effect on VHD of Bonferroni-significant gastrointestinal loci.

Disease	Gene	chr	Probe_bp	topSNP	topSNP_bp	A1	A2	Freq	b_SMR	se_SMR	p_SMR	p_HEIDI
VHD	CFDP1	16	75397489	rs7201989	75471499	G	A	0.246	-0.056	0.018	0.001	0.119
VHD	STAT3	17	40502964	rs1053004	40466092	G	A	0.380	0.093	0.047	0.048	0.948
VHD	IMPDH2	3	49064299	rs34037363	48821036	T	C	0.114	-0.209	0.105	0.047	0.089
VHD	UBE2L3	22	21941029	rs2070512	21949411	C	A	0.194	-0.029	0.009	0.002	0.623
VHD	STMN3	20	62271061	rs909333	62270637	G	T	0.207	-0.076	0.036	0.036	0.093

C) Effect of AGER gene on VHD and gastrointestinal disorders.

Disease	Gene	chr	Probe_bp	topSNP	topSNP_bp	A1	A2	Freq	b_SMR	se_SMR	p_SMR	p_HEIDI
CD	AGER	6	32150423	rs693906	31835164	C	G	0.113	-0.312	0.120	0.009	6.16E-08
GORD	AGER	6	32150423	rs693906	31835164	C	G	0.113	-0.209	0.049	2.02E-05	0.022
IBD	AGER	6	32148745	rs204995	32154285	G	A	0.217	-0.252	0.108	0.020	1.60E-04
IBS	AGER	6	32150423	rs693906	31835164	C	G	0.113	-0.168	0.052	0.001	0.045
UC	AGER	6	32148745	rs204995	32154285	G	A	0.217	-0.870	0.185	2.59E-06	0.002
VHD	AGER	6	32150423	rs693906	31835164	C	G	0.113	0.212	0.052	4.82E-05	0.092

Supplementary table 6. Results of the summary-based Mendelian randomization integrating whole-blood expression quantitative trait loci. For each disease-gene association, we report the strongest effect observed between GTEX and eQTLGen datasets.

A) Bonferroni-significant disease-gene association with HEIDI P > 0.05.

Disease	Gene	chr	gene_bp	topSNP	topSNP_bp	A1	A2	Freq	b_SMR	se_SMR	p_SMR	p_HEIDI
CD	DNMT3A	2	25510652	rs7578575	25488819	A	T	0.329	-0.834	0.164	3.51E-07	0.383
IBD	DNMT3A	2	25510652	rs7578575	25488819	A	T	0.329	-0.745	0.130	9.22E-09	0.515
UC	DNMT3A	2	25510652	rs7578575	25488819	A	T	0.329	-0.627	0.156	5.73E-05	0.541
UC	CFDP1	16	75327596	rs9934007	75390532	C	T	0.754	0.273	0.070	9.61E-05	0.973
CD	STAT3	17	40502964	rs1053004	40466092	G	A	0.380	0.702	0.128	4.40E-08	0.056
CD	IMPDH2	3	49064299	rs9813813	49207093	A	T	0.110	-1.233	0.294	2.74E-05	0.213
IBD	IMPDH2	3	49064299	rs9813813	49207093	A	T	0.110	-1.328	0.258	2.57E-07	0.071
UC	IMPDH2	3	49064299	rs9813813	49207093	A	T	0.110	-1.445	0.307	2.53E-06	0.050
IBD	UBE2L3	22	21941029	rs2070512	21949411	C	A	0.194	0.163	0.021	1.06E-14	0.169
UC	UBE2L3	22	21941029	rs2070512	21949411	C	A	0.194	0.116	0.027	1.76E-05	0.698
CD	BCL2L15	1	1.14E+08	rs1217393	1.14E+08	A	G	0.271	-0.705	0.170	3.39E-05	0.184
CD	STMN3	20	62271061	rs909333	62270637	G	T	0.207	0.547	0.102	8.31E-08	0.200
IBD	STMN3	20	62271061	rs909333	62270637	G	T	0.207	0.555	0.087	2.01E-10	0.055
UC	STMN3	20	62271061	rs909333	62270637	G	T	0.207	0.498	0.098	4.04E-07	0.062
GORD	PBX2	6	32154964	rs693906	31835164	C	G	0.113	0.154	0.036	1.65E-05	0.058
VHD	AGER	6	32150423	rs693906	31835164	C	G	0.113	0.212	0.052	4.82E-05	0.092

B) Nominally significant effect on VHD of Bonferroni-significant gastrointestinal loci.

Disease	Gene	chr	Probe_bp	topSNP	topSNP_bp	A1	A2	Freq	b_SMR	se_SMR	p_SMR	p_HEIDI
VHD	CFDP1	16	75397489	rs7201989	75471499	G	A	0.246	-0.056	0.018	0.001	0.119
VHD	STAT3	17	40502964	rs1053004	40466092	G	A	0.380	0.093	0.047	0.048	0.948
VHD	IMPDH2	3	49064299	rs34037363	48821036	T	C	0.114	-0.209	0.105	0.047	0.089
VHD	UBE2L3	22	21941029	rs2070512	21949411	C	A	0.194	-0.029	0.009	0.002	0.623
VHD	STMN3	20	62271061	rs909333	62270637	G	T	0.207	-0.076	0.036	0.036	0.093

C) Effect of AGER gene on VHD and gastrointestinal disorders.

Disease	Gene	chr	Probe_bp	topSNP	topSNP_bp	A1	A2	Freq	b_SMR	se_SMR	p_SMR	p_HEIDI
CD	AGER	6	32150423	rs693906	31835164	C	G	0.113	-0.312	0.120	0.009	6.16E-08
GORD	AGER	6	32150423	rs693906	31835164	C	G	0.113	-0.209	0.049	2.02E-05	0.022
IBD	AGER	6	32148745	rs204995	32154285	G	A	0.217	-0.252	0.108	0.020	1.60E-04
IBS	AGER	6	32150423	rs693906	31835164	C	G	0.113	-0.168	0.052	0.001	0.045
UC	AGER	6	32148745	rs204995	32154285	G	A	0.217	-0.870	0.185	2.59E-06	0.002
VHD	AGER	6	32150423	rs693906	31835164	C	G	0.113	0.212	0.052	4.82E-05	0.092

Supplementary table 6. Results of the summary-based Mendelian randomization integrating whole-blood expression quantitative trait loci. For each disease-gene association, we report the strongest effect observed between GTEX and eQTLGen datasets.

A) Bonferroni-significant disease-gene association with HEIDI P > 0.05.

Disease	Gene	chr	gene_bp	topSNP	topSNP_bp	A1	A2	Freq	b_SMR	se_SMR	p_SMR	p_HEIDI
CD	DNMT3A	2	25510652	rs7578575	25488819	A	T	0.329	-0.834	0.164	3.51E-07	0.383
IBD	DNMT3A	2	25510652	rs7578575	25488819	A	T	0.329	-0.745	0.130	9.22E-09	0.515
UC	DNMT3A	2	25510652	rs7578575	25488819	A	T	0.329	-0.627	0.156	5.73E-05	0.541
UC	CFDP1	16	75327596	rs9934007	75390532	C	T	0.754	0.273	0.070	9.61E-05	0.973
CD	STAT3	17	40502964	rs1053004	40466092	G	A	0.380	0.702	0.128	4.40E-08	0.056
CD	IMPDH2	3	49064299	rs9813813	49207093	A	T	0.110	-1.233	0.294	2.74E-05	0.213
IBD	IMPDH2	3	49064299	rs9813813	49207093	A	T	0.110	-1.328	0.258	2.57E-07	0.071
UC	IMPDH2	3	49064299	rs9813813	49207093	A	T	0.110	-1.445	0.307	2.53E-06	0.050
IBD	UBE2L3	22	21941029	rs2070512	21949411	C	A	0.194	0.163	0.021	1.06E-14	0.169
UC	UBE2L3	22	21941029	rs2070512	21949411	C	A	0.194	0.116	0.027	1.76E-05	0.698
CD	BCL2L15	1	1.14E+08	rs1217393	1.14E+08	A	G	0.271	-0.705	0.170	3.39E-05	0.184
CD	STMN3	20	62271061	rs909333	62270637	G	T	0.207	0.547	0.102	8.31E-08	0.200
IBD	STMN3	20	62271061	rs909333	62270637	G	T	0.207	0.555	0.087	2.01E-10	0.055
UC	STMN3	20	62271061	rs909333	62270637	G	T	0.207	0.498	0.098	4.04E-07	0.062
GORD	PBX2	6	32154964	rs693906	31835164	C	G	0.113	0.154	0.036	1.65E-05	0.058
VHD	AGER	6	32150423	rs693906	31835164	C	G	0.113	0.212	0.052	4.82E-05	0.092

B) Nominally significant effect on VHD of Bonferroni-significant gastrointestinal loci.

Disease	Gene	chr	Probe_bp	topSNP	topSNP_bp	A1	A2	Freq	b_SMR	se_SMR	p_SMR	p_HEIDI
VHD	CFDP1	16	75397489	rs7201989	75471499	G	A	0.246	-0.056	0.018	0.001	0.119
VHD	STAT3	17	40502964	rs1053004	40466092	G	A	0.380	0.093	0.047	0.048	0.948
VHD	IMPDH2	3	49064299	rs34037363	48821036	T	C	0.114	-0.209	0.105	0.047	0.089
VHD	UBE2L3	22	21941029	rs2070512	21949411	C	A	0.194	-0.029	0.009	0.002	0.623
VHD	STMN3	20	62271061	rs909333	62270637	G	T	0.207	-0.076	0.036	0.036	0.093

C) Effect of AGER gene on VHD and gastrointestinal disorders.

Disease	Gene	chr	Probe_bp	topSNP	topSNP_bp	A1	A2	Freq	b_SMR	se_SMR	p_SMR	p_HEIDI
CD	AGER	6	32150423	rs693906	31835164	C	G	0.113	-0.312	0.120	0.009	6.16E-08
GORD	AGER	6	32150423	rs693906	31835164	C	G	0.113	-0.209	0.049	2.02E-05	0.022
IBD	AGER	6	32148745	rs204995	32154285	G	A	0.217	-0.252	0.108	0.020	1.60E-04
IBS	AGER	6	32150423	rs693906	31835164	C	G	0.113	-0.168	0.052	0.001	0.045
UC	AGER	6	32148745	rs204995	32154285	G	A	0.217	-0.870	0.185	2.59E-06	0.002
VHD	AGER	6	32150423	rs693906	31835164	C	G	0.113	0.212	0.052	4.82E-05	0.092

Supplementary table 6. Results of the summary-based Mendelian randomization integrating whole-blood expression quantitative trait loci. For each disease-gene association, we report the strongest effect observed between GTEX and eQTLGen datasets.

A) Bonferroni-significant disease-gene association with HEIDI P > 0.05.

Disease	Gene	chr	gene_bp	topSNP	topSNP_bp	A1	A2	Freq	b_SMR	se_SMR	p_SMR	p_HEIDI
CD	DNMT3A	2	25510652	rs7578575	25488819	A	T	0.329	-0.834	0.164	3.51E-07	0.383
IBD	DNMT3A	2	25510652	rs7578575	25488819	A	T	0.329	-0.745	0.130	9.22E-09	0.515
UC	DNMT3A	2	25510652	rs7578575	25488819	A	T	0.329	-0.627	0.156	5.73E-05	0.541
UC	CFDP1	16	75327596	rs9934007	75390532	C	T	0.754	0.273	0.070	9.61E-05	0.973
CD	STAT3	17	40502964	rs1053004	40466092	G	A	0.380	0.702	0.128	4.40E-08	0.056
CD	IMPDH2	3	49064299	rs9813813	49207093	A	T	0.110	-1.233	0.294	2.74E-05	0.213
IBD	IMPDH2	3	49064299	rs9813813	49207093	A	T	0.110	-1.328	0.258	2.57E-07	0.071
UC	IMPDH2	3	49064299	rs9813813	49207093	A	T	0.110	-1.445	0.307	2.53E-06	0.050
IBD	UBE2L3	22	21941029	rs2070512	21949411	C	A	0.194	0.163	0.021	1.06E-14	0.169
UC	UBE2L3	22	21941029	rs2070512	21949411	C	A	0.194	0.116	0.027	1.76E-05	0.698
CD	BCL2L15	1	1.14E+08	rs1217393	1.14E+08	A	G	0.271	-0.705	0.170	3.39E-05	0.184
CD	STMN3	20	62271061	rs909333	62270637	G	T	0.207	0.547	0.102	8.31E-08	0.200
IBD	STMN3	20	62271061	rs909333	62270637	G	T	0.207	0.555	0.087	2.01E-10	0.055
UC	STMN3	20	62271061	rs909333	62270637	G	T	0.207	0.498	0.098	4.04E-07	0.062
GORD	PBX2	6	32154964	rs693906	31835164	C	G	0.113	0.154	0.036	1.65E-05	0.058
VHD	AGER	6	32150423	rs693906	31835164	C	G	0.113	0.212	0.052	4.82E-05	0.092

B) Nominally significant effect on VHD of Bonferroni-significant gastrointestinal loci.

Disease	Gene	chr	Probe_bp	topSNP	topSNP_bp	A1	A2	Freq	b_SMR	se_SMR	p_SMR	p_HEIDI
VHD	CFDP1	16	75397489	rs7201989	75471499	G	A	0.246	-0.056	0.018	0.001	0.119
VHD	STAT3	17	40502964	rs1053004	40466092	G	A	0.380	0.093	0.047	0.048	0.948
VHD	IMPDH2	3	49064299	rs34037363	48821036	T	C	0.114	-0.209	0.105	0.047	0.089
VHD	UBE2L3	22	21941029	rs2070512	21949411	C	A	0.194	-0.029	0.009	0.002	0.623
VHD	STMN3	20	62271061	rs909333	62270637	G	T	0.207	-0.076	0.036	0.036	0.093

C) Effect of AGER gene on VHD and gastrointestinal disorders.

Disease	Gene	chr	Probe_bp	topSNP	topSNP_bp	A1	A2	Freq	b_SMR	se_SMR	p_SMR	p_HEIDI
CD	AGER	6	32150423	rs693906	31835164	C	G	0.113	-0.312	0.120	0.009	6.16E-08
GORD	AGER	6	32150423	rs693906	31835164	C	G	0.113	-0.209	0.049	2.02E-05	0.022
IBD	AGER	6	32148745	rs204995	32154285	G	A	0.217	-0.252	0.108	0.020	1.60E-04
IBS	AGER	6	32150423	rs693906	31835164	C	G	0.113	-0.168	0.052	0.001	0.045
UC	AGER	6	32148745	rs204995	32154285	G	A	0.217	-0.870	0.185	2.59E-06	0.002
VHD	AGER	6	32150423	rs693906	31835164	C	G	0.113	0.212	0.052	4.82E-05	0.092

Supplementary table 6. Results of the summary-based Mendelian randomization integrating whole-blood expression quantitative trait loci. For each disease-gene association, we report the strongest effect observed between GTEX and eQTLGen datasets.

A) Bonferroni-significant disease-gene association with HEIDI P > 0.05.

Disease	Gene	chr	gene_bp	topSNP	topSNP_bp	A1	A2	Freq	b_SMR	se_SMR	p_SMR	p_HEIDI
CD	DNMT3A	2	25510652	rs7578575	25488819	A	T	0.329	-0.834	0.164	3.51E-07	0.383
IBD	DNMT3A	2	25510652	rs7578575	25488819	A	T	0.329	-0.745	0.130	9.22E-09	0.515
UC	DNMT3A	2	25510652	rs7578575	25488819	A	T	0.329	-0.627	0.156	5.73E-05	0.541
UC	CFDP1	16	75327596	rs9934007	75390532	C	T	0.754	0.273	0.070	9.61E-05	0.973
CD	STAT3	17	40502964	rs1053004	40466092	G	A	0.380	0.702	0.128	4.40E-08	0.056
CD	IMPDH2	3	49064299	rs9813813	49207093	A	T	0.110	-1.233	0.294	2.74E-05	0.213
IBD	IMPDH2	3	49064299	rs9813813	49207093	A	T	0.110	-1.328	0.258	2.57E-07	0.071
UC	IMPDH2	3	49064299	rs9813813	49207093	A	T	0.110	-1.445	0.307	2.53E-06	0.050
IBD	UBE2L3	22	21941029	rs2070512	21949411	C	A	0.194	0.163	0.021	1.06E-14	0.169
UC	UBE2L3	22	21941029	rs2070512	21949411	C	A	0.194	0.116	0.027	1.76E-05	0.698
CD	BCL2L15	1	1.14E+08	rs1217393	1.14E+08	A	G	0.271	-0.705	0.170	3.39E-05	0.184
CD	STMN3	20	62271061	rs909333	62270637	G	T	0.207	0.547	0.102	8.31E-08	0.200
IBD	STMN3	20	62271061	rs909333	62270637	G	T	0.207	0.555	0.087	2.01E-10	0.055
UC	STMN3	20	62271061	rs909333	62270637	G	T	0.207	0.498	0.098	4.04E-07	0.062
GORD	PBX2	6	32154964	rs693906	31835164	C	G	0.113	0.154	0.036	1.65E-05	0.058
VHD	AGER	6	32150423	rs693906	31835164	C	G	0.113	0.212	0.052	4.82E-05	0.092

B) Nominally significant effect on VHD of Bonferroni-significant gastrointestinal loci.

Disease	Gene	chr	Probe_bp	topSNP	topSNP_bp	A1	A2	Freq	b_SMR	se_SMR	p_SMR	p_HEIDI
VHD	CFDP1	16	75397489	rs7201989	75471499	G	A	0.246	-0.056	0.018	0.001	0.119
VHD	STAT3	17	40502964	rs1053004	40466092	G	A	0.380	0.093	0.047	0.048	0.948
VHD	IMPDH2	3	49064299	rs34037363	48821036	T	C	0.114	-0.209	0.105	0.047	0.089
VHD	UBE2L3	22	21941029	rs2070512	21949411	C	A	0.194	-0.029	0.009	0.002	0.623
VHD	STMN3	20	62271061	rs909333	62270637	G	T	0.207	-0.076	0.036	0.036	0.093

C) Effect of AGER gene on VHD and gastrointestinal disorders.

Disease	Gene	chr	Probe_bp	topSNP	topSNP_bp	A1	A2	Freq	b_SMR	se_SMR	p_SMR	p_HEIDI
CD	AGER	6	32150423	rs693906	31835164	C	G	0.113	-0.312	0.120	0.009	6.16E-08
GORD	AGER	6	32150423	rs693906	31835164	C	G	0.113	-0.209	0.049	2.02E-05	0.022
IBD	AGER	6	32148745	rs204995	32154285	G	A	0.217	-0.252	0.108	0.020	1.60E-04
IBS	AGER	6	32150423	rs693906	31835164	C	G	0.113	-0.168	0.052	0.001	0.045
UC	AGER	6	32148745	rs204995	32154285	G	A	0.217	-0.870	0.185	2.59E-06	0.002
VHD	AGER	6	32150423	rs693906	31835164	C	G	0.113	0.212	0.052	4.82E-05	0.092

Supplementary table 6. Results of the summary-based Mendelian randomization integrating whole-blood expression quantitative trait loci. For each disease-gene association, we report the strongest effect observed between GTEX and eQTLGen datasets.

A) Bonferroni-significant disease-gene association with HEIDI P > 0.05.

Disease	Gene	chr	gene_bp	topSNP	topSNP_bp	A1	A2	Freq	b_SMR	se_SMR	p_SMR	p_HEIDI
CD	DNMT3A	2	25510652	rs7578575	25488819	A	T	0.329	-0.834	0.164	3.51E-07	0.383
IBD	DNMT3A	2	25510652	rs7578575	25488819	A	T	0.329	-0.745	0.130	9.22E-09	0.515
UC	DNMT3A	2	25510652	rs7578575	25488819	A	T	0.329	-0.627	0.156	5.73E-05	0.541
UC	CFDP1	16	75327596	rs9934007	75390532	C	T	0.754	0.273	0.070	9.61E-05	0.973
CD	STAT3	17	40502964	rs1053004	40466092	G	A	0.380	0.702	0.128	4.40E-08	0.056
CD	IMPDH2	3	49064299	rs9813813	49207093	A	T	0.110	-1.233	0.294	2.74E-05	0.213
IBD	IMPDH2	3	49064299	rs9813813	49207093	A	T	0.110	-1.328	0.258	2.57E-07	0.071
UC	IMPDH2	3	49064299	rs9813813	49207093	A	T	0.110	-1.445	0.307	2.53E-06	0.050
IBD	UBE2L3	22	21941029	rs2070512	21949411	C	A	0.194	0.163	0.021	1.06E-14	0.169
UC	UBE2L3	22	21941029	rs2070512	21949411	C	A	0.194	0.116	0.027	1.76E-05	0.698
CD	BCL2L15	1	1.14E+08	rs1217393	1.14E+08	A	G	0.271	-0.705	0.170	3.39E-05	0.184
CD	STMN3	20	62271061	rs909333	62270637	G	T	0.207	0.547	0.102	8.31E-08	0.200
IBD	STMN3	20	62271061	rs909333	62270637	G	T	0.207	0.555	0.087	2.01E-10	0.055
UC	STMN3	20	62271061	rs909333	62270637	G	T	0.207	0.498	0.098	4.04E-07	0.062
GORD	PBX2	6	32154964	rs693906	31835164	C	G	0.113	0.154	0.036	1.65E-05	0.058
VHD	AGER	6	32150423	rs693906	31835164	C	G	0.113	0.212	0.052	4.82E-05	0.092

B) Nominally significant effect on VHD of Bonferroni-significant gastrointestinal loci.

Disease	Gene	chr	Probe_bp	topSNP	topSNP_bp	A1	A2	Freq	b_SMR	se_SMR	p_SMR	p_HEIDI
VHD	CFDP1	16	75397489	rs7201989	75471499	G	A	0.246	-0.056	0.018	0.001	0.119
VHD	STAT3	17	40502964	rs1053004	40466092	G	A	0.380	0.093	0.047	0.048	0.948
VHD	IMPDH2	3	49064299	rs34037363	48821036	T	C	0.114	-0.209	0.105	0.047	0.089
VHD	UBE2L3	22	21941029	rs2070512	21949411	C	A	0.194	-0.029	0.009	0.002	0.623
VHD	STMN3	20	62271061	rs909333	62270637	G	T	0.207	-0.076	0.036	0.036	0.093

C) Effect of AGER gene on VHD and gastrointestinal disorders.

Disease	Gene	chr	Probe_bp	topSNP	topSNP_bp	A1	A2	Freq	b_SMR	se_SMR	p_SMR	p_HEIDI
CD	AGER	6	32150423	rs693906	31835164	C	G	0.113	-0.312	0.120	0.009	6.16E-08
GORD	AGER	6	32150423	rs693906	31835164	C	G	0.113	-0.209	0.049	2.02E-05	0.022
IBD	AGER	6	32148745	rs204995	32154285	G	A	0.217	-0.252	0.108	0.020	1.60E-04
IBS	AGER	6	32150423	rs693906	31835164	C	G	0.113	-0.168	0.052	0.001	0.045
UC	AGER	6	32148745	rs204995	32154285	G	A	0.217	-0.870	0.185	2.59E-06	0.002
VHD	AGER	6	32150423	rs693906	31835164	C	G	0.113	0.212	0.052	4.82E-05	0.092

Supplementary table 6. Results of the summary-based Mendelian randomization integrating whole-blood expression quantitative trait loci. For each disease-gene association, we report the strongest effect observed between GTEX and eQTLGen datasets.

A) Bonferroni-significant disease-gene association with HEIDI P > 0.05.

Disease	Gene	chr	gene_bp	topSNP	topSNP_bp	A1	A2	Freq	b_SMR	se_SMR	p_SMR	p_HEIDI
CD	DNMT3A	2	25510652	rs7578575	25488819	A	T	0.329	-0.834	0.164	3.51E-07	0.383
IBD	DNMT3A	2	25510652	rs7578575	25488819	A	T	0.329	-0.745	0.130	9.22E-09	0.515
UC	DNMT3A	2	25510652	rs7578575	25488819	A	T	0.329	-0.627	0.156	5.73E-05	0.541
UC	CFDP1	16	75327596	rs9934007	75390532	C	T	0.754	0.273	0.070	9.61E-05	0.973
CD	STAT3	17	40502964	rs1053004	40466092	G	A	0.380	0.702	0.128	4.40E-08	0.056
CD	IMPDH2	3	49064299	rs9813813	49207093	A	T	0.110	-1.233	0.294	2.74E-05	0.213
IBD	IMPDH2	3	49064299	rs9813813	49207093	A	T	0.110	-1.328	0.258	2.57E-07	0.071
UC	IMPDH2	3	49064299	rs9813813	49207093	A	T	0.110	-1.445	0.307	2.53E-06	0.050
IBD	UBE2L3	22	21941029	rs2070512	21949411	C	A	0.194	0.163	0.021	1.06E-14	0.169
UC	UBE2L3	22	21941029	rs2070512	21949411	C	A	0.194	0.116	0.027	1.76E-05	0.698
CD	BCL2L15	1	1.14E+08	rs1217393	1.14E+08	A	G	0.271	-0.705	0.170	3.39E-05	0.184
CD	STMN3	20	62271061	rs909333	62270637	G	T	0.207	0.547	0.102	8.31E-08	0.200
IBD	STMN3	20	62271061	rs909333	62270637	G	T	0.207	0.555	0.087	2.01E-10	0.055
UC	STMN3	20	62271061	rs909333	62270637	G	T	0.207	0.498	0.098	4.04E-07	0.062
GORD	PBX2	6	32154964	rs693906	31835164	C	G	0.113	0.154	0.036	1.65E-05	0.058
VHD	AGER	6	32150423	rs693906	31835164	C	G	0.113	0.212	0.052	4.82E-05	0.092

B) Nominally significant effect on VHD of Bonferroni-significant gastrointestinal loci.

Disease	Gene	chr	Probe_bp	topSNP	topSNP_bp	A1	A2	Freq	b_SMR	se_SMR	p_SMR	p_HEIDI
VHD	CFDP1	16	75397489	rs7201989	75471499	G	A	0.246	-0.056	0.018	0.001	0.119
VHD	STAT3	17	40502964	rs1053004	40466092	G	A	0.380	0.093	0.047	0.048	0.948
VHD	IMPDH2	3	49064299	rs34037363	48821036	T	C	0.114	-0.209	0.105	0.047	0.089
VHD	UBE2L3	22	21941029	rs2070512	21949411	C	A	0.194	-0.029	0.009	0.002	0.623
VHD	STMN3	20	62271061	rs909333	62270637	G	T	0.207	-0.076	0.036	0.036	0.093

C) Effect of AGER gene on VHD and gastrointestinal disorders.

Disease	Gene	chr	Probe_bp	topSNP	topSNP_bp	A1	A2	Freq	b_SMR	se_SMR	p_SMR	p_HEIDI
CD	AGER	6	32150423	rs693906	31835164	C	G	0.113	-0.312	0.120	0.009	6.16E-08
GORD	AGER	6	32150423	rs693906	31835164	C	G	0.113	-0.209	0.049	2.02E-05	0.022
IBD	AGER	6	32148745	rs204995	32154285	G	A	0.217	-0.252	0.108	0.020	1.60E-04
IBS	AGER	6	32150423	rs693906	31835164	C	G	0.113	-0.168	0.052	0.001	0.045
UC	AGER	6	32148745	rs204995	32154285	G	A	0.217	-0.870	0.185	2.59E-06	0.002
VHD	AGER	6	32150423	rs693906	31835164	C	G	0.113	0.212	0.052	4.82E-05	0.092

Supplementary table 6. Results of the summary-based Mendelian randomization integrating whole-blood expression quantitative trait loci. For each disease-gene association, we report the strongest effect observed between GTEX and eQTLGen datasets.

A) Bonferroni-significant disease-gene association with HEIDI P > 0.05.

Disease	Gene	chr	gene_bp	topSNP	topSNP_bp	A1	A2	Freq	b_SMR	se_SMR	p_SMR	p_HEIDI
CD	DNMT3A	2	25510652	rs7578575	25488819	A	T	0.329	-0.834	0.164	3.51E-07	0.383
IBD	DNMT3A	2	25510652	rs7578575	25488819	A	T	0.329	-0.745	0.130	9.22E-09	0.515
UC	DNMT3A	2	25510652	rs7578575	25488819	A	T	0.329	-0.627	0.156	5.73E-05	0.541
UC	CFDP1	16	75327596	rs9934007	75390532	C	T	0.754	0.273	0.070	9.61E-05	0.973
CD	STAT3	17	40502964	rs1053004	40466092	G	A	0.380	0.702	0.128	4.40E-08	0.056
CD	IMPDH2	3	49064299	rs9813813	49207093	A	T	0.110	-1.233	0.294	2.74E-05	0.213
IBD	IMPDH2	3	49064299	rs9813813	49207093	A	T	0.110	-1.328	0.258	2.57E-07	0.071
UC	IMPDH2	3	49064299	rs9813813	49207093	A	T	0.110	-1.445	0.307	2.53E-06	0.050
IBD	UBE2L3	22	21941029	rs2070512	21949411	C	A	0.194	0.163	0.021	1.06E-14	0.169
UC	UBE2L3	22	21941029	rs2070512	21949411	C	A	0.194	0.116	0.027	1.76E-05	0.698
CD	BCL2L15	1	1.14E+08	rs1217393	1.14E+08	A	G	0.271	-0.705	0.170	3.39E-05	0.184
CD	STMN3	20	62271061	rs909333	62270637	G	T	0.207	0.547	0.102	8.31E-08	0.200
IBD	STMN3	20	62271061	rs909333	62270637	G	T	0.207	0.555	0.087	2.01E-10	0.055
UC	STMN3	20	62271061	rs909333	62270637	G	T	0.207	0.498	0.098	4.04E-07	0.062
GORD	PBX2	6	32154964	rs693906	31835164	C	G	0.113	0.154	0.036	1.65E-05	0.058
VHD	AGER	6	32150423	rs693906	31835164	C	G	0.113	0.212	0.052	4.82E-05	0.092

B) Nominally significant effect on VHD of Bonferroni-significant gastrointestinal loci.

Disease	Gene	chr	Probe_bp	topSNP	topSNP_bp	A1	A2	Freq	b_SMR	se_SMR	p_SMR	p_HEIDI
VHD	CFDP1	16	75397489	rs7201989	75471499	G	A	0.246	-0.056	0.018	0.001	0.119
VHD	STAT3	17	40502964	rs1053004	40466092	G	A	0.380	0.093	0.047	0.048	0.948
VHD	IMPDH2	3	49064299	rs34037363	48821036	T	C	0.114	-0.209	0.105	0.047	0.089
VHD	UBE2L3	22	21941029	rs2070512	21949411	C	A	0.194	-0.029	0.009	0.002	0.623
VHD	STMN3	20	62271061	rs909333	62270637	G	T	0.207	-0.076	0.036	0.036	0.093

C) Effect of AGER gene on VHD and gastrointestinal disorders.

Disease	Gene	chr	Probe_bp	topSNP	topSNP_bp	A1	A2	Freq	b_SMR	se_SMR	p_SMR	p_HEIDI
CD	AGER	6	32150423	rs693906	31835164	C	G	0.113	-0.312	0.120	0.009	6.16E-08
GORD	AGER	6	32150423	rs693906	31835164	C	G	0.113	-0.209	0.049	2.02E-05	0.022
IBD	AGER	6	32148745	rs204995	32154285	G	A	0.217	-0.252	0.108	0.020	1.60E-04
IBS	AGER	6	32150423	rs693906	31835164	C	G	0.113	-0.168	0.052	0.001	0.045
UC	AGER	6	32148745	rs204995	32154285	G	A	0.217	-0.870	0.185	2.59E-06	0.002
VHD	AGER	6	32150423	rs693906	31835164	C	G	0.113	0.212	0.052	4.82E-05	0.092

Supplementary table 6. Results of the summary-based Mendelian randomization integrating whole-blood expression quantitative trait loci. For each disease-gene association, we report the strongest effect observed between GTEX and eQTLGen datasets.

A) Bonferroni-significant disease-gene association with HEIDI P > 0.05.

Disease	Gene	chr	gene_bp	topSNP	topSNP_bp	A1	A2	Freq	b_SMR	se_SMR	p_SMR	p_HEIDI
CD	DNMT3A	2	25510652	rs7578575	25488819	A	T	0.329	-0.834	0.164	3.51E-07	0.383
IBD	DNMT3A	2	25510652	rs7578575	25488819	A	T	0.329	-0.745	0.130	9.22E-09	0.515
UC	DNMT3A	2	25510652	rs7578575	25488819	A	T	0.329	-0.627	0.156	5.73E-05	0.541
UC	CFDP1	16	75327596	rs9934007	75390532	C	T	0.754	0.273	0.070	9.61E-05	0.973
CD	STAT3	17	40502964	rs1053004	40466092	G	A	0.380	0.702	0.128	4.40E-08	0.056
CD	IMPDH2	3	49064299	rs9813813	49207093	A	T	0.110	-1.233	0.294	2.74E-05	0.213
IBD	IMPDH2	3	49064299	rs9813813	49207093	A	T	0.110	-1.328	0.258	2.57E-07	0.071
UC	IMPDH2	3	49064299	rs9813813	49207093	A	T	0.110	-1.445	0.307	2.53E-06	0.050
IBD	UBE2L3	22	21941029	rs2070512	21949411	C	A	0.194	0.163	0.021	1.06E-14	0.169
UC	UBE2L3	22	21941029	rs2070512	21949411	C	A	0.194	0.116	0.027	1.76E-05	0.698
CD	BCL2L15	1	1.14E+08	rs1217393	1.14E+08	A	G	0.271	-0.705	0.170	3.39E-05	0.184
CD	STMN3	20	62271061	rs909333	62270637	G	T	0.207	0.547	0.102	8.31E-08	0.200
IBD	STMN3	20	62271061	rs909333	62270637	G	T	0.207	0.555	0.087	2.01E-10	0.055
UC	STMN3	20	62271061	rs909333	62270637	G	T	0.207	0.498	0.098	4.04E-07	0.062
GORD	PBX2	6	32154964	rs693906	31835164	C	G	0.113	0.154	0.036	1.65E-05	0.058
VHD	AGER	6	32150423	rs693906	31835164	C	G	0.113	0.212	0.052	4.82E-05	0.092

B) Nominally significant effect on VHD of Bonferroni-significant gastrointestinal loci.

Disease	Gene	chr	Probe_bp	topSNP	topSNP_bp	A1	A2	Freq	b_SMR	se_SMR	p_SMR	p_HEIDI
VHD	CFDP1	16	75397489	rs7201989	75471499	G	A	0.246	-0.056	0.018	0.001	0.119
VHD	STAT3	17	40502964	rs1053004	40466092	G	A	0.380	0.093	0.047	0.048	0.948
VHD	IMPDH2	3	49064299	rs34037363	48821036	T	C	0.114	-0.209	0.105	0.047	0.089
VHD	UBE2L3	22	21941029	rs2070512	21949411	C	A	0.194	-0.029	0.009	0.002	0.623
VHD	STMN3	20	62271061	rs909333	62270637	G	T	0.207	-0.076	0.036	0.036	0.093

C) Effect of AGER gene on VHD and gastrointestinal disorders.

Disease	Gene	chr	Probe_bp	topSNP	topSNP_bp	A1	A2	Freq	b_SMR	se_SMR	p_SMR	p_HEIDI
CD	AGER	6	32150423	rs693906	31835164	C	G	0.113	-0.312	0.120	0.009	6.16E-08
GORD	AGER	6	32150423	rs693906	31835164	C	G	0.113	-0.209	0.049	2.02E-05	0.022
IBD	AGER	6	32148745	rs204995	32154285	G	A	0.217	-0.252	0.108	0.020	1.60E-04
IBS	AGER	6	32150423	rs693906	31835164	C	G	0.113	-0.168	0.052	0.001	0.045
UC	AGER	6	32148745	rs204995	32154285	G	A	0.217	-0.870	0.185	2.59E-06	0.002
VHD	AGER	6	32150423	rs693906	31835164	C	G	0.113	0.212	0.052	4.82E-05	0.092

Supplementary table 6. Results of the summary-based Mendelian randomization integrating whole-blood expression quantitative trait loci. For each disease-gene association, we report the strongest effect observed between GTEX and eQTLGen datasets.

A) Bonferroni-significant disease-gene association with HEIDI P > 0.05.

Disease	Gene	chr	gene_bp	topSNP	topSNP_bp	A1	A2	Freq	b_SMR	se_SMR	p_SMR	p_HEIDI
CD	DNMT3A	2	25510652	rs7578575	25488819	A	T	0.329	-0.834	0.164	3.51E-07	0.383
IBD	DNMT3A	2	25510652	rs7578575	25488819	A	T	0.329	-0.745	0.130	9.22E-09	0.515
UC	DNMT3A	2	25510652	rs7578575	25488819	A	T	0.329	-0.627	0.156	5.73E-05	0.541
UC	CFDP1	16	75327596	rs9934007	75390532	C	T	0.754	0.273	0.070	9.61E-05	0.973
CD	STAT3	17	40502964	rs1053004	40466092	G	A	0.380	0.702	0.128	4.40E-08	0.056
CD	IMPDH2	3	49064299	rs9813813	49207093	A	T	0.110	-1.233	0.294	2.74E-05	0.213
IBD	IMPDH2	3	49064299	rs9813813	49207093	A	T	0.110	-1.328	0.258	2.57E-07	0.071
UC	IMPDH2	3	49064299	rs9813813	49207093	A	T	0.110	-1.445	0.307	2.53E-06	0.050
IBD	UBE2L3	22	21941029	rs2070512	21949411	C	A	0.194	0.163	0.021	1.06E-14	0.169
UC	UBE2L3	22	21941029	rs2070512	21949411	C	A	0.194	0.116	0.027	1.76E-05	0.698
CD	BCL2L15	1	1.14E+08	rs1217393	1.14E+08	A	G	0.271	-0.705	0.170	3.39E-05	0.184
CD	STMN3	20	62271061	rs909333	62270637	G	T	0.207	0.547	0.102	8.31E-08	0.200
IBD	STMN3	20	62271061	rs909333	62270637	G	T	0.207	0.555	0.087	2.01E-10	0.055
UC	STMN3	20	62271061	rs909333	62270637	G	T	0.207	0.498	0.098	4.04E-07	0.062
GORD	PBX2	6	32154964	rs693906	31835164	C	G	0.113	0.154	0.036	1.65E-05	0.058
VHD	AGER	6	32150423	rs693906	31835164	C	G	0.113	0.212	0.052	4.82E-05	0.092

B) Nominally significant effect on VHD of Bonferroni-significant gastrointestinal loci.

Disease	Gene	chr	Probe_bp	topSNP	topSNP_bp	A1	A2	Freq	b_SMR	se_SMR	p_SMR	p_HEIDI
VHD	CFDP1	16	75397489	rs7201989	75471499	G	A	0.246	-0.056	0.018	0.001	0.119
VHD	STAT3	17	40502964	rs1053004	40466092	G	A	0.380	0.093	0.047	0.048	0.948
VHD	IMPDH2	3	49064299	rs34037363	48821036	T	C	0.114	-0.209	0.105	0.047	0.089
VHD	UBE2L3	22	21941029	rs2070512	21949411	C	A	0.194	-0.029	0.009	0.002	0.623
VHD	STMN3	20	62271061	rs909333	62270637	G	T	0.207	-0.076	0.036	0.036	0.093

C) Effect of AGER gene on VHD and gastrointestinal disorders.

Disease	Gene	chr	Probe_bp	topSNP	topSNP_bp	A1	A2	Freq	b_SMR	se_SMR	p_SMR	p_HEIDI
CD	AGER	6	32150423	rs693906	31835164	C	G	0.113	-0.312	0.120	0.009	6.16E-08
GORD	AGER	6	32150423	rs693906	31835164	C	G	0.113	-0.209	0.049	2.02E-05	0.022
IBD	AGER	6	32148745	rs204995	32154285	G	A	0.217	-0.252	0.108	0.020	1.60E-04
IBS	AGER	6	32150423	rs693906	31835164	C	G	0.113	-0.168	0.052	0.001	0.045
UC	AGER	6	32148745	rs204995	32154285	G	A	0.217	-0.870	0.185	2.59E-06	0.002
VHD	AGER	6	32150423	rs693906	31835164	C	G	0.113	0.212	0.052	4.82E-05	0.092

Supplementary table 6. Results of the summary-based Mendelian randomization integrating whole-blood expression quantitative trait loci. For each disease-gene association, we report the strongest effect observed between GTEX and eQTLGen datasets.

A) Bonferroni-significant disease-gene association with HEIDI P > 0.05.

Disease	Gene	chr	gene_bp	topSNP	topSNP_bp	A1	A2	Freq	b_SMR	se_SMR	p_SMR	p_HEIDI
CD	DNMT3A	2	25510652	rs7578575	25488819	A	T	0.329	-0.834	0.164	3.51E-07	0.383
IBD	DNMT3A	2	25510652	rs7578575	25488819	A	T	0.329	-0.745	0.130	9.22E-09	0.515
UC	DNMT3A	2	25510652	rs7578575	25488819	A	T	0.329	-0.627	0.156	5.73E-05	0.541
UC	CFDP1	16	75327596	rs9934007	75390532	C	T	0.754	0.273	0.070	9.61E-05	0.973
CD	STAT3	17	40502964	rs1053004	40466092	G	A	0.380	0.702	0.128	4.40E-08	0.056
CD	IMPDH2	3	49064299	rs9813813	49207093	A	T	0.110	-1.233	0.294	2.74E-05	0.213
IBD	IMPDH2	3	49064299	rs9813813	49207093	A	T	0.110	-1.328	0.258	2.57E-07	0.071
UC	IMPDH2	3	49064299	rs9813813	49207093	A	T	0.110	-1.445	0.307	2.53E-06	0.050
IBD	UBE2L3	22	21941029	rs2070512	21949411	C	A	0.194	0.163	0.021	1.06E-14	0.169
UC	UBE2L3	22	21941029	rs2070512	21949411	C	A	0.194	0.116	0.027	1.76E-05	0.698
CD	BCL2L15	1	1.14E+08	rs1217393	1.14E+08	A	G	0.271	-0.705	0.170	3.39E-05	0.184
CD	STMN3	20	62271061	rs909333	62270637	G	T	0.207	0.547	0.102	8.31E-08	0.200
IBD	STMN3	20	62271061	rs909333	62270637	G	T	0.207	0.555	0.087	2.01E-10	0.055
UC	STMN3	20	62271061	rs909333	62270637	G	T	0.207	0.498	0.098	4.04E-07	0.062
GORD	PBX2	6	32154964	rs693906	31835164	C	G	0.113	0.154	0.036	1.65E-05	0.058
VHD	AGER	6	32150423	rs693906	31835164	C	G	0.113	0.212	0.052	4.82E-05	0.092

B) Nominally significant effect on VHD of Bonferroni-significant gastrointestinal loci.

Disease	Gene	chr	Probe_bp	topSNP	topSNP_bp	A1	A2	Freq	b_SMR	se_SMR	p_SMR	p_HEIDI
VHD	CFDP1	16	75397489	rs7201989	75471499	G	A	0.246	-0.056	0.018	0.001	0.119
VHD	STAT3	17	40502964	rs1053004	40466092	G	A	0.380	0.093	0.047	0.048	0.948
VHD	IMPDH2	3	49064299	rs34037363	48821036	T	C	0.114	-0.209	0.105	0.047	0.089
VHD	UBE2L3	22	21941029	rs2070512	21949411	C	A	0.194	-0.029	0.009	0.002	0.623
VHD	STMN3	20	62271061	rs909333	62270637	G	T	0.207	-0.076	0.036	0.036	0.093

C) Effect of AGER gene on VHD and gastrointestinal disorders.

Disease	Gene	chr	Probe_bp	topSNP	topSNP_bp	A1	A2	Freq	b_SMR	se_SMR	p_SMR	p_HEIDI
CD	AGER	6	32150423	rs693906	31835164	C	G	0.113	-0.312	0.120	0.009	6.16E-08
GORD	AGER	6	32150423	rs693906	31835164	C	G	0.113	-0.209	0.049	2.02E-05	0.022
IBD	AGER	6	32148745	rs204995	32154285	G	A	0.217	-0.252	0.108	0.020	1.60E-04
IBS	AGER	6	32150423	rs693906	31835164	C	G	0.113	-0.168	0.052	0.001	0.045
UC	AGER	6	32148745	rs204995	32154285	G	A	0.217	-0.870	0.185	2.59E-06	0.002
VHD	AGER	6	32150423	rs693906	31835164	C	G	0.113	0.212	0.052	4.82E-05	0.092

Supplementary table 6. Results of the summary-based Mendelian randomization integrating whole-blood expression quantitative trait loci. For each disease-gene association, we report the strongest effect observed between GTEX and eQTLGen datasets.

A) Bonferroni-significant disease-gene association with HEIDI P > 0.05.

Disease	Gene	chr	gene_bp	topSNP	topSNP_bp	A1	A2	Freq	b_SMR	se_SMR	p_SMR	p_HEIDI
CD	DNMT3A	2	25510652	rs7578575	25488819	A	T	0.329	-0.834	0.164	3.51E-07	0.383
IBD	DNMT3A	2	25510652	rs7578575	25488819	A	T	0.329	-0.745	0.130	9.22E-09	0.515
UC	DNMT3A	2	25510652	rs7578575	25488819	A	T	0.329	-0.627	0.156	5.73E-05	0.541
UC	CFDP1	16	75327596	rs9934007	75390532	C	T	0.754	0.273	0.070	9.61E-05	0.973
CD	STAT3	17	40502964	rs1053004	40466092	G	A	0.380	0.702	0.128	4.40E-08	0.056
CD	IMPDH2	3	49064299	rs9813813	49207093	A	T	0.110	-1.233	0.294	2.74E-05	0.213
IBD	IMPDH2	3	49064299	rs9813813	49207093	A	T	0.110	-1.328	0.258	2.57E-07	0.071
UC	IMPDH2	3	49064299	rs9813813	49207093	A	T	0.110	-1.445	0.307	2.53E-06	0.050
IBD	UBE2L3	22	21941029	rs2070512	21949411	C	A	0.194	0.163	0.021	1.06E-14	0.169
UC	UBE2L3	22	21941029	rs2070512	21949411	C	A	0.194	0.116	0.027	1.76E-05	0.698
CD	BCL2L15	1	1.14E+08	rs1217393	1.14E+08	A	G	0.271	-0.705	0.170	3.39E-05	0.184
CD	STMN3	20	62271061	rs909333	62270637	G	T	0.207	0.547	0.102	8.31E-08	0.200
IBD	STMN3	20	62271061	rs909333	62270637	G	T	0.207	0.555	0.087	2.01E-10	0.055
UC	STMN3	20	62271061	rs909333	62270637	G	T	0.207	0.498	0.098	4.04E-07	0.062
GORD	PBX2	6	32154964	rs693906	31835164	C	G	0.113	0.154	0.036	1.65E-05	0.058
VHD	AGER	6	32150423	rs693906	31835164	C	G	0.113	0.212	0.052	4.82E-05	0.092

B) Nominally significant effect on VHD of Bonferroni-significant gastrointestinal loci.

Disease	Gene	chr	Probe_bp	topSNP	topSNP_bp	A1	A2	Freq	b_SMR	se_SMR	p_SMR	p_HEIDI
VHD	CFDP1	16	75397489	rs7201989	75471499	G	A	0.246	-0.056	0.018	0.001	0.119
VHD	STAT3	17	40502964	rs1053004	40466092	G	A	0.380	0.093	0.047	0.048	0.948
VHD	IMPDH2	3	49064299	rs34037363	48821036	T	C	0.114	-0.209	0.105	0.047	0.089
VHD	UBE2L3	22	21941029	rs2070512	21949411	C	A	0.194	-0.029	0.009	0.002	0.623
VHD	STMN3	20	62271061	rs909333	62270637	G	T	0.207	-0.076	0.036	0.036	0.093

C) Effect of AGER gene on VHD and gastrointestinal disorders.

Disease	Gene	chr	Probe_bp	topSNP	topSNP_bp	A1	A2	Freq	b_SMR	se_SMR	p_SMR	p_HEIDI
CD	AGER	6	32150423	rs693906	31835164	C	G	0.113	-0.312	0.120	0.009	6.16E-08
GORD	AGER	6	32150423	rs693906	31835164	C	G	0.113	-0.209	0.049	2.02E-05	0.022
IBD	AGER	6	32148745	rs204995	32154285	G	A	0.217	-0.252	0.108	0.020	1.60E-04
IBS	AGER	6	32150423	rs693906	31835164	C	G	0.113	-0.168	0.052	0.001	0.045
UC	AGER	6	32148745	rs204995	32154285	G	A	0.217	-0.870	0.185	2.59E-06	0.002
VHD	AGER	6	32150423	rs693906	31835164	C	G	0.113	0.212	0.052	4.82E-05	0.092

Supplementary table 6. Results of the summary-based Mendelian randomization integrating whole-blood expression quantitative trait loci. For each disease-gene association, we report the strongest effect observed between GTEX and eQTLGen datasets.

A) Bonferroni-significant disease-gene association with HEIDI P > 0.05.

Disease	Gene	chr	gene_bp	topSNP	topSNP_bp	A1	A2	Freq	b_SMR	se_SMR	p_SMR	p_HEIDI
CD	DNMT3A	2	25510652	rs7578575	25488819	A	T	0.329	-0.834	0.164	3.51E-07	0.383
IBD	DNMT3A	2	25510652	rs7578575	25488819	A	T	0.329	-0.745	0.130	9.22E-09	0.515
UC	DNMT3A	2	25510652	rs7578575	25488819	A	T	0.329	-0.627	0.156	5.73E-05	0.541
UC	CFDP1	16	75327596	rs9934007	75390532	C	T	0.754	0.273	0.070	9.61E-05	0.973
CD	STAT3	17	40502964	rs1053004	40466092	G	A	0.380	0.702	0.128	4.40E-08	0.056
CD	IMPDH2	3	49064299	rs9813813	49207093	A	T	0.110	-1.233	0.294	2.74E-05	0.213
IBD	IMPDH2	3	49064299	rs9813813	49207093	A	T	0.110	-1.328	0.258	2.57E-07	0.071
UC	IMPDH2	3	49064299	rs9813813	49207093	A	T	0.110	-1.445	0.307	2.53E-06	0.050
IBD	UBE2L3	22	21941029	rs2070512	21949411	C	A	0.194	0.163	0.021	1.06E-14	0.169
UC	UBE2L3	22	21941029	rs2070512	21949411	C	A	0.194	0.116	0.027	1.76E-05	0.698
CD	BCL2L15	1	1.14E+08	rs1217393	1.14E+08	A	G	0.271	-0.705	0.170	3.39E-05	0.184
CD	STMN3	20	62271061	rs909333	62270637	G	T	0.207	0.547	0.102	8.31E-08	0.200
IBD	STMN3	20	62271061	rs909333	62270637	G	T	0.207	0.555	0.087	2.01E-10	0.055
UC	STMN3	20	62271061	rs909333	62270637	G	T	0.207	0.498	0.098	4.04E-07	0.062
GORD	PBX2	6	32154964	rs693906	31835164	C	G	0.113	0.154	0.036	1.65E-05	0.058
VHD	AGER	6	32150423	rs693906	31835164	C	G	0.113	0.212	0.052	4.82E-05	0.092

B) Nominally significant effect on VHD of Bonferroni-significant gastrointestinal loci.

Disease	Gene	chr	Probe_bp	topSNP	topSNP_bp	A1	A2	Freq	b_SMR	se_SMR	p_SMR	p_HEIDI
VHD	CFDP1	16	75397489	rs7201989	75471499	G	A	0.246	-0.056	0.018	0.001	0.119
VHD	STAT3	17	40502964	rs1053004	40466092	G	A	0.380	0.093	0.047	0.048	0.948
VHD	IMPDH2	3	49064299	rs34037363	48821036	T	C	0.114	-0.209	0.105	0.047	0.089
VHD	UBE2L3	22	21941029	rs2070512	21949411	C	A	0.194	-0.029	0.009	0.002	0.623
VHD	STMN3	20	62271061	rs909333	62270637	G	T	0.207	-0.076	0.036	0.036	0.093

C) Effect of AGER gene on VHD and gastrointestinal disorders.

Disease	Gene	chr	Probe_bp	topSNP	topSNP_bp	A1	A2	Freq	b_SMR	se_SMR	p_SMR	p_HEIDI
CD	AGER	6	32150423	rs693906	31835164	C	G	0.113	-0.312	0.120	0.009	6.16E-08
GORD	AGER	6	32150423	rs693906	31835164	C	G	0.113	-0.209	0.049	2.02E-05	0.022
IBD	AGER	6	32148745	rs204995	32154285	G	A	0.217	-0.252	0.108	0.020	1.60E-04
IBS	AGER	6	32150423	rs693906	31835164	C	G	0.113	-0.168	0.052	0.001	0.045
UC	AGER	6	32148745	rs204995	32154285	G	A	0.217	-0.870	0.185	2.59E-06	0.002
VHD	AGER	6	32150423	rs693906	31835164	C	G	0.113	0.212	0.052	4.82E-05	0.092

Supplementary table 6. Results of the summary-based Mendelian randomization integrating whole-blood expression quantitative trait loci. For each disease-gene association, we report the strongest effect observed between GTEX and eQTLGen datasets.

A) Bonferroni-significant disease-gene association with HEIDI P > 0.05.

Disease	Gene	chr	gene_bp	topSNP	topSNP_bp	A1	A2	Freq	b_SMR	se_SMR	p_SMR	p_HEIDI
CD	DNMT3A	2	25510652	rs7578575	25488819	A	T	0.329	-0.834	0.164	3.51E-07	0.383
IBD	DNMT3A	2	25510652	rs7578575	25488819	A	T	0.329	-0.745	0.130	9.22E-09	0.515
UC	DNMT3A	2	25510652	rs7578575	25488819	A	T	0.329	-0.627	0.156	5.73E-05	0.541
UC	CFDP1	16	75327596	rs9934007	75390532	C	T	0.754	0.273	0.070	9.61E-05	0.973
CD	STAT3	17	40502964	rs1053004	40466092	G	A	0.380	0.702	0.128	4.40E-08	0.056
CD	IMPDH2	3	49064299	rs9813813	49207093	A	T	0.110	-1.233	0.294	2.74E-05	0.213
IBD	IMPDH2	3	49064299	rs9813813	49207093	A	T	0.110	-1.328	0.258	2.57E-07	0.071
UC	IMPDH2	3	49064299	rs9813813	49207093	A	T	0.110	-1.445	0.307	2.53E-06	0.050
IBD	UBE2L3	22	21941029	rs2070512	21949411	C	A	0.194	0.163	0.021	1.06E-14	0.169
UC	UBE2L3	22	21941029	rs2070512	21949411	C	A	0.194	0.116	0.027	1.76E-05	0.698
CD	BCL2L15	1	1.14E+08	rs1217393	1.14E+08	A	G	0.271	-0.705	0.170	3.39E-05	0.184
CD	STMN3	20	62271061	rs909333	62270637	G	T	0.207	0.547	0.102	8.31E-08	0.200
IBD	STMN3	20	62271061	rs909333	62270637	G	T	0.207	0.555	0.087	2.01E-10	0.055
UC	STMN3	20	62271061	rs909333	62270637	G	T	0.207	0.498	0.098	4.04E-07	0.062
GORD	PBX2	6	32154964	rs693906	31835164	C	G	0.113	0.154	0.036	1.65E-05	0.058
VHD	AGER	6	32150423	rs693906	31835164	C	G	0.113	0.212	0.052	4.82E-05	0.092

B) Nominally significant effect on VHD of Bonferroni-significant gastrointestinal loci.

Disease	Gene	chr	Probe_bp	topSNP	topSNP_bp	A1	A2	Freq	b_SMR	se_SMR	p_SMR	p_HEIDI
VHD	CFDP1	16	75397489	rs7201989	75471499	G	A	0.246	-0.056	0.018	0.001	0.119
VHD	STAT3	17	40502964	rs1053004	40466092	G	A	0.380	0.093	0.047	0.048	0.948
VHD	IMPDH2	3	49064299	rs34037363	48821036	T	C	0.114	-0.209	0.105	0.047	0.089
VHD	UBE2L3	22	21941029	rs2070512	21949411	C	A	0.194	-0.029	0.009	0.002	0.623
VHD	STMN3	20	62271061	rs909333	62270637	G	T	0.207	-0.076	0.036	0.036	0.093

C) Effect of AGER gene on VHD and gastrointestinal disorders.

Disease	Gene	chr	Probe_bp	topSNP	topSNP_bp	A1	A2	Freq	b_SMR	se_SMR	p_SMR	p_HEIDI
CD	AGER	6	32150423	rs693906	31835164	C	G	0.113	-0.312	0.120	0.009	6.16E-08
GORD	AGER	6	32150423	rs693906	31835164	C	G	0.113	-0.209	0.049	2.02E-05	0.022
IBD	AGER	6	32148745	rs204995	32154285	G	A	0.217	-0.252	0.108	0.020	1.60E-04
IBS	AGER	6	32150423	rs693906	31835164	C	G	0.113	-0.168	0.052	0.001	0.045
UC	AGER	6	32148745	rs204995	32154285	G	A	0.217	-0.870	0.185	2.59E-06	0.002
VHD	AGER	6	32150423	rs693906	31835164	C	G	0.113	0.212	0.052	4.82E-05	0.092

Supplementary table 6. Results of the summary-based Mendelian randomization integrating whole-blood expression quantitative trait loci. For each disease-gene association, we report the strongest effect observed between GTEX and eQTLGen datasets.

A) Bonferroni-significant disease-gene association with HEIDI P > 0.05.

Disease	Gene	chr	gene_bp	topSNP	topSNP_bp	A1	A2	Freq	b_SMR	se_SMR	p_SMR	p_HEIDI
CD	DNMT3A	2	25510652	rs7578575	25488819	A	T	0.329	-0.834	0.164	3.51E-07	0.383
IBD	DNMT3A	2	25510652	rs7578575	25488819	A	T	0.329	-0.745	0.130	9.22E-09	0.515
UC	DNMT3A	2	25510652	rs7578575	25488819	A	T	0.329	-0.627	0.156	5.73E-05	0.541
UC	CFDP1	16	75327596	rs9934007	75390532	C	T	0.754	0.273	0.070	9.61E-05	0.973
CD	STAT3	17	40502964	rs1053004	40466092	G	A	0.380	0.702	0.128	4.40E-08	0.056
CD	IMPDH2	3	49064299	rs9813813	49207093	A	T	0.110	-1.233	0.294	2.74E-05	0.213
IBD	IMPDH2	3	49064299	rs9813813	49207093	A	T	0.110	-1.328	0.258	2.57E-07	0.071
UC	IMPDH2	3	49064299	rs9813813	49207093	A	T	0.110	-1.445	0.307	2.53E-06	0.050
IBD	UBE2L3	22	21941029	rs2070512	21949411	C	A	0.194	0.163	0.021	1.06E-14	0.169
UC	UBE2L3	22	21941029	rs2070512	21949411	C	A	0.194	0.116	0.027	1.76E-05	0.698
CD	BCL2L15	1	1.14E+08	rs1217393	1.14E+08	A	G	0.271	-0.705	0.170	3.39E-05	0.184
CD	STMN3	20	62271061	rs909333	62270637	G	T	0.207	0.547	0.102	8.31E-08	0.200
IBD	STMN3	20	62271061	rs909333	62270637	G	T	0.207	0.555	0.087	2.01E-10	0.055
UC	STMN3	20	62271061	rs909333	62270637	G	T	0.207	0.498	0.098	4.04E-07	0.062
GORD	PBX2	6	32154964	rs693906	31835164	C	G	0.113	0.154	0.036	1.65E-05	0.058
VHD	AGER	6	32150423	rs693906	31835164	C	G	0.113	0.212	0.052	4.82E-05	0.092

B) Nominally significant effect on VHD of Bonferroni-significant gastrointestinal loci.

Disease	Gene	chr	Probe_bp	topSNP	topSNP_bp	A1	A2	Freq	b_SMR	se_SMR	p_SMR	p_HEIDI
VHD	CFDP1	16	75397489	rs7201989	75471499	G	A	0.246	-0.056	0.018	0.001	0.119
VHD	STAT3	17	40502964	rs1053004	40466092	G	A	0.380	0.093	0.047	0.048	0.948
VHD	IMPDH2	3	49064299	rs34037363	48821036	T	C	0.114	-0.209	0.105	0.047	0.089
VHD	UBE2L3	22	21941029	rs2070512	21949411	C	A	0.194	-0.029	0.009	0.002	0.623
VHD	STMN3	20	62271061	rs909333	62270637	G	T	0.207	-0.076	0.036	0.036	0.093

C) Effect of AGER gene on VHD and gastrointestinal disorders.

Disease	Gene	chr	Probe_bp	topSNP	topSNP_bp	A1	A2	Freq	b_SMR	se_SMR	p_SMR	p_HEIDI
CD	AGER	6	32150423	rs693906	31835164	C	G	0.113	-0.312	0.120	0.009	6.16E-08
GORD	AGER	6	32150423	rs693906	31835164	C	G	0.113	-0.209	0.049	2.02E-05	0.022
IBD	AGER	6	32148745	rs204995	32154285	G	A	0.217	-0.252	0.108	0.020	1.60E-04
IBS	AGER	6	32150423	rs693906	31835164	C	G	0.113	-0.168	0.052	0.001	0.045
UC	AGER	6	32148745	rs204995	32154285	G	A	0.217	-0.870	0.185	2.59E-06	0.002
VHD	AGER	6	32150423	rs693906	31835164	C	G	0.113	0.212	0.052	4.82E-05	0.092

Supplementary table 6. Results of the summary-based Mendelian randomization integrating whole-blood expression quantitative trait loci. For each disease-gene association, we report the strongest effect observed between GTEX and eQTLGen datasets.

A) Bonferroni-significant disease-gene association with HEIDI P > 0.05.

Disease	Gene	chr	gene_bp	topSNP	topSNP_bp	A1	A2	Freq	b_SMR	se_SMR	p_SMR	p_HEIDI
CD	DNMT3A	2	25510652	rs7578575	25488819	A	T	0.329	-0.834	0.164	3.51E-07	0.383
IBD	DNMT3A	2	25510652	rs7578575	25488819	A	T	0.329	-0.745	0.130	9.22E-09	0.515
UC	DNMT3A	2	25510652	rs7578575	25488819	A	T	0.329	-0.627	0.156	5.73E-05	0.541
UC	CFDP1	16	75327596	rs9934007	75390532	C	T	0.754	0.273	0.070	9.61E-05	0.973
CD	STAT3	17	40502964	rs1053004	40466092	G	A	0.380	0.702	0.128	4.40E-08	0.056
CD	IMPDH2	3	49064299	rs9813813	49207093	A	T	0.110	-1.233	0.294	2.74E-05	0.213
IBD	IMPDH2	3	49064299	rs9813813	49207093	A	T	0.110	-1.328	0.258	2.57E-07	0.071
UC	IMPDH2	3	49064299	rs9813813	49207093	A	T	0.110	-1.445	0.307	2.53E-06	0.050
IBD	UBE2L3	22	21941029	rs2070512	21949411	C	A	0.194	0.163	0.021	1.06E-14	0.169
UC	UBE2L3	22	21941029	rs2070512	21949411	C	A	0.194	0.116	0.027	1.76E-05	0.698
CD	BCL2L15	1	1.14E+08	rs1217393	1.14E+08	A	G	0.271	-0.705	0.170	3.39E-05	0.184
CD	STMN3	20	62271061	rs909333	62270637	G	T	0.207	0.547	0.102	8.31E-08	0.200
IBD	STMN3	20	62271061	rs909333	62270637	G	T	0.207	0.555	0.087	2.01E-10	0.055
UC	STMN3	20	62271061	rs909333	62270637	G	T	0.207	0.498	0.098	4.04E-07	0.062
GORD	PBX2	6	32154964	rs693906	31835164	C	G	0.113	0.154	0.036	1.65E-05	0.058
VHD	AGER	6	32150423	rs693906	31835164	C	G	0.113	0.212	0.052	4.82E-05	0.092

B) Nominally significant effect on VHD of Bonferroni-significant gastrointestinal loci.

Disease	Gene	chr	Probe_bp	topSNP	topSNP_bp	A1	A2	Freq	b_SMR	se_SMR	p_SMR	p_HEIDI
VHD	CFDP1	16	75397489	rs7201989	75471499	G	A	0.246	-0.056	0.018	0.001	0.119
VHD	STAT3	17	40502964	rs1053004	40466092	G	A	0.380	0.093	0.047	0.048	0.948
VHD	IMPDH2	3	49064299	rs34037363	48821036	T	C	0.114	-0.209	0.105	0.047	0.089
VHD	UBE2L3	22	21941029	rs2070512	21949411	C	A	0.194	-0.029	0.009	0.002	0.623
VHD	STMN3	20	62271061	rs909333	62270637	G	T	0.207	-0.076	0.036	0.036	0.093

C) Effect of AGER gene on VHD and gastrointestinal disorders.

Disease	Gene	chr	Probe_bp	topSNP	topSNP_bp	A1	A2	Freq	b_SMR	se_SMR	p_SMR	p_HEIDI
CD	AGER	6	32150423	rs693906	31835164	C	G	0.113	-0.312	0.120	0.009	6.16E-08
GORD	AGER	6	32150423	rs693906	31835164	C	G	0.113	-0.209	0.049	2.02E-05	0.022
IBD	AGER	6	32148745	rs204995	32154285	G	A	0.217	-0.252	0.108	0.020	1.60E-04
IBS	AGER	6	32150423	rs693906	31835164	C	G	0.113	-0.168	0.052	0.001	0.045
UC	AGER	6	32148745	rs204995	32154285	G	A	0.217	-0.870	0.185	2.59E-06	0.002
VHD	AGER	6	32150423	rs693906	31835164	C	G	0.113	0.212	0.052	4.82E-05	0.092

Supplementary table 6. Results of the summary-based Mendelian randomization integrating whole-blood expression quantitative trait loci. For each disease-gene association, we report the strongest effect observed between GTEX and eQTLGen datasets.

A) Bonferroni-significant disease-gene association with HEIDI P > 0.05.

Disease	Gene	chr	gene_bp	topSNP	topSNP_bp	A1	A2	Freq	b_SMR	se_SMR	p_SMR	p_HEIDI
CD	DNMT3A	2	25510652	rs7578575	25488819	A	T	0.329	-0.834	0.164	3.51E-07	0.383
IBD	DNMT3A	2	25510652	rs7578575	25488819	A	T	0.329	-0.745	0.130	9.22E-09	0.515
UC	DNMT3A	2	25510652	rs7578575	25488819	A	T	0.329	-0.627	0.156	5.73E-05	0.541
UC	CFDP1	16	75327596	rs9934007	75390532	C	T	0.754	0.273	0.070	9.61E-05	0.973
CD	STAT3	17	40502964	rs1053004	40466092	G	A	0.380	0.702	0.128	4.40E-08	0.056
CD	IMPDH2	3	49064299	rs9813813	49207093	A	T	0.110	-1.233	0.294	2.74E-05	0.213
IBD	IMPDH2	3	49064299	rs9813813	49207093	A	T	0.110	-1.328	0.258	2.57E-07	0.071
UC	IMPDH2	3	49064299	rs9813813	49207093	A	T	0.110	-1.445	0.307	2.53E-06	0.050
IBD	UBE2L3	22	21941029	rs2070512	21949411	C	A	0.194	0.163	0.021	1.06E-14	0.169
UC	UBE2L3	22	21941029	rs2070512	21949411	C	A	0.194	0.116	0.027	1.76E-05	0.698
CD	BCL2L15	1	1.14E+08	rs1217393	1.14E+08	A	G	0.271	-0.705	0.170	3.39E-05	0.184
CD	STMN3	20	62271061	rs909333	62270637	G	T	0.207	0.547	0.102	8.31E-08	0.200
IBD	STMN3	20	62271061	rs909333	62270637	G	T	0.207	0.555	0.087	2.01E-10	0.055
UC	STMN3	20	62271061	rs909333	62270637	G	T	0.207	0.498	0.098	4.04E-07	0.062
GORD	PBX2	6	32154964	rs693906	31835164	C	G	0.113	0.154	0.036	1.65E-05	0.058
VHD	AGER	6	32150423	rs693906	31835164	C	G	0.113	0.212	0.052	4.82E-05	0.092

B) Nominally significant effect on VHD of Bonferroni-significant gastrointestinal loci.

Disease	Gene	chr	Probe_bp	topSNP	topSNP_bp	A1	A2	Freq	b_SMR	se_SMR	p_SMR	p_HEIDI
VHD	CFDP1	16	75397489	rs7201989	75471499	G	A	0.246	-0.056	0.018	0.001	0.119
VHD	STAT3	17	40502964	rs1053004	40466092	G	A	0.380	0.093	0.047	0.048	0.948
VHD	IMPDH2	3	49064299	rs34037363	48821036	T	C	0.114	-0.209	0.105	0.047	0.089
VHD	UBE2L3	22	21941029	rs2070512	21949411	C	A	0.194	-0.029	0.009	0.002	0.623
VHD	STMN3	20	62271061	rs909333	62270637	G	T	0.207	-0.076	0.036	0.036	0.093

C) Effect of AGER gene on VHD and gastrointestinal disorders.

Disease	Gene	chr	Probe_bp	topSNP	topSNP_bp	A1	A2	Freq	b_SMR	se_SMR	p_SMR	p_HEIDI
CD	AGER	6	32150423	rs693906	31835164	C	G	0.113	-0.312	0.120	0.009	6.16E-08
GORD	AGER	6	32150423	rs693906	31835164	C	G	0.113	-0.209	0.049	2.02E-05	0.022
IBD	AGER	6	32148745	rs204995	32154285	G	A	0.217	-0.252	0.108	0.020	1.60E-04
IBS	AGER	6	32150423	rs693906	31835164	C	G	0.113	-0.168	0.052	0.001	0.045
UC	AGER	6	32148745	rs204995	32154285	G	A	0.217	-0.870	0.185	2.59E-06	0.002
VHD	AGER	6	32150423	rs693906	31835164	C	G	0.113	0.212	0.052	4.82E-05	0.092

Supplementary table 6. Results of the summary-based Mendelian randomization integrating whole-blood expression quantitative trait loci. For each disease-gene association, we report the strongest effect observed between GTEX and eQTLGen datasets.

A) Bonferroni-significant disease-gene association with HEIDI P > 0.05.

Disease	Gene	chr	gene_bp	topSNP	topSNP_bp	A1	A2	Freq	b_SMR	se_SMR	p_SMR	p_HEIDI
CD	DNMT3A	2	25510652	rs7578575	25488819	A	T	0.329	-0.834	0.164	3.51E-07	0.383
IBD	DNMT3A	2	25510652	rs7578575	25488819	A	T	0.329	-0.745	0.130	9.22E-09	0.515
UC	DNMT3A	2	25510652	rs7578575	25488819	A	T	0.329	-0.627	0.156	5.73E-05	0.541
UC	CFDP1	16	75327596	rs9934007	75390532	C	T	0.754	0.273	0.070	9.61E-05	0.973
CD	STAT3	17	40502964	rs1053004	40466092	G	A	0.380	0.702	0.128	4.40E-08	0.056
CD	IMPDH2	3	49064299	rs9813813	49207093	A	T	0.110	-1.233	0.294	2.74E-05	0.213
IBD	IMPDH2	3	49064299	rs9813813	49207093	A	T	0.110	-1.328	0.258	2.57E-07	0.071
UC	IMPDH2	3	49064299	rs9813813	49207093	A	T	0.110	-1.445	0.307	2.53E-06	0.050
IBD	UBE2L3	22	21941029	rs2070512	21949411	C	A	0.194	0.163	0.021	1.06E-14	0.169
UC	UBE2L3	22	21941029	rs2070512	21949411	C	A	0.194	0.116	0.027	1.76E-05	0.698
CD	BCL2L15	1	1.14E+08	rs1217393	1.14E+08	A	G	0.271	-0.705	0.170	3.39E-05	0.184
CD	STMN3	20	62271061	rs909333	62270637	G	T	0.207	0.547	0.102	8.31E-08	0.200
IBD	STMN3	20	62271061	rs909333	62270637	G	T	0.207	0.555	0.087	2.01E-10	0.055
UC	STMN3	20	62271061	rs909333	62270637	G	T	0.207	0.498	0.098	4.04E-07	0.062
GORD	PBX2	6	32154964	rs693906	31835164	C	G	0.113	0.154	0.036	1.65E-05	0.058
VHD	AGER	6	32150423	rs693906	31835164	C	G	0.113	0.212	0.052	4.82E-05	0.092

B) Nominally significant effect on VHD of Bonferroni-significant gastrointestinal loci.

Disease	Gene	chr	Probe_bp	topSNP	topSNP_bp	A1	A2	Freq	b_SMR	se_SMR	p_SMR	p_HEIDI
VHD	CFDP1	16	75397489	rs7201989	75471499	G	A	0.246	-0.056	0.018	0.001	0.119
VHD	STAT3	17	40502964	rs1053004	40466092	G	A	0.380	0.093	0.047	0.048	0.948
VHD	IMPDH2	3	49064299	rs34037363	48821036	T	C	0.114	-0.209	0.105	0.047	0.089
VHD	UBE2L3	22	21941029	rs2070512	21949411	C	A	0.194	-0.029	0.009	0.002	0.623
VHD	STMN3	20	62271061	rs909333	62270637	G	T	0.207	-0.076	0.036	0.036	0.093

C) Effect of AGER gene on VHD and gastrointestinal disorders.

Disease	Gene	chr	Probe_bp	topSNP	topSNP_bp	A1	A2	Freq	b_SMR	se_SMR	p_SMR	p_HEIDI
CD	AGER	6	32150423	rs693906	31835164	C	G	0.113	-0.312	0.120	0.009	6.16E-08
GORD	AGER	6	32150423	rs693906	31835164	C	G	0.113	-0.209	0.049	2.02E-05	0.022
IBD	AGER	6	32148745	rs204995	32154285	G	A	0.217	-0.252	0.108	0.020	1.60E-04
IBS	AGER	6	32150423	rs693906	31835164	C	G	0.113	-0.168	0.052	0.001	0.045
UC	AGER	6	32148745	rs204995	32154285	G	A	0.217	-0.870	0.185	2.59E-06	0.002
VHD	AGER	6	32150423	rs693906	31835164	C	G	0.113	0.212	0.052	4.82E-05	0.092

Supplementary table 6. Results of the summary-based Mendelian randomization integrating whole-blood expression quantitative trait loci. For each disease-gene association, we report the strongest effect observed between GTEX and eQTLGen datasets.

A) Bonferroni-significant disease-gene association with HEIDI P > 0.05.

Disease	Gene	chr	gene_bp	topSNP	topSNP_bp	A1	A2	Freq	b_SMR	se_SMR	p_SMR	p_HEIDI
CD	DNMT3A	2	25510652	rs7578575	25488819	A	T	0.329	-0.834	0.164	3.51E-07	0.383
IBD	DNMT3A	2	25510652	rs7578575	25488819	A	T	0.329	-0.745	0.130	9.22E-09	0.515
UC	DNMT3A	2	25510652	rs7578575	25488819	A	T	0.329	-0.627	0.156	5.73E-05	0.541
UC	CFDP1	16	75327596	rs9934007	75390532	C	T	0.754	0.273	0.070	9.61E-05	0.973
CD	STAT3	17	40502964	rs1053004	40466092	G	A	0.380	0.702	0.128	4.40E-08	0.056
CD	IMPDH2	3	49064299	rs9813813	49207093	A	T	0.110	-1.233	0.294	2.74E-05	0.213
IBD	IMPDH2	3	49064299	rs9813813	49207093	A	T	0.110	-1.328	0.258	2.57E-07	0.071
UC	IMPDH2	3	49064299	rs9813813	49207093	A	T	0.110	-1.445	0.307	2.53E-06	0.050
IBD	UBE2L3	22	21941029	rs2070512	21949411	C	A	0.194	0.163	0.021	1.06E-14	0.169
UC	UBE2L3	22	21941029	rs2070512	21949411	C	A	0.194	0.116	0.027	1.76E-05	0.698
CD	BCL2L15	1	1.14E+08	rs1217393	1.14E+08	A	G	0.271	-0.705	0.170	3.39E-05	0.184
CD	STMN3	20	62271061	rs909333	62270637	G	T	0.207	0.547	0.102	8.31E-08	0.200
IBD	STMN3	20	62271061	rs909333	62270637	G	T	0.207	0.555	0.087	2.01E-10	0.055
UC	STMN3	20	62271061	rs909333	62270637	G	T	0.207	0.498	0.098	4.04E-07	0.062
GORD	PBX2	6	32154964	rs693906	31835164	C	G	0.113	0.154	0.036	1.65E-05	0.058
VHD	AGER	6	32150423	rs693906	31835164	C	G	0.113	0.212	0.052	4.82E-05	0.092

B) Nominally significant effect on VHD of Bonferroni-significant gastrointestinal loci.

Disease	Gene	chr	Probe_bp	topSNP	topSNP_bp	A1	A2	Freq	b_SMR	se_SMR	p_SMR	p_HEIDI
VHD	CFDP1	16	75397489	rs7201989	75471499	G	A	0.246	-0.056	0.018	0.001	0.119
VHD	STAT3	17	40502964	rs1053004	40466092	G	A	0.380	0.093	0.047	0.048	0.948
VHD	IMPDH2	3	49064299	rs34037363	48821036	T	C	0.114	-0.209	0.105	0.047	0.089
VHD	UBE2L3	22	21941029	rs2070512	21949411	C	A	0.194	-0.029	0.009	0.002	0.623
VHD	STMN3	20	62271061	rs909333	62270637	G	T	0.207	-0.076	0.036	0.036	0.093

C) Effect of AGER gene on VHD and gastrointestinal disorders.

Disease	Gene	chr	Probe_bp	topSNP	topSNP_bp	A1	A2	Freq	b_SMR	se_SMR	p_SMR	p_HEIDI
CD	AGER	6	32150423	rs693906	31835164	C	G	0.113	-0.312	0.120	0.009	6.16E-08
GORD	AGER	6	32150423	rs693906	31835164	C	G	0.113	-0.209	0.049	2.02E-05	0.022
IBD	AGER	6	32148745	rs204995	32154285	G	A	0.217	-0.252	0.108	0.020	1.60E-04
IBS	AGER	6	32150423	rs693906	31835164	C	G	0.113	-0.168	0.052	0.001	0.045
UC	AGER	6	32148745	rs204995	32154285	G	A	0.217	-0.870	0.185	2.59E-06	0.002
VHD	AGER	6	32150423	rs693906	31835164	C	G	0.113	0.212	0.052	4.82E-05	0.092

Supplementary table 6. Results of the summary-based Mendelian randomization integrating whole-blood expression quantitative trait loci. For each disease-gene association, we report the strongest effect observed between GTEX and eQTLGen datasets.

A) Bonferroni-significant disease-gene association with HEIDI P > 0.05.

Disease	Gene	chr	gene_bp	topSNP	topSNP_bp	A1	A2	Freq	b_SMR	se_SMR	p_SMR	p_HEIDI
CD	DNMT3A	2	25510652	rs7578575	25488819	A	T	0.329	-0.834	0.164	3.51E-07	0.383
IBD	DNMT3A	2	25510652	rs7578575	25488819	A	T	0.329	-0.745	0.130	9.22E-09	0.515
UC	DNMT3A	2	25510652	rs7578575	25488819	A	T	0.329	-0.627	0.156	5.73E-05	0.541
UC	CFDP1	16	75327596	rs9934007	75390532	C	T	0.754	0.273	0.070	9.61E-05	0.973
CD	STAT3	17	40502964	rs1053004	40466092	G	A	0.380	0.702	0.128	4.40E-08	0.056
CD	IMPDH2	3	49064299	rs9813813	49207093	A	T	0.110	-1.233	0.294	2.74E-05	0.213
IBD	IMPDH2	3	49064299	rs9813813	49207093	A	T	0.110	-1.328	0.258	2.57E-07	0.071
UC	IMPDH2	3	49064299	rs9813813	49207093	A	T	0.110	-1.445	0.307	2.53E-06	0.050
IBD	UBE2L3	22	21941029	rs2070512	21949411	C	A	0.194	0.163	0.021	1.06E-14	0.169
UC	UBE2L3	22	21941029	rs2070512	21949411	C	A	0.194	0.116	0.027	1.76E-05	0.698
CD	BCL2L15	1	1.14E+08	rs1217393	1.14E+08	A	G	0.271	-0.705	0.170	3.39E-05	0.184
CD	STMN3	20	62271061	rs909333	62270637	G	T	0.207	0.547	0.102	8.31E-08	0.200
IBD	STMN3	20	62271061	rs909333	62270637	G	T	0.207	0.555	0.087	2.01E-10	0.055
UC	STMN3	20	62271061	rs909333	62270637	G	T	0.207	0.498	0.098	4.04E-07	0.062
GORD	PBX2	6	32154964	rs693906	31835164	C	G	0.113	0.154	0.036	1.65E-05	0.058
VHD	AGER	6	32150423	rs693906	31835164	C	G	0.113	0.212	0.052	4.82E-05	0.092

B) Nominally significant effect on VHD of Bonferroni-significant gastrointestinal loci.

Disease	Gene	chr	Probe_bp	topSNP	topSNP_bp	A1	A2	Freq	b_SMR	se_SMR	p_SMR	p_HEIDI
VHD	CFDP1	16	75397489	rs7201989	75471499	G	A	0.246	-0.056	0.018	0.001	0.119
VHD	STAT3	17	40502964	rs1053004	40466092	G	A	0.380	0.093	0.047	0.048	0.948
VHD	IMPDH2	3	49064299	rs34037363	48821036	T	C	0.114	-0.209	0.105	0.047	0.089
VHD	UBE2L3	22	21941029	rs2070512	21949411	C	A	0.194	-0.029	0.009	0.002	0.623
VHD	STMN3	20	62271061	rs909333	62270637	G	T	0.207	-0.076	0.036	0.036	0.093

C) Effect of AGER gene on VHD and gastrointestinal disorders.

Disease	Gene	chr	Probe_bp	topSNP	topSNP_bp	A1	A2	Freq	b_SMR	se_SMR	p_SMR	p_HEIDI
CD	AGER	6	32150423	rs693906	31835164	C	G	0.113	-0.312	0.120	0.009	6.16E-08
GORD	AGER	6	32150423	rs693906	31835164	C	G	0.113	-0.209	0.049	2.02E-05	0.022
IBD	AGER	6	32148745	rs204995	32154285	G	A	0.217	-0.252	0.108	0.020	1.60E-04
IBS	AGER	6	32150423	rs693906	31835164	C	G	0.113	-0.168	0.052	0.001	0.045
UC	AGER	6	32148745	rs204995	32154285	G	A	0.217	-0.870	0.185	2.59E-06	0.002
VHD	AGER	6	32150423	rs693906	31835164	C	G	0.113	0.212	0.052	4.82E-05	0.092

Supplementary table 6. Results of the summary-based Mendelian randomization integrating whole-blood expression quantitative trait loci. For each disease-gene association, we report the strongest effect observed between GTEX and eQTLGen datasets.

A) Bonferroni-significant disease-gene association with HEIDI P > 0.05.

Disease	Gene	chr	gene_bp	topSNP	topSNP_bp	A1	A2	Freq	b_SMR	se_SMR	p_SMR	p_HEIDI
CD	DNMT3A	2	25510652	rs7578575	25488819	A	T	0.329	-0.834	0.164	3.51E-07	0.383
IBD	DNMT3A	2	25510652	rs7578575	25488819	A	T	0.329	-0.745	0.130	9.22E-09	0.515
UC	DNMT3A	2	25510652	rs7578575	25488819	A	T	0.329	-0.627	0.156	5.73E-05	0.541
UC	CFDP1	16	75327596	rs9934007	75390532	C	T	0.754	0.273	0.070	9.61E-05	0.973
CD	STAT3	17	40502964	rs1053004	40466092	G	A	0.380	0.702	0.128	4.40E-08	0.056
CD	IMPDH2	3	49064299	rs9813813	49207093	A	T	0.110	-1.233	0.294	2.74E-05	0.213
IBD	IMPDH2	3	49064299	rs9813813	49207093	A	T	0.110	-1.328	0.258	2.57E-07	0.071
UC	IMPDH2	3	49064299	rs9813813	49207093	A	T	0.110	-1.445	0.307	2.53E-06	0.050
IBD	UBE2L3	22	21941029	rs2070512	21949411	C	A	0.194	0.163	0.021	1.06E-14	0.169
UC	UBE2L3	22	21941029	rs2070512	21949411	C	A	0.194	0.116	0.027	1.76E-05	0.698
CD	BCL2L15	1	1.14E+08	rs1217393	1.14E+08	A	G	0.271	-0.705	0.170	3.39E-05	0.184
CD	STMN3	20	62271061	rs909333	62270637	G	T	0.207	0.547	0.102	8.31E-08	0.200
IBD	STMN3	20	62271061	rs909333	62270637	G	T	0.207	0.555	0.087	2.01E-10	0.055
UC	STMN3	20	62271061	rs909333	62270637	G	T	0.207	0.498	0.098	4.04E-07	0.062
GORD	PBX2	6	32154964	rs693906	31835164	C	G	0.113	0.154	0.036	1.65E-05	0.058
VHD	AGER	6	32150423	rs693906	31835164	C	G	0.113	0.212	0.052	4.82E-05	0.092

B) Nominally significant effect on VHD of Bonferroni-significant gastrointestinal loci.

Disease	Gene	chr	Probe_bp	topSNP	topSNP_bp	A1	A2	Freq	b_SMR	se_SMR	p_SMR	p_HEIDI
VHD	CFDP1	16	75397489	rs7201989	75471499	G	A	0.246	-0.056	0.018	0.001	0.119
VHD	STAT3	17	40502964	rs1053004	40466092	G	A	0.380	0.093	0.047	0.048	0.948
VHD	IMPDH2	3	49064299	rs34037363	48821036	T	C	0.114	-0.209	0.105	0.047	0.089
VHD	UBE2L3	22	21941029	rs2070512	21949411	C	A	0.194	-0.029	0.009	0.002	0.623
VHD	STMN3	20	62271061	rs909333	62270637	G	T	0.207	-0.076	0.036	0.036	0.093

C) Effect of AGER gene on VHD and gastrointestinal disorders.

Disease	Gene	chr	Probe_bp	topSNP	topSNP_bp	A1	A2	Freq	b_SMR	se_SMR	p_SMR	p_HEIDI
CD	AGER	6	32150423	rs693906	31835164	C	G	0.113	-0.312	0.120	0.009	6.16E-08
GORD	AGER	6	32150423	rs693906	31835164	C	G	0.113	-0.209	0.049	2.02E-05	0.022
IBD	AGER	6	32148745	rs204995	32154285	G	A	0.217	-0.252	0.108	0.020	1.60E-04
IBS	AGER	6	32150423	rs693906	31835164	C	G	0.113	-0.168	0.052	0.001	0.045
UC	AGER	6	32148745	rs204995	32154285	G	A	0.217	-0.870	0.185	2.59E-06	0.002
VHD	AGER	6	32150423	rs693906	31835164	C	G	0.113	0.212	0.052	4.82E-05	0.092

Supplementary table 6. Results of the summary-based Mendelian randomization integrating whole-blood expression quantitative trait loci. For each disease-gene association, we report the strongest effect observed between GTEX and eQTLGen datasets.

A) Bonferroni-significant disease-gene association with HEIDI P > 0.05.

Disease	Gene	chr	gene_bp	topSNP	topSNP_bp	A1	A2	Freq	b_SMR	se_SMR	p_SMR	p_HEIDI
CD	DNMT3A	2	25510652	rs7578575	25488819	A	T	0.329	-0.834	0.164	3.51E-07	0.383
IBD	DNMT3A	2	25510652	rs7578575	25488819	A	T	0.329	-0.745	0.130	9.22E-09	0.515
UC	DNMT3A	2	25510652	rs7578575	25488819	A	T	0.329	-0.627	0.156	5.73E-05	0.541
UC	CFDP1	16	75327596	rs9934007	75390532	C	T	0.754	0.273	0.070	9.61E-05	0.973
CD	STAT3	17	40502964	rs1053004	40466092	G	A	0.380	0.702	0.128	4.40E-08	0.056
CD	IMPDH2	3	49064299	rs9813813	49207093	A	T	0.110	-1.233	0.294	2.74E-05	0.213
IBD	IMPDH2	3	49064299	rs9813813	49207093	A	T	0.110	-1.328	0.258	2.57E-07	0.071
UC	IMPDH2	3	49064299	rs9813813	49207093	A	T	0.110	-1.445	0.307	2.53E-06	0.050
IBD	UBE2L3	22	21941029	rs2070512	21949411	C	A	0.194	0.163	0.021	1.06E-14	0.169
UC	UBE2L3	22	21941029	rs2070512	21949411	C	A	0.194	0.116	0.027	1.76E-05	0.698
CD	BCL2L15	1	1.14E+08	rs1217393	1.14E+08	A	G	0.271	-0.705	0.170	3.39E-05	0.184
CD	STMN3	20	62271061	rs909333	62270637	G	T	0.207	0.547	0.102	8.31E-08	0.200
IBD	STMN3	20	62271061	rs909333	62270637	G	T	0.207	0.555	0.087	2.01E-10	0.055
UC	STMN3	20	62271061	rs909333	62270637	G	T	0.207	0.498	0.098	4.04E-07	0.062
GORD	PBX2	6	32154964	rs693906	31835164	C	G	0.113	0.154	0.036	1.65E-05	0.058
VHD	AGER	6	32150423	rs693906	31835164	C	G	0.113	0.212	0.052	4.82E-05	0.092

B) Nominally significant effect on VHD of Bonferroni-significant gastrointestinal loci.

Disease	Gene	chr	Probe_bp	topSNP	topSNP_bp	A1	A2	Freq	b_SMR	se_SMR	p_SMR	p_HEIDI
VHD	CFDP1	16	75397489	rs7201989	75471499	G	A	0.246	-0.056	0.018	0.001	0.119
VHD	STAT3	17	40502964	rs1053004	40466092	G	A	0.380	0.093	0.047	0.048	0.948
VHD	IMPDH2	3	49064299	rs34037363	48821036	T	C	0.114	-0.209	0.105	0.047	0.089
VHD	UBE2L3	22	21941029	rs2070512	21949411	C	A	0.194	-0.029	0.009	0.002	0.623
VHD	STMN3	20	62271061	rs909333	62270637	G	T	0.207	-0.076	0.036	0.036	0.093

C) Effect of AGER gene on VHD and gastrointestinal disorders.

Disease	Gene	chr	Probe_bp	topSNP	topSNP_bp	A1	A2	Freq	b_SMR	se_SMR	p_SMR	p_HEIDI
CD	AGER	6	32150423	rs693906	31835164	C	G	0.113	-0.312	0.120	0.009	6.16E-08
GORD	AGER	6	32150423	rs693906	31835164	C	G	0.113	-0.209	0.049	2.02E-05	0.022
IBD	AGER	6	32148745	rs204995	32154285	G	A	0.217	-0.252	0.108	0.020	1.60E-04
IBS	AGER	6	32150423	rs693906	31835164	C	G	0.113	-0.168	0.052	0.001	0.045
UC	AGER	6	32148745	rs204995	32154285	G	A	0.217	-0.870	0.185	2.59E-06	0.002
VHD	AGER	6	32150423	rs693906	31835164	C	G	0.113	0.212	0.052	4.82E-05	0.092

Supplementary table 6. Results of the summary-based Mendelian randomization integrating whole-blood expression quantitative trait loci. For each disease-gene association, we report the strongest effect observed between GTEX and eQTLGen datasets.

A) Bonferroni-significant disease-gene association with HEIDI P > 0.05.

Disease	Gene	chr	gene_bp	topSNP	topSNP_bp	A1	A2	Freq	b_SMR	se_SMR	p_SMR	p_HEIDI
CD	DNMT3A	2	25510652	rs7578575	25488819	A	T	0.329	-0.834	0.164	3.51E-07	0.383
IBD	DNMT3A	2	25510652	rs7578575	25488819	A	T	0.329	-0.745	0.130	9.22E-09	0.515
UC	DNMT3A	2	25510652	rs7578575	25488819	A	T	0.329	-0.627	0.156	5.73E-05	0.541
UC	CFDP1	16	75327596	rs9934007	75390532	C	T	0.754	0.273	0.070	9.61E-05	0.973
CD	STAT3	17	40502964	rs1053004	40466092	G	A	0.380	0.702	0.128	4.40E-08	0.056
CD	IMPDH2	3	49064299	rs9813813	49207093	A	T	0.110	-1.233	0.294	2.74E-05	0.213
IBD	IMPDH2	3	49064299	rs9813813	49207093	A	T	0.110	-1.328	0.258	2.57E-07	0.071
UC	IMPDH2	3	49064299	rs9813813	49207093	A	T	0.110	-1.445	0.307	2.53E-06	0.050
IBD	UBE2L3	22	21941029	rs2070512	21949411	C	A	0.194	0.163	0.021	1.06E-14	0.169
UC	UBE2L3	22	21941029	rs2070512	21949411	C	A	0.194	0.116	0.027	1.76E-05	0.698
CD	BCL2L15	1	1.14E+08	rs1217393	1.14E+08	A	G	0.271	-0.705	0.170	3.39E-05	0.184
CD	STMN3	20	62271061	rs909333	62270637	G	T	0.207	0.547	0.102	8.31E-08	0.200
IBD	STMN3	20	62271061	rs909333	62270637	G	T	0.207	0.555	0.087	2.01E-10	0.055
UC	STMN3	20	62271061	rs909333	62270637	G	T	0.207	0.498	0.098	4.04E-07	0.062
GORD	PBX2	6	32154964	rs693906	31835164	C	G	0.113	0.154	0.036	1.65E-05	0.058
VHD	AGER	6	32150423	rs693906	31835164	C	G	0.113	0.212	0.052	4.82E-05	0.092

B) Nominally significant effect on VHD of Bonferroni-significant gastrointestinal loci.

Disease	Gene	chr	Probe_bp	topSNP	topSNP_bp	A1	A2	Freq	b_SMR	se_SMR	p_SMR	p_HEIDI
VHD	CFDP1	16	75397489	rs7201989	75471499	G	A	0.246	-0.056	0.018	0.001	0.119
VHD	STAT3	17	40502964	rs1053004	40466092	G	A	0.380	0.093	0.047	0.048	0.948
VHD	IMPDH2	3	49064299	rs34037363	48821036	T	C	0.114	-0.209	0.105	0.047	0.089
VHD	UBE2L3	22	21941029	rs2070512	21949411	C	A	0.194	-0.029	0.009	0.002	0.623
VHD	STMN3	20	62271061	rs909333	62270637	G	T	0.207	-0.076	0.036	0.036	0.093

C) Effect of AGER gene on VHD and gastrointestinal disorders.

Disease	Gene	chr	Probe_bp	topSNP	topSNP_bp	A1	A2	Freq	b_SMR	se_SMR	p_SMR	p_HEIDI
CD	AGER	6	32150423	rs693906	31835164	C	G	0.113	-0.312	0.120	0.009	6.16E-08
GORD	AGER	6	32150423	rs693906	31835164	C	G	0.113	-0.209	0.049	2.02E-05	0.022
IBD	AGER	6	32148745	rs204995	32154285	G	A	0.217	-0.252	0.108	0.020	1.60E-04
IBS	AGER	6	32150423	rs693906	31835164	C	G	0.113	-0.168	0.052	0.001	0.045
UC	AGER	6	32148745	rs204995	32154285	G	A	0.217	-0.870	0.185	2.59E-06	0.002
VHD	AGER	6	32150423	rs693906	31835164	C	G	0.113	0.212	0.052	4.82E-05	0.092

Supplementary table 6. Results of the summary-based Mendelian randomization integrating whole-blood expression quantitative trait loci. For each disease-gene association, we report the strongest effect observed between GTEX and eQTLGen datasets.

A) Bonferroni-significant disease-gene association with HEIDI P > 0.05.

Disease	Gene	chr	gene_bp	topSNP	topSNP_bp	A1	A2	Freq	b_SMR	se_SMR	p_SMR	p_HEIDI
CD	DNMT3A	2	25510652	rs7578575	25488819	A	T	0.329	-0.834	0.164	3.51E-07	0.383
IBD	DNMT3A	2	25510652	rs7578575	25488819	A	T	0.329	-0.745	0.130	9.22E-09	0.515
UC	DNMT3A	2	25510652	rs7578575	25488819	A	T	0.329	-0.627	0.156	5.73E-05	0.541
UC	CFDP1	16	75327596	rs9934007	75390532	C	T	0.754	0.273	0.070	9.61E-05	0.973
CD	STAT3	17	40502964	rs1053004	40466092	G	A	0.380	0.702	0.128	4.40E-08	0.056
CD	IMPDH2	3	49064299	rs9813813	49207093	A	T	0.110	-1.233	0.294	2.74E-05	0.213
IBD	IMPDH2	3	49064299	rs9813813	49207093	A	T	0.110	-1.328	0.258	2.57E-07	0.071
UC	IMPDH2	3	49064299	rs9813813	49207093	A	T	0.110	-1.445	0.307	2.53E-06	0.050
IBD	UBE2L3	22	21941029	rs2070512	21949411	C	A	0.194	0.163	0.021	1.06E-14	0.169
UC	UBE2L3	22	21941029	rs2070512	21949411	C	A	0.194	0.116	0.027	1.76E-05	0.698
CD	BCL2L15	1	1.14E+08	rs1217393	1.14E+08	A	G	0.271	-0.705	0.170	3.39E-05	0.184
CD	STMN3	20	62271061	rs909333	62270637	G	T	0.207	0.547	0.102	8.31E-08	0.200
IBD	STMN3	20	62271061	rs909333	62270637	G	T	0.207	0.555	0.087	2.01E-10	0.055
UC	STMN3	20	62271061	rs909333	62270637	G	T	0.207	0.498	0.098	4.04E-07	0.062
GORD	PBX2	6	32154964	rs693906	31835164	C	G	0.113	0.154	0.036	1.65E-05	0.058
VHD	AGER	6	32150423	rs693906	31835164	C	G	0.113	0.212	0.052	4.82E-05	0.092

B) Nominally significant effect on VHD of Bonferroni-significant gastrointestinal loci.

Disease	Gene	chr	Probe_bp	topSNP	topSNP_bp	A1	A2	Freq	b_SMR	se_SMR	p_SMR	p_HEIDI
VHD	CFDP1	16	75397489	rs7201989	75471499	G	A	0.246	-0.056	0.018	0.001	0.119
VHD	STAT3	17	40502964	rs1053004	40466092	G	A	0.380	0.093	0.047	0.048	0.948
VHD	IMPDH2	3	49064299	rs34037363	48821036	T	C	0.114	-0.209	0.105	0.047	0.089
VHD	UBE2L3	22	21941029	rs2070512	21949411	C	A	0.194	-0.029	0.009	0.002	0.623
VHD	STMN3	20	62271061	rs909333	62270637	G	T	0.207	-0.076	0.036	0.036	0.093

C) Effect of AGER gene on VHD and gastrointestinal disorders.

Disease	Gene	chr	Probe_bp	topSNP	topSNP_bp	A1	A2	Freq	b_SMR	se_SMR	p_SMR	p_HEIDI
CD	AGER	6	32150423	rs693906	31835164	C	G	0.113	-0.312	0.120	0.009	6.16E-08
GORD	AGER	6	32150423	rs693906	31835164	C	G	0.113	-0.209	0.049	2.02E-05	0.022
IBD	AGER	6	32148745	rs204995	32154285	G	A	0.217	-0.252	0.108	0.020	1.60E-04
IBS	AGER	6	32150423	rs693906	31835164	C	G	0.113	-0.168	0.052	0.001	0.045
UC	AGER	6	32148745	rs204995	32154285	G	A	0.217	-0.870	0.185	2.59E-06	0.002
VHD	AGER	6	32150423	rs693906	31835164	C	G	0.113	0.212	0.052	4.82E-05	0.092

Supplementary table 6. Results of the summary-based Mendelian randomization integrating whole-blood expression quantitative trait loci. For each disease-gene association, we report the strongest effect observed between GTEX and eQTLGen datasets.

A) Bonferroni-significant disease-gene association with HEIDI P > 0.05.

Disease	Gene	chr	gene_bp	topSNP	topSNP_bp	A1	A2	Freq	b_SMR	se_SMR	p_SMR	p_HEIDI
CD	DNMT3A	2	25510652	rs7578575	25488819	A	T	0.329	-0.834	0.164	3.51E-07	0.383
IBD	DNMT3A	2	25510652	rs7578575	25488819	A	T	0.329	-0.745	0.130	9.22E-09	0.515
UC	DNMT3A	2	25510652	rs7578575	25488819	A	T	0.329	-0.627	0.156	5.73E-05	0.541
UC	CFDP1	16	75327596	rs9934007	75390532	C	T	0.754	0.273	0.070	9.61E-05	0.973
CD	STAT3	17	40502964	rs1053004	40466092	G	A	0.380	0.702	0.128	4.40E-08	0.056
CD	IMPDH2	3	49064299	rs9813813	49207093	A	T	0.110	-1.233	0.294	2.74E-05	0.213
IBD	IMPDH2	3	49064299	rs9813813	49207093	A	T	0.110	-1.328	0.258	2.57E-07	0.071
UC	IMPDH2	3	49064299	rs9813813	49207093	A	T	0.110	-1.445	0.307	2.53E-06	0.050
IBD	UBE2L3	22	21941029	rs2070512	21949411	C	A	0.194	0.163	0.021	1.06E-14	0.169
UC	UBE2L3	22	21941029	rs2070512	21949411	C	A	0.194	0.116	0.027	1.76E-05	0.698
CD	BCL2L15	1	1.14E+08	rs1217393	1.14E+08	A	G	0.271	-0.705	0.170	3.39E-05	0.184
CD	STMN3	20	62271061	rs909333	62270637	G	T	0.207	0.547	0.102	8.31E-08	0.200
IBD	STMN3	20	62271061	rs909333	62270637	G	T	0.207	0.555	0.087	2.01E-10	0.055
UC	STMN3	20	62271061	rs909333	62270637	G	T	0.207	0.498	0.098	4.04E-07	0.062
GORD	PBX2	6	32154964	rs693906	31835164	C	G	0.113	0.154	0.036	1.65E-05	0.058
VHD	AGER	6	32150423	rs693906	31835164	C	G	0.113	0.212	0.052	4.82E-05	0.092

B) Nominally significant effect on VHD of Bonferroni-significant gastrointestinal loci.

Disease	Gene	chr	Probe_bp	topSNP	topSNP_bp	A1	A2	Freq	b_SMR	se_SMR	p_SMR	p_HEIDI
VHD	CFDP1	16	75397489	rs7201989	75471499	G	A	0.246	-0.056	0.018	0.001	0.119
VHD	STAT3	17	40502964	rs1053004	40466092	G	A	0.380	0.093	0.047	0.048	0.948
VHD	IMPDH2	3	49064299	rs34037363	48821036	T	C	0.114	-0.209	0.105	0.047	0.089
VHD	UBE2L3	22	21941029	rs2070512	21949411	C	A	0.194	-0.029	0.009	0.002	0.623
VHD	STMN3	20	62271061	rs909333	62270637	G	T	0.207	-0.076	0.036	0.036	0.093

C) Effect of AGER gene on VHD and gastrointestinal disorders.

Disease	Gene	chr	Probe_bp	topSNP	topSNP_bp	A1	A2	Freq	b_SMR	se_SMR	p_SMR	p_HEIDI
CD	AGER	6	32150423	rs693906	31835164	C	G	0.113	-0.312	0.120	0.009	6.16E-08
GORD	AGER	6	32150423	rs693906	31835164	C	G	0.113	-0.209	0.049	2.02E-05	0.022
IBD	AGER	6	32148745	rs204995	32154285	G	A	0.217	-0.252	0.108	0.020	1.60E-04
IBS	AGER	6	32150423	rs693906	31835164	C	G	0.113	-0.168	0.052	0.001	0.045
UC	AGER	6	32148745	rs204995	32154285	G	A	0.217	-0.870	0.185	2.59E-06	0.002
VHD	AGER	6	32150423	rs693906	31835164	C	G	0.113	0.212	0.052	4.82E-05	0.092

Supplementary table 6. Results of the summary-based Mendelian randomization integrating whole-blood expression quantitative trait loci. For each disease-gene association, we report the strongest effect observed between GTEX and eQTLGen datasets.

A) Bonferroni-significant disease-gene association with HEIDI P > 0.05.

Disease	Gene	chr	gene_bp	topSNP	topSNP_bp	A1	A2	Freq	b_SMR	se_SMR	p_SMR	p_HEIDI
CD	DNMT3A	2	25510652	rs7578575	25488819	A	T	0.329	-0.834	0.164	3.51E-07	0.383
IBD	DNMT3A	2	25510652	rs7578575	25488819	A	T	0.329	-0.745	0.130	9.22E-09	0.515
UC	DNMT3A	2	25510652	rs7578575	25488819	A	T	0.329	-0.627	0.156	5.73E-05	0.541
UC	CFDP1	16	75327596	rs9934007	75390532	C	T	0.754	0.273	0.070	9.61E-05	0.973
CD	STAT3	17	40502964	rs1053004	40466092	G	A	0.380	0.702	0.128	4.40E-08	0.056
CD	IMPDH2	3	49064299	rs9813813	49207093	A	T	0.110	-1.233	0.294	2.74E-05	0.213
IBD	IMPDH2	3	49064299	rs9813813	49207093	A	T	0.110	-1.328	0.258	2.57E-07	0.071
UC	IMPDH2	3	49064299	rs9813813	49207093	A	T	0.110	-1.445	0.307	2.53E-06	0.050
IBD	UBE2L3	22	21941029	rs2070512	21949411	C	A	0.194	0.163	0.021	1.06E-14	0.169
UC	UBE2L3	22	21941029	rs2070512	21949411	C	A	0.194	0.116	0.027	1.76E-05	0.698
CD	BCL2L15	1	1.14E+08	rs1217393	1.14E+08	A	G	0.271	-0.705	0.170	3.39E-05	0.184
CD	STMN3	20	62271061	rs909333	62270637	G	T	0.207	0.547	0.102	8.31E-08	0.200
IBD	STMN3	20	62271061	rs909333	62270637	G	T	0.207	0.555	0.087	2.01E-10	0.055
UC	STMN3	20	62271061	rs909333	62270637	G	T	0.207	0.498	0.098	4.04E-07	0.062
GORD	PBX2	6	32154964	rs693906	31835164	C	G	0.113	0.154	0.036	1.65E-05	0.058
VHD	AGER	6	32150423	rs693906	31835164	C	G	0.113	0.212	0.052	4.82E-05	0.092

B) Nominally significant effect on VHD of Bonferroni-significant gastrointestinal loci.

Disease	Gene	chr	Probe_bp	topSNP	topSNP_bp	A1	A2	Freq	b_SMR	se_SMR	p_SMR	p_HEIDI
VHD	CFDP1	16	75397489	rs7201989	75471499	G	A	0.246	-0.056	0.018	0.001	0.119
VHD	STAT3	17	40502964	rs1053004	40466092	G	A	0.380	0.093	0.047	0.048	0.948
VHD	IMPDH2	3	49064299	rs34037363	48821036	T	C	0.114	-0.209	0.105	0.047	0.089
VHD	UBE2L3	22	21941029	rs2070512	21949411	C	A	0.194	-0.029	0.009	0.002	0.623
VHD	STMN3	20	62271061	rs909333	62270637	G	T	0.207	-0.076	0.036	0.036	0.093

C) Effect of AGER gene on VHD and gastrointestinal disorders.

Disease	Gene	chr	Probe_bp	topSNP	topSNP_bp	A1	A2	Freq	b_SMR	se_SMR	p_SMR	p_HEIDI
CD	AGER	6	32150423	rs693906	31835164	C	G	0.113	-0.312	0.120	0.009	6.16E-08
GORD	AGER	6	32150423	rs693906	31835164	C	G	0.113	-0.209	0.049	2.02E-05	0.022
IBD	AGER	6	32148745	rs204995	32154285	G	A	0.217	-0.252	0.108	0.020	1.60E-04
IBS	AGER	6	32150423	rs693906	31835164	C	G	0.113	-0.168	0.052	0.001	0.045
UC	AGER	6	32148745	rs204995	32154285	G	A	0.217	-0.870	0.185	2.59E-06	0.002
VHD	AGER	6	32150423	rs693906	31835164	C	G	0.113	0.212	0.052	4.82E-05	0.092

Supplementary table 6. Results of the summary-based Mendelian randomization integrating whole-blood expression quantitative trait loci. For each disease-gene association, we report the strongest effect observed between GTEX and eQTLGen datasets.

A) Bonferroni-significant disease-gene association with HEIDI P > 0.05.

Disease	Gene	chr	gene_bp	topSNP	topSNP_bp	A1	A2	Freq	b_SMR	se_SMR	p_SMR	p_HEIDI
CD	DNMT3A	2	25510652	rs7578575	25488819	A	T	0.329	-0.834	0.164	3.51E-07	0.383
IBD	DNMT3A	2	25510652	rs7578575	25488819	A	T	0.329	-0.745	0.130	9.22E-09	0.515
UC	DNMT3A	2	25510652	rs7578575	25488819	A	T	0.329	-0.627	0.156	5.73E-05	0.541
UC	CFDP1	16	75327596	rs9934007	75390532	C	T	0.754	0.273	0.070	9.61E-05	0.973
CD	STAT3	17	40502964	rs1053004	40466092	G	A	0.380	0.702	0.128	4.40E-08	0.056
CD	IMPDH2	3	49064299	rs9813813	49207093	A	T	0.110	-1.233	0.294	2.74E-05	0.213
IBD	IMPDH2	3	49064299	rs9813813	49207093	A	T	0.110	-1.328	0.258	2.57E-07	0.071
UC	IMPDH2	3	49064299	rs9813813	49207093	A	T	0.110	-1.445	0.307	2.53E-06	0.050
IBD	UBE2L3	22	21941029	rs2070512	21949411	C	A	0.194	0.163	0.021	1.06E-14	0.169
UC	UBE2L3	22	21941029	rs2070512	21949411	C	A	0.194	0.116	0.027	1.76E-05	0.698
CD	BCL2L15	1	1.14E+08	rs1217393	1.14E+08	A	G	0.271	-0.705	0.170	3.39E-05	0.184
CD	STMN3	20	62271061	rs909333	62270637	G	T	0.207	0.547	0.102	8.31E-08	0.200
IBD	STMN3	20	62271061	rs909333	62270637	G	T	0.207	0.555	0.087	2.01E-10	0.055
UC	STMN3	20	62271061	rs909333	62270637	G	T	0.207	0.498	0.098	4.04E-07	0.062
GORD	PBX2	6	32154964	rs693906	31835164	C	G	0.113	0.154	0.036	1.65E-05	0.058
VHD	AGER	6	32150423	rs693906	31835164	C	G	0.113	0.212	0.052	4.82E-05	0.092

B) Nominally significant effect on VHD of Bonferroni-significant gastrointestinal loci.

Disease	Gene	chr	Probe_bp	topSNP	topSNP_bp	A1	A2	Freq	b_SMR	se_SMR	p_SMR	p_HEIDI
VHD	CFDP1	16	75397489	rs7201989	75471499	G	A	0.246	-0.056	0.018	0.001	0.119
VHD	STAT3	17	40502964	rs1053004	40466092	G	A	0.380	0.093	0.047	0.048	0.948
VHD	IMPDH2	3	49064299	rs34037363	48821036	T	C	0.114	-0.209	0.105	0.047	0.089
VHD	UBE2L3	22	21941029	rs2070512	21949411	C	A	0.194	-0.029	0.009	0.002	0.623
VHD	STMN3	20	62271061	rs909333	62270637	G	T	0.207	-0.076	0.036	0.036	0.093

C) Effect of AGER gene on VHD and gastrointestinal disorders.

Disease	Gene	chr	Probe_bp	topSNP	topSNP_bp	A1	A2	Freq	b_SMR	se_SMR	p_SMR	p_HEIDI
CD	AGER	6	32150423	rs693906	31835164	C	G	0.113	-0.312	0.120	0.009	6.16E-08
GORD	AGER	6	32150423	rs693906	31835164	C	G	0.113	-0.209	0.049	2.02E-05	0.022
IBD	AGER	6	32148745	rs204995	32154285	G	A	0.217	-0.252	0.108	0.020	1.60E-04
IBS	AGER	6	32150423	rs693906	31835164	C	G	0.113	-0.168	0.052	0.001	0.045
UC	AGER	6	32148745	rs204995	32154285	G	A	0.217	-0.870	0.185	2.59E-06	0.002
VHD	AGER	6	32150423	rs693906	31835164	C	G	0.113	0.212	0.052	4.82E-05	0.092

Supplementary table 6. Results of the summary-based Mendelian randomization integrating whole-blood expression quantitative trait loci. For each disease-gene association, we report the strongest effect observed between GTEX and eQTLGen datasets.

A) Bonferroni-significant disease-gene association with HEIDI P > 0.05.

Disease	Gene	chr	gene_bp	topSNP	topSNP_bp	A1	A2	Freq	b_SMR	se_SMR	p_SMR	p_HEIDI
CD	DNMT3A	2	25510652	rs7578575	25488819	A	T	0.329	-0.834	0.164	3.51E-07	0.383
IBD	DNMT3A	2	25510652	rs7578575	25488819	A	T	0.329	-0.745	0.130	9.22E-09	0.515
UC	DNMT3A	2	25510652	rs7578575	25488819	A	T	0.329	-0.627	0.156	5.73E-05	0.541
UC	CFDP1	16	75327596	rs9934007	75390532	C	T	0.754	0.273	0.070	9.61E-05	0.973
CD	STAT3	17	40502964	rs1053004	40466092	G	A	0.380	0.702	0.128	4.40E-08	0.056
CD	IMPDH2	3	49064299	rs9813813	49207093	A	T	0.110	-1.233	0.294	2.74E-05	0.213
IBD	IMPDH2	3	49064299	rs9813813	49207093	A	T	0.110	-1.328	0.258	2.57E-07	0.071
UC	IMPDH2	3	49064299	rs9813813	49207093	A	T	0.110	-1.445	0.307	2.53E-06	0.050
IBD	UBE2L3	22	21941029	rs2070512	21949411	C	A	0.194	0.163	0.021	1.06E-14	0.169
UC	UBE2L3	22	21941029	rs2070512	21949411	C	A	0.194	0.116	0.027	1.76E-05	0.698
CD	BCL2L15	1	1.14E+08	rs1217393	1.14E+08	A	G	0.271	-0.705	0.170	3.39E-05	0.184
CD	STMN3	20	62271061	rs909333	62270637	G	T	0.207	0.547	0.102	8.31E-08	0.200
IBD	STMN3	20	62271061	rs909333	62270637	G	T	0.207	0.555	0.087	2.01E-10	0.055
UC	STMN3	20	62271061	rs909333	62270637	G	T	0.207	0.498	0.098	4.04E-07	0.062
GORD	PBX2	6	32154964	rs693906	31835164	C	G	0.113	0.154	0.036	1.65E-05	0.058
VHD	AGER	6	32150423	rs693906	31835164	C	G	0.113	0.212	0.052	4.82E-05	0.092

B) Nominally significant effect on VHD of Bonferroni-significant gastrointestinal loci.

Disease	Gene	chr	Probe_bp	topSNP	topSNP_bp	A1	A2	Freq	b_SMR	se_SMR	p_SMR	p_HEIDI
VHD	CFDP1	16	75397489	rs7201989	75471499	G	A	0.246	-0.056	0.018	0.001	0.119
VHD	STAT3	17	40502964	rs1053004	40466092	G	A	0.380	0.093	0.047	0.048	0.948
VHD	IMPDH2	3	49064299	rs34037363	48821036	T	C	0.114	-0.209	0.105	0.047	0.089
VHD	UBE2L3	22	21941029	rs2070512	21949411	C	A	0.194	-0.029	0.009	0.002	0.623
VHD	STMN3	20	62271061	rs909333	62270637	G	T	0.207	-0.076	0.036	0.036	0.093

C) Effect of AGER gene on VHD and gastrointestinal disorders.

Disease	Gene	chr	Probe_bp	topSNP	topSNP_bp	A1	A2	Freq	b_SMR	se_SMR	p_SMR	p_HEIDI
CD	AGER	6	32150423	rs693906	31835164	C	G	0.113	-0.312	0.120	0.009	6.16E-08
GORD	AGER	6	32150423	rs693906	31835164	C	G	0.113	-0.209	0.049	2.02E-05	0.022
IBD	AGER	6	32148745	rs204995	32154285	G	A	0.217	-0.252	0.108	0.020	1.60E-04
IBS	AGER	6	32150423	rs693906	31835164	C	G	0.113	-0.168	0.052	0.001	0.045
UC	AGER	6	32148745	rs204995	32154285	G	A	0.217	-0.870	0.185	2.59E-06	0.002
VHD	AGER	6	32150423	rs693906	31835164	C	G	0.113	0.212	0.052	4.82E-05	0.092

Supplementary table 6. Results of the summary-based Mendelian randomization integrating whole-blood expression quantitative trait loci. For each disease-gene association, we report the strongest effect observed between GTEX and eQTLGen datasets.

A) Bonferroni-significant disease-gene association with HEIDI P > 0.05.

Disease	Gene	chr	gene_bp	topSNP	topSNP_bp	A1	A2	Freq	b_SMR	se_SMR	p_SMR	p_HEIDI
CD	DNMT3A	2	25510652	rs7578575	25488819	A	T	0.329	-0.834	0.164	3.51E-07	0.383
IBD	DNMT3A	2	25510652	rs7578575	25488819	A	T	0.329	-0.745	0.130	9.22E-09	0.515
UC	DNMT3A	2	25510652	rs7578575	25488819	A	T	0.329	-0.627	0.156	5.73E-05	0.541
UC	CFDP1	16	75327596	rs9934007	75390532	C	T	0.754	0.273	0.070	9.61E-05	0.973
CD	STAT3	17	40502964	rs1053004	40466092	G	A	0.380	0.702	0.128	4.40E-08	0.056
CD	IMPDH2	3	49064299	rs9813813	49207093	A	T	0.110	-1.233	0.294	2.74E-05	0.213
IBD	IMPDH2	3	49064299	rs9813813	49207093	A	T	0.110	-1.328	0.258	2.57E-07	0.071
UC	IMPDH2	3	49064299	rs9813813	49207093	A	T	0.110	-1.445	0.307	2.53E-06	0.050
IBD	UBE2L3	22	21941029	rs2070512	21949411	C	A	0.194	0.163	0.021	1.06E-14	0.169
UC	UBE2L3	22	21941029	rs2070512	21949411	C	A	0.194	0.116	0.027	1.76E-05	0.698
CD	BCL2L15	1	1.14E+08	rs1217393	1.14E+08	A	G	0.271	-0.705	0.170	3.39E-05	0.184
CD	STMN3	20	62271061	rs909333	62270637	G	T	0.207	0.547	0.102	8.31E-08	0.200
IBD	STMN3	20	62271061	rs909333	62270637	G	T	0.207	0.555	0.087	2.01E-10	0.055
UC	STMN3	20	62271061	rs909333	62270637	G	T	0.207	0.498	0.098	4.04E-07	0.062
GORD	PBX2	6	32154964	rs693906	31835164	C	G	0.113	0.154	0.036	1.65E-05	0.058
VHD	AGER	6	32150423	rs693906	31835164	C	G	0.113	0.212	0.052	4.82E-05	0.092

B) Nominally significant effect on VHD of Bonferroni-significant gastrointestinal loci.

Disease	Gene	chr	Probe_bp	topSNP	topSNP_bp	A1	A2	Freq	b_SMR	se_SMR	p_SMR	p_HEIDI
VHD	CFDP1	16	75397489	rs7201989	75471499	G	A	0.246	-0.056	0.018	0.001	0.119
VHD	STAT3	17	40502964	rs1053004	40466092	G	A	0.380	0.093	0.047	0.048	0.948
VHD	IMPDH2	3	49064299	rs34037363	48821036	T	C	0.114	-0.209	0.105	0.047	0.089
VHD	UBE2L3	22	21941029	rs2070512	21949411	C	A	0.194	-0.029	0.009	0.002	0.623
VHD	STMN3	20	62271061	rs909333	62270637	G	T	0.207	-0.076	0.036	0.036	0.093

C) Effect of AGER gene on VHD and gastrointestinal disorders.

Disease	Gene	chr	Probe_bp	topSNP	topSNP_bp	A1	A2	Freq	b_SMR	se_SMR	p_SMR	p_HEIDI
CD	AGER	6	32150423	rs693906	31835164	C	G	0.113	-0.312	0.120	0.009	6.16E-08
GORD	AGER	6	32150423	rs693906	31835164	C	G	0.113	-0.209	0.049	2.02E-05	0.022
IBD	AGER	6	32148745	rs204995	32154285	G	A	0.217	-0.252	0.108	0.020	1.60E-04
IBS	AGER	6	32150423	rs693906	31835164	C	G	0.113	-0.168	0.052	0.001	0.045
UC	AGER	6	32148745	rs204995	32154285	G	A	0.217	-0.870	0.185	2.59E-06	0.002
VHD	AGER	6	32150423	rs693906	31835164	C	G	0.113	0.212	0.052	4.82E-05	0.092

Supplementary table 6. Results of the summary-based Mendelian randomization integrating whole-blood expression quantitative trait loci. For each disease-gene association, we report the strongest effect observed between GTEX and eQTLGen datasets.

A) Bonferroni-significant disease-gene association with HEIDI P > 0.05.

Disease	Gene	chr	gene_bp	topSNP	topSNP_bp	A1	A2	Freq	b_SMR	se_SMR	p_SMR	p_HEIDI
CD	DNMT3A	2	25510652	rs7578575	25488819	A	T	0.329	-0.834	0.164	3.51E-07	0.383
IBD	DNMT3A	2	25510652	rs7578575	25488819	A	T	0.329	-0.745	0.130	9.22E-09	0.515
UC	DNMT3A	2	25510652	rs7578575	25488819	A	T	0.329	-0.627	0.156	5.73E-05	0.541
UC	CFDP1	16	75327596	rs9934007	75390532	C	T	0.754	0.273	0.070	9.61E-05	0.973
CD	STAT3	17	40502964	rs1053004	40466092	G	A	0.380	0.702	0.128	4.40E-08	0.056
CD	IMPDH2	3	49064299	rs9813813	49207093	A	T	0.110	-1.233	0.294	2.74E-05	0.213
IBD	IMPDH2	3	49064299	rs9813813	49207093	A	T	0.110	-1.328	0.258	2.57E-07	0.071
UC	IMPDH2	3	49064299	rs9813813	49207093	A	T	0.110	-1.445	0.307	2.53E-06	0.050
IBD	UBE2L3	22	21941029	rs2070512	21949411	C	A	0.194	0.163	0.021	1.06E-14	0.169
UC	UBE2L3	22	21941029	rs2070512	21949411	C	A	0.194	0.116	0.027	1.76E-05	0.698
CD	BCL2L15	1	1.14E+08	rs1217393	1.14E+08	A	G	0.271	-0.705	0.170	3.39E-05	0.184
CD	STMN3	20	62271061	rs909333	62270637	G	T	0.207	0.547	0.102	8.31E-08	0.200
IBD	STMN3	20	62271061	rs909333	62270637	G	T	0.207	0.555	0.087	2.01E-10	0.055
UC	STMN3	20	62271061	rs909333	62270637	G	T	0.207	0.498	0.098	4.04E-07	0.062
GORD	PBX2	6	32154964	rs693906	31835164	C	G	0.113	0.154	0.036	1.65E-05	0.058
VHD	AGER	6	32150423	rs693906	31835164	C	G	0.113	0.212	0.052	4.82E-05	0.092

B) Nominally significant effect on VHD of Bonferroni-significant gastrointestinal loci.

Disease	Gene	chr	Probe_bp	topSNP	topSNP_bp	A1	A2	Freq	b_SMR	se_SMR	p_SMR	p_HEIDI
VHD	CFDP1	16	75397489	rs7201989	75471499	G	A	0.246	-0.056	0.018	0.001	0.119
VHD	STAT3	17	40502964	rs1053004	40466092	G	A	0.380	0.093	0.047	0.048	0.948
VHD	IMPDH2	3	49064299	rs34037363	48821036	T	C	0.114	-0.209	0.105	0.047	0.089
VHD	UBE2L3	22	21941029	rs2070512	21949411	C	A	0.194	-0.029	0.009	0.002	0.623
VHD	STMN3	20	62271061	rs909333	62270637	G	T	0.207	-0.076	0.036	0.036	0.093

C) Effect of AGER gene on VHD and gastrointestinal disorders.

Disease	Gene	chr	Probe_bp	topSNP	topSNP_bp	A1	A2	Freq	b_SMR	se_SMR	p_SMR	p_HEIDI
CD	AGER	6	32150423	rs693906	31835164	C	G	0.113	-0.312	0.120	0.009	6.16E-08
GORD	AGER	6	32150423	rs693906	31835164	C	G	0.113	-0.209	0.049	2.02E-05	0.022
IBD	AGER	6	32148745	rs204995	32154285	G	A	0.217	-0.252	0.108	0.020	1.60E-04
IBS	AGER	6	32150423	rs693906	31835164	C	G	0.113	-0.168	0.052	0.001	0.045
UC	AGER	6	32148745	rs204995	32154285	G	A	0.217	-0.870	0.185	2.59E-06	0.002
VHD	AGER	6	32150423	rs693906	31835164	C	G	0.113	0.212	0.052	4.82E-05	0.092

Supplementary table 6. Results of the summary-based Mendelian randomization integrating whole-blood expression quantitative trait loci. For each disease-gene association, we report the strongest effect observed between GTEX and eQTLGen datasets.

A) Bonferroni-significant disease-gene association with HEIDI P > 0.05.

Disease	Gene	chr	gene_bp	topSNP	topSNP_bp	A1	A2	Freq	b_SMR	se_SMR	p_SMR	p_HEIDI
CD	DNMT3A	2	25510652	rs7578575	25488819	A	T	0.329	-0.834	0.164	3.51E-07	0.383
IBD	DNMT3A	2	25510652	rs7578575	25488819	A	T	0.329	-0.745	0.130	9.22E-09	0.515
UC	DNMT3A	2	25510652	rs7578575	25488819	A	T	0.329	-0.627	0.156	5.73E-05	0.541
UC	CFDP1	16	75327596	rs9934007	75390532	C	T	0.754	0.273	0.070	9.61E-05	0.973
CD	STAT3	17	40502964	rs1053004	40466092	G	A	0.380	0.702	0.128	4.40E-08	0.056
CD	IMPDH2	3	49064299	rs9813813	49207093	A	T	0.110	-1.233	0.294	2.74E-05	0.213
IBD	IMPDH2	3	49064299	rs9813813	49207093	A	T	0.110	-1.328	0.258	2.57E-07	0.071
UC	IMPDH2	3	49064299	rs9813813	49207093	A	T	0.110	-1.445	0.307	2.53E-06	0.050
IBD	UBE2L3	22	21941029	rs2070512	21949411	C	A	0.194	0.163	0.021	1.06E-14	0.169
UC	UBE2L3	22	21941029	rs2070512	21949411	C	A	0.194	0.116	0.027	1.76E-05	0.698
CD	BCL2L15	1	1.14E+08	rs1217393	1.14E+08	A	G	0.271	-0.705	0.170	3.39E-05	0.184
CD	STMN3	20	62271061	rs909333	62270637	G	T	0.207	0.547	0.102	8.31E-08	0.200
IBD	STMN3	20	62271061	rs909333	62270637	G	T	0.207	0.555	0.087	2.01E-10	0.055
UC	STMN3	20	62271061	rs909333	62270637	G	T	0.207	0.498	0.098	4.04E-07	0.062
GORD	PBX2	6	32154964	rs693906	31835164	C	G	0.113	0.154	0.036	1.65E-05	0.058
VHD	AGER	6	32150423	rs693906	31835164	C	G	0.113	0.212	0.052	4.82E-05	0.092

B) Nominally significant effect on VHD of Bonferroni-significant gastrointestinal loci.

Disease	Gene	chr	Probe_bp	topSNP	topSNP_bp	A1	A2	Freq	b_SMR	se_SMR	p_SMR	p_HEIDI
VHD	CFDP1	16	75397489	rs7201989	75471499	G	A	0.246	-0.056	0.018	0.001	0.119
VHD	STAT3	17	40502964	rs1053004	40466092	G	A	0.380	0.093	0.047	0.048	0.948
VHD	IMPDH2	3	49064299	rs34037363	48821036	T	C	0.114	-0.209	0.105	0.047	0.089
VHD	UBE2L3	22	21941029	rs2070512	21949411	C	A	0.194	-0.029	0.009	0.002	0.623
VHD	STMN3	20	62271061	rs909333	62270637	G	T	0.207	-0.076	0.036	0.036	0.093

C) Effect of AGER gene on VHD and gastrointestinal disorders.

Disease	Gene	chr	Probe_bp	topSNP	topSNP_bp	A1	A2	Freq	b_SMR	se_SMR	p_SMR	p_HEIDI
CD	AGER	6	32150423	rs693906	31835164	C	G	0.113	-0.312	0.120	0.009	6.16E-08
GORD	AGER	6	32150423	rs693906	31835164	C	G	0.113	-0.209	0.049	2.02E-05	0.022
IBD	AGER	6	32148745	rs204995	32154285	G	A	0.217	-0.252	0.108	0.020	1.60E-04
IBS	AGER	6	32150423	rs693906	31835164	C	G	0.113	-0.168	0.052	0.001	0.045
UC	AGER	6	32148745	rs204995	32154285	G	A	0.217	-0.870	0.185	2.59E-06	0.002
VHD	AGER	6	32150423	rs693906	31835164	C	G	0.113	0.212	0.052	4.82E-05	0.092

Supplementary table 6. Results of the summary-based Mendelian randomization integrating whole-blood expression quantitative trait loci. For each disease-gene association, we report the strongest effect observed between GTEX and eQTLGen datasets.

A) Bonferroni-significant disease-gene association with HEIDI P > 0.05.

Disease	Gene	chr	gene_bp	topSNP	topSNP_bp	A1	A2	Freq	b_SMR	se_SMR	p_SMR	p_HEIDI
CD	DNMT3A	2	25510652	rs7578575	25488819	A	T	0.329	-0.834	0.164	3.51E-07	0.383
IBD	DNMT3A	2	25510652	rs7578575	25488819	A	T	0.329	-0.745	0.130	9.22E-09	0.515
UC	DNMT3A	2	25510652	rs7578575	25488819	A	T	0.329	-0.627	0.156	5.73E-05	0.541
UC	CFDP1	16	75327596	rs9934007	75390532	C	T	0.754	0.273	0.070	9.61E-05	0.973
CD	STAT3	17	40502964	rs1053004	40466092	G	A	0.380	0.702	0.128	4.40E-08	0.056
CD	IMPDH2	3	49064299	rs9813813	49207093	A	T	0.110	-1.233	0.294	2.74E-05	0.213
IBD	IMPDH2	3	49064299	rs9813813	49207093	A	T	0.110	-1.328	0.258	2.57E-07	0.071
UC	IMPDH2	3	49064299	rs9813813	49207093	A	T	0.110	-1.445	0.307	2.53E-06	0.050
IBD	UBE2L3	22	21941029	rs2070512	21949411	C	A	0.194	0.163	0.021	1.06E-14	0.169
UC	UBE2L3	22	21941029	rs2070512	21949411	C	A	0.194	0.116	0.027	1.76E-05	0.698
CD	BCL2L15	1	1.14E+08	rs1217393	1.14E+08	A	G	0.271	-0.705	0.170	3.39E-05	0.184
CD	STMN3	20	62271061	rs909333	62270637	G	T	0.207	0.547	0.102	8.31E-08	0.200
IBD	STMN3	20	62271061	rs909333	62270637	G	T	0.207	0.555	0.087	2.01E-10	0.055
UC	STMN3	20	62271061	rs909333	62270637	G	T	0.207	0.498	0.098	4.04E-07	0.062
GORD	PBX2	6	32154964	rs693906	31835164	C	G	0.113	0.154	0.036	1.65E-05	0.058
VHD	AGER	6	32150423	rs693906	31835164	C	G	0.113	0.212	0.052	4.82E-05	0.092

B) Nominally significant effect on VHD of Bonferroni-significant gastrointestinal loci.

Disease	Gene	chr	Probe_bp	topSNP	topSNP_bp	A1	A2	Freq	b_SMR	se_SMR	p_SMR	p_HEIDI
VHD	CFDP1	16	75397489	rs7201989	75471499	G	A	0.246	-0.056	0.018	0.001	0.119
VHD	STAT3	17	40502964	rs1053004	40466092	G	A	0.380	0.093	0.047	0.048	0.948
VHD	IMPDH2	3	49064299	rs34037363	48821036	T	C	0.114	-0.209	0.105	0.047	0.089
VHD	UBE2L3	22	21941029	rs2070512	21949411	C	A	0.194	-0.029	0.009	0.002	0.623
VHD	STMN3	20	62271061	rs909333	62270637	G	T	0.207	-0.076	0.036	0.036	0.093

C) Effect of AGER gene on VHD and gastrointestinal disorders.

Disease	Gene	chr	Probe_bp	topSNP	topSNP_bp	A1	A2	Freq	b_SMR	se_SMR	p_SMR	p_HEIDI
CD	AGER	6	32150423	rs693906	31835164	C	G	0.113	-0.312	0.120	0.009	6.16E-08
GORD	AGER	6	32150423	rs693906	31835164	C	G	0.113	-0.209	0.049	2.02E-05	0.022
IBD	AGER	6	32148745	rs204995	32154285	G	A	0.217	-0.252	0.108	0.020	1.60E-04
IBS	AGER	6	32150423	rs693906	31835164	C	G	0.113	-0.168	0.052	0.001	0.045
UC	AGER	6	32148745	rs204995	32154285	G	A	0.217	-0.870	0.185	2.59E-06	0.002
VHD	AGER	6	32150423	rs693906	31835164	C	G	0.113	0.212	0.052	4.82E-05	0.092

Supplementary table 6. Results of the summary-based Mendelian randomization integrating whole-blood expression quantitative trait loci. For each disease-gene association, we report the strongest effect observed between GTEX and eQTLGen datasets.

A) Bonferroni-significant disease-gene association with HEIDI P > 0.05.

Disease	Gene	chr	gene_bp	topSNP	topSNP_bp	A1	A2	Freq	b_SMR	se_SMR	p_SMR	p_HEIDI
CD	DNMT3A	2	25510652	rs7578575	25488819	A	T	0.329	-0.834	0.164	3.51E-07	0.383
IBD	DNMT3A	2	25510652	rs7578575	25488819	A	T	0.329	-0.745	0.130	9.22E-09	0.515
UC	DNMT3A	2	25510652	rs7578575	25488819	A	T	0.329	-0.627	0.156	5.73E-05	0.541
UC	CFDP1	16	75327596	rs9934007	75390532	C	T	0.754	0.273	0.070	9.61E-05	0.973
CD	STAT3	17	40502964	rs1053004	40466092	G	A	0.380	0.702	0.128	4.40E-08	0.056
CD	IMPDH2	3	49064299	rs9813813	49207093	A	T	0.110	-1.233	0.294	2.74E-05	0.213
IBD	IMPDH2	3	49064299	rs9813813	49207093	A	T	0.110	-1.328	0.258	2.57E-07	0.071
UC	IMPDH2	3	49064299	rs9813813	49207093	A	T	0.110	-1.445	0.307	2.53E-06	0.050
IBD	UBE2L3	22	21941029	rs2070512	21949411	C	A	0.194	0.163	0.021	1.06E-14	0.169
UC	UBE2L3	22	21941029	rs2070512	21949411	C	A	0.194	0.116	0.027	1.76E-05	0.698
CD	BCL2L15	1	1.14E+08	rs1217393	1.14E+08	A	G	0.271	-0.705	0.170	3.39E-05	0.184
CD	STMN3	20	62271061	rs909333	62270637	G	T	0.207	0.547	0.102	8.31E-08	0.200
IBD	STMN3	20	62271061	rs909333	62270637	G	T	0.207	0.555	0.087	2.01E-10	0.055
UC	STMN3	20	62271061	rs909333	62270637	G	T	0.207	0.498	0.098	4.04E-07	0.062
GORD	PBX2	6	32154964	rs693906	31835164	C	G	0.113	0.154	0.036	1.65E-05	0.058
VHD	AGER	6	32150423	rs693906	31835164	C	G	0.113	0.212	0.052	4.82E-05	0.092

B) Nominally significant effect on VHD of Bonferroni-significant gastrointestinal loci.

Disease	Gene	chr	Probe_bp	topSNP	topSNP_bp	A1	A2	Freq	b_SMR	se_SMR	p_SMR	p_HEIDI
VHD	CFDP1	16	75397489	rs7201989	75471499	G	A	0.246	-0.056	0.018	0.001	0.119
VHD	STAT3	17	40502964	rs1053004	40466092	G	A	0.380	0.093	0.047	0.048	0.948
VHD	IMPDH2	3	49064299	rs34037363	48821036	T	C	0.114	-0.209	0.105	0.047	0.089
VHD	UBE2L3	22	21941029	rs2070512	21949411	C	A	0.194	-0.029	0.009	0.002	0.623
VHD	STMN3	20	62271061	rs909333	62270637	G	T	0.207	-0.076	0.036	0.036	0.093

C) Effect of AGER gene on VHD and gastrointestinal disorders.

Disease	Gene	chr	Probe_bp	topSNP	topSNP_bp	A1	A2	Freq	b_SMR	se_SMR	p_SMR	p_HEIDI
CD	AGER	6	32150423	rs693906	31835164	C	G	0.113	-0.312	0.120	0.009	6.16E-08
GORD	AGER	6	32150423	rs693906	31835164	C	G	0.113	-0.209	0.049	2.02E-05	0.022
IBD	AGER	6	32148745	rs204995	32154285	G	A	0.217	-0.252	0.108	0.020	1.60E-04
IBS	AGER	6	32150423	rs693906	31835164	C	G	0.113	-0.168	0.052	0.001	0.045
UC	AGER	6	32148745	rs204995	32154285	G	A	0.217	-0.870	0.185	2.59E-06	0.002
VHD	AGER	6	32150423	rs693906	31835164	C	G	0.113	0.212	0.052	4.82E-05	0.092

Supplementary table 6. Results of the summary-based Mendelian randomization integrating whole-blood expression quantitative trait loci. For each disease-gene association, we report the strongest effect observed between GTEX and eQTLGen datasets.

A) Bonferroni-significant disease-gene association with HEIDI P > 0.05.

Disease	Gene	chr	gene_bp	topSNP	topSNP_bp	A1	A2	Freq	b_SMR	se_SMR	p_SMR	p_HEIDI
CD	DNMT3A	2	25510652	rs7578575	25488819	A	T	0.329	-0.834	0.164	3.51E-07	0.383
IBD	DNMT3A	2	25510652	rs7578575	25488819	A	T	0.329	-0.745	0.130	9.22E-09	0.515
UC	DNMT3A	2	25510652	rs7578575	25488819	A	T	0.329	-0.627	0.156	5.73E-05	0.541
UC	CFDP1	16	75327596	rs9934007	75390532	C	T	0.754	0.273	0.070	9.61E-05	0.973
CD	STAT3	17	40502964	rs1053004	40466092	G	A	0.380	0.702	0.128	4.40E-08	0.056
CD	IMPDH2	3	49064299	rs9813813	49207093	A	T	0.110	-1.233	0.294	2.74E-05	0.213
IBD	IMPDH2	3	49064299	rs9813813	49207093	A	T	0.110	-1.328	0.258	2.57E-07	0.071
UC	IMPDH2	3	49064299	rs9813813	49207093	A	T	0.110	-1.445	0.307	2.53E-06	0.050
IBD	UBE2L3	22	21941029	rs2070512	21949411	C	A	0.194	0.163	0.021	1.06E-14	0.169
UC	UBE2L3	22	21941029	rs2070512	21949411	C	A	0.194	0.116	0.027	1.76E-05	0.698
CD	BCL2L15	1	1.14E+08	rs1217393	1.14E+08	A	G	0.271	-0.705	0.170	3.39E-05	0.184
CD	STMN3	20	62271061	rs909333	62270637	G	T	0.207	0.547	0.102	8.31E-08	0.200
IBD	STMN3	20	62271061	rs909333	62270637	G	T	0.207	0.555	0.087	2.01E-10	0.055
UC	STMN3	20	62271061	rs909333	62270637	G	T	0.207	0.498	0.098	4.04E-07	0.062
GORD	PBX2	6	32154964	rs693906	31835164	C	G	0.113	0.154	0.036	1.65E-05	0.058
VHD	AGER	6	32150423	rs693906	31835164	C	G	0.113	0.212	0.052	4.82E-05	0.092

B) Nominally significant effect on VHD of Bonferroni-significant gastrointestinal loci.

Disease	Gene	chr	Probe_bp	topSNP	topSNP_bp	A1	A2	Freq	b_SMR	se_SMR	p_SMR	p_HEIDI
VHD	CFDP1	16	75397489	rs7201989	75471499	G	A	0.246	-0.056	0.018	0.001	0.119
VHD	STAT3	17	40502964	rs1053004	40466092	G	A	0.380	0.093	0.047	0.048	0.948
VHD	IMPDH2	3	49064299	rs34037363	48821036	T	C	0.114	-0.209	0.105	0.047	0.089
VHD	UBE2L3	22	21941029	rs2070512	21949411	C	A	0.194	-0.029	0.009	0.002	0.623
VHD	STMN3	20	62271061	rs909333	62270637	G	T	0.207	-0.076	0.036	0.036	0.093

C) Effect of AGER gene on VHD and gastrointestinal disorders.

Disease	Gene	chr	Probe_bp	topSNP	topSNP_bp	A1	A2	Freq	b_SMR	se_SMR	p_SMR	p_HEIDI
CD	AGER	6	32150423	rs693906	31835164	C	G	0.113	-0.312	0.120	0.009	6.16E-08
GORD	AGER	6	32150423	rs693906	31835164	C	G	0.113	-0.209	0.049	2.02E-05	0.022
IBD	AGER	6	32148745	rs204995	32154285	G	A	0.217	-0.252	0.108	0.020	1.60E-04
IBS	AGER	6	32150423	rs693906	31835164	C	G	0.113	-0.168	0.052	0.001	0.045
UC	AGER	6	32148745	rs204995	32154285	G	A	0.217	-0.870	0.185	2.59E-06	0.002
VHD	AGER	6	32150423	rs693906	31835164	C	G	0.113	0.212	0.052	4.82E-05	0.092

Supplementary table 6. Results of the summary-based Mendelian randomization integrating whole-blood expression quantitative trait loci. For each disease-gene association, we report the strongest effect observed between GTEX and eQTLGen datasets.

A) Bonferroni-significant disease-gene association with HEIDI P > 0.05.

Disease	Gene	chr	gene_bp	topSNP	topSNP_bp	A1	A2	Freq	b_SMR	se_SMR	p_SMR	p_HEIDI
CD	DNMT3A	2	25510652	rs7578575	25488819	A	T	0.329	-0.834	0.164	3.51E-07	0.383
IBD	DNMT3A	2	25510652	rs7578575	25488819	A	T	0.329	-0.745	0.130	9.22E-09	0.515
UC	DNMT3A	2	25510652	rs7578575	25488819	A	T	0.329	-0.627	0.156	5.73E-05	0.541
UC	CFDP1	16	75327596	rs9934007	75390532	C	T	0.754	0.273	0.070	9.61E-05	0.973
CD	STAT3	17	40502964	rs1053004	40466092	G	A	0.380	0.702	0.128	4.40E-08	0.056
CD	IMPDH2	3	49064299	rs9813813	49207093	A	T	0.110	-1.233	0.294	2.74E-05	0.213
IBD	IMPDH2	3	49064299	rs9813813	49207093	A	T	0.110	-1.328	0.258	2.57E-07	0.071
UC	IMPDH2	3	49064299	rs9813813	49207093	A	T	0.110	-1.445	0.307	2.53E-06	0.050
IBD	UBE2L3	22	21941029	rs2070512	21949411	C	A	0.194	0.163	0.021	1.06E-14	0.169
UC	UBE2L3	22	21941029	rs2070512	21949411	C	A	0.194	0.116	0.027	1.76E-05	0.698
CD	BCL2L15	1	1.14E+08	rs1217393	1.14E+08	A	G	0.271	-0.705	0.170	3.39E-05	0.184
CD	STMN3	20	62271061	rs909333	62270637	G	T	0.207	0.547	0.102	8.31E-08	0.200
IBD	STMN3	20	62271061	rs909333	62270637	G	T	0.207	0.555	0.087	2.01E-10	0.055
UC	STMN3	20	62271061	rs909333	62270637	G	T	0.207	0.498	0.098	4.04E-07	0.062
GORD	PBX2	6	32154964	rs693906	31835164	C	G	0.113	0.154	0.036	1.65E-05	0.058
VHD	AGER	6	32150423	rs693906	31835164	C	G	0.113	0.212	0.052	4.82E-05	0.092

B) Nominally significant effect on VHD of Bonferroni-significant gastrointestinal loci.

Disease	Gene	chr	Probe_bp	topSNP	topSNP_bp	A1	A2	Freq	b_SMR	se_SMR	p_SMR	p_HEIDI
VHD	CFDP1	16	75397489	rs7201989	75471499	G	A	0.246	-0.056	0.018	0.001	0.119
VHD	STAT3	17	40502964	rs1053004	40466092	G	A	0.380	0.093	0.047	0.048	0.948
VHD	IMPDH2	3	49064299	rs34037363	48821036	T	C	0.114	-0.209	0.105	0.047	0.089
VHD	UBE2L3	22	21941029	rs2070512	21949411	C	A	0.194	-0.029	0.009	0.002	0.623
VHD	STMN3	20	62271061	rs909333	62270637	G	T	0.207	-0.076	0.036	0.036	0.093

C) Effect of AGER gene on VHD and gastrointestinal disorders.

Disease	Gene	chr	Probe_bp	topSNP	topSNP_bp	A1	A2	Freq	b_SMR	se_SMR	p_SMR	p_HEIDI
CD	AGER	6	32150423	rs693906	31835164	C	G	0.113	-0.312	0.120	0.009	6.16E-08
GORD	AGER	6	32150423	rs693906	31835164	C	G	0.113	-0.209	0.049	2.02E-05	0.022
IBD	AGER	6	32148745	rs204995	32154285	G	A	0.217	-0.252	0.108	0.020	1.60E-04
IBS	AGER	6	32150423	rs693906	31835164	C	G	0.113	-0.168	0.052	0.001	0.045
UC	AGER	6	32148745	rs204995	32154285	G	A	0.217	-0.870	0.185	2.59E-06	0.002
VHD	AGER	6	32150423	rs693906	31835164	C	G	0.113	0.212	0.052	4.82E-05	0.092

Supplementary table 6. Results of the summary-based Mendelian randomization integrating whole-blood expression quantitative trait loci. For each disease-gene association, we report the strongest effect observed between GTEX and eQTLGen datasets.

A) Bonferroni-significant disease-gene association with HEIDI P > 0.05.

Disease	Gene	chr	gene_bp	topSNP	topSNP_bp	A1	A2	Freq	b_SMR	se_SMR	p_SMR	p_HEIDI
CD	DNMT3A	2	25510652	rs7578575	25488819	A	T	0.329	-0.834	0.164	3.51E-07	0.383
IBD	DNMT3A	2	25510652	rs7578575	25488819	A	T	0.329	-0.745	0.130	9.22E-09	0.515
UC	DNMT3A	2	25510652	rs7578575	25488819	A	T	0.329	-0.627	0.156	5.73E-05	0.541
UC	CFDP1	16	75327596	rs9934007	75390532	C	T	0.754	0.273	0.070	9.61E-05	0.973
CD	STAT3	17	40502964	rs1053004	40466092	G	A	0.380	0.702	0.128	4.40E-08	0.056
CD	IMPDH2	3	49064299	rs9813813	49207093	A	T	0.110	-1.233	0.294	2.74E-05	0.213
IBD	IMPDH2	3	49064299	rs9813813	49207093	A	T	0.110	-1.328	0.258	2.57E-07	0.071
UC	IMPDH2	3	49064299	rs9813813	49207093	A	T	0.110	-1.445	0.307	2.53E-06	0.050
IBD	UBE2L3	22	21941029	rs2070512	21949411	C	A	0.194	0.163	0.021	1.06E-14	0.169
UC	UBE2L3	22	21941029	rs2070512	21949411	C	A	0.194	0.116	0.027	1.76E-05	0.698
CD	BCL2L15	1	1.14E+08	rs1217393	1.14E+08	A	G	0.271	-0.705	0.170	3.39E-05	0.184
CD	STMN3	20	62271061	rs909333	62270637	G	T	0.207	0.547	0.102	8.31E-08	0.200
IBD	STMN3	20	62271061	rs909333	62270637	G	T	0.207	0.555	0.087	2.01E-10	0.055
UC	STMN3	20	62271061	rs909333	62270637	G	T	0.207	0.498	0.098	4.04E-07	0.062
GORD	PBX2	6	32154964	rs693906	31835164	C	G	0.113	0.154	0.036	1.65E-05	0.058
VHD	AGER	6	32150423	rs693906	31835164	C	G	0.113	0.212	0.052	4.82E-05	0.092

B) Nominally significant effect on VHD of Bonferroni-significant gastrointestinal loci.

Disease	Gene	chr	Probe_bp	topSNP	topSNP_bp	A1	A2	Freq	b_SMR	se_SMR	p_SMR	p_HEIDI
VHD	CFDP1	16	75397489	rs7201989	75471499	G	A	0.246	-0.056	0.018	0.001	0.119
VHD	STAT3	17	40502964	rs1053004	40466092	G	A	0.380	0.093	0.047	0.048	0.948
VHD	IMPDH2	3	49064299	rs34037363	48821036	T	C	0.114	-0.209	0.105	0.047	0.089
VHD	UBE2L3	22	21941029	rs2070512	21949411	C	A	0.194	-0.029	0.009	0.002	0.623
VHD	STMN3	20	62271061	rs909333	62270637	G	T	0.207	-0.076	0.036	0.036	0.093

C) Effect of AGER gene on VHD and gastrointestinal disorders.

Disease	Gene	chr	Probe_bp	topSNP	topSNP_bp	A1	A2	Freq	b_SMR	se_SMR	p_SMR	p_HEIDI
CD	AGER	6	32150423	rs693906	31835164	C	G	0.113	-0.312	0.120	0.009	6.16E-08
GORD	AGER	6	32150423	rs693906	31835164	C	G	0.113	-0.209	0.049	2.02E-05	0.022
IBD	AGER	6	32148745	rs204995	32154285	G	A	0.217	-0.252	0.108	0.020	1.60E-04
IBS	AGER	6	32150423	rs693906	31835164	C	G	0.113	-0.168	0.052	0.001	0.045
UC	AGER	6	32148745	rs204995	32154285	G	A	0.217	-0.870	0.185	2.59E-06	0.002
VHD	AGER	6	32150423	rs693906	31835164	C	G	0.113	0.212	0.052	4.82E-05	0.092

Supplementary table 6. Results of the summary-based Mendelian randomization integrating whole-blood expression quantitative trait loci. For each disease-gene association, we report the strongest effect observed between GTEX and eQTLGen datasets.

A) Bonferroni-significant disease-gene association with HEIDI P > 0.05.

Disease	Gene	chr	gene_bp	topSNP	topSNP_bp	A1	A2	Freq	b_SMR	se_SMR	p_SMR	p_HEIDI
CD	DNMT3A	2	25510652	rs7578575	25488819	A	T	0.329	-0.834	0.164	3.51E-07	0.383
IBD	DNMT3A	2	25510652	rs7578575	25488819	A	T	0.329	-0.745	0.130	9.22E-09	0.515
UC	DNMT3A	2	25510652	rs7578575	25488819	A	T	0.329	-0.627	0.156	5.73E-05	0.541
UC	CFDP1	16	75327596	rs9934007	75390532	C	T	0.754	0.273	0.070	9.61E-05	0.973
CD	STAT3	17	40502964	rs1053004	40466092	G	A	0.380	0.702	0.128	4.40E-08	0.056
CD	IMPDH2	3	49064299	rs9813813	49207093	A	T	0.110	-1.233	0.294	2.74E-05	0.213
IBD	IMPDH2	3	49064299	rs9813813	49207093	A	T	0.110	-1.328	0.258	2.57E-07	0.071
UC	IMPDH2	3	49064299	rs9813813	49207093	A	T	0.110	-1.445	0.307	2.53E-06	0.050
IBD	UBE2L3	22	21941029	rs2070512	21949411	C	A	0.194	0.163	0.021	1.06E-14	0.169
UC	UBE2L3	22	21941029	rs2070512	21949411	C	A	0.194	0.116	0.027	1.76E-05	0.698
CD	BCL2L15	1	1.14E+08	rs1217393	1.14E+08	A	G	0.271	-0.705	0.170	3.39E-05	0.184
CD	STMN3	20	62271061	rs909333	62270637	G	T	0.207	0.547	0.102	8.31E-08	0.200
IBD	STMN3	20	62271061	rs909333	62270637	G	T	0.207	0.555	0.087	2.01E-10	0.055
UC	STMN3	20	62271061	rs909333	62270637	G	T	0.207	0.498	0.098	4.04E-07	0.062
GORD	PBX2	6	32154964	rs693906	31835164	C	G	0.113	0.154	0.036	1.65E-05	0.058
VHD	AGER	6	32150423	rs693906	31835164	C	G	0.113	0.212	0.052	4.82E-05	0.092

B) Nominally significant effect on VHD of Bonferroni-significant gastrointestinal loci.

Disease	Gene	chr	Probe_bp	topSNP	topSNP_bp	A1	A2	Freq	b_SMR	se_SMR	p_SMR	p_HEIDI
VHD	CFDP1	16	75397489	rs7201989	75471499	G	A	0.246	-0.056	0.018	0.001	0.119
VHD	STAT3	17	40502964	rs1053004	40466092	G	A	0.380	0.093	0.047	0.048	0.948
VHD	IMPDH2	3	49064299	rs34037363	48821036	T	C	0.114	-0.209	0.105	0.047	0.089
VHD	UBE2L3	22	21941029	rs2070512	21949411	C	A	0.194	-0.029	0.009	0.002	0.623
VHD	STMN3	20	62271061	rs909333	62270637	G	T	0.207	-0.076	0.036	0.036	0.093

C) Effect of AGER gene on VHD and gastrointestinal disorders.

Disease	Gene	chr	Probe_bp	topSNP	topSNP_bp	A1	A2	Freq	b_SMR	se_SMR	p_SMR	p_HEIDI
CD	AGER	6	32150423	rs693906	31835164	C	G	0.113	-0.312	0.120	0.009	6.16E-08
GORD	AGER	6	32150423	rs693906	31835164	C	G	0.113	-0.209	0.049	2.02E-05	0.022
IBD	AGER	6	32148745	rs204995	32154285	G	A	0.217	-0.252	0.108	0.020	1.60E-04
IBS	AGER	6	32150423	rs693906	31835164	C	G	0.113	-0.168	0.052	0.001	0.045
UC	AGER	6	32148745	rs204995	32154285	G	A	0.217	-0.870	0.185	2.59E-06	0.002
VHD	AGER	6	32150423	rs693906	31835164	C	G	0.113	0.212	0.052	4.82E-05	0.092

Supplementary table 6. Results of the summary-based Mendelian randomization integrating whole-blood expression quantitative trait loci. For each disease-gene association, we report the strongest effect observed between GTEX and eQTLGen datasets.

A) Bonferroni-significant disease-gene association with HEIDI P > 0.05.

Disease	Gene	chr	gene_bp	topSNP	topSNP_bp	A1	A2	Freq	b_SMR	se_SMR	p_SMR	p_HEIDI
CD	DNMT3A	2	25510652	rs7578575	25488819	A	T	0.329	-0.834	0.164	3.51E-07	0.383
IBD	DNMT3A	2	25510652	rs7578575	25488819	A	T	0.329	-0.745	0.130	9.22E-09	0.515
UC	DNMT3A	2	25510652	rs7578575	25488819	A	T	0.329	-0.627	0.156	5.73E-05	0.541
UC	CFDP1	16	75327596	rs9934007	75390532	C	T	0.754	0.273	0.070	9.61E-05	0.973
CD	STAT3	17	40502964	rs1053004	40466092	G	A	0.380	0.702	0.128	4.40E-08	0.056
CD	IMPDH2	3	49064299	rs9813813	49207093	A	T	0.110	-1.233	0.294	2.74E-05	0.213
IBD	IMPDH2	3	49064299	rs9813813	49207093	A	T	0.110	-1.328	0.258	2.57E-07	0.071
UC	IMPDH2	3	49064299	rs9813813	49207093	A	T	0.110	-1.445	0.307	2.53E-06	0.050
IBD	UBE2L3	22	21941029	rs2070512	21949411	C	A	0.194	0.163	0.021	1.06E-14	0.169
UC	UBE2L3	22	21941029	rs2070512	21949411	C	A	0.194	0.116	0.027	1.76E-05	0.698
CD	BCL2L15	1	1.14E+08	rs1217393	1.14E+08	A	G	0.271	-0.705	0.170	3.39E-05	0.184
CD	STMN3	20	62271061	rs909333	62270637	G	T	0.207	0.547	0.102	8.31E-08	0.200
IBD	STMN3	20	62271061	rs909333	62270637	G	T	0.207	0.555	0.087	2.01E-10	0.055
UC	STMN3	20	62271061	rs909333	62270637	G	T	0.207	0.498	0.098	4.04E-07	0.062
GORD	PBX2	6	32154964	rs693906	31835164	C	G	0.113	0.154	0.036	1.65E-05	0.058
VHD	AGER	6	32150423	rs693906	31835164	C	G	0.113	0.212	0.052	4.82E-05	0.092

B) Nominally significant effect on VHD of Bonferroni-significant gastrointestinal loci.

Disease	Gene	chr	Probe_bp	topSNP	topSNP_bp	A1	A2	Freq	b_SMR	se_SMR	p_SMR	p_HEIDI
VHD	CFDP1	16	75397489	rs7201989	75471499	G	A	0.246	-0.056	0.018	0.001	0.119
VHD	STAT3	17	40502964	rs1053004	40466092	G	A	0.380	0.093	0.047	0.048	0.948
VHD	IMPDH2	3	49064299	rs34037363	48821036	T	C	0.114	-0.209	0.105	0.047	0.089
VHD	UBE2L3	22	21941029	rs2070512	21949411	C	A	0.194	-0.029	0.009	0.002	0.623
VHD	STMN3	20	62271061	rs909333	62270637	G	T	0.207	-0.076	0.036	0.036	0.093

C) Effect of AGER gene on VHD and gastrointestinal disorders.

Disease	Gene	chr	Probe_bp	topSNP	topSNP_bp	A1	A2	Freq	b_SMR	se_SMR	p_SMR	p_HEIDI
CD	AGER	6	32150423	rs693906	31835164	C	G	0.113	-0.312	0.120	0.009	6.16E-08
GORD	AGER	6	32150423	rs693906	31835164	C	G	0.113	-0.209	0.049	2.02E-05	0.022
IBD	AGER	6	32148745	rs204995	32154285	G	A	0.217	-0.252	0.108	0.020	1.60E-04
IBS	AGER	6	32150423	rs693906	31835164	C	G	0.113	-0.168	0.052	0.001	0.045
UC	AGER	6	32148745	rs204995	32154285	G	A	0.217	-0.870	0.185	2.59E-06	0.002
VHD	AGER	6	32150423	rs693906	31835164	C	G	0.113	0.212	0.052	4.82E-05	0.092

Supplementary table 6. Results of the summary-based Mendelian randomization integrating whole-blood expression quantitative trait loci. For each disease-gene association, we report the strongest effect observed between GTEX and eQTLGen datasets.

A) Bonferroni-significant disease-gene association with HEIDI P > 0.05.

Disease	Gene	chr	gene_bp	topSNP	topSNP_bp	A1	A2	Freq	b_SMR	se_SMR	p_SMR	p_HEIDI
CD	DNMT3A	2	25510652	rs7578575	25488819	A	T	0.329	-0.834	0.164	3.51E-07	0.383
IBD	DNMT3A	2	25510652	rs7578575	25488819	A	T	0.329	-0.745	0.130	9.22E-09	0.515
UC	DNMT3A	2	25510652	rs7578575	25488819	A	T	0.329	-0.627	0.156	5.73E-05	0.541
UC	CFDP1	16	75327596	rs9934007	75390532	C	T	0.754	0.273	0.070	9.61E-05	0.973
CD	STAT3	17	40502964	rs1053004	40466092	G	A	0.380	0.702	0.128	4.40E-08	0.056
CD	IMPDH2	3	49064299	rs9813813	49207093	A	T	0.110	-1.233	0.294	2.74E-05	0.213
IBD	IMPDH2	3	49064299	rs9813813	49207093	A	T	0.110	-1.328	0.258	2.57E-07	0.071
UC	IMPDH2	3	49064299	rs9813813	49207093	A	T	0.110	-1.445	0.307	2.53E-06	0.050
IBD	UBE2L3	22	21941029	rs2070512	21949411	C	A	0.194	0.163	0.021	1.06E-14	0.169
UC	UBE2L3	22	21941029	rs2070512	21949411	C	A	0.194	0.116	0.027	1.76E-05	0.698
CD	BCL2L15	1	1.14E+08	rs1217393	1.14E+08	A	G	0.271	-0.705	0.170	3.39E-05	0.184
CD	STMN3	20	62271061	rs909333	62270637	G	T	0.207	0.547	0.102	8.31E-08	0.200
IBD	STMN3	20	62271061	rs909333	62270637	G	T	0.207	0.555	0.087	2.01E-10	0.055
UC	STMN3	20	62271061	rs909333	62270637	G	T	0.207	0.498	0.098	4.04E-07	0.062
GORD	PBX2	6	32154964	rs693906	31835164	C	G	0.113	0.154	0.036	1.65E-05	0.058
VHD	AGER	6	32150423	rs693906	31835164	C	G	0.113	0.212	0.052	4.82E-05	0.092

B) Nominally significant effect on VHD of Bonferroni-significant gastrointestinal loci.

Disease	Gene	chr	Probe_bp	topSNP	topSNP_bp	A1	A2	Freq	b_SMR	se_SMR	p_SMR	p_HEIDI
VHD	CFDP1	16	75397489	rs7201989	75471499	G	A	0.246	-0.056	0.018	0.001	0.119
VHD	STAT3	17	40502964	rs1053004	40466092	G	A	0.380	0.093	0.047	0.048	0.948
VHD	IMPDH2	3	49064299	rs34037363	48821036	T	C	0.114	-0.209	0.105	0.047	0.089
VHD	UBE2L3	22	21941029	rs2070512	21949411	C	A	0.194	-0.029	0.009	0.002	0.623
VHD	STMN3	20	62271061	rs909333	62270637	G	T	0.207	-0.076	0.036	0.036	0.093

C) Effect of AGER gene on VHD and gastrointestinal disorders.

Disease	Gene	chr	Probe_bp	topSNP	topSNP_bp	A1	A2	Freq	b_SMR	se_SMR	p_SMR	p_HEIDI
CD	AGER	6	32150423	rs693906	31835164	C	G	0.113	-0.312	0.120	0.009	6.16E-08
GORD	AGER	6	32150423	rs693906	31835164	C	G	0.113	-0.209	0.049	2.02E-05	0.022
IBD	AGER	6	32148745	rs204995	32154285	G	A	0.217	-0.252	0.108	0.020	1.60E-04
IBS	AGER	6	32150423	rs693906	31835164	C	G	0.113	-0.168	0.052	0.001	0.045
UC	AGER	6	32148745	rs204995	32154285	G	A	0.217	-0.870	0.185	2.59E-06	0.002
VHD	AGER	6	32150423	rs693906	31835164	C	G	0.113	0.212	0.052	4.82E-05	0.092

Supplementary table 6. Results of the summary-based Mendelian randomization integrating whole-blood expression quantitative trait loci. For each disease-gene association, we report the strongest effect observed between GTEX and eQTLGen datasets.

A) Bonferroni-significant disease-gene association with HEIDI P > 0.05.

Disease	Gene	chr	gene_bp	topSNP	topSNP_bp	A1	A2	Freq	b_SMR	se_SMR	p_SMR	p_HEIDI
CD	DNMT3A	2	25510652	rs7578575	25488819	A	T	0.329	-0.834	0.164	3.51E-07	0.383
IBD	DNMT3A	2	25510652	rs7578575	25488819	A	T	0.329	-0.745	0.130	9.22E-09	0.515
UC	DNMT3A	2	25510652	rs7578575	25488819	A	T	0.329	-0.627	0.156	5.73E-05	0.541
UC	CFDP1	16	75327596	rs9934007	75390532	C	T	0.754	0.273	0.070	9.61E-05	0.973
CD	STAT3	17	40502964	rs1053004	40466092	G	A	0.380	0.702	0.128	4.40E-08	0.056
CD	IMPDH2	3	49064299	rs9813813	49207093	A	T	0.110	-1.233	0.294	2.74E-05	0.213
IBD	IMPDH2	3	49064299	rs9813813	49207093	A	T	0.110	-1.328	0.258	2.57E-07	0.071
UC	IMPDH2	3	49064299	rs9813813	49207093	A	T	0.110	-1.445	0.307	2.53E-06	0.050
IBD	UBE2L3	22	21941029	rs2070512	21949411	C	A	0.194	0.163	0.021	1.06E-14	0.169
UC	UBE2L3	22	21941029	rs2070512	21949411	C	A	0.194	0.116	0.027	1.76E-05	0.698
CD	BCL2L15	1	1.14E+08	rs1217393	1.14E+08	A	G	0.271	-0.705	0.170	3.39E-05	0.184
CD	STMN3	20	62271061	rs909333	62270637	G	T	0.207	0.547	0.102	8.31E-08	0.200
IBD	STMN3	20	62271061	rs909333	62270637	G	T	0.207	0.555	0.087	2.01E-10	0.055
UC	STMN3	20	62271061	rs909333	62270637	G	T	0.207	0.498	0.098	4.04E-07	0.062
GORD	PBX2	6	32154964	rs693906	31835164	C	G	0.113	0.154	0.036	1.65E-05	0.058
VHD	AGER	6	32150423	rs693906	31835164	C	G	0.113	0.212	0.052	4.82E-05	0.092

B) Nominally significant effect on VHD of Bonferroni-significant gastrointestinal loci.

Disease	Gene	chr	Probe_bp	topSNP	topSNP_bp	A1	A2	Freq	b_SMR	se_SMR	p_SMR	p_HEIDI
VHD	CFDP1	16	75397489	rs7201989	75471499	G	A	0.246	-0.056	0.018	0.001	0.119
VHD	STAT3	17	40502964	rs1053004	40466092	G	A	0.380	0.093	0.047	0.048	0.948
VHD	IMPDH2	3	49064299	rs34037363	48821036	T	C	0.114	-0.209	0.105	0.047	0.089
VHD	UBE2L3	22	21941029	rs2070512	21949411	C	A	0.194	-0.029	0.009	0.002	0.623
VHD	STMN3	20	62271061	rs909333	62270637	G	T	0.207	-0.076	0.036	0.036	0.093

C) Effect of AGER gene on VHD and gastrointestinal disorders.

Disease	Gene	chr	Probe_bp	topSNP	topSNP_bp	A1	A2	Freq	b_SMR	se_SMR	p_SMR	p_HEIDI
CD	AGER	6	32150423	rs693906	31835164	C	G	0.113	-0.312	0.120	0.009	6.16E-08
GORD	AGER	6	32150423	rs693906	31835164	C	G	0.113	-0.209	0.049	2.02E-05	0.022
IBD	AGER	6	32148745	rs204995	32154285	G	A	0.217	-0.252	0.108	0.020	1.60E-04
IBS	AGER	6	32150423	rs693906	31835164	C	G	0.113	-0.168	0.052	0.001	0.045
UC	AGER	6	32148745	rs204995	32154285	G	A	0.217	-0.870	0.185	2.59E-06	0.002
VHD	AGER	6	32150423	rs693906	31835164	C	G	0.113	0.212	0.052	4.82E-05	0.092

Supplementary table 6. Results of the summary-based Mendelian randomization integrating whole-blood expression quantitative trait loci. For each disease-gene association, we report the strongest effect observed between GTEX and eQTLGen datasets.

A) Bonferroni-significant disease-gene association with HEIDI P > 0.05.

Disease	Gene	chr	gene_bp	topSNP	topSNP_bp	A1	A2	Freq	b_SMR	se_SMR	p_SMR	p_HEIDI
CD	DNMT3A	2	25510652	rs7578575	25488819	A	T	0.329	-0.834	0.164	3.51E-07	0.383
IBD	DNMT3A	2	25510652	rs7578575	25488819	A	T	0.329	-0.745	0.130	9.22E-09	0.515
UC	DNMT3A	2	25510652	rs7578575	25488819	A	T	0.329	-0.627	0.156	5.73E-05	0.541
UC	CFDP1	16	75327596	rs9934007	75390532	C	T	0.754	0.273	0.070	9.61E-05	0.973
CD	STAT3	17	40502964	rs1053004	40466092	G	A	0.380	0.702	0.128	4.40E-08	0.056
CD	IMPDH2	3	49064299	rs9813813	49207093	A	T	0.110	-1.233	0.294	2.74E-05	0.213
IBD	IMPDH2	3	49064299	rs9813813	49207093	A	T	0.110	-1.328	0.258	2.57E-07	0.071
UC	IMPDH2	3	49064299	rs9813813	49207093	A	T	0.110	-1.445	0.307	2.53E-06	0.050
IBD	UBE2L3	22	21941029	rs2070512	21949411	C	A	0.194	0.163	0.021	1.06E-14	0.169
UC	UBE2L3	22	21941029	rs2070512	21949411	C	A	0.194	0.116	0.027	1.76E-05	0.698
CD	BCL2L15	1	1.14E+08	rs1217393	1.14E+08	A	G	0.271	-0.705	0.170	3.39E-05	0.184
CD	STMN3	20	62271061	rs909333	62270637	G	T	0.207	0.547	0.102	8.31E-08	0.200
IBD	STMN3	20	62271061	rs909333	62270637	G	T	0.207	0.555	0.087	2.01E-10	0.055
UC	STMN3	20	62271061	rs909333	62270637	G	T	0.207	0.498	0.098	4.04E-07	0.062
GORD	PBX2	6	32154964	rs693906	31835164	C	G	0.113	0.154	0.036	1.65E-05	0.058
VHD	AGER	6	32150423	rs693906	31835164	C	G	0.113	0.212	0.052	4.82E-05	0.092

B) Nominally significant effect on VHD of Bonferroni-significant gastrointestinal loci.

Disease	Gene	chr	Probe_bp	topSNP	topSNP_bp	A1	A2	Freq	b_SMR	se_SMR	p_SMR	p_HEIDI
VHD	CFDP1	16	75397489	rs7201989	75471499	G	A	0.246	-0.056	0.018	0.001	0.119
VHD	STAT3	17	40502964	rs1053004	40466092	G	A	0.380	0.093	0.047	0.048	0.948
VHD	IMPDH2	3	49064299	rs34037363	48821036	T	C	0.114	-0.209	0.105	0.047	0.089
VHD	UBE2L3	22	21941029	rs2070512	21949411	C	A	0.194	-0.029	0.009	0.002	0.623
VHD	STMN3	20	62271061	rs909333	62270637	G	T	0.207	-0.076	0.036	0.036	0.093

C) Effect of AGER gene on VHD and gastrointestinal disorders.

Disease	Gene	chr	Probe_bp	topSNP	topSNP_bp	A1	A2	Freq	b_SMR	se_SMR	p_SMR	p_HEIDI
CD	AGER	6	32150423	rs693906	31835164	C	G	0.113	-0.312	0.120	0.009	6.16E-08
GORD	AGER	6	32150423	rs693906	31835164	C	G	0.113	-0.209	0.049	2.02E-05	0.022
IBD	AGER	6	32148745	rs204995	32154285	G	A	0.217	-0.252	0.108	0.020	1.60E-04
IBS	AGER	6	32150423	rs693906	31835164	C	G	0.113	-0.168	0.052	0.001	0.045
UC	AGER	6	32148745	rs204995	32154285	G	A	0.217	-0.870	0.185	2.59E-06	0.002
VHD	AGER	6	32150423	rs693906	31835164	C	G	0.113	0.212	0.052	4.82E-05	0.092

Supplementary table 6. Results of the summary-based Mendelian randomization integrating whole-blood expression quantitative trait loci. For each disease-gene association, we report the strongest effect observed between GTEX and eQTLGen datasets.

A) Bonferroni-significant disease-gene association with HEIDI P > 0.05.

Disease	Gene	chr	gene_bp	topSNP	topSNP_bp	A1	A2	Freq	b_SMR	se_SMR	p_SMR	p_HEIDI
CD	DNMT3A	2	25510652	rs7578575	25488819	A	T	0.329	-0.834	0.164	3.51E-07	0.383
IBD	DNMT3A	2	25510652	rs7578575	25488819	A	T	0.329	-0.745	0.130	9.22E-09	0.515
UC	DNMT3A	2	25510652	rs7578575	25488819	A	T	0.329	-0.627	0.156	5.73E-05	0.541
UC	CFDP1	16	75327596	rs9934007	75390532	C	T	0.754	0.273	0.070	9.61E-05	0.973
CD	STAT3	17	40502964	rs1053004	40466092	G	A	0.380	0.702	0.128	4.40E-08	0.056
CD	IMPDH2	3	49064299	rs9813813	49207093	A	T	0.110	-1.233	0.294	2.74E-05	0.213
IBD	IMPDH2	3	49064299	rs9813813	49207093	A	T	0.110	-1.328	0.258	2.57E-07	0.071
UC	IMPDH2	3	49064299	rs9813813	49207093	A	T	0.110	-1.445	0.307	2.53E-06	0.050
IBD	UBE2L3	22	21941029	rs2070512	21949411	C	A	0.194	0.163	0.021	1.06E-14	0.169
UC	UBE2L3	22	21941029	rs2070512	21949411	C	A	0.194	0.116	0.027	1.76E-05	0.698
CD	BCL2L15	1	1.14E+08	rs1217393	1.14E+08	A	G	0.271	-0.705	0.170	3.39E-05	0.184
CD	STMN3	20	62271061	rs909333	62270637	G	T	0.207	0.547	0.102	8.31E-08	0.200
IBD	STMN3	20	62271061	rs909333	62270637	G	T	0.207	0.555	0.087	2.01E-10	0.055
UC	STMN3	20	62271061	rs909333	62270637	G	T	0.207	0.498	0.098	4.04E-07	0.062
GORD	PBX2	6	32154964	rs693906	31835164	C	G	0.113	0.154	0.036	1.65E-05	0.058
VHD	AGER	6	32150423	rs693906	31835164	C	G	0.113	0.212	0.052	4.82E-05	0.092

B) Nominally significant effect on VHD of Bonferroni-significant gastrointestinal loci.

Disease	Gene	chr	Probe_bp	topSNP	topSNP_bp	A1	A2	Freq	b_SMR	se_SMR	p_SMR	p_HEIDI
VHD	CFDP1	16	75397489	rs7201989	75471499	G	A	0.246	-0.056	0.018	0.001	0.119
VHD	STAT3	17	40502964	rs1053004	40466092	G	A	0.380	0.093	0.047	0.048	0.948
VHD	IMPDH2	3	49064299	rs34037363	48821036	T	C	0.114	-0.209	0.105	0.047	0.089
VHD	UBE2L3	22	21941029	rs2070512	21949411	C	A	0.194	-0.029	0.009	0.002	0.623
VHD	STMN3	20	62271061	rs909333	62270637	G	T	0.207	-0.076	0.036	0.036	0.093

C) Effect of AGER gene on VHD and gastrointestinal disorders.

Disease	Gene	chr	Probe_bp	topSNP	topSNP_bp	A1	A2	Freq	b_SMR	se_SMR	p_SMR	p_HEIDI
CD	AGER	6	32150423	rs693906	31835164	C	G	0.113	-0.312	0.120	0.009	6.16E-08
GORD	AGER	6	32150423	rs693906	31835164	C	G	0.113	-0.209	0.049	2.02E-05	0.022
IBD	AGER	6	32148745	rs204995	32154285	G	A	0.217	-0.252	0.108	0.020	1.60E-04
IBS	AGER	6	32150423	rs693906	31835164	C	G	0.113	-0.168	0.052	0.001	0.045
UC	AGER	6	32148745	rs204995	32154285	G	A	0.217	-0.870	0.185	2.59E-06	0.002
VHD	AGER	6	32150423	rs693906	31835164	C	G	0.113	0.212	0.052	4.82E-05	0.092

Supplementary table 6. Results of the summary-based Mendelian randomization integrating whole-blood expression quantitative trait loci. For each disease-gene association, we report the strongest effect observed between GTEX and eQTLGen datasets.

A) Bonferroni-significant disease-gene association with HEIDI P > 0.05.

Disease	Gene	chr	gene_bp	topSNP	topSNP_bp	A1	A2	Freq	b_SMR	se_SMR	p_SMR	p_HEIDI
CD	DNMT3A	2	25510652	rs7578575	25488819	A	T	0.329	-0.834	0.164	3.51E-07	0.383
IBD	DNMT3A	2	25510652	rs7578575	25488819	A	T	0.329	-0.745	0.130	9.22E-09	0.515
UC	DNMT3A	2	25510652	rs7578575	25488819	A	T	0.329	-0.627	0.156	5.73E-05	0.541
UC	CFDP1	16	75327596	rs9934007	75390532	C	T	0.754	0.273	0.070	9.61E-05	0.973
CD	STAT3	17	40502964	rs1053004	40466092	G	A	0.380	0.702	0.128	4.40E-08	0.056
CD	IMPDH2	3	49064299	rs9813813	49207093	A	T	0.110	-1.233	0.294	2.74E-05	0.213
IBD	IMPDH2	3	49064299	rs9813813	49207093	A	T	0.110	-1.328	0.258	2.57E-07	0.071
UC	IMPDH2	3	49064299	rs9813813	49207093	A	T	0.110	-1.445	0.307	2.53E-06	0.050
IBD	UBE2L3	22	21941029	rs2070512	21949411	C	A	0.194	0.163	0.021	1.06E-14	0.169
UC	UBE2L3	22	21941029	rs2070512	21949411	C	A	0.194	0.116	0.027	1.76E-05	0.698
CD	BCL2L15	1	1.14E+08	rs1217393	1.14E+08	A	G	0.271	-0.705	0.170	3.39E-05	0.184
CD	STMN3	20	62271061	rs909333	62270637	G	T	0.207	0.547	0.102	8.31E-08	0.200
IBD	STMN3	20	62271061	rs909333	62270637	G	T	0.207	0.555	0.087	2.01E-10	0.055
UC	STMN3	20	62271061	rs909333	62270637	G	T	0.207	0.498	0.098	4.04E-07	0.062
GORD	PBX2	6	32154964	rs693906	31835164	C	G	0.113	0.154	0.036	1.65E-05	0.058
VHD	AGER	6	32150423	rs693906	31835164	C	G	0.113	0.212	0.052	4.82E-05	0.092

B) Nominally significant effect on VHD of Bonferroni-significant gastrointestinal loci.

Disease	Gene	chr	Probe_bp	topSNP	topSNP_bp	A1	A2	Freq	b_SMR	se_SMR	p_SMR	p_HEIDI
VHD	CFDP1	16	75397489	rs7201989	75471499	G	A	0.246	-0.056	0.018	0.001	0.119
VHD	STAT3	17	40502964	rs1053004	40466092	G	A	0.380	0.093	0.047	0.048	0.948
VHD	IMPDH2	3	49064299	rs34037363	48821036	T	C	0.114	-0.209	0.105	0.047	0.089
VHD	UBE2L3	22	21941029	rs2070512	21949411	C	A	0.194	-0.029	0.009	0.002	0.623
VHD	STMN3	20	62271061	rs909333	62270637	G	T	0.207	-0.076	0.036	0.036	0.093

C) Effect of AGER gene on VHD and gastrointestinal disorders.

Disease	Gene	chr	Probe_bp	topSNP	topSNP_bp	A1	A2	Freq	b_SMR	se_SMR	p_SMR	p_HEIDI
CD	AGER	6	32150423	rs693906	31835164	C	G	0.113	-0.312	0.120	0.009	6.16E-08
GORD	AGER	6	32150423	rs693906	31835164	C	G	0.113	-0.209	0.049	2.02E-05	0.022
IBD	AGER	6	32148745	rs204995	32154285	G	A	0.217	-0.252	0.108	0.020	1.60E-04
IBS	AGER	6	32150423	rs693906	31835164	C	G	0.113	-0.168	0.052	0.001	0.045
UC	AGER	6	32148745	rs204995	32154285	G	A	0.217	-0.870	0.185	2.59E-06	0.002
VHD	AGER	6	32150423	rs693906	31835164	C	G	0.113	0.212	0.052	4.82E-05	0.092

Supplementary table 6. Results of the summary-based Mendelian randomization integrating whole-blood expression quantitative trait loci. For each disease-gene association, we report the strongest effect observed between GTEX and eQTLGen datasets.

A) Bonferroni-significant disease-gene association with HEIDI P > 0.05.

Disease	Gene	chr	gene_bp	topSNP	topSNP_bp	A1	A2	Freq	b_SMR	se_SMR	p_SMR	p_HEIDI
CD	DNMT3A	2	25510652	rs7578575	25488819	A	T	0.329	-0.834	0.164	3.51E-07	0.383
IBD	DNMT3A	2	25510652	rs7578575	25488819	A	T	0.329	-0.745	0.130	9.22E-09	0.515
UC	DNMT3A	2	25510652	rs7578575	25488819	A	T	0.329	-0.627	0.156	5.73E-05	0.541
UC	CFDP1	16	75327596	rs9934007	75390532	C	T	0.754	0.273	0.070	9.61E-05	0.973
CD	STAT3	17	40502964	rs1053004	40466092	G	A	0.380	0.702	0.128	4.40E-08	0.056
CD	IMPDH2	3	49064299	rs9813813	49207093	A	T	0.110	-1.233	0.294	2.74E-05	0.213
IBD	IMPDH2	3	49064299	rs9813813	49207093	A	T	0.110	-1.328	0.258	2.57E-07	0.071
UC	IMPDH2	3	49064299	rs9813813	49207093	A	T	0.110	-1.445	0.307	2.53E-06	0.050
IBD	UBE2L3	22	21941029	rs2070512	21949411	C	A	0.194	0.163	0.021	1.06E-14	0.169
UC	UBE2L3	22	21941029	rs2070512	21949411	C	A	0.194	0.116	0.027	1.76E-05	0.698
CD	BCL2L15	1	1.14E+08	rs1217393	1.14E+08	A	G	0.271	-0.705	0.170	3.39E-05	0.184
CD	STMN3	20	62271061	rs909333	62270637	G	T	0.207	0.547	0.102	8.31E-08	0.200
IBD	STMN3	20	62271061	rs909333	62270637	G	T	0.207	0.555	0.087	2.01E-10	0.055
UC	STMN3	20	62271061	rs909333	62270637	G	T	0.207	0.498	0.098	4.04E-07	0.062
GORD	PBX2	6	32154964	rs693906	31835164	C	G	0.113	0.154	0.036	1.65E-05	0.058
VHD	AGER	6	32150423	rs693906	31835164	C	G	0.113	0.212	0.052	4.82E-05	0.092

B) Nominally significant effect on VHD of Bonferroni-significant gastrointestinal loci.

Disease	Gene	chr	Probe_bp	topSNP	topSNP_bp	A1	A2	Freq	b_SMR	se_SMR	p_SMR	p_HEIDI
VHD	CFDP1	16	75397489	rs7201989	75471499	G	A	0.246	-0.056	0.018	0.001	0.119
VHD	STAT3	17	40502964	rs1053004	40466092	G	A	0.380	0.093	0.047	0.048	0.948
VHD	IMPDH2	3	49064299	rs34037363	48821036	T	C	0.114	-0.209	0.105	0.047	0.089
VHD	UBE2L3	22	21941029	rs2070512	21949411	C	A	0.194	-0.029	0.009	0.002	0.623
VHD	STMN3	20	62271061	rs909333	62270637	G	T	0.207	-0.076	0.036	0.036	0.093

C) Effect of AGER gene on VHD and gastrointestinal disorders.

Disease	Gene	chr	Probe_bp	topSNP	topSNP_bp	A1	A2	Freq	b_SMR	se_SMR	p_SMR	p_HEIDI
CD	AGER	6	32150423	rs693906	31835164	C	G	0.113	-0.312	0.120	0.009	6.16E-08
GORD	AGER	6	32150423	rs693906	31835164	C	G	0.113	-0.209	0.049	2.02E-05	0.022
IBD	AGER	6	32148745	rs204995	32154285	G	A	0.217	-0.252	0.108	0.020	1.60E-04
IBS	AGER	6	32150423	rs693906	31835164	C	G	0.113	-0.168	0.052	0.001	0.045
UC	AGER	6	32148745	rs204995	32154285	G	A	0.217	-0.870	0.185	2.59E-06	0.002
VHD	AGER	6	32150423	rs693906	31835164	C	G	0.113	0.212	0.052	4.82E-05	0.092

Supplementary table 6. Results of the summary-based Mendelian randomization integrating whole-blood expression quantitative trait loci. For each disease-gene association, we report the strongest effect observed between GTEX and eQTLGen datasets.

A) Bonferroni-significant disease-gene association with HEIDI P > 0.05.

Disease	Gene	chr	gene_bp	topSNP	topSNP_bp	A1	A2	Freq	b_SMR	se_SMR	p_SMR	p_HEIDI
CD	DNMT3A	2	25510652	rs7578575	25488819	A	T	0.329	-0.834	0.164	3.51E-07	0.383
IBD	DNMT3A	2	25510652	rs7578575	25488819	A	T	0.329	-0.745	0.130	9.22E-09	0.515
UC	DNMT3A	2	25510652	rs7578575	25488819	A	T	0.329	-0.627	0.156	5.73E-05	0.541
UC	CFDP1	16	75327596	rs9934007	75390532	C	T	0.754	0.273	0.070	9.61E-05	0.973
CD	STAT3	17	40502964	rs1053004	40466092	G	A	0.380	0.702	0.128	4.40E-08	0.056
CD	IMPDH2	3	49064299	rs9813813	49207093	A	T	0.110	-1.233	0.294	2.74E-05	0.213
IBD	IMPDH2	3	49064299	rs9813813	49207093	A	T	0.110	-1.328	0.258	2.57E-07	0.071
UC	IMPDH2	3	49064299	rs9813813	49207093	A	T	0.110	-1.445	0.307	2.53E-06	0.050
IBD	UBE2L3	22	21941029	rs2070512	21949411	C	A	0.194	0.163	0.021	1.06E-14	0.169
UC	UBE2L3	22	21941029	rs2070512	21949411	C	A	0.194	0.116	0.027	1.76E-05	0.698
CD	BCL2L15	1	1.14E+08	rs1217393	1.14E+08	A	G	0.271	-0.705	0.170	3.39E-05	0.184
CD	STMN3	20	62271061	rs909333	62270637	G	T	0.207	0.547	0.102	8.31E-08	0.200
IBD	STMN3	20	62271061	rs909333	62270637	G	T	0.207	0.555	0.087	2.01E-10	0.055
UC	STMN3	20	62271061	rs909333	62270637	G	T	0.207	0.498	0.098	4.04E-07	0.062
GORD	PBX2	6	32154964	rs693906	31835164	C	G	0.113	0.154	0.036	1.65E-05	0.058
VHD	AGER	6	32150423	rs693906	31835164	C	G	0.113	0.212	0.052	4.82E-05	0.092

B) Nominally significant effect on VHD of Bonferroni-significant gastrointestinal loci.

Disease	Gene	chr	Probe_bp	topSNP	topSNP_bp	A1	A2	Freq	b_SMR	se_SMR	p_SMR	p_HEIDI
VHD	CFDP1	16	75397489	rs7201989	75471499	G	A	0.246	-0.056	0.018	0.001	0.119
VHD	STAT3	17	40502964	rs1053004	40466092	G	A	0.380	0.093	0.047	0.048	0.948
VHD	IMPDH2	3	49064299	rs34037363	48821036	T	C	0.114	-0.209	0.105	0.047	0.089
VHD	UBE2L3	22	21941029	rs2070512	21949411	C	A	0.194	-0.029	0.009	0.002	0.623
VHD	STMN3	20	62271061	rs909333	62270637	G	T	0.207	-0.076	0.036	0.036	0.093

C) Effect of AGER gene on VHD and gastrointestinal disorders.

Disease	Gene	chr	Probe_bp	topSNP	topSNP_bp	A1	A2	Freq	b_SMR	se_SMR	p_SMR	p_HEIDI
CD	AGER	6	32150423	rs693906	31835164	C	G	0.113	-0.312	0.120	0.009	6.16E-08
GORD	AGER	6	32150423	rs693906	31835164	C	G	0.113	-0.209	0.049	2.02E-05	0.022
IBD	AGER	6	32148745	rs204995	32154285	G	A	0.217	-0.252	0.108	0.020	1.60E-04
IBS	AGER	6	32150423	rs693906	31835164	C	G	0.113	-0.168	0.052	0.001	0.045
UC	AGER	6	32148745	rs204995	32154285	G	A	0.217	-0.870	0.185	2.59E-06	0.002
VHD	AGER	6	32150423	rs693906	31835164	C	G	0.113	0.212	0.052	4.82E-05	0.092

Supplementary table 6. Results of the summary-based Mendelian randomization integrating whole-blood expression quantitative trait loci. For each disease-gene association, we report the strongest effect observed between GTEX and eQTLGen datasets.

A) Bonferroni-significant disease-gene association with HEIDI P > 0.05.

Disease	Gene	chr	gene_bp	topSNP	topSNP_bp	A1	A2	Freq	b_SMR	se_SMR	p_SMR	p_HEIDI
CD	DNMT3A	2	25510652	rs7578575	25488819	A	T	0.329	-0.834	0.164	3.51E-07	0.383
IBD	DNMT3A	2	25510652	rs7578575	25488819	A	T	0.329	-0.745	0.130	9.22E-09	0.515
UC	DNMT3A	2	25510652	rs7578575	25488819	A	T	0.329	-0.627	0.156	5.73E-05	0.541
UC	CFDP1	16	75327596	rs9934007	75390532	C	T	0.754	0.273	0.070	9.61E-05	0.973
CD	STAT3	17	40502964	rs1053004	40466092	G	A	0.380	0.702	0.128	4.40E-08	0.056
CD	IMPDH2	3	49064299	rs9813813	49207093	A	T	0.110	-1.233	0.294	2.74E-05	0.213
IBD	IMPDH2	3	49064299	rs9813813	49207093	A	T	0.110	-1.328	0.258	2.57E-07	0.071
UC	IMPDH2	3	49064299	rs9813813	49207093	A	T	0.110	-1.445	0.307	2.53E-06	0.050
IBD	UBE2L3	22	21941029	rs2070512	21949411	C	A	0.194	0.163	0.021	1.06E-14	0.169
UC	UBE2L3	22	21941029	rs2070512	21949411	C	A	0.194	0.116	0.027	1.76E-05	0.698
CD	BCL2L15	1	1.14E+08	rs1217393	1.14E+08	A	G	0.271	-0.705	0.170	3.39E-05	0.184
CD	STMN3	20	62271061	rs909333	62270637	G	T	0.207	0.547	0.102	8.31E-08	0.200
IBD	STMN3	20	62271061	rs909333	62270637	G	T	0.207	0.555	0.087	2.01E-10	0.055
UC	STMN3	20	62271061	rs909333	62270637	G	T	0.207	0.498	0.098	4.04E-07	0.062
GORD	PBX2	6	32154964	rs693906	31835164	C	G	0.113	0.154	0.036	1.65E-05	0.058
VHD	AGER	6	32150423	rs693906	31835164	C	G	0.113	0.212	0.052	4.82E-05	0.092

B) Nominally significant effect on VHD of Bonferroni-significant gastrointestinal loci.

Disease	Gene	chr	Probe_bp	topSNP	topSNP_bp	A1	A2	Freq	b_SMR	se_SMR	p_SMR	p_HEIDI
VHD	CFDP1	16	75397489	rs7201989	75471499	G	A	0.246	-0.056	0.018	0.001	0.119
VHD	STAT3	17	40502964	rs1053004	40466092	G	A	0.380	0.093	0.047	0.048	0.948
VHD	IMPDH2	3	49064299	rs34037363	48821036	T	C	0.114	-0.209	0.105	0.047	0.089
VHD	UBE2L3	22	21941029	rs2070512	21949411	C	A	0.194	-0.029	0.009	0.002	0.623
VHD	STMN3	20	62271061	rs909333	62270637	G	T	0.207	-0.076	0.036	0.036	0.093

C) Effect of AGER gene on VHD and gastrointestinal disorders.

Disease	Gene	chr	Probe_bp	topSNP	topSNP_bp	A1	A2	Freq	b_SMR	se_SMR	p_SMR	p_HEIDI
CD	AGER	6	32150423	rs693906	31835164	C	G	0.113	-0.312	0.120	0.009	6.16E-08
GORD	AGER	6	32150423	rs693906	31835164	C	G	0.113	-0.209	0.049	2.02E-05	0.022
IBD	AGER	6	32148745	rs204995	32154285	G	A	0.217	-0.252	0.108	0.020	1.60E-04
IBS	AGER	6	32150423	rs693906	31835164	C	G	0.113	-0.168	0.052	0.001	0.045
UC	AGER	6	32148745	rs204995	32154285	G	A	0.217	-0.870	0.185	2.59E-06	0.002
VHD	AGER	6	32150423	rs693906	31835164	C	G	0.113	0.212	0.052	4.82E-05	0.092

Supplementary table 6. Results of the summary-based Mendelian randomization integrating whole-blood expression quantitative trait loci. For each disease-gene association, we report the strongest effect observed between GTEX and eQTLGen datasets.

A) Bonferroni-significant disease-gene association with HEIDI P > 0.05.

Disease	Gene	chr	gene_bp	topSNP	topSNP_bp	A1	A2	Freq	b_SMR	se_SMR	p_SMR	p_HEIDI
CD	DNMT3A	2	25510652	rs7578575	25488819	A	T	0.329	-0.834	0.164	3.51E-07	0.383
IBD	DNMT3A	2	25510652	rs7578575	25488819	A	T	0.329	-0.745	0.130	9.22E-09	0.515
UC	DNMT3A	2	25510652	rs7578575	25488819	A	T	0.329	-0.627	0.156	5.73E-05	0.541
UC	CFDP1	16	75327596	rs9934007	75390532	C	T	0.754	0.273	0.070	9.61E-05	0.973
CD	STAT3	17	40502964	rs1053004	40466092	G	A	0.380	0.702	0.128	4.40E-08	0.056
CD	IMPDH2	3	49064299	rs9813813	49207093	A	T	0.110	-1.233	0.294	2.74E-05	0.213
IBD	IMPDH2	3	49064299	rs9813813	49207093	A	T	0.110	-1.328	0.258	2.57E-07	0.071
UC	IMPDH2	3	49064299	rs9813813	49207093	A	T	0.110	-1.445	0.307	2.53E-06	0.050
IBD	UBE2L3	22	21941029	rs2070512	21949411	C	A	0.194	0.163	0.021	1.06E-14	0.169
UC	UBE2L3	22	21941029	rs2070512	21949411	C	A	0.194	0.116	0.027	1.76E-05	0.698
CD	BCL2L15	1	1.14E+08	rs1217393	1.14E+08	A	G	0.271	-0.705	0.170	3.39E-05	0.184
CD	STMN3	20	62271061	rs909333	62270637	G	T	0.207	0.547	0.102	8.31E-08	0.200
IBD	STMN3	20	62271061	rs909333	62270637	G	T	0.207	0.555	0.087	2.01E-10	0.055
UC	STMN3	20	62271061	rs909333	62270637	G	T	0.207	0.498	0.098	4.04E-07	0.062
GORD	PBX2	6	32154964	rs693906	31835164	C	G	0.113	0.154	0.036	1.65E-05	0.058
VHD	AGER	6	32150423	rs693906	31835164	C	G	0.113	0.212	0.052	4.82E-05	0.092

B) Nominally significant effect on VHD of Bonferroni-significant gastrointestinal loci.

Disease	Gene	chr	Probe_bp	topSNP	topSNP_bp	A1	A2	Freq	b_SMR	se_SMR	p_SMR	p_HEIDI
VHD	CFDP1	16	75397489	rs7201989	75471499	G	A	0.246	-0.056	0.018	0.001	0.119
VHD	STAT3	17	40502964	rs1053004	40466092	G	A	0.380	0.093	0.047	0.048	0.948
VHD	IMPDH2	3	49064299	rs34037363	48821036	T	C	0.114	-0.209	0.105	0.047	0.089
VHD	UBE2L3	22	21941029	rs2070512	21949411	C	A	0.194	-0.029	0.009	0.002	0.623
VHD	STMN3	20	62271061	rs909333	62270637	G	T	0.207	-0.076	0.036	0.036	0.093

C) Effect of AGER gene on VHD and gastrointestinal disorders.

Disease	Gene	chr	Probe_bp	topSNP	topSNP_bp	A1	A2	Freq	b_SMR	se_SMR	p_SMR	p_HEIDI
CD	AGER	6	32150423	rs693906	31835164	C	G	0.113	-0.312	0.120	0.009	6.16E-08
GORD	AGER	6	32150423	rs693906	31835164	C	G	0.113	-0.209	0.049	2.02E-05	0.022
IBD	AGER	6	32148745	rs204995	32154285	G	A	0.217	-0.252	0.108	0.020	1.60E-04
IBS	AGER	6	32150423	rs693906	31835164	C	G	0.113	-0.168	0.052	0.001	0.045
UC	AGER	6	32148745	rs204995	32154285	G	A	0.217	-0.870	0.185	2.59E-06	0.002
VHD	AGER	6	32150423	rs693906	31835164	C	G	0.113	0.212	0.052	4.82E-05	0.092

Supplementary table 6. Results of the summary-based Mendelian randomization integrating whole-blood expression quantitative trait loci. For each disease-gene association, we report the strongest effect observed between GTEX and eQTLGen datasets.

A) Bonferroni-significant disease-gene association with HEIDI P > 0.05.

Disease	Gene	chr	gene_bp	topSNP	topSNP_bp	A1	A2	Freq	b_SMR	se_SMR	p_SMR	p_HEIDI
CD	DNMT3A	2	25510652	rs7578575	25488819	A	T	0.329	-0.834	0.164	3.51E-07	0.383
IBD	DNMT3A	2	25510652	rs7578575	25488819	A	T	0.329	-0.745	0.130	9.22E-09	0.515
UC	DNMT3A	2	25510652	rs7578575	25488819	A	T	0.329	-0.627	0.156	5.73E-05	0.541
UC	CFDP1	16	75327596	rs9934007	75390532	C	T	0.754	0.273	0.070	9.61E-05	0.973
CD	STAT3	17	40502964	rs1053004	40466092	G	A	0.380	0.702	0.128	4.40E-08	0.056
CD	IMPDH2	3	49064299	rs9813813	49207093	A	T	0.110	-1.233	0.294	2.74E-05	0.213
IBD	IMPDH2	3	49064299	rs9813813	49207093	A	T	0.110	-1.328	0.258	2.57E-07	0.071
UC	IMPDH2	3	49064299	rs9813813	49207093	A	T	0.110	-1.445	0.307	2.53E-06	0.050
IBD	UBE2L3	22	21941029	rs2070512	21949411	C	A	0.194	0.163	0.021	1.06E-14	0.169
UC	UBE2L3	22	21941029	rs2070512	21949411	C	A	0.194	0.116	0.027	1.76E-05	0.698
CD	BCL2L15	1	1.14E+08	rs1217393	1.14E+08	A	G	0.271	-0.705	0.170	3.39E-05	0.184
CD	STMN3	20	62271061	rs909333	62270637	G	T	0.207	0.547	0.102	8.31E-08	0.200
IBD	STMN3	20	62271061	rs909333	62270637	G	T	0.207	0.555	0.087	2.01E-10	0.055
UC	STMN3	20	62271061	rs909333	62270637	G	T	0.207	0.498	0.098	4.04E-07	0.062
GORD	PBX2	6	32154964	rs693906	31835164	C	G	0.113	0.154	0.036	1.65E-05	0.058
VHD	AGER	6	32150423	rs693906	31835164	C	G	0.113	0.212	0.052	4.82E-05	0.092

B) Nominally significant effect on VHD of Bonferroni-significant gastrointestinal loci.

Disease	Gene	chr	Probe_bp	topSNP	topSNP_bp	A1	A2	Freq	b_SMR	se_SMR	p_SMR	p_HEIDI
VHD	CFDP1	16	75397489	rs7201989	75471499	G	A	0.246	-0.056	0.018	0.001	0.119
VHD	STAT3	17	40502964	rs1053004	40466092	G	A	0.380	0.093	0.047	0.048	0.948
VHD	IMPDH2	3	49064299	rs34037363	48821036	T	C	0.114	-0.209	0.105	0.047	0.089
VHD	UBE2L3	22	21941029	rs2070512	21949411	C	A	0.194	-0.029	0.009	0.002	0.623
VHD	STMN3	20	62271061	rs909333	62270637	G	T	0.207	-0.076	0.036	0.036	0.093

C) Effect of AGER gene on VHD and gastrointestinal disorders.

Disease	Gene	chr	Probe_bp	topSNP	topSNP_bp	A1	A2	Freq	b_SMR	se_SMR	p_SMR	p_HEIDI
CD	AGER	6	32150423	rs693906	31835164	C	G	0.113	-0.312	0.120	0.009	6.16E-08
GORD	AGER	6	32150423	rs693906	31835164	C	G	0.113	-0.209	0.049	2.02E-05	0.022
IBD	AGER	6	32148745	rs204995	32154285	G	A	0.217	-0.252	0.108	0.020	1.60E-04
IBS	AGER	6	32150423	rs693906	31835164	C	G	0.113	-0.168	0.052	0.001	0.045
UC	AGER	6	32148745	rs204995	32154285	G	A	0.217	-0.870	0.185	2.59E-06	0.002
VHD	AGER	6	32150423	rs693906	31835164	C	G	0.113	0.212	0.052	4.82E-05	0.092

Supplementary table 6. Results of the summary-based Mendelian randomization integrating whole-blood expression quantitative trait loci. For each disease-gene association, we report the strongest effect observed between GTEX and eQTLGen datasets.

A) Bonferroni-significant disease-gene association with HEIDI P > 0.05.

Disease	Gene	chr	gene_bp	topSNP	topSNP_bp	A1	A2	Freq	b_SMR	se_SMR	p_SMR	p_HEIDI
CD	DNMT3A	2	25510652	rs7578575	25488819	A	T	0.329	-0.834	0.164	3.51E-07	0.383
IBD	DNMT3A	2	25510652	rs7578575	25488819	A	T	0.329	-0.745	0.130	9.22E-09	0.515
UC	DNMT3A	2	25510652	rs7578575	25488819	A	T	0.329	-0.627	0.156	5.73E-05	0.541
UC	CFDP1	16	75327596	rs9934007	75390532	C	T	0.754	0.273	0.070	9.61E-05	0.973
CD	STAT3	17	40502964	rs1053004	40466092	G	A	0.380	0.702	0.128	4.40E-08	0.056
CD	IMPDH2	3	49064299	rs9813813	49207093	A	T	0.110	-1.233	0.294	2.74E-05	0.213
IBD	IMPDH2	3	49064299	rs9813813	49207093	A	T	0.110	-1.328	0.258	2.57E-07	0.071
UC	IMPDH2	3	49064299	rs9813813	49207093	A	T	0.110	-1.445	0.307	2.53E-06	0.050
IBD	UBE2L3	22	21941029	rs2070512	21949411	C	A	0.194	0.163	0.021	1.06E-14	0.169
UC	UBE2L3	22	21941029	rs2070512	21949411	C	A	0.194	0.116	0.027	1.76E-05	0.698
CD	BCL2L15	1	1.14E+08	rs1217393	1.14E+08	A	G	0.271	-0.705	0.170	3.39E-05	0.184
CD	STMN3	20	62271061	rs909333	62270637	G	T	0.207	0.547	0.102	8.31E-08	0.200
IBD	STMN3	20	62271061	rs909333	62270637	G	T	0.207	0.555	0.087	2.01E-10	0.055
UC	STMN3	20	62271061	rs909333	62270637	G	T	0.207	0.498	0.098	4.04E-07	0.062
GORD	PBX2	6	32154964	rs693906	31835164	C	G	0.113	0.154	0.036	1.65E-05	0.058
VHD	AGER	6	32150423	rs693906	31835164	C	G	0.113	0.212	0.052	4.82E-05	0.092

B) Nominally significant effect on VHD of Bonferroni-significant gastrointestinal loci.

Disease	Gene	chr	Probe_bp	topSNP	topSNP_bp	A1	A2	Freq	b_SMR	se_SMR	p_SMR	p_HEIDI
VHD	CFDP1	16	75397489	rs7201989	75471499	G	A	0.246	-0.056	0.018	0.001	0.119
VHD	STAT3	17	40502964	rs1053004	40466092	G	A	0.380	0.093	0.047	0.048	0.948
VHD	IMPDH2	3	49064299	rs34037363	48821036	T	C	0.114	-0.209	0.105	0.047	0.089
VHD	UBE2L3	22	21941029	rs2070512	21949411	C	A	0.194	-0.029	0.009	0.002	0.623
VHD	STMN3	20	62271061	rs909333	62270637	G	T	0.207	-0.076	0.036	0.036	0.093

C) Effect of AGER gene on VHD and gastrointestinal disorders.

Disease	Gene	chr	Probe_bp	topSNP	topSNP_bp	A1	A2	Freq	b_SMR	se_SMR	p_SMR	p_HEIDI
CD	AGER	6	32150423	rs693906	31835164	C	G	0.113	-0.312	0.120	0.009	6.16E-08
GORD	AGER	6	32150423	rs693906	31835164	C	G	0.113	-0.209	0.049	2.02E-05	0.022
IBD	AGER	6	32148745	rs204995	32154285	G	A	0.217	-0.252	0.108	0.020	1.60E-04
IBS	AGER	6	32150423	rs693906	31835164	C	G	0.113	-0.168	0.052	0.001	0.045
UC	AGER	6	32148745	rs204995	32154285	G	A	0.217	-0.870	0.185	2.59E-06	0.002
VHD	AGER	6	32150423	rs693906	31835164	C	G	0.113	0.212	0.052	4.82E-05	0.092

Supplementary table 6. Results of the summary-based Mendelian randomization integrating whole-blood expression quantitative trait loci. For each disease-gene association, we report the strongest effect observed between GTEX and eQTLGen datasets.

A) Bonferroni-significant disease-gene association with HEIDI P > 0.05.

Disease	Gene	chr	gene_bp	topSNP	topSNP_bp	A1	A2	Freq	b_SMR	se_SMR	p_SMR	p_HEIDI
CD	DNMT3A	2	25510652	rs7578575	25488819	A	T	0.329	-0.834	0.164	3.51E-07	0.383
IBD	DNMT3A	2	25510652	rs7578575	25488819	A	T	0.329	-0.745	0.130	9.22E-09	0.515
UC	DNMT3A	2	25510652	rs7578575	25488819	A	T	0.329	-0.627	0.156	5.73E-05	0.541
UC	CFDP1	16	75327596	rs9934007	75390532	C	T	0.754	0.273	0.070	9.61E-05	0.973
CD	STAT3	17	40502964	rs1053004	40466092	G	A	0.380	0.702	0.128	4.40E-08	0.056
CD	IMPDH2	3	49064299	rs9813813	49207093	A	T	0.110	-1.233	0.294	2.74E-05	0.213
IBD	IMPDH2	3	49064299	rs9813813	49207093	A	T	0.110	-1.328	0.258	2.57E-07	0.071
UC	IMPDH2	3	49064299	rs9813813	49207093	A	T	0.110	-1.445	0.307	2.53E-06	0.050
IBD	UBE2L3	22	21941029	rs2070512	21949411	C	A	0.194	0.163	0.021	1.06E-14	0.169
UC	UBE2L3	22	21941029	rs2070512	21949411	C	A	0.194	0.116	0.027	1.76E-05	0.698
CD	BCL2L15	1	1.14E+08	rs1217393	1.14E+08	A	G	0.271	-0.705	0.170	3.39E-05	0.184
CD	STMN3	20	62271061	rs909333	62270637	G	T	0.207	0.547	0.102	8.31E-08	0.200
IBD	STMN3	20	62271061	rs909333	62270637	G	T	0.207	0.555	0.087	2.01E-10	0.055
UC	STMN3	20	62271061	rs909333	62270637	G	T	0.207	0.498	0.098	4.04E-07	0.062
GORD	PBX2	6	32154964	rs693906	31835164	C	G	0.113	0.154	0.036	1.65E-05	0.058
VHD	AGER	6	32150423	rs693906	31835164	C	G	0.113	0.212	0.052	4.82E-05	0.092

B) Nominally significant effect on VHD of Bonferroni-significant gastrointestinal loci.

Disease	Gene	chr	Probe_bp	topSNP	topSNP_bp	A1	A2	Freq	b_SMR	se_SMR	p_SMR	p_HEIDI
VHD	CFDP1	16	75397489	rs7201989	75471499	G	A	0.246	-0.056	0.018	0.001	0.119
VHD	STAT3	17	40502964	rs1053004	40466092	G	A	0.380	0.093	0.047	0.048	0.948
VHD	IMPDH2	3	49064299	rs34037363	48821036	T	C	0.114	-0.209	0.105	0.047	0.089
VHD	UBE2L3	22	21941029	rs2070512	21949411	C	A	0.194	-0.029	0.009	0.002	0.623
VHD	STMN3	20	62271061	rs909333	62270637	G	T	0.207	-0.076	0.036	0.036	0.093

C) Effect of AGER gene on VHD and gastrointestinal disorders.

Disease	Gene	chr	Probe_bp	topSNP	topSNP_bp	A1	A2	Freq	b_SMR	se_SMR	p_SMR	p_HEIDI
CD	AGER	6	32150423	rs693906	31835164	C	G	0.113	-0.312	0.120	0.009	6.16E-08
GORD	AGER	6	32150423	rs693906	31835164	C	G	0.113	-0.209	0.049	2.02E-05	0.022
IBD	AGER	6	32148745	rs204995	32154285	G	A	0.217	-0.252	0.108	0.020	1.60E-04
IBS	AGER	6	32150423	rs693906	31835164	C	G	0.113	-0.168	0.052	0.001	0.045
UC	AGER	6	32148745	rs204995	32154285	G	A	0.217	-0.870	0.185	2.59E-06	0.002
VHD	AGER	6	32150423	rs693906	31835164	C	G	0.113	0.212	0.052	4.82E-05	0.092

Supplementary table 6. Results of the summary-based Mendelian randomization integrating whole-blood expression quantitative trait loci. For each disease-gene association, we report the strongest effect observed between GTEX and eQTLGen datasets.

A) Bonferroni-significant disease-gene association with HEIDI P > 0.05.

Disease	Gene	chr	gene_bp	topSNP	topSNP_bp	A1	A2	Freq	b_SMR	se_SMR	p_SMR	p_HEIDI
CD	DNMT3A	2	25510652	rs7578575	25488819	A	T	0.329	-0.834	0.164	3.51E-07	0.383
IBD	DNMT3A	2	25510652	rs7578575	25488819	A	T	0.329	-0.745	0.130	9.22E-09	0.515
UC	DNMT3A	2	25510652	rs7578575	25488819	A	T	0.329	-0.627	0.156	5.73E-05	0.541
UC	CFDP1	16	75327596	rs9934007	75390532	C	T	0.754	0.273	0.070	9.61E-05	0.973
CD	STAT3	17	40502964	rs1053004	40466092	G	A	0.380	0.702	0.128	4.40E-08	0.056
CD	IMPDH2	3	49064299	rs9813813	49207093	A	T	0.110	-1.233	0.294	2.74E-05	0.213
IBD	IMPDH2	3	49064299	rs9813813	49207093	A	T	0.110	-1.328	0.258	2.57E-07	0.071
UC	IMPDH2	3	49064299	rs9813813	49207093	A	T	0.110	-1.445	0.307	2.53E-06	0.050
IBD	UBE2L3	22	21941029	rs2070512	21949411	C	A	0.194	0.163	0.021	1.06E-14	0.169
UC	UBE2L3	22	21941029	rs2070512	21949411	C	A	0.194	0.116	0.027	1.76E-05	0.698
CD	BCL2L15	1	1.14E+08	rs1217393	1.14E+08	A	G	0.271	-0.705	0.170	3.39E-05	0.184
CD	STMN3	20	62271061	rs909333	62270637	G	T	0.207	0.547	0.102	8.31E-08	0.200
IBD	STMN3	20	62271061	rs909333	62270637	G	T	0.207	0.555	0.087	2.01E-10	0.055
UC	STMN3	20	62271061	rs909333	62270637	G	T	0.207	0.498	0.098	4.04E-07	0.062
GORD	PBX2	6	32154964	rs693906	31835164	C	G	0.113	0.154	0.036	1.65E-05	0.058
VHD	AGER	6	32150423	rs693906	31835164	C	G	0.113	0.212	0.052	4.82E-05	0.092

B) Nominally significant effect on VHD of Bonferroni-significant gastrointestinal loci.

Disease	Gene	chr	Probe_bp	topSNP	topSNP_bp	A1	A2	Freq	b_SMR	se_SMR	p_SMR	p_HEIDI
VHD	CFDP1	16	75397489	rs7201989	75471499	G	A	0.246	-0.056	0.018	0.001	0.119
VHD	STAT3	17	40502964	rs1053004	40466092	G	A	0.380	0.093	0.047	0.048	0.948
VHD	IMPDH2	3	49064299	rs34037363	48821036	T	C	0.114	-0.209	0.105	0.047	0.089
VHD	UBE2L3	22	21941029	rs2070512	21949411	C	A	0.194	-0.029	0.009	0.002	0.623
VHD	STMN3	20	62271061	rs909333	62270637	G	T	0.207	-0.076	0.036	0.036	0.093

C) Effect of AGER gene on VHD and gastrointestinal disorders.

Disease	Gene	chr	Probe_bp	topSNP	topSNP_bp	A1	A2	Freq	b_SMR	se_SMR	p_SMR	p_HEIDI
CD	AGER	6	32150423	rs693906	31835164	C	G	0.113	-0.312	0.120	0.009	6.16E-08
GORD	AGER	6	32150423	rs693906	31835164	C	G	0.113	-0.209	0.049	2.02E-05	0.022
IBD	AGER	6	32148745	rs204995	32154285	G	A	0.217	-0.252	0.108	0.020	1.60E-04
IBS	AGER	6	32150423	rs693906	31835164	C	G	0.113	-0.168	0.052	0.001	0.045
UC	AGER	6	32148745	rs204995	32154285	G	A	0.217	-0.870	0.185	2.59E-06	0.002
VHD	AGER	6	32150423	rs693906	31835164	C	G	0.113	0.212	0.052	4.82E-05	0.092

Supplementary table 6. Results of the summary-based Mendelian randomization integrating whole-blood expression quantitative trait loci. For each disease-gene association, we report the strongest effect observed between GTEX and eQTLGen datasets.

A) Bonferroni-significant disease-gene association with HEIDI P > 0.05.

Disease	Gene	chr	gene_bp	topSNP	topSNP_bp	A1	A2	Freq	b_SMR	se_SMR	p_SMR	p_HEIDI
CD	DNMT3A	2	25510652	rs7578575	25488819	A	T	0.329	-0.834	0.164	3.51E-07	0.383
IBD	DNMT3A	2	25510652	rs7578575	25488819	A	T	0.329	-0.745	0.130	9.22E-09	0.515
UC	DNMT3A	2	25510652	rs7578575	25488819	A	T	0.329	-0.627	0.156	5.73E-05	0.541
UC	CFDP1	16	75327596	rs9934007	75390532	C	T	0.754	0.273	0.070	9.61E-05	0.973
CD	STAT3	17	40502964	rs1053004	40466092	G	A	0.380	0.702	0.128	4.40E-08	0.056
CD	IMPDH2	3	49064299	rs9813813	49207093	A	T	0.110	-1.233	0.294	2.74E-05	0.213
IBD	IMPDH2	3	49064299	rs9813813	49207093	A	T	0.110	-1.328	0.258	2.57E-07	0.071
UC	IMPDH2	3	49064299	rs9813813	49207093	A	T	0.110	-1.445	0.307	2.53E-06	0.050
IBD	UBE2L3	22	21941029	rs2070512	21949411	C	A	0.194	0.163	0.021	1.06E-14	0.169
UC	UBE2L3	22	21941029	rs2070512	21949411	C	A	0.194	0.116	0.027	1.76E-05	0.698
CD	BCL2L15	1	1.14E+08	rs1217393	1.14E+08	A	G	0.271	-0.705	0.170	3.39E-05	0.184
CD	STMN3	20	62271061	rs909333	62270637	G	T	0.207	0.547	0.102	8.31E-08	0.200
IBD	STMN3	20	62271061	rs909333	62270637	G	T	0.207	0.555	0.087	2.01E-10	0.055
UC	STMN3	20	62271061	rs909333	62270637	G	T	0.207	0.498	0.098	4.04E-07	0.062
GORD	PBX2	6	32154964	rs693906	31835164	C	G	0.113	0.154	0.036	1.65E-05	0.058
VHD	AGER	6	32150423	rs693906	31835164	C	G	0.113	0.212	0.052	4.82E-05	0.092

B) Nominally significant effect on VHD of Bonferroni-significant gastrointestinal loci.

Disease	Gene	chr	Probe_bp	topSNP	topSNP_bp	A1	A2	Freq	b_SMR	se_SMR	p_SMR	p_HEIDI
VHD	CFDP1	16	75397489	rs7201989	75471499	G	A	0.246	-0.056	0.018	0.001	0.119
VHD	STAT3	17	40502964	rs1053004	40466092	G	A	0.380	0.093	0.047	0.048	0.948
VHD	IMPDH2	3	49064299	rs34037363	48821036	T	C	0.114	-0.209	0.105	0.047	0.089
VHD	UBE2L3	22	21941029	rs2070512	21949411	C	A	0.194	-0.029	0.009	0.002	0.623
VHD	STMN3	20	62271061	rs909333	62270637	G	T	0.207	-0.076	0.036	0.036	0.093

C) Effect of AGER gene on VHD and gastrointestinal disorders.

Disease	Gene	chr	Probe_bp	topSNP	topSNP_bp	A1	A2	Freq	b_SMR	se_SMR	p_SMR	p_HEIDI
CD	AGER	6	32150423	rs693906	31835164	C	G	0.113	-0.312	0.120	0.009	6.16E-08
GORD	AGER	6	32150423	rs693906	31835164	C	G	0.113	-0.209	0.049	2.02E-05	0.022
IBD	AGER	6	32148745	rs204995	32154285	G	A	0.217	-0.252	0.108	0.020	1.60E-04
IBS	AGER	6	32150423	rs693906	31835164	C	G	0.113	-0.168	0.052	0.001	0.045
UC	AGER	6	32148745	rs204995	32154285	G	A	0.217	-0.870	0.185	2.59E-06	0.002
VHD	AGER	6	32150423	rs693906	31835164	C	G	0.113	0.212	0.052	4.82E-05	0.092

Supplementary table 6. Results of the summary-based Mendelian randomization integrating whole-blood expression quantitative trait loci. For each disease-gene association, we report the strongest effect observed between GTEX and eQTLGen datasets.

A) Bonferroni-significant disease-gene association with HEIDI P > 0.05.

Disease	Gene	chr	gene_bp	topSNP	topSNP_bp	A1	A2	Freq	b_SMR	se_SMR	p_SMR	p_HEIDI
CD	DNMT3A	2	25510652	rs7578575	25488819	A	T	0.329	-0.834	0.164	3.51E-07	0.383
IBD	DNMT3A	2	25510652	rs7578575	25488819	A	T	0.329	-0.745	0.130	9.22E-09	0.515
UC	DNMT3A	2	25510652	rs7578575	25488819	A	T	0.329	-0.627	0.156	5.73E-05	0.541
UC	CFDP1	16	75327596	rs9934007	75390532	C	T	0.754	0.273	0.070	9.61E-05	0.973
CD	STAT3	17	40502964	rs1053004	40466092	G	A	0.380	0.702	0.128	4.40E-08	0.056
CD	IMPDH2	3	49064299	rs9813813	49207093	A	T	0.110	-1.233	0.294	2.74E-05	0.213
IBD	IMPDH2	3	49064299	rs9813813	49207093	A	T	0.110	-1.328	0.258	2.57E-07	0.071
UC	IMPDH2	3	49064299	rs9813813	49207093	A	T	0.110	-1.445	0.307	2.53E-06	0.050
IBD	UBE2L3	22	21941029	rs2070512	21949411	C	A	0.194	0.163	0.021	1.06E-14	0.169
UC	UBE2L3	22	21941029	rs2070512	21949411	C	A	0.194	0.116	0.027	1.76E-05	0.698
CD	BCL2L15	1	1.14E+08	rs1217393	1.14E+08	A	G	0.271	-0.705	0.170	3.39E-05	0.184
CD	STMN3	20	62271061	rs909333	62270637	G	T	0.207	0.547	0.102	8.31E-08	0.200
IBD	STMN3	20	62271061	rs909333	62270637	G	T	0.207	0.555	0.087	2.01E-10	0.055
UC	STMN3	20	62271061	rs909333	62270637	G	T	0.207	0.498	0.098	4.04E-07	0.062
GORD	PBX2	6	32154964	rs693906	31835164	C	G	0.113	0.154	0.036	1.65E-05	0.058
VHD	AGER	6	32150423	rs693906	31835164	C	G	0.113	0.212	0.052	4.82E-05	0.092

B) Nominally significant effect on VHD of Bonferroni-significant gastrointestinal loci.

Disease	Gene	chr	Probe_bp	topSNP	topSNP_bp	A1	A2	Freq	b_SMR	se_SMR	p_SMR	p_HEIDI
VHD	CFDP1	16	75397489	rs7201989	75471499	G	A	0.246	-0.056	0.018	0.001	0.119
VHD	STAT3	17	40502964	rs1053004	40466092	G	A	0.380	0.093	0.047	0.048	0.948
VHD	IMPDH2	3	49064299	rs34037363	48821036	T	C	0.114	-0.209	0.105	0.047	0.089
VHD	UBE2L3	22	21941029	rs2070512	21949411	C	A	0.194	-0.029	0.009	0.002	0.623
VHD	STMN3	20	62271061	rs909333	62270637	G	T	0.207	-0.076	0.036	0.036	0.093

C) Effect of AGER gene on VHD and gastrointestinal disorders.

Disease	Gene	chr	Probe_bp	topSNP	topSNP_bp	A1	A2	Freq	b_SMR	se_SMR	p_SMR	p_HEIDI
CD	AGER	6	32150423	rs693906	31835164	C	G	0.113	-0.312	0.120	0.009	6.16E-08
GORD	AGER	6	32150423	rs693906	31835164	C	G	0.113	-0.209	0.049	2.02E-05	0.022
IBD	AGER	6	32148745	rs204995	32154285	G	A	0.217	-0.252	0.108	0.020	1.60E-04
IBS	AGER	6	32150423	rs693906	31835164	C	G	0.113	-0.168	0.052	0.001	0.045
UC	AGER	6	32148745	rs204995	32154285	G	A	0.217	-0.870	0.185	2.59E-06	0.002
VHD	AGER	6	32150423	rs693906	31835164	C	G	0.113	0.212	0.052	4.82E-05	0.092

Supplementary table 6. Results of the summary-based Mendelian randomization integrating whole-blood expression quantitative trait loci. For each disease-gene association, we report the strongest effect observed between GTEX and eQTLGen datasets.

A) Bonferroni-significant disease-gene association with HEIDI P > 0.05.

Disease	Gene	chr	gene_bp	topSNP	topSNP_bp	A1	A2	Freq	b_SMR	se_SMR	p_SMR	p_HEIDI
CD	DNMT3A	2	25510652	rs7578575	25488819	A	T	0.329	-0.834	0.164	3.51E-07	0.383
IBD	DNMT3A	2	25510652	rs7578575	25488819	A	T	0.329	-0.745	0.130	9.22E-09	0.515
UC	DNMT3A	2	25510652	rs7578575	25488819	A	T	0.329	-0.627	0.156	5.73E-05	0.541
UC	CFDP1	16	75327596	rs9934007	75390532	C	T	0.754	0.273	0.070	9.61E-05	0.973
CD	STAT3	17	40502964	rs1053004	40466092	G	A	0.380	0.702	0.128	4.40E-08	0.056
CD	IMPDH2	3	49064299	rs9813813	49207093	A	T	0.110	-1.233	0.294	2.74E-05	0.213
IBD	IMPDH2	3	49064299	rs9813813	49207093	A	T	0.110	-1.328	0.258	2.57E-07	0.071
UC	IMPDH2	3	49064299	rs9813813	49207093	A	T	0.110	-1.445	0.307	2.53E-06	0.050
IBD	UBE2L3	22	21941029	rs2070512	21949411	C	A	0.194	0.163	0.021	1.06E-14	0.169
UC	UBE2L3	22	21941029	rs2070512	21949411	C	A	0.194	0.116	0.027	1.76E-05	0.698
CD	BCL2L15	1	1.14E+08	rs1217393	1.14E+08	A	G	0.271	-0.705	0.170	3.39E-05	0.184
CD	STMN3	20	62271061	rs909333	62270637	G	T	0.207	0.547	0.102	8.31E-08	0.200
IBD	STMN3	20	62271061	rs909333	62270637	G	T	0.207	0.555	0.087	2.01E-10	0.055
UC	STMN3	20	62271061	rs909333	62270637	G	T	0.207	0.498	0.098	4.04E-07	0.062
GORD	PBX2	6	32154964	rs693906	31835164	C	G	0.113	0.154	0.036	1.65E-05	0.058
VHD	AGER	6	32150423	rs693906	31835164	C	G	0.113	0.212	0.052	4.82E-05	0.092

B) Nominally significant effect on VHD of Bonferroni-significant gastrointestinal loci.

Disease	Gene	chr	Probe_bp	topSNP	topSNP_bp	A1	A2	Freq	b_SMR	se_SMR	p_SMR	p_HEIDI
VHD	CFDP1	16	75397489	rs7201989	75471499	G	A	0.246	-0.056	0.018	0.001	0.119
VHD	STAT3	17	40502964	rs1053004	40466092	G	A	0.380	0.093	0.047	0.048	0.948
VHD	IMPDH2	3	49064299	rs34037363	48821036	T	C	0.114	-0.209	0.105	0.047	0.089
VHD	UBE2L3	22	21941029	rs2070512	21949411	C	A	0.194	-0.029	0.009	0.002	0.623
VHD	STMN3	20	62271061	rs909333	62270637	G	T	0.207	-0.076	0.036	0.036	0.093

C) Effect of AGER gene on VHD and gastrointestinal disorders.

Disease	Gene	chr	Probe_bp	topSNP	topSNP_bp	A1	A2	Freq	b_SMR	se_SMR	p_SMR	p_HEIDI
CD	AGER	6	32150423	rs693906	31835164	C	G	0.113	-0.312	0.120	0.009	6.16E-08
GORD	AGER	6	32150423	rs693906	31835164	C	G	0.113	-0.209	0.049	2.02E-05	0.022
IBD	AGER	6	32148745	rs204995	32154285	G	A	0.217	-0.252	0.108	0.020	1.60E-04
IBS	AGER	6	32150423	rs693906	31835164	C	G	0.113	-0.168	0.052	0.001	0.045
UC	AGER	6	32148745	rs204995	32154285	G	A	0.217	-0.870	0.185	2.59E-06	0.002
VHD	AGER	6	32150423	rs693906	31835164	C	G	0.113	0.212	0.052	4.82E-05	0.092

Supplementary table 6. Results of the summary-based Mendelian randomization integrating whole-blood expression quantitative trait loci. For each disease-gene association, we report the strongest effect observed between GTEX and eQTLGen datasets.

A) Bonferroni-significant disease-gene association with HEIDI P > 0.05.

Disease	Gene	chr	gene_bp	topSNP	topSNP_bp	A1	A2	Freq	b_SMR	se_SMR	p_SMR	p_HEIDI
CD	DNMT3A	2	25510652	rs7578575	25488819	A	T	0.329	-0.834	0.164	3.51E-07	0.383
IBD	DNMT3A	2	25510652	rs7578575	25488819	A	T	0.329	-0.745	0.130	9.22E-09	0.515
UC	DNMT3A	2	25510652	rs7578575	25488819	A	T	0.329	-0.627	0.156	5.73E-05	0.541
UC	CFDP1	16	75327596	rs9934007	75390532	C	T	0.754	0.273	0.070	9.61E-05	0.973
CD	STAT3	17	40502964	rs1053004	40466092	G	A	0.380	0.702	0.128	4.40E-08	0.056
CD	IMPDH2	3	49064299	rs9813813	49207093	A	T	0.110	-1.233	0.294	2.74E-05	0.213
IBD	IMPDH2	3	49064299	rs9813813	49207093	A	T	0.110	-1.328	0.258	2.57E-07	0.071
UC	IMPDH2	3	49064299	rs9813813	49207093	A	T	0.110	-1.445	0.307	2.53E-06	0.050
IBD	UBE2L3	22	21941029	rs2070512	21949411	C	A	0.194	0.163	0.021	1.06E-14	0.169
UC	UBE2L3	22	21941029	rs2070512	21949411	C	A	0.194	0.116	0.027	1.76E-05	0.698
CD	BCL2L15	1	1.14E+08	rs1217393	1.14E+08	A	G	0.271	-0.705	0.170	3.39E-05	0.184
CD	STMN3	20	62271061	rs909333	62270637	G	T	0.207	0.547	0.102	8.31E-08	0.200
IBD	STMN3	20	62271061	rs909333	62270637	G	T	0.207	0.555	0.087	2.01E-10	0.055
UC	STMN3	20	62271061	rs909333	62270637	G	T	0.207	0.498	0.098	4.04E-07	0.062
GORD	PBX2	6	32154964	rs693906	31835164	C	G	0.113	0.154	0.036	1.65E-05	0.058
VHD	AGER	6	32150423	rs693906	31835164	C	G	0.113	0.212	0.052	4.82E-05	0.092

B) Nominally significant effect on VHD of Bonferroni-significant gastrointestinal loci.

Disease	Gene	chr	Probe_bp	topSNP	topSNP_bp	A1	A2	Freq	b_SMR	se_SMR	p_SMR	p_HEIDI
VHD	CFDP1	16	75397489	rs7201989	75471499	G	A	0.246	-0.056	0.018	0.001	0.119
VHD	STAT3	17	40502964	rs1053004	40466092	G	A	0.380	0.093	0.047	0.048	0.948
VHD	IMPDH2	3	49064299	rs34037363	48821036	T	C	0.114	-0.209	0.105	0.047	0.089
VHD	UBE2L3	22	21941029	rs2070512	21949411	C	A	0.194	-0.029	0.009	0.002	0.623
VHD	STMN3	20	62271061	rs909333	62270637	G	T	0.207	-0.076	0.036	0.036	0.093

C) Effect of AGER gene on VHD and gastrointestinal disorders.

Disease	Gene	chr	Probe_bp	topSNP	topSNP_bp	A1	A2	Freq	b_SMR	se_SMR	p_SMR	p_HEIDI
CD	AGER	6	32150423	rs693906	31835164	C	G	0.113	-0.312	0.120	0.009	6.16E-08
GORD	AGER	6	32150423	rs693906	31835164	C	G	0.113	-0.209	0.049	2.02E-05	0.022
IBD	AGER	6	32148745	rs204995	32154285	G	A	0.217	-0.252	0.108	0.020	1.60E-04
IBS	AGER	6	32150423	rs693906	31835164	C	G	0.113	-0.168	0.052	0.001	0.045
UC	AGER	6	32148745	rs204995	32154285	G	A	0.217	-0.870	0.185	2.59E-06	0.002
VHD	AGER	6	32150423	rs693906	31835164	C	G	0.113	0.212	0.052	4.82E-05	0.092

Supplementary table 6. Results of the summary-based Mendelian randomization integrating whole-blood expression quantitative trait loci. For each disease-gene association, we report the strongest effect observed between GTEX and eQTLGen datasets.

A) Bonferroni-significant disease-gene association with HEIDI P > 0.05.

Disease	Gene	chr	gene_bp	topSNP	topSNP_bp	A1	A2	Freq	b_SMR	se_SMR	p_SMR	p_HEIDI
CD	DNMT3A	2	25510652	rs7578575	25488819	A	T	0.329	-0.834	0.164	3.51E-07	0.383
IBD	DNMT3A	2	25510652	rs7578575	25488819	A	T	0.329	-0.745	0.130	9.22E-09	0.515
UC	DNMT3A	2	25510652	rs7578575	25488819	A	T	0.329	-0.627	0.156	5.73E-05	0.541
UC	CFDP1	16	75327596	rs9934007	75390532	C	T	0.754	0.273	0.070	9.61E-05	0.973
CD	STAT3	17	40502964	rs1053004	40466092	G	A	0.380	0.702	0.128	4.40E-08	0.056
CD	IMPDH2	3	49064299	rs9813813	49207093	A	T	0.110	-1.233	0.294	2.74E-05	0.213
IBD	IMPDH2	3	49064299	rs9813813	49207093	A	T	0.110	-1.328	0.258	2.57E-07	0.071
UC	IMPDH2	3	49064299	rs9813813	49207093	A	T	0.110	-1.445	0.307	2.53E-06	0.050
IBD	UBE2L3	22	21941029	rs2070512	21949411	C	A	0.194	0.163	0.021	1.06E-14	0.169
UC	UBE2L3	22	21941029	rs2070512	21949411	C	A	0.194	0.116	0.027	1.76E-05	0.698
CD	BCL2L15	1	1.14E+08	rs1217393	1.14E+08	A	G	0.271	-0.705	0.170	3.39E-05	0.184
CD	STMN3	20	62271061	rs909333	62270637	G	T	0.207	0.547	0.102	8.31E-08	0.200
IBD	STMN3	20	62271061	rs909333	62270637	G	T	0.207	0.555	0.087	2.01E-10	0.055
UC	STMN3	20	62271061	rs909333	62270637	G	T	0.207	0.498	0.098	4.04E-07	0.062
GORD	PBX2	6	32154964	rs693906	31835164	C	G	0.113	0.154	0.036	1.65E-05	0.058
VHD	AGER	6	32150423	rs693906	31835164	C	G	0.113	0.212	0.052	4.82E-05	0.092

B) Nominally significant effect on VHD of Bonferroni-significant gastrointestinal loci.

Disease	Gene	chr	Probe_bp	topSNP	topSNP_bp	A1	A2	Freq	b_SMR	se_SMR	p_SMR	p_HEIDI
VHD	CFDP1	16	75397489	rs7201989	75471499	G	A	0.246	-0.056	0.018	0.001	0.119
VHD	STAT3	17	40502964	rs1053004	40466092	G	A	0.380	0.093	0.047	0.048	0.948
VHD	IMPDH2	3	49064299	rs34037363	48821036	T	C	0.114	-0.209	0.105	0.047	0.089
VHD	UBE2L3	22	21941029	rs2070512	21949411	C	A	0.194	-0.029	0.009	0.002	0.623
VHD	STMN3	20	62271061	rs909333	62270637	G	T	0.207	-0.076	0.036	0.036	0.093

C) Effect of AGER gene on VHD and gastrointestinal disorders.

Disease	Gene	chr	Probe_bp	topSNP	topSNP_bp	A1	A2	Freq	b_SMR	se_SMR	p_SMR	p_HEIDI
CD	AGER	6	32150423	rs693906	31835164	C	G	0.113	-0.312	0.120	0.009	6.16E-08
GORD	AGER	6	32150423	rs693906	31835164	C	G	0.113	-0.209	0.049	2.02E-05	0.022
IBD	AGER	6	32148745	rs204995	32154285	G	A	0.217	-0.252	0.108	0.020	1.60E-04
IBS	AGER	6	32150423	rs693906	31835164	C	G	0.113	-0.168	0.052	0.001	0.045
UC	AGER	6	32148745	rs204995	32154285	G	A	0.217	-0.870	0.185	2.59E-06	0.002
VHD	AGER	6	32150423	rs693906	31835164	C	G	0.113	0.212	0.052	4.82E-05	0.092

Supplementary table 6. Results of the summary-based Mendelian randomization integrating whole-blood expression quantitative trait loci. For each disease-gene association, we report the strongest effect observed between GTEX and eQTLGen datasets.

A) Bonferroni-significant disease-gene association with HEIDI P > 0.05.

Disease	Gene	chr	gene_bp	topSNP	topSNP_bp	A1	A2	Freq	b_SMR	se_SMR	p_SMR	p_HEIDI
CD	DNMT3A	2	25510652	rs7578575	25488819	A	T	0.329	-0.834	0.164	3.51E-07	0.383
IBD	DNMT3A	2	25510652	rs7578575	25488819	A	T	0.329	-0.745	0.130	9.22E-09	0.515
UC	DNMT3A	2	25510652	rs7578575	25488819	A	T	0.329	-0.627	0.156	5.73E-05	0.541
UC	CFDP1	16	75327596	rs9934007	75390532	C	T	0.754	0.273	0.070	9.61E-05	0.973
CD	STAT3	17	40502964	rs1053004	40466092	G	A	0.380	0.702	0.128	4.40E-08	0.056
CD	IMPDH2	3	49064299	rs9813813	49207093	A	T	0.110	-1.233	0.294	2.74E-05	0.213
IBD	IMPDH2	3	49064299	rs9813813	49207093	A	T	0.110	-1.328	0.258	2.57E-07	0.071
UC	IMPDH2	3	49064299	rs9813813	49207093	A	T	0.110	-1.445	0.307	2.53E-06	0.050
IBD	UBE2L3	22	21941029	rs2070512	21949411	C	A	0.194	0.163	0.021	1.06E-14	0.169
UC	UBE2L3	22	21941029	rs2070512	21949411	C	A	0.194	0.116	0.027	1.76E-05	0.698
CD	BCL2L15	1	1.14E+08	rs1217393	1.14E+08	A	G	0.271	-0.705	0.170	3.39E-05	0.184
CD	STMN3	20	62271061	rs909333	62270637	G	T	0.207	0.547	0.102	8.31E-08	0.200
IBD	STMN3	20	62271061	rs909333	62270637	G	T	0.207	0.555	0.087	2.01E-10	0.055
UC	STMN3	20	62271061	rs909333	62270637	G	T	0.207	0.498	0.098	4.04E-07	0.062
GORD	PBX2	6	32154964	rs693906	31835164	C	G	0.113	0.154	0.036	1.65E-05	0.058
VHD	AGER	6	32150423	rs693906	31835164	C	G	0.113	0.212	0.052	4.82E-05	0.092

B) Nominally significant effect on VHD of Bonferroni-significant gastrointestinal loci.

Disease	Gene	chr	Probe_bp	topSNP	topSNP_bp	A1	A2	Freq	b_SMR	se_SMR	p_SMR	p_HEIDI
VHD	CFDP1	16	75397489	rs7201989	75471499	G	A	0.246	-0.056	0.018	0.001	0.119
VHD	STAT3	17	40502964	rs1053004	40466092	G	A	0.380	0.093	0.047	0.048	0.948
VHD	IMPDH2	3	49064299	rs34037363	48821036	T	C	0.114	-0.209	0.105	0.047	0.089
VHD	UBE2L3	22	21941029	rs2070512	21949411	C	A	0.194	-0.029	0.009	0.002	0.623
VHD	STMN3	20	62271061	rs909333	62270637	G	T	0.207	-0.076	0.036	0.036	0.093

C) Effect of AGER gene on VHD and gastrointestinal disorders.

Disease	Gene	chr	Probe_bp	topSNP	topSNP_bp	A1	A2	Freq	b_SMR	se_SMR	p_SMR	p_HEIDI
CD	AGER	6	32150423	rs693906	31835164	C	G	0.113	-0.312	0.120	0.009	6.16E-08
GORD	AGER	6	32150423	rs693906	31835164	C	G	0.113	-0.209	0.049	2.02E-05	0.022
IBD	AGER	6	32148745	rs204995	32154285	G	A	0.217	-0.252	0.108	0.020	1.60E-04
IBS	AGER	6	32150423	rs693906	31835164	C	G	0.113	-0.168	0.052	0.001	0.045
UC	AGER	6	32148745	rs204995	32154285	G	A	0.217	-0.870	0.185	2.59E-06	0.002
VHD	AGER	6	32150423	rs693906	31835164	C	G	0.113	0.212	0.052	4.82E-05	0.092

Supplementary table 6. Results of the summary-based Mendelian randomization integrating whole-blood expression quantitative trait loci. For each disease-gene association, we report the strongest effect observed between GTEX and eQTLGen datasets.

A) Bonferroni-significant disease-gene association with HEIDI P > 0.05.

Disease	Gene	chr	gene_bp	topSNP	topSNP_bp	A1	A2	Freq	b_SMR	se_SMR	p_SMR	p_HEIDI
CD	DNMT3A	2	25510652	rs7578575	25488819	A	T	0.329	-0.834	0.164	3.51E-07	0.383
IBD	DNMT3A	2	25510652	rs7578575	25488819	A	T	0.329	-0.745	0.130	9.22E-09	0.515
UC	DNMT3A	2	25510652	rs7578575	25488819	A	T	0.329	-0.627	0.156	5.73E-05	0.541
UC	CFDP1	16	75327596	rs9934007	75390532	C	T	0.754	0.273	0.070	9.61E-05	0.973
CD	STAT3	17	40502964	rs1053004	40466092	G	A	0.380	0.702	0.128	4.40E-08	0.056
CD	IMPDH2	3	49064299	rs9813813	49207093	A	T	0.110	-1.233	0.294	2.74E-05	0.213
IBD	IMPDH2	3	49064299	rs9813813	49207093	A	T	0.110	-1.328	0.258	2.57E-07	0.071
UC	IMPDH2	3	49064299	rs9813813	49207093	A	T	0.110	-1.445	0.307	2.53E-06	0.050
IBD	UBE2L3	22	21941029	rs2070512	21949411	C	A	0.194	0.163	0.021	1.06E-14	0.169
UC	UBE2L3	22	21941029	rs2070512	21949411	C	A	0.194	0.116	0.027	1.76E-05	0.698
CD	BCL2L15	1	1.14E+08	rs1217393	1.14E+08	A	G	0.271	-0.705	0.170	3.39E-05	0.184
CD	STMN3	20	62271061	rs909333	62270637	G	T	0.207	0.547	0.102	8.31E-08	0.200
IBD	STMN3	20	62271061	rs909333	62270637	G	T	0.207	0.555	0.087	2.01E-10	0.055
UC	STMN3	20	62271061	rs909333	62270637	G	T	0.207	0.498	0.098	4.04E-07	0.062
GORD	PBX2	6	32154964	rs693906	31835164	C	G	0.113	0.154	0.036	1.65E-05	0.058
VHD	AGER	6	32150423	rs693906	31835164	C	G	0.113	0.212	0.052	4.82E-05	0.092

B) Nominally significant effect on VHD of Bonferroni-significant gastrointestinal loci.

Disease	Gene	chr	Probe_bp	topSNP	topSNP_bp	A1	A2	Freq	b_SMR	se_SMR	p_SMR	p_HEIDI
VHD	CFDP1	16	75397489	rs7201989	75471499	G	A	0.246	-0.056	0.018	0.001	0.119
VHD	STAT3	17	40502964	rs1053004	40466092	G	A	0.380	0.093	0.047	0.048	0.948
VHD	IMPDH2	3	49064299	rs34037363	48821036	T	C	0.114	-0.209	0.105	0.047	0.089
VHD	UBE2L3	22	21941029	rs2070512	21949411	C	A	0.194	-0.029	0.009	0.002	0.623
VHD	STMN3	20	62271061	rs909333	62270637	G	T	0.207	-0.076	0.036	0.036	0.093

C) Effect of AGER gene on VHD and gastrointestinal disorders.

Disease	Gene	chr	Probe_bp	topSNP	topSNP_bp	A1	A2	Freq	b_SMR	se_SMR	p_SMR	p_HEIDI
CD	AGER	6	32150423	rs693906	31835164	C	G	0.113	-0.312	0.120	0.009	6.16E-08
GORD	AGER	6	32150423	rs693906	31835164	C	G	0.113	-0.209	0.049	2.02E-05	0.022
IBD	AGER	6	32148745	rs204995	32154285	G	A	0.217	-0.252	0.108	0.020	1.60E-04
IBS	AGER	6	32150423	rs693906	31835164	C	G	0.113	-0.168	0.052	0.001	0.045
UC	AGER	6	32148745	rs204995	32154285	G	A	0.217	-0.870	0.185	2.59E-06	0.002
VHD	AGER	6	32150423	rs693906	31835164	C	G	0.113	0.212	0.052	4.82E-05	0.092

Supplementary table 6. Results of the summary-based Mendelian randomization integrating whole-blood expression quantitative trait loci. For each disease-gene association, we report the strongest effect observed between GTEX and eQTLGen datasets.

A) Bonferroni-significant disease-gene association with HEIDI P > 0.05.

Disease	Gene	chr	gene_bp	topSNP	topSNP_bp	A1	A2	Freq	b_SMR	se_SMR	p_SMR	p_HEIDI
CD	DNMT3A	2	25510652	rs7578575	25488819	A	T	0.329	-0.834	0.164	3.51E-07	0.383
IBD	DNMT3A	2	25510652	rs7578575	25488819	A	T	0.329	-0.745	0.130	9.22E-09	0.515
UC	DNMT3A	2	25510652	rs7578575	25488819	A	T	0.329	-0.627	0.156	5.73E-05	0.541
UC	CFDP1	16	75327596	rs9934007	75390532	C	T	0.754	0.273	0.070	9.61E-05	0.973
CD	STAT3	17	40502964	rs1053004	40466092	G	A	0.380	0.702	0.128	4.40E-08	0.056
CD	IMPDH2	3	49064299	rs9813813	49207093	A	T	0.110	-1.233	0.294	2.74E-05	0.213
IBD	IMPDH2	3	49064299	rs9813813	49207093	A	T	0.110	-1.328	0.258	2.57E-07	0.071
UC	IMPDH2	3	49064299	rs9813813	49207093	A	T	0.110	-1.445	0.307	2.53E-06	0.050
IBD	UBE2L3	22	21941029	rs2070512	21949411	C	A	0.194	0.163	0.021	1.06E-14	0.169
UC	UBE2L3	22	21941029	rs2070512	21949411	C	A	0.194	0.116	0.027	1.76E-05	0.698
CD	BCL2L15	1	1.14E+08	rs1217393	1.14E+08	A	G	0.271	-0.705	0.170	3.39E-05	0.184
CD	STMN3	20	62271061	rs909333	62270637	G	T	0.207	0.547	0.102	8.31E-08	0.200
IBD	STMN3	20	62271061	rs909333	62270637	G	T	0.207	0.555	0.087	2.01E-10	0.055
UC	STMN3	20	62271061	rs909333	62270637	G	T	0.207	0.498	0.098	4.04E-07	0.062
GORD	PBX2	6	32154964	rs693906	31835164	C	G	0.113	0.154	0.036	1.65E-05	0.058
VHD	AGER	6	32150423	rs693906	31835164	C	G	0.113	0.212	0.052	4.82E-05	0.092

B) Nominally significant effect on VHD of Bonferroni-significant gastrointestinal loci.

Disease	Gene	chr	Probe_bp	topSNP	topSNP_bp	A1	A2	Freq	b_SMR	se_SMR	p_SMR	p_HEIDI
VHD	CFDP1	16	75397489	rs7201989	75471499	G	A	0.246	-0.056	0.018	0.001	0.119
VHD	STAT3	17	40502964	rs1053004	40466092	G	A	0.380	0.093	0.047	0.048	0.948
VHD	IMPDH2	3	49064299	rs34037363	48821036	T	C	0.114	-0.209	0.105	0.047	0.089
VHD	UBE2L3	22	21941029	rs2070512	21949411	C	A	0.194	-0.029	0.009	0.002	0.623
VHD	STMN3	20	62271061	rs909333	62270637	G	T	0.207	-0.076	0.036	0.036	0.093

C) Effect of AGER gene on VHD and gastrointestinal disorders.

Disease	Gene	chr	Probe_bp	topSNP	topSNP_bp	A1	A2	Freq	b_SMR	se_SMR	p_SMR	p_HEIDI
CD	AGER	6	32150423	rs693906	31835164	C	G	0.113	-0.312	0.120	0.009	6.16E-08
GORD	AGER	6	32150423	rs693906	31835164	C	G	0.113	-0.209	0.049	2.02E-05	0.022
IBD	AGER	6	32148745	rs204995	32154285	G	A	0.217	-0.252	0.108	0.020	1.60E-04
IBS	AGER	6	32150423	rs693906	31835164	C	G	0.113	-0.168	0.052	0.001	0.045
UC	AGER	6	32148745	rs204995	32154285	G	A	0.217	-0.870	0.185	2.59E-06	0.002
VHD	AGER	6	32150423	rs693906	31835164	C	G	0.113	0.212	0.052	4.82E-05	0.092

Supplementary table 6. Results of the summary-based Mendelian randomization integrating whole-blood expression quantitative trait loci. For each disease-gene association, we report the strongest effect observed between GTEX and eQTLGen datasets.

A) Bonferroni-significant disease-gene association with HEIDI P > 0.05.

Disease	Gene	chr	gene_bp	topSNP	topSNP_bp	A1	A2	Freq	b_SMR	se_SMR	p_SMR	p_HEIDI
CD	DNMT3A	2	25510652	rs7578575	25488819	A	T	0.329	-0.834	0.164	3.51E-07	0.383
IBD	DNMT3A	2	25510652	rs7578575	25488819	A	T	0.329	-0.745	0.130	9.22E-09	0.515
UC	DNMT3A	2	25510652	rs7578575	25488819	A	T	0.329	-0.627	0.156	5.73E-05	0.541
UC	CFDP1	16	75327596	rs9934007	75390532	C	T	0.754	0.273	0.070	9.61E-05	0.973
CD	STAT3	17	40502964	rs1053004	40466092	G	A	0.380	0.702	0.128	4.40E-08	0.056
CD	IMPDH2	3	49064299	rs9813813	49207093	A	T	0.110	-1.233	0.294	2.74E-05	0.213
IBD	IMPDH2	3	49064299	rs9813813	49207093	A	T	0.110	-1.328	0.258	2.57E-07	0.071
UC	IMPDH2	3	49064299	rs9813813	49207093	A	T	0.110	-1.445	0.307	2.53E-06	0.050
IBD	UBE2L3	22	21941029	rs2070512	21949411	C	A	0.194	0.163	0.021	1.06E-14	0.169
UC	UBE2L3	22	21941029	rs2070512	21949411	C	A	0.194	0.116	0.027	1.76E-05	0.698
CD	BCL2L15	1	1.14E+08	rs1217393	1.14E+08	A	G	0.271	-0.705	0.170	3.39E-05	0.184
CD	STMN3	20	62271061	rs909333	62270637	G	T	0.207	0.547	0.102	8.31E-08	0.200
IBD	STMN3	20	62271061	rs909333	62270637	G	T	0.207	0.555	0.087	2.01E-10	0.055
UC	STMN3	20	62271061	rs909333	62270637	G	T	0.207	0.498	0.098	4.04E-07	0.062
GORD	PBX2	6	32154964	rs693906	31835164	C	G	0.113	0.154	0.036	1.65E-05	0.058
VHD	AGER	6	32150423	rs693906	31835164	C	G	0.113	0.212	0.052	4.82E-05	0.092

B) Nominally significant effect on VHD of Bonferroni-significant gastrointestinal loci.

Disease	Gene	chr	Probe_bp	topSNP	topSNP_bp	A1	A2	Freq	b_SMR	se_SMR	p_SMR	p_HEIDI
VHD	CFDP1	16	75397489	rs7201989	75471499	G	A	0.246	-0.056	0.018	0.001	0.119
VHD	STAT3	17	40502964	rs1053004	40466092	G	A	0.380	0.093	0.047	0.048	0.948
VHD	IMPDH2	3	49064299	rs34037363	48821036	T	C	0.114	-0.209	0.105	0.047	0.089
VHD	UBE2L3	22	21941029	rs2070512	21949411	C	A	0.194	-0.029	0.009	0.002	0.623
VHD	STMN3	20	62271061	rs909333	62270637	G	T	0.207	-0.076	0.036	0.036	0.093

C) Effect of AGER gene on VHD and gastrointestinal disorders.

Disease	Gene	chr	Probe_bp	topSNP	topSNP_bp	A1	A2	Freq	b_SMR	se_SMR	p_SMR	p_HEIDI
CD	AGER	6	32150423	rs693906	31835164	C	G	0.113	-0.312	0.120	0.009	6.16E-08
GORD	AGER	6	32150423	rs693906	31835164	C	G	0.113	-0.209	0.049	2.02E-05	0.022
IBD	AGER	6	32148745	rs204995	32154285	G	A	0.217	-0.252	0.108	0.020	1.60E-04
IBS	AGER	6	32150423	rs693906	31835164	C	G	0.113	-0.168	0.052	0.001	0.045
UC	AGER	6	32148745	rs204995	32154285	G	A	0.217	-0.870	0.185	2.59E-06	0.002
VHD	AGER	6	32150423	rs693906	31835164	C	G	0.113	0.212	0.052	4.82E-05	0.092

Supplementary table 6. Results of the summary-based Mendelian randomization integrating whole-blood expression quantitative trait loci. For each disease-gene association, we report the strongest effect observed between GTEX and eQTLGen datasets.

A) Bonferroni-significant disease-gene association with HEIDI P > 0.05.

Disease	Gene	chr	gene_bp	topSNP	topSNP_bp	A1	A2	Freq	b_SMR	se_SMR	p_SMR	p_HEIDI
CD	DNMT3A	2	25510652	rs7578575	25488819	A	T	0.329	-0.834	0.164	3.51E-07	0.383
IBD	DNMT3A	2	25510652	rs7578575	25488819	A	T	0.329	-0.745	0.130	9.22E-09	0.515
UC	DNMT3A	2	25510652	rs7578575	25488819	A	T	0.329	-0.627	0.156	5.73E-05	0.541
UC	CFDP1	16	75327596	rs9934007	75390532	C	T	0.754	0.273	0.070	9.61E-05	0.973
CD	STAT3	17	40502964	rs1053004	40466092	G	A	0.380	0.702	0.128	4.40E-08	0.056
CD	IMPDH2	3	49064299	rs9813813	49207093	A	T	0.110	-1.233	0.294	2.74E-05	0.213
IBD	IMPDH2	3	49064299	rs9813813	49207093	A	T	0.110	-1.328	0.258	2.57E-07	0.071
UC	IMPDH2	3	49064299	rs9813813	49207093	A	T	0.110	-1.445	0.307	2.53E-06	0.050
IBD	UBE2L3	22	21941029	rs2070512	21949411	C	A	0.194	0.163	0.021	1.06E-14	0.169
UC	UBE2L3	22	21941029	rs2070512	21949411	C	A	0.194	0.116	0.027	1.76E-05	0.698
CD	BCL2L15	1	1.14E+08	rs1217393	1.14E+08	A	G	0.271	-0.705	0.170	3.39E-05	0.184
CD	STMN3	20	62271061	rs909333	62270637	G	T	0.207	0.547	0.102	8.31E-08	0.200
IBD	STMN3	20	62271061	rs909333	62270637	G	T	0.207	0.555	0.087	2.01E-10	0.055
UC	STMN3	20	62271061	rs909333	62270637	G	T	0.207	0.498	0.098	4.04E-07	0.062
GORD	PBX2	6	32154964	rs693906	31835164	C	G	0.113	0.154	0.036	1.65E-05	0.058
VHD	AGER	6	32150423	rs693906	31835164	C	G	0.113	0.212	0.052	4.82E-05	0.092

B) Nominally significant effect on VHD of Bonferroni-significant gastrointestinal loci.

Disease	Gene	chr	Probe_bp	topSNP	topSNP_bp	A1	A2	Freq	b_SMR	se_SMR	p_SMR	p_HEIDI
VHD	CFDP1	16	75397489	rs7201989	75471499	G	A	0.246	-0.056	0.018	0.001	0.119
VHD	STAT3	17	40502964	rs1053004	40466092	G	A	0.380	0.093	0.047	0.048	0.948
VHD	IMPDH2	3	49064299	rs34037363	48821036	T	C	0.114	-0.209	0.105	0.047	0.089
VHD	UBE2L3	22	21941029	rs2070512	21949411	C	A	0.194	-0.029	0.009	0.002	0.623
VHD	STMN3	20	62271061	rs909333	62270637	G	T	0.207	-0.076	0.036	0.036	0.093

C) Effect of AGER gene on VHD and gastrointestinal disorders.

Disease	Gene	chr	Probe_bp	topSNP	topSNP_bp	A1	A2	Freq	b_SMR	se_SMR	p_SMR	p_HEIDI
CD	AGER	6	32150423	rs693906	31835164	C	G	0.113	-0.312	0.120	0.009	6.16E-08
GORD	AGER	6	32150423	rs693906	31835164	C	G	0.113	-0.209	0.049	2.02E-05	0.022
IBD	AGER	6	32148745	rs204995	32154285	G	A	0.217	-0.252	0.108	0.020	1.60E-04
IBS	AGER	6	32150423	rs693906	31835164	C	G	0.113	-0.168	0.052	0.001	0.045
UC	AGER	6	32148745	rs204995	32154285	G	A	0.217	-0.870	0.185	2.59E-06	0.002
VHD	AGER	6	32150423	rs693906	31835164	C	G	0.113	0.212	0.052	4.82E-05	0.092

Supplementary table 6. Results of the summary-based Mendelian randomization integrating whole-blood expression quantitative trait loci. For each disease-gene association, we report the strongest effect observed between GTEX and eQTLGen datasets.

A) Bonferroni-significant disease-gene association with HEIDI P > 0.05.

Disease	Gene	chr	gene_bp	topSNP	topSNP_bp	A1	A2	Freq	b_SMR	se_SMR	p_SMR	p_HEIDI
CD	DNMT3A	2	25510652	rs7578575	25488819	A	T	0.329	-0.834	0.164	3.51E-07	0.383
IBD	DNMT3A	2	25510652	rs7578575	25488819	A	T	0.329	-0.745	0.130	9.22E-09	0.515
UC	DNMT3A	2	25510652	rs7578575	25488819	A	T	0.329	-0.627	0.156	5.73E-05	0.541
UC	CFDP1	16	75327596	rs9934007	75390532	C	T	0.754	0.273	0.070	9.61E-05	0.973
CD	STAT3	17	40502964	rs1053004	40466092	G	A	0.380	0.702	0.128	4.40E-08	0.056
CD	IMPDH2	3	49064299	rs9813813	49207093	A	T	0.110	-1.233	0.294	2.74E-05	0.213
IBD	IMPDH2	3	49064299	rs9813813	49207093	A	T	0.110	-1.328	0.258	2.57E-07	0.071
UC	IMPDH2	3	49064299	rs9813813	49207093	A	T	0.110	-1.445	0.307	2.53E-06	0.050
IBD	UBE2L3	22	21941029	rs2070512	21949411	C	A	0.194	0.163	0.021	1.06E-14	0.169
UC	UBE2L3	22	21941029	rs2070512	21949411	C	A	0.194	0.116	0.027	1.76E-05	0.698
CD	BCL2L15	1	1.14E+08	rs1217393	1.14E+08	A	G	0.271	-0.705	0.170	3.39E-05	0.184
CD	STMN3	20	62271061	rs909333	62270637	G	T	0.207	0.547	0.102	8.31E-08	0.200
IBD	STMN3	20	62271061	rs909333	62270637	G	T	0.207	0.555	0.087	2.01E-10	0.055
UC	STMN3	20	62271061	rs909333	62270637	G	T	0.207	0.498	0.098	4.04E-07	0.062
GORD	PBX2	6	32154964	rs693906	31835164	C	G	0.113	0.154	0.036	1.65E-05	0.058
VHD	AGER	6	32150423	rs693906	31835164	C	G	0.113	0.212	0.052	4.82E-05	0.092

B) Nominally significant effect on VHD of Bonferroni-significant gastrointestinal loci.

Disease	Gene	chr	Probe_bp	topSNP	topSNP_bp	A1	A2	Freq	b_SMR	se_SMR	p_SMR	p_HEIDI
VHD	CFDP1	16	75397489	rs7201989	75471499	G	A	0.246	-0.056	0.018	0.001	0.119
VHD	STAT3	17	40502964	rs1053004	40466092	G	A	0.380	0.093	0.047	0.048	0.948
VHD	IMPDH2	3	49064299	rs34037363	48821036	T	C	0.114	-0.209	0.105	0.047	0.089
VHD	UBE2L3	22	21941029	rs2070512	21949411	C	A	0.194	-0.029	0.009	0.002	0.623
VHD	STMN3	20	62271061	rs909333	62270637	G	T	0.207	-0.076	0.036	0.036	0.093

C) Effect of AGER gene on VHD and gastrointestinal disorders.

Disease	Gene	chr	Probe_bp	topSNP	topSNP_bp	A1	A2	Freq	b_SMR	se_SMR	p_SMR	p_HEIDI
CD	AGER	6	32150423	rs693906	31835164	C	G	0.113	-0.312	0.120	0.009	6.16E-08
GORD	AGER	6	32150423	rs693906	31835164	C	G	0.113	-0.209	0.049	2.02E-05	0.022
IBD	AGER	6	32148745	rs204995	32154285	G	A	0.217	-0.252	0.108	0.020	1.60E-04
IBS	AGER	6	32150423	rs693906	31835164	C	G	0.113	-0.168	0.052	0.001	0.045
UC	AGER	6	32148745	rs204995	32154285	G	A	0.217	-0.870	0.185	2.59E-06	0.002
VHD	AGER	6	32150423	rs693906	31835164	C	G	0.113	0.212	0.052	4.82E-05	0.092

Supplementary table 6. Results of the summary-based Mendelian randomization integrating whole-blood expression quantitative trait loci. For each disease-gene association, we report the strongest effect observed between GTEX and eQTLGen datasets.

A) Bonferroni-significant disease-gene association with HEIDI P > 0.05.

Disease	Gene	chr	gene_bp	topSNP	topSNP_bp	A1	A2	Freq	b_SMR	se_SMR	p_SMR	p_HEIDI
CD	DNMT3A	2	25510652	rs7578575	25488819	A	T	0.329	-0.834	0.164	3.51E-07	0.383
IBD	DNMT3A	2	25510652	rs7578575	25488819	A	T	0.329	-0.745	0.130	9.22E-09	0.515
UC	DNMT3A	2	25510652	rs7578575	25488819	A	T	0.329	-0.627	0.156	5.73E-05	0.541
UC	CFDP1	16	75327596	rs9934007	75390532	C	T	0.754	0.273	0.070	9.61E-05	0.973
CD	STAT3	17	40502964	rs1053004	40466092	G	A	0.380	0.702	0.128	4.40E-08	0.056
CD	IMPDH2	3	49064299	rs9813813	49207093	A	T	0.110	-1.233	0.294	2.74E-05	0.213
IBD	IMPDH2	3	49064299	rs9813813	49207093	A	T	0.110	-1.328	0.258	2.57E-07	0.071
UC	IMPDH2	3	49064299	rs9813813	49207093	A	T	0.110	-1.445	0.307	2.53E-06	0.050
IBD	UBE2L3	22	21941029	rs2070512	21949411	C	A	0.194	0.163	0.021	1.06E-14	0.169
UC	UBE2L3	22	21941029	rs2070512	21949411	C	A	0.194	0.116	0.027	1.76E-05	0.698
CD	BCL2L15	1	1.14E+08	rs1217393	1.14E+08	A	G	0.271	-0.705	0.170	3.39E-05	0.184
CD	STMN3	20	62271061	rs909333	62270637	G	T	0.207	0.547	0.102	8.31E-08	0.200
IBD	STMN3	20	62271061	rs909333	62270637	G	T	0.207	0.555	0.087	2.01E-10	0.055
UC	STMN3	20	62271061	rs909333	62270637	G	T	0.207	0.498	0.098	4.04E-07	0.062
GORD	PBX2	6	32154964	rs693906	31835164	C	G	0.113	0.154	0.036	1.65E-05	0.058
VHD	AGER	6	32150423	rs693906	31835164	C	G	0.113	0.212	0.052	4.82E-05	0.092

B) Nominally significant effect on VHD of Bonferroni-significant gastrointestinal loci.

Disease	Gene	chr	Probe_bp	topSNP	topSNP_bp	A1	A2	Freq	b_SMR	se_SMR	p_SMR	p_HEIDI
VHD	CFDP1	16	75397489	rs7201989	75471499	G	A	0.246	-0.056	0.018	0.001	0.119
VHD	STAT3	17	40502964	rs1053004	40466092	G	A	0.380	0.093	0.047	0.048	0.948
VHD	IMPDH2	3	49064299	rs34037363	48821036	T	C	0.114	-0.209	0.105	0.047	0.089
VHD	UBE2L3	22	21941029	rs2070512	21949411	C	A	0.194	-0.029	0.009	0.002	0.623
VHD	STMN3	20	62271061	rs909333	62270637	G	T	0.207	-0.076	0.036	0.036	0.093

C) Effect of AGER gene on VHD and gastrointestinal disorders.

Disease	Gene	chr	Probe_bp	topSNP	topSNP_bp	A1	A2	Freq	b_SMR	se_SMR	p_SMR	p_HEIDI
CD	AGER	6	32150423	rs693906	31835164	C	G	0.113	-0.312	0.120	0.009	6.16E-08
GORD	AGER	6	32150423	rs693906	31835164	C	G	0.113	-0.209	0.049	2.02E-05	0.022
IBD	AGER	6	32148745	rs204995	32154285	G	A	0.217	-0.252	0.108	0.020	1.60E-04
IBS	AGER	6	32150423	rs693906	31835164	C	G	0.113	-0.168	0.052	0.001	0.045
UC	AGER	6	32148745	rs204995	32154285	G	A	0.217	-0.870	0.185	2.59E-06	0.002
VHD	AGER	6	32150423	rs693906	31835164	C	G	0.113	0.212	0.052	4.82E-05	0.092

Supplementary table 6. Results of the summary-based Mendelian randomization integrating whole-blood expression quantitative trait loci. For each disease-gene association, we report the strongest effect observed between GTEX and eQTLGen datasets.

A) Bonferroni-significant disease-gene association with HEIDI P > 0.05.

Disease	Gene	chr	gene_bp	topSNP	topSNP_bp	A1	A2	Freq	b_SMR	se_SMR	p_SMR	p_HEIDI
CD	DNMT3A	2	25510652	rs7578575	25488819	A	T	0.329	-0.834	0.164	3.51E-07	0.383
IBD	DNMT3A	2	25510652	rs7578575	25488819	A	T	0.329	-0.745	0.130	9.22E-09	0.515
UC	DNMT3A	2	25510652	rs7578575	25488819	A	T	0.329	-0.627	0.156	5.73E-05	0.541
UC	CFDP1	16	75327596	rs9934007	75390532	C	T	0.754	0.273	0.070	9.61E-05	0.973
CD	STAT3	17	40502964	rs1053004	40466092	G	A	0.380	0.702	0.128	4.40E-08	0.056
CD	IMPDH2	3	49064299	rs9813813	49207093	A	T	0.110	-1.233	0.294	2.74E-05	0.213
IBD	IMPDH2	3	49064299	rs9813813	49207093	A	T	0.110	-1.328	0.258	2.57E-07	0.071
UC	IMPDH2	3	49064299	rs9813813	49207093	A	T	0.110	-1.445	0.307	2.53E-06	0.050
IBD	UBE2L3	22	21941029	rs2070512	21949411	C	A	0.194	0.163	0.021	1.06E-14	0.169
UC	UBE2L3	22	21941029	rs2070512	21949411	C	A	0.194	0.116	0.027	1.76E-05	0.698
CD	BCL2L15	1	1.14E+08	rs1217393	1.14E+08	A	G	0.271	-0.705	0.170	3.39E-05	0.184
CD	STMN3	20	62271061	rs909333	62270637	G	T	0.207	0.547	0.102	8.31E-08	0.200
IBD	STMN3	20	62271061	rs909333	62270637	G	T	0.207	0.555	0.087	2.01E-10	0.055
UC	STMN3	20	62271061	rs909333	62270637	G	T	0.207	0.498	0.098	4.04E-07	0.062
GORD	PBX2	6	32154964	rs693906	31835164	C	G	0.113	0.154	0.036	1.65E-05	0.058
VHD	AGER	6	32150423	rs693906	31835164	C	G	0.113	0.212	0.052	4.82E-05	0.092

B) Nominally significant effect on VHD of Bonferroni-significant gastrointestinal loci.

Disease	Gene	chr	Probe_bp	topSNP	topSNP_bp	A1	A2	Freq	b_SMR	se_SMR	p_SMR	p_HEIDI
VHD	CFDP1	16	75397489	rs7201989	75471499	G	A	0.246	-0.056	0.018	0.001	0.119
VHD	STAT3	17	40502964	rs1053004	40466092	G	A	0.380	0.093	0.047	0.048	0.948
VHD	IMPDH2	3	49064299	rs34037363	48821036	T	C	0.114	-0.209	0.105	0.047	0.089
VHD	UBE2L3	22	21941029	rs2070512	21949411	C	A	0.194	-0.029	0.009	0.002	0.623
VHD	STMN3	20	62271061	rs909333	62270637	G	T	0.207	-0.076	0.036	0.036	0.093

C) Effect of AGER gene on VHD and gastrointestinal disorders.

Disease	Gene	chr	Probe_bp	topSNP	topSNP_bp	A1	A2	Freq	b_SMR	se_SMR	p_SMR	p_HEIDI
CD	AGER	6	32150423	rs693906	31835164	C	G	0.113	-0.312	0.120	0.009	6.16E-08
GORD	AGER	6	32150423	rs693906	31835164	C	G	0.113	-0.209	0.049	2.02E-05	0.022
IBD	AGER	6	32148745	rs204995	32154285	G	A	0.217	-0.252	0.108	0.020	1.60E-04
IBS	AGER	6	32150423	rs693906	31835164	C	G	0.113	-0.168	0.052	0.001	0.045
UC	AGER	6	32148745	rs204995	32154285	G	A	0.217	-0.870	0.185	2.59E-06	0.002
VHD	AGER	6	32150423	rs693906	31835164	C	G	0.113	0.212	0.052	4.82E-05	0.092

Supplementary table 6. Results of the summary-based Mendelian randomization integrating whole-blood expression quantitative trait loci. For each disease-gene association, we report the strongest effect observed between GTEX and eQTLGen datasets.

A) Bonferroni-significant disease-gene association with HEIDI P > 0.05.

Disease	Gene	chr	gene_bp	topSNP	topSNP_bp	A1	A2	Freq	b_SMR	se_SMR	p_SMR	p_HEIDI
CD	DNMT3A	2	25510652	rs7578575	25488819	A	T	0.329	-0.834	0.164	3.51E-07	0.383
IBD	DNMT3A	2	25510652	rs7578575	25488819	A	T	0.329	-0.745	0.130	9.22E-09	0.515
UC	DNMT3A	2	25510652	rs7578575	25488819	A	T	0.329	-0.627	0.156	5.73E-05	0.541
UC	CFDP1	16	75327596	rs9934007	75390532	C	T	0.754	0.273	0.070	9.61E-05	0.973
CD	STAT3	17	40502964	rs1053004	40466092	G	A	0.380	0.702	0.128	4.40E-08	0.056
CD	IMPDH2	3	49064299	rs9813813	49207093	A	T	0.110	-1.233	0.294	2.74E-05	0.213
IBD	IMPDH2	3	49064299	rs9813813	49207093	A	T	0.110	-1.328	0.258	2.57E-07	0.071
UC	IMPDH2	3	49064299	rs9813813	49207093	A	T	0.110	-1.445	0.307	2.53E-06	0.050
IBD	UBE2L3	22	21941029	rs2070512	21949411	C	A	0.194	0.163	0.021	1.06E-14	0.169
UC	UBE2L3	22	21941029	rs2070512	21949411	C	A	0.194	0.116	0.027	1.76E-05	0.698
CD	BCL2L15	1	1.14E+08	rs1217393	1.14E+08	A	G	0.271	-0.705	0.170	3.39E-05	0.184
CD	STMN3	20	62271061	rs909333	62270637	G	T	0.207	0.547	0.102	8.31E-08	0.200
IBD	STMN3	20	62271061	rs909333	62270637	G	T	0.207	0.555	0.087	2.01E-10	0.055
UC	STMN3	20	62271061	rs909333	62270637	G	T	0.207	0.498	0.098	4.04E-07	0.062
GORD	PBX2	6	32154964	rs693906	31835164	C	G	0.113	0.154	0.036	1.65E-05	0.058
VHD	AGER	6	32150423	rs693906	31835164	C	G	0.113	0.212	0.052	4.82E-05	0.092

B) Nominally significant effect on VHD of Bonferroni-significant gastrointestinal loci.

Disease	Gene	chr	Probe_bp	topSNP	topSNP_bp	A1	A2	Freq	b_SMR	se_SMR	p_SMR	p_HEIDI
VHD	CFDP1	16	75397489	rs7201989	75471499	G	A	0.246	-0.056	0.018	0.001	0.119
VHD	STAT3	17	40502964	rs1053004	40466092	G	A	0.380	0.093	0.047	0.048	0.948
VHD	IMPDH2	3	49064299	rs34037363	48821036	T	C	0.114	-0.209	0.105	0.047	0.089
VHD	UBE2L3	22	21941029	rs2070512	21949411	C	A	0.194	-0.029	0.009	0.002	0.623
VHD	STMN3	20	62271061	rs909333	62270637	G	T	0.207	-0.076	0.036	0.036	0.093

C) Effect of AGER gene on VHD and gastrointestinal disorders.

Disease	Gene	chr	Probe_bp	topSNP	topSNP_bp	A1	A2	Freq	b_SMR	se_SMR	p_SMR	p_HEIDI
CD	AGER	6	32150423	rs693906	31835164	C	G	0.113	-0.312	0.120	0.009	6.16E-08
GORD	AGER	6	32150423	rs693906	31835164	C	G	0.113	-0.209	0.049	2.02E-05	0.022
IBD	AGER	6	32148745	rs204995	32154285	G	A	0.217	-0.252	0.108	0.020	1.60E-04
IBS	AGER	6	32150423	rs693906	31835164	C	G	0.113	-0.168	0.052	0.001	0.045
UC	AGER	6	32148745	rs204995	32154285	G	A	0.217	-0.870	0.185	2.59E-06	0.002
VHD	AGER	6	32150423	rs693906	31835164	C	G	0.113	0.212	0.052	4.82E-05	0.092

Supplementary table 6. Results of the summary-based Mendelian randomization integrating whole-blood expression quantitative trait loci. For each disease-gene association, we report the strongest effect observed between GTEX and eQTLGen datasets.

A) Bonferroni-significant disease-gene association with HEIDI P > 0.05.

Disease	Gene	chr	gene_bp	topSNP	topSNP_bp	A1	A2	Freq	b_SMR	se_SMR	p_SMR	p_HEIDI
CD	DNMT3A	2	25510652	rs7578575	25488819	A	T	0.329	-0.834	0.164	3.51E-07	0.383
IBD	DNMT3A	2	25510652	rs7578575	25488819	A	T	0.329	-0.745	0.130	9.22E-09	0.515
UC	DNMT3A	2	25510652	rs7578575	25488819	A	T	0.329	-0.627	0.156	5.73E-05	0.541
UC	CFDP1	16	75327596	rs9934007	75390532	C	T	0.754	0.273	0.070	9.61E-05	0.973
CD	STAT3	17	40502964	rs1053004	40466092	G	A	0.380	0.702	0.128	4.40E-08	0.056
CD	IMPDH2	3	49064299	rs9813813	49207093	A	T	0.110	-1.233	0.294	2.74E-05	0.213
IBD	IMPDH2	3	49064299	rs9813813	49207093	A	T	0.110	-1.328	0.258	2.57E-07	0.071
UC	IMPDH2	3	49064299	rs9813813	49207093	A	T	0.110	-1.445	0.307	2.53E-06	0.050
IBD	UBE2L3	22	21941029	rs2070512	21949411	C	A	0.194	0.163	0.021	1.06E-14	0.169
UC	UBE2L3	22	21941029	rs2070512	21949411	C	A	0.194	0.116	0.027	1.76E-05	0.698
CD	BCL2L15	1	1.14E+08	rs1217393	1.14E+08	A	G	0.271	-0.705	0.170	3.39E-05	0.184
CD	STMN3	20	62271061	rs909333	62270637	G	T	0.207	0.547	0.102	8.31E-08	0.200
IBD	STMN3	20	62271061	rs909333	62270637	G	T	0.207	0.555	0.087	2.01E-10	0.055
UC	STMN3	20	62271061	rs909333	62270637	G	T	0.207	0.498	0.098	4.04E-07	0.062
GORD	PBX2	6	32154964	rs693906	31835164	C	G	0.113	0.154	0.036	1.65E-05	0.058
VHD	AGER	6	32150423	rs693906	31835164	C	G	0.113	0.212	0.052	4.82E-05	0.092

B) Nominally significant effect on VHD of Bonferroni-significant gastrointestinal loci.

Disease	Gene	chr	Probe_bp	topSNP	topSNP_bp	A1	A2	Freq	b_SMR	se_SMR	p_SMR	p_HEIDI
VHD	CFDP1	16	75397489	rs7201989	75471499	G	A	0.246	-0.056	0.018	0.001	0.119
VHD	STAT3	17	40502964	rs1053004	40466092	G	A	0.380	0.093	0.047	0.048	0.948
VHD	IMPDH2	3	49064299	rs34037363	48821036	T	C	0.114	-0.209	0.105	0.047	0.089
VHD	UBE2L3	22	21941029	rs2070512	21949411	C	A	0.194	-0.029	0.009	0.002	0.623
VHD	STMN3	20	62271061	rs909333	62270637	G	T	0.207	-0.076	0.036	0.036	0.093

C) Effect of AGER gene on VHD and gastrointestinal disorders.

Disease	Gene	chr	Probe_bp	topSNP	topSNP_bp	A1	A2	Freq	b_SMR	se_SMR	p_SMR	p_HEIDI
CD	AGER	6	32150423	rs693906	31835164	C	G	0.113	-0.312	0.120	0.009	6.16E-08
GORD	AGER	6	32150423	rs693906	31835164	C	G	0.113	-0.209	0.049	2.02E-05	0.022
IBD	AGER	6	32148745	rs204995	32154285	G	A	0.217	-0.252	0.108	0.020	1.60E-04
IBS	AGER	6	32150423	rs693906	31835164	C	G	0.113	-0.168	0.052	0.001	0.045
UC	AGER	6	32148745	rs204995	32154285	G	A	0.217	-0.870	0.185	2.59E-06	0.002
VHD	AGER	6	32150423	rs693906	31835164	C	G	0.113	0.212	0.052	4.82E-05	0.092

Supplementary table 6. Results of the summary-based Mendelian randomization integrating whole-blood expression quantitative trait loci. For each disease-gene association, we report the strongest effect observed between GTEX and eQTLGen datasets.

A) Bonferroni-significant disease-gene association with HEIDI P > 0.05.

Disease	Gene	chr	gene_bp	topSNP	topSNP_bp	A1	A2	Freq	b_SMR	se_SMR	p_SMR	p_HEIDI
CD	DNMT3A	2	25510652	rs7578575	25488819	A	T	0.329	-0.834	0.164	3.51E-07	0.383
IBD	DNMT3A	2	25510652	rs7578575	25488819	A	T	0.329	-0.745	0.130	9.22E-09	0.515
UC	DNMT3A	2	25510652	rs7578575	25488819	A	T	0.329	-0.627	0.156	5.73E-05	0.541
UC	CFDP1	16	75327596	rs9934007	75390532	C	T	0.754	0.273	0.070	9.61E-05	0.973
CD	STAT3	17	40502964	rs1053004	40466092	G	A	0.380	0.702	0.128	4.40E-08	0.056
CD	IMPDH2	3	49064299	rs9813813	49207093	A	T	0.110	-1.233	0.294	2.74E-05	0.213
IBD	IMPDH2	3	49064299	rs9813813	49207093	A	T	0.110	-1.328	0.258	2.57E-07	0.071
UC	IMPDH2	3	49064299	rs9813813	49207093	A	T	0.110	-1.445	0.307	2.53E-06	0.050
IBD	UBE2L3	22	21941029	rs2070512	21949411	C	A	0.194	0.163	0.021	1.06E-14	0.169
UC	UBE2L3	22	21941029	rs2070512	21949411	C	A	0.194	0.116	0.027	1.76E-05	0.698
CD	BCL2L15	1	1.14E+08	rs1217393	1.14E+08	A	G	0.271	-0.705	0.170	3.39E-05	0.184
CD	STMN3	20	62271061	rs909333	62270637	G	T	0.207	0.547	0.102	8.31E-08	0.200
IBD	STMN3	20	62271061	rs909333	62270637	G	T	0.207	0.555	0.087	2.01E-10	0.055
UC	STMN3	20	62271061	rs909333	62270637	G	T	0.207	0.498	0.098	4.04E-07	0.062
GORD	PBX2	6	32154964	rs693906	31835164	C	G	0.113	0.154	0.036	1.65E-05	0.058
VHD	AGER	6	32150423	rs693906	31835164	C	G	0.113	0.212	0.052	4.82E-05	0.092

B) Nominally significant effect on VHD of Bonferroni-significant gastrointestinal loci.

Disease	Gene	chr	Probe_bp	topSNP	topSNP_bp	A1	A2	Freq	b_SMR	se_SMR	p_SMR	p_HEIDI
VHD	CFDP1	16	75397489	rs7201989	75471499	G	A	0.246	-0.056	0.018	0.001	0.119
VHD	STAT3	17	40502964	rs1053004	40466092	G	A	0.380	0.093	0.047	0.048	0.948
VHD	IMPDH2	3	49064299	rs34037363	48821036	T	C	0.114	-0.209	0.105	0.047	0.089
VHD	UBE2L3	22	21941029	rs2070512	21949411	C	A	0.194	-0.029	0.009	0.002	0.623
VHD	STMN3	20	62271061	rs909333	62270637	G	T	0.207	-0.076	0.036	0.036	0.093

C) Effect of AGER gene on VHD and gastrointestinal disorders.

Disease	Gene	chr	Probe_bp	topSNP	topSNP_bp	A1	A2	Freq	b_SMR	se_SMR	p_SMR	p_HEIDI
CD	AGER	6	32150423	rs693906	31835164	C	G	0.113	-0.312	0.120	0.009	6.16E-08
GORD	AGER	6	32150423	rs693906	31835164	C	G	0.113	-0.209	0.049	2.02E-05	0.022
IBD	AGER	6	32148745	rs204995	32154285	G	A	0.217	-0.252	0.108	0.020	1.60E-04
IBS	AGER	6	32150423	rs693906	31835164	C	G	0.113	-0.168	0.052	0.001	0.045
UC	AGER	6	32148745	rs204995	32154285	G	A	0.217	-0.870	0.185	2.59E-06	0.002
VHD	AGER	6	32150423	rs693906	31835164	C	G	0.113	0.212	0.052	4.82E-05	0.092

Supplementary table 6. Results of the summary-based Mendelian randomization integrating whole-blood expression quantitative trait loci. For each disease-gene association, we report the strongest effect observed between GTEX and eQTLGen datasets.

A) Bonferroni-significant disease-gene association with HEIDI P > 0.05.

Disease	Gene	chr	gene_bp	topSNP	topSNP_bp	A1	A2	Freq	b_SMR	se_SMR	p_SMR	p_HEIDI
CD	DNMT3A	2	25510652	rs7578575	25488819	A	T	0.329	-0.834	0.164	3.51E-07	0.383
IBD	DNMT3A	2	25510652	rs7578575	25488819	A	T	0.329	-0.745	0.130	9.22E-09	0.515
UC	DNMT3A	2	25510652	rs7578575	25488819	A	T	0.329	-0.627	0.156	5.73E-05	0.541
UC	CFDP1	16	75327596	rs9934007	75390532	C	T	0.754	0.273	0.070	9.61E-05	0.973
CD	STAT3	17	40502964	rs1053004	40466092	G	A	0.380	0.702	0.128	4.40E-08	0.056
CD	IMPDH2	3	49064299	rs9813813	49207093	A	T	0.110	-1.233	0.294	2.74E-05	0.213
IBD	IMPDH2	3	49064299	rs9813813	49207093	A	T	0.110	-1.328	0.258	2.57E-07	0.071
UC	IMPDH2	3	49064299	rs9813813	49207093	A	T	0.110	-1.445	0.307	2.53E-06	0.050
IBD	UBE2L3	22	21941029	rs2070512	21949411	C	A	0.194	0.163	0.021	1.06E-14	0.169
UC	UBE2L3	22	21941029	rs2070512	21949411	C	A	0.194	0.116	0.027	1.76E-05	0.698
CD	BCL2L15	1	1.14E+08	rs1217393	1.14E+08	A	G	0.271	-0.705	0.170	3.39E-05	0.184
CD	STMN3	20	62271061	rs909333	62270637	G	T	0.207	0.547	0.102	8.31E-08	0.200
IBD	STMN3	20	62271061	rs909333	62270637	G	T	0.207	0.555	0.087	2.01E-10	0.055
UC	STMN3	20	62271061	rs909333	62270637	G	T	0.207	0.498	0.098	4.04E-07	0.062
GORD	PBX2	6	32154964	rs693906	31835164	C	G	0.113	0.154	0.036	1.65E-05	0.058
VHD	AGER	6	32150423	rs693906	31835164	C	G	0.113	0.212	0.052	4.82E-05	0.092

B) Nominally significant effect on VHD of Bonferroni-significant gastrointestinal loci.

Disease	Gene	chr	Probe_bp	topSNP	topSNP_bp	A1	A2	Freq	b_SMR	se_SMR	p_SMR	p_HEIDI
VHD	CFDP1	16	75397489	rs7201989	75471499	G	A	0.246	-0.056	0.018	0.001	0.119
VHD	STAT3	17	40502964	rs1053004	40466092	G	A	0.380	0.093	0.047	0.048	0.948
VHD	IMPDH2	3	49064299	rs34037363	48821036	T	C	0.114	-0.209	0.105	0.047	0.089
VHD	UBE2L3	22	21941029	rs2070512	21949411	C	A	0.194	-0.029	0.009	0.002	0.623
VHD	STMN3	20	62271061	rs909333	62270637	G	T	0.207	-0.076	0.036	0.036	0.093

C) Effect of AGER gene on VHD and gastrointestinal disorders.

Disease	Gene	chr	Probe_bp	topSNP	topSNP_bp	A1	A2	Freq	b_SMR	se_SMR	p_SMR	p_HEIDI
CD	AGER	6	32150423	rs693906	31835164	C	G	0.113	-0.312	0.120	0.009	6.16E-08
GORD	AGER	6	32150423	rs693906	31835164	C	G	0.113	-0.209	0.049	2.02E-05	0.022
IBD	AGER	6	32148745	rs204995	32154285	G	A	0.217	-0.252	0.108	0.020	1.60E-04
IBS	AGER	6	32150423	rs693906	31835164	C	G	0.113	-0.168	0.052	0.001	0.045
UC	AGER	6	32148745	rs204995	32154285	G	A	0.217	-0.870	0.185	2.59E-06	0.002
VHD	AGER	6	32150423	rs693906	31835164	C	G	0.113	0.212	0.052	4.82E-05	0.092

Supplementary table 6. Results of the summary-based Mendelian randomization integrating whole-blood expression quantitative trait loci. For each disease-gene association, we report the strongest effect observed between GTEX and eQTLGen datasets.

A) Bonferroni-significant disease-gene association with HEIDI P > 0.05.

Disease	Gene	chr	gene_bp	topSNP	topSNP_bp	A1	A2	Freq	b_SMR	se_SMR	p_SMR	p_HEIDI
CD	DNMT3A	2	25510652	rs7578575	25488819	A	T	0.329	-0.834	0.164	3.51E-07	0.383
IBD	DNMT3A	2	25510652	rs7578575	25488819	A	T	0.329	-0.745	0.130	9.22E-09	0.515
UC	DNMT3A	2	25510652	rs7578575	25488819	A	T	0.329	-0.627	0.156	5.73E-05	0.541
UC	CFDP1	16	75327596	rs9934007	75390532	C	T	0.754	0.273	0.070	9.61E-05	0.973
CD	STAT3	17	40502964	rs1053004	40466092	G	A	0.380	0.702	0.128	4.40E-08	0.056
CD	IMPDH2	3	49064299	rs9813813	49207093	A	T	0.110	-1.233	0.294	2.74E-05	0.213
IBD	IMPDH2	3	49064299	rs9813813	49207093	A	T	0.110	-1.328	0.258	2.57E-07	0.071
UC	IMPDH2	3	49064299	rs9813813	49207093	A	T	0.110	-1.445	0.307	2.53E-06	0.050
IBD	UBE2L3	22	21941029	rs2070512	21949411	C	A	0.194	0.163	0.021	1.06E-14	0.169
UC	UBE2L3	22	21941029	rs2070512	21949411	C	A	0.194	0.116	0.027	1.76E-05	0.698
CD	BCL2L15	1	1.14E+08	rs1217393	1.14E+08	A	G	0.271	-0.705	0.170	3.39E-05	0.184
CD	STMN3	20	62271061	rs909333	62270637	G	T	0.207	0.547	0.102	8.31E-08	0.200
IBD	STMN3	20	62271061	rs909333	62270637	G	T	0.207	0.555	0.087	2.01E-10	0.055
UC	STMN3	20	62271061	rs909333	62270637	G	T	0.207	0.498	0.098	4.04E-07	0.062
GORD	PBX2	6	32154964	rs693906	31835164	C	G	0.113	0.154	0.036	1.65E-05	0.058
VHD	AGER	6	32150423	rs693906	31835164	C	G	0.113	0.212	0.052	4.82E-05	0.092

B) Nominally significant effect on VHD of Bonferroni-significant gastrointestinal loci.

Disease	Gene	chr	Probe_bp	topSNP	topSNP_bp	A1	A2	Freq	b_SMR	se_SMR	p_SMR	p_HEIDI
VHD	CFDP1	16	75397489	rs7201989	75471499	G	A	0.246	-0.056	0.018	0.001	0.119
VHD	STAT3	17	40502964	rs1053004	40466092	G	A	0.380	0.093	0.047	0.048	0.948
VHD	IMPDH2	3	49064299	rs34037363	48821036	T	C	0.114	-0.209	0.105	0.047	0.089
VHD	UBE2L3	22	21941029	rs2070512	21949411	C	A	0.194	-0.029	0.009	0.002	0.623
VHD	STMN3	20	62271061	rs909333	62270637	G	T	0.207	-0.076	0.036	0.036	0.093

C) Effect of AGER gene on VHD and gastrointestinal disorders.

Disease	Gene	chr	Probe_bp	topSNP	topSNP_bp	A1	A2	Freq	b_SMR	se_SMR	p_SMR	p_HEIDI
CD	AGER	6	32150423	rs693906	31835164	C	G	0.113	-0.312	0.120	0.009	6.16E-08
GORD	AGER	6	32150423	rs693906	31835164	C	G	0.113	-0.209	0.049	2.02E-05	0.022
IBD	AGER	6	32148745	rs204995	32154285	G	A	0.217	-0.252	0.108	0.020	1.60E-04
IBS	AGER	6	32150423	rs693906	31835164	C	G	0.113	-0.168	0.052	0.001	0.045
UC	AGER	6	32148745	rs204995	32154285	G	A	0.217	-0.870	0.185	2.59E-06	0.002
VHD	AGER	6	32150423	rs693906	31835164	C	G	0.113	0.212	0.052	4.82E-05	0.092

Supplementary table 6. Results of the summary-based Mendelian randomization integrating whole-blood expression quantitative trait loci. For each disease-gene association, we report the strongest effect observed between GTEX and eQTLGen datasets.

A) Bonferroni-significant disease-gene association with HEIDI P > 0.05.

Disease	Gene	chr	gene_bp	topSNP	topSNP_bp	A1	A2	Freq	b_SMR	se_SMR	p_SMR	p_HEIDI
CD	DNMT3A	2	25510652	rs7578575	25488819	A	T	0.329	-0.834	0.164	3.51E-07	0.383
IBD	DNMT3A	2	25510652	rs7578575	25488819	A	T	0.329	-0.745	0.130	9.22E-09	0.515
UC	DNMT3A	2	25510652	rs7578575	25488819	A	T	0.329	-0.627	0.156	5.73E-05	0.541
UC	CFDP1	16	75327596	rs9934007	75390532	C	T	0.754	0.273	0.070	9.61E-05	0.973
CD	STAT3	17	40502964	rs1053004	40466092	G	A	0.380	0.702	0.128	4.40E-08	0.056
CD	IMPDH2	3	49064299	rs9813813	49207093	A	T	0.110	-1.233	0.294	2.74E-05	0.213
IBD	IMPDH2	3	49064299	rs9813813	49207093	A	T	0.110	-1.328	0.258	2.57E-07	0.071
UC	IMPDH2	3	49064299	rs9813813	49207093	A	T	0.110	-1.445	0.307	2.53E-06	0.050
IBD	UBE2L3	22	21941029	rs2070512	21949411	C	A	0.194	0.163	0.021	1.06E-14	0.169
UC	UBE2L3	22	21941029	rs2070512	21949411	C	A	0.194	0.116	0.027	1.76E-05	0.698
CD	BCL2L15	1	1.14E+08	rs1217393	1.14E+08	A	G	0.271	-0.705	0.170	3.39E-05	0.184
CD	STMN3	20	62271061	rs909333	62270637	G	T	0.207	0.547	0.102	8.31E-08	0.200
IBD	STMN3	20	62271061	rs909333	62270637	G	T	0.207	0.555	0.087	2.01E-10	0.055
UC	STMN3	20	62271061	rs909333	62270637	G	T	0.207	0.498	0.098	4.04E-07	0.062
GORD	PBX2	6	32154964	rs693906	31835164	C	G	0.113	0.154	0.036	1.65E-05	0.058
VHD	AGER	6	32150423	rs693906	31835164	C	G	0.113	0.212	0.052	4.82E-05	0.092

B) Nominally significant effect on VHD of Bonferroni-significant gastrointestinal loci.

Disease	Gene	chr	Probe_bp	topSNP	topSNP_bp	A1	A2	Freq	b_SMR	se_SMR	p_SMR	p_HEIDI
VHD	CFDP1	16	75397489	rs7201989	75471499	G	A	0.246	-0.056	0.018	0.001	0.119
VHD	STAT3	17	40502964	rs1053004	40466092	G	A	0.380	0.093	0.047	0.048	0.948
VHD	IMPDH2	3	49064299	rs34037363	48821036	T	C	0.114	-0.209	0.105	0.047	0.089
VHD	UBE2L3	22	21941029	rs2070512	21949411	C	A	0.194	-0.029	0.009	0.002	0.623
VHD	STMN3	20	62271061	rs909333	62270637	G	T	0.207	-0.076	0.036	0.036	0.093

C) Effect of AGER gene on VHD and gastrointestinal disorders.

Disease	Gene	chr	Probe_bp	topSNP	topSNP_bp	A1	A2	Freq	b_SMR	se_SMR	p_SMR	p_HEIDI
CD	AGER	6	32150423	rs693906	31835164	C	G	0.113	-0.312	0.120	0.009	6.16E-08
GORD	AGER	6	32150423	rs693906	31835164	C	G	0.113	-0.209	0.049	2.02E-05	0.022
IBD	AGER	6	32148745	rs204995	32154285	G	A	0.217	-0.252	0.108	0.020	1.60E-04
IBS	AGER	6	32150423	rs693906	31835164	C	G	0.113	-0.168	0.052	0.001	0.045
UC	AGER	6	32148745	rs204995	32154285	G	A	0.217	-0.870	0.185	2.59E-06	0.002
VHD	AGER	6	32150423	rs693906	31835164	C	G	0.113	0.212	0.052	4.82E-05	0.092

Supplementary table 6. Results of the summary-based Mendelian randomization integrating whole-blood expression quantitative trait loci. For each disease-gene association, we report the strongest effect observed between GTEX and eQTLGen datasets.

A) Bonferroni-significant disease-gene association with HEIDI P > 0.05.

Disease	Gene	chr	gene_bp	topSNP	topSNP_bp	A1	A2	Freq	b_SMR	se_SMR	p_SMR	p_HEIDI
CD	DNMT3A	2	25510652	rs7578575	25488819	A	T	0.329	-0.834	0.164	3.51E-07	0.383
IBD	DNMT3A	2	25510652	rs7578575	25488819	A	T	0.329	-0.745	0.130	9.22E-09	0.515
UC	DNMT3A	2	25510652	rs7578575	25488819	A	T	0.329	-0.627	0.156	5.73E-05	0.541
UC	CFDP1	16	75327596	rs9934007	75390532	C	T	0.754	0.273	0.070	9.61E-05	0.973
CD	STAT3	17	40502964	rs1053004	40466092	G	A	0.380	0.702	0.128	4.40E-08	0.056
CD	IMPDH2	3	49064299	rs9813813	49207093	A	T	0.110	-1.233	0.294	2.74E-05	0.213
IBD	IMPDH2	3	49064299	rs9813813	49207093	A	T	0.110	-1.328	0.258	2.57E-07	0.071
UC	IMPDH2	3	49064299	rs9813813	49207093	A	T	0.110	-1.445	0.307	2.53E-06	0.050
IBD	UBE2L3	22	21941029	rs2070512	21949411	C	A	0.194	0.163	0.021	1.06E-14	0.169
UC	UBE2L3	22	21941029	rs2070512	21949411	C	A	0.194	0.116	0.027	1.76E-05	0.698
CD	BCL2L15	1	1.14E+08	rs1217393	1.14E+08	A	G	0.271	-0.705	0.170	3.39E-05	0.184
CD	STMN3	20	62271061	rs909333	62270637	G	T	0.207	0.547	0.102	8.31E-08	0.200
IBD	STMN3	20	62271061	rs909333	62270637	G	T	0.207	0.555	0.087	2.01E-10	0.055
UC	STMN3	20	62271061	rs909333	62270637	G	T	0.207	0.498	0.098	4.04E-07	0.062
GORD	PBX2	6	32154964	rs693906	31835164	C	G	0.113	0.154	0.036	1.65E-05	0.058
VHD	AGER	6	32150423	rs693906	31835164	C	G	0.113	0.212	0.052	4.82E-05	0.092

B) Nominally significant effect on VHD of Bonferroni-significant gastrointestinal loci.

Disease	Gene	chr	Probe_bp	topSNP	topSNP_bp	A1	A2	Freq	b_SMR	se_SMR	p_SMR	p_HEIDI
VHD	CFDP1	16	75397489	rs7201989	75471499	G	A	0.246	-0.056	0.018	0.001	0.119
VHD	STAT3	17	40502964	rs1053004	40466092	G	A	0.380	0.093	0.047	0.048	0.948
VHD	IMPDH2	3	49064299	rs34037363	48821036	T	C	0.114	-0.209	0.105	0.047	0.089
VHD	UBE2L3	22	21941029	rs2070512	21949411	C	A	0.194	-0.029	0.009	0.002	0.623
VHD	STMN3	20	62271061	rs909333	62270637	G	T	0.207	-0.076	0.036	0.036	0.093

C) Effect of AGER gene on VHD and gastrointestinal disorders.

Disease	Gene	chr	Probe_bp	topSNP	topSNP_bp	A1	A2	Freq	b_SMR	se_SMR	p_SMR	p_HEIDI
CD	AGER	6	32150423	rs693906	31835164	C	G	0.113	-0.312	0.120	0.009	6.16E-08
GORD	AGER	6	32150423	rs693906	31835164	C	G	0.113	-0.209	0.049	2.02E-05	0.022
IBD	AGER	6	32148745	rs204995	32154285	G	A	0.217	-0.252	0.108	0.020	1.60E-04
IBS	AGER	6	32150423	rs693906	31835164	C	G	0.113	-0.168	0.052	0.001	0.045
UC	AGER	6	32148745	rs204995	32154285	G	A	0.217	-0.870	0.185	2.59E-06	0.002
VHD	AGER	6	32150423	rs693906	31835164	C	G	0.113	0.212	0.052	4.82E-05	0.092

Supplementary table 6. Results of the summary-based Mendelian randomization integrating whole-blood expression quantitative trait loci. For each disease-gene association, we report the strongest effect observed between GTEX and eQTLGen datasets.

A) Bonferroni-significant disease-gene association with HEIDI P > 0.05.

Disease	Gene	chr	gene_bp	topSNP	topSNP_bp	A1	A2	Freq	b_SMR	se_SMR	p_SMR	p_HEIDI
CD	DNMT3A	2	25510652	rs7578575	25488819	A	T	0.329	-0.834	0.164	3.51E-07	0.383
IBD	DNMT3A	2	25510652	rs7578575	25488819	A	T	0.329	-0.745	0.130	9.22E-09	0.515
UC	DNMT3A	2	25510652	rs7578575	25488819	A	T	0.329	-0.627	0.156	5.73E-05	0.541
UC	CFDP1	16	75327596	rs9934007	75390532	C	T	0.754	0.273	0.070	9.61E-05	0.973
CD	STAT3	17	40502964	rs1053004	40466092	G	A	0.380	0.702	0.128	4.40E-08	0.056
CD	IMPDH2	3	49064299	rs9813813	49207093	A	T	0.110	-1.233	0.294	2.74E-05	0.213
IBD	IMPDH2	3	49064299	rs9813813	49207093	A	T	0.110	-1.328	0.258	2.57E-07	0.071
UC	IMPDH2	3	49064299	rs9813813	49207093	A	T	0.110	-1.445	0.307	2.53E-06	0.050
IBD	UBE2L3	22	21941029	rs2070512	21949411	C	A	0.194	0.163	0.021	1.06E-14	0.169
UC	UBE2L3	22	21941029	rs2070512	21949411	C	A	0.194	0.116	0.027	1.76E-05	0.698
CD	BCL2L15	1	1.14E+08	rs1217393	1.14E+08	A	G	0.271	-0.705	0.170	3.39E-05	0.184
CD	STMN3	20	62271061	rs909333	62270637	G	T	0.207	0.547	0.102	8.31E-08	0.200
IBD	STMN3	20	62271061	rs909333	62270637	G	T	0.207	0.555	0.087	2.01E-10	0.055
UC	STMN3	20	62271061	rs909333	62270637	G	T	0.207	0.498	0.098	4.04E-07	0.062
GORD	PBX2	6	32154964	rs693906	31835164	C	G	0.113	0.154	0.036	1.65E-05	0.058
VHD	AGER	6	32150423	rs693906	31835164	C	G	0.113	0.212	0.052	4.82E-05	0.092

B) Nominally significant effect on VHD of Bonferroni-significant gastrointestinal loci.

Disease	Gene	chr	Probe_bp	topSNP	topSNP_bp	A1	A2	Freq	b_SMR	se_SMR	p_SMR	p_HEIDI
VHD	CFDP1	16	75397489	rs7201989	75471499	G	A	0.246	-0.056	0.018	0.001	0.119
VHD	STAT3	17	40502964	rs1053004	40466092	G	A	0.380	0.093	0.047	0.048	0.948
VHD	IMPDH2	3	49064299	rs34037363	48821036	T	C	0.114	-0.209	0.105	0.047	0.089
VHD	UBE2L3	22	21941029	rs2070512	21949411	C	A	0.194	-0.029	0.009	0.002	0.623
VHD	STMN3	20	62271061	rs909333	62270637	G	T	0.207	-0.076	0.036	0.036	0.093

C) Effect of AGER gene on VHD and gastrointestinal disorders.

Disease	Gene	chr	Probe_bp	topSNP	topSNP_bp	A1	A2	Freq	b_SMR	se_SMR	p_SMR	p_HEIDI
CD	AGER	6	32150423	rs693906	31835164	C	G	0.113	-0.312	0.120	0.009	6.16E-08
GORD	AGER	6	32150423	rs693906	31835164	C	G	0.113	-0.209	0.049	2.02E-05	0.022
IBD	AGER	6	32148745	rs204995	32154285	G	A	0.217	-0.252	0.108	0.020	1.60E-04
IBS	AGER	6	32150423	rs693906	31835164	C	G	0.113	-0.168	0.052	0.001	0.045
UC	AGER	6	32148745	rs204995	32154285	G	A	0.217	-0.870	0.185	2.59E-06	0.002
VHD	AGER	6	32150423	rs693906	31835164	C	G	0.113	0.212	0.052	4.82E-05	0.092

Supplementary table 6. Results of the summary-based Mendelian randomization integrating whole-blood expression quantitative trait loci. For each disease-gene association, we report the strongest effect observed between GTEX and eQTLGen datasets.

A) Bonferroni-significant disease-gene association with HEIDI P > 0.05.

Disease	Gene	chr	gene_bp	topSNP	topSNP_bp	A1	A2	Freq	b_SMR	se_SMR	p_SMR	p_HEIDI
CD	DNMT3A	2	25510652	rs7578575	25488819	A	T	0.329	-0.834	0.164	3.51E-07	0.383
IBD	DNMT3A	2	25510652	rs7578575	25488819	A	T	0.329	-0.745	0.130	9.22E-09	0.515
UC	DNMT3A	2	25510652	rs7578575	25488819	A	T	0.329	-0.627	0.156	5.73E-05	0.541
UC	CFDP1	16	75327596	rs9934007	75390532	C	T	0.754	0.273	0.070	9.61E-05	0.973
CD	STAT3	17	40502964	rs1053004	40466092	G	A	0.380	0.702	0.128	4.40E-08	0.056
CD	IMPDH2	3	49064299	rs9813813	49207093	A	T	0.110	-1.233	0.294	2.74E-05	0.213
IBD	IMPDH2	3	49064299	rs9813813	49207093	A	T	0.110	-1.328	0.258	2.57E-07	0.071
UC	IMPDH2	3	49064299	rs9813813	49207093	A	T	0.110	-1.445	0.307	2.53E-06	0.050
IBD	UBE2L3	22	21941029	rs2070512	21949411	C	A	0.194	0.163	0.021	1.06E-14	0.169
UC	UBE2L3	22	21941029	rs2070512	21949411	C	A	0.194	0.116	0.027	1.76E-05	0.698
CD	BCL2L15	1	1.14E+08	rs1217393	1.14E+08	A	G	0.271	-0.705	0.170	3.39E-05	0.184
CD	STMN3	20	62271061	rs909333	62270637	G	T	0.207	0.547	0.102	8.31E-08	0.200
IBD	STMN3	20	62271061	rs909333	62270637	G	T	0.207	0.555	0.087	2.01E-10	0.055
UC	STMN3	20	62271061	rs909333	62270637	G	T	0.207	0.498	0.098	4.04E-07	0.062
GORD	PBX2	6	32154964	rs693906	31835164	C	G	0.113	0.154	0.036	1.65E-05	0.058
VHD	AGER	6	32150423	rs693906	31835164	C	G	0.113	0.212	0.052	4.82E-05	0.092

B) Nominally significant effect on VHD of Bonferroni-significant gastrointestinal loci.

Disease	Gene	chr	Probe_bp	topSNP	topSNP_bp	A1	A2	Freq	b_SMR	se_SMR	p_SMR	p_HEIDI
VHD	CFDP1	16	75397489	rs7201989	75471499	G	A	0.246	-0.056	0.018	0.001	0.119
VHD	STAT3	17	40502964	rs1053004	40466092	G	A	0.380	0.093	0.047	0.048	0.948
VHD	IMPDH2	3	49064299	rs34037363	48821036	T	C	0.114	-0.209	0.105	0.047	0.089
VHD	UBE2L3	22	21941029	rs2070512	21949411	C	A	0.194	-0.029	0.009	0.002	0.623
VHD	STMN3	20	62271061	rs909333	62270637	G	T	0.207	-0.076	0.036	0.036	0.093

C) Effect of AGER gene on VHD and gastrointestinal disorders.

Disease	Gene	chr	Probe_bp	topSNP	topSNP_bp	A1	A2	Freq	b_SMR	se_SMR	p_SMR	p_HEIDI
CD	AGER	6	32150423	rs693906	31835164	C	G	0.113	-0.312	0.120	0.009	6.16E-08
GORD	AGER	6	32150423	rs693906	31835164	C	G	0.113	-0.209	0.049	2.02E-05	0.022
IBD	AGER	6	32148745	rs204995	32154285	G	A	0.217	-0.252	0.108	0.020	1.60E-04
IBS	AGER	6	32150423	rs693906	31835164	C	G	0.113	-0.168	0.052	0.001	0.045
UC	AGER	6	32148745	rs204995	32154285	G	A	0.217	-0.870	0.185	2.59E-06	0.002
VHD	AGER	6	32150423	rs693906	31835164	C	G	0.113	0.212	0.052	4.82E-05	0.092

Supplementary table 6. Results of the summary-based Mendelian randomization integrating whole-blood expression quantitative trait loci. For each disease-gene association, we report the strongest effect observed between GTEX and eQTLGen datasets.

A) Bonferroni-significant disease-gene association with HEIDI P > 0.05.

Disease	Gene	chr	gene_bp	topSNP	topSNP_bp	A1	A2	Freq	b_SMR	se_SMR	p_SMR	p_HEIDI
CD	DNMT3A	2	25510652	rs7578575	25488819	A	T	0.329	-0.834	0.164	3.51E-07	0.383
IBD	DNMT3A	2	25510652	rs7578575	25488819	A	T	0.329	-0.745	0.130	9.22E-09	0.515
UC	DNMT3A	2	25510652	rs7578575	25488819	A	T	0.329	-0.627	0.156	5.73E-05	0.541
UC	CFDP1	16	75327596	rs9934007	75390532	C	T	0.754	0.273	0.070	9.61E-05	0.973
CD	STAT3	17	40502964	rs1053004	40466092	G	A	0.380	0.702	0.128	4.40E-08	0.056
CD	IMPDH2	3	49064299	rs9813813	49207093	A	T	0.110	-1.233	0.294	2.74E-05	0.213
IBD	IMPDH2	3	49064299	rs9813813	49207093	A	T	0.110	-1.328	0.258	2.57E-07	0.071
UC	IMPDH2	3	49064299	rs9813813	49207093	A	T	0.110	-1.445	0.307	2.53E-06	0.050
IBD	UBE2L3	22	21941029	rs2070512	21949411	C	A	0.194	0.163	0.021	1.06E-14	0.169
UC	UBE2L3	22	21941029	rs2070512	21949411	C	A	0.194	0.116	0.027	1.76E-05	0.698
CD	BCL2L15	1	1.14E+08	rs1217393	1.14E+08	A	G	0.271	-0.705	0.170	3.39E-05	0.184
CD	STMN3	20	62271061	rs909333	62270637	G	T	0.207	0.547	0.102	8.31E-08	0.200
IBD	STMN3	20	62271061	rs909333	62270637	G	T	0.207	0.555	0.087	2.01E-10	0.055
UC	STMN3	20	62271061	rs909333	62270637	G	T	0.207	0.498	0.098	4.04E-07	0.062
GORD	PBX2	6	32154964	rs693906	31835164	C	G	0.113	0.154	0.036	1.65E-05	0.058
VHD	AGER	6	32150423	rs693906	31835164	C	G	0.113	0.212	0.052	4.82E-05	0.092

B) Nominally significant effect on VHD of Bonferroni-significant gastrointestinal loci.

Disease	Gene	chr	Probe_bp	topSNP	topSNP_bp	A1	A2	Freq	b_SMR	se_SMR	p_SMR	p_HEIDI
VHD	CFDP1	16	75397489	rs7201989	75471499	G	A	0.246	-0.056	0.018	0.001	0.119
VHD	STAT3	17	40502964	rs1053004	40466092	G	A	0.380	0.093	0.047	0.048	0.948
VHD	IMPDH2	3	49064299	rs34037363	48821036	T	C	0.114	-0.209	0.105	0.047	0.089
VHD	UBE2L3	22	21941029	rs2070512	21949411	C	A	0.194	-0.029	0.009	0.002	0.623
VHD	STMN3	20	62271061	rs909333	62270637	G	T	0.207	-0.076	0.036	0.036	0.093

C) Effect of AGER gene on VHD and gastrointestinal disorders.

Disease	Gene	chr	Probe_bp	topSNP	topSNP_bp	A1	A2	Freq	b_SMR	se_SMR	p_SMR	p_HEIDI
CD	AGER	6	32150423	rs693906	31835164	C	G	0.113	-0.312	0.120	0.009	6.16E-08
GORD	AGER	6	32150423	rs693906	31835164	C	G	0.113	-0.209	0.049	2.02E-05	0.022
IBD	AGER	6	32148745	rs204995	32154285	G	A	0.217	-0.252	0.108	0.020	1.60E-04
IBS	AGER	6	32150423	rs693906	31835164	C	G	0.113	-0.168	0.052	0.001	0.045
UC	AGER	6	32148745	rs204995	32154285	G	A	0.217	-0.870	0.185	2.59E-06	0.002
VHD	AGER	6	32150423	rs693906	31835164	C	G	0.113	0.212	0.052	4.82E-05	0.092

Supplementary table 6. Results of the summary-based Mendelian randomization integrating whole-blood expression quantitative trait loci. For each disease-gene association, we report the strongest effect observed between GTEX and eQTLGen datasets.

A) Bonferroni-significant disease-gene association with HEIDI P > 0.05.

Disease	Gene	chr	gene_bp	topSNP	topSNP_bp	A1	A2	Freq	b_SMR	se_SMR	p_SMR	p_HEIDI
CD	DNMT3A	2	25510652	rs7578575	25488819	A	T	0.329	-0.834	0.164	3.51E-07	0.383
IBD	DNMT3A	2	25510652	rs7578575	25488819	A	T	0.329	-0.745	0.130	9.22E-09	0.515
UC	DNMT3A	2	25510652	rs7578575	25488819	A	T	0.329	-0.627	0.156	5.73E-05	0.541
UC	CFDP1	16	75327596	rs9934007	75390532	C	T	0.754	0.273	0.070	9.61E-05	0.973
CD	STAT3	17	40502964	rs1053004	40466092	G	A	0.380	0.702	0.128	4.40E-08	0.056
CD	IMPDH2	3	49064299	rs9813813	49207093	A	T	0.110	-1.233	0.294	2.74E-05	0.213
IBD	IMPDH2	3	49064299	rs9813813	49207093	A	T	0.110	-1.328	0.258	2.57E-07	0.071
UC	IMPDH2	3	49064299	rs9813813	49207093	A	T	0.110	-1.445	0.307	2.53E-06	0.050
IBD	UBE2L3	22	21941029	rs2070512	21949411	C	A	0.194	0.163	0.021	1.06E-14	0.169
UC	UBE2L3	22	21941029	rs2070512	21949411	C	A	0.194	0.116	0.027	1.76E-05	0.698
CD	BCL2L15	1	1.14E+08	rs1217393	1.14E+08	A	G	0.271	-0.705	0.170	3.39E-05	0.184
CD	STMN3	20	62271061	rs909333	62270637	G	T	0.207	0.547	0.102	8.31E-08	0.200
IBD	STMN3	20	62271061	rs909333	62270637	G	T	0.207	0.555	0.087	2.01E-10	0.055
UC	STMN3	20	62271061	rs909333	62270637	G	T	0.207	0.498	0.098	4.04E-07	0.062
GORD	PBX2	6	32154964	rs693906	31835164	C	G	0.113	0.154	0.036	1.65E-05	0.058
VHD	AGER	6	32150423	rs693906	31835164	C	G	0.113	0.212	0.052	4.82E-05	0.092

B) Nominally significant effect on VHD of Bonferroni-significant gastrointestinal loci.

Disease	Gene	chr	Probe_bp	topSNP	topSNP_bp	A1	A2	Freq	b_SMR	se_SMR	p_SMR	p_HEIDI
VHD	CFDP1	16	75397489	rs7201989	75471499	G	A	0.246	-0.056	0.018	0.001	0.119
VHD	STAT3	17	40502964	rs1053004	40466092	G	A	0.380	0.093	0.047	0.048	0.948
VHD	IMPDH2	3	49064299	rs34037363	48821036	T	C	0.114	-0.209	0.105	0.047	0.089
VHD	UBE2L3	22	21941029	rs2070512	21949411	C	A	0.194	-0.029	0.009	0.002	0.623
VHD	STMN3	20	62271061	rs909333	62270637	G	T	0.207	-0.076	0.036	0.036	0.093

C) Effect of AGER gene on VHD and gastrointestinal disorders.

Disease	Gene	chr	Probe_bp	topSNP	topSNP_bp	A1	A2	Freq	b_SMR	se_SMR	p_SMR	p_HEIDI
CD	AGER	6	32150423	rs693906	31835164	C	G	0.113	-0.312	0.120	0.009	6.16E-08
GORD	AGER	6	32150423	rs693906	31835164	C	G	0.113	-0.209	0.049	2.02E-05	0.022
IBD	AGER	6	32148745	rs204995	32154285	G	A	0.217	-0.252	0.108	0.020	1.60E-04
IBS	AGER	6	32150423	rs693906	31835164	C	G	0.113	-0.168	0.052	0.001	0.045
UC	AGER	6	32148745	rs204995	32154285	G	A	0.217	-0.870	0.185	2.59E-06	0.002
VHD	AGER	6	32150423	rs693906	31835164	C	G	0.113	0.212	0.052	4.82E-05	0.092

Supplementary table 6. Results of the summary-based Mendelian randomization integrating whole-blood expression quantitative trait loci. For each disease-gene association, we report the strongest effect observed between GTEX and eQTLGen datasets.

A) Bonferroni-significant disease-gene association with HEIDI P > 0.05.

Disease	Gene	chr	gene_bp	topSNP	topSNP_bp	A1	A2	Freq	b_SMR	se_SMR	p_SMR	p_HEIDI
CD	DNMT3A	2	25510652	rs7578575	25488819	A	T	0.329	-0.834	0.164	3.51E-07	0.383
IBD	DNMT3A	2	25510652	rs7578575	25488819	A	T	0.329	-0.745	0.130	9.22E-09	0.515
UC	DNMT3A	2	25510652	rs7578575	25488819	A	T	0.329	-0.627	0.156	5.73E-05	0.541
UC	CFDP1	16	75327596	rs9934007	75390532	C	T	0.754	0.273	0.070	9.61E-05	0.973
CD	STAT3	17	40502964	rs1053004	40466092	G	A	0.380	0.702	0.128	4.40E-08	0.056
CD	IMPDH2	3	49064299	rs9813813	49207093	A	T	0.110	-1.233	0.294	2.74E-05	0.213
IBD	IMPDH2	3	49064299	rs9813813	49207093	A	T	0.110	-1.328	0.258	2.57E-07	0.071
UC	IMPDH2	3	49064299	rs9813813	49207093	A	T	0.110	-1.445	0.307	2.53E-06	0.050
IBD	UBE2L3	22	21941029	rs2070512	21949411	C	A	0.194	0.163	0.021	1.06E-14	0.169
UC	UBE2L3	22	21941029	rs2070512	21949411	C	A	0.194	0.116	0.027	1.76E-05	0.698
CD	BCL2L15	1	1.14E+08	rs1217393	1.14E+08	A	G	0.271	-0.705	0.170	3.39E-05	0.184
CD	STMN3	20	62271061	rs909333	62270637	G	T	0.207	0.547	0.102	8.31E-08	0.200
IBD	STMN3	20	62271061	rs909333	62270637	G	T	0.207	0.555	0.087	2.01E-10	0.055
UC	STMN3	20	62271061	rs909333	62270637	G	T	0.207	0.498	0.098	4.04E-07	0.062
GORD	PBX2	6	32154964	rs693906	31835164	C	G	0.113	0.154	0.036	1.65E-05	0.058
VHD	AGER	6	32150423	rs693906	31835164	C	G	0.113	0.212	0.052	4.82E-05	0.092

B) Nominally significant effect on VHD of Bonferroni-significant gastrointestinal loci.

Disease	Gene	chr	Probe_bp	topSNP	topSNP_bp	A1	A2	Freq	b_SMR	se_SMR	p_SMR	p_HEIDI
VHD	CFDP1	16	75397489	rs7201989	75471499	G	A	0.246	-0.056	0.018	0.001	0.119
VHD	STAT3	17	40502964	rs1053004	40466092	G	A	0.380	0.093	0.047	0.048	0.948
VHD	IMPDH2	3	49064299	rs34037363	48821036	T	C	0.114	-0.209	0.105	0.047	0.089
VHD	UBE2L3	22	21941029	rs2070512	21949411	C	A	0.194	-0.029	0.009	0.002	0.623
VHD	STMN3	20	62271061	rs909333	62270637	G	T	0.207	-0.076	0.036	0.036	0.093

C) Effect of AGER gene on VHD and gastrointestinal disorders.

Disease	Gene	chr	Probe_bp	topSNP	topSNP_bp	A1	A2	Freq	b_SMR	se_SMR	p_SMR	p_HEIDI
CD	AGER	6	32150423	rs693906	31835164	C	G	0.113	-0.312	0.120	0.009	6.16E-08
GORD	AGER	6	32150423	rs693906	31835164	C	G	0.113	-0.209	0.049	2.02E-05	0.022
IBD	AGER	6	32148745	rs204995	32154285	G	A	0.217	-0.252	0.108	0.020	1.60E-04
IBS	AGER	6	32150423	rs693906	31835164	C	G	0.113	-0.168	0.052	0.001	0.045
UC	AGER	6	32148745	rs204995	32154285	G	A	0.217	-0.870	0.185	2.59E-06	0.002
VHD	AGER	6	32150423	rs693906	31835164	C	G	0.113	0.212	0.052	4.82E-05	0.092

Supplementary table 6. Results of the summary-based Mendelian randomization integrating whole-blood expression quantitative trait loci. For each disease-gene association, we report the strongest effect observed between GTEX and eQTLGen datasets.

A) Bonferroni-significant disease-gene association with HEIDI P > 0.05.

Disease	Gene	chr	gene_bp	topSNP	topSNP_bp	A1	A2	Freq	b_SMR	se_SMR	p_SMR	p_HEIDI
CD	DNMT3A	2	25510652	rs7578575	25488819	A	T	0.329	-0.834	0.164	3.51E-07	0.383
IBD	DNMT3A	2	25510652	rs7578575	25488819	A	T	0.329	-0.745	0.130	9.22E-09	0.515
UC	DNMT3A	2	25510652	rs7578575	25488819	A	T	0.329	-0.627	0.156	5.73E-05	0.541
UC	CFDP1	16	75327596	rs9934007	75390532	C	T	0.754	0.273	0.070	9.61E-05	0.973
CD	STAT3	17	40502964	rs1053004	40466092	G	A	0.380	0.702	0.128	4.40E-08	0.056
CD	IMPDH2	3	49064299	rs9813813	49207093	A	T	0.110	-1.233	0.294	2.74E-05	0.213
IBD	IMPDH2	3	49064299	rs9813813	49207093	A	T	0.110	-1.328	0.258	2.57E-07	0.071
UC	IMPDH2	3	49064299	rs9813813	49207093	A	T	0.110	-1.445	0.307	2.53E-06	0.050
IBD	UBE2L3	22	21941029	rs2070512	21949411	C	A	0.194	0.163	0.021	1.06E-14	0.169
UC	UBE2L3	22	21941029	rs2070512	21949411	C	A	0.194	0.116	0.027	1.76E-05	0.698
CD	BCL2L15	1	1.14E+08	rs1217393	1.14E+08	A	G	0.271	-0.705	0.170	3.39E-05	0.184
CD	STMN3	20	62271061	rs909333	62270637	G	T	0.207	0.547	0.102	8.31E-08	0.200
IBD	STMN3	20	62271061	rs909333	62270637	G	T	0.207	0.555	0.087	2.01E-10	0.055
UC	STMN3	20	62271061	rs909333	62270637	G	T	0.207	0.498	0.098	4.04E-07	0.062
GORD	PBX2	6	32154964	rs693906	31835164	C	G	0.113	0.154	0.036	1.65E-05	0.058
VHD	AGER	6	32150423	rs693906	31835164	C	G	0.113	0.212	0.052	4.82E-05	0.092

B) Nominally significant effect on VHD of Bonferroni-significant gastrointestinal loci.

Disease	Gene	chr	Probe_bp	topSNP	topSNP_bp	A1	A2	Freq	b_SMR	se_SMR	p_SMR	p_HEIDI
VHD	CFDP1	16	75397489	rs7201989	75471499	G	A	0.246	-0.056	0.018	0.001	0.119
VHD	STAT3	17	40502964	rs1053004	40466092	G	A	0.380	0.093	0.047	0.048	0.948
VHD	IMPDH2	3	49064299	rs34037363	48821036	T	C	0.114	-0.209	0.105	0.047	0.089
VHD	UBE2L3	22	21941029	rs2070512	21949411	C	A	0.194	-0.029	0.009	0.002	0.623
VHD	STMN3	20	62271061	rs909333	62270637	G	T	0.207	-0.076	0.036	0.036	0.093

C) Effect of AGER gene on VHD and gastrointestinal disorders.

Disease	Gene	chr	Probe_bp	topSNP	topSNP_bp	A1	A2	Freq	b_SMR	se_SMR	p_SMR	p_HEIDI
CD	AGER	6	32150423	rs693906	31835164	C	G	0.113	-0.312	0.120	0.009	6.16E-08
GORD	AGER	6	32150423	rs693906	31835164	C	G	0.113	-0.209	0.049	2.02E-05	0.022
IBD	AGER	6	32148745	rs204995	32154285	G	A	0.217	-0.252	0.108	0.020	1.60E-04
IBS	AGER	6	32150423	rs693906	31835164	C	G	0.113	-0.168	0.052	0.001	0.045
UC	AGER	6	32148745	rs204995	32154285	G	A	0.217	-0.870	0.185	2.59E-06	0.002
VHD	AGER	6	32150423	rs693906	31835164	C	G	0.113	0.212	0.052	4.82E-05	0.092

Supplementary table 6. Results of the summary-based Mendelian randomization integrating whole-blood expression quantitative trait loci. For each disease-gene association, we report the strongest effect observed between GTEX and eQTLGen datasets.

A) Bonferroni-significant disease-gene association with HEIDI P > 0.05.

Disease	Gene	chr	gene_bp	topSNP	topSNP_bp	A1	A2	Freq	b_SMR	se_SMR	p_SMR	p_HEIDI
CD	DNMT3A	2	25510652	rs7578575	25488819	A	T	0.329	-0.834	0.164	3.51E-07	0.383
IBD	DNMT3A	2	25510652	rs7578575	25488819	A	T	0.329	-0.745	0.130	9.22E-09	0.515
UC	DNMT3A	2	25510652	rs7578575	25488819	A	T	0.329	-0.627	0.156	5.73E-05	0.541
UC	CFDP1	16	75327596	rs9934007	75390532	C	T	0.754	0.273	0.070	9.61E-05	0.973
CD	STAT3	17	40502964	rs1053004	40466092	G	A	0.380	0.702	0.128	4.40E-08	0.056
CD	IMPDH2	3	49064299	rs9813813	49207093	A	T	0.110	-1.233	0.294	2.74E-05	0.213
IBD	IMPDH2	3	49064299	rs9813813	49207093	A	T	0.110	-1.328	0.258	2.57E-07	0.071
UC	IMPDH2	3	49064299	rs9813813	49207093	A	T	0.110	-1.445	0.307	2.53E-06	0.050
IBD	UBE2L3	22	21941029	rs2070512	21949411	C	A	0.194	0.163	0.021	1.06E-14	0.169
UC	UBE2L3	22	21941029	rs2070512	21949411	C	A	0.194	0.116	0.027	1.76E-05	0.698
CD	BCL2L15	1	1.14E+08	rs1217393	1.14E+08	A	G	0.271	-0.705	0.170	3.39E-05	0.184
CD	STMN3	20	62271061	rs909333	62270637	G	T	0.207	0.547	0.102	8.31E-08	0.200
IBD	STMN3	20	62271061	rs909333	62270637	G	T	0.207	0.555	0.087	2.01E-10	0.055
UC	STMN3	20	62271061	rs909333	62270637	G	T	0.207	0.498	0.098	4.04E-07	0.062
GORD	PBX2	6	32154964	rs693906	31835164	C	G	0.113	0.154	0.036	1.65E-05	0.058
VHD	AGER	6	32150423	rs693906	31835164	C	G	0.113	0.212	0.052	4.82E-05	0.092

B) Nominally significant effect on VHD of Bonferroni-significant gastrointestinal loci.

Disease	Gene	chr	Probe_bp	topSNP	topSNP_bp	A1	A2	Freq	b_SMR	se_SMR	p_SMR	p_HEIDI
VHD	CFDP1	16	75397489	rs7201989	75471499	G	A	0.246	-0.056	0.018	0.001	0.119
VHD	STAT3	17	40502964	rs1053004	40466092	G	A	0.380	0.093	0.047	0.048	0.948
VHD	IMPDH2	3	49064299	rs34037363	48821036	T	C	0.114	-0.209	0.105	0.047	0.089
VHD	UBE2L3	22	21941029	rs2070512	21949411	C	A	0.194	-0.029	0.009	0.002	0.623
VHD	STMN3	20	62271061	rs909333	62270637	G	T	0.207	-0.076	0.036	0.036	0.093

C) Effect of AGER gene on VHD and gastrointestinal disorders.

Disease	Gene	chr	Probe_bp	topSNP	topSNP_bp	A1	A2	Freq	b_SMR	se_SMR	p_SMR	p_HEIDI
CD	AGER	6	32150423	rs693906	31835164	C	G	0.113	-0.312	0.120	0.009	6.16E-08
GORD	AGER	6	32150423	rs693906	31835164	C	G	0.113	-0.209	0.049	2.02E-05	0.022
IBD	AGER	6	32148745	rs204995	32154285	G	A	0.217	-0.252	0.108	0.020	1.60E-04
IBS	AGER	6	32150423	rs693906	31835164	C	G	0.113	-0.168	0.052	0.001	0.045
UC	AGER	6	32148745	rs204995	32154285	G	A	0.217	-0.870	0.185	2.59E-06	0.002
VHD	AGER	6	32150423	rs693906	31835164	C	G	0.113	0.212	0.052	4.82E-05	0.092

Supplementary table 6. Results of the summary-based Mendelian randomization integrating whole-blood expression quantitative trait loci. For each disease-gene association, we report the strongest effect observed between GTEX and eQTLGen datasets.

A) Bonferroni-significant disease-gene association with HEIDI P > 0.05.

Disease	Gene	chr	gene_bp	topSNP	topSNP_bp	A1	A2	Freq	b_SMR	se_SMR	p_SMR	p_HEIDI
CD	DNMT3A	2	25510652	rs7578575	25488819	A	T	0.329	-0.834	0.164	3.51E-07	0.383
IBD	DNMT3A	2	25510652	rs7578575	25488819	A	T	0.329	-0.745	0.130	9.22E-09	0.515
UC	DNMT3A	2	25510652	rs7578575	25488819	A	T	0.329	-0.627	0.156	5.73E-05	0.541
UC	CFDP1	16	75327596	rs9934007	75390532	C	T	0.754	0.273	0.070	9.61E-05	0.973
CD	STAT3	17	40502964	rs1053004	40466092	G	A	0.380	0.702	0.128	4.40E-08	0.056
CD	IMPDH2	3	49064299	rs9813813	49207093	A	T	0.110	-1.233	0.294	2.74E-05	0.213
IBD	IMPDH2	3	49064299	rs9813813	49207093	A	T	0.110	-1.328	0.258	2.57E-07	0.071
UC	IMPDH2	3	49064299	rs9813813	49207093	A	T	0.110	-1.445	0.307	2.53E-06	0.050
IBD	UBE2L3	22	21941029	rs2070512	21949411	C	A	0.194	0.163	0.021	1.06E-14	0.169
UC	UBE2L3	22	21941029	rs2070512	21949411	C	A	0.194	0.116	0.027	1.76E-05	0.698
CD	BCL2L15	1	1.14E+08	rs1217393	1.14E+08	A	G	0.271	-0.705	0.170	3.39E-05	0.184
CD	STMN3	20	62271061	rs909333	62270637	G	T	0.207	0.547	0.102	8.31E-08	0.200
IBD	STMN3	20	62271061	rs909333	62270637	G	T	0.207	0.555	0.087	2.01E-10	0.055
UC	STMN3	20	62271061	rs909333	62270637	G	T	0.207	0.498	0.098	4.04E-07	0.062
GORD	PBX2	6	32154964	rs693906	31835164	C	G	0.113	0.154	0.036	1.65E-05	0.058
VHD	AGER	6	32150423	rs693906	31835164	C	G	0.113	0.212	0.052	4.82E-05	0.092

B) Nominally significant effect on VHD of Bonferroni-significant gastrointestinal loci.

Disease	Gene	chr	Probe_bp	topSNP	topSNP_bp	A1	A2	Freq	b_SMR	se_SMR	p_SMR	p_HEIDI
VHD	CFDP1	16	75397489	rs7201989	75471499	G	A	0.246	-0.056	0.018	0.001	0.119
VHD	STAT3	17	40502964	rs1053004	40466092	G	A	0.380	0.093	0.047	0.048	0.948
VHD	IMPDH2	3	49064299	rs34037363	48821036	T	C	0.114	-0.209	0.105	0.047	0.089
VHD	UBE2L3	22	21941029	rs2070512	21949411	C	A	0.194	-0.029	0.009	0.002	0.623
VHD	STMN3	20	62271061	rs909333	62270637	G	T	0.207	-0.076	0.036	0.036	0.093

C) Effect of AGER gene on VHD and gastrointestinal disorders.

Disease	Gene	chr	Probe_bp	topSNP	topSNP_bp	A1	A2	Freq	b_SMR	se_SMR	p_SMR	p_HEIDI
CD	AGER	6	32150423	rs693906	31835164	C	G	0.113	-0.312	0.120	0.009	6.16E-08
GORD	AGER	6	32150423	rs693906	31835164	C	G	0.113	-0.209	0.049	2.02E-05	0.022
IBD	AGER	6	32148745	rs204995	32154285	G	A	0.217	-0.252	0.108	0.020	1.60E-04
IBS	AGER	6	32150423	rs693906	31835164	C	G	0.113	-0.168	0.052	0.001	0.045
UC	AGER	6	32148745	rs204995	32154285	G	A	0.217	-0.870	0.185	2.59E-06	0.002
VHD	AGER	6	32150423	rs693906	31835164	C	G	0.113	0.212	0.052	4.82E-05	0.092

Supplementary table 6. Results of the summary-based Mendelian randomization integrating whole-blood expression quantitative trait loci. For each disease-gene association, we report the strongest effect observed between GTEX and eQTLGen datasets.

A) Bonferroni-significant disease-gene association with HEIDI P > 0.05.

Disease	Gene	chr	gene_bp	topSNP	topSNP_bp	A1	A2	Freq	b_SMR	se_SMR	p_SMR	p_HEIDI
CD	DNMT3A	2	25510652	rs7578575	25488819	A	T	0.329	-0.834	0.164	3.51E-07	0.383
IBD	DNMT3A	2	25510652	rs7578575	25488819	A	T	0.329	-0.745	0.130	9.22E-09	0.515
UC	DNMT3A	2	25510652	rs7578575	25488819	A	T	0.329	-0.627	0.156	5.73E-05	0.541
UC	CFDP1	16	75327596	rs9934007	75390532	C	T	0.754	0.273	0.070	9.61E-05	0.973
CD	STAT3	17	40502964	rs1053004	40466092	G	A	0.380	0.702	0.128	4.40E-08	0.056
CD	IMPDH2	3	49064299	rs9813813	49207093	A	T	0.110	-1.233	0.294	2.74E-05	0.213
IBD	IMPDH2	3	49064299	rs9813813	49207093	A	T	0.110	-1.328	0.258	2.57E-07	0.071
UC	IMPDH2	3	49064299	rs9813813	49207093	A	T	0.110	-1.445	0.307	2.53E-06	0.050
IBD	UBE2L3	22	21941029	rs2070512	21949411	C	A	0.194	0.163	0.021	1.06E-14	0.169
UC	UBE2L3	22	21941029	rs2070512	21949411	C	A	0.194	0.116	0.027	1.76E-05	0.698
CD	BCL2L15	1	1.14E+08	rs1217393	1.14E+08	A	G	0.271	-0.705	0.170	3.39E-05	0.184
CD	STMN3	20	62271061	rs909333	62270637	G	T	0.207	0.547	0.102	8.31E-08	0.200
IBD	STMN3	20	62271061	rs909333	62270637	G	T	0.207	0.555	0.087	2.01E-10	0.055
UC	STMN3	20	62271061	rs909333	62270637	G	T	0.207	0.498	0.098	4.04E-07	0.062
GORD	PBX2	6	32154964	rs693906	31835164	C	G	0.113	0.154	0.036	1.65E-05	0.058
VHD	AGER	6	32150423	rs693906	31835164	C	G	0.113	0.212	0.052	4.82E-05	0.092

B) Nominally significant effect on VHD of Bonferroni-significant gastrointestinal loci.

Disease	Gene	chr	Probe_bp	topSNP	topSNP_bp	A1	A2	Freq	b_SMR	se_SMR	p_SMR	p_HEIDI
VHD	CFDP1	16	75397489	rs7201989	75471499	G	A	0.246	-0.056	0.018	0.001	0.119
VHD	STAT3	17	40502964	rs1053004	40466092	G	A	0.380	0.093	0.047	0.048	0.948
VHD	IMPDH2	3	49064299	rs34037363	48821036	T	C	0.114	-0.209	0.105	0.047	0.089
VHD	UBE2L3	22	21941029	rs2070512	21949411	C	A	0.194	-0.029	0.009	0.002	0.623
VHD	STMN3	20	62271061	rs909333	62270637	G	T	0.207	-0.076	0.036	0.036	0.093

C) Effect of AGER gene on VHD and gastrointestinal disorders.

Disease	Gene	chr	Probe_bp	topSNP	topSNP_bp	A1	A2	Freq	b_SMR	se_SMR	p_SMR	p_HEIDI
CD	AGER	6	32150423	rs693906	31835164	C	G	0.113	-0.312	0.120	0.009	6.16E-08
GORD	AGER	6	32150423	rs693906	31835164	C	G	0.113	-0.209	0.049	2.02E-05	0.022
IBD	AGER	6	32148745	rs204995	32154285	G	A	0.217	-0.252	0.108	0.020	1.60E-04
IBS	AGER	6	32150423	rs693906	31835164	C	G	0.113	-0.168	0.052	0.001	0.045
UC	AGER	6	32148745	rs204995	32154285	G	A	0.217	-0.870	0.185	2.59E-06	0.002
VHD	AGER	6	32150423	rs693906	31835164	C	G	0.113	0.212	0.052	4.82E-05	0.092

Supplementary table 6. Results of the summary-based Mendelian randomization integrating whole-blood expression quantitative trait loci. For each disease-gene association, we report the strongest effect observed between GTEX and eQTLGen datasets.

A) Bonferroni-significant disease-gene association with HEIDI P > 0.05.

Disease	Gene	chr	gene_bp	topSNP	topSNP_bp	A1	A2	Freq	b_SMR	se_SMR	p_SMR	p_HEIDI
CD	DNMT3A	2	25510652	rs7578575	25488819	A	T	0.329	-0.834	0.164	3.51E-07	0.383
IBD	DNMT3A	2	25510652	rs7578575	25488819	A	T	0.329	-0.745	0.130	9.22E-09	0.515
UC	DNMT3A	2	25510652	rs7578575	25488819	A	T	0.329	-0.627	0.156	5.73E-05	0.541
UC	CFDP1	16	75327596	rs9934007	75390532	C	T	0.754	0.273	0.070	9.61E-05	0.973
CD	STAT3	17	40502964	rs1053004	40466092	G	A	0.380	0.702	0.128	4.40E-08	0.056
CD	IMPDH2	3	49064299	rs9813813	49207093	A	T	0.110	-1.233	0.294	2.74E-05	0.213
IBD	IMPDH2	3	49064299	rs9813813	49207093	A	T	0.110	-1.328	0.258	2.57E-07	0.071
UC	IMPDH2	3	49064299	rs9813813	49207093	A	T	0.110	-1.445	0.307	2.53E-06	0.050
IBD	UBE2L3	22	21941029	rs2070512	21949411	C	A	0.194	0.163	0.021	1.06E-14	0.169
UC	UBE2L3	22	21941029	rs2070512	21949411	C	A	0.194	0.116	0.027	1.76E-05	0.698
CD	BCL2L15	1	1.14E+08	rs1217393	1.14E+08	A	G	0.271	-0.705	0.170	3.39E-05	0.184
CD	STMN3	20	62271061	rs909333	62270637	G	T	0.207	0.547	0.102	8.31E-08	0.200
IBD	STMN3	20	62271061	rs909333	62270637	G	T	0.207	0.555	0.087	2.01E-10	0.055
UC	STMN3	20	62271061	rs909333	62270637	G	T	0.207	0.498	0.098	4.04E-07	0.062
GORD	PBX2	6	32154964	rs693906	31835164	C	G	0.113	0.154	0.036	1.65E-05	0.058
VHD	AGER	6	32150423	rs693906	31835164	C	G	0.113	0.212	0.052	4.82E-05	0.092

B) Nominally significant effect on VHD of Bonferroni-significant gastrointestinal loci.

Disease	Gene	chr	Probe_bp	topSNP	topSNP_bp	A1	A2	Freq	b_SMR	se_SMR	p_SMR	p_HEIDI
VHD	CFDP1	16	75397489	rs7201989	75471499	G	A	0.246	-0.056	0.018	0.001	0.119
VHD	STAT3	17	40502964	rs1053004	40466092	G	A	0.380	0.093	0.047	0.048	0.948
VHD	IMPDH2	3	49064299	rs34037363	48821036	T	C	0.114	-0.209	0.105	0.047	0.089
VHD	UBE2L3	22	21941029	rs2070512	21949411	C	A	0.194	-0.029	0.009	0.002	0.623
VHD	STMN3	20	62271061	rs909333	62270637	G	T	0.207	-0.076	0.036	0.036	0.093

C) Effect of AGER gene on VHD and gastrointestinal disorders.

Disease	Gene	chr	Probe_bp	topSNP	topSNP_bp	A1	A2	Freq	b_SMR	se_SMR	p_SMR	p_HEIDI
CD	AGER	6	32150423	rs693906	31835164	C	G	0.113	-0.312	0.120	0.009	6.16E-08
GORD	AGER	6	32150423	rs693906	31835164	C	G	0.113	-0.209	0.049	2.02E-05	0.022
IBD	AGER	6	32148745	rs204995	32154285	G	A	0.217	-0.252	0.108	0.020	1.60E-04
IBS	AGER	6	32150423	rs693906	31835164	C	G	0.113	-0.168	0.052	0.001	0.045
UC	AGER	6	32148745	rs204995	32154285	G	A	0.217	-0.870	0.185	2.59E-06	0.002
VHD	AGER	6	32150423	rs693906	31835164	C	G	0.113	0.212	0.052	4.82E-05	0.092

Supplementary table 6. Results of the summary-based Mendelian randomization integrating whole-blood expression quantitative trait loci. For each disease-gene association, we report the strongest effect observed between GTEX and eQTLGen datasets.

A) Bonferroni-significant disease-gene association with HEIDI P > 0.05.

Disease	Gene	chr	gene_bp	topSNP	topSNP_bp	A1	A2	Freq	b_SMR	se_SMR	p_SMR	p_HEIDI
CD	DNMT3A	2	25510652	rs7578575	25488819	A	T	0.329	-0.834	0.164	3.51E-07	0.383
IBD	DNMT3A	2	25510652	rs7578575	25488819	A	T	0.329	-0.745	0.130	9.22E-09	0.515
UC	DNMT3A	2	25510652	rs7578575	25488819	A	T	0.329	-0.627	0.156	5.73E-05	0.541
UC	CFDP1	16	75327596	rs9934007	75390532	C	T	0.754	0.273	0.070	9.61E-05	0.973
CD	STAT3	17	40502964	rs1053004	40466092	G	A	0.380	0.702	0.128	4.40E-08	0.056
CD	IMPDH2	3	49064299	rs9813813	49207093	A	T	0.110	-1.233	0.294	2.74E-05	0.213
IBD	IMPDH2	3	49064299	rs9813813	49207093	A	T	0.110	-1.328	0.258	2.57E-07	0.071
UC	IMPDH2	3	49064299	rs9813813	49207093	A	T	0.110	-1.445	0.307	2.53E-06	0.050
IBD	UBE2L3	22	21941029	rs2070512	21949411	C	A	0.194	0.163	0.021	1.06E-14	0.169
UC	UBE2L3	22	21941029	rs2070512	21949411	C	A	0.194	0.116	0.027	1.76E-05	0.698
CD	BCL2L15	1	1.14E+08	rs1217393	1.14E+08	A	G	0.271	-0.705	0.170	3.39E-05	0.184
CD	STMN3	20	62271061	rs909333	62270637	G	T	0.207	0.547	0.102	8.31E-08	0.200
IBD	STMN3	20	62271061	rs909333	62270637	G	T	0.207	0.555	0.087	2.01E-10	0.055
UC	STMN3	20	62271061	rs909333	62270637	G	T	0.207	0.498	0.098	4.04E-07	0.062
GORD	PBX2	6	32154964	rs693906	31835164	C	G	0.113	0.154	0.036	1.65E-05	0.058
VHD	AGER	6	32150423	rs693906	31835164	C	G	0.113	0.212	0.052	4.82E-05	0.092

B) Nominally significant effect on VHD of Bonferroni-significant gastrointestinal loci.

Disease	Gene	chr	Probe_bp	topSNP	topSNP_bp	A1	A2	Freq	b_SMR	se_SMR	p_SMR	p_HEIDI
VHD	CFDP1	16	75397489	rs7201989	75471499	G	A	0.246	-0.056	0.018	0.001	0.119
VHD	STAT3	17	40502964	rs1053004	40466092	G	A	0.380	0.093	0.047	0.048	0.948
VHD	IMPDH2	3	49064299	rs34037363	48821036	T	C	0.114	-0.209	0.105	0.047	0.089
VHD	UBE2L3	22	21941029	rs2070512	21949411	C	A	0.194	-0.029	0.009	0.002	0.623
VHD	STMN3	20	62271061	rs909333	62270637	G	T	0.207	-0.076	0.036	0.036	0.093

C) Effect of AGER gene on VHD and gastrointestinal disorders.

Disease	Gene	chr	Probe_bp	topSNP	topSNP_bp	A1	A2	Freq	b_SMR	se_SMR	p_SMR	p_HEIDI
CD	AGER	6	32150423	rs693906	31835164	C	G	0.113	-0.312	0.120	0.009	6.16E-08
GORD	AGER	6	32150423	rs693906	31835164	C	G	0.113	-0.209	0.049	2.02E-05	0.022
IBD	AGER	6	32148745	rs204995	32154285	G	A	0.217	-0.252	0.108	0.020	1.60E-04
IBS	AGER	6	32150423	rs693906	31835164	C	G	0.113	-0.168	0.052	0.001	0.045
UC	AGER	6	32148745	rs204995	32154285	G	A	0.217	-0.870	0.185	2.59E-06	0.002
VHD	AGER	6	32150423	rs693906	31835164	C	G	0.113	0.212	0.052	4.82E-05	0.092

Supplementary table 6. Results of the summary-based Mendelian randomization integrating whole-blood expression quantitative trait loci. For each disease-gene association, we report the strongest effect observed between GTEX and eQTLGen datasets.

A) Bonferroni-significant disease-gene association with HEIDI P > 0.05.

Disease	Gene	chr	gene_bp	topSNP	topSNP_bp	A1	A2	Freq	b_SMR	se_SMR	p_SMR	p_HEIDI
CD	DNMT3A	2	25510652	rs7578575	25488819	A	T	0.329	-0.834	0.164	3.51E-07	0.383
IBD	DNMT3A	2	25510652	rs7578575	25488819	A	T	0.329	-0.745	0.130	9.22E-09	0.515
UC	DNMT3A	2	25510652	rs7578575	25488819	A	T	0.329	-0.627	0.156	5.73E-05	0.541
UC	CFDP1	16	75327596	rs9934007	75390532	C	T	0.754	0.273	0.070	9.61E-05	0.973
CD	STAT3	17	40502964	rs1053004	40466092	G	A	0.380	0.702	0.128	4.40E-08	0.056
CD	IMPDH2	3	49064299	rs9813813	49207093	A	T	0.110	-1.233	0.294	2.74E-05	0.213
IBD	IMPDH2	3	49064299	rs9813813	49207093	A	T	0.110	-1.328	0.258	2.57E-07	0.071
UC	IMPDH2	3	49064299	rs9813813	49207093	A	T	0.110	-1.445	0.307	2.53E-06	0.050
IBD	UBE2L3	22	21941029	rs2070512	21949411	C	A	0.194	0.163	0.021	1.06E-14	0.169
UC	UBE2L3	22	21941029	rs2070512	21949411	C	A	0.194	0.116	0.027	1.76E-05	0.698
CD	BCL2L15	1	1.14E+08	rs1217393	1.14E+08	A	G	0.271	-0.705	0.170	3.39E-05	0.184
CD	STMN3	20	62271061	rs909333	62270637	G	T	0.207	0.547	0.102	8.31E-08	0.200
IBD	STMN3	20	62271061	rs909333	62270637	G	T	0.207	0.555	0.087	2.01E-10	0.055
UC	STMN3	20	62271061	rs909333	62270637	G	T	0.207	0.498	0.098	4.04E-07	0.062
GORD	PBX2	6	32154964	rs693906	31835164	C	G	0.113	0.154	0.036	1.65E-05	0.058
VHD	AGER	6	32150423	rs693906	31835164	C	G	0.113	0.212	0.052	4.82E-05	0.092

B) Nominally significant effect on VHD of Bonferroni-significant gastrointestinal loci.

Disease	Gene	chr	Probe_bp	topSNP	topSNP_bp	A1	A2	Freq	b_SMR	se_SMR	p_SMR	p_HEIDI
VHD	CFDP1	16	75397489	rs7201989	75471499	G	A	0.246	-0.056	0.018	0.001	0.119
VHD	STAT3	17	40502964	rs1053004	40466092	G	A	0.380	0.093	0.047	0.048	0.948
VHD	IMPDH2	3	49064299	rs34037363	48821036	T	C	0.114	-0.209	0.105	0.047	0.089
VHD	UBE2L3	22	21941029	rs2070512	21949411	C	A	0.194	-0.029	0.009	0.002	0.623
VHD	STMN3	20	62271061	rs909333	62270637	G	T	0.207	-0.076	0.036	0.036	0.093

C) Effect of AGER gene on VHD and gastrointestinal disorders.

Disease	Gene	chr	Probe_bp	topSNP	topSNP_bp	A1	A2	Freq	b_SMR	se_SMR	p_SMR	p_HEIDI
CD	AGER	6	32150423	rs693906	31835164	C	G	0.113	-0.312	0.120	0.009	6.16E-08
GORD	AGER	6	32150423	rs693906	31835164	C	G	0.113	-0.209	0.049	2.02E-05	0.022
IBD	AGER	6	32148745	rs204995	32154285	G	A	0.217	-0.252	0.108	0.020	1.60E-04
IBS	AGER	6	32150423	rs693906	31835164	C	G	0.113	-0.168	0.052	0.001	0.045
UC	AGER	6	32148745	rs204995	32154285	G	A	0.217	-0.870	0.185	2.59E-06	0.002
VHD	AGER	6	32150423	rs693906	31835164	C	G	0.113	0.212	0.052	4.82E-05	0.092

Supplementary table 6. Results of the summary-based Mendelian randomization integrating whole-blood expression quantitative trait loci. For each disease-gene association, we report the strongest effect observed between GTEX and eQTLGen datasets.

A) Bonferroni-significant disease-gene association with HEIDI P > 0.05.

Disease	Gene	chr	gene_bp	topSNP	topSNP_bp	A1	A2	Freq	b_SMR	se_SMR	p_SMR	p_HEIDI
CD	DNMT3A	2	25510652	rs7578575	25488819	A	T	0.329	-0.834	0.164	3.51E-07	0.383
IBD	DNMT3A	2	25510652	rs7578575	25488819	A	T	0.329	-0.745	0.130	9.22E-09	0.515
UC	DNMT3A	2	25510652	rs7578575	25488819	A	T	0.329	-0.627	0.156	5.73E-05	0.541
UC	CFDP1	16	75327596	rs9934007	75390532	C	T	0.754	0.273	0.070	9.61E-05	0.973
CD	STAT3	17	40502964	rs1053004	40466092	G	A	0.380	0.702	0.128	4.40E-08	0.056
CD	IMPDH2	3	49064299	rs9813813	49207093	A	T	0.110	-1.233	0.294	2.74E-05	0.213
IBD	IMPDH2	3	49064299	rs9813813	49207093	A	T	0.110	-1.328	0.258	2.57E-07	0.071
UC	IMPDH2	3	49064299	rs9813813	49207093	A	T	0.110	-1.445	0.307	2.53E-06	0.050
IBD	UBE2L3	22	21941029	rs2070512	21949411	C	A	0.194	0.163	0.021	1.06E-14	0.169
UC	UBE2L3	22	21941029	rs2070512	21949411	C	A	0.194	0.116	0.027	1.76E-05	0.698
CD	BCL2L15	1	1.14E+08	rs1217393	1.14E+08	A	G	0.271	-0.705	0.170	3.39E-05	0.184
CD	STMN3	20	62271061	rs909333	62270637	G	T	0.207	0.547	0.102	8.31E-08	0.200
IBD	STMN3	20	62271061	rs909333	62270637	G	T	0.207	0.555	0.087	2.01E-10	0.055
UC	STMN3	20	62271061	rs909333	62270637	G	T	0.207	0.498	0.098	4.04E-07	0.062
GORD	PBX2	6	32154964	rs693906	31835164	C	G	0.113	0.154	0.036	1.65E-05	0.058
VHD	AGER	6	32150423	rs693906	31835164	C	G	0.113	0.212	0.052	4.82E-05	0.092

B) Nominally significant effect on VHD of Bonferroni-significant gastrointestinal loci.

Disease	Gene	chr	Probe_bp	topSNP	topSNP_bp	A1	A2	Freq	b_SMR	se_SMR	p_SMR	p_HEIDI
VHD	CFDP1	16	75397489	rs7201989	75471499	G	A	0.246	-0.056	0.018	0.001	0.119
VHD	STAT3	17	40502964	rs1053004	40466092	G	A	0.380	0.093	0.047	0.048	0.948
VHD	IMPDH2	3	49064299	rs34037363	48821036	T	C	0.114	-0.209	0.105	0.047	0.089
VHD	UBE2L3	22	21941029	rs2070512	21949411	C	A	0.194	-0.029	0.009	0.002	0.623
VHD	STMN3	20	62271061	rs909333	62270637	G	T	0.207	-0.076	0.036	0.036	0.093

C) Effect of AGER gene on VHD and gastrointestinal disorders.

Disease	Gene	chr	Probe_bp	topSNP	topSNP_bp	A1	A2	Freq	b_SMR	se_SMR	p_SMR	p_HEIDI
CD	AGER	6	32150423	rs693906	31835164	C	G	0.113	-0.312	0.120	0.009	6.16E-08
GORD	AGER	6	32150423	rs693906	31835164	C	G	0.113	-0.209	0.049	2.02E-05	0.022
IBD	AGER	6	32148745	rs204995	32154285	G	A	0.217	-0.252	0.108	0.020	1.60E-04
IBS	AGER	6	32150423	rs693906	31835164	C	G	0.113	-0.168	0.052	0.001	0.045
UC	AGER	6	32148745	rs204995	32154285	G	A	0.217	-0.870	0.185	2.59E-06	0.002
VHD	AGER	6	32150423	rs693906	31835164	C	G	0.113	0.212	0.052	4.82E-05	0.092

Supplementary table 6. Results of the summary-based Mendelian randomization integrating whole-blood expression quantitative trait loci. For each disease-gene association, we report the strongest effect observed between GTEX and eQTLGen datasets.

A) Bonferroni-significant disease-gene association with HEIDI P > 0.05.

Disease	Gene	chr	gene_bp	topSNP	topSNP_bp	A1	A2	Freq	b_SMR	se_SMR	p_SMR	p_HEIDI
CD	DNMT3A	2	25510652	rs7578575	25488819	A	T	0.329	-0.834	0.164	3.51E-07	0.383
IBD	DNMT3A	2	25510652	rs7578575	25488819	A	T	0.329	-0.745	0.130	9.22E-09	0.515
UC	DNMT3A	2	25510652	rs7578575	25488819	A	T	0.329	-0.627	0.156	5.73E-05	0.541
UC	CFDP1	16	75327596	rs9934007	75390532	C	T	0.754	0.273	0.070	9.61E-05	0.973
CD	STAT3	17	40502964	rs1053004	40466092	G	A	0.380	0.702	0.128	4.40E-08	0.056
CD	IMPDH2	3	49064299	rs9813813	49207093	A	T	0.110	-1.233	0.294	2.74E-05	0.213
IBD	IMPDH2	3	49064299	rs9813813	49207093	A	T	0.110	-1.328	0.258	2.57E-07	0.071
UC	IMPDH2	3	49064299	rs9813813	49207093	A	T	0.110	-1.445	0.307	2.53E-06	0.050
IBD	UBE2L3	22	21941029	rs2070512	21949411	C	A	0.194	0.163	0.021	1.06E-14	0.169
UC	UBE2L3	22	21941029	rs2070512	21949411	C	A	0.194	0.116	0.027	1.76E-05	0.698
CD	BCL2L15	1	1.14E+08	rs1217393	1.14E+08	A	G	0.271	-0.705	0.170	3.39E-05	0.184
CD	STMN3	20	62271061	rs909333	62270637	G	T	0.207	0.547	0.102	8.31E-08	0.200
IBD	STMN3	20	62271061	rs909333	62270637	G	T	0.207	0.555	0.087	2.01E-10	0.055
UC	STMN3	20	62271061	rs909333	62270637	G	T	0.207	0.498	0.098	4.04E-07	0.062
GORD	PBX2	6	32154964	rs693906	31835164	C	G	0.113	0.154	0.036	1.65E-05	0.058
VHD	AGER	6	32150423	rs693906	31835164	C	G	0.113	0.212	0.052	4.82E-05	0.092

B) Nominally significant effect on VHD of Bonferroni-significant gastrointestinal loci.

Disease	Gene	chr	Probe_bp	topSNP	topSNP_bp	A1	A2	Freq	b_SMR	se_SMR	p_SMR	p_HEIDI
VHD	CFDP1	16	75397489	rs7201989	75471499	G	A	0.246	-0.056	0.018	0.001	0.119
VHD	STAT3	17	40502964	rs1053004	40466092	G	A	0.380	0.093	0.047	0.048	0.948
VHD	IMPDH2	3	49064299	rs34037363	48821036	T	C	0.114	-0.209	0.105	0.047	0.089
VHD	UBE2L3	22	21941029	rs2070512	21949411	C	A	0.194	-0.029	0.009	0.002	0.623
VHD	STMN3	20	62271061	rs909333	62270637	G	T	0.207	-0.076	0.036	0.036	0.093

C) Effect of AGER gene on VHD and gastrointestinal disorders.

Disease	Gene	chr	Probe_bp	topSNP	topSNP_bp	A1	A2	Freq	b_SMR	se_SMR	p_SMR	p_HEIDI
CD	AGER	6	32150423	rs693906	31835164	C	G	0.113	-0.312	0.120	0.009	6.16E-08
GORD	AGER	6	32150423	rs693906	31835164	C	G	0.113	-0.209	0.049	2.02E-05	0.022
IBD	AGER	6	32148745	rs204995	32154285	G	A	0.217	-0.252	0.108	0.020	1.60E-04
IBS	AGER	6	32150423	rs693906	31835164	C	G	0.113	-0.168	0.052	0.001	0.045
UC	AGER	6	32148745	rs204995	32154285	G	A	0.217	-0.870	0.185	2.59E-06	0.002
VHD	AGER	6	32150423	rs693906	31835164	C	G	0.113	0.212	0.052	4.82E-05	0.092

Supplementary table 6. Results of the summary-based Mendelian randomization integrating whole-blood expression quantitative trait loci. For each disease-gene association, we report the strongest effect observed between GTEX and eQTLGen datasets.

A) Bonferroni-significant disease-gene association with HEIDI P > 0.05.

Disease	Gene	chr	gene_bp	topSNP	topSNP_bp	A1	A2	Freq	b_SMR	se_SMR	p_SMR	p_HEIDI
CD	DNMT3A	2	25510652	rs7578575	25488819	A	T	0.329	-0.834	0.164	3.51E-07	0.383
IBD	DNMT3A	2	25510652	rs7578575	25488819	A	T	0.329	-0.745	0.130	9.22E-09	0.515
UC	DNMT3A	2	25510652	rs7578575	25488819	A	T	0.329	-0.627	0.156	5.73E-05	0.541
UC	CFDP1	16	75327596	rs9934007	75390532	C	T	0.754	0.273	0.070	9.61E-05	0.973
CD	STAT3	17	40502964	rs1053004	40466092	G	A	0.380	0.702	0.128	4.40E-08	0.056
CD	IMPDH2	3	49064299	rs9813813	49207093	A	T	0.110	-1.233	0.294	2.74E-05	0.213
IBD	IMPDH2	3	49064299	rs9813813	49207093	A	T	0.110	-1.328	0.258	2.57E-07	0.071
UC	IMPDH2	3	49064299	rs9813813	49207093	A	T	0.110	-1.445	0.307	2.53E-06	0.050
IBD	UBE2L3	22	21941029	rs2070512	21949411	C	A	0.194	0.163	0.021	1.06E-14	0.169
UC	UBE2L3	22	21941029	rs2070512	21949411	C	A	0.194	0.116	0.027	1.76E-05	0.698
CD	BCL2L15	1	1.14E+08	rs1217393	1.14E+08	A	G	0.271	-0.705	0.170	3.39E-05	0.184
CD	STMN3	20	62271061	rs909333	62270637	G	T	0.207	0.547	0.102	8.31E-08	0.200
IBD	STMN3	20	62271061	rs909333	62270637	G	T	0.207	0.555	0.087	2.01E-10	0.055
UC	STMN3	20	62271061	rs909333	62270637	G	T	0.207	0.498	0.098	4.04E-07	0.062
GORD	PBX2	6	32154964	rs693906	31835164	C	G	0.113	0.154	0.036	1.65E-05	0.058
VHD	AGER	6	32150423	rs693906	31835164	C	G	0.113	0.212	0.052	4.82E-05	0.092

B) Nominally significant effect on VHD of Bonferroni-significant gastrointestinal loci.

Disease	Gene	chr	Probe_bp	topSNP	topSNP_bp	A1	A2	Freq	b_SMR	se_SMR	p_SMR	p_HEIDI
VHD	CFDP1	16	75397489	rs7201989	75471499	G	A	0.246	-0.056	0.018	0.001	0.119
VHD	STAT3	17	40502964	rs1053004	40466092	G	A	0.380	0.093	0.047	0.048	0.948
VHD	IMPDH2	3	49064299	rs34037363	48821036	T	C	0.114	-0.209	0.105	0.047	0.089
VHD	UBE2L3	22	21941029	rs2070512	21949411	C	A	0.194	-0.029	0.009	0.002	0.623
VHD	STMN3	20	62271061	rs909333	62270637	G	T	0.207	-0.076	0.036	0.036	0.093

C) Effect of AGER gene on VHD and gastrointestinal disorders.

Disease	Gene	chr	Probe_bp	topSNP	topSNP_bp	A1	A2	Freq	b_SMR	se_SMR	p_SMR	p_HEIDI
CD	AGER	6	32150423	rs693906	31835164	C	G	0.113	-0.312	0.120	0.009	6.16E-08
GORD	AGER	6	32150423	rs693906	31835164	C	G	0.113	-0.209	0.049	2.02E-05	0.022
IBD	AGER	6	32148745	rs204995	32154285	G	A	0.217	-0.252	0.108	0.020	1.60E-04
IBS	AGER	6	32150423	rs693906	31835164	C	G	0.113	-0.168	0.052	0.001	0.045
UC	AGER	6	32148745	rs204995	32154285	G	A	0.217	-0.870	0.185	2.59E-06	0.002
VHD	AGER	6	32150423	rs693906	31835164	C	G	0.113	0.212	0.052	4.82E-05	0.092

Supplementary table 6. Results of the summary-based Mendelian randomization integrating whole-blood expression quantitative trait loci. For each disease-gene association, we report the strongest effect observed between GTEX and eQTLGen datasets.

A) Bonferroni-significant disease-gene association with HEIDI P > 0.05.

Disease	Gene	chr	gene_bp	topSNP	topSNP_bp	A1	A2	Freq	b_SMR	se_SMR	p_SMR	p_HEIDI
CD	DNMT3A	2	25510652	rs7578575	25488819	A	T	0.329	-0.834	0.164	3.51E-07	0.383
IBD	DNMT3A	2	25510652	rs7578575	25488819	A	T	0.329	-0.745	0.130	9.22E-09	0.515
UC	DNMT3A	2	25510652	rs7578575	25488819	A	T	0.329	-0.627	0.156	5.73E-05	0.541
UC	CFDP1	16	75327596	rs9934007	75390532	C	T	0.754	0.273	0.070	9.61E-05	0.973
CD	STAT3	17	40502964	rs1053004	40466092	G	A	0.380	0.702	0.128	4.40E-08	0.056
CD	IMPDH2	3	49064299	rs9813813	49207093	A	T	0.110	-1.233	0.294	2.74E-05	0.213
IBD	IMPDH2	3	49064299	rs9813813	49207093	A	T	0.110	-1.328	0.258	2.57E-07	0.071
UC	IMPDH2	3	49064299	rs9813813	49207093	A	T	0.110	-1.445	0.307	2.53E-06	0.050
IBD	UBE2L3	22	21941029	rs2070512	21949411	C	A	0.194	0.163	0.021	1.06E-14	0.169
UC	UBE2L3	22	21941029	rs2070512	21949411	C	A	0.194	0.116	0.027	1.76E-05	0.698
CD	BCL2L15	1	1.14E+08	rs1217393	1.14E+08	A	G	0.271	-0.705	0.170	3.39E-05	0.184
CD	STMN3	20	62271061	rs909333	62270637	G	T	0.207	0.547	0.102	8.31E-08	0.200
IBD	STMN3	20	62271061	rs909333	62270637	G	T	0.207	0.555	0.087	2.01E-10	0.055
UC	STMN3	20	62271061	rs909333	62270637	G	T	0.207	0.498	0.098	4.04E-07	0.062
GORD	PBX2	6	32154964	rs693906	31835164	C	G	0.113	0.154	0.036	1.65E-05	0.058
VHD	AGER	6	32150423	rs693906	31835164	C	G	0.113	0.212	0.052	4.82E-05	0.092

B) Nominally significant effect on VHD of Bonferroni-significant gastrointestinal loci.

Disease	Gene	chr	Probe_bp	topSNP	topSNP_bp	A1	A2	Freq	b_SMR	se_SMR	p_SMR	p_HEIDI
VHD	CFDP1	16	75397489	rs7201989	75471499	G	A	0.246	-0.056	0.018	0.001	0.119
VHD	STAT3	17	40502964	rs1053004	40466092	G	A	0.380	0.093	0.047	0.048	0.948
VHD	IMPDH2	3	49064299	rs34037363	48821036	T	C	0.114	-0.209	0.105	0.047	0.089
VHD	UBE2L3	22	21941029	rs2070512	21949411	C	A	0.194	-0.029	0.009	0.002	0.623
VHD	STMN3	20	62271061	rs909333	62270637	G	T	0.207	-0.076	0.036	0.036	0.093

C) Effect of AGER gene on VHD and gastrointestinal disorders.

Disease	Gene	chr	Probe_bp	topSNP	topSNP_bp	A1	A2	Freq	b_SMR	se_SMR	p_SMR	p_HEIDI
CD	AGER	6	32150423	rs693906	31835164	C	G	0.113	-0.312	0.120	0.009	6.16E-08
GORD	AGER	6	32150423	rs693906	31835164	C	G	0.113	-0.209	0.049	2.02E-05	0.022
IBD	AGER	6	32148745	rs204995	32154285	G	A	0.217	-0.252	0.108	0.020	1.60E-04
IBS	AGER	6	32150423	rs693906	31835164	C	G	0.113	-0.168	0.052	0.001	0.045
UC	AGER	6	32148745	rs204995	32154285	G	A	0.217	-0.870	0.185	2.59E-06	0.002
VHD	AGER	6	32150423	rs693906	31835164	C	G	0.113	0.212	0.052	4.82E-05	0.092

Supplementary table 6. Results of the summary-based Mendelian randomization integrating whole-blood expression quantitative trait loci. For each disease-gene association, we report the strongest effect observed between GTEX and eQTLGen datasets.

A) Bonferroni-significant disease-gene association with HEIDI P > 0.05.

Disease	Gene	chr	gene_bp	topSNP	topSNP_bp	A1	A2	Freq	b_SMR	se_SMR	p_SMR	p_HEIDI
CD	DNMT3A	2	25510652	rs7578575	25488819	A	T	0.329	-0.834	0.164	3.51E-07	0.383
IBD	DNMT3A	2	25510652	rs7578575	25488819	A	T	0.329	-0.745	0.130	9.22E-09	0.515
UC	DNMT3A	2	25510652	rs7578575	25488819	A	T	0.329	-0.627	0.156	5.73E-05	0.541
UC	CFDP1	16	75327596	rs9934007	75390532	C	T	0.754	0.273	0.070	9.61E-05	0.973
CD	STAT3	17	40502964	rs1053004	40466092	G	A	0.380	0.702	0.128	4.40E-08	0.056
CD	IMPDH2	3	49064299	rs9813813	49207093	A	T	0.110	-1.233	0.294	2.74E-05	0.213
IBD	IMPDH2	3	49064299	rs9813813	49207093	A	T	0.110	-1.328	0.258	2.57E-07	0.071
UC	IMPDH2	3	49064299	rs9813813	49207093	A	T	0.110	-1.445	0.307	2.53E-06	0.050
IBD	UBE2L3	22	21941029	rs2070512	21949411	C	A	0.194	0.163	0.021	1.06E-14	0.169
UC	UBE2L3	22	21941029	rs2070512	21949411	C	A	0.194	0.116	0.027	1.76E-05	0.698
CD	BCL2L15	1	1.14E+08	rs1217393	1.14E+08	A	G	0.271	-0.705	0.170	3.39E-05	0.184
CD	STMN3	20	62271061	rs909333	62270637	G	T	0.207	0.547	0.102	8.31E-08	0.200
IBD	STMN3	20	62271061	rs909333	62270637	G	T	0.207	0.555	0.087	2.01E-10	0.055
UC	STMN3	20	62271061	rs909333	62270637	G	T	0.207	0.498	0.098	4.04E-07	0.062
GORD	PBX2	6	32154964	rs693906	31835164	C	G	0.113	0.154	0.036	1.65E-05	0.058
VHD	AGER	6	32150423	rs693906	31835164	C	G	0.113	0.212	0.052	4.82E-05	0.092

B) Nominally significant effect on VHD of Bonferroni-significant gastrointestinal loci.

Disease	Gene	chr	Probe_bp	topSNP	topSNP_bp	A1	A2	Freq	b_SMR	se_SMR	p_SMR	p_HEIDI
VHD	CFDP1	16	75397489	rs7201989	75471499	G	A	0.246	-0.056	0.018	0.001	0.119
VHD	STAT3	17	40502964	rs1053004	40466092	G	A	0.380	0.093	0.047	0.048	0.948
VHD	IMPDH2	3	49064299	rs34037363	48821036	T	C	0.114	-0.209	0.105	0.047	0.089
VHD	UBE2L3	22	21941029	rs2070512	21949411	C	A	0.194	-0.029	0.009	0.002	0.623
VHD	STMN3	20	62271061	rs909333	62270637	G	T	0.207	-0.076	0.036	0.036	0.093

C) Effect of AGER gene on VHD and gastrointestinal disorders.

Disease	Gene	chr	Probe_bp	topSNP	topSNP_bp	A1	A2	Freq	b_SMR	se_SMR	p_SMR	p_HEIDI
CD	AGER	6	32150423	rs693906	31835164	C	G	0.113	-0.312	0.120	0.009	6.16E-08
GORD	AGER	6	32150423	rs693906	31835164	C	G	0.113	-0.209	0.049	2.02E-05	0.022
IBD	AGER	6	32148745	rs204995	32154285	G	A	0.217	-0.252	0.108	0.020	1.60E-04
IBS	AGER	6	32150423	rs693906	31835164	C	G	0.113	-0.168	0.052	0.001	0.045
UC	AGER	6	32148745	rs204995	32154285	G	A	0.217	-0.870	0.185	2.59E-06	0.002
VHD	AGER	6	32150423	rs693906	31835164	C	G	0.113	0.212	0.052	4.82E-05	0.092

Supplementary table 6. Results of the summary-based Mendelian randomization integrating whole-blood expression quantitative trait loci. For each disease-gene association, we report the strongest effect observed between GTEX and eQTLGen datasets.

A) Bonferroni-significant disease-gene association with HEIDI P > 0.05.

Disease	Gene	chr	gene_bp	topSNP	topSNP_bp	A1	A2	Freq	b_SMR	se_SMR	p_SMR	p_HEIDI
CD	DNMT3A	2	25510652	rs7578575	25488819	A	T	0.329	-0.834	0.164	3.51E-07	0.383
IBD	DNMT3A	2	25510652	rs7578575	25488819	A	T	0.329	-0.745	0.130	9.22E-09	0.515
UC	DNMT3A	2	25510652	rs7578575	25488819	A	T	0.329	-0.627	0.156	5.73E-05	0.541
UC	CFDP1	16	75327596	rs9934007	75390532	C	T	0.754	0.273	0.070	9.61E-05	0.973
CD	STAT3	17	40502964	rs1053004	40466092	G	A	0.380	0.702	0.128	4.40E-08	0.056
CD	IMPDH2	3	49064299	rs9813813	49207093	A	T	0.110	-1.233	0.294	2.74E-05	0.213
IBD	IMPDH2	3	49064299	rs9813813	49207093	A	T	0.110	-1.328	0.258	2.57E-07	0.071
UC	IMPDH2	3	49064299	rs9813813	49207093	A	T	0.110	-1.445	0.307	2.53E-06	0.050
IBD	UBE2L3	22	21941029	rs2070512	21949411	C	A	0.194	0.163	0.021	1.06E-14	0.169
UC	UBE2L3	22	21941029	rs2070512	21949411	C	A	0.194	0.116	0.027	1.76E-05	0.698
CD	BCL2L15	1	1.14E+08	rs1217393	1.14E+08	A	G	0.271	-0.705	0.170	3.39E-05	0.184
CD	STMN3	20	62271061	rs909333	62270637	G	T	0.207	0.547	0.102	8.31E-08	0.200
IBD	STMN3	20	62271061	rs909333	62270637	G	T	0.207	0.555	0.087	2.01E-10	0.055
UC	STMN3	20	62271061	rs909333	62270637	G	T	0.207	0.498	0.098	4.04E-07	0.062
GORD	PBX2	6	32154964	rs693906	31835164	C	G	0.113	0.154	0.036	1.65E-05	0.058
VHD	AGER	6	32150423	rs693906	31835164	C	G	0.113	0.212	0.052	4.82E-05	0.092

B) Nominally significant effect on VHD of Bonferroni-significant gastrointestinal loci.

Disease	Gene	chr	Probe_bp	topSNP	topSNP_bp	A1	A2	Freq	b_SMR	se_SMR	p_SMR	p_HEIDI
VHD	CFDP1	16	75397489	rs7201989	75471499	G	A	0.246	-0.056	0.018	0.001	0.119
VHD	STAT3	17	40502964	rs1053004	40466092	G	A	0.380	0.093	0.047	0.048	0.948
VHD	IMPDH2	3	49064299	rs34037363	48821036	T	C	0.114	-0.209	0.105	0.047	0.089
VHD	UBE2L3	22	21941029	rs2070512	21949411	C	A	0.194	-0.029	0.009	0.002	0.623
VHD	STMN3	20	62271061	rs909333	62270637	G	T	0.207	-0.076	0.036	0.036	0.093

C) Effect of AGER gene on VHD and gastrointestinal disorders.

Disease	Gene	chr	Probe_bp	topSNP	topSNP_bp	A1	A2	Freq	b_SMR	se_SMR	p_SMR	p_HEIDI
CD	AGER	6	32150423	rs693906	31835164	C	G	0.113	-0.312	0.120	0.009	6.16E-08
GORD	AGER	6	32150423	rs693906	31835164	C	G	0.113	-0.209	0.049	2.02E-05	0.022
IBD	AGER	6	32148745	rs204995	32154285	G	A	0.217	-0.252	0.108	0.020	1.60E-04
IBS	AGER	6	32150423	rs693906	31835164	C	G	0.113	-0.168	0.052	0.001	0.045
UC	AGER	6	32148745	rs204995	32154285	G	A	0.217	-0.870	0.185	2.59E-06	0.002
VHD	AGER	6	32150423	rs693906	31835164	C	G	0.113	0.212	0.052	4.82E-05	0.092

Supplementary table 6. Results of the summary-based Mendelian randomization integrating whole-blood expression quantitative trait loci. For each disease-gene association, we report the strongest effect observed between GTEX and eQTLGen datasets.

A) Bonferroni-significant disease-gene association with HEIDI P > 0.05.

Disease	Gene	chr	gene_bp	topSNP	topSNP_bp	A1	A2	Freq	b_SMR	se_SMR	p_SMR	p_HEIDI
CD	DNMT3A	2	25510652	rs7578575	25488819	A	T	0.329	-0.834	0.164	3.51E-07	0.383
IBD	DNMT3A	2	25510652	rs7578575	25488819	A	T	0.329	-0.745	0.130	9.22E-09	0.515
UC	DNMT3A	2	25510652	rs7578575	25488819	A	T	0.329	-0.627	0.156	5.73E-05	0.541
UC	CFDP1	16	75327596	rs9934007	75390532	C	T	0.754	0.273	0.070	9.61E-05	0.973
CD	STAT3	17	40502964	rs1053004	40466092	G	A	0.380	0.702	0.128	4.40E-08	0.056
CD	IMPDH2	3	49064299	rs9813813	49207093	A	T	0.110	-1.233	0.294	2.74E-05	0.213
IBD	IMPDH2	3	49064299	rs9813813	49207093	A	T	0.110	-1.328	0.258	2.57E-07	0.071
UC	IMPDH2	3	49064299	rs9813813	49207093	A	T	0.110	-1.445	0.307	2.53E-06	0.050
IBD	UBE2L3	22	21941029	rs2070512	21949411	C	A	0.194	0.163	0.021	1.06E-14	0.169
UC	UBE2L3	22	21941029	rs2070512	21949411	C	A	0.194	0.116	0.027	1.76E-05	0.698
CD	BCL2L15	1	1.14E+08	rs1217393	1.14E+08	A	G	0.271	-0.705	0.170	3.39E-05	0.184
CD	STMN3	20	62271061	rs909333	62270637	G	T	0.207	0.547	0.102	8.31E-08	0.200
IBD	STMN3	20	62271061	rs909333	62270637	G	T	0.207	0.555	0.087	2.01E-10	0.055
UC	STMN3	20	62271061	rs909333	62270637	G	T	0.207	0.498	0.098	4.04E-07	0.062
GORD	PBX2	6	32154964	rs693906	31835164	C	G	0.113	0.154	0.036	1.65E-05	0.058
VHD	AGER	6	32150423	rs693906	31835164	C	G	0.113	0.212	0.052	4.82E-05	0.092

B) Nominally significant effect on VHD of Bonferroni-significant gastrointestinal loci.

Disease	Gene	chr	Probe_bp	topSNP	topSNP_bp	A1	A2	Freq	b_SMR	se_SMR	p_SMR	p_HEIDI
VHD	CFDP1	16	75397489	rs7201989	75471499	G	A	0.246	-0.056	0.018	0.001	0.119
VHD	STAT3	17	40502964	rs1053004	40466092	G	A	0.380	0.093	0.047	0.048	0.948
VHD	IMPDH2	3	49064299	rs34037363	48821036	T	C	0.114	-0.209	0.105	0.047	0.089
VHD	UBE2L3	22	21941029	rs2070512	21949411	C	A	0.194	-0.029	0.009	0.002	0.623
VHD	STMN3	20	62271061	rs909333	62270637	G	T	0.207	-0.076	0.036	0.036	0.093

C) Effect of AGER gene on VHD and gastrointestinal disorders.

Disease	Gene	chr	Probe_bp	topSNP	topSNP_bp	A1	A2	Freq	b_SMR	se_SMR	p_SMR	p_HEIDI
CD	AGER	6	32150423	rs693906	31835164	C	G	0.113	-0.312	0.120	0.009	6.16E-08
GORD	AGER	6	32150423	rs693906	31835164	C	G	0.113	-0.209	0.049	2.02E-05	0.022
IBD	AGER	6	32148745	rs204995	32154285	G	A	0.217	-0.252	0.108	0.020	1.60E-04
IBS	AGER	6	32150423	rs693906	31835164	C	G	0.113	-0.168	0.052	0.001	0.045
UC	AGER	6	32148745	rs204995	32154285	G	A	0.217	-0.870	0.185	2.59E-06	0.002
VHD	AGER	6	32150423	rs693906	31835164	C	G	0.113	0.212	0.052	4.82E-05	0.092

Supplementary table 6. Results of the summary-based Mendelian randomization integrating whole-blood expression quantitative trait loci. For each disease-gene association, we report the strongest effect observed between GTEX and eQTLGen datasets.

A) Bonferroni-significant disease-gene association with HEIDI P > 0.05.

Disease	Gene	chr	gene_bp	topSNP	topSNP_bp	A1	A2	Freq	b_SMR	se_SMR	p_SMR	p_HEIDI
CD	DNMT3A	2	25510652	rs7578575	25488819	A	T	0.329	-0.834	0.164	3.51E-07	0.383
IBD	DNMT3A	2	25510652	rs7578575	25488819	A	T	0.329	-0.745	0.130	9.22E-09	0.515
UC	DNMT3A	2	25510652	rs7578575	25488819	A	T	0.329	-0.627	0.156	5.73E-05	0.541
UC	CFDP1	16	75327596	rs9934007	75390532	C	T	0.754	0.273	0.070	9.61E-05	0.973
CD	STAT3	17	40502964	rs1053004	40466092	G	A	0.380	0.702	0.128	4.40E-08	0.056
CD	IMPDH2	3	49064299	rs9813813	49207093	A	T	0.110	-1.233	0.294	2.74E-05	0.213
IBD	IMPDH2	3	49064299	rs9813813	49207093	A	T	0.110	-1.328	0.258	2.57E-07	0.071
UC	IMPDH2	3	49064299	rs9813813	49207093	A	T	0.110	-1.445	0.307	2.53E-06	0.050
IBD	UBE2L3	22	21941029	rs2070512	21949411	C	A	0.194	0.163	0.021	1.06E-14	0.169
UC	UBE2L3	22	21941029	rs2070512	21949411	C	A	0.194	0.116	0.027	1.76E-05	0.698
CD	BCL2L15	1	1.14E+08	rs1217393	1.14E+08	A	G	0.271	-0.705	0.170	3.39E-05	0.184
CD	STMN3	20	62271061	rs909333	62270637	G	T	0.207	0.547	0.102	8.31E-08	0.200
IBD	STMN3	20	62271061	rs909333	62270637	G	T	0.207	0.555	0.087	2.01E-10	0.055
UC	STMN3	20	62271061	rs909333	62270637	G	T	0.207	0.498	0.098	4.04E-07	0.062
GORD	PBX2	6	32154964	rs693906	31835164	C	G	0.113	0.154	0.036	1.65E-05	0.058
VHD	AGER	6	32150423	rs693906	31835164	C	G	0.113	0.212	0.052	4.82E-05	0.092

B) Nominally significant effect on VHD of Bonferroni-significant gastrointestinal loci.

Disease	Gene	chr	Probe_bp	topSNP	topSNP_bp	A1	A2	Freq	b_SMR	se_SMR	p_SMR	p_HEIDI
VHD	CFDP1	16	75397489	rs7201989	75471499	G	A	0.246	-0.056	0.018	0.001	0.119
VHD	STAT3	17	40502964	rs1053004	40466092	G	A	0.380	0.093	0.047	0.048	0.948
VHD	IMPDH2	3	49064299	rs34037363	48821036	T	C	0.114	-0.209	0.105	0.047	0.089
VHD	UBE2L3	22	21941029	rs2070512	21949411	C	A	0.194	-0.029	0.009	0.002	0.623
VHD	STMN3	20	62271061	rs909333	62270637	G	T	0.207	-0.076	0.036	0.036	0.093

C) Effect of AGER gene on VHD and gastrointestinal disorders.

Disease	Gene	chr	Probe_bp	topSNP	topSNP_bp	A1	A2	Freq	b_SMR	se_SMR	p_SMR	p_HEIDI
CD	AGER	6	32150423	rs693906	31835164	C	G	0.113	-0.312	0.120	0.009	6.16E-08
GORD	AGER	6	32150423	rs693906	31835164	C	G	0.113	-0.209	0.049	2.02E-05	0.022
IBD	AGER	6	32148745	rs204995	32154285	G	A	0.217	-0.252	0.108	0.020	1.60E-04
IBS	AGER	6	32150423	rs693906	31835164	C	G	0.113	-0.168	0.052	0.001	0.045
UC	AGER	6	32148745	rs204995	32154285	G	A	0.217	-0.870	0.185	2.59E-06	0.002
VHD	AGER	6	32150423	rs693906	31835164	C	G	0.113	0.212	0.052	4.82E-05	0.092

Supplementary table 6. Results of the summary-based Mendelian randomization integrating whole-blood expression quantitative trait loci. For each disease-gene association, we report the strongest effect observed between GTEX and eQTLGen datasets.

A) Bonferroni-significant disease-gene association with HEIDI P > 0.05.

Disease	Gene	chr	gene_bp	topSNP	topSNP_bp	A1	A2	Freq	b_SMR	se_SMR	p_SMR	p_HEIDI
CD	DNMT3A	2	25510652	rs7578575	25488819	A	T	0.329	-0.834	0.164	3.51E-07	0.383
IBD	DNMT3A	2	25510652	rs7578575	25488819	A	T	0.329	-0.745	0.130	9.22E-09	0.515
UC	DNMT3A	2	25510652	rs7578575	25488819	A	T	0.329	-0.627	0.156	5.73E-05	0.541
UC	CFDP1	16	75327596	rs9934007	75390532	C	T	0.754	0.273	0.070	9.61E-05	0.973
CD	STAT3	17	40502964	rs1053004	40466092	G	A	0.380	0.702	0.128	4.40E-08	0.056
CD	IMPDH2	3	49064299	rs9813813	49207093	A	T	0.110	-1.233	0.294	2.74E-05	0.213
IBD	IMPDH2	3	49064299	rs9813813	49207093	A	T	0.110	-1.328	0.258	2.57E-07	0.071
UC	IMPDH2	3	49064299	rs9813813	49207093	A	T	0.110	-1.445	0.307	2.53E-06	0.050
IBD	UBE2L3	22	21941029	rs2070512	21949411	C	A	0.194	0.163	0.021	1.06E-14	0.169
UC	UBE2L3	22	21941029	rs2070512	21949411	C	A	0.194	0.116	0.027	1.76E-05	0.698
CD	BCL2L15	1	1.14E+08	rs1217393	1.14E+08	A	G	0.271	-0.705	0.170	3.39E-05	0.184
CD	STMN3	20	62271061	rs909333	62270637	G	T	0.207	0.547	0.102	8.31E-08	0.200
IBD	STMN3	20	62271061	rs909333	62270637	G	T	0.207	0.555	0.087	2.01E-10	0.055
UC	STMN3	20	62271061	rs909333	62270637	G	T	0.207	0.498	0.098	4.04E-07	0.062
GORD	PBX2	6	32154964	rs693906	31835164	C	G	0.113	0.154	0.036	1.65E-05	0.058
VHD	AGER	6	32150423	rs693906	31835164	C	G	0.113	0.212	0.052	4.82E-05	0.092

B) Nominally significant effect on VHD of Bonferroni-significant gastrointestinal loci.

Disease	Gene	chr	Probe_bp	topSNP	topSNP_bp	A1	A2	Freq	b_SMR	se_SMR	p_SMR	p_HEIDI
VHD	CFDP1	16	75397489	rs7201989	75471499	G	A	0.246	-0.056	0.018	0.001	0.119
VHD	STAT3	17	40502964	rs1053004	40466092	G	A	0.380	0.093	0.047	0.048	0.948
VHD	IMPDH2	3	49064299	rs34037363	48821036	T	C	0.114	-0.209	0.105	0.047	0.089
VHD	UBE2L3	22	21941029	rs2070512	21949411	C	A	0.194	-0.029	0.009	0.002	0.623
VHD	STMN3	20	62271061	rs909333	62270637	G	T	0.207	-0.076	0.036	0.036	0.093

C) Effect of AGER gene on VHD and gastrointestinal disorders.

Disease	Gene	chr	Probe_bp	topSNP	topSNP_bp	A1	A2	Freq	b_SMR	se_SMR	p_SMR	p_HEIDI
CD	AGER	6	32150423	rs693906	31835164	C	G	0.113	-0.312	0.120	0.009	6.16E-08
GORD	AGER	6	32150423	rs693906	31835164	C	G	0.113	-0.209	0.049	2.02E-05	0.022
IBD	AGER	6	32148745	rs204995	32154285	G	A	0.217	-0.252	0.108	0.020	1.60E-04
IBS	AGER	6	32150423	rs693906	31835164	C	G	0.113	-0.168	0.052	0.001	0.045
UC	AGER	6	32148745	rs204995	32154285	G	A	0.217	-0.870	0.185	2.59E-06	0.002
VHD	AGER	6	32150423	rs693906	31835164	C	G	0.113	0.212	0.052	4.82E-05	0.092

Supplementary table 6. Results of the summary-based Mendelian randomization integrating whole-blood expression quantitative trait loci. For each disease-gene association, we report the strongest effect observed between GTEX and eQTLGen datasets.

A) Bonferroni-significant disease-gene association with HEIDI P > 0.05.

Disease	Gene	chr	gene_bp	topSNP	topSNP_bp	A1	A2	Freq	b_SMR	se_SMR	p_SMR	p_HEIDI
CD	DNMT3A	2	25510652	rs7578575	25488819	A	T	0.329	-0.834	0.164	3.51E-07	0.383
IBD	DNMT3A	2	25510652	rs7578575	25488819	A	T	0.329	-0.745	0.130	9.22E-09	0.515
UC	DNMT3A	2	25510652	rs7578575	25488819	A	T	0.329	-0.627	0.156	5.73E-05	0.541
UC	CFDP1	16	75327596	rs9934007	75390532	C	T	0.754	0.273	0.070	9.61E-05	0.973
CD	STAT3	17	40502964	rs1053004	40466092	G	A	0.380	0.702	0.128	4.40E-08	0.056
CD	IMPDH2	3	49064299	rs9813813	49207093	A	T	0.110	-1.233	0.294	2.74E-05	0.213
IBD	IMPDH2	3	49064299	rs9813813	49207093	A	T	0.110	-1.328	0.258	2.57E-07	0.071
UC	IMPDH2	3	49064299	rs9813813	49207093	A	T	0.110	-1.445	0.307	2.53E-06	0.050
IBD	UBE2L3	22	21941029	rs2070512	21949411	C	A	0.194	0.163	0.021	1.06E-14	0.169
UC	UBE2L3	22	21941029	rs2070512	21949411	C	A	0.194	0.116	0.027	1.76E-05	0.698
CD	BCL2L15	1	1.14E+08	rs1217393	1.14E+08	A	G	0.271	-0.705	0.170	3.39E-05	0.184
CD	STMN3	20	62271061	rs909333	62270637	G	T	0.207	0.547	0.102	8.31E-08	0.200
IBD	STMN3	20	62271061	rs909333	62270637	G	T	0.207	0.555	0.087	2.01E-10	0.055
UC	STMN3	20	62271061	rs909333	62270637	G	T	0.207	0.498	0.098	4.04E-07	0.062
GORD	PBX2	6	32154964	rs693906	31835164	C	G	0.113	0.154	0.036	1.65E-05	0.058
VHD	AGER	6	32150423	rs693906	31835164	C	G	0.113	0.212	0.052	4.82E-05	0.092

B) Nominally significant effect on VHD of Bonferroni-significant gastrointestinal loci.

Disease	Gene	chr	Probe_bp	topSNP	topSNP_bp	A1	A2	Freq	b_SMR	se_SMR	p_SMR	p_HEIDI
VHD	CFDP1	16	75397489	rs7201989	75471499	G	A	0.246	-0.056	0.018	0.001	0.119
VHD	STAT3	17	40502964	rs1053004	40466092	G	A	0.380	0.093	0.047	0.048	0.948
VHD	IMPDH2	3	49064299	rs34037363	48821036	T	C	0.114	-0.209	0.105	0.047	0.089
VHD	UBE2L3	22	21941029	rs2070512	21949411	C	A	0.194	-0.029	0.009	0.002	0.623
VHD	STMN3	20	62271061	rs909333	62270637	G	T	0.207	-0.076	0.036	0.036	0.093

C) Effect of AGER gene on VHD and gastrointestinal disorders.

Disease	Gene	chr	Probe_bp	topSNP	topSNP_bp	A1	A2	Freq	b_SMR	se_SMR	p_SMR	p_HEIDI
CD	AGER	6	32150423	rs693906	31835164	C	G	0.113	-0.312	0.120	0.009	6.16E-08
GORD	AGER	6	32150423	rs693906	31835164	C	G	0.113	-0.209	0.049	2.02E-05	0.022
IBD	AGER	6	32148745	rs204995	32154285	G	A	0.217	-0.252	0.108	0.020	1.60E-04
IBS	AGER	6	32150423	rs693906	31835164	C	G	0.113	-0.168	0.052	0.001	0.045
UC	AGER	6	32148745	rs204995	32154285	G	A	0.217	-0.870	0.185	2.59E-06	0.002
VHD	AGER	6	32150423	rs693906	31835164	C	G	0.113	0.212	0.052	4.82E-05	0.092

Supplementary table 5. Gene-set enrichment with respect to gene-based pleiotropy signals survuving false discovery rate multiple testing correction (FDR q<0.05).

Pleiotropy	GeneSet	Genes, n	P-value	genes
VHD_CD	Inflammatory bowel disease	18	4.75E-21	IL23R, DNMT3A, ATG16L1, ATP6V1G2, PRRC2A, CLIC1, MSH5, VWA7, VARS, LSM2, ATF6B, PBX2, NOTCH4, CUL2, CREM, CCL2, UBE2L3, YDJC
	Ulcerative colitis	14	2.85E-17	IL23R, GTF2H4, ATP6V1G2, PRRC2A, CLIC1, MSH5, VWA7, VARS, LSM2, ATF6B, PBX2, NOTCH4, UBE2L3, YDJC
	Autism spectrum disorder or schizophrenia	13	5.09E-14	SPATS2L, TRIM15, GTF2H4, ATP6V1G2, PRRC2A, CLIC1, MSH5, VWA7, VARS, LSM2, ATF6B, PBX2, NOTCH4
	Crohn's disease	13	1.18E-13	IL23R, BCL2L15, AP4B1, DCLRE1B, DNMT3A, ATG16L1, PBX2, NOTCH4, CUL2, CREM, CCL2, UBE2L3, YDJC
	osing spondylitis, Crohn's disease, psoriasis, primary sclerosing cho	5	1.39E-06	IL23R, DNMT3A, ATG16L1, CREM, UBE2L3
	Takayasu arteritis	4	2.01E-06	PRRC2A, MSH5, ATF6B, NOTCH4
	Blood protein levels	11	2.17E-06	IL23R, TRIM15, ATP6V1G2, PRRC2A, CLIC1, MSH5, VWA7, VARS, LSM2, ATF6B, NOTCH4
	Type 1 diabetes and autoimmune thyroid diseases	3	2.36E-06	BCL2L15, PRRC2A, NOTCH4
	-induced agranulocytosis/granulocytopenia in treatment-resistant sch	3	3.18E-06	MSH5, PBX2, NOTCH4
	Sarcoidosis (Lofgren's syndrome vs non-Lofgren's syndrome)	3	8.33E-06	GTF2H4, VARS, ATF6B
	Epstein-Barr virus immune response (EBNA-1)	2	6.12E-05	PRRC2A, NOTCH4
	Conotruncal heart defects	2	7.65E-05	GTF2H4, VARS
	HIV-1 control	2	2.03E-04	VARS, NOTCH4
	Alopecia areata	3	2.21E-04	BCL2L15, AP4B1, DCLRE1B
	Systemic seropositive rheumatic diseases	2	2.58E-04	AP4B1, YDJC
[illegible]	Autism spectrum disorder or schizophrenia	12	4.00E-17	SFTA2, ATP6V1G2, PRRC2A, MSH5, VWA7, VARS, LSM2, PPT2, EGFL8, AGPAT1, RNF5, AGER, PBX2
	Ulcerative colitis	11	2.54E-16	ATP6V1G2, PRRC2A, MSH5, VWA7, VARS, LSM2, PPT2, EGFL8, AGPAT1, RNF5, AGER, PBX2
	Inflammatory bowel disease	11	5.16E-14	ATP6V1G2, PRRC2A, MSH5, VWA7, VARS, LSM2, PPT2, EGFL8, AGPAT1, RNF5, AGER, PBX2

VHD_GORD	Idiopathic membranous nephropathy	3	5.47E-08	PRRC2A, PPT2, EGFL8, RNF5
	-induced agranulocytosis/granulocytopenia in treatment-resistant sch	3	5.08E-07	MSH5, AGPAT1, PBX2
	Prostate cancer	5	2.69E-06	PPT2, EGFL8, AGPAT1, RNF5, AGER, PBX2
	Advanced glycation end-product levels	2	1.86E-05	RNF5, AGER
	Blood protein levels	7	6.03E-05	ATP6V1G2, PRRC2A, MSH5, VWA7, VARS, LSM2, AGER
	Oleic acid (18:1n-9) levels	2	6.19E-05	PPT2, EGFL8, AGPAT1
VHD_IBD	Inflammatory bowel disease	29	5.31E-38	IL23R, DNMT3A, PUS10, IMPDH2, KLHDC8B, CCDC36, POU5F1, HCG27, PRRC2A, MSH5, VWA7, VARS, LSM2, ATF6B, PPT2, EGFL8, AGPAT1, RNF5, AGER, PBX2, GPSM3, NOTCH4, HLA-DRA, CUL2, CREM, NOD2, STAT3, STMN3, UBE2L3, YDJC
	Ulcerative colitis	25	4.86E-35	IL23R, PUS10, IMPDH2, KLHDC8B, CCDC36, POU5F1, HCG27, PRRC2A, MSH5, VWA7, VARS, LSM2, ATF6B, PPT2, EGFL8, AGPAT1, RNF5, AGER, PBX2, GPSM3, NOTCH4, HLA-DRA, STAT3, STMN3, UBE2L3, YDJC
	Autism spectrum disorder or schizophrenia	16	2.48E-16	POU5F1, HCG27, PRRC2A, MSH5, VWA7, VARS, LSM2, ATF6B, PPT2, EGFL8, AGPAT1, RNF5, AGER, PBX2, GPSM3, NOTCH4, HLA-DRA
	Crohn's disease	16	6.93E-16	IL23R, DNMT3A, PUS10, IMPDH2, KLHDC8B, CCDC36, PBX2, NOTCH4, HLA-DRA, CUL2, CREM, NOD2, STAT3, STMN3, UBE2L3, YDJC
	Prostate cancer	11	5.00E-12	ATF6B, PPT2, EGFL8, AGPAT1, RNF5, AGER, PBX2, GPSM3, NOTCH4, HLA-DRA, GMEB2, STMN3
	›idosis (non-Lofgren's syndrome without extrapulmonary manifestat	5	1.18E-09	ATF6B, AGER, GPSM3, NOTCH4, HLA-DRA
	Blood protein levels	16	1.77E-09	IL23R, IMPDH2, QARS, KLHDC8B, CCDC36, HCG27, PRRC2A, MSH5, VWA7, VARS, LSM2, ATF6B, AGER, GPSM3, NOTCH4, HLA-DRA
	Takayasu arteritis	6	2.05E-09	HCG27, PRRC2A, MSH5, ATF6B, NOTCH4, HLA-DRA
	osing spondylitis, Crohn's disease, psoriasis, primary sclerosing cho	7	6.29E-09	IL23R, DNMT3A, PUS10, CREM, NOD2, STAT3, UBE2L3
	-induced agranulocytosis/granulocytopenia in treatment-resistant sch	4	5.71E-08	MSH5, AGPAT1, PBX2, NOTCH4

	Advanced glycation end-product levels	3	4.16E-07	RNF5, AGER, NOTCH4
	Idiopathic membranous nephropathy	3	8.14E-07	PRRC2A, PPT2, EGFL8, RNF5
	Atopic dermatitis	4	3.63E-06	PUS10, ATF6B, GPSM3, STAT3
	Sarcoidosis (Lofgren's syndrome vs non-Lofgren's syndrome)	3	1.96E-05	VARS, ATF6B, HLA-DRA
	Vogt-Koyanagi-Harada syndrome	2	6.29E-05	IL23R, HLA-DRA
	Cold sores	2	6.29E-05	POU5F1, HCG27
	Psoriasis	4	7.99E-05	IL23R, HCG27, STAT3, UBE2L3
	Epstein-Barr virus immune response (EBNA-1)	2	1.08E-04	PRRC2A, NOTCH4
	Food antigen IgG levels	2	1.08E-04	NOTCH4, HLA-DRA
	Hepatitis B	2	1.08E-04	HLA-DRA, UBE2L3
	Systemic lupus erythematosus	5	2.43E-04	MSH5, NOTCH4, STAT3, UBE2L3, YDJC
	Lung cancer	4	2.71E-04	HCG27, MSH5, NOTCH4, HLA-DRA
	HIV-1 control	2	3.56E-04	VARS, NOTCH4
	Oleic acid (18:1n-9) levels	2	3.56E-04	PPT2, EGFL8, AGPAT1
	Type 1 diabetes and autoimmune thyroid diseases	2	5.61E-04	PRRC2A, NOTCH4
VHD_IBS	Takayasu arteritis	2	1.34E-05	PRRC2A, MSH5
	Menopause (age at onset)	2	2.33E-05	PRRC2A, MSH5
	Ulcerative colitis	16	7.12E-23	PSORS1C1, CDSN, CCHCR1, TCF19, POU5F1, HCG27, ATF6B, PPT2, EGFL8, AGPAT1, RNF5, AGER, PBX2, GPSM3, NOTCH4, AMZ1, UBE2L3
	Inflammatory bowel disease	16	1.66E-19	PSORS1C1, CDSN, CCHCR1, TCF19, POU5F1, HCG27, ATF6B, PPT2, EGFL8, AGPAT1, RNF5, AGER, PBX2, GPSM3, NOTCH4, AMZ1, UBE2L3
	Autism spectrum disorder or schizophrenia	15	5.36E-19	PSORS1C1, CDSN, CCHCR1, TCF19, POU5F1, HCG27, ATF6B, PPT2, EGFL8, AGPAT1, RNF5, AGER, PBX2, GPSM3, NOTCH4, HLA-DPB1
	Prostate cancer	9	3.13E-11	CCHCR1, ATF6B, PPT2, EGFL8, AGPAT1, RNF5, AGER, PBX2, GPSM3, NOTCH4
	Stevens-Johnson syndrome and toxic epidermal necrolysis	4	4.08E-11	PSORS1C1, CCHCR1, TCF19, POU5F1
	Multiple myeloma	5	6.87E-11	PSORS1C1, CDSN, CCHCR1, TCF19, POU5F1

VHD_UC	idosis (non-Lofgren's syndrome without extrapulmonary manifestat	5	1.02E-10	ATF6B, AGER, GPSM3, NOTCH4, HLA-DPB1
	Idiopathic membranous nephropathy	4	3.83E-10	PSORS1C1, TCF19, PPT2, EGFL8, RNF5
	Takayasu arteritis	5	1.14E-08	PSORS1C1, CDSN, HCG27, ATF6B, NOTCH4
	Advanced glycation end-product levels	3	1.02E-07	RNF5, AGER, NOTCH4
	Chronic hepatitis B infection	3	4.39E-07	TCF19, NOTCH4, HLA-DPB1
	-induced agranulocytosis/granulocytopenia in treatment-resistant sch	3	1.85E-06	AGPAT1, PBX2, NOTCH4
	Hematology traits	3	2.42E-06	PSORS1C1, CDSN, CCHCR1
	Carotid plaque	2	1.19E-05	CFDP1, TMEM170A
	rug-induced Stevens-Johnson syndrome or toxic epidermal necrolys	2	2.51E-05	PSORS1C1, POU5F1
	Cold sores	2	2.51E-05	POU5F1, HCG27
	Systemic sclerosis	3	3.79E-05	PSORS1C1, NOTCH4, HLA-DPB1
	Hepatitis B	2	4.29E-05	HLA-DPB1, UBE2L3
	Epstein-Barr virus immune response (EBNA-1)	2	4.29E-05	PSORS1C1, NOTCH4
	White blood cell count	4	4.83E-05	PSORS1C1, CDSN, CCHCR1, HCG27
	Chronic obstructive pulmonary disease or coronary artery disease	2	1.25E-04	CFDP1, TMEM170A
	Oleic acid (18:1n-9) levels	2	1.42E-04	PPT2, EGFL8, AGPAT1
	HIV-1 control	2	1.42E-04	CDSN, NOTCH4
	Response to hepatitis B vaccine	2	1.81E-04	PSORS1C1, HLA-DPB1
	Blood protein levels	8	1.88E-04	PSORS1C1, CDSN, HCG27, ATF6B, AGER, GPSM3, NOTCH4, HLA-DPB1
	Type 1 diabetes and autoimmune thyroid diseases	2	2.25E-04	PSORS1C1, NOTCH4
	Chronic obstructive pulmonary disease-related biomarkers	2	2.99E-04	PSORS1C1, CCHCR1
	Psoriasis	3	4.06E-04	CCHCR1, HCG27, UBE2L3
	Chronic obstructive pulmonary disease	3	4.53E-04	AGER, AMZ1, CFDP1
	Night sleep phenotypes	4	6.14E-04	PSORS1C1, CCHCR1, TCF19, POU5F1
	Feeling nervous	2	8.65E-04	PSORS1C1, CCHCR1
	Automobile speeding propensity	2	8.65E-04	CDSN, ATF6B

Supplementary table 6. Results of the summary-based Mendelian randomization integrating whole-blood expression quantitative trait loci. For each disease-gene association, we report the strongest effect observed between GTEX and eQTLGen datasets.

A) Bonferroni-significant disease-gene association with HEIDI P > 0.05.

Disease	Gene	chr	gene_bp	topSNP	topSNP_bp	A1	A2	Freq	b_SMR	se_SMR	p_SMR	p_HEIDI
CD	DNMT3A	2	25510652	rs7578575	25488819	A	T	0.329	-0.834	0.164	3.51E-07	0.383
IBD	DNMT3A	2	25510652	rs7578575	25488819	A	T	0.329	-0.745	0.130	9.22E-09	0.515
UC	DNMT3A	2	25510652	rs7578575	25488819	A	T	0.329	-0.627	0.156	5.73E-05	0.541
UC	CFDP1	16	75327596	rs9934007	75390532	C	T	0.754	0.273	0.070	9.61E-05	0.973
CD	STAT3	17	40502964	rs1053004	40466092	G	A	0.380	0.702	0.128	4.40E-08	0.056
CD	IMPDH2	3	49064299	rs9813813	49207093	A	T	0.110	-1.233	0.294	2.74E-05	0.213
IBD	IMPDH2	3	49064299	rs9813813	49207093	A	T	0.110	-1.328	0.258	2.57E-07	0.071
UC	IMPDH2	3	49064299	rs9813813	49207093	A	T	0.110	-1.445	0.307	2.53E-06	0.050
IBD	UBE2L3	22	21941029	rs2070512	21949411	C	A	0.194	0.163	0.021	1.06E-14	0.169
UC	UBE2L3	22	21941029	rs2070512	21949411	C	A	0.194	0.116	0.027	1.76E-05	0.698
CD	BCL2L15	1	1.14E+08	rs1217393	1.14E+08	A	G	0.271	-0.705	0.170	3.39E-05	0.184
CD	STMN3	20	62271061	rs909333	62270637	G	T	0.207	0.547	0.102	8.31E-08	0.200
IBD	STMN3	20	62271061	rs909333	62270637	G	T	0.207	0.555	0.087	2.01E-10	0.055
UC	STMN3	20	62271061	rs909333	62270637	G	T	0.207	0.498	0.098	4.04E-07	0.062
GORD	PBX2	6	32154964	rs693906	31835164	C	G	0.113	0.154	0.036	1.65E-05	0.058
VHD	AGER	6	32150423	rs693906	31835164	C	G	0.113	0.212	0.052	4.82E-05	0.092

B) Nominally significant effect on VHD of Bonferroni-significant gastrointestinal loci.

Disease	Gene	chr	Probe_bp	topSNP	topSNP_bp	A1	A2	Freq	b_SMR	se_SMR	p_SMR	p_HEIDI
VHD	CFDP1	16	75397489	rs7201989	75471499	G	A	0.246	-0.056	0.018	0.001	0.119
VHD	STAT3	17	40502964	rs1053004	40466092	G	A	0.380	0.093	0.047	0.048	0.948
VHD	IMPDH2	3	49064299	rs34037363	48821036	T	C	0.114	-0.209	0.105	0.047	0.089
VHD	UBE2L3	22	21941029	rs2070512	21949411	C	A	0.194	-0.029	0.009	0.002	0.623
VHD	STMN3	20	62271061	rs909333	62270637	G	T	0.207	-0.076	0.036	0.036	0.093

C) Effect of AGER gene on VHD and gastrointestinal disorders.

Disease	Gene	chr	Probe_bp	topSNP	topSNP_bp	A1	A2	Freq	b_SMR	se_SMR	p_SMR	p_HEIDI
CD	AGER	6	32150423	rs693906	31835164	C	G	0.113	-0.312	0.120	0.009	6.16E-08
GORD	AGER	6	32150423	rs693906	31835164	C	G	0.113	-0.209	0.049	2.02E-05	0.022
IBD	AGER	6	32148745	rs204995	32154285	G	A	0.217	-0.252	0.108	0.020	1.60E-04
IBS	AGER	6	32150423	rs693906	31835164	C	G	0.113	-0.168	0.052	0.001	0.045
UC	AGER	6	32148745	rs204995	32154285	G	A	0.217	-0.870	0.185	2.59E-06	0.002
VHD	AGER	6	32150423	rs693906	31835164	C	G	0.113	0.212	0.052	4.82E-05	0.092

Supplementary table 7. Mendelian randomization between gastrointestinal disorders and VHD.

Exposures	Outcomes	F_min	n_snp	IVW (fixed-effects)						IVW (random effects)						Weighted Mode						Weighted Median						MR-Egger								Heterogeneity test	
				beta	SE	OR	UCI	LCI	Pvalue	beta	SE	OR	UCI	LCI	Pvalue	beta	SE	OR	Down	Up	Pval	beta	SE	OR	Down	Up	Pval	beta	SE	OR	UCI	LCI	Pvalue	intercept	intercept-Pvalue	Q	P value
CD		30.142	76	0.008	0.005	1.008	0.997	1.018	0.157	0.008	0.005	1.008	0.997	1.018	0.157	0.000	0.011	1.000	0.978	1.022	0.997	0.000	0.008	1.000	0.984	1.016	0.961	-0.001	0.014	0.999	0.971	1.027	0.931	0.002	0.503	70.440	0.628
IBD		29.860	107	0.007	0.006	1.007	0.995	1.019	0.249	0.007	0.007	1.007	0.994	1.020	0.285	0.004	0.012	1.004	0.980	1.029	0.742	0.008	0.010	1.008	0.989	1.027	0.401	-0.018	0.017	0.982	0.950	1.015	0.271	0.003	0.097	123.468	0.118
UC		30.461	61	0.007	0.007	1.007	0.995	1.020	0.260	0.007	0.007	1.007	0.995	1.020	0.260	0.010	0.016	1.010	0.980	1.042	0.505	0.010	0.009	1.010	0.992	1.029	0.276	-0.003	0.021	0.997	0.957	1.040	0.905	0.002	0.622	57.157	0.580
GORD	VHD	30.113	5	0.033	0.072	1.034	0.897	1.191	0.648	0.033	0.072	1.034	0.897	1.191	0.648	0.073	0.108	1.075	0.870	1.329	0.502	0.059	0.091	1.060	0.887	1.267	0.519	-0.115	0.610	0.891	0.128	6.200	0.862	0.006	0.822	3.436	0.488
IBS		30.506	6	-0.117	0.063	0.890	0.786	1.006	0.063	-0.117	0.078	0.890	0.764	1.036	0.131	-0.152	0.118	0.859	0.682	1.081	0.195	-0.151	0.080	0.860	0.735	1.006	0.060	-0.204	1.117	0.815	0.037	18.122	0.864	0.004	0.941	7.569	0.182
PGM		32.551	17	0.039	0.047	1.040	0.948	1.140	0.406	0.039	0.056	1.040	0.931	1.161	0.489	-0.083	0.095	0.920	0.764	1.108	0.381	-0.055	0.069	0.946	0.827	1.082	0.420	-0.047	0.221	0.954	0.596	1.527	0.833	0.004	0.691	23.041	0.113
PUD		34.809	6	0.009	0.034	1.009	0.944	1.078	0.796	0.009	0.053	1.009	0.909	1.120	0.870	-0.083	0.102	0.921	0.754	1.124	0.416	0.003	0.048	1.003	0.913	1.102	0.946	-0.265	0.296	0.767	0.337	1.744	0.421	0.023	0.400	12.421	0.029
	CD	31.598	11	0.026	0.100	1.026	0.844	1.248	0.795	0.026	0.111	1.026	0.825	1.277	0.816	0.164	0.204	1.178	0.790	1.757	0.422	0.113	0.144	1.120	0.845	1.485	0.431	0.244	0.176	1.277	0.857	1.902	0.199	-0.018	0.158	12.506	0.253
	IBD	31.598	11	0.033	0.079	1.033	0.886	1.205	0.679	0.033	0.085	1.033	0.875	1.220	0.702	0.200	0.175	1.221	0.867	1.720	0.253	0.152	0.109	1.164	0.940	1.440	0.163	0.289	0.136	1.335	1.022	1.742	0.034	-0.020	0.023	11.697	0.306
	UC	31.598	11	0.015	0.096	1.015	0.840	1.226	0.878	0.015	0.096	1.015	0.840	1.226	0.878	0.026	0.161	1.026	0.749	1.406	0.872	0.010	0.131	1.010	0.781	1.306	0.941	0.179	0.208	1.197	0.752	1.905	0.405	-0.012	0.390	9.561	0.480
VHD	GORD	31.234	12	0.036	0.038	1.036	0.962	1.116	0.350	0.036	0.039	1.036	0.959	1.119	0.367	-0.005	0.057	0.995	0.890	1.112	0.925	-0.008	0.051	0.992	0.897	1.097	0.876	-0.029	0.087	0.971	0.799	1.180	0.744	0.005	0.423	11.797	0.379
	IBS	31.234	12	-0.082	0.047	0.921	0.840	1.009	0.078	-0.082	0.047	0.921	0.840	1.009	0.078	-0.161	0.112	0.851	0.684	1.059	0.149	-0.145	0.065	0.865	0.761	0.983	0.026	-0.286	0.125	0.751	0.588	0.959	0.022	0.012	0.078	8.657	0.654
	PGM	31.234	10	0.030	0.033	1.031	0.967	1.099	0.351	0.030	0.041	1.031	0.951	1.117	0.461	0.045	0.049	1.046	0.950	1.153	0.360	0.022	0.046	1.022	0.935	1.118	0.632	0.136	0.083	1.145	0.946	1.387	0.141	-0.008	0.189	14.413	0.108
	PUD	31.359	11	0.121	0.067	1.128	0.990	1.286	0.071	0.121	0.083	1.128	0.959	1.327	0.145	-0.065	0.166	0.937	0.677	1.297	0.694	0.009	0.099	1.009	0.830	1.226	0.930	0.147	0.187	1.158	0.758	1.770	0.454	-0.002	0.879	15.316	0.121

Supplementary table 8. Mendelian randomization testing the effect of gut microbiota on VHD and gastrointestinal diseases. Effect surviving Bonferroni multiple testing correction (P<2.37E-04) are highlihgted in bold red. Effect surviving the exploratory multiple testing correction (P<0.01) are indicated in red

Gut microbiota	Group	N	beta.VHD	pvalue.VHD	beta.GORD	pvalue.GORD	beta.PUD	pvalue.PUD	beta.IBS	pvalue.IBS	beta.IBD	pvalue.IBD	beta.CD	pvalue.CD	beta.UC	pvalue.UC
Butyricicoccus	genus	7	-0.133	0.004	-0.100	0.005	-0.043	0.468	-0.010	0.794	0.058	0.427	0.230	0.020	0.119	0.465
Coprococcus2	genus	10	0.081	0.011	-0.054	0.107	-0.086	0.125	0.036	0.323	0.133	0.035	0.088	0.277	0.167	0.037
Victivallis	genus	11	0.056	0.001	0.030	0.063	-0.065	0.027	-0.005	0.778	0.059	0.091	0.012	0.801	0.050	0.261
Lentisphaeria	class	9	0.066	0.003	0.041	0.054	-0.052	0.198	-0.012	0.589	-0.042	0.480	-0.108	0.066	-0.057	0.282
Victivallales	order	9	0.066	0.003	0.041	0.054	-0.052	0.198	-0.012	0.589	-0.042	0.480	-0.108	0.066	-0.057	0.282
Ruminiclostridium5	genus	14	0.094	0.007	-0.064	0.128	0.004	0.951	0.075	0.043	-0.032	0.656	-0.030	0.765	0.052	0.536
Oscillibacter	genus	14	0.066	0.004	0.001	0.960	0.052	0.197	-0.013	0.600	-0.003	0.955	0.022	0.729	-0.028	0.648
Oscillospira	genus	8	-0.012	0.735	-0.096	0.004	0.023	0.684	-0.027	0.443	0.015	0.813	0.012	0.888	0.052	0.540
RuminococcaceaeUCG013	genus	11	0.078	0.027	-0.089	0.005	-0.028	0.616	0.002	0.951	-0.045	0.485	-0.134	0.106	0.036	0.744
Allisonella	genus	8	0.014	0.446	0.070	0.002	-0.032	0.422	0.014	0.486	0.043	0.256	0.057	0.238	0.013	0.782
Proteobacteria	phylum	12	-0.038	0.267	0.122	0.001	0.046	0.488	-0.012	0.829	-0.071	0.346	-0.051	0.603	0.001	0.992
LachnospiraceaeUCG008	genus	10	0.030	0.215	0.091	1.79E-04	0.056	0.193	-0.004	0.864	0.054	0.244	0.059	0.318	0.048	0.410
Anaerotruncus	genus	14	-0.032	0.349	-0.029	0.405	0.178	0.004	-0.055	0.142	-0.054	0.422	0.083	0.355	-0.082	0.375
Eubacteriumxylanophilumgroup	genus	11	0.002	0.942	-0.042	0.270	0.200	0.004	-0.051	0.216	-0.043	0.518	-0.053	0.585	-0.032	0.701
RuminococcaceaeUCG009	genus	9	0.028	0.300	0.035	0.260	-0.014	0.800	-0.109	2.06E-04	-0.109	0.039	-0.242	0.001	-0.024	0.725
Flavonifractor	genus	8	-0.050	0.138	-0.033	0.325	0.077	0.205	0.108	0.006	0.049	0.623	-0.033	0.790	0.257	0.004
Rikenellaceae	family	16	-0.031	0.307	-0.045	0.154	-0.007	0.900	0.081	0.007	0.073	0.178	0.055	0.448	0.104	0.133
Melainabacteria	class	11	-0.035	0.135	0.004	0.849	0.048	0.186	0.066	0.004	0.020	0.611	0.067	0.180	-0.047	0.361
Erysipelotrichia	class	11	0.088	0.027	-0.075	0.099	0.044	0.552	0.117	0.007	0.183	0.027	0.061	0.609	0.084	0.399
Erysipelotrichaceae	family	11	0.088	0.027	-0.075	0.099	0.044	0.552	0.117	0.007	0.183	0.027	0.061	0.609	0.084	0.399
Erysipelotrichales	order	11	0.088	0.027	-0.075	0.099	0.044	0.552	0.117	0.007	0.183	0.027	0.061	0.609	0.084	0.399
unknownfamily	family	10	-0.022	0.363	-0.003	0.875	0.019	0.605	0.078	0.001	0.035	0.390	0.068	0.193	-0.026	0.627
unknowngenus	genus	10	-0.022	0.363	-0.003	0.875	0.019	0.605	0.078	0.001	0.035	0.390	0.068	0.193	-0.026	0.627
Gastranaerophilales	order	10	-0.022	0.363	-0.003	0.875	0.019	0.605	0.078	0.001	0.035	0.390	0.068	0.193	-0.026	0.627
Rhodospirillales	order	13	0.011	0.643	0.000	0.997	-0.022	0.636	0.081	0.003	0.020	0.761	-0.049	0.460	0.025	0.800
unknownfamily	family	12	0.025	0.201	-0.038	0.051	-0.033	0.301	0.016	0.414	0.136	0.001	0.173	0.001	0.082	0.089
unknowngenus	genus	12	0.025	0.201	-0.038	0.051	-0.033	0.301	0.016	0.414	0.136	0.001	0.173	0.001	0.082	0.089
NB1n	order	12	0.025	0.201	-0.038	0.051	-0.033	0.301	0.016	0.414	0.136	0.001	0.173	0.001	0.082	0.089
Eubacteriumventriosumgroup	genus	14	0.005	0.864	0.055	0.109	-0.035	0.542	-0.058	0.095	-0.209	0.001	-0.056	0.502	-0.357	1.29E-05
Sutterella	genus	11	-0.051	0.122	0.001	0.978	-0.051	0.509	0.028	0.419	-0.176	0.007	0.039	0.647	-0.246	0.003
Coprococcus1	genus	12	0.035	0.283	-0.026	0.433	-0.032	0.570	0.067	0.053	0.228	0.001	0.160	0.079	0.263	0.004
RuminococcaceaeUCG014	genus	12	-0.023	0.490	-0.013	0.655	-0.038	0.455	0.015	0.644	0.189	0.013	0.198	0.006	0.208	0.006
Clostridiaceae1	family	11	-0.022	0.520	-0.027	0.440	-0.097	0.102	0.011	0.761	-0.179	0.016	-0.310	0.002	-0.161	0.088
LachnospiraceaeUCG001	genus	15	-0.039	0.108	-0.002	0.930	-0.011	0.810	-0.026	0.358	-0.092	0.056	-0.170	0.006	-0.052	0.400
Eggerthella	genus	8	-0.033	0.183	0.010	0.710	-0.036	0.451	-0.012	0.624	-0.080	0.072	-0.168	0.006	-0.044	0.441
Dialister	genus	11	-0.015	0.643	-0.005	0.903	-0.014	0.803	0.037	0.319	-0.127	0.072	-0.263	0.004	0.070	0.462
unknowngenus	genus	17	-0.011	0.663	0.004	0.898	-0.044	0.354	-0.054	0.071	-0.020	0.691	0.051	0.450	-0.195	0.004
Lactobacillus	genus	9	0.019	0.427	0.056	0.039	-0.074	0.119	-0.040	0.153	-0.105	0.044	0.021	0.766	-0.199	0.005
Eubacteriumruminantiumgroup	genus	16	-0.028	0.155	0.010	0.621	-0.008	0.828	0.030	0.168	0.105	0.010	0.038	0.477	0.141	0.007
Lactobacillaceae	family	11	0.024	0.280	-0.018	0.439	0.063	0.133	-0.023	0.388	-0.074	0.110	-0.025	0.696	-0.153	0.010
Actinomyces	genus	7	-0.041	0.200	0.029	0.336	-0.066	0.230	0.003	0.918	-0.017	0.789	0.012	0.882	-0.202	0.020
Victivallaceae	family	12	0.012	0.474	-0.020	0.216	-0.048	0.098	-0.001	0.962	0.030	0.343	-0.017	0.688	0.094	0.021
Ruminococcaceae	family	11	-0.005	0.884	-0.001	0.968	0.006	0.919	0.024	0.556	0.016	0.812	-0.116	0.190	0.217	0.022
Eubacteriumfissicatenagroup	genus	8	0.019	0.406	0.011	0.641	-0.015	0.710	-0.022	0.329	0.054	0.239	-0.103	0.076	0.132	0.023
Oxalobacteraceae	family	13	0.008	0.652	-0.022	0.284	0.011	0.742	-0.019	0.370	0.091	0.013	0.067	0.158	0.102	0.028
unknowngenus	genus	11	-0.012	0.647	-0.017	0.562	-0.022	0.662	-0.010	0.723	-0.077	0.122	-0.099	0.141	-0.147	0.029
RuminococcaceaeUCG011	genus	7	0.056	0.011	-0.010	0.683	0.060	0.113	0.027	0.253	-0.034	0.400	0.052	0.316	-0.110	0.033
Catenibacterium	genus	4	-0.022	0.443	0.055	0.025	-0.072	0.131	-0.019	0.531	-0.033	0.552	0.057	0.422	-0.131	0.033

Gut microbiota	Group	N	beta.VHD	pvalue.VHD	beta.GORD	pvalue.GORD	beta.PUD	pvalue.PUD	beta.IBS	pvalue.IBS	beta.IBD	pvalue.IBD	beta.CD	pvalue.CD	beta.UC	pvalue.UC
Clostridiales	order	10	-0.023	0.600	0.019	0.586	-0.061	0.325	-0.023	0.535	0.137	0.047	-0.020	0.821	0.179	0.035
Ruminococcus2	genus	14	-0.012	0.694	0.031	0.338	0.089	0.158	0.037	0.268	-0.094	0.161	-0.060	0.456	-0.166	0.036
Olsenella	genus	10	0.000	0.995	-0.004	0.816	0.077	0.029	-0.035	0.095	-0.067	0.062	-0.033	0.498	-0.100	0.038
Cyanobacteria	phylum	9	0.015	0.573	0.003	0.908	0.058	0.174	0.019	0.551	-0.046	0.367	0.051	0.407	-0.134	0.040
Peptococcaceae	family	9	-0.060	0.048	-0.009	0.742	0.091	0.085	-0.039	0.205	-0.118	0.040	-0.022	0.766	-0.138	0.046
LachnospiraceaeUCG004	genus	11	-0.025	0.481	-0.052	0.118	-0.120	0.039	0.029	0.419	-0.118	0.086	-0.100	0.219	-0.157	0.060
Desulfovibrionales	order	10	0.047	0.228	0.018	0.598	0.025	0.693	-0.009	0.798	0.134	0.044	0.067	0.430	0.152	0.061
Bilophila	genus	15	-0.014	0.623	-0.062	0.047	0.044	0.415	-0.015	0.636	0.096	0.127	0.037	0.652	0.149	0.063
Turicibacter	genus	10	0.057	0.041	-0.034	0.239	-0.062	0.195	-0.040	0.175	0.120	0.028	0.002	0.977	0.122	0.068
unknowngenus	genus	10	0.035	0.218	0.011	0.674	0.045	0.346	0.022	0.416	0.136	0.017	0.134	0.055	0.125	0.069
Ruminiclostridium6	genus	12	0.063	0.065	-0.015	0.571	-0.040	0.434	0.066	0.021	-0.087	0.155	-0.066	0.337	-0.139	0.073
Holdemania	genus	15	-0.011	0.645	-0.018	0.406	0.020	0.601	-0.025	0.282	0.003	0.948	-0.024	0.656	0.091	0.094
LachnospiraceaeUCG010	genus	10	-0.049	0.168	-0.083	0.017	-0.098	0.091	-0.020	0.563	0.166	0.013	0.118	0.281	0.135	0.098
Lentisphaerae	phylum	10	0.053	0.012	0.047	0.024	-0.048	0.209	-0.012	0.596	-0.061	0.285	-0.122	0.027	-0.083	0.101
RuminococcaceaeUCG002	genus	21	0.024	0.353	0.018	0.478	0.005	0.935	-0.005	0.845	-0.018	0.721	0.045	0.506	-0.099	0.108
Clostridia	class	9	-0.051	0.262	0.043	0.234	-0.073	0.250	-0.046	0.243	0.079	0.252	-0.028	0.758	0.140	0.109
Eubacteriumeligensgroup	genus	8	-0.024	0.502	-0.089	0.027	0.035	0.581	0.058	0.164	-0.128	0.156	0.141	0.421	0.159	0.113
Eubacteriumoxidoreducensgroup	genus	6	0.024	0.396	-0.036	0.374	0.039	0.502	-0.013	0.737	-0.008	0.911	-0.023	0.778	-0.125	0.115
Prevotellaceae	family	17	-0.022	0.418	0.003	0.907	-0.066	0.175	0.062	0.037	-0.119	0.030	-0.080	0.258	-0.110	0.116
RikenellaceaeRC9gutgroup	genus	11	-0.024	0.155	-0.010	0.525	0.048	0.097	0.015	0.386	-0.047	0.172	0.021	0.625	-0.070	0.121
Enterorhabdus	genus	7	-0.029	0.338	-0.016	0.710	0.034	0.495	0.021	0.488	-0.118	0.061	-0.100	0.159	-0.125	0.123
Acidaminococcaceae	family	7	0.042	0.239	0.006	0.868	0.086	0.178	-0.011	0.775	-0.017	0.815	0.219	0.029	-0.142	0.126
FamilyXIIIUCG001	genus	7	0.017	0.672	0.042	0.231	0.002	0.975	0.010	0.793	0.096	0.184	0.061	0.566	0.149	0.127
LachnospiraceaeFCS020group	genus	14	0.002	0.950	-0.044	0.111	0.053	0.249	0.009	0.812	0.130	0.012	0.099	0.137	0.104	0.127
Butyricimonas	genus	14	0.065	0.020	-0.013	0.627	-0.036	0.416	-0.046	0.088	0.025	0.632	0.012	0.867	0.105	0.131
unknowngenus	genus	12	0.012	0.698	-0.007	0.829	-0.021	0.705	-0.023	0.472	0.041	0.498	0.046	0.540	0.114	0.132
Desulfovibrionaceae	family	9	0.053	0.193	0.018	0.605	0.026	0.682	-0.014	0.704	0.112	0.103	0.050	0.569	0.122	0.145
RuminococcaceaeUCG005	genus	14	0.012	0.690	-0.005	0.866	0.021	0.683	0.021	0.496	0.080	0.167	0.037	0.622	0.104	0.146
Bacillales	order	6	0.007	0.780	0.005	0.778	-0.077	0.026	-0.008	0.692	-0.054	0.135	-0.022	0.630	-0.070	0.154
Bacteroidetes	phylum	11	0.052	0.164	0.001	0.987	-0.031	0.600	0.057	0.146	-0.109	0.162	-0.118	0.218	-0.138	0.156
Peptostreptococcaceae	family	13	0.008	0.853	-0.008	0.801	0.045	0.460	0.019	0.569	0.174	0.031	0.118	0.199	0.128	0.160
unknowngenus	genus	13	-0.014	0.439	-0.026	0.182	0.028	0.404	-0.018	0.371	0.003	0.935	0.058	0.263	-0.068	0.178
Negativicutes	class	10	0.002	0.970	0.023	0.537	0.083	0.231	-0.047	0.292	-0.020	0.797	-0.102	0.293	-0.137	0.180
Selenomonadales	order	10	0.002	0.970	0.023	0.537	0.083	0.231	-0.047	0.292	-0.020	0.797	-0.102	0.293	-0.137	0.180
Firmicutes	phylum	12	-0.074	0.042	0.005	0.884	-0.081	0.161	-0.014	0.690	0.005	0.943	-0.162	0.054	0.108	0.190
Bacteroidia	class	13	0.033	0.347	0.007	0.822	-0.012	0.827	0.013	0.705	-0.149	0.047	-0.062	0.519	-0.127	0.191
Bacteroidales	order	13	0.033	0.347	0.007	0.822	-0.012	0.827	0.013	0.705	-0.149	0.047	-0.062	0.519	-0.127	0.191
Lactobacillales	order	16	0.066	0.024	0.035	0.210	-0.015	0.762	-0.002	0.941	-0.007	0.895	-0.020	0.786	-0.102	0.193
Oxalobacter	genus	10	0.032	0.112	-0.004	0.858	-0.029	0.387	-0.036	0.092	0.084	0.025	0.092	0.058	0.059	0.214
unknowngenus	genus	6	0.076	0.062	-0.017	0.622	-0.053	0.502	-0.014	0.703	-0.107	0.112	-0.036	0.683	-0.099	0.216
FamilyXIII	family	8	-0.102	0.024	0.063	0.136	0.097	0.188	0.035	0.368	-0.154	0.062	-0.057	0.587	-0.122	0.216
Christensenellaceae	family	9	0.029	0.371	-0.047	0.177	-0.075	0.263	-0.023	0.572	-0.146	0.038	-0.241	0.014	-0.110	0.218
DefluviitaleaceaeUCG011	genus	9	-0.031	0.319	0.022	0.455	0.073	0.157	-0.008	0.787	-0.035	0.565	0.046	0.553	-0.095	0.220
Methanobacteria	class	10	0.015	0.453	-0.033	0.084	-0.057	0.076	0.008	0.673	-0.034	0.328	-0.035	0.423	-0.056	0.221
Methanobacteriaceae	family	10	0.015	0.453	-0.033	0.084	-0.057	0.076	0.008	0.673	-0.034	0.328	-0.035	0.423	-0.056	0.221
Methanobacteriales	order	10	0.015	0.453	-0.033	0.084	-0.057	0.076	0.008	0.673	-0.034	0.328	-0.035	0.423	-0.056	0.221
Roseburia	genus	14	0.022	0.497	-0.071	0.034	-0.011	0.838	-0.082	0.035	0.008	0.905	-0.009	0.914	0.106	0.228
Clostridiumsensustricto1	genus	7	0.024	0.674	0.047	0.234	0.018	0.766	0.033	0.391	-0.108	0.163	-0.154	0.212	-0.117	0.232
Bacilli	class	17	0.049	0.096	0.030	0.265	0.002	0.958	0.011	0.737	0.016	0.772	0.115	0.110	-0.087	0.243
Enterobacteriaceae	family	10	0.003	0.944	0.044	0.263	0.001	0.993	-0.006	0.893	-0.025	0.794	-0.016	0.902	-0.104	0.264
Enterobacteriales	order	10	0.003	0.944	0.044	0.263	0.001	0.993	-0.006	0.893	-0.025	0.794	-0.016	0.902	-0.104	0.264

Gut microbiota	Group	N	beta.VHD	pvalue.VHD	beta.GORD	pvalue.GORD	beta.PUD	pvalue.PUD	beta.IBS	pvalue.IBS	beta.IBD	pvalue.IBD	beta.CD	pvalue.CD	beta.UC	pvalue.UC
Tyzzerella3	genus	10	-0.050	0.027	0.007	0.749	-0.087	0.013	0.029	0.195	0.032	0.421	0.020	0.703	0.057	0.265
Actinobacteria	class	17	-0.013	0.657	-0.021	0.536	0.123	0.026	0.003	0.931	-0.078	0.158	0.002	0.973	-0.076	0.271
Haemophilus	genus	12	0.002	0.941	0.037	0.134	-0.037	0.376	-0.009	0.745	-0.129	0.026	-0.173	0.014	0.067	0.272
Veillonella	genus	6	0.063	0.096	-0.034	0.350	-0.126	0.031	-0.022	0.469	-0.057	0.351	0.033	0.694	-0.084	0.280
Veillonellaceae	family	18	-0.023	0.393	0.060	0.025	-0.063	0.172	0.014	0.715	-0.081	0.154	-0.084	0.239	-0.068	0.298
ClostridialesvadinBB60group	family	16	0.031	0.201	-0.027	0.272	-0.040	0.339	-0.023	0.398	-0.093	0.069	-0.006	0.944	-0.068	0.298
unknowngenus	genus	16	0.031	0.201	-0.027	0.272	-0.040	0.339	-0.023	0.398	-0.093	0.069	-0.006	0.944	-0.068	0.298
Pasteurellaceae	family	17	-0.016	0.425	-0.014	0.492	0.011	0.761	-0.009	0.685	-0.054	0.220	-0.084	0.137	0.052	0.305
Pasteurellales	order	17	-0.016	0.425	-0.014	0.492	0.011	0.761	-0.009	0.685	-0.054	0.220	-0.084	0.137	0.052	0.305
Euryarchaeota	phylum	10	0.030	0.113	0.020	0.314	-0.053	0.094	0.010	0.614	-0.035	0.308	-0.014	0.747	-0.044	0.310
Intestinimonas	genus	17	-0.006	0.797	-0.001	0.982	-0.012	0.781	0.014	0.590	0.045	0.383	-0.029	0.648	0.065	0.320
Eubacteriumbrachygroup	genus	11	-0.019	0.323	-0.005	0.774	-0.009	0.802	0.000	0.981	-0.046	0.232	0.012	0.803	-0.048	0.331
Blautia	genus	12	-0.036	0.331	0.031	0.422	0.039	0.568	-0.024	0.528	-0.014	0.863	0.012	0.909	-0.087	0.332
Anaerofilum	genus	8	-0.005	0.828	-0.022	0.432	-0.058	0.227	-0.025	0.325	-0.028	0.604	-0.022	0.753	-0.075	0.334
Ruminococcusgauvreauiigroup	genus	11	0.001	0.983	-0.007	0.845	0.132	0.027	0.079	0.026	-0.067	0.292	-0.006	0.943	-0.078	0.335
Ruminococcustorquesgroup	genus	10	-0.023	0.575	-0.017	0.631	0.040	0.552	-0.010	0.788	-0.058	0.412	-0.026	0.850	0.093	0.340
Erysipelatoclostridium	genus	14	0.039	0.114	0.037	0.182	-0.018	0.716	0.044	0.097	-0.048	0.504	-0.075	0.244	-0.065	0.341
Terrisporobacter	genus	6	-0.034	0.307	0.002	0.966	-0.119	0.029	-0.016	0.624	-0.072	0.238	-0.104	0.183	-0.072	0.355
FamilyXIIIAD3011group	genus	11	0.048	0.187	0.038	0.250	0.014	0.813	-0.010	0.790	-0.087	0.210	-0.086	0.366	-0.080	0.361
Faecalibacterium	genus	11	-0.046	0.179	0.033	0.285	0.003	0.958	0.005	0.889	-0.066	0.305	-0.017	0.833	0.079	0.368
RuminococcaceaeUCG004	genus	12	-0.045	0.098	0.044	0.187	-0.036	0.549	0.022	0.466	0.030	0.594	-0.041	0.567	0.085	0.385
Deltaproteobacteria	class	11	0.039	0.294	-0.006	0.847	0.034	0.559	-0.020	0.572	0.057	0.361	0.072	0.387	0.066	0.405
CandidatusSoleaferrea	genus	13	0.028	0.372	-0.003	0.928	-0.064	0.117	-0.026	0.278	-0.010	0.831	0.089	0.126	-0.048	0.407
Alistipes	genus	12	0.002	0.966	-0.087	0.019	-0.147	0.017	0.000	0.998	0.044	0.511	0.044	0.624	0.070	0.409
Ruminococcus1	genus	12	0.009	0.785	-0.028	0.562	0.008	0.902	-0.054	0.133	-0.007	0.913	-0.076	0.368	0.065	0.418
RuminococcaceaeUCG010	genus	6	0.012	0.799	-0.034	0.473	0.108	0.151	-0.008	0.862	-0.068	0.491	0.047	0.685	0.081	0.424
Senegalimassilia	genus	7	0.003	0.928	0.015	0.753	0.010	0.865	-0.035	0.298	0.037	0.530	0.071	0.353	0.062	0.434
Collinsella	genus	12	-0.022	0.536	0.028	0.415	-0.005	0.944	-0.067	0.080	0.035	0.594	0.035	0.774	0.067	0.452
ErysipelotrichaceaeUCG003	genus	17	0.021	0.442	-0.042	0.139	-0.051	0.298	-0.045	0.110	-0.024	0.659	-0.016	0.859	-0.052	0.456
Peptococcus	genus	16	-0.020	0.253	0.032	0.083	-0.008	0.795	-0.023	0.206	0.020	0.571	0.082	0.064	-0.033	0.460
Coriobacteriia	class	21	-0.024	0.355	-0.043	0.204	0.080	0.113	0.002	0.955	0.015	0.775	0.056	0.468	0.050	0.465
Coriobacteriaceae	family	21	-0.024	0.355	-0.043	0.204	0.080	0.113	0.002	0.955	0.015	0.775	0.056	0.468	0.050	0.465
Coriobacteriales	order	21	-0.024	0.355	-0.043	0.204	0.080	0.113	0.002	0.955	0.015	0.775	0.056	0.468	0.050	0.465
Prevotella7	genus	10	-0.011	0.548	0.016	0.409	0.025	0.467	-0.001	0.968	0.029	0.404	0.095	0.051	-0.034	0.466
Ruminococcusgnavusgroup	genus	10	-0.026	0.285	-0.039	0.095	0.053	0.175	0.019	0.440	-0.025	0.629	0.054	0.417	-0.041	0.478
Alphaproteobacteria	class	8	-0.015	0.660	0.013	0.700	-0.054	0.353	0.037	0.286	0.053	0.393	-0.040	0.622	0.059	0.483
Alloprevotella	genus	6	0.023	0.296	0.005	0.886	-0.001	0.982	-0.004	0.866	-0.018	0.691	-0.053	0.357	0.036	0.488
Lachnoclostridium	genus	13	0.003	0.944	0.028	0.479	0.083	0.213	-0.086	0.038	-0.130	0.067	-0.130	0.156	-0.086	0.488
Dorea	genus	13	0.047	0.147	-0.042	0.285	-0.035	0.568	0.009	0.834	0.068	0.310	0.040	0.657	0.059	0.490
Lactococcus	genus	9	-0.005	0.801	0.012	0.546	0.007	0.843	0.018	0.451	-0.031	0.422	0.010	0.843	0.030	0.509
Sellimonas	genus	8	0.003	0.855	-0.008	0.665	0.035	0.245	0.010	0.589	-0.001	0.980	0.031	0.513	-0.029	0.511
Coprobacter	genus	13	-0.022	0.308	0.021	0.299	0.007	0.859	0.012	0.574	-0.030	0.517	-0.010	0.859	-0.038	0.516
Subdoligranulum	genus	13	0.026	0.451	0.004	0.911	-0.049	0.429	0.040	0.278	0.107	0.099	0.030	0.720	0.058	0.525
Defluviitaleaceae	family	11	-0.051	0.063	0.004	0.893	0.014	0.776	0.032	0.285	-0.020	0.701	0.088	0.194	-0.043	0.538
Actinomycetaceae	family	4	-0.052	0.176	-0.040	0.265	0.028	0.802	-0.027	0.484	0.019	0.821	-0.053	0.630	0.065	0.548
Actinomycetales	order	4	-0.052	0.176	-0.040	0.267	0.027	0.803	-0.027	0.486	0.019	0.820	-0.053	0.631	0.065	0.548
RuminococcaceaeNK4A214group	genus	14	0.054	0.096	0.071	0.028	-0.046	0.560	0.019	0.561	-0.004	0.942	-0.002	0.977	0.045	0.561
ChristensenellaceaeR.7	genus	7	-0.033	0.492	0.030	0.474	0.116	0.109	-0.027	0.531	0.040	0.679	-0.030	0.794	-0.066	0.561
Mollicutes	class	8	0.001	0.976	0.063	0.223	0.103	0.091	0.007	0.838	-0.013	0.835	-0.011	0.892	-0.046	0.574
Tenericutes	phylum	8	0.001	0.976	0.063	0.223	0.103	0.091	0.007	0.838	-0.013	0.835	-0.011	0.892	-0.046	0.574
Verrucomicrobia	phylum	11	0.011	0.731	-0.044	0.213	-0.013	0.812	-0.022	0.531	-0.114	0.074	-0.103	0.191	-0.058	0.581

Gut microbiota	Group	N	beta.VHD	pvalue.VHD	beta.GORD	pvalue.GORD	beta.PUD	pvalue.PUD	beta.IBS	pvalue.IBS	beta.IBD	pvalue.IBD	beta.CD	pvalue.CD	beta.UC	pvalue.UC
Slackia	genus	7	-0.004	0.906	-0.036	0.254	-0.016	0.752	-0.049	0.092	0.004	0.948	-0.036	0.624	0.041	0.597
Parabacteroides	genus	7	0.063	0.166	-0.027	0.479	0.047	0.527	-0.049	0.289	0.019	0.803	-0.263	0.013	0.049	0.604
Desulfovibrio	genus	10	-0.006	0.856	-0.048	0.124	-0.064	0.220	-0.034	0.243	0.013	0.811	0.109	0.107	-0.036	0.622
Eisenbergiella	genus	11	0.002	0.914	0.033	0.146	-0.037	0.361	-0.046	0.055	0.047	0.303	-0.003	0.963	0.027	0.622
Ruminiclostridium9	genus	15	-0.033	0.307	-0.003	0.934	0.076	0.196	0.010	0.778	-0.017	0.808	-0.003	0.969	-0.040	0.623
Howardella	genus	9	0.023	0.253	-0.005	0.789	0.036	0.271	0.014	0.481	-0.016	0.647	0.003	0.950	-0.022	0.633
FamilyXI	family	9	-0.022	0.218	-0.023	0.328	-0.044	0.144	0.008	0.700	-0.014	0.678	-0.056	0.216	0.021	0.637
Intestinibacter	genus	13	-0.064	0.035	0.014	0.650	-0.049	0.379	-0.010	0.759	0.051	0.385	0.025	0.746	0.036	0.646
Anaerostipes	genus	12	-0.039	0.282	0.043	0.223	0.044	0.459	-0.072	0.059	0.016	0.822	0.040	0.642	-0.041	0.654
Gordonibacter	genus	13	0.033	0.043	-0.020	0.241	0.018	0.538	0.006	0.752	0.050	0.111	0.023	0.566	0.018	0.655
Paraprevotella	genus	13	-0.008	0.719	0.007	0.763	0.031	0.424	-0.040	0.094	-0.022	0.609	-0.090	0.117	0.025	0.661
Bifidobacterium	genus	14	-0.063	0.031	-0.007	0.813	0.080	0.139	0.023	0.431	-0.056	0.260	-0.054	0.414	0.030	0.670
Burkholderiales	order	12	0.008	0.820	0.009	0.810	-0.108	0.110	0.045	0.229	-0.070	0.300	-0.110	0.224	0.035	0.680
Bacteroidaceae	family	9	-0.054	0.193	-0.009	0.850	0.100	0.178	-0.009	0.846	0.125	0.204	0.090	0.451	-0.048	0.682
Bacteroides	genus	9	-0.054	0.193	-0.009	0.850	0.100	0.178	-0.009	0.846	0.125	0.204	0.090	0.451	-0.048	0.682
Parasutterella	genus	15	-0.009	0.715	0.022	0.488	0.024	0.572	0.008	0.751	0.035	0.467	0.100	0.148	-0.028	0.684
RuminococcaceaeUCG003	genus	10	0.011	0.747	-0.034	0.364	-0.072	0.244	0.036	0.305	-0.171	0.028	-0.196	0.031	-0.033	0.699
Fusicatenibacter	genus	19	-0.046	0.121	0.000	0.992	-0.024	0.679	0.000	0.995	0.057	0.353	0.064	0.561	-0.031	0.703
Lachnospiraceae	family	17	-0.015	0.644	0.032	0.296	0.073	0.187	-0.059	0.059	0.054	0.352	0.028	0.725	0.028	0.704
Streptococcus	genus	13	0.046	0.180	0.010	0.748	-0.059	0.255	-0.067	0.035	0.001	0.986	0.021	0.798	-0.029	0.713
Streptococcaceae	family	13	0.018	0.592	0.023	0.429	-0.041	0.411	0.004	0.886	-0.003	0.966	0.010	0.900	-0.027	0.719
Akkermansia	genus	12	0.012	0.692	-0.004	0.917	0.000	0.995	-0.020	0.556	-0.120	0.049	-0.199	0.012	-0.026	0.725
Verrucomicrobiaceae	family	12	0.012	0.691	-0.004	0.917	0.000	0.997	-0.019	0.558	-0.120	0.050	-0.199	0.012	-0.026	0.726
Verrucomicrobiae	class	12	0.012	0.690	-0.004	0.916	0.000	0.997	-0.019	0.559	-0.120	0.050	-0.199	0.012	-0.026	0.727
Verrucomicrobiales	order	12	0.012	0.690	-0.004	0.916	0.000	0.997	-0.019	0.559	-0.120	0.050	-0.199	0.012	-0.026	0.727
Porphyromonadaceae	family	10	0.033	0.422	0.011	0.788	0.032	0.641	0.034	0.440	0.035	0.632	-0.071	0.470	0.033	0.729
Actinobacteria	phylum	15	-0.055	0.104	-0.049	0.125	-0.049	0.382	0.040	0.212	-0.092	0.114	-0.112	0.124	-0.025	0.739
Clostridiuminnocuumgroup	genus	10	-0.035	0.061	0.024	0.291	-0.009	0.815	-0.028	0.216	-0.016	0.699	-0.031	0.586	0.017	0.749
LachnospiraceaeNC2004group	genus	9	-0.049	0.058	-0.002	0.936	0.011	0.806	-0.017	0.553	-0.009	0.866	-0.011	0.860	0.020	0.768
LachnospiraceaeNK4A136group	genus	13	0.000	0.991	-0.017	0.587	-0.025	0.697	0.021	0.545	-0.061	0.286	-0.181	0.029	-0.023	0.778
Methanobrevibacter	genus	6	0.055	0.040	-0.036	0.105	-0.052	0.176	-0.023	0.331	-0.016	0.735	0.014	0.821	0.015	0.792
Hungatella	genus	5	0.005	0.866	0.060	0.061	0.021	0.666	-0.004	0.885	-0.064	0.258	-0.109	0.186	-0.021	0.794
Eubacteriumhalliigroup	genus	12	-0.005	0.869	0.022	0.486	0.041	0.481	0.070	0.047	0.055	0.423	0.038	0.662	0.022	0.795
Barnesiella	genus	15	-0.035	0.448	-0.001	0.983	-0.027	0.608	0.021	0.504	0.006	0.914	0.071	0.339	0.018	0.801
Eubacteriumnodatumgroup	genus	10	-0.009	0.630	0.001	0.958	0.070	0.034	0.002	0.929	-0.027	0.436	-0.011	0.806	0.011	0.807
unknownfamily	family	14	0.060	0.024	0.019	0.479	-0.045	0.342	-0.004	0.886	0.020	0.703	-0.006	0.924	-0.016	0.808
unknowngenus	genus	14	0.060	0.024	0.019	0.479	-0.045	0.342	-0.004	0.886	0.020	0.703	-0.006	0.924	-0.016	0.808
MollicutesRF9	order	14	0.060	0.024	0.019	0.479	-0.045	0.342	-0.004	0.886	0.020	0.703	-0.006	0.924	-0.016	0.808
BacteroidalesS24.7	family	9	0.006	0.826	0.004	0.878	-0.036	0.464	-0.010	0.728	0.016	0.749	0.052	0.432	0.014	0.839
unknowngenus	genus	9	0.006	0.826	0.004	0.878	-0.036	0.464	-0.010	0.728	0.016	0.749	0.052	0.432	0.014	0.839
Butyrivibrio	genus	16	0.000	0.995	-0.009	0.566	-0.039	0.132	0.003	0.870	-0.002	0.942	-0.019	0.609	0.007	0.846
Eubacteriumcoprostanoligenesgroup	genus	12	-0.018	0.628	-0.002	0.955	-0.079	0.258	-0.065	0.083	0.011	0.873	0.021	0.869	0.016	0.852
Marvinbryantia	genus	9	0.013	0.721	0.028	0.432	0.159	0.010	-0.035	0.350	-0.006	0.923	-0.083	0.290	0.013	0.867
Alcaligenaceae	family	18	-0.041	0.160	0.023	0.444	-0.129	0.023	0.021	0.502	-0.030	0.618	-0.059	0.436	-0.011	0.879
Rhodospirillaceae	family	14	0.020	0.395	-0.026	0.269	0.005	0.908	0.055	0.035	0.029	0.530	-0.068	0.263	0.009	0.882
Betaproteobacteria	class	12	0.008	0.821	0.001	0.980	-0.070	0.258	0.038	0.286	-0.083	0.203	-0.165	0.050	-0.012	0.883
Phascolarctobacterium	genus	9	-0.046	0.168	-0.082	0.013	-0.026	0.619	-0.005	0.871	0.064	0.289	0.112	0.150	-0.010	0.889
Romboutsia	genus	15	-0.061	0.032	-0.014	0.647	-0.043	0.366	-0.007	0.836	-0.013	0.812	-0.092	0.204	0.009	0.898
Coprococcus3	genus	9	-0.068	0.098	-0.037	0.366	0.032	0.648	0.027	0.503	0.055	0.627	0.169	0.105	0.014	0.927
Lachnospira	genus	6	0.059	0.239	-0.012	0.805	-0.143	0.149	-0.035	0.501	0.059	0.555	-0.186	0.147	0.009	0.945
Holdemanella	genus	10	0.041	0.088	-0.014	0.609	0.004	0.936	0.002	0.929	-0.059	0.218	-0.103	0.093	0.003	0.951

Gut microbiota	Group	N	beta.VHD	pvalue.VHD	beta.GORD	pvalue.GORD	beta.PUD	pvalue.PUD	beta.IBS	pvalue.IBS	beta.IBD	pvalue.IBD	beta.CD	pvalue.CD	beta.UC	pvalue.UC
Eubacteriumrectalegroup	genus	12	-0.036	0.268	0.014	0.721	0.033	0.595	0.008	0.833	-0.005	0.947	-0.084	0.373	-0.005	0.956
Escherichia.Shigella	genus	13	-0.023	0.438	-0.001	0.986	0.018	0.735	0.044	0.187	0.038	0.550	0.075	0.345	0.004	0.965
Gammaproteobacteria	class	8	-0.019	0.671	-0.004	0.930	0.141	0.056	-0.042	0.330	-0.067	0.412	0.019	0.853	0.004	0.969
unknowngenus	genus	14	0.007	0.751	0.001	0.952	-0.021	0.632	0.054	0.044	0.012	0.797	-0.039	0.536	0.004	0.970
Prevotella9	genus	18	-0.033	0.115	0.038	0.093	0.032	0.433	0.039	0.110	-0.015	0.740	0.032	0.585	-0.002	0.973
LachnospiraceaeND3007group	genus	3	-0.060	0.403	0.058	0.504	-0.100	0.509	-0.085	0.301	0.214	0.094	0.144	0.446	0.005	0.974
Odoribacter	genus	7	-0.065	0.131	0.047	0.251	-0.035	0.640	-0.031	0.463	-0.007	0.928	0.150	0.130	-0.002	0.987
Bifidobacteriaceae	family	17	-0.028	0.321	-0.016	0.627	0.033	0.575	0.048	0.103	-0.075	0.140	-0.100	0.128	0.000	0.994
Bifidobacteriales	order	17	-0.028	0.321	-0.016	0.627	0.033	0.575	0.048	0.103	-0.075	0.140	-0.100	0.128	0.000	0.994
Adlercreutzia	genus	11	-0.020	0.467	-0.004	0.923	-0.022	0.678	0.029	0.334	0.014	0.820	0.042	0.587	0.000	0.995
